# Supplementary material for: The lipocone superfamily, a unifying theme in metabolism of lipids, peptidoglycan and exopolysaccharides, inter-organismal conflicts and immunity
Source: eLife. 2025 Sep 9;14:RP108061. doi: 10.7554/eLife.108061 (PMC12419801; doi:10.7554/eLife.108061)
Supplement: Figure 5—source data 1. [file elife-108061-fig5-data1.pdf]

**Figure 5—Source Data 1**. Lipocone family conserved contextual associations across distinct functional themes.

| Function | Family | Genome associations[1] | Phyletic pattern notes[2] |
|---|---|---|---|
| Lipid head group exchange reactions | Euk-PTDSS1/2 | Phosphatidylserine production | Pan-eukaryotic |
| | Prok-PTDSS | Archaeophosphatidylserine production | sporadic archaea, bacteria, and viruses |
| | cpCone-1 | Kleisin-ScpA, HTH-ScpB | Patescibacteria |
| | | TM+LemA, TM+TM+MPTase, single cpCone-1 KptA domain fusion | |
| | | LP+LolA, 4TM with β-curl insert, [TM+NLPC/p60] | FCB group |
| | cpCONE-DUF2585 | HslV-Peptidase, GNAT, ClpB-HslU | Alphaproteobacteria |
| | | Glycolate-oxidase-GlcE, glycolate-oxidase-GlcF | |
| Cardiolipin synthesis | Wok-DUF2238 | Synaptojanin-like, [GlycosylTFase-A], [α/β-hydrolase], [NUDIX] | Terrabacteria, Pseudomonadota |
| | | Two-gene associations with diverse phosphatases: Calcineurin, HAD, PAP2 | Terrabacteria (sp), other lineages |
| Modified isoprenoid lipid synthesis | Wok-DUF2238 | Carotenoid biosynthesis module, GlycosylTFase-A, NUDIX | Actinomycetota |
| | YfiM-1 | amidophosphoribosyltransferase, UbiA-prenylTFase, RidA deaminase, TM-containing DUF5638, HD, PHP+Ig | Calditrichota, FCB group, Gemmatimonadetes |
| Lipid head group modifications in peptidoglycan dynamics | VanZ-1 & VanZ-2 | peptidoglycan glycosylTFases, D-Ala-D-Ala-peptidases, MurE-synthetase MurD-synthetase, MATE-flippase | Gene neighborhoods in Terrabacteria, FCB group, and Pseudomonadota |
| Lipid head group modifications in exopolysaccharide metabolism | VanZ-1 & VanZ-2 | WaaL-ligase, Wzc-CpsD-N, ElyC | Gene neighborhoods in Terrabacteria, FCB & PVC groups, and Pseudomonadota |
| Uncharacterized modifications of peptidoglycan and the outer membrane | VanZ-1+VanZ-i | Ferredoxin, α/β-hydrolase lipase, D-Ala-D-Ala-MβL transpeptidase | Betaproteobacteria |
| | VanZ-2 | SprA-N, GCV-H, 2TM+proline-rich-linker+TonB-C, TonB-C+OMP-β-barrel | FCB group |
| | VanZ-2 | ABC ATPase transporter, TM+coiled-coil+Papain-like or gly-gly-peptidase, SP+LTDs | Patescibacteria |
| Lipocone domains operating in or in transit to the outer membrane | YfiM-Griddle (up to 3 copies) | OMP-β-barrel(f), extended β-hairpin(f), LolA, POTRA, PLUG, TolB-N, Patatin lipase, GlycosylTFase-B, PAP2, LP+Synaptojanin, R-P(f) | Gram-negative bacteria |
| | YfiM-DUF2279 | OMP-β-barrel(f), MltG-endolytic-TGase, LP+Cytochrome-C7, PMM/PGM, GNAT, diaminopimelate-epimerase, Lysozyme | FCB group |
| | YfiM-DUF2279 | OMP-β-barrel(f), GlycosylTFase-A, OMP-β-barrel, SP+PDZ+ClpP-protease | FCB group |
| | ClaspCone-1 | TM(f) or 5TM(f), TULIP(f) or Ig and MPTase(f), [PHP](f), GDSL-Lipase, MBOAT | Pseudomonadota, Planctomycetota |
| Membrane-anchoring linkage | Skillet-1 | Specialized lipobox(f), diverse ligand-binding domains(f): Ig, Jellyroll, βPs, Concanavalin, OB-fold, SHOCT, MORNs | Bacillota, FCB group, Pseudomonadota |

| | | | |
|---|---|---|---|
| Lipid-associated signaling systems, standalone proteins | VanZ-1 | HTH(f), RHH(f), YycI(f), RDD(f), Glyoxylase(f), NPCBM(f) | Widespread, sporadic linkages |
| | VanZ-2 | cNMPDB(f), FHA(f), KTSC(f), Papain(f), TPRs(f), Calcineurin(f), CBD9(f) | |
| | Skillet-3 | Ig(f), β-sandwich(f), helix-grip(f), βPs(f), MORNs(f), Lipocalin(f), β-barrel(f) | Pseudomonadota, FCB group, Terrabacteria |
| Lipid-associating signaling systems, multicomponent | VanZ-1 | HAAS(f), PadR-HTH | Bacillota |
| | Skillet-2 | helix+TM or ZnR+helix+TM or HTH+L12-ClpS+TM, TetR transcriptional repressor, [HMG-CoA-reductase+GHMP-kinase], [SP+Ig repeats] | Bacteriodota, Bacillota (sp) |
| | Skillet-DUF2809 | wHTH, cytoplasmic-helix+6TM protein. Joined by one or more of: ElyC, CreD, Coq4, Lcp-like, DUF1361, TGase | FCB group, Pseudomonadota (sp) |
| Antiviral immunity | Min-Wnt | DUF3892(f) | Pseudomonadota (sp) |
| | | 3-strand β-meander(f), LP+PPTs, SP+Glycosyl-hydrolase, SP+β-helix | Bacteroidota |
| | | Standalone | Cyanobacteria |
| | | helical-domain+PcfJ-GNAT(f) | Duplodnaviria |
| Toxin domains in polymorphic and allied conflict systems | Min-Wnt | SP or LP+tail(f), LP+Imm-BamE or LP+Imm-Jellyroll or Imm-4TM | Terrabacteria, Pseudomonadota, FCB group, Elusimicrobia, Acidobacteria, PVC group (sp), Archaea (sp) |
| | | Polymorphic toxin delivery systems: T1SS, T4SS, T6SS, T7SS, T9SS, DUF4157-MPTase, Immunity proteins as above | |
| | | LP+Cystatin-FD (f), LP+Imm-Jellyroll (dominant) or LP+Imm-BamE | Bacteroidota |
| | Prok-SAA | Polymorphic toxin delivery systems: T6SS, MuF, TM+[TM+TM+]Imm-SAA or LP+Imm-BamE | Spirochaetota, Nitrospirota, Acidobacteriota, Terrabacteria (sp), PVC group, Pseudomonadota (sp), Fusobacteriota, Bacteriodota (sp) |
| | | PGBD(f), TM+Imm-SAA | Pseudomonadota (sp) |
| | Prok-TelC | Polymorphic toxin delivery systems: T6SS, T7SS, MPTase-DUF4157, ZU5+vWA core {31064832}, Imm-TipC, Imm-Zu5/vWA | Bacillota, Actinomycetota (sp), Myxococcota (sp), FCB and PVC groups (sp), Pseudomonadota (sp) |
| | | SP+GbpC+MucBP-IG(f), Imm-TipC | Bacillota and Actinomycetota (sp) |
| | | TPM+TPM(f) or TPM+Ig, Imm-4TM | Bacteriodota |
| | CapCone-1 | Polymorphic toxin delivery systems: T6SS (including PsbP/MOG1-like fusion), MPTase-DUF4157, LP+Imm-BamE | Pseudomonadota, PVC group, Terrabacteria (sp), FCB group (sp) |
| | | Cystatin-FD+linker, LP+Imm-BamE | Bacteroidota |
| | CapCone-2 | SP(f), SP+Imm-BamE | Bdellovibrionota, Acidobacteria (sp) |

| | | | |
|---|---|---|---|
| | | Polymorphic toxin delivery systems: T6SS, MPTase-DUF4157 | Pseudomonadota (sp), FCB group (sp), PVC group (sp), Archaea (sp) |
| | | ANKs(f), SP+Imm-SAS6-N-like-β-sandwich | PVC group (sp), Pseudomonadota (sp) |
| | ClaspCone-2 | Polymorphic toxin delivery systems: T6SS, Imm-4TM | FCB group (sp), PVC group (sp), Pseudomonadota (sp) |
| | VanZ-1 | Polymorphic toxin delivery systems: T6SS | Bacillota |
| Toxins in predator-prey and other inter-specific conflicts | Min-Wnt | SP+half-β-barrel(f), CC-motif-containing-tail(f), C-terminal helical-extension(f) | Bacteroidota, PVC group, Terrabacteria (sp), Hemichordata, Rotifera, fungi |
| | | Broken-hairpin(f) | Alphaproteobacteria (sp), Duplodnaviria (sp), Terrabacteria (sp) |
| | CapCone-2 | SP(f), Patatin(f), Lipocalin, acyltransferase+TM+TM+TM, SP+α/β-hydrolase, SP+OMP-β-barrel | Bdellovibrionota, Holophagales, Archangium, Woeseiaceae, Labrenzia, Roseibium |
| | Prok-SAA | SP+MTPase+Prok-SAA+vWD+Ig+Ig | Gemmatimonadetes (sp), Pseudomonata (sp) |
| | Skillet | Histidine kinase-Receiver, MPTase, Papain-like, MTases, LysM & other ligand-bindings domains, etc. | Omnitrophica Patescibacteria |
| | Prok-TelC | NAGPA(f), ligand-binding(f): Ig, CW-repeats, βPs, β-sandwich, etc. | Bacillota |
| | | PGBD+PGBD+Rv2525c-like-TIM-barrel(f), SP+Ig+Ig*, 3TM-CCDN*, SP+VanY* | Bacillota, fungi (sp), Actinomycetota* (sp) |
| Resistance to antimicrobial agents | VanZ-1, VanZ-2, Skillet-DUF2809* | VanY, vancomycin resistance modules, D-Ala-D-Ala-MβL* | Terrabacteria, FCB group (sp)*, Pseudomonata (sp)* |
| | YfiM-1 | Thioredoxin, DTW-SPOUT, acetate—CoA-ligase+ATP-grasp+GNAT, HKD fold phosphatidylserine synthetase | Gammaproteobacteria (sp) |

[1](f): denotes a domain that is directly fused to the Lipocone family; *: associations present in a phylogenetically restricted subset; [x]: association is not universally observed; GlycosylTFase-A: glycosyltransferase-A; TFase: transferase; TGase: transglycosylase; CW: cell wall; MTase: Methylase; MBL: metallo-β-lactamase; TM: transmembrane; SP: signal peptide; LP: membrane-anchored lipoprotein; T[x]SS: Type-X-secretory system; Imm: immunity protein; βPs: β-propellers;

[2](sp): denotes sporadic distribution in the listed phylogeny; *: phylogenies with restricted associations
