# Supplementary material for: The lipocone superfamily, a unifying theme in metabolism of lipids, peptidoglycan and exopolysaccharides, inter-organismal conflicts and immunity
Source: eLife. 2025 Sep 9;14:RP108061. doi: 10.7554/eLife.108061 (PMC12419801; doi:10.7554/eLife.108061)
Supplement: Figure 6—source data 1. [file elife-108061-fig6-data1.pdf]

Figure 6—Source Data 1. Significant enrichment of Lipocone family contextual associations across functional categories.

**Lipocone family**	**Function**	**p-value**
Prok-TelC	Biological conflict	0.0
Min-Wnt	Biological conflict	0.0
VanZ-2	Sugar metabolism	0.0
Skillet-1	Adhesion/extracellular matrix	0.000001
VanZ-2	Peptidoglycan	0.000002
YfiM-Griddle	Outer membrane	0.000015
VanZ-1	Sugar metabolism	0.000022
YfiM-1	Isoprenoid metabolism	0.000171
Wok-DUF2238	Isoprenoid metabolism	0.000242
Skillet-DUF2809	Exopolysaccharide	0.000296
Skillet-2	Transcription	0.00053
CapCone-1	Biological conflict	0.000636
VanZ-1	Exopolysaccharide	0.000836
Skillet-3	Adhesion/extracellular matrix	0.000839
cpCone-i	Cell membrane	0.003064
CapCone-2	Biological conflict	0.003375
VanZ-i	Sugar metabolism	0.003824
cpCone-1	Cell membrane	0.007168
