# Supplementary material for: The lipocone superfamily, a unifying theme in metabolism of lipids, peptidoglycan and exopolysaccharides, inter-organismal conflicts and immunity
Source: eLife. 2025 Sep 9;14:RP108061. doi: 10.7554/eLife.108061 (PMC12419801; doi:10.7554/eLife.108061)
Supplement: Figure 5—source data 2. — Features and coloring as described in the Figure 5 legend. [file elife-108061-fig5-data2.pdf]

###########:CapCone-1; ###########;Polymorphic toxin and allied conflict system associations;
###;MPTase (DUF4157) associations

Accession	Organism	Architecture
KFB74168.1	*Candidatus Accumulibacter phosphatis*	DUF4157-MPTase, CapCone-1, Cadherin-like
TCO80656.1	*Plasticicumulans lactativorans*	DUF4157-MPTase, CapCone-1, Cadherin-like
MBM3235931.1	*Candidatus Poribacteria bacterium*	DUF4157-MPTase / DUF4157-MPTase, CapCone-1, Cadherin-like
WP_131253169.1		DUF4157-MPTase, CapCone-1, Cadherin-like, 4helix-bundle
MBF04199.1	*Flavobacterium sp.*	disordered, DUF4157-MPTase, CapCone-1, 6helix-bundle
WP_106522428.1	*Taibaiella chishuiensis*	disordered, DUF4157-MPTase, CapCone-1, 6helix-bundle
MBC8045620.1	*Chitinophagales bacterium*	disordered, DUF4157-MPTase, CapCone-1, 6helix-bundle
WP_203709243.1	*Actinoplanes digitatis*	disordered, DUF4157-MPTase, CapCone-1, 6helix-bundle
WP_218952111.1	*Amycolatopsis anabasis*	DUF4157-MPTase, CapCone-1, 6helix-bundle
WP_211845705.1	*Neoroseomonas eburnea*	disordered, DUF4157-MPTase, CapCone-1
WP_139229732.1	*Halogeometricum limi*	disordered, DUF4157-MPTase, CapCone-1
TQN44781.1	*Humibacillus xanthopallidus*	DUF4157-MPTase, CapCone-1
WP_186814740.1	*Cellulomonas terrae*	DUF4157-MPTase, CapCone-1
TQF83457.1	*Elioraea sp. Yellowstone*	DUF4157-MPTase, CapCone-1
WP_206046749.1	*Mangrovimicrobium sediminis*	DUF4157-MPTase, CapCone-1
MCB0569717.1	*Phaeodactylibacter sp.*	CapCone-1, Cadherin-like / DUF4157-MPTase
MBC7851302.1	*Chitinophagaceae bacterium*	MTPase, MTPase, CapCone-1, 4helix-bundle
MBS1133303.1	*Burkholderiaceae bacterium*	MTPase, MTPase, CapCone-1, 4helix-bundle / DUF4157-MPTase
MBO0857063.1	*Chloracidobacterium sp.*	DUF4157-MPTase, CapCone-1
WP_131170031.1	*Propioniciclava sinopodophylli*	DUF4157-MPTase, CapCone-1

#;DUF4157-MPTase+CapCone-1

Accession	Organism	Architecture
ASC72636.1	*Halomicronema hongdechloris C2206*	DUF4157-MPTase, CapCone-1
MBA2669176.1	*Gemmatimonadota bacterium*	DUF4157-MPTase, CapCone-1
MBN1323498.1	*Methanotrichaceae archaeon*	DUF4157-MPTase, CapCone-1
NJK65332.1	*Microcoleus sp. SU_5_3*	DUF4157-MPTase, CapCone-1

#;Polymorphic with immunity

Accession	Organism	Architecture
SNA88534.1	*Flavobacterium psychrophilum*	RHS-repeats, CapCone-1 / BamE-like-Imm
MBT7801657.1	*Tateyamaria sp.*	RHS-repeats, CapCone-1 / BamE-like-Imm
MBR1727693.1	*Muribaculaceae bacterium*	RHS-repeats, CapCone-1 / BamE-like-Imm
WP_233777403.1	*Fulvivirga maritima*	RHS-repeats, CapCone-1 / BamE-like-Imm
NLL51837.1	*Peptococcaceae bacterium*	RHS-repeats, CapCone-1 / BamE-like-Imm
MBP5668435.1	*Salinivirgaceae bacterium*	RHS-repeats, CapCone-1 / BamE-like-Imm
MBN1315670.1	*Anaerolineales bacterium*	RHS-repeats, CapCone-1 / BamE-like-Imm
HGV25085.1		RHS-repeats, CapCone-1 / BamE-like-Imm
RKY40123.1	*Candidatus Makaraimicrobium thalassicum*	RHS-repeats, CapCone-1 / BamE-like-Imm
MCB0401153.1	*Flavobacteriales bacterium*	RHS-repeats, CapCone-1 / BamE-like-Imm
MBE7696477.1	*Tenacibaculum finnmarkense genomovar ulcerans*	CapCone-1(frag) / BamE-like-Imm
MCB9703292.1	*Myxococcales bacterium*	CapCone-1 / BamE-like-Imm
MBK8485175.1	*Saprospiraceae bacterium*	CapCone-1(frag) / BamE-like-Imm
MBQ9509589.1		CapCone-1(frag) / BamE-like-Imm
RPD96773.1	*Aureibaculum marinum*	CapCone-1(frag) / BamE-like-Imm
WP_051663830.1	*Bacteroides sp. Ga6A1*	CapCone-1(frag) / BamE-like-Imm
MBP5549404.1	*Bacteroidales bacterium*	CapCone-1(frag) / BamE-like-Imm
MBS6329184.1	*Prevotella bivia*	CapCone-1(frag) / BamE-like-Imm
WP_232223185.1	*Prevotella sp. S7 MS 2*	CapCone-1 / BamE-like-Imm
MBP5517511.1	*Bacteroidales bacterium*	CapCone-1 / BamE-like-Imm
WP_052352632.1	*Chryseobacterium oranimense G311*	CapCone-1 / BamE-like-Imm
MBE7442818.1	*Flavobacteriales bacterium*	RHS-repeats, CapCone-1
MBI2464489.1	*Candidatus Peregrinibacteria bacterium*	RHS-repeats, CapCone-1
MBR9704654.1	*Candidatus Pacearchaeota archaeon*	RHS-repeats, CapCone-1
MBM4185661.1	*Gemmatimonadota bacterium*	RHS-repeats, CapCone-1
WP_009161933.1	*Hoylesella saccharolytica F0055*	RHS-repeats, CapCone-1
WP_187465880.1	*Neolewinella lacunae*	RHS-repeats, CapCone-1
MBR1637701.1	*Bacteroidales bacterium*	RHS-repeats, CapCone-1
MBI2547048.1	*Candidatus Aenigmatarchaeota archaeon*	RHS-repeats, CapCone-1
MBI2184782.1	*Nitrososphaerota archaeon*	RHS-repeats, CapCone-1 / ? / RHS-repeats
NOX63382.1	*Chloroflexota bacterium*	RHS-repeats, CapCone-1
NEN24156.1	*Cryomorpha ignava*	RHS-repeats, CapCone-1
MBK9402640.1	*Bacteroidota bacterium*	RHS-repeats, CapCone-1
PID57947.1	*Ignavibacteriota bacterium*	RHS-repeats, CapCone-1
MBK7444144.1	*Bacteroidota bacterium*	RHS-repeats, CapCone-1
MBU0496713.1	*Thermoplasmatota archaeon*	RHS-repeats, CapCone-1
ODS37095.1	*Candidatus Altiarchaeales archaeon WOR_SM1_86-2*	RHS-repeats, CapCone-1
MCA9931923.1	*Anaerolineales bacterium*	RHS-repeats, CapCone-1 / ? / ? / RHS-repeats
MBN2777898.1	*Bacteroidales bacterium*	RHS-repeats, CapCone-1
MBI9051560.1	*Anaerolineaceae bacterium*	RHS-repeats, CapCone-1 / ? / RHS-repeats
HAY34298.1	*Bacteroidota bacterium*	RHS-repeats, CapCone-1
MBN8641151.1	*Flavobacteriales bacterium*	RHS-repeats, CapCone-1
MBU1019176.1	*Patescibacteria group bacterium*	RHS-repeats, CapCone-1
WP_148114543.1	*Gynurincola endophyticus*	RHS-repeats, CapCone-1
PIZ95216.1	*Candidatus Magasanikbacteria bacterium CG_4_10_14_0_2_um_filter_37_12*	RHS-repeats, CapCone-1
MBD78553.1	*Crocinitomicaceae bacterium*	RHS-repeats, CapCone-1
MCB0230325.1	*Anaerolineae bacterium*	RHS-repeats, CapCone-1
MBI4154872.1	*Candidatus Woesearchaeota archaeon*	RHS-repeats, CapCone-1
MBK8458223.1	*Phyllobacteriaceae bacterium*	RHS-repeats, CapCone-1
WP_081799877.1	*Thermoanaerobaculum aquaticum*	RHS-repeats, CapCone-1
MCC6965825.1	*Nitrospira sp.*	RHS-repeats, CapCone-1
NWG91432.1	*Parvularculaceae bacterium*	RHS-repeats, CapCone-1 / ? / RHS-repeats, CapCone-1
MBC6949168.1	*Candidatus Zhuqueibacterota bacterium*	RHS-repeats, CapCone-1
MBI4209405.1	*Deltaproteobacteria bacterium*	RHS-repeats, CapCone-1
MCD4699059.1	*Phycisphaerae bacterium*	RHS-repeats, CapCone-1
NUQ86799.1	*Anaerolineales bacterium*	RHS-repeats, CapCone-1
MBK7637550.1	*Saprospiraceae bacterium*	RHS-repeats / CapCone-1-min
TAE84144.1	*Bacteroidota bacterium*	RHS-repeats, CapCone-1
MBK8268125.1	*Planctomycetota bacterium*	RHS-repeats, CapCone-1
MBV6395160.1	*Anaerolineales bacterium*	RHS-repeats, CapCone-1
MBN8618824.1	*Anaerolineae bacterium*	RHS-repeats, CapCone-1
AFM02643.1	*Bernardetia litoralis DSM 6794*	RHS-repeats, CapCone-1
MBK8478406.1	*Opitutaceae bacterium*	RHS-repeats, CapCone-1
PKO17016.1	*candidate division BRC1 bacterium HGW-BRC1-1*	RHS-repeats, CapCone-1
MBT6823893.1	*archaeon*	RHS-repeats, CapCone-1
MBP7874151.1	*Bacteroidales bacterium*	RHS-repeats, CapCone-1

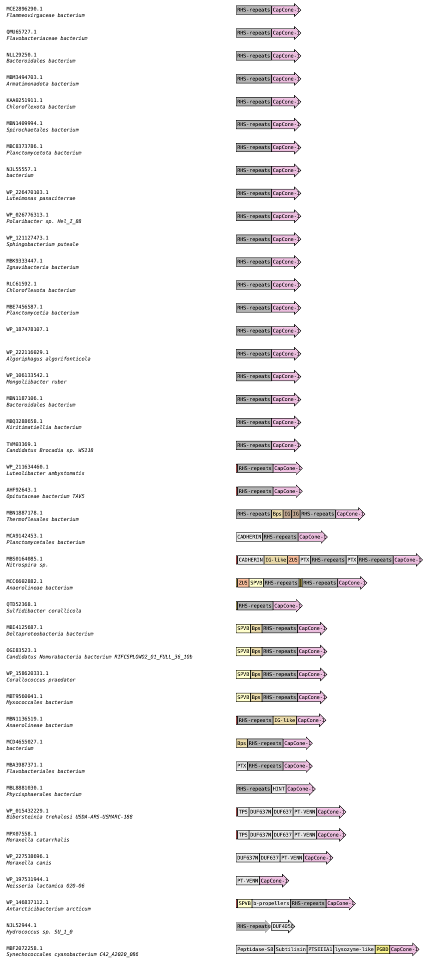

#####;PSBP-like domain transport-mediated toxin deployment systems

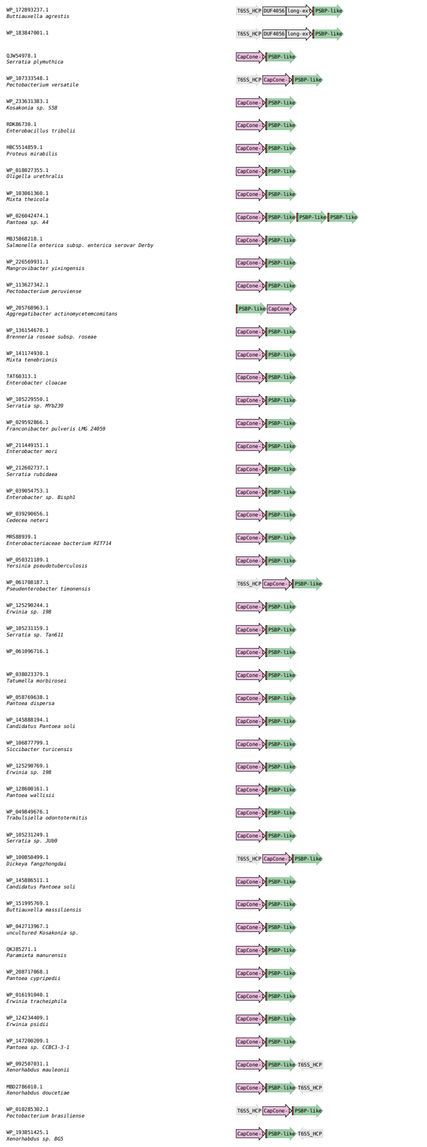

WP_139654995.1
Photorhabdus luminescens subsp. sonorensis
CapCone-1 | PSBP-like

WP_115458454.1
Enterobacillus tribolii
T6SS_HCP | CapCone-1 | PSBP-like

WP_036847594.1
Photorhabdus khanii
CapCone-1 | PSBP-like

WP_204771885.1
Lelliottia amnigena
ZapB | CapCone-1 | PSBP-like

MBM6608475.1
Enterobacteriaceae bacterium RIT 814
ZapB | CapCone-1 | PSBP-like

WP_144416807.1
Yersinia pseudotuberculosis
CapCone-1 | PSBP-like | T6SS_HCP

WP_125370365.1
Citrobacter farmeri
ZapB | CapCone-1 | PSBP-like

WP_164563256.1
Citrobacter portucalensis
ZapB | CapCone-1 | PSBP-like

WP_152082909.1
Enterobacter oligotrophicus
ZapB | CapCone-1 | PSBP-like

WP_036837749.1
Photorhabdus temperata subsp. temperata Meg1
CapCone-1 | PSBP-like | T6SS_HCP

WP_036842281.1
Photorhabdus khanii NC19
CapCone-1 | PSBP-like | PSBP-like | T6SS_HCP

WP_115459255.1
Enterobacillus tribolii
CapCone-1 | PSBP-like

WP_151056740.1
Nitrincola iocasae
CapCone-1 | PSBP-like

WP_077335186.1
Vibrio ruber DSM 16370
T6SS_HCP | CapCone-1 | PSBP-like

WP_201879068.1
Pectobacterium carotovorum
T6SS_HCP | CapCone-1 | PSBP-like

WP_142929284.1
Exilibacterium tricleocarpae
CapCone-1 | PSBP-like

WP_151032335.1
Cellvibrio sp. KY-GH-1
CapCone-1 | PSBP-like

HAU5516689.1
Cronobacter sakazakii
CapCone-1 | PSBP-like

SQK72589.1
Tatumella ptyseos
CapCone-1 | PSBP-like

VEI70089.1
Serratia rubidaea
CapCone-1 | PSBP-like

WP_051910612.1
Pectobacterium brasiliense
CapCone-1 | PSBP-like

WP_119629387.1
Methylocaldum marinum
CapCone-1(frag) | PSBP-like

MBP2196907.1
CapCone-1(frag) | CapCone-1(frag) | PSBP-like

CEK24092.1
Xenorhabdus nematophila AN6/1
CapCone-1(frag) | PSBP-like | T6SS_HCP

WP_233459377.1
Citrobacter sedlakii
ZapB | ? | CapCone-1(frag) | PSBP-like

MBG0749894.1
Pectobacterium carotovorum subsp. carotovorum PCCS1
CapCone-1(frag) | PSBP-like

WP_233596269.1
Citrobacter braakii
ZapB | CapCone-1(frag) | PSBP-like

WP_233623586.1
Citrobacter braakii
ZapB | CapCone-1(frag) | PSBP-like

WP_197085556.1
Cellvibrio sp. pealriver
CapCone-1(frag) | PSBP-like

WP_039054647.1
Enterobacter sp. Bisph1
CapCone-1(frag) | PSBP-like | T6SS_HCP

NLY35038.1
Alcaligenaceae bacterium
CapCone-1(frag) | PSBP-like

WP_151586730.1
Leclercia adecarboxylata
ZapB | CapCone-1(frag) | PSBP-like

WP_186370534.1
Pseudescherichia sp.
CapCone-1(frag) | PSBP-like

WP_139109734.1
Kosakonia oryziphila
CapCone-1(frag) | PSBP-like

EOS93040.1
Erwinia tracheiphila PSU-1
CapCone-1(frag) | ? | PSBP-like

WP_225586417.1
Pectobacterium versatile
CapCone-1(frag) | PSE | ? | PSBP-like

BBA33855.1
Methylocaldum marinum
CapCone-1(frag) | ? | ? | PSBP-like

OXU10053.1
Citrobacter braakii
CapCone-1(frag) | PSBP-like

##########;Cystastin-FD-mediated toxin deployment systems

WP_092136737.1
Bizionia paragorgiae
Cystastin-FD | CapCone-1

WP_198913931.1
Tenacibaculum finnmarkense
Cystastin-FD | CapCone-1 | BamE-like-Imm

WP_200778676.1
Flavobacterium segetis
Cystastin-FD | CapCone-1 | BamE-like-Imm

MBS3739284.1
Psychroflexus sp.
Cystastin-FD | CapCone-1

##########;rare architectural and neighborhood arrangements; ####;CapCone-1 domains featuring an unusual helical insert, predicted to play a possible role in deployment

WP_142079277.1
Roseinatronobacter monicus
DUF4506-conflict-helix-ins

WP_150526510.1
Roseibium sediminis
DUF4506-conflict-helix-ins

MBC8130556.1
Rhizobiaceae bacterium
DUF4506-conflict-helix-ins

WP_182417455.1
Bartonella sp. HY038
T6SS_HCP | ? | DUF4506-conflict-helix-ins

WP_150522800.1
Roseibium sediminis
DUF4506-conflict-helix-ins

WP_147850705.1
Vineibacter terrae
DUF4506-conflict-helix-hairpin-ins

####;prominent upstream hairpin fusion

MCC6601799.1
Anaerolineae bacterium
hairpin | CapCone-1

MBI1299820.1
bacterium
hairpin | CapCone-1

MBE7528942.1
Ardenticatenaceae bacterium
hairpin | CapCone-1

MBE2199188.1
Anaerolinea sp.
hairpin | CapCone-1

MCB9421154.1
Ardenticatenaceae bacterium
hairpin | CapCone-1

MCB0005439.1
Anaerolineales bacterium
hairpin | CapCone-1

NJN54067.1
Anaerolineae bacterium
hairpin | CapCone-1

MCA9901807.1
Anaerolineales bacterium
hairpin | CapCone-1

MCB8987325.1
Ardenticatenaceae bacterium
hairpin | CapCone-1

MBK8930791.1
Candidatus Leptovillus affinis
hairpin | CapCone-1

MBK8987259.1
Candidatus Leptovillus gracilis
hairpin | CapCone-1

MBX7237457.1
Caldilineales bacterium
hairpin | CapCone-1

MCB0035274.1
Anaerolineales bacterium
hairpin | CapCone-1

MCB0000097.1
Anaerolineales bacterium
hairpin | CapCone-1

####;IG-like domain fusion

PIE47958.1
Flavobacteriales bacterium
IG-like | CapCone-1

WP_188663739.1
IG-like | CapCone-1

WP_183424343.1
Luteibacter sp. Sphag1AF
IG-like | CapCone-1

####;LysM C-terminal domain fusion

MBT9557233.1
Myxococcales bacterium
CapCone-1 | LysM

####;TPR repeats fusion

UCH95465.1
Candidatus Aminicenantes bacterium
TPR-repeats | CapCone-1

HEU32995.1
TPR-repeats | CapCone-1

MBN2089357.1
Candidatus Zhuqueibacterota bacterium
TPR-repeats | CapCone-1

NIM96513.1
Candidatus Zixiibacteriota bacterium
TPR-repeats | CapCone-1

####;unknown N-terminal domain fusion

MBK7098056.1
Sphingobacteriales bacterium
unknown7-Nterm | CapCone-1

MBR2649842.1
Sediminibacterium sp.
unknown7-Nterm | CapCone-1

#;PGBD fusion

UCF06526.1
bacterium
PGBD | CapCone-1

###########;standalone versions on the genome

RPD94046.1
Aureibaculum marinum
CapCone-1

TVQ50982.1
Saprospirales bacterium
CapCone-1

MBL56269.1
Flavobacteriales bacterium
CapCone-1

WP_162442277.1
Rhodocytophaga rosea
CapCone-1

MBI2605685.1
Deltaproteobacteria bacterium
CapCone-1

MCA9060762.1
Planctomycetaceae bacterium
CapCone-1

WP_159296572.1
Tenacibaculum maritimum
CapCone-1

WP_232736598.1
Salmonella enterica subsp. enterica serovar Kentucky
CapCone-1

WP_192811333.1
Paracidovorax oryzae
CapCone-1

EHD06974.1
Salmonella enterica subsp. enterica serovar Urbana str. R8-2977
CapCone-1

WP_052816090.1
Agrobacterium rubi
CapCone-1

RZL85854.1
Sphingomonas sp.
CapCone-1

WP_071898495.1
Cystobacter ferrugineus
CapCone-1

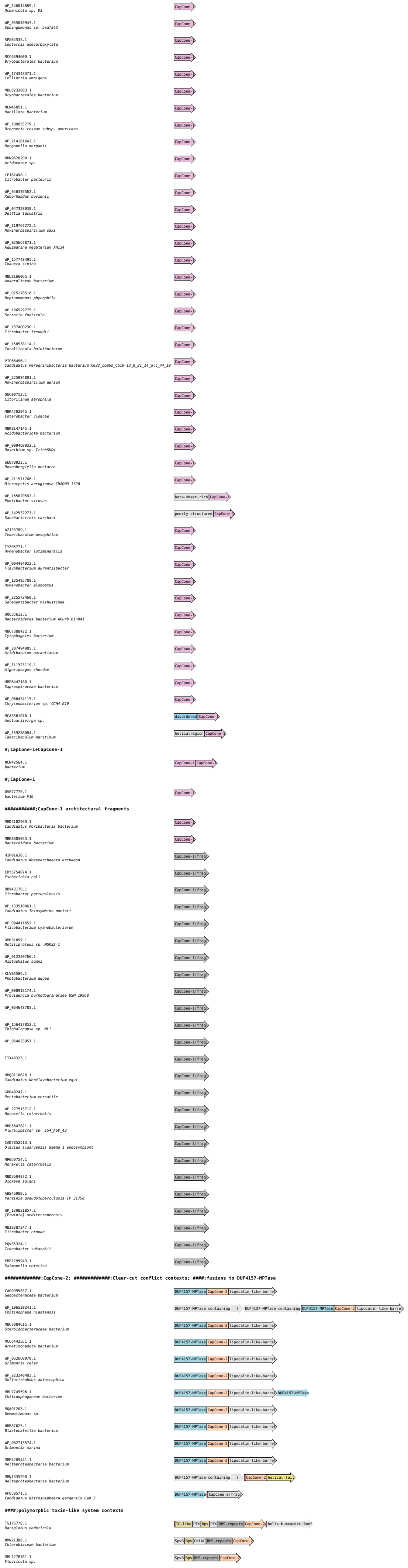
WP_140014689.1
Oceanicola sp. D3
CapCone-1
WP_055840943.1
Sphingomonas sp. Leaf343
CapCone-1
SPX64535.1
Leclercia adecarboxylata
CapCone-1
MCC6394609.1
Bryobacterales bacterium
CapCone-1
WP_174343371.1
Lelliottia amnigena
CapCone-1
MBL8233083.1
Bryobacterales bacterium
CapCone-1
NLW46851.1
Bacillota bacterium
CapCone-1
WP_109055779.1
Brenneria roseae subsp. americana
CapCone-1
WP_214181643.1
Morganella morganii
CapCone-1
MBN9626200.1
Acidovorax sp.
CapCone-1
CEJ67498.1
Citrobacter pasteurii
CapCone-1
WP_046336562.1
Xenorhabdus bovienii
CapCone-1
WP_047328038.1
Delftia lacustris
CapCone-1
WP_119767272.1
Noviherbaspirillum saxi
CapCone-1
WP_025667871.1
Aquimarina megaterium XH134
CapCone-1
WP_157748495.1
Thauera sinica
CapCone-1
MBL8166065.1
Anaerolineae bacterium
CapCone-1
WP_075178516.1
Neptunomonas phycophila
CapCone-1
WP_186529775.1
Serratia fonticola
CapCone-1
WP_137400230.1
Citrobacter freundii
CapCone-1
WP_158538114.1
Corallincola holothuriorum
CapCone-1
PIP66456.1
Candidatus Peregrinibacteria bacterium CG22_combo_CG10-13_8_21_14_all_44_10
CapCone-1
WP_225984801.1
Noviherbaspirillum aerium
CapCone-1
OUC09712.1
Litorilinea aerophila
CapCone-1
MBK4703945.1
Enterobacter cloacae
CapCone-1
MBK8147145.1
Acidobacteriota bacterium
CapCone-1
WP_009468932.1
Roseibium sp. TrichSKD4
CapCone-1
SEQ78922.1
Rosenbergiella nectarea
CapCone-1
WP_211571766.1
Microcystis aeruginosa CHAOHU 1326
CapCone-1
WP_165820592.1
Pontibacter virosus
beta-sheet-rich
CapCone-1
WP_142532273.1
Saccharicrinis carchari
poorly-structured
CapCone-1
AZJ33789.1
Tenacibaculum mesophilum
CapCone-1
TYZ05771.1
Hymenobacter lutimineralis
CapCone-1
WP_094484922.1
Flavobacterium aurantiibacter
CapCone-1
WP_135495708.1
Hymenobacter elongatus
CapCone-1
WP_225572468.1
Salegentibacter mishustinae
CapCone-1
OQC35612.1
Bacteroidetes bacterium ADurb.Bin041
CapCone-1
MBC7388422.1
Cytophagales bacterium
CapCone-1
WP_207494085.1
Aridibaculum aurantiacum
CapCone-1
WP_111323119.1
Algoriphagus chordae
CapCone-1
MBP6447180.1
Saprospiraceae bacterium
CapCone-1
WP_066434115.1
Chryseobacterium sp. CCH4-E10
CapCone-1
MCA3561076.1
Aestuariivirga sp.
disordered
CapCone-1
WP_159288084.1
Tenacibaculum maritimum
helicalregion
CapCone-1
#;CapCone-1+CapCone-1
NCN42564.1
bacterium
CapCone-1
CapCone-1
#;CapCone-1
OVE77770.1
bacterium F16
CapCone-1
###########;CapCone-1 architectural fragments
MBD3182969.1
Candidatus Poribacteria bacterium
CapCone-1
MBK8685053.1
Bacteroidota bacterium
CapCone-1
HIH91638.1
Candidatus Woesearchaeota archaeon
CapCone-1(frag
EHY3754074.1
Escherichia coli
CapCone-1(frag
BBV43170.1
Citrobacter portucalensis
CapCone-1(frag
WP_133510061.1
Candidatus Thiosymbion oneisti
CapCone-1(frag
WP_094411652.1
Flavobacterium cyanobacteriorum
CapCone-1(frag
OMH31857.1
Motiliproteus sp. MSK22-1
CapCone-1(frag
WP_012340766.1
Histophilus somni
CapCone-1(frag
KLV05586.1
Photobacterium aquae
CapCone-1(frag
WP_008913174.1
Providencia burhodogranariea DSM 19968
CapCone-1(frag
WP_064648783.1
CapCone-1(frag
WP_156427853.1
Thiohalocapsa sp. ML1
CapCone-1(frag
WP_064623957.1
CapCone-1(frag
TIX48325.1
CapCone-1(frag
MBQ0116628.1
Candidatus Neoflavobacterium equi
CapCone-1(frag
GBO49247.1
Pectobacterium versatile
CapCone-1(frag
WP_227513712.1
Moraxella catarrhalis
CapCone-1(frag
MBU5647821.1
Pluralibacter sp. S54_ASV_43
CapCone-1(frag
CAD7852513.1
Olavius algarvensis Gamma 1 endosymbiont
CapCone-1(frag
MPW59754.1
Moraxella catarrhalis
CapCone-1(frag
MBD3604872.1
Dickeya solani
CapCone-1(frag
ABS46908.1
Yersinia pseudotuberculosis IP 31758
CapCone-1(frag
WP_130831857.1
[Erwinia] mediterraneensis
CapCone-1(frag
MBJ8387247.1
Citrobacter cronae
CapCone-1(frag
PQV85324.1
Cronobacter sakazakii
CapCone-1(frag
EBF1295943.1
Salmonella enterica
CapCone-1(frag
#############;CapCone-2; #############;Clear-cut conflict contexts; ####;fusions to DUF4157-MPTase
CAG0995837.1
Geobacteraceae bacterium
DUF4157-MPTase
CapCone-2
lipocalin-like-barrel
WP_106530241.1
Chitinophaga niastensis
DUF4157-MPTase-containing
?
DUF4157-MPTase-containing
DUF4157-MPTase
CapCone-2
lipocalin-like-barrel
MBC7984615.1
Steroidobacteraceae bacterium
DUF4157-MPTase
CapCone-2
lipocalin-like-barrel
MCC6443551.1
Armatimonadota bacterium
DUF4157-MPTase
CapCone-2
lipocalin-like-barrel
WP_062660970.1
Grimontia celer
DUF4157-MPTase
CapCone-2
lipocalin-like-barrel
WP_223248483.1
Sulfurirhabdus autotrophica
DUF4157-MPTase
CapCone-2
lipocalin-like-barrel
MBL7749596.1
Chitinophagaceae bacterium
DUF4157-MPTase
CapCone-2
lipocalin-like-barrel
DUF4157-MPTase
MQA91203.1
Gemmatimonas sp.
DUF4157-MPTase
CapCone-2
lipocalin-like-barrel
HBB87625.1
Blastocatellia bacterium
DUF4157-MPTase
CapCone-2
lipocalin-like-barrel
WP_062713574.1
Grimontia marina
DUF4157-MPTase
CapCone-2
lipocalin-like-barrel
MBM4289441.1
Deltaproteobacteria bacterium
DUF4157-MPTase
CapCone-2
lipocalin-like-barrel
MBN1335290.1
Deltaproteobacteria bacterium
DUF4157-MPTase-containing
?
CapCone-2
helical-tail
AFU58571.1
Candidatus Nitrososphaera gargensis Ga9.2
DUF4157-MPTase
CapCone-2(frag
####;polymorphic toxin-like system contexts
TSJ76770.1
Rariglobus hedericola
IG-like
PTX
Bps
PTX
RHS-repeats
CapCone-2
helix-b-meander-Imm?
NMW21388.1
Chlorobiaceae bacterium
SpvB
Bps
CdiAC
RHS-repeats
CapCone-2
MBL1278781.1
Fluviicola sp.
SpvB
Bps
RHS-repeats
CapCone-2

Accession	Organism	Gene neighborhood
KAF5429684.1	*Candidatus Methanophagaceae archaeon*	RHS-repeats, CapCone-2
MBC8372044.1	*Planctomycetota bacterium*	RHS-repeats, CapCone-2
MBI9019438.1	*Phycisphaerae bacterium*	RHS-repeats, CapCone-2
MBL8820661.1	*Planctomyces sp.*	RHS-repeats, CapCone-2
RYD85565.1	*Verrucomicrobiaceae bacterium*	IG-like, RHS-repeats, CapCone-2 / helix-b-meander-Imm? / ? / RHS-repeat-containing
TWT84381.1	*Novipirellula herctigrandis*	RHS-repeats, CapCone-2 / helix-b-meander-Imm

####;association with small, secreted protein with helix and beta-meander, a subset of which contain 4 conserved cysteine residues. Potential secreted toxin with membrane-anchored immunity protein pairing, frequently observed in SAW clade systems

Accession	Organism	Gene neighborhood
MCC7441605.1	*Bdellovibrionales bacterium*	CapCone-2, helical-tail / helix-b-meander-Imm
MBX3017701.1	*Pseudobdellovibrionaceae bacterium*	CapCone-2, helical-tail / helix-b-meander-Imm
OFZ36824.1	*Bdellovibrionales bacterium RIFOXYB1_FULL_39_21*	CapCone-2, helical-tail / helix-b-meander-Imm
MAV90968.1	*Pseudobdellovibrionaceae bacterium*	CapCone-2, helical-tail / helix-b-meander-Imm
MCC6137651.1	*Pseudobdellovibrionaceae bacterium*	CapCone-2, helical-tail / helix-b-meander-Imm
RME17803.1	*Bdellovibrio sp.*	CapCone-2, helical-tail / helix-b-meander-Imm
WP_063208951.1	*Bdellovibrio bacteriovorus*	CapCone-2, helical-tail / helix-b-meander-Imm
MBL7555126.1	*Pseudobdellovibrionaceae bacterium*	CapCone-2, helical-tail / helix-b-meander-Imm
KYG65510.1	*Bdellovibrio bacteriovorus*	CapCone-2, helical-tail / helix-b-meander-Imm
NOT77854.1	*Bacteriovoracaceae bacterium*	CapCone-2, helical-tail / helix-b-meander-Imm
WP_088565924.1	*Bdellovibrio bacteriovorus*	CapCone-2, helical-tail / helix-b-meander-Imm
MCB0361366.1	*Bdellovibrionales bacterium*	CapCone-2, helical-tail / helix-b-meander-Imm
WP_115362598.1	*Halobacteriovorax sp. Y22*	CapCone-2, helical-tail / helix-b-meander-Imm
WP_021266995.1	*Bacteriovorax sp. BAL6_X*	helix-b-meander-Imm / CapCone-2, helical-tail
WP_103217241.1	*Halobacteriovorax sp. DA5*	helix-b-meander-Imm / CapCone-2, helical-tail
MCC7402765.1	*Bdellovibrionales bacterium*	CapCone-2, helical-tail / helix-b-meander-Imm
MCB0365783.1	*Bdellovibrionales bacterium*	CapCone-2, helical-tail / helix-b-meander-Imm
MBO9667284.1	*Bdellovibrio sp.*	disordered, CapCone-2 / helix-b-meander-Imm
OUR93658.1	*Halobacteriovorax marinus*	CapCone-2, helical-tail2 / helix-b-meander-Imm
OUR96354.1	*Halobacteriovorax marinus*	CapCone-2, helical-tail2 / helix-b-meander-Imm
WP_021276812.1	*Bacteriovorax sp. DB6_IX*	CapCone-2, helical-tail2 / helix-b-meander-Imm

#;direct fusion to the domain

Accession	Organism	Gene neighborhood
NOZ94751.1	*Acidobacteriota bacterium*	CapCone-2, b-meander
MBT5095663.1	*Halobacteriovoraceae bacterium*	CapCone-2, b-meander
MBD3871094.1	*Candidatus Sulfomarinibacter kjeldsenii*	CapCone-2, b-meander

##########;contexts featuring the CapCone-2 Lipocone in two-gene islands. Possible connection to the extended, C-terminal and predicted cytoplasmic helical-tail; ####;direct Lipocone fusion to ANK repeats while adjacent to membrane-anchored prokaryotic SAS-6_N-like domain

Accession	Organism	Gene neighborhood
TDJ58642.1	*Planctomycetota bacterium*	CapCone-2, ANKs / SAS-6_N-like
HGO74050.1		CapCone-2, ANKs / SAS-6_N-like
NQY92227.1	*Deltaproteobacteria bacterium*	CapCone-2, ANKs / SAS-6_N-like
MCB9844774.1	*Phycisphaeraceae bacterium*	CapCone-2, ANKs / SAS-6_N-like
UCF33030.1	*Phycisphaerales bacterium*	CapCone-2, ANKs / SAS-6_N-like
MBS1135087.1	*Burkholderiaceae bacterium*	CapCone-2, ANKs / SAS-6_N-like
MBX3644499.1	*Rubrivivax sp.*	CapCone-2, ANKs / SAS-6_N-like
QNN24147.1	*Planctomycetales bacterium ZRK34*	CapCone-2, ANKs / SAS-6_N-like
MBI1827638.1	*Planctomycetota bacterium*	CapCone-2, ANKs / SAS-6_N-like

#;fusion only

Accession	Organism	Gene neighborhood
MBA3273469.1	*Chthoniobacterales bacterium*	CapCone-2, ANKs
HHS14785.1		CapCone-2, ANKs
MCB9865247.1	*Phycisphaerales bacterium*	CapCone-2, ANKs
KPK04435.1	*Betaproteobacteria bacterium SG8_39*	CapCone-2, ANKs

####;association with OmpA-like-beta-barrel

Accession	Organism	Gene neighborhood
MCC7442396.1	*Bdellovibrionales bacterium*	CapCone-2, helical-tail / OmpA-like-beta-barrel
MBM4382838.1	*Deltaproteobacteria bacterium*	OmpA-like-beta-barrel / CapCone-2, helical-tail
RMG42956.1	*Acidobacteriota bacterium*	OmpA-like-beta-barrel / CapCone-2, helical-tail
MYA07395.1	*Holophagales bacterium*	OmpA-like-beta-barrel / CapCone-2, helical-tail
NNE06787.1	*Lysobacterales bacterium*	OmpA-like-beta-barrel / CapCone-2, helical-tail
NIV17657.1	*Woeseiaceae bacterium*	OmpA-like-beta-barrel / CapCone-2, helical-tail
NIM00358.1	*Acidobacteriota bacterium*	OmpA-like-beta-barrel / CapCone-2, helical-tail
MSQ98623.1	*Lysobacterales bacterium*	OmpA-like-beta-barrel / CapCone-2, helical-tail
NNL00071.1	*Lysobacterales bacterium*	OmpA-like-beta-barrel / CapCone-2, helical-tail
MBL0039650.1	*Lysobacterales bacterium*	OmpA-like-beta-barrel / CapCone-2, helical-tail
MBP7418754.1	*Lysobacterales bacterium*	OmpA-like-beta-barrel / CapCone-2, helical-tail
MCB1565069.1	*Lysobacterales bacterium*	OmpA-like-beta-barrel / CapCone-2, helical-tail

#;direct fusion to OmpA-like-beta-barrel

Accession	Organism	Gene neighborhood
WP_203407488.1	*Archangium violaceum*	CapCone-2, OmpA-like-beta-barrel
WP_204221556.1	*Archangium violaceum*	CapCone-2, OmpA-like-beta-barrel

####;alpha/beta-hydrolase association

Accession	Organism	Gene neighborhood
NOT80751.1	*Bacteriovoracaceae bacterium*	ABhydrolase / CapCone-2, helical-tail
NIV18685.1	*Woeseiaceae bacterium*	ABhydrolase / CapCone-2, helical-tail
MBH47743.1	*Halobacteriovorax sp.*	CapCone-2, helical-tail / ABhydrolase
MBF0315225.1	*Oligoflexia bacterium*	CapCone-2, helical-tail / ABhydrolase
MBF0363682.1	*Oligoflexia bacterium*	CapCone-2, helical-tail / ABhydrolase
REJ72939.1	*Acidobacteriota bacterium*	ABhydrolase / CapCone-2, helical-tail

#;direct fusion

Accession	Organism	Gene neighborhood
WP_021272296.1	*Bacteriovorax sp. BSW11_IV*	CapCone-2, ABhydrolase

####;OB-fold-containing domain association

Accession	Organism	Gene neighborhood
APR67620.1	*Thalassolituus oleivorans*	CapCone-2, helical-tail / DUF2835-OB
ASP39613.1	*Bacterioplanes sanyensis*	CapCone-2, helical-tail / DUF2835-OB
WP_225675410.1	*Thalassolituus marinus*	CapCone-2, helical-tail / DUF2835-OB
TNC86595.1	*Thalassolituus sp.*	CapCone-2, helical-tail / DUF2835-OB
PHR18847.1	*Sphingopyxis sp.*	CapCone-2, helical-tail / DUF2835-OB

####;association iwth Acyltransferase+TM+TM+TM protein

Accession	Organism	Gene neighborhood
MBT7524685.1	*Fidelibacterota bacterium*	CapCone-2, helical-tail / Acyltransferase
MBC8345682.1	*Fidelibacterota bacterium*	CapCone-2, helical-tail / Acyltransferase
RTZ73493.1	*Fidelibacterota bacterium*	CapCone-2, helical-tail / Acyltransferase
MBC8323274.1	*Fidelibacterota bacterium*	CapCone-2, helical-tail / Acyltransferase
MBT3591041.1	*Fidelibacterota bacterium*	CapCone-2, helical-tail / Acyltransferase
MBT3479113.1	*Fidelibacterota bacterium*	CapCone-2, helical-tail / Acyltransferase
HIN02586.1	*Fidelibacterota bacterium*	CapCone-2, helical-tail / Acyltransferase

#############;other direct fusions; ####;lipocalin-like-barrel

Accession	Organism	Gene neighborhood
WP_152502315.1	*Labrenzia sp. THAF82*	CapCone-2, lipocalin-like-barrel
WP_055678723.1		CapCone-2, lipocalin-like-barrel
MBO6892403.1	*Roseibium sp.*	CapCone-2, lipocalin-like-barrel

####;IG-like

Accession	Organism	Gene neighborhood
MAR09117.1	*Blastopirellula sp.*	CapCone-2, IG-like

####;Patatin

Accession	Organism	Gene neighborhood
RLD34100.1	*Bacteroidota bacterium*	CapCone-2, Patatin
RKX83697.1	*Spirochaetota bacterium*	CapCone-2, Patatin

#############;standalone versions

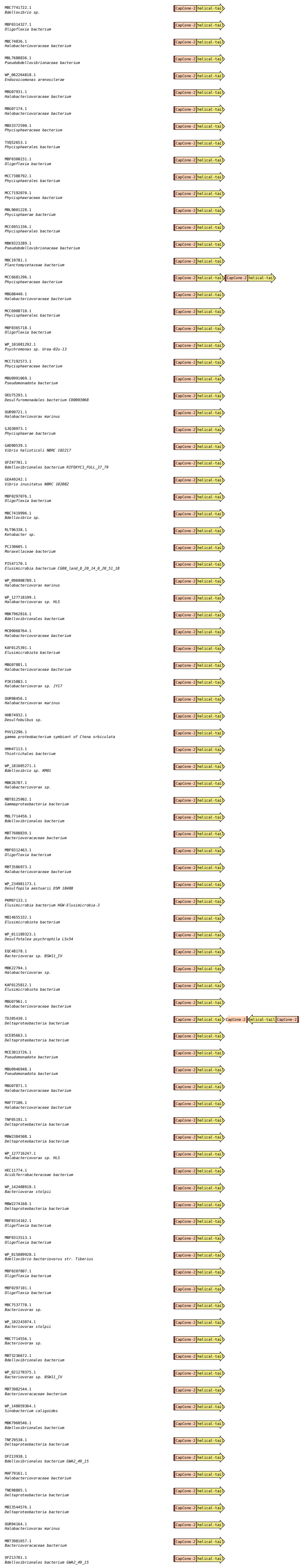

MBC7741722.1
Bdellovibrio sp.
CapCone-2 helical-tai
MBF0314327.1
Oligoflexia bacterium
CapCone-2 helical-tai
MBC74836.1
Halobacteriovoraceae bacterium
CapCone-2 helical-tai
MBL7688836.1
Pseudobdellovibrionaceae bacterium
CapCone-2 helical-tai
WP_062264818.1
Endozoicomonas arenosclerae
CapCone-2 helical-tai
MBG07931.1
Halobacteriovoraceae bacterium
CapCone-2 helical-tai
MBG07174.1
Halobacteriovoraceae bacterium
CapCone-2 helical-tai
MBX3372590.1
Phycisphaeraceae bacterium
CapCone-2 helical-tai
TVQ52653.1
Phycisphaerales bacterium
CapCone-2 helical-tai
MBF0300231.1
Oligoflexia bacterium
CapCone-2 helical-tai
MCC7388792.1
Phycisphaerales bacterium
CapCone-2 helical-tai
MCC7192070.1
Phycisphaeraceae bacterium
CapCone-2 helical-tai
MBL9001228.1
Phycisphaerae bacterium
CapCone-2 helical-tai
MCC6951336.1
Phycisphaerales bacterium
CapCone-2 helical-tai
MBK9323289.1
Pseudobdellovibrionaceae bacterium
CapCone-2 helical-tai
MBC19781.1
Planctomycetaceae bacterium
CapCone-2 helical-tai
MCC6681296.1
Phycisphaeraceae bacterium
CapCone-2 helical-tai CapCone-2 helical-tai
MBG08440.1
Halobacteriovoraceae bacterium
CapCone-2 helical-tai
MCC6908710.1
Phycisphaerales bacterium
CapCone-2 helical-tai
MBF0365718.1
Oligoflexia bacterium
CapCone-2 helical-tai
WP_101081292.1
Psychromonas sp. Urea-02u-13
CapCone-2 helical-tai
MCC7192573.1
Phycisphaeraceae bacterium
CapCone-2 helical-tai
MBU0991069.1
Pseudomonadota bacterium
CapCone-2 helical-tai
OEU75293.1
Desulfuromonadales bacterium C00003068
CapCone-2 helical-tai
OUR99721.1
Halobacteriovorax marinus
CapCone-2 helical-tai
GJQ30973.1
Phycisphaerae bacterium
CapCone-2 helical-tai
GAD90539.1
Vibrio halioticoli NBRC 102217
CapCone-2 helical-tai
OFZ47701.1
Bdellovibrionales bacterium RIFOXYC1_FULL_37_79
CapCone-2 helical-tai
GEA49242.1
Vibrio inusitatus NBRC 102082
CapCone-2 helical-tai
MBF0297076.1
Oligoflexia bacterium
CapCone-2 helical-tai
MBC7419996.1
Bdellovibrio sp.
CapCone-2 helical-tai
RLT96338.1
Ketobacter sp.
CapCone-2 helical-tai
PCJ30605.1
Moraxellaceae bacterium
CapCone-2 helical-tai
PIS47170.1
Elusimicrobia bacterium CG08_land_8_20_14_0_20_51_18
CapCone-2 helical-tai
WP_096908789.1
Halobacteriovorax marinus
CapCone-2 helical-tai
WP_127718199.1
Halobacteriovorax sp. HLS
CapCone-2 helical-tai
MBK7962816.1
Bdellovibrionales bacterium
CapCone-2 helical-tai
MCB9060764.1
Halobacteriovoraceae bacterium
CapCone-2 helical-tai
KAF0125391.1
Elusimicrobiota bacterium
CapCone-2 helical-tai
MBG07881.1
Halobacteriovoraceae bacterium
CapCone-2 helical-tai
PIK15083.1
Halobacteriovorax sp. JY17
CapCone-2 helical-tai
OUR98456.1
Halobacteriovorax marinus
CapCone-2 helical-tai
HHB74932.1
Desulfobulbus sp.
CapCone-2 helical-tai
PVV12296.1
gamma proteobacterium symbiont of Ctena orbiculata
CapCone-2 helical-tai
HHH47113.1
Thiotrichales bacterium
CapCone-2 helical-tai
WP_181605271.1
Bdellovibrio sp. KM01
CapCone-2 helical-tai
MBK26707.1
Halobacteriovorax sp.
CapCone-2 helical-tai
MBT8125902.1
Gammaproteobacteria bacterium
CapCone-2 helical-tai
MBL7714456.1
Bdellovibrionales bacterium
CapCone-2 helical-tai
MBT7608839.1
Bacteriovoracaceae bacterium
CapCone-2 helical-tai
MBF0312463.1
Oligoflexia bacterium
CapCone-2 helical-tai
MBT3586973.1
Halobacteriovoraceae bacterium
CapCone-2 helical-tai
WP_234981173.1
Desulfopila aestuarii DSM 18488
CapCone-2 helical-tai
PKM97133.1
Elusimicrobia bacterium HGW-Elusimicrobia-3
CapCone-2 helical-tai
MBI4655332.1
Elusimicrobiota bacterium
CapCone-2 helical-tai
WP_011189323.1
Desulfotalea psychrophila LSv54
CapCone-2 helical-tai
EQC48178.1
Bacteriovorax sp. BSW11_IV
CapCone-2 helical-tai
MBK22794.1
Halobacteriovorax sp.
CapCone-2 helical-tai
KAF0125812.1
Elusimicrobiota bacterium
CapCone-2 helical-tai
MBG07961.1
Halobacteriovoraceae bacterium
CapCone-2 helical-tai
TDJ05430.1
Deltaproteobacteria bacterium
CapCone-2 helical-tai CapCone-2 helical-tail CapCone-2
UCE85663.1
Deltaproteobacteria bacterium
CapCone-2 helical-tai
MCE3013726.1
Pseudomonadota bacterium
CapCone-2 helical-tai
MBU0946948.1
Pseudomonadota bacterium
CapCone-2 helical-tai
MBG07871.1
Halobacteriovoraceae bacterium
CapCone-2 helical-tai
MAF77106.1
Halobacteriovoraceae bacterium
CapCone-2 helical-tai
TNF05191.1
Deltaproteobacteria bacterium
CapCone-2 helical-tai
MBW2384508.1
Deltaproteobacteria bacterium
CapCone-2 helical-tai
WP_127716247.1
Halobacteriovorax sp. HLS
CapCone-2 helical-tai
HEC11774.1
Acidiferrobacteraceae bacterium
CapCone-2 helical-tai
WP_142408918.1
Bacteriovorax stolpii
CapCone-2 helical-tai
MBW2274168.1
Deltaproteobacteria bacterium
CapCone-2 helical-tai
MBF0314162.1
Oligoflexia bacterium
CapCone-2 helical-tai
MBF0313513.1
Oligoflexia bacterium
CapCone-2 helical-tai
WP_015089928.1
Bdellovibrio bacteriovorus str. Tiberius
CapCone-2 helical-tai
MBF0207807.1
Oligoflexia bacterium
CapCone-2 helical-tai
MBF0297101.1
Oligoflexia bacterium
CapCone-2 helical-tai
MBC7537778.1
Bacteriovorax sp.
CapCone-2 helical-tai
WP_102243074.1
Bacteriovorax stolpii
CapCone-2 helical-tai
MBC7714556.1
Bacteriovorax sp.
CapCone-2 helical-tai
MBT3236672.1
Bdellovibrionales bacterium
CapCone-2 helical-tai
WP_021270375.1
Bacteriovorax sp. BSW11_IV
CapCone-2 helical-tai
MBT3982544.1
Bacteriovoracaceae bacterium
CapCone-2 helical-tai
WP_148059364.1
Sinobacterium caligoides
CapCone-2 helical-tai
MBK7960546.1
Bdellovibrionales bacterium
CapCone-2 helical-tai
TNF29538.1
Deltaproteobacteria bacterium
CapCone-2 helical-tai
OFZ13938.1
Bdellovibrionales bacterium GWA2_49_15
CapCone-2 helical-tai
MAF79161.1
Halobacteriovoraceae bacterium
CapCone-2 helical-tai
TNE98805.1
Deltaproteobacteria bacterium
CapCone-2 helical-tai
MBI3544576.1
Deltaproteobacteria bacterium
CapCone-2 helical-tai
OUR94164.1
Halobacteriovorax marinus
CapCone-2 helical-tai
MBT3981657.1
Bacteriovoracaceae bacterium
CapCone-2 helical-tai
OFZ13701.1
Bdellovibrionales bacterium GWA2_49_15
CapCone-2 helical-tai

PIP88943.1	*Bdellovibrionales bacterium CG22_combo_CG10-13_8_21_14_all_38_13*	CapCone-2	helical-tai
MBH48325.1	*Halobacteriovorax sp.*	CapCone-2	helical-tai
MBC7537654.1	*Bacteriovorax sp.*	CapCone-2	helical-tai
MBT6326615.1	*Bdellovibrionales bacterium*	CapCone-2	helical-tai
OUR97159.1	*Halobacteriovorax marinus*	CapCone-2	helical-tai
OUR97237.1	*Halobacteriovorax marinus*	CapCone-2	helical-tai
WP_127715994.1	*Halobacteriovorax sp. HLS*	CapCone-2	helical-tai
MBT3980786.1	*Bacteriovoracaceae bacterium*	CapCone-2	helical-tai
MBF0360411.1	*Oligoflexia bacterium*	CapCone-2	helical-tai
MBT3982371.1	*Bacteriovoracaceae bacterium*	CapCone-2	helical-tai
OFZ16030.1	*Bdellovibrionales bacterium GWA2_49_15*	CapCone-2	helical-tai
OFZ16272.1	*Bdellovibrionales bacterium GWA2_49_15*	CapCone-2	helical-tai
OUR97758.1	*Halobacteriovorax marinus*	CapCone-2	helical-tai
OFZ13148.1	*Bdellovibrionales bacterium GWA2_49_15*	CapCone-2	helical-tai
RTZ76144.1	*Gammaproteobacteria bacterium*	CapCone-2	helical-tai
MBK24803.1	*Halobacteriovorax sp.*	CapCone-2	helical-tai
MBI2606046.1	*Deltaproteobacteria bacterium*	CapCone-2	helical-tai
TDJ06949.1	*Deltaproteobacteria bacterium*	CapCone-2	helical-tai
MBD66675.1	*Halobacteriovoraceae bacterium*	CapCone-2	helical-tai
MBL4818154.1	*Deltaproteobacteria bacterium*	CapCone-2	helical-tai
HBA59230.1	*Elusimicrobiota bacterium*	CapCone-2	helical-tai
MBT7766741.1	*Bdellovibrionales bacterium*	CapCone-2	helical-tai
PIR36567.1	*Bdellovibrio sp. CG11_big_fil_rev_8_21_14_0_20_39_38*	CapCone-2	helical-tai
MBT6325398.1	*Bdellovibrionales bacterium*	CapCone-2	helical-tai
MBK8971189.1	*Hahellaceae bacterium*	CapCone-2	helical-tai
MBA2494046.1	*Acidobacteriota bacterium*	CapCone-2	helical-tai
MBI3542360.1	*Deltaproteobacteria bacterium*	CapCone-2	helical-tai
MBA2621169.1	*Acidobacteriota bacterium*	CapCone-2	helical-tai
MCB1036575.1	*Acidobacteriota bacterium*	CapCone-2	helical-tai
KTD07045.1	*Legionella jamestowniensis*	CapCone-2	helical-tai
MCA1625090.1	*Acidobacteriota bacterium*	CapCone-2	helical-tai
MBN2311444.1	*Candidatus Hydrogenedentota bacterium*	CapCone-2	helical-tai
MBI1373479.1	*Phycisphaera sp.*	CapCone-2	helical-tai
QNN24695.1	*Planctomycetales bacterium ZRK34*	CapCone-2	helical-tai
OFZ13313.1	*Bdellovibrionales bacterium GWA2_49_15*	CapCone-2	helical-tai
MBK7890998.1	*Bdellovibrionales bacterium*	CapCone-2	helical-tai
NIP84080.1	*Planctomycetales bacterium*	CapCone-2	helical-tai
MBK7392791.1	*Chloracidobacterium sp.*	CapCone-2	helical-tai
MBI1368749.1	*Planctomycetota bacterium*	CapCone-2	helical-tai
NQU68194.1	*Fidelibacterota bacterium*	CapCone-2	helical-tai
MBA3335444.1	*Acidobacteriota bacterium*	CapCone-2	helical-tai
MBA3599929.1	*Acidobacteriota bacterium*	CapCone-2	helical-tai
OQX18481.1	*Desulfobulbaceae bacterium A2*	CapCone-2	helical-tai
MBX7169389.1	*Pyrinomonadaceae bacterium*	CapCone-2	helical-tai
WP_127716905.1	*Halobacteriovorax sp. HLS*	CapCone-2	helical-tai
MBK9529758.1	*Acidobacteriota bacterium*	CapCone-2	helical-tai
MBK7512707.1	*Chloracidobacterium sp.*	CapCone-2	helical-tai
MBL8617093.1	*Deltaproteobacteria bacterium*	CapCone-2	helical-tai
MBS1795306.1	*Acidobacteriota bacterium*	CapCone-2	helical-tai
MBK8466493.1	*Chloracidobacterium sp.*	CapCone-2	helical-tai
MCD9186178.1	*Pyrinomonadaceae bacterium*	CapCone-2	helical-tai
HHO52315.1	*Deltaproteobacteria bacterium*	CapCone-2	helical-tai
MBW1644625.1	*Deltaproteobacteria bacterium*	CapCone-2	helical-tai
MCC7108743.1	*Deltaproteobacteria bacterium*	CapCone-2	helical-tai
RME46381.1	*Deltaproteobacteria bacterium*	CapCone-2	helical-tai
MBA3536481.1	*Tatlockia sp.*	CapCone-2	helical-tai
RMF84550.1	*Nitrospirota bacterium*	CapCone-2	helical-tai
UCF66807.1	*Acidobacteriota bacterium*	CapCone-2	helical-tai
MBF0298269.1	*Oligoflexia bacterium*	CapCone-2	helical-tai
MBA2650932.1	*Tatlockia sp.*	CapCone-2	helical-tai
HGB40278.1		CapCone-2	helical-tai
MBI4890374.1	*Acidobacteriota bacterium*	CapCone-2	helical-tai
WP_203113849.1	*Legionella bononiensis*	CapCone-2	helical-tai
WP_131793923.1	*Fluoribacter gormanii*	CapCone-2	helical-tai
MBX7258477.1	*Candidatus Hydrogenedentota bacterium*	CapCone-2	helical-tai
MBA2710510.1	*Tatlockia sp.*	CapCone-2	helical-tai
MCC7441343.1	*Bdellovibrionales bacterium*	CapCone-2	helical-tai
MBF0106022.1	*Deltaproteobacteria bacterium*	CapCone-2	helical-tai
RKX85903.1	*Spirochaetota bacterium*	CapCone-2	helical-tai
NJL29127.1	*Thermoanaerobaculia bacterium*	CapCone-2	helical-tai
MBA2648399.1	*Legionella sp.*	CapCone-2	helical-tai
MBL7060164.1	*Fidelibacterota bacterium*	CapCone-2	helical-tai
MCC7072251.1	*Deltaproteobacteria bacterium*	CapCone-2	helical-tai
MBF0361635.1	*Oligoflexia bacterium*	CapCone-2	helical-tai
MCB9688778.1	*Alphaproteobacteria bacterium*	CapCone-2	helical-tai
MBF0314772.1	*Oligoflexia bacterium*	CapCone-2	helical-tai
MBF0363981.1	*Oligoflexia bacterium*	CapCone-2	helical-tai
RLA64279.1	*Campylobacterota bacterium*	CapCone-2	helical-tai
MCB9681432.1	*Alphaproteobacteria bacterium*	CapCone-2	helical-tai
MCB9664970.1	*Alphaproteobacteria bacterium*	CapCone-2	helical-tai
MBN1335350.1	*Deltaproteobacteria bacterium*	CapCone-2	helical-tai
MBG08241.1	*Halobacteriovoraceae bacterium*	CapCone-2	helical-tai
OGQ24958.1	*Deltaproteobacteria bacterium RBG_16_71_12*	CapCone-2	helical-tai
EPZ32613.1	*Rozella allomycis CSF55*	CapCone-2	helical-tai
MCB9674342.1	*Alphaproteobacteria bacterium*	CapCone-2	helical-tai
MBF0361596.1	*Oligoflexia bacterium*	CapCone-2	helical-tai
MBI2423266.1	*Candidatus Hydrogenedentota bacterium*	CapCone-2	helical-tai
MBC7899858.1	*Pyrinomonadaceae bacterium*	CapCone-2	helical-tai
HIJ65649.1	*Candidatus Hydrogenedentota bacterium*	CapCone-2	helical-tai
MCC6142858.1	*Candidatus Hydrogenedentota bacterium*	CapCone-2	helical-tai
WP_142701667.1	*Bdellovibrio sp. ZAP7*	CapCone-2	helical-tai
MYE10189.1	*Gammaproteobacteria bacterium*	CapCone-2	helical-tai
MAW07051.1	*Halobacteriovoraceae bacterium*	CapCone-2	helical-tai

MBG07749.1
Halobacteriovoraceae bacterium
CapCone-2 | helical-tai

MBX3180531.1
Candidatus Hydrogenedentota bacterium
CapCone-2 | helical-tai

MCC7337259.1
Pirellulaceae bacterium
CapCone-2 | helical-tai

WP_102243534.1
Bacteriovorax stolpii
CapCone-2 | helical-tai

MAW07663.1
Halobacteriovoraceae bacterium
CapCone-2 | helical-tai

MBT6325744.1
Bdellovibrionales bacterium
CapCone-2 | helical-tai

MBT6326759.1
Bdellovibrionales bacterium
CapCone-2 | helical-tai

TNE91268.1
Deltaproteobacteria bacterium
CapCone-2 | helical-tai

MBL8236529.1
Bryobacterales bacterium
CapCone-2 | helical-tai

MBD3648323.1
Pseudomonadales bacterium
CapCone-2 | helical-tai

OFZ14731.1
Bdellovibrionales bacterium GWA2_49_15
CapCone-2 | helical-tai

MBF2755659.1
Gammaproteobacteria bacterium AqS3
CapCone-2 | helical-tai

MCC7440608.1
Bdellovibrionales bacterium
CapCone-2 | helical-tai

MBP9595358.1
Fusobacteriaceae bacterium
CapCone-2 | helical-tai

MBQ74622.1
Gammaproteobacteria bacterium
CapCone-2 | helical-tai

MBJ39312.1
Gammaproteobacteria bacterium
CapCone-2 | helical-tai

MBT5441473.1
Gammaproteobacteria bacterium
CapCone-2 | helical-tai

MBC8479096.1
FCB group bacterium
CapCone-2 | helical-tai

OFY98807.1
Bdellovibrionales bacterium GWB1_52_6
CapCone-2 | helical-tai

MCA9570996.1
Myxococcales bacterium
CapCone-2 | helical-tai

OFZ56099.1
Bdellovibrionales bacterium RIFOXYC1_FULL_54_43
CapCone-2 | helical-tai

HEO70060.1
Candidatus Hydrogenedentota bacterium
CapCone-2 | helical-tai

MBM4369565.1
Deltaproteobacteria bacterium
CapCone-2 | helical-tai

MBT3586105.1
Halobacteriovoraceae bacterium
CapCone-2 | helical-tai

MCA9566880.1
Myxococcales bacterium
CapCone-2 | helical-tai

MBC85632.1
Pseudobdellovibrionaceae bacterium
CapCone-2 | helical-tai

KZY96197.1
Oleibacter sp. HI0075
CapCone-2 | helical-tai

MYF31281.1
Gammaproteobacteria bacterium
CapCone-2 | helical-tai

MCA9751105.1
Gemmatimonadota bacterium
CapCone-2 | helical-tai

MBV9217059.1
Acidobacteriota bacterium
CapCone-2 | helical-tai

MBT6326667.1
Bdellovibrionales bacterium
CapCone-2 | helical-tai

MCB1904999.1
Gammaproteobacteria bacterium
CapCone-2 | helical-tai

MCB9060082.1
Halobacteriovoraceae bacterium
CapCone-2 | helical-tai

MBT8435871.1
Gammaproteobacteria bacterium
CapCone-2 | helical-tai

MBM4250652.1
Deltaproteobacteria bacterium
helical | CapCone-2

MBF0442873.1
Oligoflexales bacterium
helical | CapCone-2

MBF0207628.1
Oligoflexia bacterium
CapCone-2

NUN49969.1
Candidatus Brocadiia bacterium
CapCone-2

PYI86971.1
Verrucomicrobiota bacterium
CapCone-2

MCB0365788.1
Bdellovibrionales bacterium
CapCone-2(frag)

#############:CapCone-DUF4056; #######;neighborhood association with two distinct OmpA-like-beta-barrel domains, including a CdiB-like OmpA-like-beta-barrel domain. Sometimes joined by secreted alpha/beta-hydrolase domain

WP_076539863.1
Shewanella sp. UCD-KL21
OmpA-like-beta-barrel | ABhydrolase | CapCone-DUF4056 | OmpA-like-beta-barrel-CdiB-like

WP_229368866.1
Shewanella sp. 1_MG-2023
OmpA-like-beta-barrel | ABhydrolase | CapCone-DUF4056 | OmpA-like-beta-barrel-CdiB-like

GIU20041.1
Shewanella sp. MBTL60-112-B1
OmpA-like-beta-barrel | ABhydrolase | CapCone-DUF4056 | OmpA-like-beta-barrel-CdiB-like

WP_095497160.1
Paraferrimonas haliotis
OmpA-like-beta-barrel | ABhydrolase | CapCone-DUF4056 | OmpA-like-beta-barrel-CdiB-like

WP_035480687.1
Aliagarivorans marinus DSM 23064
OmpA-like-beta-barrel | ABhydrolase | CapCone-DUF4056 | OmpA-like-beta-barrel-CdiB-like

WP_153447400.1
Vibrio algicola
OmpA-like-beta-barrel | OmpA-like-beta-barrel-CdiB-like | ABhydrolase | CapCone-DUF4056 | ? | ? | ? | ? | ABhydrolase

WP_144388865.1
Vibrio algivorus
OmpA-like-beta-barrel | ABhydrolase | CapCone-DUF4056

WP_232322014.1
Photobacterium jeanii
OmpA-like-beta-barrel | OmpA-like-beta-barrel-CdiB-like | CapCone-DUF4056

BCS97217.1
Desulfoluna limicola
CapCone-DUF4056 | OmpA-like-beta-barrel-CdiB-like

MBE4247003.1
Vibrio parahaemolyticus
OmpA-like-beta-barrel | OmpA-like-beta-barrel-CdiB-like | CapCone-DUF4056

WP_140363697.1
OmpA-like-beta-barrel | OmpA-like-beta-barrel-CdiB-like | CapCone-DUF4056

WP_071239015.1
OmpA-like-beta-barrel | OmpA-like-beta-barrel-CdiB-like | CapCone-DUF4056

WP_017222904.1
Moritella dasanensis ArB 0140
OmpA-like-beta-barrel | CapCone-DUF4056 | OmpA-like-beta-barrel-CdiB-like

WP_001352825.1
OmpA-like-beta-barrel | OmpA-like-beta-barrel-CdiB-like | CapCone-DUF4056

AHY11190.1
Citrobacter freundii CFNIH1
OmpA-like-beta-barrel | OmpA-like-beta-barrel-CdiB-like | CapCone-DUF4056

WP_068437389.1
Providencia heimbachae
OmpA-like-beta-barrel | OmpA-like-beta-barrel-CdiB-like | CapCone-DUF4056

CSB56380.1
Vibrio cholerae
OmpA-like-beta-barrel | OmpA-like-beta-barrel-CdiB-like | CapCone-DUF4056

WP_228206213.1
OmpA-like-beta-barrel | OmpA-like-beta-barrel-CdiB-like | CapCone-DUF4056

WP_029834637.1
Vibrio parahaemolyticus S046
OmpA-like-beta-barrel | OmpA-like-beta-barrel-CdiB-like | CapCone-DUF4056

WP_199016912.1
Shimwellia pseudoproteus
OmpA-like-beta-barrel-CdiB-like | CapCone-DUF4056

MBM7344263.1
Pantoea coffeiphila
OmpA-like-beta-barrel | OmpA-like-beta-barrel-CdiB-like | CapCone-DUF4056

WP_233599388.1
Erwinia sp. JUb26
OmpA-like-beta-barrel | OmpA-like-beta-barrel-CdiB-like | CapCone-DUF4056

WP_225577082.1
Providencia alcalifaciens
OmpA-like-beta-barrel | OmpA-like-beta-barrel-CdiB-like | CapCone-DUF4056

WP_224120491.1
Providencia alcalifaciens
OmpA-like-beta-barrel | OmpA-like-beta-barrel-CdiB-like | CapCone-DUF4056

WP_206675590.1
Solimonas terrae
OmpA-like-beta-barrel-CdiB-like | CapCone-DUF4056

HAT3810408.1
Morganella morganii
OmpA-like-beta-barrel | OmpA-like-beta-barrel-CdiB-like | CapCone-DUF4056

WP_087489019.1
Tatumella citrea
OmpA-like-beta-barrel | OmpA-like-beta-barrel-CdiB-like | CapCone-DUF4056

EEM2539391.1
Salmonella enterica subsp. enterica serovar Morehead
OmpA-like-beta-barrel-CdiB-like | CapCone-DUF4056

WP_128175548.1
[Pantoea] beijingensis
OmpA-like-beta-barrel | OmpA-like-beta-barrel-CdiB-like | CapCone-DUF4056

WP_133845808.1
Erwinia rhapontici
OmpA-like-beta-barrel | OmpA-like-beta-barrel-CdiB-like | CapCone-DUF4056

WP_212714706.1
Tatumella sp. JGM118
OmpA-like-beta-barrel | OmpA-like-beta-barrel-CdiB-like | CapCone-DUF4056

GAL05331.1
Photobacterium aphoticum
OmpA-like-beta-barrel | OmpA-like-beta-barrel-CdiB-like | OmpA-like-beta-barrel-CdiB-like | CapCone-DUF4056

KQN54795.1
Erwinia sp. Leaf53
OmpA-like-beta-barrel | OmpA-like-beta-barrel-CdiB-like | CapCone-DUF4056

WP_154753725.1
Erwinia sorbitola
OmpA-like-beta-barrel | OmpA-like-beta-barrel-CdiB-like | CapCone-DUF4056

WP_053014232.1
Xenorhabdus sp. GDc328
OmpA-like-beta-barrel | OmpA-like-beta-barrel-CdiB-like | CapCone-DUF4056

WP_158313591.1
OmpA-like-beta-barrel | OmpA-like-beta-barrel | OmpA-like-beta-barrel-CdiB-like | CapCone-DUF4056

EAW1040144.1
Salmonella enterica
OmpA-like-beta-barrel | OmpA-like-beta-barrel-CdiB-like | CapCone-DUF4056

WP_009733749.1
Vibrio sp. N418
OmpA-like-beta-barrel-CdiB-like | CapCone-DUF4056

EHO9417998.1
Salmonella enterica
OmpA-like-beta-barrel | OmpA-like-beta-barrel-CdiB-like | CapCone-DUF4056

EHY0536206.1
Salmonella enterica
OmpA-like-beta-barrel | OmpA-like-beta-barrel-CdiB-like | CapCone-DUF4056

MBD2792529.1
Xenorhabdus szentirmaii
OmpA-like-beta-barrel | OmpA-like-beta-barrel-CdiB-like | CapCone-DUF4056

WP_228067819.1
Vibrio parahaemolyticus
OmpA-like-beta-barrel | OmpA-like-beta-barrel-CdiB-like | CapCone-DUF4056

WP_065824267.1
Photorhabdus australis subsp. thailandensis
OmpA-like-beta-barrel | OmpA-like-beta-barrel-CdiB-like | CapCone-DUF4056

WP_229001489.1
Photorhabdus aegyptia
OmpA-like-beta-barrel | OmpA-like-beta-barrel-CdiB-like | CapCone-DUF4056

WP_054305766.1
Serratia rubidaea
OmpA-like-beta-barrel | OmpA-like-beta-barrel-CdiB-like | CapCone-DUF4056

WP_044554427.1
Serratia liquefaciens
OmpA-like-beta-barrel | OmpA-like-beta-barrel-CdiB-like | CapCone-DUF4056

WP_166309875.1
Photorhabdus cinerea
OmpA-like-beta-barrel | OmpA-like-beta-barrel-CdiB-like | CapCone-DUF4056

WP_036102191.1
Mangrovibacter sp. MFB070
OmpA-like-beta-barrel | OmpA-like-beta-barrel-CdiB-like | CapCone-DUF4056

HAT1681591.1
Klebsiella oxytoca
OmpA-like-beta-barrel | OmpA-like-beta-barrel-CdiB-like | CapCone-DUF4056

WP_047877259.1
Photobacterium aquae
OmpA-like-beta-barrel | OmpA-like-beta-barrel-CdiB-like | CapCone-DUF4056

EDV3839034.1
Salmonella enterica subsp. diarizonae
OmpA-like-beta-barrel | OmpA-like-beta-barrel-CdiB-like | CapCone-DUF4056

WP_015727480.1
Vibrio vulnificus
OmpA-like-beta-barrel | OmpA-like-beta-barrel-CdiB-like | CapCone-DUF4056

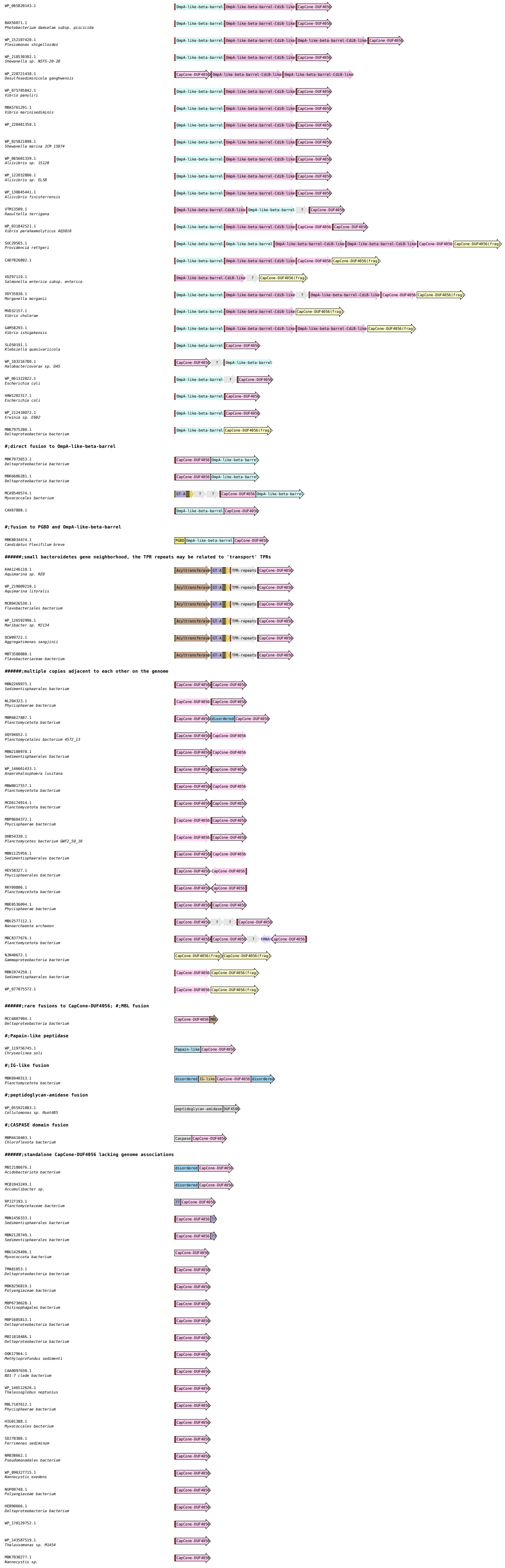

WP_065820143.1
OmpA-like-beta-barrel OmpA-like-beta-barrel-CdiB-like CapCone-DUF4056
BAX56071.1
Photobacterium damselae subsp. piscicida
OmpA-like-beta-barrel OmpA-like-beta-barrel-CdiB-like CapCone-DUF4056
WP_152107420.1
Plesiomonas shigelloides
OmpA-like-beta-barrel OmpA-like-beta-barrel-CdiB-like OmpA-like-beta-barrel-CdiB-like CapCone-DUF4056
WP_218530392.1
Shewanella sp. NIFS-20-20
OmpA-like-beta-barrel OmpA-like-beta-barrel-CdiB-like CapCone-DUF4056
WP_228721438.1
Desulfosediminicola ganghwensis
CapCone-DUF4056 OmpA-like-beta-barrel-CdiB-like OmpA-like-beta-barrel-CdiB-like
WP_075705842.1
Vibrio panuliri
OmpA-like-beta-barrel OmpA-like-beta-barrel-CdiB-like CapCone-DUF4056
MBA5761291.1
Vibrio marinisediminis
OmpA-like-beta-barrel OmpA-like-beta-barrel-CdiB-like CapCone-DUF4056
WP_228481358.1
OmpA-like-beta-barrel OmpA-like-beta-barrel-CdiB-like CapCone-DUF4056
WP_025821898.1
Shewanella marina JCM 15074
OmpA-like-beta-barrel OmpA-like-beta-barrel-CdiB-like CapCone-DUF4056
WP_065601339.1
Aliivibrio sp. 1S128
OmpA-like-beta-barrel OmpA-like-beta-barrel-CdiB-like CapCone-DUF4056
WP_122032806.1
Aliivibrio sp. EL58
OmpA-like-beta-barrel OmpA-like-beta-barrel-CdiB-like CapCone-DUF4056
WP_130045441.1
Aliivibrio finisterrensis
OmpA-like-beta-barrel OmpA-like-beta-barrel-CdiB-like CapCone-DUF4056
VTM13509.1
Raoultella terrigena
OmpA-like-beta-barrel-CdiB-like OmpA-like-beta-barrel ? CapCone-DUF4056
WP_031842521.1
Vibrio parahaemolyticus AQ3810
OmpA-like-beta-barrel OmpA-like-beta-barrel-CdiB-like CapCone-DUF4056 CapCone-DUF4056
SUC29565.1
Providencia rettgeri
OmpA-like-beta-barrel OmpA-like-beta-barrel OmpA-like-beta-barrel-CdiB-like OmpA-like-beta-barrel-CdiB-like CapCone-DUF4056 CapCone-DUF4056(frag)
CAD7826802.1
OmpA-like-beta-barrel OmpA-like-beta-barrel-CdiB-like CapCone-DUF4056 CapCone-DUF4056(frag)
VDZ97119.1
Salmonella enterica subsp. enterica
OmpA-like-beta-barrel-CdiB-like ? CapCone-DUF4056(frag)
VDY35836.1
Morganella morganii
OmpA-like-beta-barrel OmpA-like-beta-barrel-CdiB-like ? OmpA-like-beta-barrel-CdiB-like CapCone-DUF4056 CapCone-DUF4056(frag)
MVD32157.1
Vibrio cholerae
OmpA-like-beta-barrel OmpA-like-beta-barrel-CdiB-like CapCone-DUF4056(frag)
GAM58293.1
Vibrio ishigakensis
OmpA-like-beta-barrel OmpA-like-beta-barrel-CdiB-like OmpA-like-beta-barrel-CdiB-like CapCone-DUF4056(frag)
SLO50191.1
Klebsiella quasivariicola
OmpA-like-beta-barrel CapCone-DUF4056
WP_103216780.1
Halobacteriovorax sp. DA5
CapCone-DUF4056 ? OmpA-like-beta-barrel
WP_061322822.1
Escherichia coli
OmpA-like-beta-barrel ? CapCone-DUF4056
HAW1202317.1
Escherichia coli
OmpA-like-beta-barrel CapCone-DUF4056
WP_212410072.1
Erwinia sp. E602
OmpA-like-beta-barrel CapCone-DUF4056
MBK7975280.1
Deltaproteobacteria bacterium
OmpA-like-beta-barrel CapCone-DUF4056(frag)
#;direct fusion to OmpA-like-beta-barrel
MBK7973653.1
Deltaproteobacteria bacterium
CapCone-DUF4056 OmpA-like-beta-barrel
MBK6686281.1
Deltaproteobacteria bacterium
CapCone-DUF4056 OmpA-like-beta-barrel
MCA9549574.1
Myxococcales bacterium
GT-A ? ? CapCone-DUF4056 OmpA-like-beta-barrel
CAX67888.1
OmpA-like-beta-barrel CapCone-DUF4056
#;fusion to PGBD and OmpA-like-beta-barrel
MBK8034474.1
Candidatus Flexifilum breve
PGBD OmpA-like-beta-barrel CapCone-DUF4056
######;small bacteroidetes gene neighborhood, the TPR repeats may be related to 'transport' TPRs
KAA1246110.1
Aquimarina sp. RZ0
Acyltransferase GT-A TPR-repeats CapCone-DUF4056
WP_219009210.1
Aquimarina litoralis
Acyltransferase GT-A TPR-repeats CapCone-DUF4056
MCB9426530.1
Flavobacteriales bacterium
Acyltransferase GT-A TPR-repeats CapCone-DUF4056
WP_126592996.1
Maribacter sp. MJ134
Acyltransferase GT-A TPR-repeats CapCone-DUF4056
QCW99722.1
Aggregatimonas sangjinii
Acyltransferase GT-A TPR-repeats CapCone-DUF4056
MBT3588080.1
Flavobacteriaceae bacterium
Acyltransferase GT-A TPR-repeats CapCone-DUF4056
######;multiple copies adjacent to each other on the genome
MBN2269975.1
Sedimentisphaerales bacterium
CapCone-DUF4056 CapCone-DUF4056
NLZ04323.1
Phycisphaerae bacterium
CapCone-DUF4056 CapCone-DUF4056
MBM4027887.1
Planctomycetota bacterium
CapCone-DUF4056 disordered CapCone-DUF4056
OQY04652.1
Planctomycetales bacterium 4572_13
CapCone-DUF4056 CapCone-DUF4056
MBN2180978.1
Sedimentisphaerales bacterium
CapCone-DUF4056 CapCone-DUF4056
WP_146661433.1
Anaerohalosphaera lusitana
CapCone-DUF4056 CapCone-DUF4056
MBW8017557.1
Planctomycetota bacterium
CapCone-DUF4056 CapCone-DUF4056
MCD6174914.1
Planctomycetota bacterium
CapCone-DUF4056 CapCone-DUF4056
MBP8604372.1
Phycisphaerae bacterium
CapCone-DUF4056 CapCone-DUF4056
OHB54330.1
Planctomycetes bacterium GWF2_50_10
CapCone-DUF4056 CapCone-DUF4056
MBN1125956.1
Sedimentisphaerales bacterium
CapCone-DUF4056 CapCone-DUF4056
HEV58327.1
Phycisphaerales bacterium
CapCone-DUF4056 CapCone-DUF4056
RKY09806.1
Planctomycetota bacterium
CapCone-DUF4056 CapCone-DUF4056
MBE0536094.1
Phycisphaerae bacterium
CapCone-DUF4056 CapCone-DUF4056
MBU2577112.1
Nanoarchaeota archaeon
CapCone-DUF4056 ? ? CapCone-DUF4056
MBC8377676.1
Planctomycetota bacterium
CapCone-DUF4056 CapCone-DUF4056 ? tRNA CapCone-DUF4056
NJN40672.1
Gammaproteobacteria bacterium
CapCone-DUF4056(frag) CapCone-DUF4056(frag)
MBN1974258.1
Sedimentisphaerales bacterium
CapCone-DUF4056 CapCone-DUF4056(frag)
WP_077875572.1
CapCone-DUF4056 CapCone-DUF4056(frag)
######;rare fusions to CapCone-DUF4056; #;MBL fusion
MCC6807994.1
Deltaproteobacteria bacterium
CapCone-DUF4056 MBL
#;Papain-like peptidase
WP_119756745.1
Chryseolinea soli
Papain-like CapCone-DUF4056
#;IG-like fusion
MBK6940313.1
Planctomycetota bacterium
disordered IG-like CapCone-DUF4056 disordered
#;peptidoglycan-amidase fusion
WP_055921083.1
Cellulomonas sp. Root485
peptidoglycan-amidase DUF4506
#;CASPASE domain fusion
MBM4410403.1
Chloroflexota bacterium
Caspase CapCone-DUF4056
######;standalone CapCone-DUF4056 lacking genome associations
MBI2188676.1
Acidobacteriota bacterium
disordered CapCone-DUF4056
MCB1943249.1
Accumulibacter sp.
disordered CapCone-DUF4056
RPJ27193.1
Planctomycetaceae bacterium
?? CapCone-DUF4056
MBN1456333.1
Sedimentisphaerales bacterium
CapCone-DUF4056 ??
MBN2128749.1
Sedimentisphaerales bacterium
CapCone-DUF4056 ??
MBU1429496.1
Myxococcota bacterium
CapCone-DUF4056
TMA81053.1
Deltaproteobacteria bacterium
CapCone-DUF4056
MBK8256819.1
Polyangiaceae bacterium
CapCone-DUF4056
MBP6730628.1
Chitinophagales bacterium
CapCone-DUF4056
MBP1685813.1
Deltaproteobacteria bacterium
CapCone-DUF4056
MBI1818486.1
Deltaproteobacteria bacterium
CapCone-DUF4056
OQK17964.1
Methyloprofundus sedimenti
CapCone-DUF4056
CAA0097030.1
BD1-7 clade bacterium
CapCone-DUF4056
WP_146512626.1
Thalassoglobus neptunius
CapCone-DUF4056
MBL7107612.1
Phycisphaerae bacterium
CapCone-DUF4056
HIG01388.1
Myxococcales bacterium
CapCone-DUF4056
SDJ78386.1
Ferrimonas sediminum
CapCone-DUF4056
NRB38662.1
Pseudomonadales bacterium
CapCone-DUF4056
WP_096327715.1
Nannocystis exedens
CapCone-DUF4056
NUP09748.1
Polyangiaceae bacterium
CapCone-DUF4056
HEB90666.1
Deltaproteobacteria bacterium
CapCone-DUF4056
WP_170129752.1
CapCone-DUF4056
WP_143587519.1
Thalassomonas sp. M1454
CapCone-DUF4056
MBK7830277.1
Nannocystis sp.
CapCone-DUF4056

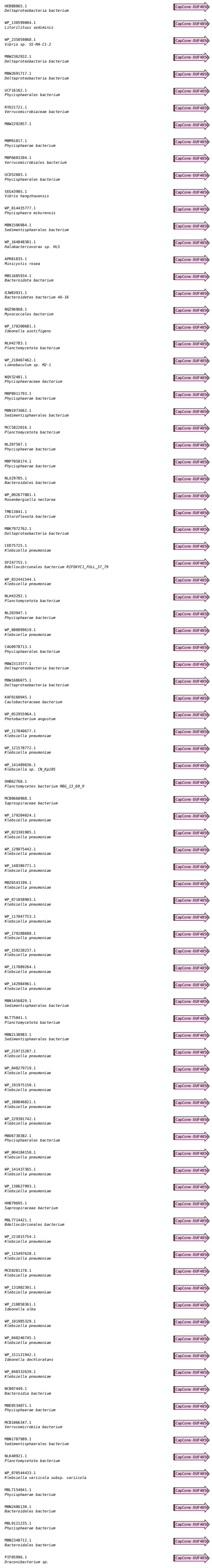
HEB88865.1
Deltaproteobacteria bacterium
CapCone-DUF4056
WP_130599004.1
Litorilituus sediminis
CapCone-DUF4056
WP_235059868.1
Vibrio sp. SS-MA-C1-2
CapCone-DUF4056
MBW2362932.1
Deltaproteobacteria bacterium
CapCone-DUF4056
MBW2691717.1
Deltaproteobacteria bacterium
CapCone-DUF4056
UCF16162.1
Phycisphaerales bacterium
CapCone-DUF4056
RYD21721.1
Verrucomicrobiaceae bacterium
CapCone-DUF4056
MBW2292857.1
CapCone-DUF4056
MBM91017.1
Phycisphaerae bacterium
CapCone-DUF4056
MBP6603204.1
Verrucomicrobiales bacterium
CapCone-DUF4056
UCD52603.1
Phycisphaerales bacterium
CapCone-DUF4056
SEG43905.1
Vibrio hangzhouensis
CapCone-DUF4056
WP_014435777.1
Phycisphaera mikurensis
CapCone-DUF4056
MBN1506984.1
Sedimentisphaerales bacterium
CapCone-DUF4056
WP_164848381.1
Halobacteriovorax sp. HLS
CapCone-DUF4056
APR81835.1
Minicystis rosea
CapCone-DUF4056
MBS1685934.1
Bacteroidota bacterium
CapCone-DUF4056
OJW83931.1
Bacteroidetes bacterium 46-16
CapCone-DUF4056
NQZ96968.1
Myxococcales bacterium
CapCone-DUF4056
WP_170200681.1
Ideonella azotifigens
CapCone-DUF4056
NLH42783.1
Planctomycetota bacterium
CapCone-DUF4056
WP_218467462.1
Limnobaculum sp. M2-1
CapCone-DUF4056
NQV32481.1
Phycisphaeraceae bacterium
CapCone-DUF4056
MBP8911793.1
Phycisphaerae bacterium
CapCone-DUF4056
MBN1973662.1
Sedimentisphaerales bacterium
CapCone-DUF4056
MCC5822016.1
Planctomycetota bacterium
CapCone-DUF4056
NLZ07507.1
Phycisphaerae bacterium
CapCone-DUF4056
MBP7050174.1
Phycisphaerae bacterium
CapCone-DUF4056
NLX29785.1
Bacteroidales bacterium
CapCone-DUF4056
WP_092677881.1
Rosenbergiella nectarea
CapCone-DUF4056
TMD13841.1
Chloroflexota bacterium
CapCone-DUF4056
MBK7972762.1
Deltaproteobacteria bacterium
CapCone-DUF4056
CED75725.1
Klebsiella pneumoniae
CapCone-DUF4056
OFZ47755.1
Bdellovibrionales bacterium RIFOXYC1_FULL_37_79
CapCone-DUF4056
WP_032441544.1
Klebsiella pneumoniae
CapCone-DUF4056
NLH43292.1
Planctomycetota bacterium
CapCone-DUF4056
NLZ03947.1
Phycisphaerae bacterium
CapCone-DUF4056
WP_080899619.1
Klebsiella pneumoniae
CapCone-DUF4056
CAG0978713.1
Phycisphaerales bacterium
CapCone-DUF4056
MBW2313577.1
Deltaproteobacteria bacterium
CapCone-DUF4056
MBW1686075.1
Deltaproteobacteria bacterium
CapCone-DUF4056
KAF0160945.1
Caulobacteraceae bacterium
CapCone-DUF4056
WP_052955964.1
Photobacterium angustum
CapCone-DUF4056
WP_117040677.1
Klebsiella pneumoniae
CapCone-DUF4056
WP_121578772.1
Klebsiella pneumoniae
CapCone-DUF4056
WP_141489836.1
Klebsiella sp. CN_Kp105
CapCone-DUF4056
OHB62768.1
Planctomycetes bacterium RBG_13_60_9
CapCone-DUF4056
MCB0668968.1
Saprospiraceae bacterium
CapCone-DUF4056
WP_179204024.1
Klebsiella pneumoniae
CapCone-DUF4056
WP_023301905.1
Klebsiella pneumoniae
CapCone-DUF4056
WP_129075442.1
Klebsiella pneumoniae
CapCone-DUF4056
WP_148386771.1
Klebsiella pneumoniae
CapCone-DUF4056
MBZ6543199.1
Klebsiella pneumoniae
CapCone-DUF4056
WP_071058903.1
Klebsiella pneumoniae
CapCone-DUF4056
WP_117047753.1
Klebsiella pneumoniae
CapCone-DUF4056
WP_179288688.1
Klebsiella pneumoniae
CapCone-DUF4056
WP_159220237.1
Klebsiella pneumoniae
CapCone-DUF4056
WP_117089264.1
Klebsiella pneumoniae
CapCone-DUF4056
WP_142984961.1
Klebsiella pneumoniae
CapCone-DUF4056
MBN1456829.1
Sedimentisphaerales bacterium
CapCone-DUF4056
NLT75041.1
Planctomycetota bacterium
CapCone-DUF4056
MBN2138983.1
Sedimentisphaerales bacterium
CapCone-DUF4056
WP_219715287.1
Klebsiella pneumoniae
CapCone-DUF4056
WP_048279719.1
Klebsiella pneumoniae
CapCone-DUF4056
WP_191975150.1
Klebsiella pneumoniae
CapCone-DUF4056
WP_180846821.1
Klebsiella pneumoniae
CapCone-DUF4056
WP_229381742.1
Klebsiella pneumoniae
CapCone-DUF4056
MBO6738382.1
Phycisphaerales bacterium
CapCone-DUF4056
WP_004184150.1
Klebsiella pneumoniae
CapCone-DUF4056
WP_141437365.1
Klebsiella pneumoniae
CapCone-DUF4056
WP_130627993.1
Klebsiella pneumoniae
CapCone-DUF4056
HHB79695.1
Saprospiraceae bacterium
CapCone-DUF4056
MBL7714421.1
Bdellovibrionales bacterium
CapCone-DUF4056
WP_221015754.1
Klebsiella pneumoniae
CapCone-DUF4056
WP_115497628.1
Klebsiella pneumoniae
CapCone-DUF4056
MCE0281178.1
Klebsiella pneumoniae
CapCone-DUF4056
WP_131082301.1
Klebsiella pneumoniae
CapCone-DUF4056
WP_210858361.1
Ideonella alba
CapCone-DUF4056
WP_101985329.1
Klebsiella pneumoniae
CapCone-DUF4056
WP_040246745.1
Klebsiella pneumoniae
CapCone-DUF4056
WP_151121942.1
Ideonella dechloratans
CapCone-DUF4056
WP_048332639.1
Klebsiella pneumoniae
CapCone-DUF4056
NCB07449.1
Bacteroidia bacterium
CapCone-DUF4056
MBE0534871.1
Phycisphaerae bacterium
CapCone-DUF4056
MCB1066347.1
Verrucomicrobiia bacterium
CapCone-DUF4056
MBN1787989.1
Sedimentisphaerales bacterium
CapCone-DUF4056
NLK40921.1
Planctomycetota bacterium
CapCone-DUF4056
WP_070544433.1
Klebsiella variicola subsp. variicola
CapCone-DUF4056
MBL7154041.1
Phycisphaerae bacterium
CapCone-DUF4056
MBN2486130.1
Bacteroidales bacterium
CapCone-DUF4056
MBL9121235.1
Phycisphaerae bacterium
CapCone-DUF4056
MBN2348712.1
Bacteroidales bacterium
CapCone-DUF4056
PIF05996.1
Draconibacterium sp.
CapCone-DUF4056

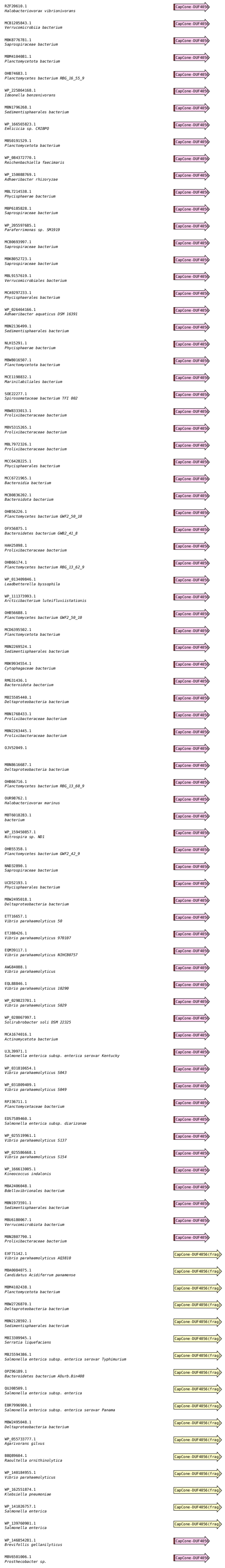

RZF20610.1
Halobacteriovorax vibrionivorans
CapCone-DUF4056
MCB1205843.1
Verrucomicrobiia bacterium
CapCone-DUF4056
MBK8776781.1
Saprospiraceae bacterium
CapCone-DUF4056
MBM4104081.1
Planctomycetota bacterium
CapCone-DUF4056
OHB74683.1
Planctomycetes bacterium RBG_16_55_9
CapCone-DUF4056
WP_225864168.1
Ideonella benzenivorans
CapCone-DUF4056
MBN1796268.1
Sedimentisphaerales bacterium
CapCone-DUF4056
WP_166565823.1
Emticicia sp. CRIBPO
CapCone-DUF4056
MBS0191529.1
Planctomycetota bacterium
CapCone-DUF4056
WP_084372770.1
Reichenbachiella faecimaris
CapCone-DUF4056
WP_150088769.1
Adhaeribacter rhizoryzae
CapCone-DUF4056
MBL7214538.1
Phycisphaerae bacterium
CapCone-DUF4056
MBP6185828.1
Saprospiraceae bacterium
CapCone-DUF4056
WP_205597685.1
Paraferrimonas sp. SM1919
CapCone-DUF4056
MCB0693997.1
Saprospiraceae bacterium
CapCone-DUF4056
MBK8052723.1
Saprospiraceae bacterium
CapCone-DUF4056
MBL9157619.1
Verrucomicrobiales bacterium
CapCone-DUF4056
MCA9297233.1
Phycisphaerales bacterium
CapCone-DUF4056
WP_026464166.1
Adhaeribacter aquaticus DSM 16391
CapCone-DUF4056
MBN2136499.1
Sedimentisphaerales bacterium
CapCone-DUF4056
NLH15291.1
Phycisphaerae bacterium
CapCone-DUF4056
MBW8016507.1
Planctomycetota bacterium
CapCone-DUF4056
MCE1198832.1
Marinilabiliales bacterium
CapCone-DUF4056
SOE22277.1
Spirosomataceae bacterium TFI 002
CapCone-DUF4056
MBW8333013.1
Prolixibacteraceae bacterium
CapCone-DUF4056
MBV5315265.1
Prolixibacteraceae bacterium
CapCone-DUF4056
MBL7972326.1
Prolixibacteraceae bacterium
CapCone-DUF4056
MCC6428225.1
Phycisphaerales bacterium
CapCone-DUF4056
MCC6721965.1
Bacteroidia bacterium
CapCone-DUF4056
MCB0836202.1
Bacteroidota bacterium
CapCone-DUF4056
OHB56226.1
Planctomycetes bacterium GWF2_50_10
CapCone-DUF4056
OFX56875.1
Bacteroidetes bacterium GWB2_41_8
CapCone-DUF4056
HAH25098.1
Prolixibacteraceae bacterium
CapCone-DUF4056
OHB66174.1
Planctomycetes bacterium RBG_13_62_9
CapCone-DUF4056
WP_013409846.1
Leadbetterella byssophila
CapCone-DUF4056
WP_111373993.1
Arcticibacterium luteifluviistationis
CapCone-DUF4056
OHB56688.1
Planctomycetes bacterium GWF2_50_10
CapCone-DUF4056
MCD6395502.1
Planctomycetota bacterium
CapCone-DUF4056
MBN2269524.1
Sedimentisphaerales bacterium
CapCone-DUF4056
MBK9934554.1
Cytophagaceae bacterium
CapCone-DUF4056
RMG31436.1
Bacteroidota bacterium
CapCone-DUF4056
MBI5505440.1
Deltaproteobacteria bacterium
CapCone-DUF4056
MBN1768433.1
Prolixibacteraceae bacterium
CapCone-DUF4056
MBN2263445.1
Prolixibacteraceae bacterium
CapCone-DUF4056
OJV52049.1
CapCone-DUF4056
MBN8616687.1
Deltaproteobacteria bacterium
CapCone-DUF4056
OHB66716.1
Planctomycetes bacterium RBG_13_60_9
CapCone-DUF4056
OUR98762.1
Halobacteriovorax marinus
CapCone-DUF4056
MBT6018283.1
bacterium
CapCone-DUF4056
WP_159450857.1
Nitrospira sp. ND1
CapCone-DUF4056
OHB55358.1
Planctomycetes bacterium GWF2_42_9
CapCone-DUF4056
NND32890.1
Saprospiraceae bacterium
CapCone-DUF4056
UCD52193.1
Phycisphaerales bacterium
CapCone-DUF4056
MBW2495018.1
Deltaproteobacteria bacterium
CapCone-DUF4056
ETT16657.1
Vibrio parahaemolyticus 50
CapCone-DUF4056
ETJ88426.1
Vibrio parahaemolyticus 970107
CapCone-DUF4056
EQM39117.1
Vibrio parahaemolyticus NIHCB0757
CapCone-DUF4056
AWG84088.1
Vibrio parahaemolyticus
CapCone-DUF4056
EQL88846.1
Vibrio parahaemolyticus 10290
CapCone-DUF4056
WP_029823701.1
Vibrio parahaemolyticus S029
CapCone-DUF4056
WP_028067997.1
Solirubrobacter soli DSM 22325
CapCone-DUF4056
MCA1674016.1
Actinomycetota bacterium
CapCone-DUF4056
UJL39971.1
Salmonella enterica subsp. enterica serovar Kentucky
CapCone-DUF4056
WP_031810654.1
Vibrio parahaemolyticus S043
CapCone-DUF4056
WP_031809409.1
Vibrio parahaemolyticus S049
CapCone-DUF4056
RPJ36711.1
Planctomycetaceae bacterium
CapCone-DUF4056
EDS7589460.1
Salmonella enterica subsp. diarizonae
CapCone-DUF4056
WP_025519961.1
Vibrio parahaemolyticus S137
CapCone-DUF4056
WP_025586668.1
Vibrio parahaemolyticus S154
CapCone-DUF4056
WP_166613005.1
Kineococcus indalonis
CapCone-DUF4056
MBA2406048.1
Bdellovibrionales bacterium
CapCone-DUF4056
MBN1973591.1
Sedimentisphaerales bacterium
CapCone-DUF4056
MBU6180067.1
Verrucomicrobiota bacterium
CapCone-DUF4056
MBN2807790.1
Prolixibacteraceae bacterium
CapCone-DUF4056
EXF71142.1
Vibrio parahaemolyticus AQ3810
CapCone-DUF4056(frag
MBA0084075.1
Candidatus Acidiferrum panamense
CapCone-DUF4056(frag
MBM4102438.1
Planctomycetota bacterium
CapCone-DUF4056(frag
MBW2726870.1
Deltaproteobacteria bacterium
CapCone-DUF4056(frag
MBN2128592.1
Sedimentisphaerales bacterium
CapCone-DUF4056(frag
MBI3309945.1
Serratia liquefaciens
CapCone-DUF4056(frag
MBJ5594386.1
Salmonella enterica subsp. enterica serovar Typhimurium
CapCone-DUF4056(frag
OPZ96189.1
Bacteroidetes bacterium ADurb.Bin408
CapCone-DUF4056(frag
QUJ08509.1
Salmonella enterica subsp. enterica
CapCone-DUF4056(frag
EBR7996900.1
Salmonella enterica subsp. enterica serovar Panama
CapCone-DUF4056(frag
MBW2495048.1
Deltaproteobacteria bacterium
CapCone-DUF4056(frag
WP_055733777.1
Agarivorans gilvus
CapCone-DUF4056(frag
BBQ89604.1
Raoultella ornithinolytica
CapCone-DUF4056(frag
WP_140184955.1
Vibrio parahaemolyticus
CapCone-DUF4056(frag
WP_162551874.1
Klebsiella pneumoniae
CapCone-DUF4056(frag
WP_141026757.1
Salmonella enterica
CapCone-DUF4056(frag
WP_139760901.1
Salmonella enterica
CapCone-DUF4056(frag
WP_146854281.1
Brevifollis gellanilyticus
CapCone-DUF4056
MBV6501006.1
Prosthecobacter sp.
CapCone-DUF4056

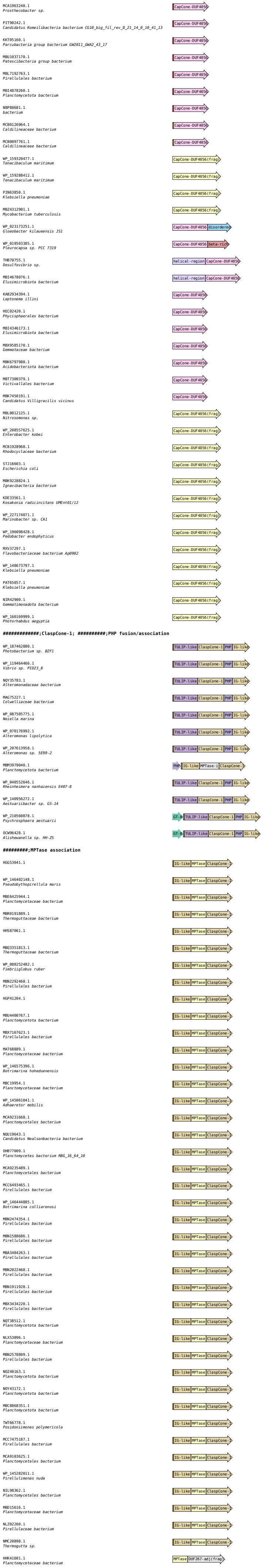
MCA1963240.1
Prosthecobacter sp.
CapCone-DUF4056
PIT90242.1
Candidatus Komeilibacteria bacterium CG10_big_fil_rev_8_21_14_0_10_41_13
CapCone-DUF4056
KKT05160.1
Parcubacteria group bacterium GW2011_GWA2_43_17
CapCone-DUF4056
MBU1037170.1
Patescibacteria group bacterium
CapCone-DUF4056
MBL7192763.1
Pirellulales bacterium
CapCone-DUF4056
MBI4878260.1
Planctomycetota bacterium
CapCone-DUF4056
NBP80681.1
bacterium
CapCone-DUF4056
MCB0126964.1
Caldilineaceae bacterium
CapCone-DUF4056
MCB0097761.1
Caldilineaceae bacterium
CapCone-DUF4056
WP_159320477.1
Tenacibaculum maritimum
CapCone-DUF4056(frag)
WP_159288412.1
Tenacibaculum maritimum
CapCone-DUF4056(frag)
PIN63850.1
Klebsiella pneumoniae
CapCone-DUF4056(frag)
MBZ4312901.1
Mycobacterium tuberculosis
CapCone-DUF4056(frag)
WP_023173251.1
Gloeobacter kilaueensis JS1
CapCone-DUF4056
disordered
WP_019503385.1
Pleurocapsa sp. PCC 7319
CapCone-DUF4056
beta-rich
THB70755.1
Desulfovibrio sp.
helical-region
CapCone-DUF4056
MBI4678076.1
Elusimicrobiota bacterium
helical-region
CapCone-DUF4056
KAB2934394.1
Leptonema illini
CapCone-DUF4056
HEC02420.1
Phycisphaerales bacterium
CapCone-DUF4056
MBI4346173.1
Elusimicrobiota bacterium
CapCone-DUF4056
MBX9585170.1
Gemmataceae bacterium
CapCone-DUF4056
MBK6797980.1
Acidobacteriota bacterium
CapCone-DUF4056
MBT7300379.1
Victivallales bacterium
CapCone-DUF4056
MBK7450191.1
Candidatus Villigracilis vicinus
CapCone-DUF4056
MBL0012125.1
Nitrosomonas sp.
CapCone-DUF4056(frag)
WP_208557625.1
Enterobacter kobei
CapCone-DUF4056(frag)
MCB1928968.1
Rhodocyclaceae bacterium
CapCone-DUF4056(frag)
STJ16603.1
Escherichia coli
CapCone-DUF4056(frag)
MBK9228824.1
Ignavibacteria bacterium
CapCone-DUF4056(frag)
KDE33561.1
Kosakonia radicincitans UMEnt01/12
CapCone-DUF4056(frag)
WP_227174071.1
Marinobacter sp. CA1
CapCone-DUF4056(frag)
WP_196098428.1
Pedobacter endophyticus
CapCone-DUF4056(frag)
MXV37297.1
Flavobacteriaceae bacterium Ap0902
CapCone-DUF4056(frag)
WP_148673797.1
Klebsiella pneumoniae
CapCone-DUF4056(frag)
PAT65057.1
Klebsiella pneumoniae
CapCone-DUF4056(frag)
NIR42900.1
Gemmatimonadota bacterium
CapCone-DUF4056(frag)
WP_160169999.1
Photorhabdus aegyptia
CapCone-DUF4056(frag)
#############;ClaspCone-1; ##########;PHP fusion/association
WP_187462880.1
Photobacterium sp. BZF1
TULIP-like
ClaspCone-1
PHP
IG-like
WP_119464466.1
Vibrio sp. PID23_8
TULIP-like
ClaspCone-1
PHP
IG-like
NQY35783.1
Alteromonadaceae bacterium
TULIP-like
ClaspCone-1
PHP
IG-like
MAG75227.1
Colwelliaceae bacterium
TULIP-like
ClaspCone-1
PHP
IG-like
WP_087505775.1
Neiella marina
TULIP-like
ClaspCone-1
PHP
IG-like
WP_070176992.1
Alteromonas lipolytica
TULIP-like
ClaspCone-1
PHP
IG-like
WP_207613958.1
Alteromonas sp. 5E99-2
TULIP-like
ClaspCone-1
PHP
IG-like
MBM3979040.1
Planctomycetota bacterium
PHP
IG-like
MPTase-i
ClaspCone-1
WP_040552646.1
Rheinheimera nanhaiensis E407-8
TULIP-like
ClaspCone-1
PHP
IG-like
WP_140956272.1
Aestuariibacter sp. GS-14
TULIP-like
ClaspCone-1
PHP
IG-like
WP_210560878.1
Psychrosphaera aestuarii
GT-B
TULIP-like
ClaspCone-1
PHP
IG-like
OCW96428.1
Alishewanella sp. HH-ZS
GT-B
TULIP-like
ClaspCone-1
PHP
IG-like
#########;MPTase association
HGG53041.1
IG-like
MPTase
ClaspCone-1
WP_146402148.1
Pseudobythopirellula maris
IG-like
MPTase
ClaspCone-1
MBE6425944.1
Planctomycetaceae bacterium
IG-like
MPTase
ClaspCone-1
MBR0191889.1
Thermoguttaceae bacterium
IG-like
MPTase
ClaspCone-1
HHS87061.1
IG-like
MPTase
ClaspCone-1
MBQ3351813.1
Thermoguttaceae bacterium
IG-like
MPTase
ClaspCone-1
WP_088252482.1
Fimbriiglobus ruber
IG-like
MPTase
ClaspCone-1
MBN2292468.1
Pirellulales bacterium
IG-like
MPTase
ClaspCone-1
HGP41204.1
IG-like
MPTase
ClaspCone-1
MBU4400767.1
Planctomycetota bacterium
IG-like
MPTase
ClaspCone-1
MBX7167623.1
Pirellulales bacterium
IG-like
MPTase
ClaspCone-1
MAT68889.1
Planctomycetaceae bacterium
IG-like
MPTase
ClaspCone-1
WP_146575396.1
Botrimarina hoheduenensis
IG-like
MPTase
ClaspCone-1
MBC19954.1
Planctomycetaceae bacterium
IG-like
MPTase
ClaspCone-1
WP_145061041.1
Adhaeretor mobilis
IG-like
MPTase
ClaspCone-1
MCA9231668.1
Planctomycetales bacterium
IG-like
MPTase
ClaspCone-1
NQU19643.1
Candidatus Nealsonbacteria bacterium
IG-like
MPTase
ClaspCone-1
OHB77009.1
Planctomycetes bacterium RBG_16_64_10
IG-like
MPTase
ClaspCone-1
MCA9235489.1
Planctomycetales bacterium
IG-like
MPTase
ClaspCone-1
MCC6493465.1
Pirellulales bacterium
IG-like
MPTase
ClaspCone-1
WP_146444885.1
Botrimarina colliarenosi
IG-like
MPTase
ClaspCone-1
MBN2474354.1
Pirellulales bacterium
IG-like
MPTase
ClaspCone-1
MBN1588686.1
Pirellulales bacterium
IG-like
MPTase
ClaspCone-1
MBA3484263.1
Pirellulales bacterium
IG-like
MPTase
ClaspCone-1
MBN2022468.1
Pirellulales bacterium
IG-like
MPTase
ClaspCone-1
MBN1911928.1
Pirellulales bacterium
IG-like
MPTase
ClaspCone-1
MBX3434220.1
Pirellulales bacterium
IG-like
MPTase
ClaspCone-1
NQT38512.1
Planctomycetota bacterium
IG-like
MPTase
ClaspCone-1
NLX53896.1
Planctomycetaceae bacterium
IG-like
MPTase
ClaspCone-1
MBN2578809.1
Pirellulales bacterium
IG-like
MPTase
ClaspCone-1
NOZ40163.1
Planctomycetota bacterium
IG-like
MPTase
ClaspCone-1
NOY43172.1
Planctomycetota bacterium
IG-like
MPTase
ClaspCone-1
MBC8868351.1
Planctomycetota bacterium
IG-like
MPTase
ClaspCone-1
TWT66778.1
Posidoniimonas polymericola
IG-like
MPTase
ClaspCone-1
MCC7475187.1
Pirellulales bacterium
IG-like
MPTase
ClaspCone-1
MCA9103625.1
Planctomycetales bacterium
IG-like
MPTase
ClaspCone-1
WP_145282011.1
Pirellulimonas nuda
IG-like
MPTase
ClaspCone-1
NIL98362.1
Planctomycetales bacterium
IG-like
MPTase
ClaspCone-1
MBD15616.1
Planctomycetaceae bacterium
IG-like
MPTase
ClaspCone-1
NLZ02260.1
Pirellulaceae bacterium
IG-like
MPTase
ClaspCone-1
NMC20898.1
Thermogutta sp.
IG-like
MPTase
ClaspCone-1
HHK41601.1
Planctomycetaceae bacterium
MPTase
DUF267-adj(frag)

RMG02391.1	*Planctomycetota bacterium*	MPTase · DUF267-adj(frag)

#;inactive MPTase

WP_149111871.1	*Limnoglobus roseus*	IG-like · MPTase-i · ClaspCone-1
MBN9520423.1	*bacterium*	IG-like · MPTase-i · ClaspCone-1
MBX3401308.1	*Gemmataceae bacterium*	IG-like · MPTase-i · ClaspCone-1
QDU56318.1	*Aeoliella mucimassa*	IG-like · MPTase-i · ClaspCone-1
MBN9119997.1	*Planctomycetota bacterium*	IG-like · MPTase-i · ClaspCone-1
WP_171474632.1	*Frigoriglobus tundricola*	IG-like · MPTase-i · ClaspCone-1
WP_010039904.1	*Gemmata obscuriglobus UQM 2246*	IG-like · MPTase-i · ClaspCone-1
MCA9240819.1	*Planctomycetales bacterium*	IG-like · MPTase-i · ClaspCone-1

#;zinc finger found in the dskA TF ** unlikely to be real?

MAW38777.1	*Gammaproteobacteria bacterium*	TULIP-like · ClaspCone-1 · zf-dskA_traR
HAG71005.1	*Gammaproteobacteria bacterium*	TULIP-like · ClaspCone-1 · zf-dskA_traR
MBL4581165.1	*Gammaproteobacteria bacterium*	TULIP-like · ClaspCone-1 · zf-dskA_traR
MBL6905536.1	*Pseudomonadales bacterium*	TULIP-like · ClaspCone-1 · zf-dskA_traR
NQV68429.1	*Pseudohongiella sp.*	TULIP-like · ClaspCone-1 · zf-dskA_traR
MAJ55600.1	*Gammaproteobacteria bacterium*	TULIP-like · ClaspCone-1 · zf-dskA_traR
MAS59893.1	*Gammaproteobacteria bacterium*	TULIP-like · ClaspCone-1 · zf-dskA_traR
HIF86131.1	*Gammaproteobacteria bacterium*	TULIP-like · ClaspCone-1 · zf-dskA_traR
MBT7678780.1	*Gammaproteobacteria bacterium*	TULIP-like · ClaspCone-1 · zf-dskA_traR
MBT73051.1	*Gammaproteobacteria bacterium*	TULIP-like · ClaspCone-1 · zf-dskA_traR
MAU05661.1	*Gammaproteobacteria bacterium*	TULIP-like · ClaspCone-1 · zf-dskA_traR
PDH33023.1	*OM182 bacterium MED-G28*	TULIP-like · ClaspCone-1 · zf-dskA_traR
MBM87740.1	*Gammaproteobacteria bacterium*	TULIP-like · ClaspCone-1 · zf-dskA_traR
NKB34122.1	*Pseudomonadales bacterium*	TULIP-like · ClaspCone-1 · zf-dskA_traR
PCH60570.1	*SAR86 cluster bacterium*	TULIP-like · ClaspCone-1 · zf-dskA_traR
MBT6043102.1	*Gammaproteobacteria bacterium*	TULIP-like · ClaspCone-1 · zf-dskA_traR
NBS24690.1	*Gammaproteobacteria bacterium*	TULIP-like · ClaspCone-1 · zf-dskA_traR
MBN4053498.1	*Haliea sp. AH-315-K21*	TULIP-like · ClaspCone-1 · zf-dskA_traR
HIF74163.1	*Gammaproteobacteria bacterium*	TULIP-like · ClaspCone-1 · zf-dskA_traR
MBN2010386.1	*Candidatus Zhuqueibacterota bacterium*	TULIP-like · ClaspCone-1 · zf-dskA_traR
HAT27683.1	*Gammaproteobacteria bacterium*	ClaspCone-1 · zf-dskA_traR
MAK94332.1	*Gammaproteobacteria bacterium*	?? · ClaspCone-1 · zf-dskA_traR
GIT21756.1	*Gammaproteobacteria bacterium*	ClaspCone-1 · zf-dskA_traR

########;association with an exopolyphosphatase of the Actin ATPase family fused to a HD domain

MBU2568725.1	*Gammaproteobacteria bacterium*	Ppx-GppA · HD · TULIP-like · ClaspCone-1
TSA41324.1	*Methylococcaceae bacterium*	Ppx-GppA · HD · TULIP-like · ClaspCone-1
MBV5302228.1	*Methylococcales bacterium*	Ppx-GppA · HD · TULIP-like · ClaspCone-1
NOR81763.1	*Methyloprofundus sp.*	Ppx-GppA · HD · TULIP-like · ClaspCone-1
WP_023496491.1	*Methyloglobulus morosus KoM1*	Ppx-GppA · HD · TULIP-like · ClaspCone-1
WP_031433404.1	*Methylomarinum vadi*	Ppx-GppA · HD · TULIP-like · ClaspCone-1
PPD36446.1	*Methylomonas sp.*	Ppx-GppA · HD · TULIP-like · ClaspCone-1
WP_159658738.1	*Methylicorpusculum oleiharenae*	Ppx-GppA · HD · TULIP-like · ClaspCone-1
WP_062329691.1	*Methylomonas methanica*	Ppx-GppA · HD · TULIP-like · ClaspCone-1
GDX85365.1	*Methylococcaceae bacterium*	Ppx-GppA · HD · TULIP-like · ClaspCone-1
BCG65421.1	*Methyloprofundus sp.*	Ppx-GppA · HD · TULIP-like · ClaspCone-1
WP_198243134.1	*methane-oxidizing endosymbiont of Gigantopelta aegis*	Ppx-GppA · HD · TULIP-like · ClaspCone-1
WP_080523605.1	*Methyloprofundus sedimenti*	Ppx-GppA · HD · TULIP-like · ClaspCone-1
NOQ35996.1	*Methylococcaceae bacterium*	Ppx-GppA · HD · TULIP-like · ClaspCone-1
WP_020481274.1	*Methylomonas sp. MK1*	Ppx-GppA · HD · TULIP-like · ClaspCone-1
TAL44255.1	*Methylovulum sp.*	Ppx-GppA · HD · TULIP-like · ClaspCone-1
KAF3983051.1	*Methylococcales symbiont of Hymedesmia sp. n. MRB-2018*	Ppx-GppA · HD · TULIP-like · ClaspCone-1
NOQ64145.1	*Methyloprofundus sp.*	Ppx-GppA · HD · TULIP-like · ClaspCone-1
MBT6523511.1	*Methylococcales bacterium*	Ppx-GppA · HD · TULIP-like · ClaspCone-1
MSP28857.1	*Methylococcales bacterium*	Ppx-GppA · HD · TULIP-like · ClaspCone-1
WP_024297998.1	*Methylomicrobium lacus LW14*	Ppx-GppA · HD · TULIP-like · ClaspCone-1
HIG91948.1	*Methylococcaceae bacterium*	Ppx-GppA · HD · TULIP-like · ClaspCone-1
MBE0468620.1	*Methyloprofundus sp.*	Ppx-GppA · HD · TULIP-like · ClaspCone-1
OYV21087.1	*Methylococcaceae bacterium NSP1-2*	Ppx-GppA · HD · TULIP-like · ClaspCone-1
NOQ15255.1	*Methyloprofundus sp.*	Ppx-GppA · HD · TULIP-like · ClaspCone-1
WP_019865881.1	*Methylovulum miyakonense HT12*	Ppx-GppA · HD · TULIP-like · ClaspCone-1
WP_103973391.1	*Methylovulum psychrotolerans*	Ppx-GppA · HD · TULIP-like · ClaspCone-1
HHZ71361.1	*Methylococcaceae bacterium*	Ppx-GppA · HD · TULIP-like · ClaspCone-1
WP_036254548.1	*Methylobacter sp. BBA5.1*	Ppx-GppA · HD · TULIP-like · ClaspCone-1
NOT83667.1	*Methylococcaceae bacterium*	Ppx-GppA · HD · TULIP-like · ClaspCone-1
WP_215585153.1		Ppx-GppA · HD · TULIP-like · ClaspCone-1
WP_196434303.1	*Methylomonas sp. LL1*	Ppx-GppA · HD · TULIP-like · ClaspCone-1
NOS88978.1	*Methylococcaceae bacterium*	Ppx-GppA · HD · TULIP-like · ClaspCone-1
WP_150048081.1	*Methylomonas sp. HYX-M1*	Ppx-GppA · HD · TULIP-like · ClaspCone-1
WP_087145206.1	*Crenothrix polyspora*	Ppx-GppA · HD · TULIP-like · ClaspCone-1
HIG08105.1	*Methylococcaceae bacterium*	Ppx-GppA · HD · TULIP-like · ClaspCone-1
RIZ65650.1	*Methylococcales bacterium*	Ppx-GppA · HD · TULIP-like · ClaspCone-1
MBN8496278.1	*Accumulibacter sp.*	Ppx-GppA · HD · TULIP-like · ClaspCone-1
WP_169065037.1	*Candidatus Accumulibacter phosphatis*	Ppx-GppA · HD · TULIP-like · ClaspCone-1
MBL8395857.1	*Accumulibacter sp.*	Ppx-GppA · HD · TULIP-like · ClaspCone-1
HCZ15527.1	*Accumulibacter sp.*	Ppx-GppA · HD · TULIP-like · ClaspCone-1
WP_186408100.1	*Candidatus Accumulibacter aalborgensis*	Ppx-GppA · HD · TULIP-like · ClaspCone-1
MCB1942871.1	*Accumulibacter sp.*	Ppx-GppA · HD · TULIP-like · ClaspCone-1
MBK7953273.1	*Candidatus Accumulibacter affinis*	Ppx-GppA · HD · TULIP-like · ClaspCone-1
MCC7183801.1	*Rhodocyclaceae bacterium*	TULIP-like · ClaspCone-1 · Ppx-GppA · HD
MCB1967072.1	*Accumulibacter sp.*	Ppx-GppA · HD · TULIP-like · ClaspCone-1
MBL8396538.1	*Accumulibacter sp.*	Ppx-GppA · HD · TULIP-like · ClaspCone-1
QLH49830.1	*Candidatus Accumulibacter cognatus*	Ppx-GppA · HD · TULIP-like · ClaspCone-1

############;associations with lipid-acting enzymes; #;MBOAT-O-acyltransferase

WP_070049557.1	*Rheinheimera salexigens*	TULIP-like · ClaspCone-1 · MBOAT-O-acyltransferase
PKM19692.1	*Gammaproteobacteria bacterium HGW-Gammaproteobacteria-15*	TULIP-like · ClaspCone-1 · ? · MBOAT-O-acyltransferase
WP_205311249.1	*Rheinheimera maricola*	TULIP-like · ClaspCone-1 · MBOAT-O-acyltransferase
WP_215398440.1	*Rheinheimera oceanensis*	TULIP-like · ClaspCone-1 · ? · MBOAT-O-acyltransferase
WP_218444867.1	*Pacificimonas pallii*	MBOAT-O-acyltransferase · ? · ? · TULIP-like · ClaspCone-1
WP_089996841.1	*Cognatiyoonia koreensis*	MBOAT-O-acyltransferase · ? · ? · ? · TULIP-like · ClaspCone-1

#;GDSL lipase

WP_229585123.1	*Rheinheimera sp. UJ51*	TULIP-like · ClaspCone-1 · Lipase_GDSL
WP_226750831.1	*Alishewanella maricola*	TULIP-like · ClaspCone-1 · Lipase_GDSL
WP_189482718.1	*Alishewanella tabrizica*	TULIP-like · ClaspCone-1 · Lipase_GDSL

Accession	Organism	Domains
MBL8446504.1	*Zoogloeaceae bacterium*	TULIP-like, ClaspCone-1, ?, Lipase_GDSL
TDI73806.1	*Betaproteobacteria bacterium*	TULIP-like, ClaspCone-1, Lipase_GDSL
TFH12685.1	*Nitrosomonadales bacterium*	TULIP-like, ClaspCone-1, Lipase_GDSL
MBL8439147.1	*Zoogloeaceae bacterium*	TULIP-like, ClaspCone-1, ?, Lipase_GDSL
TDI79938.1	*Betaproteobacteria bacterium*	TULIP-like, ClaspCone-1, Lipase_GDSL

#######;additional N-terminal fusion to 5TM region

Accession	Organism	Domains
MBM4092156.1	*Planctomycetota bacterium*	5TM, TULIP-like, ClaspCone-1
MBL9123873.1	*Planctomycetaceae bacterium*	5TM, TULIP-like, ClaspCone-1
QDV42279.1	*Stieleria neptunia*	5TM, TULIP-like, ClaspCone-1
RJP32713.1	*Phycisphaerales bacterium*	5TM, TULIP-like, ClaspCone-1
MBL7153463.1	*Phycisphaerae bacterium*	5TM, TULIP-like, ClaspCone-1
NLY99765.1	*Pirellulaceae bacterium*	5TM, TULIP-like, ClaspCone-1
MBM4028732.1	*Planctomycetota bacterium*	5TM, TULIP-like, ClaspCone-1
WP_145378196.1	*Symmachiella dynata*	5TM, TULIP-like, ClaspCone-1
MAE63896.1	*Phycisphaeraceae bacterium*	5TM, TULIP-like, ClaspCone-1
MCA9101864.1	*Planctomycetales bacterium*	5TM, TULIP-like, ClaspCone-1
MBH05774.1	*Phycisphaeraceae bacterium*	5TM, TULIP-like, ClaspCone-1
NLS92410.1	*Planctomycetaceae bacterium*	5TM, TULIP-like, ClaspCone-1
MAR10335.1	*Blastopirellula sp.*	5TM, TULIP-like, ClaspCone-1
WP_145198391.1	*Thalassoglobus polymorphus*	5TM, TULIP-like, ClaspCone-1
NQV34135.1	*Phycisphaeraceae bacterium*	5TM, TULIP-like, ClaspCone-1
MBL9125357.1	*Planctomycetaceae bacterium*	5TM, TULIP-like, ClaspCone-1
MBX9790365.1	*Pirellulales bacterium*	5TM, TULIP-like, ClaspCone-1
MCC7085534.1	*Pirellulales bacterium*	5TM, TULIP-like, ClaspCone-1
MBI3108813.1	*Candidatus Rokuibacteriota bacterium*	5TM, TULIP-like, ClaspCone-1
MBN1294591.1	*Candidatus Latescibacterota bacterium*	5TM, TULIP-like, ClaspCone-1
UCE46767.1	*Phycisphaerales bacterium*	5TM, TULIP-like, ClaspCone-1
MBX7072636.1	*Pirellulales bacterium*	5TM, TULIP-like, ClaspCone-1
MBX3707172.1	*Pseudomonadales bacterium*	5TM, TULIP-like, ClaspCone-1
MBI2827101.1	*Planctomycetia bacterium*	5TM, TULIP-like, ClaspCone-1
MBN1488751.1	*Phycisphaerae bacterium*	5TM, TULIP-like, ClaspCone-1
MXW71191.1	*Acidobacteriota bacterium*	5TM, TULIP-like, ClaspCone-1

#######;has the N-terminal 5TM region, but lacks the TULIP domain fusion

Accession	Organism	Domains
MCB1373244.1	*Paracoccaceae bacterium*	5TM, ClaspCone-1
QEG00131.1	*Stieleria maiorica*	5TM, ClaspCone-1
WP_145386181.1	*Stieleria neptunia*	5TM, ClaspCone-1
WP_197231749.1	*Novipirellula artificiosorum*	5TM, ClaspCone-1
MBX3463946.1	*Planctomycetota bacterium*	5TM, ClaspCone-1
MBP2678929.1	*Deltaproteobacteria bacterium*	5TM, ClaspCone-1
MBL8869168.1	*Planctomycetaceae bacterium*	5TM, ClaspCone-1
MBL7940080.1	*Flavobacteriales bacterium*	5TM, ClaspCone-1
MBX2962161.1	*Cyclobacteriaceae bacterium*	5TM, ClaspCone-1
MCE2727226.1	*Planctomycetaceae bacterium*	5TM, ClaspCone-1
GDX95609.1	*Planctomycetia bacterium*	5TM, ClaspCone-1
MCC6129469.1	*Acidobacteriota bacterium*	5TM, ClaspCone-1
MAG56696.1	*Planctomycetota bacterium*	5TM, ClaspCone-1
TDJ00870.1	*Caldithrix sp.*	5TM, ClaspCone-1
TVS15157.1	*Planctomycetaceae bacterium*	5TM, ClaspCone-1
MBX9791995.1	*Pirellulales bacterium*	5TM, ClaspCone-1
MBX3417227.1	*Pirellulaceae bacterium*	5TM, ClaspCone-1

##########;TULIP fusion only

Accession	Organism	Domains
WP_226697143.1	*Qipengyuania flava*	TULIP-like, ClaspCone-1
KWV94244.1	*Erythrobacter sp. AP23*	TULIP-like, ClaspCone-1
MSQ68988.1	*Gammaproteobacteria bacterium*	TULIP-like, ClaspCone-1
MBU6272590.1	*Betaproteobacteria bacterium*	TULIP-like, ClaspCone-1
MBK8766509.1	*Burkholderiaceae bacterium*	TULIP-like, ClaspCone-1
WP_224812028.1		TULIP-like, ClaspCone-1
WP_160752978.1	*Qipengyuania algicida*	TULIP-like, ClaspCone-1
PID43469.1	*Pseudomonadota bacterium*	TULIP-like, ClaspCone-1
MCC5827179.1	*Alkalimonas sp.*	TULIP-like, ClaspCone-1
WP_139243694.1	*Alkalimonas amylolytica*	TULIP-like, ClaspCone-1
MBV1878303.1	*Pseudomonadales bacterium*	TULIP-like, ClaspCone-1
WP_090198907.1	*Erythrobacter sp. HL-111*	TULIP-like, ClaspCone-1
WP_133341944.1	*Meridianimarinicoccus aquatilis*	TULIP-like, ClaspCone-1
WP_100656212.1	*Alteromonas flava*	TULIP-like, ClaspCone-1
WP_130637634.1	*Alteromonas sp. KUL49*	TULIP-like, ClaspCone-1
WP_198823725.1	*Paraglaciecola chathamensis*	TULIP-like, ClaspCone-1
MAI38383.1	*Alteromonas sp.*	TULIP-like, ClaspCone-1
WP_203565445.1	*Alteromonas profundi*	TULIP-like, ClaspCone-1
WP_133467978.1	*Paraglaciecola marina*	TULIP-like, ClaspCone-1
WP_102797413.1	*Bowmanella denitrificans*	TULIP-like, ClaspCone-1
WP_206593397.1	*Bowmanella yangjiangensis*	TULIP-like, ClaspCone-1
WP_235313129.1	*Paraglaciecola algarum*	TULIP-like, ClaspCone-1
WP_158771491.1	*Paraglaciecola sp. L1A13*	TULIP-like, ClaspCone-1
WP_007619448.1	*Paraglaciecola arctica BSs20135*	TULIP-like, ClaspCone-1
NCP63371.1	*Paraglaciecola sp.*	TULIP-like, ClaspCone-1
WP_158965844.1	*Paraglaciecola sp. L3A3*	TULIP-like, ClaspCone-1
WP_206574742.1	*Bowmanella dokdonensis*	TULIP-like, ClaspCone-1
WP_068374362.1	*Paraglaciecola hydrolytica*	TULIP-like, ClaspCone-1
WP_099033371.1	*Lacimicrobium alkaliphilum*	TULIP-like, ClaspCone-1
NVK55227.1	*Alteromonadaceae bacterium*	TULIP-like, ClaspCone-1
WP_070176352.1	*Alteromonas lipolytica*	TULIP-like, ClaspCone-1
WP_143834362.1	*Aliiglaciecola sp. M165*	TULIP-like, ClaspCone-1
WP_215930833.1	*Paraglaciecola arctica*	TULIP-like, ClaspCone-1
WP_006013687.1	*Brumicola pallidula DSM 14239 = ACAM 615*	TULIP-like, ClaspCone-1
WP_136250498.1	*Ningiella ruwaisensis*	TULIP-like, ClaspCone-1
MBE1287567.1	*Alteromonadaceae bacterium*	TULIP-like, ClaspCone-1
MBF7071885.1	*Glaciecola sp. MH2013*	TULIP-like, ClaspCone-1
WP_214450611.1	*Glaciecola sp. XM2*	TULIP-like, ClaspCone-1
WP_013785488.1	*Alteromonas naphthalenivorans*	TULIP-like, ClaspCone-1
WP_229162342.1	*Fluctibacter halophilus*	TULIP-like, ClaspCone-1
WP_156077875.1	*Alteromonas macleodii*	TULIP-like, ClaspCone-1
OFC71976.1	*Alteromonas confluentis*	TULIP-like, ClaspCone-1
WP_010180895.1	*Glaciecola sp. HTCC2999*	TULIP-like, ClaspCone-1

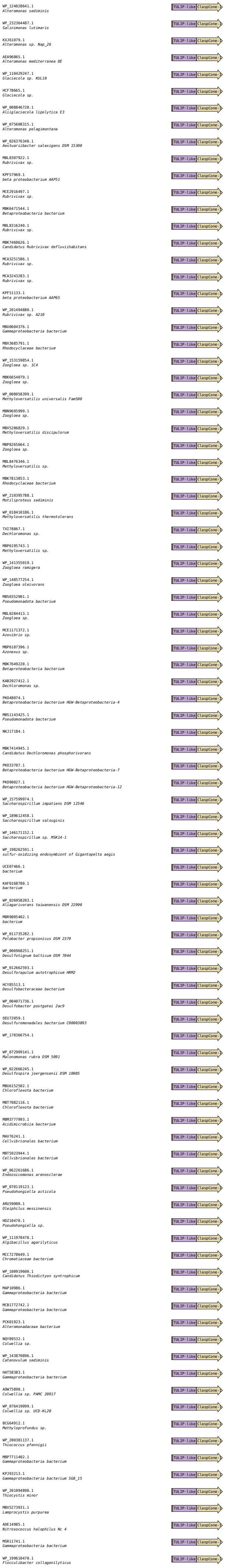
WP_124028641.1
Alteromonas sediminis
WP_232364487.1
Salinimonas lutimaris
KXJ61079.1
Alteromonas sp. Nap_26
AEA96865.1
Alteromonas mediterranea DE
WP_110429247.1
Glaciecola sp. KUL10
HCF78665.1
Glaciecola sp.
WP_008846720.1
Aliiglaciecola lipolytica E3
WP_075608315.1
Alteromonas pelagimontana
WP_026376340.1
Aestuariibacter salexigens DSM 15300
MBL8307922.1
Rubrivivax sp.
KPF57969.1
beta proteobacterium AAP51
MCE2916497.1
Rubrivivax sp.
MBK6471544.1
Betaproteobacteria bacterium
MBL8316240.1
Rubrivivax sp.
MBK7460626.1
Candidatus Rubrivivax defluviihabitans
MCA3251586.1
Rubrivivax sp.
MCA3243283.1
Rubrivivax sp.
KPF51133.1
beta proteobacterium AAP65
WP_201494880.1
Rubrivivax sp. A210
MBU0604376.1
Gammaproteobacteria bacterium
MBX3685791.1
Rhodocyclaceae bacterium
WP_153159854.1
Zoogloea sp. 1C4
MBK6654079.1
Zoogloea sp.
WP_008058399.1
Methyloversatilis universalis Fam500
MBN9695999.1
Zoogloea sp.
MBV5286829.1
Methyloversatilis discipulorum
MBP8265664.1
Zoogloea sp.
MBL8476346.1
Methyloversatilis sp.
MBK7813853.1
Rhodocyclaceae bacterium
WP_210395788.1
Motiliproteus sediminis
WP_018410186.1
Methyloversatilis thermotolerans
TXI78867.1
Dechloromonas sp.
MBP6195743.1
Methyloversatilis sp.
WP_141355019.1
Zoogloea ramigera
WP_148577254.1
Zoogloea oleivorans
MBS0352901.1
Pseudomonadota bacterium
MBL0284413.1
Zoogloea sp.
MCE1171372.1
Azovibrio sp.
MBP6187396.1
Azonexus sp.
MBK7649220.1
Betaproteobacteria bacterium
KAB2927412.1
Dechloromonas sp.
PKO48074.1
Betaproteobacteria bacterium HGW-Betaproteobacteria-4
MBS1143425.1
Pseudomonadota bacterium
NKJ17184.1
MBK7414945.1
Candidatus Dechloromonas phosphorivorans
PKO33707.1
Betaproteobacteria bacterium HGW-Betaproteobacteria-7
PKO90027.1
Betaproteobacteria bacterium HGW-Betaproteobacteria-12
WP_157599974.1
Saccharospirillum impatiens DSM 12546
WP_189612458.1
Saccharospirillum salsuginis
WP_146171152.1
Saccharospirillum sp. MSK14-1
WP_198262591.1
sulfur-oxidizing endosymbiont of Gigantopelta aegis
UCE07466.1
bacterium
KAF0168780.1
bacterium
WP_026958203.1
Aliagarivorans taiwanensis DSM 22990
MBR9805402.1
bacterium
WP_011735282.1
Pelobacter propionicus DSM 2379
WP_006968251.1
Desulfotignum balticum DSM 7044
WP_012662593.1
Desulforapulum autotrophicum HRM2
HCY85513.1
Desulfobacteraceae bacterium
WP_004071736.1
Desulfobacter postgatei 2ac9
OEU72059.1
Desulfuromonadales bacterium C00003093
WP_178366754.1
WP_072909141.1
Malonomonas rubra DSM 5091
WP_022666245.1
Desulfospira joergensenii DSM 10085
MBU6152502.1
Chloroflexota bacterium
MBT7082116.1
Chloroflexota bacterium
MBM3777993.1
Acidimicrobiia bacterium
MAV76241.1
Cellvibrionales bacterium
MBT5923944.1
Cellvibrionales bacterium
WP_062261686.1
Endozoicomonas arenosclerae
WP_070119123.1
Pseudohongiella acticola
ARU59000.1
Oleiphilus messinensis
HDZ10470.1
Pseudohongiella sp.
WP_111978478.1
Algibacillus agarilyticus
MCC7278649.1
Chromatiaceae bacterium
WP_100919600.1
Candidatus Thiodictyon syntrophicum
MAP10986.1
Gammaproteobacteria bacterium
MCB1772742.1
Gammaproteobacteria bacterium
PCK01923.1
Alteromonadaceae bacterium
NQY89532.1
Colwellia sp.
WP_143870896.1
Catenovulum sediminis
HAT58383.1
Gammaproteobacteria bacterium
AOW75898.1
Colwellia sp. PAMC 20917
WP_076419099.1
Colwellia sp. UCD-KL20
BCG64912.1
Methyloprofundus sp.
WP_200381137.1
Thiococcus pfennigii
MBP7711402.1
Gammaproteobacteria bacterium
KPJ93213.1
Gammaproteobacteria bacterium SG8_15
WP_201094898.1
Thiocystis minor
MBV5273931.1
Lamprocystis purpurea
ADE14985.1
Nitrosococcus halophilus Nc 4
MSR11741.1
Gammaproteobacteria bacterium
WP_199610470.1
Flocculibacter collagenilyticus
TULIP-like
ClaspCone-

WP_085210787.1	*Methylomagnum ishizawai*	TULIP-like ClaspCone-
MBA1445663.1	*Gammaproteobacteria bacterium*	TULIP-like ClaspCone-
WP_232768300.1	*Vibrio salinus*	TULIP-like ClaspCone-
WP_158002491.1	*Methyloterricola oryzae*	TULIP-like ClaspCone-
WP_218359786.1	*Enterovibrio paralichthyis*	TULIP-like ClaspCone-
MCB1878190.1	*Chromatiales bacterium*	TULIP-like ClaspCone-
PCI58974.1	*Gammaproteobacteria bacterium*	TULIP-like ClaspCone-
ESQ08515.1	*Thiohalocapsa sp. PB-PSB1*	TULIP-like ClaspCone-
WP_158658006.1	*Agarilytica rhodophyticola*	TULIP-like ClaspCone-
HAT42267.1	*Rheinheimera sp.*	TULIP-like ClaspCone-
WP_210205340.1	*Vibrio sp. B1REV9*	TULIP-like ClaspCone-
NQZ83854.1	*Colwellia sp.*	TULIP-like ClaspCone-
MCB1691023.1	*Halioglobus sp.*	TULIP-like ClaspCone-
MBT7334432.1	*Gammaproteobacteria bacterium*	TULIP-like ClaspCone-
HGX94037.1	*Candidatus Tenderia sp.*	TULIP-like ClaspCone-
MBU1439562.1	*Gammaproteobacteria bacterium*	TULIP-like ClaspCone-
MBK6739325.1	*Haliea sp.*	TULIP-like ClaspCone-
HCH20254.1	*Cellvibrionales bacterium*	TULIP-like ClaspCone-
HEC30343.1	*Gammaproteobacteria bacterium*	TULIP-like ClaspCone-
WP_159657842.1	*Methylicorpusculum oleiharenae*	TULIP-like ClaspCone-
WP_013816944.1	*Methylomonas methanica MC09*	TULIP-like ClaspCone-
WP_189379023.1	*Thalassotalea profundi*	TULIP-like ClaspCone-
WP_206486400.1	*Thalassotalea sp. G2M2-11*	TULIP-like ClaspCone-
MBQ85814.1	*Gammaproteobacteria bacterium*	TULIP-like ClaspCone-
WP_147302684.1	*Thalassotalea euphylliae*	TULIP-like ClaspCone-
WP_169075867.1	*Thalassotalea algicola*	TULIP-like ClaspCone-
WP_068061645.1	*Rheinheimera sp. SA_1*	TULIP-like ClaspCone-
MBS3952159.1	*Methylomicrobium sp.*	TULIP-like ClaspCone-
WP_138320414.1	*Thalassotalea litorea*	TULIP-like ClaspCone-
WP_057956499.1	*endosymbiont of Ridgeia piscesae*	TULIP-like ClaspCone-
HHI92088.1	*Gammaproteobacteria bacterium*	TULIP-like ClaspCone-
WP_064029937.1	*Methylomonas koyamae*	TULIP-like ClaspCone-
WP_010959707.1	*Methylococcus capsulatus str. Bath*	TULIP-like ClaspCone-
WP_051479664.1	*Catenovulum agarivorans DS-2*	TULIP-like ClaspCone-
WP_184423230.1	*Thalassotalea piscium*	TULIP-like ClaspCone-
WP_232322393.1	*Methylomonas lenta*	TULIP-like ClaspCone-
WP_034873241.1	*Endozoicomonas montiporae CL-33*	TULIP-like ClaspCone-
WP_208933238.1	*Rheinheimera sp. F8*	TULIP-like ClaspCone-
WP_074496671.1	*Thalassotalea sp. PP2-459*	TULIP-like ClaspCone-
NRA62454.1	*Psychrobium sp.*	TULIP-like ClaspCone-
WP_165312855.1	*Vibrio ziniensis*	TULIP-like ClaspCone-
RLA57176.1	*Gammaproteobacteria bacterium*	TULIP-like ClaspCone-
WP_156302372.1	*Methylogaea oryzae JCM 16910*	TULIP-like ClaspCone-
WP_043751531.1	*Imhoffiella purpurea*	TULIP-like ClaspCone-
WP_094786660.1	*Zooshikella ganghwensis*	TULIP-like ClaspCone-
WP_166991495.1	*Pseudomaricurvus alkylphenolicus*	TULIP-like ClaspCone-
HCU64822.1	*Rheinheimera sp.*	TULIP-like ClaspCone-
WP_212716558.1	*Zooshikella ganghwensis*	TULIP-like ClaspCone-
WP_017839855.1	*Methylotuvimicrobium buryatense*	TULIP-like ClaspCone-
WP_017444157.1	*Gayadomonas joobiniege G7*	TULIP-like ClaspCone-
PIE43751.1	*Gammaproteobacteria bacterium*	TULIP-like ClaspCone-
KRO72187.1	*OM182 bacterium BACL3 MAG-120507-bin80*	TULIP-like ClaspCone-
WP_048690617.1	*Catenovulum maritimum*	TULIP-like ClaspCone-
WP_058022421.1	*Pseudohongiella spirulinae*	TULIP-like ClaspCone-
MBK52428.1	*Gammaproteobacteria bacterium*	TULIP-like ClaspCone-
MBM4229067.1	*Gammaproteobacteria bacterium*	TULIP-like ClaspCone-
MCB1920736.1	*Candidatus Competibacteraceae bacterium*	TULIP-like ClaspCone-
MBN2699849.1	*Methylothermaceae bacterium*	TULIP-like ClaspCone-
WP_051685935.1	*Rheinheimera texasensis DSM 17496*	TULIP-like ClaspCone-
PZN78427.1	*Candidatus Methylumidiphilus alinenensis*	TULIP-like ClaspCone-
WP_007040397.1	*Thiorhodococcus drewsii AZ1*	TULIP-like ClaspCone-
NNJ92991.1	*Gammaproteobacteria bacterium*	TULIP-like ClaspCone-
TXH93756.1	*Rheinheimera sp.*	TULIP-like ClaspCone-
WP_200207379.1		TULIP-like ClaspCone-
MCB1785973.1	*Gammaproteobacteria bacterium*	TULIP-like ClaspCone-
HCB13638.1	*Gammaproteobacteria bacterium*	TULIP-like ClaspCone-
MCB1922611.1	*Gammaproteobacteria bacterium*	TULIP-like ClaspCone-
WP_163130181.1	*Agarivorans sp. Alg241-V36*	TULIP-like ClaspCone-
MBT8140111.1	*Gammaproteobacteria bacterium*	TULIP-like ClaspCone-
MBQ77226.1	*Cellvibrionales bacterium*	TULIP-like ClaspCone-
WP_132978228.1	*Thiobaca trueperi*	TULIP-like ClaspCone-
TAK63379.1	*Methylobacter sp.*	TULIP-like ClaspCone-
WP_132229087.1	*Marichromatium gracile*	TULIP-like ClaspCone-
MBU2115110.1	*Gammaproteobacteria bacterium*	TULIP-like ClaspCone-
NEX15867.1	*Halochromatium sp.*	TULIP-like ClaspCone-
NBV76335.1	*Methylococcaceae bacterium*	TULIP-like ClaspCone-
MSR13168.1	*Gammaproteobacteria bacterium*	TULIP-like ClaspCone-
NBC14045.1	*Gammaproteobacteria bacterium*	TULIP-like ClaspCone-
MCB1765328.1	*Candidatus Competibacteraceae bacterium*	TULIP-like ClaspCone-
MCB1618949.1	*Pseudomonadales bacterium*	TULIP-like ClaspCone-
HHI76830.1	*Gammaproteobacteria bacterium*	TULIP-like ClaspCone-
RDH82652.1	*endosymbiont of Galathealinum brachiosum*	TULIP-like ClaspCone-
HHX83220.1	*Pseudomonadaceae bacterium*	TULIP-like ClaspCone-
RKZ96888.1	*Gammaproteobacteria bacterium*	TULIP-like ClaspCone-
WP_036519858.1	*Nitrincola sp. A-D6*	TULIP-like ClaspCone-
MBL4781827.1	*Porticoccaceae bacterium*	TULIP-like ClaspCone-
RMG34282.1	*Gammaproteobacteria bacterium*	TULIP-like ClaspCone-
WP_058553759.1	*Thiohalocapsa sp. ML1*	TULIP-like ClaspCone-
KXJ40092.1	*Methylothermaceae bacteria B42*	TULIP-like ClaspCone-
MCB1694775.1	*Halioglobus sp.*	TULIP-like ClaspCone-
NCC28313.1	*Gammaproteobacteria bacterium*	TULIP-like ClaspCone-
NRA71363.1	*Gammaproteobacteria bacterium*	TULIP-like ClaspCone-
MBU1666416.1	*Gammaproteobacteria bacterium*	TULIP-like ClaspCone-

OED48289.1	*Endozoicomonas sp. (ex Bugula neritina AB1)*	TULIP-like ClaspCone-
NDE33659.1	*Gammaproteobacteria bacterium*	TULIP-like ClaspCone-
WP_139243689.1	*Alkalimonas amylolytica*	TULIP-like ClaspCone-
MCC5827070.1	*Alkalimonas sp.*	TULIP-like ClaspCone-
WP_137674449.1	*Agarivorans sp. Toyoura001*	TULIP-like ClaspCone-
WP_210560951.1	*Psychrosphaera aestuarii*	TULIP-like ClaspCone-
OUS13565.1	*Gammaproteobacteria bacterium 53_120_T64*	TULIP-like ClaspCone-
WP_209112186.1	*Endozoicomonas sp. G2_1*	TULIP-like ClaspCone-
MCC7120812.1	*Gammaproteobacteria bacterium*	TULIP-like ClaspCone-
WP_164450921.1	*Thiorhodococcus minor*	TULIP-like ClaspCone-
MCB1672252.1	*Pseudomonadales bacterium*	TULIP-like ClaspCone-
WP_114697218.1	*Motiliproteus coralliicola*	TULIP-like ClaspCone-
MCB1864388.1	*Chromatiales bacterium*	TULIP-like ClaspCone-
WP_041603840.1	*Thioflavicoccus mobilis 8321*	TULIP-like ClaspCone-
WP_186508144.1	*Neptunicella marina*	TULIP-like ClaspCone-
WP_200154634.1	*Chromatium okenii*	TULIP-like ClaspCone-
RLA47238.1	*Gammaproteobacteria bacterium*	TULIP-like ClaspCone-
WP_232056817.1	*Methylomonas sp. HYX-M1*	TULIP-like ClaspCone-
WP_064035318.1	*Methylomonas methanica*	TULIP-like ClaspCone-
WP_152646514.1		TULIP-like ClaspCone-
OUS18050.1	*Gammaproteobacteria bacterium 50_400_T64*	TULIP-like ClaspCone-
WP_200376552.1	*Thiocystis violacea*	TULIP-like ClaspCone-
MCC6713962.1	*Gammaproteobacteria bacterium*	TULIP-like ClaspCone-
MCB1738475.1	*Gammaproteobacteria bacterium*	TULIP-like ClaspCone-
NBC47125.1	*Gammaproteobacteria bacterium*	TULIP-like ClaspCone-
WP_152647083.1	*Thalassomonas viridans*	TULIP-like ClaspCone-
HAZ42044.1	*Methylococcaceae bacterium*	TULIP-like ClaspCone-
MBL6999626.1	*Gammaproteobacteria bacterium*	TULIP-like ClaspCone-
HEC16403.1	*Sedimenticola sp.*	TULIP-like ClaspCone-
WP_207611487.1	*Alteromonas sp. 5E99-2*	TULIP-like ClaspCone-
MBP6682485.1	*Halioglobus sp.*	TULIP-like ClaspCone-
PCJ27304.1	*SAR86 cluster bacterium*	TULIP-like ClaspCone-
WP_093190272.1		TULIP-like ClaspCone-
MBT5229200.1	*Methylococcales bacterium*	TULIP-like ClaspCone-
WP_131259595.1	*Pseudobowmanella zhangzhouensis*	TULIP-like ClaspCone-
TDF38364.1	*Alteromonadaceae bacterium M269*	TULIP-like ClaspCone-
WP_208831065.1	*Psychrosphaera ytuae*	TULIP-like ClaspCone-
WP_019528350.1	*Dasania marina DSM 21967*	TULIP-like ClaspCone-
RYU62396.1	*Methylolobus aquaticus*	TULIP-like ClaspCone-
WP_153250755.1	*Candidatus Methylospira mobilis*	TULIP-like ClaspCone-
WP_006007131.1	*Glaciecola punicea ACAM 611*	TULIP-like ClaspCone-
NJD06959.1	*Methylococcaceae bacterium*	TULIP-like ClaspCone-
HAJ91212.1	*Gammaproteobacteria bacterium*	TULIP-like ClaspCone-
WP_012969761.1	*Allochromatium vinosum DSM 180*	TULIP-like ClaspCone-
HED16578.1	*Gammaproteobacteria bacterium*	TULIP-like ClaspCone-
MCB1693122.1	*Pseudomonadales bacterium*	TULIP-like ClaspCone-
WP_200332797.1	*Thiocystis violacea*	TULIP-like ClaspCone-
WP_046556146.1	*Arsukibacterium ikkense*	TULIP-like ClaspCone-
WP_215962510.1	*Psychrosphaera sp. B3R10*	TULIP-like ClaspCone-
WP_019674643.1	*Arsukibacterium perlucidum DSM 18276*	TULIP-like ClaspCone-
WP_194971196.1	*Methylobacter sp. BlB1*	TULIP-like ClaspCone-
MCB1631867.1	*Pseudomonadales bacterium*	TULIP-like ClaspCone-
MBT8146162.1	*Gammaproteobacteria bacterium*	TULIP-like ClaspCone-
MBR9912849.1	*Gammaproteobacteria bacterium*	TULIP-like ClaspCone-
WP_180567857.1	*Spartinivicinus marinus*	TULIP-like ClaspCone-
GFZ78491.1	*Pseudohongiella nitratireducens*	TULIP-like ClaspCone-
WP_163833453.1	*Spartinivicinus ruber*	TULIP-like ClaspCone-
MCB1662651.1	*Pseudomonadales bacterium*	TULIP-like ClaspCone-
WP_189774993.1	*Thalassotalea marina*	TULIP-like ClaspCone-
RLA19779.1	*Gammaproteobacteria bacterium*	TULIP-like ClaspCone-
WP_119631773.1	*Methylocaldum marinum*	TULIP-like ClaspCone-
WP_091334968.1	*Allochromatium warmingii*	TULIP-like ClaspCone-
MBT9502856.1	*Burkholderiaceae bacterium*	TULIP-like ClaspCone-
WP_133699880.1	*Roseateles toxinivorans*	TULIP-like ClaspCone-
MCC7547113.1	*Burkholderiales bacterium*	TULIP-like ClaspCone-
WP_201209785.1	*Rhodocyclus purpureus*	TULIP-like ClaspCone-
WP_090827779.1	*Nitrosovibrio tenuis*	TULIP-like ClaspCone-
WP_074859442.1	*Nitrosospira briensis*	TULIP-like ClaspCone-
WP_090540565.1	*Nitrosomonas sp. Nm132*	TULIP-like ClaspCone-
TXI26330.1	*Nitrosomonas sp.*	TULIP-like ClaspCone-
WP_172631204.1		TULIP-like ClaspCone-
MBA4142350.1	*Nitrosospira sp.*	TULIP-like ClaspCone-
MBE7527953.1	*Burkholderiales bacterium*	TULIP-like ClaspCone-
WP_090413531.1	*Nitrosomonas halophila*	TULIP-like ClaspCone-
MBL8335240.1	*Rhodoferax sp.*	TULIP-like ClaspCone-
MBY0484063.1	*Nitrosomonas sp.*	TULIP-like ClaspCone-
WP_160809627.1		TULIP-like ClaspCone-

####;beta-rich fusions

MCC6531401.1	*Burkholderiales bacterium*	TULIP-like ClaspCone-
MBC6962655.1	*Nitrosomonas sp.*	TULIP-like ClaspCone-
WP_198111107.1	*Inhella proteolytica*	TULIP-like ClaspCone-
WP_200384062.1	*Rhodocyclus tenuis*	TULIP-like ClaspCone-
ODT80525.1	*Nitrosomonadales bacterium SCN 54-20*	TULIP-like ClaspCone-
MBK9359651.1	*Rubrivivax sp.*	TULIP-like ClaspCone-
WP_212345478.1	*Azoarcus sp. L1K30*	TULIP-like ClaspCone-
MCB1948695.1	*Nitrosomonas sp.*	TULIP-like ClaspCone-
NDG38921.1	*Betaproteobacteria bacterium*	TULIP-like ClaspCone-
WP_183106675.1	*Mitsuaria sp. WAJ17*	TULIP-like ClaspCone-
WP_092754803.1	*Rhodoferax sp. OV413*	TULIP-like ClaspCone-
WP_074974835.1	*Nitrosospira multiformis*	TULIP-like ClaspCone-
MBL8444088.1	*Zoogloeaceae bacterium*	TULIP-like ClaspCone-
MBK8119435.1	*Sulfuritalea sp.*	TULIP-like ClaspCone-
MBC7680884.1	*Rhodoferax sp.*	TULIP-like ClaspCone-

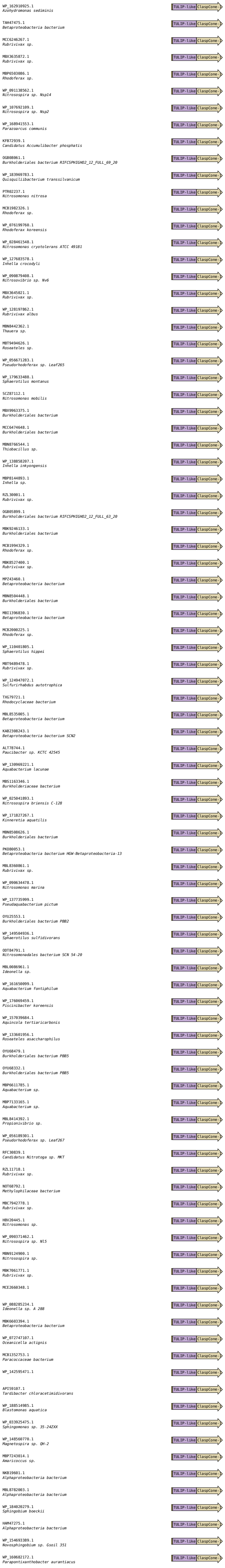
WP_162910925.1
Azohydromonas sediminis
TULIP-like ClaspCone-
TAH47475.1
Betaproteobacteria bacterium
TULIP-like ClaspCone-
MCC6246267.1
Rubrivivax sp.
TULIP-like ClaspCone-
MBX3635872.1
Rubrivivax sp.
TULIP-like ClaspCone-
MBP6503086.1
Rhodoferax sp.
TULIP-like ClaspCone-
WP_091138562.1
Nitrosospira sp. Nsp14
TULIP-like ClaspCone-
WP_107692109.1
Nitrosospira sp. Nsp2
TULIP-like ClaspCone-
WP_168941553.1
Parazoarcus communis
TULIP-like ClaspCone-
KFB72939.1
Candidatus Accumulibacter phosphatis
TULIP-like ClaspCone-
OGB08061.1
Burkholderiales bacterium RIFCSPHIGHO2_12_FULL_69_20
TULIP-like ClaspCone-
WP_183969783.1
Quisquiliibacterium transsilvanicum
TULIP-like ClaspCone-
PTR02237.1
Nitrosomonas nitrosa
TULIP-like ClaspCone-
MCB1982326.1
Rhodoferax sp.
TULIP-like ClaspCone-
WP_076199760.1
Rhodoferax koreensis
TULIP-like ClaspCone-
WP_028461548.1
Nitrosomonas cryotolerans ATCC 49181
TULIP-like ClaspCone-
WP_127683578.1
Inhella crocodyli
TULIP-like ClaspCone-
WP_090879408.1
Nitrosovibrio sp. Nv6
TULIP-like ClaspCone-
MBX3645021.1
Rubrivivax sp.
TULIP-like ClaspCone-
WP_128197862.1
Rubrivivax albus
TULIP-like ClaspCone-
MBN8442362.1
Thauera sp.
TULIP-like ClaspCone-
MBT9494626.1
Roseateles sp.
TULIP-like ClaspCone-
WP_056671283.1
Pseudorhodoferax sp. Leaf265
TULIP-like ClaspCone-
WP_179633488.1
Sphaerotilus montanus
TULIP-like ClaspCone-
SCZ87112.1
Nitrosomonas mobilis
TULIP-like ClaspCone-
MBX9963375.1
Burkholderiales bacterium
TULIP-like ClaspCone-
MCC6474648.1
Burkholderiales bacterium
TULIP-like ClaspCone-
MBN8766544.1
Thiobacillus sp.
TULIP-like ClaspCone-
WP_138858207.1
Inhella inkyongensis
TULIP-like ClaspCone-
MBP8144893.1
Inhella sp.
TULIP-like ClaspCone-
RZL30001.1
Rubrivivax sp.
TULIP-like ClaspCone-
OGB05899.1
Burkholderiales bacterium RIFCSPHIGHO2_12_FULL_63_20
TULIP-like ClaspCone-
MBK9246133.1
Burkholderiales bacterium
TULIP-like ClaspCone-
MCB1994329.1
Rhodoferax sp.
TULIP-like ClaspCone-
MBK8527400.1
Rubrivivax sp.
TULIP-like ClaspCone-
MPZ43460.1
Betaproteobacteria bacterium
TULIP-like ClaspCone-
MBN8504448.1
Burkholderiales bacterium
TULIP-like ClaspCone-
MBI1396830.1
Betaproteobacteria bacterium
TULIP-like ClaspCone-
MCB2000225.1
Rhodoferax sp.
TULIP-like ClaspCone-
WP_110401805.1
Sphaerotilus hippei
TULIP-like ClaspCone-
MBT9489478.1
Rubrivivax sp.
TULIP-like ClaspCone-
WP_124947072.1
Sulfurirhabdus autotrophica
TULIP-like ClaspCone-
TXG79721.1
Rhodocyclaceae bacterium
TULIP-like ClaspCone-
MBL8535005.1
Betaproteobacteria bacterium
TULIP-like ClaspCone-
KAB2308243.1
Betaproteobacteria bacterium SCN2
TULIP-like ClaspCone-
ALT78744.1
Paucibacter sp. KCTC 42545
TULIP-like ClaspCone-
WP_130969221.1
Aquabacterium lacunae
TULIP-like ClaspCone-
MBS1163346.1
Burkholderiaceae bacterium
TULIP-like ClaspCone-
WP_025041893.1
Nitrosospira briensis C-128
TULIP-like ClaspCone-
WP_171827267.1
Kinneretia aquatilis
TULIP-like ClaspCone-
MBN8508626.1
Burkholderiales bacterium
TULIP-like ClaspCone-
PKO80053.1
Betaproteobacteria bacterium HGW-Betaproteobacteria-13
TULIP-like ClaspCone-
MBL8360861.1
Rubrivivax sp.
TULIP-like ClaspCone-
WP_090634478.1
Nitrosomonas marina
TULIP-like ClaspCone-
WP_137735999.1
Pseudaquabacterium pictum
TULIP-like ClaspCone-
OYU25553.1
Burkholderiales bacterium PBB2
TULIP-like ClaspCone-
WP_149504936.1
Sphaerotilus sulfidivorans
TULIP-like ClaspCone-
ODT84791.1
Nitrosomonadales bacterium SCN 54-20
TULIP-like ClaspCone-
MBL0086961.1
Ideonella sp.
TULIP-like ClaspCone-
WP_161650099.1
Aquabacterium fontiphilum
TULIP-like ClaspCone-
WP_176069459.1
Piscinibacter koreensis
TULIP-like ClaspCone-
WP_157039684.1
Aquincola tertiaricarbonis
TULIP-like ClaspCone-
WP_133601956.1
Roseateles asaccharophilus
TULIP-like ClaspCone-
OYU68479.1
Burkholderiales bacterium PBB5
TULIP-like ClaspCone-
OYU68332.1
Burkholderiales bacterium PBB5
TULIP-like ClaspCone-
MBP6611785.1
Aquabacterium sp.
TULIP-like ClaspCone-
MBP7133165.1
Aquabacterium sp.
TULIP-like ClaspCone-
MBL8414392.1
Propionivibrio sp.
TULIP-like ClaspCone-
WP_056189301.1
Pseudorhodoferax sp. Leaf267
TULIP-like ClaspCone-
RFC30839.1
Candidatus Nitrotoga sp. MKT
TULIP-like ClaspCone-
RZL11718.1
Rubrivivax sp.
TULIP-like ClaspCone-
NOT68792.1
Methylophilaceae bacterium
TULIP-like ClaspCone-
MBC7942778.1
Rubrivivax sp.
TULIP-like ClaspCone-
HBV20445.1
Nitrosomonas sp.
TULIP-like ClaspCone-
WP_090371462.1
Nitrosospira sp. Nl5
TULIP-like ClaspCone-
MBN9124900.1
Nitrosospira sp.
TULIP-like ClaspCone-
MBK7061771.1
Rubrivivax sp.
TULIP-like ClaspCone-
MCE2660348.1
TULIP-like ClaspCone-
WP_088285234.1
Ideonella sp. A 288
TULIP-like ClaspCone-
MBK6603394.1
Betaproteobacteria bacterium
TULIP-like ClaspCone-
WP_072747107.1
Oceanicella actignis
TULIP-like ClaspCone-
MCB1352753.1
Paracoccaceae bacterium
TULIP-like ClaspCone-
WP_142595471.1
TULIP-like ClaspCone-
API59107.1
Tardibacter chloracetimidivorans
TULIP-like ClaspCone-
WP_188514985.1
Blastomonas aquatica
TULIP-like ClaspCone-
WP_033925475.1
Sphingomonas sp. 35-24ZXX
TULIP-like ClaspCone-
WP_148560770.1
Magnetospira sp. QH-2
TULIP-like ClaspCone-
MBP7243014.1
Amaricoccus sp.
TULIP-like ClaspCone-
NKB19601.1
Alphaproteobacteria bacterium
TULIP-like ClaspCone-
MBL8782003.1
Alphaproteobacteria bacterium
TULIP-like ClaspCone-
WP_184020279.1
Sphingobium boeckii
TULIP-like ClaspCone-
HAM47275.1
Alphaproteobacteria bacterium
TULIP-like ClaspCone-
WP_154693389.1
Novosphingobium sp. Gsoil 351
TULIP-like ClaspCone-
WP_160682172.1
Parapontixanthobacter aurantiacus
TULIP-like ClaspCone-

MCB2102319.1	*Rhodobacterales bacterium*	TULIP-like ClaspCone-
OYU16428.1	*Alphaproteobacteria bacterium PA4*	TULIP-like ClaspCone-
WP_152414453.1	*Blastomonas sp. AAP53*	TULIP-like ClaspCone-
OWV32243.1	*Pacificimonas flava*	TULIP-like ClaspCone-
WP_054136026.1	*Blastomonas sp. AAP25*	TULIP-like ClaspCone-
WP_008603845.1	*Pacificimonas flava*	TULIP-like ClaspCone-
TVQ57168.1	*Paracoccaceae bacterium*	TULIP-like ClaspCone-
MBN9505623.1	*Altererythrobacter sp.*	TULIP-like ClaspCone-
RJT25203.1	*Chakrabartia godavariana*	TULIP-like ClaspCone-
MBM3488280.1	*Alphaproteobacteria bacterium*	TULIP-like ClaspCone-
WP_129791935.1	*Sphingosinicella sp. CPCC 101087*	TULIP-like ClaspCone-
WP_184153695.1	*Amaricoccus macauensis*	TULIP-like ClaspCone-
NQU71125.1		TULIP-like ClaspCone-
WP_192126722.1		TULIP-like ClaspCone-
WP_162301998.1	*Croceibacterium ferulae*	TULIP-like ClaspCone-
WP_185965048.1	*Glacieibacterium frigidum*	TULIP-like ClaspCone-
WP_022673960.1	*Novosphingopyxis baekryungensis DSM 16222*	TULIP-like ClaspCone-
MBK8629860.1	*Sphingomonadales bacterium*	TULIP-like ClaspCone-
PZQ52504.1	*Rhodovulum sulfidophilum*	TULIP-like ClaspCone-
WP_160593693.1	*Sphingomonas changnyeongensis*	TULIP-like ClaspCone-
WP_201158267.1	*Rhodobaculum claviforme*	TULIP-like ClaspCone-
OSZ70933.1	*Sphingomonas sp. IBVSS1*	TULIP-like ClaspCone-
MBD21722.1	*Rhodospirillaceae bacterium*	TULIP-like ClaspCone-
MBT5047722.1	*Rhodospirillaceae bacterium*	TULIP-like ClaspCone-
WP_086054791.1	*Pseudoruegeria sp. SK021*	TULIP-like ClaspCone-
OYW44115.1	*Sphingomonadales bacterium 12-68-11*	TULIP-like ClaspCone-
WP_092683847.1	*Albimonas donghaensis*	TULIP-like ClaspCone-
WP_160756163.1	*Allopontixanthobacter sediminis*	TULIP-like ClaspCone-
WP_092861103.1	*Albimonas pacifica*	TULIP-like ClaspCone-
NOT41685.1	*Alphaproteobacteria bacterium*	TULIP-like ClaspCone-
WP_195822439.1	*Roseobacter sp. MH60115*	TULIP-like ClaspCone-
WP_160754170.1	*Qipengyuania algicida*	TULIP-like ClaspCone-
MBT5812504.1	*Rhodospirillaceae bacterium*	TULIP-like ClaspCone-
MBJ6122448.1	*Sphingomonas mollis*	TULIP-like ClaspCone-
WP_126174502.1	*Altericroceibacterium xinjiangense*	TULIP-like ClaspCone-
WP_184113081.1	*Sphingomonas abaci*	TULIP-like ClaspCone-
WP_092863553.1	*Albimonas pacifica*	TULIP-like ClaspCone-
WP_234869158.1	*Allitabrizicola rongguiensis*	TULIP-like ClaspCone-
MBT7975386.1	*Rhodospirillaceae bacterium*	TULIP-like ClaspCone-
WP_111403713.1	*Meridianimarinicoccus zhengii*	TULIP-like ClaspCone-
MAM68377.1	*Rhodospirillaceae bacterium*	TULIP-like ClaspCone-
MBL8648070.1	*Sphingosinicella sp.*	TULIP-like ClaspCone-
PZQ95498.1	*Cereibacter sphaeroides*	TULIP-like ClaspCone-
WP_160599445.1	*Allopontixanthobacter confluentis*	TULIP-like ClaspCone-
ESZ87681.1	*Blastomonas sp. CACIA14H2*	TULIP-like ClaspCone-
MBL9068550.1	*Sphingopyxis sp.*	TULIP-like ClaspCone-
MBL4721043.1	*Alphaproteobacteria bacterium*	TULIP-like ClaspCone-
MSP42397.1	*Alphaproteobacteria bacterium*	TULIP-like ClaspCone-
RKS88162.1	*Sphingosinicella microcystinivorans*	TULIP-like ClaspCone-
WP_149780583.1	*Roseovarius litoreus*	TULIP-like ClaspCone-
MBK9324923.1	*Hydrogenophilales bacterium*	TULIP-like ClaspCone-
NWG39006.1	*Hydrogenophilaceae bacterium*	TULIP-like ClaspCone-
OYY94281.1	*Hydrogenophilales bacterium 28-61-23*	TULIP-like ClaspCone-
MBI3710005.1	*Pseudomonadota bacterium*	TULIP-like ClaspCone-
TDJ62275.1	*Pseudomonadota bacterium*	TULIP-like ClaspCone-
MBU0971408.1	*Pseudomonadota bacterium*	TULIP-like ClaspCone-
MBU1581128.1	*Pseudomonadota bacterium*	TULIP-like ClaspCone-
OQW75194.1	*Proteobacteria bacterium ST_bin11*	TULIP-like ClaspCone-
MBE9503796.1	*Pseudomonadota bacterium*	TULIP-like ClaspCone-
MCA0241899.1	*Pseudomonadota bacterium*	TULIP-like ClaspCone-
MBS1213909.1	*Pseudomonadota bacterium*	TULIP-like ClaspCone-
MBI3432121.1	*Hydrogenophilales bacterium*	TULIP-like ClaspCone-
OYY61986.1	*Hydrogenophilales bacterium 28-61-11*	TULIP-like ClaspCone-
MBS1176659.1	*Pseudomonadota bacterium*	TULIP-like ClaspCone-
MBS0591983.1	*Pseudomonadota bacterium*	TULIP-like ClaspCone-
MBJ7722182.1		TULIP-like ClaspCone-
MBA2857223.1		TULIP-like ClaspCone-
HGE90414.1		TULIP-like ClaspCone-
HGV63414.1		TULIP-like ClaspCone-
MBI3271286.1	*Planctomycetota bacterium*	TULIP-like ClaspCone-
NOG84668.1	*Planctomycetota bacterium*	TULIP-like ClaspCone-
MBI5365567.1	*Planctomycetota bacterium*	TULIP-like ClaspCone-
MBM3998988.1	*Planctomycetota bacterium*	TULIP-like ClaspCone-
NLY00158.1	*Rhodopirellula sp.*	TULIP-like ClaspCone-
MBI5739645.1	*Nitrospirota bacterium*	TULIP-like ClaspCone-
NOY83434.1	*Candidatus Manganitrophaceae bacterium*	TULIP-like ClaspCone-
MBI5408674.1	*Nitrospirota bacterium*	TULIP-like ClaspCone-
RJQ49857.1	*Nitrospiraceae bacterium*	TULIP-like ClaspCone-
MBI4686892.1	*Nitrospirota bacterium*	TULIP-like ClaspCone-
MBC7562620.1	*Gemmatimonadaceae bacterium*	TULIP-like ClaspCone-
MBU6365096.1	*Gemmatimonadota bacterium*	TULIP-like ClaspCone-
MBX2989728.1	*Bacteroidota bacterium*	TULIP-like ClaspCone-
OGQ93299.1	*Deltaproteobacteria bacterium RIFOXYC2_FULL_48_10*	TULIP-like ClaspCone-
TMB73970.1	*Deltaproteobacteria bacterium*	TULIP-like ClaspCone-
MBM4264004.1	*Deltaproteobacteria bacterium*	TULIP-like ClaspCone-
MBP79539.1	*Deltaproteobacteria bacterium*	TULIP-like ClaspCone-
MSP38298.1	*Deltaproteobacteria bacterium*	TULIP-like ClaspCone-
PCI28320.1	*SAR324 cluster bacterium*	TULIP-like ClaspCone-
MBM2803852.1	*Deltaproteobacteria bacterium*	TULIP-like ClaspCone-
MBM4298296.1	*Deltaproteobacteria bacterium*	TULIP-like ClaspCone-
MBI3064336.1	*Deltaproteobacteria bacterium*	TULIP-like ClaspCone-
MCB9502881.1	*Deferribacteres bacterium*	TULIP-like ClaspCone-
KAA3614044.1	*Calditrichota bacterium*	TULIP-like ClaspCone-

MCC7007717.1	*Acidobacteriota bacterium*	TULIP-like · ClaspCone-1
HGH07338.1		TULIP-like · ClaspCone-1
MBI5628543.1	*Candidatus Rokuibacteriota bacterium*	TULIP-like · ClaspCone-1
MYA35939.1	*Gammaproteobacteria bacterium*	TULIP-like · ClaspCone-1
HBX38180.1	*Pseudohongiella sp.*	TULIP-like · ClaspCone-1
MAM71291.1	*Gammaproteobacteria bacterium*	TULIP-like · ClaspCone-1
NRB81439.1	*Saccharospirillaceae bacterium*	TULIP-like · ClaspCone-1
WP_044618440.1	*Gynuella sunshinyii YC6258*	TULIP-like · ClaspCone-1

#;fragments

MBL4574143.1	*Gammaproteobacteria bacterium*	ClaspCone-1
OYW32325.1	*Methyloversatilis sp. 12-65-5*	ClaspCone-1
MBC7208893.1	*Methyloversatilis sp.*	ClaspCone-1
WP_196490389.1	*Erythrobacter donghaensis*	TULIP-l

#############;lacks TULIP fusion

HHI33201.1		ClaspCone-1
MBS35316.1	*Thiotrichales bacterium*	ClaspCone-1
MBL6987197.1	*Methylobacter sp.*	ClaspCone-1
MBU4500575.1	*Gammaproteobacteria bacterium*	ClaspCone-1
MBU1697468.1	*Pseudomonadota bacterium*	ClaspCone-1
WP_148307750.1	*endosymbiont of unidentified scaly snail isolate Monju*	ClaspCone-1
KTE55442.1	*Sphingopyxis sp. H073*	ClaspCone-1
NOR68354.1	*Methylomarinum sp.*	ClaspCone-1
WP_146675740.1	*Pirellula sp. SH-Sr6A*	ClaspCone-1
PQJ97269.1	*Chromatium okenii*	ClaspCone-1
MBL4821011.1	*Gammaproteobacteria bacterium*	ClaspCone-1
NJK89107.1	*Myxococcales bacterium*	ClaspCone-1
WP_058275778.1	*Ruegeria atlantica*	ClaspCone-1
MSP67670.1	*Alphaproteobacteria bacterium*	ClaspCone-1
WP_232283470.1	*Sphingomonas sp. PAMC 26617*	ClaspCone-1
MCB1041266.1	*Acidimicrobiales bacterium*	ClaspCone-1
HAK63583.1	*Alphaproteobacteria bacterium*	ClaspCone-1
NLF07756.1	*Pirellulaceae bacterium*	ClaspCone-1
WP_198669458.1	*Blastomonas sp. UPD001*	ClaspCone-1
MBW7946615.1	*Sphingomonadaceae bacterium*	ClaspCone-1
HAC91914.1	*Planctomycetaceae bacterium*	ClaspCone-1
WP_074307363.1	*Singulisphaera sp. GP187*	ClaspCone-1
OYU74888.1	*Alphaproteobacteria bacterium PA3*	ClaspCone-1
MBU2277746.1	*Gammaproteobacteria bacterium*	ClaspCone-1
EGG98911.1	*gamma proteobacterium IMCC2047*	ClaspCone-1
HHH39782.1	*Sedimenticola sp.*	ClaspCone-1
MBI1991579.1	*Betaproteobacteria bacterium*	ClaspCone-1
MBI3463667.1	*Planctomycetota bacterium*	ClaspCone-1
MBN1490432.1	*Phycisphaerae bacterium*	ClaspCone-1
MBR9784408.1	*Gammaproteobacteria bacterium*	ClaspCone-1
MBK7813600.1	*Rhodocyclaceae bacterium*	ClaspCone-1
MBN7827827.1	*Bowmanella dokdonensis*	ClaspCone-1
NDC63918.1	*Planctomycetia bacterium*	ClaspCone-1
RMF88457.1	*Planctomycetota bacterium*	ClaspCone-1
NIL98345.1	*Planctomycetales bacterium*	ClaspCone-1
MBS04823.1	*Gammaproteobacteria bacterium*	ClaspCone-1
MBX9581943.1	*Gemmataceae bacterium*	ClaspCone-1
NOQ79711.1	*Gammaproteobacteria bacterium*	ClaspCone-1
MBK8993544.1	*Gammaproteobacteria bacterium*	ClaspCone-1
MBU2099003.1	*Gammaproteobacteria bacterium*	ClaspCone-1
NUQ62007.1	*Pirellulales bacterium*	ClaspCone-1
NLM16483.1	*Candidatus Rifleibacteriota bacterium*	ClaspCone-1
MBQ3643116.1	*Candidatus Rifleibacteriota bacterium*	ClaspCone-1
PKL45079.1	*Candidatus Riflebacteria bacterium HGW-Riflebacteria-1*	ClaspCone-1
PKL51014.1	*Candidatus Riflebacteria bacterium HGW-Riflebacteria-2*	ClaspCone-1
PKK89535.1	*Candidatus Wallbacteria bacterium HGW-Wallbacteria-1*	ClaspCone-1
MBI3037921.1	*bacterium*	ClaspCone-1
OQB46357.1	*bacterium ADurb.Bin157*	ClaspCone-1
MCA8925162.1	*Planctomycetota bacterium*	ClaspCone-1
MBL4849831.1	*Planctomycetota bacterium*	ClaspCone-1
MCA8920723.1	*Planctomycetota bacterium*	ClaspCone-1
MBX3472043.1	*Planctomycetota bacterium*	ClaspCone-1
HBP17538.1	*Planctomycetota bacterium*	ClaspCone-1
WP_115392376.1	*Rhodovulum sp. 12E13*	ClaspCone-1
MBI4346754.1	*Elusimicrobiota bacterium*	ClaspCone-1
HIF33245.1	*Planctomycetaceae bacterium*	?? · ClaspCone-1
MBX9655834.1	*bacterium*	?? · ClaspCone-1
MBY0588477.1	*bacterium*	?? · ClaspCone-1
MBI2923758.1	*Planctomycetota bacterium*	ClaspCone-1
MCC6348055.1	*Candidatus Eiseniibacteriota bacterium*	ClaspCone-1
MCD6249670.1	*Candidatus Zixiibacteriota bacterium*	ClaspCone-1
OUW62748.1	*bacterium TMED198*	ClaspCone-1
WP_204433184.1		ClaspCone-1
DAI65794.1		ClaspCone-1
RJP34599.1	*Phycisphaerales bacterium*	? · ClaspCone-1
MBC8344884.1	*Fidelibacterota bacterium*	ClaspCone-1
WP_109622307.1	*Sediminitomix flava*	ClaspCone-1
OPX30781.1	*Gemmatimonadaceae bacterium 4484_173*	ClaspCone-1
MBA8869023.1	*Pantoea agglomerans*	ClaspCone-1

#############; ClaspCone-2; ###########;RHS-containing classical ClaspCone-2 repeats

WP_126243474.1	*Chitinophaga rhizosphaerae*	RHS-repeats · ClaspCone-2
TXK75369.1	*Mesonia sp. K4-1*	RHS-repeats · ClaspCone-2 · 4TM-imm(frag)
WP_102994981.1	*Pseudotamlana carrageenivorans*	RHS-repeats · ClaspCone-2 · 4TM-imm
KII66730.1	*Thelohanellus kitauei*	RHS-repeats · ClaspCone-2
MBI1148117.1	*Escherichia coli*	RHS-repeats · ClaspCone-2 · 4TM-imm
WP_047371727.1	*Kluyvera intermedia*	RHS-repeats · ClaspCone-2 · 4TM-imm
PKP12733.1	*Bacteroidetes bacterium HGW-Bacteroidetes-3*	RHS-repeats · ClaspCone-2 · 4TM-imm
WP_192520806.1	*Psychrobacter sp. FME61*	RHS-repeats · ClaspCone-2
WP_149109332.1	*Limnoglobus roseus*	RHS-repeats · ClaspCone-2
EFP0747811.1	*Escherichia coli*	RHS-repeats · ClaspCone-2

WP_014709527.1	*Prevotella intermedia*	RHS-repeats, ClaspCone-2, 4TM-imm(frag)
WP_191132094.1		RHS-repeats, ClaspCone-2
WP_140027831.1	*Escherichia coli*	RHS-repeats, ClaspCone-2
WP_131597436.1	*Pedobacter psychroterrae*	SpvB, BetaPropellers, RHS-repeats, ClaspCone-2
WP_233027933.1	*Sinomicrobium kalidii*	IG-like-repeats, DUF6443, RHS-repeats, ClaspCone-2

#########;IG-like repeat-containing; #;IG+IG+4-strand-beta-sheet+ClaspCone-2

WP_004084771.1	*Helicobacter bilis*	IG, IG, 4-strand-beta-sheet, ClaspCone-2
WP_110580246.1	*Helicobacter cinaedi*	IG, IG, 4-strand-beta-sheet, ClaspCone-2, 4TM-imm(?)
WP_054197463.1	*Helicobacter pullorum*	IG, IG, 4-strand-beta-sheet, ClaspCone-2, 4TM-imm(?)

########;Peptidase domain-associating; #;Subtilisin-containing

MCC3503793.1	*Microcoleus sp. PH2017_19_SFW_U_A*	Subtilisin, SH3, NPLC, LysM, ClaspCone-2

#;Papain-like-containing

MBI4352443.1	*Candidatus Omnitrophota bacterium*	Papain-like, ClaspCone-2

########;HISKIN-containing

MBP0019758.1	*Cyanobacteria bacterium SBLK*	His-Kinase, SHS2, ClaspCone-2, disordered, TPRs

########;HEAT repeats fusion

MBS3121770.1	*Candidatus Woesearchaeota archaeon*	HEATs, ClaspCone-2

#########;standalone ClaspCone-2

WP_067554832.1	*Acinetobacter larvae*	ClaspCone-2
WP_210670807.1	*Helicobacter pullorum NCTC 12824*	ClaspCone-2, 4TM-imm(frag)
WP_034562942.1	*Helicobacter bilis*	ClaspCone-2
EDZ3577090.1	*Salmonella enterica*	ClaspCone-2
WP_139986992.1	*Escherichia coli*	ClaspCone-2
WP_226871960.1	*Enterobacter hormaechei*	ClaspCone-2

#############;ClaspCone-3

MBX2975608.1	*Ignavibacteriaceae bacterium*	ClaspCone-3
NUN08505.1	*Ignavibacteriaceae bacterium*	ClaspCone-3
KAA0246010.1	*Chlorobiota bacterium*	ClaspCone-3
MBE0573018.1	*Ignavibacteriaceae bacterium*	ClaspCone-3
MBI5726357.1	*Ignavibacteriales bacterium*	ClaspCone-3
HAL56057.1	*Bacteroidota bacterium*	ClaspCone-3
HDX11496.1		ClaspCone-3
RMD50784.1	*Ignavibacteria bacterium*	ClaspCone-3
RCK72239.1	*Ignavibacteriota bacterium*	ClaspCone-3
MBM4167153.1	*Ignavibacteria bacterium*	ClaspCone-3
NWF90806.1	*Ignavibacteriaceae bacterium*	ClaspCone-3
MBA4313121.1	*Chlorobiaceae bacterium*	ClaspCone-3
MBM4168851.1	*Ignavibacteria bacterium*	ClaspCone-3
MBI1804581.1	*Ignavibacteria bacterium*	ClaspCone-3
HHD22207.1		ClaspCone-3
MBS4029494.1	*Ignavibacteriales bacterium*	ClaspCone-3
OGU19325.1	*Ignavibacteria bacterium GWA2_55_25*	ClaspCone-3
MBP1679342.1	*Bacteroidota bacterium*	ClaspCone-3
TAK54873.1	*Bacteroidota bacterium*	ClaspCone-3
MBI5646067.1	*Ignavibacteriota bacterium*	ClaspCone-3
HCV41921.1	*Bacteroidota bacterium*	ClaspCone-3
PIP77491.1	*Ignavibacteria bacterium CG22_combo_CG10-13_8_21_14_all_37_15*	ClaspCone-3
NOY07076.1	*Chlorobiota bacterium*	ClaspCone-3
WP_140944637.1	*Candidatus Thermokryptus mobilis*	ClaspCone-3
CUU08697.1	*Candidatus Kryptobacter tengchongensis*	ClaspCone-3
MBI2428615.1	*Ignavibacteriales bacterium*	ClaspCone-3
PIW70416.1	*Ignavibacteriales bacterium CG12_big_fil_rev_8_21_14_0_65_30_8*	ClaspCone-3
MBM4171375.1	*Ignavibacteria bacterium*	ClaspCone-3
MBI3124650.1	*Ignavibacteriales bacterium*	ClaspCone-3
MBX3008125.1	*Melioribacteraceae bacterium*	ClaspCone-3
MBV6513292.1	*Ignavibacteriaceae bacterium*	ClaspCone-3
TSA29530.1	*Ignavibacteriales bacterium*	ClaspCone-3
MBZ0179618.1	*Melioribacteraceae bacterium*	ClaspCone-3
WP_014857104.1	*Melioribacter sp.*	ClaspCone-3
KAF0151442.1	*Ignavibacteria bacterium*	ClaspCone-3
KUO61538.1	*bacterium BRH_c32*	ClaspCone-3
NLT49644.1	*Ignavibacteria bacterium*	ClaspCone-3
UCH64513.1	*Ignavibacterium sp.*	ClaspCone-3
RJP61847.1	*Ignavibacteriales bacterium*	ClaspCone-3
MBA4250683.1	*Chlorobiaceae bacterium*	ClaspCone-3
MCE1187533.1	*Ignavibacteria bacterium*	ClaspCone-3
PJA98778.1	*Ignavibacteriales bacterium CG_4_9_14_3_um_filter_30_11*	ClaspCone-3
MBU2447054.1	*Bacteroidota bacterium*	ClaspCone-3
MBM4175545.1	*Ignavibacteria bacterium*	ClaspCone-3
QQS37426.1	*Ignavibacteriales bacterium*	ClaspCone-3
PJA96131.1	*Ignavibacteriales bacterium CG_4_9_14_3_um_filter_34_10*	ClaspCone-3
MBU1098897.1	*Bacteroidota bacterium*	ClaspCone-3
GJQ64024.1	*Melioribacteraceae bacterium*	ClaspCone-3
MCC6740020.1	*Planctomycetia bacterium*	ClaspCone-1
MBI5472923.1	*Ignavibacteriota bacterium*	ClaspCone-3
MBI4417795.1	*Ignavibacteriales bacterium*	ClaspCone-3
MBW7887590.1	*Bacteroidota bacterium*	ClaspCone-3
HCA78134.1	*Bacteroidota bacterium*	ClaspCone-3
PIU45474.1	*Ignavibacteriales bacterium CG07_land_8_20_14_0_80_59_12*	ClaspCone-3
MCC6395812.1	*Bacteroidota bacterium*	ClaspCone-3
MBI5475761.1	*Ignavibacteriales bacterium*	ClaspCone-3
HCY03691.1	*Erythrobacter sp.*	ClaspCone-3
MBZ0183738.1	*Melioribacteraceae bacterium*	ClaspCone-3
GBD91489.1	*bacterium BMS3Abin04*	ClaspCone-3
MBE0645224.1	*Bacteroidota bacterium*	ClaspCone-3
MBL0175425.1	*Ignavibacteria bacterium*	ClaspCone-3
MBI3195673.1	*Ignavibacteriota bacterium*	ClaspCone-3
MBI5663171.1	*Ignavibacterium album*	ClaspCone-3
KXK53328.1	*Chlorobi bacterium OLB7*	ClaspCone-3
NOX18075.1	*Chlorobiota bacterium*	ClaspCone-3
OLD67027.1	*Ignavibacteria bacterium 13_1_40CM_2_61_4*	ClaspCone-3
MBS1911082.1	*Bacteroidota bacterium*	ClaspCone-3
MBP8976087.1	*Bacteroidota bacterium*	ClaspCone-3
MBU1098137.1	*Bacteroidota bacterium*	ClaspCone-3
KAB2921232.1	*Bacteroidota bacterium*	ClaspCone-3

Accession	Organism	Domains
MAT39355.1	*Ectothiorhodospiraceae bacterium*	ClaspCone-3
OGU62769.1	*Ignavibacteria bacterium RBG_13_36_8*	ClaspCone-3
MBL1213662.1	*Ignavibacteriota bacterium*	ClaspCone-3
CAG0942767.1	*Candidatus Brocadiaceae bacterium*	ClaspCone-3
NOX64197.1	*Chlorobiota bacterium*	ClaspCone-3
MBN1300217.1	*Melioribacteraceae bacterium*	ClaspCone-3
MBS3945105.1	*Melioribacter sp.*	ClaspCone-3
MBU2507893.1	*Bacteroidota bacterium*	ClaspCone-3
OGU36632.1	*Ignavibacteria bacterium GWB2_35_6b*	ClaspCone-3
MBI9073278.1	*Melioribacteraceae bacterium*	ClaspCone-3
MBD3410151.1	*Ignavibacteriales bacterium*	ClaspCone-3
GJQ33735.1	*Ignavibacteriaceae bacterium*	ClaspCone-3
MBU2494845.1	*Bacteroidota bacterium*	ClaspCone-3
NUM72410.1	*Ignavibacteriaceae bacterium*	ClaspCone-3
MBS1902351.1	*Bacteroidota bacterium*	ClaspCone-3
MBE2280598.1		ClaspCone-3
RJP59761.1	*Ignavibacteriales bacterium*	ClaspCone-3
MBK8946828.1	*Ignavibacteriota bacterium*	ClaspCone-3
RPI70731.1	*Ignavibacteriales bacterium*	ClaspCone-3
MBV6446289.1	*Ignavibacteriaceae bacterium*	ClaspCone-3
MBN8546023.1	*Ignavibacteria bacterium*	ClaspCone-3
MBN1639455.1	*Ignavibacteriales bacterium*	ClaspCone-3
MBU0473575.1	*Bacteroidota bacterium*	ClaspCone-3
NLH60647.1		ClaspCone-3
MBW2998073.1	*Candidatus Woesearchaeota archaeon*	ClaspCone-3
MBL0332117.1	*Chlorobiota bacterium*	ClaspCone-3
MBN1447449.1	*Bacteroidota bacterium*	ClaspCone-3
MBU1299521.1	*Bacteroidota bacterium*	ClaspCone-3
OYV86548.1	*Ignavibacteriae bacterium 37-53-5*	ClaspCone-3
MBM2842095.1	*Bacteroidota bacterium*	ClaspCone-3
MBW7901820.1	*Rhodocyclaceae bacterium*	ClaspCone-3
PCH51773.1	*Cellvibrionales bacterium*	ClaspCone-3
RPI02349.1	*Ignavibacteriota bacterium*	ClaspCone-3
HHS61273.1		ClaspCone-3
HGS53131.1		ClaspCone-3
MCC6697771.1	*Candidatus Hydrogenedentota bacterium*	ClaspCone-3
MCC5915625.1	*Balneolaceae bacterium*	ClaspCone-3
WP_109644344.1	*Rhodohalobacter mucosus*	ClaspCone-3
PID69607.1	*Flavobacteriales bacterium*	ClaspCone-3
MCC6277004.1	*Oligoflexia bacterium*	? ClaspCone-3

#############;PTDSS1/2: automatic annotations

Accession	Organism	Domains
XP_005827092.1	*Guillardia theta CCMP2712*	PTDSS1/2
PRP83789.1	*Planoprotostelium fungivorum*	PTDSS1/2
XP_004344733.1	*Acanthamoeba castellanii str. Neff*	PTDSS1/2
XP_004368267.1	*Acanthamoeba castellanii str. Neff*	PTDSS1/2
XP_012760211.1	*Acytostelium subglobosum LB1*	PTDSS1/2
XP_003290662.1	*Dictyostelium purpureum*	PTDSS1/2
KYQ94384.1	*Tieghemostelium lacteum*	PTDSS1/2
XP_004253677.1	*Entamoeba invadens IP1*	PTDSS1/2
XP_008860538.1	*Entamoeba nuttalli P19*	PTDSS1/2
KAH3731340.1	*Pelomyxa schiedti*	PTDSS1/2
XP_013759935.1	*Thecamonas trahens ATCC 50062*	PTDSS1/2
XP_013758815.1	*Thecamonas trahens ATCC 50062*	PTDSS1/2
EPY26426.1	*Angomonas deanei*	PTDSS1/2
CUE71370.1	*Bodo saltans*	PTDSS1/2
GET87150.1	*Leishmania tarentolae*	PTDSS1/2
KNH05885.1	*Perkinsela sp. CCAP 1560/4*	PTDSS1/2
CCW64272.1	*Phytomonas sp. EM1*	PTDSS1/2
KAG5509457.1	*Porcisia hertigi*	PTDSS1/2
EPY26341.1	*Strigomonas culicis*	PTDSS1/2
XP_011774773.1	*Trypanosoma brucei gambiense DAL972*	PTDSS1/2
CCC91488.1		PTDSS1/2
EKF27542.1	*Trypanosoma cruzi marinkellei*	PTDSS1/2
XP_028880322.1	*Trypanosoma theileri*	PTDSS1/2
KAG8346950.1	*Trypanosoma vivax*	PTDSS1/2
XP_044565122.1	*Naegleria fowleri*	PTDSS1/2
OHS99411.1	*Tritrichomonas foetus*	PTDSS1/2
XP_004990775.1	*Salpingoeca rosetta*	PTDSS1/2
KJE94056.1	*Capsaspora owczarzaki ATCC 30864*	PTDSS1/2
KAG5455703.1	*Olpidium bornovanus*	PTDSS1/2
KAH8928351.1	*Atractiella rhizophila*	PTDSS1/2
MBW0512674.1	*Austropuccinia psidii MF-1*	PTDSS1/2
KAG9097087.1	*Ceratobasidium sp. 370*	PTDSS1/2
KAB5595898.1	*Ceratobasidium theobromae*	PTDSS1/2
KAF9508315.1	*Hydnum rufescens UP504*	PTDSS1/2
RDB14689.1	*Hypsizygus marmoreus*	PTDSS1/2
XP_014565616.1	*Mixia osmundae IAM 14324*	PTDSS1/2
THH05664.1	*Phellinidium pouzarii*	PTDSS1/2
KAF7791481.1	*Pleurotus ostreatoroseus*	PTDSS1/2
KAA1065295.1	*Puccinia graminis f. sp. tritici*	PTDSS1/2
XP_043180438.1	*Rhizoctonia solani*	PTDSS1/2
OCB83859.1	*Sanghuangporus baumii*	PTDSS1/2
XP_003034006.1		PTDSS1/2
PVF93111.1	*Serendipita vermifera*	PTDSS1/2
KIK37547.1	*Suillus luteus UH-Slu-Lm8-n1*	PTDSS1/2
KAG5722224.1	*Termitomyces sp. T112*	PTDSS1/2
KAG6900144.1	*Termitomyces sp. T159_Od127*	PTDSS1/2
CDO69873.1	*Trametes cinnabarina*	PTDSS1/2
KAG8952889.1	*Tulasnella sp. 424*	PTDSS1/2
KAG8977156.1	*Tulasnella sp. 425*	PTDSS1/2
KAG9013543.1	*Tulasnella sp. JGI-2019a*	PTDSS1/2
KAF8651936.1	*Volvariella volvacea WC 439*	PTDSS1/2
XP_006675932.1	*Batrachochytrium dendrobatidis JAM81*	PTDSS1/2

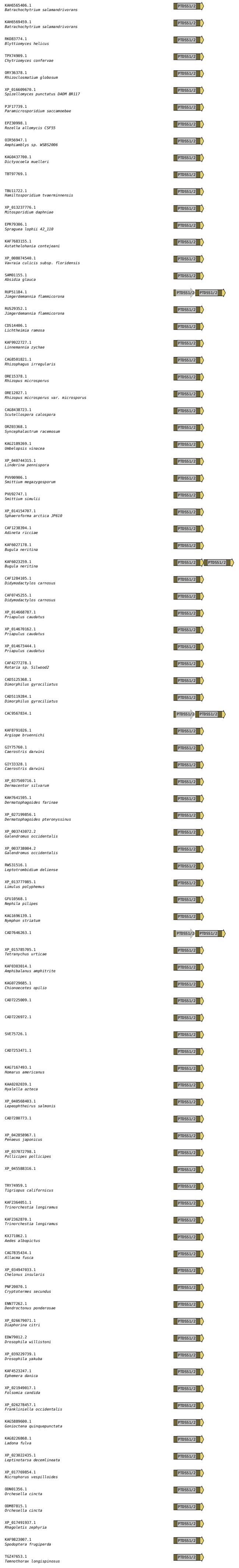
KAH6565406.1
Batrachochytrium salamandrivorans
PTDSS1/2
KAH6569459.1
Batrachochytrium salamandrivorans
PTDSS1/2
RKO83774.1
Blyttiomyces helicus
PTDSS1/2
TPX74909.1
Chytriomyces confervae
PTDSS1/2
ORY36378.1
Rhizoclosmatium globosum
PTDSS1/2
XP_016609670.1
Spizellomyces punctatus DAOM BR117
PTDSS1/2
PJF17739.1
Paramicrosporidium saccamoebae
PTDSS1/2
EPZ30998.1
Rozella allomycis CSF55
PTDSS1/2
OIR56947.1
Amphiamblys sp. WSBS2006
PTDSS1/2
KAG0437700.1
Dictyocoela muelleri
PTDSS1/2
TBT97769.1
PTDSS1/2
TBU11722.1
Hamiltosporidium tvaerminnensis
PTDSS1/2
XP_013237776.1
Mitosporidium daphniae
PTDSS1/2
EPR79306.1
Spraguea lophii 42_110
PTDSS1/2
KAF7683155.1
Astathelohania contejeani
PTDSS1/2
XP_008074540.1
Vavraia culicis subsp. floridensis
PTDSS1/2
SAM01155.1
Absidia glauca
PTDSS1/2
RUP51184.1
Jimgerdemannia flammicorona
PTDSS1/2
PTDSS1/2
RUS29352.1
Jimgerdemannia flammicorona
PTDSS1/2
CDS14406.1
Lichtheimia ramosa
PTDSS1/2
KAF9922727.1
Linnemannia zychae
PTDSS1/2
CAG8501821.1
Rhizophagus irregularis
PTDSS1/2
ORE15378.1
Rhizopus microsporus
PTDSS1/2
ORE12027.1
Rhizopus microsporus var. microsporus
PTDSS1/2
CAG8438723.1
Scutellospora calospora
PTDSS1/2
ORZ03368.1
Syncephalastrum racemosum
PTDSS1/2
KAG2189269.1
Umbelopsis vinacea
PTDSS1/2
XP_040744315.1
Linderina pennispora
PTDSS1/2
PVV00906.1
Smittium megazygosporum
PTDSS1/2
PVU92747.1
Smittium simulii
PTDSS1/2
XP_014154707.1
Sphaeroforma arctica JP610
PTDSS1/2
CAF1238394.1
Adineta ricciae
PTDSS1/2
KAF6027178.1
Bugula neritina
PTDSS1/2
KAF6023259.1
Bugula neritina
PTDSS1/2
PTDSS1/2
CAF1284105.1
Didymodactylos carnosus
PTDSS1/2
CAF0745255.1
Didymodactylos carnosus
PTDSS1/2
XP_014668787.1
Priapulus caudatus
PTDSS1/2
XP_014670162.1
Priapulus caudatus
PTDSS1/2
XP_014673444.1
Priapulus caudatus
PTDSS1/2
CAF4277278.1
Rotaria sp. Silwood2
PTDSS1/2
CAD5125368.1
Dimorphilus gyrociliatus
PTDSS1/2
CAD5119284.1
Dimorphilus gyrociliatus
PTDSS1/2
CAC9567834.1
PTDSS1/2
PTDSS1/2
KAF8791026.1
Argiope bruennichi
PTDSS1/2
GIY75760.1
Caerostris darwini
PTDSS1/2
GIY33328.1
Caerostris darwini
PTDSS1/2
XP_037569716.1
Dermacentor silvarum
PTDSS1/2
KAH7641595.1
Dermatophagoides farinae
PTDSS1/2
XP_027199856.1
Dermatophagoides pteronyssinus
PTDSS1/2
XP_003743072.2
Galendromus occidentalis
PTDSS1/2
XP_003738004.2
Galendromus occidentalis
PTDSS1/2
RWS31516.1
Leptotrombidium deliense
PTDSS1/2
XP_013777085.1
Limulus polyphemus
PTDSS1/2
GFU10568.1
Nephila pilipes
PTDSS1/2
KAG1696139.1
Nymphon striatum
PTDSS1/2
CAD7646263.1
PTDSS1/2
PTDSS1/2
XP_015785705.1
Tetranychus urticae
PTDSS1/2
KAF0303014.1
Amphibalanus amphitrite
PTDSS1/2
KAG0729685.1
Chionoecetes opilio
PTDSS1/2
CAD7225009.1
PTDSS1/2
CAD7226972.1
PTDSS1/2
SVE75726.1
PTDSS1/2
CAD7253471.1
PTDSS1/2
KAG7167493.1
Homarus americanus
PTDSS1/2
KAA0202039.1
Hyalella azteca
PTDSS1/2
XP_040568403.1
Lepeophtheirus salmonis
PTDSS1/2
CAD7280773.1
PTDSS1/2
XP_042858967.1
Penaeus japonicus
PTDSS1/2
XP_037072798.1
Pollicipes pollicipes
PTDSS1/2
XP_045588316.1
PTDSS1/2
TRY74959.1
Tigriopus californicus
PTDSS1/2
KAF2364051.1
Trinorchestia longiramus
PTDSS1/2
KAF2362870.1
Trinorchestia longiramus
PTDSS1/2
KXJ71062.1
Aedes albopictus
PTDSS1/2
CAG7835434.1
Allacma fusca
PTDSS1/2
XP_034947033.1
Chelonus insularis
PTDSS1/2
PNF20070.1
Cryptotermes secundus
PTDSS1/2
ENN77262.1
Dendroctonus ponderosae
PTDSS1/2
XP_026679071.1
Diaphorina citri
PTDSS1/2
EDW79012.2
Drosophila willistoni
PTDSS1/2
XP_039229739.1
Drosophila yakuba
PTDSS1/2
KAF4523247.1
Ephemera danica
PTDSS1/2
XP_021949017.1
Folsomia candida
PTDSS1/2
XP_026278457.1
Frankliniella occidentalis
PTDSS1/2
KAG5889600.1
Gonioctena quinquepunctata
PTDSS1/2
KAG8226868.1
Ladona fulva
PTDSS1/2
XP_023022435.1
Leptinotarsa decemlineata
PTDSS1/2
XP_017769854.1
Nicrophorus vespilloides
PTDSS1/2
ODN01356.1
Orchesella cincta
PTDSS1/2
ODM87815.1
Orchesella cincta
PTDSS1/2
XP_017491937.1
Rhagoletis zephyria
PTDSS1/2
KAF9823007.1
Spodoptera frugiperda
PTDSS1/2
TGZ47653.1
Temnothorax longispinosus
PTDSS1/2

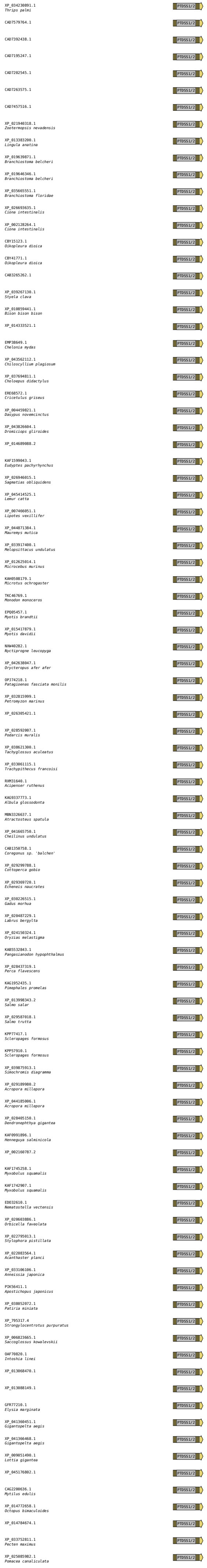

XP_034230891.1
Thrips palmi
PTDSS1/2
CAD7579764.1
PTDSS1/2
CAD7392438.1
PTDSS1/2
CAD7195247.1
PTDSS1/2
CAD7202545.1
PTDSS1/2
CAD7263575.1
PTDSS1/2
CAD7457516.1
PTDSS1/2
XP_021940318.1
Zootermopsis nevadensis
PTDSS1/2
XP_013383200.1
Lingula anatina
PTDSS1/2
XP_019639871.1
Branchiostoma belcheri
PTDSS1/2
XP_019646346.1
Branchiostoma belcheri
PTDSS1/2
XP_035665551.1
Branchiostoma floridae
PTDSS1/2
XP_026693635.1
Ciona intestinalis
PTDSS1/2
XP_002128264.1
Ciona intestinalis
PTDSS1/2
CBY15123.1
Oikopleura dioica
PTDSS1/2
CBY41771.1
Oikopleura dioica
PTDSS1/2
CAB3265262.1
PTDSS1/2
XP_039267130.1
Styela clava
PTDSS1/2
XP_010859441.1
Bison bison bison
PTDSS1/2
XP_014333521.1
PTDSS1/2
EMP38649.1
Chelonia mydas
PTDSS1/2
XP_043562112.1
Chiloscyllium plagiosum
PTDSS1/2
XP_037694811.1
Choloepus didactylus
PTDSS1/2
ERE68572.1
Cricetulus griseus
PTDSS1/2
XP_004459821.1
Dasypus novemcinctus
PTDSS1/2
XP_043826604.1
Dromiciops gliroides
PTDSS1/2
XP_014689088.2
PTDSS1/2
KAF1599043.1
Eudyptes pachyrhynchus
PTDSS1/2
XP_026946015.1
Sagmatias obliquidens
PTDSS1/2
XP_045414525.1
Lemur catta
PTDSS1/2
XP_007466051.1
Lipotes vexillifer
PTDSS1/2
XP_044871384.1
Mauremys mutica
PTDSS1/2
XP_033917400.1
Melopsittacus undulatus
PTDSS1/2
XP_012625014.1
Microcebus murinus
PTDSS1/2
KAH0508179.1
Microtus ochrogaster
PTDSS1/2
TKC46769.1
Monodon monoceros
PTDSS1/2
EPQ05457.1
Myotis brandtii
PTDSS1/2
XP_015417879.1
Myotis davidii
PTDSS1/2
NXW40282.1
Nyctiprogne leucopyga
PTDSS1/2
XP_042638047.1
Orycteropus afer afer
PTDSS1/2
OPJ74218.1
Patagioenas fasciata monilis
PTDSS1/2
XP_032815999.1
Petromyzon marinus
PTDSS1/2
XP_026305421.1
PTDSS1/2
XP_028592007.1
Podarcis muralis
PTDSS1/2
XP_038621300.1
Tachyglossus aculeatus
PTDSS1/2
XP_033061115.1
Trachypithecus francoisi
PTDSS1/2
RXM31640.1
Acipenser ruthenus
PTDSS1/2
KAG9337773.1
Albula glossodonta
PTDSS1/2
MBN3326637.1
Atractosteus spatula
PTDSS1/2
XP_041665750.1
Cheilinus undulatus
PTDSS1/2
CAB1350758.1
Coregonus sp. 'balchen'
PTDSS1/2
XP_029299788.1
Cottoperca gobio
PTDSS1/2
XP_029369720.1
Echeneis naucrates
PTDSS1/2
XP_030226515.1
Gadus morhua
PTDSS1/2
XP_020487229.1
Labrus bergylta
PTDSS1/2
XP_024150324.1
Oryzias melastigma
PTDSS1/2
KAB5532843.1
Pangasianodon hypophthalmus
PTDSS1/2
XP_028437319.1
Perca flavescens
PTDSS1/2
KAG1952435.1
Pimephales promelas
PTDSS1/2
XP_013998343.2
Salmo salar
PTDSS1/2
XP_029587018.1
Salmo trutta
PTDSS1/2
KPP77417.1
Scleropages formosus
PTDSS1/2
KPP57910.1
Scleropages formosus
PTDSS1/2
XP_039875913.1
Simochromis diagramma
PTDSS1/2
XP_029189980.2
Acropora millepora
PTDSS1/2
XP_044185006.1
Acropora millepora
PTDSS1/2
XP_028405150.1
Dendronephthya gigantea
PTDSS1/2
KAF0991896.1
Henneguya salminicola
PTDSS1/2
XP_002160787.2
PTDSS1/2
KAF1745258.1
Myxobolus squamalis
PTDSS1/2
KAF1742907.1
Myxobolus squamalis
PTDSS1/2
EDO32610.1
Nematostella vectensis
PTDSS1/2
XP_020603886.1
Orbicella faveolata
PTDSS1/2
XP_022795013.1
Stylophora pistillata
PTDSS1/2
XP_022083564.1
Acanthaster planci
PTDSS1/2
XP_033106106.1
Anneissia japonica
PTDSS1/2
PIK56411.1
Apostichopus japonicus
PTDSS1/2
XP_038052072.1
Patiria miniata
PTDSS1/2
XP_795317.4
Strongylocentrotus purpuratus
PTDSS1/2
XP_006823665.1
Saccoglossus kowalevskii
PTDSS1/2
OAF70820.1
Intoshia linei
PTDSS1/2
XP_013068470.1
PTDSS1/2
XP_013088149.1
PTDSS1/2
GFR77210.1
Elysia marginata
PTDSS1/2
XP_041360451.1
Gigantopelta aegis
PTDSS1/2
XP_041366468.1
Gigantopelta aegis
PTDSS1/2
XP_009051490.1
Lottia gigantea
PTDSS1/2
XP_045176802.1
PTDSS1/2
CAG2200636.1
Mytilus edulis
PTDSS1/2
XP_014772658.1
Octopus bimaculoides
PTDSS1/2
XP_014784674.1
PTDSS1/2
XP_033752811.1
Pecten maximus
PTDSS1/2
XP_025085982.1
Pomacea canaliculata
PTDSS1/2

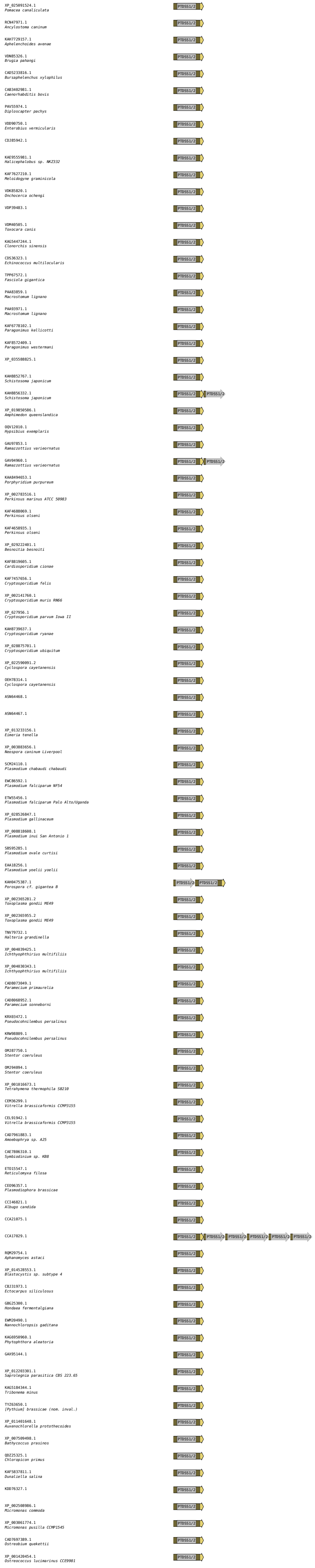

XP_025091524.1
Pomacea canaliculata
PTDSS1/2
RCN47971.1
Ancylostoma caninum
PTDSS1/2
KAH7729157.1
Aphelenchoides avenae
PTDSS1/2
VDN85326.1
Brugia pahangi
PTDSS1/2
CAD5233816.1
Bursaphelenchus xylophilus
PTDSS1/2
CAB3402981.1
Caenorhabditis bovis
PTDSS1/2
PAV55974.1
Diploscapter pachys
PTDSS1/2
VDD90750.1
Enterobius vermicularis
PTDSS1/2
CDJ85942.1
PTDSS1/2
KAE9555981.1
Halicephalobus sp. NKZ332
PTDSS1/2
KAF7627210.1
Meloidogyne graminicola
PTDSS1/2
VDK85820.1
Onchocerca ochengi
PTDSS1/2
VDP39483.1
PTDSS1/2
VDM40505.1
Toxocara canis
PTDSS1/2
KAG5447244.1
Clonorchis sinensis
PTDSS1/2
CDS36323.1
Echinococcus multilocularis
PTDSS1/2
TPP67572.1
Fasciola gigantica
PTDSS1/2
PAA83859.1
Macrostomum lignano
PTDSS1/2
PAA93971.1
Macrostomum lignano
PTDSS1/2
KAF6778102.1
Paragonimus kellicotti
PTDSS1/2
KAF8572409.1
Paragonimus westermani
PTDSS1/2
XP_035588825.1
PTDSS1/2
KAH8852767.1
Schistosoma japonicum
PTDSS1/2
KAH8856332.1
Schistosoma japonicum
PTDSS1/2 PTDSS1/2
XP_019850586.1
Amphimedon queenslandica
PTDSS1/2
OQV12010.1
Hypsibius exemplaris
PTDSS1/2
GAU97853.1
Ramazzottius varieornatus
PTDSS1/2
GAV04960.1
Ramazzottius varieornatus
PTDSS1/2 PTDSS1/2
KAA8494653.1
Porphyridium purpureum
PTDSS1/2
XP_002783516.1
Perkinsus marinus ATCC 50983
PTDSS1/2
KAF4688069.1
Perkinsus olseni
PTDSS1/2
KAF4658935.1
Perkinsus olseni
PTDSS1/2
XP_029222401.1
Besnoitia besnoiti
PTDSS1/2
KAF8819605.1
Cardiosporidium cionae
PTDSS1/2
KAF7457656.1
Cryptosporidium felis
PTDSS1/2
XP_002141760.1
Cryptosporidium muris RN66
PTDSS1/2
XP_627956.1
Cryptosporidium parvum Iowa II
PTDSS1/2
KAH8739637.1
Cryptosporidium ryanae
PTDSS1/2
XP_028875701.1
Cryptosporidium ubiquitum
PTDSS1/2
XP_022590091.2
Cyclospora cayetanensis
PTDSS1/2
OEH78314.1
Cyclospora cayetanensis
PTDSS1/2
ASN64468.1
PTDSS1/2
ASN64467.1
PTDSS1/2
XP_013233156.1
Eimeria tenella
PTDSS1/2
XP_003883656.1
Neospora caninum Liverpool
PTDSS1/2
SCM24110.1
Plasmodium chabaudi chabaudi
PTDSS1/2
EWC86592.1
Plasmodium falciparum NF54
PTDSS1/2
ETW55456.1
Plasmodium falciparum Palo Alto/Uganda
PTDSS1/2
XP_028526847.1
Plasmodium gallinaceum
PTDSS1/2
XP_008818608.1
Plasmodium inui San Antonio 1
PTDSS1/2
SBS95285.1
Plasmodium ovale curtisi
PTDSS1/2
EAA18256.1
Plasmodium yoelii yoelii
PTDSS1/2
KAH0475387.1
Porospora cf. gigantea B
PTDSS1/2 PTDSS1/2
XP_002365281.2
Toxoplasma gondii ME49
PTDSS1/2
XP_002365955.2
Toxoplasma gondii ME49
PTDSS1/2
TNV79732.1
Halteria grandinella
PTDSS1/2
XP_004039425.1
Ichthyophthirius multifiliis
PTDSS1/2
XP_004030343.1
Ichthyophthirius multifiliis
PTDSS1/2
CAD8073049.1
Paramecium primaurelia
PTDSS1/2
CAD8068952.1
Paramecium sonneborni
PTDSS1/2
KRX03472.1
Pseudocohnilembus persalinus
PTDSS1/2
KRW98809.1
Pseudocohnilembus persalinus
PTDSS1/2
OMJ87750.1
Stentor coeruleus
PTDSS1/2
OMJ94094.1
Stentor coeruleus
PTDSS1/2
XP_001016673.1
Tetrahymena thermophila SB210
PTDSS1/2
CEM36299.1
Vitrella brassicaformis CCMP3155
PTDSS1/2
CEL91942.1
Vitrella brassicaformis CCMP3155
PTDSS1/2
CAD7961883.1
Amoebophrya sp. A25
PTDSS1/2
CAE7806310.1
Symbiodinium sp. KB8
PTDSS1/2
ETO15547.1
Reticulomyxa filosa
PTDSS1/2
CEO96357.1
Plasmodiophora brassicae
PTDSS1/2
CCI46821.1
Albugo candida
PTDSS1/2
CCA21075.1
PTDSS1/2
CCA17029.1
PTDSS1/2 PTDSS1/2 PTDSS1/2 PTDSS1/2 PTDSS1/2 PTDSS1/2
RQM29754.1
Aphanomyces astaci
PTDSS1/2
XP_014528553.1
Blastocystis sp. subtype 4
PTDSS1/2
CBJ31973.1
Ectocarpus siliculosus
PTDSS1/2
GBG25300.1
Hondaea fermentalgiana
PTDSS1/2
EWM20490.1
Nannochloropsis gaditana
PTDSS1/2
KAG6950960.1
Phytophthora aleatoria
PTDSS1/2
GAX95144.1
PTDSS1/2
XP_012203301.1
Saprolegnia parasitica CBS 223.65
PTDSS1/2
KAG5184344.1
Tribonema minus
PTDSS1/2
TYZ63650.1
[Pythium] brassicae (nom. inval.)
PTDSS1/2
XP_011401648.1
Auxenochlorella protothecoides
PTDSS1/2
XP_007509498.1
Bathycoccus prasinos
PTDSS1/2
QDZ25325.1
Chloropicon primus
PTDSS1/2
KAF5837811.1
Dunaliella salina
PTDSS1/2
KDD76327.1
PTDSS1/2
XP_002508986.1
Micromonas commoda
PTDSS1/2
XP_003061774.1
Micromonas pusilla CCMP1545
PTDSS1/2
CAD7697389.1
Ostreobium quekettii
PTDSS1/2
XP_001420454.1
Ostreococcus lucimarinus CCE9901
PTDSS1/2

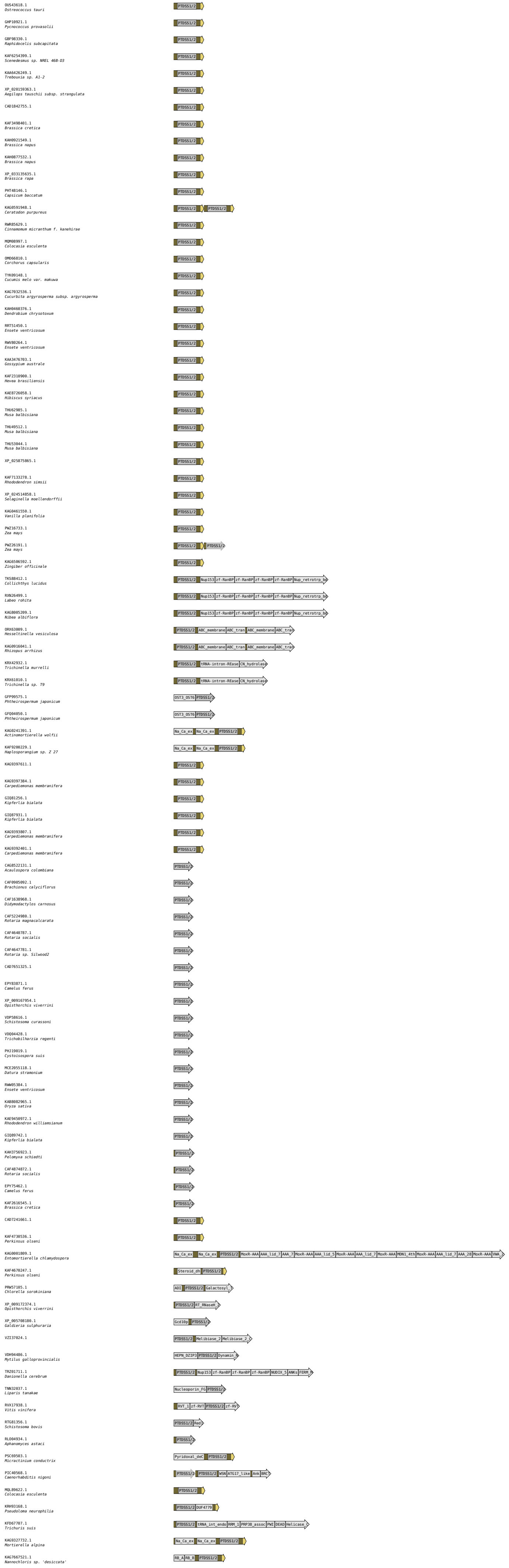
OUS43618.1
Ostreococcus tauri
GHP10921.1
Pycnococcus provasolii
GBF98330.1
Raphidocelis subcapitata
KAF6254399.1
Scenedesmus sp. NREL 46B-D3
KAA6426249.1
Trebouxia sp. A1-2
XP_020159363.1
Aegilops tauschii subsp. strangulata
CAD1842755.1
KAF3498401.1
Brassica cretica
KAH0921549.1
Brassica napus
KAH0877532.1
Brassica napus
XP_033135635.1
Brassica rapa
PHT48146.1
Capsicum baccatum
KAG0591948.1
Ceratodon purpureus
RWR85629.1
Cinnamomum micranthum f. kanehirae
MQM08997.1
Colocasia esculenta
OMO66810.1
Corchorus capsularis
TYK09148.1
Cucumis melo var. makuwa
KAG7032536.1
Cucurbita argyrosperma subsp. argyrosperma
KAH0460376.1
Dendrobium chrysotoxum
RRT51450.1
Ensete ventricosum
RWV80264.1
Ensete ventricosum
KAA3476703.1
Gossypium australe
KAF2310900.1
Hevea brasiliensis
KAE8726058.1
Hibiscus syriacus
THU62985.1
Musa balbisiana
THU49512.1
Musa balbisiana
THU53044.1
Musa balbisiana
XP_025875865.1
KAF7133278.1
Rhododendron simsii
XP_024514858.1
Selaginella moellendorffii
KAG0461550.1
Vanilla planifolia
PWZ16733.1
Zea mays
PWZ26191.1
Zea mays
KAG6506592.1
Zingiber officinale
TKS88412.1
Collichthys lucidus
PTDSS1/2 Nup153 zf-RanBP zf-RanBP zf-RanBP zf-RanBP Nup_retrotrp_bd
RXN26499.1
Labeo rohita
PTDSS1/2 Nup153 zf-RanBP zf-RanBP zf-RanBP zf-RanBP Nup_retrotrp_bd
KAG8005209.1
Nibea albiflora
PTDSS1/2 Nup153 zf-RanBP zf-RanBP zf-RanBP zf-RanBP Nup_retrotrp_bd
ORX63009.1
Hesseltinella vesiculosa
PTDSS1/2 ABC_membrane ABC_tran ABC_membrane ABC_tran
KAG0916041.1
Rhizopus arrhizus
PTDSS1/2 ABC_membrane ABC_tran ABC_membrane ABC_tran
KRX42932.1
Trichinella murrelli
PTDSS1/2 tRNA-intron-REase CN_hydrolase
KRX61010.1
Trichinella sp. T9
PTDSS1/2 tRNA-intron-REase CN_hydrolase
GFP99575.1
Phtheirospermum japonicum
OST3_OST6 PTDSS1/2
GFQ04050.1
Phtheirospermum japonicum
OST3_OST6 PTDSS1/2
KAG0241391.1
Actinomortierella wolfii
Na_Ca_ex Na_Ca_ex PTDSS1/2
KAF9200229.1
Haplosporangium sp. Z 27
Na_Ca_ex Na_Ca_ex PTDSS1/2
KAG9397611.1
KAG9397384.1
Carpediemonas membranifera
GIQ81256.1
Kipferlia bialata
GIQ87931.1
Kipferlia bialata
KAG9393807.1
Carpediemonas membranifera
KAG9392401.1
Carpediemonas membranifera
CAG8522131.1
Acaulospora colombiana
CAF0905092.1
Brachionus calyciflorus
CAF1638968.1
Didymodactylos carnosus
CAF5224980.1
Rotaria magnacalcarata
CAF4640787.1
Rotaria socialis
CAF4647781.1
Rotaria sp. Silwood2
CAD7651325.1
EPY83871.1
Camelus ferus
XP_009167954.1
Opisthorchis viverrini
VDP58616.1
Schistosoma curassoni
VDQ04428.1
Trichobilharzia regenti
PHJ19819.1
Cystoisospora suis
MCE2055118.1
Datura stramonium
RWW05384.1
Ensete ventricosum
KAB8082965.1
Oryza sativa
KAE9450972.1
Rhododendron williamsianum
GIQ89742.1
Kipferlia bialata
KAH3756923.1
Pelomyxa schiedti
CAF4874872.1
Rotaria socialis
EPY75462.1
Camelus ferus
KAF2616545.1
Brassica cretica
CAD7241661.1
KAF4730536.1
Perkinsus olseni
KAG0001809.1
Entomortierella chlamydospora
Na_Ca_ex Na_Ca_ex PTDSS1/2 MoxR-AAA AAA_lid_7 AAA_7 MoxR-AAA AAA_lid_5 MoxR-AAA AAA_lid_7 MoxR-AAA MDN1_4th MoxR-AAA AAA_lid_7 AAA_28 MoxR-AAA VWA
KAF4670247.1
Perkinsus olseni
Steroid_dh PTDSS1/2
PRW57105.1
Chlorella sorokiniana
ADI PTDSS1/2 Galactosyl_T
XP_009172374.1
Opisthorchis viverrini
PTDSS1/2 RT_RNaseH
XP_005708180.1
Galdieria sulphuraria
Gcd10p PTDSS1/2
VZI37024.1
PTDSS1/2 Melibiase_2 Melibiase_2
VDH94486.1
Mytilus galloprovincialis
HEPN_DZIP3 PTDSS1/2 Dynamin_N
TRZ81711.1
Danionella cerebrum
PTDSS1/2 Nup153 zf-RanBP zf-RanBP zf-RanBP NUDIX_5 ANKs FERM_M
TNN32037.1
Liparis tanakae
Nucleoporin_FG PTDSS1/2
RVX17938.1
Vitis vinifera
RVT_1 zf-RVT PTDSS1/2 zf-RVT
RTG81356.1
Schistosoma bovis
PTDSS1/2 Red
RLO04934.1
Aphanomyces astaci
PSC69583.1
Micractinium conductrix
Pyridoxal_deC PTDSS1/2
PIC40568.1
Caenorhabditis nigoni
PTDSS1/2 PTDSS1/2 WSN ATG17_like Ank BRCT
MQL89622.1
Colocasia esculenta
KRH93168.1
Pseudoloma neurophilia
PTDSS1/2 DUF4779
KFD67707.1
Trichuris suis
PTDSS1/2 tRNA_int_endo RRM_1 PRP38_assoc PWI DEAD Helicase_C
KAG9327732.1
Mortierella alpina
Na_Ca_ex Na_Ca_ex PTDSS1/2
KAG7667521.1
Nannochloris sp. 'desiccata'
RB_A RB_B PTDSS1/2

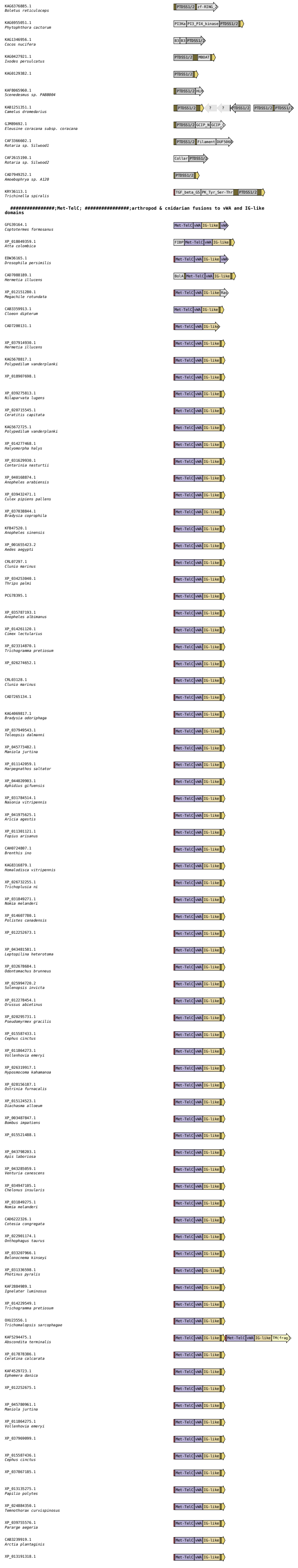
KAG6376885.1
Boletus reticuloceps
PTDSS1/2 zf-RING_2
KAG6955051.1
Phytophthora cactorum
PI3Ka PI3_PI4_kinase PTDSS1/2
KAG1346956.1
Cocos nucifera
B3 B3 PTDSS1/2
KAG0427921.1
Ixodes persulcatus
PTDSS1/2 MBOAT
KAG0129382.1
PTDSS1/2
KAF8065960.1
Scenedesmus sp. PABB004
PTDSS1/2 HLH
KAB1251351.1
Camelus dromedarius
PTDSS1/2 ? ? PTDSS1/2 PTDSS1/2 PTDSS1/2
GJM89692.1
Eleusine coracana subsp. coracana
PTDSS1/2 GCIP_N GCIP_C
CAF3366602.1
Rotaria sp. Silwood1
PTDSS1/2 Filament DUF5068
CAF2615190.1
Rotaria sp. Silwood2
Collar PTDSS1/2
CAD7949252.1
Amoebophrya sp. A120
PTDSS1/2
KRY36113.1
Trichinella spiralis
TGF_beta_GS PK_Tyr_Ser-Thr PTDSS1/2
################;Met-TelC; ################;arthropod & cnidarian fusions to vWA and IG-like domains
GFG39164.1
Coptotermes formosanus
Met-TelC vWA IG-like vWA
XP_018049359.1
Atta colombica
FIBP Met-TelC vWA IG-like
EDW36165.1
Drosophila persimilis
Met-TelC vWA IG-like vWA
CAD7088189.1
Hermetia illucens
BolA Met-TelC vWA IG-like
XP_012151280.1
Megachile rotundata
Met-TelC vWA IG-like Ras
CAB3359913.1
Cloeon dipterum
Met-TelC vWA IG-like
CAD7200131.1
Met-TelC vWA IG-like
XP_037914930.1
Hermetia illucens
Met-TelC vWA IG-like
KAG5678817.1
Polypedilum vanderplanki
Met-TelC vWA IG-like
XP_018907698.1
Met-TelC vWA IG-like
XP_039275813.1
Nilaparvata lugens
Met-TelC vWA IG-like
XP_020715545.1
Ceratitis capitata
Met-TelC vWA IG-like
KAG5672725.1
Polypedilum vanderplanki
Met-TelC vWA IG-like
XP_014277468.1
Halyomorpha halys
Met-TelC vWA IG-like
XP_031629930.1
Contarinia nasturtii
Met-TelC vWA IG-like
XP_040168874.1
Anopheles arabiensis
Met-TelC vWA IG-like
XP_039432471.1
Culex pipiens pallens
Met-TelC vWA IG-like
XP_037038844.1
Bradysia coprophila
Met-TelC vWA IG-like
KFB47520.1
Anopheles sinensis
Met-TelC vWA IG-like
XP_001655423.2
Aedes aegypti
Met-TelC vWA IG-like
CRL07297.1
Clunio marinus
Met-TelC vWA IG-like
XP_034253040.1
Thrips palmi
Met-TelC vWA IG-like
PCG78395.1
Met-TelC vWA IG-like
XP_035787193.1
Anopheles albimanus
Met-TelC vWA IG-like
XP_014261120.1
Cimex lectularius
Met-TelC vWA IG-like
XP_023314870.1
Trichogramma pretiosum
Met-TelC vWA IG-like
XP_026274652.1
Met-TelC vWA IG-like
CRL03128.1
Clunio marinus
Met-TelC vWA IG-like
CAD7265134.1
Met-TelC vWA IG-like
KAG4069817.1
Bradysia odoriphaga
Met-TelC vWA IG-like
XP_037949543.1
Teleopsis dalmanni
Met-TelC vWA IG-like
XP_045773482.1
Maniola jurtina
Met-TelC vWA IG-like
XP_011142059.1
Harpegnathos saltator
Met-TelC vWA IG-like
XP_044020903.1
Aphidius gifuensis
Met-TelC vWA IG-like
XP_031784514.1
Nasonia vitripennis
Met-TelC vWA IG-like
XP_041975625.1
Aricia agestis
Met-TelC vWA IG-like
XP_011301121.1
Fopius arisanus
Met-TelC vWA IG-like
CAH0724807.1
Brenthis ino
Met-TelC vWA IG-like
KAG8316879.1
Homalodisca vitripennis
Met-TelC vWA IG-like
XP_026732255.1
Trichoplusia ni
Met-TelC vWA IG-like
XP_031849271.1
Nomia melanderi
Met-TelC vWA IG-like
XP_014607780.1
Polistes canadensis
Met-TelC vWA IG-like
XP_012252673.1
Met-TelC vWA IG-like
XP_043481581.1
Leptopilina heterotoma
Met-TelC vWA IG-like
XP_032678684.1
Odontomachus brunneus
Met-TelC vWA IG-like
XP_025994720.2
Solenopsis invicta
Met-TelC vWA IG-like
XP_012278454.1
Orussus abietinus
Met-TelC vWA IG-like
XP_020295731.1
Pseudomyrmex gracilis
Met-TelC vWA IG-like
XP_015587433.1
Cephus cinctus
Met-TelC vWA IG-like
XP_011864273.1
Vollenhovia emeryi
Met-TelC vWA IG-like
XP_026319917.1
Hyposmocoma kahamanoa
Met-TelC vWA IG-like
XP_028156187.1
Ostrinia furnacalis
Met-TelC vWA IG-like
XP_015124523.1
Diachasma alloeum
Met-TelC vWA IG-like
XP_003487847.1
Bombus impatiens
Met-TelC vWA IG-like
XP_015521488.1
Met-TelC vWA IG-like
XP_043798203.1
Apis laboriosa
Met-TelC vWA IG-like
XP_043285059.1
Venturia canescens
Met-TelC vWA IG-like
XP_034947105.1
Chelonus insularis
Met-TelC vWA IG-like
XP_031849275.1
Nomia melanderi
Met-TelC vWA IG-like
CAD6222326.1
Cotesia congregata
Met-TelC vWA IG-like
XP_022901174.1
Onthophagus taurus
Met-TelC vWA IG-like
XP_033207966.1
Belonocnema kinseyi
Met-TelC vWA IG-like
XP_031336598.1
Photinus pyralis
Met-TelC vWA IG-like
KAF2884989.1
Ignelater luminosus
Met-TelC vWA IG-like
XP_014229549.1
Trichogramma pretiosum
Met-TelC vWA IG-like
OXU23556.1
Trichomalopsis sarcophagae
Met-TelC vWA IG-like
KAF5294475.1
Abscondita terminalis
Met-TelC vWA IG-like Met-TelC vWA IG-like TM(frag)
XP_017878386.1
Ceratina calcarata
Met-TelC vWA IG-like
KAF4529723.1
Ephemera danica
Met-TelC vWA IG-like
XP_012252675.1
Met-TelC vWA IG-like
XP_045780961.1
Maniola jurtina
Met-TelC vWA IG-like
XP_011864275.1
Vollenhovia emeryi
Met-TelC vWA IG-like
XP_037969099.1
Met-TelC vWA IG-like
XP_015587436.1
Cephus cinctus
Met-TelC vWA IG-like
XP_037867185.1
Met-TelC vWA IG-like
XP_013135275.1
Papilio polytes
Met-TelC vWA IG-like
XP_024884350.1
Temnothorax curvispinosus
Met-TelC vWA IG-like
XP_039755576.1
Pararge aegeria
Met-TelC vWA IG-like
CAB3239919.1
Arctia plantaginis
Met-TelC vWA IG-like
XP_013191318.1
Met-TelC vWA IG-like

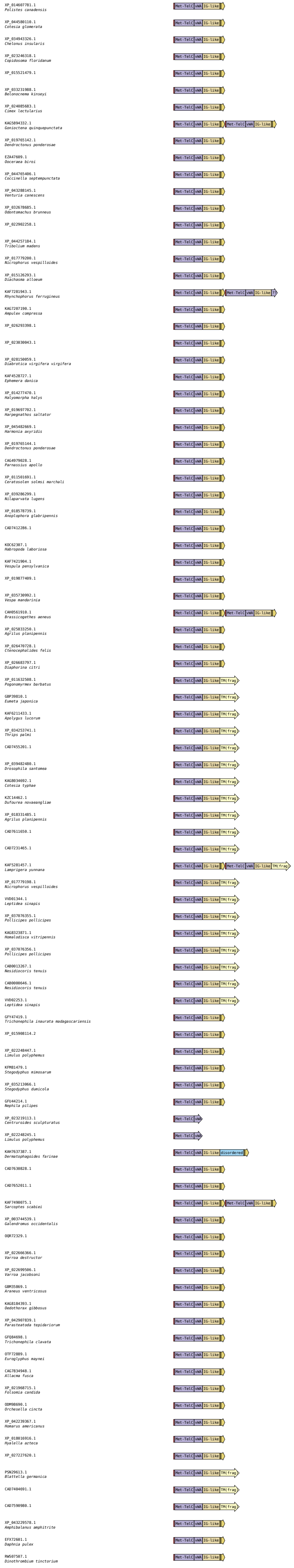
XP_014607781.1
Polistes canadensis
Met-TelC vWA IG-like
XP_044580110.1
Cotesia glomerata
Met-TelC vWA IG-like
XP_034943326.1
Chelonus insularis
Met-TelC vWA IG-like
XP_023246318.1
Copidosoma floridanum
Met-TelC vWA IG-like
XP_015521479.1
Met-TelC vWA IG-like
XP_033231988.1
Belonocnema kinseyi
Met-TelC vWA IG-like
XP_024085683.1
Cimex lectularius
Met-TelC vWA IG-like
KAG5894332.1
Gonioctena quinquepunctata
Met-TelC vWA IG-like Met-TelC vWA IG-like
XP_019765142.1
Dendroctonus ponderosae
Met-TelC vWA IG-like
EZA47609.1
Ooceraea biroi
Met-TelC vWA IG-like
XP_044765406.1
Coccinella septempunctata
Met-TelC vWA IG-like
XP_043288145.1
Venturia canescens
Met-TelC vWA IG-like
XP_032678685.1
Odontomachus brunneus
Met-TelC vWA IG-like
XP_022902258.1
Met-TelC vWA IG-like
XP_044257184.1
Tribolium madens
Met-TelC vWA IG-like
XP_017779200.1
Nicrophorus vespilloides
Met-TelC vWA IG-like
XP_015126293.1
Diachasma alloeum
Met-TelC vWA IG-like
KAF7281943.1
Rhynchophorus ferrugineus
Met-TelC vWA IG-like Met-TelC vWA IG-like ?
KAG7207190.1
Ampulex compressa
Met-TelC vWA IG-like
XP_026293398.1
Met-TelC vWA IG-like
XP_023030043.1
Met-TelC vWA IG-like
XP_028150059.1
Diabrotica virgifera virgifera
Met-TelC vWA IG-like
KAF4528727.1
Ephemera danica
Met-TelC vWA IG-like
XP_014277470.1
Halyomorpha halys
Met-TelC vWA IG-like
XP_019697702.1
Harpegnathos saltator
Met-TelC vWA IG-like
XP_045482669.1
Harmonia axyridis
Met-TelC vWA IG-like
XP_019765144.1
Dendroctonus ponderosae
Met-TelC vWA IG-like
CAG4979028.1
Parnassius apollo
Met-TelC vWA IG-like
XP_011501691.1
Ceratosolen solmsi marchali
Met-TelC vWA IG-like
XP_039286299.1
Nilaparvata lugens
Met-TelC vWA IG-like
XP_018578739.1
Anoplophora glabripennis
Met-TelC vWA IG-like
CAD7412286.1
Met-TelC vWA IG-like
KOC62307.1
Habropoda laboriosa
Met-TelC vWA IG-like
KAF7421904.1
Vespula pensylvanica
Met-TelC vWA IG-like
XP_019877409.1
Met-TelC vWA IG-like
XP_035730992.1
Vespa mandarinia
Met-TelC vWA IG-like
CAH0561910.1
Brassicogethes aeneus
Met-TelC vWA IG-like Met-TelC vWA IG-like
XP_025833250.1
Agrilus planipennis
Met-TelC vWA IG-like
XP_026470728.1
Ctenocephalides felis
Met-TelC vWA IG-like
XP_026683797.1
Diaphorina citri
Met-TelC vWA IG-like
XP_011632508.1
Pogonomyrmex barbatus
Met-TelC vWA IG-like TM(frag)
GBP39810.1
Eumeta japonica
Met-TelC vWA IG-like TM(frag)
KAF6211433.1
Apolygus lucorum
Met-TelC vWA IG-like TM(frag)
XP_034253741.1
Thrips palmi
Met-TelC vWA IG-like TM(frag)
CAD7455201.1
Met-TelC vWA IG-like TM(frag)
XP_039482480.1
Drosophila santomea
Met-TelC vWA IG-like TM(frag)
KAG8034692.1
Cotesia typhae
Met-TelC vWA IG-like TM(frag)
KZC14462.1
Dufourea novaeangliae
Met-TelC vWA IG-like TM(frag)
XP_018331485.1
Agrilus planipennis
Met-TelC vWA IG-like TM(frag)
CAD7611650.1
Met-TelC vWA IG-like TM(frag)
CAD7231465.1
Met-TelC vWA IG-like TM(frag)
KAF5281457.1
Lamprigera yunnana
Met-TelC vWA IG-like Met-TelC vWA IG-like TM(frag)
XP_017779198.1
Nicrophorus vespilloides
Met-TelC vWA IG-like TM(frag)
VVD01344.1
Leptidea sinapis
Met-TelC vWA IG-like TM(frag)
XP_037076355.1
Pollicipes pollicipes
Met-TelC vWA IG-like TM(frag)
KAG8323871.1
Homalodisca vitripennis
Met-TelC vWA IG-like TM(frag)
XP_037076356.1
Pollicipes pollicipes
Met-TelC vWA IG-like TM(frag)
CAB0013267.1
Nesidiocoris tenuis
Met-TelC vWA IG-like TM(frag)
CAB0000646.1
Nesidiocoris tenuis
Met-TelC vWA IG-like TM(frag)
VVD02253.1
Leptidea sinapis
Met-TelC vWA IG-like TM(frag)
GFY47419.1
Trichonephila inaurata madagascariensis
Met-TelC vWA IG-like
XP_015908114.2
Met-TelC vWA IG-like
XP_022248447.1
Limulus polyphemus
Met-TelC vWA IG-like
KFM81479.1
Stegodyphus mimosarum
Met-TelC vWA IG-like
XP_035213066.1
Stegodyphus dumicola
Met-TelC vWA IG-like
GFU44214.1
Nephila pilipes
Met-TelC vWA IG-like
XP_023219113.1
Centruroides sculpturatus
Met-TelC vWA
XP_022248245.1
Limulus polyphemus
Met-TelC vWA
KAH7637387.1
Dermatophagoides farinae
Met-TelC vWA IG-like disordered
CAD7630828.1
Met-TelC vWA IG-like
CAD7652011.1
Met-TelC vWA IG-like
KAF7490075.1
Sarcoptes scabiei
Met-TelC vWA IG-like Met-TelC vWA IG-like
XP_003744539.1
Galendromus occidentalis
Met-TelC vWA IG-like
OQR72329.1
Met-TelC vWA IG-like
XP_022666366.1
Varroa destructor
Met-TelC vWA IG-like
XP_022699506.1
Varroa jacobsoni
Met-TelC vWA IG-like
GBM35869.1
Araneus ventricosus
Met-TelC vWA IG-like
KAG8184393.1
Oedothorax gibbosus
Met-TelC vWA IG-like
XP_042907839.1
Parasteatoda tepidariorum
Met-TelC vWA IG-like
GFQ84698.1
Trichonephila clavata
Met-TelC vWA IG-like
OTF72889.1
Euroglyphus maynei
Met-TelC vWA IG-like
CAG7834948.1
Allacma fusca
Met-TelC vWA IG-like
XP_021968715.1
Folsomia candida
Met-TelC vWA IG-like
ODM98690.1
Orchesella cincta
Met-TelC vWA IG-like
XP_042239367.1
Homarus americanus
Met-TelC vWA IG-like
XP_018016916.1
Hyalella azteca
Met-TelC vWA IG-like
XP_027227620.1
Met-TelC vWA IG-like
PSN29613.1
Blattella germanica
Met-TelC vWA IG-like TM(frag)
CAD7404691.1
Met-TelC vWA IG-like TM(frag)
CAD7590980.1
Met-TelC vWA IG-like TM(frag)
XP_043229578.1
Amphibalanus amphitrite
Met-TelC vWA IG-like
EFX72601.1
Daphnia pulex
Met-TelC vWA IG-like
RWS07507.1
Dinothrombium tinctorium
Met-TelC vWA IG-like

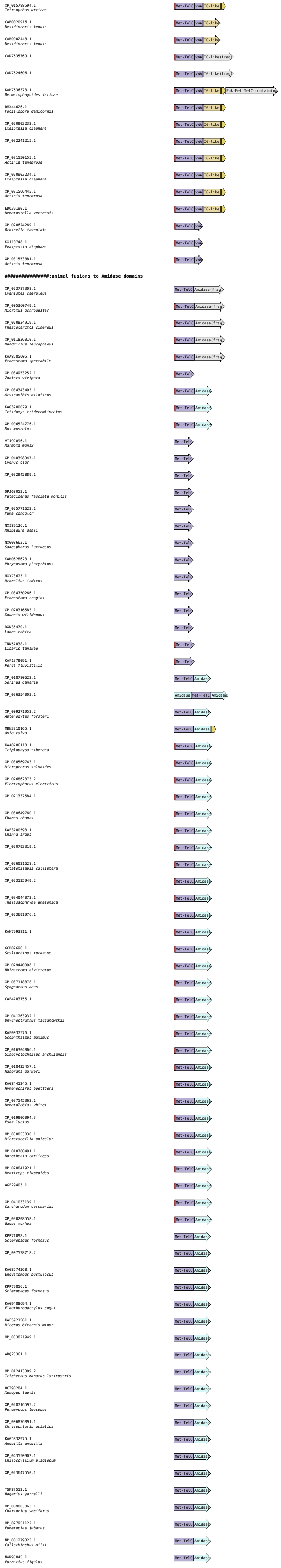
XP_015788594.1
Tetranychus urticae
Met-TelC vWA IG-like
CAB0020916.1
Nesidiocoris tenuis
Met-TelC vWA IG-like
CAB0002448.1
Nesidiocoris tenuis
Met-TelC vWA IG-like
CAD7635769.1
Met-TelC vWA IG-like(frag)
CAD7624606.1
Met-TelC vWA IG-like(frag)
KAH7636373.1
Dermatophagoides farinae
Met-TelC vWA IG-like Euk-Met-TelC-containing
RMX44626.1
Pocillopora damicornis
Met-TelC vWA IG-like
XP_020903232.1
Exaiptasia diaphana
Met-TelC vWA IG-like
XP_032241215.1
Met-TelC vWA IG-like
XP_031550155.1
Actinia tenebrosa
Met-TelC vWA IG-like
XP_020903234.1
Exaiptasia diaphana
Met-TelC vWA IG-like
XP_031566445.1
Actinia tenebrosa
Met-TelC vWA IG-like
EDO39190.1
Nematostella vectensis
Met-TelC vWA IG-like
XP_020624269.1
Orbicella faveolata
Met-TelC vWA
KXJ10748.1
Exaiptasia diaphana
Met-TelC vWA
XP_031553881.1
Actinia tenebrosa
Met-TelC vWA
################;animal fusions to Amidase domains
XP_023787308.1
Cyanistes caeruleus
Met-TelC Amidase(frag)
XP_005360749.1
Microtus ochrogaster
Met-TelC Amidase(frag)
XP_020824919.1
Phascolarctos cinereus
Met-TelC Amidase(frag)
XP_011836010.1
Mandrillus leucophaeus
Met-TelC Amidase(frag)
KAA8585605.1
Etheostoma spectabile
Met-TelC Amidase(frag)
XP_034953252.1
Zootoca vivipara
Met-TelC
XP_034343493.1
Arvicanthis niloticus
Met-TelC Amidase
KAG3280029.1
Ictidomys tridecemlineatus
Met-TelC Amidase
XP_006524776.1
Mus musculus
Met-TelC Amidase
VTJ92096.1
Marmota monax
Met-TelC
XP_040398947.1
Cygnus olor
Met-TelC
XP_032942889.1
Met-TelC
OPJ68053.1
Patagioenas fasciata monilis
Met-TelC
XP_025771622.1
Puma concolor
Met-TelC
NXI89126.1
Rhipidura dahli
Met-TelC
NXG08663.1
Sakesphorus luctuosus
Met-TelC
KAH0628623.1
Phrynosoma platyrhinos
Met-TelC
NXX73623.1
Urocolius indicus
Met-TelC
XP_034750266.1
Etheostoma cragini
Met-TelC
XP_028316583.1
Gouania willdenowi
Met-TelC
RXN35470.1
Labeo rohita
Met-TelC
TNN57838.1
Liparis tanakae
Met-TelC
KAF1379091.1
Perca fluviatilis
Met-TelC
XP_018780622.1
Serinus canaria
Met-TelC Amidase
XP_036354003.1
Amidase Met-TelC Amidase
XP_009271952.2
Aptenodytes forsteri
Met-TelC Amidase
MBN3310165.1
Amia calva
Met-TelC Amidase
KAA0706110.1
Triplophysa tibetana
Met-TelC Amidase
XP_038569743.1
Micropterus salmoides
Met-TelC Amidase
XP_026862373.2
Electrophorus electricus
Met-TelC Amidase
XP_021332584.1
Met-TelC Amidase
XP_030649760.1
Chanos chanos
Met-TelC Amidase
KAF3700593.1
Channa argus
Met-TelC Amidase
XP_020793319.1
Met-TelC Amidase
XP_026021628.1
Astatotilapia calliptera
Met-TelC Amidase
XP_023125949.2
Met-TelC Amidase
XP_034044072.1
Thalassophryne amazonica
Met-TelC Amidase
XP_023691976.1
Met-TelC Amidase
KAH7993811.1
Met-TelC Amidase
GCB82698.1
Scyliorhinus torazame
Met-TelC Amidase
XP_029440098.1
Rhinatrema bivittatum
Met-TelC Amidase
XP_037118878.1
Syngnathus acus
Met-TelC Amidase
CAF4783755.1
Met-TelC Amidase
XP_041263932.1
Onychostruthus taczanowskii
Met-TelC Amidase
KAF0037576.1
Scophthalmus maximus
Met-TelC Amidase
XP_016304066.1
Sinocyclocheilus anshuiensis
Met-TelC Amidase
XP_018422457.1
Nanorana parkeri
Met-TelC Amidase
KAG8441245.1
Hymenochirus boettgeri
Met-TelC Amidase
XP_037545362.1
Nematolebias whitei
Met-TelC Amidase
XP_019906094.3
Esox lucius
Met-TelC Amidase
XP_030053030.1
Microcaecilia unicolor
Met-TelC Amidase
XP_010788491.1
Notothenia coriiceps
Met-TelC Amidase
XP_028841921.1
Denticeps clupeoides
Met-TelC Amidase
AGF29403.1
Met-TelC Amidase
XP_041033139.1
Carcharodon carcharias
Met-TelC Amidase
XP_030208558.1
Gadus morhua
Met-TelC Amidase
KPP71098.1
Scleropages formosus
Met-TelC Amidase
XP_007538718.2
Met-TelC Amidase
KAG8574368.1
Engystomops pustulosus
Met-TelC Amidase
KPP79856.1
Scleropages formosus
Met-TelC Amidase
KAG9488694.1
Eleutherodactylus coqui
Met-TelC Amidase
KAF5921561.1
Diceros bicornis minor
Met-TelC Amidase
XP_033821949.1
Met-TelC Amidase
ABQ23361.1
Met-TelC Amidase
XP_012413309.2
Trichechus manatus latirostris
Met-TelC Amidase
OCT90284.1
Xenopus laevis
Met-TelC Amidase
XP_028716595.2
Peromyscus leucopus
Met-TelC Amidase
XP_006876891.1
Chrysochloris asiatica
Met-TelC Amidase
KAG5832975.1
Anguilla anguilla
Met-TelC Amidase
XP_043550902.1
Chiloscyllium plagiosum
Met-TelC Amidase
XP_023647550.1
Met-TelC Amidase
TSK87512.1
Bagarius yarrelli
Met-TelC Amidase
XP_009883863.1
Charadrius vociferus
Met-TelC Amidase
XP_027951122.1
Eumetopias jubatus
Met-TelC Amidase
NP_001279323.1
Callorhinchus milii
Met-TelC Amidase
NWR95845.1
Furnarius figulus
Met-TelC Amidase

XP_012686651.2	*Clupea harengus*	Met-TelC Amidase
KAG7279699.1	*Coryphaenoides rupestris*	Met-TelC Amidase
XP_015234835.1	*Cyprinodon variegatus*	Met-TelC Amidase
AIZ75056.1		Met-TelC Amidase
XP_008574627.1	*Galeopterus variegatus*	Met-TelC Amidase
NXG18851.1	*Grallaria varia*	Met-TelC Amidase
NXS63946.1	*Brachypteracias leptosomus*	Met-TelC Amidase
XP_006002990.1	*Latimeria chalumnae*	Met-TelC Amidase
KAG5279762.1	*Alosa alosa*	Met-TelC Amidase
XP_036383860.1	*Megalops cyprinoides*	Met-TelC Amidase
KAA0713384.1	*Triplophysa tibetana*	Met-TelC Amidase
XP_042748723.1	*Lagopus leucura*	Met-TelC Amidase
XP_034544840.1	*Notolabrus celidotus*	Met-TelC Amidase
XP_032906856.1	*Amblyraja radiata*	Met-TelC Amidase
KAF3846822.1	*Dissostichus mawsoni*	Met-TelC Amidase
KAF5880242.1	*Clarias magur*	Met-TelC Amidase
MBN3324215.1	*Atractosteus spatula*	Met-TelC Amidase
NWI69923.1	*Todus mexicanus*	Met-TelC Amidase
NXK69829.1	*Sylvietta virens*	Met-TelC Amidase
XP_042307698.1	*Sceloporus undulatus*	Met-TelC Amidase
NXP70488.1	*Ramphastos sulfuratus*	Met-TelC Amidase
NWY07982.1	*Nothoprocta ornata*	Met-TelC Amidase
NXS12523.1	*Neodrepanis coruscans*	Met-TelC Amidase
NXD74558.1	*Eolophus roseicapilla*	Met-TelC Amidase
NWV56848.1	*Daphoenositta chrysoptera*	Met-TelC Amidase
NWS78382.1	*Crotophaga sulcirostris*	Met-TelC Amidase
NWY22567.1	*Aphelocoma coerulescens*	Met-TelC Amidase
XP_015832183.1		Met-TelC Amidase
XP_038025267.1		Met-TelC Amidase KAP-glycosylase C2 RasGAP Amidase
EMP26326.1	*Chelonia mydas*	Met-TelC Amidase C2 RasGAP DAB2P-C
ERE72265.1	*Cricetulus griseus*	Met-TelC Amidase C2 RasGAP DUF4763
XP_030638580.1	*Chanos chanos*	Met-TelC Amidase PH C2 RasGAP UPF0242
MXQ93498.1	*Bos mutus*	Met-TelC Amidase RasGAP ZnF-repeats
KFO37403.1	*Fukomys damarensis*	Met-TelC Amidase RasGAP ATG16

#;fragments and rare architectures

XP_028518783.1	*Exaiptasia diaphana*	Met-TelC vWA EGF EGF IG-like
PFX28088.1	*Stylophora pistillata*	Met-TelC RVT VWA
EDO29847.1	*Nematostella vectensis*	Met-TelC
ADX31898.1		Met-TelC Amidase
KAF3852298.1	*Dissostichus mawsoni*	Met-TelC Amidase
ROL53015.1	*Anabarilius grahami*	Met-TelC ZnF-repeats
XP_016846275.1		Met-TelC Amidase
RMC10974.1	*Hirundo rustica rustica*	Met-TelC Amidase Lipoxygenase
RLV85401.1	*Chloebia gouldiae*	Met-TelC Amidase
RMB91427.1	*Hirundo rustica rustica*	Met-TelC ZnF-repeats
XP_013382085.1	*Lingula anatina*	Met-TelC ZnF
CAG7819605.1	*Allacma fusca*	Met-TelC
GBP98208.1	*Eumeta japonica*	Met-TelC
KAG8228521.1	*Ladona fulva*	Met-TelC
KOB78554.1	*Operophtera brumata*	Met-TelC
CAB0028367.1	*Trichogramma brassicae*	Met-TelC Anks
XP_002433161.1	*Pediculus humanus corporis*	Met-TelC
XP_043672144.1	*Vespula pensylvanica*	Met-TelC
XP_026683795.1	*Diaphorina citri*	Met-TelC
CAB3371313.1	*Cloeon dipterum*	Euk-Tel Myosin
TRY77882.1	*Tigriopus californicus*	Met-TelC
CAD7280959.1		Met-TelC
KAG0714986.1	*Chionoecetes opilio*	Met-TelC
CAD7248107.1		Met-TelC-containing
CAD7246025.1		Met-TelC
XP_037509362.1		Met-TelC
XP_023233168.1	*Centruroides sculpturatus*	Met-TelC
GBN58329.1	*Araneus ventricosus*	Met-TelC
KAG1683176.1	*Nymphon striatum*	Met-TelC
CAD7635435.1		Met-TelC
CAD7666923.1		Met-TelC
XP_029838277.3	*Ixodes scapularis*	Met-TelC vWA
XP_037561205.1		Met-TelC
EEC08684.1	*Ixodes scapularis*	Met-TelC
XP_022660505.1	*Varroa destructor*	Met-TelC
KAG1648356.1	*Nymphon striatum*	Met-TelC

#################;Prok-PTDSS; ####;versions lacking circular permutation

MAV70985.1	*Fidelibacterota bacterium*	Prok-PTDSS-no-cp
MBI89660.1	*Fidelibacterota bacterium*	Prok-PTDSS-no-cp
MBT5783009.1	*Fidelibacterota bacterium*	Prok-PTDSS-no-cp
MBQ83040.1	*Gammaproteobacteria bacterium*	Prok-PTDSS-no-cp
HIA80031.1	*Fidelibacterota bacterium*	Prok-PTDSS-no-cp
GIR75656.1	*Candidatus Poseidoniales archaeon*	Prok-PTDSS-no-cp
RLF35206.1	*Thermoplasmata archaeon*	Prok-PTDSS-no-cp
RLI51307.1	*Candidatus Bathyarchaeota archaeon*	Prok-PTDSS-no-cp
MAU36405.1	*Flavobacteriales bacterium*	Prok-PTDSS-no-cp
MAR50590.1	*Paracoccaceae bacterium*	Prok-PTDSS-no-cp
MBL4898179.1	*Colwellia sp.*	Prok-PTDSS-no-cp
MBL96243.1	*Legionellales bacterium*	Prok-PTDSS-no-cp
NBO54834.1	*Actinomycetota bacterium*	Prok-PTDSS-no-cp
NBQ43697.1	*Mycobacteriaceae bacterium*	Prok-PTDSS-no-cp
QBK85880.1		Prok-PTDSS-no-cp
QBK86384.1		Prok-PTDSS-no-cp
QBK87868.1		Prok-PTDSS-no-cp Prok-PTDSS-no-cp
AEQ60687.1	*Acanthamoeba castellanii mamavirus*	Prok-PTDSS-no-cp
BCS83038.1	*Cotonvirus japonicus*	Prok-PTDSS-no-cp
ARF09884.1	*Indivirus ILV1*	Prok-PTDSS-no-cp
YP_004894433.1	*Megavirus chiliensis*	Prok-PTDSS-no-cp
QGR53848.1	*Moumouvirus maliensis*	Prok-PTDSS-no-cp

QKU34224.1		Prok-PTDSS-no-cp
QKU34443.1		Prok-PTDSS-no-cp
QKU35776.1		Prok-PTDSS-no-cp
QIG60187.1	*Dishui Lake large algae virus 1*	Prok-PTDSS-no-cp
NDE17539.1	*bacterium*	Prok-PTDSS-no-cp
AYV81799.1	*Harvfovirus sp.*	Prok-PTDSS-no-cp
AMN83651.1	*Faustovirus*	Prok-PTDSS-no-cp
SME64917.1	*Faustovirus ST1*	Prok-PTDSS-no-cp
QYB17778.1	*Pacmanvirus S19*	Prok-PTDSS-no-cp
KPJ58557.1	*Latescibacteria bacterium DG_63*	Prok-PTDSS-no-cp
MAU36516.1	*Flavobacteriales bacterium*	Prok-PTDSS-no-cp
AET43567.1	*Micromonas pusilla virus PL1*	Prok-PTDSS-no-cp
YP_004062057.1	*Micromonas sp. RCC1109 virus MpV1*	Prok-PTDSS-no-cp
TET11121.1	*Candidatus Thorarchaeota archaeon*	Prok-PTDSS-no-cp
YP_009174733.1	*Yellowstone lake phycodnavirus 1*	Prok-PTDSS-no-cp
YP_009665611.1	*Feldmannia irregularis virus a*	Prok-PTDSS-no-cp

####;versions with only the 3-helix Lipocone core

MBL6732194.1	*Candidatus Poseidoniaceae archaeon*	Prok-PTDSS-3helix
RJU82846.1	*Candidatus Poseidoniales archaeon*	Prok-PTDSS-3helix
HIE82048.1	*Candidatus Poseidoniales archaeon*	Prok-PTDSS-3helix
MBT5274907.1	*Candidatus Woesearchaeota archaeon*	Prok-PTDSS-3helix
MAK97355.1	*Methanobacteriota archaeon*	Prok-PTDSS-3helix
MAU74776.1	*Methanobacteriota archaeon*	Prok-PTDSS-3helix
MAS62562.1	*Methanobacteriota archaeon*	Prok-PTDSS-3helix
MBQ70822.1	*Methanobacteriota archaeon*	Prok-PTDSS-3helix
MBS84244.1	*Methanobacteriota archaeon*	Prok-PTDSS-3helix
MBD18986.1	*Methanobacteriota archaeon*	Prok-PTDSS-3helix
MBA86908.1	*Methanobacteriota archaeon*	Prok-PTDSS-3helix
PXY70995.1	*Methanobacteriota archaeon*	Prok-PTDSS-3helix
MBT5284661.1	*Methanobacteriota archaeon*	Prok-PTDSS-3helix
MBT4920362.1	*Methanobacteriota archaeon*	Prok-PTDSS-3helix
RPG69422.1	*Euryarchaeota archaeon TMED99*	Prok-PTDSS-3helix
HCQ99731.1	*Acidobacteriota bacterium*	Prok-PTDSS-3helix
MAG21161.1	*Fidelibacterota bacterium*	Prok-PTDSS-3helix
MAI87067.1	*Fidelibacterota bacterium*	Prok-PTDSS-3helix
MAR75995.1	*Fidelibacterota bacterium*	Prok-PTDSS-3helix
MBA65439.1	*Fidelibacterota bacterium*	Prok-PTDSS-3helix
MBL46928.1	*Fidelibacterota bacterium*	Prok-PTDSS-3helix
MBH31785.1	*Fidelibacterota bacterium*	Prok-PTDSS-3helix
PCJ12490.1	*Fidelibacterota bacterium*	Prok-PTDSS-3helix
HIN26067.1	*Fidelibacterota bacterium*	Prok-PTDSS-3helix
MBT5782444.1	*Fidelibacterota bacterium*	Prok-PTDSS-3helix
NQT63393.1	*Fidelibacterota bacterium*	Prok-PTDSS-3helix
MBQ23302.1	*Flavobacteriales bacterium*	Prok-PTDSS-3helix
MBE26891.1	*Saprospirales bacterium*	Prok-PTDSS-3helix
HBU57294.1	*Gemmatimonadota bacterium*	Prok-PTDSS-3helix
MAV38260.1	*Planctomycetaceae bacterium*	Prok-PTDSS-3helix
MBE31674.1	*Rickettsiales bacterium*	Prok-PTDSS-3helix
MBQ82189.1	*Gammaproteobacteria bacterium*	Prok-PTDSS-3helix
MBI79525.1	*Gammaproteobacteria bacterium*	Prok-PTDSS-3helix
OUU78316.1	*Gammaproteobacteria bacterium TMED78*	Prok-PTDSS-3helix
MAD57179.1	*Porticoccus sp.*	Prok-PTDSS-3helix
OUW49837.1	*bacterium TMED190*	Prok-PTDSS-3helix

####;versions fused to DUF6122 helical domain

MBC8311969.1	*Fidelibacterota bacterium*	DUF6122 Prok-PTDSS
HIA30082.1	*Fidelibacterota bacterium*	DUF6122 Prok-PTDSS

####;version with a C-terminal cp helix

VBB18269.1	*Yasminevirus sp. GU-2018*	Prok-PTDSS

####;fused to unknown N-terminal domain

OLS19732.1	*Candidatus Heimdallarchaeota archaeon LC_2*	?? Prok-PTDSS

##################;Prok-SAA; ##################;classical polymorphic and allied systems

HES96575.1		RHS-repeats Prok-SAA
ECG1194407.1	*Salmonella bongori*	RHS-repeats Prok-SAA; SAA-imm-2
MBK6864798.1	*Ideonella sp.*	RHS-repeats Prok-SAA; Imm-SAA
MCC5846437.1	*Verrucomicrobiota bacterium*	RHS-repeats Prok-SAA
EDP8625052.1	*Salmonella bongori*	RHS-repeats Prok-SAA; SAA-imm-2
MCB1054655.1	*Acidobacteriota bacterium*	RHS-repeats Prok-SAA
MBK7706223.1	*Acidobacteriota bacterium*	RHS-repeats Prok-SAA
UGS39949.1	*Pseudocitrobacter corydidari*	RHS-repeats Prok-SAA
MBI4001192.1	*Nitrospira defluvii*	RHS-repeats Prok-SAA
KAA8997699.1	*Affinibrenneria salicis*	RHS-repeats Prok-SAA; SAA-imm-2
SDZ63196.1	*Delftia lacustris*	RHS-repeats Prok-SAA
HEI71611.1		RHS-repeats Prok-SAA; ?; RHS-repeats
QAA47302.1	*Akkermansia muciniphila*	RHS-repeats Prok-SAA; alpha-helix 2-hairpin; RHS-repeats
MBU6408680.1	*Alphaproteobacteria bacterium*	RHS-repeats Prok-SAA; Imm-SAA
UDQ98389.1	*Lentisphaerae bacterium WC36*	RHS-repeats Prok-SAA
AVJ34923.1	*Stenotrophomonas sp. MYb57*	RHS-repeats Prok-SAA
MBV9572581.1	*Terriglobales bacterium*	RHS-repeats Prok-SAA
KAB3615898.1	*Phocaeicola vulgatus*	RHS-repeats Prok-SAA; alpha-helix 2-hairpin; RHS-repeats
PIT16680.1	*Snodgrassella alvi*	RHS-repeats Prok-SAA
KAB3640194.1	*Phocaeicola vulgatus*	RHS-repeats Prok-SAA; alpha-helix 2-hairpin; RHS-repeats
MBA2586230.1	*Chthoniobacterales bacterium*	RHS-repeats Prok-SAA
MBW2562413.1	*Deltaproteobacteria bacterium*	RHS-repeats Prok-SAA; Imm-SAA
SOB59839.1	*Pseudodesulfovibrio profundus*	RHS-repeats Prok-SAA; ?; Imm-SAA
MBP7826453.1	*Verrucomicrobiota bacterium*	RHS-repeats Prok-SAA; SAA-imm-2
MBM9555392.1		RHS-repeats Prok-SAA; ?; Imm-SAA
MBY0416403.1	*Bdellovibrionales bacterium*	RHS-repeats Prok-SAA
HBA3915027.1	*Escherichia coli*	RHS-repeats Prok-SAA
MBK7657970.1	*Betaproteobacteria bacterium*	RHS-repeats Prok-SAA
MCC6709936.1	*Gammaproteobacteria bacterium*	RHS-repeats Prok-SAA
MBI3756231.1	*Deltaproteobacteria bacterium*	RHS-repeats Prok-SAA
MBK8151177.1	*Acidobacteriota bacterium*	RHS-repeats; Prok-SAA OB-like
MBA4148776.1	*Verrucomicrobiota bacterium*	RHS-repeats; Prok-SAA
AAM40934.1	*Xanthomonas campestris pv. campestris str. ATCC 33913*	RHS-repeats; Prok-SAA

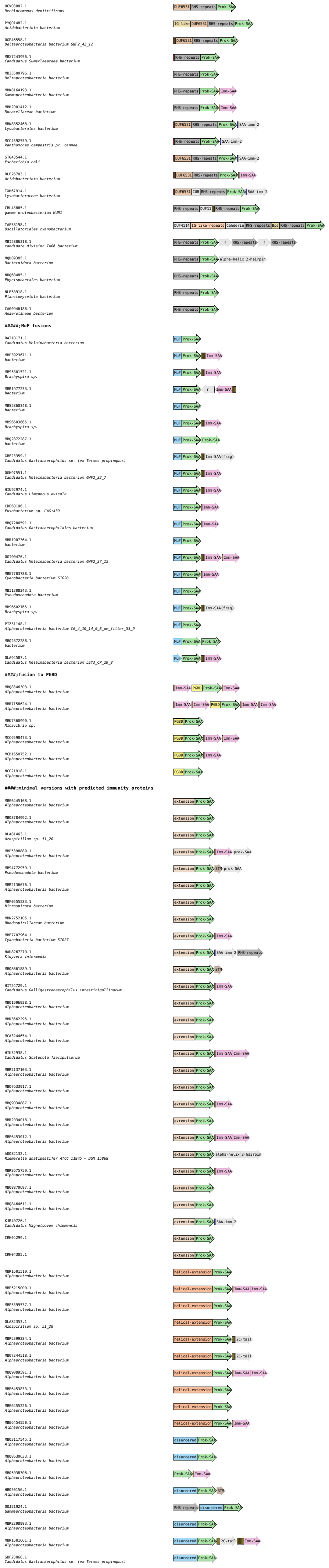
UCV03882.1
Dechloromonas denitrificans
DUF6531 RHS-repeats Prok-SAA
PYQ01482.1
Acidobacteriota bacterium
IG-like DUF6531 RHS-repeats Prok-SAA
OGP46558.1
Deltaproteobacteria bacterium GWF2_42_12
DUF6531 RHS-repeats Prok-SAA
MBX7243956.1
Candidatus Sumerlaeaceae bacterium
RHS-repeats Prok-SAA
MBI5500796.1
Deltaproteobacteria bacterium
RHS-repeats Prok-SAA
MBK8164193.1
Gammaproteobacteria bacterium
RHS-repeats Prok-SAA Imm-SAA
MBH2001412.1
Moraxellaceae bacterium
RHS-repeats Prok-SAA Imm-SAA
MBW8852468.1
Lysobacterales bacterium
DUF6531 RHS-repeats Prok-SAA SAA-imm-2
MCC4592559.1
Xanthomonas campestris pv. cannae
RHS-repeats Prok-SAA SAA-imm-2
STG43544.1
Escherichia coli
DUF6531 RHS-repeats Prok-SAA SAA-imm-2
RLE26703.1
Acidobacteriota bacterium
DUF6531 RHS-repeats Prok-SAA Imm-SAA
TXH67914.1
Lysobacteraceae bacterium
DUF6531 CUB RHS-repeats Prok-SAA SAA-imm-2
CBL43865.1
gamma proteobacterium HdN1
RHS-repeats DUF11 RHS-repeats Prok-SAA
TAF58198.1
Oscillatoriales cyanobacterium
DUF4114 IG-like-repeats Cahderin RHS-repeats Bps RHS-repeats Prok-SAA
MBI5806318.1
candidate division TA06 bacterium
RHS-repeats Prok-SAA ? RHS-repeats ? RHS-repeats
NQU89385.1
Bacteroidota bacterium
RHS-repeats Prok-SAA alpha-helix 2-hairpin
NUQ68485.1
Phycisphaerales bacterium
RHS-repeats Prok-SAA
NLE58918.1
Planctomycetota bacterium
RHS-repeats Prok-SAA
CAG0946188.1
Anaerolineae bacterium
RHS-repeats Prok-SAA
#####;MuF fusions
RAI10171.1
Candidatus Melainabacteria bacterium
MuF Prok-SAA
MBP3923671.1
bacterium
MuF Prok-SAA Imm-SAA
MBS5801521.1
Brachyspira sp.
MuF Prok-SAA Imm-SAA
MBR1977233.1
bacterium
MuF Prok-SAA ? Imm-SAA
MBS5860348.1
bacterium
MuF Prok-SAA
MBS6603665.1
Brachyspira sp.
MuF Prok-SAA Imm-SAA
MBQ2872287.1
bacterium
MuF Prok-SAA Prok-SAA
GBF23359.1
Candidatus Gastranaerophilus sp. (ex Termes propinquus)
MuF Prok-SAA Imm-SAA(frag)
OGH97551.1
Candidatus Melainabacteria bacterium GWF2_32_7
MuF Prok-SAA Imm-SAA
HIU92974.1
Candidatus Limenecus avicola
MuF Prok-SAA Imm-SAA
CDE60196.1
Fusobacterium sp. CAG:439
MuF Prok-SAA Imm-SAA
MBQ7286591.1
Candidatus Gastranaerophilales bacterium
MuF Prok-SAA Imm-SAA
MBR1907364.1
bacterium
MuF Prok-SAA
OGI00476.1
Candidatus Melainabacteria bacterium GWF2_37_15
MuF Prok-SAA Imm-SAA Imm-SAA
MBE7703788.1
Cyanobacteria bacterium SIG28
MuF Prok-SAA Imm-SAA
MBI1308243.1
Pseudomonadota bacterium
MuF Prok-SAA
MBS6602765.1
Brachyspira sp.
MuF Prok-SAA Imm-SAA(frag)
PIZ31148.1
Alphaproteobacteria bacterium CG_4_10_14_0_8_um_filter_53_9
MuF Prok-SAA
MBQ2872288.1
bacterium
MuF Prok-SAA Prok-SAA
OLA94587.1
Candidatus Melainabacteria bacterium LEY3_CP_29_8
MuF Prok-SAA Imm-SAA
####;fusion to PGBD
MBQ8346303.1
Alphaproteobacteria bacterium
Imm-SAA PGBD Prok-SAA Imm-SAA
MBR7158624.1
Alphaproteobacteria bacterium
Imm-SAA Imm-SAA PGBD Prok-SAA Imm-SAA Imm-SAA
MBK7360990.1
Micavibrio sp.
PGBD Prok-SAA
MCC6598473.1
Alphaproteobacteria bacterium
PGBD Prok-SAA Imm-SAA Imm-SAA
MCB1650752.1
Alphaproteobacteria bacterium
PGBD Prok-SAA Imm-SAA
NCC21918.1
Alphaproteobacteria bacterium
PGBD Prok-SAA
####;minimal versions with predicted immunity proteins
MBE6445168.1
Alphaproteobacteria bacterium
extension Prok-SAA
MBQ8784992.1
Alphaproteobacteria bacterium
extension Prok-SAA
OLA81463.1
Azospirillum sp. 51_20
extension Prok-SAA
MBP5398889.1
Alphaproteobacteria bacterium
extension Prok-SAA Imm-SAA prok-SAA
MBS4772959.1
Pseudomonadota bacterium
extension Prok-SAA 3TM prok-SAA
MBR2136676.1
Alphaproteobacteria bacterium
extension Prok-SAA
MBF0555583.1
Nitrospirota bacterium
extension Prok-SAA
MBN2752105.1
Rhodospirillaceae bacterium
extension Prok-SAA
MBE7707964.1
Cyanobacteria bacterium SIG27
extension Prok-SAA Imm-SAA
HAU8267270.1
Kluyvera intermedia
extension Prok-SAA SAA-imm-2 RHS-repeats
MBQ8661889.1
Alphaproteobacteria bacterium
extension Prok-SAA 3TM
HIT54729.1
Candidatus Galligastranaerophilus intestinigallinarum
extension Prok-SAA Imm-SAA
MBQ1996928.1
Alphaproteobacteria bacterium
extension Prok-SAA
MBR3662295.1
Alphaproteobacteria bacterium
extension Prok-SAA
MCA3244654.1
Alphaproteobacteria bacterium
extension Prok-SAA
HIU52938.1
Candidatus Scatocola faecipullorum
extension Prok-SAA Imm-SAA Imm-SAA
MBR2137103.1
Alphaproteobacteria bacterium
extension Prok-SAA
MBQ7633917.1
Alphaproteobacteria bacterium
extension Prok-SAA
MBQ9034887.1
Alphaproteobacteria bacterium
extension Prok-SAA Imm-SAA
MBR2034018.1
Alphaproteobacteria bacterium
extension Prok-SAA
MBE6453012.1
Alphaproteobacteria bacterium
extension Prok-SAA Imm-SAA Imm-SAA
ADQ82132.1
Riemerella anatipestifer ATCC 11845 = DSM 15868
extension Prok-SAA alpha-helix 2-hairpin
MBR3675759.1
Alphaproteobacteria bacterium
extension Prok-SAA Imm-SAA
MBQ8870697.1
Alphaproteobacteria bacterium
extension Prok-SAA
MBQ8464611.1
Alphaproteobacteria bacterium
extension Prok-SAA
KJR40726.1
Candidatus Magnetoovum chiemensis
extension Prok-SAA SAA-imm-2
CRH04299.1
extension Prok-SAA
CRH04305.1
extension Prok-SAA
MBR1601519.1
Alphaproteobacteria bacterium
helical-extension Prok-SAA
MBP5215000.1
Alphaproteobacteria bacterium
helical-extension Prok-SAA Imm-SAA Imm-SAA
MBP5399537.1
Alphaproteobacteria bacterium
helical-extension Prok-SAA
OLA82353.1
Azospirillum sp. 51_20
helical-extension Prok-SAA
MBP5399284.1
Alphaproteobacteria bacterium
helical-extension Prok-SAA 2C-tail
MBO7244518.1
Alphaproteobacteria bacterium
helical-extension Prok-SAA 2C-tail
MBQ9089591.1
Alphaproteobacteria bacterium
helical-extension Prok-SAA Imm-SAA Imm-SAA
MBE6453833.1
Alphaproteobacteria bacterium
helical-extension Prok-SAA
MBE6455226.1
Alphaproteobacteria bacterium
helical-extension Prok-SAA
MBE6454550.1
Alphaproteobacteria bacterium
helical-extension Prok-SAA Imm-SAA
MBQ3117545.1
Alphaproteobacteria bacterium
disordered Prok-SAA
MBQ8630633.1
Alphaproteobacteria bacterium
disordered Prok-SAA
MBO5038306.1
Alphaproteobacteria bacterium
Prok-SAA Imm-SAA
HBO50156.1
Alphaproteobacteria bacterium
disordered Prok-SAA 3TM
QOJ21924.1
Gammaproteobacteria bacterium
RHS-repeats disordered Prok-SAA
MBR2298983.1
Alphaproteobacteria bacterium
disordered Prok-SAA
MBR1601661.1
Alphaproteobacteria bacterium
disordered Prok-SAA 2C-tail Imm-SAA
GBF23066.1
Candidatus Gastranaerophilus sp. (ex Termes propinquus)
disordered Prok-SAA

Accession	Organism	Domain architecture
RZK39948.1	*Pedobacter sp.*	disordered, Prok-SAA, alpha-helix, 2-hairpin
MBR1649114.1	*Alphaproteobacteria bacterium*	Prok-SAA, Imm-SAA
MBR1649032.1	*Alphaproteobacteria bacterium*	Prok-SAA
CDB53504.1	*Azospirillum sp. CAG:239*	Prok-SAA, Imm-SAA
UIY30982.1	*Neorhizobium galegae*	RHS-repeats, Prok-SAA
MBO7257966.1	*Alphaproteobacteria bacterium*	Prok-SAA, Imm-SAA
MBR1605734.1	*Alphaproteobacteria bacterium*	Prok-SAA
MBP3545783.1		Prok-SAA, Imm-SAA
MBJ6610612.1	*Candidatus Thiothrix moscowensis*	Prok-SAA, Imm-SAA
OLA80983.1	*Azospirillum sp. 51_20*	Prok-SAA
MBQ8784989.1	*Alphaproteobacteria bacterium*	Prok-SAA
MBF0246770.1	*Alphaproteobacteria bacterium*	Prok-SAA
MBQ8464927.1	*Alphaproteobacteria bacterium*	Prok-SAA, Imm-SAA
MBQ8869965.1	*Alphaproteobacteria bacterium*	Prok-SAA, Imm-SAA
MBR4106372.1	*Alphaproteobacteria bacterium*	Prok-SAA, Imm-SAA
MBR1600814.1	*Alphaproteobacteria bacterium*	Prok-SAA, Imm-SAA
MBR1373509.1	*bacterium*	Prok-SAA, Imm-SAA
MBE6462763.1	*Alphaproteobacteria bacterium*	Prok-SAA, Imm-SAA
MBQ4472488.1	*Alphaproteobacteria bacterium*	Prok-SAA, Imm-SAA
MBS5833706.1	*Acetobacter sp.*	Prok-SAA, Imm-SAA
MBQ8751046.1	*Alphaproteobacteria bacterium*	Prok-SAA, Imm-SAA, Imm-SAA
MBO6281056.1	*Alphaproteobacteria bacterium*	Prok-SAA, 2C-tail
MBS5833711.1	*Acetobacter sp.*	Prok-SAA, Imm-SAA
MBS5833742.1	*Acetobacter sp.*	Prok-SAA, C-rich-tail, C-rich-tail
MBR6408927.1	*Alphaproteobacteria bacterium*	Prok-SAA, Imm-SAA, Imm-SAA
MBE6455229.1	*Alphaproteobacteria bacterium*	Prok-SAA, 2C-tail
MBQ6854347.1	*Alphaproteobacteria bacterium*	Prok-SAA, 2C-tail
MBQ8660867.1	*Alphaproteobacteria bacterium*	disordered, Prok-SAA, ?, Imm-SAA, Imm-SAA

####;fusion to broken-hairpin

Accession	Organism	Domain architecture
MBP3821680.1	*bacterium*	broken-hairpin, Prok-SAA, Imm-SAA

####;fusion to VWD domain

Accession	Organism	Domain architecture
UCC83773.1	*Gemmatimonadota bacterium*	MPTase, Prok-SAA, VWD, IG, IG
WP_097067108.1	*Nitrosovibrio sp. Nv4*	MPTase, Prok-SAA, VWD, IG, IG
RLB42500.1	*Deltaproteobacteria bacterium*	MPTase, Prok-SAA, VWD, IG, IG
MBW1755671.1	*Deltaproteobacteria bacterium*	MPTase, Prok-SAA, VWD, IG, IG
MCA9531475.1	*Myxococcales bacterium*	MPTase, Prok-SAA, VWD, IG, IG

####;standalone versions

Accession	Organism	Domain architecture
MBF0320176.1	*Nitrospirota bacterium*	Prok-SAA
KWT78410.1	*Candidatus Magnetominusculus xianensis*	Prok-SAA
MBF0553095.1	*Nitrospirota bacterium*	Prok-SAA
MBF0516514.1	*Nitrospirota bacterium*	Prok-SAA
MBR6674875.1	*Alphaproteobacteria bacterium*	Prok-SAA
MBD3759966.1	*Rhizorhabdus sp.*	Prok-SAA
MBO5284324.1	*Alphaproteobacteria bacterium*	Prok-SAA, disordered
MBQ7632800.1	*Alphaproteobacteria bacterium*	Prok-SAA
MBQ9149488.1	*bacterium*	coiled-coil, Prok-SAA, coiled-coil, Prok-SAA
DAB04142.1	*Candidatus Gastranaerophilales bacterium HUM_10*	coiled-coil, Prok-SAA, 3TM
MBQ9149487.1	*bacterium*	coiled-coil, Prok-SAA, coiled-coil, Prok-SAA
OLA95000.1	*Candidatus Melainabacteria bacterium LEY3_CP_29_8*	coiled-coil, Prok-SAA
MBQ8870602.1	*Alphaproteobacteria bacterium*	Prok-SAA
MPT36158.1	*Flavobacterium sp.*	Prok-SAA, alpha-helix, 2-hairpin
PIY60266.1	*Candidatus Woesearchaeota archaeon CG_4_10_14_0_8_um_filter_47_5*	Prok-SAA
MBO6088321.1	*bacterium*	Prok-SAA
MBP3545598.1	*Alphaproteobacteria bacterium*	Prok-SAA
MBQ2844688.1	*Alphaproteobacteria bacterium*	Prok-SAA
MBP5399724.1	*Alphaproteobacteria bacterium*	Prok-SAA
MBE6462287.1	*Alphaproteobacteria bacterium*	Prok-SAA
MBO7555782.1	*Alphaproteobacteria bacterium*	Prok-SAA
MBE6462418.1	*Alphaproteobacteria bacterium*	Prok-SAA
MBR6409295.1	*Alphaproteobacteria bacterium*	Prok-SAA
MBR4931832.1	*Alphaproteobacteria bacterium*	Prok-SAA
OLA80980.1	*Azospirillum sp. 51_20*	Prok-SAA
MBF0487980.1	*Nitrospirota bacterium*	Prok-SAA
MBP3545615.1	*Alphaproteobacteria bacterium*	Prok-SAA
MBS5802726.1	*Brachyspira sp.*	Prok-SAA
MBQ3034427.1	*Alphaproteobacteria bacterium*	Prok-SAA
HIV07674.1	*Candidatus Scatocola faecigallinarum*	Prok-SAA
MBR5130871.1	*Alphaproteobacteria bacterium*	Prok-SAA
PYQ29061.1	*Acidobacteriota bacterium*	Prok-SAA
PIP93034.1	*Bdellovibrio sp. CG22_combo_CG10-13_8_21_14_all_39_27*	Prok-SAA, 2C-Chelix
PIP91732.1	*Bdellovibrionales bacterium CG22_combo_CG10-13_8_21_14_all_38_13*	Prok-SAA, 2C-Chelix
MBT3586022.1	*Halobacteriovoraceae bacterium*	Prok-SAA, 2C-Chelix
MBR4126959.1	*Alphaproteobacteria bacterium*	Prok-SAA
MBF0459739.1	*Nitrospirota bacterium*	Prok-SAA
MBQ3642739.1	*bacterium*	SAA(frag)
MBC7168875.1	*Phenylobacterium sp.*	SAA(frag)
MBH0178856.1	*Nitrospira sp.*	SAA(frag)

################;Prok-TelC; ###########;Polymorphic toxin and related systems

Accession	Organism	Domain architecture
WP_228064066.1	*Streptococcus acidominimus*	RHS-repeats, Prok-TelC
WP_135182613.1	*Streptococcus cuniculi*	RHS-repeats, Prok-TelC, TipC-like-imm
WP_153225049.1		RHS-repeats, Prok-TelC, TipC-like-imm
WP_142997553.1	*Streptococcus minor*	RHS-repeats, Prok-TelC, TipC-like-imm
WP_136581339.1	*Streptococcus suis*	DUF6531, RHS-repeats, Prok-TelC, TipC-like-imm, ?, TipC-like-imm
PKM94319.1	*Firmicutes bacterium HGW-Firmicutes-1*	Peptidase-S8, DUF6531, RHS-repeats, Prok-TelC, BamE-like?
WP_207237989.1		RHS-repeats, Prok-TelC, BamE-like?
MBN2134727.1	*Acidobacteriota bacterium*	RHS-repeats, Prok-TelC

#;WXG/LXG-containing architectures

Accession	Organism	Domain architecture
MBD5537845.1	*Lachnospiraceae bacterium*	WXG, Prok-TelC
WP_061599910.1	*Streptococcus gordonii*	WXG, Prok-TelC
WP_051933811.1	*Carnobacterium gallinarum DSM 4847*	WXG, Prok-TelC, TipC-like-imm
WP_209525075.1	*Vagococcus allomyrinae*	WXG, Prok-TelC, TipC-like-imm, TipC-like-imm, TipC-like-imm, TipC-like-imm
WP_090154464.1	*Pseudobutyrivibrio sp. AR14*	WXG, Prok-TelC
WP_088838733.1	*Listeria sp. ILCC792*	WXG, ?, WXG, Prok-TelC, TipC-like-imm
WP_009731655.1	*Streptococcus sp. F0442*	EsxAB-like, long-hairpin, WXG, Prok-TelC, TipC-like-imm

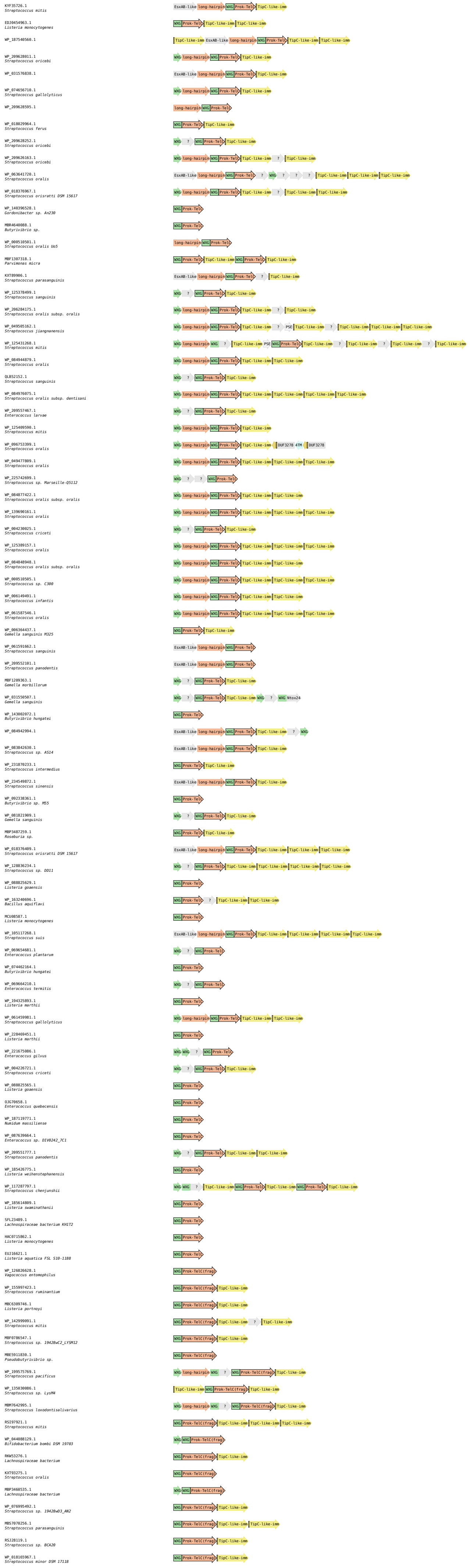
KYF35726.1
Streptococcus mitis
EDJ0454963.1
Listeria monocytogenes
WP_187540560.1
WP_209628011.1
Streptococcus oricebi
WP_031576838.1
WP_074656710.1
Streptococcus gallolyticus
WP_209628595.1
WP_018029964.1
Streptococcus ferus
WP_209628252.1
Streptococcus oricebi
WP_209626163.1
Streptococcus oricebi
WP_063641720.1
Streptococcus oralis
WP_018376967.1
Streptococcus orisratti DSM 15617
WP_140396528.1
Gordonibacter sp. An230
MBR4640088.1
Butyrivibrio sp.
WP_000510501.1
Streptococcus oralis Uo5
MBF1307318.1
Parvimonas micra
KXT89906.1
Streptococcus parasanguinis
WP_125378499.1
Streptococcus sanguinis
WP_206284175.1
Streptococcus oralis subsp. oralis
WP_049505162.1
Streptococcus jiangnanensis
WP_125431268.1
Streptococcus mitis
WP_084944879.1
Streptococcus oralis
QLB52152.1
Streptococcus sanguinis
WP_084976075.1
Streptococcus oralis subsp. dentisani
WP_209557467.1
Enterococcus larvae
WP_125409590.1
Streptococcus mitis
WP_096753399.1
Streptococcus oralis
WP_049477809.1
Streptococcus oralis
WP_225742699.1
Streptococcus sp. Marseille-Q5112
WP_084877422.1
Streptococcus oralis subsp. oralis
WP_139690161.1
Streptococcus oralis
WP_004230025.1
Streptococcus criceti
WP_125389157.1
Streptococcus oralis
WP_084848948.1
Streptococcus oralis subsp. oralis
WP_000510505.1
Streptococcus sp. C300
WP_006149491.1
Streptococcus infantis
WP_061587546.1
Streptococcus oralis
WP_006364437.1
Gemella sanguinis M325
WP_061591662.1
Streptococcus sanguinis
WP_209552101.1
Streptococcus panodentis
MBF1209363.1
Gemella morbillorum
WP_031550507.1
Gemella sanguinis
WP_143002072.1
Butyrivibrio hungatei
WP_084942994.1
WP_083842630.1
Streptococcus sp. AS14
WP_231870233.1
Streptococcus intermedius
WP_234549872.1
Streptococcus sinensis
WP_092338361.1
Butyrivibrio sp. M55
WP_081821909.1
Gemella sanguinis
MBP3487259.1
Roseburia sp.
WP_018376409.1
Streptococcus orisratti DSM 15617
WP_128836234.1
Streptococcus sp. DD11
WP_088825629.1
Listeria goaensis
WP_163240696.1
Bacillus aquiflavi
MCU08587.1
Listeria monocytogenes
WP_105117268.1
Streptococcus suis
WP_069654681.1
Enterococcus plantarum
WP_074462164.1
Butyrivibrio hungatei
WP_069664210.1
Enterococcus termitis
WP_194325893.1
Listeria marthii
WP_061459981.1
Streptococcus gallolyticus
WP_228469451.1
Listeria marthii
WP_221675086.1
Enterococcus gilvus
WP_004226721.1
Streptococcus criceti
WP_088825565.1
Listeria goaensis
OJG70658.1
Enterococcus quebecensis
WP_187119771.1
Numidum massiliense
WP_087639664.1
Enterococcus sp. DIV0242_7C1
WP_209551777.1
Streptococcus panodentis
WP_185426775.1
Listeria weihenstephanensis
WP_117287797.1
Streptococcus chenjunshii
WP_185614809.1
Listeria swaminathanii
SFL23489.1
Lachnospiraceae bacterium KH1T2
HAC0715862.1
Listeria monocytogenes
EUJ16621.1
Listeria aquatica FSL S10-1188
WP_126826628.1
Vagococcus entomophilus
WP_155997423.1
Streptococcus ruminantium
MBC6309746.1
Listeria portnoyi
WP_142999091.1
Streptococcus mitis
MBF0786547.1
Streptococcus sp. 19428wC2_LYSM12
MBE5911830.1
Pseudobutyrivibrio sp.
WP_199575769.1
Streptococcus pacificus
WP_135030086.1
Streptococcus sp. LysM4
MBM7642995.1
Streptococcus loxodontisalivarius
RSI97921.1
Streptococcus mitis
WP_044088129.1
Bifidobacterium bombi DSM 19703
RKW53276.1
Lachnospiraceae bacterium
KXT93275.1
Streptococcus oralis
MBP3460535.1
Lachnospiraceae bacterium
WP_076995492.1
Streptococcus sp. 19428wD3_AN2
MBS7070256.1
Streptococcus parasanguinis
RSJ28119.1
Streptococcus sp. BCA20
WP_018165967.1
Streptococcus minor DSM 17118

WP_050950871.1
Limosilactobacillus gastricus PS3
WXG Prok-TelC(frag)

WP_156009879.1
Streptococcus ruminantium
WXG Prok-TelC(frag) TipC-like-imm

WP_044754960.1
Streptococcus suis
WXG Prok-TelC(frag) TipC-like-imm ? TipC-like-imm

HJC03411.1
Candidatus Ligilactobacillus avistercoris
WXG Prok-TelC

WP_094785333.1
Pseudolactococcus reticulitermitis
WXG Prok-TelC(frag)

WP_185380136.1
Listeria booriae
WXG ? ? WXG Prok-TelC

WP_169298144.1
Clostridium muellerianum
WXG hel-hairpin Prok-TelC

HCW52245.1
Clostridium sp.
WXG ? WXG hel-hairpin Prok-TelC

WP_021725990.1
Olsenella profusa F0195
WXG Prok-TelC

WP_156097156.1
Bifidobacterium bombi DSM 19703
WXG Prok-TelC

HJD08797.1
Candidatus Ligilactobacillus faecavium
WXG Prok-TelC

####;ZU5+vWA core

MBO4865976.1
Ruminococcus sp.
b-helix TSP-repeats ZU5 vWA Flg-flap TSP-repeats Prok-TelC Zu5-vWA-imm

WP_024858870.1
Ruminococcus albus AD2013
b-helix TSP-repeats ZU5 vWA Flg-flap TSP-repeats Prok-TelC Zu5-vWA-imm

SCJ40748.1
uncultured Ruminococcus sp.
b-helix TSP-repeats ZU5 vWA Flg-flap TSP-repeats Prok-TelC Zu5-vWA-imm

MBQ1326801.1
Eubacterium sp.
CBM CBM CBM TSP-repeats ZU5 vWA Flg-flap TSP-repeats Prok-TelC Zu5-vWA-imm

CDE53385.1
Roseburia sp. CAG:303
CBM CBM CBM TSP-repeats ZU5 vWA Flg-flap TSP-repeats Prok-TelC Zu5-vWA-imm

OKZ61282.1
Clostridium sp. 42_12
CBM CBM CBM TSP-repeats ZU5 vWA Flg-flap TSP-repeats Prok-TelC

MBR6580339.1
Ruminococcus sp.
CBM CBM CBM TSP-repeats ZU5 vWA Flg-flap TSP-repeats Prok-TelC

SKC52739.1
[Eubacterium] yurii
CBM TSP-repeats ZU5 vWA Flg-flap TSP-repeats Prok-TelC Zu5-vWA-imm

MBQ6638652.1
Lachnospiraceae bacterium
CBM-repeats TSP-repeats ZU5 vWA Flg-flap TSP-repeats Prok-TelC Zu5-vWA-imm Zu5-vWA-imm

MBQ6638962.1
Lachnospiraceae bacterium
CBM-repeats TSP-repeats ZU5 vWA Flg-flap TSP-repeats Prok-TelC Zu5-vWA-imm

HCM23678.1
Oscillospiraceae bacterium
CBM-repeats TSP-repeats ZU5 vWA Flg-flap TSP-repeats Prok-TelC Zu5-vWA-imm Zu5-vWA-imm

WP_094691255.1
Pseudoscardovia suis
CBM-repeats TSP-repeats ZU5 vWA Flg-flap TSP-repeats Prok-TelC

WP_158216109.1
Pseudoscardovia suis
CBM-repeats TSP-repeats ZU5 vWA Flg-flap TSP-repeats Prok-TelC

WP_022234420.1
uncultured Ruminococcus sp.
Prok-TelC(frag) Zu5-vWA-imm

####;RICIN-trefoil domain-containing architectures

MBR6682345.1
Clostridia bacterium
beta-propeller RICIN-trefoil RICIN-trefoil RICIN-trefoil Prok-TelC

MBE6748308.1
Oscillospiraceae bacterium
beta-propeller RICIN-trefoil RICIN-trefoil Prok-TelC

MBQ3550484.1
Clostridia bacterium
beta-propeller RICIN-trefoil RICIN-trefoil Prok-TelC

MBR6411220.1
Clostridia bacterium
beta-propeller RICIN-trefoil RICIN-trefoil Prok-TelC

MBE6694225.1
Oscillospiraceae bacterium
RHS-repeats RICIN-trefoil RICIN-trefoil IG-like IG-like Prok-TelC

MCD7776809.1
Bacillota bacterium
RICIN-trefoil IG-like Prok-TelC

MCD7943965.1
Clostridia bacterium
RICIN-trefoil IG-like Prok-TelC

MBR5949542.1
Clostridia bacterium
RICIN-trefoil IG-like Prok-TelC

MBE6947797.1
Oscillospiraceae bacterium
beta-propeller RICIN-trefoil IG-like Prok-TelC

MBQ4037036.1
Clostridia bacterium
beta-propeller RICIN-trefoil IG-like Prok-TelC

####;GbpC beta-sandwich and MucBP IG-like domain-containing architectures

WP_204171091.1
Mammaliicoccus sciuri
linker GbpC MucBP_IG MucBP_IG linker Prok-TelC charge-helical-bundle TipC-like-imm

WP_095348358.1
Lactococcus lactis subsp. lactis
linker GbpC MucBP_IG MucBP_IG linker Prok-TelC charge-helical-bundle TipC-like-imm TipC-like-imm

WP_104142190.1
Lactococcus lactis
linker GbpC MucBP_IG MucBP_IG linker Prok-TelC charge-helical-bundle TipC-like-imm TipC-like-imm ? TipC-like-imm TipC-like-imm

WP_180960511.1
Gemella sanguinis
linker GbpC MucBP_IG MucBP_IG linker Prok-TelC charge-helical-bundle TipC-like-imm

WP_216278079.1
Collinsella sp. zg1085
linker GbpC MucBP_IG MucBP_IG linker Prok-TelC charge-helical-bundle TipC-like-imm TipC-like-imm TipC-like-imm TipC-like-imm

WP_135125370.1
Granulicatella sp. 19428wC4_WM01
linker GbpC MucBP_IG MucBP_IG linker Prok-TelC charge-helical-bundle TipC-like-imm

WP_075361645.1
Boudabousia marimammalium
linker GbpC MucBP_IG MucBP_IG linker Prok-TelC charge-helical-bundle TipC-like-imm TipC-like-imm TipC-like-imm TipC-like-imm TipC-like-imm TipC-like-imm

MCB5733571.1
Streptococcus sp. MSK15_114
linker GbpC MucBP_IG MucBP_IG linker Prok-TelC charge-helical-bundle TipC-like-imm

AVI59457.1
Streptococcus equinus
linker GbpC MucBP_IG MucBP_IG linker Prok-TelC charge-helical-bundle TipC-like-imm

SFE46632.1
Peptostreptococcaceae bacterium pGA-8
linker GbpC MucBP_IG MucBP_IG linker Prok-TelC charge-helical-bundle TipC-like-imm TipC-like-imm TipC-like-imm

WP_071129624.1
Varibaculum massiliense
linker GbpC MucBP_IG MucBP_IG linker Prok-TelC charge-helical-bundle TipC-like-imm TipC-like-imm TipC-like-imm TipC-like-imm

WP_056995285.1
Limosilactobacillus equigenerosi
linker GbpC MucBP_IG MucBP_IG linker Prok-TelC charge-helical-bundle TipC-like-imm

MBF1359753.1
Mogibacterium diversum
linker GbpC MucBP_IG MucBP_IG linker Prok-TelC charge-helical-bundle TipC-like-imm TipC-like-imm TipC-like-imm TipC-like-imm

MBB1548372.1
Mogibacterium sp.
linker GbpC MucBP_IG MucBP_IG linker Prok-TelC charge-helical-bundle

MBS5865389.1
Coriobacteriaceae bacterium
linker GbpC MucBP_IG MucBP_IG linker Prok-TelC charge-helical-bundle TipC-like-imm TipC-like-imm TipC-like-imm

WP_135157208.1
GbpC MucBP-IG MucBP-IG disordered Prok-TelC TipC-like-imm TipC-like-imm

WP_182587509.1
Limosilactobacillus balticus
GbpC MucBP-IG MucBP-IG MucBP-IG MucBP-IG Prok-TelC TipC-like-imm TipC-like-imm ? TipC-like-imm

KRL92605.1
Limosilactobacillus equigenerosi DSM 18793 = JCM 14505
MucBP_IG MucBP_IG linker Prok-TelC charge-helical-bundle TipC-like-imm TipC-like-imm TipC-like-imm

WP_228098439.1
MucBP_IG linker Prok-TelC charge-helical-bundle TipC-like-imm TipC-like-imm TipC-like-imm

HJF21761.1
Mammaliicoccus lentus
TipC-like-imm linker GbpC MucBP_IG MucBP_IG linker Prok-TelC charge-helical-bundle Prok-TelC TipC-like-imm TipC-like-imm

WP_205040894.1
Streptococcus suis
helical-linker GbpC linker Prok-TelC charge-helical-bundle TipC-like-imm TipC-like-imm TipC-like-imm

WP_146461824.1
Streptococcus sp. sy004
helical-linker GbpC linker Prok-TelC charge-helical-bundle

WP_160544910.1
helical-linker GbpC linker Prok-TelC charge-helical-bundle TipC-like-imm TipC-like-imm

WP_074626982.1
Streptococcus gallolyticus
helical-linker GbpC linker Prok-TelC charge-helical-bundle TipC-like-imm

MBF0698864.1
Streptococcus danieliae
helical-linker GbpC linker Prok-TelC charge-helical-bundle

WP_179923816.1
Streptococcus danieliae
helical-linker GbpC linker Prok-TelC charge-helical-bundle TipC-like-imm TipC-like-imm

WP_074485954.1
Streptococcus henryi
helical-linker GbpC linker Prok-TelC charge-helical-bundle TipC-like-imm TipC-like-imm TipC-like-imm

WP_003087142.1
Streptococcus ratti
helical-linker GbpC linker Prok-TelC charge-helical-bundle TipC-like-imm TipC-like-imm

WP_196967568.1
Streptococcus sp. NLN64
GbpC linker Prok-TelC charge-helical-bundle TipC-like-imm ? TipC-like-imm

WP_160332982.1
Streptococcus danieliae
Prok-TelC charge-helical-bundle TipC-like-imm

MBF1354798.1
Mogibacterium diversum
Prok-TelC charge-helical-bundle TipC-like-imm TipC-like-imm TipC-like-imm TipC-like-imm

WP_202913563.1
Streptococcus sp. S784/96/1
Prok-TelC charge-helical-bundle

WP_094233489.1
Mogibacterium pumilum
Prok-TelC charge-helical-bundle TipC-like-imm TipC-like-imm TipC-like-imm TipC-like-imm

MBS5319494.1
Gemella haemolysans
Prok-TelC charge-helical-bundle TipC-like-imm

MBQ9690682.1
Eggerthellaceae bacterium
Prok-TelC charge-helical-bundle TipC-like-imm

####;MPTase DUF4157 fusions

WP_079537109.1
Bradyrhizobium lablabi
DUF4157_MPTase Prok-TelC

WP_074238721.1
Chitinophaga niabensis
DUF4157_MPTase Prok-TelC

TLU54685.1
Chlorobium sp.
DUF4157_MPTase Prok-TelC

MBP8805133.1
Kofleriaceae bacterium
DUF4157_MPTase Prok-TelC

WP_203907874.1
Rhizocola hellebori
MPTase Prok-TelC

WP_203373918.1
Paractinoplanes ovalisporus
MPTase Prok-TelC ??

MCB9230046.1
Bacteroidia bacterium
MPTase Prok-TelC

NUN13406.1
Myxococcales bacterium
MPTase Prok-TelC

WP_233104110.1
Haliangium ochraceum DSM 14365
MPTase ?? Prok-TelC

WP_218142985.1
Nitrosomonas marina
ZnR MPTase OmpA-like-beta-barrel Prok-TelC

NIM95755.1
Anaerolineales bacterium
MPTase OmpA-like-beta-barrel Prok-TelC

MCB9232021.1
Bacteroidia bacterium
MPTase beta-barrel Prok-TelC

CAG0963931.1
Methanosarcinales archaeon
MPTase OmpA-like-beta-barrel Prok-TelC

MBK7954298.1
Candidatus Accumulibacter affinis
MPTase OmpA-like-beta-barrel Prok-TelC

WP_197995812.1
Gimesia algae
MPTase Prok-TelC

MBV9169443.1
Chloroflexota bacterium
MPTase Prok-TelC

TMD12013.1
Chloroflexota bacterium
OmpA-like-beta-barrel Prok-TelC

#;these Prok-TelC sequences cluster with DUF4157-versions based on sequence, but feature distinct (or no) fusions

MBE7519185.1
Thermoflexaceae bacterium
Prok-TelC

MBP9086829.1
Kofleriaceae bacterium
Prok-TelC

WP_204760277.1
Lentzea nigeriaca
Prok-TelC

WP_223403235.1
Occultella gossypii
Prok-TelC

WP_025142905.1
Pedobacter jeongneungensis
Prok-TelC

WP_097554513.1
Flavobacterium branchiophilum
Prok-TelC

WP_201421845.1
Myxococcus xanthus
Prok-TelC

WP_233587552.1
Corallococcus sp. CA049B
Prok-TelC

WP_116010277.1
Chryseobacterium elymi
Prok-TelC

WP_009401518.1
Capnocytophaga sp. oral taxon 338 str. F0234
Prok-TelC

WP_096585470.1
Tolypothrix tenuis PCC 7101
Prok-TelC

MBP1771601.1
Holophagaceae bacterium
Prok-TelC

WP_179813438.1
Allostreptomyces psammosilenae
Prok-TelC

MBN1306573.1
Chitinispirillaceae bacterium
Prok-TelC

WP_119652564.1
Capnocytophaga canis
Prok-TelC(frag) Prok-TelC

WP_002627747.1
Cystobacter fuscus DSM 2262
LysM Prok-TelC

WP_002613618.1
Stigmatella aurantiaca DW4/3-1
LysM Prok-TelC

WP_071899555.1
Cystobacter ferrugineus
PGBD LysM SH3 SH3 Prok-TelC

HBE17896.1
Cyanobacteria bacterium UBA11367
?? Prok-TelC

WP_232312723.1
Enterovibrio coralii
Prok-TelC

KFA87055.1
Archangium violaceum Cb vi76
SH3 Prok-TelC

NOQ74231.1
Crocinitomix sp.
?? Prok-TelC

WP_167736150.1
Nocardioides sp. 503
PGBD PGBD Prok-TelC

WP_017663710.1
Baaleninema simplex PCC 7105
Prok-TelC

WP_197060027.1
Sporocytophaga myxococcoides
Prok-TelC

WP_097107514.1
Nitrosomonas ureae
Prok-TelC

WP_183296548.1
Cellulomonas cellasea
Prok-TelC

####;TPM-like fusions

WP_150543541.1
Mesonia oceanica
TPM-like IG-like Prok-TelC

MCB9274968.1
Lewinellaceae bacterium
TPM-like TPM-like Prok-TelC TM-imm

MBK8748618.1
Candidatus Brachybacter algidus
TPM-like TPM-like Prok-TelC TM-imm

MCB0533343.1
Saprospiraceae bacterium
TPM-like TPM-like Prok-TelC

MBK6931957.1
Saprospirales bacterium
TPM-like TPM-like Prok-TelC TM-imm

MCB2090319.1
Alphaproteobacteria bacterium
TPM-like TPM-like Prok-TelC

MBK6901543.1
Saprospirales bacterium
Prok-TelC TM-imm

####;other diverse polymorphic-like system contexts; #;SpvB+BetaPropeller fusions

MBR6124050.1
Candidatus Saccharibacteria bacterium
SPVB Bps RHS-repeats Prok-TelC

#;CW-binding-beta-hairpin-repeat fusions

WP_012060545.1
Clostridium beijerinckii
Phage_GPD Phage_base_V DUF2345 CW-binding-beta-hairpin-repeats Prok-TelC BamE-like?

WP_077865328.1
Phage_GPD Phage_base_V DUF2345 CW-binding-beta-hairpin-repeats Prok-TelC

WP_117623903.1
Enterocloster bolteae
Prok-TelC CW-binding-beta-hairpin-repeats

WP_118710482.1
Enterocloster aldenensis
Prok-TelC CW-binding-beta-hairpin-repeats

#;SH3 repeats and Carboxypeptidase

MBQ3413842.1
Clostridia bacterium
SH3 SH3 SH3 SH3 SH3 Carboxypeptidase Prok-TelC

HCQ55030.1
Clostridiales bacterium
Phage_holin_4_1 SH3 SH3 SH3 SH3 SH3 Carboxypeptidase Prok-TelC

AVQ95882.1
Ethanoligenens harbinense YUAN-3
SH3 SH3 SH3 SH3 Carboxypeptidase Prok-TelC BamE-like? BamE-like?

WP_013485142.1
Ethanoligenens harbinense
BamE-like? BamE-like? ? BamE-like? BamE-like? Carboxypeptidase Prok-TelC BamE-like?

HBQ85135.1
Syntrophomonas sp.
SH3 SH3 SH3 SH3 Carboxypeptidase IG-like Prok-TelC

WP_200267842.1
Clostridium yunnanense
SH3 SH3 SH3 SH3 Carboxypeptidase SH3 PGBD Prok-TelC

WP_155642216.1
Clostridium perfringens
Carboxypeptidase SH3 Prok-TelC BamE-like?

EHR0219375.1
Clostridium perfringens
SH3 Prok-TelC BamE-like? BamE-like?

EGT3601847.1
Clostridium perfringens
SH3 Prok-TelC BamE-like? BamE-like?

WP_198615657.1
SH3 Prok-TelC BamE-like?

WP_196372130.1
SH3 Prok-TelC

#;LysM and/or Chitinase domain fusions

NQN65101.1
Streptococcus suis
LysM LysM LysM Chitinase Prok-TelC

#;PAAR fusions

WP_124065279.1
Clostridium sp. E02
PAAR Prok-TelC TipC-like-imm

WP_185959916.1
Criibacterium bergeronii
PAAR Prok-TelC TipC-like-imm

###########;Architecture typically combining Prok-TelC with putative peptidoglycan glycosylhydrolase TIM-barrel amd PGBP domains with some lineage-restricted gene associations. Actinobacterial association with the duoIG domain

WP_068212297.1
Rathayibacter tanaceti
duoIG PGBD PGBD Rv2525c_GlyHyd-like_TIM-barrel Prok-TelC

WP_104304578.1
Rathayibacter sp. AY2B3
duoIG PGBD PGBD Rv2525c_GlyHyd-like_TIM-barrel Prok-TelC

WP_104318888.1
Rathayibacter rathayi
duoIG PGBD PGBD Rv2525c_GlyHyd-like_TIM-barrel Prok-TelC

WP_104265562.1
Rathayibacter iranicus NCPPB 2253 = VKM Ac-1602
duoIG PGBD PGBD Rv2525c_GlyHyd-like_TIM-barrel Prok-TelC

WP_179389374.1
Psychromicrobium silvestre
duoIG PGBD PGBD Rv2525c_GlyHyd-like_TIM-barrel Prok-TelC

WP_006538431.1
Parafrankia sp. EUN1f
duoIG PGBD PGBD Rv2525c_GlyHyd-like_TIM-barrel Prok-TelC

WP_144761878.1
Curtobacterium sp. 9128
PGBD duoIG PGBD PGBD Rv2525c_GlyHyd-like_TIM-barrel Prok-TelC

WP_146847900.1
Cellulomonas terrae
PGBD duoIG PGBD PGBD Rv2525c_GlyHyd-like_TIM-barrel Prok-TelC

WP_209343972.1
Streptomyces montanisoli
PGBD PGBD Rv2525c_GlyHyd-like_TIM-barrel Prok-TelC duoIG

WP_090039896.1
Leifsonia sp. CL147
duoIG PGBD PGBD Rv2525c_GlyHyd-like_TIM-barrel Prok-TelC

GGM91888.1
Streptomyces fuscichromogenes
PGBD PGBD Rv2525c_GlyHyd-like_TIM-barrel Prok-TelC duoIG

WP_121687087.1
Mycetocola lacteus
duoIG PGBD PGBD Rv2525c_GlyHyd-like_TIM-barrel Prok-TelC

MBN1174476.1
Micromonosporaceae bacterium
PGBD PGBD Rv2525c_GlyHyd-like_TIM-barrel Prok-TelC(frag) duoIG

WP_109817566.1
Micromonospora acroterricola
PGBD PGBD Rv2525c_GlyHyd-like_TIM-barrel Prok-TelC duoIG

WP_155932730.1
Micrococcus luteus
duoIG PGBD PGBD Rv2525c_GlyHyd-like_TIM-barrel Prok-TelC

WP_220339548.1
Microbacterium ureisolvens
duoIG PGBD PGBD Rv2525c_GlyHyd-like_TIM-barrel Prok-TelC

WP_156458810.1
Microbacterium sp. Leaf179
duoIG PGBD PGBD Rv2525c_GlyHyd-like_TIM-barrel Prok-TelC ? PGBD PGBD Rv2525c_GlyHyd-like_TIM-barrel Prok-TelC

WP_120263064.1
Microbacterium sp. AG238
duoIG PGBD PGBD Rv2525c_GlyHyd-like_TIM-barrel Prok-TelC

MBN9154555.1
Microbacterium sp.
duoIG PGBD PGBD Rv2525c_GlyHyd-like_TIM-barrel Prok-TelC

WP_135067363.1
Microbacterium wangchenii
duoIG PGBD PGBD Rv2525c_GlyHyd-like_TIM-barrel Prok-TelC

WP_214157593.1
Kineosporia corallincola
PGBD PGBD Rv2525c_GlyHyd-like_TIM-barrel Prok-TelC duoIG

WP_167627329.1
Frigoribacterium faeni
duoIG PGBD PGBD Rv2525c_GlyHyd-like_TIM-barrel Prok-TelC

WP_194629936.1
Curtobacterium sp. VKM Ac-2865
duoIG PGBD PGBD Rv2525c_GlyHyd-like_TIM-barrel Prok-TelC

WP_058740770.1
Curtobacterium citreum
duoIG PGBD PGBD Rv2525c_GlyHyd-like_TIM-barrel Prok-TelC

WP_194665049.1
Clavibacter sp. VKM Ac-2872
duoIG PGBD PGBD Rv2525c_GlyHyd-like_TIM-barrel Prok-TelC

WP_122148954.1
Cellulomonas triticagri
duoIG PGBD PGBD Rv2525c_GlyHyd-like_TIM-barrel Prok-TelC

WP_203675048.1
Cellulomonas phragmiteti
duoIG PGBD PGBD Rv2525c_GlyHyd-like_TIM-barrel Prok-TelC

WP_203750464.1
Cellulomonas chitinilytica
duoIG PGBD PGBD Rv2525c_GlyHyd-like_TIM-barrel Prok-TelC

WP_183295238.1
Cellulomonas cellasea
duoIG PGBD PGBD Rv2525c_GlyHyd-like_TIM-barrel Prok-TelC

WP_139416608.1
Agromyces laixinhei
duoIG PGBD PGBD Rv2525c_GlyHyd-like_TIM-barrel Prok-TelC

WP_086990545.1
Agrococcus casei LMG 22410
duoIG PGBD PGBD Rv2525c_GlyHyd-like_TIM-barrel Prok-TelC

MBN9633119.1
Actinomycetota bacterium
duoIG PGBD PGBD Rv2525c_GlyHyd-like_TIM-barrel Prok-TelC

WP_053585617.1
Actinomyces sp. oral taxon 414
PGBD ? PGBD PGBD Rv2525c_GlyHyd-like_TIM-barrel Prok-TelC ? duoIG

WP_147679966.1
Actinomyces ruminicola
PGBD ? PGBD PGBD Rv2525c_GlyHyd-like_TIM-barrel Prok-TelC ? duoIG

WP_203786530.1
Paractinoplanes rishiriensis
PGBD PGBD Rv2525c_GlyHyd-like_TIM-barrel Prok-TelC duoIG

WP_052721187.1
Actinoplanes rectilineatus
PGBD PGBD Rv2525c_GlyHyd-like_TIM-barrel Prok-TelC duoIG

####;Actinobacterial association with the 3TM-CCDN and VanY-like peptidase domains

WP_199038348.1	*Glycomyces salinus*	PGBD PGBD Rv2525c_GlyHyd-like_TIM-barrel Prok-TelC VanY-like ? ? VanY-like Prok-TelC Rv2525c_GlyHyd-like_TIM-barrel PGBD PGBD PGBD
WP_043381878.1	*Streptomyces mutabilis*	PGBD PGBD Rv2525c_GlyHyd-like_TIM-barrel Prok-TelC VanY-like
NRQ39970.1	*Nonomuraea antri*	PGBD PGBD Rv2525c_GlyHyd-like_TIM-barrel Prok-TelC 3TM-CCDN VanY-like
WP_206324481.1	*Streptomyces sp. R302*	PGBD PGBD PGBD Rv2525c_GlyHyd-like_TIM-barrel Prok-TelC VanY-like
WP_106188440.1	*Umezawaea tangerina*	PGBD PGBD Rv2525c_GlyHyd-like_TIM-barrel Prok-TelC 3TM-CCDN VanY-like
WP_043669800.1	*Streptomyces xylophagus*	PGBD PGBD Rv2525c_GlyHyd-like_TIM-barrel Prok-TelC ? VanY-like
WP_053167123.1	*Streptomyces sp. NPDC016734*	PGBD PGBD Rv2525c_GlyHyd-like_TIM-barrel Prok-TelC 3TM-CCDN VanY-like
WP_098898232.1	*Streptomyces sp. st77*	PGBD PGBD Rv2525c_GlyHyd-like_TIM-barrel Prok-TelC VanY-like PGBD PGBD
WP_218956619.1	*Streptomyces sp. UH6*	PGBD PGBD PGBD PGBD Rv2525c_GlyHyd-like_TIM-barrel Prok-TelC
WP_019329720.1	*Streptomyces rochei*	PGBD PGBD Rv2525c_GlyHyd-like_TIM-barrel Prok-TelC ? VanY-like
NEA66460.1	*Streptomyces sp. SID12488*	PGBD PGBD Rv2525c_GlyHyd-like_TIM-barrel Prok-TelC 3TM-CCDN VanY-like
WP_073215091.1	*Streptomyces sp. NPDC090126*	PGBD PGBD Rv2525c_GlyHyd-like_TIM-barrel Prok-TelC VanY-like PGBD PGBD
WP_077058292.1		PGBD PGBD Rv2525c_GlyHyd-like_TIM-barrel Prok-TelC ? VanY-like
WP_214971030.1	*Streptomyces sp. ISL-100*	PGBD PGBD Rv2525c_GlyHyd-like_TIM-barrel Prok-TelC 3TM-CCDN VanY-like
WP_026169521.1	*Streptomyces sp. SID8375*	PGBD PGBD Rv2525c_GlyHyd-like_TIM-barrel Prok-TelC 3TM-CCDN VanY-like
WP_148645871.1	*Streptomyces sp. CB01881*	PGBD PGBD Rv2525c_GlyHyd-like_TIM-barrel Prok-TelC 3TM-CCDN VanY-like
NKY13791.1	*Streptomyces somaliensis DSM 40738*	PGBD PGBD Rv2525c_GlyHyd-like_TIM-barrel Prok-TelC 3TM-CCDN
WP_203697979.1	*Streptomyces rubrogriseus*	PGBD PGBD Rv2525c_GlyHyd-like_TIM-barrel Prok-TelC VanY-like
WP_190089869.1	*Streptomyces melanogenes*	PGBD PGBD Rv2525c_GlyHyd-like_TIM-barrel Prok-TelC VanY-like
WP_044570649.1	*Streptomyces iranensis*	PGBD PGBD Rv2525c_GlyHyd-like_TIM-barrel Prok-TelC 3TM-CCDN VanY-like
WP_127177053.1		PGBD PGBD Rv2525c_GlyHyd-like_TIM-barrel Prok-TelC 3TM-CCDN VanY-like
WP_079084776.1	*Streptomyces dysideae*	PGBD PGBD Rv2525c_GlyHyd-like_TIM-barrel Prok-TelC VanY-like
WP_159470564.1	*Streptomyces caniferus*	PGBD PGBD Rv2525c_GlyHyd-like_TIM-barrel Prok-TelC 3TM-CCDN VanY-like
WP_182660134.1	*Streptomyces calidiresistens*	PGBD PGBD Rv2525c_GlyHyd-like_TIM-barrel Prok-TelC(frag) 3TM-CCDN
RJQ78450.1	*Pseudonocardiaceae bacterium YIM PH 21723*	PGBD PGBD Rv2525c_GlyHyd-like_TIM-barrel Prok-TelC 3TM-CCDN
WP_189170668.1	*Pilimelia anulata*	PGBD PGBD Rv2525c_GlyHyd-like_TIM-barrel Prok-TelC VanY-like
WP_217680900.1	*Kitasatospora sp. SUK 42*	PGBD PGBD Rv2525c_GlyHyd-like_TIM-barrel Prok-TelC 3TM-CCDN VanY-like
WP_233727587.1	*Kibdelosporangium philippinense*	PGBD PGBD Rv2525c_GlyHyd-like_TIM-barrel Prok-TelC 3TM-CCDN VanY-like
WP_062428268.1	*Herbidospora daliensis*	PGBD PGBD Rv2525c_GlyHyd-like_TIM-barrel Prok-TelC 3TM-CCDN
WP_235987805.1		PGBD PGBD Rv2525c_GlyHyd-like_TIM-barrel Prok-TelC(frag) 3TM-CCDN VanY-like
WP_112134212.1	*Glycomyces dulcitolivorans*	PGBD PGBD PGBD Rv2525c_GlyHyd-like_TIM-barrel Prok-TelC VanY-like
WP_209707222.1	*Crossiella equi*	PGBD PGBD Rv2525c_GlyHyd-like_TIM-barrel Prok-TelC 3TM-CCDN VanY-like
WP_140457939.1	*Cellulomonas oligotrophica*	PGBD PGBD Rv2525c_GlyHyd-like_TIM-barrel Prok-TelC VanY-like
WP_013117675.1	*Cellulomonas flavigena DSM 20109*	PGBD PGBD Rv2525c_GlyHyd-like_TIM-barrel Prok-TelC(frag) VanY-like
WP_216212399.1	*Amycolatopsis aidingensis*	PGBD PGBD Rv2525c_GlyHyd-like_TIM-barrel Prok-TelC 3TM-CCDN VanY-like
WP_216216096.1	*Amycolatopsis aidingensis*	PGBD PGBD Rv2525c_GlyHyd-like_TIM-barrel Prok-TelC ? VanY-like
WP_217643439.1		PGBD PGBD Rv2525c_GlyHyd-like_TIM-barrel Prok-TelC 3TM-CCDN VanY-like
WP_067494945.1	*Actinoplanes sp. TFC3*	PGBD PGBD Rv2525c_GlyHyd-like_TIM-barrel Prok-TelC VanY-like
WP_015621829.1	*Actinoplanes sp. N902-109*	PGBD PGBD Rv2525c_GlyHyd-like_TIM-barrel Prok-TelC VanY-like
MBB4740352.1	*Actinoplanes octamycinicus*	PGBD PGBD Rv2525c_GlyHyd-like_TIM-barrel Prok-TelC VanY-like
WP_203841747.1	*Winogradskya humida*	PGBD PGBD Rv2525c_GlyHyd-like_TIM-barrel Prok-TelC VanY-like
WP_092782816.1	*Actinokineospora terrae*	PGBD PGBD Rv2525c_GlyHyd-like_TIM-barrel Prok-TelC 3TM-CCDN VanY-like
WP_204455565.1	*Actinokineospora baliensis*	PGBD PGBD Rv2525c_GlyHyd-like_TIM-barrel Prok-TelC 3TM-CCDN VanY-like
WP_223103469.1		PGBD PGBD PGBD Rv2525c_GlyHyd-like_TIM-barrel Prok-TelC VanY-like
WP_228771326.1	*Actinokineospora iranica*	Prok-TelC 3TM-CCDN
WP_223772468.1	*Streptomyces sp. 135*	Prok-TelC ? VanY-like
WP_039933159.1	*Streptomyces viridochromogenes DSM 40736*	Prok-TelC(frag) ? 3TM-CCDN VanY-like
PZS28266.1	*Pseudonocardiales bacterium*	SH3 SH3 Prok-TelC VanY-like

####;standalone genome versions

WP_024288003.1	*Cellulomonas sp. KRMCY2*	PGBD PGBD Rv2525c_GlyHyd-like_TIM-barrel Prok-TelC
DAS95809.1		PGBD PGBD PGBD Rv2525c_GlyHyd-like_TIM-barrel PGBD PGBD Rv2525c_GlyHyd-like_TIM-barrel Prok-TelC
DAO39384.1		PGBD PGBD PGBD Rv2525c_GlyHyd-like_TIM-barrel Prok-TelC
KFZ03760.1	*Pseudogymnoascus sp. VKM F-4520 (FW-2644)*	PGBD PGBD Rv2525c_GlyHyd-like_TIM-barrel Prok-TelC
KAF4332149.1	*Fusarium beomiforme*	PGBD PGBD Rv2525c_GlyHyd-like_TIM-barrel Prok-TelC
KFY86710.1	*Pseudogymnoascus sp. VKM F-4517 (FW-2822)*	PGBD PGBD Rv2525c_GlyHyd-like_TIM-barrel Prok-TelC
WP_205134706.1	*Virgibacillus halotolerans*	PGBD PGBD Rv2525c_GlyHyd-like_TIM-barrel Prok-TelC
WP_217323006.1	*Terribacillus sp. DMT04*	PGBD PGBD Rv2525c_GlyHyd-like_TIM-barrel Prok-TelC
WP_223362323.1	*Sporolactobacillus terrae*	PGBD PGBD Rv2525c_GlyHyd-like_TIM-barrel Prok-TelC
WP_212951000.1	*Siminovitchia terrae*	PGBD PGBD Rv2525c_GlyHyd-like_TIM-barrel Prok-TelC
WP_078395881.1	*Shouchella patagoniensis*	PGBD PGBD Rv2525c_GlyHyd-like_TIM-barrel Prok-TelC
WP_076570164.1		PGBD PGBD Rv2525c_GlyHyd-like_TIM-barrel Prok-TelC(frag)
WP_090529100.1	*Pseudobutyrivibrio sp. UC1225*	PGBD PGBD Rv2525c_GlyHyd-like_TIM-barrel Prok-TelC
WP_090483004.1	*Pseudobutyrivibrio sp. JW11*	PGBD PGBD Rv2525c_GlyHyd-like_TIM-barrel Prok-TelC
MBW4862288.1	*Paeniclostridium sp.*	PGBD PGBD Rv2525c_GlyHyd-like_TIM-barrel Prok-TelC
WP_057574211.1	*Paraclostridium sordellii*	PGBD PGBD Rv2525c_GlyHyd-like_TIM-barrel Prok-TelC
WP_090955669.1	*Paenibacillus sp. OK060*	PGBD PGBD Rv2525c_GlyHyd-like_TIM-barrel Prok-TelC
WP_179263053.1	*Paenibacillus sp. E222*	PGBD PGBD Rv2525c_GlyHyd-like_TIM-barrel Prok-TelC
WP_031462172.1	*Paenibacillus polymyxa*	PGBD PGBD Rv2525c_GlyHyd-like_TIM-barrel Prok-TelC
WP_071705073.1	*Murdochiella vaginalis*	PGBD PGBD Rv2525c_GlyHyd-like_TIM-barrel Prok-TelC
WP_231790011.1	*Metabacillus kandeliae*	PGBD PGBD Rv2525c_GlyHyd-like_TIM-barrel Prok-TelC
WP_087243591.1	*Massilimicrobiota sp. An142*	PGBD PGBD Rv2525c_GlyHyd-like_TIM-barrel Prok-TelC
WP_066144471.1		PGBD PGBD Rv2525c_GlyHyd-like_TIM-barrel Prok-TelC
WP_167841617.1	*Pseudolactococcus laudensis*	PGBD PGBD Rv2525c_GlyHyd-like_TIM-barrel Prok-TelC
WP_197908259.1	*Lactococcus garvieae*	PGBD PGBD Rv2525c_GlyHyd-like_TIM-barrel Prok-TelC
OEZ36365.1	*Lactiplantibacillus plantarum*	PGBD PGBD Rv2525c_GlyHyd-like_TIM-barrel Prok-TelC(frag)
WP_216749050.1		PGBD PGBD Rv2525c_GlyHyd-like_TIM-barrel Prok-TelC
ETW67349.1	*Lacticaseibacillus rhamnosus 2166*	PGBD PGBD Rv2525c_GlyHyd-like_TIM-barrel Prok-TelC
WP_123022583.1	*Lacticaseibacillus paracasei*	PGBD PGBD Rv2525c_GlyHyd-like_TIM-barrel Prok-TelC
RND86818.1	*Lacticaseibacillus paracasei*	PGBD PGBD Rv2525c_GlyHyd-like_TIM-barrel Prok-TelC
EKQ06135.1	*Lacticaseibacillus paracasei*	PGBD PGBD Rv2525c_GlyHyd-like_TIM-barrel Prok-TelC
WP_225877873.1	*Lacticaseibacillus mingshuiensis*	PGBD PGBD Rv2525c_GlyHyd-like_TIM-barrel Prok-TelC
MBR4083288.1	*Lachnospiraceae bacterium*	PGBD PGBD Rv2525c_GlyHyd-like_TIM-barrel Prok-TelC
WP_087308698.1	*Lachnoclostridium sp. An138*	PGBD PGBD Rv2525c_GlyHyd-like_TIM-barrel Prok-TelC
WP_196801257.1	*Lachnobacterium bovis NK4B19*	PGBD PGBD Rv2525c_GlyHyd-like_TIM-barrel Prok-TelC
WP_060931239.1	*Lachnoanaerobaculum saburreum*	PGBD PGBD Rv2525c_GlyHyd-like_TIM-barrel Prok-TelC
WP_008752943.1	*Lachnoanaerobaculum saburreum F0468*	PGBD PGBD Rv2525c_GlyHyd-like_TIM-barrel Prok-TelC
WP_179940193.1	*Gemelliphila palaticanis*	PGBD PGBD Rv2525c_GlyHyd-like_TIM-barrel Prok-TelC
RHP19389.1	*Eubacterium sp. AF34-35BH*	PGBD PGBD Rv2525c_GlyHyd-like_TIM-barrel Prok-TelC
WP_010768680.1	*Enterococcus phoeniculicola*	PGBD PGBD Rv2525c_GlyHyd-like_TIM-barrel Prok-TelC
WP_209555714.1	*Enterococcus larvae*	PGBD PGBD Rv2525c_GlyHyd-like_TIM-barrel Prok-TelC
WP_227740500.1	*Enterococcus faecium*	PGBD PGBD Rv2525c_GlyHyd-like_TIM-barrel Prok-TelC
WP_080832312.1	*Cohnella massiliensis*	PGBD PGBD Rv2525c_GlyHyd-like_TIM-barrel Prok-TelC
WP_182303179.1	*Cohnella cholangitidis*	PGBD PGBD Rv2525c_GlyHyd-like_TIM-barrel Prok-TelC

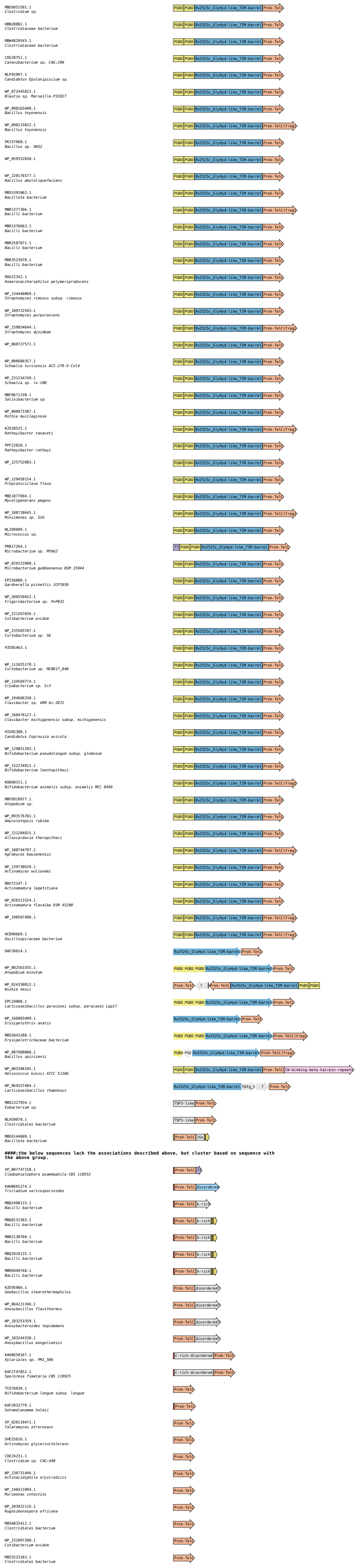
MBE6051501.1
Clostridium sp.
PGBD PGBD Rv2525c_GlyHyd-like_TIM-barrel Prok-TelC
HBN28882.1
Clostridiaceae bacterium
PGBD PGBD Rv2525c_GlyHyd-like_TIM-barrel Prok-TelC
MBW4829545.1
Clostridiaceae bacterium
PGBD PGBD Rv2525c_GlyHyd-like_TIM-barrel Prok-TelC
CDE28751.1
Catenibacterium sp. CAG:290
PGBD PGBD Rv2525c_GlyHyd-like_TIM-barrel Prok-TelC
NLP45997.1
Candidatus Epulonipiscium sp.
PGBD PGBD Rv2525c_GlyHyd-like_TIM-barrel Prok-TelC
WP_072445822.1
Blautia sp. Marseille-P3201T
PGBD PGBD Rv2525c_GlyHyd-like_TIM-barrel Prok-TelC
WP_098163408.1
Bacillus toyonensis
PGBD PGBD Rv2525c_GlyHyd-like_TIM-barrel Prok-TelC
WP_098133022.1
Bacillus toyonensis
PGBD PGBD Rv2525c_GlyHyd-like_TIM-barrel Prok-TelC(frag)
PKJ57968.1
Bacillus sp. SN32
PGBD PGBD Rv2525c_GlyHyd-like_TIM-barrel Prok-TelC
WP_059352838.1
PGBD PGBD Rv2525c_GlyHyd-like_TIM-barrel Prok-TelC
WP_220176577.1
Bacillus amyloliquefaciens
PGBD PGBD Rv2525c_GlyHyd-like_TIM-barrel Prok-TelC
MBS5391062.1
Bacillota bacterium
PGBD PGBD Rv2525c_GlyHyd-like_TIM-barrel Prok-TelC
MBR1377366.1
Bacilli bacterium
PGBD PGBD Rv2525c_GlyHyd-like_TIM-barrel Prok-TelC(frag)
MBR1376663.1
Bacilli bacterium
PGBD PGBD Rv2525c_GlyHyd-like_TIM-barrel Prok-TelC
MBR2587871.1
Bacilli bacterium
PGBD PGBD Rv2525c_GlyHyd-like_TIM-barrel Prok-TelC
MBR3523978.1
Bacilli bacterium
PGBD PGBD Rv2525c_GlyHyd-like_TIM-barrel Prok-TelC
RDU22342.1
Anaerosacchariphilus polymeriproducens
PGBD PGBD Rv2525c_GlyHyd-like_TIM-barrel Prok-TelC
WP_234440869.1
Streptomyces rimosus subsp. rimosus
PGBD PGBD Rv2525c_GlyHyd-like_TIM-barrel Prok-TelC
WP_189722593.1
Streptomyces purpurascens
PGBD PGBD Rv2525c_GlyHyd-like_TIM-barrel Prok-TelC
WP_159054644.1
Streptomyces dysideae
PGBD PGBD Rv2525c_GlyHyd-like_TIM-barrel Prok-TelC(frag)
WP_060727571.1
PGBD PGBD Rv2525c_GlyHyd-like_TIM-barrel Prok-TelC
WP_006680357.1
Schaalia turicensis ACS-279-V-Col4
PGBD PGBD Rv2525c_GlyHyd-like_TIM-barrel Prok-TelC
WP_231234749.1
Schaalia sp. lx-100
PGBD PGBD Rv2525c_GlyHyd-like_TIM-barrel Prok-TelC
MBF0671338.1
Salinibacterium sp.
PGBD PGBD Rv2525c_GlyHyd-like_TIM-barrel Prok-TelC
WP_080973387.1
Rothia mucilaginosa
PGBD PGBD Rv2525c_GlyHyd-like_TIM-barrel Prok-TelC
KZX20525.1
Rathayibacter tanaceti
PGBD PGBD Rv2525c_GlyHyd-like_TIM-barrel Prok-TelC(frag)
PPF22828.1
Rathayibacter rathayi
PGBD PGBD Rv2525c_GlyHyd-like_TIM-barrel Prok-TelC
WP_225752083.1
PGBD PGBD Rv2525c_GlyHyd-like_TIM-barrel Prok-TelC
WP_129458154.1
Propioniciclava flava
PGBD PGBD Rv2525c_GlyHyd-like_TIM-barrel Prok-TelC
MBE1877694.1
Myceligenerans pegani
PGBD PGBD Rv2525c_GlyHyd-like_TIM-barrel Prok-TelC
WP_108718645.1
Miniimonas sp. S16
PGBD PGBD Rv2525c_GlyHyd-like_TIM-barrel Prok-TelC(frag)
NLZ99099.1
Micrococcus sp.
PGBD PGBD Rv2525c_GlyHyd-like_TIM-barrel Prok-TelC
PRB17264.1
Microbacterium sp. MYb62
?? PGBD PGBD Rv2525c_GlyHyd-like_TIM-barrel Prok-TelC
WP_029153908.1
Microbacterium gubbeenense DSM 15944
PGBD PGBD Rv2525c_GlyHyd-like_TIM-barrel Prok-TelC
EPI56080.1
Gardnerella pickettii JCP7659
PGBD PGBD Rv2525c_GlyHyd-like_TIM-barrel Prok-TelC
WP_209559452.1
Frigoribacterium sp. PvP032
PGBD PGBD Rv2525c_GlyHyd-like_TIM-barrel Prok-TelC
WP_221267656.1
Cutibacterium avidum
PGBD PGBD Rv2525c_GlyHyd-like_TIM-barrel Prok-TelC
WP_235503707.1
Curtobacterium sp. S6
PGBD PGBD Rv2525c_GlyHyd-like_TIM-barrel Prok-TelC
PZE85463.1
PGBD PGBD Rv2525c_GlyHyd-like_TIM-barrel Prok-TelC
WP_111025178.1
Curtobacterium sp. MCBD17_040
PGBD PGBD Rv2525c_GlyHyd-like_TIM-barrel Prok-TelC
WP_134569774.1
Cryobacterium sp. Sr3
PGBD PGBD Rv2525c_GlyHyd-like_TIM-barrel Prok-TelC
WP_194666330.1
Clavibacter sp. VKM Ac-2872
PGBD PGBD Rv2525c_GlyHyd-like_TIM-barrel Prok-TelC
WP_160476127.1
Clavibacter michiganensis subsp. michiganensis
PGBD PGBD Rv2525c_GlyHyd-like_TIM-barrel Prok-TelC
HIU05308.1
Candidatus Coprousia avicola
PGBD PGBD Rv2525c_GlyHyd-like_TIM-barrel Prok-TelC
WP_129851293.1
Bifidobacterium pseudolongum subsp. globosum
PGBD PGBD Rv2525c_GlyHyd-like_TIM-barrel Prok-TelC
WP_152234911.1
Bifidobacterium leontopitheci
PGBD PGBD Rv2525c_GlyHyd-like_TIM-barrel Prok-TelC
KOA60311.1
Bifidobacterium animalis subsp. animalis MCC 0499
PGBD PGBD Rv2525c_GlyHyd-like_TIM-barrel Prok-TelC(frag)
MBF0910977.1
Atopobium sp.
PGBD PGBD Rv2525c_GlyHyd-like_TIM-barrel Prok-TelC
WP_093576781.1
Amycolatopsis rubida
PGBD PGBD Rv2525c_GlyHyd-like_TIM-barrel Prok-TelC
WP_131284815.1
Alloscardovia theropitheci
PGBD PGBD Rv2525c_GlyHyd-like_TIM-barrel Prok-TelC
WP_188744797.1
Agromyces bauzanensis
PGBD PGBD Rv2525c_GlyHyd-like_TIM-barrel Prok-TelC(frag)
WP_139738639.1
Actinomyces wuliandei
PGBD PGBD Rv2525c_GlyHyd-like_TIM-barrel Prok-TelC
NDU72147.1
Actinomadura lepetitiana
PGBD PGBD Rv2525c_GlyHyd-like_TIM-barrel Prok-TelC
WP_026313324.1
Actinomadura flavalba DSM 45200
PGBD PGBD Rv2525c_GlyHyd-like_TIM-barrel Prok-TelC
WP_198507480.1
PGBD PGBD Rv2525c_GlyHyd-like_TIM-barrel Prok-TelC(frag)
HCB99669.1
Oscillospiraceae bacterium
PGBD PGBD Rv2525c_GlyHyd-like_TIM-barrel Prok-TelC(frag)
DAF20014.1
Rv2525c_GlyHyd-like_TIM-barrel Prok-TelC
WP_002563355.1
Atopobium minutum
PGBD PGBD PGBD Rv2525c_GlyHyd-like_TIM-barrel Prok-TelC
WP_024330812.1
Winkia neuii
Prok-TelC ? Prok-TelC Rv2525c_GlyHyd-like_TIM-barrel PGBD PGBD
EPC24088.1
Lacticaseibacillus paracasei subsp. paracasei Lpp17
PGBD PGBD PGBD Rv2525c_GlyHyd-like_TIM-barrel Prok-TelC
WP_166083409.1
Erysipelothrix anatis
Rv2525c_GlyHyd-like_TIM-barrel Prok-TelC
MBS5043288.1
Erysipelotrichaceae bacterium
PGBD PGBD PGBD Rv2525c_GlyHyd-like_TIM-barrel Prok-TelC(frag)
WP_087988088.1
Bacillus spizizenii
PGBD PSE Rv2525c_GlyHyd-like_TIM-barrel Prok-TelC(frag)
WP_005398105.1
Helcococcus kunzii ATCC 51366
PGBD PGBD Rv2525c_GlyHyd-like_TIM-barrel Prok-TelC CW-binding-beta-hairpin-repeats
WP_064557484.1
Lacticaseibacillus rhamnosus
Rv2525c_GlyHyd-like_TIM-barrel Ybfg_C ? Prok-TelC
MBQ1327934.1
Eubacterium sp.
TSP3-like Prok-TelC
NLH59079.1
Clostridiales bacterium
TSP3-like Prok-TelC
MBE6144609.1
Bacillota bacterium
Prok-TelC IGs
####;the below sequences lack the associations described above, but cluster based on sequence with the above group.
XP_007747158.1
Cladophialophora psammophila CBS 110553
Prok-TelC ??
KAH8665274.1
Tricladium varicosporioides
Prok-TelC disordered
MBQ2408133.1
Bacilli bacterium
Prok-TelC b-rich
MBQ8131583.1
Bacilli bacterium
Prok-TelC b-rich
MBR2138760.1
Bacilli bacterium
Prok-TelC b-rich
MBQ2910135.1
Bacilli bacterium
Prok-TelC b-rich
MBR6949748.1
Bacilli bacterium
Prok-TelC b-rich
KZE95966.1
Geobacillus stearothermophilus
Prok-TelC disordered?
WP_064221346.1
Anoxybacillus flavithermus
Prok-TelC disordered?
WP_183253359.1
Anoxybacteroides tepidamans
Prok-TelC disordered?
WP_183244330.1
Anoxybacillus mongoliensis
Prok-TelC disordered?
KAH8658167.1
Xylariales sp. PMI_506
C-rich-disordered Prok-TelC
KAF2747852.1
Sporormia fimetaria CBS 119925
C-rich-disordered Prok-TelC
TCD76030.1
Bifidobacterium longum subsp. longum
Prok-TelC
KAF2032779.1
Setomelanomma holmii
Prok-TelC
XP_020119471.1
Talaromyces atroroseus
Prok-TelC
SHE25616.1
Actinomyces glycerinitolerans
Prok-TelC
CDE24231.1
Clostridium sp. CAG:440
Prok-TelC
WP_136731446.1
Actinacidiphila oryziradicis
Prok-TelC
WP_148411094.1
Murimonas intestini
Prok-TelC
WP_203922116.1
Rugosimonospora africana
Prok-TelC
MBS6833412.1
Clostridiales bacterium
Prok-TelC
WP_231097208.1
Cutibacterium avidum
Prok-TelC
MBS5523183.1
Clostridiales bacterium
Prok-TelC

SCD67710.1
Streptomyces sp. di50b
Prok-TelC

WP_055292511.1
Intestinibacter bartlettii
Prok-TelC

TKA76943.1
Cryomyces minteri
Prok-TelC

WP_125929236.1
Bacillus canaveralius
Prok-TelC

WP_045725461.1
Clostridium baratii
Prok-TelC

WP_104259208.1
Rathayibacter tritici
Prok-TelC

WP_156135622.1
Geobacillus kaustophilus
Prok-TelC

MBS7272425.1
Fibrobacter sp.
Prok-TelC

WP_181519958.1
Anoxybacillus ayderensis
Prok-TelC

PKU92147.1
Bifidobacterium pseudolongum subsp. globosum
Prok-TelC

MBR1745125.1
Fibrobacter sp.
Prok-TelC

WP_038425839.1
Bifidobacterium longum
Prok-TelC

WP_006303440.1
Fannyhessea vaginae
Prok-TelC

WP_128683296.1
Actinomyces qiguomingii
Prok-TelC

RIY26741.1
Bifidobacteriaceae bacterium WP022
Prok-TelC

MBF0930733.1
Actinomyces graevenitzii
Prok-TelC

WP_195926528.1
Sarcina ventriculi
Prok-TelC

HAQ1495430.1
Enterococcus faecium
Prok-TelC

WP_161148296.1
Streptomyces sp. SID4982
Prok-TelC

WP_159049268.1
Streptomyces canus
Prok-TelC

WP_191812428.1
Bacillus norwichensis
Prok-TelC

MBF1650658.1
Rothia dentocariosa
Prok-TelC

ACV09617.1
Jonesia denitrificans DSM 20603
Prok-TelC

WP_230068740.1
Arthrobacter cryoconiti
Prok-TelC

WP_158791405.1
Streptomyces sp. NRRL WC-3549
Prok-TelC

OFJ62189.1
Actinomyces sp. HMSC075B09
Prok-TelC

WP_005986444.1
Actinomyces graevenitzii C83
Prok-TelC

MBC8589899.1
Wansuia hejianensis
Prok-TelC

KAG8811915.1
Serendipita sp. 399
Prok-TelC

OAU42196.1
Lacticaseibacillus rhamnosus
Prok-TelC

KOA49375.1
Bifidobacterium breve MCC 1340
Prok-TelC

OOL70212.1
Enterococcus faecium
Prok-TelC

MCE2535882.1
Enterococcus faecalis
Prok-TelC

###############;Prok-TelC associations with amidase domains (possibly paralleling eukaryotic contexts)

WP_157728636.1
Actinopolymorpha singaporensis
Prok-TelC unknown-alpha/beta alpha-helical-region Amidase PGBD

WP_074665160.1
Nitrosomonas communis
PGBP unknown-alpha/beta IG-like Prok-TelC

WP_010982368.1
Streptomyces avermitilis
IG-like Prok-TelC IG-like Amidase

WP_167865039.1
Clostridium gasigenes
SH3 SH3 SH3 Amidase Prok-TelC

MBS4974575.1
Clostridium celatum
Amidase Amidase(frag) Prok-TelC

###############; NAGPA-containing architecture and associated contexts

MBR6668667.1
Clostridia bacterium
sugar-transf GT-B GT-B ? NLPC IG IG b-helix Zn-meander-repeats Prok-TelC NAGPA IG IG Adhesin-like CW-binding-beta-hairpin-repeats Phytase-like CW-binding-beta-hairpin-repeats

MBE5810035.1
Clostridiales bacterium
sugar-transf GT-B GT-B ? ?? NLPC IG IG b-helix Zn-meander-repeats Prok-TelC NAGPA IG IG Adhesin-like CW-binding-beta-hairpin-repeats EF-hand

MBE5970992.1
Lachnoclostridium sp.
HAD GT-A GT-A IG IG b-helix Zn-meander-repeats Prok-TelC NAGPA IG IG Adhesin-like CW-binding-beta-hairpin-repeats

MBR4056838.1
Oscillospiraceae bacterium
TM-Prolipoprotein-diacylglyceryl-transferase IG IG b-helix Zn-meander-repeats Prok-TelC NAGPA IG IG Adhesin-like CW-binding-beta-hairpin-repeats IG-repeats

MBE6727638.1
Oscillospiraceae bacterium
IG IG b-helix Zn-meander-repeats EFHAND Prok-TelC NAGPA IG IG Adhesin-like CW-binding-beta-hairpin-repeats Polysacc_synt Polysacc_synt_C_flippase VanZ

MBE6737637.1
Oscillospiraceae bacterium
ZU5 LytR_cpsA_psr sugar-transf GT-B GT-B GT-B GT-B GT-B GT-B ? ? GT-A ? IG IG b-helix Zn-meander-repeats Prok-TelC NAGPA IG IG Adhesin-like CW-binding-beta-hairpin-repeats Acyltransferase Polysacc_synt Polysacc_synt_C_flippase

MBE6573331.1
Oscillospiraceae bacterium
GT-A GT-A FUKUTIN Acyltransferase NAGPA CW-binding-betahairpin GT-A ? IG IG b-helix Zn-meander-repeats Prok-TelC NAGPA IG IG Adhesin-like CW-binding-beta-hairpin-repeats

MBE6770712.1
Oscillospiraceae bacterium
IG IG b-helix Zn-meander-repeats Prok-TelC NAGPA IG IG Adhesin-like CW-binding-beta-hairpin-repeats

MBR2867878.1
Clostridia bacterium
ZU5 LytR_cpsA_psr sugar-transf GT-B GT-B GT-B GT-B GT-B GT-B ? GT-B GT-B GT-A ? IG IG b-helix Zn-meander-repeats Prok-TelC NAGPA IG IG Adhesin-like CW-binding-beta-hairpin-repeats Polysacc_synt Polysacc_synt_C_flippase

MBD8961174.1
Oscillospiraceae bacterium
ZU5 LytR_cpsA_psr sugar-transf GT-B GT-B GT-B GT-B GT-B GT-B ? GT-B GT-B GT-A ? IG IG b-helix Zn-meander-repeats Prok-TelC NAGPA IG IG Adhesin-like CW-binding-beta-hairpin-repeats Polysacc_synt Polysacc_synt_C_flippase

MBR5806545.1
Oscillospiraceae bacterium
IG IG b-helix Zn-meander-repeats Prok-TelC NAGPA IG IG Adhesin-like CW-binding-beta-hairpin-repeats ? GT-A GT-A GT-A GT-A GT-A GT-A GT-A

MBQ7314915.1
Clostridia bacterium
IG IG b-helix Zn-meander-repeats Prok-TelC NAGPA IG IG Adhesin-like CW-binding-beta-hairpin-repeats

MBQ8898929.1
Clostridia bacterium
IG IG b-helix Zn-meander-repeats Prok-TelC NAGPA IG IG Adhesin-like CW-binding-beta-hairpin-repeats Polysacc_synt Polysacc_synt_C_flippase

MBQ8746651.1
Clostridia bacterium
sugar-transf Acyltransferase GT-A FUKUTIN IG IG b-helix Zn-meander-repeats Prok-TelC NAGPA IG IG Adhesin-like CW-binding-beta-hairpin-repeats ZnF-repeats

MBQ3611887.1
Bacillota bacterium
TM-Prolipoprotein-diacylglyceryl-transferase ? ? IG IG b-helix Zn-meander-repeats Prok-TelC NAGPA IG IG Adhesin-like CW-binding-betahairpin IG IG

MBR2718595.1
Clostridia bacterium
IG IG b-helix Zn-meander-repeats Prok-TelC NAGPA IG IG Adhesin-like CW-binding-betahairpin PT-repeats CW-binding-betahairpin PT-repeats IG IG ? GT-A

MBR7179005.1
Oscillospiraceae bacterium
IG IG b-helix Zn-meander-repeats Prok-TelC NAGPA IG IG Adhesin-like CW-binding-betahairpin b-sandwich ??

MBE5892566.1
Lachnospiraceae bacterium
IG IG b-helix Zn-meander-repeats Prok-TelC NAGPA IG IG Adhesin-like CW-binding-betahairpin EFHAND

MBQ7890142.1
Erysipelotrichaceae bacterium
Zn-meander-repeats Prok-TelC NAGPA IG IG Adhesin-like CW-binding-beta-hairpin-repeats IG-like b-propeller

MBR3795262.1
Clostridia bacterium
Zn-meander-repeats Prok-TelC NAGPA IG IG Adhesin-like CW-binding-beta-hairpin-repeats GT-B GT-B GT-B GT-B GT-A

MBP3478960.1
Oscillospiraceae bacterium
Zn-meander-repeats Prok-TelC NAGPA IG IG Adhesin-like CW-binding-beta-hairpin-repeats GT-B GT-B GT-B GT-B GT-A

MBP3645942.1
Clostridia bacterium
Zn-meander-repeats Prok-TelC NAGPA IG IG Adhesin-like CW-binding-beta-hairpin-repeats Polysacc_synt Polysacc_synt_C_flippase FUKUTIN

MBE6981890.1
Oscillospiraceae bacterium
sugar-transf GT-A FUKUTIN GT-A ? Zn-meander-repeats Prok-TelC NAGPA IG IG Adhesin-like CW-binding-beta-hairpin-repeats Acyltransferase Polysacc_synt Polysacc_synt_C_flippase GT-B GT-B Wzy_C-O-antigen-ligase Wzz ParA-polysaccharide-kinase Cps-CapC_TIMbarrel

MBO5050239.1
Oscillospiraceae bacterium
Zn-meander-repeats Prok-TelC NAGPA IG IG Adhesin-like CW-binding-beta-hairpin-repeats GT-B GT-B GT-B GT-B GT-A

MBE6933611.1
Oscillospiraceae bacterium
Zn-meander-repeats Prok-TelC NAGPA IG IG Adhesin-like CW-binding-beta-hairpin-repeats

MBE6903051.1
Oscillospiraceae bacterium
Zn-meander-repeats Prok-TelC NAGPA IG IG Adhesin-like CW-binding-beta-hairpin-repeats Polysacc_synt Polysacc_synt_C_flippase

MBQ6832174.1
Oscillospiraceae bacterium
Zn-meander-repeats Prok-TelC NAGPA IG IG Adhesin-like CW-binding-beta-hairpin-repeats

MBQ9860006.1
Clostridia bacterium
Zn-meander-repeats Prok-TelC NAGPA IG IG Adhesin-like CW-binding-beta-hairpin-repeats

MBQ4121201.1
Clostridia bacterium
Zn-meander-repeats Prok-TelC NAGPA IG IG Adhesin-like CW-binding-beta-hairpin-repeats

MBR5515129.1
Clostridia bacterium
GT-A FUKUTIN Acyltransferase ZnF-repeats Prok-TelC NAGPA IG IG Adhesin-like CW-binding-beta-hairpin-repeats

MBE6921550.1
Oscillospiraceae bacterium
ZnF-repeats Prok-TelC NAGPA IG IG Adhesin-like CW-binding-beta-hairpin-repeatsIG-like b-propeller

MBR4035169.1
Oscillospiraceae bacterium
ZnF-repeats Prok-TelC NAGPA IG IG Adhesin-like

MBO5396433.1
Clostridia bacterium
ZnF-repeats Prok-TelC NAGPA IG IG Adhesin-like CW-binding-beta-hairpin-repeats EFHAND

MBP1559306.1
Oscillospiraceae bacterium
ZnF-repeats Prok-TelC IG-like ??

MBQ2004578.1
Peptococcaceae bacterium
ZnF-repeats Prok-TelC NAGPA IG IG

MBQ7301185.1
Clostridia bacterium
ZnF Prok-TelC NAGPA IG IG Adhesin-like CW-binding-betahairpin ?? IG-like STY-kinase EFHAND

MBQ4565119.1
Oscillospiraceae bacterium
sugar-transf GT-A FUKUTIN GT-A GT-B GT-B Wzy_C-O-antigen-ligase Acyltransferase Polysacc_synt Polysacc_synt_C_flippase Prok-TelC NAGPA IG IG Adhesin-like CW-binding-beta-hairpin-repeats

MBQ2848041.1
Clostridia bacterium
GT-A FUKUTIN Wzy_C-O-antigen-ligase beta-domain GT-A GT-B GT-B Prok-TelC NAGPA IG IG Adhesin-like CW-binding-beta-hairpin-repeats

MBQ9991996.1
Bacillota bacterium
Prok-TelC NAGPA IG IG Adhesin-like CW-binding-beta-hairpin-repeats

MBR5283517.1
Clostridia bacterium
Prok-TelC NAGPA IG IG Adhesin-like CW-binding-beta-hairpin-repeats

MBR4881297.1
Clostridia bacterium
Prok-TelC NAGPA IG IG Adhesin-like CW-binding-beta-hairpin-repeats

MBP3706214.1
Clostridia bacterium
Prok-TelC NAGPA IG IG Adhesin-like CW-binding-beta-hairpin-repeats EFHAND Polysacc_synt Polysacc_synt_C_flippase

MBR2339001.1
Clostridia bacterium
Wzy_C-O-antigen-ligase GT-A GT-B GT-B Prok-TelC NAGPA IG IG Adhesin-like

HIZ12654.1
Candidatus Mediterraneibacter stercorigallinarum
Prok-TelC NAGPA IG IG

MBP1576272.1
Oscillospiraceae bacterium
Prok-TelC NAGPA IG IG

MBQ8396182.1
Oscillospiraceae bacterium
Prok-TelC NAGPA

MBR4880845.1
Clostridia bacterium
Prok-TelC NAGPA

MBQ8244947.1
Oscillospiraceae bacterium
beta-domain Prok-TelC NAGPA

MBR3692634.1
Clostridia bacterium
Prok-TelC

MBR4033631.1
Clostridia bacterium
Prok-TelC

RHD71643.1
Olsenella sp. AM30-3LB
TM-Prolipoprotein-diacylglyceryl-transferase ? Prok-TelC NAGPA IG IG Adhesin-like CW-binding-beta-hairpin-repeats Acyltransferase GT-A GT-A GT-B GT-B Wzy_C-O-antigen-ligase

MBR5126041.1
Oscillospiraceae bacterium
beta-domain Prok-TelC NAGPA IG IG Adhesin-like CW-binding-beta-hairpin-repeats TM-Prolipoprotein-diacylglyceryl-transferase

MBE6959783.1
Oscillospiraceae bacterium
LytR_cpsA_psr beta-domain GT-A GT-B GT-B Wzy_C-O-antigen-ligase Polysacc_synt Polysacc_synt_C_flippase Prok-TelC NAGPA IG IG Adhesin-like CW-binding-beta-hairpin-repeats

MBE6807060.1
Oscillospiraceae bacterium
Wzz-polysaccharide-kinase ParA-polysaccharide-kinase LytR_cpsA_psr sugar-transf Wzy_C-O-antigen-ligase GT-A FUKUTIN GT-A GT-B GT-B Prok-TelC NAGPA IG IG Adhesin-like CW-binding-beta-hairpin-repeats

HCG67076.1
Clostridiales bacterium
GT-A Wzz-polysaccharide-kinase ParA-polysaccharide-kinase FUKUTIN beta-domain Acyltransferase TM-Prolipoprotein-diacylglyceryl-transferase ? Prok-TelC NAGPA IG IG Adhesin-like CW-binding-beta-hairpin-repeats GT-A GT-A ? GT-B GT-B Wzy_C-O-antigen-ligase

MCC2129042.1
Brotocaccenecus cirricatena
sugar-transf GT-B GT-B GT-B GT-B GT-B ? FUKUTIN beta-domain Prok-TelC NAGPA IG IG Adhesin-like CW-binding-beta-hairpin-repeats TM-Prolipoprotein-diacylglyceryl-transferase-? GT-B GT-B GT-B Acyltransferase Polysacc_synt Polysacc_synt_C_flippase

MBR0447802.1
Clostridia bacterium
Wzz-polysaccharide-kinase LytR_cpsA_psr Wzy_C-O-antigen-ligase GT-A FUKUTIN GT-A GT-B GT-B Prok-TelC NAGPA IG IG Adhesin-like CW-binding-beta-hairpin-repeats

MBO5323388.1
Oscillospiraceae bacterium
Prok-TelC NAGPA IG IG Adhesin-like CW-binding-beta-hairpin-repeats

MBQ7124344.1
Oscillospiraceae bacterium
Prok-TelC NAGPA IG IG Adhesin-like CW-binding-beta-hairpin-repeats

MBO5797279.1
Clostridia bacterium
Prok-TelC NAGPA IG IG Adhesin-like CW-binding-beta-hairpin-repeats TM-Prolipoprotein-diacylglyceryl-transferase

MBS1407795.1
Oscillospiraceae bacterium
sugar-transf Wzz-polysaccharide-kinase ParA-polysaccharide-kinase Cps-CapC_TIMbarrel GT-A FUKUTIN ? GT-B GT-B beta-domain Prok-TelC NAGPA IG IG Adhesin-like CW-binding-beta-hairpin-repeats

PWM34743.1
Oscillospiraceae bacterium
FUKUTIN GT-B GT-B Prok-TelC NAGPA IG IG Adhesin-like CW-binding-beta-hairpin-repeats

MBR2320403.1
Clostridia bacterium
Wzz-polysaccharide-kinase LytR_cpsA_psr sugar-transf Wzy_C-O-antigen-ligase GT-A FUKUTIN GT-A GT-B GT-B Prok-TelC NAGPA IG IG Adhesin-like CW-binding-beta-hairpin-repeats

MBE6928311.1
Oscillospiraceae bacterium
FUKUTIN beta-domain GT-B GT-B Wzy_C-O-antigen-ligase GT-B GT-B Acyltransferase Polysacc_synt Polysacc_synt_C_flippase Prok-TelC NAGPA IG IG Adhesin-like CW-binding-beta-hairpin-repeats

MBE6985141.1
Oscillospiraceae bacterium
GT-B GT-B Wzy_C-O-antigen-ligase GT-B GT-B Polysacc_synt Polysacc_synt_C_flippase Prok-TelC NAGPA IG IG Adhesin-like CW-binding-beta-hairpin-repeats

MBE6682881.1
Oscillospiraceae bacterium
sugar-transf Wzy_C-O-antigen-ligase GT-A FUKUTIN GT-A GT-B GT-B Prok-TelC NAGPA IG IG Adhesin-like CW-binding-beta-hairpin-repeats TM-Prolipoprotein-diacylglyceryl-transferase

MBQ8621422.1
Oscillospiraceae bacterium
Prok-TelC NAGPA IG IG Adhesin-like CW-binding-beta-hairpin-repeats

MBQ1818153.1
Clostridia bacterium
Wzz-polysaccharide-kinase ParA-polysaccharide-kinase Cps-CapC_TIMbarrel GT-B GT-B GT-A GT-B GT-B ? GT-B GT-B ? FUKUTIN Prok-TelC NAGPA IG IG Adhesin-like CW-binding-beta-hairpin-repeats

HCP51269.1
Oscillospiraceae bacterium
beta-domain Prok-TelC NAGPA IG IG Adhesin-like CW-binding-beta-hairpin-repeats

MCD7801436.1
Clostridiales bacterium
sugar-transf GT-A FUKUTIN GT-B GT-B beta-domain Prok-TelC NAGPA IG IG Adhesin-like CW-binding-beta-hairpin-repeats

MCD7881394.1
Clostridiales bacterium
sugar-transf GT-A FUKUTIN GT-B GT-B beta-domain Prok-TelC NAGPA IG IG Adhesin-like CW-binding-beta-hairpin-repeats ? TM-Prolipoprotein-diacylglyceryl-transferase ? ? GT-B Acyltransferase Polysacc_synt Polysacc_synt_C_flippase

MBR5452463.1
Clostridia bacterium
Prok-TelC NAGPA IG IG Adhesin-like CW-binding-beta-hairpin-repeats

MBQ9228226.1
Eubacterium sp.
Wzz-polysaccharide-kinase ParA-polysaccharide-kinase GT-B GT-B GT-B GT-B GT-B ? FUKUTIN TM-Prolipoprotein-diacylglyceryl-transferase ? Prok-TelC NAGPA IG IG Adhesin-like CW-binding-beta-hairpin-repeats EFHAND GT-B GT-B Polysacc_synt Polysacc_synt_C_flippase

MBQ4141545.1
Clostridia bacterium
Prok-TelC NAGPA IG IG Adhesin-like CW-binding-beta-hairpin-repeats EFHAND TM-Prolipoprotein-diacylglyceryl-transferase

MBQ7939133.1
Clostridia bacterium
Cps-CapC_TIMbarrel ZU5 LytR_cpsA_psr sugar-transf Wzy_C-O-antigen-ligase GT-A FUKUTIN beta-domain GT-A GT-B GT-B Prok-TelC NAGPA IG IG Adhesin-like CW-binding-beta-hairpin-repeats EFHAND TM-Prolipoprotein-diacylglyceryl-transferase

MBQ7922069.1
Clostridia bacterium
Cps-CapC_TIMbarrel ZU5 LytR_cpsA_psr sugar-transf Wzy_C-O-antigen-ligase GT-A FUKUTIN beta-domain GT-A GT-B GT-B Prok-TelC NAGPA IG IG Adhesin-like CW-binding-beta-hairpin-repeats EFHAND

MBO5702767.1
Clostridia bacterium
Prok-TelC NAGPA IG IG Adhesin-like CW-binding-beta-hairpin-repeats EFHAND ? TM-Prolipoprotein-diacylglyceryl-transferase

MBQ2920052.1
Oscillospiraceae bacterium
IG-like IG-like b-helix Zn-meander-repeats Prok-TelC NAGPA IG IG Adhesin-like C2-like-repeats

MBQ8522954.1
Clostridia bacterium
Zn-meander-repeats Prok-TelC

MBE7074890.1
Clostridiales bacterium
Zn-meander-repeats Prok-TelC

MBQ7885083.1
Clostridia bacterium
Zn-meander-repeats Prok-TelC

HHQ45171.1
Candidatus Altiarchaeales archaeon
Zn-meander-repeats Prok-TelC

###############;Cyanobacterial-specific minimal family with no apparent means of secretion and no associations. Found as a single-gene island in these genomes

OUC16545.1
Alkalinema sp. CACIAM 70d
Prok-TelC

WP_190569423.1
Alkalinema sp. FACHB-956
Prok-TelC

NJM47760.1
Alkalinema sp. RU_4_3
Prok-TelC

HBE18249.1
Cyanobacteria bacterium UBA11367
Prok-TelC

HBB35205.1
Cyanobacteria bacterium UBA9273
Prok-TelC

BAU10121.1
Leptolyngbya sp. NIES-3755
Prok-TelC

WP_006515851.1
Prok-TelC

NEP15606.1
Leptolyngbya sp. SIO4C1
Prok-TelC

NEQ33788.1
Leptolyngbya sp. SIO4C5
Prok-TelC

WP_063718902.1
Prok-TelC

NJR50868.1
Leptolyngbyaceae cyanobacterium CSU_1_3
Prok-TelC

MBX2866152.1
Leptolyngbyaceae cyanobacterium MAG.088
Prok-TelC

NEO65867.1
Moorena sp. SIO4G2
Prok-TelC

WP_190569050.1
Nostoc parmelioides FACHB-3921
Prok-TelC

WP_015138189.1
Nostoc sp. PCC 7524
Prok-TelC

WP_011319773.1
Trichormus variabilis A2
Prok-TelC

WP_200664312.1
Oxynema sp. CENA135
Prok-TelC

WP_148288044.1
Prochlorothrix hollandica
Prok-TelC

MBE9031350.1
Romeriopsis navalis LEGE 11480
Prok-TelC

MBP0015391.1
Roseofilum sp. SID3
Prok-TelC

NEO30218.1
Symploca sp. SIO3C6
Prok-TelC

MBW4549266.1
Symplocastrum torsivum CPER-KK1
Prok-TelC

WP_190559384.1
Trichocoleus sp. FACHB-262
Prok-TelC

##################;other secreted architectures; #;SP/LP/TM segment fusions

MBD8953202.1
Subdoligranulum sp.
Prok-TelC helix-bundle

MBS1467592.1
Subdoligranulum sp.
Prok-TelC helix-bundle

HJB42470.1
Candidatus Gemmiger avicola
Prok-TelC helix-bundle

WP_143402728.1
Gemmiger formicilis
Prok-TelC helix-bundle

HJC02900.1
Candidatus Gemmiger stercoripullorum
Prok-TelC helix-bundle

PWM60796.1
Subdoligranulum variabile
Prok-TelC helix-bundle

HIZ61663.1
Candidatus Gemmiger avistercoris
Prok-TelC helix-bundle

WP_193503169.1
Gemmiger gallinarum
Prok-TelC helix-bundle

HJB22318.1
Candidatus Allofournierella merdavium
Prok-TelC helix-bundle

WP_087181092.1
Gemmiger sp. An50
Prok-TelC helix-bundle

WP_204657415.1
Gemmiger formicilis
Prok-TelC helix-bundle

WP_235257744.1
Prok-TelC helix-bundle

HJC54578.1
Candidatus Gemmiger stercoravium
Prok-TelC helix-bundle

NCA67961.1
Clostridia bacterium
Prok-TelC

HJA25157.1
Candidatus Allofournierella merdigallinarum
Prok-TelC

MBA4601794.1
Thermoactinomyces mirandus
Prok-TelC

#;YkuD-LD transpeptidase and IG-like fusions

MBW2662687.1
Deltaproteobacteria bacterium
YkuD-LD_transpeptidase-like IG-like Prok-TelC

#;IG-like fusions

MCA1702768.1
Actinomycetota bacterium
IG IG IG Prok-TelC

#;EF-hand fusion

MBQ3002471.1
Clostridia bacterium
Prok-TelC EF-hand

#;IG-like fusion

WP_073070405.1
Phormidesmis priestleyi ULC007
IG-like Prok-TelC

#;SH3 repeats

KFE61101.1
Hyalangium minutum
SH3 SH3 Prok-TelC

##################;other, rare architectures

PWW25182.1
Geodermatophilus normandii
alpha/beta-unknown beta-barrel Prok-TelC

NEP86156.1
Okeania sp. SIO2C2
alpha/beta-unknown beta-barrel Prok-TelC

WP_169824917.1
beta-barrel Prok-TelC

MCB9883678.1
Planctomycetota bacterium
beta-barrel Prok-TelC

#;inactive versions fused to PGBD

WP_146017566.1
Verrucosispora sp. ts21
Prok-TelC-i PGBD

BCB75745.1
Phytohabitans flavus
Prok-TelC-i PGBD

WP_066953303.1
Streptomyces lushanensis
Prok-TelC-i PGBD

WP_013734100.1
Micromonospora maris
Prok-TelC-i PGBD

WP_173035675.1
Phytohabitans flavus
Prok-TelC-i PGBD

#;fused to PGBD domains

WP_021637055.1
Eisenbergiella porci
PGBD Prok-TelC

#;a/b-repeats fusion

WP_163286858.1
Cryomorpha ignava
a/b-repeats Prok-TelC ? a/b-repeats Prok-TelC a/b-repeats Prok-TelC a/b-repeats Prok-TelC ? ? ? a/b-repeats Prok-TelC

#;NLPC and SH3-repeats

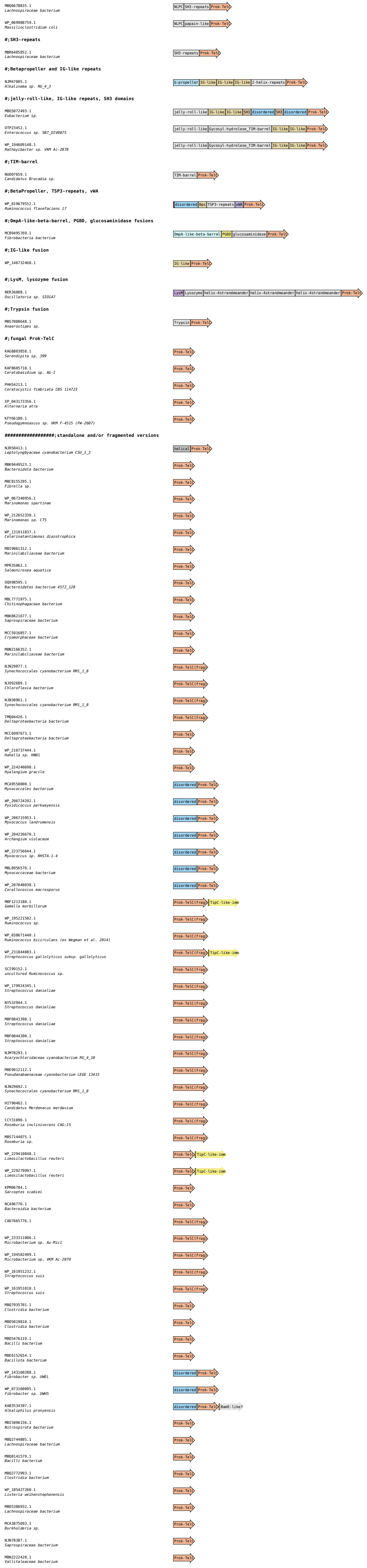
MBQ6678835.1 Lachnospiraceae bacterium — NLPC | SH3-repeats | Prok-TelC
WP_069988759.1 Massilioclostridium coli — NLPC | papain-like | Prok-TelC
#;SH3-repeats
MBR6405852.1 Lachnospiraceae bacterium — SH3-repeats | Prok-TelC
#;Betapropeller and IG-like repeats
NJM47005.1 Alkalinema sp. RU_4_3 — b-propeller | IG-like | IG-like | IG-like | 2-helix-repeats | Prok-TelC
#;jelly-roll-like, IG-like repeats, SH3 domains
MBO5072493.1 Eubacterium sp. — jelly-roll-like | IG-like | IG-like | SH3 | disordered | SH3 | disordered | Prok-TelC
OTP23452.1 Enterococcus sp. 5B7_DIV0075 — jelly-roll-like | Gycosyl-hydrolase_TIM-barrel | IG-like | IG-like | Prok-TelC
WP_194609148.1 Rathayibacter sp. VKM Ac-2878 — jelly-roll-like | Gycosyl-hydrolase_TIM-barrel | IG-like | IG-like | Prok-TelC
#;TIM-barrel
NUO07659.1 Candidatus Brocadia sp. — TIM-barrel | Prok-TelC
#;BetaPropeller, TSP3-repeats, vWA
WP_019679552.1 Ruminococcus flavefaciens 17 — disordered | Bps | TSP3-repeats | vWA | Prok-TelC
#;OmpA-like-beta-barrel, PGBD, glucosaminidase fusions
MCB9495769.1 Fibrobacteria bacterium — OmpA-like-beta-barrel | PGBD | glucosaminidase | Prok-TelC
#;IG-like fusion
WP_146732468.1 — IG-like | Prok-TelC
#;LysM, lysozyme fusion
NER36008.1 Oscillatoria sp. SIO1A7 — LysM | Lysozyme | helix-4strandmeander | helix-4strandmeander | helix-4strandmeander | Prok-TelC
#;Trypsin fusion
MBS7008648.1 Anaerostipes sp. — Trypsin | Prok-TelC
#;fungal Prok-TelC
KAG8803858.1 Serendipita sp. 399 — Prok-TelC
KAF8605710.1 Ceratobasidium sp. AG-I — Prok-TelC
PHH54213.1 Ceratocystis fimbriata CBS 114723 — Prok-TelC
XP_043173356.1 Alternaria atra — Prok-TelC
KFY66180.1 Pseudogymnoascus sp. VKM F-4515 (FW-2607) — Prok-TelC
##################;standalone and/or fragmented versions
NJR50413.1 Leptolyngbyaceae cyanobacterium CSU_1_3 — helical | Prok-TelC
MBK9449523.1 Bacteroidota bacterium — Prok-TelC
MBC8155295.1 Fibrella sp. — Prok-TelC
WP_067340956.1 Marinomonas spartinae — Prok-TelC
WP_212652330.1 Marinomonas sp. CT5 — Prok-TelC
WP_131911837.1 Celerinatantimonas diazotrophica — Prok-TelC
MBI9061312.1 Marinilabiliaceae bacterium — Prok-TelC
MPR35062.1 Salmonirosea aquatica — Prok-TelC
OQX98595.1 Bacteroidetes bacterium 4572_128 — Prok-TelC
MBL7771975.1 Chitinophagaceae bacterium — Prok-TelC
MBK8621677.1 Saprospiraceae bacterium — Prok-TelC
MCC5916857.1 Cryomorphaceae bacterium — Prok-TelC
MBN2166352.1 Marinilabiliaceae bacterium — Prok-TelC
NJN29877.1 Synechococcales cyanobacterium RM1_1_8 — Prok-TelC(frag)
NJO92689.1 Chloroflexia bacterium — Prok-TelC(frag)
NJN30961.1 Synechococcales cyanobacterium RM1_1_8 — Prok-TelC(frag)
TMQ04426.1 Deltaproteobacteria bacterium — Prok-TelC(frag)
MCC6997673.1 Deltaproteobacteria bacterium — Prok-TelC
WP_216737444.1 Hahella sp. HN01 — Prok-TelC
WP_224240698.1 Hyalangium gracile — Prok-TelC
MCA9550000.1 Myxococcales bacterium — disordered | Prok-TelC
WP_206724292.1 Pyxidicoccus parkwayensis — disordered | Prok-TelC
WP_206715953.1 Myxococcus landrumensis — disordered | Prok-TelC
WP_204226670.1 Archangium violaceum — disordered | Prok-TelC
WP_223756644.1 Myxococcus sp. RHSTA-1-4 — disordered | Prok-TelC
MBL8956570.1 Myxococcaceae bacterium — disordered | Prok-TelC
WP_207048038.1 Corallococcus macrosporus — disordered | Prok-TelC
MBF1213180.1 Gemella morbillorum — Prok-TelC(frag) | TipC-like-imm
WP_195221502.1 Ruminococcus sp. — Prok-TelC(frag)
WP_038671440.1 Ruminococcus bicirculans (ex Wegman et al. 2014) — Prok-TelC(frag)
WP_231844083.1 Streptococcus gallolyticus subsp. gallolyticus — Prok-TelC(frag) | TipC-like-imm
SCI99152.1 uncultured Ruminococcus sp. — Prok-TelC(frag)
WP_179924345.1 Streptococcus danieliae — Prok-TelC(frag)
NYS32944.1 Streptococcus danieliae — Prok-TelC(frag)
MBF0843398.1 Streptococcus danieliae — Prok-TelC(frag)
MBF0844390.1 Streptococcus danieliae — Prok-TelC(frag)
NJM78293.1 Acaryochloridaceae cyanobacterium RU_4_10 — Prok-TelC(frag)
MBE9012112.1 Pseudanabaenaceae cyanobacterium LEGE 13415 — Prok-TelC(frag)
NJN29692.1 Synechococcales cyanobacterium RM1_1_8 — Prok-TelC(frag)
HIT90462.1 Candidatus Merdenecus merdavium — Prok-TelC(frag)
CCY31096.1 Roseburia inulinivorans CAG:15 — Prok-TelC(frag)
MBS7144075.1 Roseburia sp. — Prok-TelC(frag)
WP_229410848.1 Limosilactobacillus reuteri — Prok-TelC | TipC-like-imm
WP_229279997.1 Limosilactobacillus reuteri — Prok-TelC | TipC-like-imm
KPM06784.1 Sarcoptes scabiei — Prok-TelC
NCA96776.1 Bacteroidia bacterium — Prok-TelC
CAD7665776.1 — Prok-TelC(frag)
WP_233311006.1 Microbacterium sp. Au-Mic1 — Prok-TelC(frag)
WP_194582499.1 Microbacterium sp. VKM Ac-2870 — Prok-TelC(frag)
WP_161951232.1 Streptococcus suis — Prok-TelC(frag)
WP_161951010.1 Streptococcus suis — Prok-TelC(frag)
MBQ7935701.1 Clostridia bacterium — Prok-TelC
MBO5019810.1 Clostridia bacterium — Prok-TelC
MBO5476119.1 Bacilli bacterium — Prok-TelC
MBE6152654.1 Bacillota bacterium — Prok-TelC
WP_143160288.1 Fibrobacter sp. UWEL — disordered | Prok-TelC
WP_073160095.1 Fibrobacter sp. UWH5 — disordered | Prok-TelC
KAB3534397.1 Alkaliphilus pronyensis — disordered | Prok-TelC | BamE-like?
MBI5096156.1 Nitrospirota bacterium — Prok-TelC
MBQ2744805.1 Lachnospiraceae bacterium — Prok-TelC
MBQ8141579.1 Bacilli bacterium — Prok-TelC
MBQ2772993.1 Clostridia bacterium — Prok-TelC
WP_185427260.1 Listeria weihenstephanensis — Prok-TelC
MBO5386932.1 Lachnospiraceae bacterium — Prok-TelC
MCA3875693.1 Burkholderia sp. — Prok-TelC
NJN78387.1 Saprospiraceae bacterium — Prok-TelC
MBN2222420.1 Vallitaleaceae bacterium — Prok-TelC

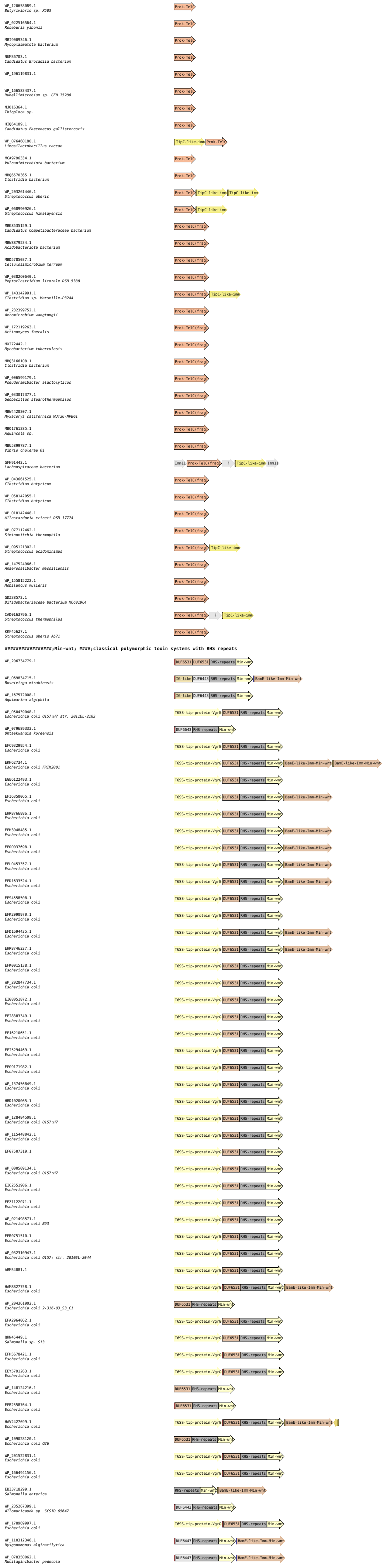
WP_120658089.1
Butyrivibrio sp. X503
Prok-TelC
WP_022516564.1
Roseburia yibonii
Prok-TelC
MBI9009346.1
Mycoplasmatota bacterium
Prok-TelC
NUM36703.1
Candidatus Brocadiia bacterium
Prok-TelC
WP_196119831.1
Prok-TelC
WP_166583437.1
Rubellimicrobium sp. CFH 75288
Prok-TelC
NJO16364.1
Thioploca sp.
Prok-TelC
HIQ64189.1
Candidatus Faecenecus gallistercoris
Prok-TelC
WP_076460180.1
Limosilactobacillus caccae
TipC-like-imm
Prok-TelC
MCA9796334.1
Vulcanimicrobiota bacterium
Prok-TelC
MBQ6570365.1
Clostridia bacterium
Prok-TelC
WP_203261446.1
Streptococcus uberis
Prok-TelC
TipC-like-imm
TipC-like-imm
WP_068990926.1
Streptococcus himalayensis
Prok-TelC
TipC-like-imm
MBK8535159.1
Candidatus Competibacteraceae bacterium
Prok-TelC(frag)
MBW8879534.1
Acidobacteriota bacterium
Prok-TelC(frag)
MBD5785037.1
Cellulosimicrobium terreum
Prok-TelC(frag)
WP_038260640.1
Peptoclostridium litorale DSM 5388
Prok-TelC(frag)
WP_143142991.1
Clostridium sp. Marseille-P3244
Prok-TelC(frag)
TipC-like-imm
WP_232399752.1
Aeromicrobium wangtongii
Prok-TelC(frag)
WP_172119263.1
Actinomyces faecalis
Prok-TelC(frag)
MXI72442.1
Mycobacterium tuberculosis
Prok-TelC(frag)
MBQ3166108.1
Clostridia bacterium
Prok-TelC(frag)
WP_006599179.1
Pseudoramibacter alactolyticus
Prok-TelC(frag)
WP_033017377.1
Geobacillus stearothermophilus
Prok-TelC(frag)
MBW4420307.1
Myxacorys californica WJT36-NPBG1
Prok-TelC(frag)
MBQ1761385.1
Aquincola sp.
Prok-TelC(frag)
MBU5899787.1
Vibrio cholerae O1
Prok-TelC(frag)
GFH91442.1
Lachnospiraceae bacterium
Imm11
Prok-TelC(frag)
?
TipC-like-imm
Imm11
WP_043661525.1
Clostridium butyricum
Prok-TelC(frag)
WP_058142055.1
Clostridium butyricum
Prok-TelC(frag)
WP_018142448.1
Alloscardovia criceti DSM 17774
Prok-TelC(frag)
WP_077112462.1
Siminovitchia thermophila
Prok-TelC(frag)
WP_095121302.1
Streptococcus acidominimus
Prok-TelC(frag)
TipC-like-imm
WP_147524966.1
Anaerosalibacter massiliensis
Prok-TelC(frag)
WP_155815222.1
Mobiluncus mulieris
Prok-TelC(frag)
GDZ38572.1
Bifidobacteriaceae bacterium MCC01964
Prok-TelC(frag)
CAD0163796.1
Streptococcus thermophilus
Prok-TelC(frag)
?
TipC-like-imm
KKF45627.1
Streptococcus uberis Ab71
Prok-TelC(frag)
#################;Min-wnt; ####;classical polymorphic toxin systems with RHS repeats
WP_206734779.1
DUF6531
DUF6531
RHS-repeats
Min-wnt
WP_069834715.1
Roseivirga misakiensis
IG-like
DUF6443
RHS-repeats
Min-wnt
BamE-like-Imm-Min-wnt
WP_167572008.1
Aquimarina algiphila
IG-like
DUF6443
RHS-repeats
Min-wnt
WP_050439048.1
Escherichia coli O157:H7 str. 2011EL-2103
T6SS-tip-protein-VgrG
DUF6531
RHS-repeats
Min-wnt
WP_079689333.1
Ohtaekwangia koreensis
DUF6643
RHS-repeats
Min-wnt
EFC9329954.1
Escherichia coli
T6SS-tip-protein-VgrG
DUF6531
RHS-repeats
Min-wnt
EKH62734.1
Escherichia coli FRIK2001
T6SS-tip-protein-VgrG
DUF6531
RHS-repeats
Min-wnt
BamE-like-Imm-Min-wnt
BamE-like-Imm-Min-wnt
EGE6122493.1
Escherichia coli
T6SS-tip-protein-VgrG
DUF6531
RHS-repeats
Min-wnt
EFI6350065.1
Escherichia coli
T6SS-tip-protein-VgrG
DUF6531
RHS-repeats
Min-wnt
BamE-like-Imm-Min-wnt
EHR8766886.1
Escherichia coli
T6SS-tip-protein-VgrG
DUF6531
RHS-repeats
Min-wnt
EFH3048485.1
Escherichia coli
T6SS-tip-protein-VgrG
DUF6531
RHS-repeats
Min-wnt
BamE-like-Imm-Min-wnt
EFO0037698.1
Escherichia coli
T6SS-tip-protein-VgrG
DUF6531
RHS-repeats
Min-wnt
BamE-like-Imm-Min-wnt
EFL0453357.1
Escherichia coli
T6SS-tip-protein-VgrG
DUF6531
RHS-repeats
Min-wnt
BamE-like-Imm-Min-wnt
EFD1633524.1
Escherichia coli
T6SS-tip-protein-VgrG
DUF6531
RHS-repeats
Min-wnt
BamE-like-Imm-Min-wnt
EES4558508.1
Escherichia coli
T6SS-tip-protein-VgrG
DUF6531
RHS-repeats
Min-wnt
EFK2090970.1
Escherichia coli
T6SS-tip-protein-VgrG
DUF6531
RHS-repeats
Min-wnt
EFD1694425.1
Escherichia coli
T6SS-tip-protein-VgrG
DUF6531
RHS-repeats
Min-wnt
BamE-like-Imm-Min-wnt
EHR8746227.1
Escherichia coli
T6SS-tip-protein-VgrG
DUF6531
RHS-repeats
Min-wnt
BamE-like-Imm-Min-wnt
EFK0015138.1
Escherichia coli
T6SS-tip-protein-VgrG
DUF6531
RHS-repeats
Min-wnt
WP_202847734.1
Escherichia coli
T6SS-tip-protein-VgrG
DUF6531
RHS-repeats
Min-wnt
EIG8051872.1
Escherichia coli
T6SS-tip-protein-VgrG
DUF6531
RHS-repeats
Min-wnt
EFI8383349.1
Escherichia coli
T6SS-tip-protein-VgrG
DUF6531
RHS-repeats
Min-wnt
EFJ6210651.1
Escherichia coli
T6SS-tip-protein-VgrG
DUF6531
RHS-repeats
Min-wnt
EFI5294469.1
Escherichia coli
T6SS-tip-protein-VgrG
DUF6531
RHS-repeats
Min-wnt
EFG9171982.1
Escherichia coli
T6SS-tip-protein-VgrG
DUF6531
RHS-repeats
Min-wnt
WP_137456849.1
Escherichia coli
T6SS-tip-protein-VgrG
DUF6531
RHS-repeats
Min-wnt
HBD1020065.1
Escherichia coli
T6SS-tip-protein-VgrG
DUF6531
RHS-repeats
Min-wnt
WP_128484508.1
Escherichia coli O157:H7
T6SS-tip-protein-VgrG
DUF6531
RHS-repeats
Min-wnt
WP_115448042.1
Escherichia coli
T6SS-tip-protein-VgrG
DUF6531
RHS-repeats
Min-wnt
EFG7507319.1
T6SS-tip-protein-VgrG
DUF6531
RHS-repeats
Min-wnt
WP_000509134.1
Escherichia coli O157:H7
T6SS-tip-protein-VgrG
DUF6531
RHS-repeats
Min-wnt
EIC2551906.1
Escherichia coli
T6SS-tip-protein-VgrG
DUF6531
RHS-repeats
Min-wnt
EEZ1122071.1
Escherichia coli
T6SS-tip-protein-VgrG
DUF6531
RHS-repeats
Min-wnt
WP_021498571.1
Escherichia coli B93
T6SS-tip-protein-VgrG
DUF6531
RHS-repeats
Min-wnt
EER0751510.1
Escherichia coli
T6SS-tip-protein-VgrG
DUF6531
RHS-repeats
Min-wnt
WP_032310943.1
Escherichia coli O157: str. 2010EL-2044
T6SS-tip-protein-VgrG
DUF6531
RHS-repeats
Min-wnt
ABM54881.1
T6SS-tip-protein-VgrG
DUF6531
RHS-repeats
Min-wnt
HAM8827758.1
Escherichia coli
T6SS-tip-protein-VgrG
DUF6531
RHS-repeats
Min-wnt
BamE-like-Imm-Min-wnt
WP_204361902.1
Escherichia coli 2-316-03_S3_C1
DUF6531
RHS-repeats
Min-wnt
EFA2964062.1
Escherichia coli
T6SS-tip-protein-VgrG
DUF6531
RHS-repeats
Min-wnt
QHN45449.1
Salmonella sp. S13
T6SS-tip-protein-VgrG
DUF6531
RHS-repeats
Min-wnt
EFH5678421.1
Escherichia coli
T6SS-tip-protein-VgrG
DUF6531
RHS-repeats
Min-wnt
EEY5791263.1
Escherichia coli
T6SS-tip-protein-VgrG
DUF6531
RHS-repeats
Min-wnt
WP_148124216.1
Escherichia coli
DUF6531
RHS-repeats
Min-wnt
EFB2558764.1
Escherichia coli
DUF6531
RHS-repeats
Min-wnt
HAV2427699.1
Escherichia coli
T6SS-tip-protein-VgrG
DUF6531
RHS-repeats
Min-wnt
BamE-like-Imm-Min-wnt
WP_109028120.1
Escherichia coli O26
DUF6531
RHS-repeats
Min-wnt
WP_201522831.1
Escherichia coli
T6SS-tip-protein-VgrG
DUF6531
RHS-repeats
Min-wnt
WP_166494156.1
Escherichia coli
T6SS-tip-protein-VgrG
DUF6531
RHS-repeats
Min-wnt
EBI3718299.1
Salmonella enterica
RHS-repeats
Min-wnt
BamE-like-Imm-Min-wnt
WP_235267399.1
Allomuricauda sp. SCSIO 65647
DUF6443
RHS-repeats
Min-wnt
WP_178969997.1
Escherichia coli
T6SS-tip-protein-VgrG
DUF6531
RHS-repeats
Min-wnt
WP_110312346.1
Dysgonomonas alginatilytica
DUF6443
RHS-repeats
Min-wnt
BamE-like-Imm-Min-wnt
WP_078350062.1
Mucilaginibacter pedocola
DUF6443
RHS-repeats
Min-wnt
BamE-like-Imm-Min-wnt

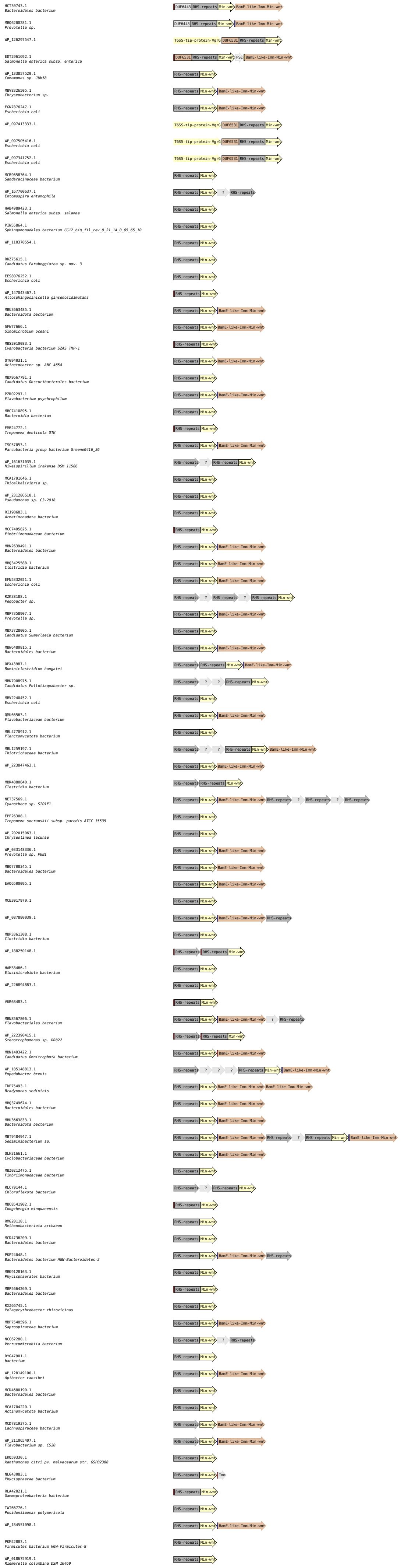
HCT30743.1
Bacteroidales bacterium
DUF6443 RHS-repeats Min-wnt BamE-like-Imm-Min-wnt
MBQ6200281.1
Prevotella sp.
DUF6443 RHS-repeats Min-wnt BamE-like-Imm-Min-wnt
WP_126297547.1
T6SS-tip-protein-VgrG DUF6531 RHS-repeats Min-wnt
EDT2961692.1
Salmonella enterica subsp. enterica
DUF6531 RHS-repeats Min-wnt PSE BamE-like-Imm-Min-wnt
WP_133857520.1
Comamonas sp. JUb58
RHS-repeats Min-wnt
MBV8326505.1
Chryseobacterium sp.
RHS-repeats Min-wnt BamE-like-Imm-Min-wnt
EGN7876247.1
Escherichia coli
RHS-repeats Min-wnt BamE-like-Imm-Min-wnt
WP_097413333.1
T6SS-tip-protein-VgrG DUF6531 RHS-repeats Min-wnt
WP_097505416.1
Escherichia coli
T6SS-tip-protein-VgrG DUF6531 RHS-repeats Min-wnt
WP_097341752.1
Escherichia coli
T6SS-tip-protein-VgrG DUF6531 RHS-repeats Min-wnt
MCB9658364.1
Sandaracinaceae bacterium
RHS-repeats Min-wnt
WP_167700637.1
Entomospira entomophila
RHS-repeats Min-wnt ? RHS-repeats
HAB4989423.1
Salmonella enterica subsp. salamae
RHS-repeats Min-wnt
PIW55864.1
Sphingomonadales bacterium CG12_big_fil_rev_8_21_14_0_65_65_10
RHS-repeats Min-wnt
WP_110370554.1
RHS-repeats Min-wnt
RKZ75615.1
Candidatus Parabeggiatoa sp. nov. 3
RHS-repeats Min-wnt
EES8076252.1
Escherichia coli
RHS-repeats Min-wnt
WP_147043467.1
Allosphingosinicella ginsenosidimutans
RHS-repeats Min-wnt
MBU3663485.1
Bacteroidota bacterium
RHS-repeats Min-wnt BamE-like-Imm-Min-wnt
SFW77666.1
Sinomicrobium oceani
RHS-repeats Min-wnt BamE-like-Imm-Min-wnt
MBS2010083.1
Cyanobacteria bacterium SZAS TMP-1
RHS-repeats Min-wnt
OTG94031.1
Acinetobacter sp. ANC 4654
RHS-repeats Min-wnt BamE-like-Imm-Min-wnt
MBX9667791.1
Candidatus Obscuribacterales bacterium
RHS-repeats Min-wnt
PZR02297.1
Flavobacterium psychrophilum
RHS-repeats Min-wnt BamE-like-Imm-Min-wnt
MBC7410895.1
Bacteroidia bacterium
RHS-repeats Min-wnt
EMB24772.1
Treponema denticola OTK
RHS-repeats Min-wnt
TSC57053.1
Parcubacteria group bacterium Greene0416_36
RHS-repeats Min-wnt BamE-like-Imm-Min-wnt
WP_161631035.1
Niveispirillum irakense DSM 11586
RHS-repeats ? RHS-repeats Min-wnt
MCA1791646.1
Thioalkalivibrio sp.
RHS-repeats Min-wnt
WP_231286510.1
Pseudomonas sp. C3-2018
RHS-repeats Min-wnt
RIJ98683.1
Armatimonadota bacterium
RHS-repeats Min-wnt
MCC7495825.1
Fimbriimonadaceae bacterium
RHS-repeats Min-wnt
MBN2639491.1
Bacteroidales bacterium
RHS-repeats Min-wnt BamE-like-Imm-Min-wnt
MBQ3425588.1
Clostridia bacterium
RHS-repeats Min-wnt BamE-like-Imm-Min-wnt
EFN5332021.1
Escherichia coli
RHS-repeats Min-wnt BamE-like-Imm-Min-wnt
RZK38188.1
Pedobacter sp.
RHS-repeats ? RHS-repeats ? RHS-repeats Min-wnt
MBP7358907.1
Prevotella sp.
RHS-repeats Min-wnt BamE-like-Imm-Min-wnt
MBX3728005.1
Candidatus Sumerlaeia bacterium
RHS-repeats Min-wnt
MBW6480815.1
Bacteroidales bacterium
RHS-repeats Min-wnt BamE-like-Imm-Min-wnt
OPX43987.1
Ruminiclostridium hungatei
RHS-repeats RHS-repeats Min-wnt BamE-like-Imm-Min-wnt
MBK7908975.1
Candidatus Pollutiaquabacter sp.
RHS-repeats ? ? RHS-repeats Min-wnt
MBV2240452.1
Escherichia coli
RHS-repeats Min-wnt
QMU66563.1
Flavobacteriaceae bacterium
RHS-repeats Min-wnt BamE-like-Imm-Min-wnt
MBL4770912.1
Planctomycetota bacterium
RHS-repeats Min-wnt
MBL1259197.1
Thiotrichaceae bacterium
RHS-repeats ? ? RHS-repeats Min-wnt BamE-like-Imm-Min-wnt
WP_223847463.1
RHS-repeats Min-wnt BamE-like-Imm-Min-wnt
MBR4880840.1
Clostridia bacterium
RHS-repeats RHS-repeats Min-wnt
NET37569.1
Cyanothece sp. SIO1E1
RHS-repeats Min-wnt BamE-like-Imm-Min-wnt RHS-repeats ? RHS-repeats ? RHS-repeats
EPF26308.1
Treponema socranskii subsp. paredis ATCC 35535
RHS-repeats Min-wnt
WP_202015063.1
Chryseolinea lacunae
RHS-repeats Min-wnt
WP_033148336.1
Prevotella sp. P6B1
RHS-repeats Min-wnt BamE-like-Imm-Min-wnt
MBQ7708345.1
Bacteroidales bacterium
RHS-repeats Min-wnt BamE-like-Imm-Min-wnt
EAQ6500095.1
RHS-repeats Min-wnt BamE-like-Imm-Min-wnt
MCE3017979.1
RHS-repeats Min-wnt
WP_087880039.1
RHS-repeats Min-wnt BamE-like-Imm-Min-wnt RHS-repeats
MBP3361308.1
Clostridia bacterium
RHS-repeats Min-wnt
WP_188250148.1
RHS-repeats RHS-repeats Min-wnt
HAM38466.1
Elusimicrobiota bacterium
RHS-repeats Min-wnt
WP_226894883.1
RHS-repeats Min-wnt
VUR68483.1
RHS-repeats Min-wnt
MBN8567806.1
Flavobacteriales bacterium
RHS-repeats Min-wnt BamE-like-Imm-Min-wnt ? RHS-repeats
WP_222390415.1
Stenotrophomonas sp. DR822
RHS-repeats RHS-repeats Min-wnt
MBN1493422.1
Candidatus Omnitrophota bacterium
RHS-repeats Min-wnt BamE-like-Imm-Min-wnt
WP_185148813.1
Empedobacter brevis
RHS-repeats ? ? ? RHS-repeats Min-wnt BamE-like-Imm-Min-wnt
TDP75493.1
Bradymonas sediminis
RHS-repeats Min-wnt BamE-like-Imm-Min-wnt BamE-like-Imm-Min-wnt
MBQ3749674.1
Bacteroidales bacterium
RHS-repeats Min-wnt BamE-like-Imm-Min-wnt
MBU3663833.1
Bacteroidota bacterium
RHS-repeats Min-wnt BamE-like-Imm-Min-wnt
MBT9484947.1
Sediminibacterium sp.
RHS-repeats Min-wnt BamE-like-Imm-Min-wnt RHS-repeats ? RHS-repeats Min-wnt BamE-like-Imm-Min-wnt
QLH31661.1
Cyclobacteriaceae bacterium
RHS-repeats Min-wnt BamE-like-Imm-Min-wnt
MBZ0212475.1
Fimbriimonadaceae bacterium
RHS-repeats Min-wnt
RLC79144.1
Chloroflexota bacterium
RHS-repeats ? RHS-repeats Min-wnt
MBC8541902.1
Congzhengia minquanensis
RHS-repeats Min-wnt
RMG20118.1
Methanobacteriota archaeon
RHS-repeats Min-wnt
MCD4736209.1
Bacteroidales bacterium
RHS-repeats Min-wnt
PKP24048.1
Bacteroidetes bacterium HGW-Bacteroidetes-2
RHS-repeats Min-wnt BamE-like-Imm-Min-wnt RHS-repeats
MBK9128163.1
Phycisphaerales bacterium
RHS-repeats Min-wnt
MBP5664269.1
Bacteroidales bacterium
RHS-repeats Min-wnt
RXZ66745.1
Pelagerythrobacter rhizovicinus
RHS-repeats Min-wnt
MBP7540596.1
Saprospiraceae bacterium
RHS-repeats Min-wnt BamE-like-Imm-Min-wnt
NCC62280.1
Verrucomicrobiia bacterium
RHS-repeats Min-wnt ? RHS-repeats
RYG47901.1
bacterium
RHS-repeats Min-wnt
WP_128149100.1
Apibacter raozihei
RHS-repeats Min-wnt BamE-like-Imm-Min-wnt
MCD4680190.1
Bacteroidales bacterium
RHS-repeats Min-wnt
MCA1704220.1
Actinomycetota bacterium
RHS-repeats Min-wnt
MCD7819375.1
Lachnospiraceae bacterium
RHS-repeats Min-wnt BamE-like-Imm-Min-wnt
WP_211065407.1
Flavobacterium sp. CS20
RHS-repeats Min-wnt BamE-like-Imm-Min-wnt
EKQ59330.1
Xanthomonas citri pv. malvacearum str. GSPB2388
RHS-repeats Min-wnt
NLG43083.1
Phycisphaerae bacterium
RHS-repeats Min-wnt Imm
RLA42021.1
Gammaproteobacteria bacterium
RHS-repeats Min-wnt
TWT66776.1
Posidoniimonas polymericola
RHS-repeats Min-wnt
WP_184551098.1
RHS-repeats Min-wnt BamE-like-Imm-Min-wnt
PKM42883.1
Firmicutes bacterium HGW-Firmicutes-8
RHS-repeats Min-wnt
WP_018675919.1
Riemerella columbina DSM 16469
RHS-repeats Min-wnt

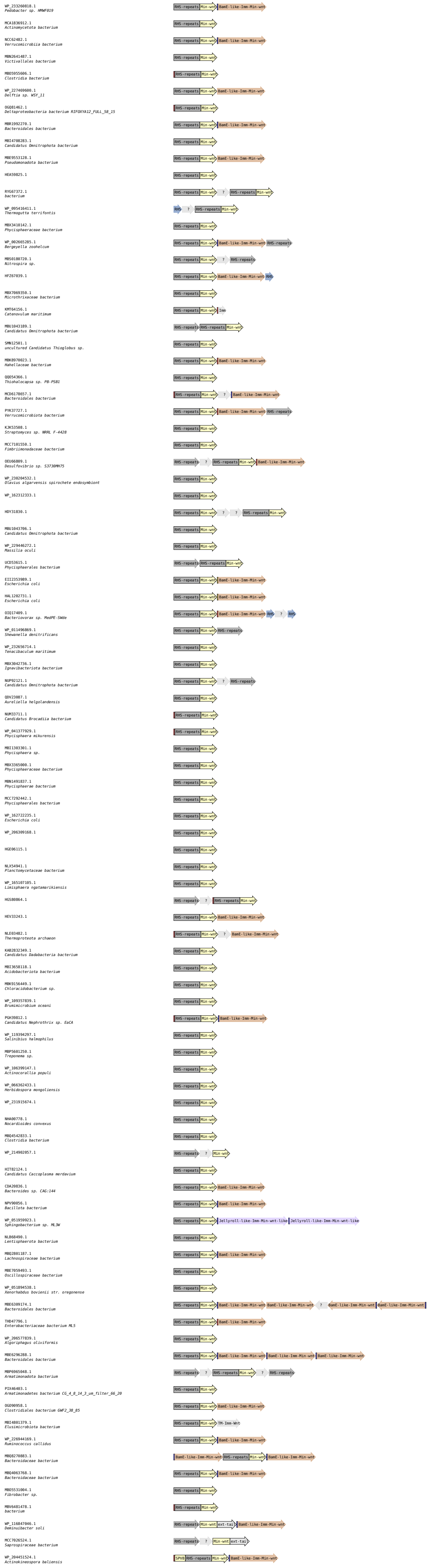
WP_233260818.1
Pedobacter sp. HMWF019
RHS-repeats Min-wnt BamE-like-Imm-Min-wnt
MCA1836912.1
Actinomycetota bacterium
RHS-repeats Min-wnt
NCC62482.1
Verrucomicrobiia bacterium
RHS-repeats Min-wnt BamE-like-Imm-Min-wnt
MBN2641487.1
Victivallales bacterium
RHS-repeats Min-wnt
MBO5955606.1
Clostridia bacterium
RHS-repeats Min-wnt
WP_227469600.1
Delftia sp. WSY_11
RHS-repeats Min-wnt BamE-like-Imm-Min-wnt
OGQ81462.1
Deltaproteobacteria bacterium RIFOXYA12_FULL_58_15
RHS-repeats Min-wnt
MBR1992270.1
Bacteroidales bacterium
RHS-repeats Min-wnt BamE-like-Imm-Min-wnt
MBI4708283.1
Candidatus Omnitrophota bacterium
RHS-repeats Min-wnt
MBE9553128.1
Pseudomonadota bacterium
RHS-repeats Min-wnt BamE-like-Imm-Min-wnt
HEA59825.1
RHS-repeats Min-wnt
RYG67372.1
bacterium
RHS-repeats Min-wnt ? RHS-repeats Min-wnt
WP_095416411.1
Thermogutta terrifontis
RHS ? RHS-repeats Min-wnt
MBX3410142.1
Phycisphaeraceae bacterium
RHS-repeats Min-wnt
WP_002665285.1
Bergeyella zoohelcum
RHS-repeats Min-wnt BamE-like-Imm-Min-wnt RHS-repeats
MBS0180720.1
Nitrospira sp.
RHS-repeats Min-wnt ? RHS-repeats
HFZ67039.1
RHS-repeats Min-wnt BamE-like-Imm-Min-wnt RHS
MBX7069350.1
Microthrixaceae bacterium
RHS-repeats Min-wnt
KMT64156.1
Catenovulum maritimum
RHS-repeats Min-wnt Imm
MBU1043189.1
Candidatus Omnitrophota bacterium
RHS-repeats RHS-repeats Min-wnt
SMN12501.1
uncultured Candidatus Thioglobus sp.
RHS-repeats Min-wnt
MBK8970023.1
Hahellaceae bacterium
RHS-repeats Min-wnt BamE-like-Imm-Min-wnt
QQO54366.1
Thiohalocapsa sp. PB-PSB1
RHS-repeats Min-wnt
MCD6178657.1
Bacteroidales bacterium
RHS-repeats Min-wnt ? BamE-like-Imm-Min-wnt
PYK37727.1
Verrucomicrobiota bacterium
RHS-repeats Min-wnt BamE-like-Imm-Min-wnt RHS-repeats
KJK53508.1
Streptomyces sp. NRRL F-4428
RHS-repeats Min-wnt
MCC7101550.1
Fimbriimonadaceae bacterium
RHS-repeats Min-wnt
OEU66809.1
Desulfovibrio sp. S3730MH75
RHS-repeats ? RHS-repeats Min-wnt BamE-like-Imm-Min-wnt
WP_230204532.1
Olavius algarvensis spirochete endosymbiont
RHS-repeats Min-wnt
WP_162312333.1
RHS-repeats Min-wnt
HDY31830.1
RHS-repeats Min-wnt ? ? RHS-repeats Min-wnt
MBU1043706.1
Candidatus Omnitrophota bacterium
RHS-repeats Min-wnt
WP_229446272.1
Massilia oculi
RHS-repeats Min-wnt
UCD53615.1
Phycisphaerales bacterium
RHS-repeats RHS-repeats Min-wnt
EII2353989.1
Escherichia coli
RHS-repeats Min-wnt BamE-like-Imm-Min-wnt
HAL1202731.1
Escherichia coli
RHS-repeats Min-wnt BamE-like-Imm-Min-wnt
OIQ17409.1
Bacteriovorax sp. MedPE-SWde
RHS-repeats Min-wnt BamE-like-Imm-Min-wnt RHS ? RHS
WP_011496869.1
Shewanella denitrificans
RHS-repeats Min-wnt RHS-repeats
WP_232656714.1
Tenacibaculum maritimum
RHS-repeats Min-wnt
MBX3042736.1
Ignavibacteriota bacterium
RHS-repeats Min-wnt
NUP92121.1
Candidatus Omnitrophota bacterium
RHS-repeats Min-wnt ? RHS-repeats
QDV23087.1
Aureliella helgolandensis
RHS-repeats Min-wnt
NUM33711.1
Candidatus Brocadiia bacterium
RHS-repeats Min-wnt
WP_041377929.1
Phycisphaera mikurensis
RHS-repeats Min-wnt
MBI1303301.1
Phycisphaera sp.
RHS-repeats Min-wnt
MBX3365000.1
Phycisphaeraceae bacterium
RHS-repeats Min-wnt
MBN1491837.1
Phycisphaerae bacterium
RHS-repeats Min-wnt
MCC7292442.1
Phycisphaerales bacterium
RHS-repeats Min-wnt
WP_162722235.1
Escherichia coli
RHS-repeats Min-wnt
WP_206309168.1
RHS-repeats Min-wnt
HGE06115.1
RHS-repeats Min-wnt
NLX54941.1
Planctomycetaceae bacterium
RHS-repeats Min-wnt
WP_165107105.1
Limisphaera ngatamarikiensis
RHS-repeats Min-wnt
HGS80864.1
RHS-repeats ? RHS-repeats Min-wnt
HEV33243.1
RHS-repeats Min-wnt BamE-like-Imm-Min-wnt
NLE03482.1
Thermoproteota archaeon
RHS-repeats Min-wnt ? BamE-like-Imm-Min-wnt
KAB2832349.1
Candidatus Dadabacteria bacterium
RHS-repeats Min-wnt
MBI3658118.1
Acidobacteriota bacterium
RHS-repeats Min-wnt
MBK9156449.1
Chloracidobacterium sp.
RHS-repeats Min-wnt
WP_109357839.1
Brumimicrobium oceani
RHS-repeats Min-wnt
PGH39812.1
Candidatus Nephrothrix sp. EaCA
RHS-repeats Min-wnt BamE-like-Imm-Min-wnt
WP_119394297.1
Salinibius halmophilus
RHS-repeats Min-wnt
MBP5601250.1
Treponema sp.
RHS-repeats Min-wnt
WP_106399147.1
Actinocorallia populi
RHS-repeats Min-wnt
WP_066362433.1
Herbidospora mongoliensis
RHS-repeats Min-wnt
WP_231915674.1
RHS-repeats Min-wnt
NHA00778.1
Nocardioides convexus
RHS-repeats Min-wnt
MBQ4542833.1
Clostridia bacterium
RHS-repeats Min-wnt
WP_214902057.1
RHS-repeats ? Min-wnt
HIT82124.1
Candidatus Caccoplasma merdavium
RHS-repeats Min-wnt
CDA20836.1
Bacteroides sp. CAG:144
RHS-repeats Min-wnt BamE-like-Imm-Min-wnt
NPV90056.1
Bacillota bacterium
RHS-repeats Min-wnt BamE-like-Imm-Min-wnt
WP_051959923.1
Sphingobacterium sp. ML3W
RHS-repeats Min-wnt Jellyroll-like-Imm-Min-wnt-like Jellyroll-like-Imm-Min-wnt-like
NLB68490.1
Lentisphaerota bacterium
RHS-repeats Min-wnt
MBQ2801187.1
Lachnospiraceae bacterium
RHS-repeats Min-wnt BamE-like-Imm-Min-wnt
MBE7059493.1
Oscillospiraceae bacterium
RHS-repeats Min-wnt
WP_051894538.1
Xenorhabdus bovienii str. oregonense
RHS-repeats Min-wnt
MBE6309174.1
Bacteroidales bacterium
RHS-repeats Min-wnt BamE-like-Imm-Min-wnt BamE-like-Imm-Min-wnt ? BamE-like-Imm-Min-wnt BamE-like-Imm-Min-wnt
THD47796.1
Enterobacteriaceae bacterium ML5
RHS-repeats Min-wnt BamE-like-Imm-Min-wnt
WP_206577839.1
Algoriphagus oliviformis
RHS-repeats Min-wnt
MBE6296288.1
Bacteroidales bacterium
RHS-repeats Min-wnt BamE-like-Imm-Min-wnt BamE-like-Imm-Min-wnt BamE-like-Imm-Min-wnt
MBP6965048.1
Armatimonadota bacterium
RHS-repeats ? RHS-repeats Min-wnt ? RHS-repeats
PIX46403.1
Armatimonadetes bacterium CG_4_8_14_3_um_filter_66_20
RHS-repeats Min-wnt
OGO90958.1
Clostridiales bacterium GWF2_38_85
RHS-repeats Min-wnt BamE-like-Imm-Min-wnt
MBI4801379.1
Elusimicrobiota bacterium
RHS-repeats Min-wnt TM-Imm-Wnt
WP_226944169.1
Ruminococcus callidus
RHS-repeats Min-wnt BamE-like-Imm-Min-wnt
MBQ8270883.1
Bacteroidaceae bacterium
BamE-like-Imm-Min-wnt RHS-repeats Min-wnt BamE-like-Imm-Min-wnt
MBQ4063768.1
Bacteroidaceae bacterium
RHS-repeats Min-wnt BamE-like-Imm-Min-wnt
MBO5531004.1
Fibrobacter sp.
RHS-repeats Min-wnt
MBV6481478.1
bacterium
RHS-repeats Min-wnt
WP_116847046.1
Deminuibacter soli
RHS-repeats Min-wnt ext-tail BamE-like-Imm-Min-wnt
MCC7026524.1
Saprospiraceae bacterium
RHS-repeats ? Min-wnt ext-tail
WP_204451524.1
Actinokineospora baliensis
SPVB RHS-repeats Min-wnt BamE-like-Imm-Min-wnt

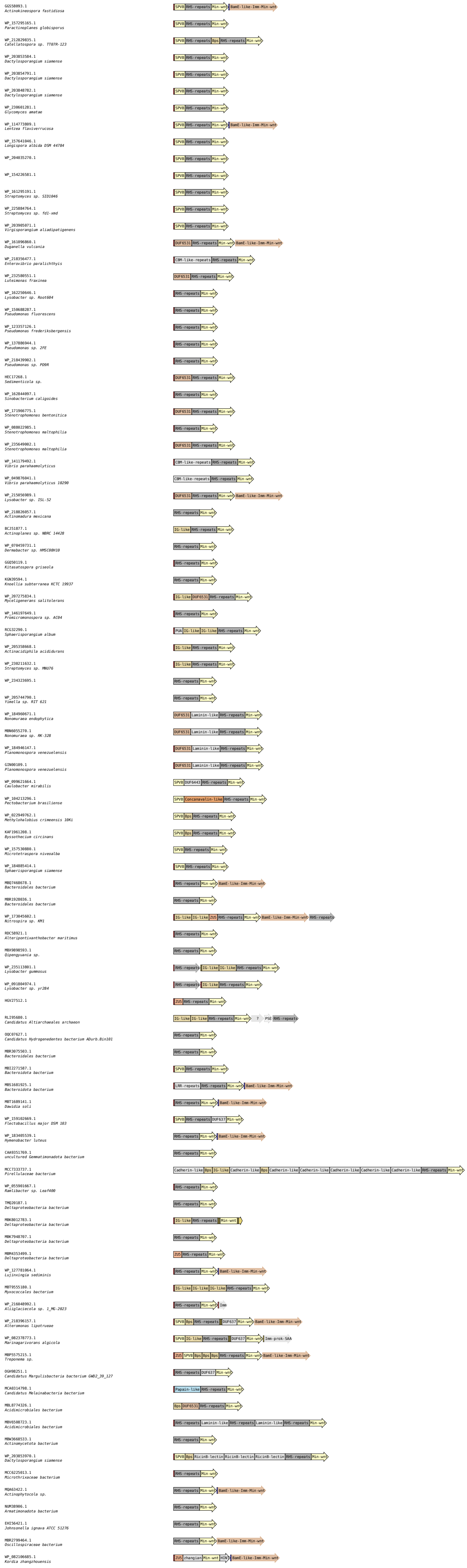
GGS58093.1
Actinokineospora fastidiosa
SPVB RHS-repeats Min-wnt BamE-like-Imm-Min-wnt
WP_157295165.1
Paractinoplanes globisporus
SPVB RHS-repeats Min-wnt
WP_212829835.1
Catellatospora sp. TT07R-123
SPVB RHS-repeats Bps RHS-repeats Min-wnt
WP_203853584.1
Dactylosporangium siamense
SPVB RHS-repeats Min-wnt
WP_203854791.1
Dactylosporangium siamense
SPVB RHS-repeats Min-wnt
WP_203848782.1
Dactylosporangium siamense
SPVB RHS-repeats Min-wnt
WP_230601281.1
Glycomyces amatae
SPVB RHS-repeats Min-wnt
WP_114773809.1
Lentzea flaviverrucosa
SPVB RHS-repeats Min-wnt BamE-like-Imm-Min-wnt
WP_157641046.1
Longispora albida DSM 44784
SPVB RHS-repeats Min-wnt
WP_204035270.1
SPVB RHS-repeats Min-wnt
WP_154226581.1
SPVB RHS-repeats Min-wnt
WP_161295191.1
Streptomyces sp. SID1046
SPVB RHS-repeats Min-wnt
WP_225884764.1
Streptomyces sp. fd1-xmd
SPVB RHS-repeats Min-wnt
WP_203905071.1
Virgisporangium aliadipatigenens
SPVB RHS-repeats Min-wnt
WP_161096860.1
Duganella vulcania
DUF6531 RHS-repeats Min-wnt BamE-like-Imm-Min-wnt
WP_218356477.1
Enterovibrio paralichthyis
CBM-like-repeats RHS-repeats Min-wnt
WP_232580551.1
Luteimonas fraxinea
DUF6531 RHS-repeats Min-wnt
WP_162250646.1
Lysobacter sp. Root604
RHS-repeats Min-wnt
WP_150688287.1
Pseudomonas fluorescens
RHS-repeats Min-wnt
WP_123357126.1
Pseudomonas frederiksbergensis
RHS-repeats Min-wnt
WP_137886944.1
Pseudomonas sp. 2FE
RHS-repeats Min-wnt
WP_218439902.1
Pseudomonas sp. PD9R
RHS-repeats Min-wnt
HEC17268.1
Sedimenticola sp.
DUF6531 RHS-repeats Min-wnt
WP_162844097.1
Sinobacterium caligoides
RHS-repeats Min-wnt
WP_171966775.1
Stenotrophomonas bentonitica
DUF6531 RHS-repeats Min-wnt
WP_088022985.1
Stenotrophomonas maltophilia
RHS-repeats Min-wnt
WP_235649002.1
Stenotrophomonas maltophilia
DUF6531 RHS-repeats Min-wnt
WP_141179492.1
Vibrio parahaemolyticus
CBM-like-repeats RHS-repeats Min-wnt
WP_049876041.1
Vibrio parahaemolyticus 10290
CBM-like-repeats RHS-repeats Min-wnt
WP_215056989.1
Lysobacter sp. ISL-52
DUF6531 RHS-repeats Min-wnt BamE-like-Imm-Min-wnt
WP_218826057.1
Actinomadura mexicana
RHS-repeats Min-wnt
BCJ51877.1
Actinoplanes sp. NBRC 14428
IG-like RHS-repeats Min-wnt
WP_070459731.1
Dermabacter sp. HMSC08H10
RHS-repeats Min-wnt
GGQ50119.1
Kitasatospora griseola
RHS-repeats Min-wnt
KGN39594.1
Knoellia subterranea KCTC 19937
RHS-repeats Min-wnt
WP_207275834.1
Myceligenerans salitolerans
IG-like DUF6531 RHS-repeats Min-wnt
WP_146197649.1
Promicromonospora sp. AC04
RHS-repeats Min-wnt
RCG32290.1
Sphaerisporangium album
PUA IG-like IG-like RHS-repeats Min-wnt
WP_205358668.1
Actinacidiphila acididurans
IG-like RHS-repeats Min-wnt
WP_230211632.1
Streptomyces sp. MNU76
IG-like RHS-repeats Min-wnt
WP_234323695.1
RHS-repeats Min-wnt
WP_205744790.1
Yimella sp. RIT 621
RHS-repeats Min-wnt
WP_184960671.1
Nonomuraea endophytica
DUF6531 Laminin-like RHS-repeats Min-wnt
MBN6055270.1
Nonomuraea sp. RK-328
DUF6531 Laminin-like RHS-repeats Min-wnt
WP_184946147.1
Planomonospora venezuelensis
DUF6531 Laminin-like RHS-repeats Min-wnt
GIN00109.1
Planomonospora venezuelensis
DUF6531 Laminin-like RHS-repeats Min-wnt
WP_099621664.1
Caulobacter mirabilis
SPVB DUF6443 RHS-repeats Min-wnt
WP_104213296.1
Pectobacterium brasiliense
SPVB Concanavalin-like RHS-repeats Min-wnt
WP_022949762.1
Methylohalobius crimeensis 10Ki
SPVB Bps RHS-repeats Min-wnt
KAF1961208.1
Byssothecium circinans
SPVB Bps RHS-repeats Min-wnt
WP_157530880.1
Microtetraspora niveoalba
SPVB RHS-repeats Min-wnt
WP_184885414.1
Sphaerisporangium siamense
SPVB RHS-repeats Min-wnt
MBQ7468678.1
Bacteroidales bacterium
RHS-repeats Min-wnt BamE-like-Imm-Min-wnt
MBR1928036.1
Bacteroidales bacterium
RHS-repeats Min-wnt
WP_173045602.1
Nitrospira sp. KM1
IG-like IG-like ZU5 RHS-repeats Min-wnt BamE-like-Imm-Min-wnt RHS-repeats
RDC58921.1
Alteripontixanthobacter maritimus
RHS-repeats Min-wnt
MBX9898593.1
Qipengyuania sp.
RHS-repeats Min-wnt
WP_235113801.1
Lysobacter gummosus
RHS-repeats IG-like IG-like RHS-repeats Min-wnt
WP_091804974.1
Lysobacter sp. yr284
RHS-repeats IG-like RHS-repeats Min-wnt
HGV27512.1
ZU5 RHS-repeats Min-wnt
RLI95680.1
Candidatus Altiarchaeales archaeon
IG-like IG-like RHS-repeats Min-wnt ? PSE RHS-repeats
OQC07627.1
Candidatus Hydrogenedentes bacterium ADurb.Bin101
RHS-repeats Min-wnt
MBR3075503.1
Bacteroidales bacterium
RHS-repeats Min-wnt
MBI2271587.1
Bacteroidota bacterium
SPVB RHS-repeats Min-wnt
MBS1681925.1
Bacteroidota bacterium
LRR-repeats RHS-repeats Min-wnt BamE-like-Imm-Min-wnt
MBT1689141.1
Dawidia soli
RHS-repeats Min-wnt BamE-like-Imm-Min-wnt
WP_159102669.1
Flectobacillus major DSM 103
SPVB RHS-repeats DUF637 Min-wnt
WP_183405539.1
Hymenobacter luteus
RHS-repeats Min-wnt BamE-like-Imm-Min-wnt
CAA9351769.1
uncultured Gemmatimonadota bacterium
RHS-repeats Min-wnt
MCC7333737.1
Pirellulaceae bacterium
Cadherin-like Bps IG-like Cadherin-like Bps Cadherin-like Cadherin-like Cadherin-like Cadherin-like Cadherin-like RHS-repeats Min-wnt
WP_055901667.1
Ramlibacter sp. Leaf400
RHS-repeats Min-wnt
TMQ20187.1
Deltaproteobacteria bacterium
RHS-repeats Min-wnt
MBK8012783.1
Deltaproteobacteria bacterium
IG-like RHS-repeats Min-wnt
MBK7948707.1
Deltaproteobacteria bacterium
RHS-repeats Min-wnt
MBM4353499.1
Deltaproteobacteria bacterium
ZU5 RHS-repeats Min-wnt
WP_127781064.1
Lujinxingia sediminis
RHS-repeats Min-wnt BamE-like-Imm-Min-wnt
MBT9555180.1
Myxococcales bacterium
IG-like IG-like IG-like RHS-repeats Min-wnt
WP_216048992.1
Aliiglaciecola sp. 1_MG-2023
RHS-repeats Min-wnt Imm
WP_218396157.1
Alteromonas lipotrueae
SPVB Bps RHS-repeats DUF637 Min-wnt BamE-like-Imm-Min-wnt
WP_082378773.1
Marinagarivorans algicola
SPVB IG-like RHS-repeats DUF637 Min-wnt Imm-prok-SAA
MBP5575215.1
Treponema sp.
ZU5 SPVB Bps Bps Bps RHS-repeats Min-wnt BamE-like-Imm-Min-wnt
OGH98251.1
Candidatus Margulisbacteria bacterium GWD2_39_127
RHS-repeats DUF637 Min-wnt
MCA0314798.1
Candidatus Melainabacteria bacterium
Papain-like RHS-repeats Min-wnt
MBL8774326.1
Acidimicrobiales bacterium
Bps DUF6531 RHS-repeats Min-wnt
MBV6508723.1
Acidimicrobiales bacterium
RHS-repeats Laminin-like RHS-repeats Laminin-like RHS-repeats Min-wnt
MBW3668533.1
Actinomycetota bacterium
RHS-repeats Min-wnt
WP_203853970.1
Dactylosporangium siamense
SPVB Bps RicinB-lectin RicinB-lectin RicinB-lectin RHS-repeats Min-wnt
MCC6225013.1
Microthrixaceae bacterium
RHS-repeats Min-wnt
MQA63422.1
Actinophytocola sp.
RHS-repeats Min-wnt BamE-like-Imm-Min-wnt
NUM38906.1
Armatimonadota bacterium
RHS-repeats Min-wnt
EHI56421.1
Johnsonella ignava ATCC 51276
RHS-repeats Min-wnt
MBR2799464.1
Oscillospiraceae bacterium
RHS-repeats Min-wnt BamE-like-Imm-Min-wnt
WP_082106685.1
Kordia zhangzhouensis
ZU5 zhangian Min-wnt HINT BamE-like-Imm-Min-wnt

EKD66724.1
groundwater metagenome
Min-wnt ? RHS-repeats

HEI00890.1
RHS-repeats Min-wnt

KAA3647500.1
Bacteroidota bacterium
RHS-repeats ? Min-wnt BamE-like-Imm-Min-wnt

STM07682.1
Escherichia coli
RHS-repeats Min-wnt BamE-like-Imm-Min-wnt

####;type VII secretion system polymorphic toxin systems with N-terminal WXG/LXG domain fusions

WP_157103763.1
Nocardia harenae NBRC 108248
WXG Min-wnt

WP_179814453.1
Allostreptomyces psammosilenae
WXG Min-wnt BamE-like-Imm-Min-wnt

WP_203911526.1
Rhizocola hellebori
WXG Min-wnt

WP_207918491.1
Saccharopolyspora karakumensis
WXG Min-wnt BamE-like-Imm-Min-wnt

AUS80094.1
Actinoalloteichus sp. AHMU CJ021
WXG Min-wnt

WP_081190183.1
Saccharomonospora piscinae
WXG Min-wnt

WP_234995790.1
Streptoalloteichus hindustanus
WXG Min-wnt BamE-like-Imm-Min-wnt

WP_203911990.1
Rhizocola hellebori
WXG Min-wnt BamE-like-Imm-Min-wnt

GIJ45295.1
Virgisporangium aliadipatigenens
WXG Min-wnt

MBR7677251.1
Streptomyces daliensis
WXG Min-wnt

WP_114016230.1
Streptomyces reniochalinae
WXG Min-wnt BamE-like-Imm-Min-wnt

NUT03391.1
Hamadaea sp.
WXG Min-wnt

WP_157102189.1
Nocardia caishijiensis
WXG WXG Min-wnt

WP_195002118.1
Nocardia higoensis
WXG WXG Min-wnt BamE-like-Imm-Min-wnt

WP_232000806.1
Mycobacterium kyorinense
WXG Min-wnt BamE-like-Imm-Min-wnt

WP_067832553.1
Nocardia lijiangensis NBRC 108240
WXG Min-wnt BamE-like-Imm-Min-wnt

WP_067466736.1
Nocardia amamiensis NBRC 102102
WXG Min-wnt BamE-like-Imm-Min-wnt

WP_140179370.1
Thermomonospora catenispora
WXG Min-wnt BamE-like-Imm-Min-wnt BamE-like-Imm-Min-wnt

WP_130344559.1
Herbihabitans rhizosphaerae
WXG WXG Min-wnt

WP_197013192.1
Actinomadura viridis
WXG Min-wnt BamE-like-Imm-Min-wnt BamE-like-Imm-Min-wnt

WP_051029087.1
Nocardia takedensis NBRC 100417
WXG Min-wnt BamE-like-Imm-Min-wnt

WP_070380418.1
Rhodococcus sp. WMMA185
WXG WXG Min-wnt BamE-like-Imm-Min-wnt

PKV77888.1
Nocardia fluminea
WXG Min-wnt BamE-like-Imm-Min-wnt

WP_180890876.1
Haloechinothrix aidingensis
WXG WXG Min-wnt BamE-like-Imm-Min-wnt

WP_156371039.1
Nocardia arizonensis
WXG WXG Min-wnt BamE-like-Imm-Min-wnt

WP_052314045.1
WXG WXG Min-wnt BamE-like-Imm-Min-wnt

WP_119727659.1
Thermomonospora amylolytica
WXG Min-wnt BamE-like-Imm-Min-wnt

WP_195024431.1
Nocardia farcinica
WXG WXG Min-wnt BamE-like-Imm-Min-wnt

WP_091455604.1
Actinokineospora iranica
WXG Min-wnt

WP_233000554.1
Nocardia asteroides
WXG WXG Min-wnt

WP_218020367.1
Nocardia sienata NBRC 100364
WXG Min-wnt BamE-like-Imm-Min-wnt

WP_146073748.1
Amycolatopsis sp. CA-126428
WXG Min-wnt

WP_216216056.1
Amycolatopsis aidingensis
WXG WXG Min-wnt BamE-like-Imm-Min-wnt

WP_216915007.1
Nocardia noduli
WXG Min-wnt BamE-like-Imm-Min-wnt

NUS43350.1
Mycobacteriaceae bacterium
WXG Min-wnt BamE-like-Imm-Min-wnt

WP_122188591.1
Nocardia stercoris
WXG Min-wnt BamE-like-Imm-Min-wnt BamE-like-Imm-Min-wnt

WP_156959396.1
Nocardia sp. BMG51109
WXG WXG Min-wnt BamE-like-Imm-Min-wnt

WP_138447736.1
Nocardia cyriacigeorgica
WXG Min-wnt BamE-like-Imm-Min-wnt

WP_051132969.1
Nocardia paucivorans NBRC 100373
WXG Min-wnt BamE-like-Imm-Min-wnt

APU15842.1
Actinoalloteichus fjordicus
WXG WXG Min-wnt BamE-like-Imm-Min-wnt

WP_185003819.1
Crossiella cryophila
WXG Min-wnt

WP_134712939.1
Saccharomonospora xinjiangensis
WXG WXG Min-wnt BamE-like-Imm-Min-wnt

WP_130343793.1
Herbihabitans rhizosphaerae
WXG WXG Min-wnt

WP_206394712.1
Pseudoclavibacter albus
WXG Min-wnt BamE-like-Imm-Min-wnt

WP_235052488.1
Yinghuangia soli
WXG Min-wnt BamE-like-Imm-Min-wnt

WP_218469396.1
Nocardia iowensis
WXG WXG Min-wnt

MCC6976088.1
Anaerolineae bacterium
WXG Min-wnt BamE-like-Imm-Min-wnt

WP_207274673.1
Myceligenerans salitolerans
WXG Min-wnt BamE-like-Imm-Min-wnt

WP_157225021.1
Nocardia thailandica NBRC 100428
WXG Min-wnt

WP_051421718.1
Arthrobacter sp. StoSoilB20
WXG Min-wnt BamE-like-Imm-Min-wnt

MCA9892283.1
Anaerolineae bacterium
WXG Min-wnt

MBK8026766.1
Candidatus Flexifilum affinis
WXG Min-wnt BamE-like-Imm-Min-wnt

WP_076465438.1
Actinomyces mediterranea
WXG Min-wnt BamE-like-Imm-Min-wnt

PJF31449.1
Candidatus Thermofonsia Clade 1 bacterium
WXG Min-wnt BamE-like-Imm-Min-wnt

WP_136017848.1
Arthrobacter silvisoli
WXG Min-wnt BamE-like-Imm-Min-wnt

WP_074587386.1
Arthrobacter cupressi
WXG Min-wnt BamE-like-Imm-Min-wnt

WP_116115352.1
Amycolatopsis thermalba
WXG Min-wnt

WP_206807945.1
Amycolatopsis sp. 195334CR
WXG Min-wnt

WP_110341740.1
Prauserella flavalba
WXG Min-wnt

WP_213170837.1
Natronoglycomyces albus
WXG Min-wnt

WP_044520534.1
Mycolicibacterium septicum DSM 44393
WXG Min-wnt BamE-like-Imm-Min-wnt

WP_171508626.1
Mycolicibacterium fortuitum
WXG Min-wnt

OBI55709.1
Mycolicibacterium fortuitum
WXG Min-wnt

WP_163679194.1
Mycolicibacterium poriferae
WXG Min-wnt BamE-like-Imm-Min-wnt

WP_064931687.1
Mycolicibacterium peregrinum
WXG Min-wnt

WP_003925047.1
Mycolicibacterium thermoresistibile
WXG Min-wnt BamE-like-Imm-Min-wnt BamE-like-Imm-Min-wnt

WP_163795138.1
WXG Min-wnt BamE-like-Imm-Min-wnt

WP_200845826.1
Mycolicibacterium vanbaalenii
WXG Min-wnt BamE-like-Imm-Min-wnt

WP_211648758.1
Leucobacter manosquensis
WXG Min-wnt BamE-like-Imm-Min-wnt BamE-like-Imm-Min-wnt

WP_051177949.1
Nocardia concava NBRC 100430
WXG WXG Min-wnt BamE-like-Imm-Min-wnt

WP_020555793.1
Embleya scabrispora DSM 41855
WXG Min-wnt BamE-like-Imm-Min-wnt

WP_053717361.1
Saccharothrix sp. NRRL B-16348
WXG WXG Min-wnt

WP_203867946.1
Plantactinospora endophytica
WXG Min-wnt

WP_063047311.1
Nocardia pseudovaccinii NBRC 100343
WXG WXG Min-wnt BamE-like-Imm-Min-wnt

WP_181856896.1
Nocardia farcinica
WXG WXG Min-wnt BamE-like-Imm-Min-wnt BamE-like-Imm-Min-wnt

WP_213559489.1
Nocardia tengchongensis
WXG WXG Min-wnt BamE-like-Imm-Min-wnt

WP_115411048.1
Nocardia farcinica
WXG WXG Min-wnt BamE-like-Imm-Min-wnt

MBU0576964.1
Patescibacteria group bacterium
WXG Min-wnt

WP_195134064.1
Nocardia amamiensis
WXG WXG Min-wnt BamE-like-Imm-Min-wnt

WP_235039099.1
Kibdelosporangium aridum
WXG WXG Min-wnt BamE-like-Imm-Min-wnt

WP_063047354.1
Nocardia pseudovaccinii NBRC 100343
WXG WXG Min-wnt BamE-like-Imm-Min-wnt

WP_233036551.1
Nocardia yamanashiensis
WXG WXG Min-wnt BamE-like-Imm-Min-wnt

WP_109529926.1
Nocardia sp.
WXG WXG Min-wnt BamE-like-Imm-Min-wnt

WP_052373280.1
Nocardia otitidiscaviarum
WXG WXG Min-wnt BamE-like-Imm-Min-wnt

WP_218471438.1
Nocardia iowensis
WXG WXG Min-wnt BamE-like-Imm-Min-wnt ? ? ? ? AraC-HTH AlcRN

WP_074849037.1
Gordonia westfalica
WXG WXG Min-wnt BamE-like-Imm-Min-wnt

MPY99544.1
Actinophytocola sp.
WXG WXG Min-wnt BamE-like-Imm-Min-wnt

WP_159843286.1
Nocardia sp. CY41
WXG WXG Min-wnt BamE-like-Imm-Min-wnt

WP_124707186.1
Gordonia insulae
WXG Min-wnt BamE-like-Imm-Min-wnt

WP_051406773.1
Nocardia sp. CNY236
WXG WXG Min-wnt BamE-like-Imm-Min-wnt

WP_067647185.1
Nocardia harenae NBRC 108248
WXG WXG Min-wnt BamE-like-Imm-Min-wnt

QKT06966.1
Gordonia sp. X0973
WXG Min-wnt BamE-like-Imm-Min-wnt BamE-like-Imm-Min-wnt

WP_157111460.1
Nocardia beijingensis NBRC 16342
WXG WXG Min-wnt BamE-like-Imm-Min-wnt

WP_195119951.1
Nocardia otitidiscaviarum
WXG WXG Min-wnt BamE-like-Imm-Min-wnt BamE-like-Imm-Min-wnt

WP_189033575.1
Nocardia rhizosphaerihabitans
WXG WXG Min-wnt BamE-like-Imm-Min-wnt

WP_157226614.1
Gordonia soli NBRC 108243
WXG Min-wnt

WP_106178393.1
Prauserella shujinwangii
WXG WXG Min-wnt BamE-like-Imm-Min-wnt

WP_069166072.1
Nocardia altamirensis NBRC 108246
WXG WXG Min-wnt BamE-like-Imm-Min-wnt

WP_218722054.1
Nocardia sp. MH4
WXG WXG Min-wnt

WP_204409836.1
Brachybacterium muris
WXG Min-wnt BamE-like-Imm-Min-wnt

WP_110078329.1
Actinokineospora spheciospongiae
WXG WXG Min-wnt BamE-like-Imm-Min-wnt

WP_159425245.1
Streptomyces sp. TLI_053
WXG Min-wnt BamE-like-Imm-Min-wnt

WP_015803454.1
Actinosynnema mirum DSM 43827
WXG WXG Min-wnt BamE-like-Imm-Min-wnt

WP_090007105.1
Lentzea albidocapillata subsp. violacea
WXG WXG Min-wnt

WP_146007714.1
Brachybacterium sp. Marseille-Q7125
WXG Min-wnt BamE-like-Imm-Min-wnt

WP_143875944.1
Nocardia fluminea
WXG Min-wnt BamE-like-Imm-Min-wnt

HJF51167.1
Brachybacterium paraconglomeratum
WXG Min-wnt BamE-like-Imm-Min-wnt

MBK8756994.1
Actinomycetales bacterium
WXG Min-wnt BamE-like-Imm-Min-wnt

WP_191297447.1
Lentzea cavernae
WXG Min-wnt

WP_139115556.1
Gordonia hongkongensis
WXG Min-wnt BamE-like-Imm-Min-wnt

WP_073824647.1
Buchananella hordeovulneris
WXG Min-wnt BamE-like-Imm-Min-wnt

WP_195154625.1
Nocardia abscessus
WXG WXG Min-wnt

WP_067534759.1
Nocardia crassostreae
WXG WXG Min-wnt Min-wnt BamE-like-Imm-Min-wnt

WP_156094708.1
Nocardia lijiangensis NBRC 108240
WXG Min-wnt

WP_195152655.1
Nocardia abscessus
WXG Min-wnt BamE-like-Imm-Min-wnt

WP_137808802.1
Gordonia sp. ALPHA1B1
WXG Min-wnt BamE-like-Imm-Min-wnt

NLE78355.1
Rhodococcus sp. (in: high G+C Gram-positive bacteria)
WXG Min-wnt BamE-like-Imm-Min-wnt BamE-like-Imm-Min-wnt

WP_063127685.1
Nocardia fusca NBRC 14340
WXG Min-wnt

WP_157112674.1
Nocardia speluncae
WXG Min-wnt

####;additional polymophic toxin-like system configurations; #;PT-VENN-containing

RPD86152.1
Neisseria weixii
DUF637 PT-VENN Min-wnt BamE-like-Imm-Min-wnt

WP_123796433.1
Neisseria animalis
PT-VENN Min-wnt BamE-like-Imm-Min-wnt

WP_228776616.1
PT-VENN Min-wnt BamE-like-Imm-Min-wnt

WP_170161440.1
Moraxella cuniculi
PT-VENN Min-wnt

WP_019671796.1
Psychrobacter lutiphocae
PT-VENN Min-wnt

WP_228259286.1
FilH FilG PT-VENN Min-wnt BamE-like-Imm-Min-wnt

#;halocin-C858-N-containing

RHS59109.1
Ruminococcus sp. AM45-9BH
halocin-C858-N Min-wnt

WP_043940202.1
Bacillus subtilis
halocin-C858-N Min-wnt

WP_212129129.1
Bacillus subtilis
halocin-C858-N Min-wnt

#;LGFP repeats-containing

WP_141720764.1
Streptomyces sp. Ncost-T10-10d
LGFP-repeats Min-wnt

WP_052844342.1
Corynebacterium testudinoris
LGFP-repeats Min-wnt

EOM75571.1
Rhodococcus rhodnii LMG 5362
LGFP-repeats Min-wnt

WP_185714199.1
LGFP-repeats Min-wnt

WP_231598733.1
Corynebacterium occultum
LGFP-repeats Min-wnt

WP_228287245.1
Rhodococcus ruber
LGFP-repeats Min-wnt

WP_110038839.1
Nocardia neocaledoniensis NBRC 108232
LGFP-repeats Min-wnt

ETK36859.1
Microbispora sp. ATCC PTA-5024
Min-wnt LGFP-repeats

#;DUF4280-containing

EIP3948310.1
Salmonella enterica
DUF4280 Min-wnt BamE-like-Imm-Min-wnt

WP_218523072.1
Rahnella sp. PD12R
DUF4280 Min-wnt BamE-like-Imm-Min-wnt

#;PGBD-containing

MBT5109073.1
Rhodospirillaceae bacterium
PGBD Min-wnt

MBL6935944.1
Alphaproteobacteria bacterium
PGBD Min-wnt

MBT5049772.1
Rhodospirillaceae bacterium
PGBD Min-wnt

MCB1720465.1
Alphaproteobacteria bacterium
PGBD Min-wnt

HIF25050.1
Micavibrio sp.
PGBD Min-wnt

MBC7953998.1
Rhodospirillales bacterium
PGBD Min-wnt

WP_092620848.1
Roseospirillum parvum
PGBD Min-wnt

#;X-Tfes_XVIPCD-containing (Type IV secretion system)

MCC5104118.1
Min-wnt X-Tfes_XVIPCD

MCC7249747.1
Lysobacter sp.
BamE-like-Imm-Min-wnt Min-wnt X-Tfes_XVIPCD

WP_052812208.1
Xanthomonas campestris
BamE-like-Imm-Min-wnt Min-wnt X-Tfes_XVIPCD

WP_147308488.1
Xanthomonas nasturtii
BamE-like-Imm-Min-wnt Min-wnt X-Tfes_XVIPCD

TXI46032.1
Lysobacter sp.
BamE-like-Imm-Min-wnt Min-wnt X-Tfes_XVIPCD

WP_215839101.1
Lysobacter sp. K5869
Min-wnt X-Tfes_XVIPCD

MCB2018383.1
Hydrogenophaga sp.
BamE-like-Imm-Min-wnt Min-wnt X-Tfes_XVIPCD

WP_036492364.1
Neisseria mucosa
Min-wnt X-Tfes_XVIPCD

NUO76909.1
Lysobacter sp.
BamE-like-Imm-Min-wnt Min-wnt X-Tfes_XVIPCD

MBW8810997.1
Lysobacter sp.
BamE-like-Imm-Min-wnt Min-wnt X-Tfes_XVIPCD

MBN8213630.1
Lysobacterales bacterium
BamE-like-Imm-Min-wnt Min-wnt X-Tfes_XVIPCD

MBW8849268.1
Lysobacterales bacterium
BamE-like-Imm-Min-wnt Min-wnt X-Tfes_XVIPCD

MBX3713666.1
Lysobacter sp.
BamE-like-Imm-Min-wnt Min-wnt X-Tfes_XVIPCD

#;DUF4157-type MPTase-containing

WP_214570603.1
Dyella sp. LX-1
DUF4157-MPTase Min-wnt

NIM16380.1
Candidatus Aminicenantes bacterium
DUF4157-MPTase Min-wnt

MCC7564815.1
Methanomicrobiaceae archaeon
DUF4157-MPTase Min-wnt

WP_212670510.1
DUF4157-MPTase Min-wnt

WP_090470862.1
Mucilaginibacter sp. OK268
DUF4157-MPTase Min-wnt

MBO7506063.1
Paludibacteraceae bacterium
DUF4157-MPTase Min-wnt

#;TPR-like-repeats-containing+Min-wnt

WP_047961772.1
Xenorhabdus khoisanae
TPR-like-repeats Min-wnt BamE-like-Imm-Min-wnt

ARD69870.1
TPR-like-repeats Min-wnt BamE-like-Imm-Min-wnt

WP_092511313.1
Xenorhabdus mauleonii
TPR-like-repeats Min-wnt BamE-like-Imm-Min-wnt

WP_214039782.1
Photorhabdus akhurstii
TPR-like-repeats Min-wnt BamE-like-Imm-Min-wnt

#;Beta-roll-repeat-containing

WP_151040627.1
Neorhizobium galegae
BamE-like-Imm-Min-wnt Min-wnt Beta-Roll-repeats

TNE40589.1
Sphingomonadales bacterium
Min-wnt Beta-Roll-repeats

WP_136347764.1
Pseudothauera nasutitermitis
Min-wnt Beta-Roll-repeats

RUR71954.1
Variovorax guangxiensis
Min-wnt Beta-Roll-repeats

WP_095193968.1
Pseudomonas sp. Irchel 3A7
BamE-like-Imm-Min-wnt Min-wnt Beta-Roll-repeats

WP_194957643.1
Xylella fastidiosa subsp. multiplex
Min-wnt Beta-Roll-repeats

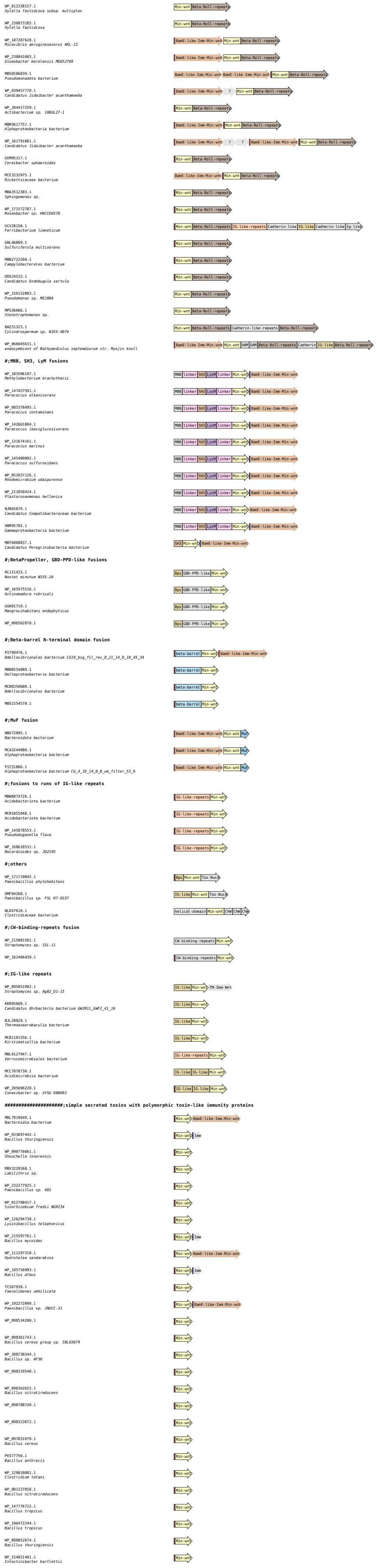

WP_012338157.1
Xylella fastidiosa subsp. multiplex
Min-wnt Beta-Roll-repeats
WP_230873185.1
Xylella fastidiosa
Min-wnt Beta-Roll-repeats
WP_187287628.1
Micavibrio aeruginosavorus ARL-13
BamE-like-Imm-Min-wnt Min-wnt Beta-Roll-repeats
WP_230841665.1
Gloeobacter morelensis MG652769
BamE-like-Imm-Min-wnt Min-wnt Beta-Roll-repeats
MBS0586839.1
Pseudomonadota bacterium
BamE-like-Imm-Min-wnt BamE-like-Imm-Min-wnt Min-wnt Beta-Roll-repeats
WP_039457770.1
Candidatus Jidaibacter acanthamoeba
BamE-like-Imm-Min-wnt ? Min-wnt Beta-Roll-repeats
WP_204417259.1
Actibacterium sp. 188UL27-1
Min-wnt Beta-Roll-repeats
MBM3617757.1
Alphaproteobacteria bacterium
BamE-like-Imm-Min-wnt Min-wnt Beta-Roll-repeats
WP_161791861.1
Candidatus Jidaibacter acanthamoeba
BamE-like-Imm-Min-wnt ? ? BamE-like-Imm-Min-wnt Min-wnt Beta-Roll-repeats
GEM95317.1
Cereibacter sphaeroides
Min-wnt Beta-Roll-repeats
MCE3232975.1
Rickettsiaceae bacterium
BamE-like-Imm-Min-wnt Min-wnt Beta-Roll-repeats
MBA3512303.1
Sphingomonas sp.
Min-wnt Beta-Roll-repeats
WP_171572787.1
Roseobacter sp. HKCCD6578
Min-wnt Beta-Roll-repeats
UCV28156.1
Ferribacterium limneticum
Min-wnt Beta-Roll-repeats IG-like-repeats Cadherin-like IG-like Cadherin-like Ig-like
GBL46869.1
Sulfuriferula multivorans
Min-wnt Beta-Roll-repeats
MBN2722260.1
Campylobacterales bacterium
Min-wnt Beta-Roll-repeats
ODS24532.1
Candidatus Endobugula sertula
Min-wnt Beta-Roll-repeats
WP_156152803.1
Pseudomonas sp. MEJ086
Min-wnt Beta-Roll-repeats
MPS36066.1
Stenotrophomonas sp.
Min-wnt Beta-Roll-repeats
BAZ31323.1
Cylindrospermum sp. NIES-4074
Min-wnt Beta-Roll-repeats Cadherin-like-repeats Beta-Roll-repeats
WP_066045631.1
endosymbiont of Bathymodiolus septemdierum str. Myojin knoll
BamE-like-Imm-Min-wnt Min-wnt DAM DAM Beta-Roll-repeats Cadherin IG-like Beta-Roll-repeats
#;MBB, SH3, LyM fusions
WP_183506197.1
Methylobacterium brachythecii
MBB linker SH3 LysM linker Min-wnt BamE-like-Imm-Min-wnt
WP_147457591.1
Paracoccus alkanivorans
MBB linker SH3 LysM linker Min-wnt BamE-like-Imm-Min-wnt
WP_085378495.1
Paracoccus contaminans
MBB linker SH3 LysM linker Min-wnt BamE-like-Imm-Min-wnt
WP_142661804.1
Paracoccus laeviglucosivorans
MBB linker SH3 LysM linker Min-wnt BamE-like-Imm-Min-wnt
WP_131674161.1
Paracoccus marinus
MBB linker SH3 LysM linker Min-wnt BamE-like-Imm-Min-wnt
WP_145400092.1
Paracoccus sulfuroxidans
MBB linker SH3 LysM linker Min-wnt BamE-like-Imm-Min-wnt
WP_052037126.1
Rhodomicrobium udaipurense
MBB linker SH3 LysM linker Min-wnt BamE-like-Imm-Min-wnt
WP_211850424.1
Plastoroseomonas hellenica
MBB linker SH3 LysM linker Min-wnt BamE-like-Imm-Min-wnt
NJN45870.1
Candidatus Competibacteraceae bacterium
MBB linker SH3 LysM linker Min-wnt BamE-like-Imm-Min-wnt
HBR95785.1
Gammaproteobacteria bacterium
MBB linker SH3 LysM linker Min-wnt BamE-like-Imm-Min-wnt
MBT6068837.1
Candidatus Peregrinibacteria bacterium
SH3 Min-wnt BamE-like-Imm-Min-wnt
#;BetaPropeller, GBD-PPD-like fusions
RCJ21433.1
Nostoc minutum NIES-26
Bps GBD-PPD-like Min-wnt
WP_165975516.1
Actinomadura rubrisoli
Bps GBD-PPD-like Min-wnt
GGK81719.1
Mangrovihabitans endophyticus
Bps GBD-PPD-like Min-wnt
WP_096562870.1
Bps GBD-PPD-like Min-wnt
#;Beta-barrel N-terminal domain fusion
PIT99476.1
Bdellovibrionales bacterium CG10_big_fil_rev_8_21_14_0_10_45_34
beta-barrel Min-wnt BamE-like-Imm-Min-wnt
MBN8554089.1
Deltaproteobacteria bacterium
beta-barrel Min-wnt
MCB0350680.1
Bdellovibrionales bacterium
beta-barrel Min-wnt
MBS1554570.1
beta-barrel Min-wnt
#;MuF fusion
NBU72095.1
Bacteroidota bacterium
BamE-like-Imm-Min-wnt Min-wnt MuF
MCA3244080.1
Alphaproteobacteria bacterium
BamE-like-Imm-Min-wnt Min-wnt MuF
PIZ31866.1
Alphaproteobacteria bacterium CG_4_10_14_0_8_um_filter_53_9
BamE-like-Imm-Min-wnt Min-wnt MuF
#;fusions to runs of IG-like repeats
MBW8874726.1
Acidobacteriota bacterium
IG-like-repeats Min-wnt
MCB1055048.1
Acidobacteriota bacterium
IG-like-repeats Min-wnt
WP_145878553.1
Pseudoduganella flava
IG-like-repeats Min-wnt
WP_168618531.1
Nocardioides sp. JQ2195
IG-like-repeats Min-wnt
#;others
WP_171719045.1
Paenibacillus phytohabitans
Bps Min-wnt Tox-NucA
OMF94260.1
Paenibacillus sp. FSL R7-0337
IG-like Min-wnt Tox-NucA
NLD47610.1
Clostridiaceae bacterium
helical-domain Min-wnt ChW ChW ChW
#;CW-binding-repeats fusion
WP_215081501.1
Streptomyces sp. ISL-11
CW-binding-repeats Min-wnt
WP_162466439.1
CW-binding-repeats Min-wnt
#;IG-like repeats
WP_095851992.1
Streptomyces sp. Ag82_O1-15
IG-like Min-wnt TM-Imm-Wnt
KKR95609.1
Candidatus Uhrbacteria bacterium GW2011_GWF2_41_16
IG-like Min-wnt
NJL28929.1
Thermoanaerobaculia bacterium
IG-like Min-wnt
MCB1101556.1
Kiritimatiellia bacterium
IG-like Min-wnt
MBL9127947.1
Verrucomicrobiales bacterium
IG-like-repeats Min-wnt
MCC7078730.1
Acidimicrobiia bacterium
IG-like IG-like Min-wnt
WP_205696228.1
Conexibacter sp. SYSU D00693
IG-like IG-like Min-wnt
####################;simple secreted toxins with polymorphic toxin-like immunity proteins
MBL7919649.1
Bacteroidia bacterium
Min-wnt BamE-like-Imm-Min-wnt
WP_033697442.1
Bacillus thuringiensis
Min-wnt Imm
WP_090776661.1
Shouchella lonarensis
Min-wnt
MBX3229568.1
Labilithrix sp.
Min-wnt
WP_232277925.1
Paenibacillus sp. 481
Min-wnt
WP_012708417.1
Sinorhizobium fredii NGR234
Min-wnt
WP_126294739.1
Lysinibacillus telephonicus
Min-wnt
WP_215597761.1
Bacillus mycoides
Min-wnt Imm
WP_111297310.1
Hydrotalea sandarakina
Min-wnt BamE-like-Imm-Min-wnt
WP_195756993.1
Bacillus albus
Min-wnt Imm
TCS67938.1
Faecalimonas umbilicata
Min-wnt
WP_192272099.1
Paenibacillus sp. JNUCC-31
Min-wnt BamE-like-Imm-Min-wnt
WP_098534280.1
Min-wnt
WP_098361743.1
Bacillus cereus group sp. IBL03679
Min-wnt
WP_208730344.1
Bacillus sp. AF56
Min-wnt
WP_098319548.1
Min-wnt
WP_098362622.1
Bacillus nitratireducens
Min-wnt
WP_098788749.1
Min-wnt
WP_098323672.1
Min-wnt
WP_097831079.1
Bacillus cereus
Min-wnt
PES77756.1
Bacillus anthracis
Min-wnt
WP_129010081.1
Clostridium tetani
Min-wnt
WP_001227858.1
Bacillus nitratireducens
Min-wnt
WP_147776722.1
Bacillus tropicus
Min-wnt
WP_196472244.1
Bacillus tropicus
Min-wnt
WP_098852674.1
Bacillus thuringiensis
Min-wnt
WP_154821401.1
Intestinibacter bartlettii
Min-wnt

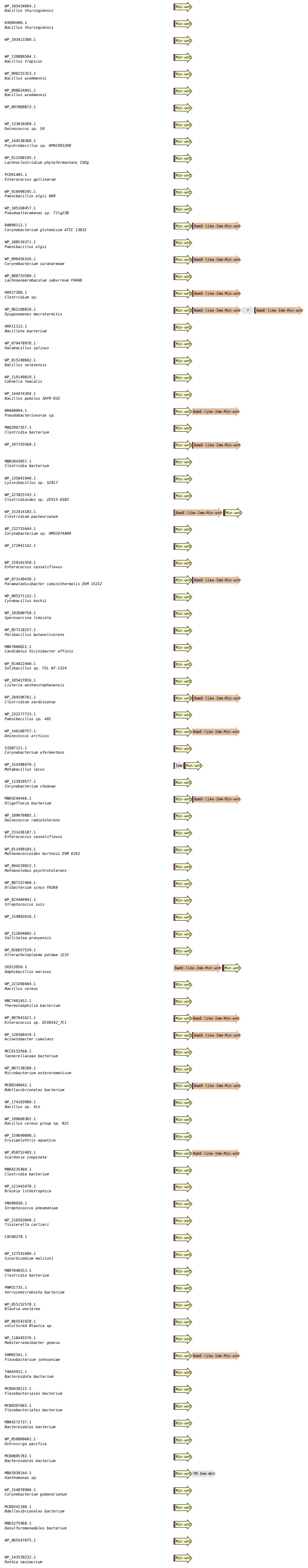
WP_105434094.1
Bacillus thuringiensis
Min-wnt
KXO04400.1
Bacillus thuringiensis
Min-wnt
WP_193413389.1
Min-wnt
WP_139889504.1
Bacillus tropicus
Min-wnt
WP_098155353.1
Bacillus wiedmannii
Min-wnt
WP_098624841.1
Bacillus wiedmannii
Min-wnt
WP_097889873.1
Min-wnt
WP_133010209.1
Deinococcus sp. S9
Min-wnt
WP_144538360.1
Psychrobacillus sp. NPDC093200
Min-wnt
WP_012200195.1
Lachnoclostridium phytofermentans ISDg
Min-wnt
PCD91405.1
Enterococcus gallinarum
Min-wnt
WP_010499245.1
Paenibacillus elgii B69
Min-wnt
WP_105168457.1
Pseudoalteromonas sp. T1lg23B
Min-wnt
BAB99112.1
Corynebacterium glutamicum ATCC 13032
Min-wnt
BamE-like-Imm-Min-wnt
WP_108534371.1
Paenibacillus elgii
Min-wnt
WP_096456326.1
Corynebacterium suranareeae
Min-wnt
BamE-like-Imm-Min-wnt
WP_008753589.1
Lachnoanaerobaculum saburreum F0468
Min-wnt
HHX17286.1
Clostridium sp.
Min-wnt
BamE-like-Imm-Min-wnt
WP_062180810.1
Dysgonomonas macrotermitis
Min-wnt
BamE-like-Imm-Min-wnt
?
BamE-like-Imm-Min-wnt
HHX11112.1
Bacillota bacterium
Min-wnt
WP_079478978.1
Halobacillus salinus
Min-wnt
WP_015240662.1
Bacillus velezensis
Min-wnt
WP_119149819.1
Cohnella faecalis
Min-wnt
WP_144474394.1
Bacillus pumilus SAFR-032
Min-wnt
NRA68094.1
Pseudobacteriovorax sp.
Min-wnt
BamE-like-Imm-Min-wnt
MBQ2967357.1
Clostridia bacterium
Min-wnt
WP_197793560.1
Min-wnt
BamE-like-Imm-Min-wnt
MBR3943057.1
Clostridia bacterium
Min-wnt
WP_135841940.1
Lysinibacillus sp. S2017
Min-wnt
WP_227825743.1
Clostridioides sp. ZZV15-6383
Min-wnt
WP_152414183.1
Clostridium pasteurianum
BamE-like-Imm-Min-wnt
Min-wnt
WP_232751644.1
Corynebacterium sp. HMSC074A09
Min-wnt
WP_172941142.1
Min-wnt
WP_159161550.1
Enterococcus casseliflavus
Min-wnt
WP_073149430.1
Paramaledivibacter caminithermalis DSM 15212
Min-wnt
BamE-like-Imm-Min-wnt
WP_095371132.1
Cytobacillus kochii
Min-wnt
WP_192600750.1
Sporosarcina limicola
Min-wnt
WP_057218257.1
Peribacillus butanolivorans
Min-wnt
MBK7800022.1
Candidatus Vicinibacter affinis
Min-wnt
WP_014822440.1
Solibacillus sp. FSL W7-1324
Min-wnt
WP_185427656.1
Listeria weihenstephanensis
Min-wnt
WP_204596761.1
Clostridium sardiniense
Min-wnt
BamE-like-Imm-Min-wnt
WP_232277733.1
Paenibacillus sp. 481
Min-wnt
WP_146160757.1
Deinococcus arcticus
Min-wnt
BamE-like-Imm-Min-wnt
SIQ07121.1
Corynebacterium afermentans
Min-wnt
WP_154308479.1
Metabacillus lacus
Imm
Min-wnt
WP_123929577.1
Corynebacterium choanae
Min-wnt
MBK9294446.1
Oligoflexia bacterium
Min-wnt
BamE-like-Imm-Min-wnt
WP_189070885.1
Deinococcus radiotolerans
Min-wnt
WP_231436187.1
Enterococcus casseliflavus
Min-wnt
WP_011499184.1
Methanococcoides burtonii DSM 6242
Min-wnt
WP_094229022.1
Methanolobus psychrotolerans
Min-wnt
WP_007157460.1
Oribacterium sinus F0268
Min-wnt
WP_024404941.1
Streptococcus suis
Min-wnt
WP_154892616.1
Min-wnt
WP_212694002.1
Vallitalea pronyensis
Min-wnt
WP_026657529.1
Alteracholeplasma palmae J233
Min-wnt
SEO13050.1
Amphibacillus marinus
BamE-like-Imm-Min-wnt
Min-wnt
WP_223260404.1
Bacillus cereus
Min-wnt
MBC7461452.1
Thermoleophilia bacterium
Min-wnt
WP_087641621.1
Enterococcus sp. DIV0242_7C1
Min-wnt
BamE-like-Imm-Min-wnt
WP_120368419.1
Acinetobacter cumulans
Min-wnt
BamE-like-Imm-Min-wnt
MCC8153568.1
Tannerellaceae bacterium
Min-wnt
WP_087130288.1
Microbacterium esteraromaticum
Min-wnt
MCB0349642.1
Bdellovibrionales bacterium
Min-wnt
BamE-like-Imm-Min-wnt
WP_174103980.1
Bacillus sp. Xin
Min-wnt
WP_199668302.1
Bacillus cereus group sp. N21
Min-wnt
WP_159649098.1
Erysipelothrix aquatica
Min-wnt
WP_050752403.1
Scardovia inopinata
Min-wnt
BamE-like-Imm-Min-wnt
MBR4235469.1
Clostridia bacterium
Min-wnt
WP_121443470.1
Brockia lithotrophica
Min-wnt
VNV09030.1
Streptococcus pneumoniae
Min-wnt
WP_216563049.1
Tissierella carlieri
Min-wnt
CAC60278.1
Min-wnt
WP_127541680.1
Sinorhizobium meliloti
Min-wnt
MBR7040553.1
Clostridia bacterium
Min-wnt
PWM31735.1
Verrucomicrobiota bacterium
Min-wnt
WP_055152578.1
Blautia wexlerae
Min-wnt
WP_065541928.1
uncultured Blautia sp.
Min-wnt
WP_118445576.1
Mediterraneibacter gnavus
Min-wnt
SHM02541.1
Flavobacterium johnsoniae
Min-wnt
BamE-like-Imm-Min-wnt
TAH43912.1
Bacteroidota bacterium
Min-wnt
MCB0430115.1
Flavobacteriales bacterium
Min-wnt
MCB0397665.1
Flavobacteriales bacterium
Min-wnt
MBR4272737.1
Bacteroidales bacterium
Min-wnt
WP_050809601.1
Ochrovirga pacifica
Min-wnt
MCB0805782.1
Bacteroidales bacterium
Min-wnt
MBA3930144.1
Xanthomonas sp.
Min-wnt
TM-Imm-Wnt
WP_154878906.1
Corynebacterium godavarianum
Min-wnt
MCB0342100.1
Bdellovibrionales bacterium
Min-wnt
MBK5275968.1
Desulfuromonadales bacterium
Min-wnt
WP_065547075.1
Min-wnt
WP_143539232.1
Rothia nasimurium
Min-wnt

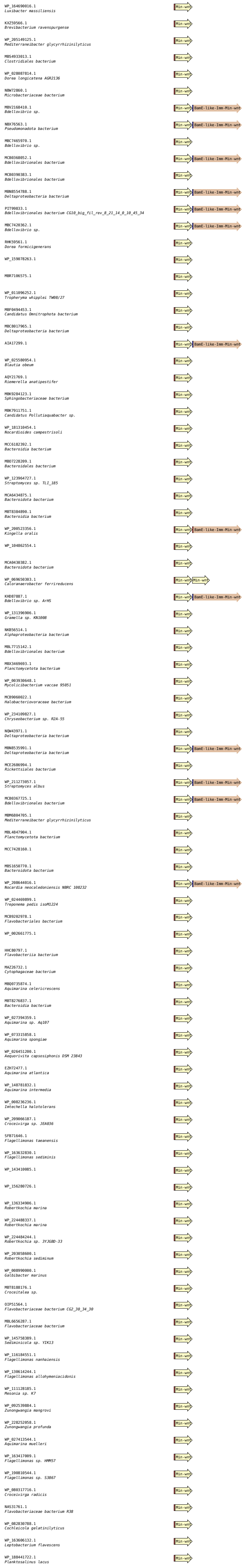

WP_164690016.1
Luxibacter massiliensis
Min-wnt
KXZ59566.1
Brevibacterium ravenspurgense
Min-wnt
WP_205149125.1
Mediterraneibacter glycyrrhizinilyticus
Min-wnt
MBS4933013.1
Clostridiales bacterium
Min-wnt
WP_028087814.1
Dorea longicatena AGR2136
Min-wnt
NBW72860.1
Microbacteriaceae bacterium
Min-wnt
MBV2168410.1
Bdellovibrio sp.
Min-wnt
BamE-like-Imm-Min-wnt
NBX76563.1
Pseudomonadota bacterium
Min-wnt
BamE-like-Imm-Min-wnt
MBC7465970.1
Bdellovibrio sp.
Min-wnt
MCB0368052.1
Bdellovibrionales bacterium
Min-wnt
BamE-like-Imm-Min-wnt
MCB0390383.1
Bdellovibrionales bacterium
Min-wnt
MBN8554788.1
Deltaproteobacteria bacterium
Min-wnt
BamE-like-Imm-Min-wnt
PIT99833.1
Bdellovibrionales bacterium CG10_big_fil_rev_8_21_14_0_10_45_34
Min-wnt
BamE-like-Imm-Min-wnt
MBC7420362.1
Bdellovibrio sp.
Min-wnt
BamE-like-Imm-Min-wnt
RHK59561.1
Dorea formicigenerans
Min-wnt
WP_159078263.1
Min-wnt
MBR7106575.1
Min-wnt
WP_011096252.1
Tropheryma whipplei TW08/27
Min-wnt
MBF0494453.1
Candidatus Omnitrophota bacterium
Min-wnt
MBC8017965.1
Deltaproteobacteria bacterium
Min-wnt
AIA17299.1
Min-wnt
BamE-like-Imm-Min-wnt
WP_025580954.1
Blautia obeum
Min-wnt
AQY21769.1
Riemerella anatipestifer
Min-wnt
MBK9284123.1
Sphingobacteriaceae bacterium
Min-wnt
MBK7911751.1
Candidatus Pollutiaquabacter sp.
Min-wnt
WP_181310454.1
Nocardioides campestrisoli
Min-wnt
MCC6182392.1
Bacteroidia bacterium
Min-wnt
MBO7228209.1
Bacteroidales bacterium
Min-wnt
WP_123964727.1
Streptomyces sp. TLI_185
Min-wnt
MCA6434875.1
Bacteroidota bacterium
Min-wnt
MBT8384890.1
Bacteroidia bacterium
Min-wnt
WP_200523356.1
Kingella oralis
Min-wnt
BamE-like-Imm-Min-wnt
WP_104862554.1
Min-wnt
MCA0430382.1
Bacteroidota bacterium
Min-wnt
WP_069650303.1
Caloranaerobacter ferrireducens
Min-wnt
Min-wnt
KHD87887.1
Bdellovibrio sp. ArHS
Min-wnt
BamE-like-Imm-Min-wnt
WP_131396906.1
Gramella sp. KN1008
Min-wnt
NKB56514.1
Alphaproteobacteria bacterium
Min-wnt
MBL7715142.1
Bdellovibrionales bacterium
Min-wnt
MBX3469693.1
Planctomycetota bacterium
Min-wnt
WP_003930648.1
Mycolicibacterium vaccae 95051
Min-wnt
MCB9060022.1
Halobacteriovoraceae bacterium
Min-wnt
WP_234109827.1
Chryseobacterium sp. R2A-55
Min-wnt
NQW43971.1
Deltaproteobacteria bacterium
Min-wnt
MBN8535991.1
Deltaproteobacteria bacterium
Min-wnt
BamE-like-Imm-Min-wnt
MCE2686994.1
Rickettsiales bacterium
Min-wnt
WP_211273057.1
Streptomyces albus
Min-wnt
BamE-like-Imm-Min-wnt
MCB0367725.1
Bdellovibrionales bacterium
Min-wnt
BamE-like-Imm-Min-wnt
MBM6804705.1
Mediterraneibacter glycyrrhizinilyticus
Min-wnt
MBL4847904.1
Planctomycetota bacterium
Min-wnt
MCC7428160.1
Min-wnt
MBS1650770.1
Bacteroidota bacterium
Min-wnt
WP_208644016.1
Nocardia neocaledoniensis NBRC 108232
Min-wnt
BamE-like-Imm-Min-wnt
WP_024469899.1
Treponema pedis isoM1224
Min-wnt
MCB9202978.1
Flavobacteriales bacterium
Min-wnt
WP_002661775.1
Min-wnt
HHC80797.1
Flavobacteriia bacterium
Min-wnt
MAZ26732.1
Cytophagaceae bacterium
Min-wnt
MBQ0735874.1
Aquimarina celericrescens
Min-wnt
MBT8276837.1
Bacteroidia bacterium
Min-wnt
WP_027394359.1
Aquimarina sp. Aq107
Min-wnt
WP_073315858.1
Aquimarina spongiae
Min-wnt
WP_026451200.1
Aequorivita capsosiphonis DSM 23843
Min-wnt
EZH72477.1
Aquimarina atlantica
Min-wnt
WP_148781832.1
Aquimarina intermedia
Min-wnt
WP_008236236.1
Imtechella halotolerans
Min-wnt
WP_209066187.1
Croceivirga sp. JEA036
Min-wnt
SFB71646.1
Flagellimonas taeanensis
Min-wnt
WP_163632830.1
Flagellimonas sediminis
Min-wnt
WP_143410085.1
Min-wnt
WP_156280726.1
Min-wnt
WP_136334906.1
Robertkochia marina
Min-wnt
WP_224488337.1
Robertkochia marina
Min-wnt
WP_224484244.1
Robertkochia sp. 3YJGBD-33
Min-wnt
WP_203058600.1
Robertkochia sediminum
Min-wnt
WP_008990000.1
Galbibacter marinus
Min-wnt
MBT8188176.1
Croceitalea sp.
Min-wnt
OIP51564.1
Flavobacteriaceae bacterium CG2_30_34_30
Min-wnt
MBL6656287.1
Flavobacteriaceae bacterium
Min-wnt
WP_145758389.1
Sediminicola sp. YIK13
Min-wnt
WP_116184551.1
Flagellimonas nanhaiensis
Min-wnt
WP_130614244.1
Flagellimonas allohymeniacidonis
Min-wnt
WP_111128185.1
Mesonia sp. K7
Min-wnt
WP_092539884.1
Zunongwangia mangrovi
Min-wnt
WP_228252058.1
Zunongwangia profunda
Min-wnt
WP_027413544.1
Aquimarina muelleri
Min-wnt
WP_163417009.1
Flagellimonas sp. HMM57
Min-wnt
WP_190810544.1
Flagellimonas sp. S3867
Min-wnt
WP_080317716.1
Croceivirga radicis
Min-wnt
NAS31761.1
Flavobacteriaceae bacterium R38
Min-wnt
WP_082830788.1
Cochleicola gelatinilyticus
Min-wnt
WP_163606132.1
Leptobacterium flavescens
Min-wnt
WP_188441722.1
Planktosalinus lacus
Min-wnt

SFW28557.1	*Sinomicrobium oceani*	Min-wnt
WP_147863634.1	*Mesonia sp. K4-1*	Min-wnt
WP_121665998.1	*Mesonia aquimarina*	Min-wnt
SFS39668.1	*Zhouia amylolytica*	Min-wnt
EIJ38465.1	*Galbibacter orientalis DSM 19592*	Min-wnt
OIQ37259.1	*Bacteroidetes bacterium MedPE-SWsnd-G1*	Min-wnt
WP_026777324.1	*Polaribacter sp. Hel_I_88*	Min-wnt
MAD96003.1	*Flavobacteriaceae bacterium*	Min-wnt
WP_215993171.1	*Polaribacter sp. 1_MG-2023*	Min-wnt
WP_147114130.1	*Polaribacter sp. IC073*	Min-wnt
WP_156099835.1	*Cellulophaga lytica*	Min-wnt
MBV1888404.1	*Urechidicola sp.*	Min-wnt
NND61619.1	*Flavobacteriaceae bacterium*	Min-wnt
NQY01540.1	*Flavobacteriaceae bacterium*	Min-wnt
MBT8312668.1	*Eudoraea sp.*	Min-wnt
MBT8385586.1	*Bacteroidia bacterium*	Min-wnt
MAN28653.1	*Mesonia sp.*	Min-wnt
MCB0460248.1	*Flavobacteriaceae bacterium*	Min-wnt
MCB0476211.1	*Flavobacteriaceae bacterium*	Min-wnt
MBC7714680.1	*Bacteriovorax sp.*	Min-wnt
WP_147120128.1	*Polaribacter sp. IC066*	Min-wnt
MBR4968312.1	*Bacteroidaceae bacterium*	Min-wnt
WP_132984256.1	*Tissierella praeacuta*	Min-wnt
WP_192014899.1		Min-wnt
MBR3884632.1	*Bacteroidaceae bacterium*	Min-wnt
MBR3612957.1	*Bacteroidaceae bacterium*	Min-wnt
WP_119057094.1	*Candidatus Ornithobacterium hominis*	Min-wnt
WP_040738792.1	*Nocardia tenerifensis*	Min-wnt
OIP00497.1	*Bacteroidetes bacterium CG2_30_33_31*	Min-wnt
MBB1561277.1	*Flavobacteriaceae bacterium*	Min-wnt
WP_074866710.1	*Lysobacter enzymogenes*	Min-wnt
WP_101523985.1	*Nocardioides houyundeii*	Min-wnt
WP_157621505.1	*Saccharothrix sp. NRRL B-16348*	Min-wnt
WP_143852580.1	*Chryseobacterium sp. SNU WT5*	Min-wnt
WP_133500097.1	*Cognatilysobacter terrigena*	Min-wnt
WP_143674228.1	*Streptomyces dengpaensis*	Min-wnt
RQO45949.1	*Variovorax sp. KBW07*	Min-wnt
WP_194705098.1	*Nocardioides islandensis*	Min-wnt
WP_145056478.1	*Lignipirellula cremea*	Min-wnt
WP_073177888.1	*Cruoricaptor ignavus*	Min-wnt
WP_039580032.1	*Xanthomonas phaseoli pv. phaseoli*	Min-wnt
WP_182042735.1	*Moheibacter lacus*	Min-wnt
MXV38614.1	*Flavobacteriaceae bacterium Ap0902*	Min-wnt
WP_147756603.1	*Chryseobacterium sp.*	Min-wnt
MBL4594774.1	*Flavobacteriales bacterium*	Min-wnt
RLD67845.1	*Bacteroidota bacterium*	Min-wnt
MBK8811790.1	*Acidobacteriota bacterium*	Min-wnt
MCB1102951.1	*Kiritimatiellia bacterium*	Min-wnt
WP_211886487.1	*Plastoroseomonas hellenica*	Min-wnt
WP_112135570.1	*Glycomyces dulcitolivorans*	Min-wnt
MBP7173906.1	*Cloacibacterium sp.*	Min-wnt
EZB52931.1	*Escherichia coli O157:H7 str. K1793*	Min-wnt
WP_157417636.1	*Nocardioides sp. Iso805N*	Min-wnt
WP_036164358.1	*Lysobacter dokdonensis DS-58*	Min-wnt
WP_158730860.1		Min-wnt
NUS60899.1	*Lysobacter sp.*	Min-wnt
WP_232704899.1		Min-wnt
WP_210537331.1		Min-wnt
NOU42947.1	*Methyloglobulus sp.*	Min-wnt
MBL7663840.1	*Bacteriovoracaceae bacterium*	Min-wnt
WP_220666750.1	*Rhodococcus sp. DMU2021*	Min-wnt
WP_050377959.1	*Chryseobacterium sp. Hurlbut01*	Min-wnt
WP_146228929.1	*Streptomyces sp. NWU339*	Min-wnt
MBP6748155.1	*Lysobacteraceae bacterium*	Min-wnt
WP_137266421.1		Min-wnt
WP_078564344.1	*Xanthomonas axonopodis pv. melhusii*	Min-wnt
MCB2221732.1	*Bacteroidota bacterium*	Min-wnt
MBA3682835.1	*Bacteroidota bacterium*	Min-wnt
WP_091124461.1	*Nocardioides terrae*	Min-wnt
WP_079667597.1	*Soonwooa buanensis*	Min-wnt
MBS9779197.1	*Moraxellaceae bacterium*	Min-wnt
WP_209889984.1	*Nocardia goodfellowii*	Min-wnt
RNM15741.1	*Nocardioides pocheonensis*	Min-wnt
WP_046833750.1	*Xanthomonas phaseoli pv. phaseoli*	Min-wnt
WP_232906477.1	*Lysobacter sp. 5GHs7-4*	Min-wnt
NOX44080.1	*Gammaproteobacteria bacterium*	Min-wnt
WP_068598667.1	*Vaginella massiliensis*	Min-wnt
RME16411.1	*Bacteroidota bacterium*	Min-wnt
MBQ2643963.1	*bacterium*	Min-wnt
SFK95479.1	*Lysobacter sp. cf310*	Min-wnt
HAI79929.1	*Chryseobacterium sp.*	Min-wnt
WP_129987047.1	*Nocardioides iriomotensis*	Min-wnt
WP_232338602.1		Min-wnt
MBP6757424.1	*Bacteroidia bacterium*	Min-wnt
MBK7708626.1	*Acidobacteriota bacterium*	Min-wnt
NOT14726.1	*Methylotenera sp.*	Min-wnt
WP_223265799.1		Min-wnt
RDY68112.1	*Lysobacter soli*	Min-wnt
WP_115858080.1	*Lysobacter silvisoli*	Min-wnt
WP_127586218.1	*Paenibacillus koleovorans NBRC 103111*	Min-wnt
MCB0471509.1	*Flavobacteriaceae bacterium*	Min-wnt
WP_092736278.1	*Riemerella columbipharyngis*	Min-wnt
RZJ44680.1	*Chryseobacterium sp.*	Min-wnt

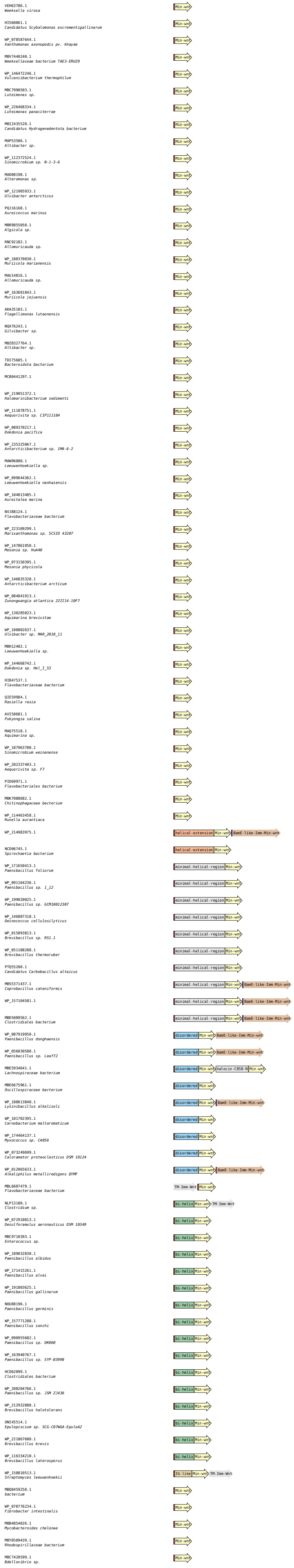
VEH63786.1
Weeksella virosa
Min-wnt
HIS60861.1
Candidatus Scybalomonas excrementigallinarum
Min-wnt
WP_078587644.1
Xanthomonas axonopodis pv. khayae
Min-wnt
MBV7440240.1
Weeksellaceae bacterium TAE3-ERU29
Min-wnt
WP_146472246.1
Vulcaniibacterium thermophilum
Min-wnt
MBC7990503.1
Luteimonas sp.
Min-wnt
WP_226468334.1
Luteimonas panaciterrae
Min-wnt
MBI2435520.1
Candidatus Hydrogenedentota bacterium
Min-wnt
MAP53586.1
Altibacter sp.
Min-wnt
WP_112372524.1
Sinomicrobium sp. N-1-3-6
Min-wnt
MAO08198.1
Alteromonas sp.
Min-wnt
WP_121905933.1
Ulvibacter antarcticus
Min-wnt
PQJ16168.1
Aureicoccus marinus
Min-wnt
MBR9855050.1
Algicola sp.
Min-wnt
RNC92182.1
Allomuricauda sp.
Min-wnt
WP_188370030.1
Muriicola marianensis
Min-wnt
MAU14816.1
Allomuricauda sp.
Min-wnt
WP_163691843.1
Muriicola jejuensis
Min-wnt
AKA35103.1
Flagellimonas lutaonensis
Min-wnt
NQX76243.1
Gilvibacter sp.
Min-wnt
MBZ0327764.1
Altibacter sp.
Min-wnt
TDI75685.1
Bacteroidota bacterium
Min-wnt
MCB0441297.1
Min-wnt
WP_219051372.1
Halomarinibacterium sedimenti
Min-wnt
WP_111878751.1
Aequorivita sp. CIP111184
Min-wnt
WP_089370217.1
Dokdonia pacifica
Min-wnt
WP_235325067.1
Antarcticibacterium sp. 1MA-6-2
Min-wnt
MAW96080.1
Leeuwenhoekiella sp.
Min-wnt
WP_099644362.1
Leeuwenhoekiella nanhaiensis
Min-wnt
WP_104813485.1
Aureitalea marina
Min-wnt
NVJ88124.1
Flavobacteriaceae bacterium
Min-wnt
WP_223109299.1
Marixanthomonas sp. SCSIO 43207
Min-wnt
WP_147861950.1
Mesonia sp. HuA40
Min-wnt
WP_073150395.1
Mesonia phycicola
Min-wnt
WP_146835328.1
Antarcticibacterium arcticum
Min-wnt
WP_084841913.1
Zunongwangia atlantica 22II14-10F7
Min-wnt
WP_130285023.1
Aquimarina brevivitae
Min-wnt
WP_100802637.1
Ulvibacter sp. MAR_2010_11
Min-wnt
MBH12402.1
Leeuwenhoekiella sp.
Min-wnt
WP_144668742.1
Dokdonia sp. Hel_I_53
Min-wnt
HIB47537.1
Flavobacteriaceae bacterium
Min-wnt
QIE59984.1
Rasiella rasia
Min-wnt
AVI50681.1
Pukyongia salina
Min-wnt
MAQ75518.1
Aquimarina sp.
Min-wnt
WP_187963780.1
Sinomicrobium weinanense
Min-wnt
WP_202337403.1
Aequorivita sp. F7
Min-wnt
PID69971.1
Flavobacteriales bacterium
Min-wnt
MBK7088682.1
Chitinophagaceae bacterium
Min-wnt
WP_114463458.1
Runella aurantiaca
Min-wnt
WP_214983975.1
helical-extension Min-wnt BamE-like-Imm-Min-wnt
NCD06745.1
Spirochaetia bacterium
helical-extension Min-wnt
WP_171650413.1
Paenibacillus foliorum
minimal-helical-region Min-wnt
WP_091164236.1
Paenibacillus sp. 1_12
minimal-helical-region Min-wnt
WP_199020025.1
Paenibacillus sp. GCM10012307
minimal-helical-region Min-wnt
WP_146887318.1
Deinococcus cellulosilyticus
minimal-helical-region Min-wnt
WP_015893813.1
Brevibacillus sp. RS1.1
minimal-helical-region Min-wnt
WP_051188288.1
Brevibacillus thermoruber
minimal-helical-region Min-wnt
PTQ55200.1
Candidatus Carbobacillus altaicus
minimal-helical-region Min-wnt
MBS5371437.1
Coprobacillus cateniformis
minimal-helical-region Min-wnt BamE-like-Imm-Min-wnt
WP_157104581.1
minimal-helical-region Min-wnt BamE-like-Imm-Min-wnt
MBD5089562.1
Clostridiales bacterium
minimal-helical-region Min-wnt BamE-like-Imm-Min-wnt
WP_087919950.1
Paenibacillus donghaensis
disordered Min-wnt BamE-like-Imm-Min-wnt
WP_056030588.1
Paenibacillus sp. Leaf72
disordered Min-wnt BamE-like-Imm-Min-wnt
MBE5934641.1
Lachnospiraceae bacterium
disordered Min-wnt halocin-C858-N Min-wnt
MBE6675961.1
Oscillospiraceae bacterium
disordered Min-wnt
WP_188613840.1
Lysinibacillus alkalisoli
disordered Min-wnt BamE-like-Imm-Min-wnt
WP_101702395.1
Carnobacterium maltaromaticum
disordered Min-wnt
WP_174464137.1
Myxococcus sp. CA056
disordered Min-wnt
WP_073249699.1
Caloramator proteoclasticus DSM 10124
disordered Min-wnt
WP_012065633.1
Alkaliphilus metalliredigens QYMF
disordered Min-wnt BamE-like-Imm-Min-wnt
MBL6607479.1
Flavobacteriaceae bacterium
TM-Imm-Wnt Min-wnt
NLP13180.1
Clostridium sp.
bi-helix Min-wnt TM-Imm-Wnt
WP_072910813.1
Desulforamulus aeronauticus DSM 10349
bi-helix Min-wnt
MBC9710393.1
Enterococcus sp.
bi-helix Min-wnt
WP_189032830.1
Paenibacillus albidus
bi-helix Min-wnt
WP_171415261.1
Paenibacillus alvei
bi-helix Min-wnt
WP_191803625.1
Paenibacillus gallinarum
bi-helix Min-wnt
NOU88196.1
Paenibacillus germinis
bi-helix Min-wnt
WP_157771288.1
Paenibacillus sonchi
bi-helix Min-wnt
WP_090955682.1
Paenibacillus sp. OK060
bi-helix Min-wnt
WP_163940767.1
Paenibacillus sp. SYP-B3998
bi-helix Min-wnt
HCO62099.1
Clostridiales bacterium
bi-helix Min-wnt
WP_208204766.1
Paenibacillus sp. JSM ZJ436
bi-helix Min-wnt
WP_212932888.1
Brevibacillus halotolerans
bi-helix Min-wnt
ONI45514.1
Epulopiscium sp. SCG-C07WGA-EpuloA2
bi-helix Min-wnt
WP_221867680.1
Brevibacillus brevis
bi-helix Min-wnt
WP_116334210.1
Brevibacillus laterosporus
bi-helix Min-wnt
WP_158810513.1
Streptomyces leeuwenhoekii
IG-like Min-wnt TM-Imm-Wnt
MBQ8459250.1
bacterium
Min-wnt
WP_078776234.1
Fibrobacter intestinalis
Min-wnt
MBB4854026.1
Mycobacteroides chelonae
Min-wnt
MBY0509439.1
Rhodospirillaceae bacterium
Min-wnt
MBC7420599.1
Bdellovibrio sp.
Min-wnt

WP_089525487.1	*Paenibacillus herberti*	Min-wnt
HGF04273.1		Min-wnt
WP_036671642.1	*Paenibacillus sp. FSL H7-0942*	Min-wnt
MBD3397849.1	*Candidatus Micrarchaeota archaeon*	Min-wnt
KKQ90637.1	*Berkelbacteria bacterium GW2011_GWA2_38_9*	Min-wnt
MBE6132356.1	*Erysipelotrichaceae bacterium*	Min-wnt
WP_184792608.1	*Phytomonospora endophytica*	Min-wnt
HBB88130.1	*Blastocatellia bacterium*	Min-wnt
WP_173526023.1	*Nonomuraea antri*	Min-wnt
TPW19080.1	*Elusimicrobiota bacterium*	Min-wnt
WP_234880925.1	*Mycobacteroides salmoniphilum*	Min-wnt
TDZ93469.1	*Mycobacteroides salmoniphilum*	Min-wnt
MCB1285170.1	*Microthrixaceae bacterium*	Min-wnt
NBW64536.1	*bacterium*	Min-wnt
WP_035875992.1	*Kitasatospora cheerisanensis KCTC 2395*	Min-wnt
TAE24342.1	*Cytophagales bacterium*	Min-wnt
WP_127503967.1	*Actinoplanes solisilvae*	Min-wnt
MBD65116.1	*Halobacteriovoraceae bacterium*	Min-wnt
OFZ30049.1	*Bdellovibrionales bacterium RIFCSPHIGHO2_01_FULL_40_29*	Min-wnt
MCB9254551.1	*Pseudobdellovibrionaceae bacterium*	Min-wnt
WP_207333950.1	*Fibrella aquatilis*	Min-wnt
SMN02529.1	*uncultured Candidatus Thioglobus sp.*	BamE-like-Imm-Min-wnt Min-wnt
HCS25143.1	*Acholeplasmatales bacterium*	Min-wnt
OPZ53515.1	*Deltaproteobacteria bacterium ADurb.BinA014*	Min-wnt
NCP12716.1	*Sphingomonadales bacterium*	BamE-like-Imm-Min-wnt Min-wnt Min-wnt
PIR53948.1	*Candidatus Peregrinibacteria bacterium CG10_big_fil_rev_8_21_14_0_10_42_8*	Min-wnt
WP_162388651.1	*Spirosoma endbachense*	Min-wnt
WP_190891945.1	*Spirosoma profusum*	Min-wnt BamE-like-Imm-Min-wnt
MBE32686.1	*bacterium*	Min-wnt
WP_099214570.1	*Tenacibaculum discolor*	Min-wnt Jellyroll-like-Imm-Min-wnt-like
MBQ3948504.1	*Ruminococcus sp.*	Min-wnt
MBF0215927.1	*Candidatus Omnitrophota bacterium*	Min-wnt
MBN8530644.1	*Alphaproteobacteria bacterium*	Min-wnt
MBX7207317.1	*Verrucomicrobiaceae bacterium*	Min-wnt
WP_218280808.1	*Verrucomicrobium spinosum DSM 4136 = JCM 18804*	Min-wnt
WP_166550548.1	*Cellulophaga sp. BC115SP*	BamE-like-Imm-Min-wnt BamE-like-Imm-Min-wnt ? Min-wnt BamE-like-Imm-Min-wnt

####;secreted fungal versions likely directed at pathogenic or competing bacteria

APU50778.1		Min-wnt
XP_002739942.1	*Saccoglossus kowalevskii*	Min-wnt
XP_002736627.1	*Saccoglossus kowalevskii*	Min-wnt
XP_002742451.1	*Saccoglossus kowalevskii*	Min-wnt
CAF0989857.1	*Didymodactylos carnosus*	Min-wnt
CAF1416688.1	*Rotaria sordida*	Min-wnt
CAF0836331.1	*Didymodactylos carnosus*	Min-wnt
CAF3251756.1	*Rotaria socialis*	Min-wnt
CAF1328615.1	*Adineta ricciae*	Min-wnt
UJR34554.1	*Adineta vaga*	Min-wnt
CAF1111402.1	*Rotaria sp. Silwood1*	Min-wnt
ORX92093.1	*Basidiobolus meristosporus CBS 931.73*	Min-wnt ? Min-wnt
PKC55642.1	*Rhizophagus irregularis*	Min-wnt
CAG8669258.1	*Ambispora gerdemannii*	Min-wnt
KAH6901204.1	*Coprinopsis sp. MPI-PUGE-AT-0042*	Min-wnt
XP_001836672.2	*Coprinopsis cinerea okayama7#130*	Min-wnt
XP_001836671.2	*Coprinopsis cinerea okayama7#130*	Min-wnt
XP_001838919.1	*Coprinopsis cinerea okayama7#130*	Min-wnt
TFK18782.1	*Coprinopsis marcescibilis*	Min-wnt
KAF2432311.1	*Tothia fuscella*	Min-wnt
CCX11736.1	*Pyronema omphalodes CBS 100304*	Min-wnt
KAG0124889.1	*Tuber indicum*	Min-wnt
CCX33658.1	*Pyronema omphalodes CBS 100304*	Min-wnt
KAF7512476.1	*Endocarpon pusillum*	Min-wnt
RPB03961.1	*Choiromyces venosus 120613-1*	Min-wnt Min-wnt
TGZ85108.1	*Ascodesmis nigricans*	Min-wnt
XP_033533576.1	*Eremomyces bilateralis CBS 781.70*	Min-wnt
XP_033535402.1	*Eremomyces bilateralis CBS 781.70*	Min-wnt
CUS11844.1	*Tuber aestivum*	Min-wnt
KAH7082508.1	*Paraphoma chrysanthemicola*	Min-wnt
XP_025540673.1	*Aspergillus costaricaensis CBS 115574*	Min-wnt
XP_041538695.1	*Aspergillus luchuensis*	Min-wnt
RSL84449.1	*Fusarium oligoseptatum*	Min-wnt
KPM37678.1	*Neonectria ditissima*	Min-wnt

#;possible comparable late acquisitions in cnidarians

XP_020610461.1	*Orbicella faveolata*	Min-wnt
PFX14481.1		Min-wnt Min-wnt
PFX22447.1		Min-wnt

####;membrane-anchored versions, potentially involved in contact-dependent conflict

NVK53065.1	*Flavobacteriaceae bacterium*	helical-extension Min-wnt C-terminal-hairpin BamE-like-Imm-Min-wnt
WP_073118127.1	*Wenyingzhuangia marina*	helical-extension Min-wnt C-terminal-hairpin BamE-like-Imm-Min-wnt BamE-like-Imm-Min-wnt BamE-like-Imm-Min-wnt
WP_051477625.1	*Aquimarina pacifica*	helical-extension Min-wnt C-terminal-hairpin
NVK51602.1	*Flavobacteriaceae bacterium*	helical-extension Min-wnt C-terminal-hairpin BamE-like-Imm-Min-wnt BamE-like-Imm-Min-wnt
MBT5090326.1	*Flavobacteriales bacterium*	helical-extension Min-wnt C-terminal-hairpin BamE-like-Imm-Min-wnt
WP_223442261.1	*Polaribacter litorisediminis*	helical-extension Min-wnt C-terminal-hairpin BamE-like-Imm-Min-wnt
WP_144430050.1	*Tenacibaculum piscium*	helical-extension Min-wnt C-terminal-hairpin BamE-like-Imm-Min-wnt
WP_075343021.1	*Tenacibaculum agarivorans*	helical-extension Min-wnt C-terminal-hairpin BamE-like-Imm-Min-wnt
WP_028887462.1	*Tenacibaculum ovolyticum DSM 18103*	helical-extension Min-wnt C-terminal-hairpin
WP_132724207.1	*uncultured Tenacibaculum sp.*	helical-extension Min-wnt C-terminal-hairpin
WP_231841460.1	*Flavobacterium cyclinae*	helical-extension Min-wnt BamE-like-Imm-Min-wnt
SOD02434.1	*bacterium JGI 053*	helical-extension Min-wnt
WP_189567959.1	*Persicitalea jodogahamensis*	helical-extension Min-wnt BamE-like-Imm-Min-wnt
WP_025742356.1	*Aquimarina pacifica*	helical-extension Min-wnt
TAH16643.1	*Runella slithyformis*	helical-extension Min-wnt
WP_053961919.1	*Lawsonella clevelandensis*	helical-extension Min-wnt
WP_019987998.1	*Rudanella lutea DSM 19387*	helical-extension Min-wnt BamE-like-Imm-Min-wnt
WP_015814409.1	*Dyadobacter fermentans DSM 18053*	helical-extension Min-wnt BamE-like-Imm-Min-wnt BamE-like-Imm-Min-wnt

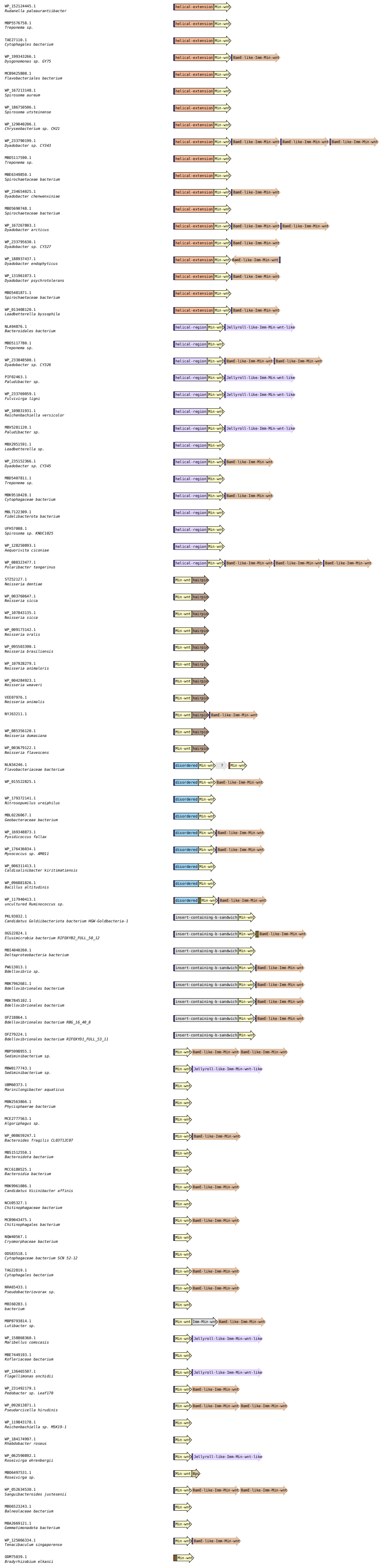

WP_152124445.1
Rudanella paleaurantiibacter
helical-extension
Min-wnt
MBP5576758.1
Treponema sp.
helical-extension
Min-wnt
TAE27110.1
Cytophagales bacterium
helical-extension
Min-wnt
WP_199343266.1
Dysgonomonas sp. GY75
helical-extension
Min-wnt
BamE-like-Imm-Min-wnt
MCB9425808.1
Flavobacteriales bacterium
helical-extension
Min-wnt
WP_167213148.1
Spirosoma aureum
helical-extension
Min-wnt
WP_186750506.1
Spirosoma utsteinense
helical-extension
Min-wnt
WP_129040206.1
Chryseobacterium sp. CH21
helical-extension
Min-wnt
WP_233790199.1
Dyadobacter sp. CY343
helical-extension
Min-wnt
BamE-like-Imm-Min-wnt
BamE-like-Imm-Min-wnt
BamE-like-Imm-Min-wnt
MBO5117590.1
Treponema sp.
helical-extension
Min-wnt
MBE6349850.1
Spirochaetaceae bacterium
helical-extension
Min-wnt
WP_234654025.1
Dyadobacter chenwenxiniae
helical-extension
Min-wnt
BamE-like-Imm-Min-wnt
MBO5690748.1
Spirochaetaceae bacterium
helical-extension
Min-wnt
WP_167267803.1
Dyadobacter arcticus
helical-extension
Min-wnt
BamE-like-Imm-Min-wnt
BamE-like-Imm-Min-wnt
WP_233795630.1
Dyadobacter sp. CY327
helical-extension
Min-wnt
BamE-like-Imm-Min-wnt
WP_188937437.1
Dyadobacter endophyticus
helical-extension
Min-wnt
BamE-like-Imm-Min-wnt
WP_131961073.1
Dyadobacter psychrotolerans
helical-extension
Min-wnt
BamE-like-Imm-Min-wnt
MBO5481871.1
Spirochaetaceae bacterium
helical-extension
Min-wnt
WP_013408120.1
Leadbetterella byssophila
helical-extension
Min-wnt
BamE-like-Imm-Min-wnt
NLA94876.1
Bacteroidales bacterium
helical-region
Min-wnt
Jellyroll-like-Imm-Min-wnt-like
MBO5117780.1
Treponema sp.
helical-region
Min-wnt
WP_233848500.1
Dyadobacter sp. CY326
helical-region
Min-wnt
BamE-like-Imm-Min-wnt
BamE-like-Imm-Min-wnt
PIF02463.1
Paludibacter sp.
helical-region
Min-wnt
Jellyroll-like-Imm-Min-wnt-like
WP_233769059.1
Fulvivirga ligni
helical-region
Min-wnt
Jellyroll-like-Imm-Min-wnt-like
WP_109831931.1
Reichenbachiella versicolor
helical-region
Min-wnt
MBV5281120.1
Paludibacter sp.
helical-region
Min-wnt
Jellyroll-like-Imm-Min-wnt-like
MBX2951591.1
Leadbetterella sp.
helical-region
Min-wnt
WP_235152366.1
Dyadobacter sp. CY345
helical-region
Min-wnt
BamE-like-Imm-Min-wnt
MBD5407811.1
Treponema sp.
helical-region
Min-wnt
MBK9510428.1
Cytophagaceae bacterium
helical-region
Min-wnt
BamE-like-Imm-Min-wnt
MBL7122309.1
Fidelibacterota bacterium
helical-region
Min-wnt
UFH57008.1
Spirosoma sp. KNUC1025
helical-region
Min-wnt
WP_128250893.1
Aequorivita ciconiae
helical-region
Min-wnt
WP_088323477.1
Polaribacter tangerinus
helical-region
Min-wnt
BamE-like-Imm-Min-wnt
BamE-like-Imm-Min-wnt
BamE-like-Imm-Min-wnt
STZ52127.1
Neisseria dentiae
Min-wnt
hairpin
WP_003760647.1
Neisseria sicca
Min-wnt
hairpin
WP_107843135.1
Neisseria sicca
Min-wnt
hairpin
WP_009173142.1
Neisseria oralis
Min-wnt
hairpin
WP_095503390.1
Neisseria brasiliensis
Min-wnt
hairpin
WP_107928279.1
Neisseria animaloris
Min-wnt
hairpin
WP_004284923.1
Neisseria weaveri
Min-wnt
hairpin
VEE07976.1
Neisseria animalis
Min-wnt
hairpin
NYJ63211.1
Min-wnt
hairpin
BamE-like-Imm-Min-wnt
WP_085356120.1
Neisseria dumasiana
Min-wnt
hairpin
WP_003679122.1
Neisseria flavescens
Min-wnt
hairpin
NLN34246.1
Flavobacteriaceae bacterium
disordered
Min-wnt
?
Min-wnt
WP_015522825.1
disordered
Min-wnt
BamE-like-Imm-Min-wnt
WP_179372141.1
Nitrosopumilus ureiphilus
disordered
Min-wnt
MBL0226067.1
Geobacteraceae bacterium
disordered
Min-wnt
WP_169348873.1
Pyxidicoccus fallax
disordered
Min-wnt
BamE-like-Imm-Min-wnt
WP_176436034.1
Myxococcus sp. AM011
disordered
Min-wnt
BamE-like-Imm-Min-wnt
WP_006311413.1
Caldisalinibacter kiritimatiensis
disordered
Min-wnt
WP_096881826.1
Bacillus altitudinis
disordered
Min-wnt
WP_117940413.1
uncultured Ruminococcus sp.
disordered
Min-wnt
BamE-like-Imm-Min-wnt
PKL93032.1
Candidatus Goldiibacteriota bacterium HGW-Goldbacteria-1
insert-containing-b-sandwich
Min-wnt
OGS22824.1
Elusimicrobia bacterium RIFOXYB2_FULL_50_12
insert-containing-b-sandwich
Min-wnt
BamE-like-Imm-Min-wnt
MBI4040260.1
Deltaproteobacteria bacterium
insert-containing-b-sandwich
Min-wnt
PWU13013.1
Bdellovibrio sp.
insert-containing-b-sandwich
Min-wnt
BamE-like-Imm-Min-wnt
MBK7962681.1
Bdellovibrionales bacterium
insert-containing-b-sandwich
Min-wnt
BamE-like-Imm-Min-wnt
MBK7845102.1
Bdellovibrionales bacterium
insert-containing-b-sandwich
Min-wnt
BamE-like-Imm-Min-wnt
OFZ18864.1
Bdellovibrionales bacterium RBG_16_40_8
insert-containing-b-sandwich
Min-wnt
BamE-like-Imm-Min-wnt
OFZ79224.1
Bdellovibrionales bacterium RIFOXYD1_FULL_53_11
insert-containing-b-sandwich
Min-wnt
MBP5998955.1
Sediminibacterium sp.
Min-wnt
BamE-like-Imm-Min-wnt
BamE-like-Imm-Min-wnt
MBW0177743.1
Sediminibacterium sp.
Min-wnt
Jellyroll-like-Imm-Min-wnt-like
UBM60373.1
Marinilongibacter aquaticus
Min-wnt
MBN2563866.1
Phycisphaerae bacterium
Min-wnt
MCE2777563.1
Algoriphagus sp.
Min-wnt
WP_008659247.1
Bacteroides fragilis CL03T12C07
Min-wnt
BamE-like-Imm-Min-wnt
MBS1512550.1
Bacteroidota bacterium
Min-wnt
MCC6180525.1
Bacteroidia bacterium
Min-wnt
MBK9961086.1
Candidatus Vicinibacter affinis
Min-wnt
BamE-like-Imm-Min-wnt
NCU05327.1
Chitinophagaceae bacterium
Min-wnt
MCB9043475.1
Chitinophagales bacterium
Min-wnt
BamE-like-Imm-Min-wnt
NQW40567.1
Cryomorphaceae bacterium
Min-wnt
ODS83518.1
Cytophagaceae bacterium SCN 52-12
Min-wnt
TAG22819.1
Cytophagales bacterium
Min-wnt
BamE-like-Imm-Min-wnt
NRA65433.1
Pseudobacteriovorax sp.
Min-wnt
BamE-like-Imm-Min-wnt
MBI60283.1
bacterium
Min-wnt
MBP8793814.1
Lutibacter sp.
Min-wnt
Imm-Min-wnt
BamE-like-Imm-Min-wnt
WP_158868360.1
Maribellus comscasis
Min-wnt
Jellyroll-like-Imm-Min-wnt-like
MBE7449193.1
Kofleriaceae bacterium
Min-wnt
WP_136465507.1
Flagellimonas onchidii
Min-wnt
Jellyroll-like-Imm-Min-wnt-like
WP_231492179.1
Pedobacter sp. Leaf170
Min-wnt
BamE-like-Imm-Min-wnt
WP_092013871.1
Pseudarcicella hirudinis
Min-wnt
BamE-like-Imm-Min-wnt
BamE-like-Imm-Min-wnt
WP_119843178.1
Reichenbachiella sp. MSK19-1
Min-wnt
WP_184174997.1
Rhabdobacter roseus
Min-wnt
WP_062590892.1
Roseivirga ehrenbergii
Min-wnt
Jellyroll-like-Imm-Min-wnt-like
MBO6497531.1
Roseivirga sp.
Min-wnt
Bps
WP_052634530.1
Sanguibacteroides justesenii
Min-wnt
BamE-like-Imm-Min-wnt
BamE-like-Imm-Min-wnt
MBO6523243.1
Balneolaceae bacterium
Min-wnt
MBA2669121.1
Gemmatimonadota bacterium
Min-wnt
WP_125066334.1
Tenacibaculum singaporense
Min-wnt
BamE-like-Imm-Min-wnt
ODM75039.1
Bradyrhizobium elkanii
Min-wnt

WP_155795458.1
Bradyrhizobium diazoefficiens
Min-wnt

MBN8589863.1
Rhodothermia bacterium
Min-wnt

NVK83062.1
Cytophagia bacterium
Min-wnt

KYF51172.1
Sorangium cellulosum
Min-wnt

###############;secreted Min-wnts which lack evidence of an immunity protein. Fused to a unique 2-cysteine-containing tail,

MBQ5404823.1
Bacteroidales bacterium
Min-wnt CC-tail

MBR4677895.1
Bacteroidales bacterium
Min-wnt CC-tail

MBN2173908.1
Bacteroidales bacterium
Min-wnt CC-tail

MBN3035588.1
Bacteroidales bacterium
Min-wnt CC-tail

MBQ2098251.1
Bacteroidales bacterium
Min-wnt CC-tail

MBO7141745.1
Bacteroidales bacterium
Min-wnt CC-tail

KGL60056.1
Polaribacter sp. Hel1_33_49
Min-wnt CC-tail

MBI3512146.1
Bacteroidota bacterium
Min-wnt CC-tail

MBQ9214466.1
Bacteroidales bacterium
Min-wnt CC-tail

MBK6833728.1
Bacteroidota bacterium
Min-wnt CC-tail

MBL0016624.1
Min-wnt CC-tail

MBK7849637.1
Bacteroidota bacterium
Min-wnt CC-tail

NOZ47015.1
Chlorobiota bacterium
Min-wnt CC-tail

OQX97227.1
Bacteroidetes bacterium 4572_128
Min-wnt CC-tail

MBV6485298.1
Flavobacteriales bacterium
Min-wnt CC-tail

NWJ50632.1
Bacteroidota bacterium
Min-wnt CC-tail

MBS7314758.1
Bacteroidales bacterium
Min-wnt CC-tail

MBP7497959.1
Bacteroidales bacterium
Min-wnt CC-tail

NMD00319.1
Bacteroidales bacterium
Min-wnt CC-tail

MBA2584636.1
Bacteroidota bacterium
Min-wnt CC-tail

MCE3229999.1
Bacteroidota bacterium
Min-wnt CC-tail

MBL4752244.1
Flavobacteriales bacterium
Min-wnt CC-tail

MBN1339793.1
Bacteroidales bacterium
Min-wnt CC-tail

WP_233188633.1
Tenacibaculum sp. SG-28
Min-wnt CC-tail

TVQ88910.1
Bacteroidota bacterium
Min-wnt CC-tail

NQY68863.1
Flavobacteriales bacterium
Min-wnt CC-tail

WP_132791479.1
Tenacibaculum skagerrakense
Min-wnt CC-tail

HAN77307.1
Bacteroidales bacterium
Min-wnt CC-tail

WP_208077094.1
Polaribacter cellanae
Min-wnt CC-tail

OFX28584.1
Bacteroidetes bacterium GWA2_31_9
Min-wnt CC-tail

MSP69517.1
Bacteroidota bacterium
Min-wnt CC-tail

MBM3436635.1
Bacteroidota bacterium
Min-wnt CC-tail

MBX7205621.1
Bacteroidia bacterium
Min-wnt CC-tail

TND09297.1
Bacteroidota bacterium
Min-wnt CC-tail

NVN95432.1
Bacteroidota bacterium
Min-wnt CC-tail

MBL4625775.1
Flavobacteriales bacterium
Min-wnt CC-tail

HHZ64125.1
Flavobacteriales bacterium
Min-wnt CC-tail

MBO7225063.1
Bacteroidales bacterium
Min-wnt CC-tail

WP_223443291.1
Polaribacter litorisediminis
Min-wnt CC-tail

WP_089378601.1
Lutibacter flavus
Min-wnt CC-tail

RUA09915.1
Flavobacteriia bacterium
Min-wnt CC-tail

OFY24287.1
Bacteroidetes bacterium GWF2_33_38
Min-wnt CC-tail

NVO03145.1
Bacteroidota bacterium
Min-wnt CC-tail

MBL7104342.1
Bacteroidales bacterium
Min-wnt CC-tail

WP_158839860.1
Polaribacter sp. L3A8
Min-wnt CC-tail

MBI2281675.1
Bacteroidota bacterium
Min-wnt CC-tail

MBK6399743.1
Bacteroidota bacterium
Min-wnt CC-tail

QCX40580.1
Aureibaculum algae
Min-wnt CC-tail

MBI5217685.1
Bacteroidia bacterium
Min-wnt CC-tail

MBL4707237.1
Flavobacteriales bacterium
Min-wnt CC-tail

MBP9847063.1
Saprospiraceae bacterium
Min-wnt CC-tail

HIW87310.1
Candidatus Onthomorpha intestinigallinarum
Min-wnt CC-tail

MBN2663662.1
Bacteroidales bacterium
Min-wnt CC-tail

MCA6364468.1
Bacteroidota bacterium
Min-wnt CC-tail

OFX66933.1
Bacteroidetes bacterium GWE2_29_8
Min-wnt CC-tail

MBK7819245.1
Sphingobacteriaceae bacterium
Min-wnt CC-tail

MCD6366401.1
Bacteroidales bacterium
Min-wnt CC-tail

WP_148868586.1
Tenacibaculum adriaticum
Min-wnt CC-tail

HCE54932.1
Lutibacter sp.
Min-wnt CC-tail

WP_132727228.1
uncultured Tenacibaculum sp.
Min-wnt CC-tail

WP_235274567.1
Tenacibaculum sp. Cn5-34
Min-wnt CC-tail

WP_123899085.1
Aureibaculum marinum
Min-wnt CC-tail

WP_218771908.1
Tenacibaculum sp. HL-MS23
Min-wnt CC-tail

WP_224928198.1
Lutimonas saemankumensis
Min-wnt CC-tail

MBS1646927.1
Bacteroidota bacterium
Min-wnt CC-tail

WP_168814578.1
Lutibacter sp. B1
Min-wnt CC-tail

WP_095074847.1
Tenacibaculum jejuense
Min-wnt CC-tail

NVK51926.1
Flavobacteriaceae bacterium
Min-wnt CC-tail

MAC94969.1
Flavobacteriales bacterium
Min-wnt CC-tail

WP_120185852.1
Tenacibaculum lutimaris
Min-wnt CC-tail

NCT08740.1
Flavobacteriia bacterium
Min-wnt CC-tail

WP_105022019.1
Polaribacter glomeratus
Min-wnt CC-tail

WP_100946674.1
Polaribacter sp. ALD11
Min-wnt CC-tail

HBX50885.1
Bacteroidales bacterium
Min-wnt CC-tail

PCJ25046.1
Flavobacteriales bacterium
Min-wnt CC-tail

PIQ14364.1
Flavobacteriales bacterium CG18_big_fil_WC_8_21_14_2_50_32_9
Min-wnt CC-tail

WP_125066378.1
Tenacibaculum singaporense
Min-wnt CC-tail

WP_099214986.1
Tenacibaculum discolor
Min-wnt CC-tail

MBK7886046.1
Bacteroidota bacterium
Min-wnt CC-tail

WP_015481371.1
Polaribacter sp. MED152
Min-wnt CC-tail

WP_113786211.1
Tenacibaculum halocynthiae
Min-wnt CC-tail

MCB0459287.1
Flavobacteriaceae bacterium
Min-wnt CC-tail

MBR4266246.1
Bacteroidales bacterium
Min-wnt CC-tail

OGS73948.1
Flavobacteria bacterium RIFCSPLOWO2_12_FULL_35_11
Min-wnt CC-tail

WP_115881866.1
Lutibacter oceani
Min-wnt CC-tail

PKP13164.1
Bacteroidetes bacterium HGW-Bacteroidetes-3
Min-wnt CC-tail

OFX28543.1
Bacteroidetes bacterium GWA2_32_17
Min-wnt CC-tail

WP_160633021.1
Acidiluteibacter ferrifornacis
Min-wnt CC-tail

WP_218732016.1	*Polaribacter sp. HaHaR_3_91*	Min-wnt CC-tail
WP_093670824.1	*Tenacibaculum sp. MAR_2009_124*	Min-wnt CC-tail
MBL4642721.1	*Flavobacteriaceae bacterium*	Min-wnt CC-tail
MBV6485765.1	*Flavobacteriales bacterium*	Min-wnt CC-tail
MBK7681056.1	*Bacteroidota bacterium*	Min-wnt CC-tail
WP_197437802.1	*Polaribacter sp. BAL334*	Min-wnt CC-tail
RLD46093.1	*Bacteroidota bacterium*	Min-wnt CC-tail
OFY37444.1	*Bacteroidetes bacterium GWF2_38_335*	Min-wnt CC-tail
MBL7901177.1	*Bacteroidia bacterium*	Min-wnt CC-tail
MBK8806701.1	*Bacteroidales bacterium*	Min-wnt CC-tail
WP_086029779.1	*Tenacibaculum holothuriorum*	Min-wnt CC-tail
HFG82817.1		Min-wnt CC-tail
WP_026776266.1	*Polaribacter sp. Hel_I_88*	Min-wnt CC-tail
MBP1646021.1	*Bacteroidota bacterium*	Min-wnt CC-tail
NEW80405.1	*Gelidibacter sp.*	Min-wnt CC-tail
HIA37132.1	*Flavobacteriales bacterium*	Min-wnt CC-tail
MBO6117412.1	*Bacteroidales bacterium*	Min-wnt CC-tail
MBC7694590.1	*Bacteroidia bacterium*	Min-wnt CC-tail
MBK6983449.1	*Bacteroidota bacterium*	Min-wnt CC-tail
MBL0103056.1	*Bacteroidota bacterium*	Min-wnt CC-tail
MBN8695019.1	*Bacteroidota bacterium*	Min-wnt CC-tail
MBI3518362.1	*Bacteroidota bacterium*	Min-wnt CC-tail
RLD42978.1	*Bacteroidota bacterium*	Min-wnt CC-tail
PID69729.1	*Flavobacteriales bacterium*	Min-wnt CC-tail
MBS1636759.1	*Bacteroidota bacterium*	Min-wnt CC-tail
NUM51418.1	*Flavobacteriales bacterium*	Min-wnt CC-tail
MBL7895996.1	*Bacteroidia bacterium*	Min-wnt CC-tail
MCD6067816.1	*Bacteroidota bacterium*	Min-wnt CC-tail
REK51504.1	*Bacteroidota bacterium*	Min-wnt CC-tail
KJS05875.1	*Flavobacteriales bacterium BRH_c54*	Min-wnt CC-tail

####;fused to an open beta-barrel

MAA80452.1	*Deltaproteobacteria bacterium*	open-beta-barrel Min-wnt
OHE23369.1	*Syntrophus sp. GWC2_56_31*	open-beta-barrel Min-wnt
NTV47549.1	*Chlorobiales bacterium*	open-beta-barrel Min-wnt
OQA57776.1	*Candidatus Omnitrophica bacterium ADurb.Bin277*	open-beta-barrel Min-wnt
RKT46056.1	*Thiocapsa rosea*	open-beta-barrel Min-wnt
MBF0216605.1	*Candidatus Omnitrophota bacterium*	open-beta-barrel Min-wnt
MBT5876446.1	*Candidatus Latescibacterota bacterium*	open-beta-barrel Min-wnt
KAA3604643.1	*Calditrichota bacterium*	open-beta-barrel Min-wnt
MBP7142267.1	*Opitutaceae bacterium*	open-beta-barrel Min-wnt
MBX7120784.1	*Opitutaceae bacterium*	open-beta-barrel Min-wnt
MBW2147936.1	*Deltaproteobacteria bacterium*	open-beta-barrel Min-wnt
MBU3759782.1	*Candidatus Omnitrophota bacterium*	open-beta-barrel Min-wnt
MBF0253684.1	*Candidatus Omnitrophota bacterium*	open-beta-barrel Min-wnt
WP_178931970.1		open-beta-barrel Min-wnt
NCA71436.1	*Sphingobacteriia bacterium*	open-beta-barrel Min-wnt
WP_093036542.1	*Thiocapsa roseopersicina*	open-beta-barrel Min-wnt
MBI4115145.1	*Candidatus Omnitrophota bacterium*	open-beta-barrel Min-wnt
MBA3016029.1	*Desulfobulbaceae bacterium*	open-beta-barrel Min-wnt
TVR50861.1	*Puniceicoccaceae bacterium*	open-beta-barrel Min-wnt
WP_200310518.1	*Persicirhabdus sediminis*	open-beta-barrel Min-wnt
MBN1567765.1	*Acidobacteriota bacterium*	open-beta-barrel Min-wnt
OGW87330.1	*Omnitrophica bacterium RIFCSPHIGHO2_02_FULL_46_11*	open-beta-barrel Min-wnt
OGW87315.1	*Omnitrophica bacterium RIFCSPLOWO2_01_FULL_45_10b*	open-beta-barrel Min-wnt
MCB0334294.1	*Bdellovibrionales bacterium*	open-beta-barrel Min-wnt
MBK8479384.1	*Opitutaceae bacterium*	open-beta-barrel Min-wnt
WP_018968713.1	*Rubritalea marina DSM 17716*	open-beta-barrel Min-wnt
RPH92671.1	*Calditrichota bacterium*	open-beta-barrel Min-wnt
MBL9205233.1	*Opitutaceae bacterium*	open-beta-barrel Min-wnt
WP_200389205.1	*Thiocapsa imhoffii*	open-beta-barrel Min-wnt
OGW84751.1	*Omnitrophica bacterium RIFCSPHIGHO2_02_FULL_46_11*	open-beta-barrel Min-wnt
WP_129589795.1	*Rubritalea profundi*	open-beta-barrel Min-wnt
RME73237.1	*Verrucomicrobiota bacterium*	open-beta-barrel Min-wnt
MAE65840.1	*Phycisphaeraceae bacterium*	open-beta-barrel Min-wnt
MBT9439492.1	*Desulfobacterales bacterium*	open-beta-barrel Min-wnt
PIU40209.1	*Candidatus Omnitrophica bacterium CG07_land_8_20_14_0_80_50_8*	open-beta-barrel Min-wnt
RLD23094.1	*Bacteroidota bacterium*	open-beta-barrel Min-wnt
NJN27320.1	*Cyclobacteriaceae bacterium*	open-beta-barrel Min-wnt
MBN1466363.1	*Candidatus Zhuqueibacterota bacterium*	open-beta-barrel Min-wnt
MBE7538033.1		open-beta-barrel Min-wnt
MBN1558755.1	*Candidatus Zhuqueibacterota bacterium*	open-beta-barrel Min-wnt
MBC9872755.1		open-beta-barrel Min-wnt
WP_148071664.1	*Bythopirellula goksoeyrii*	open-beta-barrel Min-wnt
WP_146451979.1	*Bythopirellula polymerisocia*	open-beta-barrel Min-wnt
MBC9888295.1	*Opitutia bacterium*	open-beta-barrel Min-wnt
MBH54384.1	*Opitutaceae bacterium*	open-beta-barrel Min-wnt
NQU95834.1	*Candidatus Omnitrophota bacterium*	open-beta-barrel Min-wnt
OGW75637.1	*Omnitrophica bacterium RBG_13_46_9*	open-beta-barrel Min-wnt
MCB1121479.1	*Verrucomicrobiia bacterium*	open-beta-barrel Min-wnt
MBP9866148.1	*Candidatus Omnitrophota bacterium*	open-beta-barrel Min-wnt
HCM42699.1	*Candidatus Omnitrophota bacterium*	open-beta-barrel Min-wnt

####;single additional, C-terminal helix

MBC7999242.1	*Candidatus Melainabacteria bacterium*	Min-wnt helix
MBN9393650.1	*Candidatus Melainabacteria bacterium*	Min-wnt helix
MCC6979962.1	*Candidatus Melainabacteria bacterium*	Min-wnt helix
MBK8223750.1	*Candidatus Obscuribacter sp.*	Min-wnt helix
MBK8224133.1	*Candidatus Obscuribacter sp.*	Min-wnt helix
MBK9620514.1	*Candidatus Obscuribacter sp.*	Min-wnt helix
MBX3074428.1	*Candidatus Obscuribacterales bacterium*	Min-wnt helix
MBX9939173.1	*Candidatus Obscuribacterales bacterium*	Min-wnt helix
MBX9951118.1	*Candidatus Obscuribacterales bacterium*	Min-wnt helix
MBX9770880.1	*Candidatus Obscuribacterales bacterium*	Min-wnt helix
MBY0358849.1	*Candidatus Obscuribacterales bacterium*	Min-wnt helix
MBY0547493.1	*Candidatus Obscuribacterales bacterium*	Min-wnt helix

MCB9469215.1	*Candidatus Obscuribacterales bacterium*	Min-wnt · helix
MCA9805738.1	*Cyanobacteria bacterium HKST-UBA02*	Min-wnt · helix
MBA4074806.1	*Cyanobacteria bacterium PR.023*	Min-wnt · helix
MBA3855807.1	*Cyanobacteria bacterium PR.3.49*	Min-wnt · helix
MBU6452801.1	*Cyanobacteria bacterium REEB67*	Min-wnt · helix
MBS2005454.1	*Cyanobacteria bacterium SZAS TMP-1*	Min-wnt · helix
MBS2008129.1	*Cyanobacteria bacterium SZAS TMP-1*	Min-wnt · helix
MBP7863563.1	*bacterium*	Min-wnt · helix
MBP9089629.1	*bacterium*	Min-wnt · helix
MBP9094186.1	*bacterium*	Min-wnt · helix
OPZ90265.1	*bacterium ADurb.Bin425*	Min-wnt · helix
HAK77058.1	*Runella sp.*	Min-wnt · beta-helix
WP_063006252.1	*Nocardia salmonicida subsp. cummidelens NBRC 100378*	Min-wnt · Min-wnt · extended-tail

####;other versions

WP_167267454.1	*Dyadobacter arcticus*	beta-sand · Min-wnt
WP_188932862.1	*Dyadobacter endophyticus*	beta-sand · Min-wnt
WP_234645914.1	*Dyadobacter sp. CY356*	beta-sand · Min-wnt

####;membrane-anchored versions lacking clear immunity protein signal

HCS88018.1	*Bacteroidales bacterium*	helical-region · Min-wnt
WP_109301702.1	*Aquimarina sp. AU474*	helical-region · Min-wnt
CAA9346664.1	*uncultured Gemmatimonadota bacterium*	helical-region · Min-wnt
MBQ5958943.1	*Bacteroidales bacterium*	helical-region · Min-wnt
MBQ8223186.1	*Bacteroidales bacterium*	helical-region · Min-wnt
HAQ37700.1	*Saprospirales bacterium*	helical-region · Min-wnt
MBR6686079.1	*Paludibacteraceae bacterium*	helical-region · Min-wnt
MBO8460020.1	*Candidatus Gallipaludibacter merdavium*	helical-region · Min-wnt
MBQ8956995.1	*Bacteroidales bacterium*	helical-region · Min-wnt
MBR6776016.1	*Bacteroidales bacterium*	helical-region · Min-wnt
MBO7233513.1	*Paludibacteraceae bacterium*	helical-region · Min-wnt
MBP3586647.1	*Paludibacteraceae bacterium*	helical-region · Min-wnt
MBO5974839.1	*Paludibacteraceae bacterium*	helical-region · Min-wnt
MBO7724280.1	*Paludibacteraceae bacterium*	helical-region · Min-wnt
MBO5086826.1	*Paludibacteraceae bacterium*	helical-region · Min-wnt
MBE6311997.1	*Bacteroidales bacterium*	helical-region · Min-wnt
MBR2450536.1	*Paludibacteraceae bacterium*	helical-region · Min-wnt
MBN2663636.1	*Bacteroidales bacterium*	helical-region · Min-wnt
MBO5673710.1	*Paludibacteraceae bacterium*	helical-region · Min-wnt
MBR5855305.1	*Paludibacteraceae bacterium*	helical-region · Min-wnt
MBR5912607.1	*Bacteroidales bacterium*	helical-region · Min-wnt
MBL6663806.1	*Flavobacteriales bacterium*	helical-region · Min-wnt
MAD50149.1	*Flavobacteriales bacterium*	helical-region · Min-wnt
MBO7255211.1	*Paludibacteraceae bacterium*	helical-region · Min-wnt
MCE1155467.1	*Bacteroidales bacterium*	helical-region · Min-wnt
MCB0737370.1	*Bacteroidota bacterium*	helical-region · Min-wnt
MBP6356104.1	*Paludibacter sp.*	helical-region · Min-wnt
HAR20926.1	*Cryomorphaceae bacterium*	helical-region · Min-wnt
HAR22357.1	*Cryomorphaceae bacterium*	helical-region · Min-wnt
MBQ8957810.1	*Bacteroidales bacterium*	helical-region · Min-wnt
MBT7659645.1	*Bacteroidota bacterium*	helical-region · Min-wnt
WP_038538372.1	*endosymbiont of Acanthamoeba sp. UWC8*	LysM · Min-wnt
WP_069841533.1	*Roseivirga sp. 4D4*	helical-domain · Min-wnt

#;N-terminal bi-helix configuration and C-terminal lipocalin-like domain

RYG06515.1	*Chitinophagaceae bacterium*	bi-helix · Min-wnt · Lipocalin-like
MBT1690673.1	*Dawidia soli*	bi-helix · Min-wnt · Lipocalin-like
WP_101728303.1	*Emticicia sp. TH156*	bi-helix · Min-wnt · Lipocalin-like
PKA98336.1	*Flavobacteriaceae bacterium MAR_2009_75*	bi-helix · Min-wnt · Lipocalin-like
WP_232179085.1	*Mucilaginibacter roseus*	bi-helix · Min-wnt · Lipocalin-like
KQP12035.1	*Pseudorhodoferax sp. Leaf265*	bi-helix · Min-wnt · Lipocalin-like
WP_225412036.1	*Stigmatella hybrida*	bi-helix · Min-wnt · Lipocalin-like

######################;Cystatin-fold domain (FD)-fused Min-wnts

PHS10306.1	*Kordia sp.*	Cystatin-FD · Min-wnt · Jellyroll-like-Imm-Min-wnt-like
SFN73606.1	*Algoriella xinjiangensis*	Cystatin-FD · Min-wnt · Jellyroll-like-Imm-Min-wnt-like
KAB8152947.1	*Kordia sp. TARA_039_SRF*	Cystatin-FD · Min-wnt · Jellyroll-like-Imm-Min-wnt-like
WP_188621932.1	*Flavobacterium suaedae*	Cystatin-FD · Min-wnt · Jellyroll-like-Imm-Min-wnt-like
WP_126745293.1	*Flavobacterium sp. GT3R68*	Cystatin-FD · Min-wnt · Jellyroll-like-Imm-Min-wnt-like
WP_198629127.1	*Salegentibacter maritimus*	Cystatin-FD · Min-wnt · Jellyroll-like-Imm-Min-wnt-like
OBX26441.1	*Gelidibacter algens*	Cystatin-FD · Min-wnt · BamE-like-Imm-Min-wnt
WP_199603112.1	*Gelidibacter salicanalis*	Cystatin-FD · Min-wnt · Jellyroll-like-Imm-Min-wnt-like
MBP9602236.1	*Lutibacter sp.*	Cystatin-FD · Min-wnt
PZR21233.1	*Flavobacterium psychrophilum*	Cystatin-FD · Min-wnt · Jellyroll-like-Imm-Min-wnt-like
WP_108115408.1	*Kordia periserrulae*	Cystatin-FD · Min-wnt · Jellyroll-like-Imm-Min-wnt-like · Jellyroll-like-Imm-Min-wnt-like
WP_187967619.1	*Sinomicrobium weinanense*	Cystatin-FD · Min-wnt · Jellyroll-like-Imm-Min-wnt-like
WP_215929046.1	*Winogradskyella psychrotolerans*	Cystatin-FD · Min-wnt · Jellyroll-like-Imm-Min-wnt-like
MCE2935232.1	*Flammeovirgaceae bacterium*	Cystatin-FD · Min-wnt · Jellyroll-like-Imm-Min-wnt-like
HEA20070.1	*Pricia antarctica*	Cystatin-FD · Min-wnt · Jellyroll-like-Imm-Min-wnt-like
WP_146834201.1	*Antarcticibacterium arcticum*	Cystatin-FD · Min-wnt
MBX7089883.1	*Cyclobacteriaceae bacterium*	Cystatin-FD · Min-wnt · BamE-like-Imm-Min-wnt
TRV69854.1	*Microcystis panniformis Mp_MB_F_20051200_S9D*	Cystatin-FD · Min-wnt
WP_099574008.1	*Maribacter sp. 4U21*	Cystatin-FD · Min-wnt · Jellyroll-like-Imm-Min-wnt-like
WP_073192661.1	*Psychroflexus salarius*	Cystatin-FD · Min-wnt · Jellyroll-like-Imm-Min-wnt-like · ? · ? · ? · ? · BamE-like-Imm-Min-wnt
PWI29877.1	*Flavobacteriaceae bacterium LYZ1037*	Cystatin-FD · Min-wnt · Jellyroll-like-Imm-Min-wnt-like
WP_068206098.1	*Lutibacter profundi*	Cystatin-FD · Min-wnt · Jellyroll-like-Imm-Min-wnt-like
MBN4047100.1	*bacterium AH-315-P13*	Cystatin-FD · Min-wnt · Jellyroll-like-Imm-Min-wnt-like · Jellyroll-like-Imm-Min-wnt-like
WP_235127642.1	*Dyadobacter sp. CY347*	Cystatin-FD · Min-wnt
RZL61643.1	*Pedobacter sp.*	Cystatin-FD · Min-wnt
WP_164122756.1	*Sphingobacterium sp. xlx-130*	Cystatin-FD · Min-wnt · Jellyroll-like-Imm-Min-wnt-like
WP_075350341.1	*Algoriphagus marinus*	Cystatin-FD · Min-wnt · Jellyroll-like-Imm-Min-wnt-like · Jellyroll-like-Imm-Min-wnt-like
WP_115970832.1	*Chryseobacterium pennae*	Cystatin-FD · Min-wnt(frag)
WP_115971324.1	*Chryseobacterium pennae*	Cystatin-FD · Min-wnt · Jellyroll-like-Imm-Min-wnt-like
WP_233755821.1	*Algoriphagus sp. AGSA1*	Cystatin-FD · Min-wnt
ODT53314.1	*Niastella sp. SCN 39-18*	Cystatin-FD · Min-wnt · Jellyroll-like-Imm-Min-wnt-like
WP_123204570.1	*Pedobacter jejuensis*	Cystatin-FD · Min-wnt · BamE-like-Imm-Min-wnt
MCC6307428.1	*Chitinophagaceae bacterium*	Cystatin-FD · Min-wnt · Jellyroll-like-Imm-Min-wnt-like
WP_139010570.1	*Dyadobacter flavalbus*	Cystatin-FD · Min-wnt

WP_090337864.1
Dyadobacter koreensis
Cystatin-FD | Min-wnt

WP_114067695.1
Runella rosea
Cystatin-FD | Min-wnt

PIQ22137.1
Cytophagales bacterium CG18_big_fil_WC_8_21_14_2_50_42_9
Cystatin-FD | Min-wnt | BamE-like-Imm-Min-wnt

HHH54654.1
Bacteroidota bacterium
Cystatin-FD | ?? | Min-wnt

WP_128389495.1
Cystatin-FD | Min-wnt | Min-wnt

######################;broken-hairpin fused Min-wnts; #;Min-wnt+broken-hairpin

WP_213609117.1
Roseococcus sp. SDR
Min-wnt | broken-hairpin

MBY0332355.1
Acetobacteraceae bacterium
Min-wnt | broken-hairpin

OHC80803.1
Rhodospirillales bacterium RIFCSPLOWO2_12_FULL_67_15
Min-wnt | broken-hairpin

MBL8834572.1
Rhodospirillales bacterium
Min-wnt | broken-hairpin

MBL8832351.1
Rhodospirillales bacterium
Min-wnt | broken-hairpin

MBM3565434.1
Alphaproteobacteria bacterium
Min-wnt | broken-hairpin

TAH34016.1
Alphaproteobacteria bacterium
Min-wnt | broken-hairpin

WP_213609990.1
Roseococcus sp. SDR
Min-wnt | broken-hairpin

MBR1907564.1
bacterium
Min-wnt | broken-hairpin

HIQ89771.1
Candidatus Galligastranaerophilus faecipullorum
Min-wnt | broken-hairpin

MBR1425248.1
bacterium
Min-wnt | broken-hairpin

MBO7672200.1
bacterium
Min-wnt | broken-hairpin

MBR1775717.1
bacterium
Min-wnt | broken-hairpin

MBR6127983.1
bacterium
Min-wnt | broken-hairpin

MBO6088658.1
bacterium
Min-wnt | broken-hairpin

DAA80745.1
Candidatus Gastranaerophilales bacterium HUM_2
Min-wnt | broken-hairpin

MBE7709541.1
Cyanobacteria bacterium SIG32
Min-wnt | broken-hairpin

MBI1363813.1
Pseudomonadota bacterium
Min-wnt | broken-hairpin

DAU57126.1
Min-wnt | broken-hairpin

MBD9223168.1
bacterium
Min-wnt | broken-hairpin

MCA4920503.1
Roseomonas sp.
Min-wnt | broken-hairpin

PIZ30748.1
Alphaproteobacteria bacterium CG_4_10_14_0_8_um_filter_53_9
Min-wnt | broken-hairpin

OLA94577.1
Candidatus Melainabacteria bacterium LEY3_CP_29_8
Min-wnt | broken-hairpin

MBO6181535.1
bacterium
Min-wnt | broken-hairpin

CCY23582.1
Brachyspira sp. CAG:484
Min-wnt | broken-hairpin

MBO5948269.1
bacterium
Min-wnt | broken-hairpin

##################;systems with toxins potentially directed in intracellular attack; ####;DUF3892 fusion

NQY74961.1
Candidatus Margulisiibacteriota bacterium
Min-wnt | DUF3892

WP_102242990.1
Bacteriovorax stolpii
Min-wnt | DUF3892

MBT6120324.1
bacterium
Min-wnt | DUF3892

MCE3013561.1
Pseudomonadota bacterium
TM-Imm-Wnt | Min-wnt | DUF3892

RAP33302.1
Candidatus Marinamargulisbacteria bacterium SCGC AG-439-L15
Min-wnt | DUF3892

NDB03546.1
Flavobacteriaceae bacterium
Min-wnt | DUF3892

MBT6120221.1
bacterium
BamE-like-Imm-Min-wnt | Min-wnt | DUF3892

MBP9674596.1
Bacteriovoracaceae bacterium
Min-wnt | DUF3892

MBL7664527.1
Bacteriovoracaceae bacterium
Min-wnt | DUF3892

MBU6154686.1
Bdellovibrionales bacterium
Min-wnt | DUF3892

MBT3235097.1
Bdellovibrionales bacterium
Min-wnt | DUF3892

MBT4791877.1
Halobacteriovoraceae bacterium
Min-wnt | DUF3892

GBR76018.1
Candidatus Termititenax persephonae
Min-wnt | DUF3892

MBT6120823.1
bacterium
Min-wnt | DUF3892

MBT3261272.1
bacterium
Min-wnt | DUF3892

MCB9095050.1
Halobacteriovoraceae bacterium
Min-wnt | DUF3892

MBM4252957.1
Deltaproteobacteria bacterium
Min-wnt | DUF3892

####;membrane-anchored and fused to a 3-stranded-meander.; ####;associates in genome neighborhood with membrane-bound run of pentapeptide repeats and secreted, reported alpha-L rhamnosidase

PCJ66457.1
Bacteroidota bacterium
glycosyl-hydrolase | 3-strand-meander | Min-wnt | PPTs | alpha-L-rhamnosidase-like

HAP01991.1
Bacteroidota bacterium
3-strand-meander | Min-wnt | PPTs

HAY89416.1
Bacteroidota bacterium
3-strand-meander | Min-wnt | alpha-L-rhamnosidase-like

RMG75404.1
Bacteroidota bacterium
3-strand-meander | Min-wnt | PPTs

NBV57460.1
Bacteroidota bacterium
glycosyl-hydrolase | 3-strand-meander | Min-wnt | alpha-L-rhamnosidase-like

HIF13902.1
Bacteroidota bacterium
alpha-L-rhamnosidase-like | glycosyl-hydrolase | 3-strand-meander | Min-wnt | PPTs

GDX52385.1
Bacteroidota bacterium
glycosyl-hydrolase | 3-strand-meander | Min-wnt | PPTs

MBI1307688.1
Bacteroidota bacterium
glycosyl-hydrolase | 3-strand-meander | Min-wnt | PPTs | alpha-L-rhamnosidase-like

MBL0020461.1
Bacteroidota bacterium
3-strand-meander | Min-wnt | PPTs | PPTs

MBS1658993.1
Bacteroidota bacterium
alpha-L-rhamnosidase-like | glycosyl-hydrolase | 3-strand-meander | Min-wnt | PPTs

MCB0837552.1
Bacteroidota bacterium
3-strand-meander | Min-wnt | PPTs

MBL6730440.1
Bacteroidia bacterium
glycosyl-hydrolase | 3-strand-meander | Min-wnt | alpha-L-rhamnosidase-like

MBO6516578.1
Bacteroidia bacterium
3-strand-meander | Min-wnt | PPTs | alpha-L-rhamnosidase-like

MBT8233376.1
Bacteroidia bacterium
alpha-L-rhamnosidase-like | glycosyl-hydrolase | 3-strand-meander | Min-wnt | PPTs

MBK7242586.1
Candidatus Defluviibacterium haderslevense
glycosyl-hydrolase | 3-strand-meander | Min-wnt | PPTs | alpha-L-rhamnosidase-like

MBL0235969.1
Candidatus Defluviibacterium haderslevense
3-strand-meander | Min-wnt | PPTs

MBK9103252.1
Candidatus Opimibacter iunctus
alpha-L-rhamnosidase-like | glycosyl-hydrolase | 3-strand-meander | Min-wnt | PPTs

MBL0009689.1
Candidatus Opimibacter skivensis
alpha-L-rhamnosidase-like | glycosyl-hydrolase | 3-strand-meander | Min-wnt | PPTs

MBK9981111.1
Candidatus Opimibacter skivensis
alpha-L-rhamnosidase-like | glycosyl-hydrolase | 3-strand-meander | Min-wnt | PPTs

MBX7141218.1
Chitinophagales bacterium
alpha-L-rhamnosidase-like | glycosyl-hydrolase | 3-strand-meander | Min-wnt | PPTs

MBT4774745.1
Crocinitomicaceae bacterium
glycosyl-hydrolase | 3-strand-meander | Min-wnt | PPTs | alpha-L-rhamnosidase-like

MAY83550.1
Flavobacteriales bacterium
glycosyl-hydrolase | 3-strand-meander | Min-wnt | PPTs | alpha-L-rhamnosidase-like

HAE32175.1
Flavobacteriales bacterium
glycosyl-hydrolase | 3-strand-meander | Min-wnt | PPTs

HAW19093.1
Flavobacteriales bacterium
3-strand-meander | Min-wnt | PPTs

NQV51672.1
Flavobacteriales bacterium
glycosyl-hydrolase | ? | 3-strand-meander | Min-wnt | PPTs

MBT3647031.1
Flavobacteriales bacterium
glycosyl-hydrolase | 3-strand-meander | Min-wnt

MCB9240928.1
Flavobacteriales bacterium
glycosyl-hydrolase | 3-strand-meander | Min-wnt | PPTs | alpha-L-rhamnosidase-like

MCB9262534.1
Flavobacteriales bacterium
3-strand-meander | Min-wnt | PPTs | alpha-L-rhamnosidase-like

WP_235295626.1
Portibacter marinus
glycosyl-hydrolase | 3-strand-meander | Min-wnt | PPTs

NNE26463.1
Saprospiraceae bacterium
alpha-L-rhamnosidase-like | glycosyl-hydrolase | 3-strand-meander | Min-wnt | PPTs | ? | PPTs

NOT37112.1
Saprospiraceae bacterium
glycosyl-hydrolase | 3-strand-meander | Min-wnt | PPTs | alpha-L-rhamnosidase-like

MBK7358125.1
Saprospiraceae bacterium
glycosyl-hydrolase | 3-strand-meander | Min-wnt | ? | alpha-L-rhamnosidase-like

MBK9221829.1
Saprospiraceae bacterium
glycosyl-hydrolase | 3-strand-meander | Min-wnt | PPTs | ? | alpha-L-rhamnosidase-like

MBL7817371.1
Saprospiraceae bacterium
3-strand-meander | Min-wnt | PPTs

MBP6680741.1
Saprospiraceae bacterium
glycosyl-hydrolase | 3-strand-meander | Min-wnt | PPTs

MBV6472645.1
Saprospiraceae bacterium
3-strand-meander | Min-wnt | PPTs

MBX7176553.1
Saprospiraceae bacterium
glycosyl-hydrolase | 3-strand-meander | Min-wnt | PPTs

MCB0658157.1
Saprospiraceae bacterium
alpha-L-rhamnosidase-like | glycosyl-hydrolase | 3-strand-meander | Min-wnt | PPTs

MCC6815107.1
Saprospiraceae bacterium
3-strand-meander | Min-wnt | PPTs

MBC7774907.1
Saprospiraceae bacterium
3-strand-meander | Min-wnt | PPTs

MBD98573.1
Verrucomicrobiota bacterium
glycosyl-hydrolase | 3-strand-meander | Min-wnt | PPTs

HAV26180.1
Bacteroidota bacterium
Min-wnt | PPTs | alpha-L-rhamnosidase-like

#;comparable fusions to the above

WP_189077204.1
Mangrovihabitans endophyticus
glycosyl-hydrolase | beta-rich | beta-rich | two-layer-beta | Min-wnt

WP_062721403.1
Streptomyces caeruleatus
glycosyl-hydrolase | beta-rich | beta-rich | two-layer-beta | Min-wnt

WP_062710976.1
Streptomyces regalis
glycosyl-hydrolase | beta-rich | beta-rich | two-layer-beta | Min-wnt

WP_181383867.1
Streptomyces sp. NWU339
glycosyl-hydrolase | beta-rich | beta-rich | two-layer-beta | Min-wnt

WP_037891817.1
Streptomyces viridochromogenes Tue57
glycosyl-hydrolase | beta-rich | beta-rich | two-layer-beta | Min-wnt

####;virus-encoded versions. fusions to distinctive GNAT domain, could point to intra-host conflict role.

DAL68673.1
extension | Min-wnt | BamE-like-Imm-Min-wnt

CAB4240556.1
uncultured Caudovirales phage
Min-wnt

CAB4173509.1
uncultured Caudovirales phage
Min-wnt

CAB4191049.1
uncultured Caudovirales phage
Min-wnt

CAB4215518.1
uncultured Caudovirales phage
Min-wnt

CAB5222757.1
uncultured Caudovirales phage
Min-wnt

CAB4154988.1
uncultured Caudovirales phage
coiled-coil | disordered | Min-wnt | midX | PcfJ-GNAT

CAB5224403.1
uncultured Caudovirales phage
coiled-coil | disordered | Min-wnt | midX | PcfJ-GNAT

CAB4122792.1
uncultured Caudovirales phage
Min-wnt | midX | PcfJ-GNAT

CAB4130885.1
uncultured Caudovirales phage
Min-wnt | midX | PcfJ-GNAT

QGH72181.1
Caudovirales sp. ctOwN3
helical-region | GNAT | midX2 | Min-wnt

#####################;rare fusions

WP_109598115.1
Microvirga sp. 17 mud 1-3
Min-wnt | HINT

QKK06958.1
Planctomycetota bacterium
Min-wnt | EFHAND | Imm | Imm

MCB9881206.1
Planctomycetota bacterium
Concanavalin-like | Min-wnt

NOT78922.1
Bacteriovoracaceae bacterium
Min-wnt | ANK

MCB0421258.1
Bdellovibrionales bacterium
HTH | Min-wnt

WP_202222082.1
Okeania sp. KiyG1
PPC | GBD-PPC | Min-wnt

WP_186398064.1
Stappia sp. P2PMeth1
Min-wnt | EFHAND

WP_070920415.1
Mycobacteroides chelonae
MPTase | Min-wnt

WP_083873202.1
Nocardia testacea NBRC 100365
HTH | Min-wnt

NRA87332.1
Hyphomicrobiales bacterium
Min-wnt

WP_234705188.1
Sinorhizobium meliloti Mlalz-1
Min-wnt

####################;versions with no clear means of secretion from their contexts

WP_011144708.1
Photorhabdus laumondii
coiled-coil | Min-wnt

MBT6377503.1
Flavobacterium sp.
BamE-like-Imm-Min-wnt | disordered | Min-wnt | BamE-like-Imm-Min-wnt

WP_082399502.1
helical-region | Min-wnt

WP_105408076.1
Paenibacillus sp. PCH8
extended-helix | minimal-helical-region | Min-wnt

WP_139277123.1
Salegentibacter echinorum
extended-helix | minimal-helical-region | Min-wnt | Jellyroll-like-Imm-Min-wnt-like

WP_144612392.1
Brevibacillus brevis
extended-helix | minimal-helical-region | Min-wnt

WP_218830642.1
Terribacillus saccharophilus
extended-helix | minimal-helical-region | Min-wnt

HHN36417.1
disordered | Min-wnt | Jellyroll-like-Imm-Min-wnt-like

MBK8710280.1
Niastella sp.
disordered | Min-wnt | Jellyroll-like-Imm-Min-wnt-like

MBE6293342.1
Bacteroidales bacterium
disordered | Min-wnt | BamE-like-Imm-Min-wnt

WP_181841636.1
Emticicia sp. BO119
disordered | Min-wnt | BamE-like-Imm-Min-wnt

MCB0877755.1
Thermoleophilia bacterium
disordered | Min-wnt

WP_183417847.1
Limibacillus halophilus
disordered | Min-wnt

WP_159080181.1
Nocardia suismassiliense
disordered | Min-wnt

NJB38207.1
Croceivirga sp. JEA036
disordered | Min-wnt | Jellyroll-like-Imm-Min-wnt-like

QBQ59421.1
Saccharomonospora xinjiangensis
disordered | Min-wnt | BamE-like-Imm-Min-wnt

ARV64513.1
Corynebacterium glutamicum
disordered | Min-wnt | BamE-like-Imm-Min-wnt

WP_089064943.1
Brachybacterium avium
disordered | Min-wnt | BamE-like-Imm-Min-wnt

WP_235853205.1
extended-helix | Min-wnt

WP_184487309.1
Neoroseomonas alkaliterrae
Min-wnt | disordered

MCB0878151.1
Thermoleophilia bacterium
Min-wnt | disordered

MBZ0148532.1
Pseudorhodoplanes sp.
Min-wnt | disordered

MCE1176468.1
Burkholderiales bacterium
Min-wnt | disordered

WP_003437802.1
Psychroflexus gondwanensis ACAM 44
disordered | Min-wnt | BamE-like-Imm-Min-wnt

WP_235881962.1
Mycolicibacterium vanbaalenii
disordered | Min-wnt

WP_159421612.1
[Zimmermannella] alba NBRC 15616
disordered | Min-wnt

PUU51032.1
Sinorhizobium medicae
disordered | Min-wnt

WP_080147006.1
Marinilactibacillus piezotolerans
disordered | Min-wnt

RGP48603.1
Rhodococcus erythropolis
disordered | Min-wnt

WP_225166545.1
disordered | Min-wnt

WP_137402100.1
Echinicola rosea
disordered | Min-wnt

WP_162633098.1
Echinicola strongylocentroti
helical | Min-wnt

OEJ99376.1
Flavivirga aquatica
extended-loop | Min-wnt

MBR1703978.1
Clostridia bacterium
extended-loop | Min-wnt

WP_106655149.1
Brevibacillus brevis
extended-loop | Min-wnt

MCC6230925.1
Phycisphaerales bacterium
extended-loop | Min-wnt

WP_235124081.1
Dyadobacter sp. CY347
extended-loop | Min-wnt | BamE-like-Imm-Min-wnt

WP_168703729.1
Gordonia paraffinivorans
extended-loop | Min-wnt

WP_087520383.1
Polaribacter sp. SA4-12
extended-loop | Min-wnt | BamE-like-Imm-Min-wnt

WP_147120685.1
Polaribacter sp. IC066
extended-loop | Min-wnt

MCB1167194.1
Leptospiraceae bacterium
extended-loop | Min-wnt

WP_028872776.1
Psychroserpens burtonensis
extended-loop | Min-wnt | BamE-like-Imm-Min-wnt

OHC83834.1
Rhodospirillales bacterium RIFCSPLOWO2_12_FULL_67_15
extended-loop | Min-wnt

MBT9559813.1
Myxococcales bacterium
extended-loop | Min-wnt

WP_235179042.1
Dyadobacter chenhuakuii
extended-loop | Min-wnt | BamE-like-Imm-Min-wnt

OYQ34518.1
Niveispirillum lacus
extended-loop | Min-wnt

NJL25504.1
Calothrix sp. SM1_5_4
extended-loop | Min-wnt | BamE-like-Imm-Min-wnt

WP_232142450.1
Luteimonas sp. Y-2-2-4F
BamE-like-Imm-Min-wnt | Min-wnt | helical-region

WP_192279183.1
Xanthomonas sp. XNM01
BamE-like-Imm-Min-wnt | Min-wnt | helical-region

PZO63856.1
Pseudoxanthomonas suwonensis
Min-wnt | helical-region

WP_213945600.1
Litoribacter ruber
Min-wnt | Jellyroll-like-Imm-Min-wnt-like

MBK6906589.1
Rhodocyclaceae bacterium
Min-wnt

MBK9959566.1
Chitinophagaceae bacterium
Min-wnt

TAJ44407.1
Chitinophagaceae bacterium
Min-wnt | Jellyroll-like-Imm-Min-wnt-like

MBK8774264.1
Chitinophagaceae bacterium
Min-wnt

MBS1924959.1
Bacteroidota bacterium
Min-wnt

MBS1761760.1
Bacteroidota bacterium
Min-wnt

WP_117898125.1
Ruminococcus sp. AF17-1AC
Min-wnt | imm-wnt

MBO6140976.1
Pseudomonadota bacterium
BamE-like-Imm-Min-wnt | Min-wnt | disordered

PZO62884.1
Pseudoxanthomonas suwonensis
BamE-like-Imm-Min-wnt | Min-wnt

MBD5401288.1
bacterium
Min-wnt

MCD8378167.1
Candidatus Gastranaerophilales bacterium
Min-wnt

OHQ25414.1
Neisseria sp. HMSC066H01
Min-wnt

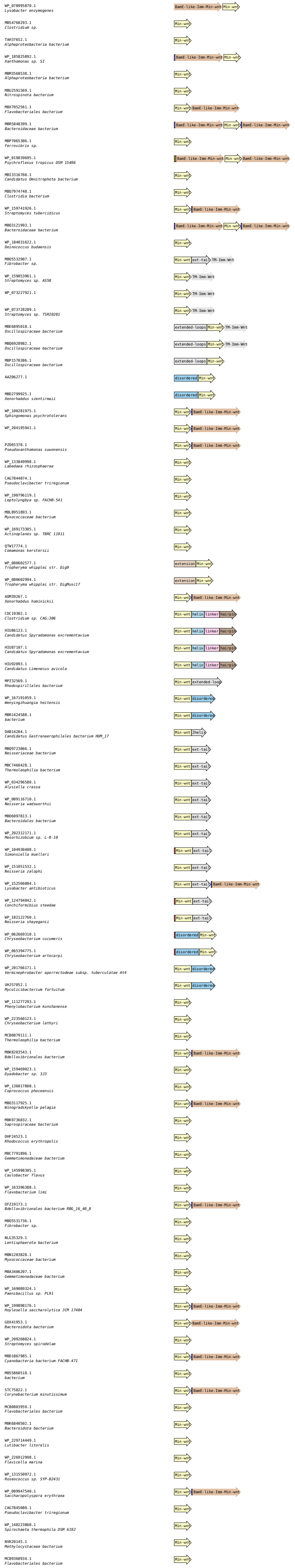

WP_078995870.1
Lysobacter enzymogenes
BamE-like-Imm-Min-wnt Min-wnt
MBS4760293.1
Clostridium sp.
Min-wnt
TAH37652.1
Alphaproteobacteria bacterium
Min-wnt
WP_185825892.1
Xanthomonas sp. SI
BamE-like-Imm-Min-wnt Min-wnt
MBM3568538.1
Alphaproteobacteria bacterium
Min-wnt
MBU2591569.1
Nitrospinota bacterium
Min-wnt
MBX7052561.1
Flavobacteriales bacterium
Min-wnt BamE-like-Imm-Min-wnt
MBR5848399.1
Bacteroidaceae bacterium
BamE-like-Imm-Min-wnt Min-wnt BamE-like-Imm-Min-wnt
MBP7065306.1
Ferrovibrio sp.
Min-wnt
WP_019039695.1
Psychroflexus tropicus DSM 15496
BamE-like-Imm-Min-wnt Min-wnt BamE-like-Imm-Min-wnt
MBI3316760.1
Candidatus Omnitrophota bacterium
Min-wnt
MBQ7974748.1
Clostridia bacterium
Min-wnt
WP_159741926.1
Streptomyces tubercidicus
Min-wnt BamE-like-Imm-Min-wnt
MBQ3121993.1
Bacteroidaceae bacterium
BamE-like-Imm-Min-wnt Min-wnt BamE-like-Imm-Min-wnt
WP_184031622.1
Deinococcus budaensis
Min-wnt
MBO5532907.1
Fibrobacter sp.
Min-wnt ext-tail TM-Imm-Wnt
WP_159053961.1
Streptomyces sp. AS58
Min-wnt TM-Imm-Wnt
WP_073227921.1
Min-wnt TM-Imm-Wnt
WP_073728289.1
Streptomyces sp. TSRI0281
Min-wnt TM-Imm-Wnt
MBE6895018.1
Oscillospiraceae bacterium
extended-loops Min-wnt TM-Imm-Wnt
MBQ6928982.1
Oscillospiraceae bacterium
extended-loops Min-wnt TM-Imm-Wnt
MBP1570386.1
Oscillospiraceae bacterium
extended-loops Min-wnt
AAZ06277.1
disordered Min-wnt
MBD2799925.1
Xenorhabdus szentirmaii
disordered Min-wnt
WP_100281975.1
Sphingomonas psychrotolerans
Min-wnt BamE-like-Imm-Min-wnt
WP_204195941.1
Min-wnt BamE-like-Imm-Min-wnt
PZO65378.1
Pseudoxanthomonas suwonensis
Min-wnt BamE-like-Imm-Min-wnt
WP_133849998.1
Labedaea rhizosphaerae
Min-wnt
CAG7844074.1
Pseudoclavibacter triregionum
Min-wnt
WP_190796119.1
Leptolyngbya sp. FACHB-541
Min-wnt
MBL8951893.1
Myxococcaceae bacterium
Min-wnt
WP_169173305.1
Actinoplanes sp. TBRC 11911
Min-wnt
QTW17774.1
Comamonas kerstersii
Min-wnt
WP_080602577.1
Tropheryma whipplei str. Dig9
extension Min-wnt
WP_080602994.1
Tropheryma whipplei str. DigMusc17
extension Min-wnt
AOM39267.1
Xenorhabdus hominickii
Min-wnt BamE-like-Imm-Min-wnt
CDC19302.1
Clostridium sp. CAG:306
Min-wnt helix linker hairpin
HIU86123.1
Candidatus Spyradomonas excrementavium
Min-wnt helix linker hairpin
HIU87107.1
Candidatus Spyradomonas excrementavium
Min-wnt helix linker hairpin
HIU92093.1
Candidatus Limenecus avicola
Min-wnt helix linker hairpin
MPZ32569.1
Rhodospirillales bacterium
Min-wnt extended-loop
WP_167191059.1
Wenyingzhuangia heitensis
Min-wnt disordered
MBR1424588.1
bacterium
Min-wnt disordered
DAB14264.1
Candidatus Gastranaerophilales bacterium HUM_17
Min-wnt 2helix
MBQ9723866.1
Neisseriaceae bacterium
Min-wnt ext-tail
MBC7460428.1
Thermoleophilia bacterium
Min-wnt ext-tail
WP_034296580.1
Alysiella crassa
Min-wnt ext-tail
WP_009116710.1
Neisseria wadsworthii
Min-wnt ext-tail
MBO6097813.1
Bacteroidales bacterium
Min-wnt ext-tail
WP_202312171.1
Mesorhizobium sp. L-8-10
Min-wnt ext-tail
WP_104930408.1
Simonsiella muelleri
Min-wnt ext-tail
WP_151051532.1
Neisseria zalophi
Min-wnt ext-tail
WP_152566004.1
Lysobacter antibioticus
Min-wnt ext-tail BamE-like-Imm-Min-wnt
WP_124794042.1
Conchiformibius steedae
Min-wnt ext-tail
WP_182122760.1
Neisseria shayeganii
Min-wnt ext-tail
WP_062669310.1
Chryseobacterium cucumeris
disordered Min-wnt
WP_065394775.1
Chryseobacterium artocarpi
disordered Min-wnt
WP_201766171.1
Verminephrobacter aporrectodeae subsp. tuberculatae At4
Min-wnt disordered
UHJ57052.1
Mycolicibacterium fortuitum
Min-wnt disordered
WP_111277293.1
Phenylobacterium kunshanense
Min-wnt
WP_223560123.1
Chryseobacterium lathyri
Min-wnt
MCB0879111.1
Thermoleophilia bacterium
Min-wnt
MBK8203543.1
Bdellovibrionales bacterium
Min-wnt BamE-like-Imm-Min-wnt
WP_159469023.1
Dyadobacter sp. 3J3
Min-wnt
WP_136017808.1
Coprococcus phoceensis
Min-wnt
MBO3117925.1
Winogradskyella pelagia
Min-wnt BamE-like-Imm-Min-wnt
MBK9736032.1
Saprospiraceae bacterium
Min-wnt
OHF24523.1
Rhodococcus erythropolis
Min-wnt
MBC7791896.1
Gemmatimonadaceae bacterium
Min-wnt
WP_145998305.1
Caulobacter flavus
Min-wnt
WP_163396388.1
Flavobacterium limi
Min-wnt
OFZ19173.1
Bdellovibrionales bacterium RBG_16_40_8
Min-wnt BamE-like-Imm-Min-wnt
MBO5531736.1
Fibrobacter sp.
Min-wnt
NLG35329.1
Lentisphaerota bacterium
Min-wnt
MBN1203828.1
Myxococcaceae bacterium
Min-wnt
MBA3406207.1
Gemmatimonadaceae bacterium
Min-wnt
WP_169089324.1
Paenibacillus sp. PL91
Min-wnt
WP_199898176.1
Hoylesella saccharolytica JCM 17484
Min-wnt BamE-like-Imm-Min-wnt
GDX41953.1
Bacteroidota bacterium
Min-wnt BamE-like-Imm-Min-wnt
WP_209268824.1
Streptomyces spirodelae
Min-wnt
MBD1867985.1
Cyanobacteria bacterium FACHB-471
Min-wnt BamE-like-Imm-Min-wnt
MBS5860518.1
bacterium
Min-wnt
STC75822.1
Corynebacterium minutissimum
Min-wnt BamE-like-Imm-Min-wnt
MCB0803959.1
Flavobacteriales bacterium
Min-wnt
MBK6840502.1
Bacteroidota bacterium
Min-wnt
WP_229714449.1
Lutibacter litoralis
Min-wnt
WP_226912998.1
Flavicella marina
Min-wnt
WP_131550972.1
Roseococcus sp. SYP-B2431
Min-wnt
WP_009947540.1
Saccharopolyspora erythraea
Min-wnt BamE-like-Imm-Min-wnt
CAG7845080.1
Pseudoclavibacter triregionum
Min-wnt
WP_148223860.1
Spirochaeta thermophila DSM 6192
Min-wnt
NVK20145.1
Methylocystaceae bacterium
Min-wnt
MCB9360934.1
Flavobacteriales bacterium
Min-wnt

HHC80911.1	*Flavobacteriia bacterium*	Min-wnt
MBG78011.1	*Alphaproteobacteria bacterium*	Min-wnt
WP_067956904.1	*Mycobacterium sp. NAZ190054*	Min-wnt
WP_208225220.1	*Salegentibacter sp. BDJ18*	Min-wnt, Jellyroll-like-Imm-Min-wnt-like
WP_100256317.1	*Reinekea forsetii*	BamE-like-Imm-Min-wnt, Min-wnt
MBX3163581.1	*Bacteroidota bacterium*	Min-wnt
WP_102144537.1	*Mycobacterium hubeiense*	Min-wnt, BamE-like-Imm-Min-wnt
MCE2733763.1	*Flammeovirgaceae bacterium*	Min-wnt
MAC86326.1	*Flavobacteriales bacterium*	Min-wnt
MBQ2366705.1	*Bacteroidaceae bacterium*	Min-wnt, BamE-like-Imm-Min-wnt
MBR1680862.1	*bacterium*	Min-wnt
MBR1619678.1	*bacterium*	Min-wnt
WP_195130086.1	*Nocardia amamiensis*	Min-wnt
MBO5964236.1	*Bacteroidales bacterium*	Min-wnt
MBL7883736.1	*Bacteroidia bacterium*	Min-wnt
NOR48851.1	*Methanosarcinaceae archaeon*	Min-wnt
MBQ4158339.1	*Clostridia bacterium*	Min-wnt, BamE-like-Imm-Min-wnt
WP_148560935.1	*Magnetospira sp. QH-2*	Min-wnt
WP_216424066.1	*Acetivibrio sp. MSJd-27*	Min-wnt, BamE-like-Imm-Min-wnt
NQY09795.1	*Flavobacteriales bacterium*	Min-wnt
WP_020591229.1	*Kiloniella laminariae DSM 19542*	Min-wnt
WP_233734980.1	*Kibdelosporangium philippinense*	Min-wnt
WP_169737023.1	*Deinococcus pimensis DSM 21231*	Min-wnt
WP_211848050.1	*Neoroseomonas eburnea*	Min-wnt
WP_211869209.1	*Neoroseomonas terrae*	Min-wnt
MBQ7878149.1	*Bacteroidaceae bacterium*	Min-wnt, BamE-like-Imm-Min-wnt
WP_176762236.1	*Eubacterium oxidoreducens*	Min-wnt
WP_211850608.1	*Plastoroseomonas hellenica*	Min-wnt
HIS74460.1	*Candidatus Galligastranaerophilus intestinavium*	Min-wnt
QQS11522.1	*Rhodospirillales bacterium*	Min-wnt
WP_144699755.1	*Cohnella terricola*	Min-wnt
WP_155153867.1	*Curvivirga aplysinae*	Min-wnt
WP_086781101.1		Min-wnt, BamE-like-Imm-Min-wnt, ?, Min-wnt, BamE-like-Imm-Min-wnt
OKL50983.1	*Bowdeniella nasicola*	Min-wnt
WP_159421664.1	*Pseudoclavibacter albus*	Min-wnt, ?, BamE-like-Imm-Min-wnt
WP_011712260.1	*Magnetococcus marinus MC-1*	Min-wnt
WP_127464603.1	*Paenibacillus sp. FSL W8-1187*	Min-wnt
MBI1308726.1	*Pseudomonadota bacterium*	Min-wnt
PHM54994.1	*Xenorhabdus hominickii*	Min-wnt
MCE3277972.1	*Bacteroidota bacterium*	Min-wnt
WP_162599722.1	*Nocardioides solisilvae*	Min-wnt
WP_051986859.1	*Clostridium hominis*	Min-wnt
WP_152664003.1	*Sphingomonas sp. SRS2*	Min-wnt
WP_230443854.1	*Nocardioides okcheonensis*	Min-wnt
WP_233427420.1	*Actinokineospora spheciospongiae*	Min-wnt, Min-wnt
WP_166320733.1	*Nocardioides piscis*	Min-wnt
WP_143343094.1	*Crossiella equi*	Min-wnt, BamE-like-Imm-Min-wnt
WP_113988059.1	*Flavobacterium tibetense*	Min-wnt, Jellyroll-like-Imm-Min-wnt-like
PVG81416.1	*Nocardioides gansuensis*	Min-wnt
WP_154516737.1	*Velocimicrobium porci*	Min-wnt, BamE-like-Imm-Min-wnt
MBR3916507.1	*Bacteroidaceae bacterium*	Min-wnt
MBA3783232.1	*Nocardioides sp.*	Min-wnt
MBK9402104.1	*Bacteroidota bacterium*	Min-wnt
WP_137173982.1	*Massilia sp. HP4*	Min-wnt, BamE-like-Imm-Min-wnt, BamE-like-Imm-Min-wnt
MBS6553427.1	*Clostridium sp.*	Min-wnt
BBZ09590.1	*Mycolicibacterium doricum*	Min-wnt
MBE8971447.1	*Nostocales cyanobacterium LEGE 12452*	Min-wnt, BamE-like-Imm-Min-wnt, BamE-like-Imm-Min-wnt
MCE2698449.1	*Anabaena sp. 49633_E8*	Min-wnt, BamE-like-Imm-Min-wnt
WP_235942527.1		Min-wnt
NVK20589.1	*Methylocystaceae bacterium*	Min-wnt
WP_069374539.1	*Herbaspirillum seropedicae*	Min-wnt
MBR1907681.1	*bacterium*	Min-wnt, ?, Min-wnt
WP_069380761.1	*Pedobacter steynii*	Min-wnt, BamE-like-Imm-Min-wnt
WP_222469327.1	*Vannielia litorea*	Min-wnt, BamE-like-Imm-Min-wnt
MBC8146645.1	*Bacteroidota bacterium*	Min-wnt
ABY46922.1	*Bacillus mycoides KBAB4*	Min-wnt
MBQ4420208.1	*Bacteroidales bacterium*	Min-wnt
HAS8474924.1	*Vibrio vulnificus*	Min-wnt, BamE-like-Imm-Min-wnt
WP_031426826.1	*Flavimarina sp. Hel_I_48*	Min-wnt
MBX7157857.1	*Verrucomicrobiia bacterium*	Min-wnt
MBL8253825.1	*Candidatus Competibacter sp.*	Min-wnt, BamE-like-Imm-Min-wnt
MBP1833499.1		Min-wnt
NLG34364.1	*Lentisphaerota bacterium*	Min-wnt, ?, BamE-like-Imm-Min-wnt
MCA0201724.1	*Pseudomonadota bacterium*	Min-wnt
WP_157694055.1	*Neisseria chenwenguii*	Min-wnt
WP_058855872.1	*Nocardia jinanensis*	Min-wnt
WP_133547962.1	*Salegentibacter sp. 24*	Min-wnt
WP_067037262.1	*Marinomonas gallaica*	BamE-like-Imm-Min-wnt, Min-wnt
WP_051678304.1	*Schinkia azotoformans*	Min-wnt
HAH33395.1	*Flavobacteriaceae bacterium*	Min-wnt
WP_116082168.1	*Tropicimonas sp. IMCC34011*	Min-wnt, BamE-like-Imm-Min-wnt
WP_131734374.1	*Gordonia paraffinivorans*	Min-wnt
MBS6532283.1	*Oscillospiraceae bacterium*	Min-wnt
MBT4679624.1	*Flavobacterium sp.*	Min-wnt
WP_222982858.1		Min-wnt
WP_143533228.1	*Salegentibacter salinarum*	Min-wnt
WP_143953308.1	*Robertkochia solimangrovi*	Min-wnt
RIV72891.1	*Flagellimonas aequoris*	Min-wnt
RYC52498.1	*Flagellimonas olearia*	Min-wnt
WP_136564918.1	*Flagellimonas alvinocaridis*	Min-wnt
NAS10862.1	*Poritiphilus flavus*	Min-wnt
WP_219039952.1	*Mesonia aestuariivivens*	Min-wnt
WP_178950411.1		Min-wnt

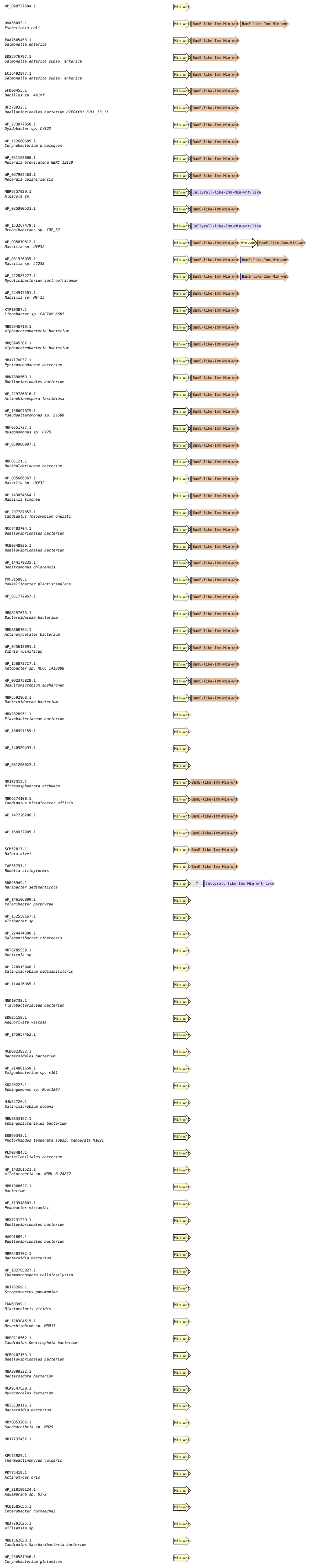
WP_099727684.1
Min-wnt
OVA56093.1
Escherichia coli
Min-wnt BamE-like-Imm-Min-wnt BamE-like-Imm-Min-wnt
EAA7605453.1
Salmonella enterica
Min-wnt BamE-like-Imm-Min-wnt
EDS5976797.1
Salmonella enterica subsp. enterica
Min-wnt BamE-like-Imm-Min-wnt
ECI6492877.1
Salmonella enterica subsp. enterica
Min-wnt BamE-like-Imm-Min-wnt
SFD00455.1
Bacillus sp. 491mf
Min-wnt BamE-like-Imm-Min-wnt
OFZ78932.1
Bdellovibrionales bacterium RIFOXYD1_FULL_53_11
Min-wnt BamE-like-Imm-Min-wnt
WP_233877850.1
Dyadobacter sp. CY323
Min-wnt BamE-like-Imm-Min-wnt
WP_152680605.1
Corynebacterium propinquum
Min-wnt BamE-like-Imm-Min-wnt
WP_051163606.1
Nocardia brevicatena NBRC 12119
Min-wnt BamE-like-Imm-Min-wnt
WP_067984463.1
Nocardia caishijiensis
Min-wnt BamE-like-Imm-Min-wnt
MBR9757929.1
Algicola sp.
Min-wnt Jellyroll-like-Imm-Min-wnt-like
WP_035000531.1
Min-wnt BamE-like-Imm-Min-wnt
WP_153267479.1
Oceanihabitans sp. IOP_32
Min-wnt Jellyroll-like-Imm-Min-wnt-like
WP_005670612.1
Massilia sp. UYP32
Min-wnt BamE-like-Imm-Min-wnt Min-wnt BamE-like-Imm-Min-wnt
WP_081926035.1
Massilia sp. LC238
Min-wnt BamE-like-Imm-Min-wnt BamE-like-Imm-Min-wnt
WP_222883277.1
Mycolicibacterium austroafricanum
Min-wnt BamE-like-Imm-Min-wnt BamE-like-Imm-Min-wnt
WP_224942501.1
Massilia sp. MS-15
Min-wnt BamE-like-Imm-Min-wnt
KYP10387.1
Limnobacter sp. CACIAM 66H1
Min-wnt BamE-like-Imm-Min-wnt
MBQ3946719.1
Alphaproteobacteria bacterium
Min-wnt BamE-like-Imm-Min-wnt
MBQ3945382.1
Alphaproteobacteria bacterium
Min-wnt BamE-like-Imm-Min-wnt
MBX7170037.1
Pyrinomonadaceae bacterium
Min-wnt BamE-like-Imm-Min-wnt
MBK7890560.1
Bdellovibrionales bacterium
Min-wnt BamE-like-Imm-Min-wnt
WP_229786816.1
Actinokineospora fastidiosa
Min-wnt BamE-like-Imm-Min-wnt
WP_138607875.1
Pseudoalteromonas sp. S1609
Min-wnt BamE-like-Imm-Min-wnt
MBF0651727.1
Dysgonomonas sp. GY75
Min-wnt BamE-like-Imm-Min-wnt
WP_050986907.1
Min-wnt BamE-like-Imm-Min-wnt
NUP85121.1
Burkholderiaceae bacterium
Min-wnt BamE-like-Imm-Min-wnt
WP_005666367.1
Massilia sp. UYP32
Min-wnt BamE-like-Imm-Min-wnt
WP_143054564.1
Massilia timonae
Min-wnt BamE-like-Imm-Min-wnt
WP_207787857.1
Candidatus Thiosymbion oneisti
Min-wnt BamE-like-Imm-Min-wnt
MCC7403704.1
Bdellovibrionales bacterium
Min-wnt BamE-like-Imm-Min-wnt
MCB0348836.1
Bdellovibrionales bacterium
Min-wnt BamE-like-Imm-Min-wnt
WP_144179235.1
Denitromonas ohlonensis
Min-wnt BamE-like-Imm-Min-wnt
PXF31500.1
Pokkaliibacter plantistimulans
Min-wnt BamE-like-Imm-Min-wnt
WP_051772967.1
Min-wnt BamE-like-Imm-Min-wnt
MBQ8257633.1
Bacteroidaceae bacterium
Min-wnt BamE-like-Imm-Min-wnt
MBK8868704.1
Actinomycetales bacterium
Min-wnt BamE-like-Imm-Min-wnt
WP_045613091.1
Vibrio vulnificus
Min-wnt BamE-like-Imm-Min-wnt
WP_156873717.1
Ketobacter sp. MCCC 1A13808
Min-wnt BamE-like-Imm-Min-wnt
WP_092375828.1
Desulfomicrobium apsheronum
Min-wnt BamE-like-Imm-Min-wnt
MBR5592968.1
Bacteroidaceae bacterium
Min-wnt BamE-like-Imm-Min-wnt
MBX2828951.1
Flavobacteriaceae bacterium
Min-wnt
WP_186991159.1
Min-wnt
WP_140989493.1
Min-wnt
WP_081208023.1
Min-wnt
NMJ87315.1
Nitrososphaerota archaeon
Min-wnt BamE-like-Imm-Min-wnt
MBK6574100.1
Candidatus Vicinibacter affinis
Min-wnt BamE-like-Imm-Min-wnt
WP_147226296.1
Min-wnt BamE-like-Imm-Min-wnt
WP_169932905.1
Min-wnt BamE-like-Imm-Min-wnt
SCM52817.1
Hafnia alvei
Min-wnt BamE-like-Imm-Min-wnt
TAE35797.1
Runella slithyformis
Min-wnt BamE-like-Imm-Min-wnt
SNR26949.1
Maribacter sedimenticola
Min-wnt ? Jellyroll-like-Imm-Min-wnt-like
WP_146106890.1
Polaribacter porphyrae
Min-wnt
WP_152558167.1
Altibacter sp.
Min-wnt
WP_224474300.1
Salegentibacter tibetensis
Min-wnt
MBT8285539.1
Muriicola sp.
Min-wnt
WP_228913946.1
Salinimicrobium sediminilitoris
Min-wnt
WP_114426065.1
Min-wnt
NNK10738.1
Flavobacteriaceae bacterium
Min-wnt
SDW25158.1
Aequorivita viscosa
Min-wnt
WP_145957462.1
Min-wnt
MCB0823832.1
Bacteroidales bacterium
Min-wnt
WP_214861650.1
Exiguobacterium sp. s161
Min-wnt
KQX26223.1
Sphingomonas sp. Root1294
Min-wnt
NJW54726.1
Salinimicrobium oceani
Min-wnt
MBN8834157.1
Sphingobacteriales bacterium
Min-wnt
EQB99348.1
Photorhabdus temperata subsp. temperata M1021
Min-wnt
PLX03484.1
Marinilabiliales bacterium
Min-wnt
WP_143261521.1
Allokutzneria sp. NRRL B-24872
Min-wnt
MBR1908627.1
bacterium
Min-wnt
WP_113948601.1
Pedobacter miscanthi
Min-wnt
MBX7231229.1
Bdellovibrionales bacterium
Min-wnt
HAG91689.1
Bdellovibrionales bacterium
Min-wnt
MBP6403702.1
Bacteroidia bacterium
Min-wnt
WP_182705027.1
Thermomonospora cellulosilytica
Min-wnt
ODJ76269.1
Streptococcus pneumoniae
Min-wnt
TKW60399.1
Blastochloris viridis
Min-wnt
WP_128304615.1
Mesorhizobium sp. M0011
Min-wnt
MBF0216562.1
Candidatus Omnitrophota bacterium
Min-wnt
MCB0407153.1
Bdellovibrionales bacterium
Min-wnt
MBA3899322.1
Bacteroidota bacterium
Min-wnt
MCA9547639.1
Myxococcales bacterium
Min-wnt
MBI5539116.1
Bacteroidia bacterium
Min-wnt
MBY8853206.1
Saccharothrix sp. MB29
Min-wnt
MBJ7737452.1
Min-wnt
KPC75929.1
Thermoactinomyces vulgaris
Min-wnt
PKY75419.1
Actinomyces oris
Min-wnt
WP_210199124.1
Aquimarina sp. U1-2
Min-wnt
MCE1685655.1
Enterobacter hormaechei
Min-wnt
MBJ7291625.1
Williamsia sp.
Min-wnt
MBB1562633.1
Candidatus Saccharibacteria bacterium
Min-wnt
WP_230581946.1
Corynebacterium glutamicum
Min-wnt

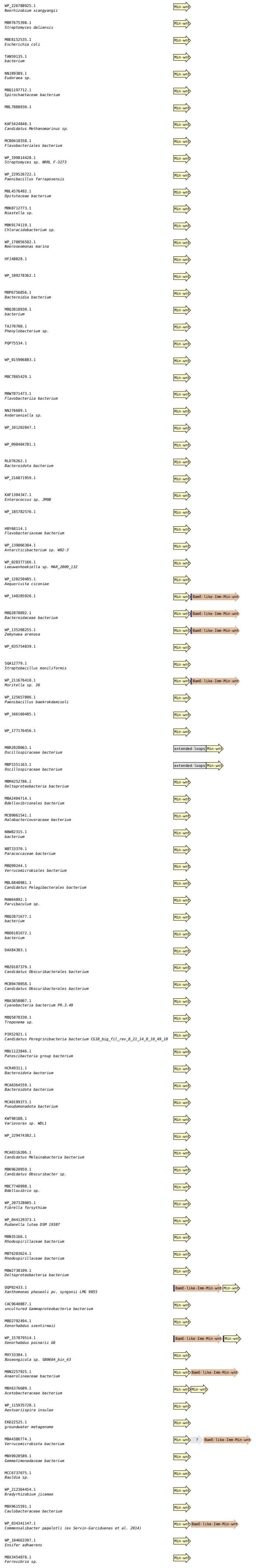

WP_226788925.1
Neorhizobium xiangyangii
Min-wnt
MBR7675398.1
Streptomyces daliensis
Min-wnt
MBE8152535.1
Escherichia coli
Min-wnt
TAN59135.1
bacterium
Min-wnt
NNJ89389.1
Eudoraea sp.
Min-wnt
MBQ1197712.1
Spirochaetaceae bacterium
Min-wnt
MBL7888930.1
Min-wnt
KAF5424840.1
Candidatus Methanomarinus sp.
Min-wnt
MCB0410358.1
Flavobacteriales bacterium
Min-wnt
WP_199814428.1
Streptomyces sp. NRRL F-3273
Min-wnt
WP_229526722.1
Paenibacillus farraposensis
Min-wnt
MBL4576492.1
Opitutaceae bacterium
Min-wnt
MBK8712773.1
Niastella sp.
Min-wnt
MBK9174119.1
Chloracidobacterium sp.
Min-wnt
WP_170056502.1
Neoroseomonas marina
Min-wnt
HFJ48828.1
Min-wnt
WP_189278362.1
Min-wnt
MBP6756856.1
Bacteroidia bacterium
Min-wnt
MBQ3818930.1
bacterium
Min-wnt
TAJ70700.1
Phenylobacterium sp.
Min-wnt
PQP75534.1
Min-wnt
WP_015906883.1
Min-wnt
MBC7865429.1
Min-wnt
MBW7871473.1
Flavobacteriia bacterium
Min-wnt
NNJ76689.1
Anderseniella sp.
Min-wnt
WP_101202847.1
Min-wnt
WP_098404781.1
Min-wnt
RLD76262.1
Bacteroidota bacterium
Min-wnt
WP_216871959.1
Min-wnt
KAF1304347.1
Enterococcus sp. JM9B
Min-wnt
WP_185782576.1
Min-wnt
HBY68114.1
Flavobacteriaceae bacterium
Min-wnt
WP_139066304.1
Antarcticibacterium sp. W02-3
Min-wnt
WP_028377166.1
Leeuwenhoekiella sp. MAR_2009_132
Min-wnt
WP_128250485.1
Aequorivita ciconiae
Min-wnt
WP_148285926.1
Min-wnt
BamE-like-Imm-Min-wnt
MBQ2878892.1
Bacteroidaceae bacterium
Min-wnt
BamE-like-Imm-Min-wnt
WP_135208255.1
Zemynaea arenosa
Min-wnt
BamE-like-Imm-Min-wnt
WP_035754839.1
Min-wnt
SQA12779.1
Streptobacillus moniliformis
Min-wnt
WP_211676410.1
Moritella sp. 36
Min-wnt
BamE-like-Imm-Min-wnt
WP_125657806.1
Paenibacillus baekrokdamisoli
Min-wnt
WP_166160485.1
Min-wnt
WP_177176456.1
Min-wnt
MBR2028063.1
Oscillospiraceae bacterium
extended-loops
Min-wnt
MBP1551163.1
Oscillospiraceae bacterium
extended-loops
Min-wnt
MBM4252786.1
Deltaproteobacteria bacterium
Min-wnt
MBA2404714.1
Bdellovibrionales bacterium
Min-wnt
MCB9061541.1
Halobacteriovoraceae bacterium
Min-wnt
NBW82315.1
bacterium
Min-wnt
NBT33370.1
Paracoccaceae bacterium
Min-wnt
MBQ99244.1
Verrucomicrobiales bacterium
Min-wnt
MBL6840981.1
Candidatus Pelagibacterales bacterium
Min-wnt
MAN64892.1
Parvibaculum sp.
Min-wnt
MBQ2871677.1
bacterium
Min-wnt
MBO6181672.1
bacterium
Min-wnt
DAX84383.1
Min-wnt
MBZ0187379.1
Candidatus Obscuribacterales bacterium
Min-wnt
MCB9470058.1
Candidatus Obscuribacterales bacterium
Min-wnt
MBA3858007.1
Cyanobacteria bacterium PR.3.49
Min-wnt
MBQ5878330.1
Treponema sp.
Min-wnt
PIR52921.1
Candidatus Peregrinibacteria bacterium CG10_big_fil_rev_8_21_14_0_10_49_10
Min-wnt
MBU1123846.1
Patescibacteria group bacterium
Min-wnt
HCR49311.1
Bacteroidota bacterium
Min-wnt
MCA0364559.1
Bacteroidota bacterium
Min-wnt
MCA0199373.1
Pseudomonadota bacterium
Min-wnt
KWT98188.1
Variovorax sp. WDL1
Min-wnt
WP_229474302.1
Min-wnt
MCA0316206.1
Candidatus Melainabacteria bacterium
Min-wnt
MBK9620959.1
Candidatus Obscuribacter sp.
Min-wnt
MBC7740998.1
Bdellovibrio sp.
Min-wnt
WP_207328005.1
Fibrella forsythiae
Min-wnt
WP_044129373.1
Rudanella lutea DSM 19387
Min-wnt
MBN35166.1
Rhodospirillaceae bacterium
Min-wnt
MBT6203624.1
Rhodospirillaceae bacterium
Min-wnt
MBW2738109.1
Deltaproteobacteria bacterium
Min-wnt
OQP82433.1
Xanthomonas phaseoli pv. syngonii LMG 9055
BamE-like-Imm-Min-wnt
Min-wnt
CAC9640887.1
uncultured Gammaproteobacteria bacterium
Min-wnt
MBD2792494.1
Xenorhabdus szentirmaii
Min-wnt
WP_157879514.1
Xenorhabdus poinarii G6
BamE-like-Imm-Min-wnt
Min-wnt
MXY33384.1
Boseongicola sp. SB0664_bin_43
Min-wnt
MBN2257925.1
Anaerolineaceae bacterium
Min-wnt
BamE-like-Imm-Min-wnt
MBX6376689.1
Acetobacteraceae bacterium
Min-wnt
Min-wnt
WP_115935728.1
Aestuariispira insulae
Min-wnt
EKD22525.1
groundwater metagenome
Min-wnt
MBA4386774.1
Verrucomicrobiota bacterium
Min-wnt
?
BamE-like-Imm-Min-wnt
MBX9928589.1
Gemmatimonadaceae bacterium
Min-wnt
MCC6737075.1
Bauldia sp.
Min-wnt
WP_212364454.1
Bradyrhizobium jicamae
Min-wnt
MBX9615591.1
Caulobacteraceae bacterium
Min-wnt
WP_034341147.1
Commensalibacter papalotli (ex Servin-Garciduenas et al. 2014)
Min-wnt
BamE-like-Imm-Min-wnt
WP_104663397.1
Ensifer adhaerens
Min-wnt
MBX3454878.1
Ferrovibrio sp.
Min-wnt

WP_172388816.1	*Sphingobacterium shayense*	Min-wnt
WP_210240483.1	*Mesorhizobium comanense*	Min-wnt
BCH22501.1	*Mesorhizobium sp. L-8-3*	Min-wnt
WP_136660824.1	*Nitratireductor sp. XY-223*	Min-wnt
OAI44670.1	*Rhizomicrobium sp. SCGC AG-212-E05*	Min-wnt
TYC64212.1	*Rhodobacterales bacterium*	Min-wnt
MBL4691664.1	*Rhodospirillales bacterium*	Min-wnt
WP_114069390.1	*Runella rosea*	Min-wnt, BamE-like-Imm-Min-wnt
WP_196900463.1		Min-wnt
NJM32144.1	*Limnobacter sp.*	Min-wnt, T6SS-tip-protein-VgrG
MCA3038947.1	*Rhodocyclaceae bacterium*	Min-wnt
WP_152122428.1	*Rudanella paleaurantiibacter*	Min-wnt
NAY91537.1	*Flagellimonas ochracea*	Min-wnt
WP_074950831.1	*Myxococcus fulvus*	Min-wnt
WP_157682360.1		Min-wnt
WP_013407698.1		Min-wnt
MBE2247691.1	*Candidatus Competibacteraceae bacterium*	Min-wnt
MYF10819.1	*Gammaproteobacteria bacterium*	Min-wnt, BamE-like-Imm-Min-wnt
MYK28248.1	*Gammaproteobacteria bacterium*	Min-wnt, BamE-like-Imm-Min-wnt
MBN8444975.1	*Gammaproteobacteria bacterium*	Min-wnt, Imm
MBC7440434.1	*Flavobacterium sp.*	Min-wnt
MCA1791955.1	*Thioalkalivibrio sp.*	Min-wnt
RRO12423.1	*Flavobacteriaceae bacterium 14752*	Min-wnt, BamE-like-Imm-Min-wnt, ?, Jellyroll-like-Imm-Min-wnt-like
SEH93468.1	*Epilithonimonas hominis*	Min-wnt
WP_025763208.1	*Dyadobacter tibetensis Y620-1*	Min-wnt
WP_234656210.1	*Dyadobacter chenwenxiniae*	Min-wnt
MBP9708436.1	*Oligoflexales bacterium*	Min-wnt
WP_131960687.1	*Dyadobacter psychrotolerans*	Min-wnt
PCJ99125.1	*Zetaproteobacteria bacterium*	Min-wnt
NQY73728.1	*Candidatus Margulisiibacteriota bacterium*	Min-wnt
OLA78696.1	*Candidatus Melainabacteria bacterium 35_41*	Min-wnt
GBR75211.1	*Candidatus Termititenax aidoneus*	Min-wnt
TPW11415.1	*Acidimicrobiaceae bacterium*	Min-wnt
MCB1040137.1	*Acidimicrobiales bacterium*	Min-wnt
WP_137339586.1	*Dyadobacter frigoris*	Min-wnt
WP_183298279.1	*Cellulomonas cellasea*	Min-wnt
ACP32342.1	*Corynebacterium aurimucosum ATCC 700975*	Min-wnt
WP_106530834.1	*Chitinophaga niastensis*	Min-wnt
WP_148565048.1		Min-wnt
WP_204293134.1	*Micromonospora gifhornensis*	Min-wnt
MCB0737404.1	*Bacteroidota bacterium*	Min-wnt
WP_065209307.1		Min-wnt
MCA0334408.1	*Bacteroidota bacterium*	Min-wnt
WP_051290269.1	*Bacteroides pyogenes DSM 20611 = JCM 6294*	Min-wnt
WP_110514182.1	*Herpetosiphon llansteffanensis*	Min-wnt, BamE-like-Imm-Min-wnt
MCD9188855.1	*Pyrinomonadaceae bacterium*	Min-wnt
OGF45205.1	*Candidatus Firestonebacteria bacterium RIFOXYD2_FULL_39_29*	Min-wnt
MCB0859975.1	*Solirubrobacterales bacterium*	Min-wnt
WP_153456833.1	*Streptomyces smaragdinus*	Min-wnt
WP_188296675.1		Min-wnt
WP_203927642.1	*Virgisporangium ochraceum*	Min-wnt
PIY43431.1	*Armatimonadetes bacterium CG_4_10_14_3_um_filter_66_18*	Min-wnt
MBQ6707247.1	*Clostridia bacterium*	Min-wnt
HIS88423.1	*Candidatus Avigastranaerophilus faecigallinarum*	Min-wnt
WP_108072139.1		Min-wnt
MBC9720664.1	*Lactobacillus sp.*	Min-wnt
WP_132282121.1	*Natranaerovirga hydrolytica*	Min-wnt, BamE-like-Imm-Min-wnt
NHI84001.1	*Candidatus Thorarchaeota archaeon*	Min-wnt
HGO42394.1		Min-wnt
MCC3659826.1	*Ruminococcus albus*	Min-wnt, BamE-like-Imm-Min-wnt
WP_183831673.1	*Aquamicrobium lusatiense*	Min-wnt
WP_093229434.1		Min-wnt
MBR6099406.1	*bacterium*	Min-wnt
TMI99395.1	*Alphaproteobacteria bacterium*	BamE-like-Imm-Min-wnt, Min-wnt
OGT88684.1	*Gammaproteobacteria bacterium RIFOXYD12_FULL_61_37*	BamE-like-Imm-Min-wnt, Min-wnt

###############;Skillet-1; ####;neighborhood with a core forming between Skillet-1 and a rapidly diverging IG-like domain fused to the SLP. Sometimes joined by a 4TM and/or HTH domain

OIP39249.1	*Deltaproteobacteria bacterium CG2_30_63_29*	Skillet-1, SLP, DUF4352-IG-like
WP_105192975.1		Skillet-1, SLP, DUF4352-IG-like
TNE54143.1	*Bacteroidota bacterium*	Skillet-1, ?, Skillet-1, SLP, DUF4352-IG-like
MBL0913309.1	*Bacteroidia bacterium*	Skillet-1, SLP, DUF4352-IG-like
WP_143884253.1	*Chryseobacterium binzhouense*	Skillet-1, SLP, DUF4352-IG-like
WP_054513071.1	*Chryseobacterium sp. ERMR1:04*	Skillet-1, SLP, DUF4352-IG-like
WP_123979592.1	*Chryseobacterium sp. G0201*	Skillet-1, SLP, DUF4352-IG-like
WP_113264972.1		Skillet-1, SLP, DUF4352-IG-like
RZJ70247.1	*Flavobacterium sp.*	Skillet-1, SLP, DUF4352-IG-like
WP_150545706.1	*Mesonia oceanica*	Skillet-1, SLP, DUF4352-IG-like
HAS42505.1	*Microscillaceae bacterium*	Skillet-1, SLP, DUF4352-IG-like
SMC75540.1	*Moheibacter sediminis*	Skillet-1, SLP, DUF4352-IG-like
WP_230848054.1	*Myroides odoratus*	Skillet-1, SLP, DUF4352-IG-like
WP_224455692.1	*Psychroflexus lacisalsi*	Skillet-1, SLP, DUF4352-IG-like
QBQ43149.1	*Sphingobacterium psychroaquaticum*	Skillet-1, SLP, DUF4352-IG-like
OJJ22503.1	*marine bacterium AO1-C*	Skillet-1, SLP, DUF4352-IG-like
WP_010933623.1	*Chlorobaculum tepidum TLS*	Skillet-1, SLP, DUF4352-IG-like
ACZ09986.1	*Sebaldella termitidis ATCC 33386*	Skillet-1, SLP, DUF4352-IG-like
MBL1153210.1	*Armatimonadota bacterium*	Skillet-1, SLP, DUF4352-IG-like
NLX91902.1	*Bacillota bacterium*	Skillet-1, SLP, DUF4352-IG-like, 4TM
MBQ4220408.1	*Butyrivibrio sp.*	Skillet-1, SLP, IG-like, IG-like, 4TM
MBQ3798017.1	*Butyrivibrio sp.*	HTH, 4TM, Skillet-1, SLP, IG-like, IG-like
WP_120742858.1	*Butyrivibrio sp. CB08*	HTH, Skillet-1, SLP, IG-like, IG-like, 4TM
WP_091825110.1	*Butyrivibrio sp. ob235*	HTH, Skillet-1, SLP, IG-like, IG-like, 4TM
MBQ1283717.1	*Clostridia bacterium*	Skillet-1, SLP, DUF5067-IG-like
NLG05346.1	*Clostridia bacterium*	Skillet-1, SLP, IG-like, IG-like, 4TM
MBQ3075845.1	*Clostridia bacterium*	Skillet-1, SLP, DUF5067-IG-like

MBQ4571706.1	*Clostridia bacterium*	HTH Skillet-1 DUF4352-IG-like SLP
MBQ3865585.1	*Clostridia bacterium*	HTH Skillet-1 DUF4352-IG-like SLP
MBQ3021985.1	*Clostridia bacterium*	HTH Skillet-1 DUF5067-IG-like SLP
MBQ9848677.1	*Clostridia bacterium*	HTH Skillet-1 DUF5067-IG-like SLP
MBQ9953103.1	*Clostridia bacterium*	HTH Skillet-1 DUF5067-IG-like SLP
MBQ6752999.1	*Clostridia bacterium*	Skillet-1 SLP DUF4352-IG-like
MBQ7603810.1	*Clostridia bacterium*	Skillet-1 SLP IG-like IG-like
MBQ8740572.1	*Clostridia bacterium*	HTH Skillet-1 DUF5067-IG-like SLP
MBQ8684488.1	*Clostridia bacterium*	HTH Skillet-1 DUF5067-IG-like SLP
MBR2240264.1	*Clostridia bacterium*	Skillet-1 SLP DUF5067-IG-like
MBQ8600127.1	*Clostridia bacterium*	HTH Skillet-1 DUF5067-IG-like SLP
MBQ8605840.1	*Clostridia bacterium*	HTH Skillet-1 DUF5067-IG-like SLP
MBR7132939.1	*Clostridia bacterium*	Skillet-1 SLP DUF5067-IG-like
MBR3381619.1	*Clostridia bacterium*	Skillet-1 SLP DUF4352-IG-like DUF4352-IG-like
MBR3145146.1	*Clostridia bacterium*	HTH Skillet-1 DUF4352-IG-like SLP
MBR2744782.1	*Clostridia bacterium*	Skillet-1 SLP DUF5067-IG-like
MBR6743333.1	*Clostridia bacterium*	Skillet-1 SLP DUF5067-IG-like
MBR6530089.1	*Clostridia bacterium*	Skillet-1 ? SLP DUF4352-IG-like
MBR5767058.1	*Clostridia bacterium*	Skillet-1 SLP IG-like IG-like
MBR4892765.1	*Clostridia bacterium*	HTH Skillet-1 DUF5067-IG-like SLP
MBP3330420.1		HTH DUF4352-IG-like SLP Skillet-1
MBP3626833.1	*Clostridia bacterium*	HTH Skillet-1 DUF5067-IG-like SLP
MBP3696375.1	*Clostridia bacterium*	Skillet-1 SLP DUF5067-IG-like
MBP3938166.1	*Clostridia bacterium*	Skillet-1 SLP DUF5067-IG-like
NBH71966.1	*Clostridiaceae bacterium*	HTH Skillet-1 SLP IG-like IG-like
MBR2524724.1	*Clostridiales bacterium*	Skillet-1 SLP IG-like IG-like
MBR6880881.1	*Clostridiales bacterium*	Skillet-1 SLP IG-like IG-like
MBR5938248.1	*Clostridiales bacterium*	Skillet-1 SLP IG-like IG-like
MBP5260682.1	*Clostridiales bacterium*	Skillet-1 SLP IG-like IG-like
MBP5655704.1	*Clostridiales bacterium*	HTH Skillet-1 SLP IG-like IG-like 4TM
MBS7354646.1	*Erysipelotrichaceae bacterium*	HTH Skillet-1 SLP IG-like IG-like
WP_117915740.1	*Lachnospira eligens*	HTH ? Skillet-1 SLP DUF4352-IG-like 4TM
MBQ9227552.1	*Eubacterium sp.*	Skillet-1 SLP IG-like IG-like
MBR1874457.1	*Eubacterium sp.*	HTH Skillet-1 DUF4352-IG-like SLP
WP_118370601.1	*Lachnospira eligens*	HTH ? ? Skillet-1 DUF4352-IG-like SLP 4TM
MBQ4521751.1	*Lachnospiraceae bacterium*	HTH Skillet-1 DUF5067-IG-like SLP
MBQ3544455.1	*Lachnospiraceae bacterium*	HTH Skillet-1 SLP IG-like IG-like
MBQ6637184.1	*Lachnospiraceae bacterium*	Skillet-1 SLP IG-like IG-like
MBQ8260974.1	*Lachnospiraceae bacterium*	HTH PSE ? Skillet-1 IG-like IG-like SLP
MBR1649539.1	*Lachnospiraceae bacterium*	Skillet-1 SLP DUF4352-IG-like IG-like IG-like
MBR1910500.1	*Lachnospiraceae bacterium*	HTH Skillet-1 IG-like IG-like SLP 4TM
MBR5799981.1	*Lachnospiraceae bacterium*	HTH Skillet-1 SLP IG-like IG-like
MBO5342345.1	*Lachnospiraceae bacterium*	Skillet-1 SLP DUF4352-IG-like DUF4352-IG-like
MBO5450926.1	*Lachnospiraceae bacterium*	HTH Skillet-1 SLP IG-like IG-like 4TM
MBP5353625.1	*Lachnospiraceae bacterium*	Skillet-1 SLP IG-like IG-like
MBP5624821.1	*Lachnospiraceae bacterium*	HTH Skillet-1 DUF4352-IG-like DUF4352-IG-like SLP 4TM
MBQ0059318.1	*Candidatus Equihabitans merdae*	Skillet-1 SLP DUF4352-IG-like DUF4352-IG-like
WP_012447277.1	*Natranaerobius thermophilus JW/NM-WN-LF*	Skillet-1 SLP DUF4352-IG-like
WP_089024113.1	*Natranaerobius trueperi*	Skillet-1 SLP DUF4352-IG-like
HCH28583.1	*Oscillospiraceae bacterium*	Skillet-1 SLP DUF4352-IG-like
MBE6540671.1	*Oscillospiraceae bacterium*	Skillet-1 SLP DUF4352-IG-like
MBE6618470.1	*Oscillospiraceae bacterium*	Skillet-1 SLP DUF5067-IG-like
MBE6781227.1	*Oscillospiraceae bacterium*	Skillet-1 SLP DUF5067-IG-like
MBE6796932.1	*Oscillospiraceae bacterium*	HTH Skillet-1 DUF5067-IG-like SLP
MBE6803255.1	*Oscillospiraceae bacterium*	Skillet-1 HTH DUF5067-IG-like SLP
MBE6809743.1	*Oscillospiraceae bacterium*	Skillet-1 SLP DUF5067-IG-like
MBE6835947.1	*Oscillospiraceae bacterium*	HTH Skillet-1 DUF5067-IG-like SLP
MBE7069319.1	*Oscillospiraceae bacterium*	HTH Skillet-1 SLP IG-like IG-like
MBQ5749538.1	*Oscillospiraceae bacterium*	Skillet-1 SLP DUF5067-IG-like
MBQ4382328.1	*Oscillospiraceae bacterium*	4TM Skillet-1 SLP IG-like IG-like
MBQ5989062.1	*Oscillospiraceae bacterium*	HTH Skillet-1 DUF4352-IG-like DUF4352-IG-like SLP
MBQ8928260.1	*Oscillospiraceae bacterium*	HTH Skillet-1 DUF4352-IG-like DUF4352-IG-like SLP
MBR2520790.1	*Oscillospiraceae bacterium*	HTH Skillet-1 SLP IG-like IG-like
MBR2807342.1	*Oscillospiraceae bacterium*	HTH 4TM Skillet-1 SLP IG-like IG-like
MBE5916564.1	*Pseudobutyrivibrio ruminis*	HTH Skillet-1 SLP IG-like IG-like 4TM
MBQ8280038.1	*Roseburia sp.*	HTH ? HTH Skillet-1 SLP IG-like IG-like
WP_186867523.1	*Roseburia zhanii*	HTH Skillet-1 SLP DUF4352-IG-like DUF4352-IG-like 4TM
SCW48603.1	*Ruminococcaceae bacterium YRB3002*	Skillet-1 SLP IG-like IG-like ? HTH
NLX01553.1	*Syntrophomonadaceae bacterium*	Skillet-1 SLP DUF4352-IG-like
WP_022770095.1	*Butyrivibrio sp. FCS006*	HTH Skillet-1 SLP IG-like IG-like 4TM
CDA97622.1	*Firmicutes bacterium CAG:65*	Skillet-1 SLP IG-like IG-like
QSZ27544.1	*Aceticella autotrophica*	4TM Skillet-1 SLP DUF4352-IG-like
HIR10589.1	*Candidatus Fimenecus excrementavium*	HTH ? Skillet-1 SLP DUF4352-IG-like
MBQ6837353.1	*Clostridia bacterium*	Skillet-1 SLP DUF4352-IG-like
WP_069157174.1	*Eisenbergiella tayi*	HTH ? Skillet-1 SLP IG-like IG-like
MBO4459030.1	*Clostridiales bacterium*	Skillet-1 SLP IG-like IG-like HTH
MBQ4424970.1	*Lachnospiraceae bacterium*	HTH Skillet-1 SLP DUF4352-IG-like DUF4352-IG-like
MBE6978386.1	*Oscillospiraceae bacterium*	HTH Skillet-1 DUF5067-IG-like DUF5067-IG-like SLP
MBR4361874.1	*Ruminococcus sp.*	Skillet-1 SLP DUF4352-IG-like
MBR3470205.1	*Lachnospiraceae bacterium*	Skillet-1 SLP DUF4352-IG-like DUF4352-IG-like

#;direct fusion

MBP5154939.1	*Lachnospiraceae bacterium*	HTH Skillet-1 IG-DUF4352 IG-DUF4352

###;another association with IG-like domains.

PIE70203.1	*Deltaproteobacteria bacterium*	disordered FliL-like ? ? beta-barrel helical-region Flg_hook Skillet-1 IG-like-repeats
MBU1169332.1	*Pseudomonadota bacterium*	Skillet-1 IG-like-repeats IG-like-repeats
WP_080806019.1	*Desulfamplus magnetovallimortis*	disordered FliL-like helical-domain DUF6115 DUF2802 beta-barrel helical-region Flg_hook Skillet-1 IG-like-repeats IG-like-repeats
MBF0241538.1	*Desulfamplus sp.*	disordered FliL-like helical-domain ? DUF6115 DUF2802 beta-barrel helical-region Flg_hook Skillet-1 IG-like-repeats
MBF0377712.1	*Desulfamplus sp.*	DUF6115 DUF2802 beta-barrel helical-region Flg_hook Skillet-1 PSE ? IG-like-repeats IG-like-repeats
MBF0468113.1	*Desulfamplus sp.*	disordered FliL-like helical-domain DUF6115 DUF2802 beta-barrel helical-region Flg_hook Skillet-1 IG-like-repeats
MBF0573275.1	*Desulfamplus sp.*	DUF6115 DUF2802 beta-barrel helical-region Flg_hook Skillet-1 IG-like-repeats
MBF0260019.1	*Desulfamplus sp.*	Skillet-1 IG-like-repeats
MCB9481272.1	*Desulfobacteraceae bacterium*	Skillet-1 IG-like-repeats IG-like-repeats

#;additional associations of a like kind

MBF0203121.1	*Desulfamplus sp.*	DUF6115 DUF2802 beta-barrel helical-region Flg_hook Skillet-1

MBF0392804.1
Desulfamplus sp.
disordered | FliL-like | helical-domain | ? | DUF6115 | DUF2802 | ? | beta-barrel | helical-region | Flg_hook | Skillet-1

MBF0225146.1
Desulfobacterales bacterium
beta-barrel | helical-region | Flg_hook | Skillet-1

MBL0024514.1
Saprospiraceae bacterium
Skillet-1 | IG-like | ? | IG-repeats | IG-repeats

#;additional IG-like domain-containing associating proteins, with MORN repeats and a helical 'linker-type' domain

HBH48084.1
Bacteroidales bacterium
Skillet-1 | SLP | MORNs | helical | IG-like | IG-like

MBE0654050.1
Bacteroidales bacterium
Skillet-1 | SLP | MORNs | helical | IG-like | IG-like

MBE0655394.1
Bacteroidales bacterium
Skillet-1 | SLP | MORNs | helical | IG-like | IG-like

MBN1415274.1
Bacteroidales bacterium
Skillet-1 | SLP | MORNs | helical | IG-like | IG-like

MBN2275042.1
Bacteroidales bacterium
Skillet-1 | SLP | MORNs | helical | IG-like | IG-like

RPI42327.1
Bacteroidota bacterium
Skillet-1 | SLP | MORNs | helical | IG-like | IG-like

NQU86513.1
Mariniphaga sp.
IG-repeats | Skillet-1 | SLP | MORNs | helical | IG-like | IG-like

MBN1819981.1
Prolixibacteraceae bacterium
Skillet-1 | SLP | MORNs | helical | IG-like | IG-like

MBT3384541.1
Prolixibacteraceae bacterium
Skillet-1 | SLP | MORNs | helical | IG-like | IG-like

MBK8053104.1
Saprospiraceae bacterium
Skillet-1 | SLP | MORNs | helical | IG-like | IG-like

MBP6185865.1
Saprospiraceae bacterium
Skillet-1 | SLP | MORNs | helical | IG-like | IG-like

RJR26729.1
Desulfobacteraceae bacterium
Skillet-1 | SLP | MORNs | helical | IG-like | IG-like

RLE01323.1
Bacteroidota bacterium
Skillet-1 | SLP | MORNs | helical | IG-like | IG-like

#;with DUF1566

PID44952.1
Pseudomonadota bacterium
Skillet-1 | SLP | IG-like | DUF1566 | SLP | IG-like | DUF1566

WP_109825164.1
Leucothrix arctica
Skillet-1 | SLP | IG-like | DUF1566 | SLP | IG-like | DUF1566

WP_109839749.1
Leucothrix pacifica
Skillet-1 | SLP | IG-like | DUF1566 | SLP | IG-like | DUF1566

WP_202718889.1
Thiothrix subterranea
SLP | IG-like | DUF1566 | SLP | IG-like | DUF1566 | ? | ? | Skillet-1 | SLP | IG-like | DUF1566 | SLP | IG-like | DUF1566

QYZ66144.1
Gammaproteobacteria bacterium (ex Lamellibrachia satsuma)
Skillet-1 | DUF1566 | DUF1566 | ??

#;additional, with jelly-roll domain also present (note subtype 2 here)

MBS0444101.1
Pseudomonadota bacterium
Skillet-1 | SLP | IG-like | jelly-roll

MBK7278700.1
Betaproteobacteria bacterium
Skillet-1 | SLP | IG-like | jelly-roll

MBK7278995.1
Betaproteobacteria bacterium
Skillet-1 | SLP | IG-like | jelly-roll

MBI3348042.1
Burkholderiales bacterium
Skillet-1 | SLP | IG-like | jelly-roll

UCG96371.1
Burkholderiales bacterium
Skillet-1 | SLP | IG-like | jelly-roll

MBK6864522.1
Ideonella sp.
Skillet-1 | SLP | IG-like | jelly-roll

MBP5990336.1
Piscinibacter sp.
Skillet-1 | SLP | IG-like | jelly-roll

TXC66490.1
Piscinibacter aquaticus
Skillet-1 | SLP | IG-like | jelly-roll

MBK6469588.1
Betaproteobacteria bacterium
Skillet-1 | SLP | IG-like | jelly-roll

#;additional, with b-propeller repeats

MBK7428995.1
Saprospiraceae bacterium
Skillet-1 | ? | b-propeller | IG-repeats

PHI21191.1
Lewinellaceae bacterium SD302
b-propeller | IG-repeats | b-propeller | IG-repeats | b-propeller | IG-repeats | Skillet-1

MBK8956696.1
Saprospiraceae bacterium
Skillet-1 | IG-like | IG-like | b-propeller | IG-repeats

MBP6397302.1
Saprospiraceae bacterium
Skillet-1 | SLP | IG-like | b-propeller | IG-repeats

MBP6695956.1
Saprospiraceae bacterium
Skillet-1 | IG-like | b-propeller | IG-repeats

MBK7220334.1
Saprospiraceae bacterium
Skillet-1 | SLP | IG-like | b-propeller | IG-repeats

MBK8701724.1
Saprospiraceae bacterium
Skillet-1 | SLP | IG-like | b-propeller | IG-repeats

MBK6365416.1
Saprospiraceae bacterium
Skillet-1 | ? | b-propeller | IG-repeats

#;only the b-propellers (these seem to have more conventional LPs)

MBR0202483.1
Bacteroidaceae bacterium
Skillet-1 | ? | ? | b-props

NBU05516.1
Candidatus Fonsibacter lacus
Skillet-1 | b-props

MBS4042873.1
Chitinophagaceae bacterium
Skillet-1 | b-props

MBD8938111.1
Leyella stercorea
Skillet-1 | b-props

MBO5242706.1
Muribaculaceae bacterium
Skillet-1 | ? | ? | b-props

MBN8880137.1
Sphingobacteriales bacterium
Skillet-1 | b-props

OJW33455.1
Sphingobacteriales bacterium 46-32
Skillet-1 | b-props

TNE80054.1
Bacteroidota bacterium
Skillet-1 | b-props

MBL6516907.1
Leyella stercorea
Skillet-1 | b-props

#;direct fusions

PID64066.1
Gammaproteobacteria bacterium
Skillet-1 | b-propeller

WP_016401261.1
Agarivorans albus
Skillet-1 | b-propeller

WP_216056082.1
Psychrosphaera sp. F3M07
Skillet-1 | b-propeller

NVK38072.1
Gammaproteobacteria bacterium
Skillet-1 | b-propeller

WP_035016664.1
Catenovulum agarivorans DS-2
Skillet-1 | b-propeller

NRA68837.1
Pseudobacteriovorax sp.
Skillet-1 | b-propeller

WP_051218825.1
Oceanobacter kriegii DSM 6294
Skillet-1 | b-propeller

KIM09320.1
Sulfurovum sp. PC08-66
Skillet-1 | b-prop

MBM4252417.1
Deltaproteobacteria bacterium
Skillet-1 | b-prop

HBS26513.1
Gammaproteobacteria bacterium
Skillet-1 | b-prop

HBR97743.1
Gammaproteobacteria bacterium
Skillet-1 | b-prop

ASJ76727.1
Granulosicoccus antarcticus IMCC3135
Skillet-1 | b-prop

NBX18695.1
Pseudomonadota bacterium
Skillet-1 | b-prop

NBW80833.1
bacterium
Skillet-1 | b-prop

MBN2803770.1
Deltaproteobacteria bacterium
Skillet-1 | b-prop

#;unknown domain with an IG-like domain-containing protein (note subtype 2 here)

MBI5716760.1
Burkholderiales bacterium
Skillet-1 | SLP | unk9 | unk9 | IG-like

MBL8326978.1
Rubrivivax sp.
Skillet-1 | SLP | unk9 | unk9 | IG-like

MBL8287061.1
Rubrivivax sp.
Skillet-1 | SLP | unk9 | unk9 | IG-like

MCC6249335.1
Rubrivivax sp.
Skillet-1 | SLP | unk9 | unk9 | IG-like

#;SLP+concavalin-like domain also present

WP_228127106.1
Kangiella aquimarina
Skillet-1 | beta-roll-repeats | IG-like | SLP | concavalin-like

WP_046561283.1
Kangiella geojedonensis
Skillet-1 | beta-roll-repeats | IG-like | SLP | concavalin-like

WP_106646533.1
Kangiella profundi
Skillet-1 | beta-roll-repeats | IG-like | SLP | concavalin-like

WP_110201838.1
Kangiella spongicola
Skillet-1 | beta-roll-repeats | IG-like | SLP | concavalin-like

WP_223577682.1
Skillet-1 | beta-roll-repeats | IG-like | SLP | concavalin-like

CAA0105169.1
BD1-7 clade bacterium
Skillet-1 | SLP | concavalin-like | beta-roll-repeats | IG-like

WP_123422433.1
Gallaecimonas pentaromativorans
Skillet-1 | SLP | concavalin-like | beta-roll-repeats | IG-like

WP_139445382.1
Arenimonas terrae
Skillet-1 | beta-roll-repeats | IG-like | SLP | concavalin-like

WP_133588697.1
Permianibacter aggregans
Skillet-1 | beta-roll-repeats | IG-like | SLP | concavalin-like

WP_154659424.1
Arenimonas composti TR7-09 = DSM 18010
Skillet-1 | beta-roll-repeats | IG-like | SLP | concavalin-like

WP_144391566.1
Pleionea sediminis
Skillet-1 | SLP | concavalin-like | beta-roll-repeats | IG-like

NVK88821.1
Gammaproteobacteria bacterium
Skillet-1 | SLP | concavalin-like

#;further associations with distinctive IG-like domains

MBU0653570.1
Gammaproteobacteria bacterium
Skillet-1 | SLP | IG-like

WP_070048945.1
Rheinheimera salexigens
Skillet-1 | SLP | IG-like

MBX3724538.1
Lysobacterales bacterium
Skillet-1 | SLP | IG-like

MBK7406275.1
Phycisphaerales bacterium
Skillet-1 | SLP | IG-like

UCG71581.1
Chromatiales bacterium
Skillet-1 | IG-like | IG-like

#another, distinct group of IG-like domains

NNF34632.1
Saprospiraceae bacterium
IG-repeats | Skillet-1

RDH87463.1
endosymbiont of Escarpia spicata
Skillet-1 | IG-repeats

Accession	Organism	Domains
MBN2554065.1	*Spirochaetales bacterium*	Skillet-1 · IG-repeats
MBP5586615.1	*Lentisphaeria bacterium*	IG-like · Skillet-1
WP_152001225.1	*Imperialibacter sp. 75*	IG-repeats · IG-repeats · Skillet-1
HIP37134.1	*Crocinitomix sp.*	Skillet-1 · IG-repeats

#;beta-rich domain. this one is a bit as the beta-rich region would necessarily be predicted to reside in the cytoplasm

Accession	Organism	Domains
MBQ5729448.1	*Bacteroidaceae bacterium*	Skillet-1 · SLP · beta-rich · SHOCT
MBQ8362083.1	*Bacteroidaceae bacterium*	Skillet-1 · SLP · beta-rich · SHOCT
MBP1615425.1	*Bacteroidota bacterium*	Skillet-1 · SLP · beta-rich · SHOCT
MBS7376718.1	*Paludibacteraceae bacterium*	Skillet-1 · SLP · beta-rich · SHOCT
OFO56675.1	*Porphyromonas sp. HMSC077F02*	Skillet-1 · SLP · beta-rich · SHOCT
WP_044111311.1	*Xylanibacter brevis P6B11*	Skillet-1 · SLP · beta-rich · SHOCT
WP_118151226.1	*Segatella copri*	Skillet-1 · SLP · beta-rich · SHOCT
EGC20899.1	*Prevotella multiformis DSM 16608*	Skillet-1 · SLP · beta-rich · SHOCT
MBO7540002.1	*Prevotella sp.*	Skillet-1 · SLP · beta-rich · SHOCT
MBQ0087754.1	*Candidatus Faecinaster equi*	Skillet-1 · SLP · beta-rich · SHOCT

###;fusions to unknown domains

Accession	Organism	Domains
MBO6058876.1	*Bacteroidaceae bacterium*	Skillet-1 · SLP · unk8
MBP3774879.1	*Bacteroidaceae bacterium*	Skillet-1 · SLP · unk8
MBQ6754738.1	*Bacteroidales bacterium*	Skillet-1 · SLP · unk8
MBR5651264.1	*Bacteroidales bacterium*	Skillet-1 · SLP · unk8
MBQ4484858.1	*Prevotella sp.*	Skillet-1 · SLP · unk8
MBR6593209.1	*Prevotella sp.*	Skillet-1 · SLP · unk8
MBS1655546.1	*Bacteroidota bacterium*	Skillet-1 · SLP · unk4
HAI82500.1	*Chitinophagaceae bacterium*	Skillet-1 · SLP · unk4
RYY38631.1	*Chitinophagaceae bacterium*	Skillet-1 · SLP · unk4
MBL7710268.1	*Chitinophagaceae bacterium*	Skillet-1 · SLP · unk4
MBL7748600.1	*Chitinophagaceae bacterium*	Skillet-1 · SLP · unk4
MBN8666025.1	*Chitinophagales bacterium*	Skillet-1 · SLP · unk4
NOS94004.1	*Cyclobacteriaceae bacterium*	Skillet-1 · SLP · unk4
WP_133995553.1	*Dinghuibacter silviterrae*	Skillet-1 · SLP · unk4
WP_072833415.1	*Flavisolibacter ginsengisoli DSM 18119*	Skillet-1 · SLP · unk4
MBK6827785.1	*Chitinophagaceae bacterium*	Skillet-1 · SLP · unk4
WP_140160602.1	*Algoriphagus antarcticus*	Skillet-1 · SLP · unk7
CAG0964140.1	*Flavobacteriales bacterium*	Skillet-1 · SLP · unk7
WP_171221859.1	*Flavobacterium rivulicola*	Skillet-1 · SLP · unk7
WP_189583583.1	*Mongoliitalea lutea*	Skillet-1 · SLP · unk7
OAD42366.1	*Polaribacter atrinae*	Skillet-1 · SLP · unk7
WP_015024990.1	*Psychroflexus torquis ATCC 700755*	Skillet-1 · SLP · unk7
WP_083727757.1	*Chitinophaga sp. LS1*	Skillet-1 · SLP · unk7
MBU4185806.1	*Pseudomonadota bacterium*	Skillet-1 · SLP · unk7
WP_151438922.1	*Thiopseudomonas alkaliphila*	Skillet-1 · SLP · unk7
WP_100728926.1	*Leptospira levettii*	Skillet-1 · SLP · unk7
OPY65614.1	*Syntrophorhabdus sp. PtaU1.Bin002*	Skillet-1 · SLP · unk7
MBU0518799.1	*bacterium*	Skillet-1 · SLP · unk7
MBC23616.1	*Phycisphaerae bacterium*	Skillet-1 · SLP · unk7 · Rossmann-MTase
UCG73358.1	*Chromatiales bacterium*	Skillet-1 · SLP · unk9 · unk9
NCF24361.1	*Gammaproteobacteria bacterium*	Skillet-1 · SLP · unk9
MBT8067917.1	*Gammaproteobacteria bacterium*	Skillet-1 · SLP · unk9 · unk9
MBT8132620.1	*Gammaproteobacteria bacterium*	Skillet-1 · SLP · unk9
TFG85103.1	*Chromatiales bacterium*	Skillet-1 · SLP · unk9
MBL7724126.1	*Chitinophagaceae bacterium*	Skillet-1 · unk18
MBL7732513.1	*Chitinophagaceae bacterium*	Skillet-1 · unk18
OSZ79849.1	*Chitinophagaceae bacterium IBVUCB2*	Skillet-1 · unk18
MBN8689019.1	*Chitinophagales bacterium*	Skillet-1 · unk18

#;another IG-like association, with AAA+ also present (see below)

Accession	Organism	Domains
MBQ6378453.1	*Prevotella sp.*	Skillet-1 · linker · AAA · lid · SLP · IG-like · helical-region · IG-like · unk10-Nterm · unk10-Cterm · unk10-Cterm · ? · ZnR · AAA · lid · AAA · lid · AAA · lid
MBQ8657388.1	*Prevotella sp.*	SLP · IG-like · helical-region · IG-like · ? · Skillet-1 · linker · AAA · lid · unk10-Nterm · unk10-Cterm · unk10-Cterm · ZnR · AAA · lid · AAA · lid · AAA · lid
MBR5325796.1	*Prevotella sp.*	Skillet-1 · ? · SLP · IG-like · helical-region · IG-like · unk10-Nterm · unk10-Cterm · unk10-Cterm
MBP5777910.1	*Prevotella sp.*	Skillet-1 · ? · ? · SLP · IG-like · helical-region · IG-like · unk10-Nterm · unk10-Cterm · unk10-Cterm
MBQ8988406.1	*Prevotella sp.*	Skillet-1 · linker · AAA · lid · unk10-Nterm · unk10-Cterm · unk10-Cterm · ? · ZnR · AAA · lid · AAA · lid · AAA · lid

######;association with AAA+-lid domain

Accession	Organism	Domains
MBQ6749511.1	*Bacteroidaceae bacterium*	Skillet-1 · linker · AAA · lid
MBQ6964767.1	*Bacteroidaceae bacterium*	Skillet-1 · linker · AAA · lid
MBQ9641250.1	*Bacteroidaceae bacterium*	Skillet-1 · linker · AAA · lid
MBR2154237.1	*Bacteroidaceae bacterium*	Skillet-1 · linker · AAA · lid
MBR1521302.1	*Bacteroidaceae bacterium*	Skillet-1 · linker · AAA · lid
MBR4533255.1	*Bacteroidaceae bacterium*	Skillet-1 · linker · AAA · lid
MBO4397119.1	*Bacteroidaceae bacterium*	Skillet-1 · linker · AAA · lid
MBO4431165.1	*Bacteroidaceae bacterium*	Skillet-1 · linker · AAA · lid
MBQ3636040.1	*Bacteroidales bacterium*	Skillet-1 · linker · AAA · lid
MBR3027431.1	*Bacteroidales bacterium*	Skillet-1 · linker · AAA · lid
MBR5716835.1	*Bacteroidales bacterium*	Skillet-1 · linker · AAA · lid
MBO4738393.1	*Bacteroidales bacterium*	Skillet-1 · linker · AAA · lid
MBQ0061046.1	*Candidatus Minthousia equi*	Skillet-1 · linker · AAA · lid
MBR4483394.1	*Paludibacteraceae bacterium*	Skillet-1 · linker · AAA · lid
MBO7634951.1	*Paludibacteraceae bacterium*	Skillet-1 · linker · AAA · lid
MBO6287863.1	*Prevotella sp.*	Skillet-1 · linker · AAA · lid
MBQ6204637.1	*Prevotella sp.*	Skillet-1 · linker · AAA · lid
MBQ6229107.1	*Prevotella sp.*	Skillet-1 · linker · AAA · lid
MBR0117600.1	*Prevotella sp.*	Skillet-1 · linker · AAA · lid
MBQ9215762.1	*Prevotella sp.*	Skillet-1 · linker · AAA · lid
MBR3473041.1	*Prevotella sp.*	Skillet-1 · linker · AAA · lid
MBR7053605.1	*Prevotella sp.*	Skillet-1 · linker · AAA · lid
MBR7018945.1	*Prevotella sp.*	Skillet-1 · linker · AAA · lid
MBR5929623.1	*Prevotella sp.*	Skillet-1 · linker · AAA · lid
MBR5655344.1	*Prevotella sp.*	Skillet-1 · linker · AAA · lid
MBO4608209.1	*Prevotella sp.*	Skillet-1 · linker · AAA · lid
MBO4821781.1	*Prevotella sp.*	Skillet-1 · linker · AAA · lid
MBO4849709.1	*Prevotella sp.*	Skillet-1 · linker · AAA · lid
MBO7578553.1	*Prevotella sp.*	Skillet-1 · linker · AAA · lid
MBP3219821.1	*Prevotella sp.*	Skillet-1 · linker · AAA · lid
MBP3286451.1	*Prevotella sp.*	Skillet-1 · linker · AAA · lid
MBP5339259.1	*Prevotella sp.*	Skillet-1 · linker · AAA · lid
MBP5424914.1	*Prevotella sp.*	Skillet-1 · linker · AAA · lid

Accession	Organism	Domains
WP_211839316.1	*Prevotella sp. ne3005*	Skillet-1 · linker · AAA · lid
WP_143096836.1	*Prevotella sp. tf2-5*	Skillet-1 · linker · AAA · lid
MBQ0024043.1	*Candidatus Minthosoma caballi*	Skillet-1 · linker · AAA · lid
MBQ0046401.1	*Candidatus Minthosoma equi*	Skillet-1 · linker · AAA · lid
MBQ4230844.1	*Salinivirgaceae bacterium*	Skillet-1 · linker · AAA · lid
MBO7145864.1	*Salinivirgaceae bacterium*	Skillet-1 · linker · AAA · lid
MBO7497542.1	*Salinivirgaceae bacterium*	Skillet-1 · linker · AAA · lid

########;association with SLP-bound OB-like domain

Accession	Organism	Domains
MBF0106912.1	*Deltaproteobacteria bacterium*	TPRs · OB-like · SLP · OB-like · Skillet-1
MSP70485.1	*Bacteroidota bacterium*	Skillet-1 · SLP · OB-like
WP_230636229.1	*Chryseobacterium sp. LC2016-29*	Skillet-1 · SLP · OB-like
OGS61252.1	*Flavobacteria bacterium GWF1_32_7*	Skillet-1 · SLP · OB-like
MBR98422.1	*Flavobacteriaceae bacterium*	Skillet-1 · SLP · OB-like
NVJ64489.1	*Flavobacteriaceae bacterium*	Skillet-1 · SLP · OB-like
WP_089050044.1		Skillet-1 · SLP · OB-like · DUF4236 · Excalibur · TerB-like
WP_073408820.1	*Flavobacterium johnsoniae*	Skillet-1 · SLP · OB-like
SNR33382.1	*Flavobacterium sp. ov086*	Skillet-1 · SLP · OB-like · DUF3892-like
PHR20653.1	*Fluviicola sp.*	Skillet-1 · SLP · OB-like
ADF53592.1	*Zunongwangia profunda SM-A87*	Skillet-1 · SLP · OB-like
WP_154781898.1	*Flavobacterium sp. ANB*	Skillet-1 · SLP · OB-like · DUF3892-like · DUF4236 · Excalibur · TerB-like
WP_073485256.1	*Flavobacterium sp. CF108*	Skillet-1 · SLP · OB-like
MBN1634755.1	*Ignavibacteria bacterium*	Skillet-1 · SLP · OB-like
MCA9585465.1	*Myxococcales bacterium*	Skillet-1 · SLP · OB-like
PCJ51989.1	*Planctomycetota bacterium*	Skillet-1 · SLP · OB-like
KPF98188.1	*Rhodopseudomonas sp. AAP120*	Skillet-1 · SLP · OB-like
WP_038031126.1	*Thioalkalivibrio sp. ALE11*	SLP · OB-like · Skillet-1
WP_135590132.1	*Leptospira gomenensis*	Skillet-1 · SLP · OB-like
TGK11254.1	*Leptospira stimsonii*	Skillet-1 · SLP · OB-like
MBN4054930.1	*Acidimicrobium ferrooxidans*	Skillet-1 · SLP · OB-like · TMs · OB-like
HHZ19957.1	*Bacillota bacterium*	Skillet-1 · SLP · OB-like
MBI1223643.1	*Bacteroidota bacterium*	SLP · OB-like · ? · ? · Skillet-1 · unk20
MBK9016585.1	*Saprospiraceae bacterium*	SLP · OB-like · ? · ? · Skillet-1 · unk20
MBJ7391863.1	*Chthoniobacterales bacterium*	Skillet-1 · linker · ?? · SLP · OB-like
EAU74896.1	*Synechococcus sp. RS9916*	Skillet-1 · SLP · OB-like
MBM3399079.1	*Bacteroidota bacterium*	SLP · OB-like · Skillet-1
MBL6611361.1	*Flavobacteriaceae bacterium*	Skillet-1 · SLP · OB-like
WP_128266528.1	*Bradyrhizobium yuanmingense*	Skillet-1 · SLP · OB-like

#;only the Excalibur association

Accession	Organism	Domains
WP_107933538.1	*Ureibacillus chungkukjangi*	Skillet-1 · helix-spine · Excalibur
DAJ26802.1		Excalibur · Skillet-1
DAU42958.1		Excalibur · Skillet-1
OCS93085.1	*Caryophanon latum*	Skillet-1 · helix-spine · Excalibur

############;combined association with Rossmann MTase domain and sometimes one variable additional enzymatic domain

Accession	Organism	Domains
MCB1061172.1	*Calditrichota bacterium*	Rossmann-MTase · Skillet-1
NMN31942.1		DUF2236-rubber-oxygenase · Rossmann-MTase · Skillet-1
PZR09278.1	*Flavobacterium psychrophilum*	DUF2236-rubber-oxygenase · PSE-Rossmann-MTase · Skillet-1
WP_146261463.1	*Apibacter muscae*	Rossmann-MTase · Skillet-1 · Calcineurin
MBC7865445.1	*Bacteroidia bacterium*	Rossmann-MTase · Skillet-1
MBO9729752.1	*Chitinophaga sp.*	Rossmann-MTase · Skillet-1
RYY59127.1	*Chitinophagaceae bacterium*	Rossmann-MTase · Skillet-1 · Calcineurin
RYY56397.1	*Chitinophagaceae bacterium*	Rossmann-MTase · Skillet-1 · Calcineurin · Rossmann-MTase
RYY71716.1	*Chitinophagaceae bacterium*	Rossmann-MTase · Skillet-1
RYZ25341.1	*Chitinophagaceae bacterium*	Rossmann-MTase · Skillet-1
KUJ52752.1	*Chryseobacterium sp. JAH*	Rossmann-MTase · Skillet-1
WP_229984765.1	*Chryseobacterium sp. C-71*	Rossmann-MTase · Skillet-1
MBT1697357.1	*Chryseosolibacter histidini*	Rossmann-MTase · Skillet-1 · DAGK_prokar
KYP15338.1	*Flavihumibacter sp. CACIAM 22H1*	Calcineurin · Spo0M · Rossmann-MTase · Skillet-1
APD07678.1	*Flavobacteriaceae bacterium UJ101*	Rossmann-MTase · Skillet-1
NQX91511.1	*Flavobacteriales bacterium*	IG-repeats · IG-repeats · Rossmann-MTase · Skillet-1
MBL4709098.1	*Flavobacteriales bacterium*	Rossmann-MTase · Skillet-1
MCB9202068.1	*Flavobacteriales bacterium*	Rossmann-MTase · Skillet-1
MCB9425686.1	*Flavobacteriales bacterium*	Rossmann-MTase · Skillet-1
AWH86435.1	*Flavobacterium album*	HTH · DUF2236-rubber-oxygenase · Rossmann-MTase · Skillet-1
WP_205509831.1	*Longitalea arenae*	Rossmann-MTase · Skillet-1
EHQ25060.1	*Mucilaginibacter paludis DSM 18603*	Rossmann-MTase · Skillet-1 · DAGK_prokar
MBB5395327.1	*Mucilaginibacter sp. AK015*	Rossmann-MTase · Skillet-1 · DAGK_prokar
WP_081154102.1		Rossmann-MTase · Skillet-1
PQJ17899.1	*Nonlabens xylanidelens*	Rossmann-MTase · Skillet-1
WP_113635119.1	*Nubsella zeaxanthinifaciens*	Rossmann-MTase · Skillet-1 · DAGK_prokar
AXY78662.1	*Paraflavitalea soli*	MBL · Rossmann-MTase · Skillet-1
KIA91947.1	*Pedobacter kyungheensis*	Rossmann-MTase · Skillet-1
WP_136844193.1	*Pedobacter polaris*	Rossmann-MTase · Skillet-1
WP_223833509.1	*Pedobacter riviphilus*	DinB-like · Rossmann-MTase · Skillet-1
WP_112746010.1	*Pseudochryseolinea flava*	Rossmann-MTase · Skillet-1 · DAGK_prokar
RYZ31252.1	*Sphingobacteriales bacterium*	DAGK_prokar · Rossmann-MTase · Skillet-1
WP_162390127.1	*Spirosoma endbachense*	Rossmann-MTase · Skillet-1 · DAGK_prokar
MBD2703133.1	*Spirosoma profusum*	Rossmann-MTase · Skillet-1 · DAGK_prokar
WP_150031696.1	*Taibaiella lutea*	DAGK_prokar · Rossmann-MTase · Skillet-1
WP_116875188.1	*Terrimonas sp.*	Rossmann-MTase · Skillet-1
WP_233603947.1	*Pedobacter sp. HDW13*	DinB-like · Rossmann-MTase · Skillet-1
MBK8801800.1	*Fibrobacterota bacterium*	Rossmann-MTase · Skillet-1 · Calcineurin
MBK9578729.1	*Fibrobacterota bacterium*	Rossmann-MTase · Skillet-1 · Calcineurin
MBK7988162.1	*Ignavibacteria bacterium*	Rossmann-MTase · Skillet-1 · Calcineurin
WP_170156918.1	*Roseimicrobium gellanilyticum*	Rossmann-MTase · Skillet-1 · Calcineurin
WP_151078071.1	*Hymenobacter busanensis*	Rossmann-MTase · Skillet-1 · GT-A
GGF03330.1	*Hymenobacter cavernae*	Rossmann-MTase · Skillet-1 · GT-A
WP_198074647.1	*Hymenobacter negativus*	Rossmann-MTase · Skillet-1 · GT-A
TKJ17046.1	*Candidatus Woesearchaeota archaeon B3_Woes*	Rossmann-MTase · PKinase · Skillet-1
WP_216714546.1	*Taishania pollutisoli*	Skillet-1 · Calcineurin

#########;tolB-N association

Accession	Organism	Domains
RMG69653.1	*Bacteroidota bacterium*	Skillet-1 · SLP? · tolB-N
NCT93157.1	*Chitinophagaceae bacterium*	Skillet-1 · SLP? · tolB-N
NBT09074.1	*Chitinophagia bacterium*	Skillet-1 · SLP? · tolB-N
WP_220275194.1	*Emticicia sp. BO119*	Skillet-1 · SLP? · tolB-N

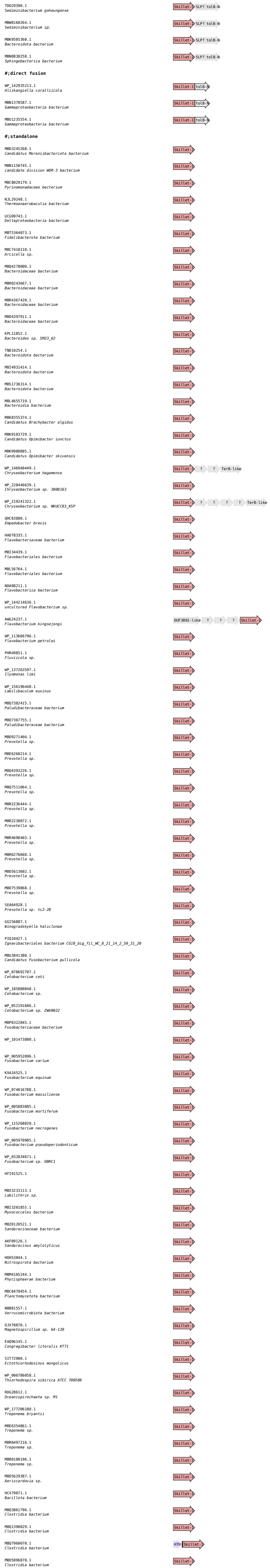
TDO29390.1
Sediminibacterium goheungense
Skillet-1 SLP? tolB-N
MBW0160264.1
Sediminibacterium sp.
Skillet-1 SLP? tolB-N
MBK9505360.1
Bacteroidota bacterium
Skillet-1 SLP? tolB-N
MBN8838258.1
Sphingobacteriia bacterium
Skillet-1 SLP? tolB-N
#;direct fusion
WP_142935213.1
Aliikangiella coralliicola
Skillet-1 tolB-N
MBN1378587.1
Gammaproteobacteria bacterium
Skillet-1 tolB-N
MBU1235554.1
Gammaproteobacteria bacterium
Skillet-1 tolB-N
#;standalone
MBD3245268.1
Candidatus Moraniibacteriota bacterium
Skillet-1
MBN1150745.1
candidate division WOR-3 bacterium
Skillet-1
MBC8029179.1
Pyrinomonadaceae bacterium
Skillet-1
NJL29248.1
Thermoanaerobaculia bacterium
Skillet-1
UCG99743.1
Deltaproteobacteria bacterium
Skillet-1
MBT5364073.1
Fidelibacterota bacterium
Skillet-1
MBC7410110.1
Arcicella sp.
Skillet-1
MBQ4278000.1
Bacteroidaceae bacterium
Skillet-1
MBR0243667.1
Bacteroidaceae bacterium
Skillet-1
MBR4367420.1
Bacteroidaceae bacterium
Skillet-1
MBO4397911.1
Bacteroidaceae bacterium
Skillet-1
KPL11852.1
Bacteroides sp. SM23_62
Skillet-1
TND10254.1
Bacteroidota bacterium
Skillet-1
MBI4931414.1
Bacteroidota bacterium
Skillet-1
MBS1736314.1
Bacteroidota bacterium
Skillet-1
MBL4655719.1
Bacteroidia bacterium
Skillet-1
MBK8355374.1
Candidatus Brachybacter algidus
Skillet-1
MBK9103729.1
Candidatus Opimibacter iunctus
Skillet-1
MBK9980885.1
Candidatus Opimibacter skivensis
Skillet-1
WP_146940449.1
Chryseobacterium hagamense
Skillet-1 ? ? TerB-like
WP_228446639.1
Chryseobacterium sp. 3008163
Skillet-1
WP_219241322.1
Chryseobacterium sp. NKUCC03_KSP
Skillet-1 ? ? ? ? TerB-like
QHC83880.1
Empedobacter brevis
Skillet-1
HAD78335.1
Flavobacteriaceae bacterium
Skillet-1
MBI34439.1
Flavobacteriales bacterium
Skillet-1
MBL56764.1
Flavobacteriales bacterium
Skillet-1
NDA98211.1
Flavobacteriia bacterium
Skillet-1
WP_144214636.1
uncultured Flavobacterium sp.
Skillet-1
AWG24237.1
Flavobacterium kingsejongi
DUF3892-like ? ? ? Skillet-1
WP_113666796.1
Flavobacterium petrolei
Skillet-1
PHR49851.1
Fluviicola sp.
Skillet-1
WP_137263597.1
Ilyomonas limi
Skillet-1
WP_156196448.1
Labilibaculum euxinus
Skillet-1
MBQ7382415.1
Paludibacteraceae bacterium
Skillet-1
MBO7367755.1
Paludibacteraceae bacterium
Skillet-1
MBD9271466.1
Prevotella sp.
Skillet-1
MBE6260214.1
Prevotella sp.
Skillet-1
MBQ4393226.1
Prevotella sp.
Skillet-1
MBQ7511064.1
Prevotella sp.
Skillet-1
MBR2236444.1
Prevotella sp.
Skillet-1
MBR2238072.1
Prevotella sp.
Skillet-1
MBR4698403.1
Prevotella sp.
Skillet-1
MBR6276668.1
Prevotella sp.
Skillet-1
MBO5613602.1
Prevotella sp.
Skillet-1
MBO7539868.1
Prevotella sp.
Skillet-1
SEA64928.1
Prevotella sp. tc2-28
Skillet-1
GGI56887.1
Winogradskyella haliclonae
Skillet-1
PIQ10427.1
Ignavibacteriales bacterium CG18_big_fil_WC_8_21_14_2_50_31_20
Skillet-1
MBU3841380.1
Candidatus Fusobacterium pullicola
Skillet-1
WP_078692707.1
Cetobacterium ceti
Skillet-1
WP_185880848.1
Cetobacterium sp.
Skillet-1
WP_052191686.1
Cetobacterium sp. ZWU0022
Skillet-1
MBP6322845.1
Fusobacteriaceae bacterium
Skillet-1
WP_101473880.1
Skillet-1
WP_005952096.1
Fusobacterium varium
Skillet-1
KXA16525.1
Fusobacterium equinum
Skillet-1
WP_074016788.1
Fusobacterium massiliense
Skillet-1
WP_005883885.1
Fusobacterium mortiferum
Skillet-1
WP_115268820.1
Fusobacterium necrogenes
Skillet-1
WP_005970985.1
Fusobacterium pseudoperiodonticum
Skillet-1
WP_032834671.1
Fusobacterium sp. OBRC1
Skillet-1
HFI91525.1
Skillet-1
MBX3233113.1
Labilithrix sp.
Skillet-1
MBI3201855.1
Myxococcales bacterium
Skillet-1
MBZ0120521.1
Sandaracinaceae bacterium
Skillet-1
AKF09126.1
Sandaracinus amylolyticus
Skillet-1
HDH53844.1
Nitrospirota bacterium
Skillet-1
MBM4105244.1
Phycisphaerae bacterium
Skillet-1
MBC8470454.1
Planctomycetota bacterium
Skillet-1
NBB81557.1
Verrucomicrobiota bacterium
Skillet-1
OJX70870.1
Magnetospirillum sp. 64-120
Skillet-1
EAQ96145.1
Congregibacter litoralis KT71
Skillet-1
SIT72900.1
Ectothiorhodosinus mongolicus
Skillet-1
WP_006786058.1
Thiorhodospira sibirica ATCC 700588
Skillet-1
RDG28612.1
Oceanispirochaeta sp. M1
Skillet-1
WP_177206180.1
Treponema bryantii
Skillet-1
MBE6354861.1
Treponema sp.
Skillet-1
MBR0497216.1
Treponema sp.
Skillet-1
MBR0100196.1
Treponema sp.
Skillet-1
MBO5629387.1
Aeriscardovia sp.
Skillet-1
HCX79871.1
Bacillota bacterium
Skillet-1
MBQ3861796.1
Clostridia bacterium
Skillet-1
MBQ1390029.1
Clostridia bacterium
Skillet-1
MBQ7960070.1
Clostridia bacterium
HTH Skillet-1
MBO5896870.1
Clostridia bacterium
Skillet-1

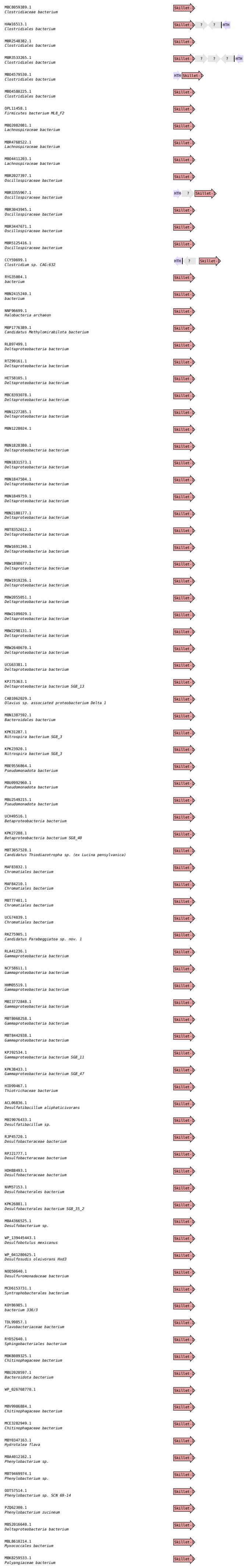
MBC8059389.1
Clostridiaceae bacterium
Skillet-1
HAW16513.1
Clostridiales bacterium
Skillet-1 ? ? HTH
MBR2548382.1
Clostridiales bacterium
Skillet-1
MBR3533265.1
Clostridiales bacterium
Skillet-1 ? ? ? HTH
MBO4579530.1
Clostridiales bacterium
HTH Skillet-1
MBO4580225.1
Clostridiales bacterium
Skillet-1
OPL11458.1
Firmicutes bacterium ML8_F2
Skillet-1
MBQ2082081.1
Lachnospiraceae bacterium
Skillet-1
MBR4768522.1
Lachnospiraceae bacterium
Skillet-1
MBO4411203.1
Lachnospiraceae bacterium
Skillet-1
MBR2027397.1
Oscillospiraceae bacterium
Skillet-1
MBR3355967.1
Oscillospiraceae bacterium
HTH ? Skillet-1
MBR3043945.1
Oscillospiraceae bacterium
Skillet-1
MBR3447671.1
Oscillospiraceae bacterium
Skillet-1
MBR5125416.1
Oscillospiraceae bacterium
Skillet-1
CCY59699.1
Clostridium sp. CAG:632
HTH ? Skillet-1
RYG35004.1
bacterium
Skillet-1
MBN2415240.1
bacterium
Skillet-1
NNF96699.1
Halobacteria archaeon
Skillet-1
MBP1776389.1
Candidatus Methylomirabilota bacterium
Skillet-1
RLB97499.1
Deltaproteobacteria bacterium
Skillet-1
RTZ99161.1
Deltaproteobacteria bacterium
Skillet-1
HET58105.1
Deltaproteobacteria bacterium
Skillet-1
MBC8393078.1
Deltaproteobacteria bacterium
Skillet-1
MBN1227285.1
Deltaproteobacteria bacterium
Skillet-1
MBN1228024.1
Skillet-1
MBN1828380.1
Deltaproteobacteria bacterium
Skillet-1
MBN1831573.1
Deltaproteobacteria bacterium
Skillet-1
MBN1847504.1
Deltaproteobacteria bacterium
Skillet-1
MBN1849759.1
Deltaproteobacteria bacterium
Skillet-1
MBN2180177.1
Deltaproteobacteria bacterium
Skillet-1
MBT8352612.1
Deltaproteobacteria bacterium
Skillet-1
MBW1691240.1
Deltaproteobacteria bacterium
Skillet-1
MBW1898677.1
Deltaproteobacteria bacterium
Skillet-1
MBW1919236.1
Deltaproteobacteria bacterium
Skillet-1
MBW2055051.1
Deltaproteobacteria bacterium
Skillet-1
MBW2109029.1
Deltaproteobacteria bacterium
Skillet-1
MBW2298131.1
Deltaproteobacteria bacterium
Skillet-1
MBW2640670.1
Deltaproteobacteria bacterium
Skillet-1
UCG63381.1
Deltaproteobacteria bacterium
Skillet-1
KPJ75363.1
Deltaproteobacteria bacterium SG8_13
Skillet-1
CAB1062029.1
Olavius sp. associated proteobacterium Delta 1
Skillet-1
MBN1387592.1
Bacteroidales bacterium
Skillet-1
KPK31287.1
Nitrospira bacterium SG8_3
Skillet-1
KPK23920.1
Nitrospira bacterium SG8_3
Skillet-1
MBE9556864.1
Pseudomonadota bacterium
Skillet-1
MBU0992960.1
Pseudomonadota bacterium
Skillet-1
MBU2549215.1
Pseudomonadota bacterium
Skillet-1
UCH49516.1
Betaproteobacteria bacterium
Skillet-1
KPK27288.1
Betaproteobacteria bacterium SG8_40
Skillet-1
MBT3057528.1
Candidatus Thiodiazotropha sp. (ex Lucina pensylvanica)
Skillet-1
MAF83832.1
Chromatiales bacterium
Skillet-1
MAF84210.1
Chromatiales bacterium
Skillet-1
MBT77401.1
Chromatiales bacterium
Skillet-1
UCG74039.1
Chromatiales bacterium
Skillet-1
RKZ75905.1
Candidatus Parabeggiatoa sp. nov. 1
Skillet-1
RLA41236.1
Gammaproteobacteria bacterium
Skillet-1
NCF58611.1
Gammaproteobacteria bacterium
Skillet-1
HHM05519.1
Gammaproteobacteria bacterium
Skillet-1
MBI3772848.1
Gammaproteobacteria bacterium
Skillet-1
MBT8068258.1
Gammaproteobacteria bacterium
Skillet-1
MBT8442938.1
Gammaproteobacteria bacterium
Skillet-1
KPJ92534.1
Gammaproteobacteria bacterium SG8_11
Skillet-1
KPK38433.1
Gammaproteobacteria bacterium SG8_47
Skillet-1
HID99467.1
Thiotrichaceae bacterium
Skillet-1
ACL06836.1
Desulfatibacillum aliphaticivorans
Skillet-1
MBI9076433.1
Desulfatibacillum sp.
Skillet-1
RJP45720.1
Desulfobacteraceae bacterium
Skillet-1
RPJ21777.1
Desulfobacteraceae bacterium
Skillet-1
HDH88493.1
Desulfobacteraceae bacterium
Skillet-1
NVM57153.1
Desulfobacterales bacterium
Skillet-1
KPK26881.1
Desulfobacterales bacterium SG8_35_2
Skillet-1
MBA4366525.1
Desulfobacterium sp.
Skillet-1
WP_139445443.1
Desulfobotulus mexicanus
Skillet-1
WP_041280625.1
Desulfosudis oleivorans Hxd3
Skillet-1
NOQ50640.1
Desulfuromonadaceae bacterium
Skillet-1
MCD6153731.1
Syntrophobacterales bacterium
Skillet-1
KOY86985.1
bacterium 336/3
Skillet-1
TDL99857.1
Flavobacteriaceae bacterium
Skillet-1
RYD52640.1
Sphingobacteriales bacterium
Skillet-1
MBK8089325.1
Chitinophagaceae bacterium
Skillet-1
MBU2020597.1
Bacteroidota bacterium
Skillet-1
WP_026768770.1
Skillet-1
MBV9986884.1
Chitinophagaceae bacterium
Skillet-1
MCE3282949.1
Chitinophagaceae bacterium
Skillet-1
MBY0347163.1
Hydrotalea flava
Skillet-1
MBA4012162.1
Phenylobacterium sp.
Skillet-1
MBT9469974.1
Phenylobacterium sp.
Skillet-1
ODT57514.1
Phenylobacterium sp. SCN 69-14
Skillet-1
PZQ62300.1
Phenylobacterium zucineum
Skillet-1
MBS2016640.1
Deltaproteobacteria bacterium
Skillet-1
MBL8610214.1
Myxococcales bacterium
Skillet-1
MBK8259533.1
Polyangiaceae bacterium
Skillet-1

Accession	Organism	Gene neighborhood
RYY59103.1	*Chitinophagaceae bacterium*	Skillet-1
NOT91857.1	*Ferruginibacter sp.*	Skillet-1
MBP6144501.1	*Sediminibacterium sp.*	Skillet-1
MBQ4021075.1	*Bacteroidales bacterium*	Skillet-1
MBP5559379.1	*Bacteroidales bacterium*	Skillet-1
KKA01519.1	*Hanseniaspora uvarum DSM 2768*	Skillet-1
OBA29225.1	*Hanseniaspora valbyensis NRRL Y-1626*	Skillet-1
NCF38274.1	*Gammaproteobacteria bacterium*	Skillet-1
MBT8436446.1	*Gammaproteobacteria bacterium*	Skillet-1
HBB13652.1	*Treponema sp.*	Skillet-1
MBQ6568345.1	*Treponema sp.*	Skillet-1
MBP5568347.1	*Treponema sp.*	Skillet-1
KKS33035.1	*Candidatus Amesbacteria bacterium GW2011_GWA2_42_12*	Skillet-1
OGZ27905.1	*Candidatus Nealsonbacteria bacterium RIFOXYC1_FULL_40_7*	Skillet-1
MBI3619996.1	*Candidatus Roizmanbacteria bacterium*	Skillet-1
MBI3559200.1	*Candidatus Gottesmanbacteria bacterium*	Skillet-1
MBL0135267.1	*Chitinophagaceae bacterium*	Skillet-1
MBY0536730.1	*Chitinophagaceae bacterium*	Skillet-1
MBA4418195.1	*Syntrophus sp. (in: bacteria)*	Skillet-1
MCA9620362.1	*Myxococcales bacterium*	FA_desaturase | Skillet-1
MCC6644250.1	*Polyangiaceae bacterium*	FA_desaturase | Skillet-1
MCC8119585.1	*Bacteroidales bacterium*	Skillet-1 | vWA
WP_076561902.1	*Chryseobacterium indoltheticum*	Skillet-1 | ??
RRD00948.1	*Prevotella sp. OH937_COT-195*	Skillet-1
MBK8637877.1	*Chromatiaceae bacterium*	Skillet-1
NNF16822.1	*Gammaproteobacteria bacterium*	Skillet-1
NHF59878.1	*Pelagihabitans pacificus*	Skillet-1
WP_216800485.1	*Geomonas oryzisoli*	Skillet-1
OFX47446.1	*Bacteroidetes bacterium GWA2_33_15*	Skillet-1
WP_114338822.1	*Corallincola holothuriorum*	Skillet-1
TKB77859.1	*Nitrospira sp.*	Skillet-1
MBQ4639451.1	*Clostridia bacterium*	Skillet-1
MCB5285234.1	*Candidatus Cloacimonadota bacterium*	Skillet-1
MBP3542016.1	*Clostridia bacterium*	Skillet-1
MBT5950617.1	*Betaproteobacteria bacterium*	Skillet-1
EJW20742.1	*alpha proteobacterium IMCC14465*	Skillet-1
MBA3890606.1	*Gemmatimonadaceae bacterium*	Skillet-1
SEI81795.1	*Lachnospiraceae bacterium A10*	Skillet-1
WP_067096882.1	*Synechococcus sp. MIT S9508*	Skillet-1
MBI5389001.1	*Candidatus Woesearchaeota archaeon*	Skillet-1
MCA9835373.1	*Trueperaceae bacterium*	Skillet-1
CRK75240.1	*Nereida ignava*	Skillet-1
MBU0470879.1	*Nanoarchaeota archaeon*	Skillet-1
MBP5576054.1	*Treponema sp.*	Skillet-1
WP_009196844.1	*Cesiribacter andamanensis AMV16*	Skillet-1
MBT8135614.1	*Gammaproteobacteria bacterium*	Skillet-1
NNC82286.1	*Flavobacteriales bacterium*	Skillet-1
MBA3502626.1	*Deltaproteobacteria bacterium*	Skillet-1
MBT4604001.1	*archaeon*	Skillet-1
MBX3199516.1	*Labilithrix sp.*	Skillet-1
MBZ1350600.1	*Zwartia hollandica*	Skillet-1
RPI03228.1	*Ignavibacteriota bacterium*	Skillet-1
MCC8992010.1	*Streptococcus sp.*	Skillet-1
MBQ0736167.1	*Aquimarina celericrescens*	Skillet-1
MBR6785786.1	*Clostridia bacterium*	Skillet-1
MBP1741275.1	*Deltaproteobacteria bacterium*	Skillet-1
MBQ0116004.1	*Candidatus Sodaliphilus aphodohippi*	Skillet-1
MBW1894534.1	*Deltaproteobacteria bacterium*	Skillet-1
HCE85612.1	*Bacteroidota bacterium*	Skillet-1
MBS0659353.1	*Verrucomicrobiota bacterium*	Skillet-1
PSR03535.1	*Bacteroidetes bacterium SW_8_64_56*	Skillet-1
MBW2219784.1	*Deltaproteobacteria bacterium*	Skillet-1
RLJ08067.1	*Candidatus Aenigmatarchaeota archaeon*	Skillet-1
MBI5477539.1	*Deltaproteobacteria bacterium*	Skillet-1
RUA19199.1	*Flavobacteriia bacterium*	Skillet-1
MCB1185320.1	*bacterium*	Skillet-1
HHH47304.1	*Gammaproteobacteria bacterium*	Skillet-1
MBD5233972.1	*Bacteroidales bacterium*	Skillet-1

###############;Skillet-2 associations; ###most common association with helix+TM or some elaboration on that core. That protein often contains an upstream ZnR. In the first set, there is a further possible association with a FAD-NAD-dependent oxidoreductase

Accession	Organism	Gene neighborhood
TYP96466.1	*Tenacibaculum adriaticum*	unk2-N | unk2-C | ? | FAD-NAD-dep-oxidoreductase | ZnR | helix | Skillet-2 | ? | ? | ? | TM(frag) | helix
MCB0760568.1	*Flavobacteriales bacterium*	FAD-NAD-dep-oxidoreductase | ? | ZnR | helix | Skillet-2
MBI1267037.1	*Cryomorphaceae bacterium*	FAD-NAD-dep-oxidoreductase | ? | ZnR | helix | Skillet-2
WP_218732827.1	*Polaribacter sp. HaHaR_3_91*	unk2-N | unk2-C | ? | ? | ? | ? | FAD-NAD-dep-oxidoreductase | ZnR | helix | Skillet-2
WP_188597397.1	*Polaribacter pacificus*	unk2-N | unk2-C | FAD-NAD-dep-oxidoreductase | ZnR | helix | Skillet-2
AZJ31159.1	*Tenacibaculum mesophilum*	unk2-N | unk2-C | ? | FAD-NAD-dep-oxidoreductase | ZnR | helix | Skillet-2 | HTH | SHS2
WP_028887732.1	*Tenacibaculum ovolyticum DSM 18103*	unk2-N | unk2-C | ? | FAD-NAD-dep-oxidoreductase | ZnR | helix | Skillet-2
WP_159244229.1	*Tenacibaculum maritimum*	unk2-N | unk2-C | FAD-NAD-dep-oxidoreductase | ZnR | helix | Skillet-2
WP_091891722.1	*Polaribacter sp. KT25b*	FAD-NAD-dep-oxidoreductase | ZnR | helix | Skillet-2 | HTH | SHS2
WP_093870692.1	*Tenacibaculum sp. MAR_2010_89*	FAD-NAD-dep-oxidoreductase | ZnR | helix | Skillet-2 | HTH | SHS2
WP_038006153.1	*Tenacibaculum sp. 47A_GOM-205m*	unk2-N | unk2-C | ? | FAD-NAD-dep-oxidoreductase | ZnR | helix | Skillet-2
WP_187481131.1	*Polaribacter pectinis*	unk2-N | unk2-C | FAD-NAD-dep-oxidoreductase | ZnR | helix | Skillet-2 | HTH | SHS2
WP_158840908.1	*Polaribacter sp. L3A8*	unk2-N | unk2-C | FAD-NAD-dep-oxidoreductase | ZnR | helix | Skillet-2
WP_068706093.1	*Tenacibaculum soleae*	unk2-N | unk2-C | ? | FAD-NAD-dep-oxidoreductase | ZnR | helix | Skillet-2 | HTH | SHS2
WP_218772946.1	*Tenacibaculum sp. AHE14PA*	unk2-N | unk2-C | ? | FAD-NAD-dep-oxidoreductase | ZnR | helix | Skillet-2 | ? | HTH | SHS2
PQJ23032.1	*Tenacibaculum sp. SG-28*	FAD-NAD-dep-oxidoreductase | ZnR | helix | Skillet-2
NVK51361.1	*Flavobacteriaceae bacterium*	unk2-N | unk2-C | FAD-NAD-dep-oxidoreductase | ZnR | helix | Skillet-2 | HTH | SHS2
RSC94077.1	*Tenacibaculum singaporense*	unk2-N | unk2-C | ? | FAD-NAD-dep-oxidoreductase | ZnR | helix | Skillet-2 | ? | HTH | SHS2
KOY50838.1	*Polaribacter dokdonensis DSW-5*	unk2-C | unk2-N | FAD-NAD-dep-oxidoreductase | helix | ZnR | ? | Skillet-2
WP_233883459.1	*Tenacibaculum piscium*	unk2-N | unk2-C | ? | helix | ZnR | Skillet-2
WP_058885516.1	*Tenacibaculum finnmarkense genomovar ulcerans*	unk2-N | unk2-C | ? | helix | ZnR | Skillet-2

#;another limited set of associations, this one may be informative: GHMP-like kinase and HMG-CoA_red could combine to act on an isoprenoid precursor. could be swapping an OH-containing molecule for the phosphate group of 5-Phosphomevalonate.

Accession	Organism	Gene neighborhood
NNC82547.1	*Flavobacteriales bacterium*	IG-repeats | ZnR | Skillet-2
WP_014203851.1	*Owenweeksia hongkongensis DSM 17368*	IG-repeats | GHMP-kinase-like | HMG-CoA_red | ZnR | Skillet-2

WP_210758976.1
Croceimicrobium hydrocarbonivorans
IG-repeats GHMP-kinase-like HMG-CoA_red ZnR Skillet-2

NVK04292.1
Flavobacteriia bacterium
IG-repeats GHMP-kinase-like HMG-CoA_red ZnR Skillet-2

WP_151693292.1
Phaeocystidibacter marisrubri
IG-repeats GHMP-kinase-like HMG-CoA_red ZnR Skillet-2

MBA2328928.1
Flavisolibacter sp.
helix Skillet-2 ? PSE IG-repeats

PTM05274.1
Bacteroidota bacterium
IG-repeats GHMP-kinase-like HMG-CoA_red ZnR Skillet-2

MCE2815657.1
Cryomorphaceae bacterium
helix Skillet-2 HMG-CoA_red GHMP-kinase-like IG-repeats

WP_151667170.1
Phaeocystidibacter luteus
PBP HTH ? ? ? IG-repeats GHMP-kinase-like HMG-CoA_red ZnR Skillet-2

NVK27426.1
Flavobacteriia bacterium
PBP HTH ? ? ? IG-repeats GHMP-kinase-like HMG-CoA_red ZnR Skillet-2

MBT3678323.1
Flavobacteriales bacterium
helix Skillet-2 HMG-CoA_red GHMP-kinase-like IG-repeats

KRO72982.1
Cryomorphaceae bacterium BACL18 MAG-120924-bin36
helix Skillet-2 HMG-CoA_red GHMP-kinase-like IG-repeats

KRO69083.1
Cryomorphaceae bacterium BACL7 MAG-120322-bin74
helix Skillet-2 NUDIX wHTH SSF HMG-CoA_red GHMP-kinase-like IG-repeats

MBL6627389.1
Cryomorphaceae bacterium
helix Skillet-2 NUDIX wHTH SSF HMG-CoA_red GHMP-kinase-like IG-repeats

NQW41690.1
Cryomorphaceae bacterium
helix Skillet-2 HMG-CoA_red GHMP-kinase-like IG-repeats

MCC5918265.1
Cryomorphaceae bacterium
GHMP-kinase-like HMG-CoA_red ZnR Skillet-2

#;association with Rossmann-MTase

WP_233757602.1
Algoriphagus sp. AGSA1
a-helical Rossman-MTase ZnR helix Skillet-2

AEL25435.1
Cyclobacterium marinum DSM 745
ZnR helix Skillet-2 a-helical Rossman-MTase

WP_232826707.1
Cognataquiflexum aquatile
ZnR helix Skillet-2 a-helical Rossman-MTase

WP_089240094.1
Belliella buryatensis
ZnR helix Skillet-2 a-helical Rossman-MTase

WP_154855172.1
Cyclobacterium xiamenense
ZnR helix Skillet-2 a-helical Rossman-MTase

WP_189586684.1
Mongoliitalea lutea
ZnR helix Skillet-2 a-helical Rossman-MTase

WP_143962725.1
Litoribacter populi
ZnR helix Skillet-2 ? a-helical Rossman-MTase

MBD3630658.1
Cyclobacterium sp.
ZnR helix Skillet-2 a-helical Rossman-MTase

WP_158856060.1
Lunatibacter salilacus
ZnR helix Skillet-2 a-helical Rossman-MTase

WP_232834793.1
Pleomorphovibrio marinus
ZnR helix Skillet-2 a-helical Rossman-MTase

TVP44510.1
Mongoliibacter sp.
Skillet-2 a-helical Rossman-MTase

#;with GNAT

WP_106931834.1
Adhaeribacter arboris
GNAT ZnR helix Skillet-2 GNAT

WP_115374057.1
Adhaeribacter pallidiroseus
GNAT ? ZnR helix Skillet-2 GNAT

WP_146898159.1
Adhaeribacter aerolatus
GNAT ZnR helix Skillet-2 GNAT

#;possible transcription factor regulator in neighborhood

MBL0911423.1
Bacteroidia bacterium
TetR HTH helix ZnR Skillet-2

WP_207435467.1
Sabulibacter ruber
TetR HTH helix ZnR Skillet-2

WP_147420351.1
Sphingobacterium detergens
TetR HTH helix Skillet-2

PSL25346.1
Chitinophaga ginsengisoli
TetR HTH helix Skillet-2

MBB2950515.1
TetR HTH helix Skillet-2

WP_202100977.1
Sphingobacterium faecale
TetR HTH helix Skillet-2

WP_089765753.1
Chitinophaga terrae (ex Kim and Jung 2007)
TetR HTH helix Skillet-2

MBP1791650.1
TetR HTH helix Skillet-2

WP_169191245.1
Chitinophaga sp. Ak27
TetR HTH helix Skillet-2

MBO9729241.1
Chitinophaga sp.
TetR HTH helix Skillet-2

WP_123873701.1
Chryseobacterium flavum
TetR HTH TM(fragment) helix Skillet-2

WP_127043898.1
TetR HTH helix Skillet-2

SHK67050.1
Chryseobacterium polytrichastri
TetR HTH TM(fragment) helix Skillet-2

WP_111599539.1
Chitinophaga skermanii
TetR HTH helix Skillet-2

WP_121488802.1
Chryseobacterium sp. 7
TetR HTH TM(fragment) helix Skillet-2

WP_235552776.1
Niabella agricola
TetR HTH helix Skillet-2

WP_048505957.1
Chryseobacterium angstadtii
TetR HTH TM(fragment) helix Skillet-2

WP_073335164.1
Chryseobacterium carnipullorum
TetR HTH TM(fragment) helix Skillet-2

WP_099768486.1
Chryseobacterium sp. 52
TetR HTH TM(fragment) helix Skillet-2

WP_223712300.1
Niabella beijingensis
TetR HTH helix Skillet-2

WP_126245241.1
Chitinophaga rhizosphaerae
TetR HTH helix Skillet-2

WP_172280139.1
Chryseobacterium sp. LAM-KRS1
TetR HTH TM(fragment) helix Skillet-2

WP_184556960.1
Chryseobacterium sediminis
TetR HTH TM(fragment) helix Skillet-2

WP_046674493.1
Sphingobacterium sp. Ag1
TetR HTH helix Skillet-2

WP_109693899.1
Chitinophaga deserti
TetR HTH helix Skillet-2

WP_205942023.1
Pedobacter chitinilyticus
TetR HTH helix Skillet-2

MBN9296023.1
Filimonas sp.
TetR HTH helix Skillet-2

MBB2871765.1
TetR HTH helix Skillet-2

RKF42335.1
Sphingobacterium siyangense
TetR HTH helix Skillet-2

PUV25932.1
Sphingobacterium athyrii
TetR HTH helix Skillet-2

WP_200248218.1
Chryseobacterium paridis
TetR HTH TM(fragment) helix Skillet-2

MBN9293350.1
Flavobacteriia bacterium
TetR HTH helix ZnR Skillet-2

WP_146060575.1
Sphingobacterium lactis
TetR HTH helix Skillet-2

WP_027374673.1
Chryseobacterium indologenes
TetR HTH TM(fragment) helix Skillet-2

WP_232720851.1
Chryseobacterium gleum
TetR HTH TM(fragment) helix Skillet-2

WP_106914842.1
Chryseobacterium aurantiacum
TetR HTH TM(fragment) helix Skillet-2

WP_044217629.1
Phaeodactylibacter xiamenensis
TetR HTH ? Skillet-2

GGC48521.1
Parapedobacter defluvii
TetR HTH ? ? ? Skillet-2

MCB0595720.1
Phaeodactylibacter sp.
TetR HTH ? Skillet-2

WP_130541124.1
Pseudobacter ginsenosidimutans
TetR HTH ? Skillet-2

MBX7142044.1
Chitinophagales bacterium
HTH TetR Skillet-2

WP_066679672.1
Chryseobacterium sp. BGARF1
TetR HTH ? Skillet-2

MBX2874423.1
Saprospiraceae bacterium
TetR HTH ? Skillet-2

MBT2623017.1
Chryseobacterium sp. ISL-6
HTH TetR Skillet-2

MCB9277376.1
Lewinellaceae bacterium
TetR HTH ? Skillet-2

MBK6642010.1
Bacteroidota bacterium
HTH TetR Skillet-2

MCB0632659.1
Lewinella sp.
TetR HTH ? Skillet-2

WP_020539357.1
Lewinella cohaerens DSM 23179
TetR HTH ? Skillet-2

MBK7873574.1
Saprospiraceae bacterium
TetR HTH ? Skillet-2

GJM34386.1
Saprospiraceae bacterium
TetR HTH ? Skillet-2

MCB9273996.1
Lewinellaceae bacterium
TetR HTH ? Skillet-2

MBK6619936.1
Saprospirales bacterium
TetR HTH ? Skillet-2

MCB0556168.1
Phaeodactylibacter sp.
TetR HTH ? Skillet-2

WP_147167466.1
Phaeodactylibacter luteus
TetR HTH ? Skillet-2

PHN02032.1
Flavilitoribacter nigricans DSM 23189 = NBRC 102662
TetR HTH ? Skillet-2

MBV6652203.1
Phaeodactylibacter sp.
TetR HTH ? Skillet-2

NBC08817.1
Bacteroidota bacterium
TetR HTH ? Skillet-2

#;distinct TF

WP_163283227.1
Cryomorpha ignava
HTH PBP ZnR helix Skillet-2

TVR38631.1
Cryomorphaceae bacterium
HTH PBP ZnR helix Skillet-2

MCA1762090.1
Flavobacteriales bacterium
HTH PBP ZnR helix Skillet-2

WP_149034209.1
Skillet-2 PSE

#;additional L17-ClpS-related domain in the helix+TM component

Accession	Organism	Architecture
WP_118641403.1	*Pseudoflavonifractor sp. AF19-9AC*	HTH ZnR L7/L12-ClpS-related Skillet-2
HJB53903.1	*Candidatus Oscillibacter pullicola*	ZnR L7/L12-ClpS-related Skillet-2
MBS6290302.1	*Oscillibacter sp.*	HTH ZnR L7/L12-ClpS-related Skillet-2
MBD9197649.1	*Clostridiales bacterium*	HTH ZnR L7/L12-ClpS-related Skillet-2
MBR1690493.1	*Oscillibacter sp.*	HTH ZnR L7/L12-ClpS-related Skillet-2
MBD9197683.1	*Clostridiales bacterium*	HTH ZnR L7/L12-ClpS-related Skillet-2
MBR4954249.1	*Oscillospiraceae bacterium*	HTH L7/L12-ClpS-related Skillet-2
WP_143072729.1	*Oscillibacter sp. PC13*	HTH ZnR L7/L12-ClpS-related Skillet-2
WP_006572962.1	*Pseudoflavonifractor capillosus ATCC 29799*	HTH L7/L12-ClpS-related Skillet-2 ZnR L17-ClpS-related

#;also with a GNAT

Accession	Organism	Architecture
MBQ3535025.1	*Clostridia bacterium*	HTH ZnR L7/L12-ClpS-related Skillet-2 GNAT
MBR5244537.1	*Clostridia bacterium*	HTH ZnR L7/L12-ClpS-related Skillet-2 GNAT
MBR4992123.1	*Clostridia bacterium*	HTH ZnR L7/L12-ClpS-related Skillet-2 GNAT

#;Just the ZnR+helix

Accession	Organism	Architecture
WP_231739662.1	*Flavobacterium sp. TAB 87*	ZnR helix Skillet-2 ? helix TM(frag)
WP_022828902.1	*Flavobacterium antarcticum*	helix TM(frag) ZnR helix Skillet-2
MBC5840627.1	*Flavobacterium kayseriense*	ZnR helix Skillet-2 helix TM(frag)
WP_215594859.1	*Hymenobacter piscis*	ZnR helix Skillet-2
WP_150452522.1	*Arenibacter lacus*	ZnR helix Skillet-2
WP_051315801.1	*Arenibacter certesii*	ZnR helix Skillet-2
WP_051254240.1	*Arenibacter latericius DSM 15913*	ZnR helix Skillet-2
WP_068837780.1	*Pontibacter akesuensis*	ZnR helix Skillet-2
PRX54659.1	*Flagellimonas meridianipacifica*	ZnR helix Skillet-2
MBK7129372.1	*Crocinitomicaceae bacterium*	ZnR helix Skillet-2
MBK9193077.1	*Crocinitomicaceae bacterium*	ZnR helix Skillet-2
WP_210469302.1	*Sporosarcina sp. 6E9*	ZnR helix Skillet-2
WP_140465428.1	*Hymenobacter nivis*	ZnR helix Skillet-2
TVQ80320.1	*Flavobacteriales bacterium*	ZnR helix Skillet-2
WP_179197704.1	*Hymenobacter crusticola*	ZnR helix Skillet-2
WP_170865205.1	*Hymenobacter psychrotolerans DSM 18569*	ZnR helix Skillet-2
WP_155522691.1	*[Bacillus] selenitireducens MLS10*	ZnR helix Skillet-2
WP_201429382.1	*Marivirga aurantiaca*	ZnR helix Skillet-2
MBU4487124.1	*Candidatus Delongbacteria bacterium*	ZnR helix Skillet-2
WP_160849752.1	*Pontibacillus yanchengensis*	ZnR helix Skillet-2
WP_085517203.1	*Marivirga sericea*	ZnR helix Skillet-2
MBT2559378.1	*Hymenobacter sp. ISL-91*	ZnR helix Skillet-2
HEH07054.1		ZnR helix Skillet-2
WP_123877544.1	*Flagellimonas maritima*	ZnR helix Skillet-2
WP_115360579.1	*Sporosarcina pasteurii*	ZnR helix Skillet-2
WP_206983783.1	*Hymenobacter telluris*	ZnR helix Skillet-2
MCB9224042.1	*Crocinitomicaceae bacterium*	ZnR helix Skillet-2
WP_150903151.1	*Adhaeribacter soli*	ZnR helix Skillet-2
MBI3135033.1	*Bacteroidota bacterium*	ZnR helix Skillet-2
NVK65413.1	*Flavobacteriales bacterium*	ZnR helix Skillet-2
MBU2268048.1	*Bacteroidota bacterium*	ZnR helix Skillet-2
RYF85663.1	*Chitinophagaceae bacterium*	ZnR helix Skillet-2
WP_138940566.1	*Aquimarina agarivorans*	ZnR helix Skillet-2
WP_116695693.1	*Marixanthomonas spongiae*	ZnR helix Skillet-2
WP_187318118.1	*Hymenobacter citatus*	ZnR helix Skillet-2
MBC8213173.1	*Fidelibacterota bacterium*	ZnR helix Skillet-2
WP_125221913.1	*Maribacter algicola*	ZnR helix Skillet-2
HAA01078.1	*Flavobacteriales bacterium*	ZnR helix Skillet-2
RYD71058.1	*Sphingobacteriales bacterium*	ZnR helix Skillet-2
WP_006259544.1	*Myroides odoratimimus*	ZnR helix Skillet-2
MCB9196672.1	*Flavobacteriales bacterium*	ZnR helix Skillet-2
WP_138078984.1	*Hymenobacter jeollabukensis*	ZnR helix Skillet-2
MBP9150766.1	*Flavobacteriales bacterium*	ZnR helix Skillet-2
WP_211222903.1	*Gelidibacter mesophilus DSM 14095*	ZnR helix Skillet-2
HAW19950.1	*Flavobacteriales bacterium*	ZnR helix Skillet-2
WP_169605535.1	*Mucilaginibacter robiniae*	ZnR helix Skillet-2
MAD96682.1	*Flavobacteriaceae bacterium*	ZnR helix Skillet-2
NQV53732.1	*Flavobacteriales bacterium*	ZnR helix Skillet-2
GGF71362.1	*Wenyingzhuangia marina*	ZnR helix Skillet-2
MCB9188775.1	*Flavobacteriales bacterium*	ZnR helix Skillet-2
MCC5932375.1	*Cyclobacteriaceae bacterium*	ZnR helix Skillet-2
RZJ93774.1	*Hymenobacter sp.*	ZnR helix Skillet-2
RZK28333.1	*Hymenobacter sp.*	ZnR helix Skillet-2
MBS1521875.1	*Bacteroidota bacterium*	ZnR helix ZnR helix Skillet-2
MBE7174943.1	*Mucilaginibacter polytrichastri*	ZnR helix Skillet-2
MBD3750560.1	*Sphingobacteriales bacterium*	ZnR helix Skillet-2
WP_188548531.1	*Hymenobacter qilianensis*	ZnR helix Skillet-2
WP_133685759.1	*Maribacter spongiicola*	ZnR helix Skillet-2
TAH02698.1	*Sphingobacteriales bacterium*	ZnR helix Skillet-2
HAD10837.1	*Saprospirales bacterium*	ZnR helix Skillet-2
MCB9192481.1	*Flavobacteriales bacterium*	ZnR helix Skillet-2
WP_073569809.1	*Algoriphagus zhangzhouensis*	ZnR helix Skillet-2
WP_089683472.1	*Catalinimonas alkaloidigena*	ZnR helix Skillet-2
WP_144603137.1	*uncultured Algoriphagus sp.*	ZnR helix Skillet-2
WP_161889628.1	*Pontibacter russatus*	ZnR helix Skillet-2
WP_219876150.1	*Pontibacter aydingkolensis*	ZnR helix Skillet-2
WP_125047741.1	*Pontibacter arcticus*	ZnR helix Skillet-2
WP_221834350.1	*Paracrocinitomix mangrovi*	ZnR helix Skillet-2 >
WP_220612553.1	*Hymenobacter sp. HSC-4F20*	ZnR helix Skillet-2
WP_119432321.1	*Pontibacter oryzae*	ZnR helix Skillet-2
WP_225600640.1	*Myroides odoratimimus*	ZnR helix Skillet-2
MCB0820450.1	*Bacteroidota bacterium*	ZnR helix Skillet-2
WP_073406934.1	*Mucilaginibacter sp. OK098*	ZnR helix Skillet-2
WP_147030077.1	*Mucilaginibacter ginsenosidivorans*	ZnR helix ZnR helix Skillet-2
MCC7453016.1	*Crocinitomix sp.*	ZnR helix Skillet-2
WP_048921345.1	*Rufibacter radiotolerans*	ZnR helix Skillet-2
WP_144911657.1	*Mucilaginibacter frigoritolerans*	ZnR helix Skillet-2
WP_186636648.1	*Rufibacter sediminis*	ZnR helix Skillet-2
WP_112087041.1	*Flavobacterium lacus*	ZnR helix Skillet-2

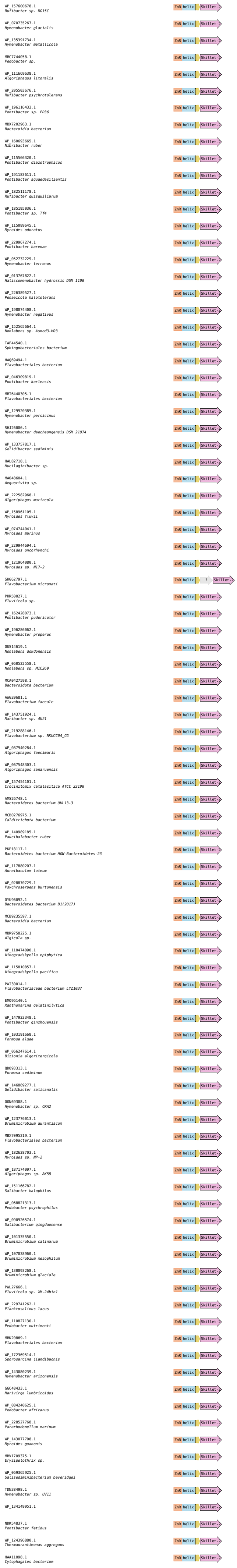
WP_157600678.1
Rufibacter sp. DG15C
ZnR helix Skillet
WP_070735267.1
Hymenobacter glacialis
ZnR helix Skillet
WP_135391734.1
Hymenobacter metallicola
ZnR helix Skillet
MBC7744058.1
Pedobacter sp.
ZnR helix Skillet
WP_111669638.1
Algoriphagus litoralis
ZnR helix Skillet
WP_205503676.1
Rufibacter psychrotolerans
ZnR helix Skillet
WP_196116433.1
Pontibacter sp. FD36
ZnR helix Skillet
MBX7202963.1
Bacteroidia bacterium
ZnR helix Skillet
WP_160693665.1
Nibribacter ruber
ZnR helix Skillet
WP_115566320.1
Pontibacter diazotrophicus
ZnR helix Skillet
WP_191183611.1
Pontibacter aquaedesilientis
ZnR helix Skillet
WP_182511178.1
Rufibacter quisquiliarum
ZnR helix Skillet
WP_185195036.1
Pontibacter sp. Tf4
ZnR helix Skillet
WP_115089645.1
Myroides odoratus
ZnR helix Skillet
WP_229967274.1
Pontibacter harenae
ZnR helix Skillet
WP_052732229.1
Hymenobacter terrenus
ZnR helix Skillet
WP_013767822.1
Haliscomenobacter hydrossis DSM 1100
ZnR helix Skillet
WP_226389527.1
Penaeicola halotolerans
ZnR helix Skillet
WP_198074408.1
Hymenobacter negativus
ZnR helix Skillet
WP_152565664.1
Nonlabens sp. Asnod3-H03
ZnR helix Skillet
TAF44540.1
Sphingobacteriales bacterium
ZnR helix Skillet
HAQ69494.1
Flavobacteriales bacterium
ZnR helix Skillet
WP_046309819.1
Pontibacter korlensis
ZnR helix Skillet
MBT6440305.1
Flavobacteriales bacterium
ZnR helix Skillet
WP_129920385.1
Hymenobacter persicinus
ZnR helix Skillet
SHJ26006.1
Hymenobacter daecheongensis DSM 21074
ZnR helix Skillet
WP_133757817.1
Gelidibacter sediminis
ZnR helix Skillet
HAL82718.1
Mucilaginibacter sp.
ZnR helix Skillet
MAO48604.1
Aequorivita sp.
ZnR helix Skillet
WP_222582968.1
Algoriphagus marincola
ZnR helix Skillet
WP_158961105.1
Myroides fluvii
ZnR helix Skillet
WP_074744041.1
Myroides marinus
ZnR helix Skillet
WP_229944694.1
Myroides oncorhynchi
ZnR helix Skillet
WP_121964080.1
Myroides sp. N17-2
ZnR helix Skillet
SHG62797.1
Flavobacterium micromati
ZnR helix ? Skillet
PHR50027.1
Fluviicola sp.
ZnR helix Skillet
WP_162428073.1
Pontibacter pudoricolor
ZnR helix Skillet
WP_196286062.1
Hymenobacter properus
ZnR helix Skillet
OUS14619.1
Nonlabens dokdonensis
ZnR helix Skillet
WP_060522558.1
Nonlabens sp. MIC269
ZnR helix Skillet
MCA0427598.1
Bacteroidota bacterium
ZnR helix Skillet
AWG20681.1
Flavobacterium faecale
ZnR helix Skillet
WP_143751924.1
Maribacter sp. 4U21
ZnR helix Skillet
WP_219288146.1
Flavobacterium sp. NKUCC04_CG
ZnR helix Skillet
WP_087940284.1
Algoriphagus faecimaris
ZnR helix Skillet
WP_067548303.1
Algoriphagus sanaruensis
ZnR helix Skillet
WP_157454101.1
Crocinitomix catalasitica ATCC 23190
ZnR helix Skillet
AMS26748.1
Bacteroidetes bacterium UKL13-3
ZnR helix Skillet
MCB0276975.1
Calditrichota bacterium
ZnR helix Skillet
WP_140989185.1
Paucihalobacter ruber
ZnR helix Skillet
PKP18117.1
Bacteroidetes bacterium HGW-Bacteroidetes-23
ZnR helix Skillet
WP_117880207.1
Aureibaculum luteum
ZnR helix Skillet
WP_028870729.1
Psychroserpens burtonensis
ZnR helix Skillet
OYU96092.1
Bacteroidetes bacterium B1(2017)
ZnR helix Skillet
MCB9235597.1
Bacteroidia bacterium
ZnR helix Skillet
MBR9758225.1
Algicola sp.
ZnR helix Skillet
WP_110474090.1
Winogradskyella epiphytica
ZnR helix Skillet
WP_115810857.1
Winogradskyella pacifica
ZnR helix Skillet
PWI30014.1
Flavobacteriaceae bacterium LYZ1037
ZnR helix Skillet
EMQ96140.1
Xanthomarina gelatinilytica
ZnR helix Skillet
WP_147923348.1
Pontibacter qinzhouensis
ZnR helix Skillet
WP_103191668.1
Formosa algae
ZnR helix Skillet
WP_066247614.1
Bizionia algoritergicola
ZnR helix Skillet
QDO93313.1
Formosa sediminum
ZnR helix Skillet
WP_146889277.1
Gelidibacter salicanalis
ZnR helix Skillet
OON69308.1
Hymenobacter sp. CRA2
ZnR helix Skillet
WP_123776013.1
Brumimicrobium aurantiacum
ZnR helix Skillet
MBX7095219.1
Flavobacteriales bacterium
ZnR helix Skillet
WP_182628703.1
Myroides sp. NP-2
ZnR helix Skillet
WP_187174097.1
Algoriphagus sp. AK58
ZnR helix Skillet
WP_151166782.1
Salibacter halophilus
ZnR helix Skillet
WP_068821313.1
Pedobacter psychrophilus
ZnR helix Skillet
WP_090926574.1
Salibacterium qingdaonense
ZnR helix Skillet
WP_101335550.1
Brumimicrobium salinarum
ZnR helix Skillet
WP_107038960.1
Brumimicrobium mesophilum
ZnR helix Skillet
WP_130093268.1
Brumimicrobium glaciale
ZnR helix Skillet
PWL27666.1
Fluviicola sp. XM-24bin1
ZnR helix Skillet
WP_229741262.1
Planktosalinus lacus
ZnR helix Skillet
WP_110827130.1
Pedobacter nutrimenti
ZnR helix Skillet
MBK20869.1
Flavobacteriales bacterium
ZnR helix Skillet
WP_172369514.1
Sporosarcina jiandibaonis
ZnR helix Skillet
WP_143080239.1
Hymenobacter arizonensis
ZnR helix Skillet
GGC48433.1
Marivirga lumbricoides
ZnR helix Skillet
WP_084240625.1
Pedobacter africanus
ZnR helix Skillet
WP_228527768.1
Pararhodonellum marinum
ZnR helix Skillet
WP_143077708.1
Myroides guanonis
ZnR helix Skillet
MBV1709375.1
Erysipelothrix sp.
ZnR helix Skillet
WP_069365925.1
Salisediminibacterium beveridgei
ZnR helix Skillet
TDN38498.1
Hymenobacter sp. UV11
ZnR helix Skillet
WP_134149951.1
ZnR helix Skillet
NDK54837.1
Pontibacter fetidus
ZnR helix Skillet
WP_124396880.1
Thermaurantimonas aggregans
ZnR helix Skillet
HAA11098.1
Cytophagales bacterium
ZnR helix Skillet

RYD69216.1
Sphingobacteriales bacterium
ZnR helix Skillet

WP_200506390.1
Adhaeribacter terrigena
ZnR helix Skillet

WP_229962667.1
Pontibacter silvestris
ZnR helix Skillet

WP_152969681.1
Pedobacter sp. Hv1
ZnR helix Skillet

WP_197063062.1
Pontibacillus marinus BH030004 = DSM 16465
ZnR helix Skillet

ARN76558.1
Nonlabens spongiae
ZnR helix Skillet

MAX80670.1
Crocinitomicaceae bacterium
ZnR helix Skillet

MBT3648558.1
Flavobacteriales bacterium
ZnR helix Skillet

WP_118455588.1
Aquimarina sp. BL5
ZnR helix Skillet

MCC5923047.1
Crocinitomicaceae bacterium
ZnR helix Skillet

WP_181186024.1
Alkalicoccus urumqiensis
ZnR helix Skillet

WP_147415810.1
Ulvibacterium marinum
ZnR helix Skillet

WP_146105152.1
Nonlabens arenilitoris
ZnR helix Skillet

WP_124579329.1
Pedobacter sp. KBW06
ZnR helix Skillet

QNK64073.1
Pedobacter sp. PAMC26386
ZnR helix Skillet

WP_007658320.1
Pontibacter sp. BAB1700
ZnR helix Skillet

WP_013489383.1
Evansella cellulosilytica DSM 2522
ZnR helix Skillet

KPM32732.1
Croceitalea dokdonensis DOKDO 023
ZnR helix Skillet

BAO56167.1
Nonlabens marinus S1-08
ZnR helix Skillet

WP_133272816.1
Hymenobacter radiodurans
ZnR helix Skillet

MAW65542.1
Flavobacteriales bacterium
ZnR helix Skillet

MAS51597.1
Flavobacteriales bacterium
ZnR helix Skillet

SDW65095.1
Aequorivita viscosa
ZnR helix Skillet

MBI33947.1
Flavobacteriales bacterium
ZnR helix Skillet

WP_176008179.1
Paenalkalicoccus suaedae
Skillet ZnR heliv

WP_088074059.1
Evansella alkalicola
ZnR helix Skillet

MBK6950549.1
Crocinitomicaceae bacterium
ZnR helix Skillet

WP_153266489.1
Oceanihabitans sp. IOP_32
ZnR helix Skillet

WP_160367529.1
Sphingobacterium humi
helix Skillet

WP_134088610.1
Olivibacter sp. XZL3
helix Skillet

MBO5965875.1
Methanobrevibacter sp.
helix Skillet

WP_148386378.1
Chitinophaga sp. XS-30
helix Skillet

WP_141421655.1
Haloflavibacter putidus
helix Skillet

WP_143305087.1
Chitinophaga vietnamensis
helix Skillet

WP_118195682.1
Albibacterium indicum
helix Skillet

WP_073192247.1
Psychroflexus salarius
helix Skillet

PRD56413.1
Sphingobacterium gobiense
helix Skillet

WP_227694430.1
Sphingobacterium sp. FBM7-1
helix Skillet

MBQ6813860.1
Methanobrevibacter sp.
helix Skillet

WP_187340370.1
Olivibacter sp. SDN3
helix Skillet

MBL7706725.1
Taibaiella sp.
helix Skillet

WP_168874096.1
Chitinophaga varians
helix Skillet

WP_028121403.1
Epilithonimonas tenax DSM 16811
helix TM(frag?) Skillet

MBA2746341.1
Flavisolibacter sp.
helix Skillet

WP_167017108.1
Chitinophaga sp. Cy-1792
helix Skillet

WP_066437428.1
Gelidibacter algens
helix Skillet

WP_187342693.1
Olivibacter sp. SDN3
helix Skillet

WP_133269856.1
Segetibacter sp. 3557_3
helix Skillet

WP_034980092.1
Chryseobacterium carnipullorum
helix TM(frag?) Skillet

MBV8255549.1
Chitinophaga sp.
helix Skillet

WP_013597979.1
Weeksella virosa
helix Skillet

PVH24690.1
Sphingobacterium corticibacter
helix Skillet

AHM59668.1
Flammeovirgaceae bacterium 311
helix Skillet

WP_034972685.1
Chryseobacterium sp. CCH4-E10
helix TM(frag?) Skillet

WP_095839259.1
Chitinophaga pendula
helix Skillet

WP_154662303.1
Vaginella massiliensis
helix Skillet

WP_078672539.1
Chitinophaga eiseniae
helix Skillet

MBI1315690.1
bacterium
helix Skillet

PVH24254.1
Sphingobacterium corticibacter
helix Skillet

MTI20609.1
Fulvivirga aurantia
helix Skillet

WP_224460798.1
Psychroflexus longus
helix Skillet

WP_218379043.1
Chitinophaga sp. sic0106
helix Skillet

WP_111591842.1
Chitinophaga dinghuensis
helix Skillet

WP_234865444.1
Flavihumibacter fluminis
helix Skillet

ATL49225.1
Chitinophaga caeni
helix Skillet

WP_139067221.1
Antarcticibacterium sp. W02-3
helix Skillet

NBR83839.1
Flavobacteriia bacterium
helix Skillet

WP_153043523.1
Psychroflexus sp. S27
helix Skillet

WP_202246243.1
Fulvivirga sediminis
helix Skillet

WP_177222690.1
Chitinophaga sp. YR627
helix Skillet

WP_141877075.1
Gramella sp. Hel_I_59
helix Skillet

WP_026915398.1
Christiangramia portivictoriae DSM 23547
helix Skillet

WP_189604789.1
Salinimicrobium marinum
helix Skillet

WP_143544537.1
Salinimicrobium sediminis
helix Skillet

WP_146833278.1
Antarcticibacterium arcticum
helix Skillet

WP_156879273.1
Salinimicrobium xinjiangense DSM 19287
helix Skillet

WP_143019305.1
Salinimicrobium catena
helix Skillet

WP_139419513.1
Chryseobacterium arthrosphaerae
helix TM(frag?) Skillet

#####;association with MTase-Rossmann

MBL6765358.1
Verrucomicrobiia bacterium
Skillet MTase-Rossmann

MBP7948399.1
Verrucomicrobiales bacterium
Skillet MTase-Rossmann

WP_156345593.1
Verrucomicrobium spinosum DSM 4136 = JCM 18804
Skillet MTase-Rossmann MTase-Rossmann

NBQ23168.1
Verrucomicrobiota bacterium
Skillet MTase-Rossmann

WP_221029358.1
Actomonas aquatica
Skillet MTase-Rossmann

MBT3596718.1
Verrucomicrobiota bacterium
Skillet MTase-Rossmann

WP_087457970.1
Tumebacillus avium
MTase-Rossmann ? ? Skillet

MCC5022584.1
Candidatus Synoicihabitans palmerolidicus
Skillet MTase-Rossmann

WP_007413972.1
Pedosphaera parvula Ellin514
Skillet MTase-Rossmann

MBU6399222.1
Verrucomicrobiota bacterium
Skillet MTase-Rossmann

MBK8000075.1
Verrucomicrobiota bacterium
Skillet MTase-Rossmann

MCC7374339.1
Verrucomicrobiales bacterium
Skillet MTase-Rossmann

MCC7179015.1
Acidobacteriota bacterium
Skillet MTase-Rossmann

NBV23291.1
Pseudomonadota bacterium
Skillet MTase-Rossmann

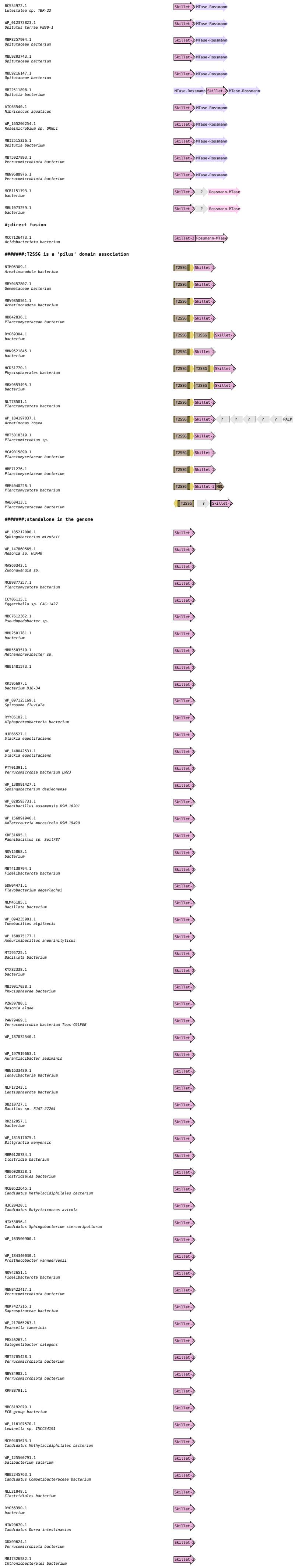
BCS34972.1
Luteitalea sp. TBR-22
Skillet-2 MTase-Rossmann
WP_012373823.1
Opitutus terrae PB90-1
Skillet-2 MTase-Rossmann
MBP8257904.1
Opitutaceae bacterium
Skillet-2 MTase-Rossmann
MBL9203743.1
Opitutaceae bacterium
Skillet-2 MTase-Rossmann
MBL9216147.1
Opitutaceae bacterium
Skillet-2 MTase-Rossmann
MBI2511898.1
Opitutia bacterium
MTase-Rossmann Skillet-2 MTase-Rossmann
ATC63540.1
Nibricoccus aquaticus
Skillet-2 MTase-Rossmann
WP_165206254.1
Roseimicrobium sp. ORNL1
Skillet-2 MTase-Rossmann
MBI2515326.1
Opitutia bacterium
Skillet-2 MTase-Rossmann
MBT5927893.1
Verrucomicrobiota bacterium
Skillet-2 MTase-Rossmann
MBN9688976.1
Verrucomicrobiota bacterium
Skillet-2 MTase-Rossmann
MCB1151793.1
bacterium
Skillet-2 ? Rossmann-MTase
MBU1073259.1
bacterium
Skillet-2 ? Rossmann-MTase
#;direct fusion
MCC7126473.1
Acidobacteriota bacterium
Skillet-2 Rossmann-MTase
#######;T2SSG is a 'pilus' domain association
NIM06309.1
Armatimonadota bacterium
T2SSG Skillet-2
MBY0457807.1
Gemmataceae bacterium
T2SSG Skillet-2
MBV9850561.1
Armatimonadota bacterium
T2SSG Skillet-2
HBO42836.1
Planctomycetaceae bacterium
T2SSG Skillet-2
RYG69304.1
bacterium
T2SSG T2SSG Skillet-2
MBN9521845.1
bacterium
T2SSG Skillet-2
HCD31770.1
Phycisphaerales bacterium
T2SSG T2SSG Skillet-2
MBX9653495.1
bacterium
T2SSG T2SSG Skillet-2
NLT78501.1
Planctomycetota bacterium
T2SSG Skillet-2
WP_184197037.1
Armatimonas rosea
T2SSG Skillet-2 ? ? ? ? ? PALP
MBT5018319.1
Planctomicrobium sp.
T2SSG Skillet-2
MCA9015890.1
Planctomycetaceae bacterium
T2SSG Skillet-2
HBE71276.1
Planctomycetaceae bacterium
T2SSG Skillet-2
MBM4040228.1
Planctomycetota bacterium
T2SSG Skillet-2 MBL
MAE60413.1
Planctomycetaceae bacterium
T2SSG ? Skillet-2
#######;standalone in the genome
WP_185212000.1
Sphingobacterium mizutaii
Skillet-2
WP_147860565.1
Mesonia sp. HuA40
Skillet-2
MAS69343.1
Zunongwangia sp.
Skillet-2
MCB9877257.1
Planctomycetota bacterium
Skillet-2
CCY06115.1
Eggerthella sp. CAG:1427
Skillet-2
MBC7612362.1
Pseudopedobacter sp.
Skillet-2
MBU2501781.1
bacterium
Skillet-2
MBR5503519.1
Methanobrevibacter sp.
Skillet-2
MBE1481573.1
Skillet-2
RKI95697.1
bacterium D16-34
Skillet-2
WP_097125169.1
Spirosoma fluviale
Skillet-2
RYY05102.1
Alphaproteobacteria bacterium
Skillet-2
HJF66527.1
Slackia equolifaciens
Skillet-2
WP_148042531.1
Slackia equolifaciens
Skillet-2
PTY01391.1
Verrucomicrobia bacterium LW23
Skillet-2
WP_138091427.1
Sphingobacterium daejeonense
Skillet-2
WP_028593731.1
Paenibacillus assamensis DSM 18201
Skillet-2
WP_156891946.1
Adlercreutzia mucosicola DSM 19490
Skillet-2
KRF31695.1
Paenibacillus sp. Soil787
Skillet-2
NQV15868.1
bacterium
Skillet-2
MBT4130794.1
Fidelibacterota bacterium
Skillet-2
SDW04471.1
Flavobacterium degerlachei
Skillet-2
NLM45185.1
Bacillota bacterium
Skillet-2
WP_094235901.1
Tumebacillus algifaecis
Skillet-2
WP_168975177.1
Aneurinibacillus aneurinilyticus
Skillet-2
MTI95725.1
Bacillota bacterium
Skillet-2
RYX82338.1
bacterium
Skillet-2
MBI9017038.1
Phycisphaerae bacterium
Skillet-2
PZW39780.1
Mesonia algae
Skillet-2
PAW79469.1
Verrucomicrobia bacterium Tous-C9LFEB
Skillet-2
WP_187032540.1
Skillet-2
WP_197919663.1
Aurantiacibacter sediminis
Skillet-2
MBN1633489.1
Ignavibacteria bacterium
Skillet-2
NLF17243.1
Lentisphaerota bacterium
Skillet-2
OBZ10727.1
Bacillus sp. FJAT-27264
Skillet-2
RKZ12957.1
bacterium
Skillet-2
WP_181517075.1
Billgrantia kenyensis
Skillet-2
MBR0120784.1
Clostridia bacterium
Skillet-2
MBE6020228.1
Clostridiales bacterium
Skillet-2
MCE0522645.1
Candidatus Methylacidiphilales bacterium
Skillet-2
HJC20420.1
Candidatus Butyricicoccus avicola
Skillet-2
HIX53896.1
Candidatus Sphingobacterium stercoripullorum
Skillet-2
WP_163500900.1
Skillet-2
WP_184340030.1
Prosthecobacter vanneervenii
Skillet-2
NQV42651.1
Fidelibacterota bacterium
Skillet-2
MBN8422417.1
Verrucomicrobiota bacterium
Skillet-2
MBK7427215.1
Saprospiraceae bacterium
Skillet-2
WP_217065263.1
Evansella tamaricis
Skillet-2
PRX46267.1
Salegentibacter salegens
Skillet-2
MBT5705428.1
Verrucomicrobiota bacterium
Skillet-2
NBV84982.1
Verrucomicrobiota bacterium
Skillet-2
RRF88791.1
Skillet-2
MBC8192079.1
FCB group bacterium
Skillet-2
WP_116107570.1
Lewinella sp. IMCC34191
Skillet-2
MCE0483673.1
Candidatus Methylacidiphilales bacterium
Skillet-2
WP_125560791.1
Salibacterium salarium
Skillet-2
MBE2245763.1
Candidatus Competibacteraceae bacterium
Skillet-2
NLL31048.1
Clostridiales bacterium
Skillet-2
RYG56390.1
bacterium
Skillet-2
HIW20670.1
Candidatus Dorea intestinavium
Skillet-2
GDX09624.1
Verrucomicrobiota bacterium
Skillet-2
MBJ7326582.1
Chthoniobacterales bacterium
Skillet-2

MBI3868641.1	*Verrucomicrobiota bacterium*	Skillet-2
MBQ4454139.1	*Clostridia bacterium*	Skillet-2
WP_185212261.1	*Sphingobacterium mizutaii*	Skillet-2
HJC65678.1	*Candidatus Enterocloster excrementigallinarum*	Skillet-2
HCE13637.1	*Clostridiales bacterium*	Skillet-2
SEG77287.1	*Paenibacillus sp. UNC499MF*	Skillet-2
KAB1637406.1	*Ellagibacter isourolithinifaciens*	Skillet-2
HAS36533.1	*Flavobacteriales bacterium*	Skillet-2
MBI3874691.1	*Verrucomicrobiota bacterium*	Skillet-2
HIW28140.1	*Candidatus Lawsonibacter pullicola*	Skillet-2
WP_052595397.1	*Aureispira sp. CCB-E*	Skillet-2
MTI67369.1	*Bacillota bacterium*	Skillet-2
RYG57576.1	*bacterium*	Skillet-2
WP_197464893.1	*Sphingobacterium sp. CFCC 11742*	Skillet-2
WP_019976465.1	*Empedobacter brevis NBRC 14943 = ATCC 43319*	Skillet-2
HCC93990.1	*Flavobacteriaceae bacterium*	Skillet-2
NQV49860.1	*Fidelibacterota bacterium*	Skillet-2
WP_224462562.1	*Psychroflexus montanilacus*	Skillet-2
MCB0482267.1	*Flavobacteriales bacterium*	Skillet-2
MBQ7766597.1	*Lachnospiraceae bacterium*	Skillet-2
SDG61271.1	*Pedobacter terrae*	Skillet-2
WP_138931787.1	*Mesohalobacter halotolerans*	Skillet-2
KUK72728.1	*Methanobacterium sp. 42_16*	Skillet-2
NUM71708.1	*Ignavibacteriaceae bacterium*	Skillet-2
MBX7075803.1	*Methanobacteriaceae archaeon*	Skillet-2
MBS5072320.1	*Hungatella hathewayi*	Skillet-2
PKL67890.1	*Methanobacteriales archaeon HGW-Methanobacteriales-1*	Skillet-2
MBS0657176.1	*Verrucomicrobiota bacterium*	Skillet-2
NQU66845.1	*Fidelibacterota bacterium*	Skillet-2
MBX3751168.1	*Opitutaceae bacterium*	Skillet-2
MBL4651115.1	*Aureispira sp.*	Skillet-2
HAC62108.1	*Cyanothece sp. UBA12306*	Skillet-2
WP_104418971.1	*Neolewinella xylanilytica*	Skillet-2
HET88423.1		Skillet-2
WP_089317942.1	*Pontibacter ummariensis*	Skillet-2
MBU3665943.1	*Chthoniobacterales bacterium*	Skillet-2
MCB0738549.1	*Bacteroidota bacterium*	Skillet-2
NUQ26157.1	*Saprospiraceae bacterium*	Skillet-2
KAA3611375.1	*Calditrichota bacterium*	Skillet-2
MBP3494488.1	*Oscillospiraceae bacterium*	Skillet-2
MBQ6900024.1	*Bacillota bacterium*	Skillet-2
MBA2582819.1	*Bacteroidota bacterium*	Skillet-2
KAA3648924.1	*Bacteroidota bacterium*	Skillet-2
NBH51637.1		Skillet-2
APY09643.1	*Winogradskyella sp. J14-2*	Skillet-2
MBO6211993.1	*Algoriella sp.*	Skillet-2
WP_201919443.1	*Marivirga atlantica*	Skillet-2
MCB0278896.1	*Calditrichota bacterium*	Skillet-2
MBI3886758.1	*Opitutia bacterium*	Skillet-2
WP_217789496.1	*Pinibacter aurantiacus*	Skillet-2
MCB0707117.1	*Saprospiraceae bacterium*	Skillet-2
OAB43486.1	*Paenibacillus glacialis*	Skillet-2
MCA9139653.1	*Planctomycetales bacterium*	Skillet-2
ARU63663.1	*Tumebacillus avium*	Skillet-2
WP_132644051.1	*Roseivirga ehrenbergii*	Skillet-2
WP_113988465.1	*Flavobacterium tibetense*	Skillet-2
WP_139212038.1	*Neolewinella agarilytica*	Skillet-2
MBR9833411.1	*bacterium*	Skillet-2
REC42008.1	*Chryseobacterium sp. 5_R23647*	Skillet-2
WP_134271870.1	*Lewinella sp. W8*	Skillet-2
MBD3375473.1	*Candidatus Zhuqueibacterota bacterium*	Skillet-2
KAA3621618.1	*Bacteroidota bacterium*	Skillet-2
MBI1229508.1	*Cytophagales bacterium*	Skillet-2
PHI20845.1	*Lewinellaceae bacterium SD302*	Skillet-2
WP_116668885.1	*Methanobrevibacter woesei*	Skillet-2
KAA3655944.1	*Calditrichota bacterium*	Skillet-2
NCQ35500.1	*bacterium*	Skillet-2
MBP3901519.1	*Blautia sp.*	Skillet-2
RHP88364.1	*Roseburia sp. AM59-24XD*	Skillet-2
WP_137760545.1	*Pontibacter sp. SGAir0037*	Skillet-2
PID79506.1	*bacterium DOLZORAL124_64_63*	Skillet-2
WP_029143286.1	*Methanobrevibacter smithii TS94C*	Skillet-2
MBK7406795.1	*Saprospirales bacterium*	Skillet-2
MBS1271802.1	*Fidelibacterota bacterium*	Skillet-2
PAY99812.1	*Spartobacteria bacterium AMD-G4*	Skillet-2
NBR48242.1	*bacterium*	Skillet-2
WP_157540376.1	*Mucilaginibacter aquatilis*	Skillet-2
NQV36777.1	*Fidelibacterota bacterium*	Skillet-2
MCB0635111.1	*Lewinella sp.*	Skillet-2
MAB84784.1	*Cryomorphaceae bacterium*	Skillet-2
WP_209037988.1	*Neolewinella antarctica*	Skillet-2
MBR9921539.1	*Bacteroidota bacterium*	Skillet-2
WP_189626183.1	*Sphingobacterium griseoflavum*	Skillet-2
HAG06071.1	*Cryomorphaceae bacterium*	Skillet-2
MBT5538047.1	*Fidelibacterota bacterium*	Skillet-2
WP_162330072.1	*Chitinophaga agri*	Skillet-2
WP_184469979.1	*Pedobacter sp. AK013*	Skillet-2
RYD99009.1	*Sphingobacteriales bacterium*	Skillet-2
WP_157472255.1	*Neolewinella persica DSM 23188*	Skillet-2
WP_226515183.1	*Bacillus shivajii*	Skillet-2
MCB0648912.1	*Saprospiraceae bacterium*	Skillet-2
MCB0654097.1	*Saprospiraceae bacterium*	Skillet-2
HAD96795.1	*Cryomorphaceae bacterium*	Skillet-2

Accession	Organism	Domain architecture
WP_092370881.1	*Enterocloster lavalensis*	Skillet-2
MBS1527765.1	*Bacteroidota bacterium*	Skillet-2
SKB57705.1	*Parapedobacter luteus*	Skillet-2
WP_183494257.1	*Neolewinella aquimaris*	Skillet-2
RXG21107.1	*Leeuwenhoekiella aequorea*	Skillet-2
MBT3316519.1	*bacterium*	Skillet-2
NOZ74892.1	*FCB group bacterium*	Skillet-2
MCB1185233.1	*bacterium*	Skillet-2
NBI31583.1	*Enterorhabdus sp. P55*	Skillet-2
MBX7157304.1	*Verrucomicrobiia bacterium*	Skillet-2
WP_147407710.1	*Roseivirga pacifica*	Skillet-2
MBP3330820.1	*Clostridia bacterium*	Skillet-2
MBA3315345.1	*Planctomycetaceae bacterium*	Skillet-2
WP_146399311.1	*Pseudobythopirellula maris*	Skillet-2
MCB7512475.1	*bacterium 210917-SL.2.15*	ZnR, ??, Skillet-2
PIZ70383.1	*Fidelibacterota bacterium CG_4_10_14_0_2_um_filter_48_9*	Skillet-2, DCC1-l-thiol-disulfide-oxidoreductase
WP_154932088.1	*Gimesia maris*	Skillet-2, IG-like
EDL57035.1	*Gimesia maris DSM 8797*	Skillet-2
GGE24283.1	*Psychroflexus planctonicus*	Skillet-2
MCA1752502.1	*Flavobacteriales bacterium*	Skillet-2
RGC73295.1	*Lachnospiraceae bacterium AM23-2LB*	Skillet-2
MBQ3560930.1	*Oscillospiraceae bacterium*	Skillet-2
MBI3192139.1	*Pedosphaera parvula*	Skillet-2

################;Skillet-3 associations; #######;diversity of associations indicating potential 1-component response systems. These proteins possibly respond to detection of some conflict-originating signal in the extracellular space, given the recombinatorial 'swapping' observed in these direct fusions.; ####;association with two distinct beta-sandwiches

Accession	Organism	Domain architecture
MCB9637172.1	*Sandaracinus sp.*	Skillet-3, T6SS-SciN-like-beta-sandwich, extended-beta-sandwich
MBU1750711.1	*Chloroflexota bacterium*	Skillet-3, T6SS-SciN-like-beta-sandwich, extended-beta-sandwich
MAT24021.1	*Sandaracinus sp.*	Skillet-3, T6SS-SciN-like-beta-sandwich, extended-beta-sandwich
MCA9636769.1	*Myxococcales bacterium*	Skillet-3, T6SS-SciN-like-beta-sandwich, extended-beta-sandwich
NUQ79672.1	*Polyangiaceae bacterium*	Skillet-3, T6SS-SciN-like-beta-sandwich, extended-beta-sandwich
MBK8264578.1	*Nannocystis sp.*	Skillet-3, T6SS-SciN-like-beta-sandwich, extended-beta-sandwich
MCA9658252.1	*Myxococcales bacterium*	Skillet-3, T6SS-SciN-like-beta-sandwich, extended-beta-sandwich
HGG58092.1	*Nannocystis exedens*	Skillet-3, T6SS-SciN-like-beta-sandwich, extended-beta-sandwich
HHH11712.1	*Sorangium sp.*	Skillet-3, T6SS-SciN-like-beta-sandwich, extended-beta-sandwich
PJB46667.1	*Deltaproteobacteria bacterium CG_4_9_14_3_um_filter_63_12*	Skillet-3, T6SS-SciN-like-beta-sandwich, extended-beta-sandwich
MBC8067950.1	*Nannocystaceae bacterium*	Skillet-3, T6SS-SciN-like-beta-sandwich, extended-beta-sandwich
MCA9687561.1	*Myxococcales bacterium*	Skillet-3, T6SS-SciN-like-beta-sandwich, extended-beta-sandwich
MBA2661648.1	*Lujinxingiaceae bacterium*	Skillet-3, T6SS-SciN-like-beta-sandwich, extended-beta-sandwich
MBL8610074.1	*Myxococcales bacterium*	Skillet-3, T6SS-SciN-like-beta-sandwich, extended-beta-sandwich
MCB9594217.1	*Sandaracinaceae bacterium*	Skillet-3, T6SS-SciN-like-beta-sandwich, extended-beta-sandwich
MCC7537213.1	*Deltaproteobacteria bacterium*	Skillet-3, T6SS-SciN-like-beta-sandwich, extended-beta-sandwich
AKU99067.1	*Labilithrix luteola*	Skillet-3, T6SS-SciN-like-beta-sandwich, extended-beta-sandwich
MCA9706924.1	*Myxococcales bacterium*	Skillet-3, T6SS-SciN-like-beta-sandwich, extended-beta-sandwich
MBQ9818337.1	*Pseudomonadota bacterium*	Skillet-3, T6SS-SciN-like-beta-sandwich, extended-beta-sandwich
MBX3193116.1	*Labilithrix sp.*	Skillet-3, T6SS-SciN-like-beta-sandwich
PZN20666.1		Skillet-3, T6SS-SciN-like-beta-sandwich

###;further association with membrane-anchored vWA domain inserved itself into a beta-sandwhich

Accession	Organism	Domain architecture
MBX3251404.1	*Myxococcales bacterium*	Skillet-3, T6SS-SciN-like-beta-sandwich, extended-beta-sandwich, beta-sandwich, vWA-insert, 3helix
WP_176396631.1	*Pseudenhygromyxa sp. WMMC2535*	Skillet-3, T6SS-SciN-like-beta-sandwich, extended-beta-sandwich, beta-sandwich, vWA-insert, 3helix
MBK7829855.1	*Nannocystis sp.*	Skillet-3, T6SS-SciN-like-beta-sandwich, extended-beta-sandwich, beta-sandwich, vWA-insert, 3helix
MBA3545477.1	*Nannocystis sp.*	Skillet-3, T6SS-SciN-like-beta-sandwich, extended-beta-sandwich, beta-sandwich, vWA-insert, 3helix
WP_050434967.1	*Chondromyces crocatus*	Skillet-3, T6SS-SciN-like-beta-sandwich, extended-beta-sandwich, beta-sandwich, vWA-insert, 3helix
MCB9753143.1	*Myxococcales bacterium*	Skillet-3, T6SS-SciN-like-beta-sandwich, extended-beta-sandwich, beta-sandwich, vWA-insert, 3helix
MBL4686393.1	*Nannocystaceae bacterium*	Skillet-3, T6SS-SciN-like-beta-sandwich, extended-beta-sandwich, beta-sandwich, vWA-insert, 3helix
MBL8971143.1	*Myxococcales bacterium*	Skillet-3, T6SS-SciN-like-beta-sandwich, extended-beta-sandwich, beta-sandwich, vWA-insert, 3helix
MCA9707220.1	*Myxococcales bacterium*	Skillet-3, T6SS-SciN-like-beta-sandwich, extended-beta-sandwich, beta-sandwich, vWA-insert, 3helix
MCA9650035.1	*Myxococcales bacterium*	Skillet-3, T6SS-SciN-like-beta-sandwich, extended-beta-sandwich, beta-sandwich, vWA-insert, 3helix
RMG93710.1	*Deltaproteobacteria bacterium*	Skillet-3, T6SS-SciN-like-beta-sandwich, extended-beta-sandwich, beta-sandwich, vWA-insert, 3helix
MCA9655129.1	*Myxococcales bacterium*	Skillet-3, T6SS-SciN-like-beta-sandwich, extended-beta-sandwich, beta-sandwich, vWA-insert, 3helix
WP_224193894.1	*Nannocystis pusilla*	Skillet-3, T6SS-SciN-like-beta-sandwich, extended-beta-sandwich, beta-sandwich, vWA-insert, 3helix
MBK6922903.1	*Deltaproteobacteria bacterium*	Skillet-3, T6SS-SciN-like-beta-sandwich, extended-beta-sandwich, beta-sandwich, vWA-insert, 3helix
MBH23090.1	*Myxococcales bacterium*	Skillet-3, T6SS-SciN-like-beta-sandwich, extended-beta-sandwich, beta-sandwich, vWA-insert, 3helix
MSQ82863.1	*Myxococcales bacterium*	Skillet-3, T6SS-SciN-like-beta-sandwich, extended-beta-sandwich, beta-sandwich, vWA-insert, 3helix
WP_199693995.1	*Sorangium cellulosum*	Skillet-3, T6SS-SciN-like-beta-sandwich, extended-beta-sandwich, beta-sandwich, vWA-insert, 3helix
WP_052375628.1	*Chondromyces apiculatus DSM 436*	Skillet-3, T6SS-SciN-like-beta-sandwich, extended-beta-sandwich, beta-sandwich, vWA-insert, 3helix
MBI4956548.1	*Myxococcales bacterium*	Skillet-3, T6SS-SciN-like-beta-sandwich, extended-beta-sandwich, beta-sandwich, vWA-insert, 3helix
WP_206079567.1	*Polyangium spumosum*	Skillet-3, T6SS-SciN-like-beta-sandwich, extended-beta-sandwich, beta-sandwich, vWA-insert, 3helix
WP_211365581.1	*Polyangium fumosum*	Skillet-3, T6SS-SciN-like-beta-sandwich, extended-beta-sandwich, beta-sandwich, vWA-insert, 3helix
MBL8945853.1	*Myxococcales bacterium*	Skillet-3, T6SS-SciN-like-beta-sandwich, extended-beta-sandwich, beta-sandwich, vWA-insert, 3helix
WP_199789567.1	*Sorangium cellulosum*	Skillet-3, T6SS-SciN-like-beta-sandwich, extended-beta-sandwich, beta-sandwich, vWA-insert, 3helix
MCA9689186.1	*Myxococcales bacterium*	Skillet-3, T6SS-SciN-like-beta-sandwich, extended-beta-sandwich, beta-sandwich, vWA-insert, 3helix
MBL4684908.1	*Nannocystaceae bacterium*	Skillet-3, T6SS-SciN-like-beta-sandwich, extended-beta-sandwich, beta-sandwich, vWA-insert, 3helix
NUN12184.1	*Myxococcales bacterium*	Skillet-3, T6SS-SciN-like-beta-sandwich, extended-beta-sandwich, beta-sandwich, vWA-insert, 3helix
MBK8264465.1	*Nannocystis sp.*	Skillet-3, T6SS-SciN-like-beta-sandwich, extended-beta-sandwich, beta-sandwich, vWA-insert, 3helix
MCB9523897.1	*Myxococcales bacterium*	Skillet-3, T6SS-SciN-like-beta-sandwich, extended-beta-sandwich, beta-sandwich, vWA-insert, 3helix
MBX2798886.1	*Myxococcales bacterium*	Skillet-3, T6SS-SciN-like-beta-sandwich, extended-beta-sandwich, beta-sandwich, vWA-insert, 3helix
MCB9548551.1	*Myxococcales bacterium*	Skillet-3, T6SS-SciN-like-beta-sandwich, extended-beta-sandwich, beta-sandwich, vWA-insert, 3helix
MBO6939888.1	*Deltaproteobacteria bacterium*	Skillet-3, T6SS-SciN-like-beta-sandwich, extended-beta-sandwich, beta-sandwich, vWA-insert, 3helix
MBL9006494.1	*Myxococcales bacterium*	Skillet-3, T6SS-SciN-like-beta-sandwich, extended-beta-sandwich, beta-sandwich, vWA-insert, 3helix
MBT9556384.1	*Myxococcales bacterium*	Skillet-3, T6SS-SciN-like-beta-sandwich, extended-beta-sandwich, beta-sandwich, vWA-insert, 3helix
MBU1431645.1	*Myxococcota bacterium*	Skillet-3, T6SS-SciN-like-beta-sandwich, extended-beta-sandwich, beta-sandwich, vWA-insert, 3helix
MBX7191092.1	*Sandaracinaceae bacterium*	Skillet-3, T6SS-SciN-like-beta-sandwich, extended-beta-sandwich, beta-sandwich, vWA-insert, 3helix
RZO64849.1	*Sandaracinaceae bacterium*	Skillet-3, T6SS-SciN-like-beta-sandwich, extended-beta-sandwich, beta-sandwich, vWA-insert, 3helix
MBX3270787.1	*Sandaracinaceae bacterium*	Skillet-3, T6SS-SciN-like-beta-sandwich, extended-beta-sandwich, beta-sandwich, vWA-insert, 3helix
MCB9595418.1	*Sandaracinaceae bacterium*	Skillet-3, T6SS-SciN-like-beta-sandwich, extended-beta-sandwich, beta-sandwich, vWA-insert, 3helix
MBK8254449.1	*Polyangiaceae bacterium*	Skillet-3, T6SS-SciN-like-beta-sandwich, extended-beta-sandwich, beta-sandwich, vWA-insert, 3helix
WP_199693870.1	*Sorangium cellulosum*	Skillet-3, T6SS-SciN-like-beta-sandwich, extended-beta-sandwich, beta-sandwich, vWA-insert, 3helix

#;just the vWA association

Accession	Organism	Domain architecture
MBH23268.1	*Myxococcales bacterium*	Skillet-3, beta-sandwich, vWA-insert, 3helix
MBI1945973.1	*Deltaproteobacteria bacterium*	Skillet-3, ?, ?, 3helix, vWA-insert, beta-sandwich

####;BetaPropeller associations; #;3-bladed b-propeller associations are often found in 2-3 copies on the genome

Accession	Organism	Domain architecture
GII04781.1	*Planobispora takensis*	Skillet-3, Bps, TM-region, Skillet-3, Bps, TM-region
WP_184958997.1	*Nonomuraea endophytica*	Skillet-3, Bps, TM-region, Skillet-3, Bps, TM-region
WP_173528063.1	*Nonomuraea antri*	Skillet-3, Bps, TM-region, Skillet-3, Bps, TM-region

WP_204066002.1
Planobispora siamensis

WP_188125530.1
Actinoplanes lobatus

WP_181019641.1
Nonomuraea typhae

WP_156045535.1
Herbidospora cretacea

WP_061290881.1
Herbidospora cretacea

WP_161480790.1
Herbidospora solisilvae

WP_062336485.1
Herbidospora sakaeratensis

WP_093621772.1
Actinoplanes philippinensis

WP_062356462.1
Herbidospora yilanensis

WP_066366734.1
Herbidospora mongoliensis

WP_198169090.1
Herbidospora daliensis

WP_203737131.1
Actinoplanes italicus

WP_146008400.1
Chromobacterium subtsugae

MBZ0284694.1
Anaerolineae bacterium

WP_185037845.1
Actinoplanes octamycinicus

GIE83025.1
Actinoplanes philippinensis

PKQ25123.1
Actinobacteria bacterium HGW-Actinobacteria-4

WP_226094707.1
Demequina sp. TTPB684

MBN2007583.1
Anaerolineae bacterium

#;Skillet-3+BetaPropeller+TM-region

MAS34693.1
Anaerolineaceae bacterium

HGW79031.1

MBN1247649.1
Anaerolineae bacterium

MBO9362869.1
Thermoflexus sp.

HDV36356.1

MBN2302947.1
Anaerolineae bacterium

MBL8046484.1
Anaerolineales bacterium

MBI3737974.1
Chloroflexota bacterium

MBI5033144.1
Chloroflexota bacterium

MBF8257235.1
Anaerolineales bacterium

MBI5652716.1
Chloroflexota bacterium

OGO18880.1
Chloroflexi bacterium RBG_16_48_8

KAA3647090.1
Chloroflexota bacterium

OQY83413.1
Chloroflexi bacterium UTCFX4

MCC7361577.1
Anaerolineales bacterium

MBI4310596.1
Chloroflexota bacterium

MBL8118409.1
Anaerolineae bacterium

MBN1966549.1
Anaerolineae bacterium

NUM45247.1
Anaerolineales bacterium

NPA72101.1
Gammaproteobacteria bacterium

NMB68823.1
Chloroflexota bacterium

MBN1120012.1
Anaerolineae bacterium

PZC49205.1
Chloroflexota bacterium

HGV42010.1

MCC6905721.1
Anaerolineae bacterium

MBN1200514.1
Anaerolineae bacterium

MCB0018341.1
Anaerolineales bacterium

HGO80939.1

MBN1121247.1
Anaerolineae bacterium

OQY30717.1
Anaerolineaceae bacterium 4572_5.2

MBI3152180.1
Chloroflexota bacterium

OQX61058.1
Anaerolinea sp. 4484_236

NLG96553.1
Chloroflexota bacterium

HFI29751.1

NLE45145.1
Chloroflexota bacterium

MBL8090790.1
Anaerolineales bacterium

HEY43292.1
Anaerolineae bacterium

RIK51005.1
Chloroflexota bacterium

HEY45603.1
Anaerolineae bacterium

MBC7878873.1
Anaerolineales bacterium

HFK84425.1

MBE0698512.1
Anaerolineaceae bacterium

OQZ00107.1
Chloroflexi bacterium UTCFX4

MBI5932014.1
Chloroflexota bacterium

MCC6501137.1
Anaerolineales bacterium

MBI5932272.1
Chloroflexota bacterium

MCD6284360.1
Anaerolineae bacterium

MBP7228206.1
Longilinea sp.

MBI5933707.1
Chloroflexota bacterium

OGO70489.1
Chloroflexi bacterium RBG_19FT_COMBO_62_14

MBK9601527.1
Candidatus Villigracilis affinis

MBL8045802.1
Anaerolineales bacterium

HDV36579.1

MBM3933592.1
SAR202 cluster bacterium

UCF61634.1
Anaerolineaceae bacterium

NLD76727.1
Acidimicrobiales bacterium

NLD77901.1
Acidimicrobiales bacterium

MBP7630984.1

MCB0961787.1
Acidimicrobiales bacterium

MBK5221569.1
Acidimicrobiia bacterium

MBX3314528.1
Actinomycetota bacterium

MCA0297187.1
Actinomycetota bacterium

NNE12877.1
Ilumatobacter sp.

MBN9106592.1
Propionibacteriaceae bacterium

MCB0912419.1
Propionibacteriaceae bacterium

TQL32049.1
Barrientosiimonas humi

AKU17942.1
Luteipulveratus mongoliensis

GJM37224.1
Acidimicrobiales bacterium

MBI9051699.1
Anaerolineaceae bacterium

MBL8092957.1
Anaerolineales bacterium

MBN1371706.1
Anaerolineaceae bacterium

MCA1701846.1
Actinomycetota bacterium

MCC7162695.1
Anaerolineae bacterium

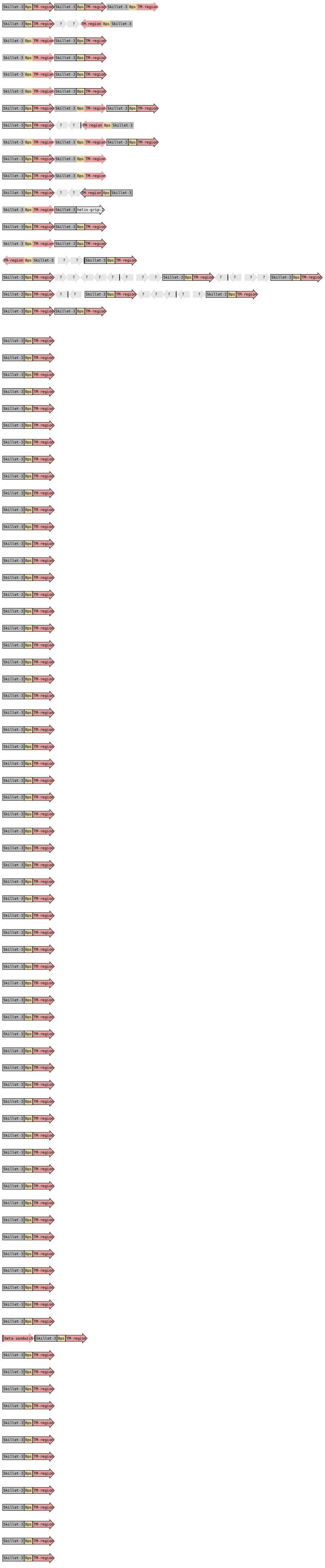

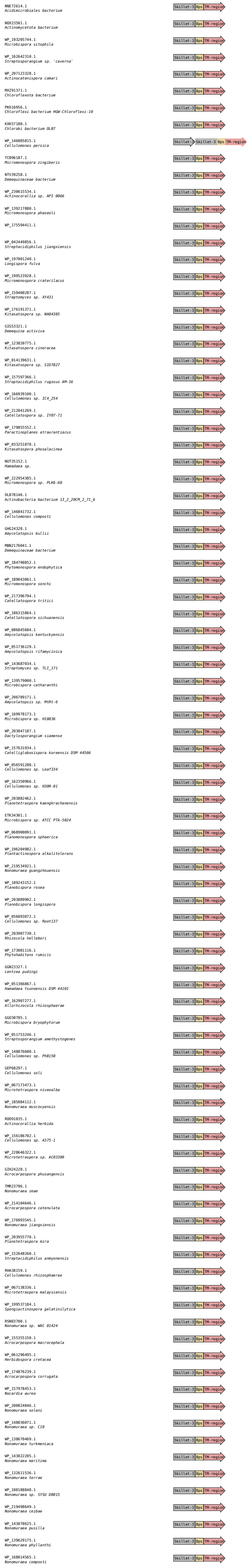
NNE72614.1
Acidimicrobiales bacterium
Skillet-3 Bps TM-region
NOX23501.1
Actinomycetota bacterium
Skillet-3 Bps TM-region
WP_193205744.1
Microbispora sitophila
Skillet-3 Bps TM-region
WP_162642310.1
Streptosporangium sp. 'caverna'
Skillet-3 Bps TM-region
WP_207123328.1
Actinocatenispora comari
Skillet-3 Bps TM-region
MXZ91371.1
Chloroflexota bacterium
Skillet-3 Bps TM-region
PKO16956.1
Chloroflexi bacterium HGW-Chloroflexi-10
Skillet-3 Bps TM-region
KXK57180.1
Chlorobi bacterium OLB7
Skillet-3 Bps TM-region
WP_146805815.1
Cellulomonas persica
Skillet-3 Skillet-3 Bps TM-region
TCB96187.1
Micromonospora zingiberis
Skillet-3 Bps TM-region
NTV39258.1
Demequinaceae bacterium
Skillet-3 Bps TM-region
WP_230615534.1
Actinocorallia sp. API 0066
Skillet-3 Bps TM-region
WP_139217880.1
Micromonospora phaseoli
Skillet-3 Bps TM-region
WP_175594411.1
Skillet-3 Bps TM-region
WP_042449856.1
Streptacidiphilus jiangxiensis
Skillet-3 Bps TM-region
WP_197001240.1
Longispora fulva
Skillet-3 Bps TM-region
WP_199523928.1
Micromonospora craterilacus
Skillet-3 Bps TM-region
WP_159400287.1
Streptomyces sp. XY431
Skillet-3 Bps TM-region
WP_176191371.1
Kitasatospora sp. NA04385
Skillet-3 Bps TM-region
GIG53321.1
Demequina activiva
Skillet-3 Bps TM-region
WP_123820775.1
Kitasatospora cineracea
Skillet-3 Bps TM-region
WP_014139631.1
Kitasatospora sp. SID7827
Skillet-3 Bps TM-region
WP_157597366.1
Streptacidiphilus rugosus AM-16
Skillet-3 Bps TM-region
WP_166939100.1
Cellulomonas sp. IC4_254
Skillet-3 Bps TM-region
WP_212841269.1
Catellatospora sp. IY07-71
Skillet-3 Bps TM-region
WP_179855552.1
Paractinoplanes atraurantiacus
Skillet-3 Bps TM-region
WP_033251878.1
Kitasatospora phosalacinea
Skillet-3 Bps TM-region
NUT35152.1
Hamadaea sp.
Skillet-3 Bps TM-region
WP_222954385.1
Micromonospora sp. PLK6-60
Skillet-3 Bps TM-region
OLB78146.1
Actinobacteria bacterium 13_2_20CM_2_71_6
Skillet-3 Bps TM-region
WP_146841732.1
Cellulomonas composti
Skillet-3 Bps TM-region
GHG24328.1
Amycolatopsis bullii
Skillet-3 Bps TM-region
MBN2176041.1
Demequinaceae bacterium
Skillet-3 Bps TM-region
WP_184790852.1
Phytomonospora endophytica
Skillet-3 Bps TM-region
WP_189043061.1
Micromonospora sonchi
Skillet-3 Bps TM-region
WP_217396794.1
Catellatospora tritici
Skillet-3 Bps TM-region
WP_186315864.1
Catellatospora sichuanensis
Skillet-3 Bps TM-region
WP_086845604.1
Amycolatopsis kentuckyensis
Skillet-3 Bps TM-region
WP_051736129.1
Amycolatopsis rifamycinica
Skillet-3 Bps TM-region
WP_143687034.1
Streptomyces sp. TLI_171
Skillet-3 Bps TM-region
WP_139579000.1
Microbispora catharanthi
Skillet-3 Bps TM-region
WP_206789171.1
Amycolatopsis sp. MtRt-6
Skillet-3 Bps TM-region
WP_169978173.1
Microbispora sp. H10836
Skillet-3 Bps TM-region
WP_203847187.1
Dactylosporangium siamense
Skillet-3 Bps TM-region
WP_157631934.1
Catelliglobosispora koreensis DSM 44566
Skillet-3 Bps TM-region
WP_056591288.1
Cellulomonas sp. Leaf334
Skillet-3 Bps TM-region
WP_162350966.1
Cellulomonas sp. H30R-01
Skillet-3 Bps TM-region
WP_203882462.1
Planotetraspora kaengkrachanensis
Skillet-3 Bps TM-region
ETK34301.1
Microbispora sp. ATCC PTA-5024
Skillet-3 Bps TM-region
WP_068900091.1
Planomonospora sphaerica
Skillet-3 Bps TM-region
WP_196204982.1
Plantactinospora alkalitolerans
Skillet-3 Bps TM-region
WP_219534921.1
Nonomuraea guangzhouensis
Skillet-3 Bps TM-region
WP_189243152.1
Planobispora rosea
Skillet-3 Bps TM-region
WP_203889902.1
Planobispora longispora
Skillet-3 Bps TM-region
WP_056093072.1
Cellulomonas sp. Root137
Skillet-3 Bps TM-region
WP_203907730.1
Rhizocola hellebori
Skillet-3 Bps TM-region
WP_173081116.1
Phytohabitans rumicis
Skillet-3 Bps TM-region
GGN23327.1
Lentzea pudingi
Skillet-3 Bps TM-region
WP_051366867.1
Hamadaea tsunoensis DSM 44101
Skillet-3 Bps TM-region
WP_162907277.1
Allorhizocola rhizosphaerae
Skillet-3 Bps TM-region
GGO30785.1
Microbispora bryophytorum
Skillet-3 Bps TM-region
WP_051753246.1
Streptosporangium amethystogenes
Skillet-3 Bps TM-region
WP_148076600.1
Cellulomonas sp. PhB150
Skillet-3 Bps TM-region
GEP68297.1
Cellulomonas soli
Skillet-3 Bps TM-region
WP_067173473.1
Microtetraspora niveoalba
Skillet-3 Bps TM-region
WP_185084112.1
Nonomuraea muscovyensis
Skillet-3 Bps TM-region
ROO91035.1
Actinocorallia herbida
Skillet-3 Bps TM-region
WP_156186702.1
Cellulomonas sp. A375-1
Skillet-3 Bps TM-region
WP_228646322.1
Microtetraspora sp. AC03309
Skillet-3 Bps TM-region
GIH24228.1
Acrocarpospora phusangensis
Skillet-3 Bps TM-region
TMR23796.1
Nonomuraea zeae
Skillet-3 Bps TM-region
WP_214104646.1
Acrocarpospora catenulata
Skillet-3 Bps TM-region
WP_176993545.1
Nonomuraea jiangxiensis
Skillet-3 Bps TM-region
WP_203955770.1
Planotetraspora mira
Skillet-3 Bps TM-region
WP_152648260.1
Streptacidiphilus anmyonensis
Skillet-3 Bps TM-region
RHA38159.1
Cellulomonas rhizosphaerae
Skillet-3 Bps TM-region
WP_067138336.1
Microtetraspora malaysiensis
Skillet-3 Bps TM-region
WP_199537184.1
Spongiactinospora gelatinilytica
Skillet-3 Bps TM-region
RSN03700.1
Nonomuraea sp. WAC 01424
Skillet-3 Bps TM-region
WP_155355150.1
Acrocarpospora macrocephala
Skillet-3 Bps TM-region
WP_061296495.1
Herbidospora cretacea
Skillet-3 Bps TM-region
WP_174876239.1
Acrocarpospora corrugata
Skillet-3 Bps TM-region
WP_157978453.1
Nocardia aurea
Skillet-3 Bps TM-region
WP_200824046.1
Nonomuraea solani
Skillet-3 Bps TM-region
WP_148036071.1
Nonomuraea sp. C10
Skillet-3 Bps TM-region
WP_138670469.1
Nonomuraea turkmeniaca
Skillet-3 Bps TM-region
WP_143022205.1
Nonomuraea maritima
Skillet-3 Bps TM-region
WP_132611536.1
Nonomuraea terrae
Skillet-3 Bps TM-region
WP_188188848.1
Nonomuraea sp. SYSU D8015
Skillet-3 Bps TM-region
WP_219498649.1
Nonomuraea ceibae
Skillet-3 Bps TM-region
WP_143078625.1
Nonomuraea pusilla
Skillet-3 Bps TM-region
WP_139629175.1
Nonomuraea phyllanthi
Skillet-3 Bps TM-region
WP_168014565.1
Nonomuraea composti
Skillet-3 Bps TM-region

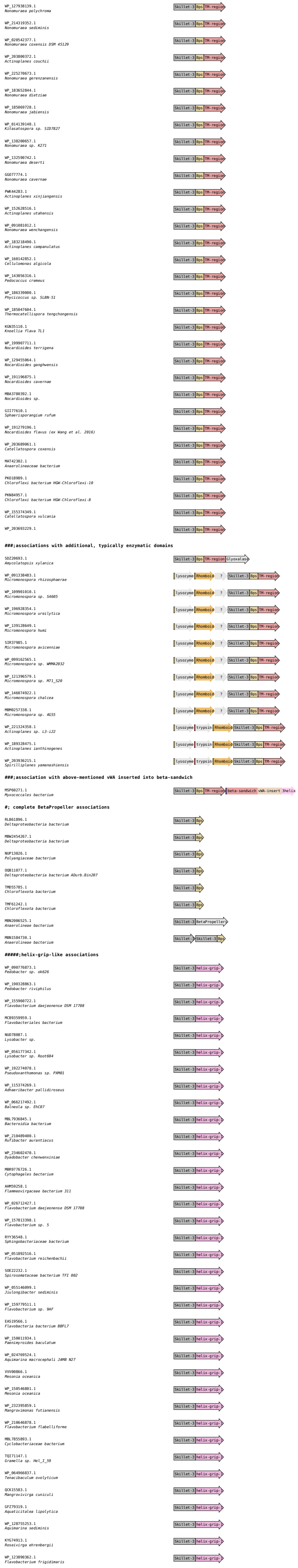

WP_127938139.1
Nonomuraea polychroma
Skillet-3 Bps TM-region
WP_214319352.1
Nonomuraea sediminis
Skillet-3 Bps TM-region
WP_020542377.1
Nonomuraea coxensis DSM 45129
Skillet-3 Bps TM-region
WP_203800372.1
Actinoplanes couchii
Skillet-3 Bps TM-region
WP_225270673.1
Nonomuraea gerenzanensis
Skillet-3 Bps TM-region
WP_183652844.1
Nonomuraea dietziae
Skillet-3 Bps TM-region
WP_185069728.1
Nonomuraea jabiensis
Skillet-3 Bps TM-region
WP_014139148.1
Kitasatospora sp. SID7827
Skillet-3 Bps TM-region
WP_138200657.1
Nonomuraea sp. K271
Skillet-3 Bps TM-region
WP_132590742.1
Nonomuraea deserti
Skillet-3 Bps TM-region
GGO77774.1
Nonomuraea cavernae
Skillet-3 Bps TM-region
PWK44283.1
Actinoplanes xinjiangensis
Skillet-3 Bps TM-region
WP_152628516.1
Actinoplanes utahensis
Skillet-3 Bps TM-region
WP_091081012.1
Nonomuraea wenchangensis
Skillet-3 Bps TM-region
WP_183218490.1
Actinoplanes campanulatus
Skillet-3 Bps TM-region
WP_160142852.1
Cellulomonas algicola
Skillet-3 Bps TM-region
WP_143056316.1
Pedococcus cremeus
Skillet-3 Bps TM-region
WP_186339000.1
Phycicoccus sp. SLBN-51
Skillet-3 Bps TM-region
WP_185047604.1
Thermocatellispora tengchongensis
Skillet-3 Bps TM-region
KGN35110.1
Knoellia flava TL1
Skillet-3 Bps TM-region
WP_199907711.1
Nocardioides terrigena
Skillet-3 Bps TM-region
WP_129455064.1
Nocardioides ganghwensis
Skillet-3 Bps TM-region
WP_191196875.1
Nocardioides cavernae
Skillet-3 Bps TM-region
MBA3780392.1
Nocardioides sp.
Skillet-3 Bps TM-region
GII77610.1
Sphaerisporangium rufum
Skillet-3 Bps TM-region
WP_191279196.1
Nocardioides flavus (ex Wang et al. 2016)
Skillet-3 Bps TM-region
WP_203689061.1
Catellatospora coxensis
Skillet-3 Bps TM-region
MAT42302.1
Anaerolineaceae bacterium
Skillet-3 Bps TM-region
PKO18989.1
Chloroflexi bacterium HGW-Chloroflexi-10
Skillet-3 Bps TM-region
PKN84957.1
Chloroflexi bacterium HGW-Chloroflexi-8
Skillet-3 Bps TM-region
WP_155374349.1
Catellatospora vulcania
Skillet-3 Bps TM-region
WP_203693229.1
Skillet-3 Bps TM-region
###;associations with additional, typically enzymatic domains
SDZ20693.1
Amycolatopsis xylanica
Skillet-3 Bps TM-region Glyoxalase
WP_091338483.1
Micromonospora rhizosphaerae
lysozyme Rhomboid ? Skillet-3 Bps TM-region
WP_109901010.1
Micromonospora sp. S4605
lysozyme Rhomboid ? Skillet-3 Bps TM-region
WP_196928354.1
Micromonospora ureilytica
lysozyme Rhomboid ? Skillet-3 Bps TM-region
WP_139128649.1
Micromonospora humi
lysozyme Rhomboid ? Skillet-3 Bps TM-region
SIR37985.1
Micromonospora avicenniae
lysozyme Rhomboid ? Skillet-3 Bps TM-region
WP_099162565.1
Micromonospora sp. WMMA2032
lysozyme Rhomboid ? Skillet-3 Bps TM-region
WP_121396579.1
Micromonospora sp. M71_S20
lysozyme Rhomboid ? Skillet-3 Bps TM-region
WP_146074922.1
Micromonospora chalcea
lysozyme Rhomboid ? Skillet-3 Bps TM-region
MBM0257338.1
Micromonospora sp. 4G55
lysozyme Rhomboid ? Skillet-3 Bps TM-region
WP_221324358.1
Actinoplanes sp. L3-i22
lysozyme trypsin Rhomboid Skillet-3 Bps TM-region
WP_189328475.1
Actinoplanes ianthinogenes
lysozyme trypsin Rhomboid Skillet-3 Bps TM-region
WP_203936215.1
Spirilliplanes yamanashiensis
lysozyme trypsin Rhomboid Skillet-3 Bps TM-region
###;association with above-mentioned vWA inserted into beta-sandwich
MSP60271.1
Myxococcales bacterium
Skillet-3 Bps TM-region beta-sandwich vWA-insert 3helix
#; complete BetaPropeller associations
RLB61896.1
Deltaproteobacteria bacterium
Skillet-3 Bps
MBW2454267.1
Deltaproteobacteria bacterium
Skillet-3 Bps
NUP13026.1
Polyangiaceae bacterium
Skillet-3 Bps
OQB11077.1
Deltaproteobacteria bacterium ADurb.Bin207
Skillet-3 Bps
TMD55785.1
Chloroflexota bacterium
Skillet-3 Bps
TMF61242.1
Chloroflexota bacterium
Skillet-3 Bps
MBN2006525.1
Anaerolineae bacterium
Skillet-3 BetaPropellers
MBN1584730.1
Anaerolineae bacterium
Skillet-3 Skillet-3 Bps
#####;helix-grip-like associations
WP_090776873.1
Pedobacter sp. ok626
Skillet-3 helix-grip-1
WP_190328863.1
Pedobacter riviphilus
Skillet-3 helix-grip-1
WP_155960722.1
Flavobacterium daejeonense DSM 17708
Skillet-3 helix-grip-1
MCB9359959.1
Flavobacteriales bacterium
Skillet-3 helix-grip-1
NUO78087.1
Lysobacter sp.
Skillet-3 helix-grip-1
WP_056177342.1
Lysobacter sp. Root604
Skillet-3 helix-grip-1
WP_192274078.1
Pseudoxanthomonas sp. PXM01
Skillet-3 helix-grip-1
WP_115374269.1
Adhaeribacter pallidiroseus
Skillet-3 helix-grip-1
WP_066217492.1
Balneola sp. EhC07
Skillet-3 helix-grip-1
MBL7936845.1
Bacteroidia bacterium
Skillet-3 helix-grip-1
WP_210489400.1
Rufibacter aurantiacus
Skillet-3 helix-grip-1
WP_234602470.1
Dyadobacter chenwenxiniae
Skillet-3 helix-grip-1
MBR9776726.1
Cytophagales bacterium
Skillet-3 helix-grip-1
AHM59258.1
Flammeovirgaceae bacterium 311
Skillet-3 helix-grip-1
WP_026712427.1
Flavobacterium daejeonense DSM 17708
Skillet-3 helix-grip-1
WP_157813398.1
Flavobacterium sp. 5
Skillet-3 helix-grip-1
RYY36548.1
Sphingobacteriaceae bacterium
Skillet-3 helix-grip-1
WP_051892516.1
Flavobacterium reichenbachii
Skillet-3 helix-grip-1
SOE22232.1
Spirosomataceae bacterium TFI 002
Skillet-3 helix-grip-1
WP_055146099.1
Jiulongibacter sediminis
Skillet-3 helix-grip-1
WP_159779511.1
Flavobacterium sp. 9AF
Skillet-3 helix-grip-1
EAS19566.1
Flavobacteria bacterium BBFL7
Skillet-3 helix-grip-1
WP_150011934.1
Paenimyroides baculatum
Skillet-3 helix-grip-1
WP_024769524.1
Aquimarina macrocephali JAMB N27
Skillet-3 helix-grip-1
VVV00866.1
Mesonia oceanica
Skillet-3 helix-grip-1
WP_150546801.1
Mesonia oceanica
Skillet-3 helix-grip-1
WP_232395859.1
Mangrovimonas futianensis
Skillet-3 helix-grip-1
WP_210646878.1
Flavobacterium flabelliforme
Skillet-3 helix-grip-1
MBL7855893.1
Cyclobacteriaceae bacterium
Skillet-3 helix-grip-1
TQI71147.1
Gramella sp. Hel_I_59
Skillet-3 helix-grip-1
WP_064966837.1
Tenacibaculum ovolyticum
Skillet-3 helix-grip-1
QCK15583.1
Mangrovivirga cuniculi
Skillet-3 helix-grip-1
GFZ79319.1
Aquaticitalea lipolytica
Skillet-3 helix-grip-1
WP_128755253.1
Aquimarina sediminis
Skillet-3 helix-grip-1
KYG74913.1
Roseivirga ehrenbergii
Skillet-3 helix-grip-1
WP_123890362.1
Flavobacterium frigidimaris
Skillet-3 helix-grip-1

WP_132065866.1 *Aquimarina spinulae*	Skillet-3, helix-grip-1
EDM42840.1 *unidentified eubacterium SCB49*	Skillet-3, helix-grip-1
KOY86277.1 *bacterium 336/3*	Skillet-3, helix-grip-1
WP_162926991.1 *Flavobacterium psychrotrophum*	Skillet-3, helix-grip-1
NTS39554.1 *Flavisolibacter longurius*	Skillet-3, helix-grip-1
SHL76569.1 *Chryseobacterium polytrichastri*	Skillet-3, helix-grip-1, Glyoxalase
WP_055980756.1 *Chryseobacterium sp. Leaf405*	Skillet-3, helix-grip-1, Glyoxalase
MPS65650.1 *Chryseobacterium sp.*	Skillet-3, helix-grip-1, Glyoxalase
WP_133943808.1 *Epilithonimonas xixisoli*	Skillet-3, helix-grip-1, Glyoxalase
KPE53010.1 *Chryseobacterium indologenes*	Skillet-3, helix-grip-1, Glyoxalase
WP_080780009.1 *Chryseobacterium phocaeense*	Skillet-3, helix-grip-1, Glyoxalase
MBB1575037.1 *Flavobacteriaceae bacterium*	Skillet-3, helix-grip-1
WP_123843025.1 *Chryseobacterium arthrosphaerae*	Skillet-3, helix-grip-1, Glyoxalase
MBB1284794.1 *Flavisolibacter longurius*	Skillet-3, helix-grip-1
WP_162944594.1 *Flavisolibacter nicotianae*	Skillet-3, helix-grip-1
MCE3282721.1 *Chitinophagaceae bacterium*	Skillet-3, helix-grip-1
PZR24321.1 *Citrobacter freundii*	Skillet-3, helix-grip-1
TXJ23263.1 *Chitinophagaceae bacterium*	Skillet-3, helix-grip-1
WP_020537522.1 *Lewinella cohaerens DSM 23179*	Skillet-3, helix-grip-1
WP_101688334.1 *Dysgonomonas massiliensis*	Skillet-3, helix-grip-1
WP_129004767.1 *Filimonas effusa*	Skillet-3, helix-grip-1
MBL7881621.1 *Chryseobacterium gambrini*	Skillet-3, helix-grip-1
WP_211073041.1 *Aquimarina sp. MMG016*	Skillet-3, helix-grip-1
WP_163491948.1	Skillet-3, helix-grip-1
MBL0911917.1 *Bacteroidia bacterium*	Skillet-3, helix-grip-1
WP_163309302.1 *Dysgonomonas sp. 521*	Skillet-3, helix-grip-1
PZR20902.1 *Flavobacterium psychrophilum*	Skillet-3, helix-grip-1
MCB0642798.1 *Phaeodactylibacter sp.*	Skillet-3, helix-grip-1
WP_139258328.1 *Epilithonimonas mollis*	Skillet-3, helix-grip-1, Glyoxalase
MBS0647318.1 *Verrucomicrobiota bacterium*	Skillet-3, helix-grip-1
WP_123892020.1 *Chryseobacterium mucoviscidosis*	Skillet-3, helix-grip-1
MBX3239198.1 *Chitinophagaceae bacterium*	Skillet-3, helix-grip-1
WP_078011720.1 *Hymenobacter sp. CRA2*	Skillet-3, helix-grip-1
MBX2924303.1 *Chitinophagaceae bacterium*	Skillet-3, helix-grip-1
WP_194778631.1 *Pararhodonellum marinum*	Skillet-3, helix-grip-1
WP_007841231.1 *Chryseobacterium populi*	Skillet-3, helix-grip-1
WP_091210905.1 *Mucilaginibacter gossypiicola*	Skillet-3, helix-grip-1
WP_113648841.1 *Pedobacter chitinilyticus*	Skillet-3, helix-grip-1
WP_089029940.1 *Chryseobacterium sp. T16E-39*	Skillet-3, helix-grip-1
WP_172284135.1 *Chryseobacterium sp. LAM-KRS1*	Skillet-3, helix-grip-1
MBO9563898.1 *Niastella sp.*	Skillet-3, helix-grip-1
WP_076451012.1 *Chryseobacterium piscicola*	Skillet-3, helix-grip-1
OJV55812.1 *Bacteroidetes bacterium 43-16*	Skillet-3, helix-grip-1
WP_230935102.1 *Psychroserpens luteolus*	Skillet-3, helix-grip-1
WP_118950197.1 *Taibaiella helva*	Glyoxalase, Skillet-3, helix-grip-1
HAR73989.1 *Flavobacteriaceae bacterium*	Skillet-3, helix-grip-1
WP_204345033.1 *Psychroserpens algicola*	Skillet-3, helix-grip-1
WP_118976843.1 *Taibaiella koreensis*	Glyoxalase, Skillet-3, helix-grip-1
MBP1616869.1 *Bacteroidota bacterium*	Skillet-3, helix-grip-1
WP_212228692.1 *Carboxylicivirga mesophila*	Skillet-3, helix-grip-1
WP_108823610.1 *Dysgonomonas sp. Marseille-P4361*	Skillet-3, helix-grip-1
WP_169673071.1 *Flavivirga algicola*	Skillet-3, helix-grip-1
MBR9846974.1 *Algicola sp.*	Skillet-3, helix-grip-1
WP_185112969.1 *Dysgonomonas sp. BGC7*	Skillet-3, helix-grip-1
WP_139997818.1 *Flavobacterium microcysteis*	Skillet-3, helix-grip-1
MCB0570023.1 *Phaeodactylibacter sp.*	Skillet-3, helix-grip-1
MBK9461772.1 *Sphingobacteriales bacterium*	Skillet-3, helix-grip-1
WP_118456461.1 *Aquimarina sp. BL5*	Skillet-3, helix-grip-1
HIA35943.1 *Flavobacteriales bacterium*	Skillet-3, helix-grip-1
MBO6516886.1 *Bacteroidia bacterium*	Skillet-3, helix-grip-1
WP_229685113.1 *Pedobacter zeae*	Skillet-3, helix-grip-1
EON77179.1 *Lunatimonas lonarensis*	Skillet-3, helix-grip-1
RME97920.1 *Bacteroidota bacterium*	Skillet-3, helix-grip-1
WP_090001916.1 *Chryseobacterium taichungense*	Skillet-3, helix-grip-1, ?, Glyoxalase
MCC7401054.1 *Chitinophagaceae bacterium*	Skillet-3, helix-grip-1
WP_228448779.1 *Chryseobacterium mulctrae*	Skillet-3, helix-grip-1
WP_199119073.1 *Pedobacter sp. ASV28*	Skillet-3, helix-grip-1
WP_165796683.1 *Chryseobacterium sp. HMWF001*	Skillet-3, helix-grip-1
PTT72499.1 *Chryseobacterium sp. HMWF001*	Skillet-3, helix-grip-1
WP_153797571.1 *Foetidibacter luteolus*	Skillet-3, helix-grip-1

###;helix-grip family 2

WP_155454294.1 *Massilia eburnea*	Skillet-3, helix-grip-2
QQS14653.1 *Rhodospirillales bacterium*	Skillet-3, helix-grip-2
ATP27080.1 *Chromobacterium violaceum*	Skillet-3, helix-grip-2
WP_124538919.1 *Piscinibacter terrae*	Skillet-3, helix-grip-2
WP_180568093.1 *Spartinivicinus marinus*	Skillet-3, helix-grip-2
WP_019866960.1 *Methylovulum miyakonense HT12*	Skillet-3, helix-grip-2
MBL8512530.1 *Betaproteobacteria bacterium*	Skillet-3, helix-grip-2
WP_020482437.1 *Methylomonas sp. MK1*	Skillet-3, helix-grip-2
APV51982.1 *Betaproteobacteria bacterium GR16-43*	Skillet-3, helix-grip-2
RPI60854.1 *Lysobacterales bacterium*	Skillet-3, helix-grip-2, helix-grip-2

#;helix-grip family3

WP_187964232.1 *Sinomicrobium weinanense*	Skillet-3, helix-grip-3
MBK9453552.1 *Bacteroidota bacterium*	Skillet-3, helix-grip-3
MBU0488134.1 *Bacteroidota bacterium*	Skillet-3, helix-grip-3
MBU0763530.1 *Bacteroidota bacterium*	Skillet-3, helix-grip-3
WP_229944612.1 *Myroides oncorhynchi*	Skillet-3, helix-grip-3
WP_121964010.1 *Myroides sp. N17-2*	Skillet-3, helix-grip-3
WP_163266358.1 *Dysgonomonas sp. 216*	Skillet-3, helix-grip-3
MBK9460716.1 *Sphingobacteriales bacterium*	Skillet-3, helix-grip-3
WP_013767659.1 *Haliscomenobacter hydrossis DSM 1100*	Skillet-3, helix-grip-3
WP_133229434.1 *Mucilaginibacter psychrotolerans*	Skillet-3, helix-grip-3
WP_090651321.1 *Mucilaginibacter sp. OK283*	Skillet-3, helix-grip-3

Accession	Organism	Domains
WP_191191530.1	*Mucilaginibacter pankratovii*	Skillet-3 helix-grip-3
WP_078349962.1	*Mucilaginibacter pedocola*	Skillet-3 helix-grip-3
WP_166114357.1	*Dysgonomonas sp. HDW5A*	Skillet-3 helix-grip-3
WP_025124041.1	*Myroides odoratimimus*	Skillet-3 helix-grip-3
QQS33276.1	*Acidobacteriota bacterium*	Skillet-3 helix-grip-3 HAD
GGX16867.1	*Aquimarina muelleri*	Skillet-3 helix-grip-3
WP_100342688.1	*Mucilaginibacter auburnensis*	Skillet-3 helix-grip-3
WP_188495236.1	*Flavobacterium palustre*	Skillet-3 helix-grip-3
WP_163608325.1	*Leptobacterium flavescens*	Skillet-3 helix-grip-3
HCY83002.1	*Xanthomarina gelatinilytica*	Skillet-3 helix-grip-3
MBO9594801.1		Skillet-3 helix-grip-3
WP_090390571.1	*Niabella drilacis*	Skillet-3 helix-grip-3
WP_223710797.1	*Niabella beijingensis*	Skillet-3 helix-grip-3
MBE7171504.1	*Williamsia sp.*	Skillet-3 helix-grip-3
WP_143095679.1	*Paenimyroides ummariense*	Skillet-3 helix-grip-3
MCB0711005.1	*Ignavibacteriota bacterium*	Skillet-3 helix-grip-3
MBS1635262.1	*Bacteroidota bacterium*	Skillet-3 helix-grip-3
MCB0853723.1	*Bacteroidota bacterium*	Skillet-3 helix-grip-3
MCB0607389.1	*Lewinella sp.*	Skillet-3 helix-grip-3
MBX3291063.1	*Acidobacteriota bacterium*	Skillet-3 helix-grip-3
NVO04301.1	*Bacteroidota bacterium*	Skillet-3 helix-grip-3
TNE54518.1	*Bacteroidota bacterium*	Skillet-3 helix-grip-3
OJJ17672.1	*marine bacterium AO1-C*	Skillet-3 helix-grip-3
MBX2889400.1	*Saprospiraceae bacterium*	Skillet-3 helix-grip-3
MBV9923479.1	*Acidobacteriota bacterium*	Skillet-3 helix-grip-3
WP_013766120.1	*Haliscomenobacter hydrossis DSM 1100*	Skillet-3 helix-grip-3
MBS1538491.1	*Bacteroidota bacterium*	Skillet-3 helix-grip-3
MBA3805470.1	*Acidobacteriota bacterium*	Skillet-3 helix-grip-3
WP_044227546.1	*Phaeodactylibacter xiamenensis*	Skillet-3 helix-grip-3
WP_048921677.1	*Rufibacter radiotolerans*	Skillet-3 helix-grip-3
NBC07322.1		Skillet-3 helix-grip-3
MBN8679959.1	*Chitinophagales bacterium*	Skillet-3 helix-grip-3
MBL7803504.1	*Saprospiraceae bacterium*	Skillet-3 helix-grip-3
GGK80604.1	*Rufibacter glacialis*	Skillet-3 helix-grip-3
MCA1762042.1	*Flavobacteriales bacterium*	Skillet-3 helix-grip-3
NJM53048.1	*Blastocatellia bacterium*	Skillet-3 helix-grip-3 HAD

#;single association with vWA mentioned above

Accession	Organism	Domains
MBE2246313.1	*Candidatus Competibacteraceae bacterium*	Skillet-3 helix-grip-3 ? 3helix vWA-insert beta-sandwich

######;other beta-sandwich associations

Accession	Organism	Domains
MCC6526117.1	*Polyangiaceae bacterium*	Skillet-3 beta-sandwich
WP_068219004.1	*Lewinella sp. 4G2*	Skillet-3 beta-sandwich
MBR9916069.1	*Algicola sp.*	Skillet-3 beta-sandwich
WP_090170771.1	*Neolewinella agarilytica*	Skillet-3 beta-sandwich
PHI21237.1	*Lewinellaceae bacterium SD302*	Skillet-3 beta-sandwich
MCA9717328.1	*Myxococcales bacterium*	Skillet-3 beta-sandwich
MCA9693128.1	*Myxococcales bacterium*	Skillet-3 beta-sandwich
WP_205271485.1	*Leptospira chreensis*	Skillet-3 beta-sandwich
MBL9022273.1	*Myxococcales bacterium*	Skillet-3 beta-sandwich
MBT3202175.1	*Phycisphaerales bacterium*	Skillet-3 beta-sandwich
MBN2352678.1	*Spirochaetales bacterium*	Skillet-3 beta-sandwich
MBN2436298.1	*Spirochaetota bacterium*	Skillet-3 beta-sandwich
MBL9023633.1	*Myxococcales bacterium*	Skillet-3 beta-sandwich
NUO47458.1	*Polyangiaceae bacterium*	Skillet-3 beta-sandwich
NUP14112.1	*Polyangiaceae bacterium*	Skillet-3 beta-sandwich beta-sandwich
MCB1192126.1	*Leptospiraceae bacterium*	Skillet-3 beta-sandwich
WP_156895957.1	*Leptospira broomii serovar Hurstbridge str. 5399*	Skillet-3 beta-sandwich
MCB1325414.1	*Leptospiraceae bacterium*	Skillet-3 beta-sandwich
MCB1178902.1	*Leptospiraceae bacterium*	Skillet-3 beta-sandwich
MBL8019247.1	*Leptospirales bacterium*	Skillet-3 beta-sandwich
MBK8219257.1	*Myxococcales bacterium*	Skillet-3 beta-sandwich

#;additional lipocalin-like domain C-terminal fusion

Accession	Organism	Domains
WP_109191099.1	*Flavobacterium crocinum*	Skillet-3 CDC-like-b-sandwich-2-helix-insert lipocalin-like
WP_051886117.1	*Flavobacterium hydatis*	Skillet-3 CDC-like-b-sandwich-2-helix-insert lipocalin-like
WP_089480459.1	*Flavobacterium araucananum*	Skillet-3 CDC-like-b-sandwich-2-helix-insert lipocalin-like
OXA68422.1	*Flavobacterium aquidurense*	Skillet-3 CDC-like-b-sandwich-2-helix-insert lipocalin-like
OMQ12700.1	*[Flexibacter] sp. ATCC 35103*	Skillet-3 CDC-like-b-sandwich-2-helix-insert lipocalin-like
WP_213258757.1	*Flavobacterium branchiicola*	Skillet-3 CDC-like-b-sandwich-2-helix-insert lipocalin-like
WP_144216596.1	*uncultured Flavobacterium sp.*	Skillet-3 CDC-like-b-sandwich-2-helix-insert lipocalin-like
WP_154281116.1	*Pedobacter petrophilus*	Skillet-3 CDC-like-b-sandwich-2-helix-insert lipocalin-like
RZK35606.1	*Pedobacter sp.*	Skillet-3 CDC-like-b-sandwich-2-helix-insert lipocalin-like

#;further association with membrane-anchored cysteine-rich helical bundle

Accession	Organism	Domains
WP_100790291.1	*Leptospira brenneri*	C-rich-helical-bundle Skillet-3 beta-sandwich
MCB1159793.1	*Leptospiraceae bacterium*	C-rich-helical-bundle Skillet-3 beta-sandwich
WP_135693056.1	*Leptospira mtsangambouensis*	C-rich-helical-bundle Skillet-3 beta-sandwich
NCN08976.1	*Leptospira sp.*	C-rich-helical-bundle Skillet-3 beta-sandwich
WP_002973701.1	*Leptospira terpstrae serovar Hualin str. LT 11-33 = ATCC 700639*	C-rich-helical-bundle Skillet-3 beta-sandwich
WP_002975892.1	*Leptospira vanthielii serovar Holland str. Waz Holland = ATCC 700522*	C-rich-helical-bundle Skillet-3 beta-sandwich
WP_125232048.1	*Leptospira brenneri*	C-rich-helical-bundle Skillet-3 beta-sandwich
WP_135633884.1	*Leptospira kanakyensis*	C-rich-helical-bundle Skillet-3 beta-sandwich
WP_002979207.1		C-rich-helical-bundle Skillet-3 beta-sandwich
MBK6608971.1	*Leptospiraceae bacterium*	C-rich-helical-bundle Skillet-3 beta-sandwich

#;operonic association

Accession	Organism	Domains
WP_127783222.1	*Rhodococcus sp. X156*	beta-sandwich Skillet-3
WP_203675411.1	*Cellulomonas phragmiteti*	beta-sandwich Skillet-3
WP_207339233.1	*Cellulomonas wangleii*	beta-sandwich Skillet-3
MTA64236.1	*Actinomycetota bacterium*	beta-sandwich Skillet-3
HHX86444.1	*Actinomycetales bacterium*	beta-sandwich Skillet-3
WP_073566134.1	*Archangium sp. Cb G35*	beta-sandwich ? Skillet-3
NVJ08600.1	*Myxococcus sp. AM001*	beta-sandwich Skillet-3
WP_234063231.1	*Myxococcus stipitatus*	beta-sandwich Skillet-3
NLG47033.1	*Gordonia sp. (in: high G+C Gram-positive bacteria)*	beta-sandwich Skillet-3
WP_120608997.1	*Corallococcus sp. CA053C*	beta-sandwich Skillet-3
AEI63086.1	*Corallococcus macrosporus*	beta-sandwich Skillet-3
WP_163867588.1	*Myxococcus eversor*	beta-sandwich Skillet-3

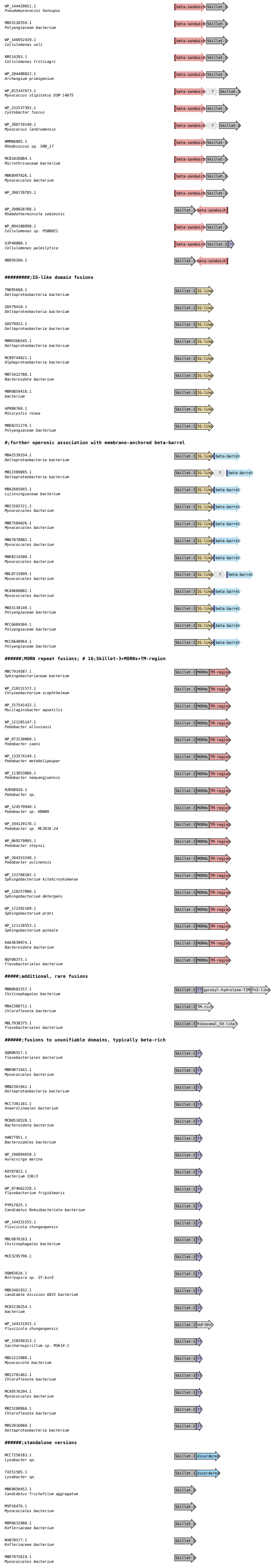
WP_144429651.1
Pseudomyxococcus hansupus
beta-sandwich Skillet-3
MBX3128359.1
Polyangiaceae bacterium
beta-sandwich Skillet-3
WP_146952439.1
Cellulomonas soli
beta-sandwich Skillet-3
RMI14293.1
Cellulomonas triticagri
beta-sandwich Skillet-3
WP_204488822.1
Archangium primigenium
beta-sandwich Skillet-3
WP_015347673.1
Myxococcus stipitatus DSM 14675
beta-sandwich ? Skillet-3
WP_232537392.1
Cystobacter fuscus
beta-sandwich Skillet-3
WP_206719140.1
Myxococcus landrumensis
beta-sandwich ? Skillet-3
NMM86005.1
Rhodococcus sp. SRB_17
beta-sandwich Skillet-3
MCB1026884.1
Microthrixaceae bacterium
beta-sandwich Skillet-3
MBK8997826.1
Myxococcales bacterium
beta-sandwich Skillet-3
WP_206729785.1
beta-sandwich Skillet-3
WP_208028788.1
Rhabdothermincola sediminis
Skillet-3 beta-sandwich
WP_094180898.1
Cellulomonas sp. PSBB021
beta-sandwich Skillet-3
UJP40000.1
Cellulomonas palmilytica
beta-sandwich Skillet-3 ??
NBO56266.1
Skillet-3 beta-sandwich
#########;IG-like domain fusions
TNE85668.1
Deltaproteobacteria bacterium
Skillet-3 IG-like
GDX79410.1
Deltaproteobacteria bacterium
Skillet-3 IG-like
GDX79922.1
Deltaproteobacteria bacterium
Skillet-3 IG-like
MBM4366545.1
Deltaproteobacteria bacterium
Skillet-3 IG-like
MCB9744821.1
Alphaproteobacteria bacterium
Skillet-3 IG-like
MBT3422788.1
Bacteroidota bacterium
Skillet-3 IG-like
MBR9859418.1
bacterium
Skillet-3 IG-like
APR80760.1
Minicystis rosea
Skillet-3 IG-like
MBK8251270.1
Polyangiaceae bacterium
Skillet-3 IG-like
#;further operonic association with membrane-anchored beta-barrel
MBA2539334.1
Deltaproteobacteria bacterium
Skillet-3 IG-like beta-barrel
MBI2389095.1
Deltaproteobacteria bacterium
Skillet-3 IG-like ? beta-barrel
MBA2665045.1
Lujinxingiaceae bacterium
Skillet-3 IG-like beta-barrel
MBI3203721.1
Myxococcales bacterium
Skillet-3 IG-like beta-barrel
MBK7584026.1
Myxococcales bacterium
Skillet-3 IG-like beta-barrel
MBK7078802.1
Myxococcales bacterium
Skillet-3 IG-like beta-barrel
MBK8214500.1
Myxococcales bacterium
Skillet-3 IG-like beta-barrel
MBL8715099.1
Myxococcales bacterium
Skillet-3 IG-like ? beta-barrel
MCA9668002.1
Myxococcales bacterium
Skillet-3 IG-like beta-barrel
MBX3130149.1
Polyangiaceae bacterium
Skillet-3 IG-like beta-barrel
MCC6669304.1
Polyangiaceae bacterium
Skillet-3 IG-like beta-barrel
MCC6648964.1
Polyangiaceae bacterium
Skillet-3 IG-like beta-barrel
######;MORN repeat fusions; # 16;Skillet-3+MORNs+TM-region
MBC7914587.1
Sphingobacteriaceae bacterium
Skillet-3 MORNs TM-region
WP_210151572.1
Chryseobacterium scophthalmum
Skillet-3 MORNs TM-region
WP_157541432.1
Mucilaginibacter aquatilis
Skillet-3 MORNs TM-region
WP_121285147.1
Pedobacter alluvionis
Skillet-3 MORNs TM-region
WP_073230000.1
Pedobacter caeni
Skillet-3 MORNs TM-region
WP_133574149.1
Pedobacter metabolipauper
Skillet-3 MORNs TM-region
WP_113653086.1
Pedobacter namyangjuensis
Skillet-3 MORNs TM-region
RZK68926.1
Pedobacter sp.
Skillet-3 MORNs TM-region
WP_124579940.1
Pedobacter sp. KBW06
Skillet-3 MORNs TM-region
WP_194129178.1
Pedobacter sp. MC2016-24
Skillet-3 MORNs TM-region
WP_069379095.1
Pedobacter steynii
Skillet-3 MORNs TM-region
WP_204353248.1
Pedobacter yulinensis
Skillet-3 MORNs TM-region
WP_132768182.1
Sphingobacterium kitahiroshimense
Skillet-3 MORNs TM-region
WP_120257080.1
Sphingobacterium detergens
Skillet-3 MORNs TM-region
WP_172392109.1
Sphingobacterium prati
Skillet-3 MORNs TM-region
WP_121126553.1
Sphingobacterium puteale
Skillet-3 MORNs TM-region
KAA3639074.1
Bacteroidota bacterium
Skillet-3 MORNs TM-region
NQY08375.1
Flavobacteriales bacterium
Skillet-3 MORNs TM-region
#####;additional, rare fusions
MBN8683257.1
Chitinophagales bacterium
Skillet-3 ?? gycosyl-hydrolase-TIM Fn3-like
MBA2380712.1
Chloroflexota bacterium
Skillet-3 TM-rich
MBL7938375.1
Flavobacteriales bacterium
Skillet-3 Ribosomal_S9-like?
######;fusions to ununifiable domains, typically beta-rich
QQR86557.1
Flavobacteriales bacterium
Skillet-3 ??
MBK9071641.1
Myxococcales bacterium
Skillet-3 ??
MBN2361941.1
Deltaproteobacteria bacterium
Skillet-3 ??
MCC7361181.1
Anaerolineales bacterium
Skillet-3 ??
MCB0510528.1
Bacteroidota bacterium
Skillet-3 ??
HAN77051.1
Bacteroidales bacterium
Skillet-3 ??
WP_196894658.1
Aureivirga marina
Skillet-3 ??
KOY87822.1
bacterium 336/3
Skillet-3 ??
WP_074662228.1
Flavobacterium frigidimaris
Skillet-3 ??
PYM17625.1
Candidatus Rokuibacteriota bacterium
Skillet-3 ??
WP_144331555.1
Fluviicola chungangensis
Skillet-3 ??
MBL6876163.1
Chitinophagales bacterium
Skillet-3 ??
MCE3295796.1
Skillet-3 ??
OQW63616.1
Nitrospira sp. ST-bin5
Skillet-3 ??
MBD3401932.1
candidate division GN15 bacterium
Skillet-3 ??
MCB2230254.1
bacterium
Skillet-3 ??
WP_144331915.1
Fluviicola chungangensis
Skillet-3 DUF3997
WP_158269313.1
Saccharospirillum sp. MSK14-1
Skillet-3 ??
MBU1222088.1
Myxococcota bacterium
Skillet-3 ??
MBI2781462.1
Chloroflexota bacterium
Skillet-3 ??
MCA9576294.1
Myxococcales bacterium
Skillet-3 ??
MBI5288860.1
Chloroflexota bacterium
Skillet-3 ??
MBS2016060.1
Deltaproteobacteria bacterium
Skillet-3 ??
######;standalone versions
MCC7250183.1
Lysobacter sp.
Skillet-3 disordered
TXI51585.1
Lysobacter sp.
Skillet-3 disordered
MBK9050452.1
Candidatus Trichofilum aggregatum
Skillet-3
MSP16476.1
Myxococcales bacterium
Skillet-3
MBP6632980.1
Kofleriaceae bacterium
Skillet-3
NVB79577.1
Kofleriaceae bacterium
Skillet-3
MBK7075619.1
Myxococcales bacterium
Skillet-3

RMH45202.1	*Deltaproteobacteria bacterium*	Skillet-3
MBP8808786.1	*Kofleriaceae bacterium*	Skillet-3
MBA2540833.1	*Deltaproteobacteria bacterium*	Skillet-3
MBN1372124.1	*Anaerolineaceae bacterium*	Skillet-3
WP_207620898.1	*Streptosporangium minutum*	Skillet-3
WP_170229405.1	*Polyangium fumosum*	Skillet-3
MBL8918970.1	*Myxococcaceae bacterium*	Skillet-3
MBK9450291.1	*Bacteroidota bacterium*	Skillet-3
MCE2469667.1	*Dehalococcoidia bacterium*	Skillet-3
MBK7534963.1	*Myxococcales bacterium*	Skillet-3
NLH08736.1	*Chloroflexota bacterium*	Skillet-3
MBL8617663.1	*Deltaproteobacteria bacterium*	Skillet-3
WP_157518663.1	*Herbidospora mongoliensis*	Skillet-3
NUQ77337.1	*Polyangiaceae bacterium*	Skillet-3
MBT9561123.1	*Myxococcales bacterium*	Skillet-3
MBL8944833.1	*Myxococcales bacterium*	Skillet-3
MBM4282926.1	*Deltaproteobacteria bacterium*	Skillet-3
MBI2893864.1	*Deltaproteobacteria bacterium*	Skillet-3
MBP9088704.1	*Kofleriaceae bacterium*	Skillet-3
MSW41544.1	*Actinomycetota bacterium*	Skillet-3
WP_206798330.1	*Corallococcus sp. NCRR*	Skillet-3
WP_169819517.1		Skillet-3
WP_172808305.1	*Corallococcus exiguus*	Skillet-3
MBK7579745.1	*Myxococcales bacterium*	Skillet-3
NQW72417.1	*Actinomycetota bacterium*	Skillet-3
MBX2812729.1	*Myxococcales bacterium*	Skillet-3
MBJ81165.1	*Myxococcales bacterium*	Skillet-3
MCA9584409.1	*Myxococcales bacterium*	Skillet-3
MBI5512624.1	*Deltaproteobacteria bacterium*	Skillet-3
MCA9718925.1	*Myxococcales bacterium*	Skillet-3
MCB9677400.1	*Alphaproteobacteria bacterium*	Skillet-3
MBM4364401.1	*Deltaproteobacteria bacterium*	Skillet-3
WP_095977183.1	*Melittangium boletus DSM 14713*	Skillet-3
MCB1041181.1	*Acidimicrobiales bacterium*	Skillet-3
MBN8609777.1	*Deltaproteobacteria bacterium*	Skillet-3
MBA2557804.1	*Chloroflexota bacterium*	Skillet-3
WP_056155251.1	*Nocardioides sp. Root224*	Skillet-3
WP_012830915.1	*Haliangium ochraceum DSM 14365*	Skillet-3
MCB9637728.1	*Myxococcales bacterium*	Skillet-3
MBP9111757.1	*Polyangiaceae bacterium*	Skillet-3
MBN2528196.1	*Deltaproteobacteria bacterium*	Skillet-3
HHK71217.1		Skillet-3
MBX3207483.1	*Labilithrix sp.*	Skillet-3
WP_210728663.1	*Cellulomonas septica*	Skillet-3
MCA9847682.1	*Dehalococcoidia bacterium*	Skillet-3
MCB9593239.1	*Sandaracinaceae bacterium*	Skillet-3
MAC29264.1	*Myxococcales bacterium*	Skillet-3
MBN2718263.1	*Deltaproteobacteria bacterium*	Skillet-3
WP_203558465.1	*Bacteroides sp. 519*	Skillet-3
MBI5526247.1	*Deltaproteobacteria bacterium*	Skillet-3
MBO6940435.1	*Deltaproteobacteria bacterium*	Skillet-3
WP_136927659.1	*Polyangium fumosum*	Skillet-3
MBK7992883.1	*Blastocatellia bacterium*	Skillet-3
SEU13722.1	*Stigmatella erecta*	Skillet-3
MBI5515898.1	*Deltaproteobacteria bacterium*	Skillet-3
MBL8677871.1	*Myxococcales bacterium*	Skillet-3
MCC7111552.1	*Deltaproteobacteria bacterium*	Skillet-3
MCC7074865.1	*Deltaproteobacteria bacterium*	Skillet-3
MBT8493616.1	*Deltaproteobacteria bacterium*	Skillet-3
RYZ08386.1	*Myxococcales bacterium*	Skillet-3
WP_218041224.1	*Acrocarpospora macrocephala*	Skillet-3
MCA2959111.1	*Silvanigrellales bacterium*	Skillet-3
AKF83620.1	*Myxococcus fulvus 124B02*	Skillet-3
TXH40965.1	*Actinomycetota bacterium*	Skillet-3
GDX81409.1	*Deltaproteobacteria bacterium*	Skillet-3
MBA2544672.1	*Deltaproteobacteria bacterium*	Skillet-3
MCA9714922.1	*Myxococcales bacterium*	Skillet-3
GEK21191.1	*Cellulomonas xylanilytica*	Skillet-3
MBT3223204.1	*Pseudomonadota bacterium*	Skillet-3
MBM4251995.1	*Deltaproteobacteria bacterium*	Skillet-3
MBA3457633.1	*Deltaproteobacteria bacterium*	Skillet-3
OGO57905.1	*Chloroflexi bacterium RBG_16_72_14*	Skillet-3
TNE90033.1	*Deltaproteobacteria bacterium*	Skillet-3
NOU28796.1	*Polyangiaceae bacterium*	Skillet-3
HIN92492.1	*Alphaproteobacteria bacterium*	Skillet-3
RMG11865.1	*Deltaproteobacteria bacterium*	Skillet-3
MBH24953.1	*Myxococcales bacterium*	Skillet-3
GDX79267.1	*Deltaproteobacteria bacterium*	Skillet-3
PKB56707.1	*SAR202 cluster bacterium Casp-Chloro-G1*	Skillet-3
OJT18898.1	*Archangium sp. Cb G35*	Skillet-3
KAB2901926.1	*Kofleriaceae bacterium*	Skillet-3
WP_002636913.1	*Pseudomyxococcus hansupus*	Skillet-3
MBV8756985.1	*Deltaproteobacteria bacterium*	Skillet-3
WP_114339532.1	*Corallincola holothuriorum*	Skillet-3
MCA2977953.1	*Myxococcaceae bacterium*	Skillet-3
RYZ48588.1	*Sphingobacteriales bacterium*	Skillet-3
MBL4847335.1	*Planctomycetota bacterium*	Skillet-3
AZZ37968.1	*Bdellovibrio sp. qaytius*	Skillet-3
MBK6847076.1	*Pseudomonadota bacterium*	Skillet-3
MBC7464362.1	*Bdellovibrio sp.*	Skillet-3
MBI4509851.1	*Deltaproteobacteria bacterium*	Skillet-3
KYF88378.1	*Sorangium cellulosum*	Skillet-3
MUH52293.1	*Actinomycetota bacterium*	Skillet-3

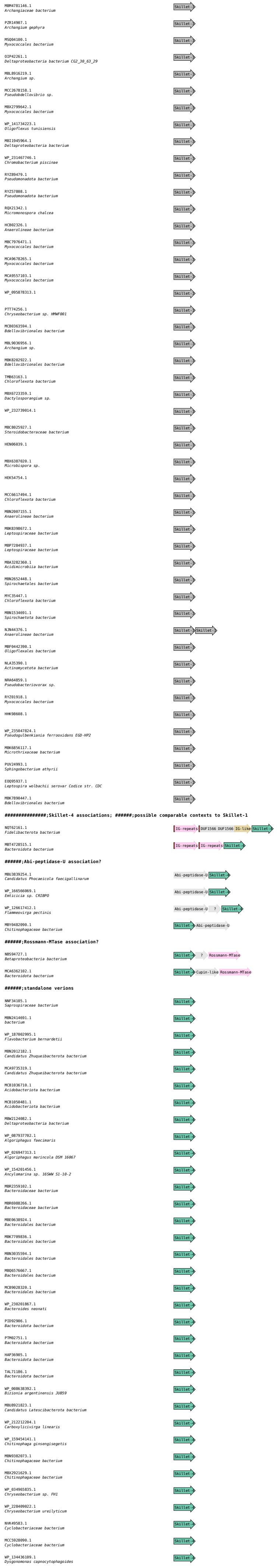
MBM4781146.1
Archangiaceae bacterium
Skillet-3
PZR14907.1
Archangium gephyra
Skillet-3
MSQ04100.1
Myxococcales bacterium
Skillet-3
OIP42261.1
Deltaproteobacteria bacterium CG2_30_63_29
Skillet-3
MBL8916219.1
Archangium sp.
Skillet-3
MCC2678158.1
Pseudobdellovibrio sp.
Skillet-3
MBX2799642.1
Myxococcales bacterium
Skillet-3
WP_141734223.1
Oligoflexus tunisiensis
Skillet-3
MBI1945964.1
Deltaproteobacteria bacterium
Skillet-3
WP_231467746.1
Chromobacterium piscinae
Skillet-3
RYZ89479.1
Pseudomonadota bacterium
Skillet-3
RYZ57808.1
Pseudomonadota bacterium
Skillet-3
RQX21342.1
Micromonospora chalcea
Skillet-3
HCB02326.1
Anaerolineae bacterium
Skillet-3
MBC7976471.1
Myxococcales bacterium
Skillet-3
MCA9678265.1
Myxococcales bacterium
Skillet-3
MCA9557103.1
Myxococcales bacterium
Skillet-3
WP_095878313.1
Skillet-3
PTT74256.1
Chryseobacterium sp. HMWF001
Skillet-3
MCB0363594.1
Bdellovibrionales bacterium
Skillet-3
MBL9036956.1
Archangium sp.
Skillet-3
MBK8202922.1
Bdellovibrionales bacterium
Skillet-3
TMB63163.1
Chloroflexota bacterium
Skillet-3
MBX6723359.1
Dactylosporangium sp.
Skillet-3
WP_232739014.1
Skillet-3
MBC8025927.1
Steroidobacteraceae bacterium
Skillet-3
HEN06039.1
Skillet-3
MBX6387020.1
Microbispora sp.
Skillet-3
HEK54754.1
Skillet-3
MCC6617494.1
Chloroflexota bacterium
Skillet-3
MBN2007155.1
Anaerolineae bacterium
Skillet-3
MBK8398672.1
Leptospiraceae bacterium
Skillet-3
MBP7284937.1
Leptospiraceae bacterium
Skillet-3
MBA3282360.1
Acidimicrobiia bacterium
Skillet-3
MBN2652448.1
Spirochaetales bacterium
Skillet-3
MYC35447.1
Chloroflexota bacterium
Skillet-3
MBN1534691.1
Spirochaetota bacterium
Skillet-3
NJN44376.1
Anaerolineae bacterium
Skillet-3
Skillet-3
MBF0442390.1
Oligoflexales bacterium
Skillet-3
NLA35390.1
Actinomycetota bacterium
Skillet-3
NRA64859.1
Pseudobacteriovorax sp.
Skillet-3
RYZ01918.1
Myxococcales bacterium
Skillet-3
HHK98608.1
Skillet-3
WP_235047824.1
Pseudogulbenkiania ferrooxidans EGD-HP2
Skillet-3
MBK6856117.1
Microthrixaceae bacterium
Skillet-3
PUV24993.1
Sphingobacterium athyrii
Skillet-3
EOQ95937.1
Leptospira wolbachii serovar Codice str. CDC
Skillet-3
MBK7890447.1
Bdellovibrionales bacterium
Skillet-3
###############;Skillet-4 associations; ######;possible comparable contexts to Skillet-1
NQT62161.1
Fidelibacterota bacterium
IG-repeats
DUF1566
DUF1566
IG-like
Skillet-4
MBT4728515.1
Bacteroidota bacterium
IG-repeats
IG-repeats
Skillet-4
######;Abi-peptidase-U association?
MBU3839254.1
Candidatus Phocaeicola faecigallinarum
Abi-peptidase-U
Skillet-4
WP_166566069.1
Emticicia sp. CRIBPO
Abi-peptidase-U
Skillet-4
WP_126617412.1
Flammeovirga pectinis
Abi-peptidase-U
?
Skillet-4
MBY0482090.1
Chitinophagaceae bacterium
Skillet-4
Abi-peptidase-U
######;Rossmann-MTase association?
NBS94727.1
Betaproteobacteria bacterium
Skillet-4
?
Rossmann-MTase
MCA6362102.1
Bacteroidota bacterium
Skillet-4
Cupin-like
Rossmann-MTase
######;standalone verions
NNF34105.1
Saprospiraceae bacterium
Skillet-4
MBN2414691.1
bacterium
Skillet-4
WP_187002995.1
Flavobacterium bernardetii
Skillet-4
MBN2012182.1
Candidatus Zhuqueibacterota bacterium
Skillet-4
MCA9735319.1
Candidatus Zhuqueibacterota bacterium
Skillet-4
MCB1036710.1
Acidobacteriota bacterium
Skillet-4
MCB1050481.1
Acidobacteriota bacterium
Skillet-4
MBW2124082.1
Deltaproteobacteria bacterium
Skillet-4
WP_087937702.1
Algoriphagus faecimaris
Skillet-4
WP_026947313.1
Algoriphagus marincola DSM 16067
Skillet-4
WP_154201456.1
Ancylomarina sp. 16SWW S1-10-2
Skillet-4
MBR2359102.1
Bacteroidaceae bacterium
Skillet-4
MBR6988266.1
Bacteroidaceae bacterium
Skillet-4
MBE0638924.1
Bacteroidales bacterium
Skillet-4
MBK7709836.1
Bacteroidales bacterium
Skillet-4
MBN3035594.1
Bacteroidales bacterium
Skillet-4
MBQ6576667.1
Bacteroidales bacterium
Skillet-4
MCB9028320.1
Bacteroidales bacterium
Skillet-4
WP_230201867.1
Bacteroides neonati
Skillet-4
PID92906.1
Bacteroidota bacterium
Skillet-4
PTM02751.1
Bacteroidota bacterium
Skillet-4
HAP36905.1
Bacteroidota bacterium
Skillet-4
TAL71186.1
Bacteroidota bacterium
Skillet-4
WP_008638392.1
Bizionia argentinensis JUB59
Skillet-4
MBU8921823.1
Candidatus Latescibacterota bacterium
Skillet-4
WP_212212204.1
Carboxylicivirga linearis
Skillet-4
WP_159454141.1
Chitinophaga ginsengisegetis
Skillet-4
MBN9382073.1
Chitinophagaceae bacterium
Skillet-4
MBX2921629.1
Chitinophagaceae bacterium
Skillet-4
WP_034965835.1
Chryseobacterium sp. FH1
Skillet-4
WP_228409022.1
Chryseobacterium ureilyticum
Skillet-4
NVK49583.1
Cyclobacteriaceae bacterium
Skillet-4
MCC5928090.1
Cyclobacteriaceae bacterium
Skillet-4
WP_134436109.1
Dysgonomonas capnocytophagoides
Skillet-4

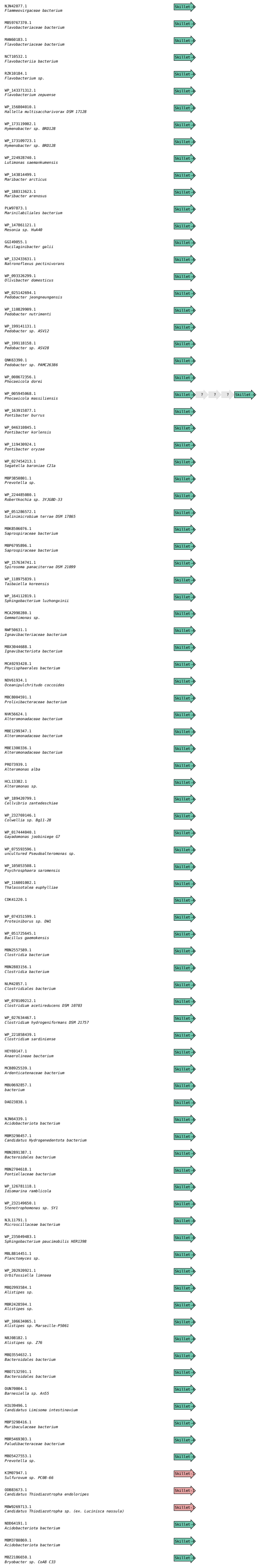

NJN42077.1
Flammeovirgaceae bacterium
MBS9767370.1
Flavobacteriaceae bacterium
MAN60183.1
Flavobacteriaceae bacterium
NCT10532.1
Flavobacteriia bacterium
RZK10184.1
Flavobacterium sp.
WP_143371312.1
Flavobacterium zepuense
WP_156804010.1
Hallella multisaccharivorax DSM 17128
WP_173119002.1
Hymenobacter sp. BRD128
WP_173109723.1
Hymenobacter sp. BRD128
WP_224928740.1
Lutimonas saemankumensis
WP_143814499.1
Maribacter arcticus
WP_188313623.1
Maribacter arenosus
PLW97873.1
Marinilabiliales bacterium
WP_147861121.1
Mesonia sp. HuA40
GGI49055.1
Mucilaginibacter galii
WP_132433631.1
Natronoflexus pectinivorans
WP_093326299.1
Olivibacter domesticus
WP_025142694.1
Pedobacter jeongneungensis
WP_110829909.1
Pedobacter nutrimenti
WP_199141131.1
Pedobacter sp. ASV12
WP_199118158.1
Pedobacter sp. ASV28
QNK63390.1
Pedobacter sp. PAMC26386
WP_008672356.1
Phocaeicola dorei
WP_005945068.1
Phocaeicola massiliensis
WP_163915877.1
Pontibacter burrus
WP_046310845.1
Pontibacter korlensis
WP_119430924.1
Pontibacter oryzae
WP_027454213.1
Segatella baroniae C21a
MBP3850801.1
Prevotella sp.
WP_224485080.1
Robertkochia sp. 3YJGBD-33
WP_051286572.1
Salinimicrobium terrae DSM 17865
MBK8506076.1
Saprospiraceae bacterium
MBP6795896.1
Saprospiraceae bacterium
WP_157634741.1
Spirosoma panaciterrae DSM 21099
WP_118975839.1
Taibaiella koreensis
WP_164112819.1
Sphingobacterium luzhongxinii
MCA2990280.1
Gemmatimonas sp.
NWF50631.1
Ignavibacteriaceae bacterium
MBX3044688.1
Ignavibacteriota bacterium
MCA9293428.1
Phycisphaerales bacterium
NDV61934.1
Oceanipulchritudo coccoides
MBC8004591.1
Prolixibacteraceae bacterium
NVK56624.1
Alteromonadaceae bacterium
MBE1299347.1
Alteromonadaceae bacterium
MBE1300336.1
Alteromonadaceae bacterium
PRO73939.1
Alteromonas alba
HCL13382.1
Alteromonas sp.
WP_189420799.1
Cellvibrio zantedeschiae
WP_232769146.1
Colwellia sp. Bg11-28
WP_017444040.1
Gayadomonas joobiniege G7
WP_075593596.1
uncultured Pseudoalteromonas sp.
WP_105053508.1
Psychrosphaera saromensis
WP_116001002.1
Thalassotalea euphylliae
CDK41220.1
WP_074351599.1
Proteiniborus sp. DW1
WP_051725645.1
Bacillus gaemokensis
MBN2557589.1
Clostridia bacterium
MBN2883156.1
Clostridia bacterium
NLM42857.1
Clostridiales bacterium
WP_070109212.1
Clostridium acetireducens DSM 10703
WP_027634467.1
Clostridium hydrogeniformans DSM 21757
WP_221858439.1
Clostridium sardiniense
HEY69147.1
Anaerolineae bacterium
MCB8925539.1
Ardenticatenaceae bacterium
MBU0692857.1
bacterium
DAO23838.1
NJN64339.1
Acidobacteriota bacterium
MBM3290457.1
Candidatus Hydrogenedentota bacterium
MBN2891387.1
Bacteroidales bacterium
MBN2704618.1
Pontiellaceae bacterium
WP_126781118.1
Idiomarina ramblicola
WP_232149650.1
Stenotrophomonas sp. SY1
NJL11791.1
Microscillaceae bacterium
WP_235049483.1
Sphingobacterium paucimobilis HER1398
MBL8814451.1
Planctomyces sp.
WP_202920921.1
Urbifossiella limnaea
MBQ2993584.1
Alistipes sp.
MBR2428594.1
Alistipes sp.
WP_106634065.1
Alistipes sp. Marseille-P5061
NBJ08182.1
Alistipes sp. Z76
MBQ3554632.1
Bacteroidales bacterium
MBO7132591.1
Bacteroidales bacterium
OUN70004.1
Barnesiella sp. An55
HIU39496.1
Candidatus Limisoma intestinavium
MBP3298416.1
Muribaculaceae bacterium
MBR5469303.1
Paludibacteraceae bacterium
MBO5427553.1
Prevotella sp.
KIM07947.1
Sulfurovum sp. PC08-66
ODB83673.1
Candidatus Thiodiazotropha endoloripes
MBW9269713.1
Candidatus Thiodiazotropha sp. (ex. Lucinisca nassula)
NDD64191.1
Acidobacteriota bacterium
MBM3780869.1
Acidobacteriota bacterium
MBZ2186650.1
Bryobacter sp. CoA8 C33

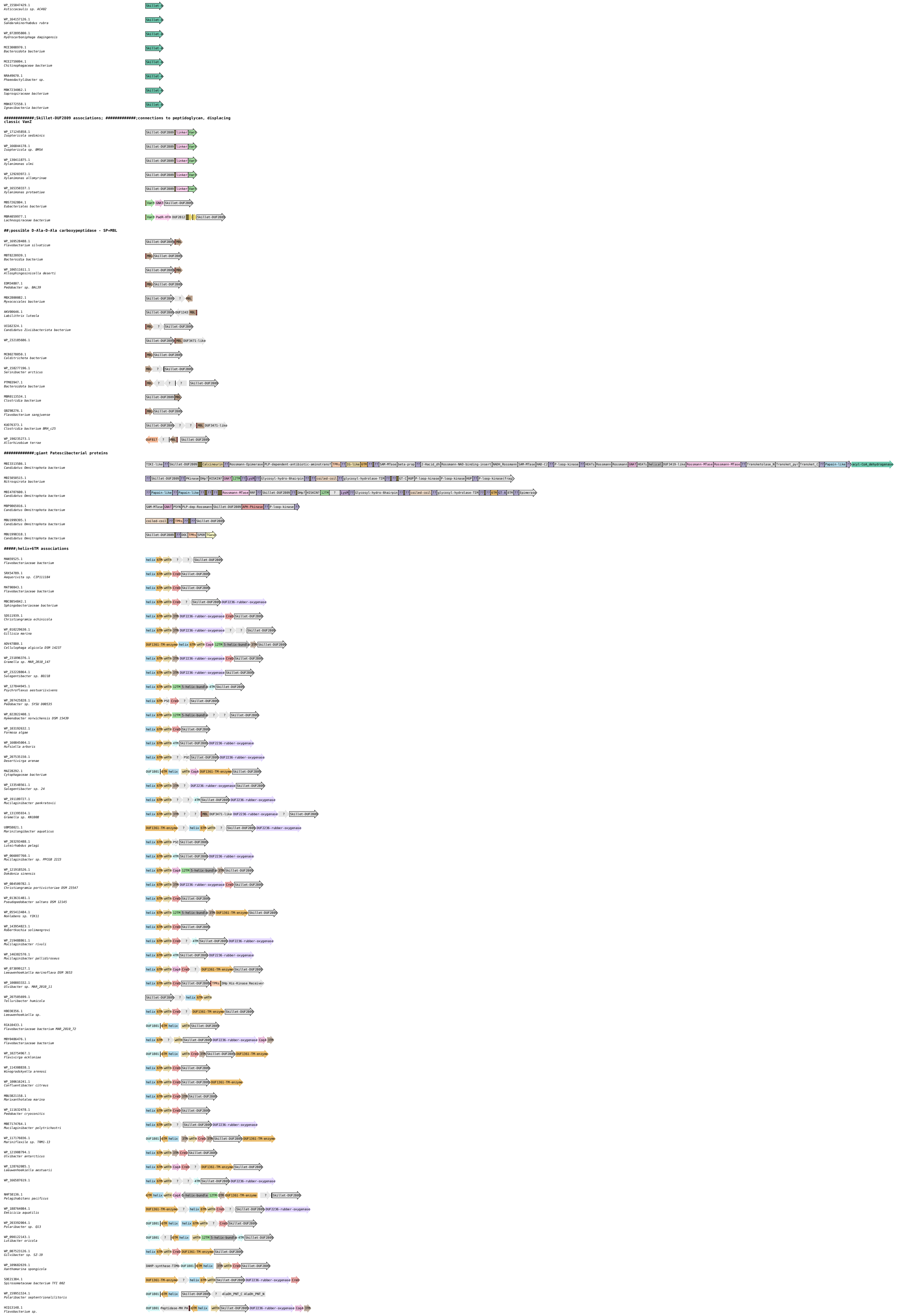
WP_155847429.1
Asticcacaulis sp. AC402
WP_164157126.1
Sandarakinorhabdus rubra
WP_072895800.1
Hydrocarboniphaga daqingensis
MCE3008970.1
Bacteroidota bacterium
MCE2759094.1
Chitinophagaceae bacterium
NRA49670.1
Phaeodactylibacter sp.
MBK7234062.1
Saprospiraceae bacterium
MBK6772558.1
Ignavibacteria bacterium
#############;Skillet-DUF2809 associations; #############;connections to peptidoglycan, displacing classic VanZ
WP_171245858.1
Isoptericola sediminis
WP_166844178.1
Isoptericola sp. BMS4
WP_130411875.1
Xylanimonas ulmi
WP_129203972.1
Xylanimonas allomyrinae
WP_165350337.1
Xylanimonas protaetiae
MBS7262804.1
Eubacteriales bacterium
MBR4059977.1
Lachnospiraceae bacterium
##;possible D-Ala-D-Ala carboxypeptidase - SP+MBL
WP_169528488.1
Flavobacterium silvaticum
MBT8220939.1
Bacteroidia bacterium
WP_106511611.1
Allosphingosinicella deserti
EDM34807.1
Pedobacter sp. BAL39
MBX2800082.1
Myxococcales bacterium
AKV00646.1
Labilithrix luteola
UCG62324.1
Candidatus Zixiibacteriota bacterium
WP_232185686.1
MCB0278050.1
Calditrichota bacterium
WP_158277196.1
Serinibacter arcticus
PTM03947.1
Bacteroidota bacterium
MBR0113534.1
Clostridia bacterium
QBZ98276.1
Flavobacterium sangjuense
KUO76373.1
Clostridia bacterium BRH_c25
WP_190235273.1
Allorhizobium terrae
#############;giant Patescibacterial proteins
MBI3313586.1
Candidatus Omnitrophota bacterium
MBI5050515.1
Nitrospirota bacterium
MBI4707600.1
Candidatus Omnitrophota bacterium
MBP9865016.1
Candidatus Omnitrophota bacterium
MBU1999395.1
Candidatus Omnitrophota bacterium
MBU1998318.1
Candidatus Omnitrophota bacterium
#####;helix+6TM associations
MAN59525.1
Flavobacteriaceae bacterium
SRX54789.1
Aequorivita sp. CIP111184
MAT90843.1
Flavobacteriaceae bacterium
MBC8054842.1
Sphingobacteriaceae bacterium
SDS11939.1
Christiangramia echinicola
WP_010229630.1
Gillisia marina
ADV47880.1
Cellulophaga algicola DSM 14237
WP_231896376.1
Gramella sp. MAR_2010_147
WP_232228864.1
Salegentibacter sp. BDJ18
WP_127844945.1
Psychroflexus aestuariivivens
WP_207425828.1
Pedobacter sp. SYSU D00535
WP_022822408.1
Hymenobacter norwichensis DSM 15439
WP_103192632.1
Formosa algae
WP_160845004.1
Hufsiella arboris
WP_207535150.1
Desertivirga arenae
MAZ28292.1
Cytophagaceae bacterium
WP_133548561.1
Salegentibacter sp. 24
WP_191189727.1
Mucilaginibacter pankratovii
WP_131395934.1
Gramella sp. KN1008
UBM58021.1
Marinilongibacter aquaticus
WP_203293488.1
Luteirhabdus pelagi
WP_066007760.1
Mucilaginibacter sp. PPCGB 2223
WP_121918526.1
Dokdonia sinensis
WP_084509782.1
Christiangramia portivictoriae DSM 23547
WP_013631481.1
Pseudopedobacter saltans DSM 12145
WP_055413484.1
Nonlabens sp. YIK11
WP_143954823.1
Robertkochia solimangrovi
WP_219488861.1
Mucilaginibacter rivuli
WP_146382570.1
Mucilaginibacter pallidiroseus
WP_073099127.1
Leeuwenhoekiella marinoflava DSM 3653
WP_100803332.1
Ulvibacter sp. MAR_2010_11
WP_207505699.1
Telluribacter humicola
HBO30356.1
Leeuwenhoekiella sp.
RIA10433.1
Flavobacteriaceae bacterium MAR_2010_72
MBY0486476.1
Flavobacteriaceae bacterium
WP_102754967.1
Flavivirga eckloniae
WP_114308838.1
Winogradskyella arenosi
WP_100616241.1
Confluentibacter citreus
MBU3821158.1
Marixanthotalea marina
WP_111632478.1
Pedobacter cryoconitis
MBE7174764.1
Mucilaginibacter polytrichastri
WP_117176036.1
Mariniflexile sp. TRM1-13
WP_121908794.1
Ulvibacter antarcticus
WP_128762085.1
Leeuwenhoekiella aestuarii
WP_166587619.1
NHF58136.1
Pelagihabitans pacificus
WP_188764084.1
Emticicia aquatilis
WP_203392004.1
Polaribacter sp. Q13
WP_090122143.1
Lutibacter oricola
WP_087523126.1
Gilvibacter sp. SZ-19
WP_109682639.1
Xanthomarina spongicola
SOE21384.1
Spirosomataceae bacterium TFI 002
WP_159951534.1
Polaribacter septentrionalilitoris
HCQ13148.1
Flavobacterium sp.

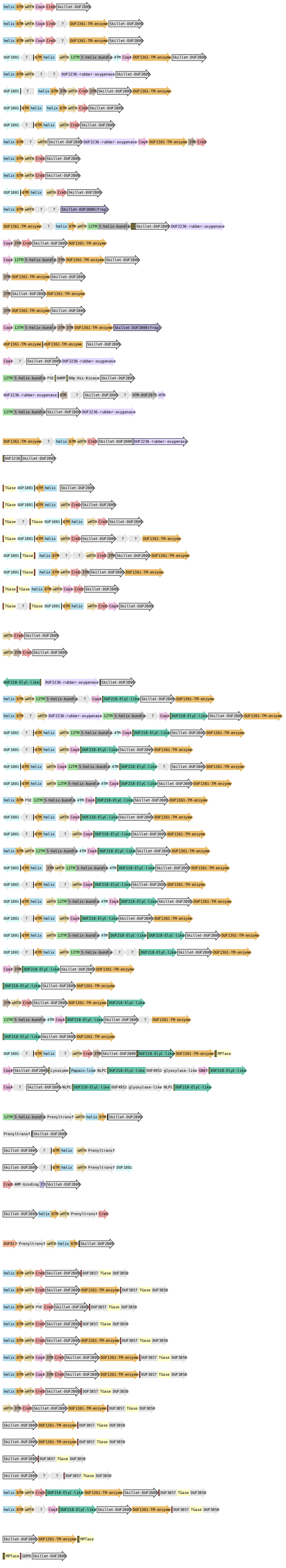

WP_105214366.1
Pukyongia salina

WP_099647089.1
Leeuwenhoekiella nanhaiensis

WP_228230976.1
Leeuwenhoekiella parthenopeia

WP_083265086.1
Urechidicola croceus

WP_029036417.1
Salinimicrobium xinjiangense DSM 19287

MBO6606124.1
Psychroserpens sp.

WP_197437746.1
Polaribacter sp. BAL334

WP_134247506.1
Lutibacter litoralis

MBN8642381.1
Flavobacteriales bacterium

WP_043706349.1
Tenacibaculum ovolyticum

WP_115880947.1
Lutibacter oceani

MCB0458632.1
Flavobacteriaceae bacterium

WP_160166673.1
Arcticibacter svalbardensis MN12-7

WP_229235540.1
Emticicia oligotrophica

WP_151674425.1
Patiriisocius marinus

WP_169933419.1
Nonlabens sp. Ci31

WP_036579470.1

WP_130963224.1
Hyunsoonleella flava

EAS19373.1
Flavobacteria bacterium BBFL7

MBV1925229.1
Dokdonia sp.

MBF4983086.1
Nonlabens mediterrranea

WP_166563521.1
Emticicia sp. CRIBPO

WP_222576626.1
Methylosinus sp. Sm6

WP_026981686.1
Flavobacterium suncheonense GH29-5 = DSM 17707

OYU92863.1
Bacteroidetes bacterium B1(2017)

#;direct fusion to DUF2236

WP_055147631.1
Jiulongibacter sediminis

MBU6121791.1
Hymenobacter siberiensis

#;with transglutaminase

RCS28202.1
Polaribacter sp. WD7

WP_115900388.1
Tenacibaculum gallaicum

GFD73882.1
Tenacibaculum sp. KUL113

WP_095073430.1
Tenacibaculum jejuense

WP_055443044.1
Lacinutrix himadriensis

WP_099562957.1

WP_093668771.1
Tenacibaculum sp. MAR_2009_124

WP_132725452.1
uncultured Tenacibaculum sp.

#;fragments?

WP_147136520.1
Seonamhaeicola algicola

NND64042.1
Flavobacteriaceae bacterium

#;DUF218-ElyC-like-containing

WP_226269223.1
Hymenobacter pini

WP_115817334.1
Winogradskyella eximia

WP_173281525.1
Winogradskyella eckloniae

WP_147766232.1
Seonamhaeicola maritimus

WP_116824243.1

WP_181364058.1
Algibacter marinivivus

WP_026753731.1
Sediminibacter sp. Hel_I_10

WP_204346419.1
Psychroserpens algicola

WP_106464338.1
Aurantibacter aestuarii

WP_145002676.1
Olleya sp.

WP_218700376.1
Olleya sp. HaHaR_3_96

MBR9914064.1
Algicola sp.

WP_099569870.1

WP_040251560.1
Psychroserpens mesophilus

WP_106678914.1
Mesoflavibacter zeaxanthinifaciens subsp. sabulilitoris

WP_148540230.1
Seonamhaeicola marinus

NQY28973.1
Flavobacteriaceae bacterium

WP_151895213.1
Patiriisocius marinistellae

NRA91944.1
Psychroserpens sp.

NQX85194.1
Flavobacteriaceae bacterium

WP_147088298.1
Subsaximicrobium wynnwilliamsii

WP_169673034.1
Flavivirga algicola

WP_132705360.1
Winogradskyella wandonensis

OQX16500.1
Thiothrix lacustris

WP_210219162.1
Thiothrix unzii

#;prenyltransferase

MBL8837034.1
Alphaproteobacteria bacterium

MBL0170627.1
Gemmatimonadaceae bacterium

MCA2990858.1
Gemmatimonas sp.

MBY0491654.1
Gemmatimonadaceae bacterium

MCD4796330.1
Candidatus Cloacimonadota bacterium

#;prenyltransferase+Abhydrolase

MBE7541481.1
Solibacteraceae bacterium

#;also with DUF817

MTK64597.1
Methanobacterium sp.

#;SP+DUF3857+TGase+DUF3858

MAO09402.1
Flavobacteriaceae bacterium

WP_186987819.1
Constantimarinum furrinae

MAP54607.1
Altibacter sp.

WP_093145158.1
Ulvibacter litoralis

WP_114492788.1
Candidatus Ulvibacter alkanivorans

QIE59100.1
Rasiella rasia

MBX2829544.1
Flavobacteriaceae bacterium

WP_214113909.1
Aequorivita echinoideorum

PHR11101.1
Aequorivita sp.

EDM43363.1
unidentified eubacterium SCB49

MCB0441901.1
Flavobacteriaceae bacterium

MBZ0328384.1
Altibacter sp.

NQY06666.1
Flavobacteriaceae bacterium

WP_068588187.1
Cochleicola gelatinilyticus

MAK34762.1
Flavobacteriaceae bacterium

#;TM+TM+MPTase

MBL86093.1
Winogradskyella sp.

WP_046573848.1
Spirosoma radiotolerans

#;fragments

Accession	Organism	Gene neighborhood
MCC8153085.1	*Tannerellaceae bacterium*	CreD, Skillet-DUF2809
MBU0697119.1	*Bacteroidota bacterium*	CreD, Skillet-DUF2809, DUF2236-rubber-oxygenase
OYY02236.1	*Sphingobacteriia bacterium 35-40-5*	CreD, Skillet-DUF2809, DUF2236-rubber-oxygenase
WP_127788831.1	*Rhodovarius crocodyli*	CreD, Skillet-DUF2809(frag)
WP_188627731.1	*Pedobacter quisquiliarum*	HD, CreD, Skillet-DUF2809
RYG64100.1	*bacterium*	DUF1361-TM-enzyme, Skillet-DUF2809
WP_085768013.1	*Nonlabens spongiae*	DUF1361-TM-enzyme, Skillet-DUF2809
WP_186558798.1	*Hyunsoonleella aquatilis*	Skillet-DUF2809, DUF1361-TM-enzyme
WP_124980077.1	*Nonlabens xiamenensis*	DUF1361-TM-enzyme, Skillet-DUF2809
OBY61920.1	*Polaribacter vadi*	Skillet-DUF2809, DUF1361-TM-enzyme
WP_034060023.1	*Lacinutrix jangbogonensis*	Skillet-DUF2809, DUF1361-TM-enzyme
WP_228850808.1		Skillet-DUF2809, DUF1361-TM-enzyme
WP_105015851.1	*Polaribacter porphyrae*	Skillet-DUF2809, DUF1361-TM-enzyme
WP_085774926.1	*Nonlabens tegetincola*	DUF1361-TM-enzyme, Skillet-DUF2809
WP_144886105.1	*Lacibacter cauensis*	Skillet-DUF2809, ?, 3TM

#########;Distinctive theme with 12TM+5-helix-bundle, also containing a HTH and 4TM+DUF2975 (also an apparently genuine HISKIN) or RADICAL_SAM

Accession	Organism	Gene neighborhood
WP_136496752.1		12TM 5-helix-bundle, ?, HAMP DHp His-Kinase, Skillet-DUF2809
GAU85619.1	*Bosea sp. BIWAKO-01*	12TM 5-helix-bundle, ?, HAMP DHp His-Kinase, Skillet-DUF2809
MBP2654676.1	*Bacillota bacterium*	4TM-DUF2975, HTH, 12TM 5-helix-bundle, Skillet-DUF2809
WP_110955433.1	*Massilibacillus massiliensis*	4TM-DUF2975, HTH, 12TM 5-helix-bundle, Skillet-DUF2809
MBD8472417.1	*Sphingomonas sp. CFBP 8765*	12TM 5-helix-bundle, Receiver OmpR-HTH, HAMP DHp His-Kinase, Skillet-DUF2809
MBE6847121.1	*Ruminococcus sp.*	4TM-DUF2975, HTH, 12TM 5-helix-bundle, Skillet-DUF2809
HBI85749.1	*Ruminococcus sp.*	4TM-DUF2975, HTH, ?, ?, ?, 12TM 5-helix-bundle, ?, ?, Skillet-DUF2809
MBO4878253.1	*Ruminococcus sp.*	4TM-DUF2975, HTH, 12TM 5-helix-bundle, Skillet-DUF2809
MBR6717568.1	*Oscillospiraceae bacterium*	HTH, 12TM 5-helix-bundle, ?, 4TM, ?, ?, ?, ?, Skillet-DUF2809
MBR3679290.1	*Oscillospiraceae bacterium*	4TM-DUF2975, HTH, 12TM 5-helix-bundle, ?, Skillet-DUF2809
WP_125995282.1	*Sphingomonas sp. S-NIH.Pt15_0812*	12TM 5-helix-bundle, ?, Cache DHp His-Kinase, Skillet-DUF2809
MBQ3842699.1	*Ruminiclostridium sp.*	4TM-DUF2975, HTH, 12TM 5-helix-bundle, Skillet-DUF2809
MBO5349147.1	*Clostridia bacterium*	4TM-DUF2975, HTH, 12TM 5-helix-bundle, Skillet-DUF2809
MBQ9126806.1	*Thermoguttaceae bacterium*	12TM 5-helix-bundle, Skillet-DUF2809
WP_088270951.1	*Enterococcus wangshanyuanii*	4TM-DUF2975, HTH, 12TM 5-helix-bundle, Skillet-DUF2809
MBQ6851574.1	*Oscillospiraceae bacterium*	4TM-DUF2975, HTH, ?, 12TM 5-helix-bundle, Skillet-DUF2809
MBQ2704859.1	*Clostridia bacterium*	Skillet-DUF2809, ?, ?, 4TM-DUF2975, HTH, 12TM 5-helix-bundle
MBR4022619.1	*Ruminococcus sp.*	4TM-DUF2975, HTH, Skillet-DUF2809, 12TM 5-helix-bundle
MBQ5712641.1	*Oscillospiraceae bacterium*	4TM-DUF2975, HTH, 12TM 5-helix-bundle, Skillet-DUF2809
MBQ8928159.1	*Oscillospiraceae bacterium*	4TM-DUF2975, HTH, Skillet-DUF2809, 12TM 5-helix-bundle
MBQ4600659.1	*Oscillospiraceae bacterium*	4TM-DUF2975, HTH, 12TM 5-helix-bundle, Skillet-DUF2809
HAP79347.1	*Ruminococcus sp.*	Skillet-DUF2809, 12TM 5-helix-bundle
WP_219749194.1		12TM 5-helix-bundle, ?, HAMP DHp His-Kinase, Skillet-DUF2809
WP_220712446.1		12TM 5-helix-bundle, ?, Skillet-DUF2809
ACB96860.1	*Beijerinckia indica subsp. indica ATCC 9039*	12TM 5-helix-bundle, ?, sCache HAMP DHp His-Kinase, Skillet-DUF2809
MBR3043307.1	*Oscillospiraceae bacterium*	12TM 5-helix-bundle, 4TM, ?, Skillet-DUF2809
WP_209680985.1	*Arthrobacter stackebrandtii*	HTH, 4TM-DUF2975, ?, Skillet-DUF2809
CDD37297.1	*Clostridium sp. CAG:356*	Skillet-DUF2809, 4TM-DUF2975, HTH
OKZ66078.1	*Clostridium sp. 28_12*	Skillet-DUF2809, 4TM-DUF2975, HTH
WP_094368234.1		4TM-DUF2975, Skillet-DUF2809
MBQ2061822.1	*Oscillospiraceae bacterium*	4TM-DUF2975, HTH, Skillet-DUF2809
MBQ9695918.1	*Oscillospiraceae bacterium*	4TM-DUF2975, HTH, Skillet-DUF2809
MBQ7354075.1	*Clostridia bacterium*	4TM-DUF2975, HTH, Skillet-DUF2809
WP_211233745.1	*Comamonas composti DSM 21721*	4TM-DUF2975, HTH, ?, Skillet-DUF2809
MBQ4466289.1	*Oscillospiraceae bacterium*	HTH, Skillet-DUF2809, 12TM 5-helix-bundle, ?, RADICAL-SAM Radical_SAM-C
MBQ7002763.1	*Oscillospiraceae bacterium*	4TM-DUF2975, HTH, Skillet-DUF2809 ??, 12TM 5-helix-bundle, RADICAL-SAM Radical_SAM-C
MBP0973621.1	*Oscillospiraceae bacterium*	4TM-DUF2975, HTH, Skillet-DUF2809, ?, 12TM 5-helix-bundle, ?, RADICAL-SAM Radical_SAM-C
MBR7037871.1	*Oscillospiraceae bacterium*	4TM-DUF2975, HTH, Skillet-DUF2809, 12TM 5-helix-bundle, RADICAL-SAM Radical_SAM-C
MBR2086672.1	*Oscillospiraceae bacterium*	4TM-DUF2975, HTH, Skillet-DUF2809, 12TM 5-helix-bundle, RADICAL-SAM Radical_SAM-C
MBR0484948.1	*Oscillospiraceae bacterium*	4TM-DUF2975, HTH, Skillet-DUF2809, ?, 12TM 5-helix-bundle, RADICAL-SAM Radical_SAM-C
MBQ9110345.1	*Oscillospiraceae bacterium*	4TM-DUF2975, HTH, ?, Skillet-DUF2809, 12TM 5-helix-bundle, RADICAL-SAM Radical_SAM-C
MBR4101559.1	*Oscillospiraceae bacterium*	Skillet-DUF2809 ??, ?, 12TM 5-helix-bundle, RADICAL-SAM Radical_SAM-C
WP_192127868.1	*Sphingomonas sp. CFBP 8760*	12TM 5-helix-bundle, ?, ?, Skillet-DUF2809

########;associations with some of the components in the systems above; #;DUF1801

Accession	Organism	Gene neighborhood
WP_127066474.1	*Cellulosilyticum sp. ST5*	HD, Skillet-DUF2809, ?, ?, DUF1801
MBB2877810.1		Skillet-DUF2809, ?, DUF1801, DUF1801
MBK7030302.1	*Bacteroidales bacterium*	Skillet-DUF2809, OmdA DUF1801
WP_221583400.1	*Microbacterium sp. G2-8*	Skillet-DUF2809, DUF1801
NLE75534.1	*Chloroflexota bacterium*	Skillet-DUF2809, DUF1801
WP_087055153.1	*Microbacterium sp. JB110*	Skillet-DUF2809, DUF1801
WP_019990722.1	*Rudanella lutea DSM 19387*	DUF1801 OmdA, Skillet-DUF2809
MCA9190426.1	*Planctomycetales bacterium*	Skillet-DUF2809, ?, DUF1801
OYY22639.1	*Sphingobacteriia bacterium 35-40-8*	DUF1801 OmdA, Skillet-DUF2809
MVQ41954.1	*Microbacterium sp. MAH-37*	DUF1801, Skillet-DUF2809
MCB0647256.1	*Saprospiraceae bacterium*	Skillet-DUF2809, ?, ?, DUF1801
RYG03498.1	*Chitinophagaceae bacterium*	Skillet-DUF2809, DUF1801
WP_210759870.1	*Croceimicrobium hydrocarbonivorans*	DUF1801 OmdA, Skillet-DUF2809
WP_143852656.1	*Chryseobacterium sp. SNU WT5*	Skillet-DUF2809, ?, ?, DUF1801
WP_128757520.1	*Leeuwenhoekiella aequorea*	DAHP-synthase-TIMb, DUF1801, ?, ?, ?, Skillet-DUF2809
MBS1833758.1	*Acidobacteriota bacterium*	HTH, ?, DUF1801, ?, ?, Skillet-DUF2809
WP_051216778.1	*Microbacterium gubbeenense DSM 15944*	Skillet-DUF2809, DUF1801
MBR10402.1	*Rickettsiales bacterium*	HTH, ?, ?, DUF1801 OmdA, DUF1801 OmdA, Skillet-DUF2809
RMZ49683.1	*Flavobacteriaceae bacterium PRS1*	DAHP-synthase-TIMb, DUF1801, Skillet-DUF2809
MCB9778560.1	*Alphaproteobacteria bacterium*	Skillet-DUF2809, ?, ?, ?, ?, ?, ?, DUF1801
WP_110926866.1		DUF1801, Skillet-DUF2809
RYY98001.1	*Alphaproteobacteria bacterium*	DUF1801, DUF1801, Skillet-DUF2809(frag)
WP_143372356.1	*Flavobacterium zepuense*	Skillet-DUF2809, UvrD-helicase UvrD_C, DUF1801

#;DUF218-ElyC-like

Accession	Organism	Gene neighborhood
MBL8206283.1	*Blastocatellia bacterium*	DUF218-ElyC-like, Skillet-DUF2809
MBE5952041.1	*Lachnospiraceae bacterium*	DUF218-ElyC-like, ?, Skillet-DUF2809
WP_208232769.1	*Gelidibacter pelagius*	DUF218-ElyC-like, Skillet-DUF2809
MBP3579575.1	*Lachnospiraceae bacterium*	DUF218-ElyC-like, NUDIX, Skillet-DUF2809
QMR43051.1	*Klebsiella aerogenes*	Skillet-DUF2809, ?, ?, ?, DUF218-ElyC-like
MBO5230066.1	*Clostridia bacterium*	DUF218-ElyC-like, ?, ?, ?, Skillet-DUF2809
WP_104734499.1	*Hanstruepera ponticola*	DUF218-ElyC-like, Skillet-DUF2809
MCB0434245.1	*Mangrovimonas sp.*	DUF218-ElyC-like, Skillet-DUF2809(frag)
MBR5272029.1	*Clostridia bacterium*	Skillet-DUF2809, Aldolase, ?, DUF218-ElyC-like

#;DUF2236-rubber-oxygenase form a two-gene core, with third DUF817 gene

Accession	Organism	Gene neighborhood
SDD92077.1	*Mucilaginibacter pineti*	Skillet-DUF2809 DUF2236-rubber-oxygenase DUF817
WP_172336793.1	*Mucilaginibacter sp. SG564*	Skillet-DUF2809 DUF2236-rubber-oxygenase DUF817
PTQ94107.1	*Mucilaginibacter yixingensis*	Skillet-DUF2809 DUF2236-rubber-oxygenase DUF817
WP_121199103.1	*Mucilaginibacter gracilis*	Skillet-DUF2809 DUF2236-rubber-oxygenase DUF817
NBP69763.1	*Cytophagia bacterium*	DUF817 Skillet-DUF2809 DUF2236-rubber-oxygenase

#;with 4TM

Accession	Organism	Gene neighborhood
RYD89130.1	*Sphingobacteriales bacterium*	4TM Skillet-DUF2809 DUF2236-rubber-oxygenase
RZL45319.1	*Pedobacter sp.*	4TM Skillet-DUF2809 DUF2236-rubber-oxygenase
WP_078349731.1	*Mucilaginibacter pedocola*	4TM Skillet-DUF2809-i DUF2236-rubber-oxygenase
WP_133231464.1	*Mucilaginibacter psychrotolerans*	4TM Skillet-DUF2809 DUF2236-rubber-oxygenase
WP_131553725.1	*Pedobacter frigiditerrae*	4TM Skillet-DUF2809 DUF2236-rubber-oxygenase
RZL36776.1	*Pedobacter sp.*	4TM Skillet-DUF2809 DUF2236-rubber-oxygenase

#;with 3TM

Accession	Organism	Gene neighborhood
WP_224469834.1	*Salegentibacter lacus*	3TM DUF2236-rubber-oxygenase Skillet-DUF2809 AlaDh_PNT_C AlaDh_PNT_N

#;3TM and SP+MBL

Accession	Organism	Gene neighborhood
MBX2970503.1	*Cyclobacteriaceae bacterium*	3TM Skillet-DUF2809 DUF2236-rubber-oxygenase ? MBL DUF3471-like

#;core associations only

Accession	Organism	Gene neighborhood
WP_230687479.1	*Hymenobacter ruricola*	DUF2236-rubber-oxygenase Skillet-DUF2809
WP_229598457.1	*Runella zeae DSM 19591*	Skillet-DUF2809 DUF2236-rubber-oxygenase
WP_119655303.1	*Hymenobacter rubripertinctus*	DUF2236-rubber-oxygenase Skillet-DUF2809
WP_106596867.1	*Dyadobacter jiangsuensis*	Skillet-DUF2809 DUF2236-rubber-oxygenase
WP_194112812.1	*Mucilaginibacter myungsuensis*	Skillet-DUF2809 DUF2236-rubber-oxygenase
WP_126692462.1	*Hymenobacter gummosus*	DUF2236-rubber-oxygenase Skillet-DUF2809
WP_208176818.1	*Hymenobacter negativus*	DUF2236-rubber-oxygenase Skillet-DUF2809
WP_130023256.1	*Emticicia agri*	Skillet-DUF2809 DUF2236-rubber-oxygenase
WP_129002810.1	*Filimonas effusa*	Skillet-DUF2809 DUF2236-rubber-oxygenase
MBN9384957.1	*Chitinophagaceae bacterium*	Skillet-DUF2809 DUF2236-rubber-oxygenase
WP_173811783.1	*Hymenobacter caeli*	DUF2236-rubber-oxygenase Skillet-DUF2809
RZJ49576.1	*Flavobacterium sp.*	Skillet-DUF2809 DUF2236-rubber-oxygenase
WP_188460546.1	*Marivirga lumbricoides*	Skillet-DUF2809 DUF2236-rubber-oxygenase
RZL06104.1	*Hymenobacter sp.*	DUF2236-rubber-oxygenase Skillet-DUF2809
WP_214073774.1	*Mucilaginibacter sp. dw_454*	Skillet-DUF2809 DUF2236-rubber-oxygenase
WP_171592441.1	*Hymenobacter sp. 5414T-23*	DUF2236-rubber-oxygenase Skillet-DUF2809
WP_035562422.1	*Hymenobacter sp. IS2118*	DUF2236-rubber-oxygenase Skillet-DUF2809
WP_100340755.1	*Mucilaginibacter auburnensis*	Skillet-DUF2809 4TM DUF2236-rubber-oxygenase
WP_147032090.1	*Mucilaginibacter ginsenosidivorans*	Skillet-DUF2809 DUF2236-rubber-oxygenase
WP_211365417.1	*Polyangium fumosum*	Skillet-DUF2809 DUF2236-rubber-oxygenase
AMR34030.1	*Mucilaginibacter sp. PAMC 26640*	Skillet-DUF2809 DUF2236-rubber-oxygenase
MBT1687090.1	*Dawidia soli*	Skillet-DUF2809 DUF2236-rubber-oxygenase
WP_184177983.1	*Rhabdobacter roseus*	Skillet-DUF2809 DUF2236-rubber-oxygenase
WP_114937235.1	*Mucilaginibacter endophyticus*	Skillet-DUF2809 DUF2236-rubber-oxygenase
WP_208308187.1		DUF2236-rubber-oxygenase Skillet-DUF2809
MCD9018791.1	*Parachryseolinea silvisoli*	Skillet-DUF2809 DUF2236-rubber-oxygenase
MBN8853370.1	*Sphingobacteriales bacterium*	Skillet-DUF2809 DUF2236-rubber-oxygenase
WP_013930438.1	*Runella slithyformis DSM 19594*	Skillet-DUF2809 DUF2236-rubber-oxygenase
WP_188928026.1	*Puia dinghuensis*	Skillet-DUF2809 DUF2236-rubber-oxygenase
MBT1695380.1	*Chryseosolibacter histidini*	Skillet-DUF2809 DUF2236-rubber-oxygenase
RPD48561.1	*Hymenobacter sediminis*	DUF2236-rubber-oxygenase Skillet-DUF2809
WP_173112402.1	*Hymenobacter sp. BRD128*	DUF2236-rubber-oxygenase Skillet-DUF2809
RVU00410.1	*Mucilaginibacter limnophilus*	Skillet-DUF2809 DUF2236-rubber-oxygenase
WP_232175575.1	*Mucilaginibacter roseus*	Skillet-DUF2809 DUF2236-rubber-oxygenase
WP_169529478.1	*Hymenobacter polaris*	DUF2236-rubber-oxygenase Skillet-DUF2809
WP_138475601.1	*Dyadobacter bucti*	Skillet-DUF2809 DUF2236-rubber-oxygenase
WP_160906657.1	*Hufsiella ginkgonis*	Skillet-DUF2809 DUF2236-rubber-oxygenase
WP_115831518.1	*Dyadobacter luteus*	Skillet-DUF2809 DUF2236-rubber-oxygenase
RYZ94082.1	*Sphingobacteriaceae bacterium*	Skillet-DUF2809 DUF2236-rubber-oxygenase
WP_208130676.1	*Hymenobacter ginsengisoli*	DUF2236-rubber-oxygenase Skillet-DUF2809
MBS1607064.1	*Bacteroidota bacterium*	Skillet-DUF2809 DUF2236-rubber-oxygenase
WP_196284882.1	*Hymenobacter properus*	DUF2236-rubber-oxygenase Skillet-DUF2809
MBL7878048.1	*Cyclobacteriaceae bacterium*	Skillet-DUF2809 DUF2236-rubber-oxygenase
WP_162549782.1	*Hymenobacter nivis*	DUF2236-rubber-oxygenase Skillet-DUF2809

#;DUF817 association

Accession	Organism	Gene neighborhood
GJE37023.1	*Methylobacterium persicinum*	DUF817 Skillet-DUF2809
WP_092773199.1	*Agrobacterium fabrum*	DUF817 Skillet-DUF2809
WP_233136590.1	*Agrobacterium vaccinii*	CN_hydrolase DUF817 Skillet-DUF2809
WP_192086682.1		Skillet-DUF2809 DUF817
GEL54271.1	*Asaia bogorensis NBRC 16594*	DUF817 Skillet-DUF2809
MBA3043257.1	*Rhizobiaceae bacterium*	DUF817 Skillet-DUF2809
WP_158625366.1	*Asaia sp. HN128*	DUF817 Skillet-DUF2809
NIE80765.1	*Asaia sp. As-1742*	DUF817 Skillet-DUF2809
WP_020809723.1	*Agrobacterium sp. TS43*	DUF817 Skillet-DUF2809
WP_112554634.1	*Agrobacterium sp. CMT1*	DUF817 Skillet-DUF2809
MWV24215.1	*Methylobacterium sp. 2A*	DUF817 Skillet-DUF2809
WP_103659155.1	*Agrobacterium rosae*	DUF817 Skillet-DUF2809
WP_182304365.1	*Ciceribacter thiooxidans*	DUF817 Skillet-DUF2809
WP_147761926.1	*Methylobacterium sp. WL103*	DUF817 Skillet-DUF2809
WP_174243197.1	*Beijerinckia sp. L45*	HTH PBPII DUF817 Skillet-DUF2809
WP_025886174.1	*Asaia prunellae JCM 25354*	DUF817 Skillet-DUF2809
WP_234891987.1	*Agrobacterium vitis*	DUF817 Skillet-DUF2809
MBB3290380.1	*Rhizobium sp. BK252*	DUF817 Skillet-DUF2809
WP_133315645.1	*Rhizobium deserti*	DUF817 Skillet-DUF2809
KQZ93159.1	*Rhizobium sp. Root564*	DUF817 Skillet-DUF2809
WP_208549440.1	*Ciceribacter sp. L1K22*	DUF817 Skillet-DUF2809
MBX9848088.1	*Rhodocyclaceae bacterium*	DUF817 Skillet-DUF2809
WP_025841327.1	*Asaia platycodi JCM 25414*	DUF817 Skillet-DUF2809
WP_203429027.1	*Rhizobium sp. BG4*	DUF817 Skillet-DUF2809
WP_144761880.1	*Methylobacterium dankookense*	DUF817 Skillet-DUF2809
TKD07832.1		DUF817 Skillet-DUF2809
WP_209943154.1	*Rhizobium halophytocola*	DUF817 Skillet-DUF2809
GGC39382.1	*Asaia siamensis*	DUF817 Skillet-DUF2809
QRG09493.1	*Xanthobacter dioxanivorans*	DUF817 Skillet-DUF2809
WP_182978329.1	*Gluconacetobacter asukensis*	DUF817 Skillet-DUF2809
MBI1203359.1	*Rhodopseudomonas sp.*	DUF817 Skillet-DUF2809
WP_126109462.1		DUF817 Skillet-DUF2809

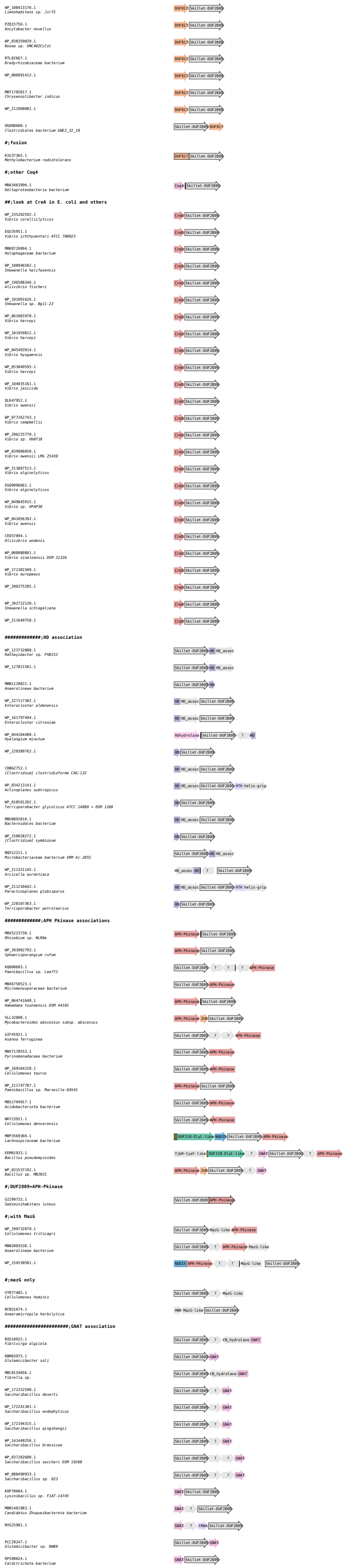
WP_108413176.1
Limnohabitans sp. Jir72
DUF817 Skillet-DUF2809
PZQ15756.1
Ancylobacter novellus
DUF817 Skillet-DUF2809
WP_038359029.1
Bosea sp. UNC402CLCol
DUF817 Skillet-DUF2809
RTL82667.1
Bradyrhizobiaceae bacterium
DUF817 Skillet-DUF2809
WP_086091412.1
DUF817 Skillet-DUF2809
MBT1705817.1
Chryseosolibacter indicus
DUF817 Skillet-DUF2809
WP_211968081.1
DUF817 Skillet-DUF2809
OGO88600.1
Clostridiales bacterium GWE2_32_10
Skillet-DUF2809 DUF817
#;fusion
KIU37365.1
Methylobacterium radiotolerans
DUF817 Skillet-DUF2809
#;other Coq4
MBA3463996.1
Deltaproteobacteria bacterium
Coq4 Skillet-DUF2809
##;look at CreA in E. coli and others
WP_235202592.1
Vibrio coralliilyticus
CreD Skillet-DUF2809
EGU35951.1
Vibrio ichthyoenteri ATCC 700023
CreD Skillet-DUF2809
MBK8726994.1
Holophagaceae bacterium
CreD Skillet-DUF2809
WP_108946502.1
Shewanella halifaxensis
CreD Skillet-DUF2809
WP_196588346.1
Aliivibrio fischeri
CreD Skillet-DUF2809
WP_101091626.1
Shewanella sp. Bg11-22
CreD Skillet-DUF2809
WP_061065978.1
Vibrio harveyi
CreD Skillet-DUF2809
WP_101959812.1
Vibrio harveyi
CreD Skillet-DUF2809
WP_045493914.1
Vibrio hyugaensis
CreD Skillet-DUF2809
WP_053049595.1
Vibrio harveyi
CreD Skillet-DUF2809
WP_104035161.1
Vibrio jasicida
CreD Skillet-DUF2809
QLK47952.1
Vibrio owensii
CreD Skillet-DUF2809
WP_077262743.1
Vibrio campbellii
CreD Skillet-DUF2809
WP_206225770.1
Vibrio sp. Vb0718
CreD Skillet-DUF2809
WP_039986050.1
Vibrio owensii LMG 25430
CreD Skillet-DUF2809
WP_213897513.1
Vibrio alginolyticus
CreD Skillet-DUF2809
EGQ9096661.1
Vibrio alginolyticus
CreD Skillet-DUF2809
WP_049845915.1
Vibrio sp. VPAP30
CreD Skillet-DUF2809
WP_041056392.1
Vibrio owensii
CreD Skillet-DUF2809
CED57894.1
Aliivibrio wodanis
CreD Skillet-DUF2809
WP_008080801.1
Vibrio sinaloensis DSM 21326
CreD Skillet-DUF2809
WP_171381509.1
Vibrio europaeus
CreD Skillet-DUF2809
WP_206375285.1
CreD Skillet-DUF2809
WP_202722120.1
Shewanella schlegeliana
CreD Skillet-DUF2809
WP_211649758.1
CreD Skillet-DUF2809
#############;HD association
WP_123732800.1
Rathayibacter sp. PhB152
Skillet-DUF2809 HD HD_assoc
WP_127821501.1
Skillet-DUF2809 HD HD_assoc
MBN1120831.1
Anaerolineae bacterium
Skillet-DUF2809 HD
WP_227117302.1
Enterocloster aldenensis
HD HD_assoc Skillet-DUF2809
WP_161797494.1
Enterocloster citroniae
HD HD_assoc Skillet-DUF2809
WP_044184480.1
Hyalangium minutum
Abhydrolase Skillet-DUF2809 ? HD
WP_228389762.1
HD Skillet-DUF2809
CDB62752.1
[Clostridium] clostridioforme CAG:132
HD HD_assoc Skillet-DUF2809
WP_034213141.1
Actinoplanes subtropicus
HD HD_assoc Skillet-DUF2809 HTH helix-grip
WP_018591202.1
Terrisporobacter glycolicus ATCC 14880 = DSM 1288
HD Skillet-DUF2809
MBU8893016.1
Bacteroidales bacterium
HD HD_assoc Skillet-DUF2809
WP_150028272.1
[Clostridium] symbiosum
HD Skillet-DUF2809
NQX12211.1
Microbacteriaceae bacterium VKM Ac-2855
Skillet-DUF2809 HD HD_assoc
WP_211321145.1
Arcicella aurantiaca
HD_assoc HD ? Skillet-DUF2809
WP_211216642.1
Paractinoplanes globisporus
HD HD_assoc Skillet-DUF2809 HTH helix-grip
WP_228107363.1
Terrisporobacter petrolearius
HD Skillet-DUF2809
#############;APH Pkinase associations
MBX5223730.1
Rhizobium sp. NLR8a
APH-Pkinase Skillet-DUF2809
WP_203982793.1
Sphaerisporangium rufum
APH-Pkinase Skillet-DUF2809
KQO00603.1
Paenibacillus sp. Leaf72
Skillet-DUF2809 ? ? ? APH-Pkinase
MBX6750523.1
Micromonosporaceae bacterium
Skillet-DUF2809 APH-Pkinase
WP_064741648.1
Hamadaea tsunoensis DSM 44101
APH-Pkinase Skillet-DUF2809
SLL32008.1
Mycobacteroides abscessus subsp. abscessus
APH-Pkinase ZnR Skillet-DUF2809
GIF45921.1
Asanoa ferruginea
Skillet-DUF2809 ? ? APH-Pkinase
MBX7170553.1
Pyrinomonadaceae bacterium
Skillet-DUF2809 APH-Pkinase
WP_169164228.1
Cellulomonas taurus
Skillet-DUF2809 APH-Pkinase
WP_211747787.1
Paenibacillus sp. Marseille-Q4541
APH-Pkinase Skillet-DUF2809
MBS1794917.1
Acidobacteriota bacterium
Skillet-DUF2809 APH-Pkinase
NKY23921.1
Cellulomonas denverensis
Skillet-DUF2809 APH-Pkinase
MBP3569369.1
Lachnospiraceae bacterium
DUF218-ElyC-like NUDIX Skillet-DUF2809 APH-Pkinase
EEM01933.1
Bacillus pseudomycoides
YjbR-CyaY-like DUF218-ElyC-like ? GNAT Skillet-DUF2809 ? APH-Pkinase
WP_031537192.1
Bacillus sp. MB2021
APH-Pkinase ZnR Skillet-DUF2809 ? GNAT
#;DUF2809+APH-Pkinase
GII99733.1
Sediminihabitans luteus
Skillet-DUF2809 APH-Pkinase
#;with MazG
WP_199732079.1
Cellulomonas triticagri
Skillet-DUF2809 MazG-like APH-Pkinase
MBN2003538.1
Anaerolineae bacterium
Skillet-DUF2809 ? APH-Pkinase MazG-like
WP_154530581.1
NUDIX APH-Pkinase ? ? MazG-like Skillet-DUF2809
#;mazG only
VTR77485.1
Cellulomonas hominis
Skillet-DUF2809 ? MazG-like
BCN31674.1
Anaeromicropila herbilytica
HNH MazG-like Skillet-DUF2809
#######################;GNAT association
NID10922.1
Fibrivirga algicola
Skillet-DUF2809 ? CN_hydrolase GNAT
RBM03975.1
Glutamicibacter soli
Skillet-DUF2809 GNAT
MBC8154056.1
Fibrella sp.
Skillet-DUF2809 CN_hydrolase GNAT
WP_172252590.1
Saccharibacillus deserti
Skillet-DUF2809 ? GNAT
WP_172241301.1
Saccharibacillus endophyticus
Skillet-DUF2809 ? GNAT
WP_172194315.1
Saccharibacillus qingshengii
Skillet-DUF2809 ? GNAT
WP_141449256.1
Saccharibacillus brassicae
Skillet-DUF2809 ? GNAT
WP_037282680.1
Saccharibacillus sacchari DSM 19268
Skillet-DUF2809 ? ? GNAT
WP_088490933.1
Saccharibacillus sp. O23
Skillet-DUF2809 ? ? GNAT
KOP78604.1
Lysinibacillus sp. FJAT-14745
GNAT Skillet-DUF2809
MBN1481983.1
Candidatus Zhuqueibacterota bacterium
GNAT ? Skillet-DUF2809
NYG25981.1
GNAT ? tRNA Skillet-DUF2809
PCC29247.1
Glutamicibacter sp. BW80
Skillet-DUF2809 GNAT
RPI00024.1
Calditrichota bacterium
GNAT Skillet-DUF2809

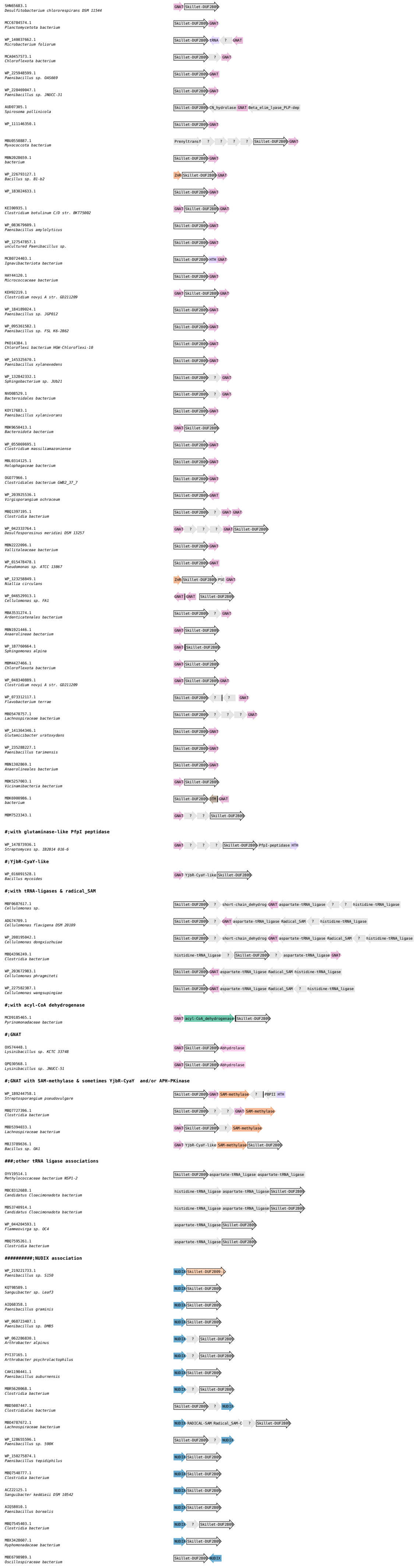
SHN65683.1
Desulfitobacterium chlororespirans DSM 11544
GNAT Skillet-DUF2809
MCC6784574.1
Planctomycetota bacterium
Skillet-DUF2809 GNAT
WP_140037662.1
Microbacterium foliorum
Skillet-DUF2809 tRNA ? GNAT
MCA0457573.1
Chloroflexota bacterium
Skillet-DUF2809 ? GNAT
WP_225948599.1
Paenibacillus sp. OAS669
Skillet-DUF2809 GNAT
WP_228469047.1
Paenibacillus sp. JNUCC-31
Skillet-DUF2809 GNAT
AUD07305.1
Spirosoma pollinicola
Skillet-DUF2809 CN_hydrolase GNAT Beta_elim_lyase_PLP-dep
WP_111146350.1
Skillet-DUF2809 GNAT
MBU0550887.1
Myxococcota bacterium
Prenyltransf ? ? ? ? Skillet-DUF2809 GNAT
MBN2028659.1
bacterium
Skillet-DUF2809 GNAT
WP_226793127.1
Bacillus sp. B1-b2
ZnR Skillet-DUF2809 GNAT
WP_183024633.1
Skillet-DUF2809 GNAT
KEI00935.1
Clostridium botulinum C/D str. BKT75002
GNAT Skillet-DUF2809 GNAT
WP_083679609.1
Paenibacillus amylolyticus
Skillet-DUF2809 GNAT
WP_127547857.1
uncultured Paenibacillus sp.
Skillet-DUF2809 GNAT
MCB0724403.1
Ignavibacteriota bacterium
Skillet-DUF2809 HTH GNAT
HAY44120.1
Micrococcaceae bacterium
Skillet-DUF2809 GNAT
KEH92219.1
Clostridium novyi A str. GD211209
GNAT Skillet-DUF2809 GNAT
WP_184189024.1
Paenibacillus sp. JGP012
Skillet-DUF2809 GNAT
WP_095361582.1
Paenibacillus sp. FSL K6-2862
Skillet-DUF2809 GNAT
PKO14384.1
Chloroflexi bacterium HGW-Chloroflexi-10
Skillet-DUF2809 GNAT
WP_145325670.1
Paenibacillus xylanexedens
Skillet-DUF2809 GNAT
WP_132842332.1
Sphingobacterium sp. JUb21
Skillet-DUF2809 ? GNAT
NVO08529.1
Bacteroidales bacterium
Skillet-DUF2809 ? GNAT
KOY17683.1
Paenibacillus xylanivorans
Skillet-DUF2809 GNAT
MBK9650413.1
Bacteroidota bacterium
GNAT Skillet-DUF2809
WP_055069695.1
Clostridium massiliamazoniense
Skillet-DUF2809 GNAT
MBL0314125.1
Holophagaceae bacterium
Skillet-DUF2809 GNAT
OGO77966.1
Clostridiales bacterium GWB2_37_7
Skillet-DUF2809 GNAT
WP_203925536.1
Virgisporangium ochraceum
Skillet-DUF2809 GNAT
MBQ1397195.1
Clostridia bacterium
Skillet-DUF2809 ? GNAT GNAT
WP_042333764.1
Desulfosporosinus meridiei DSM 13257
GNAT ? ? ? GNAT Skillet-DUF2809
MBN2222096.1
Vallitaleaceae bacterium
Skillet-DUF2809 GNAT
WP_015478478.1
Pseudomonas sp. ATCC 13867
Skillet-DUF2809 GNAT
WP_123258849.1
Niallia circulans
ZnR Skillet-DUF2809 PSE GNAT
WP_046529913.1
Cellulomonas sp. FA1
GNAT GNAT Skillet-DUF2809
MBA3531274.1
Ardenticatenales bacterium
Skillet-DUF2809 ? GNAT
MBN1921446.1
Anaerolineae bacterium
GNAT Skillet-DUF2809
WP_187760664.1
Sphingomonas alpina
GNAT Skillet-DUF2809
MBM4427466.1
Chloroflexota bacterium
GNAT Skillet-DUF2809
WP_048340889.1
Clostridium novyi A str. GD211209
GNAT Skillet-DUF2809 GNAT
WP_073312117.1
Flavobacterium terrae
Skillet-DUF2809 ? ? GNAT
MBO5470757.1
Lachnospiraceae bacterium
Skillet-DUF2809 ? ? ? GNAT
WP_141364346.1
Glutamicibacter uratoxydans
Skillet-DUF2809 GNAT
WP_235288227.1
Paenibacillus tarimensis
Skillet-DUF2809 GNAT
MBN1302869.1
Anaerolineales bacterium
Skillet-DUF2809 GNAT
MBK5257003.1
Vicinamibacteria bacterium
GNAT Skillet-DUF2809
MBK6900986.1
bacterium
Skillet-DUF2809 3TM GNAT
MBM7523343.1
GNAT ? ? Skillet-DUF2809
#;with glutaminase-like PfpI peptidase
WP_147873936.1
Streptomyces sp. IB2014 016-6
GNAT ? ? ? Skillet-DUF2809 PfpI-peptidase HTH
#;YjbR-CyaY-like
WP_016091528.1
Bacillus mycoides
GNAT YjbR-CyaY-like Skillet-DUF2809
#;with tRNA-ligases & radical_SAM
MBF0687617.1
Cellulomonas sp.
Skillet-DUF2809 ? short-chain_dehydrog GNAT aspartate-tRNA_ligase ? ? histidine-tRNA_ligase
ADG74709.1
Cellulomonas flavigena DSM 20109
Skillet-DUF2809 ? GNAT aspartate-tRNA_ligase Radical_SAM ? histidine-tRNA_ligase
WP_208195042.1
Cellulomonas dongxiuzhuiae
Skillet-DUF2809 ? short-chain_dehydrog GNAT aspartate-tRNA_ligase Radical_SAM ? histidine-tRNA_ligase
MBQ4396249.1
Clostridia bacterium
histidine-tRNA_ligase ? Skillet-DUF2809 ? aspartate-tRNA_ligase GNAT
WP_203672983.1
Cellulomonas phragmiteti
Skillet-DUF2809 GNAT aspartate-tRNA_ligase Radical_SAM histidine-tRNA_ligase
WP_227582387.1
Cellulomonas wangsupingiae
Skillet-DUF2809 GNAT aspartate-tRNA_ligase Radical_SAM ? histidine-tRNA_ligase
#;with acyl-CoA dehydrogenase
MCD9185465.1
Pyrinomonadaceae bacterium
GNAT acyl-CoA_dehydrogenase Skillet-DUF2809
#;GNAT
OXS74448.1
Lysinibacillus sp. KCTC 33748
GNAT Skillet-DUF2809 Abhydrolase
QPQ30568.1
Lysinibacillus sp. JNUCC-51
GNAT Skillet-DUF2809 Abhydrolase
#;GNAT with SAM-methylase & sometimes YjbR-CyaY and/or APH-PKinase
WP_189244758.1
Streptosporangium pseudovulgare
Skillet-DUF2809 GNAT SAM-methylase ? PBPII HTH
MBQ7727396.1
Clostridia bacterium
Skillet-DUF2809 ? ? GNAT SAM-methylase
MBD5394033.1
Lachnospiraceae bacterium
GNAT Skillet-DUF2809 ? SAM-methylase
MBJ3789636.1
Bacillus sp. OA1
GNAT YjbR-CyaY-like SAM-methylase Skillet-DUF2809
###;other tRNA ligase associations
OYV19514.1
Methylococcaceae bacterium NSP1-2
Skillet-DUF2809 aspartate-tRNA_ligase aspartate-tRNA_ligase
MBC8312688.1
Candidatus Cloacimonadota bacterium
histidine-tRNA_ligase aspartate-tRNA_ligase Skillet-DUF2809
MBS3740914.1
Candidatus Cloacimonadota bacterium
histidine-tRNA_ligase aspartate-tRNA_ligase Skillet-DUF2809
WP_044204593.1
Flammeovirga sp. OC4
aspartate-tRNA_ligase Skillet-DUF2809
MBQ7595261.1
Clostridia bacterium
aspartate-tRNA_ligase Skillet-DUF2809
##########;NUDIX association
WP_219221733.1
Paenibacillus sp. S150
NUDIX Skillet-DUF2809-1
KQT98589.1
Sanguibacter sp. Leaf3
NUDIX Skillet-DUF2809
AIQ68358.1
Paenibacillus graminis
NUDIX Skillet-DUF2809
WP_068723407.1
Paenibacillus sp. DMB5
NUDIX Skillet-DUF2809
WP_062286830.1
Arthrobacter alpinus
NUDIX ? Skillet-DUF2809
PYI37165.1
Arthrobacter psychrolactophilus
NUDIX ? Skillet-DUF2809
CAH1190441.1
Paenibacillus auburnensis
NUDIX Skillet-DUF2809
MBR5620068.1
Clostridia bacterium
NUDIX ? Skillet-DUF2809
MBD5087447.1
Clostridiales bacterium
Skillet-DUF2809 ? NUDIX
MBO4787672.1
Lachnospiraceae bacterium
NUDIX RADICAL-SAM Radical_SAM-C ? Skillet-DUF2809
WP_128655596.1
Paenibacillus sp. 598K
Skillet-DUF2809 ? NUDIX
WP_150275874.1
Paenibacillus tepidiphilus
NUDIX Skillet-DUF2809
MBQ7540777.1
Clostridia bacterium
NUDIX Skillet-DUF2809
ACZ22125.1
Sanguibacter keddieii DSM 10542
NUDIX Skillet-DUF2809
AIQ58010.1
Paenibacillus borealis
NUDIX Skillet-DUF2809
MBQ7545403.1
Clostridia bacterium
NUDIX ? Skillet-DUF2809
MBX3428607.1
Hyphomonadaceae bacterium
NUDIX Skillet-DUF2809
MBE6798989.1
Oscillospiraceae bacterium
Skillet-DUF2809 NUDIX

Accession	Organism	Gene neighborhood
HGG32052.1		NUDIX, Skillet-DUF2809
WP_009640938.1		NUDIX, Skillet-DUF2809
MBS1825775.1	*Acidobacteriota bacterium*	Skillet-DUF2809, ?, NUDIX
KWX88798.1	*Paenibacillus riograndensis*	NUDIX, Skillet-DUF2809(frag)
RKW14383.1	*Capnocytophaga sp.*	NUDIX, Skillet-DUF2809(frag)
MBL8160278.1	*Candidatus Saccharibacteria bacterium*	NUDIX, ?, ?, Skillet-DUF2809
MBI4038439.1	*Candidatus Daviesbacteria bacterium*	Skillet-DUF2809, ?, NUDIX

#;NUDIX fused to PIN (limited distribution)

Accession	Organism	Gene neighborhood
MBN9225431.1	*Microbacterium sp.*	NUDIX, PIN, Skillet-DUF2809
WP_229661574.1	*Microbacterium lacticum*	NUDIX, PIN, Skillet-DUF2809
WP_223878449.1	*Microbacterium radiodurans*	NUDIX, PIN, Skillet-DUF2809
OAH51998.1	*Microbacterium oleivorans*	NUDIX, PIN, Skillet-DUF2809
ODT40214.1	*Microbacterium sp. SCN 70-200*	NUDIX, PIN, Skillet-DUF2809
WP_112617607.1	*Microbacterium plantarum*	NUDIX, PIN, Skillet-DUF2809
RLK49724.1	*Microbacterium telephonicum*	NUDIX, PIN, Skillet-DUF2809
SBS74802.1	*uncultured Microbacterium sp.*	NUDIX, PIN, Skillet-DUF2809
WP_156366507.1	*Microbacterium sp. No. 7*	NUDIX, PIN, Skillet-DUF2809
KRA25812.1	*Microbacterium sp. Root61*	NUDIX, PIN, Skillet-DUF2809

#;DUF2809+NUDIX

Accession	Organism	Gene neighborhood
MBR3778706.1	*Clostridia bacterium*	Skillet-DUF2809, NUDIX

###########;SAM-methylase

Accession	Organism	Gene neighborhood
WP_161918043.1	*Cellulomonas sp. APG4*	Skillet-DUF2809, SAM-methylase
PST34888.1	*Enterocloster lavalensis*	Skillet-DUF2809, SAM-methylase
OGX11569.1	*Omnitrophica bacterium RIFOXYB12_FULL_50_7*	Skillet-DUF2809, ?, PSE, ?, SAM-methylase
WP_230684911.1	*Cellulomonas sp. JZ18*	Skillet-DUF2809, SAM-methylase
WP_233404439.1	*Actinotalea solisilvae*	Skillet-DUF2809, SAM-methylase
WP_222928251.1	*Cellulomonas telluris*	Skillet-DUF2809, SAM-methylase
MBK9503922.1	*Bacteroidota bacterium*	Skillet-DUF2809, ?, ?, SAM-methylase
WP_182112712.1	*Actinotalea sp. JY-7885*	Skillet-DUF2809, SAM-methylase
TFH64889.1	*Candidatus Zixiibacteriota bacterium*	Skillet-DUF2809, SAM-methylase
MBI9049120.1	*Anaerolineaceae bacterium*	Skillet-DUF2809, ?, ?, ?, Skillet-DUF2809, SAM-methylase
WP_077350224.1	*Tessaracoccus flavescens*	SAM-methylase, Skillet-DUF2809
MBN8878954.1	*Sphingobacteriales bacterium*	SAM-methylase, Skillet-DUF2809
NOQ40148.1	*Anaerolineales bacterium*	SAM-methylase, ?, Skillet-DUF2809
WP_194846558.1	*Mumia zhuanghuii*	SAM-methylase, Skillet-DUF2809
WP_234794990.1	*Hymenobacter arizonensis*	Skillet-DUF2809, SAM-methylase

#;with glutaminase-like PfpI peptidase

Accession	Organism	Gene neighborhood
WP_052098919.1	*uncultured Blautia sp.*	PfpI-peptidase, ?, PSE, SAM-methylase, Skillet-DUF2809

#;with YjbR-CyaY

Accession	Organism	Gene neighborhood
AIE79744.1	*Bacillus cereus*	YjbR-CyaY-like, SAM-methylase, Skillet-DUF2809

###;only YjbR-CyaY

Accession	Organism	Gene neighborhood
HCL02006.1	*Lachnoclostridium phytofermentans*	YjbR-CyaY-like, Skillet-DUF2809
WP_093247829.1	*Tessaracoccus oleiagri*	YjbR-CyaY-like, ?, ?, Skillet-DUF2809
WP_079423289.1	*Clostridium oryzae*	YjbR-CyaY-like, Skillet-DUF2809(frag)
WP_025414103.1	*Gemmatirosa kalamazoonensis*	YjbR-CyaY-like, Skillet-DUF2809

##########;Abhydrolase

Accession	Organism	Gene neighborhood
WP_008963824.1	*Bradyrhizobium sp. STM 3809*	Skillet-DUF2809, ?, Abhydrolase
WP_082642255.1		Abhydrolase, ?, Skillet-DUF2809
WP_116536568.1	*Microbacterium testaceum*	Abhydrolase, ?, Skillet-DUF2809
OZD55655.1	*Rhodococcus sp. 06-1477-1B*	Abhydrolase, ?, Skillet-DUF2809
WP_203975028.1	*Planotetraspora silvatica*	Abhydrolase, Skillet-DUF2809
WP_111490920.1		Abhydrolase, ?, Skillet-DUF2809
WP_013583228.1	*Microbacterium testaceum StLB037*	Abhydrolase, ?, Skillet-DUF2809
WP_112527963.1	*Phyllobacterium sp. T1293*	Skillet-DUF2809, Abhydrolase
WP_058622198.1	*Microbacterium testaceum*	Abhydrolase, ?, Skillet-DUF2809
WP_055835439.1	*Microbacterium sp. Leaf436*	Abhydrolase, ?, Skillet-DUF2809
HHX62735.1	*Candidatus Epulonipiscium sp.*	Skillet-DUF2809, ?, Abhydrolase
MBL6949557.1	*Bacteroidales bacterium*	Abhydrolase, Skillet-DUF2809
WP_082509351.1	*Microbacterium sp. Leaf179*	Abhydrolase, ?, Skillet-DUF2809
WP_230401736.1		Skillet-DUF2809, Abhydrolase
MBC7774050.1	*Saprospiraceae bacterium*	Abhydrolase, Skillet-DUF2809
WP_197140720.1	*Lysinibacillus sphaericus*	Skillet-DUF2809, Abhydrolase
MCB0635049.1	*Lewinella sp.*	Abhydrolase, ?, Skillet-DUF2809
WP_138177924.1		Abhydrolase, ?, Skillet-DUF2809
WP_228170885.1	*Mucilaginibacter daejeonensis*	Skillet-DUF2809, Abhydrolase
WP_158437685.1	*Naasia lichenicola*	Abhydrolase, Skillet-DUF2809
NJR70374.1	*Synechococcales cyanobacterium CRU_2_2*	Skillet-DUF2809, Abhydrolase
WP_167455984.1	*Buttiauxella izardii*	Skillet-DUF2809, ?, Abhydrolase
MCB0856203.1	*Bacteroidota bacterium*	Skillet-DUF2809, ?, Abhydrolase
MBN1326911.1	*Candidatus Cloacimonadota bacterium*	Abhydrolase, Skillet-DUF2809(frag)
MBI2022818.1	*Candidatus Daviesbacteria bacterium*	Skillet-DUF2809, ?, ?, Abhydrolase

#;with possible acyl--CoA ligase

Accession	Organism	Gene neighborhood
WP_184791569.1	*Phytomonospora endophytica*	Abhydrolase, AMP-binding, AMP-binding_C, ?, ?, Skillet-DUF2809

##############;possible acyl--CoA ligase association (AMP-binding+AMP-binding_C)

Accession	Organism	Gene neighborhood
MBN2031142.1	*bacterium*	Skillet-DUF2809, 1,4-dihydroxy-2-naphthoate_polyprenyltransferase
WP_017711501.1	*Prochlorothrix hollandica PCC 9006 = CALU 1027*	Skillet-DUF2809, ACAS_N, AMP-binding, AMP-binding_C
WP_165314301.1	*Agromyces protaetiae*	AMP-binding, AMP-binding_C, ?, PSE, PSE, ?, Skillet-DUF2809
MBR6800934.1	*Eubacteriaceae bacterium*	Skillet-DUF2809, ?, ?, ?, AMP-binding_C, AMP-binding
MBK9096615.1	*bacterium*	AMP-binding_C, AMP-binding, ?, ?, ?, ?, Skillet-DUF2809
WP_211331170.1	*Flavobacterium endophyticum*	Skillet-DUF2809, AMP-binding, ??
WP_182492162.1	*Flavobacterium gossypii*	Skillet-DUF2809, AMP-binding, ??
WP_100281091.1	*Sphingomonas psychrotolerans*	Skillet-DUF2809, AMP-binding_C, AMP-binding
WP_077508952.1	*Sphingomonas sp. LM7*	Skillet-DUF2809, ?, AMP-binding_C, AMP-binding
WP_221227354.1	*Stakelama sediminis*	Skillet-DUF2809, ??, AMP-binding

#############;possible enoyl-CoA hydratase/isomerase association (ENOYCOADEHYD)

Accession	Organism	Gene neighborhood
MBI5824488.1	*Chloroflexota bacterium*	Chorismate_bind, ?, ENOYCOADEHYD, Skillet-DUF2809, AMP-binding, AMP-binding_C, DAHP-synthase-TIMb, Abhydrolase, 1,4-dihydroxy-2-naphthoate_polyprenyltransferase
WP_074701130.1		ENOYCOADEHYD, AMP-binding, AMP-binding_C, 1,4-dihydroxy-2-naphthoate_polyprenyltransferase, ?, ?, Skillet-DUF2809, ?, ?, ?, ?, ?, beta-barrel
MBO1223911.1	*Candidatus Scalindua sediminis*	ENOYCOADEHYD, ?, ?, Skillet-DUF2809
WP_198979064.1	*Hymenobacter sp. BT523*	4HBT, Chorismate_bind, ?, ENOYCOADEHYD, Skillet-DUF2809
MBI3967005.1	*Chloroflexota bacterium*	ENOYCOADEHYD, ENOYCOADEHYD, ENOYCOADEHYD, ?, ENOYCOADEHYD, Skillet-DUF2809
MCB0823430.1	*Bacteroidales bacterium*	Skillet-DUF2809, ?, 4HBT, Chorismate_bind, ?, ENOYCOADEHYD, 1,4-dihydroxy-2-naphthoate_polyprenyltransferase
MCB9553808.1	*Myxococcales bacterium*	Skillet-DUF2809, ?, ENOYCOADEHYD
RAJ91569.1	*Streptomyces sp. PsTaAH-137*	Skillet-DUF2809, ENOYCOADEHYD
WP_020598087.1	*Spirosoma panaciterrae DSM 21099*	ENOYCOADEHYD, ?, ?, Skillet-DUF2809

#############;glycerophosphodiester phosphodiesterase; ##;here it could act on the glycerol phosphate or in conjunction with it? strange coupling as the GDPD is predicted to remove ethanolamine, etc. from glycerol-phosphate.

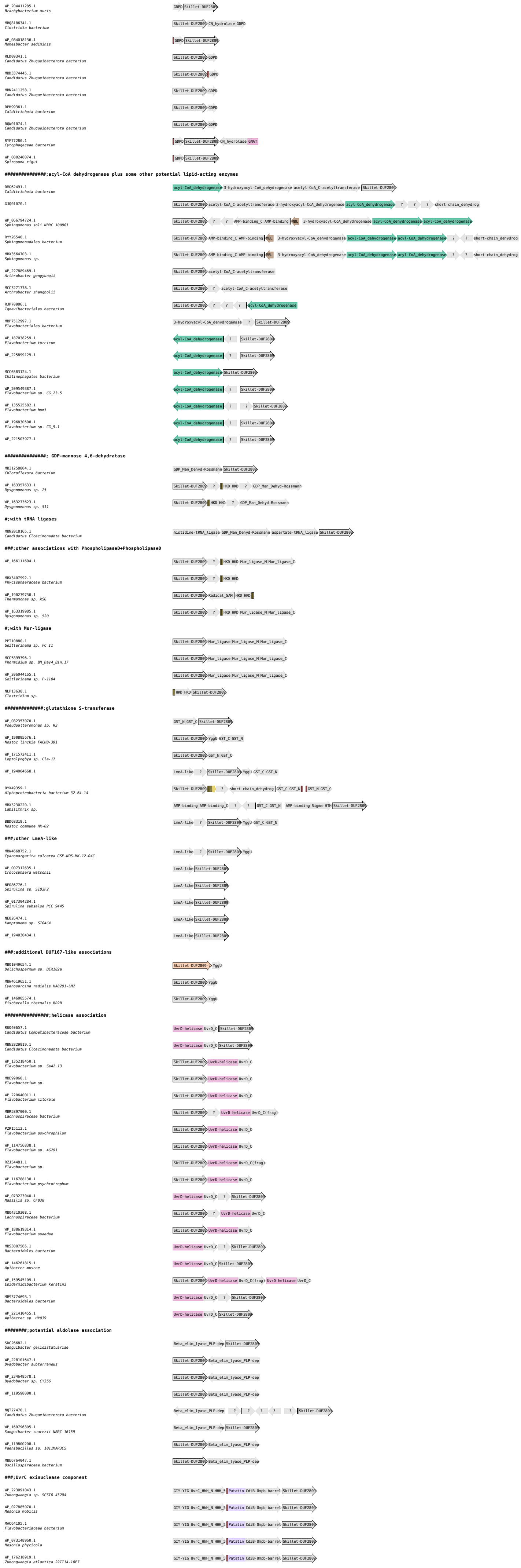
WP_204411285.1 Brachybacterium muris
GDPD Skillet-DUF2809
MBQ8186341.1 Clostridia bacterium
Skillet-DUF2809 CN_hydrolase GDPD
WP_084018136.1 Moheibacter sediminis
GDPD Skillet-DUF2809
RLD09341.1 Candidatus Zhuqueibacterota bacterium
Skillet-DUF2809 GDPD
MBD3374445.1 Candidatus Zhuqueibacterota bacterium
Skillet-DUF2809 GDPD
MBN2411258.1 Candidatus Zhuqueibacterota bacterium
Skillet-DUF2809 GDPD
RPH99361.1 Calditrichota bacterium
Skillet-DUF2809 GDPD
RQW01074.1 Candidatus Zhuqueibacterota bacterium
Skillet-DUF2809 GDPD
RYF77280.1 Cytophagaceae bacterium
GDPD Skillet-DUF2809 CN_hydrolase GNAT
WP_080240074.1 Spirosoma rigui
GDPD Skillet-DUF2809
###############;acyl-CoA dehydrogenase plus some other potential lipid-acting enzymes
RMG62491.1 Calditrichota bacterium
acyl-CoA_dehydrogenase 3-hydroxyacyl-CoA_dehydrogenase acetyl-CoA_C-acetyltransferase Skillet-DUF2809
GJQ01070.1
Skillet-DUF2809 acetyl-CoA_C-acetyltransferase 3-hydroxyacyl-CoA_dehydrogenase acyl-CoA_dehydrogenase ? ? ? short-chain_dehydrog
WP_066794724.1 Sphingomonas soli NBRC 100801
Skillet-DUF2809 ? ? AMP-binding_C AMP-binding MBL 3-hydroxyacyl-CoA_dehydrogenase acyl-CoA_dehydrogenase acyl-CoA_dehydrogenase
RYY26540.1 Sphingomonadales bacterium
Skillet-DUF2809 AMP-binding_C AMP-binding MBL 3-hydroxyacyl-CoA_dehydrogenase acyl-CoA_dehydrogenase acyl-CoA_dehydrogenase ? ? short-chain_dehydrog
MBX3564703.1 Sphingomonas sp.
Skillet-DUF2809 AMP-binding_C AMP-binding MBL 3-hydroxyacyl-CoA_dehydrogenase acyl-CoA_dehydrogenase acyl-CoA_dehydrogenase ? ? short-chain_dehydrog
WP_227889469.1 Arthrobacter gengyunqii
Skillet-DUF2809 acetyl-CoA_C-acetyltransferase
MCC3271778.1 Arthrobacter zhangbolii
Skillet-DUF2809 ? acetyl-CoA_C-acetyltransferase
RJP70906.1 Ignavibacteriales bacterium
Skillet-DUF2809 ? ? ? acyl-CoA_dehydrogenase
MBP7512997.1 Flavobacteriales bacterium
3-hydroxyacyl-CoA_dehydrogenase ? Skillet-DUF2809
WP_187038259.1 Flavobacterium turcicum
acyl-CoA_dehydrogenase ? Skillet-DUF2809
WP_225899129.1
acyl-CoA_dehydrogenase ? Skillet-DUF2809
MCC6583124.1 Chitinophagales bacterium
acyl-CoA_dehydrogenase Skillet-DUF2809
WP_209549387.1 Flavobacterium sp. CG_23.5
acyl-CoA_dehydrogenase ? Skillet-DUF2809
WP_135525582.1 Flavobacterium humi
acyl-CoA_dehydrogenase ? ? Skillet-DUF2809
WP_196830508.1 Flavobacterium sp. CG_9.1
acyl-CoA_dehydrogenase ? Skillet-DUF2809
WP_221503977.1
acyl-CoA_dehydrogenase ? Skillet-DUF2809
###############; GDP-mannose 4,6-dehydratase
MBI1258804.1 Chloroflexota bacterium
GDP_Man_Dehyd-Rossmann Skillet-DUF2809
WP_163357633.1 Dysgonomonas sp. 25
Skillet-DUF2809 ? HKD HKD ? GDP_Man_Dehyd-Rossmann
WP_163273623.1 Dysgonomonas sp. 511
Skillet-DUF2809 HKD HKD ? GDP_Man_Dehyd-Rossmann
#;with tRNA ligases
MBN2018165.1 Candidatus Cloacimonadota bacterium
histidine-tRNA_ligase GDP_Man_Dehyd-Rossmann aspartate-tRNA_ligase Skillet-DUF2809
###;other associations with PhospholipaseD+PhospholipaseD
WP_166111604.1
Skillet-DUF2809 ? HKD HKD Mur_ligase_M Mur_ligase_C
MBX3407992.1 Phycisphaeraceae bacterium
Skillet-DUF2809 ? HKD HKD
WP_190279730.1 Thermomonas sp. XSG
Skillet-DUF2809 Radical_SAM HKD HKD
WP_163319985.1 Dysgonomonas sp. 520
Skillet-DUF2809 ? HKD HKD Mur_ligase_M Mur_ligase_C
#;with Mur-ligase
PPT10880.1 Geitlerinema sp. FC II
Skillet-DUF2809 Mur_ligase Mur_ligase_M Mur_ligase_C
MCC5899396.1 Phormidium sp. BM_Day4_Bin.17
Skillet-DUF2809 Mur_ligase Mur_ligase_M Mur_ligase_C
WP_206044165.1 Geitlerinema sp. P-1104
Skillet-DUF2809 Mur_ligase Mur_ligase_M Mur_ligase_C
NLP13638.1 Clostridium sp.
HKD HKD Skillet-DUF2809
##############;glutathione S-transferase
WP_082353070.1 Pseudoalteromonas sp. R3
GST_N GST_C Skillet-DUF2809
WP_190895676.1 Nostoc linckia FACHB-391
Skillet-DUF2809 YggU GST_C GST_N
WP_171572411.1 Leptolyngbya sp. Cla-17
Skillet-DUF2809 GST_N GST_C
WP_194004668.1
LmeA-like ? Skillet-DUF2809 YggU GST_C GST_N
OYX49359.1 Alphaproteobacteria bacterium 32-64-14
Skillet-DUF2809 ? short-chain_dehydrog GST_C GST_N GST_N GST_C
MBX3230220.1 Labilithrix sp.
AMP-binding AMP-binding_C ? ? GST_C GST_N AMP-binding Sigma-HTH Skillet-DUF2809
BBD68319.1 Nostoc commune HK-02
LmeA-like ? Skillet-DUF2809 YggU GST_C GST_N
###;other LmeA-like
MBW4668752.1 Cyanomargarita calcarea GSE-NOS-MK-12-04C
LmeA-like ? Skillet-DUF2809 YggU
WP_007312635.1 Crocosphaera watsonii
LmeA-like Skillet-DUF2809
NEO86776.1 Spirulina sp. SIO3F2
LmeA-like Skillet-DUF2809
WP_017304284.1 Spirulina subsalsa PCC 9445
LmeA-like Skillet-DUF2809
NEO26474.1 Kamptonema sp. SIO4C4
LmeA-like Skillet-DUF2809
WP_194030434.1
LmeA-like Skillet-DUF2809
###;additional DUF167-like associations
MBO1049654.1 Dolichospermum sp. DEX182a
Skillet-DUF2809-i YggU
MBW4619651.1 Cyanosarcina radialis HA8281-LM2
Skillet-DUF2809 YggU
WP_146005574.1 Fischerella thermalis BR2B
Skillet-DUF2809 YggU
################;helicase association
RUQ40657.1 Candidatus Competibacteraceae bacterium
UvrD-helicase UvrD_C Skillet-DUF2809
MBN2829919.1 Candidatus Cloacimonadota bacterium
UvrD-helicase UvrD_C Skillet-DUF2809
WP_135218450.1 Flavobacterium sp. SaA2.13
Skillet-DUF2809 UvrD-helicase UvrD_C
MBE99060.1 Flavobacterium sp.
Skillet-DUF2809 UvrD-helicase UvrD_C
WP_220640011.1 Flavobacterium litorale
Skillet-DUF2809 UvrD-helicase UvrD_C
MBR5897000.1 Lachnospiraceae bacterium
Skillet-DUF2809 ? UvrD-helicase UvrD_C(frag)
PZR15112.1 Flavobacterium psychrophilum
Skillet-DUF2809 UvrD-helicase UvrD_C
WP_114756838.1 Flavobacterium sp. AG291
Skillet-DUF2809 UvrD-helicase UvrD_C
RZJ54481.1 Flavobacterium sp.
Skillet-DUF2809 UvrD-helicase UvrD_C(frag)
WP_116788138.1 Flavobacterium psychrotrophum
Skillet-DUF2809 UvrD-helicase UvrD_C
WP_073223040.1 Massilia sp. CF038
UvrD-helicase UvrD_C ? Skillet-DUF2809
MBO4310308.1 Lachnospiraceae bacterium
Skillet-DUF2809 ? UvrD-helicase UvrD_C
WP_188619314.1 Flavobacterium suaedae
Skillet-DUF2809 UvrD-helicase UvrD_C
MBS3807565.1 Bacteroidales bacterium
UvrD-helicase UvrD_C ? Skillet-DUF2809
WP_146261815.1 Apibacter muscae
UvrD-helicase UvrD_C Skillet-DUF2809
WP_159545109.1 Epidermidibacterium keratini
Skillet-DUF2809 UvrD-helicase UvrD_C(frag) UvrD-helicase UvrD_C
MBS3774693.1 Bacteroidales bacterium
UvrD-helicase UvrD_C ? Skillet-DUF2809
WP_221410455.1 Apibacter sp. HY039
UvrD-helicase UvrD_C Skillet-DUF2809
########;potential aldolase association
SDC26682.1 Sanguibacter gelidistatuariae
Beta_elim_lyase_PLP-dep Skillet-DUF2809
WP_228101647.1 Dyadobacter subterraneus
Skillet-DUF2809 Beta_elim_lyase_PLP-dep
WP_234648578.1 Dyadobacter sp. CY356
Skillet-DUF2809 Beta_elim_lyase_PLP-dep
WP_119598000.1
Skillet-DUF2809 Beta_elim_lyase_PLP-dep
NQT27470.1 Candidatus Zhuqueibacterota bacterium
Beta_elim_lyase_PLP-dep ? ? ? ? ? Skillet-DUF2809
WP_169796305.1 Sanguibacter suarezii NBRC 16159
Beta_elim_lyase_PLP-dep Skillet-DUF2809
WP_119800208.1 Paenibacillus sp. 1011MAR3C5
Skillet-DUF2809 Beta_elim_lyase_PLP-dep
MBE6764047.1 Oscillospiraceae bacterium
Skillet-DUF2809 Beta_elim_lyase_PLP-dep
###;UvrC exinuclease component
WP_223091043.1 Zunongwangia sp. SCSIO 43204
GIY-YIG UvrC_HhH_N HHH_5 Patatin CdiB-Ompb-barrel Skillet-DUF2809
WP_027885070.1 Mesonia mobilis
GIY-YIG UvrC_HhH_N HHH_5 Patatin CdiB-Ompb-barrel Skillet-DUF2809
MAC64105.1 Flavobacteriaceae bacterium
GIY-YIG UvrC_HhH_N HHH_5 Patatin CdiB-Ompb-barrel Skillet-DUF2809
WP_073148960.1 Mesonia phycicola
GIY-YIG UvrC_HhH_N HHH_5 Patatin CdiB-Ompb-barrel Skillet-DUF2809
WP_176218919.1 Zunongwangia atlantica 22II14-10F7
GIY-YIG UvrC_HhH_N HHH_5 Patatin CdiB-Ompb-barrel Skillet-DUF2809

Accession	Organism	Domains
WP_219038619.1	*Mesonia aestuariivivens*	GIY-YIG UvrC_HhH_N HHH_5 Patatin CdiB-Ompb-barrel Skillet-DUF2809
MBT3606256.1	*Candidatus Latescibacterota bacterium*	Skillet-DUF2809 ? ? CdiB-Ompb-barrel Patatin

##############;possible peptidase associations

Accession	Organism	Domains
WP_205086079.1		acyl-CoA_dehydrogenase ? ? ? PfpI-peptidase HTH Skillet-DUF2809
WP_202418091.1		short-chain_dehydrog acyl-CoA_dehydrogenase ? ? ? HTH PfpI-peptidase ? Skillet-DUF2809
SBU99983.1	*Streptomyces sp. MnatMP-M77*	short-chain_dehydrog acyl-CoA_dehydrogenase ? ? ? HTH PfpI-peptidase ? Skillet-DUF2809
RYJ24499.1	*Streptomyces sp. L-9-10*	Skillet-DUF2809 PfpI-peptidase HTH
WP_084895883.1	*Streptomyces sp. CB03238*	Skillet-DUF2809 PfpI-peptidase HTH
NRQ31527.1	*Nonomuraea antri*	Skillet-DUF2809 ? PfpI-peptidase
WP_175595156.1	*Nonomuraea montanisoli*	Skillet-DUF2809 PfpI-peptidase
HBD63717.1	*Clostridiales bacterium*	Skillet-DUF2809 PfpI-peptidase
WP_219912012.1	*Nonomuraea fuscirosea*	Skillet-DUF2809 ? short-chain_dehydrog PSE PfpI-peptidase
PZG21250.1	*Nonomuraea aridisoli*	Skillet-DUF2809 PfpI-peptidase
WP_181616191.1	*Nonomuraea soli*	Skillet-DUF2809 PSE PfpI-peptidase
WP_222871144.1	*Nonomuraea sp. PA05*	Skillet-DUF2809 PSE PfpI-peptidase
WP_192779386.1	*Nonomuraea africana*	Skillet-DUF2809 PSE PfpI-peptidase
MBQ9768867.1	*Clostridia bacterium*	Skillet-DUF2809 ? ? PfpI-peptidase

#;DUF2809+PfpI-peptidase

Accession	Organism	Domains
WP_180903365.1		Skillet-DUF2809 PfpI-peptidase

#;MPTase

Accession	Organism	Domains
SMC46658.1	*Oscillospiraceae bacterium*	Skillet-DUF2809 MPTase
MBO4635937.1	*Clostridiales bacterium*	Skillet-DUF2809 MPTase
MBP5492626.1	*Clostridiales bacterium*	Skillet-DUF2809 MPTase
MBQ8950464.1	*Eubacterium sp.*	Skillet-DUF2809 MPTase
WP_078765238.1	*Eubacterium uniforme*	Skillet-DUF2809 MPTase
MBO7355706.1	*Lachnospiraceae bacterium*	Skillet-DUF2809 MPTase
SEW20056.1	*Ruminococcaceae bacterium KH2T8*	Skillet-DUF2809 MPTase
MBQ0012941.1	*Candidatus Scatonaster coprocaballi*	Skillet-DUF2809 MPTase

#;Papain-like-peptidase fusion

Accession	Organism	Domains
MBQ8398058.1	*Clostridia bacterium*	Skillet-DUF2809 Papain-like-peptidase

#############;PLP-dependent possible aminotransferase fused to HTH transcriptional regulator

Accession	Organism	Domains
MBO5213962.1	*Clostridia bacterium*	HTH PLP-d Skillet-DUF2809
PTS91548.1	*Pedobacter sp. HMWF019*	Skillet-DUF2809 PLP-d HTH ? His-Kinase DHp TPRs
CEJ15011.1	*bacterium YEK0313*	Skillet-DUF2809 PLP-d HTH
WP_108869164.1	*Aquimarina aquimarini*	Skillet-DUF2809 PLP-d HTH
WP_025740339.1	*Aquimarina pacifica*	Skillet-DUF2809 PLP-d HTH ? ? ? ? AlaDh_PNT_C AlaDh_PNT_N
MBO5248764.1	*Clostridia bacterium*	HTH PLP-d Skillet-DUF2809
WP_222536778.1	*Pedobacter gandavensis*	Skillet-DUF2809 PLP-d HTH
EKY12161.1	*Capnocytophaga sp. oral taxon 332 str. F0381*	Skillet-DUF2809 PLP-d
MBC8183670.1	*Candidatus Zhuqueibacterota bacterium*	Skillet-DUF2809 PLP-d
MBS5081508.1	*Clostridiales bacterium*	PLP-d ? Skillet-DUF2809
WP_213531582.1	*Paenibacillus sp. GYB006*	PLP-d Skillet-DUF2809

#############;other rare fusions

Accession	Organism	Domains
MBQ7821214.1	*Clostridia bacterium*	Skillet-DUF2809 7TMR Glyco-hydro-IG Glyco-hydro-TIM
WP_109027756.1	*Methylosinus sporium*	12TM 5-helix-bundle ? HAMP DHp His-Kinase Skillet-DUF2809 Glyco-hydro-TIM
WP_153540271.1	*Actinomadura macrotermitis*	short-chain_dehydrog ? ? ? DUF2809 SUKH HEATs
WP_223227812.1	*Campylobacter concisus*	Skillet-DUF2809 ANKs
WP_132848672.1	*Serpentinicella alkaliphila*	Skillet-DUF2809 Rossmann-dehydrogenase
MBQ6570306.1	*Clostridia bacterium*	PLP-d ? ? ? Skillet-DUF2809 Cytidylate_kin
MCC6994969.1	*Deltaproteobacteria bacterium*	Skillet-DUF2809 DUF1990
WP_231921355.1	*Micromonospora auratinigra*	Skillet-DUF2809 HU HU
NLX06208.1	*Phycisphaerae bacterium*	Skillet-DUF2809(frag) REase
MBO5450336.1	*Lachnospiraceae bacterium*	His-Kinase Skillet-DUF2809
WP_129227915.1	*Lichenibacterium minor*	sCache HAMP DHp His-Kinase Skillet-DUF2809
RZJ33412.1	*Flavobacterium sp.*	Skillet-DUF2809 PepSY-like

###########;standalone

Accession	Organism	Domains
HAH18296.1	*Eubacterium sp.*	Skillet-DUF2809-i
HFA50449.1	*Bacteroidota bacterium*	Skillet-DUF2809-i
KAB2908868.1	*Ignavibacteriaceae bacterium*	Skillet-DUF2809-i
MBE0583637.1	*Desulfofustis sp.*	Skillet-DUF2809-i
MBK6912096.1	*bacterium*	Skillet-DUF2809-i
MBN2020309.1	*Sedimentisphaerales bacterium*	Skillet-DUF2809-i
MBP6628591.1	*Kofleriaceae bacterium*	Skillet-DUF2809-i
MBR1443724.1	*Bacillota bacterium*	Skillet-DUF2809-i
MBT8361120.1	*Deltaproteobacteria bacterium*	Skillet-DUF2809-i
NES83530.1	*Moorena sp. SIO2B7*	Skillet-DUF2809-i
NNK13361.1	*Desulfofustis sp.*	Skillet-DUF2809-i
PSQ81324.1	*Bacteroidetes bacterium QS_1_63_11*	Skillet-DUF2809-i
QIH33709.1	*Sphingobacterium sp. DR205*	Skillet-DUF2809-i
QUW95925.1	*Streptomyces sp. V17-9*	Skillet-DUF2809-i
RKY09755.1	*Planctomycetota bacterium*	Skillet-DUF2809-i
WP_118221172.1	*Bacteroides intestinalis*	Skillet-DUF2809-i
WP_205233186.1	*Desulfopila inferna*	Skillet-DUF2809-i
WP_216464061.1	*Clostridium bornimense*	Skillet-DUF2809-i
WP_207071220.1	*[Muricauda] lutisoli*	Skillet-DUF2809
WP_210509162.1	*Naasia sp. SYSU D00057*	Skillet-DUF2809
OYX52743.1	*Sphingomonas sp. 32-66-10*	Skillet-DUF2809
WP_216324463.1	*Sphingomonas quercus*	Skillet-DUF2809
WP_184189063.1	*Chryseobacterium defluvii*	Skillet-DUF2809
WP_007844512.1	*Chryseobacterium populi*	Skillet-DUF2809
WP_111928098.1	*Clostridium tertium*	Skillet-DUF2809
WP_167509236.1	*Pseudomonas oryzihabitans*	Skillet-DUF2809
KAA3611053.1	*Calditrichota bacterium*	Skillet-DUF2809
MBP9716978.1	*Candidatus Levyibacteriota bacterium*	Skillet-DUF2809
MBE7073288.1	*Oscillospiraceae bacterium*	Skillet-DUF2809
SDB48407.1	*Flavobacteriaceae bacterium MAR_2010_188*	Skillet-DUF2809
WP_019388807.1	*Algibacter luteus*	Skillet-DUF2809
WP_073242717.1	*Maribacter aquivivus*	Skillet-DUF2809
WP_041494927.1	*Nonlabens marinus S1-08*	Skillet-DUF2809
WP_090751613.1	*Nonlabens sp. Hel1_33_55*	Skillet-DUF2809
WP_105050413.1	*Polaribacter butkevichii*	Skillet-DUF2809
WP_218761508.1	*Polaribacter sp. AHE13PA*	Skillet-DUF2809
WP_058103729.1		Skillet-DUF2809
SNR45987.1	*Lutibacter agarilyticus*	Skillet-DUF2809
HBQ31823.1	*Brucella anthropi*	Skillet-DUF2809

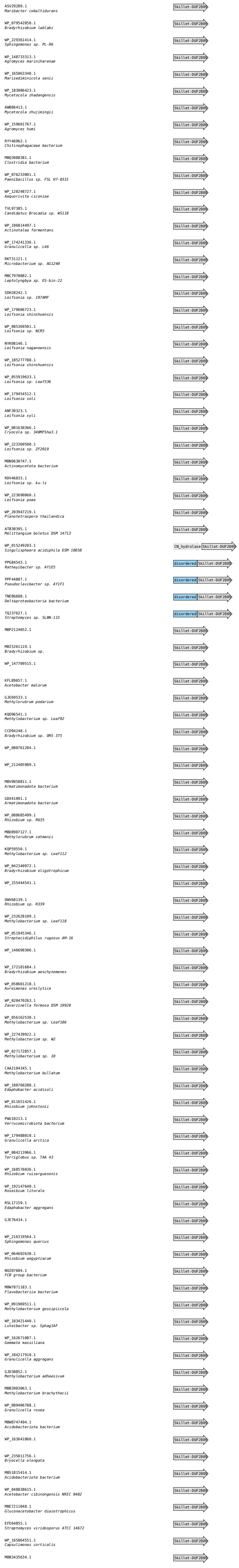

ASV29289.1
Maribacter cobaltidurans
Skillet-DUF2809
WP_079542858.1
Bradyrhizobium lablabi
Skillet-DUF2809
WP_229361414.1
Sphingomonas sp. PL-96
Skillet-DUF2809
WP_148733313.1
Agromyces mariniharenae
Skillet-DUF2809
WP_165063340.1
Marisediminicola senii
Skillet-DUF2809
WP_183086423.1
Mycetocola zhadangensis
Skillet-DUF2809
AWB86413.1
Mycetocola zhujimingii
Skillet-DUF2809
WP_159601767.1
Agromyces humi
Skillet-DUF2809
RYY46962.1
Chitinophagaceae bacterium
Skillet-DUF2809
MBQ3088381.1
Clostridia bacterium
Skillet-DUF2809
WP_076233001.1
Paenibacillus sp. FSL H7-0331
Skillet-DUF2809
WP_128248727.1
Aequorivita ciconiae
Skillet-DUF2809
TVL97385.1
Candidatus Brocadia sp. WS118
Skillet-DUF2809
WP_186814497.1
Actinotalea fermentans
Skillet-DUF2809
WP_174241336.1
Granulicella sp. L46
Skillet-DUF2809
RKT31121.1
Microbacterium sp. AG1240
Skillet-DUF2809
MBC7970082.1
Leptolyngbya sp. ES-bin-22
Skillet-DUF2809
SDH28242.1
Leifsonia sp. 197AMF
Skillet-DUF2809
WP_179606723.1
Leifsonia shinshuensis
Skillet-DUF2809
WP_085369501.1
Leifsonia sp. NCR5
Skillet-DUF2809
NYK08146.1
Leifsonia naganoensis
Skillet-DUF2809
WP_185277788.1
Leifsonia shinshuensis
Skillet-DUF2809
WP_055919633.1
Leifsonia sp. Leaf336
Skillet-DUF2809
WP_179454512.1
Leifsonia soli
Skillet-DUF2809
ANF30323.1
Leifsonia xyli
Skillet-DUF2809
WP_081630366.1
Cryocola sp. 340MFSha3.1
Skillet-DUF2809
WP_223360500.1
Leifsonia sp. ZF2019
Skillet-DUF2809
MBN9630747.1
Actinomycetota bacterium
Skillet-DUF2809
RDV46833.1
Leifsonia sp. ku-ls
Skillet-DUF2809
WP_223690060.1
Leifsonia poae
Skillet-DUF2809
WP_203947219.1
Planotetraspora thailandica
Skillet-DUF2809
ATB30395.1
Melittangium boletus DSM 14713
Skillet-DUF2809
WP_015249203.1
Singulisphaera acidiphila DSM 18658
CN_hydrolase
Skillet-DUF2809
PPG84543.1
Rathayibacter sp. AY1E5
disordered
Skillet-DUF2809
PPF44807.1
Pseudoclavibacter sp. AY1F1
disordered
Skillet-DUF2809
TNE86608.1
Deltaproteobacteria bacterium
disordered
Skillet-DUF2809
TQJ37627.1
Streptomyces sp. SLBN-115
disordered
Skillet-DUF2809
MBP2124052.1
Skillet-DUF2809
MBI5261119.1
Bradyrhizobium sp.
Skillet-DUF2809
WP_147709515.1
Skillet-DUF2809
KFL89657.1
Acetobacter malorum
Skillet-DUF2809
GJE69533.1
Methylorubrum podarium
Skillet-DUF2809
KQO96541.1
Methylobacterium sp. Leaf92
Skillet-DUF2809
CCD94248.1
Bradyrhizobium sp. ORS 375
Skillet-DUF2809
WP_080761204.1
Skillet-DUF2809
WP_212405989.1
Skillet-DUF2809
MBV9850811.1
Armatimonadota bacterium
Skillet-DUF2809
GDX41091.1
Armatimonadota bacterium
Skillet-DUF2809
WP_088685499.1
Rhizobium sp. R635
Skillet-DUF2809
MBD8907127.1
Methylorubrum zatmanii
Skillet-DUF2809
KQP59550.1
Methylobacterium sp. Leaf112
Skillet-DUF2809
WP_042340972.1
Bradyrhizobium oligotrophicum
Skillet-DUF2809
WP_155444541.1
Skillet-DUF2809
OWV68139.1
Rhizobium sp. R339
Skillet-DUF2809
WP_232628109.1
Methylobacterium sp. Leaf118
Skillet-DUF2809
WP_051945346.1
Streptacidiphilus rugosus AM-16
Skillet-DUF2809
WP_146690306.1
Skillet-DUF2809
WP_172181684.1
Bradyrhizobium aeschynomenes
Skillet-DUF2809
WP_058601218.1
Aureimonas ureilytica
Skillet-DUF2809
WP_020470263.1
Zavarzinella formosa DSM 19928
Skillet-DUF2809
WP_056162530.1
Methylobacterium sp. Leaf106
Skillet-DUF2809
WP_227439922.1
Methylobacterium sp. W2
Skillet-DUF2809
WP_027172857.1
Methylobacterium sp. 10
Skillet-DUF2809
CAA2104345.1
Methylobacterium bullatum
Skillet-DUF2809
WP_188760288.1
Edaphobacter acidisoli
Skillet-DUF2809
WP_011651426.1
Rhizobium johnstonii
Skillet-DUF2809
PWU10213.1
Verrucomicrobiota bacterium
Skillet-DUF2809
WP_179488010.1
Granulicella arctica
Skillet-DUF2809
WP_084213966.1
Terriglobus sp. TAA 43
Skillet-DUF2809
WP_168576036.1
Rhizobium ruizarguesonis
Skillet-DUF2809
WP_192147640.1
Roseibium litorale
Skillet-DUF2809
RSL17159.1
Edaphobacter aggregans
Skillet-DUF2809
GJE76434.1
Skillet-DUF2809
WP_216319564.1
Sphingomonas quercus
Skillet-DUF2809
WP_064692638.1
Rhizobium aegyptiacum
Skillet-DUF2809
NOZ07604.1
FCB group bacterium
Skillet-DUF2809
MBW7871183.1
Flavobacteriia bacterium
Skillet-DUF2809
WP_091969511.1
Methylobacterium gossipiicola
Skillet-DUF2809
WP_183421440.1
Luteibacter sp. Sphag1AF
Skillet-DUF2809
WP_162671087.1
Gemmata massiliana
Skillet-DUF2809
WP_184217910.1
Granulicella aggregans
Skillet-DUF2809
GJD30852.1
Methylobacterium adhaesivum
Skillet-DUF2809
MBB3903063.1
Methylobacterium brachythecii
Skillet-DUF2809
WP_089406788.1
Granulicella rosea
Skillet-DUF2809
MBW8747494.1
Acidobacteriota bacterium
Skillet-DUF2809
WP_163641860.1
Skillet-DUF2809
WP_235011756.1
Bryocella elongata
Skillet-DUF2809
MBS1815414.1
Acidobacteriota bacterium
Skillet-DUF2809
WP_048838615.1
Acetobacter cibinongensis NRIC 0482
Skillet-DUF2809
MBE7211048.1
Gluconacetobacter diazotrophicus
Skillet-DUF2809
EFE64855.1
Streptomyces viridosporus ATCC 14672
Skillet-DUF2809
WP_165864551.1
Capsulimonas corticalis
Skillet-DUF2809
MBB3435634.1
Skillet-DUF2809

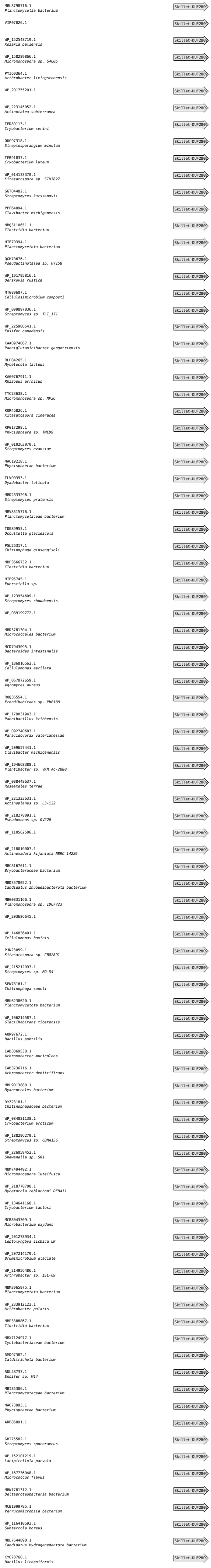
MBL8798716.1
Planctomycetia bacterium
Skillet-DUF2809
VIP07026.1
Skillet-DUF2809
WP_152548719.1
Kozakia baliensis
Skillet-DUF2809
WP_158289866.1
Micromonospora sp. S4605
Skillet-DUF2809
PYI69364.1
Arthrobacter livingstonensis
Skillet-DUF2809
WP_201755201.1
Skillet-DUF2809
WP_223145052.1
Actinotalea subterranea
Skillet-DUF2809
TFD89113.1
Cryobacterium serini
Skillet-DUF2809
OUC97318.1
Streptosporangium minutum
Skillet-DUF2809
TFB91837.1
Cryobacterium luteum
Skillet-DUF2809
WP_014133370.1
Kitasatospora sp. SID7827
Skillet-DUF2809
GGT04482.1
Streptomyces kurssanovii
Skillet-DUF2809
PPF64094.1
Clavibacter michiganensis
Skillet-DUF2809
MBQ3110651.1
Clostridia bacterium
Skillet-DUF2809
HIE70394.1
Planctomycetota bacterium
Skillet-DUF2809
QGH70676.1
Pseudactinotalea sp. HY158
Skillet-DUF2809
WP_191795816.1
Oerskovia rustica
Skillet-DUF2809
MTG89607.1
Cellulosimicrobium composti
Skillet-DUF2809
WP_099897036.1
Streptomyces sp. TLI_171
Skillet-DUF2809
WP_225906541.1
Ensifer canadensis
Skillet-DUF2809
KAA0974067.1
Paeniglutamicibacter gangotriensis
Skillet-DUF2809
RLP84265.1
Mycetocola lacteus
Skillet-DUF2809
KAG0767911.1
Rhizopus arrhizus
Skillet-DUF2809
TYC23638.1
Micromonospora sp. MP36
Skillet-DUF2809
ROR46826.1
Kitasatospora cineracea
Skillet-DUF2809
RPG17298.1
Phycisphaera sp. TMED9
Skillet-DUF2809
WP_010263970.1
Streptomyces evansiae
Skillet-DUF2809
MAC19218.1
Phycisphaerae bacterium
Skillet-DUF2809
TLV00393.1
Dyadobacter luticola
Skillet-DUF2809
MBD2833296.1
Streptomyces pratensis
Skillet-DUF2809
MBV8315776.1
Planctomycetaceae bacterium
Skillet-DUF2809
TDE89953.1
Occultella glacieicola
Skillet-DUF2809
PSL26317.1
Chitinophaga ginsengisoli
Skillet-DUF2809
MBP3666732.1
Clostridia bacterium
Skillet-DUF2809
HIE95745.1
Fuerstiella sp.
Skillet-DUF2809
WP_123954609.1
Streptomyces showdoensis
Skillet-DUF2809
WP_089199772.1
Skillet-DUF2809
MBD3781304.1
Micrococcales bacterium
Skillet-DUF2809
MCD7943095.1
Bacteroides intestinalis
Skillet-DUF2809
WP_186816562.1
Cellulomonas aerilata
Skillet-DUF2809
WP_067872659.1
Agromyces aureus
Skillet-DUF2809
ROQ36554.1
Frondihabitans sp. PhB188
Skillet-DUF2809
WP_179031943.1
Paenibacillus kribbensis
Skillet-DUF2809
WP_092740683.1
Paracidovorax valerianellae
Skillet-DUF2809
WP_209657441.1
Clavibacter michiganensis
Skillet-DUF2809
WP_194668388.1
Plantibacter sp. VKM Ac-2880
Skillet-DUF2809
WP_088448637.1
Roseateles terrae
Skillet-DUF2809
WP_221323631.1
Actinoplanes sp. L3-i22
Skillet-DUF2809
WP_218278091.1
Pseudomonas sp. OV226
Skillet-DUF2809
WP_110562506.1
Skillet-DUF2809
WP_218010007.1
Actinomadura kijaniata NBRC 14229
Skillet-DUF2809
MBC8167611.1
Bryobacteraceae bacterium
Skillet-DUF2809
MBD3378052.1
Candidatus Zhuqueibacterota bacterium
Skillet-DUF2809
MBG0831166.1
Planomonospora sp. ID67723
Skillet-DUF2809
WP_203686645.1
Skillet-DUF2809
WP_146836401.1
Cellulomonas hominis
Skillet-DUF2809
PJN23059.1
Kitasatospora sp. CB02891
Skillet-DUF2809
WP_215212993.1
Streptomyces sp. RO-S4
Skillet-DUF2809
SFW78161.1
Chitinophaga sancti
Skillet-DUF2809
MBU6238620.1
Planctomycetota bacterium
Skillet-DUF2809
WP_106214587.1
Glaciihabitans tibetensis
Skillet-DUF2809
AOR97672.1
Bacillus subtilis
Skillet-DUF2809
CAB3869538.1
Achromobacter mucicolens
Skillet-DUF2809
CAB3736716.1
Achromobacter denitrificans
Skillet-DUF2809
MBL9013880.1
Myxococcales bacterium
Skillet-DUF2809
RYZ23181.1
Chitinophagaceae bacterium
Skillet-DUF2809
WP_084021138.1
Cryobacterium arcticum
Skillet-DUF2809
WP_188296279.1
Streptomyces sp. CBMA156
Skillet-DUF2809
WP_226059452.1
Shewanella sp. SR1
Skillet-DUF2809
MBM7494492.1
Micromonospora luteifusca
Skillet-DUF2809
WP_218778708.1
Mycetocola reblochoni REB411
Skillet-DUF2809
WP_134641168.1
Cryobacterium lactosi
Skillet-DUF2809
MCB8043309.1
Microbacterium oxydans
Skillet-DUF2809
WP_201278934.1
Leptolyngbya iicbica LK
Skillet-DUF2809
WP_207214179.1
Brumimicrobium glaciale
Skillet-DUF2809
WP_214956486.1
Arthrobacter sp. ISL-69
Skillet-DUF2809
MBM3965975.1
Planctomycetota bacterium
Skillet-DUF2809
WP_233912123.1
Arthrobacter polaris
Skillet-DUF2809
MBP3388867.1
Clostridia bacterium
Skillet-DUF2809
MBX7124977.1
Cyclobacteriaceae bacterium
Skillet-DUF2809
RMD97382.1
Calditrichota bacterium
Skillet-DUF2809
RDL48737.1
Ensifer sp. M14
Skillet-DUF2809
MBI85306.1
Planctomycetaceae bacterium
Skillet-DUF2809
MAC73993.1
Phycisphaerae bacterium
Skillet-DUF2809
ARE86891.1
Skillet-DUF2809
GHI75582.1
Streptomyces spororaveus
Skillet-DUF2809
WP_152101219.1
Lacipirellula parvula
Skillet-DUF2809
WP_167736948.1
Micrococcus flavus
Skillet-DUF2809
MBW1781312.1
Deltaproteobacteria bacterium
Skillet-DUF2809
MCB1099795.1
Verrucomicrobiia bacterium
Skillet-DUF2809
WP_116410593.1
Subtercola boreus
Skillet-DUF2809
MBL7644890.1
Candidatus Hydrogenedentota bacterium
Skillet-DUF2809
KYC78760.1
Bacillus licheniformis
Skillet-DUF2809

MBP7333607.1	*Candidatus Cloacimonas sp.*	Skillet-DUF2809
WP_202721636.1	*Shewanella schlegeliana*	Skillet-DUF2809
MYV73181.1	*Streptomyces sp. SID1046*	Skillet-DUF2809
MBT7065150.1	*Verrucomicrobiota bacterium*	Skillet-DUF2809
WP_060534647.1	*Paenibacillus bovis*	Skillet-DUF2809
MYV52579.1	*Streptomyces sp. SID3212*	Skillet-DUF2809
WP_083325815.1	*Hymenobacter coccineus*	Skillet-DUF2809
WP_074317526.1	*Micromonospora cremea*	Skillet-DUF2809
WP_204224364.1	*Archangium violaceum*	Skillet-DUF2809
WP_068707032.1	*Oerskovia enterophila*	Skillet-DUF2809
MSU32602.1	*Pedosphaera sp.*	Skillet-DUF2809
WP_158612703.1	*Frigoribacterium sp. PhB24*	Skillet-DUF2809
NRD26229.1	*Frigoribacterium sp. VKM Ac-2836*	Skillet-DUF2809
WP_055916286.1	*Cellulomonas sp. Root485*	Skillet-DUF2809
WP_020015857.1	*Promicromonospora sukumoe 327MFSha3.1*	Skillet-DUF2809
MBO5326158.1	*Lachnospiraceae bacterium*	Skillet-DUF2809
WP_230647965.1	*Streptacidiphilus sp. ASG 303*	Skillet-DUF2809
MBI1913862.1	*Planctomycetota bacterium*	Skillet-DUF2809
WP_200669599.1	*Oxynema sp. CENA135*	Skillet-DUF2809
MBK9925138.1	*Candidatus Defluviilinea proxima*	Skillet-DUF2809
WP_221474463.1	*Planomonospora venezuelensis*	Skillet-DUF2809
WP_190533482.1	*Limnothrix sp. FACHB-406*	Skillet-DUF2809
WP_235338349.1	*Paenibacillus wulumuqiensis*	Skillet-DUF2809
WP_221055266.1	*Shewanella algae*	Skillet-DUF2809
GHI86794.1	*Streptomyces xanthophaeus*	Skillet-DUF2809
WP_102159973.1	*Zhihengliuella halotolerans*	Skillet-DUF2809
MBG6238056.1		Skillet-DUF2809
MCB1128472.1	*Verrucomicrobiia bacterium*	Skillet-DUF2809
NER00206.1	*Cyanothece sp. SIO2G6*	Skillet-DUF2809
MBQ1761797.1	*Aquincola sp.*	Skillet-DUF2809
HHO52447.1	*Deltaproteobacteria bacterium*	Skillet-DUF2809
WP_146929901.1	*Cellulomonas xylanilytica*	Skillet-DUF2809
MBL8021849.1	*Leptospirales bacterium*	Skillet-DUF2809
HDQ44274.1	*bacterium*	Skillet-DUF2809
WP_224356068.1	*Micrococcus porci*	Skillet-DUF2809
WP_090589583.1	*Auraticoccus monumenti*	Skillet-DUF2809
WP_229214240.1	*Dyadobacter flavalbus*	Skillet-DUF2809
WP_137249311.1		Skillet-DUF2809
WP_109131184.1	*Cellulomonas sp. WB94*	Skillet-DUF2809
HAB17517.1	*Verrucomicrobiales bacterium*	Skillet-DUF2809
GIF94580.1	*Catellatospora chokoriensis*	Skillet-DUF2809
WP_172138586.1		Skillet-DUF2809
WP_214925130.1		Skillet-DUF2809
WP_174224519.1		Skillet-DUF2809
MBV9868430.1	*Abditibacteriaceae bacterium*	Skillet-DUF2809
SOD03731.1	*bacterium JGI 053*	Skillet-DUF2809
NCF67560.1	*Chloroflexota bacterium*	Skillet-DUF2809
MBF8642864.1	*Pseudomonas luteola*	Skillet-DUF2809
WP_052265457.1	*Ruegeria sp. ANG-R*	Skillet-DUF2809
WP_235818787.1	*Brucella rhizosphaerae*	Skillet-DUF2809
GIG84617.1	*Planotetraspora kaengkrachanensis*	Skillet-DUF2809
WP_229383794.1	*Microbacterium allomyrinae*	Skillet-DUF2809
WP_219820077.1	*Pseudoclavibacter sp. RFBH5*	Skillet-DUF2809
WP_081859693.1	*Microbacterium sp. K21*	Skillet-DUF2809
MBP9940220.1	*Comamonas sp.*	Skillet-DUF2809
WP_105672395.1	*Microbacterium natoriense*	Skillet-DUF2809
WP_183150421.1	*Microbacterium sp. Se63.02b*	Skillet-DUF2809
WP_045256095.1	*Microbacterium hydrocarbonoxydans*	Skillet-DUF2809
WP_141386240.1	*Microbacterium maritypicum*	Skillet-DUF2809
WP_015593380.1		Skillet-DUF2809
WP_116636337.1	*Microbacterium sp. TPD7012*	Skillet-DUF2809
TSA40399.1	*Verrucomicrobiales bacterium*	Skillet-DUF2809
WP_190469874.1	*Aerosakkonema funiforme FACHB-1375*	Skillet-DUF2809
GDY00647.1	*Phycisphaerae bacterium*	Skillet-DUF2809
MBI4750719.1	*Acidobacteriota bacterium*	Skillet-DUF2809
MBV8708831.1	*Acidobacteriaceae bacterium*	Skillet-DUF2809
GHE32890.1	*Streptosporangium violaceochromogenes*	Skillet-DUF2809
WP_018883241.1	*Paenibacillus massiliensis 2301065 = DSM 16942*	Skillet-DUF2809
NET11239.1	*Symploca sp. SIO2B6*	Skillet-DUF2809
MBW8763638.1	*Microbacterium sp.*	Skillet-DUF2809
WP_202994563.1		Skillet-DUF2809
WP_106834010.1		Skillet-DUF2809
WP_083090220.1	*Actinomyces vulturis*	Skillet-DUF2809
MBL8231882.1	*Bryobacterales bacterium*	Skillet-DUF2809
WP_168914379.1	*Microbacterium salsuginis*	Skillet-DUF2809
PVU67919.1	*Plesiomonas shigelloides*	Skillet-DUF2809
WP_210230996.1	*Arthrobacter jiangjiafuii*	Skillet-DUF2809
WP_144615390.1	*Brevibacillus brevis*	Skillet-DUF2809
GDY14802.1	*Planctomycetota bacterium*	Skillet-DUF2809
NUL48947.1	*Cellulosimicrobium funkei*	Skillet-DUF2809
WP_218026454.1	*Cellulomonas cellasea*	Skillet-DUF2809
KEG38152.1	*Streptomyces griseorubens*	Skillet-DUF2809
MCC7062530.1	*Planctomycetota bacterium*	Skillet-DUF2809
WP_146494193.1		Skillet-DUF2809
MBE7386140.1	*Leptolyngbya sp. SIO1E4*	Skillet-DUF2809
WP_215049267.1	*Streptomyces sp. McG7*	Skillet-DUF2809
GGU71084.1	*Kitasatospora aureofaciens*	Skillet-DUF2809
WP_228447342.1		Skillet-DUF2809
WP_130505200.1	*Microterricola gilva*	Skillet-DUF2809
WP_214466728.1	*Microbacterium flavescens*	Skillet-DUF2809
WP_203894627.1	*Planobispora longispora*	Skillet-DUF2809
PSB12460.1	*filamentous cyanobacterium CCP2*	Skillet-DUF2809
RZU75578.1	*Micromonospora kangleipakensis*	Skillet-DUF2809

WP_189274040.1	*Kitasatospora griseola*	Skillet-DUF2809
SEH35422.1	*Chryseobacterium culicis*	Skillet-DUF2809
NJM77114.1	*Acaryochloridaceae cyanobacterium RU_4_10*	Skillet-DUF2809
WP_109945656.1	*Micromonospora globispora*	Skillet-DUF2809
MCA2966765.1	*Acidobacteriaceae bacterium*	Skillet-DUF2809
WP_220198102.1	*Ktedonospora formicarum*	Skillet-DUF2809
WP_224997207.1	*Cesiribacter sp. SM1*	Skillet-DUF2809
MBF8276231.1	*Candidatus Brocadiaceae bacterium*	Skillet-DUF2809
MBL7114689.1	*Kiritimatiellia bacterium*	Skillet-DUF2809
WP_144705793.1		Skillet-DUF2809
WP_210929615.1	*Comamonas aquatica*	Skillet-DUF2809
MBE6564747.1	*Oscillospiraceae bacterium*	Skillet-DUF2809
WP_118768388.1	*Cellulomonas rhizosphaerae*	Skillet-DUF2809
TAD75540.1	*Oscillatoriales cyanobacterium*	Skillet-DUF2809
WP_018975176.1	*Saccharibacillus kuerlensis*	Skillet-DUF2809
HAC90310.1	*Planctomycetaceae bacterium*	Skillet-DUF2809
MBT40068.1	*Deltaproteobacteria bacterium*	Skillet-DUF2809
NER38162.1	*Oscillatoria sp. SIO1A7*	Skillet-DUF2809
MBR2807068.1	*Oscillospiraceae bacterium*	Skillet-DUF2809
WP_015150388.1	*Oscillatoria acuminata PCC 6304*	Skillet-DUF2809
VVS91275.1	*Desulfoluna spongiiphila*	Skillet-DUF2809
WP_203661268.1		Skillet-DUF2809
MBE1364052.1		Skillet-DUF2809
WP_006929124.1	*Caldithrix abyssi DSM 13497*	Skillet-DUF2809
NIO68139.1	*Anaerolineae bacterium*	Skillet-DUF2809
WP_150307947.1	*Planctomonas psychrotolerans*	Skillet-DUF2809
WP_200277728.1	*Haloferula rosea*	Skillet-DUF2809
MBO4290271.1	*Lachnospiraceae bacterium*	Skillet-DUF2809
WP_055806309.1	*Arthrobacter sp. Leaf337*	Skillet-DUF2809
MBX3409652.1	*Phycisphaeraceae bacterium*	Skillet-DUF2809
MBA3398014.1	*Deltaproteobacteria bacterium*	Skillet-DUF2809
PWF55394.1	*Massilia glaciei*	Skillet-DUF2809
WP_211341471.1	*Myceligenerans xiligouense*	Skillet-DUF2809
WP_070248255.1	*Duganella phyllosphaerae*	Skillet-DUF2809
MBC8134026.1	*Deltaproteobacteria bacterium*	Skillet-DUF2809
WP_184038018.1	*Chitinivorax tropicus*	Skillet-DUF2809
RDI58110.1	*Flavobacterium glaciei*	Skillet-DUF2809
MBW1796494.1	*Deltaproteobacteria bacterium*	Skillet-DUF2809
MCC6811204.1	*Deltaproteobacteria bacterium*	Skillet-DUF2809
RZK33702.1	*Pedobacter sp.*	Skillet-DUF2809
WP_123739700.1	*Salana multivorans*	Skillet-DUF2809
WP_093618583.1	*Actinoplanes philippinensis*	Skillet-DUF2809
WP_163542150.1	*Occultella kanbiaonis*	Skillet-DUF2809
MBD9698004.1	*Flavimobilis rhizosphaerae*	Skillet-DUF2809
MBC8277566.1	*FCB group bacterium*	Skillet-DUF2809
WP_101585104.1	*Bacillus sp. T33-2*	Skillet-DUF2809
MBY0423240.1	*Parvularculaceae bacterium*	Skillet-DUF2809
WP_093223655.1	*Sphingomonas sp. NFR15*	Skillet-DUF2809
WP_191828420.1	*Cellulosimicrobium arenosum*	Skillet-DUF2809
WP_010094754.1	*Ornithinibacillus scapharcae TW25*	Skillet-DUF2809
WP_071517642.1	*Geitlerinema sp. PCC 9228*	Skillet-DUF2809
WP_081978605.1	*Cellulomonas carbonis*	Skillet-DUF2809
WP_233257406.1	*Promicromonospora sp. AC04*	Skillet-DUF2809
WP_056018788.1	*Cellulomonas sp. Leaf395*	Skillet-DUF2809
MCB1088549.1	*Verrucomicrobiia bacterium*	Skillet-DUF2809
WP_100747885.1	*Leptospira ellisii*	Skillet-DUF2809
WP_073565666.1	*Archangium sp. Cb G35*	Skillet-DUF2809
HCD46917.1	*Lachnoclostridium sp.*	Skillet-DUF2809
WP_190429984.1	*Coleofasciculus sp. FACHB-712*	Skillet-DUF2809
WP_211263678.1	*Stenotrophomonas terrae*	Skillet-DUF2809
MBN8729629.1	*Acidobacteriota bacterium*	Skillet-DUF2809
WP_214867709.1	*Agromyces sp. ISL-38*	Skillet-DUF2809
WP_230067256.1	*Arthrobacter cryoconiti*	Skillet-DUF2809
WP_083363579.1	*Microterricola viridarii*	Skillet-DUF2809
NOT35527.1	*Candidatus Eiseniibacteriota bacterium*	Skillet-DUF2809
AFJ61325.1	*Bacillus velezensis YAU B9601-Y2*	Skillet-DUF2809
MBL8160529.1	*Anaerolineae bacterium*	Skillet-DUF2809
WP_148818169.1	*Pigmentiphaga aceris*	Skillet-DUF2809
PSQ76559.1	*Bacteroidetes bacterium QH_6_63_17*	Skillet-DUF2809
UCF64537.1	*bacterium*	Skillet-DUF2809
WP_229262401.1	*Duganella guangzhouensis*	Skillet-DUF2809
WP_166256817.1		Skillet-DUF2809
MCD4746448.1	*Bacteroidales bacterium*	Skillet-DUF2809
MBM6621320.1	*Micrococcaceae bacterium RIT 802*	Skillet-DUF2809
WP_045275346.1	*Microbacterium terrae*	Skillet-DUF2809
WP_173922860.1	*Agromyces sp. Marseille-P2726*	Skillet-DUF2809
WP_011320698.1		Skillet-DUF2809
WP_051858144.1	*Streptomyces cellulosae*	Skillet-DUF2809
MCB1174773.1	*Leptospiraceae bacterium*	Skillet-DUF2809
WP_225583289.1		Skillet-DUF2809
PLS83161.1	*Chloroflexota bacterium*	Skillet-DUF2809
PSB04838.1	*Merismopedia glauca CCAP 1448/3*	Skillet-DUF2809
TXH84431.1	*Pseudoxanthomonas sp.*	Skillet-DUF2809
WP_223227581.1	*Campylobacter concisus*	Skillet-DUF2809
WP_225724104.1	*Campylobacter concisus*	Skillet-DUF2809
WP_224076484.1	*Planococcus chinensis*	Skillet-DUF2809
WP_230855924.1	*Campylobacter concisus*	Skillet-DUF2809
WP_036803552.1	*Planococcus glaciei CHR43*	Skillet-DUF2809
MBR6198260.1	*Bacteroidaceae bacterium*	Skillet-DUF2809
MCC7001012.1	*Gemmatimonadaceae bacterium*	Skillet-DUF2809
WP_232711478.1	*Microbacterium lacus*	Skillet-DUF2809
WP_116414053.1	*Subtercola boreus*	Skillet-DUF2809
MCA9261348.1	*Planctomycetales bacterium*	Skillet-DUF2809

MCA9149139.1	*Planctomycetales bacterium*	Skillet-DUF2809
NOZ56964.1	*Calditrichota bacterium*	Skillet-DUF2809
MBC8591729.1	*Jilunia laotingensis*	Skillet-DUF2809
WP_227933313.1	*Arthrobacter sp. zg-Y1110*	Skillet-DUF2809
NUQ53103.1	*Phycisphaerales bacterium*	Skillet-DUF2809
WP_230981054.1	*Gemmatimonas groenlandica*	Skillet-DUF2809
MBN1348072.1	*Candidatus Zhuqueibacterota bacterium*	Skillet-DUF2809
MBJ7285597.1	*Akkermansiaceae bacterium*	Skillet-DUF2809
HDP98872.1	*bacterium*	Skillet-DUF2809
WP_052346517.1	*Hymenobacter swuensis DY53*	Skillet-DUF2809
WP_020380616.1	*Leptospira interrogans serovar Szwajizak*	Skillet-DUF2809
WP_235819463.1	*Caldifermentibacillus hisashii*	Skillet-DUF2809
WP_166414818.1	*Sanguibacter sp. HDW7*	Skillet-DUF2809
WP_150241004.1		Skillet-DUF2809
MBV1757183.1	*Dethiosulfatibacter sp.*	Skillet-DUF2809
MBW4489260.1	*Trichocoleus desertorum ATA4-8-CV12*	Skillet-DUF2809
MBC7291704.1	*Actinotalea sp.*	Skillet-DUF2809
NQX03098.1	*bacterium*	Skillet-DUF2809
WP_199213975.1	*Streptomyces sp. BSE7-9*	Skillet-DUF2809
WP_130059614.1	*Bacteroides salyersiae*	Skillet-DUF2809
WP_200938427.1	*Cellulomonas sp. Root137*	Skillet-DUF2809
HAM80585.1	*Ornithinibacillus sp.*	Skillet-DUF2809
MBV9930267.1	*Alphaproteobacteria bacterium*	Skillet-DUF2809
WP_157425243.1	*Agromyces lapidis*	Skillet-DUF2809
WP_015181839.1	*Allocoleopsis franciscana PCC 7113*	Skillet-DUF2809
BAZ17541.1	*Calothrix sp. NIES-4071*	Skillet-DUF2809
MBA3871793.1	*Anaerolineae bacterium*	Skillet-DUF2809
WP_063364108.1	*Pseudoalteromonas luteoviolacea H33*	Skillet-DUF2809
WP_162907364.1	*Allorhizocola rhizosphaerae*	Skillet-DUF2809
HIK28330.1	*Oscillatoriaceae cyanobacterium M7585_C2015_266*	Skillet-DUF2809
MBK6464950.1	*Myxococcales bacterium*	Skillet-DUF2809
WP_223102909.1	*Dactylosporangium vinaceum*	Skillet-DUF2809
WP_224249876.1	*Hyalangium gracile*	Skillet-DUF2809
WP_039983077.1	*Vibrio sagamiensis NBRC 104589*	Skillet-DUF2809
RNC81216.1	*Phycisphaera sp.*	Skillet-DUF2809
MBC8109258.1	*Phycisphaerae bacterium*	Skillet-DUF2809
RZL58204.1	*Variovorax sp.*	Skillet-DUF2809
WP_079411363.1	*Paenibacillus ferrarius*	Skillet-DUF2809
WP_233245088.1	*Caulobacter sp. HMWF009*	Skillet-DUF2809
HEG44456.1	*Phycisphaerales bacterium*	Skillet-DUF2809
MSR59442.1	*Planctomycetaceae bacterium*	Skillet-DUF2809
PZO50131.1	*Phormidesmis priestleyi*	Skillet-DUF2809
GID45769.1	*Actinoplanes campanulatus*	Skillet-DUF2809
WP_009194305.1	*Cesiribacter andamanensis AMV16*	Skillet-DUF2809
WP_214452322.1	*Glaciecola sp. XM2*	Skillet-DUF2809
WP_145271467.1	*Tautonia plasticadhaerens*	Skillet-DUF2809
MBN8594185.1	*Anaerolineae bacterium*	Skillet-DUF2809
RKE89811.1	*Epilithonimonas arachidiradicis*	Skillet-DUF2809
KAF0095800.1	*Puniceicoccaceae bacterium 5H*	Skillet-DUF2809
MBT5926658.1	*Verrucomicrobiota bacterium*	Skillet-DUF2809
MBX3156644.1	*Deltaproteobacteria bacterium*	Skillet-DUF2809
VVV07077.1		Skillet-DUF2809
WP_093060677.1	*Psychrobacillus sp. OK028*	Skillet-DUF2809
MBC8047186.1	*Chitinophagales bacterium*	Skillet-DUF2809
WP_184634747.1		Skillet-DUF2809
WP_135850881.1	*Serinibacter arcticus*	Skillet-DUF2809
MBI1322673.1	*bacterium*	Skillet-DUF2809
MBO9699876.1	*Sporocytophaga sp.*	Skillet-DUF2809
MCB9809050.1	*Candidatus Nomurabacteria bacterium*	Skillet-DUF2809
WP_034357272.1	*Comamonas thiooxydans*	Skillet-DUF2809
WP_123607098.1	*Micromonospora sp. Llam0*	Skillet-DUF2809
RYD68364.1	*Verrucomicrobiaceae bacterium*	Skillet-DUF2809
NLH72727.1	*Verrucomicrobiota bacterium*	Skillet-DUF2809
WP_125660382.1	*Sphingomonas sp. 2R-10*	Skillet-DUF2809
WP_203704809.1	*Asanoa iriomotensis*	Skillet-DUF2809
WP_056005949.1	*Agromyces sp. Root1464*	Skillet-DUF2809
WP_166874312.1	*Salinibacterium sp. ZJ450*	Skillet-DUF2809
WP_082450957.1	*Sphingomonas sp. Leaf22*	Skillet-DUF2809
HHR90551.1		Skillet-DUF2809
WP_035446086.1	*Bacillus sp. UNC41MFS5*	Skillet-DUF2809
WP_169282344.1		Skillet-DUF2809
MBL8241309.1	*Bryobacterales bacterium*	Skillet-DUF2809
MCA0382170.1	*Bacteroidota bacterium*	Skillet-DUF2809
WP_210661353.1	*Paenibacillus lignilyticus*	Skillet-DUF2809
MBL8220159.1	*Bryobacterales bacterium*	Skillet-DUF2809
WP_129340801.1	*Sphingomonas desiccabilis*	Skillet-DUF2809
WP_217387813.1	*Paeniglutamicibacter quisquiliarum*	Skillet-DUF2809
NJN15431.1	*Oscillochloris sp.*	Skillet-DUF2809
RQM70508.1	*Aeromonas jandaei*	Skillet-DUF2809
WP_075085542.1	*Mariniblastus fucicola*	Skillet-DUF2809
RZI78849.1	*Microbacterium sp.*	Skillet-DUF2809
OAB47055.1	*Paenibacillus antarcticus*	Skillet-DUF2809
MBL0158789.1	*Bryobacterales bacterium*	Skillet-DUF2809
MBD1886325.1	*Microcoleus sp. FACHB-84*	Skillet-DUF2809
MBE6648286.1	*Oscillospiraceae bacterium*	Skillet-DUF2809
WP_135223646.1	*Flavobacterium foetidum*	Skillet-DUF2809
WP_183512550.1	*Paeniglutamicibacter cryotolerans*	Skillet-DUF2809
WP_203750925.1	*Cellulomonas chitinilytica*	Skillet-DUF2809
NET44044.1	*Okeania sp. SIO2B3*	Skillet-DUF2809
MBQ3065197.1	*Clostridia bacterium*	Skillet-DUF2809
MCC3528760.1	*Microcoleus sp. PH2017_21_RUC_O_A*	Skillet-DUF2809
HEN18667.1		Skillet-DUF2809
GGA79581.1	*Ornithinibacillus halotolerans*	Skillet-DUF2809

WP_056494583.1	*Sphingomonas sp. Leaf25*	Skillet-DUF2809
MBN2412795.1	*Candidatus Zhuqueibacterota bacterium*	Skillet-DUF2809
MBC7798156.1	*Pyrinomonadaceae bacterium*	Skillet-DUF2809
WP_125431106.1	*Hymenobacter metallilatus*	Skillet-DUF2809
OUS27592.1	*Gammaproteobacteria bacterium 45_16_T64*	Skillet-DUF2809
MBZ0154473.1	*Planctomycetota bacterium*	Skillet-DUF2809
MBC7807956.1	*Armatimonadota bacterium*	Skillet-DUF2809
WP_035836190.1	*Cryobacterium roopkundense*	Skillet-DUF2809
MBW4518788.1	*Scytolyngbya sp. HA4215-MV1*	Skillet-DUF2809
MBT5928408.1	*Verrucomicrobiota bacterium*	Skillet-DUF2809
WP_119974224.1	*Cryobacterium melibiosiphilum*	Skillet-DUF2809
NQT64251.1	*Fidelibacterota bacterium*	Skillet-DUF2809
MBL0940352.1	*Gemmatimonadaceae bacterium*	Skillet-DUF2809
HHO51757.1	*Deltaproteobacteria bacterium*	Skillet-DUF2809
WP_194595836.1	*Frigoribacterium sp. VKM Ac-1396*	Skillet-DUF2809
WP_232885869.1	*Buttiauxella ferragutiae*	Skillet-DUF2809
MCB9496142.1	*Fibrobacteria bacterium*	Skillet-DUF2809
WP_033106887.1	*Microbacterium sp.*	Skillet-DUF2809
WP_179552006.1	*Agromyces hippuratus*	Skillet-DUF2809
WP_022889328.1	*Agromyces italicus*	Skillet-DUF2809
MCB0009005.1		Skillet-DUF2809
MBS1830169.1	*Acidobacteriota bacterium*	Skillet-DUF2809
WP_100293080.1	*Aeromonas cavernicola*	Skillet-DUF2809
TAE59561.1	*Nostocales cyanobacterium*	Skillet-DUF2809
MBD0363472.1	*Coleofasciculus sp. C3-bin4*	Skillet-DUF2809
WP_187118935.1	*Bacillus marasmi*	Skillet-DUF2809
WP_034529223.1		Skillet-DUF2809
MBL7136044.1	*Fidelibacterota bacterium*	Skillet-DUF2809
WP_131838629.1	*Acetobacteroides hydrogenigenes*	Skillet-DUF2809
WP_157509428.1	*Glaciibacter superstes DSM 21135*	Skillet-DUF2809
WP_200352775.1	*Luteolibacter yonseiensis*	Skillet-DUF2809
MBE2220146.1	*Anaerolineae bacterium*	Skillet-DUF2809
WP_075721316.1	*Roseburia sp. 499*	Skillet-DUF2809
WP_228484499.1	*Microbacterium cremeum*	Skillet-DUF2809
KAF0198991.1	*Bacteroidota bacterium*	Skillet-DUF2809
WP_115540996.1	*Xanthomonas campestris pv. campestris*	Skillet-DUF2809
WP_149160567.1	*Agromyces intestinalis*	Skillet-DUF2809
MBI4893712.1	*Acidobacteriota bacterium*	Skillet-DUF2809
WP_075514757.1	*Rothia kristinae*	Skillet-DUF2809
WP_127846322.1	*Psychroflexus aestuariivivens*	Skillet-DUF2809
WP_069070375.1	*Nostoc sp. KVJ20*	Skillet-DUF2809
NJL84105.1	*Chloroflexaceae bacterium*	Skillet-DUF2809
MBZ0265048.1	*bacterium*	Skillet-DUF2809
WP_190958072.1	*Nostoc paludosum FACHB-159*	Skillet-DUF2809
WP_045515789.1	*Clostridium sporogenes*	Skillet-DUF2809
WP_196991181.1	*Panacibacter microcysteis*	Skillet-DUF2809
WP_230103874.1	*Microbacterium sp. Bi121*	Skillet-DUF2809
WP_179600538.1	*Neobacillus driksii*	Skillet-DUF2809
MBU0494179.1	*Chloroflexota bacterium*	Skillet-DUF2809
MBO9698522.1	*Sphingopyxis sp.*	Skillet-DUF2809
RKJ06765.1	*bacterium D16-54*	Skillet-DUF2809
NTX07441.1	*Myxococcus sp. CA040A*	Skillet-DUF2809
NTX60168.1	*Myxococcus sp. CA051A*	Skillet-DUF2809
WP_234375540.1	*Streptomyces sp. NPDC058451*	Skillet-DUF2809
KAB8144834.1	*Chloroflexia bacterium SDU3-3*	Skillet-DUF2809
MBE6902733.1	*Oscillospiraceae bacterium*	Skillet-DUF2809
MBN1670750.1	*Kiritimatiellia bacterium*	Skillet-DUF2809
WP_190756916.1	*Microcoleus sp. FACHB-68*	Skillet-DUF2809
RZL19551.1	*Pedobacter sp.*	Skillet-DUF2809
NDU77289.1	*Actinomadura lepetitiana*	Skillet-DUF2809
WP_198485942.1	*Achromobacter deleyi*	Skillet-DUF2809
WP_148599020.1	*Aquisphaera giovannonii*	Skillet-DUF2809
SJM59844.1	*Arthrobacter rhombi*	Skillet-DUF2809
MBW4533794.1	*Pleurocapsa minor HA4230-MV1*	Skillet-DUF2809
MCC3467402.1	*Microcoleus sp. PH2017_06_SFM_O_A*	Skillet-DUF2809
WP_211229264.1	*Nakamurella lactea DSM 19367*	Skillet-DUF2809
MBZ2186635.1	*Bryobacter sp. CoA8 C33*	Skillet-DUF2809
WP_143386128.1	*Flavobacterium sp. ZB4R12*	Skillet-DUF2809
WP_100350128.1	*Luteimicrobium subarcticum*	Skillet-DUF2809
MBX9579999.1	*Gemmataceae bacterium*	Skillet-DUF2809
WP_088318314.1	*Kineosporia sp. R_H_3*	Skillet-DUF2809
WP_190446966.1		Skillet-DUF2809
MBE3066779.1	*Chloroflexota bacterium*	Skillet-DUF2809
MBK7992617.1	*Blastocatellia bacterium*	Skillet-DUF2809
MBE1353981.1		Skillet-DUF2809
ONI61902.1	*Leifsonia sp. ALI-44-B*	Skillet-DUF2809
WP_147188099.1	*Panacibacter ginsenosidivorans*	Skillet-DUF2809
MBN6057176.1	*Nonomuraea sp. RK-328*	Skillet-DUF2809
MBN1895779.1	*bacterium*	Skillet-DUF2809
WP_038464104.1	*Arthrobacter sp. PAMC 25486*	Skillet-DUF2809
RZJ81002.1	*Flavobacterium sp.*	Skillet-DUF2809
WP_229640102.1	*Waterburya agarophytonicola KI4*	Skillet-DUF2809
MBE7174212.1	*Williamsia sp.*	Skillet-DUF2809
WP_137261503.1	*Ilyomonas limi*	Skillet-DUF2809
NII70708.1	*Microbacterium ulmi*	Skillet-DUF2809
WP_104534629.1	*Xanthomonas arboricola pv. populi*	Skillet-DUF2809
MBI5516596.1		Skillet-DUF2809
NLD45996.1	*Clostridiaceae bacterium*	Skillet-DUF2809
QUG42090.1	*Psychrobacillus sp. INOP01*	Skillet-DUF2809
WP_204998784.1	*Parashewanella hymeniacidonis*	Skillet-DUF2809
WP_052807313.1	*Risungbinella massiliensis*	Skillet-DUF2809
MBL8872950.1	*Planctomycetaceae bacterium*	Skillet-DUF2809
WP_188405502.1	*Psychroflexus salis*	Skillet-DUF2809

MCC6603223.1	*Anaerolineae bacterium*	Skillet-DUF2809
WP_102585262.1	*Xanthomonas arboricola pv. fragariae*	Skillet-DUF2809
WP_104543371.1	*Xanthomonas codiaei*	Skillet-DUF2809
WP_184650310.1	*Xanthomonas euroxanthea*	Skillet-DUF2809
WP_010365544.1	*Xanthomonas vasicola pv. vasculorum*	Skillet-DUF2809
HAA76444.1	*Candidatus Latescibacterota bacterium*	Skillet-DUF2809
WP_167865475.1	*Clostridium gasigenes*	Skillet-DUF2809
UCG51353.1	*Candidatus Latescibacterota bacterium*	Skillet-DUF2809
WP_188867010.1	*Pseudomonas asuensis*	Skillet-DUF2809
TAE73983.1	*Verrucomicrobiota bacterium*	Skillet-DUF2809
UCH82541.1	*Candidatus Latescibacterota bacterium*	Skillet-DUF2809
NBQ23100.1	*Verrucomicrobiota bacterium*	Skillet-DUF2809
MBU1106487.1	*Candidatus Rifleibacteriota bacterium*	Skillet-DUF2809
MBU0711667.1	*bacterium*	Skillet-DUF2809
WP_078715579.1	*Agreia bicolorata*	Skillet-DUF2809
MBP8220615.1	*Aeromonadaceae bacterium*	Skillet-DUF2809
PIS27783.1	*Fidelibacterota bacterium CG08_land_8_20_14_0_20_45_22*	Skillet-DUF2809
WP_221857561.1	*Microbacterium marinilacus*	Skillet-DUF2809
UCH63281.1	*Fidelibacterota bacterium*	Skillet-DUF2809
GBF80082.1	*Aphanothece sacrum FPU1*	Skillet-DUF2809
WP_158559273.1	*Deminuibacter soli*	Skillet-DUF2809
MBN1827474.1	*Candidatus Eiseniibacteriota bacterium*	Skillet-DUF2809
MCA9759376.1	*Candidatus Eiseniibacteriota bacterium*	Skillet-DUF2809
WP_157549976.1	*Microbacterium sp. Leaf161*	Skillet-DUF2809
NUN99599.1	*Saprospiraceae bacterium*	Skillet-DUF2809
NQT85101.1	*bacterium*	Skillet-DUF2809
MBN1523204.1	*Spirochaetales bacterium*	Skillet-DUF2809
MBT4798802.1	*Verrucomicrobiota bacterium*	Skillet-DUF2809
KEI12101.1	*Clostridium novyi B str. ATCC 27606*	Skillet-DUF2809
MBT7959372.1	*Akkermansiaceae bacterium*	Skillet-DUF2809
WP_205958132.1	*Flammeovirga yaeyamensis*	Skillet-DUF2809
WP_013325442.1		Skillet-DUF2809
WP_152666325.1	*Xanthomonas hortorum pv. gardneri*	Skillet-DUF2809
MBI9061322.1	*Marinilabiliaceae bacterium*	Skillet-DUF2809
WP_093278910.1		Skillet-DUF2809
WP_075062895.1	*Ornatilinea apprima*	Skillet-DUF2809
MAE65466.1	*Phycisphaeraceae bacterium*	Skillet-DUF2809
WP_146602090.1	*Novipirellula aureliae*	Skillet-DUF2809
MBL0136258.1	*Chitinophagaceae bacterium*	Skillet-DUF2809
WP_035748791.1	*Arthrobacter sp. 35W*	Skillet-DUF2809
WP_015121977.1	*Rivularia sp. PCC 7116*	Skillet-DUF2809
NOQ25029.1	*Bacteroidales bacterium*	Skillet-DUF2809
WP_146833206.1	*Antarcticibacterium arcticum*	Skillet-DUF2809
MBL8177812.1	*Bryobacterales bacterium*	Skillet-DUF2809
WP_115480201.1	*Anaerosacchariphilus polymeriproducens*	Skillet-DUF2809
WP_167016665.1	*Chitinophaga sp. Cy-1792*	Skillet-DUF2809
WP_212121067.1	*Niallia circulans*	Skillet-DUF2809
WP_157001665.1	*Agromyces laixinhei*	Skillet-DUF2809
MBP1657042.1	*Bacteroidota bacterium*	Skillet-DUF2809
MCB0632186.1	*Lewinella sp.*	Skillet-DUF2809
TFH03006.1	*Calditrichales bacterium*	Skillet-DUF2809
NTU75771.1	*Anaerolineaceae bacterium*	Skillet-DUF2809
WP_235525955.1	*Caulobacter sp. Root1472*	Skillet-DUF2809
NOZ60046.1	*Calditrichota bacterium*	Skillet-DUF2809
WP_103601087.1	*Campylobacter concisus*	Skillet-DUF2809
WP_103606159.1	*Campylobacter concisus*	Skillet-DUF2809
WP_138531597.1		Skillet-DUF2809
MBL8988802.1	*Gemmatimonadota bacterium*	Skillet-DUF2809
RCJ20421.1	*Nostoc sp. ATCC 43529*	Skillet-DUF2809
WP_230679899.1	*Pontibacter rufus*	Skillet-DUF2809
MBN1603093.1	*Chitinispirillaceae bacterium*	Skillet-DUF2809
WP_067026111.1	*Microbacterium sediminis*	Skillet-DUF2809
WP_129462075.1	*Flavobacterium stagni*	Skillet-DUF2809
MBC8140309.1	*Armatimonadota bacterium*	Skillet-DUF2809
MBT8260041.1	*Bacteroidia bacterium*	Skillet-DUF2809
OUC11617.1	*Alkalinema sp. CACIAM 70d*	Skillet-DUF2809
MBD3316007.1	*Chitinivibrionales bacterium*	Skillet-DUF2809
WP_188414490.1	*Mucilaginibacter galii*	Skillet-DUF2809
MBF0432785.1	*Fibrobacteria bacterium*	Skillet-DUF2809
MBI1926946.1	*Candidatus Poribacteria bacterium*	Skillet-DUF2809
MBP3326465.1	*Coprococcus sp.*	Skillet-DUF2809
WP_051400603.1	*Enterocloster hominis (ex Hitch et al. 2024)*	Skillet-DUF2809
MBP7887075.1	*Fidelibacterota bacterium*	Skillet-DUF2809
RJP72933.1	*Candidatus Zixiibacteriota bacterium*	Skillet-DUF2809
WP_200309731.1	*Persicirhabdus sediminis*	Skillet-DUF2809
WP_013498049.1	*Ruminococcus albus 7 = DSM 20455*	Skillet-DUF2809
WP_135154395.1	*Cohnella luojiensis*	Skillet-DUF2809
WP_202565082.1	*Agreia sp. COWG*	Skillet-DUF2809
MBL8897607.1	*Planctomycetota bacterium*	Skillet-DUF2809
RKY08790.1	*Planctomycetota bacterium*	Skillet-DUF2809
WP_221239833.1	*Sphingomonas abaci*	Skillet-DUF2809
WP_202766357.1	*Clostridium paridis*	Skillet-DUF2809
MBI4784696.1	*Oscillatoriophycideae cyanobacterium NC_groundwater_1537_Pr4_S-0.65um_50_18*	Skillet-DUF2809
NRB77564.1	*Saccharospirillaceae bacterium*	Skillet-DUF2809
MCA9157964.1	*Planctomycetales bacterium*	Skillet-DUF2809
BAU85943.1	*Streptomyces laurentii*	Skillet-DUF2809
TXH75538.1	*Lysobacteraceae bacterium*	Skillet-DUF2809
WP_230113781.1	*Microbacterium oxydans*	Skillet-DUF2809
WP_046357078.1	*Pseudoalteromonas luteoviolacea*	Skillet-DUF2809
MBP3321902.1	*Clostridia bacterium*	Skillet-DUF2809
WP_194764409.1	*Microbacterium sp. UFMG61*	Skillet-DUF2809
WP_226098977.1	*Clostridium tagluense*	Skillet-DUF2809
WP_118975986.1	*Taibaiella koreensis*	Skillet-DUF2809

WP_150223597.1	*Bacillus atrophaeus*	Skillet-DUF2809
WP_217181389.1	*Streptomyces sp. AC495_CC817*	Skillet-DUF2809
WP_145059437.1	*Adhaeretor mobilis*	Skillet-DUF2809
RMH87474.1	*Calditrichota bacterium*	Skillet-DUF2809
MCC6449666.1	*Candidatus Auribacterota bacterium*	Skillet-DUF2809
WP_014085455.1	*Flavobacterium branchiophilum*	Skillet-DUF2809
MBQ7045817.1	*Clostridia bacterium*	Skillet-DUF2809
MBE0685966.1	*Anaerolineaceae bacterium*	Skillet-DUF2809
MCD6375012.1	*Caldisericaceae bacterium*	Skillet-DUF2809
RZL17224.1	*Pedobacter sp.*	Skillet-DUF2809
WP_123610761.1		Skillet-DUF2809
WP_135102207.1	*Cryobacterium sp. TMT3-29-2*	Skillet-DUF2809
WP_018615377.1	*Segetibacter koreensis DSM 18137*	Skillet-DUF2809
WP_226639013.1	*Priestia flexa*	Skillet-DUF2809
WP_125154709.1	*Clostridium rectalis*	Skillet-DUF2809
MBJ7722230.1		Skillet-DUF2809
WP_131888061.1	*Priestia flexa*	Skillet-DUF2809
MBP9761269.1	*Candidatus Magasanikiibacteriota bacterium*	Skillet-DUF2809
MBQ8164958.1	*Clostridia bacterium*	Skillet-DUF2809
WP_161594057.1	*Microbacterium sp. RG1*	Skillet-DUF2809
MCB1061378.1	*Verrucomicrobiia bacterium*	Skillet-DUF2809
WP_112383127.1	*Sphingomonas carotinifaciens*	Skillet-DUF2809
NLP12740.1	*bacterium*	Skillet-DUF2809
RYD45767.1	*Verrucomicrobiaceae bacterium*	Skillet-DUF2809
MBK7929053.1	*Bryobacterales bacterium*	Skillet-DUF2809
WP_226080678.1	*Mycetocola spongiae*	Skillet-DUF2809
NQZ58790.1	*Lentisphaeraceae bacterium*	Skillet-DUF2809
MBR4510670.1	*Ruminococcus sp.*	Skillet-DUF2809
NES87516.1	*Moorena sp. SIO2B7*	Skillet-DUF2809
MBK8014591.1	*Deltaproteobacteria bacterium*	Skillet-DUF2809
WP_096237981.1	*Janthinobacterium svalbardensis*	Skillet-DUF2809
WP_012417457.1	*Bordetella avium*	Skillet-DUF2809
WP_100788265.1	*Leptospira adleri*	Skillet-DUF2809
NJM38439.1	*Akkermansiaceae bacterium*	Skillet-DUF2809
MBO4523128.1	*Ruminococcus sp.*	Skillet-DUF2809
MCB0274004.1	*Calditrichota bacterium*	Skillet-DUF2809
MBN1997907.1	*Candidatus Zhuqueibacterota bacterium*	Skillet-DUF2809
WP_234638246.1	*Delftia tsuruhatensis*	Skillet-DUF2809
MCD4832363.1	*Bacteroidales bacterium*	Skillet-DUF2809
MPV88944.1	*Georgenia ruanii*	Skillet-DUF2809
WP_106381427.1	*Abditibacterium utsteinense*	Skillet-DUF2809
MBL7852704.1	*Cyclobacteriaceae bacterium*	Skillet-DUF2809
MBN2801956.1	*Deltaproteobacteria bacterium*	Skillet-DUF2809
WP_105549487.1	*Arthrobacter sp. MYb227*	Skillet-DUF2809
WP_090712858.1	*Mycetocola miduiensis*	Skillet-DUF2809
MCA9246989.1	*Planctomycetales bacterium*	Skillet-DUF2809
MCA9299187.1	*Phycisphaerales bacterium*	Skillet-DUF2809
MBQ3523381.1	*Clostridia bacterium*	Skillet-DUF2809
WP_105328540.1	*Bremerella cremea*	Skillet-DUF2809
MCC6127103.1	*Pirellulales bacterium*	Skillet-DUF2809
WP_206603262.1	*Leptolyngbya ohadii IS1*	Skillet-DUF2809
WP_225216011.1		Skillet-DUF2809
HEM60891.1	*Chloroflexota bacterium*	Skillet-DUF2809
MBS7369957.1	*Oscillospiraceae bacterium*	Skillet-DUF2809
NIL95769.1	*Planctomycetales bacterium*	Skillet-DUF2809
MBI5086925.1	*Acidobacteriota bacterium*	Skillet-DUF2809
MBQ9839957.1	*Oscillospiraceae bacterium*	Skillet-DUF2809
MBP0029499.1	*Roseofilum sp. Guam*	Skillet-DUF2809
NLN34181.1	*Flavobacteriaceae bacterium*	Skillet-DUF2809
MBU3684295.1	*Phycisphaerales bacterium*	Skillet-DUF2809
WP_219873406.1	*Paenibacillus oenotherae*	Skillet-DUF2809
WP_073156208.1	*Seinonella peptonophila*	Skillet-DUF2809
WP_216848979.1	*Pedobacter sp. L105*	Skillet-DUF2809
WP_202924267.1	*Cellulomonas sp. H30R-01*	Skillet-DUF2809
MBX2850952.1	*Phycisphaeraceae bacterium*	Skillet-DUF2809
WP_146617094.1	*Actinoplanes lutulentus*	Skillet-DUF2809
MBG7609603.1	*Anaerolineae bacterium*	Skillet-DUF2809
MBW8002043.1	*Planctomycetota bacterium*	Skillet-DUF2809
MBI4918334.1	*archaeon*	Skillet-DUF2809
GGP97658.1	*Streptomyces griseomycini*	Skillet-DUF2809
WP_138729349.1	*Pedobacter helvus*	Skillet-DUF2809
WP_232073916.1	*Spirosoma aureum*	Skillet-DUF2809
MBK8420402.1	*Candidatus Villigracilis saccharophilus*	Skillet-DUF2809
WP_205125531.1	*Flammeovirga kamogawensis*	Skillet-DUF2809
WP_119050750.1	*Paraflavitalea soli*	Skillet-DUF2809
WP_043211890.1	*Bordetella pseudohinzii*	Skillet-DUF2809
OIO72858.1	*Zetaproteobacteria bacterium CG1_02_49_23*	Skillet-DUF2809
EOS51963.1	*Lachnospiraceae bacterium MD335*	Skillet-DUF2809
WP_152242704.1	*Janthinobacterium sp. FT58W*	Skillet-DUF2809
WP_183003786.1	*Achromobacter sp. UMC71*	Skillet-DUF2809
MBL7075360.1	*Candidatus Zhuqueibacterota bacterium*	Skillet-DUF2809
KPV48972.1	*Kouleothrix aurantiaca*	Skillet-DUF2809
WP_153021784.1	*Luteimicrobium xylanilyticum*	Skillet-DUF2809
WP_166578200.1		Skillet-DUF2809
NUO78726.1	*Candidatus Zhuqueibacterota bacterium*	Skillet-DUF2809
WP_170034991.1	*Longimicrobium terrae*	Skillet-DUF2809
WP_201498435.1	*Psychrobacter arenosus*	Skillet-DUF2809
MBK8397356.1	*Leptospiraceae bacterium*	Skillet-DUF2809
MBU6365939.1	*Gemmatimonadota bacterium*	Skillet-DUF2809
TMM37863.1	*Actinomycetota bacterium*	Skillet-DUF2809
MBR5512411.1	*Ruminococcus sp.*	Skillet-DUF2809
WP_074607873.1		Skillet-DUF2809
MBL8982526.1	*Gemmatimonadota bacterium*	Skillet-DUF2809

MBD3224348.1	*Caldithrix sp.*	Skillet-DUF2809
WP_136736879.1	*Thalassotalea mangrovi*	Skillet-DUF2809
MBQ8960561.1	*Ruminococcus sp.*	Skillet-DUF2809
MBU5617229.1	*Psychrobacter sp. TAE2020*	Skillet-DUF2809
WP_052387240.1	*Dactylosporangium aurantiacum*	Skillet-DUF2809
WP_226891981.1	*Paraclostridium bifermentans*	Skillet-DUF2809
MBE6805670.1	*Oscillospiraceae bacterium*	Skillet-DUF2809
WP_233105853.1	*[Clostridium] innocuum*	Skillet-DUF2809
HHK71285.1		Skillet-DUF2809
WP_216120860.1	*Clostridium estertheticum*	Skillet-DUF2809
WP_089281178.1	*Anaerovirgula multivorans*	Skillet-DUF2809
MBN1760206.1	*Chitinispirillaceae bacterium*	Skillet-DUF2809
WP_094345309.1	*Nostoc sp.*	Skillet-DUF2809
WP_161596753.1	*Chitinophaga vietnamensis*	Skillet-DUF2809
MBE5888394.1	*Lachnospiraceae bacterium*	Skillet-DUF2809
MBN2015312.1	*Candidatus Dojkabacteria bacterium*	Skillet-DUF2809
HCJ42176.1	*Ruminococcus sp.*	Skillet-DUF2809
WP_093139375.1	*Pseudoxanthomonas sp. GM95*	Skillet-DUF2809
MCC2669773.1	*Armatimonadota bacterium*	Skillet-DUF2809
NRH21466.1	*Candidatus Altimarinota bacterium*	Skillet-DUF2809
WP_130636978.1	*Alteromonas sp. KUL49*	Skillet-DUF2809
HGI22390.1		Skillet-DUF2809
WP_200356486.1	*Pelagicoccus mobilis*	Skillet-DUF2809
MSU85166.1	*Pedosphaera sp.*	Skillet-DUF2809
WP_216117099.1	*Clostridium gasigenes*	Skillet-DUF2809
MCA8976605.1	*Planctomycetota bacterium*	Skillet-DUF2809
WP_198988368.1	*Hymenobacter sp. BT559*	Skillet-DUF2809
MBI1764280.1		Skillet-DUF2809
TWT83080.1	*Novipirellula herctigrandis*	Skillet-DUF2809
WP_183739418.1	*Janthinobacterium sp. S3T4*	Skillet-DUF2809
NVL92267.1	*Desulfobacterales bacterium*	Skillet-DUF2809
WP_008507654.1	*Mucilaginibacter paludis DSM 18603*	Skillet-DUF2809
RWX48840.1	*Candidatus Electrothrix marina*	Skillet-DUF2809
WP_056670602.1	*Rhizobacter sp. Root1221*	Skillet-DUF2809
KPL91633.1	*Herpetosiphon geysericola*	Skillet-DUF2809
MBL8231904.1	*Bryobacterales bacterium*	Skillet-DUF2809
WP_146729347.1		Skillet-DUF2809
MBO4696025.1	*Lachnospiraceae bacterium*	Skillet-DUF2809
WP_094442120.1	*Janthinobacterium sp. PC23-8*	Skillet-DUF2809
NOQ97619.1	*Calditrichia bacterium*	Skillet-DUF2809
MBO4846317.1	*Lachnospiraceae bacterium*	Skillet-DUF2809
NTU80015.1	*Chloroflexales bacterium*	Skillet-DUF2809
NBW10589.1	*Caulobacteraceae bacterium*	Skillet-DUF2809
WP_138321163.1	*Thalassotalea litorea*	Skillet-DUF2809
WP_102293550.1		Skillet-DUF2809
WP_123308462.1	*Cellulomonas sp. PhB143*	Skillet-DUF2809
MCD6588192.1	*Candidatus Fermentibacteraceae bacterium*	Skillet-DUF2809
MBT3229628.1	*Fidelibacterota bacterium*	Skillet-DUF2809
WP_095382220.1		Skillet-DUF2809
MBO7402242.1	*Lachnospiraceae bacterium*	Skillet-DUF2809
MBS5932675.1	*Clostridiales bacterium*	Skillet-DUF2809
MBQ3357036.1	*Oscillospiraceae bacterium*	Skillet-DUF2809
WP_214122161.1	*Curvibacter sp. CHRR-16*	Skillet-DUF2809
NOY06652.1	*Chlorobiota bacterium*	Skillet-DUF2809
BAP56383.1	*Thioploca ingrica*	Skillet-DUF2809
WP_196887810.1	*Aureivirga sp. CE67*	Skillet-DUF2809
WP_122012348.1		Skillet-DUF2809
WP_083558583.1	*Flavobacterium johnsoniae*	Skillet-DUF2809
MBE6598413.1	*Oscillospiraceae bacterium*	Skillet-DUF2809
MBN2349084.1	*Bacteroidales bacterium*	Skillet-DUF2809
MBE4060346.1	*Vibrio parahaemolyticus*	Skillet-DUF2809
WP_112882388.1	*Paenibacillus montanisoli*	Skillet-DUF2809
WP_078711700.1	*Anaerorhabdus furcosa*	Skillet-DUF2809
PFG37201.1	*Flavimobilis soli*	Skillet-DUF2809
MCD8043972.1	*Tannerellaceae bacterium*	Skillet-DUF2809
WP_099155640.1	*Flavilitoribacter nigricans DSM 23189 = NBRC 102662*	Skillet-DUF2809
NEP62657.1	*Symploca sp. SIO2G7*	Skillet-DUF2809
MBT4601846.1	*Bacteroidota Order II. bacterium*	Skillet-DUF2809
WP_168882335.1	*Flammeovirga agarivorans*	Skillet-DUF2809
WP_227570802.1	*Cellulomonas chengniuliangii*	Skillet-DUF2809
MBQ8687624.1	*Ruminococcus sp.*	Skillet-DUF2809
MBK8848147.1	*Bacteroidota bacterium*	Skillet-DUF2809
MBQ8515731.1	*Ruminococcus sp.*	Skillet-DUF2809
WP_124586366.1	*Pedobacter sp. KBW06*	Skillet-DUF2809
WP_106456679.1	*Aphanothece hegewaldii CCALA 016*	Skillet-DUF2809
WP_199181995.1	*Siphonobacter sp. BAB-5405*	Skillet-DUF2809
MBP1649040.1	*Bacteroidota bacterium*	Skillet-DUF2809
WP_190593823.1	*Arthrospira platensis FACHB-439*	Skillet-DUF2809
MBQ3915893.1	*Ruminococcus sp.*	Skillet-DUF2809
MBT3424093.1	*Bacteroidota bacterium*	Skillet-DUF2809
WP_106309485.1	*Chamaesiphon polymorphus CCALA 037*	Skillet-DUF2809
WP_190715941.1	*Oscillatoria sp. FACHB-1406*	Skillet-DUF2809
WP_017290015.1	*Leptolyngbya boryana IU 594*	Skillet-DUF2809
MBQ7800333.1	*Oscillospiraceae bacterium*	Skillet-DUF2809
WP_097055025.1	*Salinimicrobium sediminis*	Skillet-DUF2809
WP_103126733.1	*Nostoc cycadae WK-1*	Skillet-DUF2809
RYY60140.1	*Chitinophagaceae bacterium*	Skillet-DUF2809
NJR37870.1	*Leptolyngbyaceae cyanobacterium CSU_1_4*	Skillet-DUF2809
WP_003441827.1	*Clostridium pasteurianum*	Skillet-DUF2809
RYZ45543.1	*Chitinophagaceae bacterium*	Skillet-DUF2809
WP_112746062.1	*Pseudochryseolinea flava*	Skillet-DUF2809
WP_002687208.1	*Bergeyella zoohelcum*	Skillet-DUF2809
WP_129535415.1	*Chryseobacterium sp. 3008163*	Skillet-DUF2809

WP_214830163.1	*Chryseobacterium sp. ISL-6*	Skillet-DUF2809
MBQ5764976.1	*Clostridia bacterium*	Skillet-DUF2809
WP_076757951.1	*Bacillus swezeyi*	Skillet-DUF2809
WP_135088084.1	*Sphingomonas parva*	Skillet-DUF2809
WP_101238729.1	*Chryseobacterium sp. PMSZPI*	Skillet-DUF2809
WP_084815986.1	*Frondihabitans sp. Leaf304*	Skillet-DUF2809
MCB0521189.1	*Saprospiraceae bacterium*	Skillet-DUF2809
MBX3593544.1	*Sphingomonas sp.*	Skillet-DUF2809
MBL7847424.1	*Cyclobacteriaceae bacterium*	Skillet-DUF2809
MCB0738609.1	*Bacteroidota bacterium*	Skillet-DUF2809
MCA0398331.1	*Bacteroidota bacterium*	Skillet-DUF2809
MBU0765750.1	*Bacteroidota bacterium*	Skillet-DUF2809
WP_224724284.1	*Paenibacillus vietnamensis*	Skillet-DUF2809
OBZ13494.1	*Bacillus sp. FJAT-26390*	Skillet-DUF2809
WP_028395800.1		Skillet-DUF2809
RZA03721.1	*Sphingobacteriaceae bacterium*	Skillet-DUF2809
WP_190351254.1		Skillet-DUF2809
WP_021778094.1	*Dokdonia sp. MED134*	Skillet-DUF2809
NRA29907.1	*Parvularculaceae bacterium*	Skillet-DUF2809
OMF18865.1	*Paenibacillus sp. FSL H8-0548*	Skillet-DUF2809
MCC8146499.1	*Bacteroidales bacterium*	Skillet-DUF2809
WP_015808198.1	*Pedobacter heparinus DSM 2366*	Skillet-DUF2809
MBR2328038.1	*Clostridia bacterium*	Skillet-DUF2809
MBL7769438.1	*Flavipsychrobacter sp.*	Skillet-DUF2809
MBN2611700.1	*Bacteroidales bacterium*	Skillet-DUF2809
WP_007127779.1	*Paenibacillus lactis*	Skillet-DUF2809
MBN1598049.1	*Bacteroidales bacterium*	Skillet-DUF2809
MBN1184721.1	*Bacteroidales bacterium*	Skillet-DUF2809
WP_106396380.1	*Actinocorallia populi*	Skillet-DUF2809
WP_226671599.1	*Metabacillus litoralis*	Skillet-DUF2809
CDZ24383.1	*[Clostridium] cellulosi*	Skillet-DUF2809
NLK87397.1	*Clostridiaceae bacterium*	Skillet-DUF2809
NQV19592.1	*Armatimonadota bacterium*	Skillet-DUF2809
WP_127507151.1	*Actinoplanes solisilvae*	Skillet-DUF2809
WP_066309445.1	*Aquimarina aggregata*	Skillet-DUF2809
UCD38012.1	*Fidelibacterota bacterium*	Skillet-DUF2809
MBW2342795.1	*Deltaproteobacteria bacterium*	Skillet-DUF2809
MBW2059905.1	*Deltaproteobacteria bacterium*	Skillet-DUF2809
MBO4749294.1	*Lachnospiraceae bacterium*	Skillet-DUF2809
MCA9664889.1	*Myxococcales bacterium*	Skillet-DUF2809
WP_052875173.1	*Clostridium botulinum C/D*	Skillet-DUF2809
PKN90580.1	*Chloroflexi bacterium HGW-Chloroflexi-6*	Skillet-DUF2809
TFH13127.1	*Lentisphaerales bacterium*	Skillet-DUF2809
OQY58690.1	*Desulfobacteraceae bacterium 4572_88*	Skillet-DUF2809
MBM3758036.1	*Acidobacteriota bacterium*	Skillet-DUF2809
RZM02481.1	*Variovorax sp.*	Skillet-DUF2809
OGU06512.1	*Geobacteraceae bacterium GWC2_58_44*	Skillet-DUF2809
MBA3314179.1	*Planctomycetaceae bacterium*	Skillet-DUF2809
WP_072830733.1	*Clostridium collagenovorans DSM 3089*	Skillet-DUF2809
MCA9731061.1	*Candidatus Zhuqueibacterota bacterium*	Skillet-DUF2809
WP_220613715.1	*Hymenobacter sp. HSC-4F20*	Skillet-DUF2809
MBC7808368.1	*Armatimonadota bacterium*	Skillet-DUF2809
NJL60920.1	*Candidatus Methylacidiphilales bacterium*	Skillet-DUF2809
WP_153286392.1	*Streptomyces fagopyri*	Skillet-DUF2809
PIQ23728.1	*bacterium (Candidatus Blackallbacteria) CG18_big_fil_WC_8_21_14_2_50_49_26*	Skillet-DUF2809
MBF0548751.1	*Candidatus Rifleibacteriota bacterium*	Skillet-DUF2809
WP_028294067.1	*Oceanobacter kriegii DSM 6294*	Skillet-DUF2809
MBM3213217.1	*Candidatus Poribacteria bacterium*	Skillet-DUF2809
WP_051799901.1	*Catenuloplanes japonicus*	Skillet-DUF2809
WP_116125853.1	*Lewinella sp. IMCC34183*	Skillet-DUF2809
MBL7737963.1	*Chitinophagaceae bacterium*	Skillet-DUF2809
HAT4252095.1	*Clostridium perfringens*	Skillet-DUF2809
HAT4315177.1	*Clostridium perfringens*	Skillet-DUF2809
EGT3599705.1	*Clostridium perfringens*	Skillet-DUF2809
NLJ07355.1	*Sphingobacteriales bacterium*	Skillet-DUF2809
MBQ9394993.1	*Pseudomonadota bacterium*	Skillet-DUF2809
WP_099313357.1	*Clostridium paraputrificum*	Skillet-DUF2809
WP_096379634.1		Skillet-DUF2809
RYZ55727.1	*Sphingobacteriales bacterium*	Skillet-DUF2809
WP_160686747.1	*Clostridium sp. C2-6-12*	Skillet-DUF2809
MBK9990652.1	*Verrucomicrobiota bacterium*	Skillet-DUF2809
MAH13145.1	*Verrucomicrobiota bacterium*	Skillet-DUF2809
WP_048354511.1	*Bacillus glycinifermentans*	Skillet-DUF2809
MBT5692694.1	*Opitutia bacterium*	Skillet-DUF2809
RKY81352.1	*Candidatus Zhuqueibacterota bacterium*	Skillet-DUF2809
GIF67635.1	*Asanoa ishikariensis*	Skillet-DUF2809
WP_148315565.1	*Clostridium culturomicium*	Skillet-DUF2809
MBN2091890.1	*Candidatus Zhuqueibacterota bacterium*	Skillet-DUF2809
MBM3974147.1	*Planctomycetota bacterium*	Skillet-DUF2809
MBX9735825.1	*Phycisphaerales bacterium*	Skillet-DUF2809
CAG0942864.1	*Candidatus Brocadiaceae bacterium*	Skillet-DUF2809
HAG14751.1	*Ruminococcus sp.*	Skillet-DUF2809
MCD6117512.1	*bacterium*	Skillet-DUF2809
KPK29800.1	*Nitrospira bacterium SG8_3*	Skillet-DUF2809
WP_200463124.1	*uncultured Carboxylicivirga sp.*	Skillet-DUF2809
WP_095837827.1	*Chitinophaga pendula*	Skillet-DUF2809
WP_134290221.1	*Psychromonas sp. RZ5*	Skillet-DUF2809
MBQ0083003.1	*Candidatus Colousia faecequi*	Skillet-DUF2809
WP_170137718.1	*Chitinophaga dinghuensis*	Skillet-DUF2809
MBL8240673.1	*Bryobacterales bacterium*	Skillet-DUF2809
WP_157950089.1	*Vallitalea okinawensis*	Skillet-DUF2809
WP_073565103.1	*Archangium sp. Cb G35*	Skillet-DUF2809
TYR81276.1	*Priestia megaterium*	Skillet-DUF2809

PZQ33631.1	*Stenotrophomonas acidaminiphila*	Skillet-DUF2809
MBV8253298.1	*Chitinophaga sp.*	Skillet-DUF2809
WP_045745901.1	*Actinoplanes sp. NBRC 101535*	Skillet-DUF2809
RYY65853.1	*Chitinophagaceae bacterium*	Skillet-DUF2809
OYU80855.1	*Flavobacterium sp. BFFFF1*	Skillet-DUF2809
NUN10706.1	*Ignavibacteriaceae bacterium*	Skillet-DUF2809
WP_016763522.1	*Priestia megaterium*	Skillet-DUF2809
WP_137937372.1	*Chitinivorax sp. B*	Skillet-DUF2809
WP_207977704.1		Skillet-DUF2809
WP_216302967.1	*Clostridium sp. CF011*	Skillet-DUF2809
NEQ54755.1	*Leptolyngbya sp. SIO3F4*	Skillet-DUF2809
WP_146950931.1	*Metabacillus litoralis*	Skillet-DUF2809
MBM4274625.1	*Deltaproteobacteria bacterium*	Skillet-DUF2809
MBP5661525.1	*Clostridia bacterium*	Skillet-DUF2809
WP_106231591.1	*Orenia metallireducens*	Skillet-DUF2809
NLE00885.1	*Fibrobacter sp.*	Skillet-DUF2809
MBQ5820352.1	*Clostridia bacterium*	Skillet-DUF2809
MBE6710472.1	*Oscillospiraceae bacterium*	Skillet-DUF2809
UJP11578.1	*Microbacterium sp. KUDC0406*	Skillet-DUF2809
MCC6241457.1	*Gemmatimonadaceae bacterium*	Skillet-DUF2809
WP_167057848.1	*Paenibacillus aceris*	Skillet-DUF2809
MCB5284778.1	*Candidatus Cloacimonadota bacterium*	Skillet-DUF2809
WP_160165868.1	*Actinoplanes sp. OR16*	Skillet-DUF2809
MBA3985941.1	*Flavobacteriales bacterium*	Skillet-DUF2809
MBL8981062.1	*Gemmatimonadota bacterium*	Skillet-DUF2809
TDO92503.1	*Enemella evansiae*	Skillet-DUF2809
QCW98616.1	*Aggregatimonas sangjinii*	Skillet-DUF2809
UCF40930.1	*Gemmatimonadota bacterium*	Skillet-DUF2809
WP_154832624.1	*Xanthomonas arboricola*	Skillet-DUF2809
WP_143048627.1	*Frigoribacterium sp. MCBA15_019*	Skillet-DUF2809
MBR6682043.1	*Clostridia bacterium*	Skillet-DUF2809
WP_171684968.1	*Paenibacillus planticolens*	Skillet-DUF2809
MBS1272491.1	*Fidelibacterota bacterium*	Skillet-DUF2809
WP_185815979.1	*Xanthomonas sp. SS*	Skillet-DUF2809
MBP8083594.1	*Spirochaetota bacterium*	Skillet-DUF2809
MBQ9947152.1	*Oscillospiraceae bacterium*	Skillet-DUF2809
WP_108464580.1	*Paenibacillus sp. CAA11*	Skillet-DUF2809
CAA6804757.1	*uncultured Thiotrichaceae bacterium*	Skillet-DUF2809
MBC7534982.1	*Ferruginibacter sp.*	Skillet-DUF2809
WP_082218162.1	*Dyadobacter psychrophilus*	Skillet-DUF2809
WP_197936170.1	*Paenibacillus sp. GSMTC-2017*	Skillet-DUF2809
WP_019974589.1	*Empedobacter brevis*	Skillet-DUF2809
RZK75640.1	*Pedobacter sp.*	Skillet-DUF2809
WP_133231976.1		Skillet-DUF2809
MBP6772705.1	*Gemmatimonadaceae bacterium*	Skillet-DUF2809
MBR5221757.1	*Clostridia bacterium*	Skillet-DUF2809
WP_194129619.1	*Pedobacter sp. MC2016-24*	Skillet-DUF2809
WP_055774370.1	*Sphingomonas sp. Leaf357*	Skillet-DUF2809
GGP17111.1	*Nonomuraea glycinis*	Skillet-DUF2809
MBN9418893.1	*Vulcanimicrobiota bacterium*	Skillet-DUF2809
MBQ3002869.1	*Clostridia bacterium*	Skillet-DUF2809
MBL8985506.1	*Gemmatimonadota bacterium*	Skillet-DUF2809
NIM59226.1	*Candidatus Aminicenantes bacterium*	Skillet-DUF2809
NQX28165.1	*Microbacteriaceae bacterium VKM Ac-2854*	Skillet-DUF2809
MCB5246851.1	*Candidatus Cloacimonadota bacterium*	Skillet-DUF2809
MBK7946543.1	*Flavobacteriales bacterium*	Skillet-DUF2809
MBQ3598769.1	*Clostridia bacterium*	Skillet-DUF2809
WP_094411528.1	*Flavobacterium cyanobacteriorum*	Skillet-DUF2809
MBQ3141176.1	*Clostridia bacterium*	Skillet-DUF2809
CAH0136839.1	*Chryseobacterium sp. Bi04*	Skillet-DUF2809
CDA90140.1	*Ruminococcus sp. CAG:563*	Skillet-DUF2809
MBE6551717.1		Skillet-DUF2809
TSE03898.1	*Aquimarina algiphila*	Skillet-DUF2809
MBR3067011.1	*Prevotella sp.*	Skillet-DUF2809
MCC7054614.1	*Gemmatimonadaceae bacterium*	Skillet-DUF2809
WP_155113084.1	*Metabacillus mangrovi*	Skillet-DUF2809
MBN8685747.1	*Chitinophagales bacterium*	Skillet-DUF2809
MCE3282695.1	*Chitinophagaceae bacterium*	Skillet-DUF2809
WP_160427037.1	*Agromyces seonyuensis*	Skillet-DUF2809
MBP9213711.1	*Chitinophagaceae bacterium*	Skillet-DUF2809
WP_028489994.1	*Thiothrix lacustris*	Skillet-DUF2809
MBL0358081.1	*Chitinophagaceae bacterium*	Skillet-DUF2809
SNY66403.1	*Paractinoplanes atraurantiacus*	Skillet-DUF2809
WP_184833570.1	*Allocatelliglobosispora scoriae*	Skillet-DUF2809
RYY55032.1	*Chitinophagaceae bacterium*	Skillet-DUF2809
MBC7936810.1	*Ferruginibacter sp.*	Skillet-DUF2809
WP_117361496.1	*Actinomadura logoneensis*	Skillet-DUF2809
CUI02680.1	*Janthinobacterium sp. CG23_2*	Skillet-DUF2809
WP_207632826.1	*Foetidibacter luteolus*	Skillet-DUF2809
NUP09751.1	*Polyangiaceae bacterium*	Skillet-DUF2809
WP_212214652.1	*Carboxylicivirga linearis*	Skillet-DUF2809
WP_150415336.1	*Ginsengibacter hankyongi*	Skillet-DUF2809
KYF74242.1	*Sorangium cellulosum*	Skillet-DUF2809
WP_228913823.1	*Salinimicrobium sediminilitoris*	Skillet-DUF2809
WP_099192562.1	*Tepidibacter mesophilus*	Skillet-DUF2809
WP_029033386.1	*Salinimicrobium terrae DSM 17865*	Skillet-DUF2809
MBQ8791929.1	*Ruminiclostridium sp.*	Skillet-DUF2809
PLX22993.1	*Salinivirgaceae bacterium*	Skillet-DUF2809
WP_105336222.1	*Blastopirellula marina*	Skillet-DUF2809
WP_184193224.1	*Armatimonas rosea*	Skillet-DUF2809
WP_024861324.1	*Ruminococcus flavefaciens*	Skillet-DUF2809
WP_021754505.1	*Leifsonia xyli subsp. cynodontis DSM 46306*	Skillet-DUF2809
MBQ0101556.1	*Candidatus Colimorpha enterica*	Skillet-DUF2809

WP_029419460.1	*Bacillus sonorensis*	Skillet-DUF2809
WP_163217006.1		Skillet-DUF2809
WP_203686405.1	*Phytomonospora endophytica*	Skillet-DUF2809
MBN2169776.1	*Candidatus Krumholzibacteriota bacterium*	Skillet-DUF2809
MBQ9816896.1	*Pseudomonadota bacterium*	Skillet-DUF2809
PSR13542.1	*Bacteroidota bacterium*	Skillet-DUF2809
MBR4362105.1	*Ruminococcus sp.*	Skillet-DUF2809
WP_015392111.1	*Clostridium saccharoperbutylacetonicum*	Skillet-DUF2809
MBE9491556.1	*Bacteroidota bacterium*	Skillet-DUF2809
MBE9031725.1	*Romeriopsis navalis LEGE 11480*	Skillet-DUF2809
MBD5160079.1	*Ruminococcus sp.*	Skillet-DUF2809
MCD4682057.1	*Bacteroidales bacterium*	Skillet-DUF2809
WP_109617367.1	*Sediminitomix flava*	Skillet-DUF2809
WP_046586675.1	*Bacillus sp. SA1-12*	Skillet-DUF2809
WP_037344585.1	*Ruminococcus sp. FC2018*	Skillet-DUF2809
WP_102231505.1		Skillet-DUF2809
WP_037300494.1	*Ruminococcus flavefaciens 007c*	Skillet-DUF2809
MCE2939966.1	*Fimbriimonadaceae bacterium*	Skillet-DUF2809
MBI4645558.1	*Bacteroidia bacterium*	Skillet-DUF2809
WP_120489352.1	*Bergeyella cardium*	Skillet-DUF2809
WP_006599672.1	*Pseudoramibacter alactolyticus*	Skillet-DUF2809
WP_146867517.1	*Chitinophaga cymbidii*	Skillet-DUF2809
WP_209524307.1	*Vagococcus allomyrinae*	Skillet-DUF2809
WP_089912608.1	*Chitinophaga rupis*	Skillet-DUF2809
WP_167297098.1	*Pedobacter sp. SG918*	Skillet-DUF2809
WP_019136462.1	*Cellulomonas massiliensis*	Skillet-DUF2809
UCE22833.1	*Candidatus Aminicenantes bacterium*	Skillet-DUF2809
RYY32827.1	*Sphingomonadales bacterium*	Skillet-DUF2809
WP_075600812.1	*Leptolyngbya sp. 'hensonii'*	Skillet-DUF2809
MBK8803108.1	*Fibrobacterota bacterium*	Skillet-DUF2809
MBQ0151960.1	*Candidatus Chryseobacterium enterohippi*	Skillet-DUF2809
PQL90682.1	*Apibacter adventoris*	Skillet-DUF2809
MBQ2768105.1	*Clostridia bacterium*	Skillet-DUF2809
MBQ4604422.1	*Clostridia bacterium*	Skillet-DUF2809
MBE9598797.1	*Pedobacter sp. MC2016-24*	Skillet-DUF2809
MBQ7522555.1	*Clostridia bacterium*	Skillet-DUF2809
MCB1174533.1	*Leptospiraceae bacterium*	Skillet-DUF2809
WP_136838133.1	*Pedobacter polaris*	Skillet-DUF2809
WP_133574719.1	*Pedobacter metabolipauper*	Skillet-DUF2809
MBR7184595.1	*Clostridia bacterium*	Skillet-DUF2809
MBR6701710.1	*Clostridia bacterium*	Skillet-DUF2809
MBO4219596.1	*Clostridia bacterium*	Skillet-DUF2809
NCT16841.1	*Flavobacteriia bacterium*	Skillet-DUF2809
WP_091144007.1	*Flavobacterium caeni*	Skillet-DUF2809
MBP3938653.1	*Clostridia bacterium*	Skillet-DUF2809
MBN2717999.1	*Deltaproteobacteria bacterium*	Skillet-DUF2809
WP_194115574.1	*Chitinilyticum piscinae*	Skillet-DUF2809
NIM49128.1	*Gemmatimonadales bacterium*	Skillet-DUF2809
WP_211561435.1	*Metabacillus flavus*	Skillet-DUF2809
NLK95567.1	*Clostridiales bacterium*	Skillet-DUF2809
MBA4275579.1	*Flavobacterium sp.*	Skillet-DUF2809
WP_046352825.1	*Janthinobacterium sp. B9-8*	Skillet-DUF2809
MBE2187091.1	*Rhodothermales bacterium*	Skillet-DUF2809
WP_044037073.1	*Clostridium bornimense*	Skillet-DUF2809
WP_018353371.1	*Longispora albida DSM 44784*	Skillet-DUF2809
WP_184547622.1	*Mucilaginibacter sp. FT3.2*	Skillet-DUF2809
WP_188833618.1	*Mucilaginibacter rubeus*	Skillet-DUF2809
MBN1609711.1	*Polyangiaceae bacterium*	Skillet-DUF2809
MBK9714460.1	*Candidatus Ribeiella danica*	Skillet-DUF2809
MBK9945274.1	*Candidatus Kouleothrix ribensis*	Skillet-DUF2809
HAR64700.1	*Lentisphaeria bacterium*	Skillet-DUF2809
RSK49065.1	*Hymenobacter rigui*	Skillet-DUF2809
WP_124564610.1	*Ectobacillus antri*	Skillet-DUF2809
AMB58178.1	*Microterricola viridarii*	Skillet-DUF2809
KLU98814.1	*Photobacterium aphoticum*	Skillet-DUF2809
WP_181600613.1	*Clostridium intestinale*	Skillet-DUF2809
MBQ8170648.1	*Oscillospiraceae bacterium*	Skillet-DUF2809
MBU0679733.1	*Verrucomicrobiota bacterium*	Skillet-DUF2809
QLQ33372.1	*Candidatus Thiothrix singaporensis*	Skillet-DUF2809
WP_198650426.1	*Saccharospirillum mangrovi*	Skillet-DUF2809
WP_012158856.1	*Alkaliphilus oremlandii OhILAs*	Skillet-DUF2809
WP_190749142.1	*Leptolyngbya subtilissima ST-M1*	Skillet-DUF2809
MCB1327702.1	*Leptospiraceae bacterium*	Skillet-DUF2809
WP_104561132.1	*Xanthomonas arboricola pv. guizotiae*	Skillet-DUF2809
WP_087016454.1	*Thaumasiovibrio subtropicus*	Skillet-DUF2809
RKZ53530.1	*Candidatus Parabeggiatoa sp. nov. 3*	Skillet-DUF2809
WP_205729312.1	*Ferrimonas aestuarii*	Skillet-DUF2809
RYD58130.1	*Sphingomonadales bacterium*	Skillet-DUF2809
WP_137101597.1	*Phreatobacter aquaticus*	Skillet-DUF2809
PPC78677.1	*Pokkaliibacter plantistimulans*	Skillet-DUF2809
RYD71264.1	*Verrucomicrobiaceae bacterium*	Skillet-DUF2809
MBO5324199.1	*Oscillospiraceae bacterium*	Skillet-DUF2809
NLT77734.1	*Planctomycetota bacterium*	Skillet-DUF2809
RYZ04892.1	*Myxococcales bacterium*	Skillet-DUF2809
MBP8807935.1	*Kofleriaceae bacterium*	Skillet-DUF2809
NNE46635.1	*Rhodothermales bacterium*	Skillet-DUF2809
MBK7821668.1	*Tessaracoccus sp.*	Skillet-DUF2809
MBK6766768.1	*bacterium*	Skillet-DUF2809
WP_013598287.1	*Weeksella virosa*	Skillet-DUF2809
MCD7936198.1	*Tannerellaceae bacterium*	Skillet-DUF2809
WP_129718190.1	*Pedobacter sp. SYP-B3415*	Skillet-DUF2809
MBR2869105.1	*Clostridia bacterium*	Skillet-DUF2809
MBP3697681.1	*Clostridia bacterium*	Skillet-DUF2809

WP_222428677.1		Skillet-DUF2809
RZK61623.1	*Hymenobacter sp.*	Skillet-DUF2809
RZJ68089.1	*Flavobacterium sp.*	Skillet-DUF2809
WP_169523058.1	*Flavobacterium solisilvae*	Skillet-DUF2809
WP_129757926.1	*Flavobacterium dauae*	Skillet-DUF2809
WP_231834685.1	*Flavobacterium channae*	Skillet-DUF2809
MBL0043513.1	*Flavobacteriales bacterium*	Skillet-DUF2809
WP_209970503.1	*Paenibacillus eucommiae*	Skillet-DUF2809
WP_013919336.1	*Paenibacillus mucilaginosus*	Skillet-DUF2809
WP_057536551.1	*Paraclostridium sordellii*	Skillet-DUF2809
WP_116093052.1	*Chryseobacterium sp. 5_R23647*	Skillet-DUF2809
WP_117775746.1		Skillet-DUF2809
WP_080064499.1	*Ruminiclostridium hungatei*	Skillet-DUF2809
WP_074963180.1	*Ruminococcus albus*	Skillet-DUF2809
MBR6385812.1	*Ruminococcus sp.*	Skillet-DUF2809
MBI9036945.1	*Bacteroidales bacterium*	Skillet-DUF2809
WP_168548561.1	*Streptococcus ovuberis*	Skillet-DUF2809
WP_235325116.1	*Antarcticibacterium sp. 1MA-6-2*	Skillet-DUF2809
MBS2020521.1	*Deltaproteobacteria bacterium*	Skillet-DUF2809
MBA2545192.1	*Deltaproteobacteria bacterium*	Skillet-DUF2809
MBN1407881.1	*Calditrichaceae bacterium*	Skillet-DUF2809
MBL8142043.1	*Acidobacteriota bacterium*	Skillet-DUF2809
NOR45972.1	*Candidatus Delongbacteria bacterium*	Skillet-DUF2809
WP_169364967.1	*Pseudanabaena yagii GIHE-NHR1*	Skillet-DUF2809
MCB9081060.1	*Lewinellaceae bacterium*	Skillet-DUF2809
MBJ7428045.1	*Bacteroidia bacterium*	Skillet-DUF2809
WP_194765846.1	*Tamlana sp. I1*	Skillet-DUF2809
OYU94810.1	*Bacteroidetes bacterium B1(2017)*	Skillet-DUF2809
WP_150031856.1	*Taibaiella lutea*	Skillet-DUF2809
WP_014798834.1	*Bernardetia litoralis DSM 6794*	Skillet-DUF2809
WP_101301074.1	*Acetivibrio saccincola*	Skillet-DUF2809
WP_055862213.1		Skillet-DUF2809
MBN1402701.1	*Anaerolineae bacterium*	Skillet-DUF2809
MBU1822052.1	*Bacteroidota bacterium*	Skillet-DUF2809
WP_205174731.1		Skillet-DUF2809
WP_233313855.1	*Niallia taxi*	Skillet-DUF2809
WP_079667579.1	*Soonwooa buanensis*	Skillet-DUF2809
NJD18921.1	*Gemmatimonadota bacterium*	Skillet-DUF2809
WP_235118915.1	*Paenibacillus hexagrammi*	Skillet-DUF2809
WP_189605598.1	*Salinimicrobium marinum*	Skillet-DUF2809
MBY0491620.1	*Gemmatimonadaceae bacterium*	Skillet-DUF2809
MBP6385715.1	*Pseudarcicella sp.*	Skillet-DUF2809
MBL6963121.1	*Bacteroidota bacterium*	Skillet-DUF2809
MBQ7101758.1	*Clostridia bacterium*	Skillet-DUF2809
RYE94585.1	*Myxococcales bacterium*	Skillet-DUF2809
MBQ6818401.1	*Clostridia bacterium*	Skillet-DUF2809
MBQ7298668.1	*Clostridia bacterium*	Skillet-DUF2809
MBX3080595.1	*Anaerolineae bacterium*	Skillet-DUF2809
WP_105038323.1	*Flavipsychrobacter stenotrophus*	Skillet-DUF2809
RZK81993.1	*Pedobacter sp.*	Skillet-DUF2809
MBQ7982925.1	*Clostridia bacterium*	Skillet-DUF2809
MBR2901706.1	*Clostridia bacterium*	Skillet-DUF2809
OGM09108.1	*Candidatus Woesebacteria bacterium RBG_13_34_9*	Skillet-DUF2809
MBR6507161.1	*Clostridia bacterium*	Skillet-DUF2809
MBR4857950.1	*Clostridia bacterium*	Skillet-DUF2809
MBR5825858.1	*Clostridia bacterium*	Skillet-DUF2809
WP_196887540.1	*Aureivirga sp. CE67*	Skillet-DUF2809
MBC8002294.1	*Verrucomicrobiales bacterium*	Skillet-DUF2809
MBK7704471.1	*Acidobacteriota bacterium*	Skillet-DUF2809
KAF2412978.1	*Microbacterium sp. B35-04*	Skillet-DUF2809
WP_133531474.1	*Flavobacterium dankookense*	Skillet-DUF2809
MBO4530980.1	*Paludibacteraceae bacterium*	Skillet-DUF2809
WP_190376132.1	*Leptolyngbya sp. AS-A5*	Skillet-DUF2809
WP_091428762.1	*Flavobacterium degerlachei*	Skillet-DUF2809
MBM3796094.1	*Acidobacteriota bacterium*	Skillet-DUF2809
HHT96568.1	*Clostridiales bacterium*	Skillet-DUF2809
WP_183622598.1	*Pseudoclavibacter helvolus*	Skillet-DUF2809
WP_162458426.1	*Pseudactinotalea terrae*	Skillet-DUF2809
MBP5368354.1	*Bacteroidales bacterium*	Skillet-DUF2809
WP_099575960.1	*Caulobacter sp. FWC2*	Skillet-DUF2809
KSB89914.1	*Caulobacter vibrioides*	Skillet-DUF2809
WP_183987354.1	*Deinobacterium chartae*	Skillet-DUF2809
MBN2519435.1	*Bacteroidales bacterium*	Skillet-DUF2809
MBN2274370.1	*Bacteroidales bacterium*	Skillet-DUF2809
MBK6605149.1	*Leptospiraceae bacterium*	Skillet-DUF2809
WP_213490701.1	*Mucilaginibacter sp. Bleaf8*	Skillet-DUF2809
WP_191019585.1	*Flavobacterium selenitireducens*	Skillet-DUF2809
WP_157524703.1	*Mucilaginibacter ginkgonis*	Skillet-DUF2809
WP_131476476.1	*Flavobacterium silvisoli*	Skillet-DUF2809
MBK8455505.1	*Thiofilum sp.*	Skillet-DUF2809
WP_020559389.1	*Thiofilum flexile DSM 14609*	Skillet-DUF2809
WP_086940606.1		Skillet-DUF2809
WP_083954287.1	*Sphingomonas koreensis*	Skillet-DUF2809
MBA4133196.1	*Flavobacterium sp.*	Skillet-DUF2809
QDQ03456.1	*Lysinibacillus fusiformis*	Skillet-DUF2809
MBP3460520.1	*Lachnospiraceae bacterium*	Skillet-DUF2809
WP_116752145.1	*Flavobacterium sp. HTF*	Skillet-DUF2809
KQO23028.1	*Flavobacterium sp. Leaf82*	Skillet-DUF2809
WP_115991351.1	*Cohnella lupini*	Skillet-DUF2809
WP_113989829.1		Skillet-DUF2809
RZK61073.1	*Hymenobacter sp.*	Skillet-DUF2809
WP_184646587.1	*Sphingopyxis sp. JAI128*	Skillet-DUF2809
WP_155181064.1	*Fulvivirga lutimaris*	Skillet-DUF2809

MBL8175389.1	*Bryobacterales bacterium*	Skillet-DUF2809
WP_188810203.1	*Hymenobacter cavernae*	Skillet-DUF2809
WP_028454507.1	*Chitinilyticum litopenaei DSM 21440*	Skillet-DUF2809
MBY0374827.1	*Bryobacteraceae bacterium*	Skillet-DUF2809
MBD5541087.1	*Lachnospiraceae bacterium*	Skillet-DUF2809
MBI3230901.1	*Burkholderiales bacterium*	Skillet-DUF2809
WP_004623256.1	*Ruminiclostridium cellobioparum subsp. termitidis CT1112*	Skillet-DUF2809
MBX7221605.1	*Blastocatellia bacterium*	Skillet-DUF2809
MBQ1172780.1	*Lachnospiraceae bacterium*	Skillet-DUF2809
MCA0426291.1	*Bacteroidota bacterium*	Skillet-DUF2809
WP_111595836.1	*Chitinophaga skermanii*	Skillet-DUF2809
WP_014254256.1	*Acetivibrio clariflavus*	Skillet-DUF2809
WP_010251080.1	*Acetivibrio cellulolyticus CD2*	Skillet-DUF2809
NLM48329.1	*Candidatus Epulonipiscium sp.*	Skillet-DUF2809
RKZ58327.1	*Candidatus Parabeggiatoa sp. nov. 2*	Skillet-DUF2809
MBO4565512.1	*Bacteroidaceae bacterium*	Skillet-DUF2809
MBP7173332.1	*Cloacibacterium sp.*	Skillet-DUF2809
HFC92738.1	*Leucothrix mucor*	Skillet-DUF2809
NLA09337.1	*Microbacteriaceae bacterium*	Skillet-DUF2809
MBN1130882.1	*Chitinispirillaceae bacterium*	Skillet-DUF2809
WP_047000502.1	*Clostridium sp. C8*	Skillet-DUF2809
MBP5489606.1	*Lachnospiraceae bacterium*	Skillet-DUF2809
MBO4500184.1	*Bacteroidaceae bacterium*	Skillet-DUF2809
MBK7467201.1	*Saprospiraceae bacterium*	Skillet-DUF2809
RZK10840.1	*Flavobacterium sp.*	Skillet-DUF2809
WP_136004303.1	*Clostridium sartagoforme*	Skillet-DUF2809
WP_113675058.1	*Vallitalea guaymasensis*	Skillet-DUF2809
WP_139064719.1	*Antarcticibacterium sp. W02-3*	Skillet-DUF2809
SCJ26782.1	*uncultured Clostridium sp.*	Skillet-DUF2809
WP_091209959.1	*Mucilaginibacter gossypiicola*	Skillet-DUF2809
MBQ6906543.1	*Clostridia bacterium*	Skillet-DUF2809
MBC5841176.1	*Flavobacterium kayseriense*	Skillet-DUF2809
MBQ0160258.1	*Candidatus Colimorpha merdihippi*	Skillet-DUF2809
WP_073362520.1	*Flavobacterium fontis*	Skillet-DUF2809
MBK8218584.1	*Myxococcales bacterium*	Skillet-DUF2809
WP_162457719.1	*Actinotalea caeni*	Skillet-DUF2809
MBN9662003.1	*Acidobacteriota bacterium*	Skillet-DUF2809
MBC8059853.1	*Clostridiaceae bacterium*	Skillet-DUF2809
MBD5150140.1	*Oscillibacter sp.*	Skillet-DUF2809
NHM01514.1	*Flavobacterium difficile*	Skillet-DUF2809
NEQ32940.1	*Leptolyngbya sp. SIO4C5*	Skillet-DUF2809
WP_199137065.1	*Pedobacter sp. ASV12*	Skillet-DUF2809
MBK1853717.1	*Oceaniferula flava*	Skillet-DUF2809
MBE6889077.1	*Oscillospiraceae bacterium*	Skillet-DUF2809
MCA9569915.1	*Myxococcales bacterium*	Skillet-DUF2809
MCA9625641.1	*Myxococcales bacterium*	Skillet-DUF2809
NOU30248.1	*Polyangiaceae bacterium*	Skillet-DUF2809
HAP34837.1	*Bacteroidota bacterium*	Skillet-DUF2809
MBR6429149.1	*Clostridia bacterium*	Skillet-DUF2809
WP_092019581.1	*Pseudarcicella hirudinis*	Skillet-DUF2809
MBB1542626.1	*Flavobacteriaceae bacterium*	Skillet-DUF2809
WP_189020774.1	*Paenibacillus marchantiophytorum*	Skillet-DUF2809
MCC6605047.1	*Anaerolineae bacterium*	Skillet-DUF2809
MBL7188616.1	*Phycisphaerae bacterium*	Skillet-DUF2809
WP_211359695.1	*Actinocorallia herbida*	Skillet-DUF2809
MCD4698306.1	*Bacteroidales bacterium*	Skillet-DUF2809
QDV25783.1	*Aureliella helgolandensis*	Skillet-DUF2809
MBR5524447.1	*Clostridia bacterium*	Skillet-DUF2809
MBS6445145.1	*Ruminococcus sp.*	Skillet-DUF2809
MBR5871317.1	*Clostridia bacterium*	Skillet-DUF2809
WP_127455997.1	*Paenibacillus chondroitinus*	Skillet-DUF2809
MCB0270739.1	*Calditrichota bacterium*	Skillet-DUF2809
MBR6042998.1	*Paludibacteraceae bacterium*	Skillet-DUF2809
WP_104418902.1	*Neolewinella xylanilytica*	Skillet-DUF2809
MBK9030296.1	*Myxococcales bacterium*	Skillet-DUF2809
WP_031238085.1	*Asticcacaulis sp. AC466*	Skillet-DUF2809
MBK7535814.1	*Myxococcales bacterium*	Skillet-DUF2809
MBL8955534.1	*Myxococcaceae bacterium*	Skillet-DUF2809
WP_185881359.1	*Cetobacterium sp. 2A*	Skillet-DUF2809
WP_119406649.1	*Mucilaginibacter celer*	Skillet-DUF2809
MBQ2694062.1	*Clostridia bacterium*	Skillet-DUF2809
NCC86923.1	*Clostridia bacterium*	Skillet-DUF2809
PZP46648.1	*Pseudopedobacter saltans*	Skillet-DUF2809
MBN2390668.1	*Anaerolineae bacterium*	Skillet-DUF2809
MBC8204690.1	*FCB group bacterium*	Skillet-DUF2809
RLJ08721.1	*Candidatus Aenigmatarchaeota archaeon*	Skillet-DUF2809
WP_203904867.1		Skillet-DUF2809
WP_203903394.1	*Virgisporangium aliadipatigenens*	Skillet-DUF2809
WP_019227459.1	*Sedimentibacter sp. B4*	Skillet-DUF2809
WP_077850044.1	*Clostridium puniceum*	Skillet-DUF2809
PWB67844.1	*Holophagae bacterium*	Skillet-DUF2809
MCC7245635.1	*Saprospiraceae bacterium*	Skillet-DUF2809
ACQ81970.1	*Beutenbergia cavernae DSM 12333*	Skillet-DUF2809
WP_152892480.1	*Clostridium tarantellae*	Skillet-DUF2809
WP_206733684.1	*Pseudoxanthomonas composti*	Skillet-DUF2809
KAA0259939.1	*Chlorobiota bacterium*	Skillet-DUF2809
WP_106637875.1	*Siphonobacter sp. SORGH_AS_0500*	Skillet-DUF2809
MBN1461531.1	*Armatimonadota bacterium*	Skillet-DUF2809
WP_084708184.1	*Leucothrix mucor DSM 2157*	Skillet-DUF2809
WP_066631197.1	*Labilibacter marinus*	Skillet-DUF2809
WP_029289426.1	*Cellulomonas sp. HZM*	Skillet-DUF2809
WP_109822879.1	*Leucothrix arctica*	Skillet-DUF2809
WP_077434136.1		Skillet-DUF2809

WP_196281608.1	*Hymenobacter jeongseonensis*	Skillet-DUF2809
WP_173777686.1	*Frigoriflavimonas asaccharolytica*	Skillet-DUF2809
WP_088842385.1	*Hymenobacter gelipurpurascens*	Skillet-DUF2809
GAE89989.1	*Acetivibrio straminisolvens JCM 21531*	Skillet-DUF2809
OPZ21066.1	*Firmicutes bacterium ADurb.BinA205*	Skillet-DUF2809
WP_068263594.1	*Janibacter limosus NBRC 16128*	Skillet-DUF2809
GHD39998.1	*Mycetocola manganoxydans*	Skillet-DUF2809
MBC7881140.1	*Gloeobacterales cyanobacterium ES-bin-313*	Skillet-DUF2809
WP_155061880.1	*Turicibacter sanguinis*	Skillet-DUF2809
MBR6892490.1	*Bacteroidaceae bacterium*	Skillet-DUF2809
WP_168567711.1	*Oxynema aestuarii AP17*	Skillet-DUF2809
NND80486.1	*Maribacter sp.*	Skillet-DUF2809
WP_190509509.1	*Oscillatoria sp. FACHB-1407*	Skillet-DUF2809
WP_066892212.1	*Clostridium nigeriense*	Skillet-DUF2809
EDS73391.1	*Anaerofustis stercorihominis DSM 17244*	Skillet-DUF2809
WP_212692928.1	*Vallitalea guaymasensis*	Skillet-DUF2809
WP_137695882.1	*Ruminiclostridium herbifermentans*	Skillet-DUF2809
WP_132000930.1	*Flavobacterium cellulosilyticum*	Skillet-DUF2809
WP_067787853.1	*Paraurantiacibacter namhicola*	Skillet-DUF2809
MBS1742852.1	*Bacteroidota bacterium*	Skillet-DUF2809
WP_195160275.1	*Clostridium sp. 'deep sea'*	Skillet-DUF2809
WP_160358646.1	*Clostridium chromiireducens*	Skillet-DUF2809
MBP1605898.1	*Acidobacteriota bacterium*	Skillet-DUF2809
WP_216389897.1	*Clostridium sp. MSJ-8*	Skillet-DUF2809
MBQ6085445.1	*Bacteroidaceae bacterium*	Skillet-DUF2809
MBK9409268.1	*Gemmatimonadota bacterium*	Skillet-DUF2809
MCB0704208.1	*Saprospiraceae bacterium*	Skillet-DUF2809
MBT4496158.1	*Gemmatimonadota bacterium*	Skillet-DUF2809
WP_023452697.1	*Asticcacaulis sp. AC460*	Skillet-DUF2809
WP_144256373.1	*Flavobacterium restrictum*	Skillet-DUF2809
WP_023451306.1	*Asticcacaulis sp. AC402*	Skillet-DUF2809
MBS1532961.1	*Bacteroidota bacterium*	Skillet-DUF2809
WP_209715034.1	*Asticcacaulis sp. BE141*	Skillet-DUF2809
WP_006273839.1	*Asticcacaulis biprosthecium C19*	Skillet-DUF2809
NNK74086.1	*Flavobacteriaceae bacterium*	Skillet-DUF2809
MBA3918785.1	*Gemmatimonas sp.*	Skillet-DUF2809
MBN8547198.1	*Ignavibacteria bacterium*	Skillet-DUF2809
WP_079736996.1	*Polaribacter sp. KT 15*	Skillet-DUF2809
WP_077809766.1	*Polaribacter sp. BM10*	Skillet-DUF2809
WP_018080355.1	*Asticcacaulis benevestitus DSM 16100 = ATCC BAA-896*	Skillet-DUF2809
WP_222823599.1	*Alteriqipengyuania abyssalis*	Skillet-DUF2809
MBQ3269386.1	*Clostridia bacterium*	Skillet-DUF2809
MBP5422365.1	*Paludibacteraceae bacterium*	Skillet-DUF2809
MBQ2939521.1	*Clostridia bacterium*	Skillet-DUF2809
MBK7267842.1	*Ignavibacteriales bacterium*	Skillet-DUF2809
WP_221836864.1	*Paracrocinitomix mangrovi*	Skillet-DUF2809
MBN1468993.1	*Fusobacteriaceae bacterium*	Skillet-DUF2809
MBI2146410.1	*Candidatus Woesearchaeota archaeon*	Skillet-DUF2809
RKZ70105.1	*Candidatus Parabeggiatoa sp. nov. 1*	Skillet-DUF2809
BCJ53554.1	*Actinoplanes sp. NBRC 14428*	Skillet-DUF2809
WP_229491795.1	*Enterovibrio norvegicus*	Skillet-DUF2809
NIM90192.1	*Candidatus Aminicenantes bacterium*	Skillet-DUF2809
NOQ72123.1	*Crocinitomix sp.*	Skillet-DUF2809
MBQ8402784.1	*Clostridia bacterium*	Skillet-DUF2809
PLX06253.1	*Marinilabiliales bacterium*	Skillet-DUF2809
MCB9549165.1	*Myxococcales bacterium*	Skillet-DUF2809
GDX80663.1	*Deltaproteobacteria bacterium*	Skillet-DUF2809
MBM3903378.1	*Verrucomicrobiota bacterium*	Skillet-DUF2809
MBL8616067.1	*Deltaproteobacteria bacterium*	Skillet-DUF2809
MBR1821702.1	*Clostridia bacterium*	Skillet-DUF2809
MBN1450401.1	*Anaerolineales bacterium*	Skillet-DUF2809
MBK8808785.1	*Bacteroidales bacterium*	Skillet-DUF2809
WP_090080229.1	*Halpernia frigidisoli*	Skillet-DUF2809
WP_167050370.1		Skillet-DUF2809
PWC03786.1	*Agromyces badenianii*	Skillet-DUF2809
WP_055741140.1	*Heyndrickxia shackletonii*	Skillet-DUF2809
MBI2894626.1	*Deltaproteobacteria bacterium*	Skillet-DUF2809
MBR5980070.1	*Oscillospiraceae bacterium*	Skillet-DUF2809
MBL0056745.1	*Chitinophagaceae bacterium*	Skillet-DUF2809
HAG03797.1	*Lachnospiraceae bacterium*	Skillet-DUF2809
WP_165327567.1	*Sphingosinithalassobacter tenebrarum*	Skillet-DUF2809
WP_199118246.1	*Pedobacter sp. ASV28*	Skillet-DUF2809
MCA0347764.1	*Bacteroidota bacterium*	Skillet-DUF2809
WP_121934756.1	*Faecalibacter macacae*	Skillet-DUF2809
MBQ2803861.1	*Lachnospiraceae bacterium*	Skillet-DUF2809
WP_024979788.1	*Flavobacterium succinicans*	Skillet-DUF2809
WP_215138323.1	*Hymenobacter sp. ISL-91*	Skillet-DUF2809
WP_130736226.1	*Flavobacterium sp. J27*	Skillet-DUF2809
MBO4804708.1	*Paludibacteraceae bacterium*	Skillet-DUF2809
MCC6539551.1	*Bryobacterales bacterium*	Skillet-DUF2809
WP_036944838.1	*Pseudobacteroides cellulosolvens ATCC 35603 = DSM 2933*	Skillet-DUF2809
MBR4840676.1	*Paludibacteraceae bacterium*	Skillet-DUF2809
NLD48105.1	*Clostridiaceae bacterium*	Skillet-DUF2809
WP_200267850.1	*Clostridium yunnanense*	Skillet-DUF2809
WP_026705605.1	*Flavobacterium soli DSM 19725*	Skillet-DUF2809
OIQ25311.1	*Crocinitomix sp. MedPE-SWsnd*	Skillet-DUF2809
MBC7904715.1	*Chitinophagaceae bacterium*	Skillet-DUF2809
WP_111972953.1	*Capnocytophaga ochracea*	Skillet-DUF2809
MBI5954732.1	*Chloroflexota bacterium*	Skillet-DUF2809
MBX9807849.1	*Flavobacteriaceae bacterium*	Skillet-DUF2809
CAA6813681.1	*uncultured Aureispira sp.*	Skillet-DUF2809
MBI3139108.1	*Sphingobacteriales bacterium*	Skillet-DUF2809
WP_099077467.1		Skillet-DUF2809

MBO4398395.1	*Bacteroidaceae bacterium*	Skillet-DUF2800
WP_095135105.1	*Anaeromicrobium sediminis*	Skillet-DUF2800
HHC79377.1	*Flavobacteriia bacterium*	Skillet-DUF2800
WP_217650508.1	*Spirulina major PCC 6313*	Skillet-DUF2800
MBN9165310.1	*Myxococcales bacterium*	Skillet-DUF2800
WP_212695042.1	*Vallitalea pronyensis*	Skillet-DUF2800
WP_108778602.1	*Flavobacterium album*	Skillet-DUF2800
MBN1154161.1	*Candidatus Zhuqueibacterota bacterium*	Skillet-DUF2800
WP_208361770.1	*Clostridium perfringens*	Skillet-DUF2800
WP_212923387.1	*Capnocytophaga cynodegmi*	Skillet-DUF2800
GEO10868.1	*Segetibacter aerophilus*	Skillet-DUF2800
WP_014388784.1	*Flavobacterium indicum GPTSA100-9 = DSM 17447*	Skillet-DUF2800
MBX7085051.1	*Leptospirales bacterium*	Skillet-DUF2800
MBN1876045.1	*Anaerolineae bacterium*	Skillet-DUF2800
MBQ9665730.1	*Bacteroidaceae bacterium*	Skillet-DUF2800
WP_121196897.1	*Mucilaginibacter gracilis*	Skillet-DUF2800
MBQ3692507.1	*Clostridia bacterium*	Skillet-DUF2800
MBA4760714.1	*Sphingomonas sp.*	Skillet-DUF2800
WP_103091201.1	*Clostridium thermosuccinogenes*	Skillet-DUF2800
WP_034957767.1	*Erythrobacter longus*	Skillet-DUF2800
MBI5943208.1	*Chloroflexota bacterium*	Skillet-DUF2800
MCD8080419.1	*Bacteroides sp.*	Skillet-DUF2800
MBC7524716.1	*Flavobacterium sp.*	Skillet-DUF2800
MBO4332335.1	*Paludibacteraceae bacterium*	Skillet-DUF2800
MBK7073276.1	*Myxococcales bacterium*	Skillet-DUF2800
MBS1913387.1	*Bacteroidota bacterium*	Skillet-DUF2800
MCD7926485.1	*Bacteroides sp.*	Skillet-DUF2800
MBD5496116.1	*Lachnospiraceae bacterium*	Skillet-DUF2800
RZK59978.1	*Pedobacter sp.*	Skillet-DUF2800
WP_113648441.1	*Pedobacter chitinilyticus*	Skillet-DUF2800
WP_229760370.1	*Ferruginibacter lapsinanis*	Skillet-DUF2800
WP_018368253.1	*Streptococcus entericus DSM 14446*	Skillet-DUF2800
WP_165356317.1	*Sphingosinicella sp. BN140058*	Skillet-DUF2800
WP_129019978.1	*Edaphocola flava*	Skillet-DUF2800
WP_184309623.1	*Anaerosolibacter carboniphilus*	Skillet-DUF2800
NJK39537.1	*Oscillatoriales cyanobacterium SM2_3_0*	Skillet-DUF2800
WP_224034075.1	*Clostridium gelidum*	Skillet-DUF2800
WP_062616202.1		Skillet-DUF2800
MBK8315987.1	*Acidobacteriota bacterium*	Skillet-DUF2800
WP_222165451.1	*Edaphocola aurantiacus*	Skillet-DUF2800
WP_156864428.1	*Casimicrobium huifangae*	Skillet-DUF2800
WP_018371515.1	*Streptococcus massiliensis*	Skillet-DUF2800
WP_116393318.1	*Parvularcula marina*	Skillet-DUF2800
WP_139958158.1	*Flavicella sediminum*	Skillet-DUF2800
HAN76484.1	*Bacteroidales bacterium*	Skillet-DUF2800
MBR1599080.1	*Lachnospiraceae bacterium*	Skillet-DUF2800
WP_202747137.1		Skillet-DUF2800
WP_073620362.1		Skillet-DUF2800
MBL4634424.1	*Kofleriaceae bacterium*	Skillet-DUF2800
MCC8028003.1	*Clostridium sp.*	Skillet-DUF2800
WP_215992928.1	*Polaribacter sp. 1_MG-2023*	Skillet-DUF2800
WP_027639356.1	*Clostridium cadaveris*	Skillet-DUF2800
MBS3053098.1	*Candidatus Aenigmatarchaeota archaeon*	Skillet-DUF2800
WP_213213448.1	*Flavobacterium sp. CYK-55*	Skillet-DUF2800
ERI91929.1	*Clostridiales bacterium oral taxon 876 str. F0540*	Skillet-DUF2800
WP_147714490.1	*Reinekea thalattae*	Skillet-DUF2800
MBJ6610240.1	*Candidatus Thiothrix moscowensis*	Skillet-DUF2800
WP_077968294.1	*Streptomyces tsukubensis*	Skillet-DUF2800
MBK7498188.1	*Ignavibacteriales bacterium*	Skillet-DUF2800
MCA8941811.1	*Planctomycetota bacterium*	Skillet-DUF2800
MBS3772060.1	*Bacteroidales bacterium*	Skillet-DUF2800
WP_173200532.1	*Parvularcula mediterranea*	Skillet-DUF2800
WP_175634107.1	*Pedobacter ghigonis*	Skillet-DUF2800
WP_189575738.1	*Parvularcula lutaonensis*	Skillet-DUF2800
WP_125013474.1	*Flavobacterium macacae*	Skillet-DUF2800
WP_221859162.1	*Clostridium sardiniense*	Skillet-DUF2800
WP_204596136.1	*Clostridium sardiniense*	Skillet-DUF2800
WP_008419228.1	*Clostridium sp. LS*	Skillet-DUF2800
MBI2129179.1	*Candidatus Woesearchaeota archaeon*	Skillet-DUF2800
MBO5746888.1	*Clostridia bacterium*	Skillet-DUF2800
NJN60145.1	*Coleofasciculaceae cyanobacterium RL_1_1*	Skillet-DUF2800
PIZ96292.1	*Candidatus Magasanikbacteria bacterium CG_4_10_14_0_2_um_filter_33_14*	Skillet-DUF2800
MBL8678138.1	*Myxococcales bacterium*	Skillet-DUF2800
OYN93796.1	*Enemella evansiae*	Skillet-DUF2800
MBU0692150.1	*bacterium*	Skillet-DUF2800
WP_189493134.1	*Formosimonas limnophila*	Skillet-DUF2800
WP_135531028.1		Skillet-DUF2800
WP_131555918.1	*Pedobacter frigidisoli*	Skillet-DUF2800
WP_048341160.1	*Clostridium haemolyticum NCTC 8350*	Skillet-DUF2800
HCT59207.1	*Gemmatimonas aurantiaca*	Skillet-DUF2800
MBR5091741.1	*Ruminiclostridium sp.*	Skillet-DUF2800
MBK8837616.1	*Hyphomonadaceae bacterium*	Skillet-DUF2800
MBW8733186.1	*Asticcacaulis sp.*	Skillet-DUF2800
MCC7454360.1	*Crocinitomix sp.*	Skillet-DUF2800
NUR90544.1	*Nonomuraea sp.*	Skillet-DUF2800
MBT8260122.1	*Bacteroidia bacterium*	Skillet-DUF2800
MBI5021014.1	*Ignavibacteriales bacterium*	Skillet-DUF2800
MBW4660549.1	*Drouetiella hepatica Uher 2000/2452*	Skillet-DUF2800
MCB8937887.1	*Ardenticatenaceae bacterium*	Skillet-DUF2800
NIA29087.1	*Actinomycetota bacterium*	Skillet-DUF2800
REE94523.1	*Paenibacillus taihuensis*	Skillet-DUF2800
WP_221239583.1	*Sphingomonas xinjiangensis*	Skillet-DUF2800
MBO9532902.1	*Solirubrobacteraceae bacterium*	Skillet-DUF2800

WP_185949776.1	*Microbispora sp. KK1-11*	Skillet-DUF2809
WP_175347044.1	*Cellulomonas humilata*	Skillet-DUF2809
KQY48053.1	*Cellulomonas sp. Root137*	Skillet-DUF2809
MBL0212294.1	*Myxococcales bacterium*	Skillet-DUF2809
NJD20447.1	*Gemmatimonadota bacterium*	Skillet-DUF2809
WP_183651606.1	*Nonomuraea dietziae*	Skillet-DUF2809
WP_206687987.1		Skillet-DUF2809
MBK9317352.1	*Acidobacteriota bacterium*	Skillet-DUF2809
MBD5112299.1	*Oscillospiraceae bacterium*	Skillet-DUF2809
TDQ15081.1	*Algoriphagus boseongensis*	Skillet-DUF2809
RKJ86255.1	*Aeromonas veronii*	Skillet-DUF2809
WP_051670669.1	*Bryobacter aggregatus MPL3*	Skillet-DUF2809
WP_119928501.1	*Bailinhaonella thermotolerans*	Skillet-DUF2809
SHE52369.1	*Pedobacter caeni*	Skillet-DUF2809
WP_051275129.1	*Cellulomonas sp. URHD0024*	Skillet-DUF2809
MBN1988769.1	*Bacteroidales bacterium*	Skillet-DUF2809
MBD5128412.1	*Oscillospiraceae bacterium*	Skillet-DUF2809
WP_207544642.1	*Sphingomonas jatrophae*	Skillet-DUF2809
WP_145796804.1	*Kitasatospora atroaurantiaca*	Skillet-DUF2809
MBS2035985.1	*bacterium*	Skillet-DUF2809
WP_199851616.1	*Brumimicrobium mesophilum*	Skillet-DUF2809
NUQ41591.1	*Calditrichaceae bacterium*	Skillet-DUF2809
TQL02583.1	*Cellulomonas sp. SLBN-39*	Skillet-DUF2809
SEN36439.1	*Paenibacillus sp. OV219*	Skillet-DUF2809
WP_132597178.1	*Nonomuraea deserti*	Skillet-DUF2809
WP_229685892.1		Skillet-DUF2809
WP_225224390.1		Skillet-DUF2809
MBL8211753.1	*Bryobacterales bacterium*	Skillet-DUF2809
WP_138755363.1		Skillet-DUF2809
WP_170297168.1	*Georgenia thermotolerans*	Skillet-DUF2809
WP_171636535.1		Skillet-DUF2809
MBP6307374.1	*Opitutaceae bacterium*	Skillet-DUF2809
MBP9780156.1	*Candidatus Altimarinota bacterium*	Skillet-DUF2809
MBC8531908.1	*Gehongia tenuis*	Skillet-DUF2809
MBQ3688533.1	*Bacteroidales bacterium*	Skillet-DUF2809
WP_214449538.1	*Flavihumibacter rivuli*	Skillet-DUF2809
MBQ9768219.1	*Lachnospiraceae bacterium*	Skillet-DUF2809
HBY59282.1	*Bryobacterales bacterium*	Skillet-DUF2809
WP_119423156.1	*Galactobacter valiniphilus*	Skillet-DUF2809
WP_203793489.1	*Cellulomonas oligotrophica*	Skillet-DUF2809
BDC48966.1	*Bryobacterales bacterium F-183*	Skillet-DUF2809
WP_083853799.1		Skillet-DUF2809
WP_206965628.1		Skillet-DUF2809
KAB2922171.1	*Bacteroidota bacterium*	Skillet-DUF2809
MBP1688072.1	*Deltaproteobacteria bacterium*	Skillet-DUF2809
WP_221416242.1	*Sphingomonas kyeonggiensis*	Skillet-DUF2809
WP_110128555.1		Skillet-DUF2809
MBT8257625.1	*Bacteroidia bacterium*	Skillet-DUF2809
QNF95688.1	*Janibacter sp. YB324*	Skillet-DUF2809
MBK8228581.1	*Flavobacteriales bacterium*	Skillet-DUF2809
MBI3681637.1	*Acidobacteriota bacterium*	Skillet-DUF2809
NOU18337.1	*Bacteroidales bacterium*	Skillet-DUF2809
WP_232800222.1	*Motilimonas sp. E26*	Skillet-DUF2809
WP_227422169.1	*Pengzhenrongella sicca*	Skillet-DUF2809
MBM3247235.1	*Candidatus Pacearchaeota archaeon*	Skillet-DUF2809
SEB58921.1	*Paramicrobacterium humi*	Skillet-DUF2809
MBC7498079.1	*Candidatus Altimarinota bacterium*	Skillet-DUF2809
MBN1246423.1	*Anaerolineae bacterium*	Skillet-DUF2809
MBT8256609.1	*Bacteroidia bacterium*	Skillet-DUF2809
WP_051191456.1	*Flavobacterium gelidilacus*	Skillet-DUF2809
WP_072956079.1		Skillet-DUF2809
MBP8856567.1	*Anaerolineaceae bacterium*	Skillet-DUF2809
WP_197029939.1	*Cellulomonas sp. URHE0023*	Skillet-DUF2809
MBT3194616.1	*Verrucomicrobiota bacterium*	Skillet-DUF2809
WP_205960040.1	*Flammeovirga aprica JL-4*	Skillet-DUF2809
MBU4465055.1	*Actinomycetota bacterium*	Skillet-DUF2809
HBT80720.1	*Candidatus Yanofskybacteria bacterium*	Skillet-DUF2809
MBN1500111.1	*Spirochaetota bacterium*	Skillet-DUF2809
WP_175269247.1	*Mucilaginibacter humi*	Skillet-DUF2809
WP_224772478.1		Skillet-DUF2809
ACN29727.1		Skillet-DUF2809
MBC7841653.1	*Gemmatimonadaceae bacterium*	Skillet-DUF2809
MBI3883468.1	*Sphingobacteriales bacterium*	Skillet-DUF2809
WP_227909603.1		Skillet-DUF2809
MBY0506876.1	*Bryobacteraceae bacterium*	Skillet-DUF2809
GEL98942.1	*Cellulomonas terrae*	Skillet-DUF2809
NET54347.1	*Merismopedia sp. SIO2A8*	Skillet-DUF2809
WP_207425217.1	*Pedobacter sp. SYSU D00535*	Skillet-DUF2809
WP_069095596.1	*Methyloligella halotolerans*	Skillet-DUF2809
MBQ8196302.1	*Oscillospiraceae bacterium*	Skillet-DUF2809
NJO83648.1	*Blastochloris sp.*	Skillet-DUF2809
WP_050990208.1	*Sphingomonas sp. HT-1*	Skillet-DUF2809
MBA3923948.1	*Nostocaceae cyanobacterium*	Skillet-DUF2809
MBC7651519.1	*Chitinophagaceae bacterium*	Skillet-DUF2809
HDU67608.1		Skillet-DUF2809
WP_231843916.1		Skillet-DUF2809
MCA9130161.1	*Planctomycetales bacterium*	Skillet-DUF2809
MBI5957075.1	*Chloroflexota bacterium*	Skillet-DUF2809
MBC7383586.1	*Bacteroidia bacterium*	Skillet-DUF2809
NJL65801.1	*Microcoleus sp. SM1_3_4*	Skillet-DUF2809
RLS72880.1	*Planctomycetota bacterium*	Skillet-DUF2809
MCB9850390.1	*Phycisphaerales bacterium*	Skillet-DUF2809
OYU98017.1	*Verrucomicrobiales bacterium VVV1*	Skillet-DUF2809

GGG35190.1	*Dokdonia pacifica*	Skillet-DUF2809
WP_233509567.1		Skillet-DUF2809
WP_083934972.1	*Segetibacter koreensis DSM 18137*	Skillet-DUF2809
NLK97663.1	*Candidatus Epulonipiscium sp.*	Skillet-DUF2809
HIB66327.1	*Phycisphaerales bacterium*	Skillet-DUF2809, Skillet-DUF2809
MBU1162313.1	*Pseudomonadota bacterium*	Skillet-DUF2809
SHN30816.1	*Mucilaginibacter sp. OK098*	Skillet-DUF2809
SNT59638.1	*Asanoa hainanensis*	Skillet-DUF2809
MBC7827608.1	*Chitinophagaceae bacterium*	Skillet-DUF2809
WP_080567358.1	*Streptococcus sobrinus*	Skillet-DUF2809
WP_228711995.1	*Halarcobacter ebronensis*	Skillet-DUF2809
MBP6689746.1	*Hyphomonadaceae bacterium*	Skillet-DUF2809
RZM03789.1	*Pedobacter sp.*	Skillet-DUF2809
HAB59231.1	*Lachnospiraceae bacterium*	Skillet-DUF2809
MBQ1183733.1	*Clostridia bacterium*	Skillet-DUF2809
WP_220640179.1	*Flavobacterium litorale*	Skillet-DUF2809
GFE69657.1	*Chroococcus sp. FPU101*	Skillet-DUF2809
MBQ4097527.1	*Clostridia bacterium*	Skillet-DUF2809
MBQ6841939.1	*Bacillota bacterium*	Skillet-DUF2809
NND52428.1	*Flavobacteriaceae bacterium*	Skillet-DUF2809
EMQ94880.1	*Xanthomarina gelatinilytica*	Skillet-DUF2809
MBN1172493.1	*Micromonosporaceae bacterium*	Skillet-DUF2809(frag)
MBS6287880.1	*Campylobacter concisus*	Skillet-DUF2809(frag)
AFZ14467.1	*Crinalium epipsammum PCC 9333*	Skillet-DUF2809(frag)
NLE46150.1	*Chloroflexota bacterium*	Skillet-DUF2809(frag)
MBS3777406.1	*Bacteroidales bacterium*	Skillet-DUF2809(frag)
SHK28822.1	*Tepidibacter formicigenes DSM 15518*	Skillet-DUF2809(frag)
MBK7271420.1	*Flavobacteriales bacterium*	Skillet-DUF2809(frag)
WP_130181915.1	*Blautia producta*	Skillet-DUF2809(frag)
RLD51080.1	*Bacteroidota bacterium*	Skillet-DUF2809(frag)
WP_006843272.1	*Dysgonomonas mossii*	Skillet-DUF2809(frag)
WP_227163787.1	*Thomasclavelia ramosa*	Skillet-DUF2809(frag)
MBW8065672.1	*Nitrospira sp.*	Skillet-DUF2809(frag)
RYD65368.1	*Verrucomicrobiaceae bacterium*	Skillet-DUF2809(frag)
MAN30766.1	*Pedosphaera sp.*	Skillet-DUF2809(frag)
AET69071.1	*Desulfosporosinus orientis DSM 765*	Skillet-DUF2809(frag)
MBL7813510.1	*Saprospiraceae bacterium*	Skillet-DUF2809(frag)
MBM3759939.1	*Acidobacteriota bacterium*	Skillet-DUF2809(frag)
MBL1153363.1	*Ignavibacteriota bacterium*	Skillet-DUF2809(frag)
MBA3557375.1	*Gemmatimonadaceae bacterium*	Skillet-DUF2809(frag)
WP_139068386.1	*Flavobacterium glycines*	Skillet-DUF2809(frag)
NIN12539.1	*Gemmatimonadales bacterium*	Skillet-DUF2809(frag)
NOY78779.1	*Calditrichota bacterium*	Skillet-DUF2809(frag)
NQT26415.1	*Candidatus Zhuqueibacterota bacterium*	Skillet-DUF2809
MBN2001007.1	*Candidatus Zhuqueibacterota bacterium*	Skillet-DUF2809
MCA9734823.1	*Candidatus Zhuqueibacterota bacterium*	Skillet-DUF2809
MCA9734928.1	*Candidatus Zhuqueibacterota bacterium*	Skillet-DUF2809
MBQ00758.1	*Acidobacteriota bacterium*	Skillet-DUF2809
MBI2212674.1	*Acidobacteriota bacterium*	Skillet-DUF2809
MBI4474344.1	*Acidobacteriota bacterium*	Skillet-DUF2809
OFW05378.1	*Acidobacteria bacterium RIFCSPLOWO2_02_FULL_67_36*	Skillet-DUF2809
MBL0158316.1	*Bryobacterales bacterium*	Skillet-DUF2809
MBL8232911.1	*Bryobacterales bacterium*	Skillet-DUF2809
BCS32451.2	*Luteitalea sp. TBR-22*	Skillet-DUF2809
MBZ0110840.1	*Thermoanaerobaculia bacterium*	Skillet-DUF2809
KAA3615791.1	*Calditrichota bacterium*	Skillet-DUF2809
MBC8403128.1	*Fidelibacterota bacterium*	Skillet-DUF2809
MBD3299126.1	*Candidatus Zixiibacteriota bacterium*	Skillet-DUF2809
MBE9490982.1	*Bacteroidota bacterium*	Skillet-DUF2809
MBS1509098.1	*Bacteroidota bacterium*	Skillet-DUF2809
MBT3209753.1	*Bacteroidota bacterium*	Skillet-DUF2809
MBL7850853.1	*Cyclobacteriaceae bacterium*	Skillet-DUF2809
HBK89140.1	*Cytophagales bacterium*	Skillet-DUF2809
TAJ12575.1	*Marinilabiliaceae bacterium JC017*	Skillet-DUF2809
PLX09859.1	*Marinilabiliales bacterium*	Skillet-DUF2809
PLW99707.1	*Marinilabiliales bacterium*	Skillet-DUF2809
MCE1200173.1	*Marinilabiliales bacterium*	Skillet-DUF2809
MBK6779168.1	*Gemmatimonadota bacterium*	Skillet-DUF2809
MBK6454479.1	*Gemmatimonadota bacterium*	Skillet-DUF2809
MBT8403668.1	*Gemmatimonadota bacterium*	Skillet-DUF2809
MBT8336048.1	*Gemmatimonadota bacterium*	Skillet-DUF2809
MBL0171020.1	*Gemmatimonadaceae bacterium*	Skillet-DUF2809
MCC7003701.1	*Gemmatimonadaceae bacterium*	Skillet-DUF2809
NNE33768.1	*Rhodothermales bacterium*	Skillet-DUF2809
NQT01068.1	*Planctomycetota bacterium*	Skillet-DUF2809
MBE9548329.1	*Pseudomonadota bacterium*	Skillet-DUF2809
HIE87310.1	*Gammaproteobacteria bacterium*	Skillet-DUF2809
MBN1781496.1	*bacterium*	Skillet-DUF2809
MCD6501307.1	*bacterium*	Skillet-DUF2809
MBN1999515.1	*Candidatus Zhuqueibacterota bacterium*	Skillet-DUF2809
MBN2292632.1	*Pirellulales bacterium*	Skillet-DUF2809
MBL0124261.1	*Betaproteobacteria bacterium*	Skillet-DUF2809
MBK7047242.1	*bacterium*	Skillet-DUF2809
MBQ5564412.1	*Clostridia bacterium*	Skillet-DUF2809
MBQ9461374.1	*Clostridia bacterium*	Skillet-DUF2809
HCA56043.1	*Oscillospiraceae bacterium*	Skillet-DUF2809
NQX01663.1	*bacterium*	Skillet-DUF2809
WP_092944521.1	*Roseateles sp. YR242*	Skillet-DUF2809, ??
EAZ59541.1	*Pseudomonas aeruginosa 2192*	Skillet-DUF2809, ??
MBB5864688.1	*Xanthomonas sp. 3058*	??, Skillet-DUF2809
MBB4131174.1	*Xanthomonas sp. 3075*	??, Skillet-DUF2809
WP_224997917.1	*Cesiribacter sp. SM1*	Skillet-DUF2809
AHM60065.1	*Flammeovirgaceae bacterium 311*	Skillet-DUF2809

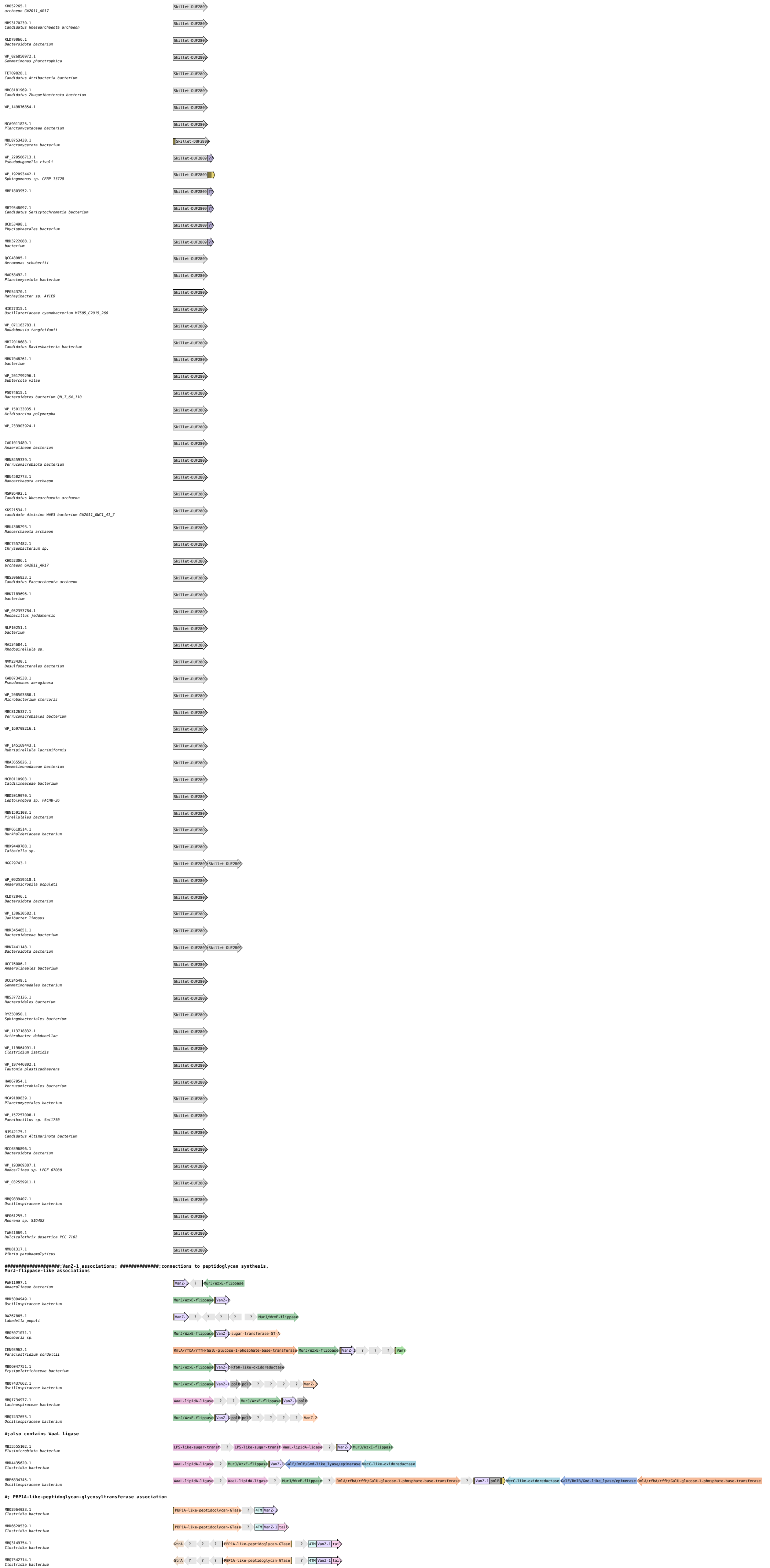
KHO52265.1
archaeon GW2011_AR17
MBS3170230.1
Candidatus Woesearchaeota archaeon
RLD79066.1
Bacteroidota bacterium
WP_026850972.1
Gemmatimonas phototrophica
TET09828.1
Candidatus Atribacteria bacterium
MBC8181969.1
Candidatus Zhuqueibacterota bacterium
WP_149876854.1
MCA9011825.1
Planctomycetaceae bacterium
MBL8753430.1
Planctomycetota bacterium
WP_229506713.1
Pseudoduganella rivuli
WP_192093442.1
Sphingomonas sp. CFBP 13720
MBP1803952.1
MBT9548097.1
Candidatus Sericytochromatia bacterium
UCD53498.1
Phycisphaerales bacterium
MBD3222088.1
bacterium
QCG48985.1
Aeromonas schubertii
MAG58492.1
Planctomycetota bacterium
PPG54370.1
Rathayibacter sp. AY1E9
HIK27315.1
Oscillatoriaceae cyanobacterium M7585_C2015_266
WP_071163783.1
Boudabousia tangfeifanii
MBI2018683.1
Candidatus Daviesbacteria bacterium
MBK7048261.1
bacterium
WP_201799296.1
Subtercola vilae
PSQ74615.1
Bacteroidetes bacterium QH_7_64_110
WP_150133035.1
Acidisarcina polymorpha
WP_233903924.1
CAG1013489.1
Anaerolineae bacterium
MBN8459339.1
Verrucomicrobiota bacterium
MBU4502773.1
Nanoarchaeota archaeon
MSR86492.1
Candidatus Woesearchaeota archaeon
KKS21534.1
candidate division WWE3 bacterium GW2011_GWC1_41_7
MBU4308293.1
Nanoarchaeota archaeon
MBC7557482.1
Chryseobacterium sp.
KHO52306.1
archaeon GW2011_AR17
MBS3066933.1
Candidatus Pacearchaeota archaeon
MBK7189696.1
bacterium
WP_052353784.1
Neobacillus jeddahensis
NLP10251.1
bacterium
MAI34684.1
Rhodopirellula sp.
NVM23430.1
Desulfobacterales bacterium
KAB0734538.1
Pseudomonas aeruginosa
WP_208503880.1
Microbacterium stercoris
MBC8126337.1
Verrucomicrobiales bacterium
WP_169708216.1
WP_145169443.1
Rubripirellula lacrimiformis
MBA3655826.1
Gemmatimonadaceae bacterium
MCB0110903.1
Caldilineaceae bacterium
MBD2019070.1
Leptolyngbya sp. FACHB-36
MBN1591108.1
Pirellulales bacterium
MBP6618514.1
Burkholderiaceae bacterium
MBX9449788.1
Taibaiella sp.
HGG29743.1
WP_092559518.1
Anaeromicropila populeti
RLD72046.1
Bacteroidota bacterium
WP_130630582.1
Janibacter limosus
MBR3454851.1
Bacteroidaceae bacterium
MBK7441148.1
Bacteroidota bacterium
UCC76006.1
Anaerolineales bacterium
UCC24549.1
Gemmatimonadales bacterium
MBS3772126.1
Bacteroidales bacterium
RYZ50050.1
Sphingobacteriales bacterium
WP_113718832.1
Arthrobacter dokdonellae
WP_119864991.1
Clostridium isatidis
WP_197446802.1
Tautonia plasticadhaerens
HAO67954.1
Verrucomicrobiales bacterium
MCA9189839.1
Planctomycetales bacterium
WP_157257008.1
Paenibacillus sp. Soil750
NJS42175.1
Candidatus Altimarinota bacterium
MCC6396896.1
Bacteroidota bacterium
WP_193969387.1
Nodosilinea sp. LEGE 07088
WP_032559911.1
MBQ9839407.1
Oscillospiraceae bacterium
NEO61255.1
Moorena sp. SIO4G2
TWH41069.1
Dulcicalothrix desertica PCC 7102
NMU81317.1
Vibrio parahaemolyticus
####################;VanZ-1 associations; ##############;connections to peptidoglycan synthesis,
MurJ-flippase-like associations
PWH11997.1
Anaerolineae bacterium
MBR5094949.1
Oscillospiraceae bacterium
RWZ67865.1
Labedella populi
MBO5071071.1
Roseburia sp.
CEN93962.1
Paraclostridium sordellii
MBO6047751.1
Erysipelotrichaceae bacterium
MBQ7437662.1
Oscillospiraceae bacterium
MBQ1734977.1
Lachnospiraceae bacterium
MBQ7437655.1
Oscillospiraceae bacterium
#;also contains WaaL ligase
MBI5555102.1
Elusimicrobiota bacterium
MBR4435620.1
Clostridia bacterium
MBE6834745.1
Oscillospiraceae bacterium
#; PBP1A-like-peptidoglycan-glycosyltransferase association
MBQ2964033.1
Clostridia bacterium
MBR6620539.1
Clostridia bacterium
MBQ3149754.1
Clostridia bacterium
MBQ7542714.1
Clostridia bacterium
Skillet-DUF2809
MurJ/WzxE-flippase
VanZ-1
WaaL-lipidA-ligase
LPS-like-sugar-transf
PBP1A-like-peptidoglycan-GTase
GtrA
4TM
polB
RfbH-like-oxidoreductase
WecC-like-oxidoreductase
GalE/RmlB/Gmd-like_lyase/epimerase
RmlA/rfbA/rffH/GalU-glucose-1-phosphate-base-transferase
sugar-transferase-GT-A
tail

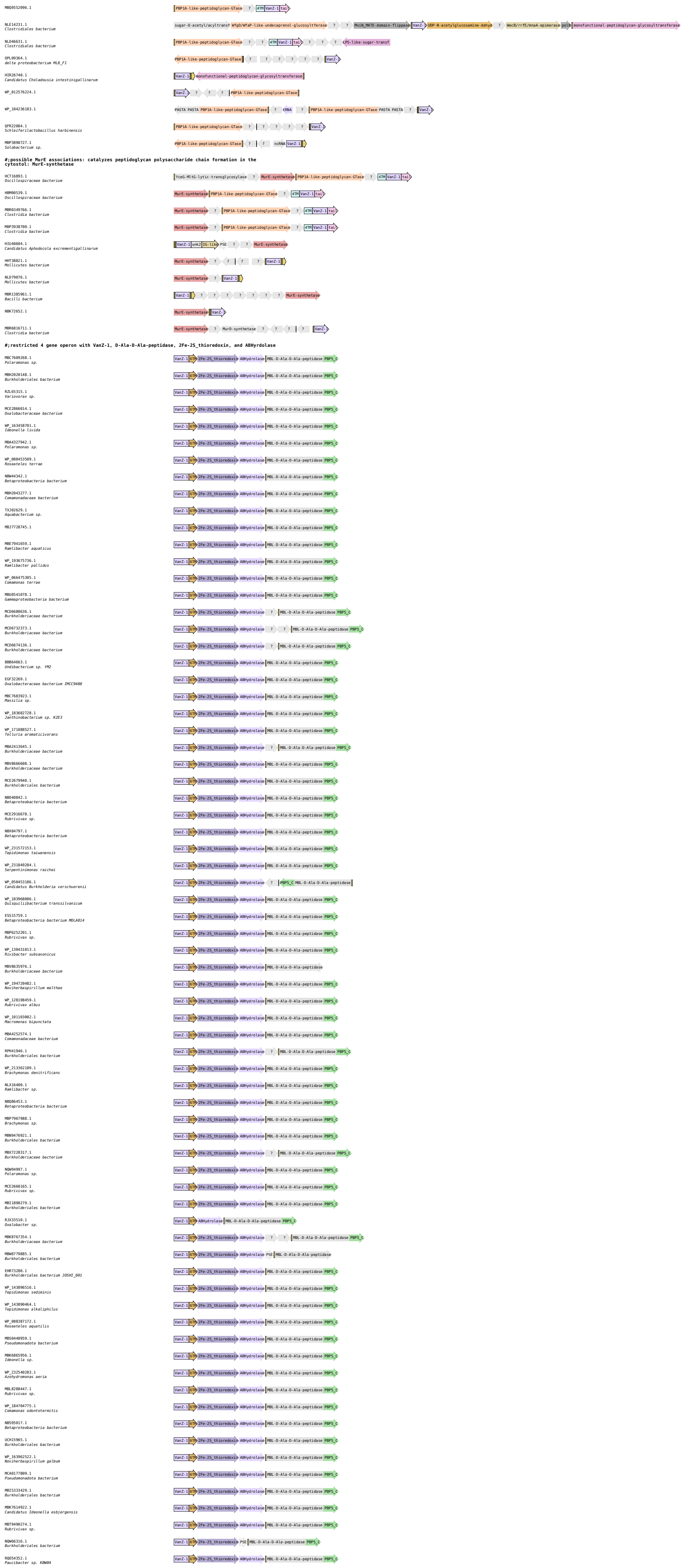

MBQ9552096.1
PBP1A-like-peptidoglycan-GTase ? 4TM VanZ-1 tail
NLE14231.1
Clostridiales bacterium
sugar-O-acetyl/acyltransf WfgD/WfaP-like-undecaprenol-glucosyltferase ? ? MviN_MATE-domain-flippase VanZ-1 UDP-N-acetylglucosamine-dehyd ? WecB/rrfE/mnaA-epimerase polb monofunctional-peptidoglycan-glycosyltransferase
NLO46631.1
Clostridiales bacterium
PBP1A-like-peptidoglycan-GTase ? ? 4TM VanZ-1 tail ? ? ? LPS-like-sugar-transf
OPL09364.1
delta proteobacterium ML8_F1
PBP1A-like-peptidoglycan-GTase ? ? ? ? ? ? ? VanZ-1
HIR26740.1
Candidatus Choladousia intestinigallinarum
VanZ-1 monofunctional-peptidoglycan-glycosyltransferase
WP_012576224.1
VanZ-1 ? ? ? PBP1A-like-peptidoglycan-GTase
WP_104236183.1
PASTA PASTA PBP1A-like-peptidoglycan-GTase ? tRNA ? PBP1A-like-peptidoglycan-GTase PASTA PASTA ? VanZ-1
QFR22084.1
Schleiferilactobacillus harbinensis
PBP1A-like-peptidoglycan-GTase ? ? ? ? ? VanZ-1
MBP3890727.1
Solobacterium sp.
PBP1A-like-peptidoglycan-GTase ? ? ncRNA VanZ-1
#;possible MurE associations: catalyzes peptidoglycan polysaccharide chain formation in the cytostol: MurE-synthetase
HCT16893.1
Oscillospiraceae bacterium
YceG-MltG-lytic-transglycosylase ? MurE-synthetase PBP1A-like-peptidoglycan-GTase ? 4TM VanZ-1 tail
HBM00539.1
Oscillospiraceae bacterium
MurE-synthetase PBP1A-like-peptidoglycan-GTase ? 4TM VanZ-1 tail
MBR0349766.1
Clostridia bacterium
MurE-synthetase ? PBP1A-like-peptidoglycan-GTase ? 4TM VanZ-1 tail
MBP3938700.1
Clostridia bacterium
MurE-synthetase ? PBP1A-like-peptidoglycan-GTase ? 4TM VanZ-1 tail
HIU40604.1
Candidatus Aphodocola excrementigallinarum
VanZ-1 unk2 IG-like PSE ? ? MurE-synthetase
HHT38821.1
Mollicutes bacterium
MurE-synthetase ? ? ? ? VanZ-1
NLD79076.1
Mollicutes bacterium
MurE-synthetase ? VanZ-1
MBR1385961.1
Bacilli bacterium
VanZ-1 ? ? ? ? ? ? ? MurE-synthetase
NBK72652.1
MurE-synthetase VanZ-1
MBR6816711.1
Clostridia bacterium
MurE-synthetase ? MurD-synthetase ? ? ? ? VanZ-1
#;restricted 4 gene operon with VanZ-1, D-Ala-D-Ala-peptidase, 2Fe-2S_thioredoxin, and ABHyrdolase
MBC7609268.1
Polaromonas sp.
VanZ-1 6TM 2Fe-2S_thioredoxin ABHydrolase MBL-D-Ala-D-Ala-peptidase PBP5_C
MBH2020148.1
Burkholderiales bacterium
VanZ-1 6TM 2Fe-2S_thioredoxin ABHydrolase MBL-D-Ala-D-Ala-peptidase PBP5_C
RZL65315.1
Variovorax sp.
VanZ-1 6TM 2Fe-2S_thioredoxin ABHydrolase MBL-D-Ala-D-Ala-peptidase PBP5_C
MCE2866014.1
Oxalobacteraceae bacterium
VanZ-1 6TM 2Fe-2S_thioredoxin ABHydrolase MBL-D-Ala-D-Ala-peptidase PBP5_C
WP_163458701.1
Ideonella livida
VanZ-1 6TM 2Fe-2S_thioredoxin ABHydrolase MBL-D-Ala-D-Ala-peptidase PBP5_C
MBA4327942.1
Polaromonas sp.
VanZ-1 6TM 2Fe-2S_thioredoxin ABHydrolase MBL-D-Ala-D-Ala-peptidase PBP5_C
WP_088453509.1
Roseateles terrae
VanZ-1 6TM 2Fe-2S_thioredoxin ABHydrolase MBL-D-Ala-D-Ala-peptidase PBP5_C
NBW44342.1
Betaproteobacteria bacterium
VanZ-1 6TM 2Fe-2S_thioredoxin ABHydrolase MBL-D-Ala-D-Ala-peptidase PBP5_C
MBH2043277.1
Comamonadaceae bacterium
VanZ-1 6TM 2Fe-2S_thioredoxin ABHydrolase MBL-D-Ala-D-Ala-peptidase PBP5_C
TXJ02629.1
Aquabacterium sp.
VanZ-1 6TM 2Fe-2S_thioredoxin ABHydrolase MBL-D-Ala-D-Ala-peptidase PBP5_C
MBJ7728745.1
VanZ-1 6TM 2Fe-2S_thioredoxin ABHydrolase MBL-D-Ala-D-Ala-peptidase PBP5_C
MBE7941659.1
Ramlibacter aquaticus
VanZ-1 6TM 2Fe-2S_thioredoxin ABHydrolase MBL-D-Ala-D-Ala-peptidase PBP5_C
WP_193675736.1
Ramlibacter pallidus
VanZ-1 6TM 2Fe-2S_thioredoxin ABHydrolase MBL-D-Ala-D-Ala-peptidase PBP5_C
WP_066475305.1
Comamonas terrae
VanZ-1 6TM 2Fe-2S_thioredoxin ABHydrolase MBL-D-Ala-D-Ala-peptidase PBP5_C
MBU0541078.1
Gammaproteobacteria bacterium
VanZ-1 6TM 2Fe-2S_thioredoxin ABHydrolase MBL-D-Ala-D-Ala-peptidase PBP5_C
MCD6680636.1
Burkholderiaceae bacterium
VanZ-1 6TM 2Fe-2S_thioredoxin ABHydrolase ? MBL-D-Ala-D-Ala-peptidase PBP5_C
MCD6732373.1
Burkholderiaceae bacterium
VanZ-1 6TM 2Fe-2S_thioredoxin ABHydrolase ? ? MBL-D-Ala-D-Ala-peptidase PBP5_C
MCD6674136.1
Burkholderiaceae bacterium
VanZ-1 6TM 2Fe-2S_thioredoxin ABHydrolase ? MBL-D-Ala-D-Ala-peptidase PBP5_C
BBB64663.1
Undibacterium sp. YM2
VanZ-1 6TM 2Fe-2S_thioredoxin ABHydrolase MBL-D-Ala-D-Ala-peptidase PBP5_C
EGF32269.1
Oxalobacteraceae bacterium IMCC9480
VanZ-1 6TM 2Fe-2S_thioredoxin ABHydrolase MBL-D-Ala-D-Ala-peptidase PBP5_C
MBC7683923.1
Massilia sp.
VanZ-1 6TM 2Fe-2S_thioredoxin ABHydrolase MBL-D-Ala-D-Ala-peptidase PBP5_C
WP_183682728.1
Janthinobacterium sp. K2E3
VanZ-1 6TM 2Fe-2S_thioredoxin ABHydrolase MBL-D-Ala-D-Ala-peptidase PBP5_C
WP_171088527.1
Telluria aromaticivorans
VanZ-1 6TM 2Fe-2S_thioredoxin ABHydrolase MBL-D-Ala-D-Ala-peptidase PBP5_C
MBA2413645.1
Burkholderiaceae bacterium
VanZ-1 6TM 2Fe-2S_thioredoxin ABHydrolase ? MBL-D-Ala-D-Ala-peptidase PBP5_C
MBV8666608.1
Burkholderiaceae bacterium
VanZ-1 6TM 2Fe-2S_thioredoxin ABHydrolase MBL-D-Ala-D-Ala-peptidase PBP5_C
MCE2679940.1
Burkholderiales bacterium
VanZ-1 6TM 2Fe-2S_thioredoxin ABHydrolase MBL-D-Ala-D-Ala-peptidase PBP5_C
NBO40842.1
Betaproteobacteria bacterium
VanZ-1 6TM 2Fe-2S_thioredoxin ABHydrolase MBL-D-Ala-D-Ala-peptidase PBP5_C
MCE2916678.1
Rubrivivax sp.
VanZ-1 6TM 2Fe-2S_thioredoxin ABHydrolase MBL-D-Ala-D-Ala-peptidase PBP5_C
NBX04797.1
Betaproteobacteria bacterium
VanZ-1 6TM 2Fe-2S_thioredoxin ABHydrolase MBL-D-Ala-D-Ala-peptidase PBP5_C
WP_231572153.1
Tepidimonas taiwanensis
VanZ-1 6TM 2Fe-2S_thioredoxin ABHydrolase MBL-D-Ala-D-Ala-peptidase PBP5_C
WP_231849284.1
Serpentinimonas raichei
VanZ-1 6TM 2Fe-2S_thioredoxin ABHydrolase MBL-D-Ala-D-Ala-peptidase PBP5_C
WP_050453186.1
Candidatus Burkholderia verschuerenii
VanZ-1 6TM 2Fe-2S_thioredoxin ABHydrolase ? PBP5_C MBL-D-Ala-D-Ala-peptidase
WP_183968086.1
Quisquiliibacterium transsilvanicum
VanZ-1 6TM 2Fe-2S_thioredoxin ABHydrolase MBL-D-Ala-D-Ala-peptidase PBP5_C
ESS15759.1
Betaproteobacteria bacterium MOLA814
VanZ-1 6TM 2Fe-2S_thioredoxin ABHydrolase MBL-D-Ala-D-Ala-peptidase PBP5_C
MBP6252201.1
Rubrivivax sp.
VanZ-1 6TM 2Fe-2S_thioredoxin ABHydrolase MBL-D-Ala-D-Ala-peptidase PBP5_C
WP_130431013.1
Rivibacter subsaxonicus
VanZ-1 6TM 2Fe-2S_thioredoxin ABHydrolase MBL-D-Ala-D-Ala-peptidase PBP5_C
MBV8635976.1
Burkholderiaceae bacterium
VanZ-1 6TM 2Fe-2S_thioredoxin ABHydrolase MBL-D-Ala-D-Ala-peptidase
WP_194720482.1
Noviherbaspirillum malthae
VanZ-1 6TM 2Fe-2S_thioredoxin ABHydrolase MBL-D-Ala-D-Ala-peptidase PBP5_C
WP_128198459.1
Rubrivivax albus
VanZ-1 6TM 2Fe-2S_thioredoxin ABHydrolase MBL-D-Ala-D-Ala-peptidase PBP5_C
WP_101103002.1
Macromonas bipunctata
VanZ-1 6TM 2Fe-2S_thioredoxin ABHydrolase MBL-D-Ala-D-Ala-peptidase PBP5_C
MBA4252574.1
Comamonadaceae bacterium
VanZ-1 6TM 2Fe-2S_thioredoxin ABHydrolase MBL-D-Ala-D-Ala-peptidase PBP5_C
RPH41946.1
Burkholderiales bacterium
VanZ-1 6TM 2Fe-2S_thioredoxin ABHydrolase ? MBL-D-Ala-D-Ala-peptidase PBP5_C
WP_213362189.1
Brachymonas denitrificans
VanZ-1 6TM 2Fe-2S_thioredoxin ABHydrolase MBL-D-Ala-D-Ala-peptidase PBP5_C
NLX16400.1
Ramlibacter sp.
VanZ-1 6TM 2Fe-2S_thioredoxin ABHydrolase MBL-D-Ala-D-Ala-peptidase PBP5_C
NBQ86453.1
Betaproteobacteria bacterium
VanZ-1 6TM 2Fe-2S_thioredoxin ABHydrolase MBL-D-Ala-D-Ala-peptidase PBP5_C
MBP7967988.1
Brachymonas sp.
VanZ-1 6TM 2Fe-2S_thioredoxin ABHydrolase MBL-D-Ala-D-Ala-peptidase PBP5_C
MBN9476921.1
Burkholderiales bacterium
VanZ-1 6TM 2Fe-2S_thioredoxin ABHydrolase MBL-D-Ala-D-Ala-peptidase PBP5_C
MBX7228317.1
Burkholderiaceae bacterium
VanZ-1 6TM 2Fe-2S_thioredoxin ABHydrolase ? MBL-D-Ala-D-Ala-peptidase PBP5_C
NQW94997.1
Polaromonas sp.
VanZ-1 6TM 2Fe-2S_thioredoxin ABHydrolase MBL-D-Ala-D-Ala-peptidase PBP5_C
MCE2660165.1
Rubrivivax sp.
VanZ-1 6TM 2Fe-2S_thioredoxin ABHydrolase MBL-D-Ala-D-Ala-peptidase PBP5_C
MBI1890279.1
Burkholderiales bacterium
VanZ-1 6TM 2Fe-2S_thioredoxin ABHydrolase MBL-D-Ala-D-Ala-peptidase PBP5_C
RJX33510.1
Oxalobacter sp.
VanZ-1 6TM ABHydrolase MBL-D-Ala-D-Ala-peptidase PBP5_C
MBK8767354.1
Burkholderiaceae bacterium
VanZ-1 6TM 2Fe-2S_thioredoxin ABHydrolase ? ? MBL-D-Ala-D-Ala-peptidase PBP5_C
MBW8779885.1
Burkholderiales bacterium
VanZ-1 6TM 2Fe-2S_thioredoxin ABHydrolase PSE MBL-D-Ala-D-Ala-peptidase
EHR73280.1
Burkholderiales bacterium JOSHI_001
VanZ-1 6TM 2Fe-2S_thioredoxin ABHydrolase MBL-D-Ala-D-Ala-peptidase PBP5_C
WP_143896516.1
Tepidimonas sediminis
VanZ-1 6TM 2Fe-2S_thioredoxin ABHydrolase MBL-D-Ala-D-Ala-peptidase PBP5_C
WP_143890464.1
Tepidimonas alkaliphilus
VanZ-1 6TM 2Fe-2S_thioredoxin ABHydrolase MBL-D-Ala-D-Ala-peptidase PBP5_C
WP_088387172.1
Roseateles aquatilis
VanZ-1 6TM 2Fe-2S_thioredoxin ABHydrolase MBL-D-Ala-D-Ala-peptidase PBP5_C
MBS0448959.1
Pseudomonadota bacterium
VanZ-1 6TM 2Fe-2S_thioredoxin ABHydrolase MBL-D-Ala-D-Ala-peptidase PBP5_C
MBK6865956.1
Ideonella sp.
VanZ-1 6TM 2Fe-2S_thioredoxin ABHydrolase MBL-D-Ala-D-Ala-peptidase PBP5_C
WP_232540283.1
Azohydromonas aeria
VanZ-1 6TM 2Fe-2S_thioredoxin ABHydrolase MBL-D-Ala-D-Ala-peptidase PBP5_C
MBL8288447.1
Rubrivivax sp.
VanZ-1 6TM 2Fe-2S_thioredoxin ABHydrolase MBL-D-Ala-D-Ala-peptidase PBP5_C
WP_184704775.1
Comamonas odontotermitis
VanZ-1 6TM 2Fe-2S_thioredoxin ABHydrolase MBL-D-Ala-D-Ala-peptidase PBP5_C
NBS95017.1
Betaproteobacteria bacterium
VanZ-1 6TM 2Fe-2S_thioredoxin ABHydrolase MBL-D-Ala-D-Ala-peptidase PBP5_C
UCH15965.1
Burkholderiales bacterium
VanZ-1 6TM 2Fe-2S_thioredoxin ABHydrolase MBL-D-Ala-D-Ala-peptidase PBP5_C
WP_163962522.1
Noviherbaspirillum galbum
VanZ-1 6TM 2Fe-2S_thioredoxin ABHydrolase MBL-D-Ala-D-Ala-peptidase PBP5_C
MCA0177809.1
Pseudomonadota bacterium
VanZ-1 6TM 2Fe-2S_thioredoxin ABHydrolase MBL-D-Ala-D-Ala-peptidase PBP5_C
MBI5333429.1
Burkholderiales bacterium
VanZ-1 6TM 2Fe-2S_thioredoxin ABHydrolase MBL-D-Ala-D-Ala-peptidase PBP5_C
MBK7614922.1
Candidatus Ideonella esbjergensis
VanZ-1 6TM 2Fe-2S_thioredoxin ABHydrolase MBL-D-Ala-D-Ala-peptidase PBP5_C
MBT9490274.1
Rubrivivax sp.
VanZ-1 6TM 2Fe-2S_thioredoxin ABHydrolase MBL-D-Ala-D-Ala-peptidase PBP5_C
NQW66316.1
Burkholderiales bacterium
VanZ-1 6TM 2Fe-2S_thioredoxin PSE MBL-D-Ala-D-Ala-peptidase PBP5_C
RQO54352.1
Paucibacter sp. KBW04
VanZ-1 6TM 2Fe-2S_thioredoxin ABHydrolase MBL-D-Ala-D-Ala-peptidase PBP5_C

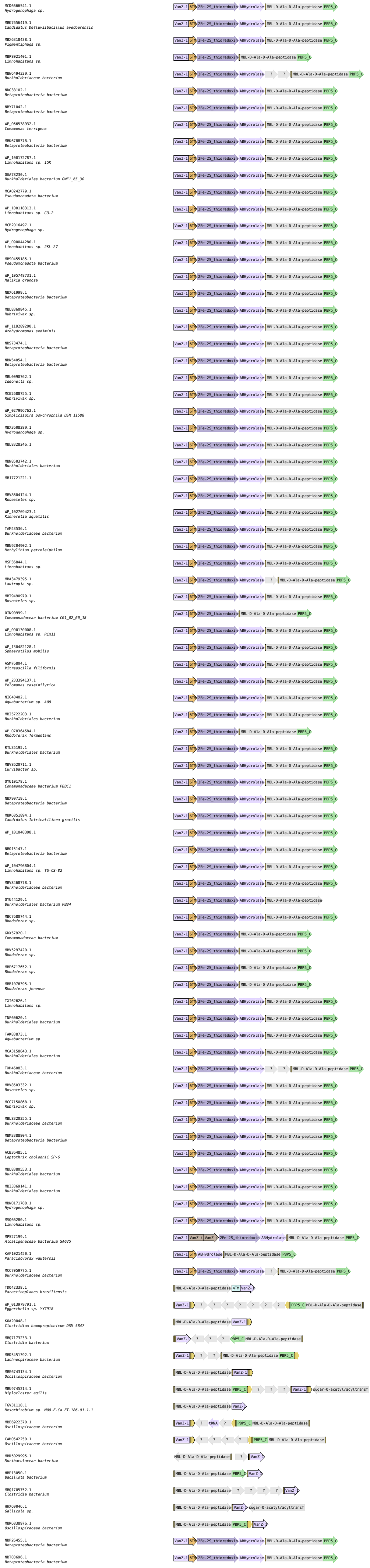
MCD6666541.1 Hydrogenophaga sp.
VanZ-1 6TM 2Fe-2S_thioredoxin ABHydrolase MBL-D-Ala-D-Ala-peptidase PBP5_C
MBK7656419.1 Candidatus Defluviibacillus avedoerensis
VanZ-1 6TM 2Fe-2S_thioredoxin ABHydrolase MBL-D-Ala-D-Ala-peptidase PBP5_C
MBX6318438.1 Pigmentiphaga sp.
VanZ-1 6TM 2Fe-2S_thioredoxin ABHydrolase MBL-D-Ala-D-Ala-peptidase PBP5_C
MBP8021401.1 Limnohabitans sp.
VanZ-1 6TM 2Fe-2S_thioredoxin MBL-D-Ala-D-Ala-peptidase PBP5_C
MBW6494329.1 Burkholderiaceae bacterium
VanZ-1 6TM 2Fe-2S_thioredoxin ABHydrolase ? ? MBL-D-Ala-D-Ala-peptidase PBP5_C
NDG38102.1 Betaproteobacteria bacterium
VanZ-1 6TM 2Fe-2S_thioredoxin ABHydrolase MBL-D-Ala-D-Ala-peptidase PBP5_C
NBY71042.1 Betaproteobacteria bacterium
VanZ-1 6TM 2Fe-2S_thioredoxin ABHydrolase MBL-D-Ala-D-Ala-peptidase PBP5_C
WP_066538932.1 Comamonas terrigena
VanZ-1 6TM 2Fe-2S_thioredoxin ABHydrolase MBL-D-Ala-D-Ala-peptidase PBP5_C
MBK6788378.1 Betaproteobacteria bacterium
VanZ-1 6TM 2Fe-2S_thioredoxin ABHydrolase MBL-D-Ala-D-Ala-peptidase PBP5_C
WP_100172787.1 Limnohabitans sp. 15K
VanZ-1 6TM 2Fe-2S_thioredoxin ABHydrolase MBL-D-Ala-D-Ala-peptidase PBP5_C
OGA78230.1 Burkholderiales bacterium GWE1_65_30
VanZ-1 6TM 2Fe-2S_thioredoxin ABHydrolase MBL-D-Ala-D-Ala-peptidase PBP5_C
MCA0242779.1 Pseudomonadota bacterium
VanZ-1 6TM 2Fe-2S_thioredoxin ABHydrolase MBL-D-Ala-D-Ala-peptidase PBP5_C
WP_100118313.1 Limnohabitans sp. G3-2
VanZ-1 6TM 2Fe-2S_thioredoxin ABHydrolase MBL-D-Ala-D-Ala-peptidase PBP5_C
MCB2016497.1 Hydrogenophaga sp.
VanZ-1 6TM 2Fe-2S_thioredoxin ABHydrolase MBL-D-Ala-D-Ala-peptidase PBP5_C
WP_090044280.1 Limnohabitans sp. 2KL-27
VanZ-1 6TM 2Fe-2S_thioredoxin ABHydrolase MBL-D-Ala-D-Ala-peptidase PBP5_C
MBS0455185.1 Pseudomonadota bacterium
VanZ-1 6TM 2Fe-2S_thioredoxin ABHydrolase MBL-D-Ala-D-Ala-peptidase PBP5_C
WP_105748731.1 Malikia granosa
VanZ-1 6TM 2Fe-2S_thioredoxin ABHydrolase MBL-D-Ala-D-Ala-peptidase PBP5_C
NBX61999.1 Betaproteobacteria bacterium
VanZ-1 6TM 2Fe-2S_thioredoxin ABHydrolase MBL-D-Ala-D-Ala-peptidase PBP5_C
MBL8360845.1 Rubrivivax sp.
VanZ-1 6TM 2Fe-2S_thioredoxin ABHydrolase MBL-D-Ala-D-Ala-peptidase PBP5_C
WP_119289200.1 Azohydromonas sediminis
VanZ-1 6TM 2Fe-2S_thioredoxin ABHydrolase MBL-D-Ala-D-Ala-peptidase PBP5_C
NBS73474.1 Betaproteobacteria bacterium
VanZ-1 6TM 2Fe-2S_thioredoxin ABHydrolase MBL-D-Ala-D-Ala-peptidase PBP5_C
NBW54054.1 Betaproteobacteria bacterium
VanZ-1 6TM 2Fe-2S_thioredoxin ABHydrolase MBL-D-Ala-D-Ala-peptidase PBP5_C
MBL0090762.1 Ideonella sp.
VanZ-1 6TM 2Fe-2S_thioredoxin ABHydrolase MBL-D-Ala-D-Ala-peptidase PBP5_C
MCE2688755.1 Rubrivivax sp.
VanZ-1 6TM 2Fe-2S_thioredoxin ABHydrolase MBL-D-Ala-D-Ala-peptidase PBP5_C
WP_027996762.1 Simplicispira psychrophila DSM 11588
VanZ-1 6TM 2Fe-2S_thioredoxin ABHydrolase MBL-D-Ala-D-Ala-peptidase PBP5_C
MBX3608289.1 Hydrogenophaga sp.
VanZ-1 6TM 2Fe-2S_thioredoxin ABHydrolase MBL-D-Ala-D-Ala-peptidase PBP5_C
MBL8328246.1
VanZ-1 6TM 2Fe-2S_thioredoxin ABHydrolase MBL-D-Ala-D-Ala-peptidase PBP5_C
MBN8503742.1 Burkholderiales bacterium
VanZ-1 6TM 2Fe-2S_thioredoxin ABHydrolase MBL-D-Ala-D-Ala-peptidase PBP5_C
MBJ7721221.1
VanZ-1 6TM 2Fe-2S_thioredoxin ABHydrolase MBL-D-Ala-D-Ala-peptidase PBP5_C
MBV8604124.1 Roseateles sp.
VanZ-1 6TM 2Fe-2S_thioredoxin ABHydrolase MBL-D-Ala-D-Ala-peptidase PBP5_C
WP_102769423.1 Kinneretia aquatilis
VanZ-1 6TM 2Fe-2S_thioredoxin ABHydrolase MBL-D-Ala-D-Ala-peptidase PBP5_C
TAM43536.1 Burkholderiaceae bacterium
VanZ-1 6TM 2Fe-2S_thioredoxin ABHydrolase MBL-D-Ala-D-Ala-peptidase PBP5_C
MBN9204902.1 Methylibium petroleiphilum
VanZ-1 6TM 2Fe-2S_thioredoxin ABHydrolase MBL-D-Ala-D-Ala-peptidase PBP5_C
MSP36844.1 Limnohabitans sp.
VanZ-1 6TM 2Fe-2S_thioredoxin ABHydrolase MBL-D-Ala-D-Ala-peptidase PBP5_C
MBA3479395.1 Lautropia sp.
VanZ-1 6TM 2Fe-2S_thioredoxin ABHydrolase ? MBL-D-Ala-D-Ala-peptidase PBP5_C
MBT9490979.1 Roseateles sp.
VanZ-1 6TM 2Fe-2S_thioredoxin ABHydrolase MBL-D-Ala-D-Ala-peptidase PBP5_C
OIN90999.1 Comamonadaceae bacterium CG1_02_60_18
VanZ-1 6TM 2Fe-2S_thioredoxin MBL-D-Ala-D-Ala-peptidase PBP5_C
WP_090130008.1 Limnohabitans sp. Rim11
VanZ-1 6TM 2Fe-2S_thioredoxin ABHydrolase MBL-D-Ala-D-Ala-peptidase PBP5_C
WP_130482128.1 Sphaerotilus mobilis
VanZ-1 6TM 2Fe-2S_thioredoxin ABHydrolase MBL-D-Ala-D-Ala-peptidase PBP5_C
ASM76804.1 Vitreoscilla filiformis
VanZ-1 6TM 2Fe-2S_thioredoxin ABHydrolase MBL-D-Ala-D-Ala-peptidase PBP5_C
WP_233394137.1 Pelomonas caseinilytica
VanZ-1 6TM 2Fe-2S_thioredoxin ABHydrolase MBL-D-Ala-D-Ala-peptidase PBP5_C
NIC40402.1 Aquabacterium sp. A08
VanZ-1 6TM 2Fe-2S_thioredoxin ABHydrolase MBL-D-Ala-D-Ala-peptidase PBP5_C
MBI5722203.1 Burkholderiales bacterium
VanZ-1 6TM 2Fe-2S_thioredoxin ABHydrolase MBL-D-Ala-D-Ala-peptidase PBP5_C
WP_078364584.1 Rhodoferax fermentans
VanZ-1 6TM 2Fe-2S_thioredoxin MBL-D-Ala-D-Ala-peptidase PBP5_C
RTL35195.1 Burkholderiales bacterium
VanZ-1 6TM 2Fe-2S_thioredoxin ABHydrolase MBL-D-Ala-D-Ala-peptidase PBP5_C
MBV8620711.1 Curvibacter sp.
VanZ-1 6TM 2Fe-2S_thioredoxin ABHydrolase MBL-D-Ala-D-Ala-peptidase PBP5_C
OYU10178.1 Comamonadaceae bacterium PBBC1
VanZ-1 6TM 2Fe-2S_thioredoxin ABHydrolase MBL-D-Ala-D-Ala-peptidase PBP5_C
NBX90719.1 Betaproteobacteria bacterium
VanZ-1 6TM 2Fe-2S_thioredoxin ABHydrolase MBL-D-Ala-D-Ala-peptidase PBP5_C
MBK6851894.1 Candidatus Intricatilinea gracilis
VanZ-1 6TM 2Fe-2S_thioredoxin ABHydrolase MBL-D-Ala-D-Ala-peptidase PBP5_C
WP_101048308.1
VanZ-1 6TM 2Fe-2S_thioredoxin ABHydrolase MBL-D-Ala-D-Ala-peptidase PBP5_C
NBO15147.1 Betaproteobacteria bacterium
VanZ-1 6TM 2Fe-2S_thioredoxin ABHydrolase MBL-D-Ala-D-Ala-peptidase PBP5_C
WP_104796804.1 Limnohabitans sp. TS-CS-82
VanZ-1 6TM 2Fe-2S_thioredoxin ABHydrolase MBL-D-Ala-D-Ala-peptidase PBP5_C
MBV8468778.1 Burkholderiaceae bacterium
VanZ-1 6TM 2Fe-2S_thioredoxin ABHydrolase MBL-D-Ala-D-Ala-peptidase PBP5_C
OYU44129.1 Burkholderiales bacterium PBB4
VanZ-1 6TM 2Fe-2S_thioredoxin ABHydrolase MBL-D-Ala-D-Ala-peptidase
MBC7680744.1 Rhodoferax sp.
VanZ-1 6TM 2Fe-2S_thioredoxin ABHydrolase MBL-D-Ala-D-Ala-peptidase PBP5_C
GDX57920.1 Comamonadaceae bacterium
VanZ-1 6TM 2Fe-2S_thioredoxin MBL-D-Ala-D-Ala-peptidase PBP5_C
MBV5297420.1 Rhodoferax sp.
VanZ-1 6TM 2Fe-2S_thioredoxin MBL-D-Ala-D-Ala-peptidase PBP5_C
MBP6717652.1 Rhodoferax sp.
VanZ-1 6TM 2Fe-2S_thioredoxin MBL-D-Ala-D-Ala-peptidase PBP5_C
MBB1076395.1 Rhodoferax jenense
VanZ-1 6TM 2Fe-2S_thioredoxin MBL-D-Ala-D-Ala-peptidase PBP5_C
TXI62626.1 Limnohabitans sp.
VanZ-1 6TM 2Fe-2S_thioredoxin ABHydrolase MBL-D-Ala-D-Ala-peptidase PBP5_C
TNF60620.1 Burkholderiales bacterium
VanZ-1 6TM 2Fe-2S_thioredoxin ABHydrolase MBL-D-Ala-D-Ala-peptidase PBP5_C
TAK83873.1 Aquabacterium sp.
VanZ-1 6TM 2Fe-2S_thioredoxin ABHydrolase MBL-D-Ala-D-Ala-peptidase PBP5_C
MCA3158843.1 Burkholderiales bacterium
VanZ-1 6TM 2Fe-2S_thioredoxin ABHydrolase MBL-D-Ala-D-Ala-peptidase PBP5_C
TXH46083.1 Burkholderiaceae bacterium
VanZ-1 6TM 2Fe-2S_thioredoxin ABHydrolase ? ? MBL-D-Ala-D-Ala-peptidase PBP5_C
MBV8503332.1 Roseateles sp.
VanZ-1 6TM 2Fe-2S_thioredoxin ABHydrolase MBL-D-Ala-D-Ala-peptidase PBP5_C
MCC7150868.1 Rubrivivax sp.
VanZ-1 6TM 2Fe-2S_thioredoxin ABHydrolase MBL-D-Ala-D-Ala-peptidase PBP5_C
MBL8320355.1 Burkholderiaceae bacterium
VanZ-1 6TM 2Fe-2S_thioredoxin ABHydrolase MBL-D-Ala-D-Ala-peptidase PBP5_C
MBM3388804.1 Betaproteobacteria bacterium
VanZ-1 6TM 2Fe-2S_thioredoxin ABHydrolase MBL-D-Ala-D-Ala-peptidase PBP5_C
ACB36485.1 Leptothrix cholodnii SP-6
VanZ-1 6TM 2Fe-2S_thioredoxin ABHydrolase MBL-D-Ala-D-Ala-peptidase PBP5_C
MBL8380553.1 Burkholderiales bacterium
VanZ-1 6TM 2Fe-2S_thioredoxin ABHydrolase MBL-D-Ala-D-Ala-peptidase PBP5_C
MBI3369141.1 Burkholderiales bacterium
VanZ-1 6TM 2Fe-2S_thioredoxin ABHydrolase MBL-D-Ala-D-Ala-peptidase PBP5_C
MBW0171788.1 Hydrogenophaga sp.
VanZ-1 6TM 2Fe-2S_thioredoxin ABHydrolase MBL-D-Ala-D-Ala-peptidase PBP5_C
MSQ66280.1 Limnohabitans sp.
VanZ-1 6TM 2Fe-2S_thioredoxin ABHydrolase MBL-D-Ala-D-Ala-peptidase PBP5_C
MPS27199.1 Alcaligenaceae bacterium SAGV5
VanZ-1 VanZ-i VanZ-1 2Fe-2S_thioredoxin ABHydrolase MBL-D-Ala-D-Ala-peptidase PBP5_C
KAF1021450.1 Paracidovorax wautersii
VanZ-1 6TM ABHydrolase MBL-D-Ala-D-Ala-peptidase PBP5_C
MCC7059775.1 Burkholderiaceae bacterium
VanZ-1 6TM 2Fe-2S_thioredoxin ABHydrolase ? MBL-D-Ala-D-Ala-peptidase PBP5_C
TDO42338.1 Paractinoplanes brasiliensis
MBL-D-Ala-D-Ala-peptidase 4TM VanZ-1
WP_013979791.1 Eggerthella sp. YY7918
VanZ-1 ? ? ? ? ? ? ? PBP5_C MBL-D-Ala-D-Ala-peptidase
KOA20048.1 Clostridium homopropionicum DSM 5847
MBL-D-Ala-D-Ala-peptidase VanZ-1
MBQ7173233.1 Clostridia bacterium
VanZ-1 ? ? ? PBP5_C MBL-D-Ala-D-Ala-peptidase
MBD5451392.1 Lachnospiraceae bacterium
VanZ-1 ? ? MBL-D-Ala-D-Ala-peptidase PBP5_C
MBE6743134.1 Oscillospiraceae bacterium
MBL-D-Ala-D-Ala-peptidase VanZ-1
MBU9745214.1 Diplocloster agilis
MBL-D-Ala-D-Ala-peptidase PBP5_C ? ? ? VanZ-1 sugar-O-acetyl/acyltransf
TGV31118.1 Mesorhizobium sp. M00.F.Ca.ET.186.01.1.1
MBL-D-Ala-D-Ala-peptidase VanZ-1
MBE6922370.1 Oscillospiraceae bacterium
VanZ-1 ? tRNA ? PBP5_C MBL-D-Ala-D-Ala-peptidase
CAH0542250.1 Oscillospiraceae bacterium
VanZ-1 ? ? ? ? PBP5_C MBL-D-Ala-D-Ala-peptidase
MBR5029995.1 Muribaculaceae bacterium
MBL-D-Ala-D-Ala-peptidase ? VanZ-1
HBP13050.1 Bacillota bacterium
MBL-D-Ala-D-Ala-peptidase PBP5_C VanZ-1
MBQ1705752.1 Clostridia bacterium
MBL-D-Ala-D-Ala-peptidase ? ? ? ? VanZ-1
HHX69046.1 Gallicola sp.
MBL-D-Ala-D-Ala-peptidase VanZ-1 sugar-O-acetyl/acyltransf
MBR6838976.1 Oscillospiraceae bacterium
MBL-D-Ala-D-Ala-peptidase PBP5_C VanZ-1
NBP26455.1 Betaproteobacteria bacterium
VanZ-1 6TM 2Fe-2S_thioredoxin ABHydrolase MBL-D-Ala-D-Ala-peptidase PBP5_C
NBT83696.1 Betaproteobacteria bacterium
VanZ-1 6TM 2Fe-2S_thioredoxin ABHydrolase MBL-D-Ala-D-Ala-peptidase PBP5_C

Accession	Organism	Gene neighborhood
NBT34341.1	*Betaproteobacteria bacterium*	VanZ-1, 6TM, 2Fe-2S_thioredoxin, ABHydrolase, MBL-D-Ala-D-Ala-peptidase, PBP5_C
MBM3376702.1	*Betaproteobacteria bacterium*	VanZ-1, 6TM, 2Fe-2S_thioredoxin, ABHydrolase, MBL-D-Ala-D-Ala-peptidase, PBP5_C
MBM3375930.1	*Betaproteobacteria bacterium*	VanZ-1, 6TM, 2Fe-2S_thioredoxin, ABHydrolase, MBL-D-Ala-D-Ala-peptidase, PBP5_C
MBU6304536.1	*Betaproteobacteria bacterium*	VanZ-1, 6TM, 2Fe-2S_thioredoxin, ABHydrolase, MBL-D-Ala-D-Ala-peptidase, PBP5_C
HCO57476.1		VanZ-1, 6TM, 2Fe-2S_thioredoxin, ABHydrolase, MBL-D-Ala-D-Ala-peptidase, PBP5_C
MRD56168.1	*Burkholderiales bacterium LSUCC0115*	VanZ-1, 6TM, 2Fe-2S_thioredoxin, ABHydrolase, MBL-D-Ala-D-Ala-peptidase, PBP5_C

#;fragmented operons

Accession	Organism	Gene neighborhood
MBE6898164.1	*Oscillospiraceae bacterium*	MBL-D-Ala-D-Ala-peptidase, PBP5_C, VanZ-1
MBR5219806.1	*Clostridia bacterium*	VanZ-1, PBP5_C, MBL-D-Ala-D-Ala-peptidase
MBQ8598517.1	*Oscillospiraceae bacterium*	MBL-D-Ala-D-Ala-peptidase, PBP5_C, VanZ-1
TDQ93056.1	*Paraburkholderia silvatlantica*	VanZ-1, 6TM, 2Fe-2S_thioredoxin, ABHydrolase
MBK9608303.1	*Betaproteobacteria bacterium*	VanZ-1, 6TM, 2Fe-2S_thioredoxin, ABHydrolase
MCE2948107.1	*Betaproteobacteria bacterium*	VanZ-1, 6TM, 2Fe-2S_thioredoxin, ABHydrolase
MBP6780509.1	*Ottowia sp.*	VanZ-1, 6TM, 2Fe-2S_thioredoxin
WP_051516766.1	*Herbaspirillum sp. RV1423*	VanZ-1, 6TM, 2Fe-2S_thioredoxin, ABHydrolase
MBP6138873.1	*Brachymonas sp.*	VanZ-1, 6TM, ABHydrolase
EAY63574.1	*Burkholderia cenocepacia PC184*	VanZ-1, 6TM, 2Fe-2S_thioredoxin, ABHydrolase
NBY63474.1	*Betaproteobacteria bacterium*	VanZ-1, 6TM, 2Fe-2S_thioredoxin, ABHydrolase
NBU78724.1	*Sphingomonadaceae bacterium*	VanZ-1, 6TM, 2Fe-2S_thioredoxin, ABHydrolase
BBJ00516.1	*Ferrigenium kumadai*	VanZ-1, 6TM, 2Fe-2S_thioredoxin, ABHydrolase
MCC2634540.1	*Ramlibacter sp.*	VanZ-1, 6TM, 2Fe-2S_thioredoxin, ABHydrolase
HGO59253.1		VanZ-1, 6TM, 2Fe-2S_thioredoxin, ABHydrolase
MBJ7379611.1	*Polynucleobacter sp.*	VanZ-1, 6TM, 2Fe-2S_thioredoxin, ABHydrolase
WP_153076440.1	*Paraburkholderia bonniea*	VanZ-1, 6TM, ?, 2Fe-2S_thioredoxin, ABHydrolase
MBM3337693.1	*Betaproteobacteria bacterium*	VanZ-1, 6TM, 2Fe-2S_thioredoxin, ABHydrolase
MCD6026695.1	*Solimicrobium sp.*	VanZ-1, 6TM
TAK92431.1	*Burkholderiaceae bacterium*	VanZ-1, 6TM
MCA3213372.1	*Burkholderiales bacterium*	VanZ-1, 6TM, 2Fe-2S_thioredoxin
WP_025918200.1	*Herminiimonas sp. CN*	VanZ-1, 6TM
KND54701.1	*Candidatus Paraburkholderia kirkii*	VanZ-1, 6TM, 2Fe-2S_thioredoxin, ABHydrolase
OZB69745.1	*Thiomonas sp. 13-64-67*	VanZ-1, 6TM, 2Fe-2S_thioredoxin, ABHydrolase
MBM3342085.1	*Betaproteobacteria bacterium*	VanZ-1, 6TM, 2Fe-2S_thioredoxin, ABHydrolase
MBP9217324.1	*Sterolibacterium sp.*	VanZ-1, 6TM
MBA3033266.1	*Rhodocyclaceae bacterium*	VanZ-1, 6TM
OQY65211.1	*Rhodocyclaceae bacterium UTPRO2*	VanZ-1, 6TM, ?, 2Fe-2S_thioredoxin, ABHydrolase
MSQ63003.1	*Betaproteobacteria bacterium*	VanZ-1, 6TM, 2Fe-2S_thioredoxin, ABHydrolase
PHV12718.1	*Chitinimonas sp. BJB300*	VanZ-1, 6TM
MBM3374007.1	*Betaproteobacteria bacterium*	VanZ-1, 6TM, ?, 2Fe-2S_thioredoxin
MCA2997960.1	*Rhodocyclaceae bacterium*	sugar-O-acetyl/acyltransf, VanZ-1, 6TM, 2Fe-2S_thioredoxin, ABHydrolase
NLD53590.1	*Burkholderiaceae bacterium*	VanZ-1, 6TM
WP_153472265.1	*Rhodocyclus gracilis*	VanZ-1, 6TM, 2Fe-2S_thioredoxin, ABHydrolase
MCC7483763.1	*Burkholderiales bacterium*	VanZ-1, 6TM, 2Fe-2S_thioredoxin, ABHydrolase
EPZ16806.1	*Thauera terpenica 58Eu*	VanZ-1, 6TM, 2Fe-2S_thioredoxin, ABHydrolase
SFZ76075.1	*Chitinimonas taiwanensis DSM 18899*	VanZ-1, 6TM
WP_144329177.1	*Tepidimonas charontis*	VanZ-1, 6TM, 2Fe-2S_thioredoxin, ABHydrolase
MBY0572652.1	*Undibacterium sp.*	VanZ-1, 6TM
MCE2947406.1	*Betaproteobacteria bacterium*	VanZ-1, 6TM
MCC7114398.1	*Burkholderiales bacterium*	VanZ-1, 6TM, 2Fe-2S_thioredoxin, ABHydrolase
RPH59047.1	*Burkholderiales bacterium*	VanZ-1, 6TM
MBS1154325.1	*Pseudomonadota bacterium*	VanZ-1, 6TM
WP_153114966.1	*Rhodocyclus tenuis*	VanZ-1, 6TM, 2Fe-2S_thioredoxin, ABHydrolase
MCE2989545.1	*Nitrosomonadaceae bacterium*	sugar-O-acetyl/acyltransf, VanZ-1, 6TM, 2Fe-2S_thioredoxin, ABHydrolase
MBC7647722.1	*Polaromonas sp.*	VanZ-1, 6TM, 2Fe-2S_thioredoxin, ABHydrolase
KPF66953.1	*beta proteobacterium AAP99*	VanZ-1, 6TM, 2Fe-2S_thioredoxin, ABHydrolase
WP_216183048.1	*Verticiella sp. GG226*	VanZ-1, 6TM
EXI69791.1	*Candidatus Accumulibacter adjunctus*	VanZ-1, 6TM
MBV8211259.1	*Burkholderiaceae bacterium*	VanZ-1, 6TM, 2Fe-2S_thioredoxin, ABHydrolase
MCC7549771.1	*Burkholderiales bacterium*	VanZ-1, 6TM, ?, ?, ?, ?, 2Fe-2S_thioredoxin, ABHydrolase
MBE2242040.1	*Burkholderiaceae bacterium*	VanZ-1, 6TM
CAI08153.1	*Aromatoleum aromaticum EbN1*	VanZ-1, 6TM, 2Fe-2S_thioredoxin, ABHydrolase
MBK7502120.1	*Candidatus Aalborgiella aggregata*	VanZ-1, 6TM
WP_026840907.1	*Citrifermentans bremense R1*	VanZ-1, 6TM
QHB57916.1	*Ralstonia solanacearum*	VanZ-1, 6TM
WP_144830617.1		VanZ-1, 6TM
WP_059414704.1	*Cupriavidus basilensis*	VanZ-1, 6TM
KKB64501.1	*Robbsia andropogonis*	VanZ-1, 6TM
AMS31533.1	*Betaproteobacteria bacterium UKL13-2*	sugar-O-acetyl/acyltransf, VanZ-1, 6TM, 2Fe-2S_thioredoxin, ABHydrolase
MBL8470573.1		VanZ-1, 6TM
NDH81855.1	*Burkholderiaceae bacterium*	VanZ-1, 6TM, 2Fe-2S_thioredoxin, ABHydrolase
NTU42495.1	*Nitrospirales bacterium*	VanZ-1, 6TM
MBV8465866.1	*Burkholderiales bacterium*	VanZ-1, 6TM
MBI5430611.1	*Nitrosomonadales bacterium*	VanZ-1, 6TM
WP_171013696.1	*Chitinivorax sp. B*	VanZ-1, 6TM
MBL8335113.1	*Rubrivivax sp.*	VanZ-1, 6TM, 2Fe-2S_thioredoxin
MBY0438033.1	*Burkholderiales bacterium*	VanZ-1, 6TM
PWT71962.1	*Pseudomonadota bacterium*	VanZ-1, 6TM
MBY0465077.1	*Burkholderiales bacterium*	VanZ-1, 6TM, 2Fe-2S_thioredoxin
NBU89200.1	*Betaproteobacteria bacterium*	VanZ-1, 6TM, 2Fe-2S_thioredoxin, ABHydrolase
MBI3528825.1	*Betaproteobacteria bacterium*	VanZ-1, 6TM, ?, 2Fe-2S_thioredoxin, ABHydrolase
OZB46058.1	*Thiomonas sp. 15-66-11*	VanZ-1, 6TM, 2Fe-2S_thioredoxin, ABHydrolase
MBI3043273.1	*Betaproteobacteria bacterium*	VanZ-1, 6TM, 2Fe-2S_thioredoxin, ABHydrolase
OFZ68967.1	*Betaproteobacteria bacterium RBG_16_56_24*	VanZ-1, 6TM, 2Fe-2S_thioredoxin
WP_169163860.1	*Azohydromonas caseinilytica*	VanZ-1, 6TM
EXI73578.1	*Candidatus Accumulibacter sp. SK-11*	VanZ-1, 6TM, ?, ?, ?, 2Fe-2S_thioredoxin, ?, ABHydrolase
MCA3134262.1	*Rhodocyclaceae bacterium*	VanZ-1, 6TM, 2Fe-2S_thioredoxin, ABHydrolase
MCC6867392.1	*Burkholderiales bacterium*	VanZ-1, 6TM, 2Fe-2S_thioredoxin, ABHydrolase
HCZ17264.1		VanZ-1, 6TM
MCE2969783.1	*Burkholderiales bacterium*	VanZ-1, 6TM, 2Fe-2S_thioredoxin, ABHydrolase
MSQ58127.1	*Betaproteobacteria bacterium*	VanZ-1, 6TM
MBV2235847.1	*Sterolibacterium sp.*	VanZ-1, 6TM
WP_170235417.1	*Verticiella sediminum*	VanZ-1, 6TM
WP_163314824.1		VanZ-1, 6TM
MCB1886598.1	*Rhodocyclaceae bacterium*	VanZ-1, 6TM, 2Fe-2S_thioredoxin, ABHydrolase

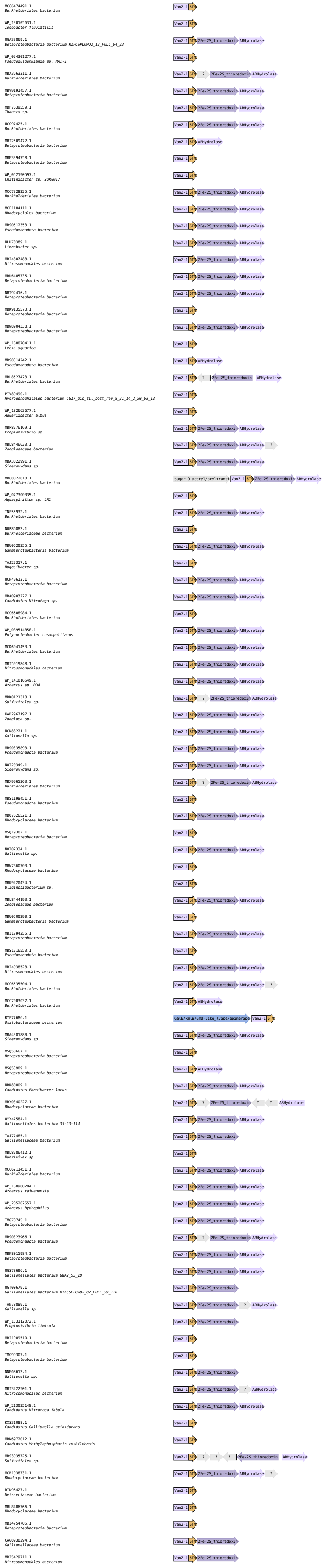
MCC6474491.1
Burkholderiales bacterium
VanZ-1 6TM
WP_130105631.1
Iodobacter fluviatilis
VanZ-1 6TM
OGA33869.1
Betaproteobacteria bacterium RIFCSPLOWO2_12_FULL_64_23
VanZ-1 6TM 2Fe-2S_thioredoxin ABHydrolase
WP_024301277.1
Pseudogulbenkiania sp. MAI-1
VanZ-1 6TM
MBX3663211.1
Burkholderiales bacterium
VanZ-1 6TM ? 2Fe-2S_thioredoxin ABHydrolase
MBV9191457.1
Betaproteobacteria bacterium
VanZ-1 6TM 2Fe-2S_thioredoxin ABHydrolase
MBP7639559.1
Thauera sp.
VanZ-1 6TM 2Fe-2S_thioredoxin ABHydrolase
UCG97425.1
Burkholderiales bacterium
VanZ-1 6TM 2Fe-2S_thioredoxin ABHydrolase
MBI2509472.1
Betaproteobacteria bacterium
VanZ-1 6TM ABHydrolase
MBM3394758.1
Betaproteobacteria bacterium
VanZ-1 6TM
WP_052190597.1
Chitinibacter sp. ZOR0017
VanZ-1 6TM
MCC7328225.1
Burkholderiales bacterium
VanZ-1 6TM 2Fe-2S_thioredoxin ABHydrolase
MCE1184111.1
Rhodocyclales bacterium
VanZ-1 6TM 2Fe-2S_thioredoxin ABHydrolase
MBS0512353.1
Pseudomonadota bacterium
VanZ-1 6TM 2Fe-2S_thioredoxin ABHydrolase
NLD70389.1
Limnobacter sp.
VanZ-1 6TM 2Fe-2S_thioredoxin ABHydrolase
MBI4807488.1
Nitrosomonadales bacterium
VanZ-1 6TM 2Fe-2S_thioredoxin ABHydrolase
MBU6485735.1
Betaproteobacteria bacterium
VanZ-1 6TM 2Fe-2S_thioredoxin ABHydrolase
NBT92416.1
Betaproteobacteria bacterium
VanZ-1 6TM 2Fe-2S_thioredoxin ABHydrolase
MBK9135573.1
Betaproteobacteria bacterium
VanZ-1 6TM
MBW8904338.1
Betaproteobacteria bacterium
VanZ-1 6TM 2Fe-2S_thioredoxin ABHydrolase
WP_168878411.1
Leeia aquatica
VanZ-1 6TM
MBS0314242.1
Pseudomonadota bacterium
VanZ-1 6TM ABHydrolase
MBL8527423.1
Burkholderiales bacterium
VanZ-1 6TM ? 2Fe-2S_thioredoxin ABHydrolase
PIV89490.1
Hydrogenophilales bacterium CG17_big_fil_post_rev_8_21_14_2_50_63_12
VanZ-1 6TM
WP_182663677.1
Aquariibacter albus
VanZ-1 6TM
MBP8276169.1
Propionivibrio sp.
VanZ-1 6TM 2Fe-2S_thioredoxin ABHydrolase
MBL8446623.1
Zoogloeaceae bacterium
VanZ-1 6TM 2Fe-2S_thioredoxin ABHydrolase ?
MBA3022991.1
Sideroxydans sp.
VanZ-1 6TM 2Fe-2S_thioredoxin ABHydrolase
MBC8022810.1
Burkholderiales bacterium
sugar-O-acetyl/acyltransf VanZ-1 6TM 2Fe-2S_thioredoxin ABHydrolase
WP_077300335.1
Aquaspirillum sp. LM1
VanZ-1 6TM
TNF55932.1
Burkholderiales bacterium
VanZ-1 6TM 2Fe-2S_thioredoxin ABHydrolase
NUP86882.1
Burkholderiaceae bacterium
VanZ-1 6TM
MBU0620355.1
Gammaproteobacteria bacterium
VanZ-1 6TM 2Fe-2S_thioredoxin ABHydrolase
TAJ22317.1
Rugosibacter sp.
VanZ-1 6TM
UCH49612.1
Betaproteobacteria bacterium
VanZ-1 6TM 2Fe-2S_thioredoxin ABHydrolase
MBA0903227.1
Candidatus Nitrotoga sp.
VanZ-1 6TM 2Fe-2S_thioredoxin ABHydrolase
MCC6608984.1
Burkholderiales bacterium
VanZ-1 6TM
WP_089514858.1
Polynucleobacter cosmopolitanus
VanZ-1 6TM 2Fe-2S_thioredoxin ABHydrolase
MCD6041453.1
Burkholderiales bacterium
VanZ-1 6TM 2Fe-2S_thioredoxin ABHydrolase
MBI5919848.1
Nitrosomonadales bacterium
VanZ-1 6TM 2Fe-2S_thioredoxin ABHydrolase
WP_141016549.1
Azoarcus sp. DD4
VanZ-1 6TM 2Fe-2S_thioredoxin ABHydrolase
MBK8121318.1
Sulfuritalea sp.
VanZ-1 6TM ? 2Fe-2S_thioredoxin ABHydrolase
KAB2967197.1
Zoogloea sp.
VanZ-1 6TM 2Fe-2S_thioredoxin ABHydrolase
NCN88221.1
Gallionella sp.
VanZ-1 6TM 2Fe-2S_thioredoxin ABHydrolase
MBS0335893.1
Pseudomonadota bacterium
VanZ-1 6TM 2Fe-2S_thioredoxin ABHydrolase
NOT20349.1
Sideroxydans sp.
VanZ-1 6TM 2Fe-2S_thioredoxin ABHydrolase
MBX9965363.1
Burkholderiales bacterium
VanZ-1 6TM ? 2Fe-2S_thioredoxin ABHydrolase
MBS1198451.1
Pseudomonadota bacterium
VanZ-1 6TM
MBQ7626521.1
Rhodocyclaceae bacterium
VanZ-1 6TM 2Fe-2S_thioredoxin ABHydrolase
MSQ19382.1
Betaproteobacteria bacterium
VanZ-1 6TM
NOT82334.1
Gallionella sp.
VanZ-1 6TM 2Fe-2S_thioredoxin ABHydrolase
MBW7860703.1
Rhodocyclaceae bacterium
VanZ-1 6TM
MBK9220434.1
Uliginosibacterium sp.
VanZ-1 6TM
MBL8444193.1
Zoogloeaceae bacterium
VanZ-1 6TM 2Fe-2S_thioredoxin ABHydrolase
MBU0500290.1
Gammaproteobacteria bacterium
VanZ-1 6TM
MBI1394355.1
Betaproteobacteria bacterium
VanZ-1 6TM 2Fe-2S_thioredoxin ABHydrolase
MBS1216553.1
Pseudomonadota bacterium
VanZ-1 6TM
MBI4938528.1
Nitrosomonadales bacterium
VanZ-1 6TM 2Fe-2S_thioredoxin ABHydrolase
MCC6535504.1
Burkholderiales bacterium
VanZ-1 6TM 2Fe-2S_thioredoxin ABHydrolase ?
MCC7083037.1
Burkholderiales bacterium
VanZ-1 6TM ABHydrolase
RYE77686.1
Oxalobacteraceae bacterium
GalE/RmlB/Gmd-like_lyase/epimerase VanZ-1 6TM
MBA4381880.1
Sideroxydans sp.
VanZ-1 6TM 2Fe-2S_thioredoxin ABHydrolase
MSQ50667.1
Betaproteobacteria bacterium
VanZ-1 6TM
MSQ53909.1
Betaproteobacteria bacterium
VanZ-1 6TM ABHydrolase
NBR80809.1
Candidatus Fonsibacter lacus
VanZ-1 6TM 2Fe-2S_thioredoxin ABHydrolase
MBY0340227.1
Rhodocyclaceae bacterium
VanZ-1 6TM ? 2Fe-2S_thioredoxin ? ? ABHydrolase
OYY47584.1
Gallionellales bacterium 35-53-114
VanZ-1 6TM 2Fe-2S_thioredoxin ABHydrolase
TAJ77485.1
Gallionellaceae bacterium
VanZ-1 6TM 2Fe-2S_thioredoxin
MBL8286412.1
Rubrivivax sp.
VanZ-1 6TM
MCC6211451.1
Burkholderiales bacterium
VanZ-1 6TM 2Fe-2S_thioredoxin ABHydrolase
WP_168988204.1
Azoarcus taiwanensis
VanZ-1 6TM 2Fe-2S_thioredoxin ABHydrolase
WP_205202557.1
Azonexus hydrophilus
VanZ-1 6TM 2Fe-2S_thioredoxin ABHydrolase
TMG78745.1
Betaproteobacteria bacterium
VanZ-1 6TM 2Fe-2S_thioredoxin ABHydrolase
MBS0323966.1
Pseudomonadota bacterium
VanZ-1 6TM ? 2Fe-2S_thioredoxin ABHydrolase
MBK8015984.1
Betaproteobacteria bacterium
VanZ-1 6TM 2Fe-2S_thioredoxin ABHydrolase
OGS78696.1
Gallionellales bacterium GWA2_55_18
VanZ-1 6TM 2Fe-2S_thioredoxin ABHydrolase
OGT00679.1
Gallionellales bacterium RIFCSPLOWO2_02_FULL_59_110
VanZ-1 6TM 2Fe-2S_thioredoxin
TAN78889.1
Gallionella sp.
VanZ-1 6TM 2Fe-2S_thioredoxin ? ABHydrolase
WP_153112072.1
Propionivibrio limicola
VanZ-1 6TM 2Fe-2S_thioredoxin
MBI1989510.1
Betaproteobacteria bacterium
VanZ-1 6TM
TMG99307.1
Betaproteobacteria bacterium
VanZ-1 6TM
NNM68612.1
Gallionella sp.
VanZ-1 6TM 2Fe-2S_thioredoxin
MBI3222501.1
Nitrosomonadales bacterium
VanZ-1 6TM 2Fe-2S_thioredoxin ? ABHydrolase
WP_213035148.1
Candidatus Nitrotoga fabula
VanZ-1 6TM 2Fe-2S_thioredoxin ABHydrolase
KXS31088.1
Candidatus Gallionella acididurans
VanZ-1 6TM
MBK6972012.1
Candidatus Methylophosphatis roskildensis
VanZ-1 6TM
MBS3935725.1
Sulfuritalea sp.
VanZ-1 6TM ? ? ? 2Fe-2S_thioredoxin ABHydrolase
MCB1938731.1
Rhodocyclaceae bacterium
VanZ-1 6TM 2Fe-2S_thioredoxin ABHydrolase ?
RTK96427.1
Neisseriaceae bacterium
VanZ-1 6TM
MBL8486766.1
Rhodocyclaceae bacterium
VanZ-1 6TM
MBI4754705.1
Betaproteobacteria bacterium
VanZ-1 6TM
CAG0938294.1
Gallionellaceae bacterium
VanZ-1 6TM 2Fe-2S_thioredoxin
MBI5429711.1
Nitrosomonadales bacterium
VanZ-1 6TM 2Fe-2S_thioredoxin

MBI5329165.1	*Betaproteobacteria bacterium*	VanZ-1 6TM
WP_206254728.1	*Niveibacterium microcysteis*	VanZ-1 6TM
MBI5862033.1	*Rhodocyclales bacterium*	VanZ-1 6TM
TAG49056.1	*Betaproteobacteria bacterium*	VanZ-1 6TM ABHydrolase
MBI3376163.1	*Betaproteobacteria bacterium*	VanZ-1 6TM
MBL8311938.1	*Burkholderiales bacterium*	VanZ-1 6TM ABHydrolase
MBS1191959.1	*Rhodocyclaceae bacterium*	VanZ-1 6TM ? 2Fe-2S_thioredoxin ABHydrolase
MBM4181758.1	*Betaproteobacteria bacterium*	VanZ-1 6TM
MBK7417409.1	*Candidatus Dechloromonas phosphorivorans*	VanZ-1 6TM PSE 2Fe-2S_thioredoxin ABHydrolase
TAK88250.1	*Betaproteobacteria bacterium*	VanZ-1 6TM 2Fe-2S_thioredoxin ABHydrolase
MBV2192844.1	*Azonexus sp.*	VanZ-1 6TM ? ? ? ? 2Fe-2S_thioredoxin ABHydrolase
MBY0346102.1	*Neisseriaceae bacterium*	VanZ-1 6TM
QWT47598.1	*Azospira inquinata*	VanZ-1 6TM 2Fe-2S_thioredoxin ABHydrolase
OGA16356.1	*Betaproteobacteria bacterium RIFCSPLOWO2_02_FULL_66_14*	VanZ-1 6TM 2Fe-2S_thioredoxin ABHydrolase
WP_102245898.1	*Pseudazoarcus pumilus*	VanZ-1 6TM 2Fe-2S_thioredoxin ABHydrolase
MBU1362693.1	*Gammaproteobacteria bacterium*	VanZ-1 6TM ? ? 2Fe-2S_thioredoxin ABHydrolase
MBI4985876.1	*Rhodocyclales bacterium*	VanZ-1 6TM ? 2Fe-2S_thioredoxin ABHydrolase
WP_152090126.1	*Azospira sp. I09*	VanZ-1 6TM 2Fe-2S_thioredoxin ABHydrolase
MBI4998323.1	*Rhodocyclales bacterium*	VanZ-1 6TM
NWG31865.1	*Rhodocyclaceae bacterium*	VanZ-1 6TM ? ? 2Fe-2S_thioredoxin ABHydrolase
WP_170019784.1	*Uliginosibacterium aquaticum*	VanZ-1 6TM
MBL8448306.1	*Dechloromonas sp.*	VanZ-1 6TM
MBP5997119.1	*Azonexus sp.*	VanZ-1 6TM
MCC7005559.1	*Ottowia sp.*	VanZ-1 6TM ABHydrolase
RMH61493.1	*Calditrichota bacterium*	VanZ-1 6TM
MBV8032672.1	*Betaproteobacteria bacterium*	VanZ-1 6TM 2Fe-2S_thioredoxin ABHydrolase
MBK6639566.1	*Rhodocyclaceae bacterium*	VanZ-1 6TM ? 2Fe-2S_thioredoxin ABHydrolase
OGA44847.1	*Betaproteobacteria bacterium RIFCSPLOWO2_12_FULL_62_13*	VanZ-1 6TM
NJD87723.1	*Betaproteobacteria bacterium*	sugar-O-acetyl/acyltransf VanZ-1 6TM 2Fe-2S_thioredoxin ABHydrolase
MBK9623633.1	*Rhodocyclaceae bacterium*	VanZ-1 6TM ? 2Fe-2S_thioredoxin ABHydrolase
MBI3939095.1	*Betaproteobacteria bacterium*	VanZ-1 6TM
MBS1218012.1	*Pseudomonadota bacterium*	VanZ-1 6TM ? ? ABHydrolase
WP_171165194.1	*Usitatibacter palustris*	sugar-O-acetyl/acyltransf VanZ-1 6TM 2Fe-2S_thioredoxin ABHydrolase
MBC7945139.1	*Burkholderiales bacterium*	VanZ-1 6TM 2Fe-2S_thioredoxin ? ABHydrolase
MBL8516153.1	*Betaproteobacteria bacterium*	sugar-O-acetyl/acyltransf VanZ-1 6TM 2Fe-2S_thioredoxin ABHydrolase
NWG86457.1	*Hydrogenophilaceae bacterium*	VanZ-1 6TM
MBI5937993.1	*Betaproteobacteria bacterium*	VanZ-1 6TM
MBP8867252.1	*Propionivibrio sp.*	VanZ-1 6TM
MBL8523752.1	*Betaproteobacteria bacterium*	sugar-O-acetyl/acyltransf VanZ-1 6TM 2Fe-2S_thioredoxin ABHydrolase
TAG84828.1	*Burkholderiales bacterium*	VanZ-1 6TM
PIU16653.1	*Gallionellales bacterium CG08_land_8_20_14_0_20_59_87*	VanZ-1 6TM 2Fe-2S_thioredoxin ABHydrolase
HEB98136.1	*Thiotrichales bacterium*	WecC-like-oxidoreductase GalE/RmlB/Gmd-like_lyase/epimerase VanZ-1 6TM
PIV72159.1	*Rhodocyclales bacterium CG17_big_fil_post_rev_8_21_14_2_50_68_7*	VanZ-1 6TM ? ? ? ? ?
MBU0750635.1	*Gammaproteobacteria bacterium*	VanZ-1 6TM
MBI3148183.1	*Betaproteobacteria bacterium*	VanZ-1 6TM 2Fe-2S_thioredoxin ABHydrolase
APV49382.1	*Betaproteobacteria bacterium GR16-43*	sugar-O-acetyl/acyltransf VanZ-1 6TM 2Fe-2S_thioredoxin ABHydrolase
MBM4190122.1	*Betaproteobacteria bacterium*	VanZ-1 6TM
MBX9904329.1	*Burkholderiales bacterium*	VanZ-1 6TM ? 2Fe-2S_thioredoxin ABHydrolase
NNM82409.1	*Burkholderiales bacterium*	VanZ-1 6TM 2Fe-2S_thioredoxin ABHydrolase
OGA53003.1	*Betaproteobacteria bacterium RIFCSPLOWO2_12_FULL_63_13*	VanZ-1 6TM 2Fe-2S_thioredoxin ?
OAI53413.1	*Betaproteobacteria bacterium SCGC AG-212-J23*	VanZ-1 6TM 2Fe-2S_thioredoxin ABHydrolase
MBI2311470.1	*Betaproteobacteria bacterium*	VanZ-1 6TM
NJR72246.1	*Gammaproteobacteria bacterium*	VanZ-1 6TM
OGB85771.1	*Burkholderiales bacterium RIFOXYD2_FULL_59_8*	VanZ-1 6TM 2Fe-2S_thioredoxin
OYU30031.1	*Comamonadaceae bacterium PBBC2*	VanZ-1 6TM
OYU67796.1	*Burkholderiales bacterium PBB5*	VanZ-1 6TM
MBM3385699.1	*Betaproteobacteria bacterium*	VanZ-1 6TM
MBM3345394.1	*Betaproteobacteria bacterium*	VanZ-1 6TM 2Fe-2S_thioredoxin ABHydrolase
TMH55556.1	*Betaproteobacteria bacterium*	VanZ-1 6TM 2Fe-2S_thioredoxin ABHydrolase
OGA26975.1	*Betaproteobacteria bacterium RIFCSPLOWO2_02_FULL_65_24*	VanZ-1 6TM 2Fe-2S_thioredoxin ABHydrolase
MBI2319314.1	*Betaproteobacteria bacterium*	VanZ-1 6TM 2Fe-2S_thioredoxin ABHydrolase

#;direct fusions VanZ-1 and D-Ala-D-Ala-peptidase

WP_051280491.1	*Anaerovorax odorimutans DSM 5092*	VanZ-1 MBL-D-Ala-D-Ala-peptidase
MBQ4435587.1	*Clostridia bacterium*	VanZ-1 MBL-D-Ala-D-Ala-peptidase
MBQ3278630.1	*Clostridia bacterium*	VanZ-1 MBL-D-Ala-D-Ala-peptidase
MBQ1365495.1	*Clostridia bacterium*	VanZ-1 MBL-D-Ala-D-Ala-peptidase
MBR3333151.1	*Clostridia bacterium*	VanZ-1 MBL-D-Ala-D-Ala-peptidase
MBR6028784.1	*Clostridia bacterium*	VanZ-1 MBL-D-Ala-D-Ala-peptidase
MBR5109406.1	*Clostridia bacterium*	VanZ-1 MBL-D-Ala-D-Ala-peptidase
HJA42093.1	*Bacillota bacterium*	VanZ-1 MBL-D-Ala-D-Ala-peptidase
PKM71660.1	*Firmicutes bacterium HGW-Firmicutes-16*	VanZ-1 MBL-D-Ala-D-Ala-peptidase
OUQ27743.1	*Lachnoclostridium sp. An131*	VanZ-1 MBL-D-Ala-D-Ala-peptidase
HAB60708.1	*Lachnospiraceae bacterium*	VanZ-1 MBL-D-Ala-D-Ala-peptidase
WP_170164349.1	*Mobilisporobacter senegalensis*	VanZ-1 MBL-D-Ala-D-Ala-peptidase
WP_049898308.1	*Ruminococcus gauvreauii*	VanZ-1 MBL-D-Ala-D-Ala-peptidase
SCI57623.1	*uncultured Clostridium sp.*	VanZ-1 MBL-D-Ala-D-Ala-peptidase
BBF43414.1	*Lachnospiraceae bacterium KM106-2*	VanZ-1 MBL-D-Ala-D-Ala-peptidase monofunctional-peptidoglycan-glycosyltransferase
MBQ8683165.1	*Clostridia bacterium*	VanZ-1 VanZ-1
OGX08794.1	*Omnitrophica WOR_2 bacterium GWA2_47_8*	VanZ-1
ASS41582.1	*Eubacterium minutum ATCC 700079*	VanZ-1

#;MurB reductase association

PKG22275.1	*Niallia nealsonii*	VanZ-1 MurB-reductase
WP_213168301.1	*Alkalicella caledoniensis*	VanZ-1 MurB-reductase
WP_090858450.1	*Paraliobacillus sp. PM-2*	MurB-reductase VanZ-1
PVE19077.1	*Arthrobacter sp. Bz4*	MurB-reductase ? ? ? VanZ-1 WecC-like-oxidoreductase ? GalE/RmlB/Gmd-like_lyase/epimerase GDP-L-fucose-synthase RfbH-like-oxidoreductase
WP_066039403.1	*Herbiconiux solani NBRC 106740*	VanZ-1 ? ? ? ? MurB-reductase
WP_007547505.1	*Listeria fleischmannii subsp. coloradonensis*	MurB-reductase ? ? ? VanZ-1
WP_185348099.1	*Listeria immobilis*	MurB-reductase ? ? ? VanZ-1
WP_099221243.1	*Listeria costaricensis*	MurB-reductase ? ? ? VanZ-1
MBS6371236.1	*Oscillospiraceae bacterium*	MurB-reductase ? ? ? VanZ-1
MBE6891130.1	*Oscillospiraceae bacterium*	VanZ-1 ? ? ? ? ? ? MurB-reductase

#;MraY-associating-this is probably adding to the sugar chain in some pathways...

MBW1692087.1	*Deltaproteobacteria bacterium*	GalE/RmlB/Gmd-like_lyase/epimerase MraY/WecA-GTase VanZ-1
MBZ9578193.1	*Patescibacteria group bacterium*	LPS-like-sugar-transf GalE/RmlB/Gmd-like_lyase/epimerase MraY/WecA-GTase WaaL-lipidA-ligase VanZ-1

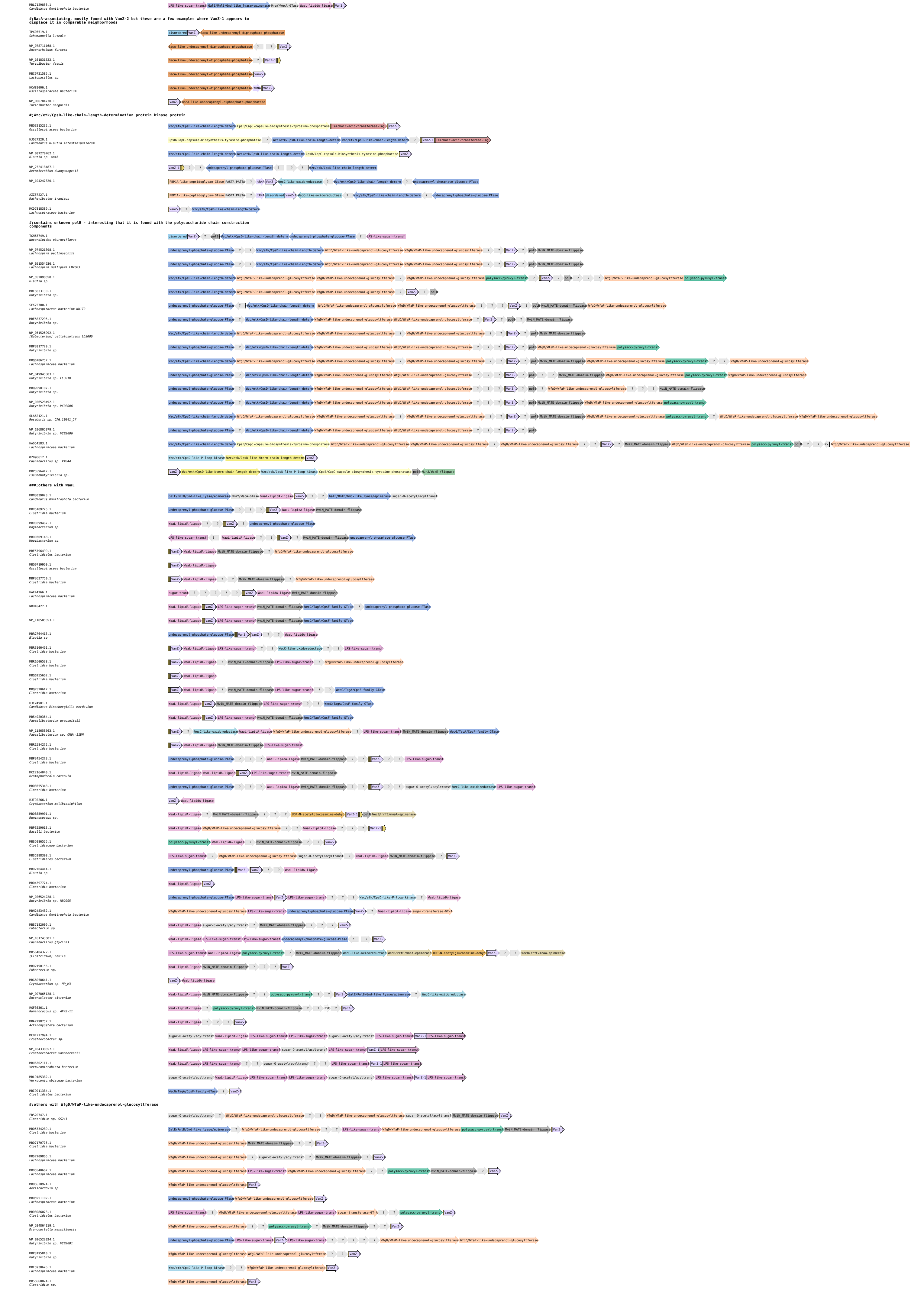
#;BacA-associating, mostly found with VanZ-2 but these are a few examples where VanZ-1 appears to displace it in comparable neighborhoods
#;Wzc/etk/CpsD-like-chain-length-determination protein kinase protein
#;contains unknown polB - interesting that it is found with the polysaccharide chain construction components
###;others with WaaL
#;others with WfgD/WfaP-like-undecaprenol-glucosyltransferase

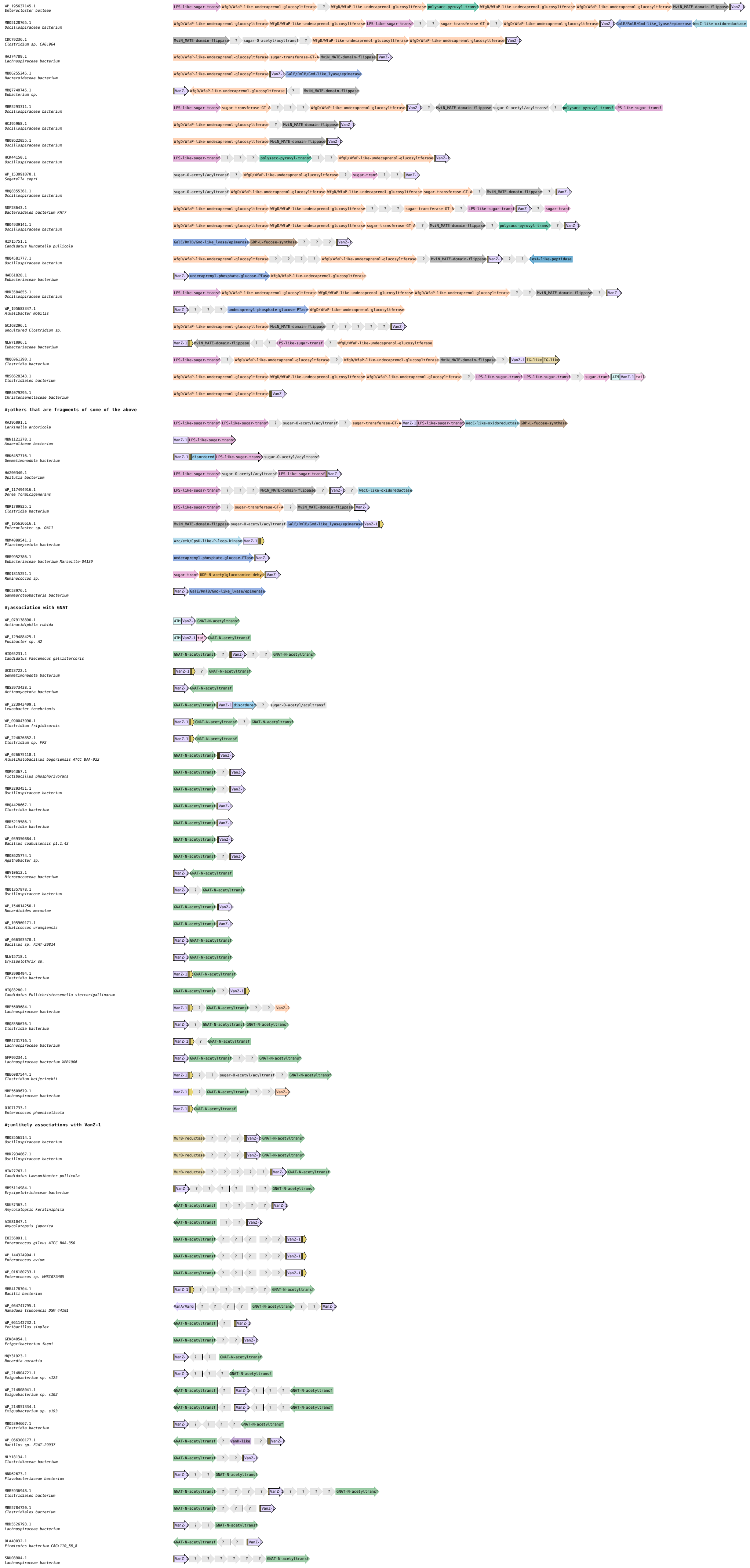
WP_195637145.1
Enterocloster bolteae
MBO5128765.1
Oscillospiraceae bacterium
CDC79236.1
Clostridium sp. CAG:964
HAJ74789.1
Lachnospiraceae bacterium
MBO6255245.1
Bacteroidaceae bacterium
MBQ7740745.1
Eubacterium sp.
MBR5293311.1
Oscillospiraceae bacterium
HCJ95968.1
Oscillospiraceae bacterium
MBO8622055.1
Oscillospiraceae bacterium
HCK44150.1
Oscillospiraceae bacterium
WP_153091070.1
Segatella copri
MBQ8355361.1
Oscillospiraceae bacterium
SDF28643.1
Bacteroidales bacterium KHT7
MBO4939141.1
Oscillospiraceae bacterium
HIX15751.1
Candidatus Hungatella pullicola
MBQ4581777.1
Oscillospiraceae bacterium
HAE61828.1
Eubacteriaceae bacterium
MBR3584855.1
Oscillospiraceae bacterium
WP_195683347.1
Alkalibacter mobilis
SCJ68296.1
uncultured Clostridium sp.
NLW71096.1
Eubacteriaceae bacterium
MBO6961290.1
Clostridia bacterium
MBS6628343.1
Clostridiales bacterium
MBR4079295.1
Christensenellaceae bacterium
#;others that are fragments of some of the above
RAJ96091.1
Larkinella arboricola
MBN1121278.1
Anaerolineae bacterium
MBK6457716.1
Gemmatimonadota bacterium
HAZ00340.1
Opitutia bacterium
WP_117494916.1
Dorea formicigenerans
MBR1709825.1
Clostridia bacterium
WP_195626616.1
Enterocloster sp. OA11
MBM4099541.1
Planctomycetota bacterium
MBR9952386.1
Eubacteriaceae bacterium Marseille-Q4139
MBQ1815251.1
Ruminococcus sp.
MBC53976.1
Gammaproteobacteria bacterium
#;association with GNAT
WP_079138890.1
Actinacidiphila rubida
WP_129488425.1
Fusibacter sp. A2
HIQ65231.1
Candidatus Faecenecus gallistercoris
UCD23722.1
Gemmatimonadota bacterium
MBS3973438.1
Actinomycetota bacterium
WP_223043409.1
Leucobacter tenebrionis
WP_090043090.1
Clostridium frigidicarnis
WP_224626852.1
Clostridium sp. FP2
WP_026675118.1
Alkalihalobacillus bogoriensis ATCC BAA-922
MQR94367.1
Fictibacillus phosphorivorans
MBR3293451.1
Oscillospiraceae bacterium
MBQ4428667.1
Clostridia bacterium
MBR5219586.1
Clostridia bacterium
WP_059350884.1
Bacillus coahuilensis p1.1.43
MBQ8625774.1
Agathobacter sp.
HBV10612.1
Micrococcaceae bacterium
MBQ1357878.1
Oscillospiraceae bacterium
WP_154614250.1
Nocardioides marmotae
WP_105960171.1
Alkalicoccus urumqiensis
WP_066303570.1
Bacillus sp. FJAT-29814
NLW15718.1
Erysipelothrix sp.
MBR3998494.1
Clostridia bacterium
HIQ83280.1
Candidatus Pullichristensenella stercorigallinarum
MBP5609684.1
Lachnospiraceae bacterium
MBO8556676.1
Clostridia bacterium
MBR4731716.1
Lachnospiraceae bacterium
SFP99234.1
Lachnospiraceae bacterium XBB1006
MBE6087544.1
Clostridium beijerinckii
MBP5609679.1
Lachnospiraceae bacterium
OJG71733.1
Enterococcus phoeniculicola
#;unlikely associations with VanZ-1
MBQ3556514.1
Oscillospiraceae bacterium
MBR2934867.1
Oscillospiraceae bacterium
HIW27767.1
Candidatus Lawsonibacter pullicola
MBS5114984.1
Erysipelotrichaceae bacterium
SDU57363.1
Amycolatopsis keratiniphila
AIG81047.1
Amycolatopsis japonica
EOI56091.1
Enterococcus gilvus ATCC BAA-350
WP_144324904.1
Enterococcus avium
WP_016180733.1
Enterococcus sp. HMSC072H05
MBR4178704.1
Bacilli bacterium
WP_064741795.1
Hamadaea tsunoensis DSM 44101
WP_061142732.1
Peribacillus simplex
GEK84054.1
Frigoribacterium faeni
MQY31923.1
Nocardia aurantia
WP_214804721.1
Exiguobacterium sp. s125
WP_214808041.1
Exiguobacterium sp. s102
WP_214851334.1
Exiguobacterium sp. s193
MBO5394667.1
Clostridia bacterium
WP_066300177.1
Bacillus sp. FJAT-29037
NLY18134.1
Clostridiaceae bacterium
NAD62673.1
Flavobacteriaceae bacterium
MBR5936948.1
Clostridiales bacterium
MBE5784720.1
Clostridiales bacterium
MBO5526793.1
Lachnospiraceae bacterium
OLA40032.1
Firmicutes bacterium CAG:110_56_8
SNU08904.1
Lachnospiraceae bacterium

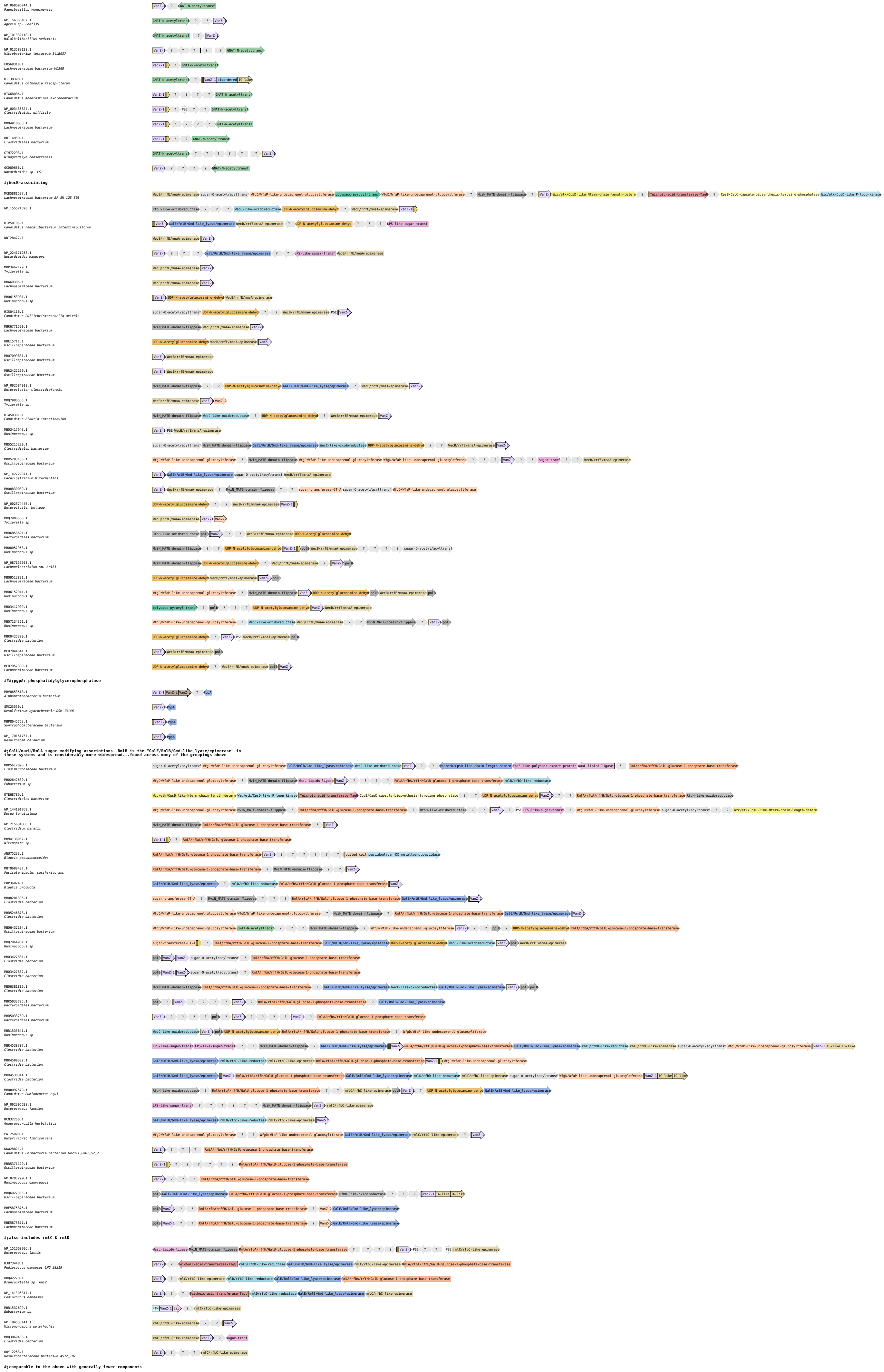
WP_068696744.1
Paenibacillus yonginensis
WP_156366107.1
Agreia sp. Leaf335
WP_101332118.1
Halalkalibacillus sediminis
WP_013583128.1
Microbacterium testaceum StLB037
EOS68310.1
Lachnospiraceae bacterium MD308
HIT38390.1
Candidatus Onthousia faecipullorum
HIX68086.1
Candidatus Anaerostipes excrementavium
WP_003436824.1
Clostridioides difficile
MBO4910663.1
Lachnospiraceae bacterium
HHT14950.1
Clostridiales bacterium
GIM72293.1
Winogradskya consettensis
GCD89666.1
Nocardioides sp. LS1
#;WecB-associating
MCB5881527.1
Lachnospiraceae bacterium EP-SM-12S-S03
WP_155521598.1
HIV50105.1
Candidatus Faecalibacterium intestinipullorum
NDI28477.1
WP_224121258.1
Nocardioides mangrovi
MBP3462129.1
Tyzzerella sp.
HBA69385.1
Lachnospiraceae bacterium
MBQ6153981.1
Ruminococcus sp.
HIS04116.1
Candidatus Pullichristensenella avicola
MBR6771520.1
Lachnospiraceae bacterium
HBE15711.1
Oscillospiraceae bacterium
MBQ7099881.1
Oscillospiraceae bacterium
MBR2422160.1
Oscillospiraceae bacterium
WP_002584018.1
Enterocloster clostridioformis
MBQ2986565.1
Tyzzerella sp.
HIW50301.1
Candidatus Blautia intestinavium
MBQ3417943.1
Ruminococcus sp.
MBS5215230.1
Clostridiales bacterium
MBR5293186.1
Oscillospiraceae bacterium
WP_142729871.1
Paraclostridium bifermentans
MBQ8830089.1
Oscillospiraceae bacterium
WP_002574446.1
Enterocloster bolteae
MBQ2986566.1
Tyzzerella sp.
MBR6858691.1
Bacteroidales bacterium
MBQ8057958.1
Ruminococcus sp.
WP_087156508.1
Lachnoclostridium sp. An181
MBQ9512831.1
Lachnospiraceae bacterium
MBQ6152941.1
Ruminococcus sp.
MBQ3417909.1
Ruminococcus sp.
MBQ7134361.1
Ruminococcus sp.
MBR0425380.1
Clostridia bacterium
MCD7844641.1
Oscillospiraceae bacterium
MCD7957300.1
Lachnospiraceae bacterium
###;pgpA: phosphatidylglycerophosphatase
MBV8653518.1
Alphaproteobacteria bacterium
SMC23550.1
Desulfacinum hydrothermale DSM 13146
MBP8645753.1
Syntrophobacteraceae bacterium
WP_170161757.1
Desulfosoma caldarium
#;GalU/murU/RmlA sugar modifying associations. RmlB is the "GalE/RmlB/Gmd-like_lyase/epimerase" in
these systems and is considerably more widespread...found across many of the groupings above
MBP5617096.1
Elusimicrobiaceae bacterium
MBQ2642680.1
Eubacterium sp.
QTE68789.1
Clostridiales bacterium
WP_144101769.1
Dorea longicatena
WP_215634868.1
Clostridium baratii
MBM4138957.1
Nitrospira sp.
ANU75233.1
Blautia pseudococcoides
MBT9688487.1
Fusicatenibacter saccharivorans
POP36074.1
Blautia producta
MBQ8201306.1
Clostridia bacterium
MBR5246878.1
Clostridia bacterium
MBQ6432104.1
Oscillospiraceae bacterium
MBQ7964961.1
Ruminococcus sp.
MBQ3427881.1
Clostridia bacterium
MBQ3427882.1
Clostridia bacterium
MBQ6381819.1
Clostridia bacterium
MBR5033725.1
Bacteroidales bacterium
MBR5033730.1
Bacteroidales bacterium
MBR1533641.1
Ruminococcus sp.
MBR4538307.1
Clostridia bacterium
MBR4500332.1
Clostridia bacterium
MBR4538314.1
Clostridia bacterium
MBQ0097579.1
Candidatus Ruminococcus equi
WP_002305628.1
Enterococcus faecium
BCN32266.1
Anaeromicropila herbilytica
PWT25990.1
Butyrivibrio fibrisolvens
KKW28821.1
Candidatus Uhrbacteria bacterium GW2011_GWD2_52_7
MBR5371120.1
Oscillospiraceae bacterium
WP_028529961.1
Ruminococcus gauvreauii
MBQ6927335.1
Oscillospiraceae bacterium
MBE5875876.1
Lachnospiraceae bacterium
MBE5875871.1
Lachnospiraceae bacterium
#;also includes rmlC & rmlD
WP_151668996.1
Enterococcus lactis
KJU73448.1
Pediococcus damnosus LMG 28219
OUQ42278.1
Drancourtella sp. An12
WP_141306347.1
Pediococcus damnosus
MBR1532699.1
Eubacterium sp.
WP_184535141.1
Micromonospora polyrhachis
MBQ3069433.1
Clostridia bacterium
OQY12263.1
Desulfobacteraceae bacterium 4572_187
#;comparable to the above with generally fewer components

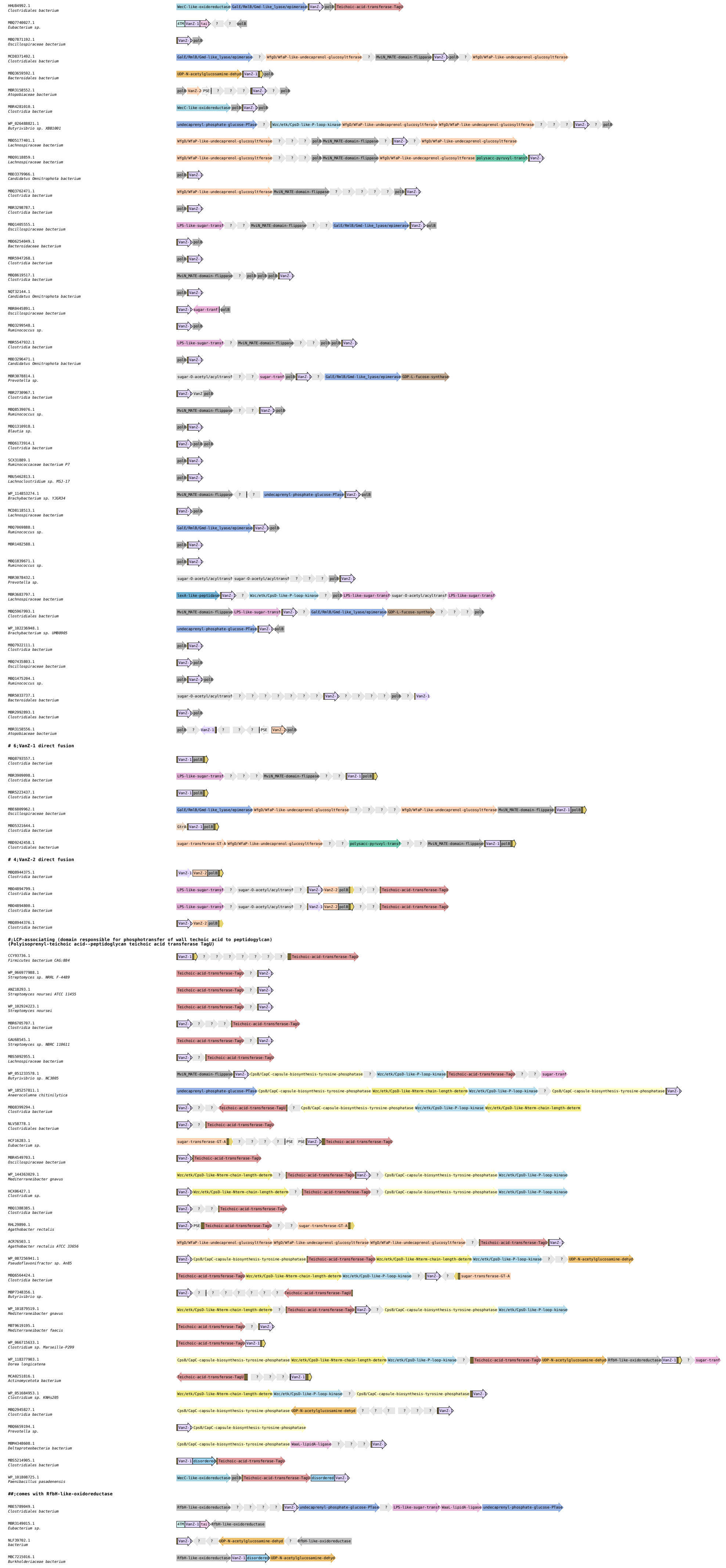
HHU84992.1
Clostridiales bacterium
MBQ7740027.1
Eubacterium sp.
MBQ7871192.1
Oscillospiraceae bacterium
MCD8371492.1
Clostridiales bacterium
MBQ3659592.1
Bacteroidales bacterium
MBR3158552.1
Atopobiaceae bacterium
MBR4281010.1
Clostridia bacterium
WP_026488821.1
Butyrivibrio sp. XBB1001
MBO5177401.1
Lachnospiraceae bacterium
MBQ9118859.1
Lachnospiraceae bacterium
MBD3379966.1
Candidatus Omnitrophota bacterium
MBQ3762471.1
Clostridia bacterium
MBR3298787.1
Clostridia bacterium
MBQ1405555.1
Oscillospiraceae bacterium
MBO6254049.1
Bacteroidaceae bacterium
MBR5947268.1
Clostridia bacterium
MBQ8619517.1
Clostridia bacterium
NQT32144.1
Candidatus Omnitrophota bacterium
MBR0445891.1
Oscillospiraceae bacterium
MBQ3299548.1
Ruminococcus sp.
MBR5547932.1
Clostridia bacterium
MBD3296471.1
Candidatus Omnitrophota bacterium
MBR3078814.1
Prevotella sp.
MBR2730967.1
Clostridia bacterium
MBQ8539076.1
Ruminococcus sp.
MBQ1310918.1
Blautia sp.
MBQ6173914.1
Clostridia bacterium
SCX31889.1
Ruminococcaceae bacterium P7
MBU5462813.1
Lachnoclostridium sp. MSJ-17
WP_114853274.1
Brachybacterium sp. YJGR34
MCD8118513.1
Lachnospiraceae bacterium
MBQ7069888.1
Ruminococcus sp.
MBR1482588.1
MBQ1839671.1
Ruminococcus sp.
MBR3078432.1
Prevotella sp.
MBR3683797.1
Lachnospiraceae bacterium
MBQ5967993.1
Clostridiales bacterium
WP_102236948.1
Brachybacterium sp. UMB0905
MBQ7922111.1
Clostridia bacterium
MBQ7435803.1
Oscillospiraceae bacterium
MBQ1475204.1
Ruminococcus sp.
MBR5033737.1
Bacteroidales bacterium
MBR2992893.1
Clostridiales bacterium
MBR3158556.1
Atopobiaceae bacterium
6;VanZ-1 direct fusion
MBQ8793557.1
Clostridia bacterium
MBR3909098.1
Clostridia bacterium
MBR5223437.1
Clostridia bacterium
MBE6809962.1
Oscillospiraceae bacterium
MBO5321644.1
Clostridia bacterium
MBD9242458.1
Clostridiales bacterium
4;VanZ-2 direct fusion
MBQ8944375.1
Clostridia bacterium
MBO4894799.1
Clostridia bacterium
MBO4894800.1
Clostridia bacterium
MBQ8944376.1
Clostridia bacterium
#;LCP-associating (domain responsible for phosphotransfer of wall techoic acid to peptidogylcan)
(Polyisoprenyl-teichoic acid--peptidoglycan teichoic acid transferase TagU)
CCY93736.1
Firmicutes bacterium CAG:884
WP_066977988.1
Streptomyces sp. NRRL F-4489
ANZ18293.1
Streptomyces noursei ATCC 11455
WP_102924223.1
Streptomyces noursei
MBR6705707.1
Clostridia bacterium
GAU68545.1
Streptomyces sp. NBRC 110611
MBS5092955.1
Lachnospiraceae bacterium
WP_051233578.1
Butyrivibrio sp. NC3005
WP_185257811.1
Anaerocolumna chitinilytica
MBQ8399294.1
Clostridia bacterium
NLV58778.1
Clostridiales bacterium
HCF16283.1
Eubacterium sp.
MBR4549703.1
Oscillospiraceae bacterium
WP_144363029.1
Mediterraneibacter gnavus
HCX06427.1
Clostridium sp.
MBQ1388385.1
Clostridia bacterium
RHL29890.1
Agathobacter rectalis
ACR76503.1
Agathobacter rectalis ATCC 33656
WP_087256941.1
Pseudoflavonifractor sp. An85
MBQ6564424.1
Clostridia bacterium
MBP7348356.1
Butyrivibrio sp.
WP_101879519.1
Mediterraneibacter gnavus
MBT9619195.1
Mediterraneibacter faecis
WP_066715633.1
Clostridium sp. Marseille-P299
WP_118377903.1
Dorea longicatena
MCA0251816.1
Actinomycetota bacterium
WP_051684953.1
Clostridium sp. KNHs205
MBQ2945827.1
Clostridia bacterium
MBQ6659194.1
Prevotella sp.
MBM4348608.1
Deltaproteobacteria bacterium
MBS5214905.1
Clostridiales bacterium
WP_101808725.1
Paenibacillus pasadenensis
##;comes with RfbH-like-oxidoreductase
MBE5789049.1
Clostridiales bacterium
MBR3149015.1
Eubacterium sp.
NLF39702.1
bacterium
MBC7215016.1
Burkholderiaceae bacterium

MBO2496208.1
VanZ-1 ? PSE RfbH-like-oxidoreductase

ERL06323.1
Olsenella profusa F0195
sugar-tranf LPS-like-sugar-transf WfgD/WfaP-like-undecaprenol-glucosyltferase ? ? ? VanZ-1 MviN_MATE-domain-flippase sugar-transferase-GT-A RfbH-like-oxidoreductase

KXS42829.1
Methanolobus sp. T82-4
GalE/RmlB/Gmd-like_lyase/epimerase VanZ-1 WecC-like-oxidoreductase ? RfbH-like-oxidoreductase sugar-O-acetyl/acyltransf

AFV22920.1
Methanolobus psychrophilus R15
GalE/RmlB/Gmd-like_lyase/epimerase VanZ-1 WecC-like-oxidoreductase ? RfbH-like-oxidoreductase sugar-O-acetyl/acyltransf

MBX3441592.1
Planctomyces sp.
GalE/RmlB/Gmd-like_lyase/epimerase RfbH-like-oxidoreductase sugar-tranf VanZ-1 ? VanZ-assorted-associations

WP_074761781.1
Xylanibacter ruminicola
sugar-transferase-GT-A ? sugar-transferase-GT-A ? MviN_MATE-domain-flippase ? VanZ-1 LPS-like-sugar-transf ? sugar-tranf RfbH-like-oxidoreductase

MBR5678261.1
Paludibacteraceae bacterium
RfbH-like-oxidoreductase VanZ-1

MBS6180982.1
Erysipelotrichaceae bacterium
RfbH-like-oxidoreductase ? ? ? ? VanZ-1

MBQ8541059.1
Clostridia bacterium
polysacc-pyruvyl-transf WfgD/WfaP-like-undecaprenol-glucosyltferase ? polysacc-pyruvyl-transf ? VanZ-1 ? RfbH-like-oxidoreductase ? ? ? ? polysacc-pyruvyl-transf

MBR6027686.1
Clostridia bacterium
UDP-N-acetylglucosamine-dehyd VanZ-1 ? RfbH-like-oxidoreductase sugar-tranf ? ? ? WfgD/WfaP-like-undecaprenol-glucosyltferase

MBQ6383732.1
Clostridia bacterium
Wzc/etk/CpsD-like-P-loop-kinase ? LPS-like-sugar-transf ? WfgD/WfaP-like-undecaprenol-glucosyltferase sugar-transferase-GT-A MviN_MATE-domain-flippase VanZ-1 RfbH-like-oxidoreductase

MBI4125426.1
Deltaproteobacteria bacterium
RfbH-like-oxidoreductase GalE/RmlB/Gmd-like_lyase/epimerase ? ? VanZ-1

MBR0410331.1
UDP-N-acetylglucosamine-dehyd VanZ-1 ? ? ? RfbH-like-oxidoreductase VanZ-2 sugar-tranf

MBR0410336.1
Eubacterium sp.
UDP-N-acetylglucosamine-dehyd VanZ-1 ? ? ? RfbH-like-oxidoreductase VanZ-2 sugar-tranf ? ? LPS-like-sugar-transf ? ? ? LPS-like-sugar-transf

MBX3441590.1
Planctomyces sp.
GalE/RmlB/Gmd-like_lyase/epimerase RfbH-like-oxidoreductase sugar-tranf VanZ-1 ? VanZ-assorted-associations

MBR2716068.1
Ruminococcus sp.
RfbH-like-oxidoreductase ? WaaL-lipidA-ligase VanZ-1 polB GalE/RmlB/Gmd-like_lyase/epimerase WecC-like-oxidoreductase

MBE6949663.1
Oscillospiraceae bacterium
sugar-O-acetyl/acyltransf RfbH-like-oxidoreductase ? WaaL-lipidA-ligase VanZ-1 WfgD/WfaP-like-undecaprenol-glucosyltferase polB GalE/RmlB/Gmd-like_lyase/epimerase WecC-like-oxidoreductase

MBQ2971220.1
Ruminococcus sp.
LPS-like-sugar-transf WecC-like-oxidoreductase ? ? sugar-O-acetyl/acyltransf RfbH-like-oxidoreductase ? WaaL-lipidA-ligase VanZ-1 polB GalE/RmlB/Gmd-like_lyase/epimerase WecC-like-oxidoreductase sugar-O-acetyl/acyltransf ? WfgD/WfaP-like-undecaprenol-glucosyltferase WfgD/WfaP-like-undecaprenol-glucosyltferase

MBO4843974.1
Bacteroidales bacterium
LPS-like-sugar-transf ? ? sugar-tranf ? ? RfbH-like-oxidoreductase ? VanZ-1 polB

MBQ4128740.1
Ruminococcus sp.
VanZ-1 polB ? RfbH-like-oxidoreductase sugar-tranf ? LPS-like-sugar-transf WfgD/WfaP-like-undecaprenol-glucosyltferase

MBQ6625795.1
Ruminococcus sp.
RfbH-like-oxidoreductase ? VanZ-1 polB

##;GtrA-associated, direct fusion

MBQ3861965.1
Clostridia bacterium
VanZ-1 GtrA-TM

MBQ2547199.1
Clostridia bacterium
VanZ-1 GtrA-TM

MBE6726418.1
Oscillospiraceae bacterium
VanZ-1 GtrA-TM

MBR4205840.1
Clostridia bacterium
VanZ-1 GtrA-TM

HIW74926.1
Bacillota bacterium
Amidohydro-TIM ? VanZ-1 GtrA-TM

HIZ20919.1
Bacillota bacterium
VanZ-1 GtrA-TM

HIV19646.1
Candidatus Merdivicinus intestinigallinarum
VanZ-1 GtrA-TM

#;operonic

HIQ58264.1
Candidatus Merdivicinus intestinavium
VanZ-1 GtrA ? ? ? ? ? ? GNAT-N-acetyltransf

WP_181556000.1
Thermaerobacillus caldiproteolyticus
sugar-transferase-GT-A GtrA RmlA/rfbA/rffH/GalU-glucose-1-phosphate-base-transferase VanZ-1

MBQ9229407.1
Eubacterium sp.
4TM VanZ-1 tail GtrA ? polB ? RfbH-like-oxidoreductase GalE/RmlB/Gmd-like_lyase/epimerase RmlA/rfbA/rffH/GalU-glucose-1-phosphate-base-transferase

MBP5489580.1
Lachnospiraceae bacterium
GtrA ? ? ? VanZ-1

WP_195199162.1
Faecalispora jeddahensis
GtrA ? ? VanZ-1

WP_050699177.1
Anaeromassilibacillus senegalensis
GtrA VanZ-1

HIT07766.1
Candidatus Merdivicinus faecavium
VanZ-1 GtrA

MBW7909261.1
Kiritimatiellia bacterium
GtrA ? ? ? VanZ-1 ?? VanZ-1 ??

#;four-gene-island (see VanZ-2)

MBX7260264.1
Chitinophagales bacterium
sprA-typeIX-ss GCV_H VanZ-1 Acyl_transf-TM

HGG45444.1
GCV_H ? ? VanZ-1

SEP94288.1
Virgibacillus subterraneus
GCV_H ? VanZ-1

MBQ2376433.1
Bacteroidales bacterium
GCV_H VanZ-1

QQR98518.1
Sphingobacteriales bacterium
GCV_H VanZ-1 Acyl_transf-TM

#;embedded in polysaccharide synthesis/transport/processing neighborhoods

MBQ6289849.1
Clostridia bacterium
LPS-like-sugar-transf LPS-like-sugar-transf WaaL-lipidA-ligase ? ? RHS VanZ-1 polB sugar-O-acetyl/acyltransf WfgD/WfaP-like-undecaprenol-glucosyltferase ? MurJ/WzxE-flippase ? UDP-N-acetylglucosamine-dehyd

MBQ9872431.1
Eubacterium sp.
UDP-N-acetylglucosamine-dehyd RHS VanZ-1 ? PSE WecB/rrfE/mnaA-epimerase

MBR4607578.1
Lachnospiraceae bacterium
RmlA/rfbA/rffH/GalU-glucose-1-phosphate-base-transferase ? PSE PSE RHS VanZ-1

MBR0162528.1
Oscillospiraceae bacterium
MviN_MATE-domain-flippase RmlA/rfbA/rffH/GalU-glucose-1-phosphate-base-transferase GalE/RmlB/Gmd-like_lyase/epimerase RHS VanZ-1

MBO5570449.1
Clostridia bacterium
GalE/RmlB/Gmd-like_lyase/epimerase rmlD/rfbD-like-reductase rmlC/rfbC-like-epimerase RmlA/rfbA/rffH/GalU-glucose-1-phosphate-base-transferase RHS VanZ-1 RmlA/rfbA/rffH/GalU-glucose-1-phosphate-base-transferase

MBR6165045.1
Clostridia bacterium
sugar-O-acetyl/acyltransf ? MviN_MATE-domain-flippase ? polB RHS VanZ-1 WecC-like-oxidoreductase

QUC68206.1
Aristaeella hokkaidonensis
polB RHS VanZ-1

MBP3198579.1
Butyrivibrio sp.
GalE/RmlB/Gmd-like_lyase/epimerase ? ? ? MviN_MATE-domain-flippase RHS VanZ-1 Teichoic-acid-transferase-TagO

QTE67405.1
Clostridiales bacterium
sugar-transferase-GT-A MviN_MATE-domain-flippase ? ? ? RfbH-like-oxidoreductase polB RHS VanZ-1 GalE/RmlB/Gmd-like_lyase/epimerase WecC-like-oxidoreductase

WP_118253968.1
Blautia sp. AF19-10LB
WecC-like-oxidoreductase RHS VanZ-1

############################other associations of note; #;found adjacent to polymorphic toxin-like architectures of VanZ-1

MBR3241687.1
Parasporobacterium sp.
RHS VanZ-1 VanZ-2

QTE75004.1
Clostridiales bacterium FE2010
sugar-O-acetyl/acyltransf RHS VanZ-1

WP_204870246.1
Pseudoflavonifractor capillosus
WfgD/WfaP-like-undecaprenol-glucosyltferase ? ? LPS-like-sugar-transf ? ? ? MviN_MATE-domain-flippase RHS VanZ-1

MBR4606786.1
Lachnospiraceae bacterium
RHS VanZ-1 GalE/RmlB/Gmd-like_lyase/epimerase

MBO4456371.1
Butyrivibrio sp.
MviN_MATE-domain-flippase ? ? RHS VanZ-1 ? ? WfgD/WfaP-like-undecaprenol-glucosyltferase

#;standalone

WP_118659076.1
Faecalibacterium sp. OM04-11BH
RHS VanZ-1 helical

MBR4455128.1
Solobacterium sp.
RHS VanZ-1

WP_051527076.1
[Eubacterium] cellulosolvens LD2006
RHS VanZ-1

MBQ9664126.1
Oscillospiraceae bacterium
RHS VanZ-1

MBQ3477092.1
Clostridia bacterium
RHS VanZ-1

MBR2824097.1
Clostridia bacterium
RHS VanZ-1

MBO6162908.1
Eubacterium sp.
RHS VanZ-1

MBS4903996.1
Subdoligranulum variabile
RHS VanZ-1

MBN2950792.1
Holdemanella sp.
RHS VanZ-1

MBQ8095334.1
Clostridia bacterium
RHS VanZ-1

MBR3243711.1
Parasporobacterium sp.
RHS VanZ-1

WP_074648334.1
[Clostridium] aminophilum
RHS VanZ-1

MBR3241686.1
Parasporobacterium sp.
RHS VanZ-1 VanZ-2

WP_197026405.1
Oribacterium sp. P6A1
RHS VanZ-1

QUA54033.1
Aristaeella lactis
RHS VanZ-1

WP_204550198.1
RHS VanZ-1

WP_162607067.1
Thomasclavelia spiroformis
RHS VanZ-1

####;VanY. While VanY is only fused to VanZ-1, there are genuine cases where VanY associates with a VanZ-2. Association with other vancomycin-resistance genes

SHN13205.1
Cryptosporangium aurantiacum
VanH-like VanA/VanG VanX ? ? 4TM VanZ-1

MBO0841867.1
Nocardioides sp.
VanH-like VanA/VanG VanX ? VanZ-1

REK38735.1
Actinomycetota bacterium
VanZ-1 ? VanA/VanG MurF-UDP-N-acetylmuramoyl-tripeptide--D-alanyl-D-alanine-ligase

NBI64690.1
Clostridiales bacterium
MurC-UDP-N-acetylmuramate--alanine-ligase ? ? ? ? ? VanA/VanG VanY VanZ-1

OLA15812.1
Coprococcus sp. CAG:131-related_45_246
VanZ-1 VanA/VanG VanY VanT

MBB5978386.1
Kribbella solani
VanY ? VanZ-1 VanH-like VanA/VanG VanX

MBQ6387895.1
Ruminococcus sp.
VanZ-1 ? VanA/VanG VanY VanT

HIZ08381.1
Candidatus Eubacterium avistercoris
VanA/VanG VanY PSE VanZ-1

SCH46280.1
uncultured Clostridium sp.
VanA/VanG VanY VanT-N VanZ-1

NBK07538.1
Enterococcus asini
VanZ-1 VanY VanA/VanG

#;VanZ & VanY two-gene associations

MBE6881451.1
Oscillospiraceae bacterium
VanZ-1 ? GalE/RmlB/Gmd-like_lyase/epimerase VanY

WP_129230075.1
Agromyces fucosus
VanY ? ? VanZ-1

BBH18462.1
Nocardioides baekrokdamisoli
VanY VanZ-1

HIS17443.1
Candidatus Coprovivens excrementavium
VanZ-1 ? ? ? ? ? ? VanY

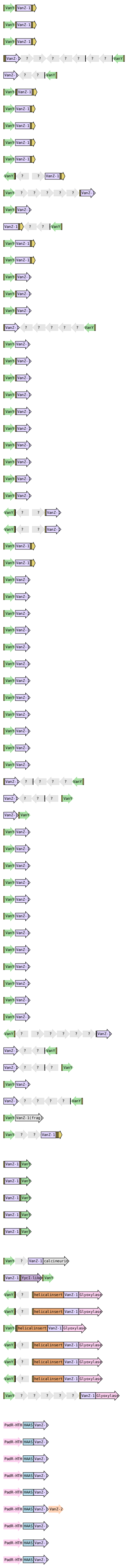

WP_012878195.1
Xylanimonas cellulosilytica DSM 15894

WP_100351002.1
Luteimicrobium subarcticum

RZS66405.1
Agromyces ramosus

PVW03558.1
Microbacterium sp. Gd 4-13

PPF44958.1
Pseudoclavibacter sp. AY1F1

MBF0987235.1
Clostridiales bacterium

WP_051136515.1
Luteimicrobium xylanilyticum

WP_013771048.1
Cellulomonas fimi

SDS99873.1
Agromyces flavus

NYI53081.1

WP_162231059.1
Leucobacter musarum subsp. musarum

BBF42493.1
Lachnospiraceae bacterium KM106-2

MBQ9939351.1
Oscillospiraceae bacterium

WP_223043623.1
Leucobacter tenebrionis

HAQ1772353.1
Enterococcus faecium

WP_013860941.1
Microlunatus phosphovorus NM-1

MBM7630212.1

WP_120109814.1
Paenibacillus pinisoli

WP_171241598.1
Knoellia koreensis

MBR6955203.1
Clostridia bacterium

SDT34573.1
Paenibacillaceae bacterium GAS479

WP_225282882.1
Nocardioides nematodiphilus

WP_120059603.1
Nocardioides cavernaquae

MCC2683648.1
Paenibacillaceae bacterium

WP_051620544.1
Paenibacillus sp. UNC451MF

WP_140738586.1
Pedococcus bigeumensis

WP_145052322.1
Paenibacillus xylanexedens

CAH1223019.1
Paenibacillus sp. JJ-223

OAS16492.1
Paenibacillus oryzisoli

KOP68695.1
Bacillus sp. FJAT-18019

WP_155229838.1
Turicibacter sanguinis

WP_141164463.1
Schumannella soli

NQX28669.1
Microbacteriaceae bacterium VKM Ac-2854

QHW34867.1
Paenibacillus rhizovicinus

WP_185122411.1
Cohnella thailandensis

WP_116190341.1
Paenibacillus taihuensis

OAB40611.1
Paenibacillus macquariensis subsp. defensor

SLL32712.1
Mycobacteroides abscessus subsp. abscessus

CQR55820.1
Paenibacillus riograndensis SBR5

QGR00339.1
Paenibacillus psychroresistens

WP_076140625.1
Paenibacillus sp. FSL F4-0097

WP_077565686.1
Paenibacillus ihbetae

WP_144870112.1
Paenibacillus lactis

GIO43079.1
Paenibacillus apis

WP_059041455.1
Paenibacillus rubinfantis

WP_023958861.1
Paenibacillus sp. JCM 10914

TDS75821.1
Amnibacterium kyonggiense

OZD55733.1
Rhodococcus sp. 06-1477-1B

WP_060702374.1
Glutamicibacter halophytocola

WP_205004881.1
Scopulibacillus daqui

WP_052087918.1
Paenibacillus wynnii

WP_068649767.1
Paenibacillus antarcticus

WP_144529519.1
Peribacillus simplex

MBP2014364.1

HIW35106.1
Candidatus Paenibacillus intestinavium

WP_213514574.1
Paenibacillus montaniterrae

WP_076112172.1
Paenibacillus sp. FSL H3-0319

WP_060626140.1
Paenibacillus etheri

WP_206097805.1
Paenibacillus apiarius

WP_151734863.1
Paenibacillus tengchongensis

KKO55237.1
Paenibacillus sp. DMB20

MBP1834788.1

WP_162564376.1
Microbacterium ureisolvens

WP_058626864.1
Microbacterium testaceum

WP_153685386.1

WP_076682122.1
Microbacterium sp. RU33B

WP_116334817.1

MBN9105355.1
Propionibacteriaceae bacterium

#;direct fusions

WP_152727776.1
Goekera deserti

QXG76294.1
Modestobacter sp. L9-4

MBQ4068733.1
Lachnospiraceae bacterium

MBQ3584723.1
Lachnospiraceae bacterium

CCZ04694.1
Eubacterium sp. CAG:603

#;VanZ is fused to other enzymatic domains

MBQ0065529.1
Bacillota bacterium

MBQ6654018.1
Erysipelotrichaceae bacterium

MBE5790091.1
Clostridiales bacterium

MBQ6061722.1
Clostridia bacterium

MBR3357146.1
Solobacterium sp.

QTE72013.1
Clostridiales bacterium FE2011

QUA53093.1
Aristaeella lactis

MBR4576947.1
Clostridia bacterium

MBP0955642.1
Oscillospiraceae bacterium

####;HAAS fusion

CAG5091277.1
Thermobacillus xylanilyticus

TYO68598.1
Rossellomorea marisflavi

PFW75808.1
Priestia aryabhattai

WP_098833512.1

BAR74173.1
Bacillus anthracis

WP_100012421.1
Lentibacillus sediminis

NWO13822.1
Virgibacillus sp.

WP_232231331.1
Virgibacillus pantothenticus

WP_115748993.1
Oceanobacillus chungangensis

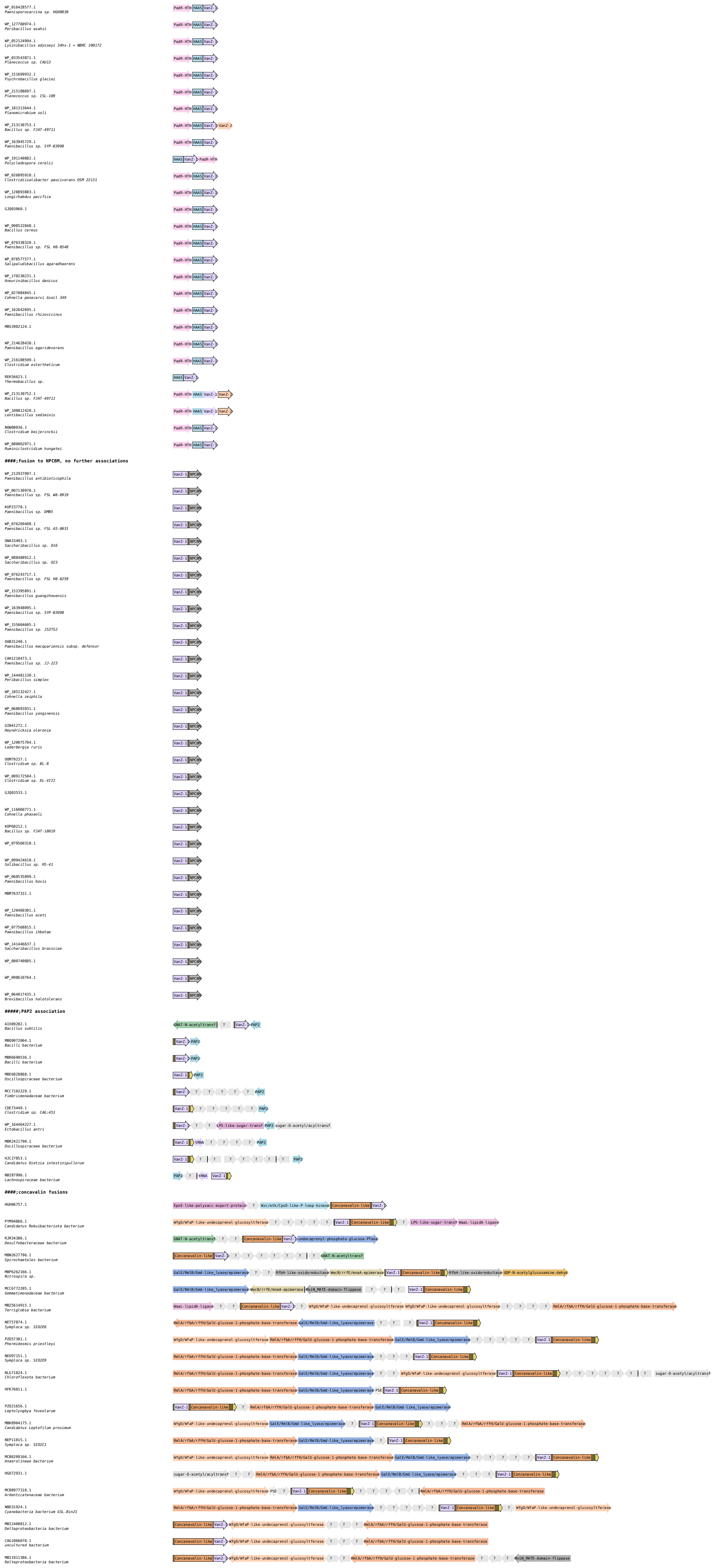

WP_016428577.1 Paenisporosarcina sp. HGH0030
PadR-HTH HAAS VanZ-1
WP_127760974.1 Peribacillus asahii
PadR-HTH HAAS VanZ-1
WP_052124994.1 Lysinibacillus odysseyi 34hs-1 = NBRC 100172
PadR-HTH HAAS VanZ-1
WP_033543871.1 Planococcus sp. CAU13
PadR-HTH HAAS VanZ-1
WP_151699932.1 Psychrobacillus glaciei
PadR-HTH HAAS VanZ-1
WP_215108697.1 Planococcus sp. ISL-109
PadR-HTH HAAS VanZ-1
WP_181313644.1 Planomicrobium soli
PadR-HTH HAAS VanZ-1
WP_213130753.1 Bacillus sp. FJAT-49711
PadR-HTH HAAS VanZ-1 VanZ-2
WP_163945729.1 Paenibacillus sp. SYP-B3998
PadR-HTH HAAS VanZ-1
WP_191140882.1 Polycladospora coralii
HAAS VanZ-1 PadR-HTH
WP_026895918.1 Clostridiisalibacter paucivorans DSM 22131
PadR-HTH HAAS VanZ-1
WP_128893883.1 Longirhabdus pacifica
PadR-HTH HAAS VanZ-1
GJQ03060.1
PadR-HTH HAAS VanZ-1
WP_098522848.1 Bacillus cereus
PadR-HTH HAAS VanZ-1
WP_076338320.1 Paenibacillus sp. FSL H8-0548
PadR-HTH HAAS VanZ-1
WP_078577577.1 Salipaludibacillus agaradhaerens
PadR-HTH HAAS VanZ-1
WP_170230231.1 Aneurinibacillus danicus
PadR-HTH HAAS VanZ-1
WP_027084845.1 Cohnella panacarvi Gsoil 349
PadR-HTH HAAS VanZ-1
WP_162642695.1 Paenibacillus rhizovicinus
PadR-HTH HAAS VanZ-1
MBS3902124.1
PadR-HTH HAAS VanZ-1
WP_214628430.1 Paenibacillus agaridevorans
PadR-HTH HAAS VanZ-1
WP_216108599.1 Clostridium estertheticum
PadR-HTH HAAS VanZ-1
REK56623.1 Thermobacillus sp.
HAAS VanZ-1
WP_213130752.1 Bacillus sp. FJAT-49711
PadR-HTH HAAS VanZ-1 VanZ-2
WP_100012420.1 Lentibacillus sediminis
PadR-HTH HAAS VanZ-1 VanZ-2
NOW08036.1 Clostridium beijerinckii
PadR-HTH HAAS VanZ-1
WP_080062971.1 Ruminiclostridium hungatei
PadR-HTH HAAS VanZ-1
####;fusion to NPCBM, no further associations
WP_212937997.1 Paenibacillus antibioticophila
VanZ-1 NPCBM
WP_007130970.1 Paenibacillus sp. FSL W8-0919
VanZ-1 NPCBM
KUP23778.1 Paenibacillus sp. DMB5
VanZ-1 NPCBM
WP_076269408.1 Paenibacillus sp. FSL A5-0031
VanZ-1 NPCBM
OWA33403.1 Saccharibacillus sp. O16
VanZ-1 NPCBM
WP_088488912.1 Saccharibacillus sp. O23
VanZ-1 NPCBM
WP_076243717.1 Paenibacillus sp. FSL H8-0259
VanZ-1 NPCBM
WP_152395891.1 Paenibacillus guangzhouensis
VanZ-1 NPCBM
WP_163948095.1 Paenibacillus sp. SYP-B3998
VanZ-1 NPCBM
WP_155604605.1 Paenibacillus sp. J53TS2
VanZ-1 NPCBM
OAB31240.1 Paenibacillus macquariensis subsp. defensor
VanZ-1 NPCBM
CAH1210473.1 Paenibacillus sp. JJ-223
VanZ-1 NPCBM
WP_144481130.1 Peribacillus simplex
VanZ-1 NPCBM
WP_185132427.1 Cohnella zeiphila
VanZ-1 NPCBM
WP_068693931.1 Paenibacillus yonginensis
VanZ-1 NPCBM
GIN41272.1 Heyndrickxia oleronia
VanZ-1 NPCBM
WP_120075704.1 Lederbergia ruris
VanZ-1 NPCBM
OOM79237.1 Clostridium sp. BL-8
VanZ-1 NPCBM
WP_009172504.1 Clostridium sp. DL-VIII
VanZ-1 NPCBM
GJQ03533.1
VanZ-1 NPCBM
WP_116060771.1 Cohnella phaseoli
VanZ-1 NPCBM
KOP68212.1 Bacillus sp. FJAT-18019
VanZ-1 NPCBM
WP_079560310.1
VanZ-1 NPCBM
WP_099424610.1 Solibacillus sp. R5-41
VanZ-1 NPCBM
WP_060535899.1 Paenibacillus bovis
VanZ-1 NPCBM
MBM7637331.1
VanZ-1 NPCBM
WP_120460301.1 Paenibacillus aceti
VanZ-1 NPCBM
WP_077568815.1 Paenibacillus ihbetae
VanZ-1 NPCBM
WP_141446637.1 Saccharibacillus brassicae
VanZ-1 NPCBM
WP_080740805.1
VanZ-1 NPCBM
WP_098610764.1
VanZ-1 NPCBM
WP_064017435.1 Brevibacillus halotolerans
VanZ-1 NPCBM
#####;PAP2 association
AIX09282.1 Bacillus subtilis
GNAT-N-acetyltransf ? VanZ-1 PAP2
MBQ9072004.1 Bacilli bacterium
VanZ-1 PAP2
MBR6690536.1 Bacilli bacterium
VanZ-1 PAP2
MBE6828868.1 Oscillospiraceae bacterium
VanZ-1 PAP2
MCC7102229.1 Fimbriimonadaceae bacterium
VanZ-1 ? ? ? ? ? PAP2
CDE73449.1 Clostridium sp. CAG:451
VanZ-1 ? ? ? ? ? PAP2
WP_164464227.1 Ectobacillus antri
VanZ-1 ? ? LPS-like-sugar-transf PAP2 sugar-O-acetyl/acyltransf
MBR2421790.1 Oscillospiraceae bacterium
VanZ-1 tRNA ? ? ? ? PAP2
HJC27853.1 Candidatus Dietzia intestinipullorum
VanZ-1 ? ? ? ? ? ? ? PAP2
NBI87996.1 Lachnospiraceae bacterium
PAP2 ? tRNA VanZ-1
####;concavalin fusions
HGH06757.1
EpsE-like-polysacc-export-protein ? Wzc/etk/CpsD-like-P-loop-kinase Concanavalin-like VanZ-1
PYM94866.1 Candidatus Rokuibacteriota bacterium
WfgD/WfaP-like-undecaprenol-glucosyltferase ? ? ? ? ? VanZ-1 Concanavalin-like ? LPS-like-sugar-transf WaaL-lipidA-ligase
RJR34386.1 Desulfobacteraceae bacterium
GNAT-N-acetyltransf ? ? Concanavalin-like VanZ-1 undecaprenyl-phosphate-glucose-PTase
MBN2627796.1 Spirochaetales bacterium
Concanavalin-like VanZ-1 ? ? ? ? ? ? ? GNAT-N-acetyltransf
MBP6262166.1 Nitrospira sp.
GalE/RmlB/Gmd-like_lyase/epimerase ? ? RfbH-like-oxidoreductase WecB/rrfE/mnaA-epimerase VanZ-1 Concanavalin-like RfbH-like-oxidoreductase UDP-N-acetylglucosamine-dehyd
MCC6772205.1 Gemmatimonadaceae bacterium
GalE/RmlB/Gmd-like_lyase/epimerase WecB/rrfE/mnaA-epimerase MviN_MATE-domain-flippase ? ? ? VanZ-1 Concanavalin-like
MBZ5614915.1 Terriglobia bacterium
WaaL-lipidA-ligase ? ? Concanavalin-like VanZ-1 ? WfgD/WfaP-like-undecaprenol-glucosyltferase WfgD/WfaP-like-undecaprenol-glucosyltferase ? ? ? ? RmlA/rfbA/rffH/GalU-glucose-1-phosphate-base-transferase
NET57874.1 Symploca sp. SIO2E6
RmlA/rfbA/rffH/GalU-glucose-1-phosphate-base-transferase GalE/RmlB/Gmd-like_lyase/epimerase ? ? VanZ-1 Concanavalin-like
PZO57301.1 Phormidesmis priestleyi
WfgD/WfaP-like-undecaprenol-glucosyltferase RmlA/rfbA/rffH/GalU-glucose-1-phosphate-base-transferase GalE/RmlB/Gmd-like_lyase/epimerase ? ? ? ? ? ? VanZ-1 Concanavalin-like
NEO97151.1 Symploca sp. SIO2E9
RmlA/rfbA/rffH/GalU-glucose-1-phosphate-base-transferase GalE/RmlB/Gmd-like_lyase/epimerase ? ? ? VanZ-1 Concanavalin-like
NLG71824.1 Chloroflexota bacterium
RmlA/rfbA/rffH/GalU-glucose-1-phosphate-base-transferase GalE/RmlB/Gmd-like_lyase/epimerase ? ? WfgD/WfaP-like-undecaprenol-glucosyltferase VanZ-1 Concanavalin-like ? ? ? ? ? ? ? sugar-O-acetyl/acyltransf
HFK76011.1
RmlA/rfbA/rffH/GalU-glucose-1-phosphate-base-transferase GalE/RmlB/Gmd-like_lyase/epimerase PSE VanZ-1 Concanavalin-like
PZO21656.1 Leptolyngbya foveolarum
VanZ-1 Concanavalin-like ? RmlA/rfbA/rffH/GalU-glucose-1-phosphate-base-transferase GalE/RmlB/Gmd-like_lyase/epimerase
MBK8904175.1 Candidatus Leptofilum proximum
WfgD/WfaP-like-undecaprenol-glucosyltferase GalE/RmlB/Gmd-like_lyase/epimerase ? VanZ-1 Concanavalin-like ? ? ? RmlA/rfbA/rffH/GalU-glucose-1-phosphate-base-transferase
NEP11015.1 Symploca sp. SIO2C1
RmlA/rfbA/rffH/GalU-glucose-1-phosphate-base-transferase GalE/RmlB/Gmd-like_lyase/epimerase ? VanZ-1 Concanavalin-like
MCB0209166.1 Anaerolineae bacterium
WfgD/WfaP-like-undecaprenol-glucosyltferase RmlA/rfbA/rffH/GalU-glucose-1-phosphate-base-transferase GalE/RmlB/Gmd-like_lyase/epimerase ? ? ? ? ? VanZ-1 Concanavalin-like
HGO72931.1
sugar-O-acetyl/acyltransf ? ? RmlA/rfbA/rffH/GalU-glucose-1-phosphate-base-transferase GalE/RmlB/Gmd-like_lyase/epimerase ? ? ? VanZ-1 Concanavalin-like
MCB8977318.1 Ardenticatenaceae bacterium
WfgD/WfaP-like-undecaprenol-glucosyltferase PSE ? VanZ-1 Concanavalin-like ? ? ? ? ? RmlA/rfbA/rffH/GalU-glucose-1-phosphate-base-transferase
NBD31924.1 Cyanobacteria bacterium GSL.Bin21
RmlA/rfbA/rffH/GalU-glucose-1-phosphate-base-transferase GalE/RmlB/Gmd-like_lyase/epimerase ? ? ? ? ? VanZ-1 Concanavalin-like ? WfgD/WfaP-like-undecaprenol-glucosyltferase
MBI2400812.1 Deltaproteobacteria bacterium
Concanavalin-like VanZ-1 WfgD/WfaP-like-undecaprenol-glucosyltferase ? ? ? RmlA/rfbA/rffH/GalU-glucose-1-phosphate-base-transferase
CAG1066070.1 uncultured bacterium
Concanavalin-like VanZ-1 WfgD/WfaP-like-undecaprenol-glucosyltferase ? ? ? RmlA/rfbA/rffH/GalU-glucose-1-phosphate-base-transferase
MBI1911386.1 Deltaproteobacteria bacterium
Concanavalin-like VanZ-1 WfgD/WfaP-like-undecaprenol-glucosyltferase ? ? RmlA/rfbA/rffH/GalU-glucose-1-phosphate-base-transferase ? ? ? MviN_MATE-domain-flippase

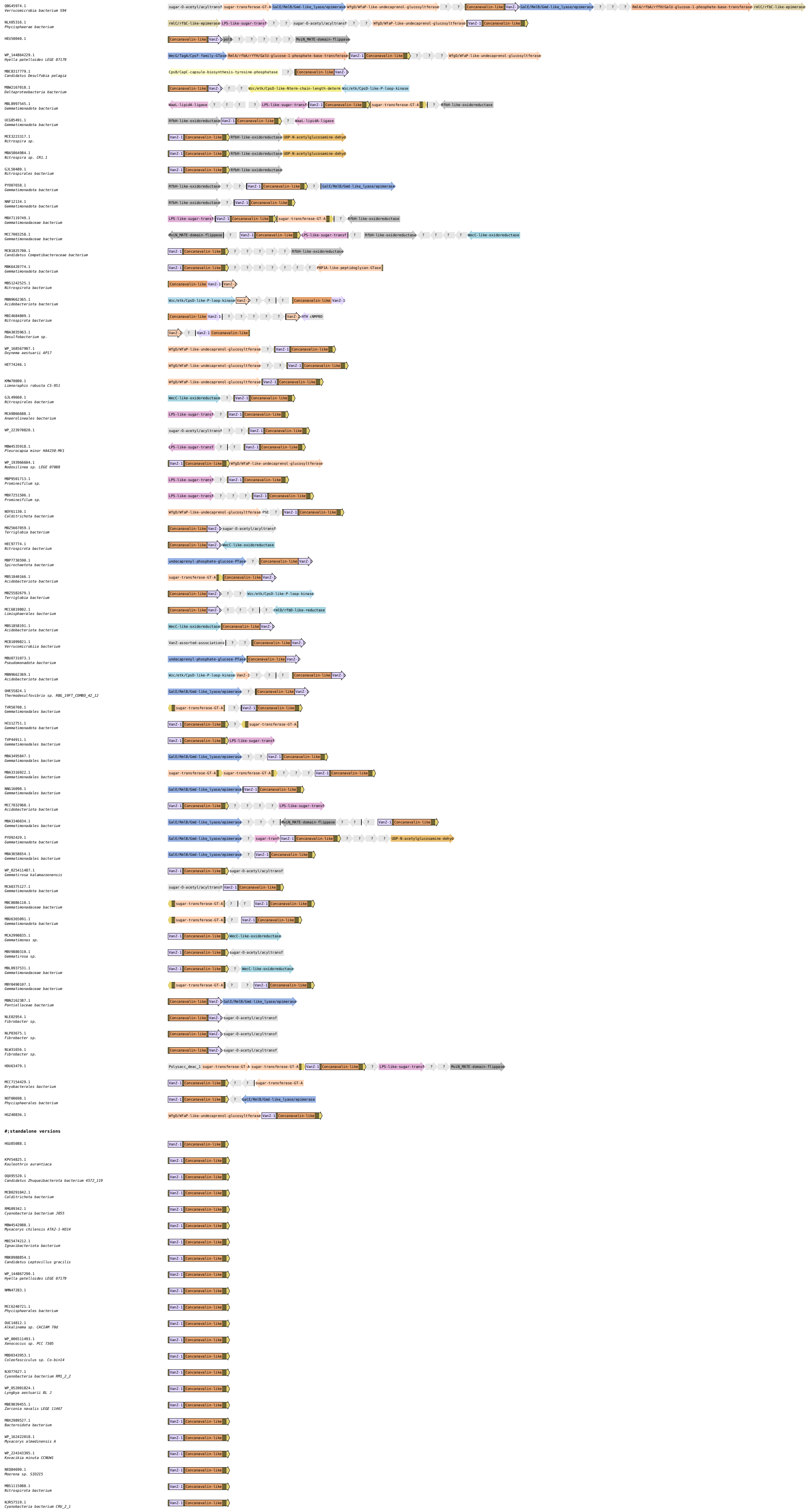

QBG45974.1
Verrucomicrobia bacterium S94
NLX05316.1
Phycisphaerae bacterium
HEU50040.1
WP_144864229.1
Hyella patelloides LEGE 07179
MBC8317779.1
Candidatus Desulfobia pelagia
MBW2167018.1
Deltaproteobacteria bacterium
MBL8997545.1
Gemmatimonadota bacterium
UCG85491.1
Gemmatimonadota bacterium
MCE3223317.1
Nitrospira sp.
MBA5864984.1
Nitrospira sp. CR1.1
GJL50480.1
Nitrospirales bacterium
PYO07658.1
Gemmatimonadota bacterium
NNF12134.1
Gemmatimonadota bacterium
MBX7119749.1
Gemmatimonadaceae bacterium
MCC7003258.1
Gemmatimonadaceae bacterium
MCB1825700.1
Candidatus Competibacteraceae bacterium
MBK6420774.1
Gemmatimonadota bacterium
MBS1242525.1
Nitrospirota bacterium
MBN9662365.1
Acidobacteriota bacterium
MBI4684809.1
Nitrospirota bacterium
MBA3035963.1
Desulfobacterium sp.
WP_168567907.1
Oxynema aestuarii AP17
HET74246.1
KMW70000.1
Limnoraphis robusta CS-951
GJL49660.1
Nitrospirales bacterium
MCA9866688.1
Anaerolineales bacterium
WP_223970820.1
MBW4535918.1
Pleurocapsa minor HA4230-MV1
WP_193966604.1
Nodosilinea sp. LEGE 07088
MBP9501713.1
Promineifilum sp.
MBX7251506.1
Promineifilum sp.
NOY61130.1
Calditrichota bacterium
MBZ5667059.1
Terriglobia bacterium
HEC97774.1
Nitrospirota bacterium
MBP7736590.1
Spirochaetota bacterium
MBS1840166.1
Acidobacteriota bacterium
MBZ5582679.1
Terriglobia bacterium
MCC6819802.1
Limisphaerales bacterium
MBS1858191.1
Acidobacteriota bacterium
MCB1099821.1
Verrucomicrobiia bacterium
MBU0731073.1
Pseudomonadota bacterium
MBN9662369.1
Acidobacteriota bacterium
OHE55824.1
Thermodesulfovibrio sp. RBG_19FT_COMBO_42_12
TVR50708.1
Gemmatimonadales bacterium
HCU12751.1
Gemmatimonadota bacterium
TVP44911.1
Gemmatimonadales bacterium
MBA3495847.1
Gemmatimonadales bacterium
MBA3316922.1
Gemmatimonadales bacterium
NNG16098.1
Gemmatimonadales bacterium
MCC7032960.1
Acidobacteriota bacterium
MBA3346034.1
Gemmatimonadales bacterium
PYO92429.1
Gemmatimonadota bacterium
MBA3658654.1
Gemmatimonadales bacterium
WP_025411487.1
Gemmatirosa kalamazoonensis
MCA0375127.1
Gemmatimonadota bacterium
MBC8086110.1
Gemmatimonadaceae bacterium
MBU6365091.1
Gemmatimonadota bacterium
MCA2990835.1
Gemmatimonas sp.
MBV9880310.1
Gemmatirosa sp.
MBL0937531.1
Gemmatimonadaceae bacterium
MBY0490107.1
Gemmatimonadaceae bacterium
MBN2162387.1
Pontiellaceae bacterium
NLE02954.1
Fibrobacter sp.
NLP03675.1
Fibrobacter sp.
NLW31656.1
Fibrobacter sp.
HDU63479.1
MCC7154429.1
Bryobacterales bacterium
NOT00698.1
Phycisphaerales bacterium
HGZ40836.1
#;standalone versions
HGU85088.1
KPV54825.1
Kouleothrix aurantiaca
OQX95520.1
Candidatus Zhuqueibacterota bacterium 4572_119
MCB0291042.1
Calditrichota bacterium
RMG09342.1
Cyanobacteria bacterium J055
MBW4542088.1
Myxacorys chilensis ATA2-1-KO14
MBI5474212.1
Ignavibacteriota bacterium
MBK8988854.1
Candidatus Leptovillus gracilis
WP_144867290.1
Hyella patelloides LEGE 07179
NMN47283.1
MCC6240721.1
Phycisphaerales bacterium
OUC14812.1
Alkalinema sp. CACIAM 70d
WP_006511493.1
Xenococcus sp. PCC 7305
MBD0343953.1
Coleofasciculus sp. Co-bin14
NJO77627.1
Cyanobacteria bacterium RM1_2_2
WP_052001824.1
Lyngbya aestuarii BL J
MBE9039455.1
Zarconia navalis LEGE 11467
MBX2989527.1
Bacteroidota bacterium
WP_162422018.1
Myxacorys almedinensis A
WP_224343395.1
Kovacikia minuta CCNUW1
NEQ84690.1
Moorena sp. SIO2I5
MBS1115088.1
Nitrospirota bacterium
NJR57519.1
Cyanobacteria bacterium CRU_2_1

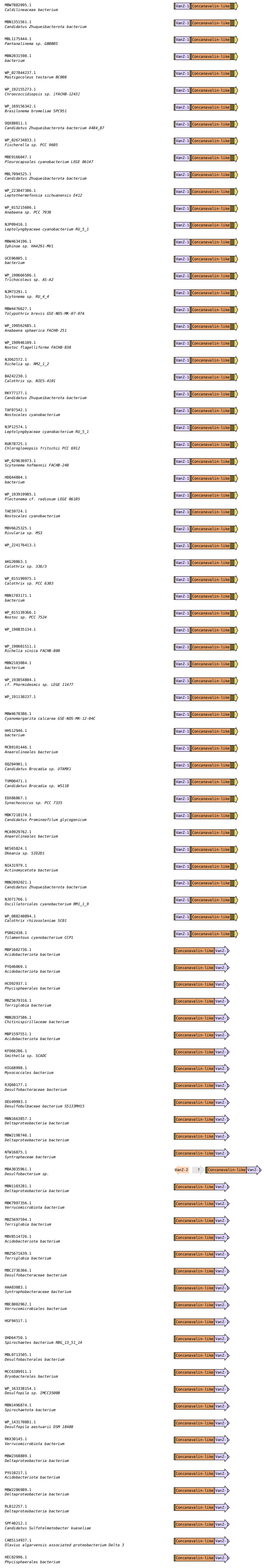

MBW7882095.1
Caldilineaceae bacterium
VanZ-1 Concanavalin-like
MBN1351561.1
Candidatus Zhuqueibacterota bacterium
VanZ-1 Concanavalin-like
MBL1175444.1
Pantanalinema sp. GBBB05
VanZ-1 Concanavalin-like
MBN2031598.1
bacterium
VanZ-1 Concanavalin-like
WP_027844237.1
Mastigocoleus testarum BC008
VanZ-1 Concanavalin-like
WP_192155273.1
Chroococcidiopsis sp. [FACHB-1243]
VanZ-1 Concanavalin-like
WP_169156342.1
Brasilonema bromeliae SPC951
VanZ-1 Concanavalin-like
OQX88811.1
Candidatus Zhuqueibacterota bacterium 4484_87
VanZ-1 Concanavalin-like
WP_026734833.1
Fischerella sp. PCC 9605
VanZ-1 Concanavalin-like
MBE9166047.1
Pleurocapsales cyanobacterium LEGE 06147
VanZ-1 Concanavalin-like
MBL7094525.1
Candidatus Zhuqueibacterota bacterium
VanZ-1 Concanavalin-like
WP_223047386.1
Leptothermofonsia sichuanensis E412
VanZ-1 Concanavalin-like
WP_015215606.1
Anabaena sp. PCC 7938
VanZ-1 Concanavalin-like
NJP09416.1
Leptolyngbyaceae cyanobacterium RU_5_1
VanZ-1 Concanavalin-like
MBW4634196.1
Iphinoe sp. HA4291-MV1
VanZ-1 Concanavalin-like
UCE06005.1
bacterium
VanZ-1 Concanavalin-like
WP_190666506.1
Trichocoleus sp. AS-A2
VanZ-1 Concanavalin-like
NJM73291.1
Scytonema sp. RU_4_4
VanZ-1 Concanavalin-like
MBW4476627.1
Tolypothrix brevis GSE-NOS-MK-07-07A
VanZ-1 Concanavalin-like
WP_190562685.1
Anabaena sphaerica FACHB-251
VanZ-1 Concanavalin-like
WP_190946109.1
Nostoc flagelliforme FACHB-838
VanZ-1 Concanavalin-like
NJO62572.1
Richelia sp. RM2_1_2
VanZ-1 Concanavalin-like
BAZ42230.1
Calothrix sp. NIES-4101
VanZ-1 Concanavalin-like
RKY77177.1
Candidatus Zhuqueibacterota bacterium
VanZ-1 Concanavalin-like
TAF07542.1
Nostocales cyanobacterium
VanZ-1 Concanavalin-like
NJP12574.1
Leptolyngbyaceae cyanobacterium RU_5_1
VanZ-1 Concanavalin-like
RUR78725.1
Chlorogloeopsis fritschii PCC 6912
VanZ-1 Concanavalin-like
WP_029636973.1
Scytonema hofmannii FACHB-248
VanZ-1 Concanavalin-like
HDQ44804.1
bacterium
VanZ-1 Concanavalin-like
WP_193919985.1
Plectonema cf. radiosum LEGE 06105
VanZ-1 Concanavalin-like
TAE59724.1
Nostocales cyanobacterium
VanZ-1 Concanavalin-like
MBV6625325.1
Rivularia sp. MS3
VanZ-1 Concanavalin-like
WP_224176413.1
VanZ-1 Concanavalin-like
AKG20863.1
Calothrix sp. 336/3
VanZ-1 Concanavalin-like
WP_015199975.1
Calothrix sp. PCC 6303
VanZ-1 Concanavalin-like
MBN1783171.1
bacterium
VanZ-1 Concanavalin-like
WP_015139366.1
Nostoc sp. PCC 7524
VanZ-1 Concanavalin-like
WP_190835134.1
VanZ-1 Concanavalin-like
WP_190601511.1
Richelia sinica FACHB-800
VanZ-1 Concanavalin-like
MBN2103084.1
bacterium
VanZ-1 Concanavalin-like
WP_193854804.1
cf. Phormidesmis sp. LEGE 11477
VanZ-1 Concanavalin-like
WP_191130237.1
VanZ-1 Concanavalin-like
MBW4670386.1
Cyanomargarita calcarea GSE-NOS-MK-12-04C
VanZ-1 Concanavalin-like
HHS12946.1
bacterium
VanZ-1 Concanavalin-like
MCB9101446.1
Anaerolineales bacterium
VanZ-1 Concanavalin-like
OQZ04901.1
Candidatus Brocadia sp. UTAMX1
VanZ-1 Concanavalin-like
TVM00471.1
Candidatus Brocadia sp. WS118
VanZ-1 Concanavalin-like
EDX86867.1
Synechococcus sp. PCC 7335
VanZ-1 Concanavalin-like
MBK7218174.1
Candidatus Promineofilum glycogenicum
VanZ-1 Concanavalin-like
MCA9929762.1
Anaerolineales bacterium
VanZ-1 Concanavalin-like
NES65824.1
Okeania sp. SIO2D1
VanZ-1 Concanavalin-like
NIA31979.1
Actinomycetota bacterium
VanZ-1 Concanavalin-like
MBN2092021.1
Candidatus Zhuqueibacterota bacterium
VanZ-1 Concanavalin-like
NJO71766.1
Oscillatoriales cyanobacterium RM1_1_9
VanZ-1 Concanavalin-like
WP_088240894.1
Calothrix rhizosoleniae SC01
VanZ-1 Concanavalin-like
PSB62438.1
filamentous cyanobacterium CCP1
VanZ-1 Concanavalin-like
MBP1602736.1
Acidobacteriota bacterium
Concanavalin-like VanZ-
PYQ46069.1
Acidobacteriota bacterium
Concanavalin-like VanZ-
HCO92937.1
Phycisphaerales bacterium
Concanavalin-like VanZ-
MBZ5679316.1
Terriglobia bacterium
Concanavalin-like VanZ-
MBN2037586.1
Chitinispirillaceae bacterium
Concanavalin-like VanZ-
MBP1597551.1
Acidobacteriota bacterium
Concanavalin-like VanZ-
KFO66286.1
Smithella sp. SCADC
Concanavalin-like VanZ-
HIG68998.1
Myxococcales bacterium
Concanavalin-like VanZ-
RJQ60177.1
Desulfobacteraceae bacterium
Concanavalin-like VanZ-
OEU49993.1
Desulfobulbaceae bacterium S5133MH15
Concanavalin-like VanZ-
MBN1663857.1
Deltaproteobacteria bacterium
Concanavalin-like VanZ-
MBW2198746.1
Deltaproteobacteria bacterium
Concanavalin-like VanZ-
NTW16875.1
Syntrophaceae bacterium
Concanavalin-like VanZ-
MBA3035961.1
Desulfobacterium sp.
VanZ-2 ? Concanavalin-like VanZ-
MBN1103281.1
Deltaproteobacteria bacterium
Concanavalin-like VanZ-
MBK7997356.1
Verrucomicrobiota bacterium
Concanavalin-like VanZ-
MBZ5697594.1
Terriglobia bacterium
Concanavalin-like VanZ-
MBV8514726.1
Acidobacteriota bacterium
Concanavalin-like VanZ-
MBZ5671639.1
Terriglobia bacterium
Concanavalin-like VanZ-
MBC2736366.1
Desulfobacteraceae bacterium
Concanavalin-like VanZ-
HAA03083.1
Syntrophobacteraceae bacterium
Concanavalin-like VanZ-
MBC8002962.1
Verrucomicrobiales bacterium
Concanavalin-like VanZ-
HGF94517.1
Concanavalin-like VanZ-
OHD64750.1
Spirochaetes bacterium RBG_13_51_14
Concanavalin-like VanZ-
MBL0713505.1
Desulfobacterales bacterium
Concanavalin-like VanZ-
MCC6389911.1
Bryobacterales bacterium
Concanavalin-like VanZ-
WP_163338154.1
Desulfopila sp. IMCC35008
Concanavalin-like VanZ-
MBN1496874.1
Spirochaetota bacterium
Concanavalin-like VanZ-
WP_143170881.1
Desulfopila aestuarii DSM 18488
Concanavalin-like VanZ-
RKX30145.1
Verrucomicrobiota bacterium
Concanavalin-like VanZ-
MBW2368889.1
Deltaproteobacteria bacterium
Concanavalin-like VanZ-
PYU10217.1
Acidobacteriota bacterium
Concanavalin-like VanZ-
MBW2286989.1
Deltaproteobacteria bacterium
Concanavalin-like VanZ-
RLB12257.1
Deltaproteobacteria bacterium
Concanavalin-like VanZ-
SPF40212.1
Candidatus Sulfotelmatobacter kueseliae
Concanavalin-like VanZ-
CAB5114937.1
Olavius algarvensis associated proteobacterium Delta 3
Concanavalin-like VanZ-
HEC02996.1
Phycisphaerales bacterium
Concanavalin-like VanZ-

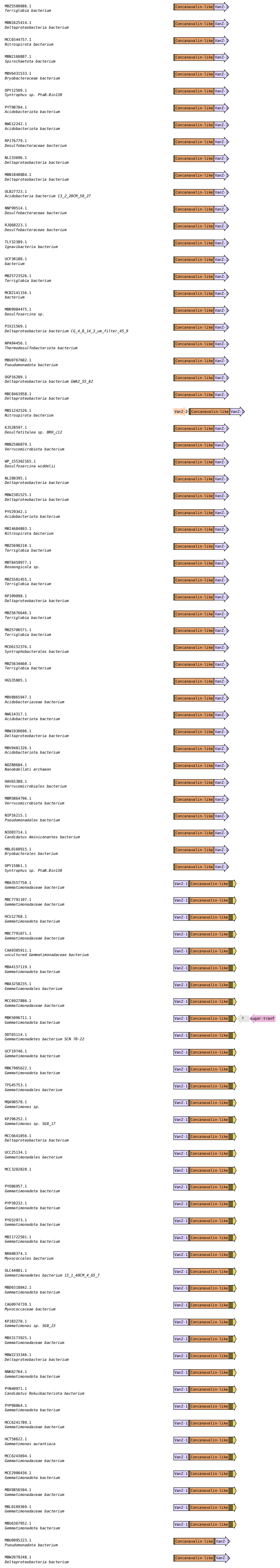

MBZ5500086.1
Terriglobia bacterium
MBN1625414.1
Deltaproteobacteria bacterium
MCC6544757.1
Nitrospirota bacterium
MBN2160887.1
Spirochaetota bacterium
MBV6431533.1
Bryobacteraceae bacterium
OPY12509.1
Syntrophus sp. PtaB.Bin138
PYT98704.1
Acidobacteriota bacterium
NWG12242.1
Acidobacteriota bacterium
RPJ76779.1
Desulfobacteraceae bacterium
NLI33696.1
Deltaproteobacteria bacterium
MBN1848884.1
Deltaproteobacteria bacterium
OLB27723.1
Acidobacteria bacterium 13_2_20CM_58_27
NNF99514.1
Desulfobacteraceae bacterium
RJQ68223.1
Desulfobacteraceae bacterium
TLY32389.1
Ignavibacteria bacterium
UCF30188.1
bacterium
MBZ5723526.1
Terriglobia bacterium
MCB2141156.1
bacterium
MBR9984475.1
Desulfosarcina sp.
PIX21569.1
Deltaproteobacteria bacterium CG_4_8_14_3_um_filter_45_9
NPA94456.1
Thermodesulfobacteriota bacterium
MBU0767602.1
Pseudomonadota bacterium
OGP16289.1
Deltaproteobacteria bacterium GWA2_55_82
MBC8463958.1
Deltaproteobacteria bacterium
MBS1242526.1
Nitrospirota bacterium
KJS28597.1
Desulfatitalea sp. BRH_c12
MBN2506879.1
Verrucomicrobiota bacterium
WP_155302165.1
Desulfosarcina widdelii
NLI80395.1
Deltaproteobacteria bacterium
MBW2381525.1
Deltaproteobacteria bacterium
PYV29342.1
Acidobacteriota bacterium
MBI4684803.1
Nitrospirota bacterium
MBZ5690210.1
Terriglobia bacterium
MBT8459977.1
Boseongicola sp.
MBZ5581455.1
Terriglobia bacterium
RPJ09098.1
Deltaproteobacteria bacterium
MBZ5676646.1
Terriglobia bacterium
MBZ5706571.1
Terriglobia bacterium
MCD6152376.1
Syntrophobacterales bacterium
MBZ5634660.1
Terriglobia bacterium
HGS35805.1
MBV8865947.1
Acidobacteriaceae bacterium
NWG14317.1
Acidobacteriota bacterium
MBW1930696.1
Deltaproteobacteria bacterium
MBV9481326.1
Acidobacteriota bacterium
NOZ80684.1
Nanobdellati archaeon
HAV65388.1
Verrucomicrobiales bacterium
MBM3864796.1
Verrucomicrobiota bacterium
NIP16215.1
Pseudomonadales bacterium
NIO83714.1
Candidatus Aminicenantes bacterium
MBL0160915.1
Bryobacterales bacterium
OPY15861.1
Syntrophus sp. PtaB.Bin138
MBA3557750.1
Gemmatimonadaceae bacterium
MBC7791107.1
Gemmatimonadaceae bacterium
HCU12768.1
Gemmatimonadota bacterium
MBC7791071.1
Gemmatimonadaceae bacterium
CAA9305911.1
uncultured Gemmatimonadaceae bacterium
MBA4157119.1
Gemmatimonadota bacterium
MBA3258235.1
Gemmatimonadales bacterium
MCC6927886.1
Gemmatimonadaceae bacterium
MBK5096711.1
Gemmatimonadota bacterium
ODT03114.1
Gemmatimonadetes bacterium SCN 70-22
UCF19746.1
Gemmatimonadota bacterium
MBK7905622.1
Gemmatimonadota bacterium
TFG45753.1
Gemmatimonadales bacterium
MQA90578.1
Gemmatimonas sp.
KPJ96252.1
Gemmatimonas sp. SG8_17
MCC6641056.1
Deltaproteobacteria bacterium
UCC25134.1
Gemmatimonadales bacterium
MCC3202828.1
PYO86957.1
Gemmatimonadota bacterium
PYP39232.1
Gemmatimonadota bacterium
PYO32973.1
Gemmatimonadota bacterium
MBI1722501.1
Gemmatimonadota bacterium
NRA08374.1
Myxococcales bacterium
OLC44001.1
Gemmatimonadetes bacterium 13_1_40CM_4_65_7
MBD0318842.1
Gemmatimonadota bacterium
CAG0974739.1
Myxococcaceae bacterium
KPJ83270.1
Gemmatimonas sp. SG8_23
MBX3173925.1
Gemmatimonadaceae bacterium
MBW2233346.1
Deltaproteobacteria bacterium
NNK62764.1
Gemmatimonadota bacterium
PYN40971.1
Candidatus Rokuibacteriota bacterium
PYP98864.1
Gemmatimonadota bacterium
MCC6241789.1
Gemmatimonadaceae bacterium
HCT58622.1
Gemmatimonas aurantiaca
MCC6243694.1
Gemmatimonadaceae bacterium
MCE2900436.1
Gemmatimonadota bacterium
MBX9856504.1
Gemmatimonadaceae bacterium
MBL0169369.1
Gemmatimonadaceae bacterium
MBU6367952.1
Gemmatimonadota bacterium
MBU0995223.1
Pseudomonadota bacterium
MBW2079248.1
Deltaproteobacteria bacterium
Concanavalin-like
VanZ-1
VanZ-2
?
sugar-tranf

MBW2741458.1	*Deltaproteobacteria bacterium*	Concanavalin-like · VanZ-1
UCG13349.1	*Deltaproteobacteria bacterium*	Concanavalin-like · VanZ-1
MCD4762466.1	*Desulfobacterales bacterium*	Concanavalin-like · VanZ-1
OEU77936.1	*Desulfobacterales bacterium S5133MH4*	Concanavalin-like · VanZ-1
MBR9984676.1	*Desulfosarcina sp.*	Concanavalin-like · VanZ-1
MBA3731161.1	*Gammaproteobacteria bacterium*	Concanavalin-like · VanZ-1
WP_215619381.1	*Leptothoe kymatousa TAU-MAC 1615*	Concanavalin-like · VanZ-1
PIE73935.1	*Deltaproteobacteria bacterium*	Concanavalin-like · VanZ-1
RLT94731.1	*Ketobacter sp.*	Concanavalin-like · VanZ-1
RLU00726.1	*Ketobacter sp.*	Concanavalin-like · VanZ-1
WP_184742180.1	*Spirochaeta isovalerica*	Concanavalin-like · VanZ-1
HFG18631.1		Concanavalin-like · VanZ-1
HCV42380.1	*Bacteroidota bacterium*	Concanavalin-like · VanZ-1
MBM2846637.1	*Bacteroidota bacterium*	Concanavalin-like · VanZ-1
HEU17959.1	*Deltaproteobacteria bacterium*	Concanavalin-like · VanZ-1
MAI77350.1	*Deltaproteobacteria bacterium*	Concanavalin-like · VanZ-1
MBW2273468.1	*Deltaproteobacteria bacterium*	Concanavalin-like · VanZ-1
MBW2294426.1	*Deltaproteobacteria bacterium*	Concanavalin-like · VanZ-1
HIG72766.1	*Myxococcales bacterium*	Concanavalin-like · VanZ-1
MBN2254264.1	*Deltaproteobacteria bacterium*	Concanavalin-like · VanZ-1
KMQ51151.1	*Chitinispirillum alkaliphilum*	Concanavalin-like · VanZ-1
UCE89991.1	*Pseudomonadota bacterium*	Concanavalin-like · VanZ-1
MCC2662295.1	*Geminicoccaceae bacterium*	Concanavalin-like · VanZ-1
KAF0190927.1	*Gammaproteobacteria bacterium*	Concanavalin-like · VanZ-1
HED18413.1	*Gammaproteobacteria bacterium*	Concanavalin-like · VanZ-1
MBG7601273.1	*Gammaproteobacteria bacterium*	Concanavalin-like · VanZ-1
UCG50819.1	*Candidatus Latescibacterota bacterium*	VanZ-1 · Concanavalin-like
UCH85207.1	*Candidatus Latescibacterota bacterium*	VanZ-1 · Concanavalin-like
MCA9457733.1	*Nitrospira sp.*	VanZ-1 · Concanavalin-like
THJ16495.1	*Nitrospira sp. CG24B*	VanZ-1 · Concanavalin-like
MBK6971784.1	*Candidatus Methylophosphatis roskildensis*	VanZ-1 · Concanavalin-like
MBK7189037.1	*bacterium*	VanZ-1 · Concanavalin-like
PYU29720.1	*Acidobacteriota bacterium*	VanZ-1 · Concanavalin-like
MBS0242551.1	*Pseudomonadota bacterium*	VanZ-1 · Concanavalin-like
TPO11627.1	*Mesorhizobium sp. B1-1-5*	VanZ-1 · Concanavalin-like
KPJ78710.1	*Gammaproteobacteria bacterium SG8_30*	VanZ-1 · Concanavalin-like
NQT27874.1	*Candidatus Zhuqueibacterota bacterium*	VanZ-1 · Concanavalin-like
HGI21652.1		VanZ-1 · Concanavalin-like
MBN2416148.1	*bacterium*	VanZ-1 · Concanavalin-like
RPI29768.1	*Acidobacteriota bacterium*	VanZ-1 · Concanavalin-like
NLU19380.1	*Phycisphaerae bacterium*	VanZ-1 · Concanavalin-like
MBN1342102.1	*Phycisphaerae bacterium*	VanZ-1 · Concanavalin-like
UCF33308.1	*Phycisphaerales bacterium*	VanZ-1 · Concanavalin-like
MBI3805553.1	*Candidatus Manganitrophaceae bacterium*	VanZ-1 · Concanavalin-like
MBP1609242.1	*Acidobacteriota bacterium*	Concanavalin-like · VanZ-1 · MTase
OPY66802.1	*Syntrophorhabdus sp. PtaU1.Bin050*	Concanavalin-like · VanZ-1 · Exosortase_EpsH
MCB1099818.1	*Verrucomicrobiia bacterium*	Concanavalin-like · VanZ-1 · ? · ? · VanZ-assorted-associations

####;OB domain fusions

WP_066020421.1	*Clostridium sp. CT7*	VanZ-1 · OB-like · OB-like · MBL-D-Ala-D-Ala-peptidase · ? · ? · GNAT-N-acetyltransf
TMI73279.1	*Bacteroidota bacterium*	MBL-D-Ala-D-Ala-peptidase · VanZ-1 · OB-like
MBD5392778.1	*Lachnospiraceae bacterium*	VanZ-1 · OB-like · OB-like
WP_191201708.1	*Paenibacillus terricola*	VanZ-1 · OB-like · OB-like
HIX67483.1	*Candidatus Anaerostipes excrementavium*	VanZ-1 · OB-like · OB-like
WP_006565608.1	*Anaerostipes caccae*	VanZ-1 · OB-like · OB-like
GFI30250.1	*Lachnospiraceae bacterium*	VanZ-1 · OB-like · OB-like
MBN2414511.1	*bacterium*	VanZ-1 · OB-like · OB-like
WP_010964147.1	*Clostridium acetobutylicum*	VanZ-1 · OB-like · OB-like
WP_129026844.1	*Aquirufa rosea*	VanZ-1 · OB-like
NBT08678.1	*Chitinophagia bacterium*	VanZ-1 · OB-like
GDX44374.1	*Bacteroidota bacterium*	VanZ-1 · OB-like
NBO58052.1	*Chitinophagia bacterium*	VanZ-1 · OB-like
RVU24117.1	*Sandaracinomonas limnophila*	VanZ-1 · OB-like
WP_214229614.1	*Pedobacter sp. B4-66*	VanZ-1 · OB-like
RJQ34257.1	*Candidatus Parcubacteria bacterium*	VanZ-1 · OB-like
HIU66162.1	*Candidatus Caccomorpha excrementavium*	VanZ-1 · OB-like
HIV91450.1	*Candidatus Eisenbergiella stercoravium*	VanZ-1 · OB-like

####;PepSY domain fusions

WP_184936023.1	*Kitasatospora kifunensis*	VanZ-1 · PepSY · PepSY · PepSY · MBL-D-Ala-D-Ala-peptidase
PKW07964.1	*Streptomyces sp. 5112.2*	VanZ-1 · PepSY · PepSY · PepSY
WP_029384318.1	*Streptomyces leeuwenhoekii*	VanZ-1 · PepSY · PepSY · PepSY
WP_030422562.1	*Streptomyces sp. NRRL F-5065*	VanZ-1 · PepSY · PepSY · PepSY
HBF79841.1	*Streptomyces sp.*	VanZ-1 · PepSY · PepSY · PepSY
WP_102925636.1	*Streptomyces noursei*	VanZ-1 · PepSY · PepSY · PepSY
GHC06899.1	*Streptomyces albogriseolus*	VanZ-1 · PepSY · PepSY · PepSY
WP_129292778.1	*Streptomyces lydicus*	VanZ-1 · PepSY · PepSY · PepSY
NKQ28440.1	*Streptomyces galbus*	VanZ-1 · PepSY · PepSY · PepSY
WP_079075542.1	*Streptomyces griseorubiginosus*	VanZ-1 · PepSY · PepSY · PepSY
WP_161225817.1	*Streptomyces sp. SID8352*	VanZ-1 · PepSY · PepSY · PepSY
OSZ57919.1	*Streptomyces pharetrae CZA14*	VanZ-1 · PepSY · PepSY · PepSY
WP_033211810.1	*Streptomyces viridochromogenes*	VanZ-1 · PepSY · PepSY · PepSY
WP_205077178.1		VanZ-1 · PepSY · PepSY · PepSY
WP_030739228.1	*Streptomyces sp. NRRL F-2890*	VanZ-1 · PepSY · PepSY · PepSY
WP_079048101.1	*Streptomyces hirsutus*	VanZ-1 · PepSY · PepSY · PepSY
GGU74033.1	*Streptomyces filipinensis*	VanZ-1 · PepSY · PepSY · PepSY
WP_205020915.1	*Streptomyces sp. HB132*	VanZ-1 · PepSY · PepSY · PepSY
WP_079159701.1	*Streptomyces griseus*	VanZ-1 · PepSY · PepSY · PepSY
WP_156726991.1	*Streptomyces apocyni*	VanZ-1 · PepSY · PepSY · PepSY
WP_078857989.1	*Streptomyces sp. BI87*	VanZ-1 · PepSY · PepSY · PepSY
WP_170994733.1	*Streptomyces sp. BPSDS2*	VanZ-1 · PepSY · PepSY · PepSY
RPK74565.1	*Streptomyces sp. ADI97-07*	VanZ-1 · PepSY · PepSY · PepSY
WP_182313754.1	*Streptomyces sp. SCUT-3*	VanZ-1 · PepSY · PepSY · PepSY
WP_099156137.1	*Streptomyces viridosporus*	VanZ-1 · PepSY · PepSY · PepSY
QCW77972.1	*Streptomyces sp. S6*	VanZ-1 · PepSY · PepSY · PepSY

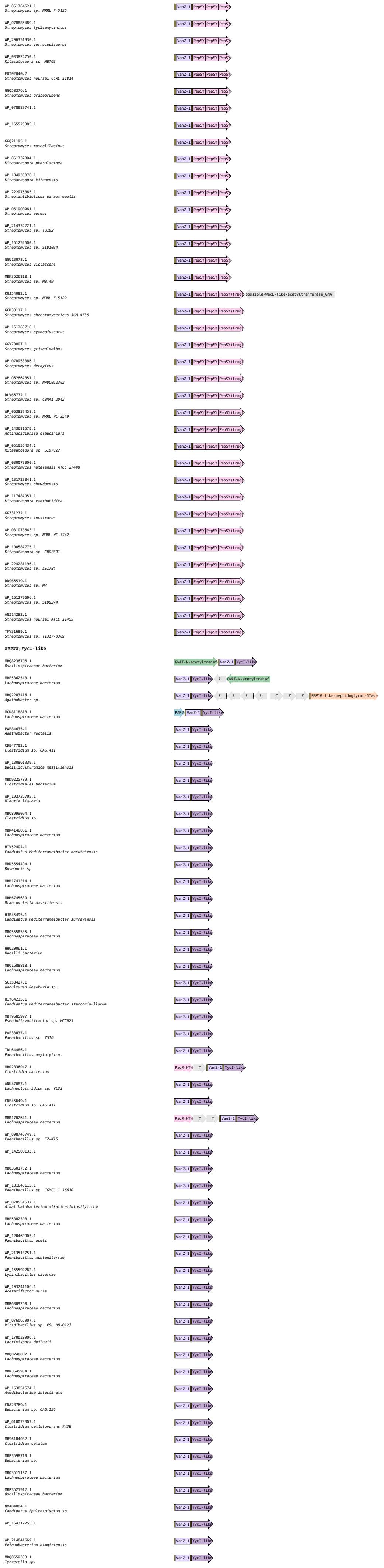
WP_051764621.1
Streptomyces sp. NRRL F-5135
WP_078885489.1
Streptomyces lydicamycinicus
WP_206351930.1
Streptomyces verrucosisporus
WP_033824750.1
Kitasatospora sp. MBT63
EOT02040.2
Streptomyces noursei CCRC 11814
GGQ58376.1
Streptomyces griseorubens
WP_078983741.1
WP_155525305.1
GGQ21195.1
Streptomyces roseolilacinus
WP_051732094.1
Kitasatospora phosalacinea
WP_184935876.1
Kitasatospora kifunensis
WP_222975865.1
Streptantibioticus parmotrematis
WP_051900961.1
Streptomyces aureus
WP_214334221.1
Streptomyces sp. Tu102
WP_161252600.1
Streptomyces sp. SID1034
GGU13078.1
Streptomyces violascens
MBK3626818.1
Streptomyces sp. MBT49
KUJ54082.1
Streptomyces sp. NRRL F-5122
GCD38117.1
Streptomyces chrestomyceticus JCM 4735
WP_161263716.1
Streptomyces cyaneofuscatus
GGV70007.1
Streptomyces griseoloalbus
WP_078953386.1
Streptomyces decoyicus
WP_062667857.1
Streptomyces sp. NPDC052302
RLV66772.1
Streptomyces sp. CBMAI 2042
WP_063837458.1
Streptomyces sp. NRRL WC-3549
WP_143681579.1
Actinacidiphila glaucinigra
WP_051055434.1
Kitasatospora sp. SID7827
WP_030073000.1
Streptomyces natalensis ATCC 27448
WP_131723841.1
Streptomyces showdoensis
WP_117487057.1
Kitasatospora xanthocidica
GGZ31272.1
Streptomyces inusitatus
WP_031078643.1
Streptomyces sp. NRRL WC-3742
WP_100587775.1
Kitasatospora sp. CB02891
WP_224281196.1
Streptomyces sp. LS1784
RDS66519.1
Streptomyces sp. M7
WP_161279696.1
Streptomyces sp. SID8374
ANZ14282.1
Streptomyces noursei ATCC 11455
TFV31689.1
Streptomyces sp. T1317-0309
VanZ-1
PepSY
PepSY(frag)
possible-WecE-like-acetyltranferase_GNAT
#####;YycI-like
MBQ8236706.1
Oscillospiraceae bacterium
MBE5862548.1
Lachnospiraceae bacterium
MBQ2283416.1
Agathobacter sp.
MCD8118818.1
Lachnospiraceae bacterium
PWE84635.1
Agathobacter rectalis
CDE47702.1
Clostridium sp. CAG:411
WP_130861339.1
Bacilliculturomica massiliensis
MBD9225789.1
Clostridiales bacterium
WP_193735705.1
Blautia liquoris
MBQ8999094.1
Clostridium sp.
MBR4146061.1
Lachnospiraceae bacterium
HIV52404.1
Candidatus Mediterraneibacter norwichensis
MBD5554494.1
Roseburia sp.
MBR1741214.1
Lachnospiraceae bacterium
MBM6745630.1
Drancourtella massiliensis
HJB45495.1
Candidatus Mediterraneibacter surreyensis
MBQ5558535.1
Lachnospiraceae bacterium
HHU20061.1
Bacilli bacterium
MBQ1688818.1
Lachnospiraceae bacterium
SCI58427.1
uncultured Roseburia sp.
HIY64235.1
Candidatus Mediterraneibacter stercoripullorum
MBT9685997.1
Pseudoflavonifractor sp. MCC625
PAF33837.1
Paenibacillus sp. 7516
TDL64486.1
Paenibacillus amylolyticus
MBQ2836047.1
Clostridia bacterium
ANU47087.1
Lachnoclostridium sp. YL32
CDE45649.1
Clostridium sp. CAG:411
MBR1702641.1
Lachnospiraceae bacterium
WP_098746749.1
Paenibacillus sp. EZ-K15
WP_142508133.1
MBQ3601752.1
Lachnospiraceae bacterium
WP_181646115.1
Paenibacillus sp. CGMCC 1.16610
WP_078551637.1
Alkalihalobacterium alkalicellulosilyticum
MBE5882308.1
Lachnospiraceae bacterium
WP_120460905.1
Paenibacillus aceti
WP_213518751.1
Paenibacillus montaniterrae
WP_155592262.1
Lysinibacillus cavernae
WP_103241106.1
Acetatifactor muris
MBR6309260.1
Lachnospiraceae bacterium
WP_076065907.1
Viridibacillus sp. FSL H8-0123
WP_170822900.1
Lacrimispora defluvii
MBQ8248002.1
Lachnospiraceae bacterium
MBR3645934.1
Lachnospiraceae bacterium
WP_163051674.1
Amedibacterium intestinale
CDA28769.1
Eubacterium sp. CAG:156
WP_010073307.1
Clostridium cellulovorans 743B
MBS6184082.1
Clostridium celatum
MBP3598710.1
Eubacterium sp.
MBQ3515187.1
Lachnospiraceae bacterium
MBP3521912.1
Oscillospiraceae bacterium
NMA84884.1
Candidatus Epulonipiscium sp.
WP_154312255.1
WP_214841669.1
Exiguobacterium himgiriensis
MBQ8559333.1
Tyzzerella sp.
GNAT-N-acetyltransf
VanZ-1
YycI-like
?
PBP1A-like-peptidoglycan-GTase
PAP2
PadR-HTH

Accession	Organism	Gene neighborhood
WP_169360455.1	*uncultured Psychrobacillus sp.*	VanZ-1 · YycI-like
MBO5324249.1	*Oscillospiraceae bacterium*	VanZ-1 · YycI-like
WP_168391897.1	*Bacillus cereus*	VanZ-1 · YycI-like
HAU85933.1	*Lachnospiraceae bacterium*	VanZ-1 · YycI-like · sugar-O-acetyl/acyltransf
MBQ9839249.1	*Oscillospiraceae bacterium*	VanZ-1 · YycI-like
HJD04312.1	*Candidatus Mediterraneibacter caccogallinarum*	VanZ-1 · YycI-like
MBO4781936.1	*Lachnospiraceae bacterium*	VanZ-1 · YycI-like
OUQ01110.1	*Thomasclavelia spiroformis*	VanZ-1 · YycI-like
WP_115481809.1	*Anaerosacchariphilus polymeriproducens*	VanZ-1 · YycI-like
WP_193831998.1	*Lysinibacillus fusiformis*	VanZ-1 · YycI-like
WP_162360984.1	*Aminipila terrae*	VanZ-1 · YycI-like · ? · PadR-HTH
MBS4974529.1	*Clostridium celatum*	VanZ-1 · YycI-like
MBE6034172.1	*Clostridiales bacterium*	VanZ-1 · YycI-like · PadR-HTH
KMW27912.1	*Clostridium sp. 1_1_41A1FAA*	VanZ-1 · YycI-like
WP_195929588.1	*uncultured Intestinibacter sp.*	VanZ-1 · YycI-like
MBO4938902.1	*Oscillospiraceae bacterium*	VanZ-1 · YycI-like
OQA73886.1	*Firmicutes bacterium ADurb.Bin248*	VanZ-1 · YycI-like
MBQ8802298.1	*Tyzzerella sp.*	VanZ-1 · YycI-like
WP_066446601.1	*Longibaculum muris*	VanZ-1 · YycI-like
MBR3973982.1	*Oscillospiraceae bacterium*	VanZ-1 · YycI-like
HAQ40227.1	*Clostridiales bacterium*	VanZ-1 · YycI-like
MBS5112776.1	*Coprobacillus cateniformis*	VanZ-1 · YycI-like
PWL88833.1	*Escherichia coli*	VanZ-1 · YycI-like
WP_213166706.1	*Alkalicella caledoniensis*	VanZ-1 · YycI-like
WP_118558155.1	*Lachnotalea sp. AF33-28*	VanZ-1 · YycI-like
NLM35434.1	*Clostridiales bacterium*	VanZ-1 · YycI-like
MBW4828104.1	*Clostridiaceae bacterium*	VanZ-1 · YycI-like
MBQ4598543.1	*Clostridia bacterium*	VanZ-1 · YycI-like
MBQ9842677.1	*Oscillospiraceae bacterium*	VanZ-1 · YycI-like
EGG83601.1	*Lachnospiraceae bacterium 2_1_46FAA*	VanZ-1 · YycI-like · ? · LPS-like-sugar-transf
MBO5525132.1	*Clostridia bacterium*	VanZ-1 · YycI-like
OUQ34247.1	*Massilimicrobiota timonensis*	VanZ-1 · YycI-like
HAU84392.1	*Lachnospiraceae bacterium*	VanZ-1 · YycI-like
MCD8189145.1	*Clostridiales bacterium*	VanZ-1 · YycI-like
MBR5993179.1	*Lachnospiraceae bacterium*	VanZ-1 · YycI-like
WP_062106777.1	*Bacillus niameyensis*	VanZ-1 · YycI-like
HIZ11660.1	*Candidatus Eubacterium faecavium*	VanZ-1 · YycI-like · VanZ-1
WP_075689288.1	*Bacillus sp. MRMR6*	VanZ-1 · YycI-like
WP_013490775.1	*Evansella cellulosilytica DSM 2522*	VanZ-1 · YycI-like
HBI05444.1	*Paenibacillaceae bacterium*	VanZ-1 · YycI-like
MBE6695043.1	*Oscillospiraceae bacterium*	VanZ-1 · YycI-like
WP_066229071.1	*Metabacillus fastidiosus NBRC 101226*	VanZ-1 · YycI-like
MBP3633658.1	*Oscillospiraceae bacterium*	VanZ-1 · YycI-like
WP_066867353.1	*Neglectibacter timonensis*	VanZ-1 · YycI-like
WP_143849781.1	*Alkalicoccobacillus porphyridii*	VanZ-1 · YycI-like
MBS7210589.1	*Lachnospiraceae bacterium*	VanZ-1 · YycI-like · ? · LPS-like-sugar-transf
MBE6980445.1	*Oscillospiraceae bacterium*	VanZ-1 · YycI-like · VanH-like
WP_129736950.1	*Massilimicrobiota timonensis*	VanZ-1 · YycI-like
RSK25152.1	*Bhargavaea beijingensis*	VanZ-1 · YycI-like
WP_212974333.1	*Bacillus sp. J14TS2*	sugar-O-acetyl/acyltransf · ? · VanZ-1 · YycI-like
MCA9748456.1	*Romboutsia sp.*	VanZ-1 · YycI-like
WP_010290048.1	*Kurthia massiliensis*	VanZ-1 · YycI-like
KIR01817.1	*Lachnospiraceae bacterium TWA4*	VanZ-1 · YycI-like
MCD7894980.1	*Erysipelotrichaceae bacterium*	VanZ-1 · YycI-like
HIT61744.1	*Candidatus Fimousia stercorigallinarum*	VanZ-1 · YycI-like
MBQ2920852.1	*Oscillospiraceae bacterium*	VanZ-1 · YycI-like
NLZ47315.1	*Clostridiales bacterium*	VanZ-1 · YycI-like
MBR6595390.1	*Oscillospiraceae bacterium*	VanZ-1 · YycI-like
MBM0067515.1	*Alkalicoccobacillus gibsonii*	VanZ-1 · YycI-like
MBQ2911085.1	*Clostridia bacterium*	PadR-HTH · ? · VanZ-1 · YycI-like
MBQ8208266.1	*Clostridia bacterium*	VanZ-1 · YycI-like
HJA65460.1	*Candidatus Mediterraneibacter cottocaccae*	VanZ-1 · YycI-like
MBO6300309.1	*Lachnospiraceae bacterium*	VanZ-1 · YycI-like
NLB41743.1	*Clostridiales bacterium*	VanZ-1 · YycI-like
MBR2423454.1	*Oscillospiraceae bacterium*	VanZ-1 · YycI-like
MBO4678927.1	*Lachnospiraceae bacterium*	VanZ-1 · YycI-like
MBP5281885.1	*Lachnospiraceae bacterium*	VanZ-1 · YycI-like
HIZ11659.1	*Candidatus Eubacterium faecavium*	VanZ-1 · YycI-like · VanZ-1

####;RDD fusions

Accession	Organism	Gene neighborhood
WP_065960714.1	*Streptomyces sparsogenes DSM 40356*	VanZ-1 · RDD · disordered · ? · ? · PBP5_C · MBL-D-Ala-D-Ala-peptidase
WP_063734910.1	*Streptomyces sp. RTd22*	VanZ-1 · RDD · disordered · ? · ? · PBP5_C · MBL-D-Ala-D-Ala-peptidase
NUR92617.1	*Nonomuraea sp.*	VanZ-1 · RDD · PSE · MBL-D-Ala-D-Ala-peptidase
WP_181611200.1	*Nonomuraea soli*	VanZ-1 · RDD · ? · MBL-D-Ala-D-Ala-peptidase
WP_143913018.1	*Aeromicrobium piscarium*	MurB-reductase · ? · tRNA · tRNA · ? · VanZ-1 · RDD
WP_002195087.1		BacA-like-undecaprenyl-diphosphate-phosphatase · ? · VanZ-1 · RDD · disordered

#;some genuine association between the GNAT and VanZ-1, with RDD fusion?

Accession	Organism	Gene neighborhood
WP_143446677.1	*Kibdelosporangium aridum*	VanZ-1 · RDD · GNAT-N-acetyltransf · PBP1A-like-peptidoglycan-GTase
ERJ01056.1	*Clostridium sp. ATCC 29733*	VanZ-1 · RDD · GNAT-N-acetyltransf
TDP97334.1	*Labedaea rhizosphaerae*	GNAT-N-acetyltransf · VanZ-1 · RDD
HJE21155.1	*Bifidobacterium pullorum*	GNAT-N-acetyltransf · ? · VanZ-1 · RDD
SHE14848.1	*Chlamydia abortus*	GNAT-N-acetyltransf · ? · ? · ? · ? · ? · VanZ-1 · RDD
NLD76284.1	*Acidimicrobiales bacterium*	VanZ-1 · RDD · ? · ? · GNAT-N-acetyltransf
MBP7630316.1	*Acidimicrobiales bacterium*	VanZ-1 · RDD · ? · ? · GNAT-N-acetyltransf
WP_105567200.1	*Microbacterium halophytorum*	VanZ-1 · RDD · ? · GNAT-N-acetyltransf
WP_132122783.1	*Actinocrispum wychmicini*	Teichoic-acid-transferase-TagU · ? · VanZ-1 · RDD
WP_231951179.1		VanZ-1 · RDD · ? · ? · Teichoic-acid-transferase-TagU
WP_195308928.1	*Flavonifractor plautii*	VanZ-1 · RDD
MBK7822290.1	*Tessaracoccus sp.*	VanZ-1 · RDD · ? · ? · ? · PBP1A-like-peptidoglycan-GTase
MBP3920671.1	*Bacilli bacterium*	VanZ-1 · RDD · ? · monofunctional-peptidoglycan-glycosyltransferase
WP_075891414.1	*Actinomyces provencensis*	VanZ-1 · RDD · disordered · ? · ? · PSE · ? · ? · ? · disordered · RDD · VanZ-1
WP_075891410.1	*Actinomyces provencensis*	disordered · RDD · VanZ-1 · ? · ? · PSE · ? · ? · ? · VanZ-1 · RDD · disordered
WP_184749061.1	*Microbacterium thalassium*	PAP2 · VanZ-1 · RDD

#;fusion to YjeF_N-Rossmann

Accession	Organism	Gene neighborhood
HIW77125.1	*Candidatus Gordonibacter avicola*	VanZ-1 · RDD · YjeF_N-Rossmann

MBL6464128.1 *Senegalimassilia sp.* — VanZ-1 | RDD | YjeF_N-Rossmann

MSA62973.1 *Gordonibacter pamelaeae* — VanZ-1 | RDD | YjeF_N-Rossmann

PWM43534.1 *Coriobacteriia bacterium* — VanZ-1 | RDD | YjeF_N-Rossmann

WP_087193424.1 *Gordonibacter sp. An230* — VanZ-1 | RDD | YjeF_N-Rossmann

HIY84462.1 *Candidatus Rubneribacter avistercoris* — VanZ-1 | RDD | YjeF_N-Rossmann

#;fusion to HisKin

MBR5231476.1 *Clostridia bacterium* — VanZ-1 | RDD | HisKin

MBO7252323.1 *Oscillospiraceae bacterium* — VanZ-1 | RDD | HisKin

MBS7305272.1 *Lachnospiraceae bacterium* — VanZ-1 | RDD | HisKin

MBR4208559.1 *Lachnospiraceae bacterium* — VanZ-1 | RDD | HisKin

MBO5842950.1 *Clostridia bacterium* — VanZ-1 | RDD | HisKin

# 10;fusion to DUF218-Rossmann (linked to ECA inhibition PMID: 34809459)

NLG21421.1 *Actinomycetales bacterium* — VanZ-1 | RDD | DUF218-Rossmann

NLH71094.1 *Brooklawnia sp.* — VanZ-1 | RDD | DUF218-Rossmann

MCB1255567.1 *Microthrixaceae bacterium* — VanZ-1 | RDD | DUF218-Rossmann

WP_129399018.1 *Nocardioides oleivorans* — VanZ-1 | RDD | DUF218-Rossmann

WP_141818919.1 *Ornithinimicrobium humiphilum* — VanZ-1 | RDD | DUF218-Rossmann

WP_204133255.1 *Phycicoccus sp. CSK15P-2* — VanZ-1 | RDD | DUF218-Rossmann

WP_114930383.1 — VanZ-1 | RDD | DUF218-Rossmann | Sulfatase

#;standalone

MCB0986830.1 *Acidimicrobiales bacterium* — VanZ-1 | RDD

MBK6855591.1 *Microthrixaceae bacterium* — VanZ-1 | RDD

WP_090289103.1 — VanZ-1 | RDD

HIS87452.1 *Candidatus Caccenecus avistercoris* — VanZ-1 | RDD

CDF11165.1 *Mycoplasma sp. CAG:776* — VanZ-1 | RDD

WP_154053153.1 *Olsenella uli* — VanZ-1 | RDD | disordered

WP_051739122.1 — VanZ-1 | RDD | disordered

NUS85626.1 *Streptomyces sp.* — VanZ-1 | RDD | disordered

GHJ40216.1 *Streptomyces sp. TS71-3* — VanZ-1 | RDD | disordered

WP_130861110.1 *Bacilliculturomica massiliensis* — VanZ-1 | RDD | disordered

WP_029088409.1 *Brevibacterium album DSM 18261* — VanZ-1 | RDD | disordered

SCH23382.1 *uncultured Collinsella sp.* — VanZ-1 | RDD | disordered

TFF12875.1 *Cellulosimicrobium funkei* — disordered | VanZ-1 | RDD

QOW00907.1 *Rhodococcus pyridinivorans* — disordered | VanZ-1 | RDD

PPG82256.1 *Rathayibacter sp. AY1E5* — disordered | VanZ-1 | RDD

MBW3086510.1 *Austwickia sp. TVS 96-490-7B* — disordered | VanZ-1 | RDD

WP_206093914.1 *Paenibacillus apiarius* — VanZ-1 | RDD | disordered

WP_232275338.1 *Paenibacillus sp. 481* — VanZ-1 | RDD | disordered

WP_028544165.1 — VanZ-1 | RDD | disordered

GJQ02612.1 — VanZ-1 | RDD | disordered

PAU69801.1 *Bifidobacterium italicum* — VanZ-1 | RDD | disordered

GBQ00469.1 *Streptomyces spongiicola* — VanZ-1 | RDD | disordered

WP_087204272.1 *Collinsella sp. An268* — VanZ-1 | RDD | disordered

WP_087430970.1 *Collinsella sp. An307* — VanZ-1 | RDD | disordered

WP_190852722.1 *Streptomyces aurantiacus* — VanZ-1 | RDD | disordered

GHG71073.1 *Amycolatopsis acidiphila* — VanZ-1 | RDD | disordered

MBQ6585716.1 *Coriobacteriales bacterium* — VanZ-1 | RDD | disordered

WP_100201693.1 *Streptomyces carminius* — VanZ-1 | RDD | disordered

RUQ09192.1 *Curtobacterium sp. HSID17257* — VanZ-1 | RDD | disordered

MBS6374933.1 *Erysipelotrichaceae bacterium* — VanZ-1 | RDD | disordered

MUH59252.1 *Bifidobacterium canis* — VanZ-1 | RDD | disordered

MBK0865816.1 *Saccharopolyspora galaxeae* — VanZ-1 | RDD | disordered

WP_129621529.1 *Candidatus Nanoperiomorbus periodonticus* — VanZ-1 | RDD | disordered

NBH68886.1 *Bifidobacterium pseudolongum* — VanZ-1 | RDD | disordered

WP_225048478.1 *Lacticaseibacillus kribbianus* — VanZ-1 | RDD | disordered

SNT16058.1 *Micrococcales bacterium KH10* — VanZ-1 | RDD | disordered

WP_164493191.1 *Streptomyces sp. ADI96-02* — VanZ-1 | RDD | disordered

AYW77454.1 *Propionibacterium acidifaciens* — VanZ-1 | RDD | disordered

WP_184862519.1 *Kutzneria kofuensis* — VanZ-1 | RDD | disordered

HBS89531.1 *Eubacterium sp.* — VanZ-1 | RDD | disordered

NLI20253.1 *Clostridiales bacterium* — VanZ-1 | RDD | disordered

PWM98587.1 *Propionibacterium sp.* — VanZ-1 | RDD | disordered

RVU97592.1 *Coriobacteriales bacterium OH1046* — VanZ-1 | RDD | disordered

WP_075739877.1 *Actinoalloteichus sp. GBA129-24* — VanZ-1 | RDD | disordered

WP_224957090.1 *Saccharopolyspora sp. 6T* — VanZ-1 | RDD | disordered

RGD41769.1 *Erysipelotrichaceae bacterium AM07-12* — VanZ-1 | RDD | disordered

WP_196028888.1 *Longicatena caecimuris* — VanZ-1 | RDD | disordered

EFE45421.1 *Erysipelotrichaceae bacterium 5_2_54FAA* — VanZ-1 | RDD | disordered

WP_012797967.1 *Slackia heliotrinireducens* — VanZ-1 | RDD | disordered

MBS6865524.1 *Oscillospiraceae bacterium* — VanZ-1 | RDD | disordered

NCC07405.1 *Clostridia bacterium* — VanZ-1 | RDD | disordered

MBF1035736.1 *Candidatus Nanosynbacter sp.* — VanZ-1 | RDD | disordered

WP_233108137.1 *[Clostridium] innocuum* — VanZ-1 | RDD | disordered

MBQ1443278.1 *Renibacterium sp.* — VanZ-1 | RDD | disordered

WP_016312517.1 *Paenibacillus sp. 3LSP* — VanZ-1 | RDD | disordered

WP_030013352.1 *Curtobacterium sp. S6* — VanZ-1 | RDD | disordered

HIU66620.1 *Candidatus Caccomorpha excrementavium* — sugar-O-acetyl/acyltransf | ? | VanZ-1 | RDD | disordered

QCT37070.1 *Arachnia propionica* — VanZ-1 | RDD | disordered

WP_119212381.1 *Lachnospira eligens* — VanZ-1 | RDD | disordered

WP_193118718.1 *Brachybacterium tyrofermentans* — VanZ-1 | RDD | disordered

WP_078787523.1 *Eubacterium ruminantium* — VanZ-1 | RDD | disordered

WP_185000953.1 *Crossiella cryophila* — VanZ-1 | RDD | VanZ-1 | RDD | disordered

MBS5451795.1 *Coriobacteriia bacterium* — VanZ-1 | RDD | disordered

HIU23206.1 *Candidatus Fimihabitans intestinipullorum* — VanZ-1 | RDD | disordered

HIY66629.1 *Candidatus Agrococcus pullicola* — VanZ-1 | RDD | disordered

HIR81890.1 *Candidatus Limiplasma merdipullorum* — VanZ-1 | RDD | disordered

RJQ77605.1 *Pseudonocardiaceae bacterium YIM PH 21723* — VanZ-1 | RDD | disordered

WP_077167370.1 *Lacticaseibacillus rhamnosus* — VanZ-1 | RDD | disordered

MBR1848579.1 *Lachnospiraceae bacterium* — VanZ-1 | RDD | disordered

NBK96952.1 *Erysipelotrichia bacterium* — VanZ-1 | RDD | disordered

WP_203951104.1 *Planotetraspora mira* — VanZ-1 | RDD | disordered

WP_078410419.1 *Priestia abyssalis* — VanZ-1 | RDD-inactive

WP_010290980.1 *Kurthia massiliensis* — VanZ-1 | RDD-inactive

WP_068592158.1 *Paenibacillus macquariensis* — VanZ-1 | RDD-inactive

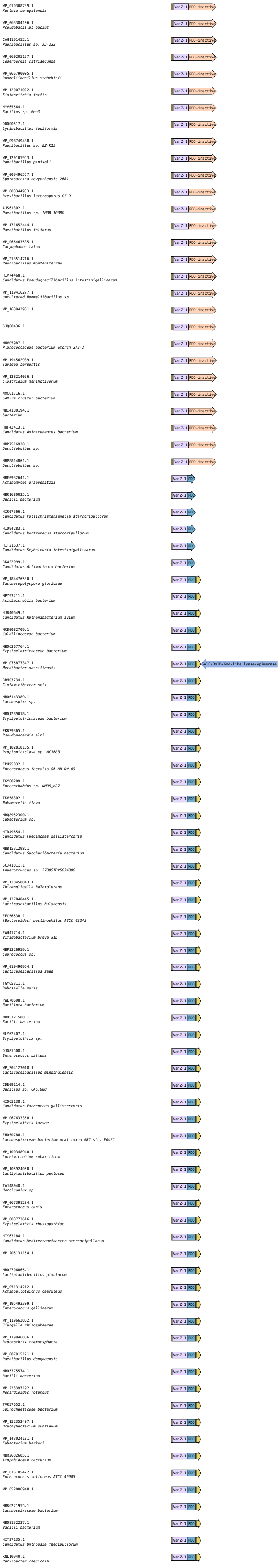
WP_010308739.1
Kurthia senegalensis
VanZ-1 RDD-inactive
WP_063384106.1
Pseudobacillus badius
VanZ-1 RDD-inactive
CAH1191452.1
Paenibacillus sp. JJ-223
VanZ-1 RDD-inactive
WP_060205127.1
Lederbergia citrisecunda
VanZ-1 RDD-inactive
WP_066790005.1
Rummeliibacillus stabekisii
VanZ-1 RDD-inactive
WP_120071022.1
Siminovitchia fortis
VanZ-1 RDD-inactive
NYV65564.1
Bacillus sp. Gen3
VanZ-1 RDD-inactive
QDQ00517.1
Lysinibacillus fusiformis
VanZ-1 RDD-inactive
WP_098749488.1
Paenibacillus sp. EZ-K15
VanZ-1 RDD-inactive
WP_120105953.1
Paenibacillus pinisoli
VanZ-1 RDD-inactive
WP_009496557.1
Sporosarcina newyorkensis 2681
VanZ-1 RDD-inactive
WP_003344933.1
Brevibacillus laterosporus GI-9
VanZ-1 RDD-inactive
AJS61392.1
Paenibacillus sp. IHBB 10380
VanZ-1 RDD-inactive
WP_171652444.1
Paenibacillus foliorum
VanZ-1 RDD-inactive
WP_066463585.1
Caryophanon latum
VanZ-1 RDD-inactive
WP_213514716.1
Paenibacillus montaniterrae
VanZ-1 RDD-inactive
HIV74468.1
Candidatus Pseudogracilibacillus intestinigallinarum
VanZ-1 RDD-inactive
WP_119416277.1
uncultured Rummeliibacillus sp.
VanZ-1 RDD-inactive
WP_163942901.1
VanZ-1 RDD-inactive
GJQ00436.1
VanZ-1 RDD-inactive
MUV05987.1
Planococcaceae bacterium Storch 2/2-2
VanZ-1 RDD-inactive
WP_194562989.1
Savagea serpentis
VanZ-1 RDD-inactive
WP_128214026.1
Clostridium manihotivorum
VanZ-1 RDD-inactive
NMC61716.1
SAR324 cluster bacterium
VanZ-1 RDD-inactive
MBI4180194.1
bacterium
VanZ-1 RDD-inactive
HHF43413.1
Candidatus Aminicenantes bacterium
VanZ-1 RDD-inactive
MBP7516920.1
Desulfobulbus sp.
VanZ-1 RDD-inactive
MBP8814861.1
Desulfobulbus sp.
VanZ-1 RDD-inactive
MBF0932641.1
Actinomyces graevenitzii
VanZ-1 RDD
MBR1680035.1
Bacilli bacterium
VanZ-1 RDD
HIR07366.1
Candidatus Pullichristensenella stercoripullorum
VanZ-1 RDD
HIQ94283.1
Candidatus Ventrenecus stercoripullorum
VanZ-1 RDD
HIT21637.1
Candidatus Scybalousia intestinigallinarum
VanZ-1 RDD
RKW22099.1
Candidatus Altimarinota bacterium
VanZ-1 RDD
WP_184476530.1
Saccharopolyspora gloriosae
VanZ-1 RDD
MPY93211.1
Acidimicrobiia bacterium
VanZ-1 RDD
HJB40649.1
Candidatus Ruthenibacterium avium
VanZ-1 RDD
MCB0082709.1
Caldilineaceae bacterium
VanZ-1 RDD
MBQ6367764.1
Erysipelotrichaceae bacterium
VanZ-1 RDD
WP_075877347.1
Merdibacter massiliensis
VanZ-1 RDD GalE/RmlB/Gmd-like_lyase/epimerase
RBM03734.1
Glutamicibacter soli
VanZ-1 RDD
MBO6143389.1
Lachnospira sp.
VanZ-1 RDD
MBQ1289018.1
Erysipelotrichaceae bacterium
VanZ-1 RDD
PKB29365.1
Pseudonocardia alni
VanZ-1 RDD
WP_182818185.1
Propioniciclava sp. MC1683
VanZ-1 RDD
EPH95032.1
Enterococcus faecalis 06-MB-DW-09
VanZ-1 RDD
TGY68289.1
Enterorhabdus sp. NM05_H27
VanZ-1 RDD
TKV58392.1
Nakamurella flava
VanZ-1 RDD
MBQ8952300.1
Eubacterium sp.
VanZ-1 RDD
HIR49654.1
Candidatus Faecimonas gallistercoris
VanZ-1 RDD
MBB1531298.1
Candidatus Saccharibacteria bacterium
VanZ-1 RDD
SCJ41011.1
Anaerotruncus sp. 2789STDY5834896
VanZ-1 RDD
WP_130450843.1
Zhihengliuella halotolerans
VanZ-1 RDD
WP_127848445.1
Lacticaseibacillus hulanensis
VanZ-1 RDD
EEC56538.1
[Bacteroides] pectinophilus ATCC 43243
VanZ-1 RDD
EWH41714.1
Bifidobacterium breve 31L
VanZ-1 RDD
MBP3326959.1
Coprococcus sp.
VanZ-1 RDD
WP_010490964.1
Lacticaseibacillus zeae
VanZ-1 RDD
TGY65311.1
Dubosiella muris
VanZ-1 RDD
PWL70698.1
Bacillota bacterium
VanZ-1 RDD
MBO5121508.1
Bacilli bacterium
VanZ-1 RDD
NLY62407.1
Erysipelothrix sp.
VanZ-1 RDD
OJG81508.1
Enterococcus pallens
VanZ-1 RDD
WP_204123018.1
Lacticaseibacillus mingshuiensis
VanZ-1 RDD
CDE09114.1
Bacillus sp. CAG:988
VanZ-1 RDD
HIQ65138.1
Candidatus Faecenecus gallistercoris
VanZ-1 RDD
WP_067633358.1
Erysipelothrix larvae
VanZ-1 RDD
EHO50788.1
Lachnospiraceae bacterium oral taxon 082 str. F0431
VanZ-1 RDD
WP_100348940.1
Luteimicrobium subarcticum
VanZ-1 RDD
WP_105924058.1
Lactiplantibacillus pentosus
VanZ-1 RDD
TAJ48048.1
Herbiconiux sp.
VanZ-1 RDD
WP_067391284.1
Enterococcus canis
VanZ-1 RDD
WP_003773616.1
Erysipelothrix rhusiopathiae
VanZ-1 RDD
HIY63184.1
Candidatus Mediterraneibacter stercoripullorum
VanZ-1 RDD
WP_205131154.1
VanZ-1 RDD
MBO2706865.1
Lactiplantibacillus plantarum
VanZ-1 RDD
WP_051314212.1
Actinoalloteichus caeruleus
VanZ-1 RDD
WP_195493309.1
Enterococcus gallinarum
VanZ-1 RDD
WP_119662862.1
Jiangella rhizosphaerae
VanZ-1 RDD
WP_119946066.1
Brochothrix thermosphacta
VanZ-1 RDD
WP_087915171.1
Paenibacillus donghaensis
VanZ-1 RDD
MBO5375574.1
Bacilli bacterium
VanZ-1 RDD
WP_223397192.1
Nocardioides rotundus
VanZ-1 RDD
TVR57652.1
Spirochaetaceae bacterium
VanZ-1 RDD
WP_152352407.1
Brachybacterium subflavum
VanZ-1 RDD
WP_143024181.1
Eubacterium barkeri
VanZ-1 RDD
MBR2682685.1
Atopobiaceae bacterium
VanZ-1 RDD
WP_016185422.1
Enterococcus sulfureus ATCC 49903
VanZ-1 RDD
WP_052006948.1
VanZ-1 RDD
MBR6221955.1
Lachnospiraceae bacterium
VanZ-1 RDD
MBQ8132237.1
Bacilli bacterium
VanZ-1 RDD
HIT37135.1
Candidatus Onthousia faecipullorum
VanZ-1 RDD
RNL10940.1
Parvibacter caecicola
VanZ-1 RDD

MBO5458748.1	*Lachnospira sp.*	VanZ-1 RDD
ERF58552.1	*Cutibacterium avidum TM16*	VanZ-1 RDD
WP_161899768.1	*Candidatus Enterococcus leclercqii*	VanZ-1 RDD
MBQ6359814.1	*Lachnospiraceae bacterium*	VanZ-1 RDD
WP_104963191.1		VanZ-1 RDD
RXZ84878.1	*Paenibacillaceae bacterium*	VanZ-1 RDD
SCI90390.1	*uncultured Flavonifractor sp.*	VanZ-1 RDD
WP_161903267.1	*Enterococcus saccharolyticus*	VanZ-1 RDD
WP_170156186.1	*Umezawaea tangerina*	VanZ-1 RDD
WP_100365064.1	*Diaminobutyricimonas aerilata*	VanZ-1 RDD
MBQ9867131.1	*Lachnospiraceae bacterium*	VanZ-1 RDD
WP_169393689.1	*Pseudonocardia xinjiangensis*	VanZ-1 RDD
WP_142059785.1	*Pseudonocardia kunmingensis*	VanZ-1 RDD
WP_051985849.1	*Paenibacillus macerans*	VanZ-1 RDD
WP_087230243.1	*Faecalibacterium sp. An58*	VanZ-1 RDD
WP_205216445.1	*Enterococcus hirae*	VanZ-1 RDD
OWW68154.1	*Enterococcus hirae 57-03-H11*	VanZ-1 RDD
HIY58458.1	*Candidatus Tetragenococcus pullicola*	VanZ-1 RDD
EHK9995798.1		VanZ-1 RDD
PSL51572.1	*Saccharothrix carnea*	VanZ-1 RDD
EGP5137020.1		VanZ-1 RDD
MBK9156922.1	*Micropruina sp.*	VanZ-1 RDD
WP_061982938.1	*Amycolatopsis regifaucium*	VanZ-1 RDD
EGP5457292.1		VanZ-1 RDD
OMG46852.1	*Paenibacillus macerans*	VanZ-1 RDD
EGP5397467.1		VanZ-1 RDD
NLH63401.1	*Erysipelotrichaceae bacterium*	VanZ-1 RDD
RKI95517.1	*bacterium D16-34*	VanZ-1 RDD; sugar-O-acetyl/acyltransf
CCX37400.1	*Clostridium sp. CAG:1013*	VanZ-1 RDD
MTG90676.1	*Cellulosimicrobium composti*	VanZ-1 RDD
OHE66870.1	*Treponema sp. GWA1_62_8*	VanZ-1 RDD
WP_080063509.1	*Ruminiclostridium hungatei*	VanZ-1 RDD
MBO4360012.1	*Eubacteriaceae bacterium*	VanZ-1 RDD
MCD8010387.1	*Lachnospiraceae bacterium*	VanZ-1 RDD
HIS38159.1	*Candidatus Onthousia faecavium*	VanZ-1 RDD
SFR85743.1	*Agromyces sp. CF514*	VanZ-1 RDD
WP_183512867.1	*Paeniglutamicibacter cryotolerans*	VanZ-1 RDD
CDE74027.1	*Clostridium sp. CAG:451*	VanZ-1 RDD
WP_184511288.1	*Terracoccus luteus*	VanZ-1 RDD
WP_185000952.1	*Crossiella cryophila*	VanZ-1 RDD; VanZ-1 RDD disordered
WP_010009360.1	*Loigolactobacillus coryniformis subsp. torquens*	VanZ-1 RDD
MCA0330909.1	*Actinomycetota bacterium*	VanZ-1 RDD
WP_024833738.1	*Ruminiclostridium josui JCM 17888*	VanZ-1 RDD
WP_144935539.1	*Paenibacillus sp. 320-W*	VanZ-1 RDD
WP_129009343.1	*Clostridium tetani*	VanZ-1 RDD
WP_051224752.1	*Pseudoclavibacter soli DSM 23366*	VanZ-1 RDD
WP_163652381.1	*Listeria sp. PSOL-1*	VanZ-1 RDD
WP_128805879.1		VanZ-1 RDD
WP_023438285.1	*Clostridium tetani*	VanZ-1 RDD
MBQ3428189.1	*Mogibacterium sp.*	VanZ-1 RDD
WP_061963154.1	*Demequina aurantiaca*	VanZ-1 RDD
TPF85921.1	*Bifidobacterium sp. UTCIF-37*	VanZ-1 RDD
WP_130005603.1		VanZ-1 RDD
WP_190917227.1	*Paenibacillus sabuli*	VanZ-1 RDD
HIT10573.1	*Candidatus Onthousia faecigallinarum*	VanZ-1 RDD
WP_213585581.1	*Paenibacillus sp. J2TS4*	VanZ-1 RDD
WP_089556147.1	*Latilactobacillus curvatus*	VanZ-1 RDD
MBR2524692.1	*Clostridiales bacterium*	VanZ-1 RDD
WP_131282896.1	*Alloscardovia theropitheci*	VanZ-1 RDD
CQR55486.1	*Paenibacillus riograndensis SBR5*	VanZ-1 RDD
HJA44050.1	*Candidatus Dorea stercoravium*	VanZ-1 RDD
QNG37160.1	*Geodermatophilaceae bacterium NBWT11*	VanZ-1 RDD
HIZ57803.1	*Candidatus Faecalibacterium gallistercoris*	VanZ-1 RDD
WP_062521652.1	*Paenibacillus jilunlii*	VanZ-1 RDD
AUD87319.1	*Bifidobacterium breve*	VanZ-1 RDD
MBK0867439.1	*Saccharopolyspora galaxeae*	VanZ-1 RDD
HIV25401.1	*Candidatus Scatomonas pullistercoris*	VanZ-1 RDD; ?; sugar-O-acetyl/acyltransf
MBZ0293522.1	*Anaerolineae bacterium*	VanZ-1 RDD
MCD1286097.1	*Brevibacterium sp. CCUG 69071*	VanZ-1 RDD
HIQ86275.1	*Candidatus Scatomorpha gallistercoris*	VanZ-1 RDD
CAM02204.1	*Saccharopolyspora erythraea NRRL 2338*	VanZ-1 RDD
WP_062465328.1	*Demequina soli*	VanZ-1 RDD
WP_062518020.1	*Demequina gelatinilytica*	VanZ-1 RDD
SDT65876.1	*Jiangella sp. DSM 45060*	VanZ-1 RDD
WP_062527122.1	*Demequina rhizosphaerae*	VanZ-1 RDD
HIS45292.1	*Candidatus Scatomorpha merdigallinarum*	VanZ-1 RDD
NRN65029.1	*Kibdelosporangium persicum*	VanZ-1 RDD
NJM31926.1	*Limnobacter sp.*	VanZ-1 RDD
HIS72865.1	*Candidatus Scatomorpha pullicola*	VanZ-1 RDD
WP_215660959.1	*Secundilactobacillus kimchicus*	VanZ-1 RDD
WP_171020610.1	*Anaerofustis stercorihominis*	VanZ-1 RDD
WP_061774870.1	*Pseudolactococcus raffinolactis*	VanZ-1 RDD
MBQ9119821.1	*Lachnospiraceae bacterium*	VanZ-1 RDD
WP_025360465.1	*Kutzneria albida DSM 43870*	VanZ-1 RDD
WP_154077559.1	*Allosaccharopolyspora coralli*	VanZ-1 RDD
MBX3314778.1	*Actinomycetota bacterium*	VanZ-1 RDD
WP_061564344.1	*Streptococcus australis*	VanZ-1 RDD
MBB4905620.1	*Actinophytocola algeriensis*	VanZ-1 RDD
MBI1351196.1	*Actinomycetales bacterium*	VanZ-1 RDD
WP_014215231.1	*Pediococcus claussenii*	VanZ-1 RDD
WP_008753940.1	*Lachnoanaerobaculum saburreum F0468*	VanZ-1 RDD
WP_184791977.1	*Phytomonospora endophytica*	VanZ-1 RDD
WP_003496416.1	*Clostridium sporogenes*	VanZ-1 RDD

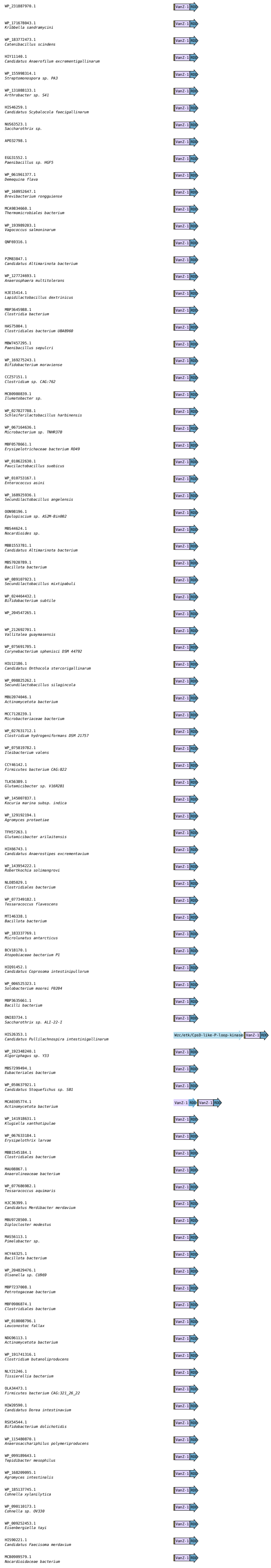
WP_231887970.1
VanZ-1 RDD
WP_171678043.1
Kribbella sandramycini
VanZ-1 RDD
WP_183772473.1
Catenibacillus scindens
VanZ-1 RDD
HIY11140.1
Candidatus Anaerofilum excrementigallinarum
VanZ-1 RDD
WP_155998314.1
Streptomonospora sp. PA3
VanZ-1 RDD
WP_131088133.1
Arthrobacter sp. S41
VanZ-1 RDD
HIS46259.1
Candidatus Scybalocola faecigallinarum
VanZ-1 RDD
NUS63523.1
Saccharothrix sp.
VanZ-1 RDD
APO32798.1
VanZ-1 RDD
EGG31552.1
Paenibacillus sp. HGF5
VanZ-1 RDD
WP_061961377.1
Demequina flava
VanZ-1 RDD
WP_160952647.1
Brevibacterium rongguiense
VanZ-1 RDD
MCA9834660.1
Thermomicrobiales bacterium
VanZ-1 RDD
WP_193989203.1
Vagococcus salmoninarum
VanZ-1 RDD
QNF69316.1
VanZ-1 RDD
PZM83847.1
Candidatus Altimarinota bacterium
VanZ-1 RDD
WP_127724693.1
Anaerosphaera multitolerans
VanZ-1 RDD
HJE15414.1
Lapidilactobacillus dextrinicus
VanZ-1 RDD
MBP3645988.1
Clostridia bacterium
VanZ-1 RDD
HAS75004.1
Clostridiales bacterium UBA8960
VanZ-1 RDD
MBW7457295.1
Paenibacillus sepulcri
VanZ-1 RDD
WP_169275243.1
Bifidobacterium moraviense
VanZ-1 RDD
CCZ57151.1
Clostridium sp. CAG:762
VanZ-1 RDD
MCB0980839.1
Ilumatobacter sp.
VanZ-1 RDD
WP_027827788.1
Schleiferilactobacillus harbinensis
VanZ-1 RDD
WP_067164636.1
Microbacterium sp. TNHR37B
VanZ-1 RDD
MBF0578661.1
Erysipelotrichaceae bacterium RD49
VanZ-1 RDD
WP_010622630.1
Paucilactobacillus suebicus
VanZ-1 RDD
WP_010753167.1
Enterococcus asini
VanZ-1 RDD
WP_168925936.1
Secundilactobacillus angelensis
VanZ-1 RDD
OON98196.1
Epulopiscium sp. AS2M-Bin002
VanZ-1 RDD
MBS44624.1
Nocardioides sp.
VanZ-1 RDD
MBB1553781.1
Candidatus Altimarinota bacterium
VanZ-1 RDD
MBS7020789.1
Bacillota bacterium
VanZ-1 RDD
WP_089107923.1
Secundilactobacillus mixtipabuli
VanZ-1 RDD
WP_024464432.1
Bifidobacterium subtile
VanZ-1 RDD
WP_204547265.1
VanZ-1 RDD
WP_212692701.1
Vallitalea guaymasensis
VanZ-1 RDD
WP_075691705.1
Corynebacterium sphenisci DSM 44792
VanZ-1 RDD
HIU12186.1
Candidatus Onthocola stercorigallinarum
VanZ-1 RDD
WP_098825262.1
Secundilactobacillus silagincola
VanZ-1 RDD
MBU2074046.1
Actinomycetota bacterium
VanZ-1 RDD
MCC7128239.1
Microbacteriaceae bacterium
VanZ-1 RDD
WP_027631712.1
Clostridium hydrogeniformans DSM 21757
VanZ-1 RDD
WP_075819782.1
Ileibacterium valens
VanZ-1 RDD
CCY46142.1
Firmicutes bacterium CAG:822
VanZ-1 RDD
TLK56389.1
Glutamicibacter sp. V16R2B1
VanZ-1 RDD
WP_145007837.1
Kocuria marina subsp. indica
VanZ-1 RDD
WP_129192194.1
Agromyces protaetiae
VanZ-1 RDD
TFH57263.1
Glutamicibacter arilaitensis
VanZ-1 RDD
HIX66743.1
Candidatus Anaerostipes excrementavium
VanZ-1 RDD
WP_143954222.1
Robertkochia solimangrovi
VanZ-1 RDD
NLO85029.1
Clostridiales bacterium
VanZ-1 RDD
WP_077349182.1
Tessaracoccus flavescens
VanZ-1 RDD
MTI46338.1
Bacillota bacterium
VanZ-1 RDD
WP_183337769.1
Microlunatus antarcticus
VanZ-1 RDD
BCV18170.1
Atopobiaceae bacterium P1
VanZ-1 RDD
HIQ91452.1
Candidatus Coprosoma intestinipullorum
VanZ-1 RDD
WP_006525323.1
Solobacterium moorei F0204
VanZ-1 RDD
MBP3635661.1
Bacilli bacterium
VanZ-1 RDD
ONI83734.1
Saccharothrix sp. ALI-22-I
VanZ-1 RDD
HIS26353.1
Candidatus Pullilachnospira intestinigallinarum
Wzc/etk/CpsD-like-P-loop-kinase
VanZ-1 RDD
WP_192348240.1
Algoriphagus sp. Y33
VanZ-1 RDD
MBS7299494.1
Eubacteriales bacterium
VanZ-1 RDD
WP_050637921.1
Candidatus Stoquefichus sp. SB1
VanZ-1 RDD
MCA0305774.1
Actinomycetota bacterium
VanZ-1 RDD
VanZ-1 RDD
WP_141918631.1
Klugiella xanthotipulae
VanZ-1 RDD
WP_067633184.1
Erysipelothrix larvae
VanZ-1 RDD
MBB1545184.1
Clostridiales bacterium
VanZ-1 RDD
MAU08867.1
Anaerolineaceae bacterium
VanZ-1 RDD
WP_077686982.1
Tessaracoccus aquimaris
VanZ-1 RDD
HJC36399.1
Candidatus Merdibacter merdavium
VanZ-1 RDD
MBU9728500.1
Diplocloster modestus
VanZ-1 RDD
MAS56113.1
Pimelobacter sp.
VanZ-1 RDD
HCY44325.1
Bacillota bacterium
VanZ-1 RDD
WP_204829476.1
Olsenella sp. CU969
VanZ-1 RDD
MBP7237008.1
Petrotogaceae bacterium
VanZ-1 RDD
MBF0986874.1
Clostridiales bacterium
VanZ-1 RDD
WP_010008796.1
Leuconostoc fallax
VanZ-1 RDD
NDG96113.1
Actinomycetota bacterium
VanZ-1 RDD
WP_191741316.1
Clostridium butanoliproducens
VanZ-1 RDD
NLY21246.1
Tissierellia bacterium
VanZ-1 RDD
OLA34473.1
Firmicutes bacterium CAG:321_26_22
VanZ-1 RDD
HIW20590.1
Candidatus Dorea intestinavium
VanZ-1 RDD
RSX54544.1
Bifidobacterium dolichotidis
VanZ-1 RDD
WP_115480870.1
Anaerosacchariphilus polymeriproducens
VanZ-1 RDD
WP_099189643.1
Tepidibacter mesophilus
VanZ-1 RDD
WP_168209095.1
Agromyces intestinalis
VanZ-1 RDD
WP_185137745.1
Cohnella xylanilytica
VanZ-1 RDD
WP_090110173.1
Cohnella sp. OV330
VanZ-1 RDD
WP_009252453.1
Eisenbergiella tayi
VanZ-1 RDD
HIS90221.1
Candidatus Faecisoma merdavium
VanZ-1 RDD
MCB0909579.1
Nocardioidaceae bacterium
VanZ-1 RDD

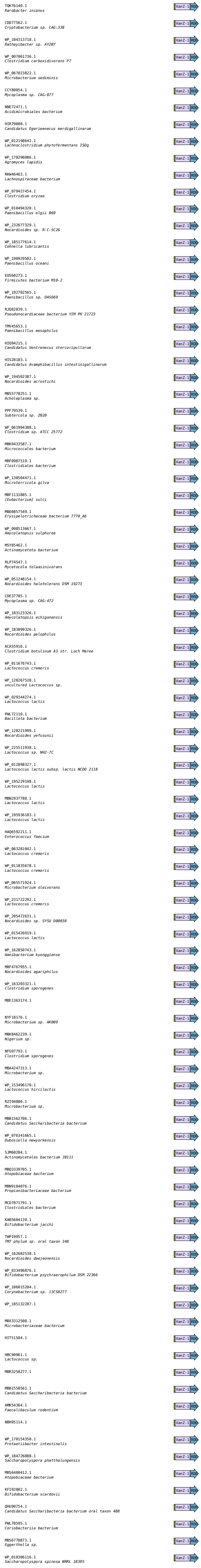
TQK76140.1 *Rarobacter incanus* VanZ-1 RDD
CDD77562.1 *Cryptobacterium sp. CAG:338* VanZ-1 RDD
WP_104313718.1 *Rathayibacter sp. AY2B7* VanZ-1 RDD
WP_007061736.1 *Clostridium carboxidivorans P7* VanZ-1 RDD
WP_067023822.1 *Microbacterium sediminis* VanZ-1 RDD
CCY80054.1 *Mycoplasma sp. CAG:877* VanZ-1 RDD
NNE72471.1 *Acidimicrobiales bacterium* VanZ-1 RDD
HIR79008.1 *Candidatus Egerieenecus merdigallinarum* VanZ-1 RDD
WP_012198642.1 *Lachnoclostridium phytofermentans ISDg* VanZ-1 RDD
WP_170296086.1 *Agromyces lapidis* VanZ-1 RDD
RKW46463.1 *Lachnospiraceae bacterium* VanZ-1 RDD
WP_079427454.1 *Clostridium oryzae* VanZ-1 RDD
WP_010494320.1 *Paenibacillus elgii B69* VanZ-1 RDD
WP_232677329.1 *Nocardioides sp. R-C-SC26* VanZ-1 RDD
WP_185177614.1 *Cohnella lubricantis* VanZ-1 RDD
WP_190929502.1 *Paenibacillus oceani* VanZ-1 RDD
EOS60273.1 *Firmicutes bacterium M10-2* VanZ-1 RDD
WP_192702565.1 *Paenibacillus sp. OAS669* VanZ-1 RDD
RJQ82839.1 *Pseudonocardiaceae bacterium YIM PH 21723* VanZ-1 RDD
TMV45653.1 *Paenibacillus mesophilus* VanZ-1 RDD
HIQ94215.1 *Candidatus Ventrenecus stercoripullorum* VanZ-1 RDD
HIS28183.1 *Candidatus Avamphibacillus intestinigallinarum* VanZ-1 RDD
WP_194502387.1 *Nocardioides acrostichi* VanZ-1 RDD
MBS5778251.1 *Acholeplasma sp.* VanZ-1 RDD
PPF79539.1 *Subtercola sp. Z020* VanZ-1 RDD
WP_061994388.1 *Clostridium sp. ATCC 25772* VanZ-1 RDD
MBK9433587.1 *Micrococcales bacterium* VanZ-1 RDD
MBF0987519.1 *Clostridiales bacterium* VanZ-1 RDD
WP_130504471.1 *Microterricola gilva* VanZ-1 RDD
MBF1131885.1 *[Eubacterium] sulci* VanZ-1 RDD
MBD0857549.1 *Erysipelotrichaceae bacterium 7770_A6* VanZ-1 RDD
WP_098513667.1 *Amycolatopsis sulphurea* VanZ-1 RDD
MSY85462.1 *Actinomycetota bacterium* VanZ-1 RDD
RLP74547.1 *Mycetocola tolaasinivorans* VanZ-1 RDD
WP_051248154.1 *Nocardioides halotolerans DSM 19273* VanZ-1 RDD
CDE37785.1 *Mycoplasma sp. CAG:472* VanZ-1 RDD
WP_183123326.1 *Amycolatopsis echigonensis* VanZ-1 RDD
WP_183099326.1 *Nocardioides pelophilus* VanZ-1 RDD
ACA55916.1 *Clostridium botulinum A3 str. Loch Maree* VanZ-1 RDD
WP_011676743.1 *Lactococcus cremoris* VanZ-1 RDD
WP_128267528.1 *uncultured Lactococcus sp.* VanZ-1 RDD
WP_029344274.1 *Lactococcus lactis* VanZ-1 RDD
PWL72110.1 *Bacillota bacterium* VanZ-1 RDD
WP_128221999.1 *Nocardioides yefusunii* VanZ-1 RDD
WP_225511938.1 *Lactococcus sp. NH2-7C* VanZ-1 RDD
WP_012898327.1 *Lactococcus lactis subsp. lactis NCDO 2118* VanZ-1 RDD
WP_195229198.1 *Lactococcus lactis* VanZ-1 RDD
MBN2937780.1 *Lactococcus lactis* VanZ-1 RDD
WP_195936183.1 *Lactococcus lactis* VanZ-1 RDD
HAQ6592211.1 *Enterococcus faecium* VanZ-1 RDD
WP_063281042.1 *Lactococcus cremoris* VanZ-1 RDD
WP_011835678.1 *Lactococcus cremoris* VanZ-1 RDD
WP_065571924.1 *Microbacterium oleivorans* VanZ-1 RDD
WP_231722292.1 *Lactococcus cremoris* VanZ-1 RDD
WP_205472631.1 *Nocardioides sp. SYSU D00038* VanZ-1 RDD
WP_015426919.1 *Lactococcus lactis* VanZ-1 RDD
WP_162850743.1 *Amnibacterium kyonggiense* VanZ-1 RDD
MBF4767955.1 *Nocardioides agariphilus* VanZ-1 RDD
WP_163203321.1 *Clostridium sporogenes* VanZ-1 RDD
MBE1363174.1 VanZ-1 RDD
NYF18176.1 *Microbacterium sp. AK009* VanZ-1 RDD
MBK8462239.1 *Nigerium sp.* VanZ-1 RDD
NFG97793.1 *Clostridium sporogenes* VanZ-1 RDD
MBA4247313.1 *Microbacterium sp.* VanZ-1 RDD
WP_153496170.1 *Lactococcus hircilactis* VanZ-1 RDD
RZI94880.1 *Microbacterium sp.* VanZ-1 RDD
MBB1562706.1 *Candidatus Saccharibacteria bacterium* VanZ-1 RDD
WP_076341665.1 *Dubosiella newyorkensis* VanZ-1 RDD
SJM60284.1 *Actinomycetales bacterium JB111* VanZ-1 RDD
MBQ3339705.1 *Atopobiaceae bacterium* VanZ-1 RDD
MBN9104076.1 *Propionibacteriaceae bacterium* VanZ-1 RDD
MCD7871791.1 *Clostridiales bacterium* VanZ-1 RDD
KAB5604139.1 *Bifidobacterium jacchi* VanZ-1 RDD
TWP19957.1 *TM7 phylum sp. oral taxon 348* VanZ-1 RDD
WP_162602538.1 *Nocardioides daejeonensis* VanZ-1 RDD
WP_033496876.1 *Bifidobacterium psychraerophilum DSM 22366* VanZ-1 RDD
WP_106015204.1 *Corynebacterium sp. 13CS0277* VanZ-1 RDD
WP_185132287.1 VanZ-1 RDD
MBX3312508.1 *Microbacteriaceae bacterium* VanZ-1 RDD
HIT51504.1 VanZ-1 RDD
HBC90961.1 *Lactococcus sp.* VanZ-1 RDD
MBR3258277.1 VanZ-1 RDD
MBB1558561.1 *Candidatus Saccharibacteria bacterium* VanZ-1 RDD
AMK54364.1 *Faecalibaculum rodentium* VanZ-1 RDD
NBH95114.1 VanZ-1 RDD
WP_170154350.1 *Protaetiibacter intestinalis* VanZ-1 RDD
WP_184726888.1 *Saccharopolyspora phatthalungensis* VanZ-1 RDD
MBS6400412.1 *Atopobiaceae bacterium* VanZ-1 RDD
KFI92002.1 *Bifidobacterium scardovii* VanZ-1 RDD
QHU90754.1 *Candidatus Saccharibacteria bacterium oral taxon 488* VanZ-1 RDD
PWL78595.1 *Coriobacteriia bacterium* VanZ-1 RDD
MBS6778873.1 *Eggerthella sp.* VanZ-1 RDD
WP_010306116.1 *Saccharopolyspora spinosa NRRL 18395* VanZ-1 RDD

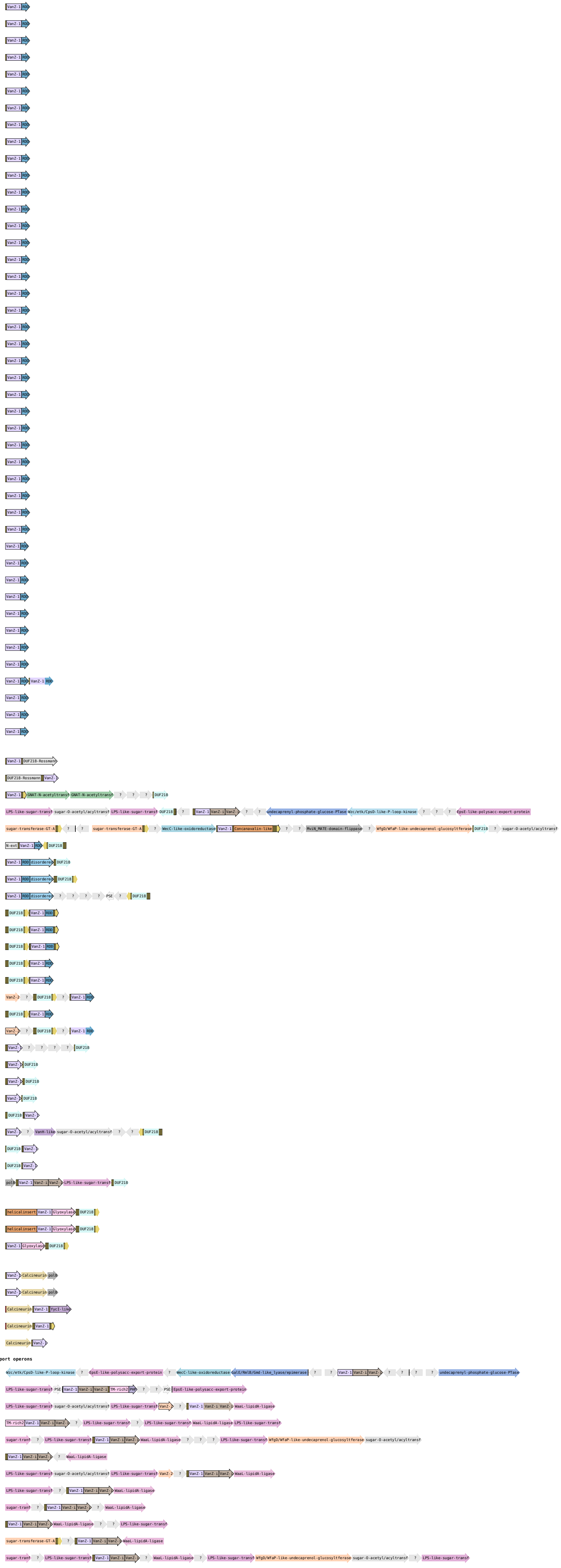

MBY0689619.1
Microbacterium marinilacus

HIS41050.1
Candidatus Aphodovivens avistercoris

RQP11328.1
Microbacteriaceae bacterium

HJF64676.1
Slackia equolifaciens

MBN9140130.1
Micrococcales bacterium

WP_204442204.1
Bifidobacterium adolescentis ATCC 15703

WP_065467454.1
Bifidobacterium dentium

KFI86913.1
Bifidobacterium pullorum subsp. saeculare DSM 6531 = LMG 14934

WP_204570503.1

ALG86837.1
Gordonia phthalatica

MBS5401178.1
Bifidobacterium sp.

HIY52300.1
Candidatus Olsenella avicola

WP_204584778.1
Collinsella intestinalis

WP_204672572.1
Enorma phocaeensis

WP_087410084.1
Collinsella sp. An2

WP_102371591.1
Enorma phocaeensis

WP_204562316.1
Collinsella tanakaei

HIU05966.1
Candidatus Coprousia avicola

WP_193927788.1
Saccharopolyspora montiporae

WP_087315349.1
Olsenella sp. An293

RYX78664.1
bacterium

PWM15911.1
Collinsella tanakaei

WP_117679610.1
Collinsella tanakaei

HIR48681.1
Candidatus Faecimonas gallistercoris

SNT63038.1
Streptosporangium subroseum

MCB0883978.1
Propionibacteriaceae bacterium

WP_025083727.1
Latilactobacillus fuchuensis DSM 14340 = JCM 11249

MBR4208234.1
Lachnospiraceae bacterium

AFM71313.1
Enterococcus hirae ATCC 9790

WP_052118247.1
Bifidobacterium bohemicum

WP_223876881.1
Lactiplantibacillus daoliensis

OJG11733.1
Enterococcus aquimarinus

GEN86049.1
Oceanobacillus sojae

EOT29314.1
Enterococcus saccharolyticus subsp. saccharolyticus ATCC 43076

MBO9706213.1
Arthrobacter sp.

MBR4122738.1
Erysipelotrichaceae bacterium

MBB1563312.1
Candidatus Saccharibacteria bacterium

HIR70968.1
Candidatus Pullilachnospira gallistercoris

WP_051876488.1

HIR43153.1
Candidatus Aphodovivens avicola

MCA0305775.1
Actinomycetota bacterium

WP_162231421.1
Secundilactobacillus similis DSM 23365 = JCM 2765

MCA9725900.1
Kurthia sp.

HAB66149.1
Bacillota bacterium

##;other DUF218 fusions or associations

MBO4731175.1
Spirochaetaceae bacterium

NQU25988.1
Candidatus Nealsonbacteria bacterium

MBS5124791.1
Clostridium sp.

MBS1817549.1
Acidobacteriota bacterium

HEE46848.1

RRD07365.1
Arachnia propionica

WP_060536007.1
Paenibacillus bovis

HAT54800.1
Lactobacillus sp.

WP_225436015.1
Lacticaseibacillus paracasei

RKT81959.1
Saccharopolyspora antimicrobica

WP_164702743.1
Modestobacter sp. KNN46-3

MBM7402094.1

PZA23261.1
Modestobacter versicolor

WP_051596650.1
Curtobacterium sp. UNCCL17

WP_079492338.1
Maledivibacter halophilus

WP_231849993.1
Saccharopolyspora erythraea

WP_079492344.1
Maledivibacter halophilus

MBQ2285869.1
Clostridia bacterium

WP_143094066.1
Caldicoprobacter faecalis

MBE5917859.1
Pseudobutyrivibrio ruminis

MBQ1172124.1
Lachnospiraceae bacterium

MBF0187352.1
Magnetococcales bacterium

MBR3082731.1
Clostridiales bacterium

MBI4444451.1
Acidobacteriota bacterium

WP_119402142.1

WP_145345480.1
Rosistilla ulvae

#;additional fusion/association with Glyoxylase domain

MBR3494663.1
Clostridia bacterium

MBR6953781.1
Clostridia bacterium

MBR5344543.1
Clostridia bacterium

####;calcinuerin associations

MBQ5347682.1
Ruminococcus sp.

MBR2280672.1
Ruminococcus sp.

MBQ9718699.1
Clostridia bacterium

WP_195159510.1
Clostridium sp. 'deep sea'

MBP3348470.1
Clostridia bacterium

####;fusion to VanZ-inactive repeats. Sugar synthesis/transport operons

WP_101522342.1
Kineobactrum sediminis

WP_163111583.1
Alteromonas hispanica

MCC7009129.1
Acidobacteriota bacterium

AGA29604.1
Singulisphaera acidiphila DSM 18658

MBX9789747.1
Pirellulales bacterium

RFC32324.1
Candidatus Nitrotoga sp. SPKER

MCC7009131.1
Acidobacteriota bacterium

NJM02122.1
Desulfobacula sp.

MBI3463910.1
Planctomycetota bacterium

OQW88761.1
Rhodoferax ferrireducens

MCC6531370.1
Burkholderiales bacterium

MBX7074758.1
Pirellulales bacterium

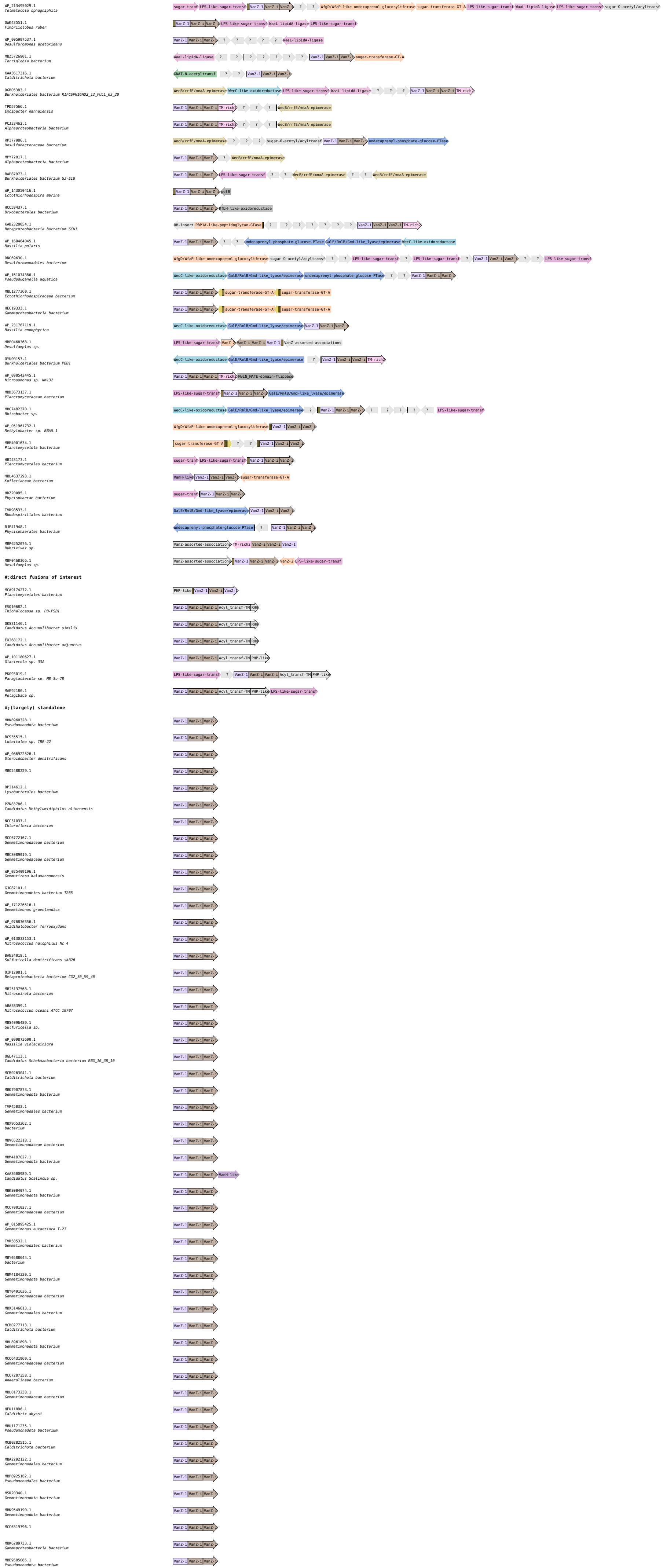
WP_213495029.1
Telmatocola sphagniphila
OWK43551.1
Fimbriiglobus ruber
WP_005997537.1
Desulfuromonas acetoxidans
MBZ5726901.1
Terriglobia bacterium
KAA3617316.1
Calditrichota bacterium
OGB05383.1
Burkholderiales bacterium RIFCSPHIGHO2_12_FULL_63_20
TPD57566.1
Emcibacter nanhaiensis
PCJ33462.1
Alphaproteobacteria bacterium
RPI77986.1
Desulfobacteraceae bacterium
MPY72017.1
Alphaproteobacteria bacterium
BAP87973.1
Burkholderiales bacterium GJ-E10
WP_143050416.1
Ectothiorhodospira marina
HCC59437.1
Bryobacterales bacterium
KAB2320054.1
Betaproteobacteria bacterium SCN1
WP_169464045.1
Massilia polaris
RNC69630.1
Desulfuromonadales bacterium
WP_161074380.1
Pseudoduganella aquatica
MBL1277360.1
Ectothiorhodospiraceae bacterium
HEC19333.1
Gammaproteobacteria bacterium
WP_231767119.1
Massilia endophytica
MBF0468368.1
Desulfamplus sp.
OYU00153.1
Burkholderiales bacterium PBB1
WP_090542445.1
Nitrosomonas sp. Nm132
MBD3673137.1
Planctomycetaceae bacterium
MBC7482370.1
Rhizobacter sp.
WP_051961732.1
Methylobacter sp. BBA5.1
MBM4001634.1
Planctomycetota bacterium
HBI43173.1
Planctomycetales bacterium
MBL4637293.1
Kofleriaceae bacterium
HDZ20095.1
Phycisphaerae bacterium
TVR98533.1
Rhodospirillales bacterium
RJP41948.1
Phycisphaerales bacterium
MBP6252076.1
Rubrivivax sp.
MBF0468366.1
Desulfamplus sp.
#;direct fusions of interest
MCA9174272.1
Planctomycetales bacterium
ESQ10682.1
Thiohalocapsa sp. PB-PSB1
QKS31146.1
Candidatus Accumulibacter similis
EXI68172.1
Candidatus Accumulibacter adjunctus
WP_101180627.1
Glaciecola sp. 33A
PKG93019.1
Paraglaciecola sp. MB-3u-78
MAE92180.1
Pelagibaca sp.
#;(largely) standalone
MBK8960328.1
Pseudomonadota bacterium
BCS35515.1
Luteitalea sp. TBR-22
WP_066922526.1
Steroidobacter denitrificans
MBO2488229.1
RPI14612.1
Lysobacterales bacterium
PZN83786.1
Candidatus Methylumidiphilus alinenensis
NCC31037.1
Chloroflexia bacterium
MCC6772167.1
Gemmatimonadaceae bacterium
MBC8089019.1
Gemmatimonadaceae bacterium
WP_025409196.1
Gemmatirosa kalamazoonensis
GJG87101.1
Gemmatimonadetes bacterium T265
WP_171226516.1
Gemmatimonas groenlandica
WP_076836356.1
Acidihalobacter ferrooxydans
WP_013033153.1
Nitrosococcus halophilus Nc 4
BAN34018.1
Sulfuricella denitrificans skB26
OIP12981.1
Betaproteobacteria bacterium CG2_30_59_46
MBI5137568.1
Nitrospirota bacterium
ABA58399.1
Nitrosococcus oceani ATCC 19707
MBS4096489.1
Sulfuricella sp.
WP_099873600.1
Massilia violaceinigra
OGL47113.1
Candidatus Schekmanbacteria bacterium RBG_16_38_10
MCB0263041.1
Calditrichota bacterium
MBK7907873.1
Gemmatimonadota bacterium
TVP45033.1
Gemmatimonadales bacterium
MBX9653362.1
bacterium
MBV6522318.1
Gemmatimonadaceae bacterium
MBM4187027.1
Gemmatimonadota bacterium
KAA3600989.1
Candidatus Scalindua sp.
MBK8004074.1
Gemmatimonadota bacterium
MCC7001027.1
Gemmatimonadaceae bacterium
WP_015895425.1
Gemmatimonas aurantiaca T-27
TVR58532.1
Gemmatimonadales bacterium
MBY0588644.1
bacterium
MBM4184320.1
Gemmatimonadota bacterium
MBY0491636.1
Gemmatimonadaceae bacterium
MBX3146613.1
Gemmatimonadales bacterium
MCB0277713.1
Calditrichota bacterium
MBL8961898.1
Gemmatimonadota bacterium
MCC6431969.1
Gemmatimonadaceae bacterium
MCC7207358.1
Anaerolineae bacterium
MBL0173238.1
Gemmatimonadaceae bacterium
HED11896.1
Caldithrix abyssi
MBU1171235.1
Pseudomonadota bacterium
MCB0282515.1
Calditrichota bacterium
MBA2292122.1
Gemmatimonadales bacterium
MBP8925182.1
Pseudomonadales bacterium
MSR20340.1
Gemmatimonadota bacterium
MBK9549190.1
Gemmatimonadota bacterium
MCC6319796.1
MBK6289733.1
Gammaproteobacteria bacterium
MBE9505065.1
Pseudomonadota bacterium

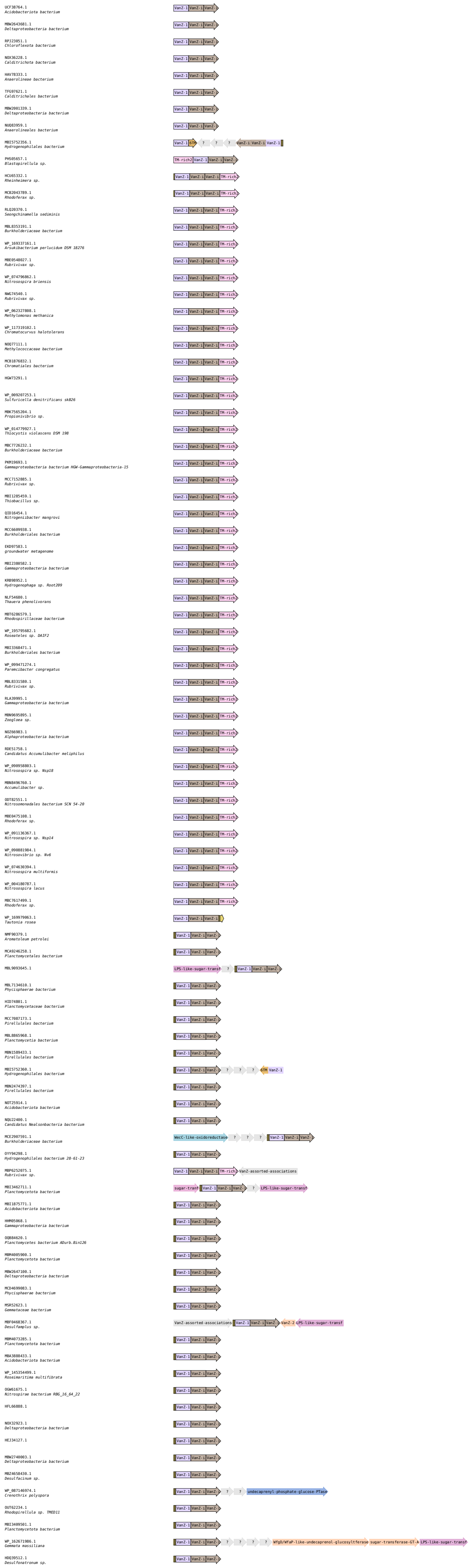
UCF38764.1
Acidobacteriota bacterium
MBW2643681.1
Deltaproteobacteria bacterium
RPJ23051.1
Chloroflexota bacterium
NOX36228.1
Calditrichota bacterium
HAV78333.1
Anaerolineae bacterium
TFG97621.1
Calditrichales bacterium
MBW2001339.1
Deltaproteobacteria bacterium
NUQ83959.1
Anaerolineales bacterium
MBI5752356.1
Hydrogenophilales bacterium
PHS05657.1
Blastopirellula sp.
HCU65332.1
Rheinheimera sp.
MCB2043789.1
Rhodoferax sp.
RLQ20370.1
Seongchinamella sediminis
MBL8353191.1
Burkholderiaceae bacterium
WP_169337161.1
Arsukibacterium perlucidum DSM 18276
MBE0548027.1
Rubrivivax sp.
WP_074796862.1
Nitrosospira briensis
NWG74540.1
Rubrivivax sp.
WP_062327808.1
Methylomonas methanica
WP_117319102.1
Chromatocurvus halotolerans
NOQ77111.1
Methylococcaceae bacterium
MCB1876832.1
Chromatiales bacterium
HGW73291.1
WP_009207253.1
Sulfuricella denitrificans skB26
MBK7565204.1
Propionivibrio sp.
WP_014779927.1
Thiocystis violascens DSM 198
MBC7726232.1
Burkholderiaceae bacterium
PKM19693.1
Gammaproteobacteria bacterium HGW-Gammaproteobacteria-15
MCC7152885.1
Rubrivivax sp.
MBI1285459.1
Thiobacillus sp.
QID16454.1
Nitrogeniibacter mangrovi
MCC6609938.1
Burkholderiales bacterium
EKD97583.1
groundwater metagenome
MBI2380582.1
Gammaproteobacteria bacterium
KRB98952.1
Hydrogenophaga sp. Root209
NLF54680.1
Thauera phenolivorans
MBT6286579.1
Rhodospirillaceae bacterium
WP_195795682.1
Roseateles sp. DAIF2
MBI3368471.1
Burkholderiales bacterium
WP_099471274.1
Paremcibacter congregatus
MBL8331580.1
Rubrivivax sp.
RLA39995.1
Gammaproteobacteria bacterium
MBN9695895.1
Zoogloea sp.
NOZ66983.1
Alphaproteobacteria bacterium
RDE51758.1
Candidatus Accumulibacter meliphilus
WP_090958803.1
Nitrosospira sp. Nsp18
MBN8496760.1
Accumulibacter sp.
ODT82551.1
Nitrosomonadales bacterium SCN 54-20
MBE0475108.1
Rhodoferax sp.
WP_091136367.1
Nitrosospira sp. Nsp14
WP_090881984.1
Nitrosovibrio sp. Nv6
WP_074630394.1
Nitrosospira multiformis
WP_004180787.1
Nitrosospira lacus
MBC7617499.1
Rhodoferax sp.
WP_169979063.1
Tautonia rosea
NMF90379.1
Aromatoleum petrolei
MCA9246258.1
Planctomycetales bacterium
MBL9093645.1
MBL7134610.1
Phycisphaerae bacterium
HID74801.1
Planctomycetaceae bacterium
MCC7087173.1
Pirellulales bacterium
MBL8865968.1
Planctomycetia bacterium
MBN1589433.1
Pirellulales bacterium
MBI5752360.1
Hydrogenophilales bacterium
MBN2474397.1
Pirellulales bacterium
NOT25914.1
Acidobacteriota bacterium
NQU22400.1
Candidatus Nealsonbacteria bacterium
MCE2907591.1
Burkholderiaceae bacterium
OYY94298.1
Hydrogenophilales bacterium 28-61-23
MBP6252075.1
Rubrivivax sp.
MBI3462711.1
Planctomycetota bacterium
MBI1875771.1
Acidobacteriota bacterium
HHM05068.1
Gammaproteobacteria bacterium
OQB84620.1
Planctomycetes bacterium ADurb.Bin126
MBM4005900.1
Planctomycetota bacterium
MBW2647100.1
Deltaproteobacteria bacterium
MCD4699083.1
Phycisphaerae bacterium
MSR52623.1
Gemmataceae bacterium
MBF0468367.1
Desulfamplus sp.
MBM4073285.1
Planctomycetota bacterium
MBA3888433.1
Acidobacteriota bacterium
WP_145354499.1
Roseimaritima multifibrata
OGW61675.1
Nitrospirae bacterium RBG_16_64_22
HFL66888.1
NOX32923.1
Deltaproteobacteria bacterium
HEJ34127.1
MBW2740003.1
Deltaproteobacteria bacterium
MBZ4658430.1
Desulfacinum sp.
WP_087146974.1
Crenothrix polyspora
OUT62234.1
Rhodopirellula sp. TMED11
MBI3409501.1
Planctomycetota bacterium
WP_162671986.1
Gemmata massiliana
HDQ39512.1
Desulfonatronum sp.

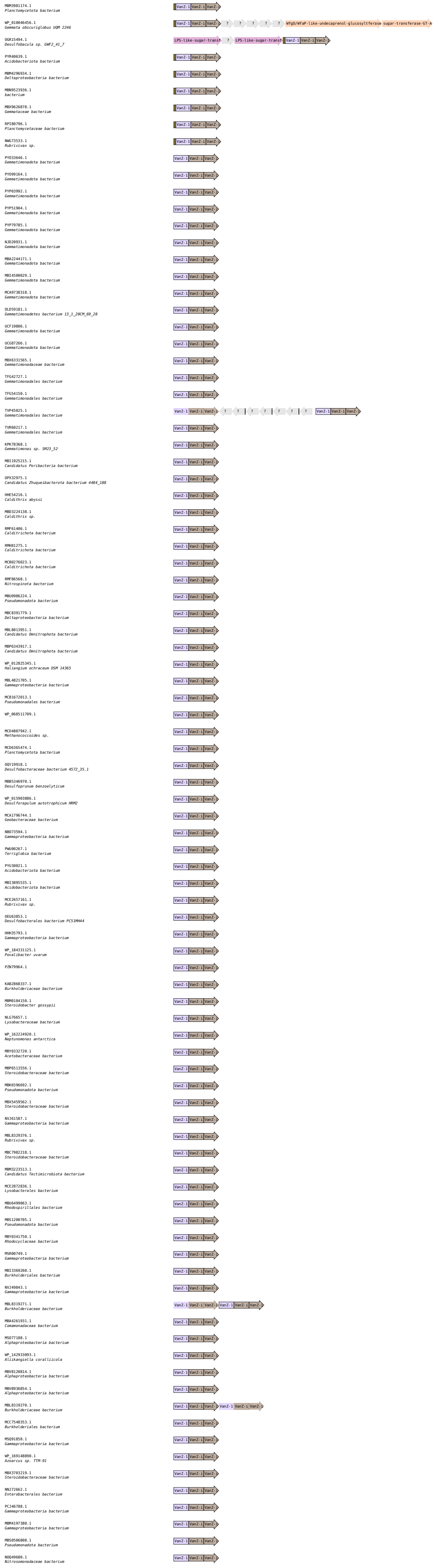
MBM3981174.1
Planctomycetota bacterium
WP_010046456.1
Gemmata obscuriglobus UQM 2246
VanZ-1 VanZ-i VanZ-i ? ? ? ? ? WfgD/WfaP-like-undecaprenol-glucosyltferase sugar-transferase-GT-A
OGR15494.1
Desulfobacula sp. GWF2_41_7
LPS-like-sugar-transf ? LPS-like-sugar-transf VanZ-1 VanZ-i VanZ-i
PYR40639.1
Acidobacteriota bacterium
MBM4296934.1
Deltaproteobacteria bacterium
MBN9523936.1
bacterium
MBX9626878.1
Gemmataceae bacterium
RPI80796.1
Planctomycetaceae bacterium
NWG73533.1
Rubrivivax sp.
PYO33646.1
Gemmatimonadota bacterium
PYO99164.1
Gemmatimonadota bacterium
PYP03992.1
Gemmatimonadota bacterium
PYP51904.1
Gemmatimonadota bacterium
PYP79785.1
Gemmatimonadota bacterium
NJD20931.1
Gemmatimonadota bacterium
MBA2244171.1
Gemmatimonadota bacterium
MBI4500029.1
Gemmatimonadota bacterium
MCA9738318.1
Gemmatimonadota bacterium
OLD59181.1
Gemmatimonadetes bacterium 13_1_20CM_69_28
UCF19806.1
Gemmatimonadota bacterium
UCG87266.1
Gemmatimonadota bacterium
MBX6331565.1
Gemmatimonadaceae bacterium
TFG42727.1
Gemmatimonadales bacterium
TFG54150.1
Gemmatimonadales bacterium
TVP45025.1
Gemmatimonadales bacterium
VanZ-1 VanZ-i VanZ-i ? ? ? ? ? ? ? VanZ-1 VanZ-i VanZ-i
TVR60217.1
Gemmatimonadales bacterium
KPK78368.1
Gemmatimonas sp. SM23_52
MBI1925215.1
Candidatus Poribacteria bacterium
OPX32975.1
Candidatus Zhuqueibacterota bacterium 4484_188
HHE54216.1
Caldithrix abyssi
MBD3224138.1
Caldithrix sp.
RMF61406.1
Calditrichota bacterium
RMH81275.1
Calditrichota bacterium
MCB0276023.1
Calditrichota bacterium
RMF86568.1
Nitrospinota bacterium
MBU0986224.1
Pseudomonadota bacterium
MBC8391779.1
Deltaproteobacteria bacterium
MBL8013951.1
Candidatus Omnitrophota bacterium
MBP6343917.1
Candidatus Omnitrophota bacterium
WP_012825345.1
Haliangium ochraceum DSM 14365
MBL4821705.1
Gammaproteobacteria bacterium
MCB1672013.1
Pseudomonadales bacterium
WP_068511709.1
MCD4807942.1
Methanococcoides sp.
MCD6365474.1
Planctomycetota bacterium
OQY19918.1
Desulfobacteraceae bacterium 4572_35.1
MBB5346970.1
Desulfoprunum benzoelyticum
WP_015903886.1
Desulforapulum autotrophicum HRM2
MCA1796744.1
Geobacteraceae bacterium
NBO73594.1
Gammaproteobacteria bacterium
PWU00267.1
Terriglobia bacterium
PYU30021.1
Acidobacteriota bacterium
MBI3895535.1
Acidobacteriota bacterium
MCE2657161.1
Rubrivivax sp.
OEU63853.1
Desulfobacterales bacterium PC51MH44
HHH35793.1
Gammaproteobacteria bacterium
WP_184331125.1
Povalibacter uvarum
PZN79964.1
KAB2868337.1
Burkholderiaceae bacterium
MBM0104150.1
Steroidobacter gossypii
NLG76657.1
Lysobacteraceae bacterium
WP_162224920.1
Neptunomonas antarctica
MBY0332720.1
Acetobacteraceae bacterium
MBP6513556.1
Steroidobacteraceae bacterium
MBK6596692.1
Pseudomonadota bacterium
MBX5459562.1
Steroidobacteraceae bacterium
NVJ61587.1
Gammaproteobacteria bacterium
MBL8329376.1
Rubrivivax sp.
MBC7982218.1
Steroidobacteraceae bacterium
MBM3223513.1
Candidatus Tectimicrobiota bacterium
MCE2872836.1
Lysobacterales bacterium
MBU6499063.1
Rhodospirillales bacterium
MBS1200705.1
Pseudomonadota bacterium
MBY0341750.1
Rhodocyclaceae bacterium
MSR00749.1
Gammaproteobacteria bacterium
MBI3369260.1
Burkholderiales bacterium
NVJ49843.1
Gammaproteobacteria bacterium
MBL8319271.1
Burkholderiaceae bacterium
VanZ-1 VanZ-i VanZ-i VanZ-1 VanZ-i VanZ-i
MBA4261931.1
Comamonadaceae bacterium
MSO77108.1
Alphaproteobacteria bacterium
WP_142933093.1
Aliikangiella coralliicola
MBV8120814.1
Alphaproteobacteria bacterium
MBV8936854.1
Alphaproteobacteria bacterium
MBL8319270.1
Burkholderiaceae bacterium
VanZ-1 VanZ-i VanZ-i VanZ-1 VanZ-i VanZ-i
MCC7548353.1
Burkholderiales bacterium
MSQ91858.1
Gammaproteobacteria bacterium
WP_169148890.1
Azoarcus sp. TTM-91
MBX3703219.1
Steroidobacteraceae bacterium
NNJ72662.1
Enterobacterales bacterium
PCJ46788.1
Gammaproteobacteria bacterium
MBM4197380.1
Gammaproteobacteria bacterium
MBS0506808.1
Pseudomonadota bacterium
NOQ49680.1
Nitrosomonadaceae bacterium
VanZ-1 VanZ-i VanZ-i

MBL8838770.1
Alphaproteobacteria bacterium

MBP9693524.1
Alphaproteobacteria bacterium

MBS0392086.1
Pseudomonadota bacterium

MBC8165977.1
Bryobacteraceae bacterium

MAT91606.1
Halioglobus sp.

WP_196139368.1
Aliikangiella sp. G2MR2-5

MCC6364535.1
Bryobacterales bacterium

MBP6105955.1
Steroidobacteraceae bacterium

WP_120098335.1
Salinisphaera sp. Q1T1-3

MBH03251.1
Lysobacterales bacterium

WP_168986616.1
Azoarcus taiwanensis

NDJ12731.1
Terriglobia bacterium

WP_232226001.1
Salinisphaera hydrothermalis C41B8

MCC6719065.1
Acetobacteraceae bacterium

WP_154246679.1
Kangiella sp. HZ709

NVJ68553.1
Gammaproteobacteria bacterium

NVK22033.1
Kangiellaceae bacterium

MBV6425193.1

MCC6782299.1
Planctomycetota bacterium

HHH47452.1
Gammaproteobacteria bacterium

WP_068636885.1
Thauera butanivorans NBRC 103042

MBS1857298.1
Acidobacteriota bacterium

MBZ5605842.1
Terriglobia bacterium

MBE7417236.1
Ideonella sp.

MCB1625099.1
Pseudomonadales bacterium

NJO13179.1
Gammaproteobacteria bacterium

UCC30749.1
Phycisphaerales bacterium

UCE60011.1
Phycisphaerales bacterium

UCF32977.1
Phycisphaerales bacterium

RME39885.1
Planctomycetota bacterium

MBI4716307.1
Planctomycetota bacterium

MBU0717700.1
Planctomycetota bacterium

AMY12512.1
Luteitalea pratensis

MBX3590924.1
Burkholderiaceae bacterium

RIK97786.1
Burkholderiales bacterium

NLD68257.1
Limnobacter sp.

WP_183967288.1
Quisquiliibacterium transsilvanicum

WP_168711020.1
Ningiella ruwaisensis

MBC7564479.1
Gemmatimonadaceae bacterium

MBC7843644.1
Gemmatimonadaceae bacterium

###;WaaL-like-ligase fusions (Wzy-like fusions). Association with polysaccharide-modifiers

MBN2702320.1
Methylothermaceae bacterium

MBI3345428.1
Gammaproteobacteria bacterium

MBI3345425.1
Gammaproteobacteria bacterium

MBX9965073.1
Burkholderiales bacterium

WP_153110270.1
Propionivibrio limicola

MBK6472898.1
Betaproteobacteria bacterium

PPD05220.1
Methylobacter sp.

RIZ64889.1
Methylococcales bacterium

NKJ16862.1

MBI2798870.1
Gammaproteobacteria bacterium

PCJ37922.1
Cellvibrionales bacterium

QHE76703.1
Hydrogenophaga sp. PBL-H3

OJY08070.1
Nitrosospira sp. 56-18

RIX45493.1
Rhodocyclales bacterium GT-UBC

HEC16060.1
Sedimenticola sp.

#;standalone

MBP6225634.1
Rhizobacter sp.

TVR57199.1
Candidatus Competibacteraceae bacterium

HCZ15237.1
Accumulibacter sp.

WP_058554639.1
Thiohalocapsa sp. ML1

WP_100918587.1
Candidatus Thiodictyon syntrophicum

RFC34179.1
Candidatus Nitrotoga sp. MKT

SFM65602.1
Nitrosomonas nitrosa

MBT9569027.1
Thiobacillus sp.

TVR57198.1
Candidatus Competibacteraceae bacterium

WP_011627860.1
Alkalilimnicola ehrlichii

MBH1975865.1
Rhodocyclales bacterium

MCB1889385.1
Rhodocyclaceae bacterium

MBI3155038.1
Burkholderiales bacterium

RMF07751.1
Alphaproteobacteria bacterium

MCB1921233.1
Candidatus Competibacteraceae bacterium

TLD47610.1
Accumulibacter sp.

MBU2570302.1
Gammaproteobacteria bacterium

WP_031432751.1
Methylomarinum vadi

KFB71463.1
Candidatus Accumulibacter phosphatis

MBS3964743.1
Methylomonas sp.

WP_013820477.1
Methylomonas methanica MC09

WP_161984295.1
Sulfuriferula plumbiphila

WP_196436118.1
Methylomonas sp. LL1

TAK65173.1
Methylobacter sp.

MBK5913836.1
Rhodocyclus purpureus

WP_051537544.1
Nitrosomonas cryotolerans ATCC 49181

WP_077280096.1
Thioalkalivibrio denitrificans

WP_140910626.1
Methylomonas koyamae

MBI3708769.1
Pseudomonadota bacterium

MCC6852357.1
Rubrivivax sp.

MBK8889956.1
Candidatus Dechloromonas phosphorivorans

WP_205429692.1
Methylococcus sp. EFPC2

NOR68460.1
Methylomarinum sp.

MBL8447018.1
Zoogloeaceae bacterium

MBK8895006.1
Propionivibrio sp.

MAT52742.1
Porticoccaceae bacterium

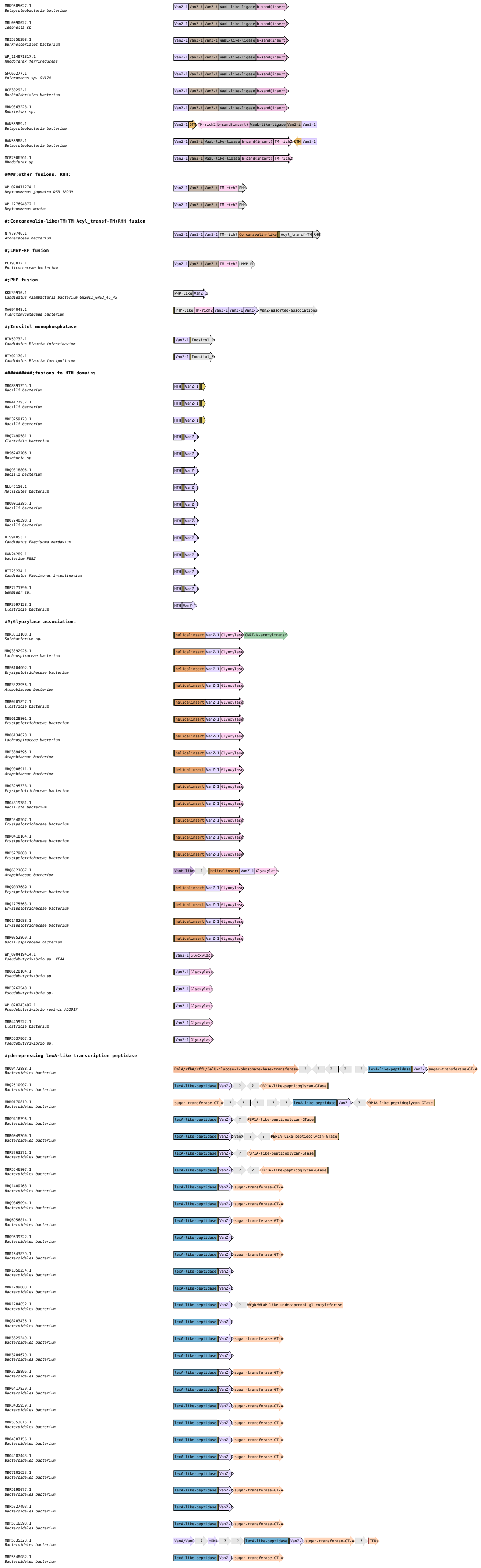
MBK9685627.1
Betaproteobacteria bacterium
VanZ-1 VanZ-i VanZ-i WaaL-like-ligase b-sand(insert)
MBL0090022.1
Ideonella sp.
VanZ-1 VanZ-i VanZ-i WaaL-like-ligase b-sand(insert)
MBI5256398.1
Burkholderiales bacterium
VanZ-1 VanZ-i VanZ-i WaaL-like-ligase b-sand(insert)
WP_114971817.1
Rhodoferax ferrireducens
VanZ-1 VanZ-i VanZ-i WaaL-like-ligase b-sand(insert)
SFC66277.1
Polaromonas sp. OV174
VanZ-1 VanZ-i VanZ-i WaaL-like-ligase b-sand(insert)
UCE30292.1
Burkholderiales bacterium
VanZ-1 VanZ-i VanZ-i WaaL-like-ligase b-sand(insert)
MBK9363228.1
Rubrivivax sp.
VanZ-1 VanZ-i VanZ-i WaaL-like-ligase b-sand(insert)
HAN56989.1
Betaproteobacteria bacterium
VanZ-1 6TM TM-rich2 b-sand(insert) WaaL-like-ligase VanZ-i VanZ-1
HAN56988.1
Betaproteobacteria bacterium
VanZ-1 VanZ-i WaaL-like-ligase b-sand(insert) TM-rich2 6TM VanZ-1
MCB2006561.1
Rhodoferax sp.
VanZ-1 VanZ-i WaaL-like-ligase b-sand(insert) TM-rich2
####;other fusions. RHH:
WP_028471274.1
Neptunomonas japonica DSM 18939
VanZ-1 VanZ-i VanZ-i TM-rich2 RHH
WP_127694872.1
Neptunomonas marina
VanZ-1 VanZ-i VanZ-i TM-rich2 RHH
#;Concanavalin-like+TM+TM+Acyl_transf-TM+RHH fusion
NTV70746.1
Azonexaceae bacterium
VanZ-1 VanZ-1 VanZ-1 TM-rich? Concanavalin-like Acyl_transf-TM RHH
#;LMWP-RP fusion
PCJ93812.1
Porticoccaceae bacterium
VanZ-1 VanZ-i VanZ-i TM-rich2 LMWP-RP
#;PHP fusion
KKU39910.1
Candidatus Azambacteria bacterium GW2011_GWE2_46_45
PHP-like VanZ-1
MAG94048.1
Planctomycetaceae bacterium
PHP-like TM-rich2 VanZ-1 VanZ-1 VanZ-1 VanZ-assorted-associations
#;Inositol monophosphatase
HIW50732.1
Candidatus Blautia intestinavium
VanZ-1 Inositol_P
HIY02170.1
Candidatus Blautia faecipullorum
VanZ-1 Inositol_P
##########;fusions to HTH domains
MBQ8891355.1
Bacilli bacterium
HTH VanZ-1
MBR4177937.1
Bacilli bacterium
HTH VanZ-1
MBP3259173.1
Bacilli bacterium
HTH VanZ-1
MBQ7499581.1
Clostridia bacterium
HTH VanZ-1
MBS6242206.1
Roseburia sp.
HTH VanZ-1
MBQ9318806.1
Bacilli bacterium
HTH VanZ-1
NLL45150.1
Mollicutes bacterium
HTH VanZ-1
MBQ9013285.1
Bacilli bacterium
HTH VanZ-1
MBQ7240398.1
Bacilli bacterium
HTH VanZ-1
HIS91053.1
Candidatus Faecisoma merdavium
HTH VanZ-1
KWW24209.1
bacterium F082
HTH VanZ-1
HIT23224.1
Candidatus Faecimonas intestinavium
HTH VanZ-1
MBP7271790.1
Gemmiger sp.
HTH VanZ-1
MBR3997128.1
Clostridia bacterium
HTH VanZ-1
##;Glyoxylase association.
MBR3311108.1
Solobacterium sp.
helicalinsert VanZ-1 Glyoxylase GNAT-N-acetyltransf
MBQ3392926.1
Lachnospiraceae bacterium
helicalinsert VanZ-1 Glyoxylase
MBE6104002.1
Erysipelotrichaceae bacterium
helicalinsert VanZ-1 Glyoxylase
MBR3327956.1
Atopobiaceae bacterium
helicalinsert VanZ-1 Glyoxylase
MBR0205857.1
Clostridia bacterium
helicalinsert VanZ-1 Glyoxylase
MBE6128801.1
Erysipelotrichaceae bacterium
helicalinsert VanZ-1 Glyoxylase
MBO6134028.1
Lachnospiraceae bacterium
helicalinsert VanZ-1 Glyoxylase
MBP3894595.1
Atopobiaceae bacterium
helicalinsert VanZ-1 Glyoxylase
MBQ9006911.1
Atopobiaceae bacterium
helicalinsert VanZ-1 Glyoxylase
MBQ3295338.1
Erysipelotrichaceae bacterium
helicalinsert VanZ-1 Glyoxylase
MBO4819381.1
Bacillota bacterium
helicalinsert VanZ-1 Glyoxylase
MBR5340567.1
Erysipelotrichaceae bacterium
helicalinsert VanZ-1 Glyoxylase
MBR0418164.1
Erysipelotrichaceae bacterium
helicalinsert VanZ-1 Glyoxylase
MBP5279088.1
Erysipelotrichaceae bacterium
helicalinsert VanZ-1 Glyoxylase
MBQ6521667.1
Atopobiaceae bacterium
VanH-like ? helicalinsert VanZ-1 Glyoxylase
MBQ9037689.1
Erysipelotrichaceae bacterium
helicalinsert VanZ-1 Glyoxylase
MBQ1775563.1
Erysipelotrichaceae bacterium
helicalinsert VanZ-1 Glyoxylase
MBQ1482688.1
Erysipelotrichaceae bacterium
helicalinsert VanZ-1 Glyoxylase
MBR0352869.1
Oscillospiraceae bacterium
helicalinsert VanZ-1 Glyoxylase
WP_090419414.1
Pseudobutyrivibrio sp. YE44
VanZ-1 Glyoxylase
MBO6128104.1
Pseudobutyrivibrio sp.
VanZ-1 Glyoxylase
MBP3262548.1
Pseudobutyrivibrio sp.
VanZ-1 Glyoxylase
WP_028243492.1
Pseudobutyrivibrio ruminis AD2017
VanZ-1 Glyoxylase
MBR4459522.1
Clostridia bacterium
VanZ-1 Glyoxylase
MBR5637967.1
Pseudobutyrivibrio sp.
VanZ-1 Glyoxylase
#;derepressing lexA-like transcription peptidase
MBQ9472888.1
Bacteroidales bacterium
RmlA/rfbA/rffH/GalU-glucose-1-phosphate-base-transferase ? ? ? ? ? lexA-like-peptidase VanZ-1 sugar-transferase-GT-A
MBQ2510907.1
Bacteroidales bacterium
lexA-like-peptidase VanZ-1 ? ? PBP1A-like-peptidoglycan-GTase
MBR0170819.1
Bacteroidales bacterium
sugar-transferase-GT-A ? ? ? ? ? lexA-like-peptidase VanZ-1 ? PBP1A-like-peptidoglycan-GTase
MBQ9418396.1
Bacteroidales bacterium
lexA-like-peptidase VanZ-1 ? PBP1A-like-peptidoglycan-GTase
MBR6049260.1
Bacteroidales bacterium
lexA-like-peptidase VanZ-1 VanX ? ? PBP1A-like-peptidoglycan-GTase
MBP3763371.1
Bacteroidales bacterium
lexA-like-peptidase VanZ-1 ? PBP1A-like-peptidoglycan-GTase
MBP5546807.1
Bacteroidales bacterium
lexA-like-peptidase VanZ-1 ? ? PBP1A-like-peptidoglycan-GTase
MBQ1409268.1
Bacteroidales bacterium
lexA-like-peptidase VanZ-1 sugar-transferase-GT-A
MBQ9865094.1
Bacteroidales bacterium
lexA-like-peptidase VanZ-1 sugar-transferase-GT-A
MBQ6956814.1
Bacteroidales bacterium
lexA-like-peptidase VanZ-1 sugar-transferase-GT-A
MBQ9639322.1
Bacteroidales bacterium
lexA-like-peptidase VanZ-1
MBR1643839.1
Bacteroidales bacterium
lexA-like-peptidase VanZ-1 sugar-transferase-GT-A
MBR1850254.1
Bacteroidales bacterium
lexA-like-peptidase VanZ-1
MBR1799803.1
Bacteroidales bacterium
lexA-like-peptidase VanZ-1
MBR1784652.1
Bacteroidales bacterium
lexA-like-peptidase VanZ-1 ? WfgD/WfaP-like-undecaprenol-glucosyltferase
MBQ8703436.1
Bacteroidales bacterium
lexA-like-peptidase VanZ-1
MBR3829249.1
Bacteroidales bacterium
lexA-like-peptidase VanZ-1 sugar-transferase-GT-A
MBR3784679.1
Bacteroidales bacterium
lexA-like-peptidase VanZ-1
MBR3528896.1
Bacteroidales bacterium
lexA-like-peptidase VanZ-1 sugar-transferase-GT-A
MBR6417829.1
Bacteroidales bacterium
lexA-like-peptidase VanZ-1 sugar-transferase-GT-A
MBR3435959.1
Bacteroidales bacterium
lexA-like-peptidase VanZ-1 sugar-transferase-GT-A
MBR5353615.1
Bacteroidales bacterium
lexA-like-peptidase VanZ-1 sugar-transferase-GT-A
MBO4307156.1
Bacteroidales bacterium
lexA-like-peptidase VanZ-1 sugar-transferase-GT-A
MBO4587443.1
Bacteroidales bacterium
lexA-like-peptidase VanZ-1 sugar-transferase-GT-A
MBO7101623.1
Bacteroidales bacterium
lexA-like-peptidase VanZ-1
MBP5190077.1
Bacteroidales bacterium
lexA-like-peptidase VanZ-1 sugar-transferase-GT-A
MBP5327493.1
Bacteroidales bacterium
lexA-like-peptidase VanZ-1
MBP5516593.1
Bacteroidales bacterium
lexA-like-peptidase VanZ-1 sugar-transferase-GT-A
MBP5535323.1
Bacteroidales bacterium
VanA/VanG ? tRNA ? ? lexA-like-peptidase VanZ-1 sugar-transferase-GT-A ? TPRs
MBP5548082.1
Bacteroidales bacterium
lexA-like-peptidase VanZ-1 sugar-transferase-GT-A

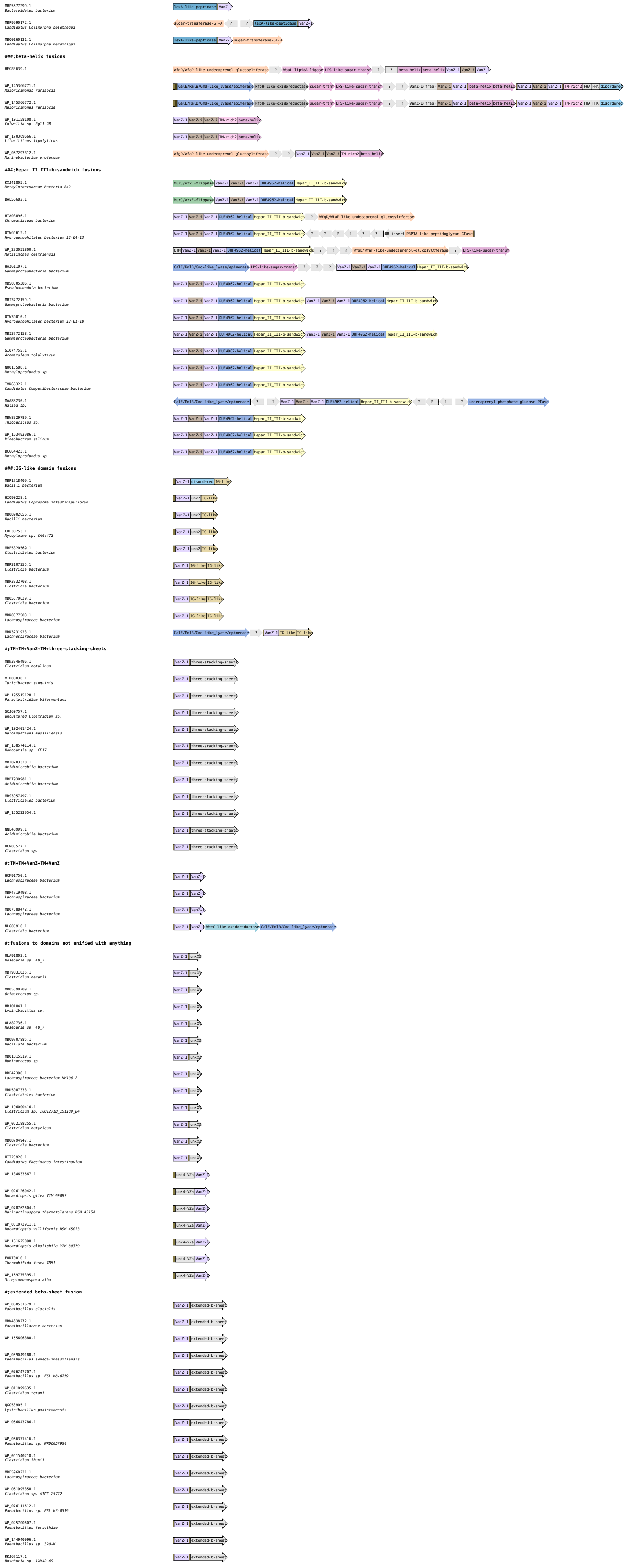
MBP5677299.1
Bacteroidales bacterium
MBP9990172.1
Candidatus Colimorpha pelethequi
MBQ0160121.1
Candidatus Colimorpha merdihippi
###;beta-helix fusions
HEG83639.1
WP_145366771.1
Maioricimonas rarisocia
WP_145366772.1
Maioricimonas rarisocia
WP_101158108.1
Colwellia sp. Bg11-28
WP_170309666.1
Litorilituus lipolyticus
WP_067297812.1
Marinobacterium profundum
###;Hepar_II_III-b-sandwich fusions
KXJ41805.1
Methylothermaceae bacteria B42
BAL56682.1
HIA08896.1
Chromatiaceae bacterium
OYW65615.1
Hydrogenophilales bacterium 12-64-13
WP_233051800.1
Motilimonas cestriensis
HAZ61107.1
Gammaproteobacteria bacterium
MBS0305386.1
Pseudomonadota bacterium
MBI3772159.1
Gammaproteobacteria bacterium
OYW36010.1
Hydrogenophilales bacterium 12-61-10
MBI3772158.1
Gammaproteobacteria bacterium
SIQ74755.1
Aromatoleum tolulyticum
NOQ15588.1
Methyloprofundus sp.
TVR66322.1
Candidatus Competibacteraceae bacterium
MAA88230.1
Haliea sp.
MBW8329789.1
Thiobacillus sp.
WP_163493986.1
Kineobactrum salinum
BCG64423.1
Methyloprofundus sp.
###;IG-like domain fusions
MBR1718409.1
Bacilli bacterium
HIQ90228.1
Candidatus Coprosoma intestinipullorum
MBQ8902656.1
Bacilli bacterium
CDE38253.1
Mycoplasma sp. CAG:472
MBE5820569.1
Clostridiales bacterium
MBR3107355.1
Clostridia bacterium
MBR3332708.1
Clostridia bacterium
MBO5570629.1
Clostridia bacterium
MBR0377503.1
Lachnospiraceae bacterium
MBR3231923.1
Lachnospiraceae bacterium
#;TM+TM+VanZ+TM+three-stacking-sheets
MBN3346496.1
Clostridium botulinum
MTH08030.1
Turicibacter sanguinis
WP_195515128.1
Paraclostridium bifermentans
SCJ60757.1
uncultured Clostridium sp.
WP_102401424.1
Haloimpatiens massiliensis
WP_168574114.1
Romboutsia sp. CE17
MBT8203320.1
Acidimicrobiia bacterium
MBP7930981.1
Acidimicrobiia bacterium
MBS3957497.1
Clostridiales bacterium
WP_155223954.1
NNL48999.1
Acidimicrobiia bacterium
HCW03577.1
Clostridium sp.
#;TM+TM+VanZ+TM+VanZ
HCM91750.1
Lachnospiraceae bacterium
MBR4719498.1
Lachnospiraceae bacterium
MBQ7588472.1
Lachnospiraceae bacterium
NLG05910.1
Clostridia bacterium
#;fusions to domains not unified with anything
OLA91803.1
Roseburia sp. 40_7
MBT9831035.1
Clostridium baratii
MBO5598289.1
Oribacterium sp.
HBJ01847.1
Lysinibacillus sp.
OLA82736.1
Roseburia sp. 40_7
MBQ9707885.1
Bacillota bacterium
MBQ1815519.1
Ruminococcus sp.
BBF42398.1
Lachnospiraceae bacterium KM106-2
MBD5087338.1
Clostridiales bacterium
WP_196000416.1
Clostridium sp. 1001271B_151109_B4
WP_052188255.1
Clostridium butyricum
MBQ8794947.1
Clostridia bacterium
HIT23928.1
Candidatus Faecimonas intestinavium
WP_184633667.1
WP_026126042.1
Nocardiopsis gilva YIM 90087
WP_078762604.1
Marinactinospora thermotolerans DSM 45154
WP_051072911.1
Nocardiopsis valliformis DSM 45023
WP_161625098.1
Nocardiopsis alkaliphila YIM 80379
EOR70010.1
Thermobifida fusca TM51
WP_169775395.1
Streptomonospora alba
#;extended beta-sheet fusion
WP_068531679.1
Paenibacillus glacialis
MBW4838272.1
Paenibacillaceae bacterium
WP_155606880.1
WP_059049188.1
Paenibacillus senegalimassiliensis
WP_076247707.1
Paenibacillus sp. FSL H8-0259
WP_011099635.1
Clostridium tetani
QGG53905.1
Lysinibacillus pakistanensis
WP_066643786.1
WP_066371416.1
Paenibacillus sp. NPDC057934
WP_051540218.1
Clostridium ihumii
MBE5960221.1
Lachnospiraceae bacterium
WP_061995858.1
Clostridium sp. ATCC 25772
WP_076111612.1
Paenibacillus sp. FSL H3-0319
WP_025700607.1
Paenibacillus forsythiae
WP_144940096.1
Paenibacillus sp. 32O-W
RKJ67117.1
Roseburia sp. 1XD42-69

#;4TM fusion

MBR3031801.1
Clostridiales bacterium
4TM VanZ-1

MBQ6271024.1
Clostridiales bacterium
4TM VanZ-1

MBO4928602.1
Clostridiales bacterium
4TM VanZ-1

WP_192119875.1
Frigoribacterium sp. CFBP 8751
4TM VanZ-1

OQA65862.1
Firmicutes bacterium ADurb.Bin262
4TM VanZ-1

PZF45567.1
4TM VanZ-1 ? ? GDP-L-fucose-synthase GalE/RmlB/Gmd-like_lyase/epimerase ? sugar-O-acetyl/acyltransf sugar-transferase-GT-A

RKI38166.1
bacterium D16-59
4TM VanZ-1 tail

TAH71122.1
Anaerolineaceae bacterium
4TM VanZ-1 tail

HBY72587.1
Lachnospiraceae bacterium
4TM VanZ-1 tail

NLO10276.1
Clostridiales bacterium
4TM VanZ-1 tail

CDA87802.1
Clostridium sp. CAG:230
4TM VanZ-1 tail

MBR1743780.1
Lachnospiraceae bacterium
4TM VanZ-1 tail

HHT88879.1
Clostridiales bacterium
4TM VanZ-1 tail

RHT93371.1
Clostridium sp. AM27-31LB
4TM VanZ-1 tail

MBP1755980.1
Bacillota bacterium
4TM VanZ-1 tail

NLK27827.1
Clostridiales bacterium
4TM VanZ-1 tail

WP_184095885.1
Anaerocolumna cellulosilytica
4TM VanZ-1 tail

WP_184289528.1
Nocardiopsis algeriensis
4TM VanZ-1 tail

MCD7825585.1
Clostridiaceae bacterium
4TM VanZ-1 tail

NLK73727.1
Clostridiales bacterium
4TM VanZ-1 tail

MBQ1994222.1
Lachnospiraceae bacterium
4TM VanZ-1 tail

NLL73265.1
Clostridiales bacterium
4TM VanZ-1 tail

HHU73095.1
Clostridiales bacterium
4TM VanZ-1 tail

MBR7072367.1
Eubacterium sp.
4TM VanZ-1 tail

NLJ87337.1
Candidatus Epulonipiscium sp.
4TM VanZ-1 tail

MBO5199228.1
Lachnospiraceae bacterium
4TM VanZ-1 tail

MBR5620314.1
Clostridia bacterium
4TM VanZ-1 tail

NLB36280.1
Clostridiales bacterium
4TM VanZ-1 tail

NLM48728.1
Candidatus Epulonipiscium sp.
4TM VanZ-1 tail

HHT76871.1
Clostridiaceae bacterium
4TM VanZ-1 tail

MBE5950925.1
Lachnospiraceae bacterium
4TM VanZ-1 tail

WP_154516530.1
Velocimicrobium porci
4TM VanZ-1 tail

WP_006316531.1
Caldisalinibacter kiritimatiensis
4TM VanZ-1 tail

NLC17902.1
Clostridiales bacterium
4TM VanZ-1 tail

PWL72990.1
Clostridiales bacterium
4TM VanZ-1 tail

RHP88144.1
Roseburia sp. AM59-24XD
4TM VanZ-1 tail

HIU66407.1
Candidatus Caccomorpha excrementavium
4TM VanZ-1 tail

MBD8929839.1
Clostridiales bacterium
4TM VanZ-1 tail

MBR3738484.1
Eubacterium sp.
4TM VanZ-1 tail

MBQ9516681.1
Eubacterium sp.
4TM VanZ-1 tail

MBR2099804.1
Eubacterium sp.
4TM VanZ-1 tail

MCC8073430.1
Clostridiales bacterium
4TM VanZ-1 tail

MBR1762700.1
Eubacterium sp.
4TM VanZ-1 tail

MBA4293364.1
bacterium
4TM VanZ-1

MBS1705112.1
Armatimonadota bacterium
4TM VanZ-1

MBL1151576.1
Armatimonadota bacterium
4TM VanZ-1

MBL8065108.1
Chthonomonadaceae bacterium
4TM VanZ-1

AUY52359.1
Streptomyces sp. CB01881
4TM VanZ-1

GID56733.1
Actinoplanes couchii
4TM VanZ-1

KAB1141601.1
Streptomyces luteolifulvus
4TM VanZ-1

GIE19264.1
Winogradskya humida
4TM VanZ-1

AWW43400.1
Streptomyces cadmiisoli
4TM VanZ-1

WP_196452672.1
4TM VanZ-1

WP_206074611.1
Antribacter gilvus
4TM VanZ-1

SDC04353.1
Raineyella antarctica
4TM VanZ-1

WP_231935165.1
Micromonospora viridifaciens
4TM VanZ-1

KFF95042.1
Streptomyces scabiei
4TM VanZ-1

HHX70683.1
Gallicola sp.
4TM VanZ-1 tail

WP_100065894.1
Miniphocaeibacter massiliensis
4TM VanZ-1 tail

SBN62278.1
Curtobacterium sp. 9128
4TM VanZ-1 tail

#########;additional N- or C-terminal TMs; #;TM+TM+VanZ+TM+TM

WP_029072970.1
Kandleria vitulina WCE2011
VanZ-1 VanZ-1 ? ? ? sugar-O-acetyl/acyltransf

MBP3277092.1
Kandleria sp.
VanZ-1

WP_051550726.1
Kandleria vitulina WCE2011
VanZ-1 VanZ-1 ? ? ? sugar-O-acetyl/acyltransf

WP_074686441.1
Kandleria vitulina
VanZ-1 ? ? ? ? sugar-O-acetyl/acyltransf

TWP16042.1
TM7 phylum sp. oral taxon 353
VanZ-1

TWP28947.1
TM7 phylum sp. oral taxon 348
VanZ-1

#;TM+VanZ+TM+TM+TM+TM

MBL8431067.1
Dechloromonas sp.
VanZ-1

EDR97276.1
Anaerostipes caccae L1-92
VanZ-1

HJC49194.1
Candidatus Anaerostipes avistercoris
VanZ-1

MBE5942639.1
Lachnospiraceae bacterium
VanZ-1

WP_075712898.1
Hornefia porci
VanZ-1

MCB6992488.1
bacterium 210820-DFI.6.37
VanZ-1

MBQ4194241.1
Clostridia bacterium
VanZ-1

#;VanZ+TM+TM+TM

MBU6438124.1
Betaproteobacteria bacterium
VanZ-1

MBC7716130.1
Rhizobacter sp.
VanZ-1

OGA45961.1
Betaproteobacteria bacterium RIFCSPLOWO2_12_FULL_62_58
VanZ-1

HAJ91024.1
Gammaproteobacteria bacterium
VanZ-1

MBL0890274.1
Gemmatimonadaceae bacterium
VanZ-1

MBS6521339.1
Clostridiales bacterium
VanZ-1

HIV17932.1
Candidatus Merdivicinus intestinigallinarum
VanZ-1

WP_058963680.1
Allofournierella massiliensis
VanZ-1

HCF36668.1
Oscillospiraceae bacterium
VanZ-1 3TM

WP_204649300.1
Gemmiger formicilis
VanZ-1 3TM

MBD8952826.1
Subdoligranulum sp.
VanZ-1

#;disordered+TM+VanZ-1

SEF16236.1
Streptomyces sp. Ag109_O5-10
disordered VanZ-1

SCK32456.1
Streptomyces sp. WMMB 714
disordered VanZ-1

SHH57995.1
Streptomyces sp. 3214.6
disordered VanZ-1

RPE46240.1
Streptomyces sp. Ag109_O5-1
disordered VanZ-1

#;TM+TM+VanZ-1+disordered

WP_215549012.1
Amycolatopsis sp. CA-230715
VanZ-1 disordered

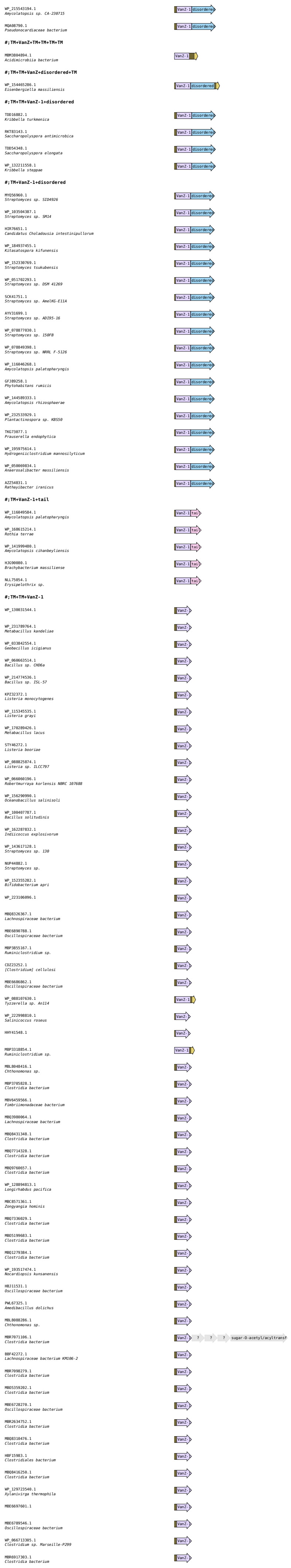
WP_215543194.1
Amycolatopsis sp. CA-230715
VanZ-1 disordered
MQA08790.1
Pseudonocardiaceae bacterium
VanZ-1 disordered
#;TM+VanZ+TM+TM+TM+TM
MBM3804894.1
Acidimicrobiia bacterium
VanZ-1
#;TM+TM+VanZ+disordered+TM
WP_154465286.1
Eisenbergiella massiliensis
VanZ-1 disordered
#;TM+TM+VanZ-1+disordered
TDD16882.1
Kribbella turkmenica
VanZ-1 disordered
RKT83143.1
Saccharopolyspora antimicrobica
VanZ-1 disordered
TDD54348.1
Saccharopolyspora elongata
VanZ-1 disordered
WP_132211558.1
Kribbella steppae
VanZ-1 disordered
#;TM+VanZ-1+disordered
MYQ56960.1
Streptomyces sp. SID4926
VanZ-1 disordered
WP_103504387.1
Streptomyces sp. SM14
VanZ-1 disordered
HIR76651.1
Candidatus Choladousia intestinipullorum
VanZ-1 disordered
WP_184937455.1
Kitasatospora kifunensis
VanZ-1 disordered
WP_152330769.1
Streptomyces tsukubensis
VanZ-1 disordered
WP_051702293.1
Streptomyces sp. DSM 41269
VanZ-1 disordered
SCK41751.1
Streptomyces sp. AmelKG-E11A
VanZ-1 disordered
AYV31699.1
Streptomyces sp. ADI95-16
VanZ-1 disordered
WP_078877030.1
Streptomyces sp. 150FB
VanZ-1 disordered
WP_078849398.1
Streptomyces sp. NRRL F-5126
VanZ-1 disordered
WP_116046268.1
Amycolatopsis palatopharyngis
VanZ-1 disordered
GFJ89258.1
Phytohabitans rumicis
VanZ-1 disordered
WP_144589333.1
Amycolatopsis rhizosphaerae
VanZ-1 disordered
WP_232533929.1
Plantactinospora sp. KBS50
VanZ-1 disordered
TKG73077.1
Prauserella endophytica
VanZ-1 disordered
WP_195975614.1
Hydrogeniiclostridium mannosilyticum
VanZ-1 disordered
WP_050069834.1
Anaerosalibacter massiliensis
VanZ-1 disordered
AZZ54831.1
Rathayibacter iranicus
VanZ-1 disordered
#;TM+VanZ-1+tail
WP_116049584.1
Amycolatopsis palatopharyngis
VanZ-1 tail
WP_168615214.1
Rothia terrae
VanZ-1 tail
WP_141999480.1
Amycolatopsis cihanbeyliensis
VanZ-1 tail
HJG90080.1
Brachybacterium massiliense
VanZ-1 tail
NLL75054.1
Erysipelothrix sp.
VanZ-1 tail
#;TM+TM+VanZ-1
WP_130031544.1
VanZ-1
WP_231789764.1
Metabacillus kandeliae
VanZ-1
WP_033842554.1
Geobacillus icigianus
VanZ-1
WP_060663514.1
Bacillus sp. CHD6a
VanZ-1
WP_214774536.1
Bacillus sp. ISL-57
VanZ-1
KPZ32372.1
Listeria monocytogenes
VanZ-1
WP_115345535.1
Listeria grayi
VanZ-1
WP_170289426.1
Metabacillus lacus
VanZ-1
STY46272.1
Listeria booriae
VanZ-1
WP_088825874.1
Listeria sp. ILCC797
VanZ-1
WP_066060196.1
Robertmurraya korlensis NBRC 107688
VanZ-1
WP_156290990.1
Oceanobacillus salinisoli
VanZ-1
WP_100407787.1
Bacillus solitudinis
VanZ-1
WP_162287832.1
Indiicoccus explosivorum
VanZ-1
WP_143617128.1
Streptomyces sp. 130
VanZ-1
NUP44882.1
Streptomyces sp.
VanZ-1
WP_152355282.1
Bifidobacterium apri
VanZ-1
WP_223106096.1
VanZ-1
MBQ8326367.1
Lachnospiraceae bacterium
VanZ-1
MBE6890788.1
Oscillospiraceae bacterium
VanZ-1
MBP3855167.1
Ruminiclostridium sp.
VanZ-1
CDZ23252.1
[Clostridium] cellulosi
VanZ-1
MBE6686862.1
Oscillospiraceae bacterium
VanZ-1
WP_088107630.1
Tyzzerella sp. An114
VanZ-1
WP_222998810.1
Salinicoccus roseus
VanZ-1
HHY41548.1
VanZ-1
MBP3318854.1
Ruminiclostridium sp.
VanZ-1
MBL8048416.1
Chthonomonas sp.
VanZ-1
MBP3705828.1
Clostridia bacterium
VanZ-1
MBV6459566.1
Fimbriimonadaceae bacterium
VanZ-1
MBQ3980064.1
Lachnospiraceae bacterium
VanZ-1
MBQ8431348.1
Clostridia bacterium
VanZ-1
MBQ7714328.1
Clostridia bacterium
VanZ-1
MBQ9760657.1
Clostridia bacterium
VanZ-1
WP_128894813.1
Longirhabdus pacifica
VanZ-1
MBC8571361.1
Zongyangia hominis
VanZ-1
MBQ7336029.1
Clostridia bacterium
VanZ-1
MBO5199683.1
Clostridia bacterium
VanZ-1
MBQ1279384.1
Clostridia bacterium
VanZ-1
WP_193517474.1
Nocardiopsis kunsanensis
VanZ-1
HBJ11531.1
Oscillospiraceae bacterium
VanZ-1
PWL67325.1
Amedibacillus dolichus
VanZ-1
MBL8088286.1
Chthonomonas sp.
VanZ-1
MBR7071106.1
Clostridia bacterium
VanZ-1 ? ? ? sugar-O-acetyl/acyltransf
BBF42272.1
Lachnospiraceae bacterium KM106-2
VanZ-1
MBR7098279.1
Clostridia bacterium
VanZ-1
MBO5359202.1
Clostridia bacterium
VanZ-1
MBE6728270.1
Oscillospiraceae bacterium
VanZ-1
MBR2634752.1
Clostridia bacterium
VanZ-1
MBQ8310476.1
Clostridia bacterium
VanZ-1
HBF15983.1
Clostridiales bacterium
VanZ-1
MBQ8416250.1
Clostridia bacterium
VanZ-1
WP_129723540.1
Xylanivirga thermophila
VanZ-1
MBE6697601.1
VanZ-1
MBE6789546.1
Oscillospiraceae bacterium
VanZ-1
WP_066713305.1
Clostridium sp. Marseille-P299
VanZ-1
MBR6917303.1
Clostridia bacterium
VanZ-1

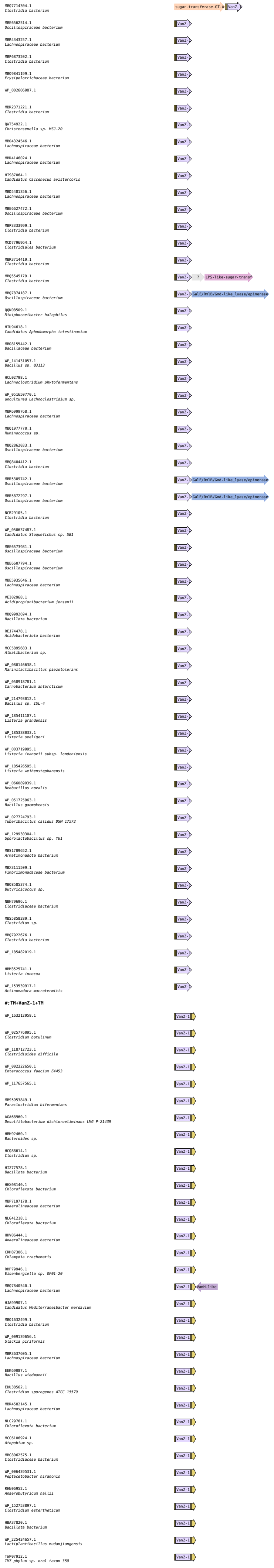
MBQ7714304.1
Clostridia bacterium
sugar-transferase-GT-A
VanZ-1
MBE6562514.1
Oscillospiraceae bacterium
VanZ-1
MBR4343257.1
Lachnospiraceae bacterium
VanZ-1
MBP6873202.1
Clostridia bacterium
VanZ-1
MBQ9841199.1
Erysipelotrichaceae bacterium
VanZ-1
WP_002606987.1
VanZ-1
MBR2371221.1
Clostridia bacterium
VanZ-1
QWT54922.1
Christensenella sp. MSJ-20
VanZ-1
MBO4324546.1
Lachnospiraceae bacterium
VanZ-1
MBR4146024.1
Lachnospiraceae bacterium
VanZ-1
HIS87064.1
Candidatus Caccenecus avistercoris
VanZ-1
MBD5481356.1
Lachnospiraceae bacterium
VanZ-1
MBE6627472.1
Oscillospiraceae bacterium
VanZ-1
MBP3333999.1
Clostridia bacterium
VanZ-1
MCD7796964.1
Clostridiales bacterium
VanZ-1
MBR3714419.1
Clostridia bacterium
VanZ-1
MBQ5545179.1
Clostridia bacterium
VanZ-1
?
LPS-like-sugar-transf
MBQ7874187.1
Oscillospiraceae bacterium
VanZ-1
GalE/RmlB/Gmd-like_lyase/epimerase
QQK08509.1
Miniphocaeibacter halophilus
VanZ-1
HIU94618.1
Candidatus Aphodomorpha intestinavium
VanZ-1
MBO8155442.1
Bacillaceae bacterium
VanZ-1
WP_141431057.1
Bacillus sp. 03113
VanZ-1
HCL02798.1
Lachnoclostridium phytofermentans
VanZ-1
WP_051650770.1
uncultured Lachnoclostridium sp.
VanZ-1
MBR6999768.1
Lachnospiraceae bacterium
VanZ-1
MBQ1977770.1
Ruminococcus sp.
VanZ-1
MBQ2862033.1
Oscillospiraceae bacterium
VanZ-1
MBQ8404412.1
Clostridia bacterium
VanZ-1
MBR5309742.1
Oscillospiraceae bacterium
VanZ-1
GalE/RmlB/Gmd-like_lyase/epimerase
MBR5872297.1
Oscillospiraceae bacterium
VanZ-1
GalE/RmlB/Gmd-like_lyase/epimerase
NCB29105.1
Clostridia bacterium
VanZ-1
WP_050637487.1
Candidatus Stoquefichus sp. SB1
VanZ-1
MBE6573981.1
Oscillospiraceae bacterium
VanZ-1
MBE6607794.1
Oscillospiraceae bacterium
VanZ-1
MBE5935646.1
Lachnospiraceae bacterium
VanZ-1
VEI02968.1
Acidipropionibacterium jensenii
VanZ-1
MBQ9992694.1
Bacillota bacterium
VanZ-1
REJ74478.1
Acidobacteriota bacterium
VanZ-1
MCC5895683.1
Alkalibacterium sp.
VanZ-1
WP_080146638.1
Marinilactibacillus piezotolerans
VanZ-1
WP_058918781.1
Carnobacterium antarcticum
VanZ-1
WP_214793012.1
Bacillus sp. ISL-4
VanZ-1
WP_185411107.1
Listeria grandensis
VanZ-1
WP_185338033.1
Listeria seeligeri
VanZ-1
WP_003719995.1
Listeria ivanovii subsp. londoniensis
VanZ-1
WP_185426595.1
Listeria weihenstephanensis
VanZ-1
WP_066089939.1
Neobacillus novalis
VanZ-1
WP_051725963.1
Bacillus gaemokensis
VanZ-1
WP_027724793.1
Tuberibacillus calidus DSM 17572
VanZ-1
WP_129930304.1
Sporolactobacillus sp. Y61
VanZ-1
MBS1709652.1
Armatimonadota bacterium
VanZ-1
MBX3111509.1
Fimbriimonadaceae bacterium
VanZ-1
MBQ8585374.1
Butyricicoccus sp.
VanZ-1
NBH79696.1
Clostridiaceae bacterium
VanZ-1
MBS5858289.1
Clostridium sp.
VanZ-1
MBQ7922676.1
Clostridia bacterium
VanZ-1
WP_185482019.1
VanZ-1
HBM3525741.1
Listeria innocua
VanZ-1
WP_153539917.1
Actinomadura macrotermitis
VanZ-1
#;TM+VanZ-1+TM
WP_163212958.1
VanZ-1
WP_025776095.1
Clostridium botulinum
VanZ-1
WP_118712723.1
Clostridioides difficile
VanZ-1
WP_002322650.1
Enterococcus faecium E4453
VanZ-1
WP_117657565.1
VanZ-1
MBS5953849.1
Paraclostridium bifermentans
VanZ-1
AGA68960.1
Desulfitobacterium dichloroeliminans LMG P-21439
VanZ-1
HBH92460.1
Bacteroides sp.
VanZ-1
HCQ88614.1
Clostridium sp.
VanZ-1
HIZ77578.1
Bacillota bacterium
VanZ-1
HHX08140.1
Chloroflexota bacterium
VanZ-1
MBP7197178.1
Anaerolineaceae bacterium
VanZ-1
NLG41218.1
Chloroflexota bacterium
VanZ-1
HHV06444.1
Anaerolineaceae bacterium
VanZ-1
CRH87306.1
Chlamydia trachomatis
VanZ-1
RHP79946.1
Eisenbergiella sp. OF01-20
VanZ-1
MBQ7840540.1
Lachnospiraceae bacterium
VanZ-1
VanH-like
HJA99907.1
Candidatus Mediterraneibacter merdavium
VanZ-1
MBQ1632499.1
Clostridia bacterium
VanZ-1
WP_009139656.1
Slackia piriformis
VanZ-1
MBR3637605.1
Lachnospiraceae bacterium
VanZ-1
EEK69087.1
Bacillus wiedmannii
VanZ-1
EDU38562.1
Clostridium sporogenes ATCC 15579
VanZ-1
MBR4582145.1
Lachnospiraceae bacterium
VanZ-1
NLC29761.1
Chloroflexota bacterium
VanZ-1
MCC6106924.1
Atopobium sp.
VanZ-1
MBC8062575.1
Clostridiaceae bacterium
VanZ-1
WP_006439531.1
Peptacetobacter hiranonis
VanZ-1
RHN06952.1
Anaerobutyricum hallii
VanZ-1
WP_152753897.1
Clostridium estertheticum
VanZ-1
HBA37820.1
Bacillota bacterium
VanZ-1
WP_225424657.1
Lactiplantibacillus mudanjiangensis
VanZ-1
TWP07912.1
TM7 phylum sp. oral taxon 350
VanZ-1

Accession	Organism	Domains
MBO6040661.1	*Oscillospiraceae bacterium*	VanZ-1
WP_204459663.1	*Agromyces cerinus subsp. nitratus*	VanZ-1
WP_005947324.1	*Blautia hansenii*	VanZ-1
WP_066731080.1	*uncultured Bariatricus sp.*	VanZ-1
WP_066675791.1	*Clostridium septicum*	VanZ-1
WP_117632530.1	*Hungatella sp.*	VanZ-1
WP_161593648.1	*Clostridium botulinum*	VanZ-1
HJB83074.1	*Candidatus Mediterraneibacter intestinavium*	VanZ-1

#;TM+VanZ-1+beta-hairpin-tail

Accession	Organism	Domains
WP_184892879.1	*Amycolatopsis umgeniensis*	VanZ-1, beta-hairpin-tail
WP_192744346.1	*Amycolatopsis roodepoortensis*	VanZ-1, beta-hairpin-tail

#;VanZ-1+TM

Accession	Organism	Domains
EHQ89040.1	*Desulfosporosinus youngiae DSM 17734*	VanZ-1
CCY59899.1	*Clostridium sp. CAG:264*	VanZ-1
MBQ7323818.1	*Clostridia bacterium*	VanZ-1
MBR3875206.1	*Clostridia bacterium*	VanZ-1
MBQ1229805.1	*Clostridia bacterium*	VanZ-1
MBR5120667.1	*Clostridia bacterium*	VanZ-1
MBU0617030.1	*Planctomycetota bacterium*	VanZ-1

#;TM+TM+VanZ-1+TM

Accession	Organism	Domains
NMC16283.1	*Chloroflexota bacterium*	2TM, VanZ-1
MBP7032151.1	*Anaerolineaceae bacterium*	2TM, VanZ-1
NLW72273.1	*Chloroflexota bacterium*	2TM, VanZ-1
MBQ8472988.1	*Bacilli bacterium*	VanZ-1
HIS12469.1	*Candidatus Onthocola stercoravium*	VanZ-1
CCY79972.1	*Mycoplasma sp. CAG:877*	VanZ-1
MCD7753813.1	*Clostridiales bacterium*	VanZ-1
CCY88023.1	*Mycoplasma sp. CAG:956*	VanZ-1
CDA60492.1	*Clostridium sp. CAG:524*	VanZ-1
MBM4455210.1	*Verrucomicrobiota bacterium*	VanZ-1
MUV06720.1	*Planococcaceae bacterium Storch 2/2-2*	VanZ-1
WP_195933688.1	*Turicibacter sanguinis*	VanZ-1
WP_006785174.1	*uncultured Turicibacter sp.*	VanZ-1

#;TM+TM+TM+VanZ-1

Accession	Organism	Domains
MBQ6150695.1	*Mogibacterium sp.*	VanZ-1
WP_087368629.1	*Faecalibacterium sp. An192*	VanZ-1
MBQ2992066.1	*Clostridia bacterium*	VanZ-1

#;TM+VanZ-1

Accession	Organism	Domains
MBQ7727164.1	*Clostridia bacterium*	VanZ-1
KID29218.1	*Prauserella sp. Am3*	VanZ-1
PZF48717.1		VanZ-1
MCB0194463.1	*Anaerolineae bacterium*	VanZ-1
RXR32406.1	*Oerskovia turbata*	VanZ-1
MWK33507.1	*Actinomadura sp. J1-007*	VanZ-1
TFH71085.1	*Cellulomonas sp. HD19AZ1*	VanZ-1
OEI67510.1	*Curtobacterium sp. ER1/6*	VanZ-1
KAF0209279.1	*Actinomycetota bacterium*	VanZ-1
WP_195446111.1	*Flavonifractor plautii*	VanZ-1
WP_087330275.1	*Flavonifractor sp. An4*	VanZ-1
WP_087240593.1	*Flavonifractor sp. An10*	VanZ-1
WP_118642929.1	*Pseudoflavonifractor sp. AF19-9AC*	VanZ-1
WP_204883750.1	*Pseudoflavonifractor phocaeensis*	VanZ-1
MTQ97346.1	*Pseudoflavonifractor sp. BIOML-A16*	VanZ-1
PPI55439.1	*Rathayibacter toxicus*	VanZ-1
MCD8340723.1	*Clostridiales bacterium*	VanZ-1
MBD9119012.1	*Clostridiales bacterium*	VanZ-1
RKI70680.1	*bacterium 1xD42-67*	VanZ-1
HJB70028.1	*Candidatus Flavonifractor avistercoris*	VanZ-1
WP_161845843.1	*Pseudoflavonifractor sp. 524-17*	VanZ-1
MBO4460111.1	*Clostridiales bacterium*	VanZ-1
PDO09495.1	*Candidatus Reconcilbacillus cellulovorans*	VanZ-1
MBQ6420355.1	*Clostridia bacterium*	VanZ-1
WP_058593612.1		VanZ-1
PFG94300.1	*Saccharopolyspora erythraea NRRL 2338*	VanZ-1
MBO5649502.1	*Clostridia bacterium*	VanZ-1
QES02867.1	*Streptomyces venezuelae ATCC 10712*	VanZ-1
EFB63156.1	*Lactobacillus gasseri 224-1*	VanZ-1
MCB5936377.1	*Bacillus sp. DFI.2.34*	VanZ-1
EEL88525.1	*Bacillus cereus AH1272*	VanZ-1
WP_101659345.1	*Aerococcus christensenii*	VanZ-1
EEG28963.1	*[Clostridium] methylpentosum DSM 5476*	VanZ-1
HIT32911.1	*Candidatus Enterenecus stercoripullorum*	VanZ-1
MBQ7672978.1	*Alphaproteobacteria bacterium*	VanZ-1
WP_225253509.1	*Jidongwangia harbinensis*	VanZ-1
NLC65169.1	*Erysipelothrix sp.*	VanZ-1
BCN31062.1	*Anaeromicropila herbilytica*	VanZ-1
MBE5960864.1	*Lachnospiraceae bacterium*	VanZ-1
WP_028852265.1	*Thermocrispum municipale DSM 44069*	VanZ-1
WP_026360566.1	*Amycolatopsis nigrescens CSC17Ta-90*	VanZ-1
WP_075717470.1	*Roseburia sp. 499*	VanZ-1
MBS6833835.1	*Bacillota bacterium*	VanZ-1
MBP3569904.1	*Lachnospiraceae bacterium*	VanZ-1
MBO5353111.1	*Lachnospiraceae bacterium*	VanZ-1
MBQ3513118.1	*Lachnospiraceae bacterium*	VanZ-1
WP_051309732.1	*Thermocrispum sp.*	VanZ-1
NLK76989.1	*Clostridiales bacterium*	VanZ-1
WP_204861432.1	*Pseudoflavonifractor phocaeensis*	VanZ-1
SCJ12606.1	*uncultured Flavonifractor sp.*	VanZ-1
HIR83991.1	*Candidatus Galloscillospira excrementavium*	VanZ-1
WP_195396606.1	*uncultured Holdemania sp.*	VanZ-1
WP_003678475.1	*Loigolactobacillus coryniformis subsp. coryniformis*	VanZ-1
WP_225418255.1	*Companilactobacillus mishanensis*	VanZ-1
AEB30341.1	*Carnobacterium sp. 17-4*	VanZ-1
MBM6614403.1	*Desemzia sp. RIT 804*	VanZ-1

GEB90489.1
Lactobacillus delbrueckii subsp. bulgaricus VanZ-1

HIZ12297.1
Candidatus Eubacterium faecavium VanZ-1

HIW86560.1
Candidatus Eubacterium faecipullorum VanZ-1

CCY67293.1
Clostridium sp. CAG:678 VanZ-1

AUH40987.1
Streptomyces sp. CMB-StM0423 VanZ-1

BAC19651.1
Corynebacterium efficiens YS-314 VanZ-1

SCE00796.1
Streptomyces sp. SolWspMP-sol7th VanZ-1

SOD62030.1
Streptomyces zhaozhouensis VanZ-1

GHH57169.1
Streptomyces candidus disordered VanZ-1

EGX56167.1
Streptomyces zinciresistens K42 disordered VanZ-1

EST33339.1
Streptomycetaceae bacterium MP113-05 disordered VanZ-1

MTG88089.1
Cellulosimicrobium composti disordered VanZ-1

WP_206098686.1
Paenibacillus silvae disordered VanZ-1

PYC77572.1
Streptomyces tateyamensis disordered VanZ-1

WP_164311796.1
Streptomyces sp. SID12501 disordered VanZ-1

WP_105969626.1
Streptomyces geranii disordered VanZ-1

NMO37066.1
Streptomyces sp. GMY02 disordered VanZ-1

QNF41243.1 disordered VanZ-1

WP_184967025.1
Streptomyces echinatus disordered VanZ-1

WP_030172422.1
Streptomyces sp. NRRL S-813 disordered VanZ-1

WP_014676614.1
Streptomyces olivaceoviridis disordered VanZ-1

KPI14353.1
Actinobacteria bacterium OK074 disordered VanZ-1

MBM3286795.1
Candidatus Eiseniibacteriota bacterium disordered VanZ-1

SEB82716.1
Streptomyces misionensis disordered VanZ-1

AZC14992.1
Microbacterium sp. ABRD28 disordered VanZ-1

WP_051450566.1
Actinospica robiniae DSM 44927 disordered VanZ-1

MBE6470240.1
Coriobacteriaceae bacterium disordered VanZ-1

RKE23096.1
Streptomyces sp. TLI_171 disordered VanZ-1

MBM4266523.1
Deltaproteobacteria bacterium disordered VanZ-1

WP_064537786.1
Streptomyces sp. NPDC058319 disordered VanZ-1

GGZ46963.1
Streptomyces subrutilus disordered VanZ-1

GFE23553.1
Streptomyces nigrescens disordered VanZ-1

RXA20936.1
Methanosarcina sp. MSH10X1 disordered VanZ-1

HGS36588.1 disordered VanZ-1

MBA9057601.1
Streptomyces murinus disordered VanZ-1

TYL54095.1
Agromyces mariniharenae disordered VanZ-1

WP_031172400.1
Streptomyces durhamensis disordered VanZ-1

#;VanZ-1+disordered

WP_152770923.1
Streptomyces spongiae VanZ-1 disordered

WP_079034869.1 VanZ-1 disordered

WP_079047832.1
Streptomyces hirsutus VanZ-1 disordered

SDT74966.1
Streptomyces sp. TLI_053 VanZ-1 disordered

WP_051811611.1
Kitasatospora sp. MBT63 VanZ-1 disordered

#;VanZ-1+tail

GHJ35226.1
Streptomyces sp. TS71-3 VanZ-1 tail

AXG82818.1
Streptomyces paludis VanZ-1 tail

GGZ18781.1
Streptomyces inusitatus VanZ-1 tail

AGK79601.1
Streptomyces microflavus DSM 40593 VanZ-1 tail

GHF68392.1
Streptomyces thermodiastaticus VanZ-1 tail

RAJ71703.1
Streptomyces sp. Amel2xB2 VanZ-1 tail

MBR7675206.1
Streptomyces daliensis VanZ-1 tail

#;VanZ-1

TFC74528.1
Cryobacterium sp. TMS1-20-1 VanZ-1

MUN08977.1
Agromyces luteolus VanZ-1

#;helical-bundle+VanZ-1+TM

CAB0934445.1
Corynebacterium diphtheriae helical-bundle VanZ-1

#;TM+VanZ-1+TM

WP_008976313.1 VanZ-1

HJC90253.1
Candidatus Mediterraneibacter excrementigallinarum VanZ-1

OJG14216.1
Enterococcus canintestini VanZ-1

NBJ03979.1
Lachnospiraceae bacterium VanZ-1

#;TM+TM+VanZ-1

MBO4668477.1
Lachnospiraceae bacterium VanZ-1

MBR6020280.1
Lachnospiraceae bacterium VanZ-1 VanH-like

MBO4626611.1
Lachnospiraceae bacterium VanZ-1 VanH-like

#;TM+VanZ-1

SCI43563.1
uncultured Flavonifractor sp. VanZ-1

#;TM+TM+VanZ-1

WP_191565297.1
Metabacillus idriensis VanZ-1

EHK2346353.1
Clostridium perfringens VanZ-1

MBQ5561023.1
Lachnospiraceae bacterium VanZ-1

WP_196313093.1
Streptococcus pseudopneumoniae VanZ-1

WP_213536212.1
Lactococcus nasutitermitis VanZ-1

MBO4635570.1
Clostridiales bacterium VanZ-1

WP_078831611.1 VanZ-1

MBS1721601.1
Armatimonadota bacterium VanZ-1

WP_004803350.1
Eggerthia catenaformis VanZ-1

MBR6564460.1
Clostridia bacterium VanZ-1

PWM47919.1
Clostridia bacterium VanZ-1

TAK30621.1
Chloroflexota bacterium VanZ-1

MBE6899757.1
Oscillospiraceae bacterium VanZ-1

MBQ9318470.1
Bacilli bacterium VanZ-1

SMC34352.1
Oscillospiraceae bacterium VanZ-1

PLR74734.1
Bacillus sp. UMB0728 VanZ-1

WP_204593185.1
Clostridium sardiniense VanZ-1

NLN55737.1
Clostridiales bacterium VanZ-1

MBR2523185.1
Clostridiales bacterium VanZ-1

MBO4972444.1
Clostridia bacterium VanZ-1 ? VanH-like

HCV46927.1
Catenibacterium sp. VanZ-1

TMA60636.1
Deltaproteobacteria bacterium VanZ-1

BAL70062.1
Streptococcus mutans LJ23 VanZ-1

WP_224809887.1
Halobacillus yeomjeoni VanZ-1

HBA93960.1
Oscillospiraceae bacterium VanZ-1

NLW69855.1
Eubacteriaceae bacterium VanZ-1

RYG37322.1
bacterium VanZ-1

#;strand-run+VanZ-1

WP_100209233.1		strand-run VanZ-1

#;TM+VanZ-1

WP_015405266.1	*Desulfocapsa sulfexigens DSM 10523*	VanZ-1
MBQ7549773.1	*Clostridia bacterium*	VanZ-1
WP_004851584.1	*Coprococcus eutactus*	VanZ-1
MBQ1840766.1	*Atopobiaceae bacterium*	sugar-tranf LPS-like-sugar-transf VanZ-1
WP_204899309.1	*Pseudoflavonifractor capillosus*	VanZ-1
HBL41209.1	*Oscillospiraceae bacterium*	VanZ-1

#;3-helix+VanZ-1

WP_191751068.1	*Clostridium gallinarum*	3-helix VanZ-1

#;TM+VanZ-1+TM

HCT92777.1	*Lachnospiraceae bacterium*	VanZ-1

#;TM+VanZ-1+TM

WP_118383843.1	*Dorea sp. AM58-8*	VanZ-1
MCD8356861.1	*Clostridia bacterium*	VanZ-1
MBS6951020.1	*Blautia sp.*	VanZ-1
MBQ6837143.1	*Clostridia bacterium*	VanZ-1
WP_105176707.1		VanZ-1
MBR6014763.1	*Bacillota bacterium*	VanZ-1
HIQ81416.1	*Candidatus Scatavimonas merdigallinarum*	VanZ-1
AZV57952.1	*Clostridium sp. AWRP*	VanZ-1
PRR82472.1	*Clostridium luticellarii*	VanZ-1
WP_101722495.1	*Eggerthella timonensis*	VanZ-1
WP_024622513.1	*Clostridioides mangenotii TR*	VanZ-1
NBI91263.1	*Lachnospiraceae bacterium*	VanZ-1
MBS5788172.1	*Clostridioides difficile*	VanZ-1
MBQ7972388.1	*Lachnospiraceae bacterium*	VanZ-1
MBD5551227.1	*Lachnospiraceae bacterium*	VanZ-1
NLL75820.1	*Clostridiales bacterium*	VanZ-1
MBD5493789.1	*Lachnospiraceae bacterium*	VanZ-1
WP_160941526.1	*Eggerthella guodeyinii*	VanZ-1
WP_155550097.1	*Paraclostridium sordellii*	VanZ-1
MBD5492727.1	*Lachnospiraceae bacterium*	VanZ-1
WP_066730942.1	*uncultured Bariatricus sp.*	VanZ-1
HIV03161.1	*Candidatus Aphodoplasma excrementigallinarum*	VanZ-1
MBQ8199169.1	*Lachnospiraceae bacterium*	VanZ-1
OQB24683.1	*Firmicutes bacterium ADurb.Bin182*	VanZ-1
MBR5430331.1	*Bacillota bacterium*	VanZ-1
PWM33956.1	*Clostridiales bacterium*	VanZ-1
RAQ28137.1	*Hydrogeniiclostridium mannosilyticum*	VanZ-1

#;TM+TM+VanZ-1

WP_061466429.1	*Peribacillus simplex*	VanZ-1
MBU1726384.1	*Candidatus Omnitrophota bacterium*	VanZ-1
HCI28825.1	*Fervidobacterium sp.*	VanZ-1

#;TM+VanZ

MBS6283035.1	*Oscillospiraceae bacterium*	VanZ-1
MBS4916191.1	*Clostridiales bacterium*	VanZ-1
CDC61335.1	*Clostridium sp. CAG:448*	VanZ-1
WP_205186164.1	*Priestia iocasae*	VanZ-1
CUX32703.1	*Clostridium sp. C105KSO15*	VanZ-1
NCB63877.1	*Clostridia bacterium*	VanZ-1
TMA48935.1	*Deltaproteobacteria bacterium*	VanZ-1
MBQ7591270.1	*Clostridia bacterium*	VanZ-1
PEJ60539.1	*Bacillus sp. AFS002410*	VanZ-1
MBO4791979.1	*Clostridia bacterium*	VanZ-1
WP_087456657.1	*Tumebacillus avium*	VanZ-1
WP_015310700.1	*Thermoanaerobacterium thermosaccharolyticum M0795*	VanZ-1
WP_144989566.1	*Paenibacillus agilis*	VanZ-1
WP_106508087.1	*Brachybacterium timonense*	VanZ-1
WP_100205321.1	*Streptomyces carminius*	VanZ-1
WP_143649754.1	*Streptomyces alkaliterrae*	VanZ-1
PWL42445.1	*Clostridiales bacterium*	VanZ-1
WP_170154021.1	*Actinoplanes italicus*	VanZ-1
KAB1864463.1	*Microbacterium algeriense*	VanZ-1
MBR2892446.1	*Clostridia bacterium*	VanZ-1
MBQ6118525.1	*Clostridia bacterium*	VanZ-1
WP_144755243.1	*Amycolatopsis acidicola*	VanZ-1

#;TM+TM+beta-hairpin+VanZ-1

MBQ8763431.1	*Clostridia bacterium*	beta-hairpin VanZ-1 ? ? ? ? ? VanZ-1

#;VanZ-1+disordered

BAU85646.1	*Streptomyces laurentii*	VanZ-1 disordered
MQS37373.1	*Streptomyces katsurahamanus*	VanZ-1 disordered
WP_141635622.1	*Kitasatospora acidiphila*	VanZ-1 disordered
GHH40878.1	*Streptomyces candidus*	VanZ-1 disordered

#;VanZ-1+disordered

MRX44538.1	*Agromyces kandeliae*	VanZ-1 disordered
WP_100573380.1	*Streptomyces sp. CB01201*	VanZ-1 disordered
WP_224737221.1	*Streptomyces sp. PSKA30*	VanZ-1 disordered
TWE17691.1	*Kitasatospora atroaurantiaca*	VanZ-1 disordered
GHA20979.1	*Streptomyces spiroverticillatus*	VanZ-1 disordered
RKN05267.1	*Streptomyces radicis*	VanZ-1 disordered
WP_168220752.1	*Streptomyces sp. RFCAC02*	VanZ-1 disordered
WP_141583184.1	*Streptomyces ipomoeae*	VanZ-1 disordered
WP_206318328.1		VanZ-1 disordered
WP_014136141.1	*Kitasatospora sp. SID7827*	VanZ-1 disordered
CCA57857.1	*Streptomyces venezuelae ATCC 10712*	VanZ-1 disordered
WP_077059414.1	*Streptomyces sp. MP131-18*	VanZ-1 disordered

#;VanZ-1+TM

MBR6959113.1	*Clostridiales bacterium*	VanZ-1
WP_051259824.1	*Corynebacterium sputi DSM 45148*	VanZ-1
MBP6544706.1	*Piscinibacter sp.*	VanZ-1
MBO5907112.1	*Clostridia bacterium*	VanZ-1
MBQ3016677.1	*Clostridia bacterium*	VanZ-1

#;VanZ-1+TM+TM

NNM61142.1	*Steroidobacteraceae bacterium*	VanZ-1

MBS0326150.1 — VanZ-1
Pseudomonadota bacterium

#;TM+VanZ-1

WP_116199426.1 — VanZ-1
Amycolatopsis circi

WP_204092503.1 — VanZ-1
Amycolatopsis sp. FDAARGOS 1241

RPJ59330.1 — VanZ-1
Acidobacteriota bacterium

#;TM+VanZ-1+disordered

BCJ53212.1 — VanZ-1 disordered
Actinoplanes sp. NBRC 14428

WP_195098034.1 — VanZ-1 disordered
Nocardia gipuzkoensis

#;TM+VanZ-1

WP_062551708.1 — VanZ-1
Peptoniphilus phoceensis

SLF17567.1 — VanZ-1
Mycobacteroides abscessus subsp. bolletii

MBZ0294933.1 — VanZ-1
Anaerolineae bacterium

WP_106317411.1 — VanZ-1
Actinoplanes italicus

MBE0418372.1 — VanZ-1
Coriobacteriia bacterium

MBQ2694205.1 — VanZ-1
Clostridia bacterium

WP_204842610.1 — VanZ-1
Catellatospora bangladeshensis

WP_142652095.1 — VanZ-1
Microbispora hainanensis

MBO7360868.1 — VanZ-1
Clostridia bacterium

GFE05017.1 — VanZ-1
Streptomyces caniferus

WP_161837642.1 — VanZ-1
Anaerocolumna sedimenticola

WP_100202529.1 — VanZ-1
Streptomyces carminius

WP_143864011.1 — VanZ-1
Brevibacillus sp. LEMMJ03

WP_130001331.1 — VanZ-1

KAB8145863.1 — VanZ-1
Chloroflexia bacterium SDU3-3

WP_195615448.1 — VanZ-1
Intestinimonas butyriciproducens

#;TM+TM+VanZ-1

MBD5103952.1 — VanZ-1
Oscillospiraceae bacterium

MCA0456829.1 — VanZ-1
Chloroflexota bacterium

WP_141994339.1 — VanZ-1
Bacillus sp. B4EP4a

MBA3872964.1 — VanZ-1
Anaerolineae bacterium

#;VanZ-1+TM

AKA97678.1 — VanZ-1
Corynebacterium ulcerans

#;TM+VanZ-1+disordered

QFU94411.1 — VanZ-1 disordered
Amycolatopsis sp. YIM 10

#;TM+VanZ-1+TM

HHW62191.1 — VanZ-1
Syntrophomonadaceae bacterium

MBQ9031145.1 — VanZ-1
Parasporobacterium sp.

ERL12817.1 — VanZ-1
Coriobacteriaceae bacterium BV3Ac1

WP_009319829.1 — VanZ-1 GalE/RmlB/Gmd-like_lyase/epimerase
[Ruminococcus] torques

WP_007882603.1 — VanZ-1
Roseburia inulinivorans DSM 16841

MBS5084054.1 — VanZ-1
Clostridiales bacterium

WP_067629804.1 — VanZ-1
Erysipelothrix larvae

WP_163067535.1 — VanZ-1
Aminipila butyrica

WP_155486240.1 — VanZ-1
Paraclostridium sordellii

HBA96623.1 — VanZ-1
Lachnospiraceae bacterium

WP_087244602.1 — VanZ-1
Massilimicrobiota sp. An142

WP_051540465.1 — VanZ-1
Clostridium ihumii

GFI36763.1 — VanZ-1
Lachnospiraceae bacterium

MBS6525634.1 — VanZ-1
Peptoniphilaceae bacterium

SCI95074.1 — VanZ-1
uncultured Clostridium sp.

WP_087913515.1 — VanZ-1
Paenibacillus donghaensis

OXX82983.1 — VanZ-1
Paraclostridium benzoelyticum

WP_066676425.1 — VanZ-1
Clostridium septicum

WP_216108293.1 — VanZ-1
Clostridium estertheticum

MBR1740512.1 — VanZ-1
Lachnospiraceae bacterium

WP_213234894.1 — VanZ-1
Fusibacter paucivorans

WP_161837973.1 — VanZ-1
Anaerocolumna sedimenticola

#;TM+TM+VanZ-2+TM

MBD3319623.1 — VanZ-1
Chitinivibrionales bacterium

#;TM+VanZ-1

ONI92192.1 — VanZ-1
Saccharothrix sp. ALI-22-I

WP_161832563.1 — VanZ-1
Turicibacter faecis

GAD35223.1 — VanZ-1
Microbacterium sp. TS-1

PZF79996.1 — VanZ-1
Jiangella anatolica

RHL27044.1 — VanZ-1
Agathobacter rectalis

RSK31941.1 — VanZ-1
Bhargavaea beijingensis

EEG55335.1 — VanZ-1
[Clostridium] asparagiforme DSM 15981

PIQ36578.1 — VanZ-1
Lysobacterales bacterium CG17_big_fil_post_rev_8_21_14_2_50_64_11

MCD7981824.1 — VanZ-1
Clostridiales bacterium

MBP3908403.1 — VanZ-1
Turicibacter sp.

PGZ94223.1 — VanZ-1
Bacillus sp. AFS029533

WP_117361427.1 — VanZ-1
Actinomadura logoneensis

HEM24314.1 — VanZ-1

WP_143111248.1 — GalE/RmlB/Gmd-like_lyase/epimerase GDP-L-fucose-synthase ? ? VanZ-1
Enterocloster citroniae

MYA05004.1 — VanZ-1
Caldilineaceae bacterium SB0664_bin_22

#;disordered+VanZ-1

OWP20948.1 — disordered VanZ-1
Microbacterium sp. AIS03

WP_191794516.1 — disordered VanZ-1

WP_161181010.1 — disordered VanZ-1
Streptomyces sp. SID4985

SFJ36357.1 — disordered VanZ-1
Paenibacillus sp. UNC496MF

PYS99411.1 — disordered VanZ-1
Acidobacteriota bacterium

WP_051527067.1 — disordered VanZ-1
[Eubacterium] cellulosolvens LD2006

#;TM+VanZ-1

MBQ2061793.1 — VanZ-1 VanZ-1
Oscillospiraceae bacterium

#;TM+VanZ-1+TM

MCD7964495.1 — VanZ-1
Clostridiaceae bacterium

#;VanZ-1+TM

WP_154831008.1 — VanZ-1

WP_099723859.1 — VanZ-1
Leucobacter sp. OAMLP11

#;VanZ-1

MBN8879498.1 — VanZ-1
Sphingobacteriales bacterium

RAJ86399.1 — VanZ-1
Streptomyces sp. PsTaAH-137

MQY15892.1 — VanZ-1
Streptomyces smaragdinus

GHD58449.1 — VanZ-1
Streptomyces goshikiensis

#;TM+VanZ-1+TM

HIZ62183.1 — VanZ-1
Candidatus Gemmiger avistercoris

WP_006861283.1 — VanZ-1
Ruminococcus sp. Marseille-P328

MBQ9227394.1 *Eubacterium sp.*	VanZ-1
RKI75666.1 *bacterium 1xD42-87*	VanZ-1
**#;TM+VanZ-1**	
WP_215115173.1 *Exiguobacterium sp. s80*	VanZ-1
**#;VanZ-1+TM**	
MBQ7407678.1 *Clostridia bacterium*	VanZ-1
MBQ3014766.1 *Clostridia bacterium*	VanZ-1 VanZ-1
MBQ3014765.1 *Clostridia bacterium*	VanZ-1 VanZ-1
**#;TM+VanZ-1+TM**	
HAM31046.1 *Erysipelotrichaceae bacterium*	VanZ-1
OJU09691.1 *Clostridiales bacterium 43-6*	VanZ-1
WP_163225468.1 *Clostridium sporogenes*	VanZ-1
MBD5144071.1 *Ruminococcus sp.*	VanZ-1
HIT94235.1 *Candidatus Faecivivens stercoripullorum*	VanZ-1
RKJ75568.1 *Butyricicoccus sp. 1XD8-22*	VanZ-1
MBQ3287388.1 *Bacillota bacterium*	VanZ-1
MBQ6406743.1 *Butyrivibrio sp.*	VanZ-1
MBR1701009.1 *Lachnospiraceae bacterium*	VanZ-1
SCJ34326.1 *uncultured Clostridium sp.*	VanZ-1
HJC21279.1 *Candidatus Butyricicoccus avicola*	VanZ-1
WP_063602606.1 *Clostridium coskatii*	VanZ-1
TDA22394.1 *Extibacter muris*	VanZ-1
HIV87402.1 *Candidatus Pygmaiobacter gallistercoris*	VanZ-1
MBQ3637122.1 *Clostridia bacterium*	VanZ-1
TGY64428.1 *Dubosiella muris*	VanZ-1
WP_051643210.1 *Agathobaculum desmolans ATCC 43058*	VanZ-1
MBR5343692.1 *Oscillospiraceae bacterium*	VanZ-1
HIY38964.1 *Candidatus Agathobaculum merdigallinarum*	VanZ-1
MBR3524765.1 *Lachnospiraceae bacterium*	VanZ-1
MBQ2873124.1 *Bacilli bacterium*	VanZ-1
HIT07962.1 *Candidatus Merdivicinus faecavium*	VanZ-1
NBH82225.1 *Lachnospiraceae bacterium*	VanZ-1
HCR43240.1 *Oscillospiraceae bacterium*	VanZ-1
WP_013625888.1 *Syntrophobotulus glycolicus DSM 8271*	VanZ-1
WP_195975474.1 *Hydrogeniiclostridium mannosilyticum*	VanZ-1
HIU43210.1 *Candidatus Ventrousia excrementavium*	VanZ-1
EOS69619.1 *Lachnospiraceae bacterium MD308*	VanZ-1
WP_101695768.1 *Dorea phocaeensis*	VanZ-1
**#;VanZ-1+TM**	
WP_185176764.1 *Corynebacterium incognita*	VanZ-1
**#;TM+VanZ-1+TM**	
MBS6395961.1 *Clostridiales bacterium*	VanZ-1
MBR7042137.1 *Bacilli bacterium*	VanZ-1
MBR1416404.1 *Bacilli bacterium*	VanZ-1
MBR3039163.1 *Lachnospiraceae bacterium*	VanZ-1
MBQ8219083.1 *Bacilli bacterium*	VanZ-1
MBR4619129.1 *Bacilli bacterium*	VanZ-1
TLD01883.1 *Robinsoniella peoriensis*	VanZ-1
MBE6148813.1 *Bacillota bacterium*	VanZ-1
MBQ7240275.1 *Bacilli bacterium*	VanZ-1
MBS5142371.1 *Bacillota bacterium*	VanZ-1
HHX52431.1 *Erysipelothrix sp.*	VanZ-1
WP_087157683.1 *Lachnoclostridium sp. An181*	VanZ-1
MBE5879354.1 *Lachnospiraceae bacterium*	VanZ-1
WP_154274047.1 *Enterococcus faecalis*	VanZ-1
MBQ9954184.1 *Eggerthellaceae bacterium*	VanZ-1
MBR3503024.1 *Clostridia bacterium*	VanZ-1
NLA33579.1 *Mycoplasmatota bacterium*	VanZ-1
HBA37360.1 *Bacillota bacterium*	VanZ-1
MBQ4031370.1 *Bacilli bacterium*	VanZ-1
MBQ6494516.1 *Bacilli bacterium*	VanZ-1
HIU23063.1 *Candidatus Fimihabitans intestinipullorum*	VanZ-1
MBR5662574.1 *Bacilli bacterium*	VanZ-1
MBQ8193188.1 *Bacilli bacterium*	VanZ-1
MBR1718156.1 *Bacilli bacterium*	VanZ-1
HJC36773.1 *Candidatus Merdibacter merdavium*	VanZ-1
MBQ4262630.1 *Bacilli bacterium*	VanZ-1
HIS18039.1 *Candidatus Coprovivens excrementavium*	VanZ-1
RDB56361.1 *Paraeggerthella hongkongensis*	VanZ-1
MBR6949143.1 *Bacilli bacterium*	VanZ-1
MBS7020284.1 *Bacillota bacterium*	VanZ-1
CDE99445.1 *Clostridium sp. CAG:628*	VanZ-1
HBP13186.1 *Bacillota bacterium*	VanZ-1
MBO6195238.1 *Bacilli bacterium*	GalE/RmlB/Gmd-like_lyase/epimerase ? ? ? VanZ-1
MCD8280882.1 *Enterocloster citroniae*	VanZ-1
MBP3623360.1 *Oscillospiraceae bacterium*	VanZ-1
HIR87245.1 *Candidatus Limivicinus faecipullorum*	VanZ-1
CUO07176.1 *Hungatella hathewayi*	VanZ-1
HIT94699.1 *Candidatus Faecivivens stercoripullorum*	VanZ-1
MBE6725582.1 *Oscillospiraceae bacterium*	VanZ-1
WP_075884537.1 *Faecalibaculum rodentium*	VanZ-1
MBO5523889.1 *Roseburia sp.*	VanZ-1
MBD9074573.1	VanZ-1
MBR2833821.1 *Bacilli bacterium*	VanZ-1
CDB92135.1 *Clostridium sp. CAG:302*	VanZ-1
MBQ1551260.1 *Bacilli bacterium*	VanZ-1
HHX93985.1 *Mycoplasmatota bacterium*	VanZ-1
MBQ7790108.1 *Bacilli bacterium*	VanZ-1
MBO4245308.1 *Bacilli bacterium*	VanZ-1
MBO6145604.1 *Bacilli bacterium*	VanZ-1
MBQ8534352.1 *Bacilli bacterium*	VanZ-1
MBT8207639.1 *Acidimicrobiia bacterium*	VanZ-1
WP_060815471.1	VanZ-1
MBQ8681691.1 *Bacilli bacterium*	VanZ-1

WP_009004012.1	*Clostridium sp. D5*	VanZ-1
NLY09164.1	*Tissierellia bacterium*	VanZ-1
HIS87388.1	*Candidatus Caccenecus avistercoris*	VanZ-1
MBE5990482.1	*Paenibacillaceae bacterium*	VanZ-1
WP_049898098.1	*Ruminococcus gauvreauii*	VanZ-1
WP_074759283.1		VanZ-1
MBS5862809.1	*Bacillota bacterium*	VanZ-1
MBR3661357.1	*Bacilli bacterium*	VanZ-1
HIU40693.1	*Candidatus Aphodocola excrementigallinarum*	VanZ-1
CDA52610.1	*Clostridium sp. CAG:533*	VanZ-1
HIT35472.1	*Candidatus Faecousia intestinavium*	VanZ-1
MBE5967314.1	*Lachnospiraceae bacterium*	VanZ-1
MBP3312887.1	*Butyricicoccus sp.*	lexA-like-peptidase, ?, VanZ-1, GalE/RmlB/Gmd-like_lyase/epimerase
MBS5703461.1	*Butyricicoccus pullicaecorum*	VanZ-1, GalE/RmlB/Gmd-like_lyase/epimerase
HAK43998.1	*Clostridium sp.*	VanZ-1
MBP3487501.1	*Roseburia sp.*	sugar-O-acetyl/acyltransf, ?, ?, ?, ?, ?, ?, VanZ-1
MBP3327972.1	*Clostridia bacterium*	WecC-like-oxidoreductase, VanZ-1, GalE/RmlB/Gmd-like_lyase/epimerase
MBR6094297.1	*Lachnospiraceae bacterium*	VanZ-1
MBP3444351.1	*Bacilli bacterium*	VanZ-1
PWL73577.1	*Bacillota bacterium*	VanZ-1
MBR1377223.1	*Bacilli bacterium*	VanZ-1
MBE6147129.1	*Bacillota bacterium*	VanZ-1
MBO4601071.1	*Bacilli bacterium*	VanZ-1
WP_118476571.1		sugar-O-acetyl/acyltransf, ?, VanZ-1
MBQ3297660.1	*Bacilli bacterium*	VanZ-1
HIU75403.1	*Candidatus Pelethocola excrementipullorum*	VanZ-1
EGT3615146.1	*Clostridium perfringens*	VanZ-1
WP_050741824.1	*Acetobacterium bakii*	VanZ-1
HIQ90025.1	*Candidatus Coprosoma intestinipullorum*	VanZ-1
WP_012260465.1	*Bacillus mycoides KBAB4*	VanZ-1
MBR3523592.1	*Bacilli bacterium*	VanZ-1
CCZ89122.1	*Coprobacillus sp. CAG:605*	VanZ-1
NLV89873.1	*Mycoplasmatota bacterium*	VanZ-1
MBQ9710090.1	*Clostridia bacterium*	VanZ-1
WP_007050316.1	*Anaerofustis stercorihominis*	VanZ-1
MBR3898448.1	*Bacilli bacterium*	VanZ-1
NBI87286.1	*Lachnospiraceae bacterium*	VanZ-1
SHN69418.1	*Desulfitobacterium chlororespirans DSM 11544*	VanZ-1
MBE6153325.1	*Bacillota bacterium*	VanZ-1
MBE6724603.1	*Oscillospiraceae bacterium*	VanZ-1
WP_215677627.1	*Roseburia inulinivorans*	VanZ-1
OGW76097.1	*Omnitrophica bacterium RBG_13_46_9*	VanZ-1
MBD9106113.1	*bacterium*	VanZ-1
MBQ7140702.1	*Bacilli bacterium*	VanZ-1
MBR3199073.1	*Bacilli bacterium*	VanZ-1
CCY88741.1	*Mycoplasma sp. CAG:956*	VanZ-1
MBO5169669.1	*Oscillospiraceae bacterium*	VanZ-1
MBQ8901894.1	*Bacilli bacterium*	VanZ-1
MBE6151040.1	*Bacillota bacterium*	VanZ-1
WP_050639972.1	*Faecalicatena contorta*	VanZ-1
WP_026883508.1	*Clostridium akagii DSM 12554*	VanZ-1
CCY26884.1	*Firmicutes bacterium CAG:114*	VanZ-1
WP_058953639.1	*Clostridium tyrobutyricum*	VanZ-1
HIQ94764.1	*Candidatus Ventrenecus stercoripullorum*	VanZ-1
WP_142433140.1	*Enterococcus mundtii*	VanZ-1
WP_153830158.1	*Enterococcus mundtii*	VanZ-1
MBD9114793.1	*bacterium*	VanZ-1
HIR61803.1	*Candidatus Faecivivens stercoravium*	VanZ-1
MBR1414140.1	*Bacilli bacterium*	VanZ-1

#;VanZ-1+TM

MBO7274018.1	*Clostridia bacterium*	VanZ-1
MBQ7356391.1	*Clostridia bacterium*	VanZ-1
MBR2956139.1	*Clostridia bacterium*	VanZ-1
MBE5764768.1	*Clostridiales bacterium*	VanZ-1, VanZ-1
MBQ2891325.1	*Bacilli bacterium*	VanZ-1
MBR3684993.1	*Clostridia bacterium*	VanZ-1
MBP5261561.1	*Clostridiales bacterium*	VanZ-1
MBQ2914897.1	*Clostridia bacterium*	VanZ-1
MBR2484883.1	*Clostridia bacterium*	VanZ-1
MCD8502893.1	*Bacillaceae bacterium*	VanZ-1
MCC8022185.1	*Clostridiales bacterium*	VanZ-1

#;VanZ-1+TM

TCK41716.1	*Leucobacter luti*	VanZ-1
BCJ28736.1	*Actinocatenispora sera*	VanZ-1
WP_183298017.1	*Cellulomonas cellasea*	VanZ-1
MBN9612348.1	*Actinomycetota bacterium*	VanZ-1

#;TM+VanZ-1+TM

HAU85161.1	*Lachnospiraceae bacterium*	VanZ-1
HBN56939.1	*Lachnospiraceae bacterium*	VanZ-1
MCA5963160.1	*Blautia parvula*	VanZ-1
MBQ9866581.1	*Lachnospiraceae bacterium*	VanZ-1
MBP3198773.1	*Butyrivibrio sp.*	VanZ-1
PWJ23157.1	*Faecalicatena orotica*	VanZ-1
HIV12598.1	*Candidatus Pullilachnospira stercoravium*	VanZ-1
MBR5430563.1	*Bacillota bacterium*	VanZ-1

#;VanZ-1+TM

NMB23515.1	*Corynebacterium sp.*	VanZ-1
HCG47286.1	*Corynebacterium flavescens*	VanZ-1

#;TM+VanZ-1

WP_067836624.1	*Amphibacillus sediminis NBRC 103570*	VanZ-1
MBD5518480.1	*Lachnospiraceae bacterium*	VanZ-1
NOK64351.1	*Chloroflexi bacterium AL-N1*	VanZ-1
WP_224146323.1	*Bacillus paralicheniformis*	VanZ-1
WP_195839064.1	*Anaerosalibacter bizertensis*	VanZ-1

WP_231788893.1	*Metabacillus kandeliae*	VanZ-1
WP_171464747.1	*Bacillus safensis*	VanZ-1
MBI1793781.1	*Chloroflexota bacterium*	VanZ-1
MBG9567016.1	*Brevibacillus agri*	VanZ-1
MAS35097.1	*Anaerolineaceae bacterium*	VanZ-1
WP_214803827.1	*Bacillus sp. ISL-51*	VanZ-1
PGT19818.1	*Bacillus cereus*	VanZ-1
MBE6995228.1	*Oscillospiraceae bacterium*	VanZ-1
WP_078380679.1	*Sutcliffiella halmapala*	VanZ-1
MBS6510572.1	*Clostridiales bacterium*	VanZ-1
MBS3975027.1	*Actinomycetota bacterium*	VanZ-1
MBQ9329925.1	*Oscillibacter sp.*	VanZ-1
WP_078173633.1	*Bacillus sp. FSL M7-1345*	VanZ-1
WP_140417264.1	*Bacillus cereus*	VanZ-1
WP_142316293.1	*Bacillus toyonensis*	VanZ-1
WP_000281848.1	*Bacillus pacificus*	VanZ-1
OUB49467.1	*Bacillus thuringiensis serovar higo*	VanZ-1
WP_140163808.1	*Bacillus thuringiensis serovar higo*	VanZ-1
WP_223493756.1	*Sutcliffiella horikoshii*	VanZ-1
ALG15633.1	*Kibdelosporangium phytohabitans*	VanZ-1
WP_154761221.1	*Amycolatopsis pithecellobii*	VanZ-1
MBS1797421.1	*Acidobacteriota bacterium*	VanZ-1
NCC87514.1	*Clostridia bacterium*	VanZ-1
WP_212832904.1	*Catellatospora sp. TT07R-123*	VanZ-1
MBR6507870.1	*Clostridia bacterium*	VanZ-1
WP_067825837.1	*Nocardia inohanensis NBRC 100128*	VanZ-1
WP_233035271.1	*Nocardia yamanashiensis*	VanZ-1
NLO26719.1	*Actinomycetota bacterium*	VanZ-1
MBQ1598814.1	*Lachnospiraceae bacterium*	VanZ-1
WP_224241501.1	*Hyalangium gracile*	VanZ-1
WP_164491624.1	*Staphylospora marina*	VanZ-1
WP_101524652.1	*Nocardioides houyundeii*	VanZ-1
MTO23995.1	*Turicibacter sanguinis*	VanZ-1
MBR3634553.1	*Lachnospiraceae bacterium*	VanZ-1
MBP3600578.1	*Clostridia bacterium*	VanZ-1
HCI66033.1	*Lachnospiraceae bacterium*	VanZ-1
MBQ9885501.1	*Lachnospiraceae bacterium*	VanZ-1
SCW38090.1	*Lachnospiraceae bacterium C10*	VanZ-1
OGX15055.1	*Omnitrophica WOR_2 bacterium RBG_13_41_10*	VanZ-1
EOS34645.1	*Lachnospiraceae bacterium A4*	VanZ-1
MBE5869923.1	*Lachnospiraceae bacterium*	VanZ-1
NBK16570.1		VanZ-1
OQY90090.1	*Anaerolineae bacterium UTCFX1*	VanZ-1
MBN1883403.1	*Candidatus Zymogenaceae bacterium*	VanZ-1
MBI2758633.1	*Chloroflexota bacterium*	VanZ-1
WP_026316361.1	*Actinokineospora enzanensis DSM 44649*	VanZ-1
PKN84951.1	*Chloroflexi bacterium HGW-Chloroflexi-8*	VanZ-1
NMB67234.1	*Chloroflexota bacterium*	VanZ-1
NUU24311.1	*Streptomycetaceae bacterium*	VanZ-1
MBK8138706.1	*Candidatus Flexicrinis proximus*	VanZ-1
WP_106403010.1	*Actinocorallia populi*	VanZ-1
WP_067281985.1	*Streptomyces jeddahensis*	VanZ-1
WP_181863164.1	*Streptomyces himalayensis subsp. aureolus*	VanZ-1
WP_224938065.1	*Cytobacillus sp. FSL W7-1323*	VanZ-1
NTW44555.1	*Anaerolineaceae bacterium*	VanZ-1
WP_164410857.1	*Streptomyces sp. SID12488*	VanZ-1
WP_118212496.1	*[Ruminococcus] lactaris*	VanZ-1
WP_076114205.1	*Paenibacillus sp. sgz500992*	VanZ-1
MBD5449357.1	*Lachnospiraceae bacterium*	VanZ-1
MBQ9229497.1	*Eubacterium sp.*	VanZ-1
WP_051082826.1	*Microbacterium sp. 77mftsu3.1*	VanZ-1
GIE46832.1	*Actinoplanes nipponensis*	VanZ-1
WP_052103332.1	*Cellulomonas cellasea*	VanZ-1
MBR4759283.1	*Lachnospiraceae bacterium*	VanZ-1
MBP9852293.1	*Candidatus Paceibacterota bacterium*	VanZ-1
RQD69217.1	*Tindallia sp. MSAO_Bac2*	VanZ-1
PKQ33438.1	*Actinobacteria bacterium HGW-Actinobacteria-11*	VanZ-1
NYG40503.1		VanZ-1
MBE5901836.1	*Lachnospiraceae bacterium*	VanZ-1
WP_204888481.1		VanZ-1
OGO57012.1	*Chloroflexi bacterium RBG_16_70_13*	VanZ-1
WP_016321196.1	*Oscillibacter sp. 1-3*	VanZ-1
WP_192204168.1	*Peribacillus simplex*	VanZ-1
WP_062422153.1	*Leptolinea tardivitalis*	VanZ-1
WP_087297307.1	*Massilimicrobiota sp. An134*	VanZ-1
WP_195607553.1	*Intestinimonas butyriciproducens*	VanZ-1
MBQ9673655.1	*Ruminococcus sp.*	VanZ-1
MBR1390329.1	*Lachnospiraceae bacterium*	VanZ-1
SDP88425.1	*Lentzea jiangxiensis*	VanZ-1
WP_102157654.1	*Zhihengliuella halotolerans*	VanZ-1
MYB78002.1	*Acidimicrobiia bacterium*	VanZ-1
HIU94781.1	*Candidatus Aphodomorpha intestinavium*	VanZ-1
WP_232398558.1	*Aeromicrobium wangtongii*	VanZ-1
WP_156758701.1	*Actinokineospora pegani*	VanZ-1
SDP35307.1	*Klenkia soli*	VanZ-1
WP_141162190.1	*Schumannella soli*	VanZ-1
HAB94707.1	*Lachnospiraceae bacterium*	VanZ-1
WP_143538094.1	*Saccharopolyspora erythraea*	VanZ-1
MBO9556700.1	*Cellulomonas sp.*	VanZ-1
WP_106178957.1		VanZ-1
SFJ59227.1	*Cellulomonas sp. KH9*	VanZ-1
MBR4724536.1	*Lachnospiraceae bacterium*	VanZ-1
MQA25112.1	*Micromonosporaceae bacterium*	VanZ-1

PZS17900.1 *Pseudonocardiales bacterium* — VanZ-1

MBR1930764.1 *Lachnospiraceae bacterium* — VanZ-1

MBQ2100836.1 *Lachnospiraceae bacterium* — VanZ-1

MBR1761104.1 *Schwartzia sp. (in: firmicutes)* — VanZ-1

WP_009950518.1 *Saccharopolyspora erythraea* — VanZ-1

#;VanZ-1+TM

AHI03396.1 *Corynebacterium falsenii DSM 44353* — VanH-like ? VanZ-1

EKX88600.1 *Corynebacterium durum F0235* — VanZ-1

BAU97364.1 *Corynebacterium suranareeae* — VanZ-1

SPJ42324.1 *Corynebacterium diphtheriae subsp. lausannense* — VanZ-1

#;VanZ-1

MBE5957224.1 *Lachnospiraceae bacterium* — VanZ-1

#;VanZ-1+TM

APT89726.1 *Corynebacterium frankenforstense DSM 45800* — VanZ-1

#;VanZ-1

WP_196198224.1 *Streptacidiphilus fuscans* — VanZ-1

WP_156758983.1 *Microbacterium karelineae* — VanZ-1

#;TM+TM+VanZ-1

HBY91928.1 *Streptococcus sp.* — VanZ-1

WP_003090988.1 *Streptococcus ratti* — VanZ-1

EGP5893539.1 — VanZ-1

WP_088087741.1 *Bacillus sp. OV166* — VanZ-1

WP_090765328.1 *Bacillus sp. OK048* — VanZ-1

MCD8502957.1 *Bacillaceae bacterium* — VanZ-1

WP_215001294.1 *Bacillus sp. ISL-7* — VanZ-1

WP_115749350.1 *Oceanobacillus chungangensis* — VanZ-1

RWZ54762.1 *Halobacillus fulvus* — VanZ-1

WP_088076693.1 *Evansella alkalicola* — VanZ-1

WP_066265052.1 *Heyndrickxia acidicola* — VanZ-1

WP_026576085.1 *Bacillus sp. UNC438CL73TsuS30* — VanZ-1

RGJ72134.1 *Eubacterium sp. TM05-53* — VanZ-1

MBR6173457.1 *Eubacterium sp.* — VanZ-1 ? sugar-transferase-GT-A

WP_171311425.1 *Enterococcus cecorum* — VanZ-1

HHW94750.1 *Mogibacterium sp.* — VanZ-1

ANU25820.1 *Planococcus versutus* — VanZ-1

WP_194350776.1 *Listeria seeligeri* — VanZ-1

SCH12257.1 *uncultured Ruminococcus sp.* — VanZ-1

MBD9120784.1 *Oscillospiraceae bacterium* — VanZ-1

WP_196321160.1 *Bifidobacterium dentium* — VanZ-1

OCN06153.1 *Erysipelotrichaceae bacterium MTC7* — VanZ-1

WP_066870738.1 *Clostridium mediterraneense* — VanZ-1 VanZ-1

WP_120745965.1 *Paenibacillus ginsengarvi* — VanZ-1

EFU74927.1 *Enterococcus italicus DSM 15952* — VanZ-1

WP_014123873.1 *Tetragenococcus halophilus* — VanZ-1

WP_161843336.1 *Candidatus Enterococcus huntleyi* — VanZ-1

WP_016182414.1 *Enterococcus columbae DSM 7374 = ATCC 51263* — VanZ-1

WP_214358246.1 *Bifidobacterium santillanense* — VanZ-1

WP_100043526.1 *Lacrimispora sphenoides* — VanZ-1

WP_065531148.1 *[Clostridium] innocuum* — VanZ-1

WP_204595009.1 *Clostridium sardiniense* — VanZ-1

ERI95401.1 *Clostridiales bacterium oral taxon 876 str. F0540* — VanZ-1

WP_051317000.1 *Ectobacillus panaciterrae DSM 19096* — VanZ-1

#;TM+VanZ-1

CAG7586438.1 *Peptoniphilus tyrrelliae* — VanZ-1

#;TM+TM+VanZ-1

MCB5927231.1 *bacterium 210820-DFI.5.26* — VanZ-1

MBS4879632.1 *Bacillota bacterium* — VanZ-1

#;TM+TM+VanZ-1

MBS5704230.1 *Butyricicoccus pullicaecorum* — VanZ-1

MBS7224349.1 *Clostridiaceae bacterium* — VanZ-1

MUL64958.1 *Mycolicibacterium sp. CBMA 234* — VanZ-1

WP_162460182.1 *Mycolicibacterium sp. CBMA 213* — VanZ-1

#;TM+VanZ-1

MBW8769415.1 *Gemmatimonadota bacterium* — VanZ-1

PYO79041.1 *Gemmatimonadota bacterium* — VanZ-1

NUO37321.1 *Gemmatimonadaceae bacterium* — VanZ-1

OUQ26366.1 *Flavonifractor sp. An135* — VanZ-1

#;TM+VanZ-1

WP_118048814.1 *Blautia obeum* — VanZ-1

WP_205154678.1 — VanZ-1

WP_204647928.1 *Gemmiger formicilis* — VanZ-1

CDB69471.1 *Eubacterium sp. CAG:252* — VanZ-1

EFF28745.1 *Enterococcus faecium U0317* — VanZ-1

HJC63747.1 *Candidatus Blautia merdavium* — VanZ-1

WP_118486150.1 *Anaerobutyricum hallii* — VanZ-1

MBD9219579.1 *Clostridiales bacterium* — VanZ-1

WP_118170487.1 — VanZ-1

EFV17027.1 *Lachnospiraceae bacterium 5_1_63FAA* — MviN_MATE-domain-flippase polysacc-pyruvyl-transf ? ? VanZ-1

MBP5772770.1 *Eubacterium sp.* — polysacc-pyruvyl-transf ? ? MviN_MATE-domain-flippase polysacc-pyruvyl-transf sugar-transferase-GT-A ? ? VanZ-1

MBQ7655236.1 *Clostridia bacterium* — VanZ-1

MBR3173777.1 *Eubacterium sp.* — MviN_MATE-domain-flippase ? ? ? VanZ-1

HIQ99439.1 *Candidatus Scybalocola faecavium* — VanZ-1

WP_183776011.1 *Catenibacillus scindens* — VanZ-1

MBS6921946.1 *Lachnospiraceae bacterium* — GalE/RmlB/Gmd-like_lyase/epimerase GDP-L-fucose-synthase ? ? ? VanZ-1

WP_128519861.1 *Absicoccus porci* — VanZ-1

HIZ43622.1 *Bacillota bacterium* — VanZ-1

#;3-helix+VanZ-1

CDB74510.1 *Clostridium sp. CAG:265* — 3-helix VanZ-1

#;TM+VanZ-1

MQM73055.1 *Candidatus Pseudoramibacter fermentans* — GalE/RmlB/Gmd-like_lyase/epimerase GalE/RmlB/Gmd-like_lyase/epimerase ? ? tRNA tRNA VanZ-1

SMB77998.1 *Peptoniphilus asaccharolyticus DSM 20463* — VanZ-1

WP_051656742.1 *Butyrivibrio sp. AE3004* — VanZ-1

#;TM+TM+VanZ-1

MBO6576774.1 *Rhodothermales bacterium* — VanZ-1

WP_016148732.1 *Butyricicoccus pullicaecorum* — VanZ-1

MBS5145486.1 *Butyricicoccus pullicaecorum* — VanZ-1

#;TM+VanZ-1

TQL01589.1	*Cellulomonas sp. SLBN-39*	VanZ-1
GAW51950.1	*Nocardioides sp. PD653-B2*	VanZ-1
HIT19909.1	*Candidatus Scybalosoma faecavium*	VanZ-1
HJH62223.1	*Bacillota bacterium*	VanZ-1
MBD5151017.1	*Oscillibacter sp.*	VanZ-1
MBQ9375163.1	*Ruminococcus sp.*	VanZ-1
MCD8225317.1	*Clostridiales bacterium*	sugar-O-acetyl/acyltransf · MviN_MATE-domain-flippase · ? · ? · VanZ-1
MBS5088037.1	*Clostridiaceae bacterium*	VanZ-1
PWW66730.1	*Actinokineospora spheciospongiae*	VanZ-1
MBD5113157.1	*Oscillospiraceae bacterium*	VanZ-1
MBE6688948.1	*Oscillospiraceae bacterium*	VanZ-1
HJA36536.1	*Bacillota bacterium*	VanZ-1
CCZ92527.1	*Coprococcus eutactus CAG:665*	VanZ-1
WP_087348886.1	*Flavonifractor sp. An306*	VanZ-1
ERI79699.1	*[Clostridium] symbiosum ATCC 14940*	VanZ-1
WP_155834907.1	*Butyrivibrio sp. FCS006*	undecaprenyl-phosphate-glucose-PTase · LPS-like-sugar-transf · VanZ-1 · LPS-like-sugar-transf
MBE6636590.1	*Oscillospiraceae bacterium*	VanZ-1
MBS5182608.1	*Anaerotruncus sp.*	sugar-O-acetyl/acyltransf · ? · ? · sugar-O-acetyl/acyltransf · MviN_MATE-domain-flippase · ? · ? · ? · VanZ-1
WP_079525039.1	*Halobacillus ihumii*	VanZ-1
MBQ1846994.1	*Clostridia bacterium*	VanZ-1
RGZ33503.1	*Mediterraneibacter gnavus*	VanZ-1 · ? · Peptidase_M29
RHQ56964.1	*Dorea sp. AF24-7LB*	WecC-like-oxidoreductase · ? · VanZ-1
WP_090048685.1	*Lentzea fradiae*	VanZ-1
MBQ7591032.1	*Clostridia bacterium*	VanZ-1
WP_128992692.1	*Clostridium tetani*	VanZ-1
NBK16368.1		VanZ-1
WP_051226703.1	*Butyrivibrio sp. MC2013*	VanZ-1
MBQ6042393.1	*Oscillospiraceae bacterium*	VanZ-1
GFI11880.1	*Lachnospiraceae bacterium*	VanZ-1
HIT32952.1	*Candidatus Enterenecus stercoripullorum*	VanZ-1
MBQ3569413.1	*Methanocorpusculum sp.*	VanZ-1
NBI61774.1	*Clostridiales bacterium*	VanZ-1
MBE6822428.1	*Oscillospiraceae bacterium*	VanZ-1
MBO7630790.1	*Lachnospiraceae bacterium*	VanZ-1
MBQ6386788.1	*Lachnospiraceae bacterium*	VanZ-1
NLJ30570.1	*Clostridiales bacterium*	VanZ-1
MBQ3393084.1	*Lachnospiraceae bacterium*	VanZ-1
MBS6629343.1	*Clostridiales bacterium*	VanZ-1
HJB26272.1	*Bacillota bacterium*	VanZ-1
MBK5268879.1	*Acidimicrobiia bacterium*	VanZ-1
MBE6957667.1	*Oscillospiraceae bacterium*	VanZ-1
RHV16293.1	*Clostridium sp. OM05-9BH*	VanZ-1
MCB6993890.1	*bacterium 210820-DFI.6.37*	VanZ-1
HCR07115.1	*Oscillospiraceae bacterium*	VanZ-1
WP_098766991.1		VanZ-1
WP_205142918.1	*Periweissella beninensis*	VanZ-1
WP_068167803.1	*Rothia sp. ND6WE1A*	VanZ-1
MBS6719803.1	*Peptoniphilus harei*	VanZ-1
HAX73021.1	*Bacillota bacterium*	VanZ-1
WP_060207484.1	*Sporosarcina koreensis*	VanZ-1
WP_012160189.1	*Alkaliphilus oremlandii OhILAs*	VanZ-1
WP_213038680.1		VanZ-1
MBR6607560.1	*Oscillospiraceae bacterium*	VanZ-1
ODU54324.1	*Clostridium sp. SCN 57-10*	VanZ-1
HIR11034.1	*Candidatus Fimenecus excrementavium*	VanZ-1
MBQ8165255.1	*Clostridia bacterium*	VanZ-1
MBD5130015.1	*Oscillospiraceae bacterium*	VanZ-1
MCC8153569.1	*Tannerellaceae bacterium*	VanZ-1

#;TM+TM+VanZ-1

WP_213130280.1	*Bacillus sp. FJAT-49711*	VanZ-1
NMP37982.1	*Clostridiales bacterium*	VanZ-1

#;TM+VanZ-1

WP_066389419.1	*Neobacillus mesonae*	VanZ-1
NLO40638.1	*Ruminiclostridium sp.*	VanZ-1
WP_066677789.1	*Clostridium septicum*	VanZ-1
GFI70840.1	*Clostridiales bacterium*	VanZ-1
MBK8139300.1	*Candidatus Flexicrinis proximus*	VanZ-1
WP_066287682.1	*Fictibacillus arsenicus*	VanZ-1
WP_155669460.1	*Ornithinibacillus caprae*	VanZ-1
MAI91469.1	*Ponticaulis sp.*	VanZ-1
WP_066087143.1	*Neobacillus novalis*	VanZ-1
WP_059174024.1	*Bacillus sp. FJAT-27445*	VanZ-1
WP_078553506.1	*Alkalihalobacterium alkalicellulosilyticum*	VanZ-1
MBS5730414.1	*Clostridium sp.*	VanZ-1
MBC7886737.1	*Ferruginibacter sp.*	VanZ-1
WP_090042413.1	*Clostridium frigidicarnis*	VanZ-1
KFF57105.1	*Bacillus stercoris*	VanZ-1
ASB61041.1	*Bacillus sp. MD-5*	VanZ-1
QQF61997.1	*Bacillus mojavensis*	VanZ-1
WP_010532842.1	*Brachybacterium squillarum M-6-3*	VanZ-1
WP_215004996.1	*Bacillus sp. ISL-7*	VanZ-1
MBE1407462.1		VanZ-1
MBO5095028.1	*Lachnospiraceae bacterium*	VanZ-1
NLL64413.1	*Clostridiaceae bacterium*	VanZ-1
MBQ6819665.1	*Clostridium sp.*	VanZ-1
HAF61528.1	*Anaerolineaceae bacterium*	VanZ-1
MBP3041282.1	*Bacillaceae bacterium Marseille-Q3522*	VanZ-1
EHL03908.1	*Desulfitobacterium hafniense DP7*	VanZ-1
WP_066392087.1	*Cytobacillus eiseniae*	VanZ-1
MAT34990.1	*Ponticaulis sp.*	VanZ-1
MAK60658.1	*Ponticaulis sp.*	VanZ-1
HJE15951.1	*Lapidilactobacillus dextrinicus*	VanZ-1
WP_231505705.1	*Bacillus sp. EB01*	VanZ-1

MCD8088734.1	*Oscillospiraceae bacterium*	VanZ-1
MBK1813000.1	*Clostridium yunnanense*	VanZ-1
MAJ09509.1	*Ponticaulis sp.*	VanZ-1
HAU86078.1	*Lachnospiraceae bacterium*	VanZ-1

#;TM+TM+VanZ-1

HHV35197.1	*Syntrophomonadaceae bacterium*	VanZ-1
GIN86315.1	*Heyndrickxia sporothermodurans*	VanZ-1
MBH0168279.1	*Fictibacillus sp. 18YEL24*	VanZ-1
MCB0323967.1	*Bdellovibrionales bacterium*	VanZ-1
WP_016200863.1	*Niallia alba*	VanZ-1
HDY74348.1	*Methanobacteriota archaeon*	VanZ-1
NLJ27383.1	*Deltaproteobacteria bacterium*	VanZ-1
WP_213093091.1	*Bacillus sp. FJAT-49736*	VanZ-1
WP_066266719.1	*Heyndrickxia acidicola*	VanZ-1
WP_102379811.1	*Raoultibacter timonensis*	VanZ-1

#;TM+VanZ-1

MBQ9489134.1	*Lachnospiraceae bacterium*	VanZ-1
MBR4731410.1	*Lachnospiraceae bacterium*	VanZ-1
MBQ2100329.1	*Lachnospiraceae bacterium*	VanZ-1

#;TM+VanZ-1

MBF1725354.1	*Streptococcus sp.*	VanZ-1
WP_129044751.1	*Companilactobacillus metriopterae*	VanZ-1
WP_076618997.1		VanZ-1
WP_025023996.1	*Companilactobacillus nodensis DSM 19682 = JCM 14932 = NBRC 107160*	VanZ-1
WP_008473416.1	*Lactobacillus gigeriorum DSM 23908 = CRBIP 24.85*	VanZ-1
WP_154547063.1	*Lactobacillus porci*	VanZ-1
WP_106482910.1	*Latilactobacillus fuchuensis*	VanZ-1
KRL44707.1	*Companilactobacillus mindensis DSM 14500*	VanZ-1
WP_154242256.1		VanZ-1
WP_118910413.1		VanZ-1
WP_089746903.1	*Granulicatella balaenopterae*	VanZ-1
WP_067979186.1	*Aerococcus urinaehominis*	VanZ-1

#;TM+VanZ-1+TM

MBQ3193381.1	*Oscillospiraceae bacterium*	VanZ-1
HIS59293.1	*Candidatus Faecousia faecipullorum*	VanZ-1
MBP3684379.1	*Oscillospiraceae bacterium*	VanZ-1
MBQ8834876.1	*Oscillospiraceae bacterium*	VanZ-1
HAL88877.1	*Clostridiales bacterium*	VanZ-1
MBQ6284742.1	*Bacilli bacterium*	VanZ-1
MBP5683646.1	*Bacilli bacterium*	VanZ-1
MBQ3307999.1	*Bacilli bacterium*	VanZ-1
MBS5719208.1	*Clostridium sp.*	VanZ-1
MBQ8473048.1	*Bacilli bacterium*	VanZ-1
CCY27512.1	*Acholeplasma sp. CAG:878*	VanZ-1
WP_009248717.1	*[Clostridium] scindens*	VanZ-1
MBP5427340.1	*Clostridiales bacterium*	VanZ-1
WP_010963971.1	*Clostridium acetobutylicum*	VanZ-1
CDA23370.1	*Mycoplasma sp. CAG:611*	VanZ-1
MBS5115641.1	*Erysipelotrichaceae bacterium*	VanZ-1
RHU82668.1	*Clostridiaceae bacterium OM08-6BH*	VanZ-1
MBK9006611.1	*Candidatus Defluviilinea gracilis*	VanZ-1
CCX91002.1	*Firmicutes bacterium CAG:110*	VanZ-1
HIU10640.1	*Candidatus Avidehalobacter gallistercoris*	VanZ-1
WP_065547893.1	*[Clostridium] clostridioforme CAG:132*	VanZ-1
MBR4185492.1	*Clostridia bacterium*	VanZ-1
MBE6230711.1	*Bacteroidales bacterium*	VanZ-1
HIS51479.1	*Candidatus Onthomonas avicola*	VanZ-1
MBR5365930.1	*Clostridia bacterium*	VanZ-1
OAD88304.1	*Clostridiales bacterium KLE1615*	VanZ-1
MBQ2854572.1	*Oscillospiraceae bacterium*	VanZ-1
MBQ3504790.1	*Oscillospiraceae bacterium*	VanZ-1
WP_195257356.1	*Dorea longicatena*	VanZ-1
RHW04090.1	*Clostridiaceae bacterium OF09-1*	VanZ-1
MBR1972764.1	*Oscillospiraceae bacterium*	VanZ-1
HIT35166.1	*Candidatus Faecousia intestinavium*	VanZ-1
CDE38145.1	*Mycoplasma sp. CAG:472*	VanZ-1
MBQ2546356.1	*Clostridia bacterium*	VanZ-1
HIR31389.1	*Candidatus Faecousia faecavium*	VanZ-1
MBR3298033.1	*Clostridia bacterium*	VanZ-1
HIT37850.1	*Candidatus Onthousia faecipullorum*	VanZ-1
HIS90449.1	*Candidatus Faecisoma merdavium*	VanZ-1
MBP3841084.1	*Bacilli bacterium*	VanZ-1
NCC01322.1	*Clostridia bacterium*	VanZ-1
NMA51276.1	*Mollicutes bacterium*	VanZ-1
WP_204612535.1		VanZ-1
MBS4879350.1	*Bacillota bacterium*	VanZ-1
WP_195941945.1	*Mediterraneibacter faecis*	VanZ-1
MBO5324466.1	*Oscillospiraceae bacterium*	VanZ-1
MBQ7293502.1	*Clostridia bacterium*	VanZ-1
MBU5452231.1	*Pseudoflavonifractor sp. MSJ-30*	VanZ-1
MBF0980266.1	*Clostridiales bacterium*	VanZ-1
EOS60580.1	*Firmicutes bacterium M10-2*	VanZ-1
MBQ6718820.1	*Oscillospiraceae bacterium*	VanZ-1
HAT90109.1	*Roseburia sp.*	VanZ-1
MBQ8243857.1	*Oscillospiraceae bacterium*	sugar-O-acetyl/acyltransf VanZ-1
MBR5616896.1	*Oscillospiraceae bacterium*	VanZ-1
MBP3347826.1	*Clostridia bacterium*	VanZ-1
NRP30824.1	*Aliiroseovarius sp. xm-m-314*	VanZ-1
MBQ1734898.1	*Lachnospiraceae bacterium*	VanZ-1
CDA51141.1	*Clostridium sp. CAG:138*	VanZ-1
QKN24647.1	*Caproicibacterium lactatifermentans*	VanZ-1
AEE47859.1	*Cellulomonas fimi ATCC 484*	VanZ-1
MBS7220191.1	*Clostridiales bacterium*	VanZ-1

#;VanZ-1+TM

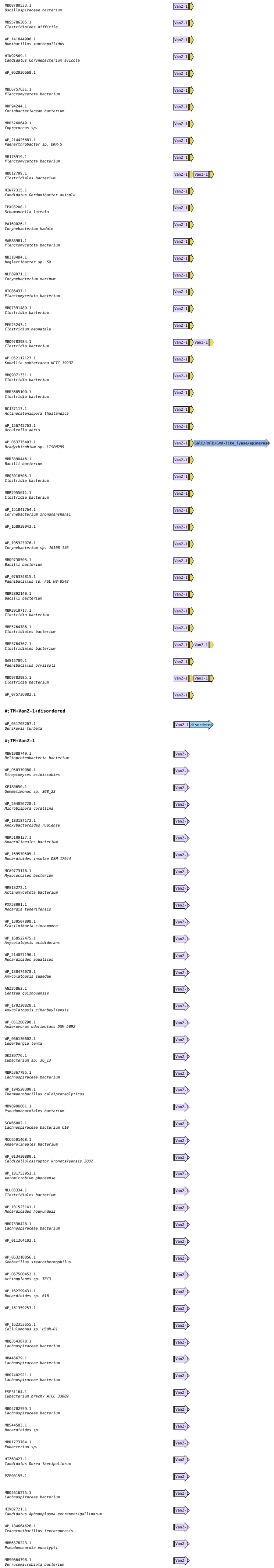
MBQ8780533.1
Oscillospiraceae bacterium
VanZ-1
MBS5786305.1
Clostridioides difficile
VanZ-1
WP_141844906.1
Humibacillus xanthopallidus
VanZ-1
HIW92569.1
Candidatus Corynebacterium avicola
VanZ-1
WP_062036668.1
VanZ-1
MBL6757631.1
Planctomycetota bacterium
VanZ-1
RRF94244.1
Coriobacteriaceae bacterium
VanZ-1
MBO5260649.1
Coprococcus sp.
VanZ-1
WP_214425681.1
Paenarthrobacter sp. DKR-5
VanZ-1
MBJ76919.1
Planctomycetota bacterium
VanZ-1
HBU12799.1
Clostridiales bacterium
VanZ-1
VanZ-1
HIW77315.1
Candidatus Gordonibacter avicola
VanZ-1
TPX03208.1
Schumannella luteola
VanZ-1
PAJ69026.1
Corynebacterium hadale
VanZ-1
MAB88981.1
Planctomycetota bacterium
VanZ-1
NBI18484.1
Neglectibacter sp. 59
VanZ-1
NLF89971.1
Corynebacterium marinum
VanZ-1
HIG86437.1
Planctomycetota bacterium
VanZ-1
MBQ7391489.1
Clostridia bacterium
VanZ-1
PEG25243.1
Clostridium neonatale
VanZ-1
MBQ9783984.1
Clostridia bacterium
VanZ-1
VanZ-1
WP_052112127.1
Knoellia subterranea KCTC 19937
VanZ-1
MBQ9071331.1
Clostridia bacterium
VanZ-1
MBR3685100.1
Clostridia bacterium
VanZ-1
BCJ37117.1
Actinocatenispora thailandica
VanZ-1
WP_156742763.1
Occultella aeris
VanZ-1
WP_063775403.1
Bradyrhizobium sp. LTSPM299
VanZ-1
GalE/RmlB/Gmd-like_lyase/epimerase
MBR3890446.1
Bacilli bacterium
VanZ-1
MBQ3016505.1
Clostridia bacterium
VanZ-1
MBR2955611.1
Clostridia bacterium
VanZ-1
WP_151841764.1
Corynebacterium zhongnanshanii
VanZ-1
WP_168938943.1
VanZ-1
WP_105323976.1
Corynebacterium sp. J010B-136
VanZ-1
MBQ9730505.1
Bacilli bacterium
VanZ-1
WP_076334815.1
Paenibacillus sp. FSL H8-0548
VanZ-1
MBR2892140.1
Bacilli bacterium
VanZ-1
MBR2919717.1
Clostridia bacterium
VanZ-1
MBE5764786.1
Clostridiales bacterium
VanZ-1
MBE5764767.1
Clostridiales bacterium
VanZ-1
VanZ-1
OAS15709.1
Paenibacillus oryzisoli
VanZ-1
MBQ9783985.1
Clostridia bacterium
VanZ-1
VanZ-1
WP_075736082.1
VanZ-1
#;TM+VanZ-1+disordered
WP_051703207.1
Oerskovia turbata
VanZ-1
disordered
#;TM+VanZ-1
MBW1988749.1
Deltaproteobacteria bacterium
VanZ-
WP_050370980.1
Streptomyces acidiscabies
VanZ-
KPJ80650.1
Gemmatimonas sp. SG8_23
VanZ-
WP_204056728.1
Microbispora corallina
VanZ-
WP_183187172.1
Anoxybacteroides rupiense
VanZ-
MBK5109127.1
Anaerolineales bacterium
VanZ-
WP_169570505.1
Nocardioides insulae DSM 17944
VanZ-
MCA9773176.1
Myxococcales bacterium
VanZ-
MRS13272.1
Actinomycetota bacterium
VanZ-
PXX58091.1
Nocardia tenerifensis
VanZ-
WP_130507898.1
Krasilnikovia cinnamomea
VanZ-
WP_168522475.1
Amycolatopsis acididurans
VanZ-
WP_214057196.1
Nocardioides aquaticus
VanZ-
WP_130474078.1
Amycolatopsis suaedae
VanZ-
ANZ35863.1
Lentzea guizhouensis
VanZ-
WP_170220828.1
Amycolatopsis cihanbeyliensis
VanZ-
WP_051280290.1
Anaerovorax odorimutans DSM 5092
VanZ-
WP_066136602.1
Lederbergia lenta
VanZ-
OKZ89776.1
Eubacterium sp. 36_13
VanZ-
MBR5367795.1
Lachnospiraceae bacterium
VanZ-
WP_194520300.1
Thermaerobacillus caldiproteolyticus
VanZ-
MBV8996801.1
Pseudonocardiales bacterium
VanZ-
SCW66002.1
Lachnospiraceae bacterium C10
VanZ-
MCC6501468.1
Anaerolineales bacterium
VanZ-
WP_013430880.1
Caldicellulosiruptor kronotskyensis 2002
VanZ-
WP_181753952.1
Aeromicrobium phoceense
VanZ-
NLL03334.1
Clostridiales bacterium
VanZ-
WP_101523141.1
Nocardioides houyundeii
VanZ-
MBO7336428.1
Lachnospiraceae bacterium
VanZ-
WP_011264102.1
VanZ-
WP_063210856.1
Geobacillus stearothermophilus
VanZ-
WP_067500452.1
Actinoplanes sp. TFC3
VanZ-
WP_162799431.1
Nocardioides sp. 616
VanZ-
WP_161359253.1
VanZ-
WP_162353655.1
Cellulomonas sp. H30R-01
VanZ-
MBQ3543878.1
Lachnospiraceae bacterium
VanZ-
HBA46679.1
Lachnospiraceae bacterium
VanZ-
MBO7402921.1
Lachnospiraceae bacterium
VanZ-
ESE31164.1
Eubacterium brachy ATCC 33089
VanZ-
MBO4782559.1
Lachnospiraceae bacterium
VanZ-
MBS44583.1
Nocardioides sp.
VanZ-
MBR1773784.1
Eubacterium sp.
VanZ-
HIZ60427.1
Candidatus Dorea faecipullorum
VanZ-
PZF00155.1
VanZ-
MBO4616275.1
Lachnospiraceae bacterium
VanZ-
HIV02721.1
Candidatus Aphodoplasma excrementigallinarum
VanZ-
WP_184664626.1
Texcoconibacillus texcoconensis
VanZ-
MBB6378223.1
Pseudonocardia eucalypti
VanZ-
MBS0664798.1
Verrucomicrobiota bacterium
VanZ-

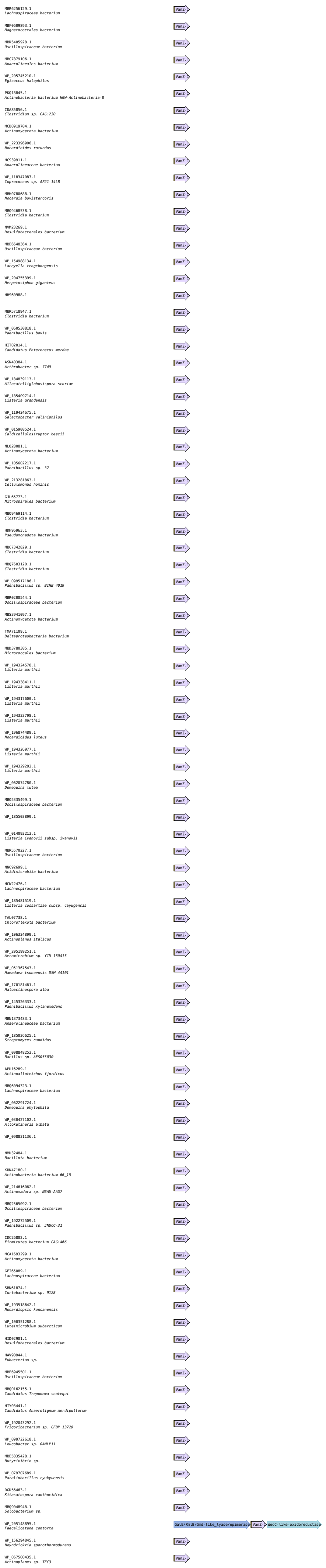

MBR6256129.1
Lachnospiraceae bacterium
VanZ-1
MBF0609893.1
Magnetococcales bacterium
VanZ-1
MBR5405928.1
Oscillospiraceae bacterium
VanZ-1
MBC7879106.1
Anaerolineales bacterium
VanZ-1
WP_205745210.1
Egicoccus halophilus
VanZ-1
PKQ18845.1
Actinobacteria bacterium HGW-Actinobacteria-8
VanZ-1
CDA85856.1
Clostridium sp. CAG:230
VanZ-1
MCB0919704.1
Actinomycetota bacterium
VanZ-1
WP_223396906.1
Nocardioides rotundus
VanZ-1
HCS39911.1
Anaerolineaceae bacterium
VanZ-1
WP_118347087.1
Coprococcus sp. AF21-14LB
VanZ-1
MBH0780688.1
Nocardia bovistercoris
VanZ-1
MBQ9468538.1
Clostridia bacterium
VanZ-1
NVM23269.1
Desulfobacterales bacterium
VanZ-1
MBE6648364.1
Oscillospiraceae bacterium
VanZ-1
WP_154988134.1
Laceyella tengchongensis
VanZ-1
WP_204755399.1
Herpetosiphon giganteus
VanZ-1
HHS60988.1
VanZ-1
MBR5718947.1
Clostridia bacterium
VanZ-1
WP_060530818.1
Paenibacillus bovis
VanZ-1
HIT02014.1
Candidatus Enterenecus merdae
VanZ-1
ASN40384.1
Arthrobacter sp. 7749
VanZ-1
WP_184839113.1
Allocatelliglobosispora scoriae
VanZ-1
WP_185409714.1
Listeria grandensis
VanZ-1
WP_119424675.1
Galactobacter valiniphilus
VanZ-1
WP_015908524.1
Caldicellulosiruptor bescii
VanZ-1
NLO28081.1
Actinomycetota bacterium
VanZ-1
WP_105602217.1
Paenibacillus sp. 37
VanZ-1
WP_213281863.1
Cellulomonas hominis
VanZ-1
GJL65773.1
Nitrospirales bacterium
VanZ-1
MBQ9469114.1
Clostridia bacterium
VanZ-1
HDH96963.1
Pseudomonadota bacterium
VanZ-1
MBC7342829.1
Clostridia bacterium
VanZ-1
MBQ7603120.1
Clostridia bacterium
VanZ-1
WP_099517186.1
Paenibacillus sp. BIHB 4019
VanZ-1
MBR0200544.1
Oscillospiraceae bacterium
VanZ-1
MBS3941097.1
Actinomycetota bacterium
VanZ-1
TMA71109.1
Deltaproteobacteria bacterium
VanZ-1
MBD3780385.1
Micrococcales bacterium
VanZ-1
WP_194324578.1
Listeria marthii
VanZ-1
WP_194338411.1
Listeria marthii
VanZ-1
WP_194317600.1
Listeria marthii
VanZ-1
WP_194333798.1
Listeria marthii
VanZ-1
WP_196874489.1
Nocardioides luteus
VanZ-1
WP_194326977.1
Listeria marthii
VanZ-1
WP_194329202.1
Listeria marthii
VanZ-1
WP_062074780.1
Demequina lutea
VanZ-1
MBQ5335499.1
Oscillospiraceae bacterium
VanZ-1
WP_185503899.1
VanZ-1
WP_014092213.1
Listeria ivanovii subsp. ivanovii
VanZ-1
MBR5570227.1
Oscillospiraceae bacterium
VanZ-1
NNC92699.1
Acidimicrobiia bacterium
VanZ-1
HCW22476.1
Lachnospiraceae bacterium
VanZ-1
WP_185481519.1
Listeria cossartiae subsp. cayugensis
VanZ-1
TAL07738.1
Chloroflexota bacterium
VanZ-1
WP_106324899.1
Actinoplanes italicus
VanZ-1
WP_205199251.1
Aeromicrobium sp. YIM 150415
VanZ-1
WP_051367543.1
Hamadaea tsunoensis DSM 44101
VanZ-1
WP_170181461.1
Haloactinospora alba
VanZ-1
WP_145326333.1
Paenibacillus xylanexedens
VanZ-1
MBN1373483.1
Anaerolineaceae bacterium
VanZ-1
WP_185036625.1
Streptomyces candidus
VanZ-1
WP_098848253.1
Bacillus sp. AFS055030
VanZ-1
APU16289.1
Actinoalloteichus fjordicus
VanZ-1
MBQ6094323.1
Lachnospiraceae bacterium
VanZ-1
WP_062291724.1
Demequina phytophila
VanZ-1
WP_030427102.1
Allokutzneria albata
VanZ-1
WP_098831136.1
VanZ-1
NMD32484.1
Bacillota bacterium
VanZ-1
KUK47180.1
Actinobacteria bacterium 66_15
VanZ-1
WP_214616062.1
Actinomadura sp. NEAU-AAG7
VanZ-1
MBQ2565092.1
Oscillospiraceae bacterium
VanZ-1
WP_192272509.1
Paenibacillus sp. JNUCC-31
VanZ-1
CDC26802.1
Firmicutes bacterium CAG:466
VanZ-1
MCA1693299.1
Actinomycetota bacterium
VanZ-1
GFI65089.1
Lachnospiraceae bacterium
VanZ-1
SBN61874.1
Curtobacterium sp. 9128
VanZ-1
WP_193518642.1
Nocardiopsis kunsanensis
VanZ-1
WP_100351288.1
Luteimicrobium subarcticum
VanZ-1
HID02901.1
Desulfobacterales bacterium
VanZ-1
HAV90944.1
Eubacterium sp.
VanZ-1
MBE6945501.1
Oscillospiraceae bacterium
VanZ-1
MBQ0162155.1
Candidatus Treponema scatequi
VanZ-1
HIY03441.1
Candidatus Anaerotignum merdipullorum
VanZ-1
WP_192043292.1
Frigoribacterium sp. CFBP 13729
VanZ-1
WP_099722618.1
Leucobacter sp. OAMLP11
VanZ-1
MBE5835428.1
Butyrivibrio sp.
VanZ-1
WP_079707689.1
Paraliobacillus ryukyuensis
VanZ-1
RGD56463.1
Kitasatospora xanthocidica
VanZ-1
MBQ9048948.1
Solobacterium sp.
VanZ-1
WP_205148895.1
Faecalicatena contorta
GalE/RmlB/Gmd-like_lyase/epimerase
VanZ-1
WecC-like-oxidoreductase
WP_156294845.1
Heyndrickxia sporothermodurans
VanZ-1
WP_067500435.1
Actinoplanes sp. TFC3
VanZ-1

WP_051563209.1	*Butyrivibrio sp. LB2008*	VanZ-1
QQD77862.1	*Curtobacterium sp. YC1*	VanZ-1
WP_014445937.1	*Actinoplanes missouriensis*	VanZ-1, LPS-like-sugar-transf
WP_074461632.1	*Butyrivibrio hungatei*	VanZ-1
WP_184691367.1	*Saccharothrix tamanrassetensis*	VanZ-1
MCB6992684.1	*bacterium 210820-DFI.6.37*	VanZ-1

#;TM+TM+VanZ-1

WP_078552868.1	*Alkalihalobacterium alkalicellulosilyticum*	VanZ-1
MBO6161825.1	*Eubacterium sp.*	VanZ-1
WP_129080508.1	*Anaerobacillus alkaliphilus*	VanZ-1
WP_181471777.1	*Halobacillus locisalis*	VanZ-1
WP_088013275.1	*Gottfriedia acidiceleris*	VanZ-1
WP_016264384.1	*Latilactobacillus sakei*	VanZ-1
WP_003323640.1	*Alkalihalobacillus alcalophilus*	VanZ-1
MBD9217857.1	*Clostridiales bacterium*	VanZ-1
WP_101648534.1		VanZ-1
WP_007083109.1	*Neobacillus bataviensis LMG 21833*	VanZ-1
QVK17303.1	*Mycoplasmatota bacterium*	VanZ-1
WP_205010590.1	*Streptococcus loxodontisalivarius*	VanZ-1
WP_090093184.1	*Lactobacillus bombicola*	VanZ-1
WP_016184706.1	*Enterococcus sulfureus ATCC 49903*	VanZ-1
MBS6600760.1	*Clostridium sp.*	VanZ-1
WP_233144154.1		VanZ-1
WP_004266047.1	*Latilactobacillus curvatus*	VanZ-1
WP_016183957.1	*Enterococcus columbae DSM 7374 = ATCC 51263*	VanZ-1
WP_066306867.1	*Bacillus sp. FJAT-29814*	VanZ-1
WP_206152586.1	*Streptococcus equi subsp. zooepidemicus*	VanZ-1
MBM7669955.1		VanZ-1
WP_223589180.1	*Neobacillus bataviensis*	VanZ-1
WP_204413143.1	*Sutcliffiella tianshenii*	VanZ-1
WP_026571919.1	*Sediminibacillus sp. JSM 1682029*	VanZ-1
MBO4998407.1	*Lachnospira sp.*	VanZ-1
WP_161979965.1	*Streptococcus sp. S784/96/1*	VanZ-1
WP_066914502.1	*Streptococcus sp. DD12*	VanZ-1
WP_184523373.1	*Bacillus benzoevorans*	VanZ-1
WP_143892630.1	*Radiobacillus deserti*	VanZ-1
WP_066870736.1	*Clostridium mediterraneense*	VanZ-1, VanZ-1
WP_114981056.1	*Clostridium botulinum*	VanZ-1
WP_225196750.1	*Halobacillus litoralis*	VanZ-1
WP_067088510.1	*Streptococcus marmotae*	VanZ-1
WP_051271287.1	*Shimazuella kribbensis DSM 45090*	VanZ-1
WP_079477465.1	*Halobacillus salinus*	VanZ-1
WP_009659410.1	*Streptococcus sp. AS14*	VanZ-1
MBP3872025.1	*Faecalicoccus sp.*	VanZ-1
WP_131237722.1	*Cytobacillus praedii*	VanZ-1
WP_078378546.1	*Sutcliffiella halmapala*	VanZ-1
WP_119545669.1	*Bacillus salacetis*	VanZ-1
MBS6206565.1	*Bacillota bacterium*	VanZ-1
KMJ59314.1	*Bacillus sp. LL01*	VanZ-1
MBD0858248.1	*Erysipelotrichaceae bacterium 7770_A6*	VanZ-1
WP_075690626.1	*Bacillus sp. MRMR6*	VanZ-1
HJB33571.1	*Candidatus Blautia merdipullorum*	VanZ-1
NLU24494.1	*Clostridiales bacterium*	VanZ-1
CDB28953.1	*Firmicutes bacterium CAG:582*	VanZ-1
WP_115873553.1		VanZ-1

#;TM+VanZ-1

SCK00054.1	*uncultured Eubacterium sp.*	VanZ-1
WP_004825717.1	*Peptoniphilus lacrimalis 315-B*	VanZ-1
KYD24248.1	*Geobacillus stearothermophilus*	VanZ-1
MBR6635674.1	*Clostridia bacterium*	VanZ-1
WP_225046690.1	*Lacticaseibacillus kribbianus*	VanZ-1
MCB4340380.1	*Bacillus subtilis*	VanZ-1
WP_196202742.1	*Plantactinospora alkalitolerans*	VanZ-1
CDZ24488.1	*[Clostridium] cellulosi*	VanZ-1
MBQ8997814.1	*Clostridium sp.*	VanZ-1
MBD5090094.1	*Clostridiales bacterium*	VanZ-1
HAE62899.1	*Eubacteriaceae bacterium*	VanZ-1
WP_051638743.1	*Butyrivibrio sp. NC2002*	VanZ-1
MBO5229162.1	*Lachnospiraceae bacterium*	VanZ-1
WP_016439008.1		sugar-O-acetyl/acyltransf, ?, VanZ-1
HIU42992.1	*Candidatus Ventrousia excrementavium*	VanZ-1
MBD5143153.1	*Ruminococcus sp.*	VanZ-1
WP_214800307.1	*Bacillus sp. ISL-51*	VanZ-1
SLK31194.1	*Mycobacteroides abscessus subsp. abscessus*	VanZ-1
MBS5335859.1	*Bacillota bacterium*	VanZ-1
MCB1482752.1	*Rhodobiaceae bacterium*	VanZ-1
WP_224130944.1	*Nocardioides sp. TRM66260-LWL*	VanZ-1
MBS5806188.1	*Bacillota bacterium*	VanZ-1
WP_062076277.1	*Demequina lutea*	VanZ-1
MBM7671746.1		VanZ-1
MBP7321371.1	*Deltaproteobacteria bacterium*	VanZ-1
WP_099208648.1	*Urinicoccus timonensis*	VanZ-1
MBE6919122.1	*Oscillospiraceae bacterium*	VanZ-1
MBN1959435.1	*Deltaproteobacteria bacterium*	VanZ-1
WP_183278064.1	*Clostridium fungisolvens*	VanZ-1
WP_075717569.1	*Roseburia sp. 499*	VanZ-1
MBQ2117302.1	*Lachnospiraceae bacterium*	GalE/RmlB/Gmd-like_lyase/epimerase, WecC-like-oxidoreductase, VanZ-1
HBI59816.1	*Lachnospiraceae bacterium*	VanZ-1
WP_026670895.1	*Butyrivibrio sp. AE3006*	VanZ-1
WP_051933777.1	*Carnobacterium gallinarum DSM 4847*	VanZ-1
HIS24818.1	*Candidatus Faeciplasma gallinarum*	VanZ-1
MBQ3598257.1	*Clostridia bacterium*	VanZ-1
HIR85312.1	*Candidatus Galloscillospira excrementavium*	VanZ-1

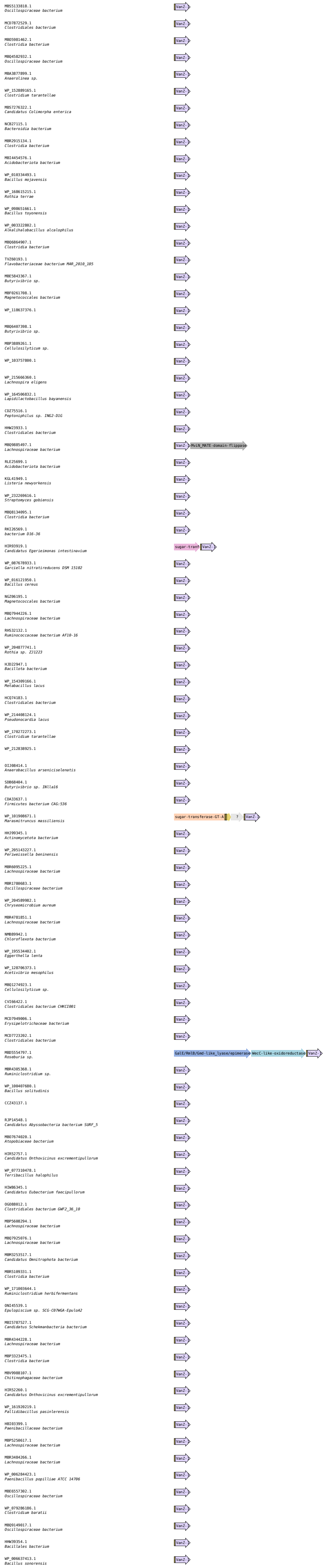
MBS5133818.1
Oscillospiraceae bacterium
VanZ-1
MCD7872529.1
Clostridiales bacterium
VanZ-1
MBO5981462.1
Clostridia bacterium
VanZ-1
MBQ4582932.1
Oscillospiraceae bacterium
VanZ-1
MBA3877899.1
Anaerolinea sp.
VanZ-1
WP_152889165.1
Clostridium tarantellae
VanZ-1
MBS7276322.1
Candidatus Colimorpha enterica
VanZ-1
NCB27115.1
Bacteroidia bacterium
VanZ-1
MBR2915134.1
Clostridia bacterium
VanZ-1
MBI4454576.1
Acidobacteriota bacterium
VanZ-1
WP_010334493.1
Bacillus mojavensis
VanZ-1
WP_168615215.1
Rothia terrae
VanZ-1
WP_098651661.1
Bacillus toyonensis
VanZ-1
WP_003322802.1
Alkalihalobacillus alcalophilus
VanZ-1
MBQ6864907.1
Clostridia bacterium
VanZ-1
TVZ60193.1
Flavobacteriaceae bacterium MAR_2010_105
VanZ-1
MBE5843367.1
Butyrivibrio sp.
VanZ-1
MBF0261708.1
Magnetococcales bacterium
VanZ-1
WP_118637376.1
VanZ-1
MBQ6407398.1
Butyrivibrio sp.
VanZ-1
MBP3889261.1
Cellulosilyticum sp.
VanZ-1
WP_103757800.1
VanZ-1
WP_215666360.1
Lachnospira eligens
VanZ-1
WP_164506832.1
Lapidilactobacillus bayanensis
VanZ-1
CDZ75516.1
Peptoniphilus sp. ING2-D1G
VanZ-1
HHW23933.1
Clostridiales bacterium
VanZ-1
MBQ9885497.1
Lachnospiraceae bacterium
VanZ-1
MviN_MATE-domain-flippase
RLE25699.1
Acidobacteriota bacterium
VanZ-1
KGL41949.1
Listeria newyorkensis
VanZ-1
WP_232269616.1
Streptomyces gobiensis
VanZ-1
MBQ8134095.1
Clostridia bacterium
VanZ-1
RKI26569.1
bacterium D16-36
VanZ-1
HIR93919.1
Candidatus Egerieimonas intestinavium
sugar-tranf
VanZ-1
WP_087678933.1
Garciella nitratireducens DSM 15102
VanZ-1
WP_016121950.1
Bacillus cereus
VanZ-1
NGZ06195.1
Magnetococcales bacterium
VanZ-1
MBQ7944226.1
Lachnospiraceae bacterium
VanZ-1
RHS32132.1
Ruminococcaceae bacterium AF10-16
VanZ-1
WP_204877741.1
Rothia sp. ZJ1223
VanZ-1
HJD22947.1
Bacillota bacterium
VanZ-1
WP_154309166.1
Metabacillus lacus
VanZ-1
HCQ74183.1
Clostridiales bacterium
VanZ-1
WP_214408124.1
Pseudonocardia lacus
VanZ-1
WP_170272273.1
Clostridium tarantellae
VanZ-1
WP_212838925.1
VanZ-1
OIJ08414.1
Anaerobacillus arseniciselenatis
VanZ-1
SDB68404.1
Butyrivibrio sp. INlla16
VanZ-1
CDA33637.1
Firmicutes bacterium CAG:536
VanZ-1
WP_101908671.1
Marasmitruncus massiliensis
sugar-transferase-GT-A
?
VanZ-1
HHJ99345.1
Actinomycetota bacterium
VanZ-1
WP_205143227.1
Periweissella beninensis
VanZ-1
MBR6095225.1
Lachnospiraceae bacterium
VanZ-1
MBR1780683.1
Oscillospiraceae bacterium
VanZ-1
WP_204589982.1
Chryseomicrobium aureum
VanZ-1
MBR4781851.1
Lachnospiraceae bacterium
VanZ-1
NMB89942.1
Chloroflexota bacterium
VanZ-1
WP_195534402.1
Eggerthella lenta
VanZ-1
WP_128706373.1
Acetivibrio mesophilus
VanZ-1
MBQ1274923.1
Cellulosilyticum sp.
VanZ-1
CVI66422.1
Clostridiales bacterium CHKCI001
VanZ-1
MCD7949006.1
Erysipelotrichaceae bacterium
VanZ-1
MCD7723202.1
Clostridiales bacterium
VanZ-1
MBD5554797.1
Roseburia sp.
GalE/RmlB/Gmd-like_lyase/epimerase
WecC-like-oxidoreductase
VanZ-1
MBR4305368.1
Ruminiclostridium sp.
VanZ-1
WP_100407680.1
Bacillus solitudinis
VanZ-1
CCZ43137.1
VanZ-1
RJP14548.1
Candidatus Abyssobacteria bacterium SURF_5
VanZ-1
MBO7674020.1
Atopobiaceae bacterium
VanZ-1
HIR52757.1
Candidatus Onthovicinus excrementipullorum
VanZ-1
WP_077310478.1
Terribacillus halophilus
VanZ-1
HIW86345.1
Candidatus Eubacterium faecipullorum
VanZ-1
OGO88012.1
Clostridiales bacterium GWF2_36_10
VanZ-1
MBP5608294.1
Lachnospiraceae bacterium
VanZ-1
MBQ7925076.1
Lachnospiraceae bacterium
VanZ-1
MBM3253517.1
Candidatus Omnitrophota bacterium
VanZ-1
MBR5109331.1
Clostridia bacterium
VanZ-1
WP_171003644.1
Ruminiclostridium herbifermentans
VanZ-1
ONI45539.1
Epulopiscium sp. SCG-C07WGA-EpuloA2
VanZ-1
MBI5787527.1
Candidatus Schekmanbacteria bacterium
VanZ-1
MBR4344228.1
Lachnospiraceae bacterium
VanZ-1
MBP3323475.1
Clostridia bacterium
VanZ-1
MBV9988107.1
Chitinophagaceae bacterium
VanZ-1
HIR52260.1
Candidatus Onthovicinus excrementipullorum
VanZ-1
WP_161920219.1
Pallidibacillus pasinlerensis
VanZ-1
HBI03399.1
Paenibacillaceae bacterium
VanZ-1
MBP5250617.1
Lachnospiraceae bacterium
VanZ-1
MBR3484266.1
Lachnospiraceae bacterium
VanZ-1
WP_006284423.1
Paenibacillus popilliae ATCC 14706
VanZ-1
MBE6557302.1
Oscillospiraceae bacterium
VanZ-1
WP_079286186.1
Clostridium baratii
VanZ-1
MBQ9149017.1
Oscillospiraceae bacterium
VanZ-1
HHW39354.1
Bacillales bacterium
VanZ-1
WP_006637413.1
Bacillus sonorensis
VanZ-1

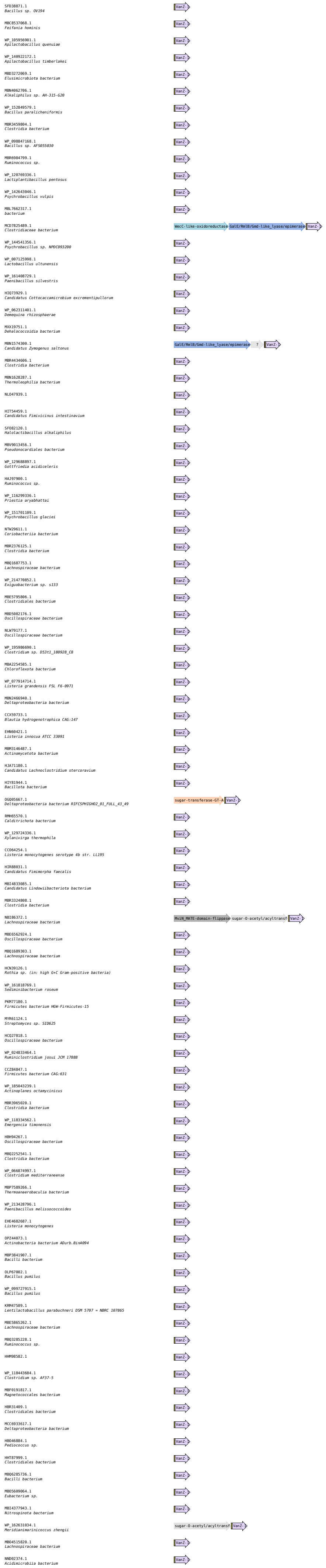
SFD38871.1
Bacillus sp. OV194
VanZ-1
MBC8537068.1
Feifania hominis
VanZ-1
WP_105956901.1
Apilactobacillus quenuiae
VanZ-1
WP_140922172.1
Apilactobacillus timberlakei
VanZ-1
MBD3272069.1
Elusimicrobiota bacterium
VanZ-1
MBN4062706.1
Alkaliphilus sp. AH-315-G20
VanZ-1
WP_152849579.1
Bacillus paralicheniformis
VanZ-1
MBR3459804.1
Clostridia bacterium
VanZ-1
WP_098847168.1
Bacillus sp. AFS055030
VanZ-1
MBR6984799.1
Ruminococcus sp.
VanZ-1
WP_120769336.1
Lactiplantibacillus pentosus
VanZ-1
WP_142643046.1
Psychrobacillus vulpis
VanZ-1
MBL7662317.1
bacterium
VanZ-1
MCD7825489.1
Clostridiaceae bacterium
WecC-like-oxidoreductase
GalE/RmlB/Gmd-like_lyase/epimerase
VanZ-1
WP_144541356.1
Psychrobacillus sp. NPDC093200
VanZ-1
WP_007125998.1
Lactobacillus ultunensis
VanZ-1
WP_161408729.1
Paenibacillus silvestris
VanZ-1
HIQ73929.1
Candidatus Cottocaccamicrobium excrementipullorum
VanZ-1
WP_062311401.1
Demequina rhizosphaerae
VanZ-1
MXX19751.1
Dehalococcoidia bacterium
VanZ-1
MBN1574300.1
Candidatus Zymogenus saltonus
GalE/RmlB/Gmd-like_lyase/epimerase
?
VanZ-1
MBR4434606.1
Clostridia bacterium
VanZ-1
MBN1628287.1
Thermoleophilia bacterium
VanZ-1
NLO47939.1
VanZ-1
HIT54459.1
Candidatus Fimivicinus intestinavium
VanZ-1
SFO82120.1
Halolactibacillus alkaliphilus
VanZ-1
MBV9013456.1
Pseudonocardiales bacterium
VanZ-1
WP_129688897.1
Gottfriedia acidiceleris
VanZ-1
HAJ97900.1
Ruminococcus sp.
VanZ-1
WP_116299336.1
Priestia aryabhattai
VanZ-1
WP_151701109.1
Psychrobacillus glaciei
VanZ-1
NTW29611.1
Coriobacteriia bacterium
VanZ-1
MBR2376125.1
Clostridia bacterium
VanZ-1
MBQ1687753.1
Lachnospiraceae bacterium
VanZ-1
WP_214770852.1
Exiguobacterium sp. s133
VanZ-1
MBE5795806.1
Clostridiales bacterium
VanZ-1
MBD5082176.1
Oscillospiraceae bacterium
VanZ-1
NLW79177.1
Oscillospiraceae bacterium
VanZ-1
WP_195986690.1
Clostridium sp. D53t1_180928_C8
VanZ-1
MBA2254585.1
Chloroflexota bacterium
VanZ-1
WP_077914714.1
Listeria grandensis FSL F6-0971
VanZ-1
MBN2466940.1
Deltaproteobacteria bacterium
VanZ-1
CCX59733.1
Blautia hydrogenotrophica CAG:147
VanZ-1
EHN60421.1
Listeria innocua ATCC 33091
VanZ-1
MBM3146487.1
Actinomycetota bacterium
VanZ-1
HJA71180.1
Candidatus Lachnoclostridium stercoravium
VanZ-1
HIY81944.1
Bacillota bacterium
VanZ-1
OGQ05667.1
Deltaproteobacteria bacterium RIFCSPHIGHO2_01_FULL_43_49
sugar-transferase-GT-A
VanZ-1
RMH65570.1
Calditrichota bacterium
VanZ-1
WP_129724336.1
Xylanivirga thermophila
VanZ-1
CCO64254.1
Listeria monocytogenes serotype 4b str. LL195
VanZ-1
HIR88031.1
Candidatus Fimimorpha faecalis
VanZ-1
MBI4833085.1
Candidatus Lindowiibacteriota bacterium
VanZ-1
MBR3324808.1
Clostridia bacterium
VanZ-1
NBI86372.1
Lachnospiraceae bacterium
MviN_MATE-domain-flippase
sugar-O-acetyl/acyltransf
VanZ-1
MBE6562924.1
Oscillospiraceae bacterium
VanZ-1
MBQ1689303.1
Lachnospiraceae bacterium
VanZ-1
HCN39126.1
Rothia sp. (in: high G+C Gram-positive bacteria)
VanZ-1
WP_161818769.1
Sediminibacterium roseum
VanZ-1
PKM77180.1
Firmicutes bacterium HGW-Firmicutes-15
VanZ-1
MYR61124.1
Streptomyces sp. SID625
VanZ-1
HCQ27818.1
Oscillospiraceae bacterium
VanZ-1
WP_024833464.1
Ruminiclostridium josui JCM 17888
VanZ-1
CCZ84847.1
Firmicutes bacterium CAG:631
VanZ-1
WP_185043239.1
Actinoplanes octamycinicus
VanZ-1
MBR3965020.1
Clostridia bacterium
VanZ-1
WP_118334562.1
Emergencia timonensis
VanZ-1
HBH94267.1
Oscillospiraceae bacterium
VanZ-1
MBQ2252541.1
Clostridia bacterium
VanZ-1
WP_066874997.1
Clostridium mediterraneense
VanZ-1
MBP7589266.1
Thermoanaerobaculia bacterium
VanZ-1
WP_213428796.1
Paenibacillus melissococcoides
VanZ-1
EHE4682687.1
Listeria monocytogenes
VanZ-1
OPZ44073.1
Actinobacteria bacterium ADurb.BinA094
VanZ-1
MBP3841907.1
Bacilli bacterium
VanZ-1
OLP67002.1
Bacillus pumilus
VanZ-1
WP_099727915.1
Bacillus pumilus
VanZ-1
KRM47589.1
Lentilactobacillus parabuchneri DSM 5707 = NBRC 107865
VanZ-1
MBE5865262.1
Lachnospiraceae bacterium
VanZ-1
MBQ3285228.1
Ruminococcus sp.
VanZ-1
HHM98582.1
VanZ-1
WP_118443684.1
Clostridium sp. AF37-5
VanZ-1
MBF0191817.1
Magnetococcales bacterium
VanZ-1
HBR31409.1
Clostridiales bacterium
VanZ-1
MCC6933617.1
Deltaproteobacteria bacterium
VanZ-1
HBO46884.1
Pediococcus sp.
VanZ-1
HHT87999.1
Clostridiales bacterium
VanZ-1
MBQ6285736.1
Bacilli bacterium
VanZ-1
MBO5609064.1
Eubacterium sp.
VanZ-1
MBI4377943.1
Nitrospinota bacterium
VanZ-1
WP_162631034.1
Meridianimarinicoccus zhengii
sugar-O-acetyl/acyltransf
VanZ-1
MBO4515820.1
Lachnospiraceae bacterium
VanZ-1
NND02374.1
Acidimicrobiia bacterium
VanZ-1

Accession	Organism	Genes
WP_091022253.1	*Bacillus sp. UNCCL81*	VanZ-1
WP_091024835.1		VanZ-1
MBR3033692.1	*Clostridiales bacterium*	VanZ-1
MCA1634647.1	*Acidobacteriota bacterium*	VanZ-1
WP_068693240.1	*Paenibacillus yonginensis*	VanZ-1
NIQ83614.1	*Acidobacteriota bacterium*	VanZ-1
WP_128219303.1	*Nocardioides yefusunii*	VanZ-1
WP_077735324.1	*Bacillus sonorensis*	VanZ-1
MBF0370631.1	*Magnetococcales bacterium*	VanZ-1
MBT8264945.1	*Bacteroidia bacterium*	VanZ-1
HAM43106.1	*Candidatus Avelusimicrobium stercoris*	VanZ-1
MBO8434652.1	*Candidatus Fimicola merdigallinarum*	VanZ-1
WP_050697042.1	*Anaeromassilibacillus senegalensis*	VanZ-1
SDP81223.1	*Actinopolyspora xinjiangensis*	VanZ-1
CDD26767.1	*Clostridium sp. CAG:452*	VanZ-1
PLW84925.1	*Halioglobus japonicus*	VanZ-1
WP_233315048.1	*Bacillus sp. Au-Bac7*	VanZ-1
WP_169189768.1	*Niallia alba*	VanZ-1
WP_011640170.1	*Syntrophomonas wolfei subsp. wolfei str. Goettingen G311*	VanZ-1
MBD9225492.1	*Clostridiales bacterium*	VanZ-1
WP_128213250.1	*Clostridium manihotivorum*	VanZ-1
GIG11047.1	*Catellatospora coxensis*	VanZ-1
WP_078552536.1	*Alkalihalobacterium alkalicellulosilyticum*	VanZ-1
WP_077110183.1	*Siminovitchia thermophila*	VanZ-1
WP_212962920.1	*Siminovitchia fordii*	VanZ-1
WP_118734611.1	*Coprococcus hominis (ex Arizal et al. 2022)*	VanZ-1
MBQ8201013.1	*Clostridia bacterium*	VanZ-1
CDE53924.1	*Roseburia sp. CAG:303*	VanZ-1
MBP3588135.1	*Clostridia bacterium*	VanZ-1
WP_090841540.1	*Alkalicoccus daliensis*	VanZ-1
MBR2742329.1	*Clostridia bacterium*	VanZ-1
NJS37736.1	*Paracoccaceae bacterium*	sugar-O-acetyl/acyltransf; VanZ-1
MBR2699186.1	*Clostridia bacterium*	VanZ-1
MBQ6873913.1	*Clostridia bacterium*	VanZ-1
RKI63722.1	*bacterium 1xD42-67*	VanZ-1
WP_090060941.1	*Celeribacter neptunius*	sugar-O-acetyl/acyltransf; VanZ-1
MBQ6156617.1	*Bacteroidales bacterium*	VanZ-1; GalE/RmlB/Gmd-like_lyase/epimerase; GDP-L-fucose-synthase
MBP5172001.1	*Bacteroidales bacterium*	VanZ-2; VanZ-1
MBA3468760.1	*Herpetosiphonaceae bacterium*	VanZ-1
MBR2892085.1	*Bacilli bacterium*	VanZ-1
WP_024056670.1	*Intestinibacter bartlettii*	VanZ-1
HIR67158.1	*Candidatus Coproplasma avicola*	VanZ-1

#;TM+VanZ-1

Accession	Organism	Genes
WP_204417645.1	*Actibacterium sp. 188UL27-1*	VanZ-1
MBR5667514.1	*Lachnospiraceae bacterium*	VanZ-1
TAN63393.1	*bacterium*	VanZ-1
HIQ93243.1	*Candidatus Copromonas avistercoris (nom. illeg.)*	VanZ-1
NLC67283.1	*Clostridiaceae bacterium*	VanZ-1; UDP-N-acetylglucosamine-dehyd

#;TM+TM+VanZ-1

Accession	Organism	Genes
WP_195632067.1	*Clostridium paraputrificum*	VanZ-1
MBQ3611268.1	*Bacillota bacterium*	VanZ-1
OJG58325.1	*Enterococcus mundtii*	VanZ-1
MBF1084223.1	*Solobacterium sp.*	VanZ-1
HCQ27279.1	*Oscillospiraceae bacterium*	VanZ-1
CCX72402.1	*Firmicutes bacterium CAG:83*	VanZ-1
WP_162473822.1	*endosymbiont 'TC1' of Trimyema compressum*	VanZ-1

#;TM+VanZ-1+TM

Accession	Organism	Genes
MBQ3165801.1	*Clostridia bacterium*	VanZ-1
MBQ2183727.1	*Lachnospiraceae bacterium*	VanZ-1
MBS1315496.1	*Anaerotignum sp.*	VanZ-1
WP_076342320.1	*Dubosiella newyorkensis*	VanZ-1

#;VanZ-1

Accession	Organism	Genes
MBE3143068.1	*Planctomycetota bacterium*	VanZ-1

#;TM+VanZ-1

Accession	Organism	Genes
MCA0273915.1	*Pseudomonadota bacterium*	VanZ-1
WP_087190483.1	*Gordonibacter urolithinfaciens*	VanZ-1
WP_232747482.1	*Ornithinimicrobium sediminis*	VanZ-1
WP_088233650.1	*Haematobacter missouriensis*	VanZ-1
WP_067691523.1	*Nocardia jejuensis NBRC 103114*	VanZ-1
MBR5536782.1	*Clostridia bacterium*	VanZ-1
HAL74486.1	*Clostridiales bacterium*	VanZ-1
WP_118731598.1	*Clostridium sp. AM33-3*	VanZ-1
NLY32009.1	*Bacillota bacterium*	VanZ-1
MBS6215704.1	*Clostridiales bacterium*	VanZ-1
WP_067071511.1	*Carbonactinospora thermoautotrophica*	VanZ-1
MBQ1435813.1	*Clostridia bacterium*	VanZ-1
NLG25114.1	*Clostridiales bacterium*	VanZ-1
MBL6469213.1	*Mogibacterium sp.*	VanZ-1
SFK48833.1	*Lachnospiraceae bacterium KH1T2*	VanZ-1
WP_170151898.1	*Subtercola boreus*	VanZ-1
RLE18068.1	*Acidobacteriota bacterium*	VanZ-1
HHV19779.1	*Thermoanaerobacterales bacterium*	VanZ-1
MBQ8316292.1	*Lachnospiraceae bacterium*	VanZ-1
MBP99748.1	*Acidobacteriota bacterium*	VanZ-1
WP_144598545.1	*Aeribacillus composti*	VanZ-1
MBQ1238984.1	*Ruminococcus sp.*	VanZ-1
WP_162355816.1	*Paenibacillus lycopersici*	VanZ-1
MBQ5842143.1	*Clostridia bacterium*	VanZ-1
WP_076229158.1	*Paenibacillus sp. FSL H7-0331*	VanZ-1
WP_030562559.1	*Streptomyces sp. KY75*	VanZ-1
MBE6615986.1	*Oscillospiraceae bacterium*	VanZ-1
HJB74353.1	*Candidatus Eubacterium faecale*	VanZ-1
TMK18898.1	*Actinomycetota bacterium*	VanZ-1
MBN1192094.1	*Coriobacteriia bacterium*	VanZ-1

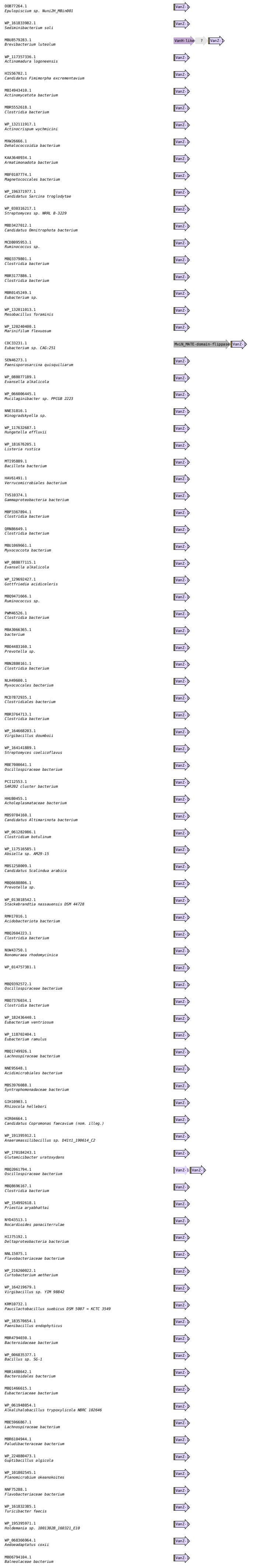
OOB77264.1
Epulopiscium sp. Nuni2H_MBin001
VanZ-1
WP_161833982.1
Sediminibacterium soli
VanZ-1
MBU8579283.1
Brevibacterium luteolum
VanH-like
?
VanZ-1
WP_117357336.1
Actinomadura logoneensis
VanZ-1
HIS56702.1
Candidatus Fimimorpha excrementavium
VanZ-1
MBI4943410.1
Actinomycetota bacterium
VanZ-1
MBR5552618.1
Clostridia bacterium
VanZ-1
WP_132111917.1
Actinocrispum wychmicini
VanZ-1
MXW26666.1
Dehalococcoidia bacterium
VanZ-1
KAA3640934.1
Armatimonadota bacterium
VanZ-1
MBF0107774.1
Magnetococcales bacterium
VanZ-1
WP_196371977.1
Candidatus Sarcina troglodytae
VanZ-1
WP_030316217.1
Streptomyces sp. NRRL B-3229
VanZ-1
MBD3427012.1
Candidatus Omnitrophota bacterium
VanZ-1
MCD8095953.1
Ruminococcus sp.
VanZ-1
MBQ3379801.1
Clostridia bacterium
VanZ-1
MBR3177886.1
Clostridia bacterium
VanZ-1
MBR0145249.1
Eubacterium sp.
VanZ-1
WP_132011013.1
Mesobacillus foraminis
VanZ-1
WP_120240400.1
Marinifilum flexuosum
VanZ-1
CDC33231.1
Eubacterium sp. CAG:251
MviN_MATE-domain-flippase
VanZ-1
SEN46273.1
Paenisporosarcina quisquiliarum
VanZ-1
WP_088077189.1
Evansella alkalicola
VanZ-1
WP_066006445.1
Mucilaginibacter sp. PPCGB 2223
VanZ-1
NNE31816.1
Winogradskyella sp.
VanZ-1
WP_117632687.1
Hungatella effluvii
VanZ-1
WP_181676205.1
Listeria rustica
VanZ-1
MTI95889.1
Bacillota bacterium
VanZ-1
HAV61491.1
Verrucomicrobiales bacterium
VanZ-1
TVS10374.1
Gammaproteobacteria bacterium
VanZ-1
MBP3367894.1
Clostridia bacterium
VanZ-1
QRN86649.1
Clostridia bacterium
VanZ-1
MBU1069661.1
Myxococcota bacterium
VanZ-1
WP_088077115.1
Evansella alkalicola
VanZ-1
WP_129692427.1
Gottfriedia acidiceleris
VanZ-1
MBQ9471666.1
Ruminococcus sp.
VanZ-1
PWM46526.1
Clostridia bacterium
VanZ-1
MBA3066365.1
bacterium
VanZ-1
MBO4483160.1
Prevotella sp.
VanZ-1
MBN2880161.1
Clostridia bacterium
VanZ-1
NLH49600.1
Myxococcales bacterium
VanZ-1
MCD7872935.1
Clostridiales bacterium
VanZ-1
MBR3764713.1
Clostridia bacterium
VanZ-1
WP_164668203.1
Virgibacillus doumboii
VanZ-1
WP_164141889.1
Streptomyces coelicoflavus
VanZ-1
MBE7000641.1
Oscillospiraceae bacterium
VanZ-1
PCI12553.1
SAR202 cluster bacterium
VanZ-1
HHU80455.1
Acholeplasmataceae bacterium
VanZ-1
MBS9784160.1
Candidatus Altimarinota bacterium
VanZ-1
WP_061282086.1
Clostridium botulinum
VanZ-1
WP_117516585.1
Absiella sp. AM29-15
VanZ-1
MBS1258009.1
Candidatus Scalindua arabica
VanZ-1
MBQ6680806.1
Prevotella sp.
VanZ-1
WP_013018542.1
Stackebrandtia nassauensis DSM 44728
VanZ-1
RMH17016.1
Acidobacteriota bacterium
VanZ-1
MBQ2604223.1
Clostridia bacterium
VanZ-1
NUW43750.1
Nonomuraea rhodomycinica
VanZ-1
WP_014757381.1
VanZ-1
MBQ9392572.1
Oscillospiraceae bacterium
VanZ-1
MBO7376034.1
Clostridia bacterium
VanZ-1
WP_182436440.1
Eubacterium ventriosum
VanZ-1
WP_118702404.1
Eubacterium ramulus
VanZ-1
MBQ1749926.1
Lachnospiraceae bacterium
VanZ-1
NNE95648.1
Acidimicrobiales bacterium
VanZ-1
MBS3976088.1
Syntrophomonadaceae bacterium
VanZ-1
GIH10903.1
Rhizocola hellebori
VanZ-1
HIR04664.1
Candidatus Copromonas faecavium (nom. illeg.)
VanZ-1
WP_191395912.1
Anaeromassilibacillus sp. D41t1_190614_C2
VanZ-1
WP_170184243.1
Glutamicibacter uratoxydans
VanZ-1
MBQ2061794.1
Oscillospiraceae bacterium
VanZ-1
VanZ-1
MBQ8696167.1
Clostridia bacterium
VanZ-1
WP_154992618.1
Priestia aryabhattai
VanZ-1
NYD43513.1
Nocardioides panaciterrulae
VanZ-1
HIJ75192.1
Deltaproteobacteria bacterium
VanZ-1
NNL15075.1
Flavobacteriaceae bacterium
VanZ-1
WP_216260022.1
Curtobacterium aetherium
VanZ-1
WP_164219679.1
Virgibacillus sp. YIM 98842
VanZ-1
KRM10732.1
Paucilactobacillus suebicus DSM 5007 = KCTC 3549
VanZ-1
WP_183570654.1
Paenibacillus endophyticus
VanZ-1
MBR4794030.1
Bacteroidaceae bacterium
VanZ-1
WP_006835377.1
Bacillus sp. SG-1
VanZ-1
MBR1488642.1
Bacteroidales bacterium
VanZ-1
MBQ1466615.1
Eubacteriaceae bacterium
VanZ-1
WP_061948054.1
Alkalihalobacillus trypoxylicola NBRC 102646
VanZ-1
MBE5966867.1
Lachnospiraceae bacterium
VanZ-1
MBR6104944.1
Paludibacteraceae bacterium
VanZ-1
WP_224880473.1
Guptibacillus algicola
VanZ-1
WP_101802545.1
Planomicrobium okeanokoites
VanZ-1
NNF75288.1
Flavobacteriaceae bacterium
VanZ-1
WP_161832385.1
Turicibacter faecis
VanZ-1
WP_195395971.1
Holdemania sp. 1001302B_160321_E10
VanZ-1
WP_068366964.1
Aedoeadaptatus coxii
VanZ-1
MBO6794104.1
Balneolaceae bacterium
VanZ-1

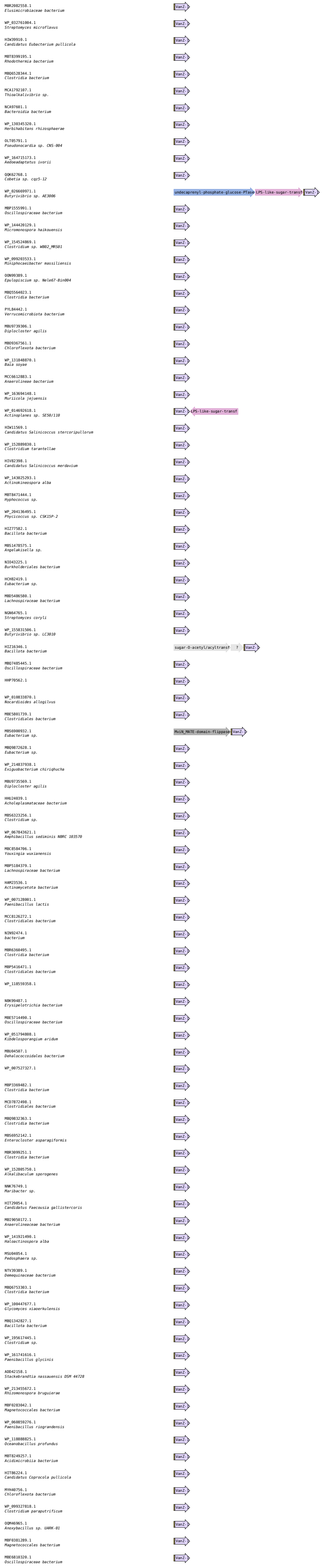
MBR2082558.1 Elusimicrobiaceae bacterium
WP_032761004.1 Streptomyces microflavus
HIW39910.1 Candidatus Eubacterium pullicola
MBT8399195.1 Rhodothermia bacterium
MBQ6528344.1 Clostridia bacterium
MCA1792107.1 Thioalkalivibrio sp.
NCA97601.1 Bacteroidia bacterium
WP_130345320.1 Herbihabitans rhizosphaerae
OLT05791.1 Pseudonocardia sp. CNS-004
WP_164715173.1 Aedoeadaptatus ivorii
QQK62768.1 Cobetia sp. cqz5-12
WP_026669971.1 Butyrivibrio sp. AE3006
undecaprenyl-phosphate-glucose-PTase
LPS-like-sugar-transf
MBP1555991.1 Oscillospiraceae bacterium
WP_144420129.1 Micromonospora haikouensis
WP_154524869.1 Clostridium sp. WB02_MRS01
WP_099203533.1 Miniphocaeibacter massiliensis
OON99389.1 Epulopiscium sp. Nele67-Bin004
MBQ5564023.1 Clostridia bacterium
PYL84442.1 Verrucomicrobiota bacterium
MBU9739306.1 Diplocloster agilis
MBO9367561.1 Chloroflexota bacterium
WP_131848870.1 Baia soyae
MCC6612883.1 Anaerolineae bacterium
WP_163694148.1 Muriicola jejuensis
WP_014692618.1 Actinoplanes sp. SE50/110
LPS-like-sugar-transf
HIW11569.1 Candidatus Salinicoccus stercoripullorum
WP_152889830.1 Clostridium tarantellae
HIV82398.1 Candidatus Salinicoccus merdavium
WP_143025293.1 Actinokineospora alba
MBT8471444.1 Hyphococcus sp.
WP_204136495.1 Phycicoccus sp. CSK15P-2
HIZ77582.1 Bacillota bacterium
MBS1478575.1 Angelakisella sp.
NIO43225.1 Burkholderiales bacterium
HCH82419.1 Eubacterium sp.
MBD5486580.1 Lachnospiraceae bacterium
NGN64765.1 Streptomyces coryli
WP_155831506.1 Butyrivibrio sp. LC3010
HIZ16346.1 Bacillota bacterium
sugar-O-acetyl/acyltransf
?
MBQ7485445.1 Oscillospiraceae bacterium
HHP70562.1
WP_010833870.1 Nocardioides allogilvus
MBE5801739.1 Clostridiales bacterium
MBS6900932.1 Eubacterium sp.
MviN_MATE-domain-flippase
MBQ9872628.1 Eubacterium sp.
WP_214837938.1 Exiguobacterium chiriqhucha
MBU9735569.1 Diplocloster agilis
HHU24039.1 Acholeplasmataceae bacterium
MBS6323256.1 Clostridium sp.
WP_067843621.1 Amphibacillus sediminis NBRC 103570
MBC8584706.1 Youxingia wuxianensis
MBP5184379.1 Lachnospiraceae bacterium
HAM23536.1 Actinomycetota bacterium
WP_007128001.1 Paenibacillus lactis
MCC8126272.1 Clostridiales bacterium
NIN92474.1 bacterium
MBR6360495.1 Clostridia bacterium
MBP5416471.1 Clostridiales bacterium
WP_118559358.1
NBK99487.1 Erysipelotrichia bacterium
MBE5714490.1 Oscillospiraceae bacterium
WP_051794808.1 Kibdelosporangium aridum
MBU04507.1 Dehalococcoidales bacterium
WP_007527327.1
MBP3369482.1 Clostridia bacterium
MCD7872498.1 Clostridiales bacterium
MBQ9832363.1 Clostridia bacterium
MBS6952142.1 Enterocloster asparagiformis
MBR3099251.1 Clostridia bacterium
WP_152805750.1 Alkalibaculum sporogenes
NNK76749.1 Maribacter sp.
HIT29054.1 Candidatus Faecousia gallistercoris
MBI9050172.1 Anaerolineaceae bacterium
WP_141921490.1 Haloactinospora alba
MSU04054.1 Pedosphaera sp.
NTV39389.1 Demequinaceae bacterium
MBQ6753303.1 Clostridia bacterium
WP_100447677.1 Glycomyces xiaoerkulensis
MBQ1342827.1 Bacillota bacterium
WP_195617445.1 Clostridium sp.
WP_161741616.1 Paenibacillus glycinis
ADD42158.1 Stackebrandtia nassauensis DSM 44728
WP_213455672.1 Rhizomonospora bruguierae
MBF0283042.1 Magnetococcales bacterium
WP_060859276.1 Paenibacillus riograndensis
WP_118888825.1 Oceanobacillus profundus
MBT8249257.1 Acidimicrobiia bacterium
HIT86224.1 Candidatus Coprocola pullicola
MYH40756.1 Chloroflexota bacterium
WP_099327818.1 Clostridium paraputrificum
OQM46965.1 Anoxybacillus sp. UARK-01
MBF0381289.1 Magnetococcales bacterium
MBE6810320.1 Oscillospiraceae bacterium

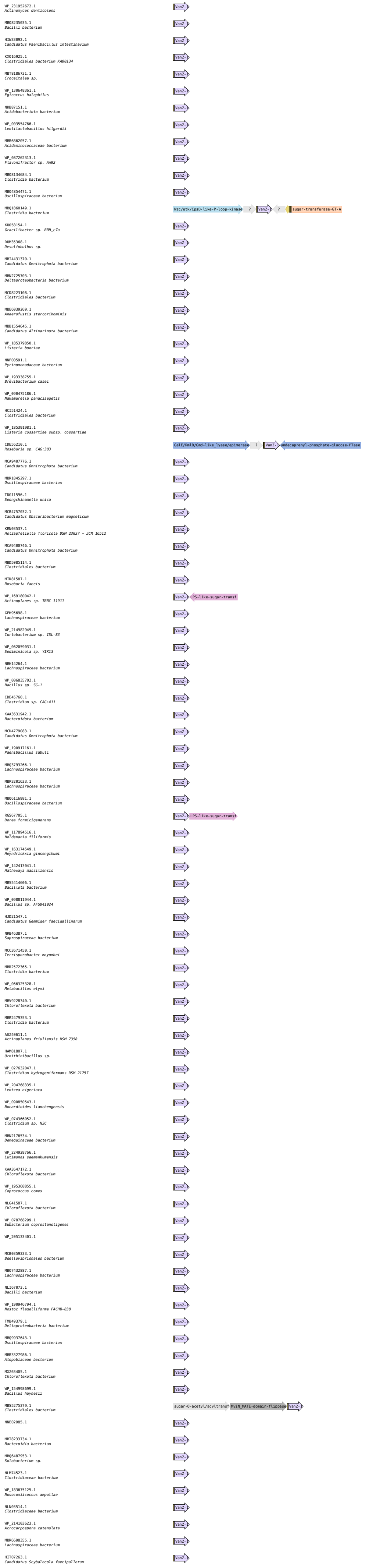
WP_231952672.1 Actinomyces denticolens VanZ-1
MBQ8235035.1 Bacilli bacterium VanZ-1
HIW33092.1 Candidatus Paenibacillus intestinavium VanZ-1
KXO16925.1 Clostridiales bacterium KA00134 VanZ-1
MBT8186731.1 Croceitalea sp. VanZ-1
WP_130648361.1 Egicoccus halophilus VanZ-1
NKB87151.1 Acidobacteriota bacterium VanZ-1
WP_003554766.1 Lentilactobacillus hilgardii VanZ-1
MBR6862057.1 Acidaminococcaceae bacterium VanZ-1
WP_087262313.1 Flavonifractor sp. An92 VanZ-1
MBQ8134684.1 Clostridia bacterium VanZ-1
MBO4854471.1 Oscillospiraceae bacterium VanZ-1
MBQ1860149.1 Clostridia bacterium Wzc/etk/CpsD-like-P-loop-kinase ? VanZ-1 ? sugar-transferase-GT-A
KUO58154.1 Gracilibacter sp. BRH_c7a VanZ-1
RUM35368.1 Desulfobulbus sp. VanZ-1
MBI4431370.1 Candidatus Omnitrophota bacterium VanZ-1
MBN2725703.1 Deltaproteobacteria bacterium VanZ-1
MCD8223108.1 Clostridiales bacterium VanZ-1
MBE6039269.1 Anaerofustis stercorihominis VanZ-1
MBB1554645.1 Candidatus Altimarinota bacterium VanZ-1
WP_185379850.1 Listeria booriae VanZ-1
NNF00591.1 Pyrinomonadaceae bacterium VanZ-1
WP_193338755.1 Brevibacterium casei VanZ-1
WP_090475186.1 Nakamurella panacisegetis VanZ-1
HCI51424.1 Clostridiales bacterium VanZ-1
WP_185391981.1 Listeria cossartiae subsp. cossartiae VanZ-1
CDE56210.1 Roseburia sp. CAG:303 GalE/RmlB/Gmd-like_lyase/epimerase ? VanZ-1 undecaprenyl-phosphate-glucose-PTase
MCA9407776.1 Candidatus Omnitrophota bacterium VanZ-1
MBR1845297.1 Oscillospiraceae bacterium VanZ-1
TDG11596.1 Seongchinamella unica VanZ-1
MCB4757032.1 Candidatus Obscuribacterium magneticum VanZ-1
KRN03537.1 Holzapfeliella floricola DSM 23037 = JCM 16512 VanZ-1
MCA9400746.1 Candidatus Omnitrophota bacterium VanZ-1
MBD5085114.1 Clostridiales bacterium VanZ-1
MTR81587.1 Roseburia faecis VanZ-1
WP_169180042.1 Actinoplanes sp. TBRC 11911 VanZ-1 LPS-like-sugar-transf
GFH95698.1 Lachnospiraceae bacterium VanZ-1
WP_214982949.1 Curtobacterium sp. ISL-83 VanZ-1
WP_062059031.1 Sediminicola sp. YIK13 VanZ-1
NBH14264.1 Lachnospiraceae bacterium VanZ-1
WP_006835702.1 Bacillus sp. SG-1 VanZ-1
CDE45760.1 Clostridium sp. CAG:411 VanZ-1
KAA3631942.1 Bacteroidota bacterium VanZ-1
MCD4779083.1 Candidatus Omnitrophota bacterium VanZ-1
WP_190917161.1 Paenibacillus sabuli VanZ-1
MBQ3793266.1 Lachnospiraceae bacterium VanZ-1
MBP3201633.1 Lachnospiraceae bacterium VanZ-1
MBQ6116981.1 Oscillospiraceae bacterium VanZ-1
RGS67705.1 Dorea formicigenerans VanZ-1 LPS-like-sugar-transf
WP_117894516.1 Holdemania filiformis VanZ-1
WP_163174549.1 Heyndrickxia ginsengihumi VanZ-1
WP_142413041.1 Hathewaya massiliensis VanZ-1
MBS5414606.1 Bacillota bacterium VanZ-1
WP_098811944.1 Bacillus sp. AFS041924 VanZ-1
HJD21547.1 Candidatus Gemmiger faecigallinarum VanZ-1
NRB46387.1 Saprospiraceae bacterium VanZ-1
MCC3671450.1 Terrisporobacter mayombei VanZ-1
MBR2572365.1 Clostridia bacterium VanZ-1
WP_066325328.1 Metabacillus elymi VanZ-1
MBV9228340.1 Chloroflexota bacterium VanZ-1
MBR2479353.1 Clostridia bacterium VanZ-1
AGZ40611.1 Actinoplanes friuliensis DSM 7358 VanZ-1
HAM81807.1 Ornithinibacillus sp. VanZ-1
WP_027632047.1 Clostridium hydrogeniformans DSM 21757 VanZ-1
WP_204768335.1 Lentzea nigeriaca VanZ-1
WP_090850543.1 Nocardioides lianchengensis VanZ-1
WP_074366052.1 Clostridium sp. N3C VanZ-1
MBN2176534.1 Demequinaceae bacterium VanZ-1
WP_224928766.1 Lutimonas saemankumensis VanZ-1
KAA3647172.1 Chloroflexota bacterium VanZ-1
WP_195368855.1 Coprococcus comes VanZ-1
NLG41587.1 Chloroflexota bacterium VanZ-1
WP_078768299.1 Eubacterium coprostanoligenes VanZ-1
WP_205133401.1 VanZ-1
MCB0359333.1 Bdellovibrionales bacterium VanZ-1
MBQ7432887.1 Lachnospiraceae bacterium VanZ-1
NLI67073.1 Bacilli bacterium VanZ-1
WP_190946794.1 Nostoc flagelliforme FACHB-838 VanZ-1
TMB49379.1 Deltaproteobacteria bacterium VanZ-1
MBQ9937643.1 Oscillospiraceae bacterium VanZ-1
MBR3327986.1 Atopobiaceae bacterium VanZ-1
MXZ63405.1 Chloroflexota bacterium VanZ-1
WP_154998699.1 Bacillus haynesii VanZ-1
MBS5275379.1 Clostridiales bacterium sugar-O-acetyl/acyltransf MviN_MATE-domain-flippase VanZ-1
NNE02985.1 VanZ-1
MBT8233734.1 Bacteroidia bacterium VanZ-1
MBQ6487953.1 Solobacterium sp. VanZ-1
NLM74523.1 Clostridiaceae bacterium VanZ-1
WP_183675125.1 Nosocomiicoccus ampullae VanZ-1
NLN03514.1 Clostridiaceae bacterium VanZ-1
WP_214103623.1 Acrocarpospora catenulata VanZ-1
MBR6698355.1 Lachnospiraceae bacterium VanZ-1
HIT07263.1 Candidatus Scybalocola faecipullorum VanZ-1

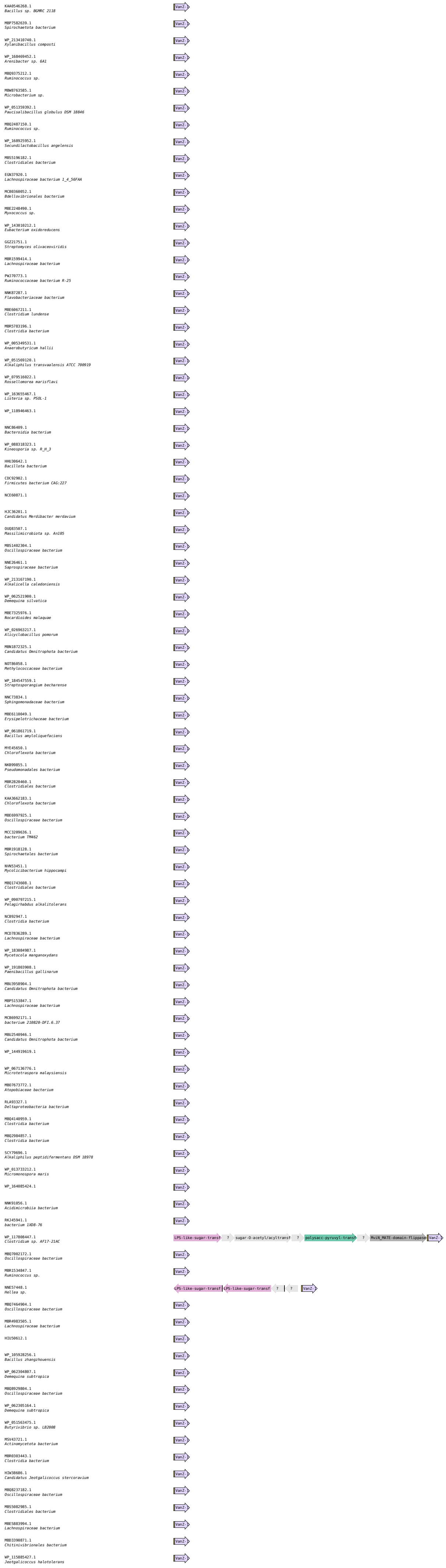
KAA0546268.1
Bacillus sp. BGMRC 2118
VanZ-1
MBP7582639.1
Spirochaetota bacterium
VanZ-1
WP_213410740.1
Xylanibacillus composti
VanZ-1
WP_168469452.1
Arenibacter sp. 6A1
VanZ-1
MBQ9375212.1
Ruminococcus sp.
VanZ-1
MBW8763585.1
Microbacterium sp.
VanZ-1
WP_051359392.1
Paucisalibacillus globulus DSM 18846
VanZ-1
MBQ2487150.1
Ruminococcus sp.
VanZ-1
WP_168925952.1
Secundilactobacillus angelensis
VanZ-1
MBS5196182.1
Clostridiales bacterium
VanZ-1
EGN37920.1
Lachnospiraceae bacterium 1_4_56FAA
VanZ-1
MCB0360052.1
Bdellovibrionales bacterium
VanZ-1
MBE2248490.1
Myxococcus sp.
VanZ-1
WP_143010212.1
Eubacterium oxidoreducens
VanZ-1
GGZ21751.1
Streptomyces olivaceoviridis
VanZ-1
MBR1599414.1
Lachnospiraceae bacterium
VanZ-1
PWJ70773.1
Ruminococcaceae bacterium R-25
VanZ-1
NNK87287.1
Flavobacteriaceae bacterium
VanZ-1
MBE6067211.1
Clostridium lundense
VanZ-1
MBR5783196.1
Clostridia bacterium
VanZ-1
WP_005349531.1
Anaerobutyricum hallii
VanZ-1
WP_051569120.1
Alkaliphilus transvaalensis ATCC 700919
VanZ-1
WP_079516022.1
Rossellomorea marisflavi
VanZ-1
WP_163655467.1
Listeria sp. PSOL-1
VanZ-1
WP_118946463.1
VanZ-1
NNC86409.1
Bacteroidia bacterium
VanZ-1
WP_088318323.1
Kineosporia sp. R_H_3
VanZ-1
HHU30642.1
Bacillota bacterium
VanZ-1
CDC92902.1
Firmicutes bacterium CAG:227
VanZ-1
NCE60871.1
VanZ-1
HJC36201.1
Candidatus Merdibacter merdavium
VanZ-1
OUQ83507.1
Massilimicrobiota sp. An105
VanZ-1
MBS1402304.1
Oscillospiraceae bacterium
VanZ-1
NNE26461.1
Saprospiraceae bacterium
VanZ-1
WP_213167190.1
Alkalicella caledoniensis
VanZ-1
WP_062521900.1
Demequina silvatica
VanZ-1
MBE7325976.1
Nocardioides malaquae
VanZ-1
WP_026963217.1
Alicyclobacillus pomorum
VanZ-1
MBN1872325.1
Candidatus Omnitrophota bacterium
VanZ-1
NOT86058.1
Methylococcaceae bacterium
VanZ-1
WP_184547559.1
Streptosporangium becharense
VanZ-1
NNC73834.1
Sphingomonadaceae bacterium
VanZ-1
MBE6110049.1
Erysipelotrichaceae bacterium
VanZ-1
WP_061861719.1
Bacillus amyloliquefaciens
VanZ-1
MYE45650.1
Chloroflexota bacterium
VanZ-1
NKB99855.1
Pseudomonadales bacterium
VanZ-1
MBR2820460.1
Clostridiales bacterium
VanZ-1
KAA3662183.1
Chloroflexota bacterium
VanZ-1
MBE6997925.1
Oscillospiraceae bacterium
VanZ-1
MCC3209636.1
bacterium TM462
VanZ-1
MBR1918128.1
Spirochaetales bacterium
VanZ-1
NVN53451.1
Mycolicibacterium hippocampi
VanZ-1
MBQ1743608.1
Clostridiales bacterium
VanZ-1
WP_090797215.1
Pelagirhabdus alkalitolerans
VanZ-1
NCB92947.1
Clostridia bacterium
VanZ-1
MCD7836289.1
Lachnospiraceae bacterium
VanZ-1
WP_183084987.1
Mycetocola manganoxydans
VanZ-1
WP_191803908.1
Paenibacillus gallinarum
VanZ-1
MBU3958904.1
Candidatus Omnitrophota bacterium
VanZ-1
MBP5153847.1
Lachnospiraceae bacterium
VanZ-1
MCB6992171.1
bacterium 210820-DFI.6.37
VanZ-1
MBU2540946.1
Candidatus Omnitrophota bacterium
VanZ-1
WP_144919619.1
VanZ-1
WP_067136776.1
Microtetraspora malaysiensis
VanZ-1
MBO7673772.1
Atopobiaceae bacterium
VanZ-1
RLA93327.1
Deltaproteobacteria bacterium
VanZ-1
MBQ4140959.1
Clostridia bacterium
VanZ-1
MBQ2984857.1
Clostridia bacterium
VanZ-1
SCY79696.1
Alkaliphilus peptidifermentans DSM 18978
VanZ-1
WP_013733212.1
Micromonospora maris
VanZ-1
WP_164085424.1
VanZ-1
NNK91056.1
Acidimicrobiia bacterium
VanZ-1
RKJ45941.1
bacterium 1XD8-76
VanZ-1
WP_117808447.1
Clostridium sp. AF17-21AC
LPS-like-sugar-transf
?
sugar-O-acetyl/acyltransf
?
polysacc-pyruvyl-transf
?
MviN_MATE-domain-flippase
VanZ-1
MBQ7002172.1
Oscillospiraceae bacterium
VanZ-1
MBR1534847.1
Ruminococcus sp.
VanZ-1
NNE57448.1
Hellea sp.
LPS-like-sugar-transf
LPS-like-sugar-transf
?
?
VanZ-1
MBQ7464904.1
Oscillospiraceae bacterium
VanZ-1
MBR4983505.1
Lachnospiraceae bacterium
VanZ-1
HIU50612.1
VanZ-1
WP_105928256.1
Bacillus zhangzhouensis
VanZ-1
WP_062304807.1
Demequina subtropica
VanZ-1
MBQ8929804.1
Oscillospiraceae bacterium
VanZ-1
WP_062305164.1
Demequina subtropica
VanZ-1
WP_051563475.1
Butyrivibrio sp. LB2008
VanZ-1
MSV43721.1
Actinomycetota bacterium
VanZ-1
MBR0303443.1
Clostridia bacterium
VanZ-1
HIW38686.1
Candidatus Jeotgalicoccus stercoravium
VanZ-1
MBQ8237182.1
Oscillospiraceae bacterium
VanZ-1
MBS5082985.1
Clostridiales bacterium
VanZ-1
MBE5883994.1
Lachnospiraceae bacterium
VanZ-1
MBD3390871.1
Chitinivibrionales bacterium
VanZ-1
WP_115885427.1
Jeotgalicoccus halotolerans
VanZ-1

WP_102273666.1	*Cytobacillus massiliigabonensis*	VanZ-1
MBD5508182.1	*Lachnospiraceae bacterium*	VanZ-1
WP_161219854.1	*Streptomyces murinus*	VanZ-1
QWC83828.1	*Nocardioidaceae bacterium*	VanZ-1
MBL6982076.1	*Anaerolineales bacterium*	VanZ-1
MBQ8763437.1	*Clostridia bacterium*	VanZ-1
MBR2177457.1	*Clostridia bacterium*	VanZ-1
MBQ8577352.1	*Clostridia bacterium*	sugar-transferase-GT-A; VanZ-1
MBI4934591.1	*Actinomycetota bacterium*	VanZ-1
AMT92596.1	*Brevibacterium linens*	VanZ-1
MCD8154308.1	*Clostridiales bacterium*	VanZ-1
MBR4941587.1	*Clostridia bacterium*	VanZ-1
KAA3625024.1	*Flavobacterium sp.*	VanZ-1
WP_143235023.1	*Paractinoplanes atraurantiacus*	VanZ-1; LPS-like-sugar-transf
MCC8067777.1	*Clostridiales bacterium*	VanZ-1
MBF0131221.1	*Magnetococcales bacterium*	VanZ-1
MBR3950070.1	*Oscillospiraceae bacterium*	VanZ-1
NNF33390.1	*Saprospiraceae bacterium*	VanZ-1
WP_052164910.1	*Listeria sp. SHR_NRA_18*	VanZ-1
GBU11202.1	*Erysipelotrichaceae bacterium*	VanZ-1
WP_191271324.1	*Nocardiopsis terrae*	VanZ-1
WP_087201072.1		VanZ-1
WP_205150583.1	*Mediterraneibacter glycyrrhizinilyticus*	VanZ-1
WP_195251918.1	*Romboutsia sp. 1001713B170207_170306_H8*	VanZ-1
WP_087305350.1	*Massilimicrobiota sp. An105*	VanZ-1
WP_101661653.1	*Bacillus canaveralius*	VanZ-1
QNM01408.1	*Simiaoa sunii*	VanZ-1
MBO4980423.1	*Lachnospiraceae bacterium*	VanZ-1
HJC31797.1	*Candidatus Anaerobutyricum faecale*	VanZ-1
WP_183933346.1	*Sphingomicrobium lutaoense*	VanZ-1
WP_075126798.1	*Actinophytocola xanthii*	VanZ-1
WP_002569795.1	*Enterocloster bolteae*	VanZ-1
MBO5337622.1	*Lachnospiraceae bacterium*	VanZ-1
WP_010073653.1	*Clostridium cellulovorans 743B*	VanZ-1
WP_152936752.1	*Limosilactobacillus pontis*	VanZ-1
CCX50136.1	*Clostridium sp. CAG:226*	VanZ-1
MXW33275.1	*Rhodothermaceae bacterium*	VanZ-1
MBO5219092.1	*Clostridia bacterium*	VanZ-1
WP_156278285.1	*Paenibacillus sp. NEAU-GSW1*	VanZ-1
MCB9070573.1	*Calditrichia bacterium*	VanZ-1
MBS6510642.1	*Clostridiales bacterium*	VanZ-1
MBA3691514.1	*Actinomycetota bacterium*	VanZ-1
WP_170219750.1	*Leucobacter komagatae*	VanZ-1
MCB9136635.1	*Anaerolineales bacterium*	VanZ-1
HAM69156.1	*Ruminococcus sp.*	VanZ-1
WP_030437014.1	*Actinoplanes subtropicus*	VanZ-1
MBQ9790284.1	*Clostridia bacterium*	VanZ-1
MBO7699661.1	*Eubacteriaceae bacterium*	VanZ-1
MBQ7521858.1	*Clostridia bacterium*	VanZ-1
WP_010680983.1	*Acetivibrio cellulolyticus CD2*	VanZ-1
WP_164744461.1	*Paenibacillus xylaniclasticus*	VanZ-1
HIZ19399.1	*Bacillota bacterium*	VanZ-1
CVI64999.1	*Eubacteriaceae bacterium CHKCI004*	VanZ-1
MBN1870222.1	*Candidatus Omnitrophota bacterium*	VanZ-1
WP_025907620.1	*Priestia flexa*	VanZ-1
CUO29345.1	*Hungatella hathewayi*	VanZ-1
MBO9658983.1	*Chitinophagaceae bacterium*	VanZ-1
WP_062137268.1	*Demequina aestuarii*	VanZ-1
WP_051860812.1	*Bacillus sp. SJS*	VanZ-1
MBQ5733888.1	*Lachnospiraceae bacterium*	VanZ-1
WP_007200159.1	*Fictibacillus macauensis ZFHKF-1*	VanZ-1
NLO86355.1	*Clostridiales bacterium*	VanZ-1
MBS6546054.1	*Butyricicoccus pullicaecorum*	VanZ-1
MBR2836028.1	*Coriobacteriales bacterium*	VanZ-1
MBE6692737.1	*Oscillospiraceae bacterium*	VanZ-1
MBU1221288.1	*Myxococcota bacterium*	VanZ-1
MBD9225130.1	*Clostridiales bacterium*	VanZ-1
MBF0293364.1	*Nitrospinota bacterium*	VanZ-1
MBQ1855676.1	*Anaerovibrio sp.*	sugar-tranf; VanZ-1
HIA54222.1	*Candidatus Melainabacteria bacterium*	VanZ-1
WP_119600938.1	*Paenibacillus nanensis*	VanZ-1
MBS1509495.1	*Bacteroidota bacterium*	VanZ-1
MBP2108001.1		VanZ-1
KAA3633050.1	*Calditrichota bacterium*	VanZ-1
HIT64549.1	*Candidatus Ventrimonas merdavium*	VanZ-1
MBO4404268.1	*Treponema sp.*	VanZ-1
MBE6718549.1	*Oscillospiraceae bacterium*	VanZ-1
WP_205144251.1	*Periweissella beninensis*	VanZ-1
WP_063696948.1	*Pediococcus inopinatus*	VanZ-1
MBU2540343.1	*Candidatus Omnitrophota bacterium*	VanZ-1
MBQ3756096.1	*Oscillospiraceae bacterium*	VanZ-1
WP_128219877.1	*Nocardioides yefusunii*	VanZ-1
MBC7279174.1	*Nocardioides sp.*	VanZ-1
MBQ6116437.1	*Oscillospiraceae bacterium*	VanZ-1
WP_058729036.1	*Curtobacterium oceanosedimentum*	VanZ-1
OGX33832.1	*Omnitrophica WOR_2 bacterium RIFCSPHIGHO2_02_FULL_52_10*	VanZ-1
WP_118604937.1		VanZ-1
MBO7506280.1	*Paludibacteraceae bacterium*	VanZ-1; LPS-like-sugar-transf
WP_184662438.1	*Texcoconibacillus texcoconensis*	VanZ-1
WP_184941963.1	*Planomonospora venezuelensis*	VanZ-1
MBQ8245350.1	*Oscillospiraceae bacterium*	VanZ-1
NQU36786.1	*Actinomycetota bacterium*	VanZ-1
NBI67180.1	*Pseudoflavonifractor sp. 60*	VanZ-1

KAB1439613.1 VanZ-1
Candidatus Galacturonibacter soehngenii

WP_184543094.1 VanZ-1
Streptosporangium becharense

SCJ20773.1 VanZ-1
uncultured Ruminococcus sp.

WP_205232316.1 VanZ-1
Desulfopila inferna

MBO4799667.1 VanZ-1
Lachnospiraceae bacterium

MBT8259218.1 VanZ-1
Bacteroidia bacterium

HIR28508.1 VanZ-1
Candidatus Choladousia intestinigallinarum

MBR2265043.1 VanZ-1
Bacillota bacterium

MBR5490278.1 VanZ-1
Oscillospiraceae bacterium

WP_063754410.1 VanZ-1
Streptomyces sp. NRRL S-1813

MBQ6370887.1 VanZ-1
Bacillota bacterium

MBA8931211.1 VanZ-1
Kutzneria viridogrisea

MBR5473205.1 VanZ-1
Clostridia bacterium

MBS3902583.1 VanZ-1
Dethiobacter sp.

MBO5373274.1 VanZ-1
Lachnospiraceae bacterium

WP_215701295.1 VanZ-1
Clostridium sp. MCC353

OCN05669.1 VanZ-1
Erysipelotrichaceae bacterium MTC7

NNC83491.1 VanZ-1
Flavobacteriales bacterium

WP_101790547.1 VanZ-1
Nonomuraea indica

MBS6347364.1 VanZ-1
Oscillospiraceae bacterium

RZT23896.1 VanZ-1
Fictibacillus sp. BK138

WP_118558339.1 VanZ-1
Lachnotalea sp. AF33-28

NLK95191.1 VanZ-1
Clostridiales bacterium

HIT23271.1 VanZ-1
Candidatus Faecimonas intestinavium

MBI4666823.1 VanZ-1
Nitrospinota bacterium

MBQ8993567.1 VanZ-1
Turicibacter sp.

CUN02273.1 VanZ-1
Faecalibacterium prausnitzii

#;VanZ-1+TM

HBU12798.1 VanZ-1 VanZ-1
Clostridiales bacterium

WP_205908472.1 VanZ-1

MBO5065980.1 VanZ-1
Clostridia bacterium

HCT95716.1 VanZ-1
Vagococcus sp.

WP_169332150.1 VanZ-1
Corynebacterium lubricantis DSM 45231

#;TM+VanZ-1

PZN12445.1 VanZ-1
Bacillota bacterium

#;TM+VanZ-1

MBR6073223.1 VanZ-1
Bacilli bacterium

MBA3781969.1 VanZ-1
Nocardioides sp.

WP_162253156.1 VanZ-1
Secundilactobacillus kimchicus

WP_002575896.1 VanZ-1
Enterocloster bolteae

MBQ4060294.1 VanZ-1
Lachnospiraceae bacterium

WP_232365334.1 VanZ-1
Lentilactobacillus fungorum

WP_087279653.1 VanZ-1
Eubacterium sp. An3

#;TM+VanZ-1

MBQ4542489.1 VanZ-1
Clostridia bacterium

HHZ17936.1 VanZ-1
Acholeplasmataceae bacterium

PZN77895.1 VanZ-1
Candidatus Methylumidiphilus alinenensis

OYT68965.1 VanZ-1
Armatimonadetes bacterium CP1_7O

NVP17652.1 VanZ-1
Candidatus Altimarinota bacterium

MBS6627832.1 VanZ-1
Clostridiales bacterium

NLC96234.1 VanZ-1
Erysipelotrichaceae bacterium

MBQ6365876.1 VanZ-1
Oscillospiraceae bacterium

WP_051515538.1 VanZ-1
Candidatus Blastococcus massiliensis

MBQ4537215.1 VanZ-1
Lachnospiraceae bacterium

WP_087159791.1 VanZ-1

HEG67073.1 VanZ-1

MBF0171042.1 VanZ-1
Nitrospinota bacterium

MBO4458382.1 VanZ-1
Butyrivibrio sp.

MBR3610088.1 VanZ-1
Oscillospiraceae bacterium

MBO6255257.1 VanZ-1
Bacteroidaceae bacterium

CDA59755.1 VanZ-1
Clostridium sp. CAG:524

MBF0352774.1 VanZ-1
SAR324 cluster bacterium

MBX3029569.1 VanZ-1
Chloroflexota bacterium

HIT74253.1 VanZ-1
Candidatus Avipropionibacterium avicola

WP_203865789.1 VanZ-1

MBI2905163.1 VanZ-1
Chloroflexota bacterium

WP_075680149.1 VanZ-1
Roseburia sp. 831b

NCB33599.1 VanZ-1
Erysipelotrichia bacterium

PZM97779.1 VanZ-1
Actinomycetota bacterium

MBQ9942855.1 VanZ-1
Clostridia bacterium

PZM94822.1 VanZ-1
Actinomycetota bacterium

MBP5631643.1 VanZ-1
Clostridia bacterium

KAB2889764.1 VanZ-1
Desulfobulbaceae bacterium

WP_160849799.1 VanZ-1
Pontibacillus yanchengensis

MBU0469011.1 VanZ-1
Candidatus Omnitrophota bacterium

MBQ5564913.1 VanZ-1
Clostridia bacterium

HIX68363.1 VanZ-1
Candidatus Anaerostipes excrementavium

MBO5022250.1 VanZ-1
Clostridia bacterium

MBP5331415.1 VanZ-1
Lachnospiraceae bacterium

WP_214413266.1 VanZ-1
Sphaerisporangium fuscum

GII67401.1 VanZ-1
Sphaerisporangium krabiense

RCG29115.1 VanZ-1
Sphaerisporangium album

WP_066717485.1 VanZ-1
Clostridium sp. Marseille-P299

MBA2569918.1 VanZ-1
Chloroflexota bacterium

KRN97108.1 VanZ-1
Furfurilactobacillus siliginis

PIW64851.1 VanZ-1
Candidatus Omnitrophica bacterium CG12_big_fil_rev_8_21_14_0_65_45_16

WP_203937943.1 VanZ-1
Spirilliplanes yamanashiensis

MBP5383997.1 VanZ-1
Lachnospiraceae bacterium

MBQ2854229.1 VanZ-1
Oscillospiraceae bacterium

WP_204464893.1 VanZ-1
Shouchella xiaoxiensis

WP_061961495.1 VanZ-1
Demequina flava

CRH08233.1 VanZ-1

MBI1839673.1 VanZ-1
Verrucomicrobiota bacterium

OGQ44634.1 VanZ-1
Deltaproteobacteria bacterium RIFCSPLOWO2_02_FULL_46_8

MBR5217269.1	*Oscillospiraceae bacterium*	VanZ-1
WP_129400483.1	*Nocardioides oleivorans*	VanZ-1
SCI76787.1	*uncultured Clostridium sp.*	VanZ-1
MCC6617555.1	*Chloroflexota bacterium*	VanZ-1
MBX6751154.1	*Micromonosporaceae bacterium*	VanZ-1
MBP8037171.1	*Desulfobulbus sp.*	VanZ-1
MBQ9324409.1	*Clostridia bacterium*	VanZ-1
MBA3883440.1	*Chthoniobacterales bacterium*	VanZ-1
MBQ8909309.1	*Clostridia bacterium*	VanZ-1
MBO5744209.1	*Clostridia bacterium*	VanZ-1
MBB2986412.1	*Terracoccus luteus*	VanZ-1
WP_131891407.1	*Jiangella asiatica*	VanZ-1
MBD9207147.1	*Clostridiales bacterium*	VanZ-1
MBQ5866679.1	*Oscillospiraceae bacterium*	VanZ-1
MBP3453520.1	*Clostridia bacterium*	WecC-like-oxidoreductase, VanZ-1
WP_169052728.1	*Pseudooceanicola onchidii*	sugar-O-acetyl/acyltransf, VanZ-1
MBN9098035.1	*Pseudonocardia sp.*	VanZ-1
WP_131847364.1	*Baia soyae*	VanZ-1
MBW1965706.1	*Deltaproteobacteria bacterium*	VanZ-1
OLB79602.1	*Actinobacteria bacterium 13_2_20CM_2_71_6*	VanZ-1
MBO6133192.1	*Lachnospiraceae bacterium*	VanZ-1
NDK07818.1	*Candidatus Altimarinota bacterium*	VanZ-1
MBQ6387134.1	*Ruminococcus sp.*	VanZ-1
MBQ7717850.1	*Clostridia bacterium*	VanZ-1
RJO63671.1	*Candidatus Omnitrophota bacterium*	VanZ-1
MBQ6931512.1	*Clostridia bacterium*	VanZ-1
MBD5480272.1	*Lachnospiraceae bacterium*	VanZ-1
OON98411.1	*Epulopiscium sp. Nele67-Bin004*	VanZ-1
RHC56509.1	*Enterocloster bolteae*	VanZ-1
MBS7240287.1	*Acetatifactor sp.*	VanZ-1
RHQ63393.1	*Blautia sp. AF25-12LB*	VanZ-1
MBR0091223.1	*Lachnospiraceae bacterium*	VanZ-1
HIR64993.1	*Candidatus Faecousia faecigallinarum*	VanZ-1
MBQ7714105.1	*Oscillospiraceae bacterium*	VanZ-1
MBD9050140.1	*Ruminococcus sp.*	VanZ-1
MBQ8683166.1	*Clostridia bacterium*	VanZ-1, VanZ-1
MBR4744447.1	*Oscillospiraceae bacterium*	VanZ-1
MBQ9791770.1	*Clostridia bacterium*	VanZ-1
WP_203900709.1	*Virgisporangium aliadipatigenens*	VanZ-1
WP_101574806.1	*Brevibacterium sp. 239c*	VanZ-1
SEF18951.1	*Jiangella alba*	VanZ-1
MBD8926157.1	*Agathobacter rectalis*	VanZ-1
WP_100407673.1	*Bacillus solitudinis*	VanZ-1
GGU97691.1	*Streptomyces albospinus*	VanZ-1
MBP0968113.1	*Oscillospiraceae bacterium*	VanZ-1
WP_170191803.1	*Saccharothrix syringae*	VanZ-1
WP_213138234.1	*Bacillus sp. FJAT-50079*	VanZ-1
MBN1622599.1	*Endomicrobiales bacterium*	VanZ-1
MBQ2311584.1	*Bacillota bacterium*	VanZ-1
MBE5811146.1	*Clostridiales bacterium*	VanZ-1
MTD60829.1	*Blautia luti DSM 14534 = JCM 17040*	VanZ-1
WP_078554389.1	*Alkalihalobacterium alkalicellulosilyticum*	VanZ-1
HFP63407.1		VanZ-1
MBQ5896896.1	*Oscillospiraceae bacterium*	VanZ-1
MBQ7327768.1	*Clostridia bacterium*	VanZ-1
PZM85731.1	*Candidatus Altimarinota bacterium*	VanZ-1
MBN1522659.1	*Candidatus Auribacterota bacterium*	VanZ-1
HJG06605.1	*Ligilactobacillus agilis*	VanZ-1
WP_181804210.1	*Streptomyces shenzhenensis subsp. oryzicola*	VanZ-1
MBL7198371.1	*Candidatus Omnitrophota bacterium*	VanZ-1
WP_102344810.1	*Bacillus sp. Marseille-P3661*	VanZ-1
MBR5248157.1	*Lachnospiraceae bacterium*	VanZ-1
HIV30150.1	*Candidatus Pullichristensenella excrementipullorum*	VanZ-1
MCD8331998.1	*Oscillospiraceae bacterium*	VanZ-1
EEJ39944.1	*Limosilactobacillus vaginalis DSM 5837 = ATCC 49540*	VanZ-1
HCH29717.1	*Oscillospiraceae bacterium*	VanZ-1
WP_088809892.1	*Listeria sp. ILCC810*	VanZ-1
HJB14189.1	*Candidatus Oscillibacter excrementigallinarum*	VanZ-1
MCA9405321.1	*Candidatus Omnitrophota bacterium*	VanZ-1
HJA21709.1	*Candidatus Limosilactobacillus intestinipullorum*	VanZ-1
PYS81151.1	*Acidobacteriota bacterium*	VanZ-1
MBR0130371.1	*Bacillota bacterium*	VanZ-1
WP_204595709.1	*Clostridium sardiniense*	VanZ-1
WP_075133631.1	*Actinophytocola xinjiangensis*	VanZ-1
MBQ8823440.1	*Lachnospiraceae bacterium*	VanZ-1
WP_154539057.1	*Parafannyhessea umbonata*	VanZ-1
GFI71467.1	*Clostridiales bacterium*	VanZ-1
WP_225254866.1	*Jidongwangia harbinensis*	VanZ-1
MBR4003446.1	*Clostridia bacterium*	VanZ-1
MBE6016176.1	*Lachnospiraceae bacterium*	VanZ-1
MBE5962350.1	*Lachnospiraceae bacterium*	VanZ-1, t
MBQ7933675.1	*Lachnospiraceae bacterium*	VanZ-1
MBQ7926336.1	*Lachnospiraceae bacterium*	VanZ-1
HIS45334.1	*Candidatus Scatomorpha merdigallinarum*	VanZ-1
WP_117153726.1	*Paraliobacillus quinghaiensis*	VanZ-1
NLK16949.1	*Clostridiales bacterium*	VanZ-1
WP_161804069.1	*Lysinibacillus macroides*	VanZ-1
WP_204436225.1	*Amphibacillus cookii*	VanZ-1
MBE6117425.1	*Erysipelotrichaceae bacterium*	VanZ-1
WP_119326161.1	*Companilactobacillus musae*	VanZ-1
HBA49132.1	*Lachnospiraceae bacterium*	VanZ-1
HIU11019.1	*Candidatus Avidehalobacter gallistercoris*	VanZ-1
MBE5974646.1	*Paenibacillaceae bacterium*	VanZ-1

NLY39560.1
Bacillota bacterium VanZ-1

HIY30803.1
Candidatus Mediterraneibacter avicola VanZ-1

MBQ9387568.1
Lachnospiraceae bacterium VanZ-1

WP_025580016.1
uncultured Blautia sp. VanZ-1

MCC5697775.1
Klebsiella pneumoniae VanZ-1

WP_026884898.1
Clostridium akagii DSM 12554 VanZ-1

MBS6518104.1
Clostridium sp. VanZ-1

MBE5807192.1
Clostridiales bacterium VanZ-1

WP_089949258.1
Lentzea xinjiangensis VanZ-1

MBR2812227.1
Solobacterium sp. VanZ-1

MBR3311000.1
Solobacterium sp. VanZ-1

WP_118523540.1
Clostridium sp. AF02-29 VanZ-1

WP_161831047.1
Turicibacter sp. KK003 VanZ-1

HHU20565.1
Bacilli bacterium VanZ-1

WP_062071122.1
Demequina sediminicola VanZ-1

MBI4436934.1
Candidatus Omnitrophota bacterium VanZ-1

AGY82056.1
Carnobacterium inhibens subsp. gilichinskyi VanZ-1

WP_152656082.1
Oceanobacillus sp. CFH 90083 VanZ-1

PWJ70817.1
Ruminococcaceae bacterium R-25 VanZ-1

HIZ08123.1
Candidatus Eubacterium avistercoris VanZ-1

GIG58018.1
Longispora fulva VanZ-1

KUK47160.1
Actinobacteria bacterium 66_15 VanZ-1

NNH75409.1
Nocardia uniformis VanZ-1

RMA94430.1
Priestia megaterium VanZ-1

MBQ7799324.1
Oscillospiraceae bacterium VanZ-1

MBD5542746.1
Lachnospiraceae bacterium VanZ-1

MBS6834880.1
Bacillota bacterium VanZ-1

MBX3438519.1
Planctomycetaceae bacterium VanZ-1

MBR3033197.1
Clostridiales bacterium VanZ-1

WP_090793342.1
Pelagirhabdus alkalitolerans VanZ-1

WP_182581705.1
Limosilactobacillus fastidiosus VanZ-1

WP_129399622.1
Nocardioides oleivorans VanZ-1

HCK44379.1
Oscillospiraceae bacterium VanZ-1

WP_010624151.1
Companilactobacillus versmoldensis DSM 14857 = KCTC 3814 VanZ-1

MBA3234838.1
Chloroflexota bacterium VanZ-1

KPK38012.1
Omnitrophica WOR_2 bacterium SM23_29 VanZ-1

HIW40131.1
Candidatus Eubacterium pullicola VanZ-1

MBQ9265347.1
Clostridia bacterium VanZ-1

NGY85603.1
Priestia megaterium VanZ-1

WP_116046177.1
Amycolatopsis palatopharyngis VanZ-1

NLF27127.1
Clostridiales bacterium VanZ-1

WP_015009153.1
Amphibacillus xylanus NBRC 15112 VanZ-1

WP_079710358.1
Paraliobacillus ryukyuensis VanZ-1

KRO14322.1
Lactiplantibacillus xiangfangensis VanZ-1

HGC23456.1 VanZ-1

MBQ7919220.1
Lachnospiraceae bacterium VanZ-1

HCX62175.1
Clostridiales bacterium VanZ-1

MBW1659676.1
Deltaproteobacteria bacterium VanZ-1

NBJ63594.1
bacterium c-19 VanZ-1

HJB94735.1
Candidatus Mediterraneibacter intestinigallinarum VanZ-1

HJA66378.1
Candidatus Mediterraneibacter cottocaccae VanZ-1

WP_075828626.1 VanZ-1

MBR6627438.1
Lachnospiraceae bacterium VanZ-1

MTA26161.1
Actinomycetota bacterium VanZ-1

MBO6195397.1
Bacilli bacterium VanZ-1

WP_119482839.1
Amnibacterium setariae VanZ-1

WP_129032483.1
Levilactobacillus suantsaii VanZ-1

ALS74109.2
Planococcus rifietoensis VanZ-1

MBR1984616.1
Clostridia bacterium VanZ-1

MBA3350200.1
Actinomycetota bacterium VanZ-1

WP_013257198.1
Desulfarculus baarsii DSM 2075 VanZ-1

MBT8206155.1
Eudoraea sp. VanZ-1

MBR3343867.1
Solobacterium sp. VanZ-1

MBE6816200.1
Oscillospiraceae bacterium VanZ-1

MBO5114098.1
Lachnospiraceae bacterium VanZ-1

WP_008395246.1
Clostridium sp. M62/1 VanZ-1

MCC5948946.1
Nitriliruptoraceae bacterium VanZ-1

WP_014446010.1
Actinoplanes missouriensis VanZ-1

MBP5198768.1
Lachnospiraceae bacterium VanZ-1

WP_155936897.1
Mycobacteroides sp. CBMA 326 VanZ-1

WP_117942177.1
Ruminococcus sp. AF17-22AC VanZ-1

HIU43323.1
Candidatus Ventrousia excrementavium VanZ-1

MBE7082511.1
Clostridiales bacterium VanZ-1

MBD8608454.1
Aeromicrobium sp. CFBP 8757 VanZ-1

WP_132029144.1
Keratinibaculum paraultunense VanZ-1

MBD8972671.1
Clostridiales bacterium VanZ-1

MBQ1994330.1
Lachnospiraceae bacterium VanZ-1

ACZ21392.1
Sanguibacter keddieii DSM 10542 VanZ-1

WP_163178901.1 VanZ-1

WP_204026847.1
Sinosporangium siamense VanZ-1

WP_119247748.1 VanZ-1

NLP29977.1
Clostridiales bacterium VanZ-1

MBP3351055.1
Lachnospiraceae bacterium VanZ-1

QRQ96909.1
Lactiplantibacillus plantarum VanZ-1

MBO0850898.1
Pseudonocardia sp. VanZ-1

NQT23346.1
Candidatus Omnitrophota bacterium VanZ-1

WP_075517193.1 VanZ-1

MCD7922723.1
Clostridiales bacterium VanZ-1

EEQ59567.1
Clostridiales bacterium 1_7_47FAA VanZ-1

RUM57830.1
Persephonella sp. VanZ-1

MBM3251917.1
Candidatus Omnitrophota bacterium VanZ-1

NLL74288.1
Erysipelothrix sp. VanZ-1

HHU56021.1
Acholeplasmataceae bacterium VanZ-1

MBQ7248004.1
Lachnospiraceae bacterium
VanZ-1

MBQ6374890.1
Clostridia bacterium
VanZ-1

WP_196438971.1
Planomonospora sp. ID67723
VanZ-1

WP_203872779.1
Planobispora takensis
VanZ-1

WP_087442275.1
Paenibacillus thiaminolyticus
VanZ-1

WP_163770442.1
Nocardioides zeae
VanZ-1

WP_015473929.1
Levilactobacillus brevis
VanZ-1

MBR6666241.1
Lachnospiraceae bacterium
VanZ-1

WP_090063074.1
Lentzea flaviverrucosa
VanZ-1

WP_169440981.1
Streptomyces sp. HNM0574
VanZ-1

MBR7186985.1
Clostridia bacterium
VanZ-1

MBQ7777185.1
Lachnospiraceae bacterium
VanZ-1

CDA29539.1
Eubacterium sp. CAG:156
VanZ-1

HHI96293.1
Thermodesulfatator atlanticus
VanZ-1

MBN2297606.1
Deltaproteobacteria bacterium
VanZ-1

NLF26184.1
Deltaproteobacteria bacterium
VanZ-1

WP_181436430.1
Curtobacterium sp. MCBD17_026
VanZ-1

ENH96193.1
Gracilibacillus halophilus YIM-C55.5
VanZ-1

GIG45951.1
Dactylosporangium siamense
VanZ-1

WP_161635193.1
Desulfovermiculus halophilus DSM 18834
VanZ-1

NLM03978.1
Clostridiales bacterium
VanZ-1

QTU82509.1
Carnobacteriaceae bacterium zg-C25
VanZ-1

MBP1533609.1
Ruminococcus sp.
VanZ-1

MBE6158297.1
Bacillota bacterium
VanZ-1

WP_184951104.1
Paractinoplanes abujensis
VanZ-1

WP_106214395.1
Glaciihabitans tibetensis
VanZ-1

WP_162129534.1
Algibacter luteus
VanZ-1

HHU20043.1
Bacilli bacterium
VanZ-1

HCJ40659.1
Ruminococcus sp.
VanZ-1

WP_231952375.1
Latilactobacillus sakei
VanZ-1

NCD23942.1
Spartobacteria bacterium
VanZ-1

WP_087150177.1
Mediterraneibacter catenae
VanZ-1

MBQ7230589.1
Oscillospiraceae bacterium
VanZ-1

WP_191258743.1
Amycolatopsis oliviviridis
VanZ-1

CDA80032.1
Clostridium sp. CAG:594
VanZ-1

WP_223877025.1
Loigolactobacillus binensis
VanZ-1

MCC5872111.1
Gammaproteobacteria bacterium
WecC-like-oxidoreductase VanZ-1

MBE6730754.1
Oscillospiraceae bacterium
VanZ-1

WP_101330500.1
Halalkalibacillus sediminis
VanZ-1

CDD04677.1
Prevotella sp. CAG:592
VanZ-1

MBR3017325.1
Clostridia bacterium
VanZ-1

MBR2660031.1
Clostridia bacterium
VanZ-1

HIQ81871.1
Candidatus Pullichristensenella stercorigallinarum
VanZ-1

WP_191142139.1
Polycladospora coralii
VanZ-1

HIR08467.1
Candidatus Pullichristensenella stercoripullorum
VanZ-1

WP_170219526.1
Nocardioides litoris
VanZ-1

MBP3410177.1
Clostridia bacterium
VanZ-1

MBP5303805.1
Clostridia bacterium
VanZ-1

MBQ1234666.1
Oscillospiraceae bacterium
VanZ-1

MBR5094874.1
Oscillospiraceae bacterium
VanZ-1

HCK87283.1
Erysipelotrichaceae bacterium
VanZ-1

MBA2439734.1
Thermoleophilaceae bacterium
VanZ-1

WP_102195649.1
VanZ-1

KRK78961.1
Companilactobacillus nodensis DSM 19682 = JCM 14932 = NBRC 107160
VanZ-1

NLG21331.1
Actinomycetales bacterium
VanZ-1

MBR0136855.1
Erysipelotrichaceae bacterium
VanZ-1

MBR3342168.1
Clostridiales bacterium
VanZ-1

WP_075887636.1
Companilactobacillus crustorum
VanZ-1

MBQ2803660.1
Lachnospiraceae bacterium
VanZ-1

WP_033610703.1
Lactiplantibacillus paraplantarum
VanZ-1

MBK6762713.1
Micrococcales bacterium
VanZ-1

WP_051688388.1
Butyrivibrio sp. AE2032
VanZ-1

HIX28904.1
Candidatus Blautia stercoravium
VanZ-1

MBQ8185645.1
Clostridia bacterium
VanZ-1

MBQ4259798.1
Lachnospiraceae bacterium
VanZ-1

BCJ54240.1
Actinoplanes sp. NBRC 14428
VanZ-1

MBS6941666.1
Slackia piriformis
VanZ-1

WP_062419972.1
Levilinea saccharolytica
VanZ-1

#;VanZ-1+TM+TM

OLD98368.1
Candidatus Rokubacteria bacterium 13_1_20CM_4_68_9
VanZ-1

MCC6774310.1
Gemmatimonadaceae bacterium
VanZ-1

MBO5867025.1
Clostridia bacterium
VanZ-1

MCB6993118.1
bacterium 210820-DFI.6.37
VanZ-1

PYM11649.1
Candidatus Rokuibacteriota bacterium
VanZ-1

#;VanZ-1+TM

MBP1580774.1
Oscillospiraceae bacterium
VanZ-1

MBQ8566021.1
Clostridia bacterium
VanZ-1

HIW23564.1
Candidatus Acutalibacter stercoravium
VanZ-1

WP_119477981.1
Algoriphagus lacus
VanZ-1

WP_016147685.1
Butyricicoccus pullicaecorum
VanZ-1

ROR25818.1
Mobilisporobacter senegalensis
VanZ-1

WP_074223111.1
Algoriphagus halophilus
VanZ-1

WP_005287799.1
Corynebacterium sp. CCUG 65737
VanZ-1

KFL43753.1
Lysinibacillus sp. BF-4
VanZ-1

MBQ7375095.1
Clostridia bacterium
VanZ-1

WP_051643034.1
Agathobaculum desmolans ATCC 43058
VanZ-1

KHL04808.1
Sinomonas humi
VanZ-1

QDV05680.1
Saltatorellus ferox
VanZ-1

MBS5315232.1
Clostridiales bacterium
VanZ-1

MBS5084277.1
Clostridiales bacterium
VanZ-1

WP_024348283.1
Lacrimispora indolis SR3
VanZ-1

NBV01327.1
Burkholderiaceae bacterium
VanZ-1

WP_224117506.1
Flavihumibacter profundi
VanZ-1

MBQ1261674.1
Clostridia bacterium
VanZ-1

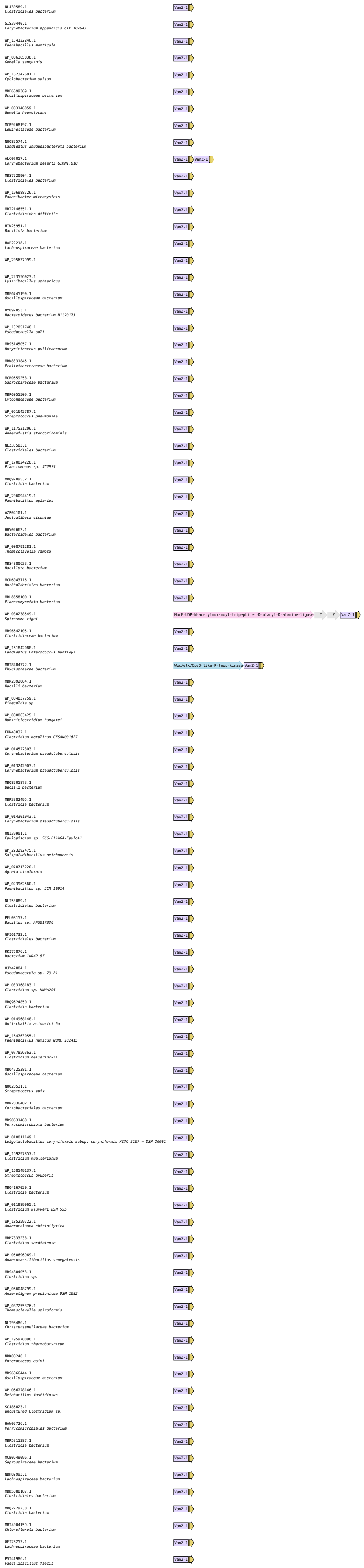
NLJ30589.1
Clostridiales bacterium
VanZ-1
SIS39440.1
Corynebacterium appendicis CIP 107643
VanZ-1
WP_154122246.1
Paenibacillus monticola
VanZ-1
WP_006365038.1
Gemella sanguinis
VanZ-1
WP_162342681.1
Cyclobacterium salsum
VanZ-1
MBE6699369.1
Oscillospiraceae bacterium
VanZ-1
WP_003146059.1
Gemella haemolysans
VanZ-1
MCB9268197.1
Lewinellaceae bacterium
VanZ-1
NUO82574.1
Candidatus Zhuqueibacterota bacterium
VanZ-1
ALC07057.1
Corynebacterium deserti GIMN1.010
VanZ-1
VanZ-1
MBS7220904.1
Clostridiales bacterium
VanZ-1
WP_196988726.1
Panacibacter microcysteis
VanZ-1
MBT2146551.1
Clostridioides difficile
VanZ-1
HIW25951.1
Bacillota bacterium
VanZ-1
HAP22218.1
Lachnospiraceae bacterium
VanZ-1
WP_205637999.1
VanZ-1
WP_223556023.1
Lysinibacillus sphaericus
VanZ-1
MBE6745190.1
Oscillospiraceae bacterium
VanZ-1
OYU92853.1
Bacteroidetes bacterium B1(2017)
VanZ-1
WP_132051748.1
Pseudocnuella soli
VanZ-1
MBS5145057.1
Butyricicoccus pullicaecorum
VanZ-1
MBW8331845.1
Prolixibacteraceae bacterium
VanZ-1
MCB0659258.1
Saprospiraceae bacterium
VanZ-1
MBP6055509.1
Cytophagaceae bacterium
VanZ-1
WP_061642787.1
Streptococcus pneumoniae
VanZ-1
WP_117531206.1
Anaerofustis stercorihominis
VanZ-1
NLZ33583.1
Clostridiales bacterium
VanZ-1
WP_170024228.1
Planctomonas sp. JC2975
VanZ-1
MBQ9709532.1
Clostridia bacterium
VanZ-1
WP_206094419.1
Paenibacillus apiarius
VanZ-1
AZP04101.1
Jeotgalibaca ciconiae
VanZ-1
HHV02662.1
Bacteroidales bacterium
VanZ-1
WP_008791281.1
Thomasclavelia ramosa
VanZ-1
MBS4880633.1
Bacillota bacterium
VanZ-1
MCD6043716.1
Burkholderiales bacterium
VanZ-1
MBL8858100.1
Planctomycetota bacterium
VanZ-1
WP_080238549.1
Spirosoma rigui
MurF-UDP-N-acetylmuramoyl-tripeptide--D-alanyl-D-alanine-ligase
?
?
VanZ-1
MBS6642105.1
Clostridiaceae bacterium
VanZ-1
WP_161842088.1
Candidatus Enterococcus huntleyi
VanZ-1
MBT8484772.1
Phycisphaerae bacterium
Wzc/etk/CpsD-like-P-loop-kinase
VanZ-1
MBR2892064.1
Bacilli bacterium
VanZ-1
WP_004837759.1
Finegoldia sp.
VanZ-1
WP_080063425.1
Ruminiclostridium hungatei
VanZ-1
EKN40832.1
Clostridium botulinum CFSAN001627
VanZ-1
WP_014522303.1
Corynebacterium pseudotuberculosis
VanZ-1
WP_013242903.1
Corynebacterium pseudotuberculosis
VanZ-1
MBQ8205873.1
Bacilli bacterium
VanZ-1
MBR3382495.1
Clostridia bacterium
VanZ-1
WP_014301043.1
Corynebacterium pseudotuberculosis
VanZ-1
ONI39901.1
Epulopiscium sp. SCG-B11WGA-EpuloA1
VanZ-1
WP_223292475.1
Salipaludibacillus neizhouensis
VanZ-1
WP_078713220.1
Agreia bicolorata
VanZ-1
WP_023962560.1
Paenibacillus sp. JCM 10914
VanZ-1
NLI53089.1
Clostridiales bacterium
VanZ-1
PEL08157.1
Bacillus sp. AFS017336
VanZ-1
GFI61732.1
Clostridiales bacterium
VanZ-1
RKI75876.1
bacterium 1xD42-87
VanZ-1
OJY47804.1
Pseudonocardia sp. 73-21
VanZ-1
WP_033168183.1
Clostridium sp. KNHs205
VanZ-1
MBQ9624850.1
Clostridia bacterium
VanZ-1
WP_014968148.1
Gottschalkia acidurici 9a
VanZ-1
WP_164763055.1
Paenibacillus humicus NBRC 102415
VanZ-1
WP_077856363.1
Clostridium beijerinckii
VanZ-1
MBQ4225281.1
Oscillospiraceae bacterium
VanZ-1
NQQ28531.1
Streptococcus suis
VanZ-1
MBR2836482.1
Coriobacteriales bacterium
VanZ-1
MBS0631468.1
Verrucomicrobiota bacterium
VanZ-1
WP_010011149.1
Loigolactobacillus coryniformis subsp. coryniformis KCTC 3167 = DSM 20001
VanZ-1
WP_169297857.1
Clostridium muellerianum
VanZ-1
WP_168549137.1
Streptococcus ovuberis
VanZ-1
MBQ4167020.1
Clostridia bacterium
VanZ-1
WP_011989065.1
Clostridium kluyveri DSM 555
VanZ-1
WP_185259722.1
Anaerocolumna chitinilytica
VanZ-1
MBM7833238.1
Clostridium sardiniense
VanZ-1
WP_050696969.1
Anaeromassilibacillus senegalensis
VanZ-1
MBS4804053.1
Clostridium sp.
VanZ-1
WP_066048799.1
Anaerotignum propionicum DSM 1682
VanZ-1
WP_087255376.1
Thomasclavelia spiroformis
VanZ-1
NLT98486.1
Christensenellaceae bacterium
VanZ-1
WP_195970098.1
Clostridium thermobutyricum
VanZ-1
NBK08240.1
Enterococcus asini
VanZ-1
MBS6866444.1
Oscillospiraceae bacterium
VanZ-1
WP_066228146.1
Metabacillus fastidiosus
VanZ-1
SCJ86823.1
uncultured Clostridium sp.
VanZ-1
HAW02726.1
Verrucomicrobiales bacterium
VanZ-1
MBR5311387.1
Clostridia bacterium
VanZ-1
MCB0649096.1
Saprospiraceae bacterium
VanZ-1
NBH82993.1
Lachnospiraceae bacterium
VanZ-1
MBD5088187.1
Clostridiales bacterium
VanZ-1
MBQ2729238.1
Clostridia bacterium
VanZ-1
MBT4004159.1
Chloroflexota bacterium
VanZ-1
GFI28253.1
Lachnospiraceae bacterium
VanZ-1
PST41986.1
Faecalibacillus faecis
VanZ-1

Accession / Organism	Genes
MBR2030286.1 *Clostridia bacterium*	VanZ-1
MBQ5355742.1 *Clostridia bacterium*	VanZ-1
WP_010756003.1 *Enterococcus pallens*	VanZ-1
MCC8099412.1 *Clostridiales bacterium*	VanZ-1
MBE5870997.1 *Lachnospiraceae bacterium*	VanZ-1
WP_190998768.1 *Peribacillus faecalis*	VanZ-1
PAW86734.1 *Opitutia bacterium Tous-C10FEB*	VanZ-1
HIX82231.1 *Candidatus Erysipelatoclostridium merdavium*	VanZ-1
WP_065547567.1	VanZ-1
WP_010740811.1 *Enterococcus malodoratus*	VanZ-1
MBK9258426.1 *Polyangiaceae bacterium*	VanZ-1
NRA95250.1 *Planctomycetota bacterium*	VanZ-1
EFM44892.1 *Corynebacterium accolens ATCC 49726*	VanZ-1
ALC07056.1 *Corynebacterium deserti GIMN1.010*	VanZ-1 VanZ-1
MBL8863491.1 *Planctomycetota bacterium*	VanZ-1
MBM3991310.1 *Planctomycetota bacterium*	VanZ-1
APT85723.1 *Corynebacterium aquilae DSM 44791*	VanZ-1
EOS45756.1 *Lachnospiraceae bacterium A2*	VanZ-1

#;TM+VanZ-1

Accession / Organism	Genes
WP_144720575.1 *Agrococcus jejuensis*	VanZ-1
MBS7141414.1 *Clostridiales bacterium*	VanZ-1
WP_117208784.1 *Allorhizocola rhizosphaerae*	VanZ-1
WP_066980074.1 *Streptomyces sp. NRRL F-4489*	VanZ-1
WP_051551188.1 *Kandleria vitulina MC3001*	VanZ-1
WP_024285109.1 *Cellulomonas sp. KRMCY2*	VanZ-1
WP_130848030.1 *Lactiplantibacillus mudanjiangensis*	VanZ-1
NLA34793.1 *Actinomycetota bacterium*	VanZ-1
WP_051999882.1 *Schleiferilactobacillus shenzhenensis LY-73*	VanZ-1
WP_102235265.1 *Brachybacterium sp. Marseille-Q7125*	VanZ-1
WP_075132164.1 *Actinophytocola xinjiangensis*	VanZ-1
WP_061776519.1 *Levilactobacillus senmaizukei DSM 21775 = NBRC 103853*	VanZ-1
MBQ9951103.1 *Clostridia bacterium*	VanZ-1
WP_141859506.1 *Curtobacterium citreum*	VanZ-1
WP_033289204.1 *Amycolatopsis jejuensis*	VanZ-1
WP_033293241.1 *Amycolatopsis jejuensis*	VanZ-1
WP_106186639.1 *Umezawaea tangerina*	VanZ-1
MBR3289867.1 *Clostridia bacterium*	MviN_MATE-domain-flippase ? VanZ-1
WP_116209256.1 *Streptoverticillium olivoreticuli subsp. olivoreticuli*	VanZ-1
WP_183392953.1 *Kineococcus radiotolerans*	VanZ-1
WP_118620472.1 *Faecalicatena orotica*	VanZ-1
RUQ07173.1 *Curtobacterium sp. HSID17257*	VanZ-1
WP_131403522.1 *Aeromicrobium sp. IC_218*	VanZ-1
WP_103565808.1 *Actinomadura rubteroloni*	VanZ-1
KFB73491.1 *Candidatus Accumulibacter phosphatis*	VanZ-1
WP_183392490.1 *Kineococcus radiotolerans*	VanZ-1
MBW7456678.1 *Paenibacillus sepulcri*	VanZ-1
WP_066987495.1 *Streptomyces sp. NRRL F-4489*	VanZ-1
WP_156370024.1 *Frigoribacterium sp. Leaf164*	VanZ-1
WP_156178931.1 *Saccharothrix sp. ST-888*	VanZ-1
WP_130981690.1 *Glaciihabitans arcticus*	VanZ-1

#;VanZ-1+TM

Accession / Organism	Genes
RYZ44694.1 *Chitinophagaceae bacterium*	VanZ-1
WP_066686367.1 *Caulobacter sp. CCH9-E1*	VanZ-1
HIU44371.1 *Candidatus Ventrousia excrementavium*	VanZ-1
WP_077349208.1 *Algoriphagus sp. A40*	VanZ-1
MBQ8474615.1 *Clostridia bacterium*	VanZ-1
HGY91640.1 *Planctomycetota bacterium*	VanZ-1
MBC8581529.1 *Zhenhengia yiwuensis*	VanZ-1
NLY20428.1 *Tissierellia bacterium*	VanZ-1
WP_182533868.1 *Fontibacillus solani*	VanZ-1
WP_027643593.1 *Enterocloster clostridioformis*	VanZ-1
MBD5542729.1 *Lachnospiraceae bacterium*	VanZ-1
NFS30019.1 *Clostridium botulinum*	VanZ-1
MBN1047070.1 *Clostridium botulinum*	VanZ-1
NSB30214.1 *Clostridium saccharoperbutylacetonicum*	VanZ-1
MBR4186545.1 *Clostridia bacterium*	VanZ-1
MCC6726721.1 *Saprospiraceae bacterium*	VanZ-1
AHI23896.1 *Corynebacterium vitaeruminis DSM 20294*	VanZ-1
WP_075347444.1	VanZ-1
HJB18243.1 *Candidatus Bariatricus faecipullorum*	VanZ-1
MBQ1821632.1 *Clostridia bacterium*	VanZ-1
WP_170924762.1 *Candidatus Enterococcus clewellii*	VanZ-1
MBS6163268.1 *Clostridiales bacterium*	VanZ-1
MBQ1787058.1 *Turicibacter sp.*	VanZ-1
MCA1596301.1 *Chloroflexota bacterium*	VanZ-1
GFI50535.1 *Lachnospiraceae bacterium*	VanZ-1
TMI95841.1 *Bacteroidota bacterium*	VanZ-1
MBQ3527523.1 *Clostridia bacterium*	VanZ-1
HJB46565.1 *Candidatus Mediterraneibacter surreyensis*	VanZ-1
MBP6887950.1 *Oscillospiraceae bacterium*	VanZ-1
MCA8950573.1 *Planctomycetota bacterium*	VanZ-1
MBW1925291.1 *Deltaproteobacteria bacterium*	sugar-O-acetyl/acyltransf VanZ-1
MBQ2764657.1 *Bacillota bacterium*	VanZ-1
HIV68138.1 *Candidatus Butyricicoccus stercorigallinarum*	VanZ-1
MBO4563573.1 *Clostridia bacterium*	VanZ-1
MBQ7836028.1 *Clostridia bacterium*	VanZ-1
KAA3612782.1 *Planctomycetota bacterium*	VanZ-1
MBO46030.1 *Planctomycetota bacterium*	VanZ-1
WP_049945194.1 *Butyrivibrio sp. AC2005*	VanZ-1
MBQ1871065.1 *Lachnospiraceae bacterium*	VanZ-1
MBR6428522.1 *Clostridia bacterium*	VanZ-1
MBR6039945.1 *Clostridia bacterium*	VanZ-1
MBR4435364.1 *Clostridia bacterium*	VanZ-1
RKW24701.1 *Candidatus Altimarinota bacterium*	VanZ-1

UFH57430.1	*Spirosoma sp. KNUC1025*	VanZ-1
WP_151844004.1	*Corynebacterium sp. zg254*	VanZ-1
MBR5366023.1	*Clostridia bacterium*	VanZ-1
HIY32026.1	*Candidatus Evtepia faecavium*	VanZ-1
WP_102273890.1	*Cytobacillus massiliigabonensis*	VanZ-1
MBL7008699.1	*Planctomycetota bacterium*	VanZ-1
WP_008088534.1	*Streptococcus ictaluri 707-05*	VanZ-1
MBO6053772.1	*Clostridia bacterium*	VanZ-1
MBP5230635.1	*Clostridia bacterium*	VanZ-1
MBP5661889.1	*Clostridia bacterium*	VanZ-1
MBS5703747.1	*Butyricicoccus pullicaecorum*	VanZ-1
WP_023607000.1	*Vagococcus lutrae*	VanZ-1
MBQ9843405.1	*Oscillospiraceae bacterium*	VanZ-1
MBE6574108.1	*Oscillospiraceae bacterium*	VanZ-1
MBQ3894582.1	*Clostridia bacterium*	VanZ-1
MBQ3572016.1	*Clostridia bacterium*	VanZ-1
MBQ3154566.1	*Clostridia bacterium*	VanZ-1
MBQ1704965.1	*Clostridia bacterium*	VanZ-1
QJA08624.1	*Romboutsia sp. CE17*	VanZ-1
HJC51132.1	*Candidatus Anaerostipes avistercoris*	VanZ-1
MBS4969033.1	*Lachnospiraceae bacterium*	VanZ-1
HIY56664.1	*Candidatus Tetragenococcus pullicola*	VanZ-1
MBS5129342.1	*Lachnospiraceae bacterium*	VanZ-1
MBQ9716099.1	*Clostridia bacterium*	VanZ-1
MBQ7241584.1	*Bacillota bacterium*	VanZ-1
MBR4926610.1	*Prevotella sp.*	VanZ-1
MBL8765953.1	*Planctomycetota bacterium*	VanZ-1
MAW62313.1	*Planctomycetota bacterium*	VanZ-1
WP_011835330.1	*Lactococcus cremoris*	VanZ-1
RRC92698.1	*Erysipelotrichaceae bacterium OH741_COT-311*	VanZ-1
ERI68052.1	*Clostridium sp. KLE 1755*	VanZ-1
MBQ4350496.1	*Clostridia bacterium*	VanZ-1
HIT35214.1	*Candidatus Faecousia intestinavium*	VanZ-1
MBP5245907.1	*Clostridia bacterium*	VanZ-1
HIS59614.1	*Candidatus Faecousia faecipullorum*	VanZ-1
MBO7184917.1	*Oscillospiraceae bacterium*	VanZ-1
WP_087371456.1	*Butyricicoccus pullicaecorum*	VanZ-1
WP_142535328.1	*Peptacetobacter hominis*	VanZ-1
MBO5522195.1	*Roseburia sp.*	VanZ-1
MBO4384315.1	*Clostridia bacterium*	VanZ-1
MBO4883917.1	*Clostridia bacterium*	VanZ-1
HBI59909.1	*Lachnospiraceae bacterium*	VanZ-1
WP_051188347.1	*Streptococcus castoreus DSM 17536*	VanZ-1
WP_067088895.1	*Streptococcus marmotae*	VanZ-1
WP_087167978.1	*Sellimonas sp.*	VanZ-1
HIQ81960.1		VanZ-1
SKC64281.1	*[Eubacterium] yurii*	VanZ-1
CCZ33661.1	*Firmicutes bacterium CAG:646*	VanZ-1
MBR3397397.1	*Lachnospiraceae bacterium*	VanZ-1
KAA8704479.1	*Lactococcus lactis subsp. hordniae*	VanZ-1
WP_009615701.1	*Desulfosporosinus sp. OT*	VanZ-1
HBF3389408.1	*Clostridioides difficile*	VanZ-1
HJA06916.1	*Candidatus Mediterraneibacter pullicola*	VanZ-1
HJB01049.1	*Candidatus Mediterraneibacter merdavium*	VanZ-1
MBR5291153.1	*Erysipelotrichaceae bacterium*	VanZ-1
MBL4771754.1	*Planctomycetota bacterium*	VanZ-1
NLA80821.1	*Chloroflexota bacterium*	VanZ-1
NLZ37706.1	*Clostridiales bacterium*	VanZ-1
MCB5881296.1	*Lachnospiraceae bacterium EP-SM-12S-S03*	VanZ-1
MBP8900963.1	*Blautia sp.*	VanZ-1
WP_118245307.1		VanZ-1
MUV07118.1	*Planococcaceae bacterium Storch 2/2-2*	VanZ-1
WP_130434185.1		VanZ-1
WP_130589805.1	*Enterococcus faecium*	VanZ-1
MBA3893409.1	*Gemmatimonadales bacterium*	VanZ-1
OQX23250.1	*Desulfobacteraceae bacterium IS3*	VanZ-1
WP_067101744.1	*Streptococcus sp. DD13*	VanZ-1
ERL11861.1	*Coriobacteriaceae bacterium BV3Ac1*	VanZ-1
WP_076995559.1	*Streptococcus sp. 19428wD3_AN2*	VanZ-1
CDD06841.1	*Dorea sp. CAG:317*	VanZ-1
MCA8965247.1	*Planctomycetota bacterium*	VanZ-1
MBQ7077567.1	*Lachnospiraceae bacterium*	VanZ-1
MBM3977037.1	*Planctomycetota bacterium*	VanZ-1
MAG55487.1	*Planctomycetota bacterium*	VanZ-1
PCJ52481.1	*Planctomycetota bacterium*	VanZ-1
KKP70346.1	*candidate division CPR3 bacterium GW2011_GWF2_35_18*	VanZ-1
MBQ7595436.1	*Clostridia bacterium*	undecaprenyl-phosphate-glucose-PTase, VanZ-1
WP_016458501.1	*Corynebacterium pyruviciproducens ATCC BAA-1742*	VanZ-1
MCB9833947.1	*Planctomycetota bacterium*	VanZ-1
WP_142766775.1	*Lactococcus protaetiae*	VanZ-1
WP_164709455.1	*Enterococcus mediterraneensis*	qVanZ-1
TFG61922.1	*Gemmatimonadales bacterium*	VanZ-1
OFS21742.1	*Corynebacterium sp. HMSC04H06*	VanZ-1
AGS35978.1	*Corynebacterium maris DSM 45190*	VanZ-1
WP_105118724.1	*Streptococcus suis*	VanZ-1
WP_216279861.1	*Gemella sp. zg-570*	VanZ-1
HIV17370.1	*Candidatus Alectryocaccobium stercorigallinarum*	VanZ-1
WP_052298614.1	*Syntrophobotulus glycolicus DSM 8271*	VanZ-1
WP_087211700.1	*Mediterraneibacter glycyrrhizinilyticus*	VanZ-1
MBQ2723421.1	*Clostridia bacterium*	VanZ-1
MBR0104891.1	*Bacillota bacterium*	VanZ-1
WP_052113620.1	*Actinotalea fermentans*	VanZ-1
MCA8970273.1	*Planctomycetota bacterium*	VanZ-1

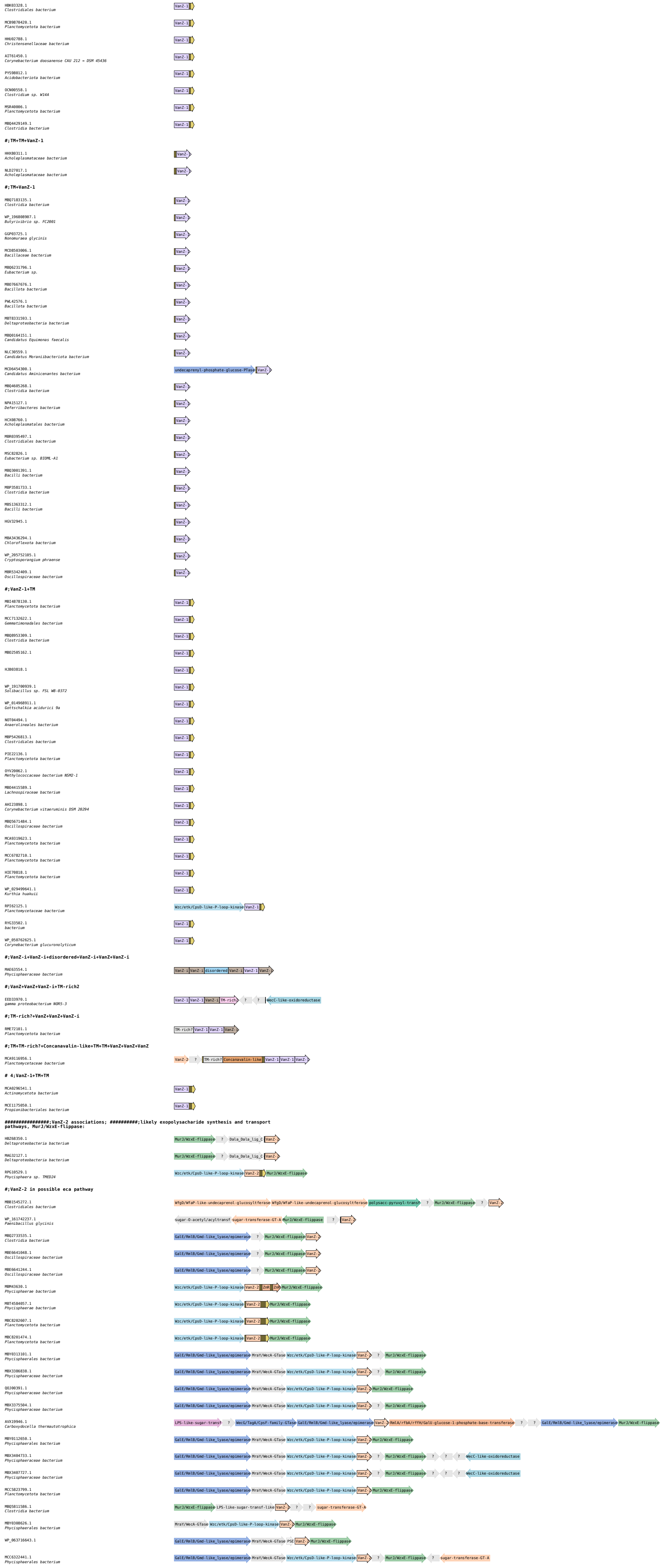

HBK03328.1
Clostridiales bacterium
VanZ-1
MCB9870420.1
Planctomycetota bacterium
VanZ-1
HHU02788.1
Christensenellaceae bacterium
VanZ-1
AIT61450.1
Corynebacterium doosanense CAU 212 = DSM 45436
VanZ-1
PYS98012.1
Acidobacteriota bacterium
VanZ-1
OCN00558.1
Clostridium sp. W14A
VanZ-1
MSR40006.1
Planctomycetota bacterium
VanZ-1
MBQ4429149.1
Clostridia bacterium
VanZ-1
#;TM+TM+VanZ-1
HHX80311.1
Acholeplasmataceae bacterium
VanZ-1
NLD27017.1
Acholeplasmataceae bacterium
VanZ-1
#;TM+VanZ-1
MBQ7183135.1
Clostridia bacterium
VanZ-1
WP_196808907.1
Butyrivibrio sp. FC2001
VanZ-1
GGP03725.1
Nonomuraea glycinis
VanZ-1
MCD8503006.1
Bacillaceae bacterium
VanZ-1
MBQ6231796.1
Eubacterium sp.
VanZ-1
MBO7667676.1
Bacillota bacterium
VanZ-1
PWL42576.1
Bacillota bacterium
VanZ-1
MBT8331593.1
Deltaproteobacteria bacterium
VanZ-1
MBQ0164151.1
Candidatus Equimonas faecalis
VanZ-1
NLC30559.1
Candidatus Moraniibacteriota bacterium
VanZ-1
MCD6454300.1
Candidatus Aminicenantes bacterium
undecaprenyl-phosphate-glucose-PTase VanZ-1
MBQ4605268.1
Clostridia bacterium
VanZ-1
NPA15127.1
Deferribacteres bacterium
VanZ-1
HCX08760.1
Acholeplasmatales bacterium
VanZ-1
MBR0395497.1
Clostridiales bacterium
VanZ-1
MSC82826.1
Eubacterium sp. BIOML-A1
VanZ-1
MBQ3001391.1
Bacilli bacterium
VanZ-1
MBP3581733.1
Clostridia bacterium
VanZ-1
MBS1363312.1
Bacilli bacterium
VanZ-1
HGV32945.1
VanZ-1
MBA3436294.1
Chloroflexota bacterium
VanZ-1
WP_205752105.1
Cryptosporangium phraense
VanZ-1
MBR5342409.1
Oscillospiraceae bacterium
VanZ-1
#;VanZ-1+TM
MBI4878130.1
Planctomycetota bacterium
VanZ-1
MCC7132622.1
Gemmatimonadales bacterium
VanZ-1
MBQ8953309.1
Clostridia bacterium
VanZ-1
MBO2505162.1
VanZ-1
HJB03818.1
VanZ-1
WP_191700939.1
Solibacillus sp. FSL W8-0372
VanZ-1
WP_014968911.1
Gottschalkia acidurici 9a
VanZ-1
NOT04494.1
Anaerolineales bacterium
VanZ-1
MBP5426813.1
Clostridiales bacterium
VanZ-1
PIE22136.1
Planctomycetota bacterium
VanZ-1
OYV20062.1
Methylococcaceae bacterium NSM2-1
VanZ-1
MBO4415589.1
Lachnospiraceae bacterium
VanZ-1
AHI23898.1
Corynebacterium vitaeruminis DSM 20294
VanZ-1
MBQ5671484.1
Oscillospiraceae bacterium
VanZ-1
MCA9319623.1
Planctomycetota bacterium
VanZ-1
MCC6782710.1
Planctomycetota bacterium
VanZ-1
HIE70818.1
Planctomycetota bacterium
VanZ-1
WP_029499641.1
Kurthia huakuii
VanZ-1
RPI62125.1
Planctomycetaceae bacterium
Wzc/etk/CpsD-like-P-loop-kinase VanZ-1
RYG33502.1
bacterium
VanZ-1
WP_050762625.1
Corynebacterium glucuronolyticum
VanZ-1
#;VanZ-i+VanZ-i+disordered+VanZ-i+VanZ+VanZ-i
MAE63554.1
Phycisphaeraceae bacterium
VanZ-i VanZ-i disordered VanZ-i VanZ-1 VanZ-i
#;VanZ+VanZ+VanZ-i+TM-rich2
EED33970.1
gamma proteobacterium NOR5-3
VanZ-1 VanZ-1 VanZ-i TM-rich2 ? ? WecC-like-oxidoreductase
#;TM-rich?+VanZ+VanZ+VanZ-i
RME72101.1
Planctomycetota bacterium
TM-rich? VanZ-1 VanZ-1 VanZ-i
#;TM+TM-rich?+Concanavalin-like+TM+TM+VanZ+VanZ+VanZ
MCA9116956.1
Planctomycetaceae bacterium
VanZ-2 ? TM-rich? Concanavalin-like VanZ-1 VanZ-1 VanZ-1
4;VanZ-1+TM+TM
MCA0296541.1
Actinomycetota bacterium
VanZ-1
MCE1175050.1
Propionibacteriales bacterium
VanZ-1
################;VanZ-2 associations; ##########;likely exopolysacharide synthesis and transport pathways, MurJ/WzxE-flippase:
HBZ68350.1
Deltaproteobacteria bacterium
MurJ/WzxE-flippase ? Dala_Dala_lig_C VanZ-2
MAG32127.1
Deltaproteobacteria bacterium
MurJ/WzxE-flippase ? Dala_Dala_lig_C VanZ-2
RPG10529.1
Phycisphaera sp. TMED24
Wzc/etk/CpsD-like-P-loop-kinase VanZ-2 MurJ/WzxE-flippase
#;VanZ-2 in possible eca pathway
MBB1545272.1
Clostridiales bacterium
WfgD/WfaP-like-undecaprenol-glucosyltferase WfgD/WfaP-like-undecaprenol-glucosyltferase polysacc-pyruvyl-transf ? MurJ/WzxE-flippase ? VanZ-2
WP_161742237.1
Paenibacillus glycinis
sugar-O-acetyl/acyltransf sugar-transferase-GT-A MurJ/WzxE-flippase ? VanZ-2
MBQ2733535.1
Clostridia bacterium
GalE/RmlB/Gmd-like_lyase/epimerase ? MurJ/WzxE-flippase VanZ-2
MBE6641048.1
Oscillospiraceae bacterium
GalE/RmlB/Gmd-like_lyase/epimerase ? MurJ/WzxE-flippase VanZ-2
MBE6641244.1
Oscillospiraceae bacterium
GalE/RmlB/Gmd-like_lyase/epimerase ? MurJ/WzxE-flippase VanZ-2
MBM43630.1
Phycisphaerae bacterium
Wzc/etk/CpsD-like-P-loop-kinase VanZ-2 ZnR ZnR MurJ/WzxE-flippase
MBT4584057.1
Phycisphaerae bacterium
Wzc/etk/CpsD-like-P-loop-kinase VanZ-2 MurJ/WzxE-flippase
MBC8202607.1
Planctomycetota bacterium
Wzc/etk/CpsD-like-P-loop-kinase VanZ-2 MurJ/WzxE-flippase
MBC8201474.1
Planctomycetota bacterium
Wzc/etk/CpsD-like-P-loop-kinase VanZ-2 MurJ/WzxE-flippase
MBY0313101.1
Phycisphaerales bacterium
GalE/RmlB/Gmd-like_lyase/epimerase MraY/WecA-GTase Wzc/etk/CpsD-like-P-loop-kinase VanZ-2 ? MurJ/WzxE-flippase
MBX3386838.1
Phycisphaeraceae bacterium
GalE/RmlB/Gmd-like_lyase/epimerase MraY/WecA-GTase Wzc/etk/CpsD-like-P-loop-kinase VanZ-2 ? MurJ/WzxE-flippase
QOJ00391.1
Phycisphaeraceae bacterium
GalE/RmlB/Gmd-like_lyase/epimerase MraY/WecA-GTase Wzc/etk/CpsD-like-P-loop-kinase VanZ-2 MurJ/WzxE-flippase
MBX3375504.1
Phycisphaeraceae bacterium
GalE/RmlB/Gmd-like_lyase/epimerase MraY/WecA-GTase Wzc/etk/CpsD-like-P-loop-kinase VanZ-2 ? MurJ/WzxE-flippase
AVX19946.1
Carboxydocella thermautotrophica
LPS-like-sugar-transf ? WecG/TagA/CpsF-family-GTase GalE/RmlB/Gmd-like_lyase/epimerase VanZ-2 RmlA/rfbA/rffH/GalU-glucose-1-phosphate-base-transferase ? ? GalE/RmlB/Gmd-like_lyase/epimerase MurJ/WzxE-flippase
MBY0112650.1
Phycisphaerales bacterium
GalE/RmlB/Gmd-like_lyase/epimerase MraY/WecA-GTase Wzc/etk/CpsD-like-P-loop-kinase VanZ-2 MurJ/WzxE-flippase
MBX3404733.1
Phycisphaeraceae bacterium
GalE/RmlB/Gmd-like_lyase/epimerase MraY/WecA-GTase Wzc/etk/CpsD-like-P-loop-kinase VanZ-2 ? MurJ/WzxE-flippase ? ? ? WecC-like-oxidoreductase
MBX3407727.1
Phycisphaeraceae bacterium
GalE/RmlB/Gmd-like_lyase/epimerase MraY/WecA-GTase Wzc/etk/CpsD-like-P-loop-kinase VanZ-2 ? MurJ/WzxE-flippase ? ? ? WecC-like-oxidoreductase
MCC5823799.1
Planctomycetota bacterium
GalE/RmlB/Gmd-like_lyase/epimerase MraY/WecA-GTase Wzc/etk/CpsD-like-P-loop-kinase VanZ-2 MurJ/WzxE-flippase
MBQ5811586.1
Clostridia bacterium
MurJ/WzxE-flippase LPS-like-sugar-transf-like VanZ-2 ? ? sugar-transferase-GT-A
MBY0308626.1
Phycisphaerales bacterium
MraY/WecA-GTase Wzc/etk/CpsD-like-P-loop-kinase VanZ-2 MurJ/WzxE-flippase
WP_063716643.1
GalE/RmlB/Gmd-like_lyase/epimerase MraY/WecA-GTase PSE VanZ-2 MurJ/WzxE-flippase
MCC6322441.1
Phycisphaerales bacterium
GalE/RmlB/Gmd-like_lyase/epimerase MraY/WecA-GTase Wzc/etk/CpsD-like-P-loop-kinase VanZ-2 ? MurJ/WzxE-flippase ? sugar-transferase-GT-A

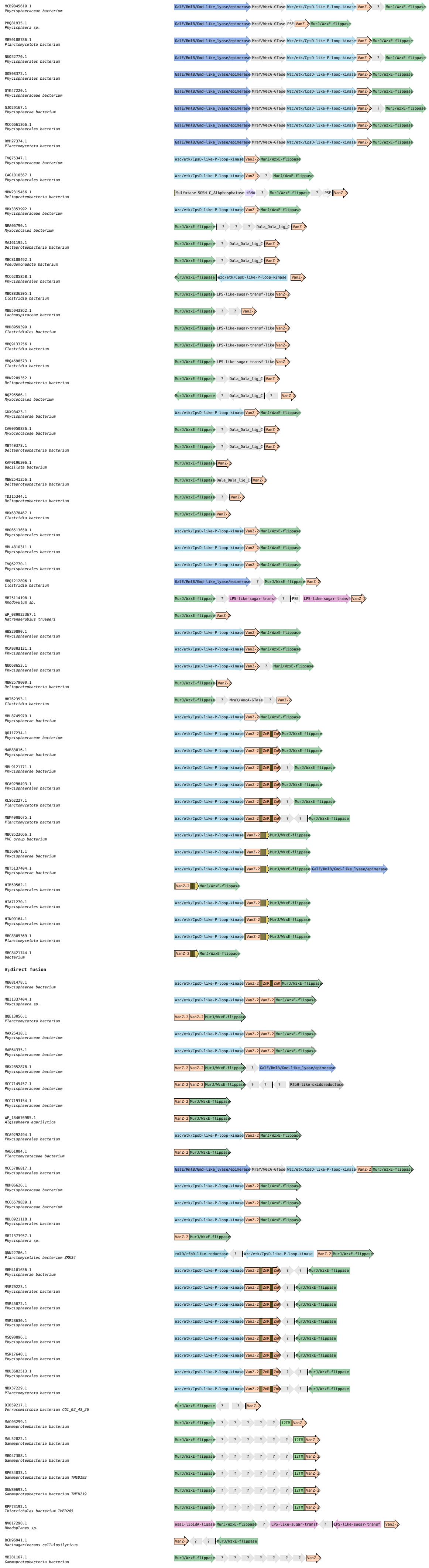
MCB9845619.1 Phycisphaeraceae bacterium: GalE/RmlB/Gmd-like_lyase/epimerase MraY/WecA-GTase Wzc/etk/CpsD-like-P-loop-kinase VanZ-2 ? MurJ/WzxE-flippase
PHQ81935.1 Phycisphaera sp.: GalE/RmlB/Gmd-like_lyase/epimerase MraY/WecA-GTase PSE VanZ-2 MurJ/WzxE-flippase
MBS0188786.1 Planctomycetota bacterium: GalE/RmlB/Gmd-like_lyase/epimerase MraY/WecA-GTase Wzc/etk/CpsD-like-P-loop-kinase VanZ-2 MurJ/WzxE-flippase
NUQ52770.1 Phycisphaerales bacterium: GalE/RmlB/Gmd-like_lyase/epimerase MraY/WecA-GTase Wzc/etk/CpsD-like-P-loop-kinase VanZ-2 ? MurJ/WzxE-flippase
QQS08372.1 Phycisphaerales bacterium: GalE/RmlB/Gmd-like_lyase/epimerase MraY/WecA-GTase Wzc/etk/CpsD-like-P-loop-kinase VanZ-2 MurJ/WzxE-flippase
QYK47220.1 Phycisphaeraceae bacterium: GalE/RmlB/Gmd-like_lyase/epimerase MraY/WecA-GTase Wzc/etk/CpsD-like-P-loop-kinase VanZ-2 MurJ/WzxE-flippase
GJQ29167.1 Phycisphaerae bacterium: GalE/RmlB/Gmd-like_lyase/epimerase MraY/WecA-GTase Wzc/etk/CpsD-like-P-loop-kinase VanZ-2 ? MurJ/WzxE-flippase
MCC6661366.1 Phycisphaerales bacterium: GalE/RmlB/Gmd-like_lyase/epimerase MraY/WecA-GTase Wzc/etk/CpsD-like-P-loop-kinase VanZ-2 MurJ/WzxE-flippase
RMH27374.1 Planctomycetota bacterium: GalE/RmlB/Gmd-like_lyase/epimerase MraY/WecA-GTase Wzc/etk/CpsD-like-P-loop-kinase VanZ-2 MurJ/WzxE-flippase
TVQ75347.1 Phycisphaeraceae bacterium: Wzc/etk/CpsD-like-P-loop-kinase VanZ-2 MurJ/WzxE-flippase
CAG1010567.1 Phycisphaerales bacterium: Wzc/etk/CpsD-like-P-loop-kinase VanZ-2 ? MurJ/WzxE-flippase
MBW2315456.1 Deltaproteobacteria bacterium: Sulfatase SGSH-C_Alkphosphatase tRNA ? MurJ/WzxE-flippase ? PSE VanZ-2
MBX3353992.1 Phycisphaeraceae bacterium: Wzc/etk/CpsD-like-P-loop-kinase VanZ-2 MurJ/WzxE-flippase
NRA06790.1 Myxococcales bacterium: MurJ/WzxE-flippase ? ? ? Dala_Dala_lig_C VanZ-2
MAJ61195.1 Deltaproteobacteria bacterium: MurJ/WzxE-flippase ? Dala_Dala_lig_C VanZ-2
MBC8188492.1 Pseudomonadota bacterium: MurJ/WzxE-flippase ? Dala_Dala_lig_C VanZ-2
MCC6285858.1 Phycisphaerales bacterium: MurJ/WzxE-flippase Wzc/etk/CpsD-like-P-loop-kinase VanZ-2
MBQ8836205.1 Clostridia bacterium: MurJ/WzxE-flippase LPS-like-sugar-transf-like VanZ-2
MBE5943862.1 Lachnospiraceae bacterium: MurJ/WzxE-flippase ? ? VanZ-2
MBD8959399.1 Clostridiales bacterium: MurJ/WzxE-flippase LPS-like-sugar-transf-like VanZ-2
MBQ9133256.1 Clostridia bacterium: MurJ/WzxE-flippase LPS-like-sugar-transf-like VanZ-2
MBQ4598573.1 Clostridia bacterium: MurJ/WzxE-flippase LPS-like-sugar-transf-like VanZ-2
MBW2289352.1 Deltaproteobacteria bacterium: MurJ/WzxE-flippase ? Dala_Dala_lig_C VanZ-2
NQZ95566.1 Myxococcales bacterium: MurJ/WzxE-flippase ? Dala_Dala_lig_C ? VanZ-2
GDX98423.1 Phycisphaerae bacterium: Wzc/etk/CpsD-like-P-loop-kinase VanZ-2 MurJ/WzxE-flippase
CAG0950836.1 Myxococcaceae bacterium: MurJ/WzxE-flippase ? Dala_Dala_lig_C VanZ-2
MBT40378.1 Deltaproteobacteria bacterium: MurJ/WzxE-flippase ? Dala_Dala_lig_C VanZ-2
KAF0196306.1 Bacillota bacterium: MurJ/WzxE-flippase VanZ-2
MBW2541356.1 Deltaproteobacteria bacterium: MurJ/WzxE-flippase Dala_Dala_lig_C VanZ-2
TDJ15344.1 Deltaproteobacteria bacterium: MurJ/WzxE-flippase ? VanZ-2
MBX6378467.1 Clostridia bacterium: MurJ/WzxE-flippase VanZ-2
MBO6513650.1 Phycisphaerales bacterium: Wzc/etk/CpsD-like-P-loop-kinase VanZ-2 MurJ/WzxE-flippase
MBL4810311.1 Phycisphaerales bacterium: Wzc/etk/CpsD-like-P-loop-kinase VanZ-2 MurJ/WzxE-flippase
TVQ62770.1 Phycisphaerales bacterium: Wzc/etk/CpsD-like-P-loop-kinase VanZ-2 MurJ/WzxE-flippase
MBQ1212896.1 Clostridia bacterium: GalE/RmlB/Gmd-like_lyase/epimerase ? MurJ/WzxE-flippase VanZ-2
MBI5114198.1 Rhodovulum sp.: MurJ/WzxE-flippase ? LPS-like-sugar-transf ? PSE LPS-like-sugar-transf VanZ-2
WP_089022367.1 Natranaerobius trueperi: MurJ/WzxE-flippase VanZ-2
HBS29890.1 Phycisphaerales bacterium: Wzc/etk/CpsD-like-P-loop-kinase VanZ-2 MurJ/WzxE-flippase
MCA9303121.1 Phycisphaerales bacterium: Wzc/etk/CpsD-like-P-loop-kinase VanZ-2 MurJ/WzxE-flippase
NUQ68653.1 Phycisphaerales bacterium: Wzc/etk/CpsD-like-P-loop-kinase VanZ-2 ? MurJ/WzxE-flippase
MBW2579000.1 Deltaproteobacteria bacterium: MurJ/WzxE-flippase VanZ-2
HHT62353.1 Clostridia bacterium: MurJ/WzxE-flippase ? MraY/WecA-GTase ? VanZ-2
MBL8745979.1 Phycisphaerae bacterium: Wzc/etk/CpsD-like-P-loop-kinase VanZ-2 MurJ/WzxE-flippase
QOJ17234.1 Phycisphaeraceae bacterium: Wzc/etk/CpsD-like-P-loop-kinase VanZ-2 ZnR ZnR MurJ/WzxE-flippase
MAB83016.1 Phycisphaerae bacterium: Wzc/etk/CpsD-like-P-loop-kinase VanZ-2 ZnR ZnR MurJ/WzxE-flippase
MBL9121771.1 Phycisphaerae bacterium: Wzc/etk/CpsD-like-P-loop-kinase VanZ-2 ZnR ZnR ? MurJ/WzxE-flippase
MCA9296493.1 Phycisphaerales bacterium: Wzc/etk/CpsD-like-P-loop-kinase VanZ-2 ZnR ZnR MurJ/WzxE-flippase
RLS62227.1 Planctomycetota bacterium: Wzc/etk/CpsD-like-P-loop-kinase VanZ-2 ZnR ZnR ? MurJ/WzxE-flippase
MBM4008675.1 Planctomycetota bacterium: Wzc/etk/CpsD-like-P-loop-kinase VanZ-2 ZnR ZnR ? ? MurJ/WzxE-flippase
MBC8523666.1 PVC group bacterium: Wzc/etk/CpsD-like-P-loop-kinase VanZ-2 MurJ/WzxE-flippase
MBI69671.1 Phycisphaerae bacterium: Wzc/etk/CpsD-like-P-loop-kinase VanZ-2 MurJ/WzxE-flippase
MBT5137404.1 Phycisphaerae bacterium: Wzc/etk/CpsD-like-P-loop-kinase VanZ-2 MurJ/WzxE-flippase GalE/RmlB/Gmd-like_lyase/epimerase
HIB50562.1 Phycisphaerales bacterium: VanZ-2 MurJ/WzxE-flippase
HIA71270.1 Phycisphaerales bacterium: Wzc/etk/CpsD-like-P-loop-kinase VanZ-2 MurJ/WzxE-flippase
HIN09164.1 Phycisphaerales bacterium: Wzc/etk/CpsD-like-P-loop-kinase VanZ-2 MurJ/WzxE-flippase
MBC8309369.1 Planctomycetota bacterium: Wzc/etk/CpsD-like-P-loop-kinase VanZ-2 MurJ/WzxE-flippase
MBC8421744.1 bacterium: VanZ-2 MurJ/WzxE-flippase
#;direct fusion
MBG81478.1 Phycisphaerae bacterium: Wzc/etk/CpsD-like-P-loop-kinase VanZ-2 ZnR ZnR MurJ/WzxE-flippase
MBI1337404.1 Phycisphaera sp.: Wzc/etk/CpsD-like-P-loop-kinase VanZ-2 VanZ-2 MurJ/WzxE-flippase
QQE13056.1 Planctomycetota bacterium: VanZ-2 VanZ-2 MurJ/WzxE-flippase
MAX25418.1 Phycisphaeraceae bacterium: Wzc/etk/CpsD-like-P-loop-kinase VanZ-2 VanZ-2 MurJ/WzxE-flippase
MAE64335.1 Phycisphaeraceae bacterium: Wzc/etk/CpsD-like-P-loop-kinase VanZ-2 VanZ-2 MurJ/WzxE-flippase
MBX2852878.1 Phycisphaeraceae bacterium: VanZ-2 VanZ-2 MurJ/WzxE-flippase ? GalE/RmlB/Gmd-like_lyase/epimerase
MCC7145457.1 Phycisphaeraceae bacterium: VanZ-2 VanZ-2 MurJ/WzxE-flippase ? ? ? RfbH-like-oxidoreductase
MCC7193154.1 Phycisphaeraceae bacterium: VanZ-2 MurJ/WzxE-flippase
WP_184676985.1 Algisphaera agarilytica: VanZ-2 MurJ/WzxE-flippase
MCA9292494.1 Phycisphaerales bacterium: Wzc/etk/CpsD-like-P-loop-kinase VanZ-2 MurJ/WzxE-flippase
MAE61004.1 Planctomycetaceae bacterium: VanZ-2 MurJ/WzxE-flippase
MCC5786817.1 Phycisphaerales bacterium: GalE/RmlB/Gmd-like_lyase/epimerase MraY/WecA-GTase Wzc/etk/CpsD-like-P-loop-kinase VanZ-2 MurJ/WzxE-flippase
MBH06626.1 Phycisphaeraceae bacterium: Wzc/etk/CpsD-like-P-loop-kinase VanZ-2 MurJ/WzxE-flippase
MCC6579839.1 Phycisphaeraceae bacterium: Wzc/etk/CpsD-like-P-loop-kinase VanZ-2 MurJ/WzxE-flippase
MBL0921118.1 Phycisphaerales bacterium: Wzc/etk/CpsD-like-P-loop-kinase VanZ-2 MurJ/WzxE-flippase
MBI1373957.1 Phycisphaera sp.: VanZ-2 MurJ/WzxE-flippase
QNN22786.1 Planctomycetales bacterium ZRK34: rmlD/rfbD-like-reductase ? Wzc/etk/CpsD-like-P-loop-kinase VanZ-2 MurJ/WzxE-flippase
MBM4101636.1 Phycisphaerae bacterium: Wzc/etk/CpsD-like-P-loop-kinase VanZ-2 ZnR ZnR ? ? MurJ/WzxE-flippase
MSR70223.1 Phycisphaerales bacterium: Wzc/etk/CpsD-like-P-loop-kinase VanZ-2 ZnR ZnR ? MurJ/WzxE-flippase
MSR45072.1 Phycisphaerales bacterium: Wzc/etk/CpsD-like-P-loop-kinase VanZ-2 ZnR ZnR ? MurJ/WzxE-flippase
MSR28630.1 Phycisphaerales bacterium: Wzc/etk/CpsD-like-P-loop-kinase VanZ-2 ZnR ZnR ? MurJ/WzxE-flippase
MSQ90896.1 Phycisphaerales bacterium: Wzc/etk/CpsD-like-P-loop-kinase VanZ-2 ZnR ZnR ? MurJ/WzxE-flippase
MSR17640.1 Phycisphaerales bacterium: Wzc/etk/CpsD-like-P-loop-kinase VanZ-2 ZnR ZnR ? MurJ/WzxE-flippase
MBU3682513.1 Phycisphaerales bacterium: Wzc/etk/CpsD-like-P-loop-kinase VanZ-2 ZnR ZnR ? ? MurJ/WzxE-flippase
NBX37229.1 Planctomycetota bacterium: Wzc/etk/CpsD-like-P-loop-kinase VanZ-2 ZnR ZnR ? ? MurJ/WzxE-flippase
OIO59217.1 Verrucomicrobia bacterium CG1_02_43_26: MurJ/WzxE-flippase ? ? VanZ-2
MAC03299.1 Gammaproteobacteria bacterium: MurJ/WzxE-flippase ? ? ? ? ? 12TM VanZ-2
MAL52822.1 Gammaproteobacteria bacterium: MurJ/WzxE-flippase ? ? ? ? ? ? 12TM VanZ-2
MBO47388.1 Gammaproteobacteria bacterium: MurJ/WzxE-flippase ? ? ? ? ? ? 12TM VanZ-2
RPG34833.1 Gammaproteobacteria bacterium TMED193: MurJ/WzxE-flippase ? ? ? ? ? ? 12TM VanZ-2
OUW80693.1 Gammaproteobacteria bacterium TMED219: MurJ/WzxE-flippase ? ? ? ? ? ? 12TM VanZ-2
RPF73192.1 Thiotrichales bacterium TMED285: MurJ/WzxE-flippase ? ? ? ? ? ? 12TM VanZ-2
NVO17290.1 Rhodoplanes sp.: WaaL-lipidA-ligase MurJ/WzxE-flippase ? LPS-like-sugar-transf ? LPS-like-sugar-transf VanZ-2
BCD96941.1 Marinagarivorans cellulosilyticus: VanZ-2 ? ? MurJ/WzxE-flippase
MBI81167.1 Gammaproteobacteria bacterium: MurJ/WzxE-flippase ? ? ? ? ? ? ? VanZ-2

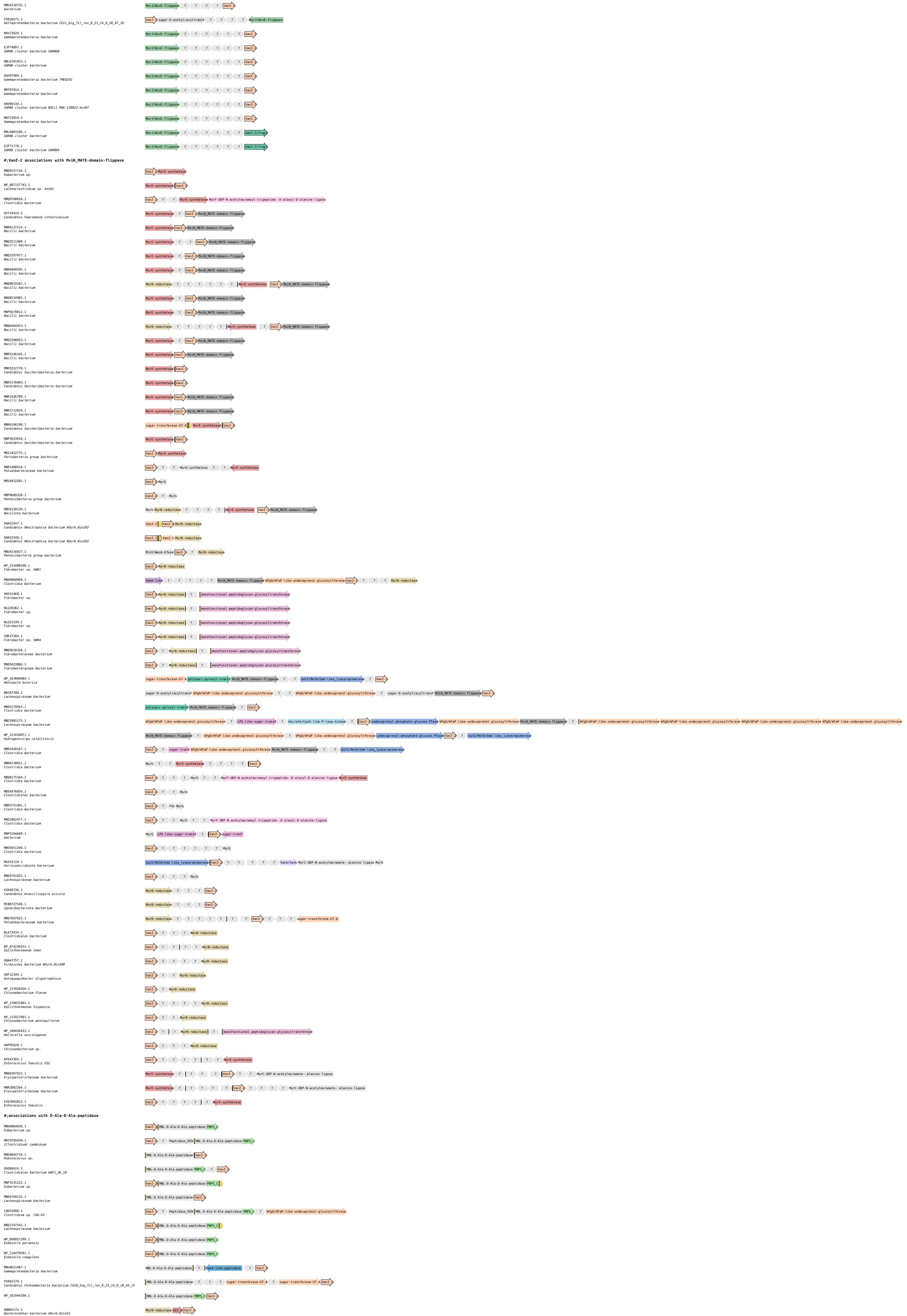
MBU4310735.1
bacterium
PIR20575.1
Deltaproteobacteria bacterium CG11_big_fil_rev_8_21_14_0_20_47_16
MAV23620.1
Gammaproteobacteria bacterium
EJP74097.1
SAR86 cluster bacterium SAR86B
MBL6701453.1
SAR86 cluster bacterium
OUX07989.1
Gammaproteobacteria bacterium TMED242
MBT87914.1
Gammaproteobacteria bacterium
KRO99134.1
SAR86 cluster bacterium BACL1 MAG-120823-bin87
MBT23659.1
Gammaproteobacteria bacterium
MBL6903106.1
SAR86 cluster bacterium
EJP71770.1
SAR86 cluster bacterium SAR86A
#;VanZ-2 associations with MviN_MATE-domain-flippase
MBQ9157116.1
Eubacterium sp.
WP_087157743.1
Lachnoclostridium sp. An181
MBQ9760016.1
Clostridia bacterium
HIT24410.1
Candidatus Faecimonas intestinavium
MBR6137214.1
Bacilli bacterium
MBQ3511409.1
Bacilli bacterium
MBQ3297977.1
Bacilli bacterium
MBR6949295.1
Bacilli bacterium
MBQ9019207.1
Bacilli bacterium
MBQ8534985.1
Bacilli bacterium
MBP5679012.1
Bacilli bacterium
MBQ6404453.1
Bacilli bacterium
MBQ3308052.1
Bacilli bacterium
MBR3146165.1
Bacilli bacterium
MBR3322778.1
Candidatus Saccharibacteria bacterium
MBR3176403.1
Candidatus Saccharibacteria bacterium
MBR1936799.1
Bacilli bacterium
MBR2712029.1
Bacilli bacterium
MBR6166190.1
Candidatus Saccharibacteria bacterium
MBP5633910.1
Candidatus Saccharibacteria bacterium
MBI2452775.1
Parcubacteria group bacterium
MBR1480524.1
Paludibacteraceae bacterium
MBS4932201.1
MBP8689320.1
Patescibacteria group bacterium
MBE6158139.1
Bacillota bacterium
OQA52547.1
Candidatus Omnitrophica bacterium ADurb.Bin292
OQA52548.1
Candidatus Omnitrophica bacterium ADurb.Bin292
MBU4216927.1
Patescibacteria group bacterium
WP_233488100.1
Fibrobacter sp. UWR1
MBQ9860009.1
Clostridia bacterium
HHX15468.1
Fibrobacter sp.
NLG39362.1
Fibrobacter sp.
NLO23339.1
Fibrobacter sp.
SHK27284.1
Fibrobacter sp. UWH4
MBQ5610356.1
Fibrobacteraceae bacterium
MBO5423866.1
Fibrobacteraceae bacterium
WP_163066084.1
Aminipila butyrica
NBI87708.1
Lachnospiraceae bacterium
MBO5179563.1
Clostridia bacterium
MBE5905175.1
Lachnospiraceae bacterium
WP_121010071.1
Hydrogenivirga caldilitoris
MBR2449167.1
Clostridia bacterium
MBR6720031.1
Clostridia bacterium
MBQ8175164.1
Clostridia bacterium
MBS6476056.1
Clostridiales bacterium
MBR3751401.1
Clostridia bacterium
MBQ1862477.1
Clostridia bacterium
MBP5204048.1
bacterium
MBO5051260.1
Clostridia bacterium
RKX33110.1
Verrucomicrobiota bacterium
MBO4761825.1
Lachnospiraceae bacterium
HIR49736.1
Candidatus Avoscillospira avicola
MCB0727546.1
Ignavibacteriota bacterium
MBQ7697622.1
Paludibacteraceae bacterium
NLA73434.1
Clostridiales bacterium
WP_074236552.1
Epilithonimonas zeae
OQA47757.1
Firmicutes bacterium ADurb.Bin300
UDF12349.1
Antiquaquibacter oligotrophicus
WP_115958356.1
Chryseobacterium flavum
WP_116031401.1
Epilithonimonas hispanica
WP_115927891.1
Chryseobacterium pennipullorum
WP_100426433.1
Hallerella succinigenes
HAP95628.1
Chryseobacterium sp.
AFO43365.1
Enterococcus faecalis D32
MBQ6367622.1
Erysipelotrichaceae bacterium
MBR2802264.1
Erysipelotrichaceae bacterium
EIQ7093912.1
Enterococcus faecalis
#;associations with D-Ala-D-Ala-peptidase
MBO4864036.1
Eubacterium sp.
MBT9785939.1
[Clostridium] symbiosum
MBE6844719.1
Ruminococcus sp.
OGO88424.1
Clostridiales bacterium GWF2_36_10
MBP3235122.1
Eubacterium sp.
MBR4769132.1
Lachnospiraceae bacterium
CDD55098.1
Clostridium sp. CAG:43
MBQ7247542.1
Lachnospiraceae bacterium
WP_099951299.1
Ezakiella peruensis
WP_116479591.1
Ezakiella coagulans
MBU0621487.1
Gammaproteobacteria bacterium
PIR92179.1
Candidatus Falkowbacteria bacterium CG10_big_fil_rev_8_21_14_0_10_44_15
WP_101944199.1
OQB65174.1
Bacteroidetes bacterium ADurb.Bin141

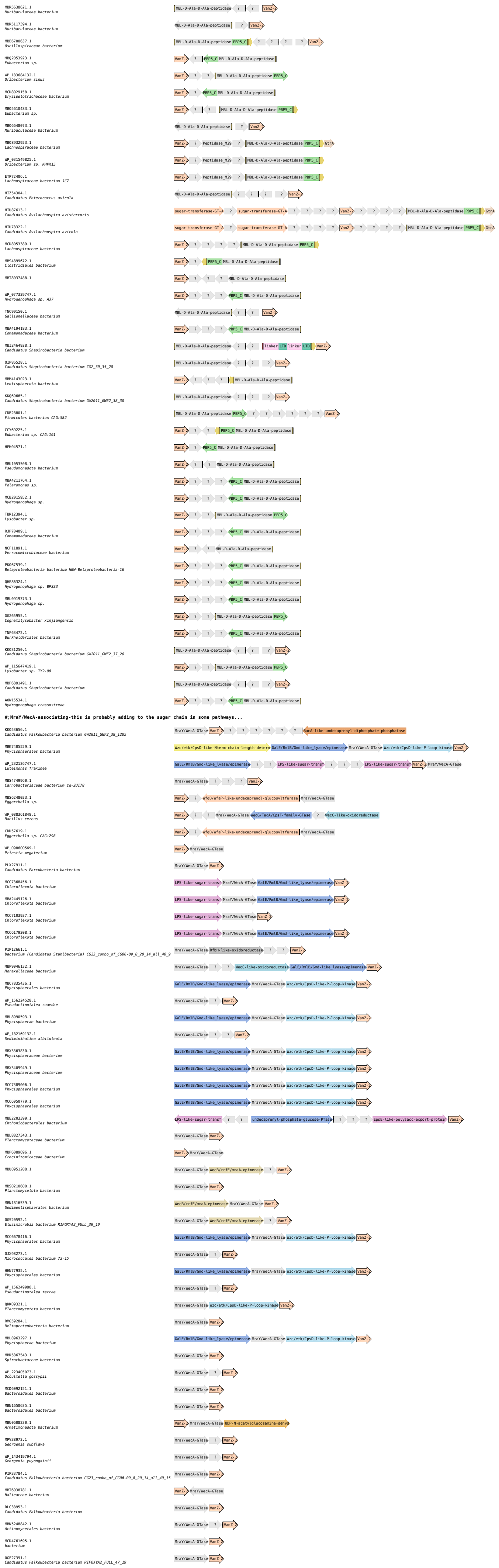

MBR5638621.1
Muribaculaceae bacterium
MBR5117394.1
Muribaculaceae bacterium
MBE6700637.1
Oscillospiraceae bacterium
MBQ2053923.1
Eubacterium sp.
WP_183684132.1
Oribacterium sinus
MCD8029158.1
Erysipelotrichaceae bacterium
MBO5610483.1
Eubacterium sp.
MBQ6648073.1
Muribaculaceae bacterium
MBQ8932923.1
Lachnospiraceae bacterium
WP_031549825.1
Oribacterium sp. KHPX15
ETP72406.1
Lachnospiraceae bacterium JC7
HIZ54304.1
Candidatus Enterococcus avicola
HIU87613.1
Candidatus Avilachnospira avistercoris
HIU78322.1
Candidatus Avilachnospira avicola
MCD8053389.1
Lachnospiraceae bacterium
MBS4899672.1
Clostridiales bacterium
MBT8037488.1
WP_077329747.1
Hydrogenophaga sp. A37
TNC99150.1
Gallionellaceae bacterium
MBA4194183.1
Comamonadaceae bacterium
MBI2464928.1
Candidatus Shapirobacteria bacterium
OIP86528.1
Candidatus Shapirobacteria bacterium CG2_30_35_20
MBM4143023.1
Lentisphaerota bacterium
KKQ69665.1
Candidatus Shapirobacteria bacterium GW2011_GWE2_38_30
CDB28801.1
Firmicutes bacterium CAG:582
CCY69225.1
Eubacterium sp. CAG:161
HFH04571.1
MBU1053508.1
Pseudomonadota bacterium
MBA4211764.1
Polaromonas sp.
MCB2015952.1
Hydrogenophaga sp.
TBR12394.1
Lysobacter sp.
RJP70409.1
Comamonadaceae bacterium
NCF11891.1
Verrucomicrobiaceae bacterium
PKO67539.1
Betaproteobacteria bacterium HGW-Betaproteobacteria-16
QHE86324.1
Hydrogenophaga sp. BPS33
MBL0919373.1
Hydrogenophaga sp.
GGZ65955.1
Cognatilysobacter xinjiangensis
TNF63472.1
Burkholderiales bacterium
KKQ31250.1
Candidatus Shapirobacteria bacterium GW2011_GWF2_37_20
WP_115647419.1
Lysobacter sp. TY2-98
MBP6891491.1
Candidatus Shapirobacteria bacterium
AOW15534.1
Hydrogenophaga crassostreae
#;MraY/WecA-associating-this is probably adding to the sugar chain in some pathways...
KKQ53656.1
Candidatus Falkowbacteria bacterium GW2011_GWF2_38_1205
MBK7405529.1
Phycisphaerales bacterium
WP_232136747.1
Luteimonas fraxinea
MBS4749960.1
Carnobacteriaceae bacterium zg-ZUI78
MBS6248023.1
Eggerthella sp.
WP_088361848.1
Bacillus cereus
CDD57619.1
Eggerthella sp. CAG:298
WP_098600569.1
Priestia megaterium
PLX27911.1
Candidatus Parcubacteria bacterium
MCC7368456.1
Chloroflexota bacterium
MBA2449126.1
Chloroflexota bacterium
MCC7103937.1
Chloroflexota bacterium
MCC6179208.1
Chloroflexota bacterium
PIP12661.1
bacterium (Candidatus Stahlbacteria) CG23_combo_of_CG06-09_8_20_14_all_40_9
MBP9046132.1
Moraxellaceae bacterium
MBC7835436.1
Phycisphaerales bacterium
WP_156224528.1
Pseudactinotalea suaedae
MBL8990593.1
Phycisphaerae bacterium
WP_182169132.1
Sediminihaliea albiluteola
MBX3363830.1
Phycisphaeraceae bacterium
MBX3409949.1
Phycisphaeraceae bacterium
MCC7389006.1
Phycisphaerales bacterium
MCC6950779.1
Phycisphaerales bacterium
MBE2203399.1
Chthoniobacterales bacterium
MBL8827343.1
Planctomycetaceae bacterium
MBP6089696.1
Crocinitomicaceae bacterium
MBU0951208.1
MBS0210600.1
Planctomycetota bacterium
MBN1816539.1
Sedimentisphaerales bacterium
OGS20592.1
Elusimicrobia bacterium RIFOXYA2_FULL_39_19
MCC6678416.1
Phycisphaerales bacterium
OJX98273.1
Micrococcales bacterium 73-15
HHN77935.1
Phycisphaerales bacterium
WP_156249988.1
Pseudactinotalea terrae
QKK09321.1
Planctomycetota bacterium
RMG59284.1
Deltaproteobacteria bacterium
MBL8963297.1
Phycisphaerae bacterium
MBR5867543.1
Spirochaetaceae bacterium
WP_223405073.1
Occultella gossypii
MCD6092151.1
Bacteroidales bacterium
MBN1650635.1
Bacteroidales bacterium
MBU0608230.1
Armatimonadota bacterium
MPV38972.1
Georgenia subflava
WP_143419794.1
Georgenia yuyongxinii
PIP33784.1
Candidatus Falkowbacteria bacterium CG23_combo_of_CG06-09_8_20_14_all_49_15
MBT6038781.1
Halieaceae bacterium
RLC38953.1
Candidatus Falkowbacteria bacterium
MBK5248842.1
Actinomycetales bacterium
MCD4761695.1
bacterium
OGF27391.1
Candidatus Falkowbacteria bacterium RIFOXYA2_FULL_47_19
VanZ-2
MBL-D-Ala-D-Ala-peptidase
PBP5_C
Peptidase_M29
GtrA
sugar-transferase-GT-A
linker
LTD
MraY/WecA-GTase
BacA-like-undecaprenyl-diphosphate-phosphatase
Wzc/etk/CpsD-like-Nterm-chain-length-determ
GalE/RmlB/Gmd-like_lyase/epimerase
Wzc/etk/CpsD-like-P-loop-kinase
LPS-like-sugar-transf
WfgD/WfaP-like-undecaprenol-glucosyltferase
WecG/TagA/CpsF-family-GTase
WecC-like-oxidoreductase
RfbH-like-oxidoreductase
undecaprenyl-phosphate-glucose-PTase
EpsE-like-polysacc-export-protein
WecB/rrfE/mnaA-epimerase
UDP-N-acetylglucosamine-dehyd

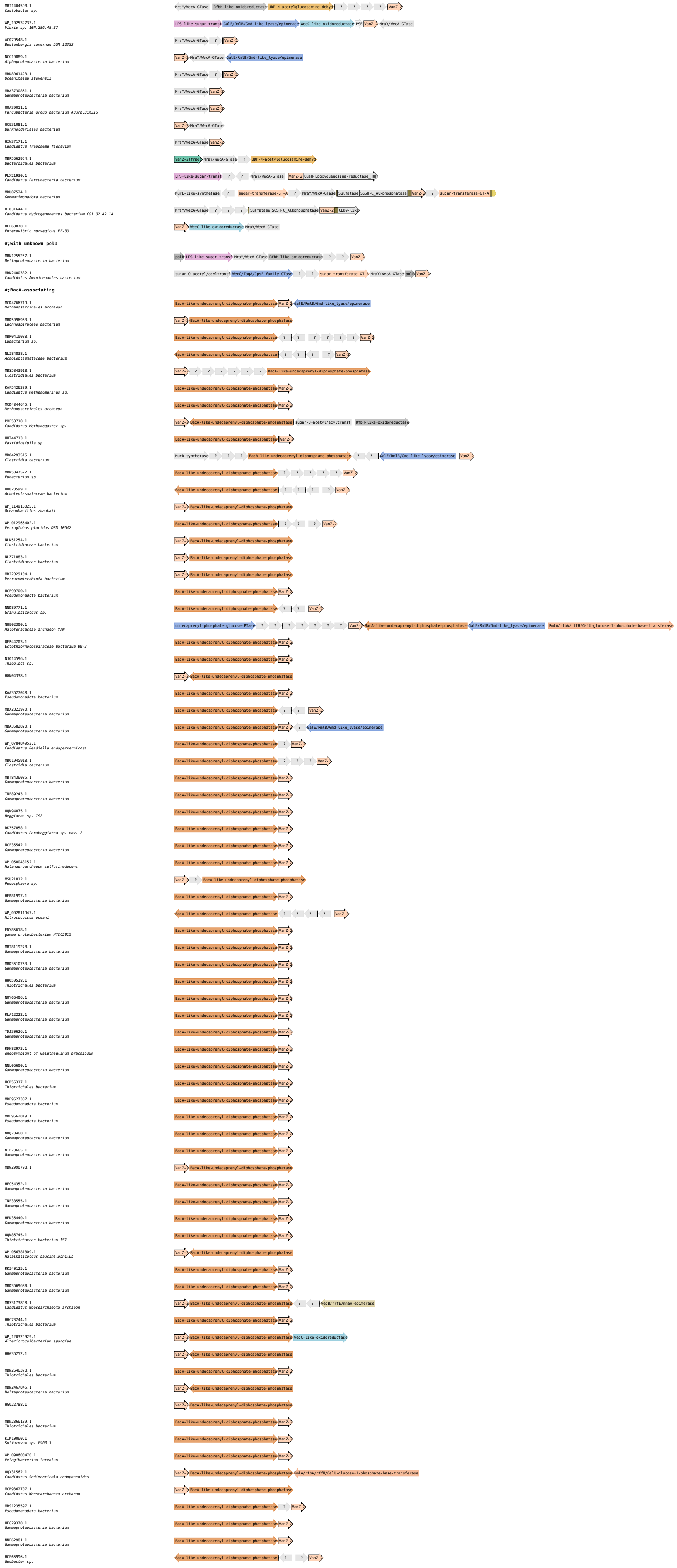
MBI1404598.1
Caulobacter sp.
WP_102532733.1
Vibrio sp. 10N.286.48.B7
ACQ79548.1
Beutenbergia cavernae DSM 12333
NCG10889.1
Alphaproteobacteria bacterium
MBD8061423.1
Oceanitalea stevensii
MBA3730861.1
Gammaproteobacteria bacterium
OQA39011.1
Parcubacteria group bacterium ADurb.Bin316
UCE31081.1
Burkholderiales bacterium
HIW37171.1
Candidatus Treponema faecavium
MBP5662954.1
Bacteroidales bacterium
PLX21930.1
Candidatus Parcubacteria bacterium
MBU07524.1
Gemmatimonadota bacterium
OIO31644.1
Candidatus Hydrogenedentes bacterium CG1_02_42_14
OEE68070.1
Enterovibrio norvegicus FF-33
#;with unknown polB
MBN1255257.1
Deltaproteobacteria bacterium
MBN2400382.1
Candidatus Aminicenantes bacterium
#;BacA-associating
MCD4766719.1
Methanosarcinales archaeon
MBD5096963.1
Lachnospiraceae bacterium
MBR0410088.1
Eubacterium sp.
NLZ84838.1
Acholeplasmataceae bacterium
MBS5843918.1
Clostridiales bacterium
KAF5426389.1
Candidatus Methanomarinus sp.
MCD4844645.1
Methanosarcinales archaeon
PXF58718.1
Candidatus Methanogaster sp.
HHT44713.1
Fastidiosipila sp.
MBO4293515.1
Clostridia bacterium
MBR5047572.1
Eubacterium sp.
HHU23599.1
Acholeplasmataceae bacterium
WP_114916025.1
Oceanobacillus zhaokaii
WP_012966402.1
Ferroglobus placidus DSM 10642
NLN51254.1
Clostridiaceae bacterium
NLZ71883.1
Clostridiaceae bacterium
MBI2929104.1
Verrucomicrobiota bacterium
UCE90700.1
Pseudomonadota bacterium
NND89771.1
Granulosicoccus sp.
NUE02300.1
Haloferacaceae archaeon YAN
QEP44203.1
Ectothiorhodospiraceae bacterium BW-2
NJO14596.1
Thioploca sp.
HGN04338.1
KAA3627048.1
Pseudomonadota bacterium
MBX2823970.1
Gammaproteobacteria bacterium
MBA3582820.1
Gammaproteobacteria bacterium
WP_078484952.1
Candidatus Reidiella endopervernicosa
MBQ1945918.1
Clostridia bacterium
MBT8436085.1
Gammaproteobacteria bacterium
TNF89243.1
Gammaproteobacteria bacterium
OQW94075.1
Beggiatoa sp. IS2
RKZ57858.1
Candidatus Parabeggiatoa sp. nov. 2
NCF35542.1
Gammaproteobacteria bacterium
WP_050048152.1
Halanaeroarchaeum sulfurireducens
MSU21812.1
Pedosphaera sp.
HEB81997.1
Gammaproteobacteria bacterium
WP_002811947.1
Nitrosococcus oceani
EDY85618.1
gamma proteobacterium HTCC5015
MBT8119278.1
Gammaproteobacteria bacterium
MBD3610763.1
Gammaproteobacteria bacterium
HHO59518.1
Thiotrichales bacterium
NOY66406.1
Gammaproteobacteria bacterium
RLA12222.1
Gammaproteobacteria bacterium
TDJ30626.1
Gammaproteobacteria bacterium
RDH82973.1
endosymbiont of Galathealinum brachiosum
NNL06600.1
Gammaproteobacteria bacterium
UCB55317.1
Thiotrichales bacterium
MBE9527307.1
Pseudomonadota bacterium
MBE9562019.1
Pseudomonadota bacterium
NOQ78468.1
Gammaproteobacteria bacterium
NIP73665.1
Gammaproteobacteria bacterium
MBW2990798.1
HFC54352.1
Gammaproteobacteria bacterium
TNF38555.1
Gammaproteobacteria bacterium
HED36440.1
Gammaproteobacteria bacterium
OQW86745.1
Thiotrichaceae bacterium IS1
WP_066381809.1
Halalkalicoccus paucihalophilus
RKZ40125.1
Gammaproteobacteria bacterium
MBD3669680.1
Gammaproteobacteria bacterium
MBS3173858.1
Candidatus Woesearchaeota archaeon
HHC73244.1
Thiotrichales bacterium
WP_120325929.1
Altericroceibacterium spongiae
HHG36252.1
MBN2646378.1
Thiotrichales bacterium
MBN2467845.1
Deltaproteobacteria bacterium
HGU22788.1
MBN2866189.1
Thiotrichales bacterium
KIM10060.1
Sulfurovum sp. FS08-3
WP_090600470.1
Pelagibacterium luteolum
OQX31562.1
Candidatus Sedimenticola endophacoides
MCB9362707.1
Candidatus Woesearchaeota archaeon
MBS1235597.1
Pseudomonadota bacterium
HEC29370.1
Gammaproteobacteria bacterium
NNE62981.1
Gammaproteobacteria bacterium
HCE66996.1
Geobacter sp.

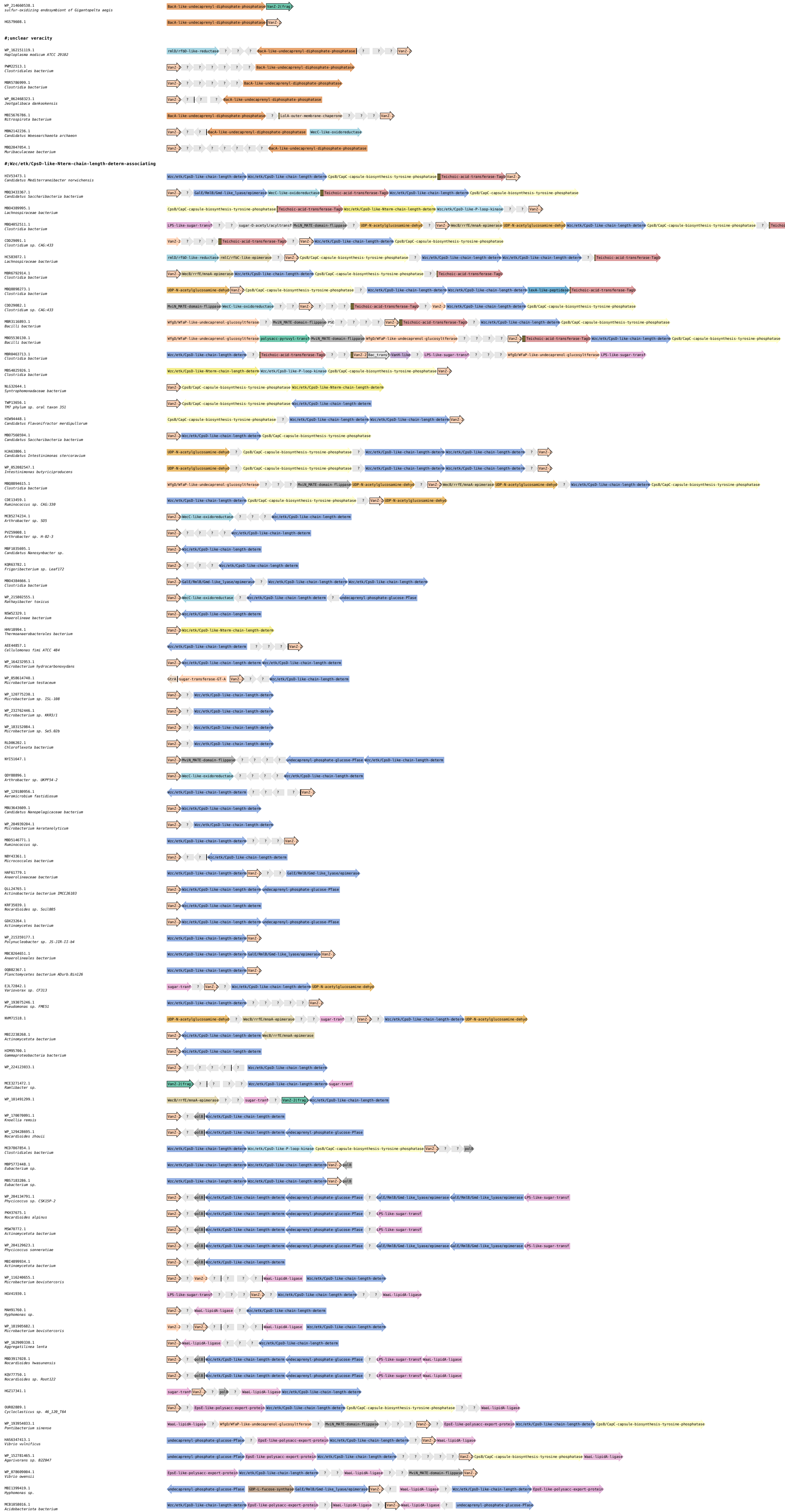
WP_214660538.1
sulfur-oxidizing endosymbiont of Gigantopelta aegis
HGS79608.1
#;unclear veracity
WP_162151119.1
Haploplasma modicum ATCC 29102
PWM22513.1
Clostridiales bacterium
MBR5786999.1
Clostridia bacterium
WP_062468323.1
Jeotgalibaca dankookensis
MBI5676786.1
Nitrospirota bacterium
MBN2142236.1
Candidatus Woesearchaeota archaeon
MBQ2047054.1
Muribaculaceae bacterium
#;Wzc/etk/CpsD-like-Nterm-chain-length-determ-associating
HIV53473.1
Candidatus Mediterraneibacter norwichensis
MBQ3433367.1
Candidatus Saccharibacteria bacterium
MBO4389995.1
Lachnospiraceae bacterium
MBQ4052511.1
Clostridia bacterium
CDD29091.1
Clostridium sp. CAG:433
HCS83072.1
Lachnospiraceae bacterium
MBR6792914.1
Clostridia bacterium
MBQ8898273.1
Clostridia bacterium
CDD29082.1
Clostridium sp. CAG:433
MBR3116893.1
Bacilli bacterium
MBO5530130.1
Bacilli bacterium
MBR0463713.1
Clostridia bacterium
MBS4025926.1
Clostridia bacterium
NLG32644.1
Syntrophomonadaceae bacterium
TWP13656.1
TM7 phylum sp. oral taxon 351
HIW94448.1
Candidatus Flavonifractor merdipullorum
MBO7560594.1
Candidatus Saccharibacteria bacterium
HJA63806.1
Candidatus Intestinimonas stercoravium
WP_052082547.1
Intestinimonas butyriciproducens
MBQ8894615.1
Clostridia bacterium
CDE13459.1
Ruminococcus sp. CAG:330
MCB5274234.1
Arthrobacter sp. S05
PVZ59008.1
Arthrobacter sp. H-02-3
MBF1035695.1
Candidatus Nanosynbacter sp.
KQR63782.1
Frigoribacterium sp. Leaf172
MBO4384666.1
Clostridia bacterium
WP_215802555.1
Rathayibacter toxicus
NSW52329.1
Anaerolineae bacterium
HHV18994.1
Thermoanaerobacterales bacterium
AEE44857.1
Cellulomonas fimi ATCC 484
WP_164232953.1
Microbacterium hydrocarbonoxydans
WP_058614740.1
Microbacterium testaceum
WP_120775230.1
Microbacterium sp. ISL-108
WP_232762446.1
Microbacterium sp. KKR3/1
WP_183152084.1
Microbacterium sp. Se5.02b
RLD06202.1
Chloroflexota bacterium
NYI51647.1
QDY88896.1
Arthrobacter sp. UKPF54-2
WP_129180956.1
Aeromicrobium fastidiosum
MBU3643609.1
Candidatus Nanopelagicaceae bacterium
WP_204939204.1
Microbacterium keratanolyticum
MBD5146771.1
Ruminococcus sp.
NBY43361.1
Micrococcales bacterium
HAF61779.1
Anaerolineaceae bacterium
QLL24765.1
Actinobacteria bacterium IMCC26103
KRF35039.1
Nocardioides sp. Soil805
GDX23264.1
Actinomycetes bacterium
WP_215359177.1
Polynucleobacter sp. JS-JIR-II-b4
MBC8264651.1
Anaerolineales bacterium
OQB82367.1
Planctomycetes bacterium ADurb.Bin126
EJL72842.1
Variovorax sp. CF313
WP_193075246.1
Pseudomonas sp. FME51
NVM71518.1
MBI2238268.1
Actinomycetota bacterium
HIM95700.1
Gammaproteobacteria bacterium
WP_224123033.1
MCE3271472.1
Ramlibacter sp.
WP_101491299.1
WP_170070091.1
Knoellia remsis
WP_129428695.1
Nocardioides zhouii
MCD7867854.1
Clostridiales bacterium
MBP5772448.1
Eubacterium sp.
MBS7183286.1
Eubacterium sp.
WP_204134791.1
Phycicoccus sp. CSK15P-2
PKH37675.1
Nocardioides alpinus
MSW70772.1
Actinomycetota bacterium
WP_204129623.1
Phycicoccus sonneratiae
MBI4899934.1
Actinomycetota bacterium
WP_116240655.1
Microbacterium bovistercoris
HGV41930.1
MAH91760.1
Hyphomonas sp.
WP_181905682.1
Microbacterium bovistercoris
WP_162909330.1
Aggregatilinea lenta
MBD3917028.1
Nocardioides hwasunensis
KQV77750.1
Nocardioides sp. Root122
HGZ17341.1
OUR82889.1
Cycloclasticus sp. 46_120_T64
WP_193954033.1
Pontibacterium sinense
HAS6347413.1
Vibrio vulnificus
WP_152781465.1
Agarivorans sp. B2Z047
WP_078609004.1
Vibrio owensii
MBI1399419.1
Hyphomonas sp.
MCB1058016.1
Acidobacteriota bacterium

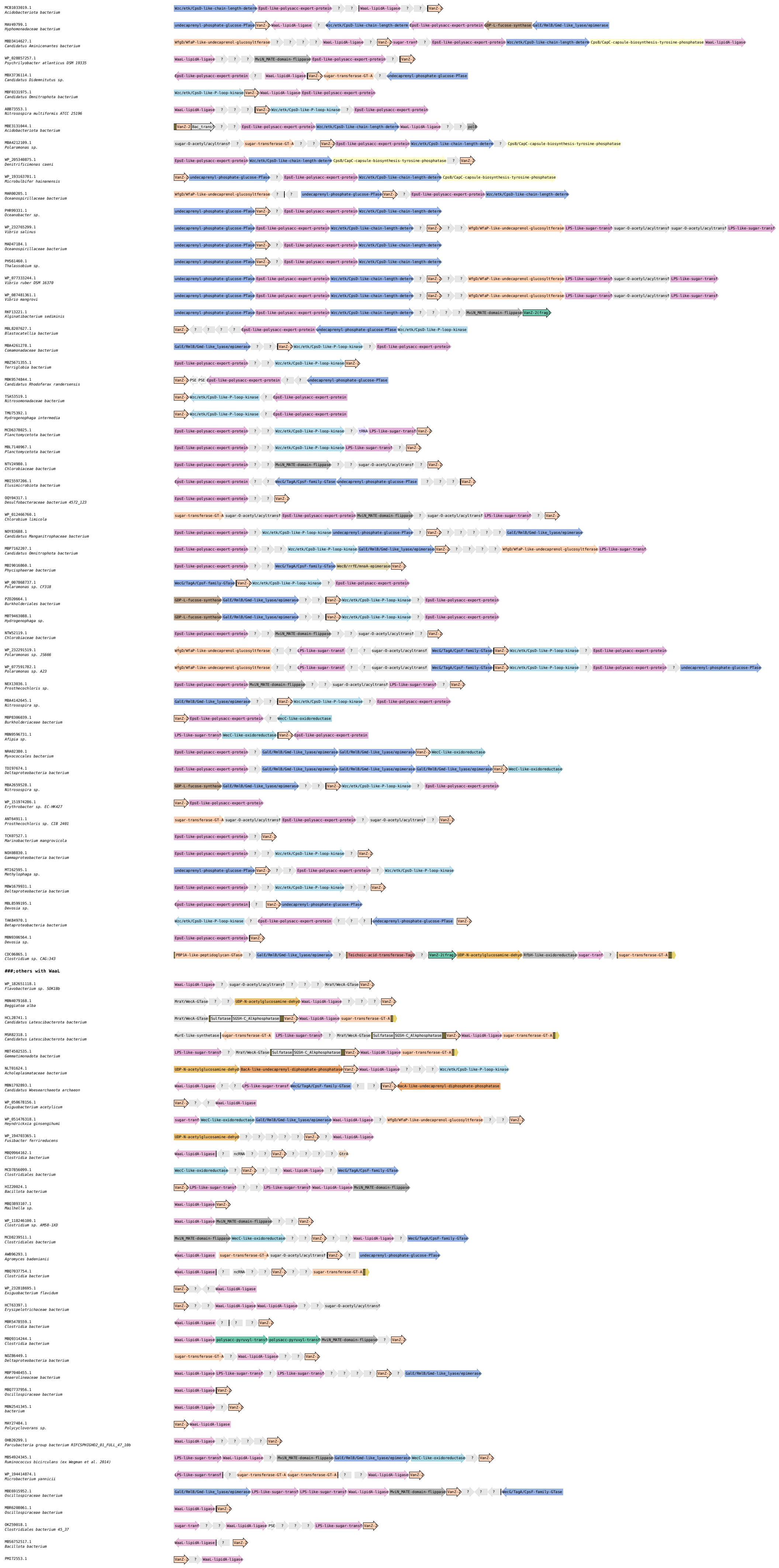

MCB1833019.1
Acidobacteriota bacterium
MAV49799.1
Hyphomonadaceae bacterium
MBD3414627.1
Candidatus Aminicenantes bacterium
WP_028857257.1
Psychrilyobacter atlanticus DSM 19335
MBX3736114.1
Candidatus Didemnitutus sp.
MBF0331975.1
Candidatus Omnitrophota bacterium
ABB73553.1
Nitrosospira multiformis ATCC 25196
MBE3131044.1
Acidobacteriota bacterium
MBA4212109.1
Polaromonas sp.
WP_205340875.1
Denitrificimonas caeni
WP_193163701.1
Microbulbifer hainanensis
MAR00205.1
Oceanospirillaceae bacterium
PHR99331.1
Oceanobacter sp.
WP_232765299.1
Vibrio salinus
MAD47184.1
Oceanospirillaceae bacterium
PHS61460.1
Thalassobium sp.
WP_077333244.1
Vibrio ruber DSM 16370
WP_087481361.1
Vibrio mangrovi
RKF13221.1
Alginatibacterium sediminis
MBL8207627.1
Blastocatellia bacterium
MBA4261278.1
Comamonadaceae bacterium
MBZ5671355.1
Terriglobia bacterium
MBK9574844.1
Candidatus Rhodoferax randersensis
TSA53519.1
Nitrosomonadaceae bacterium
TMJ75392.1
Hydrogenophaga intermedia
MCD6378025.1
Planctomycetota bacterium
MBL7140967.1
Planctomycetota bacterium
NTV24980.1
Chlorobiaceae bacterium
MBI5597206.1
Elusimicrobiota bacterium
OQY04317.1
Desulfobacteraceae bacterium 4572_123
WP_012466760.1
Chlorobium limicola
NOY83688.1
Candidatus Manganitrophaceae bacterium
MBP7162207.1
Candidatus Omnitrophota bacterium
MBI9016860.1
Phycisphaerae bacterium
WP_007868737.1
Polaromonas sp. CF318
PZO20664.1
Burkholderiales bacterium
MBT9463088.1
Hydrogenophaga sp.
NTW52119.1
Chlorobiaceae bacterium
WP_232291519.1
Polaromonas sp. JS666
WP_077591782.1
Polaromonas sp. A23
NEX13036.1
Prosthecochloris sp.
MBA4142645.1
Nitrosospira sp.
MBP8300039.1
Burkholderiaceae bacterium
MBN9596731.1
Afipia sp.
NRA02380.1
Myxococcales bacterium
TDI97674.1
Deltaproteobacteria bacterium
MBA2659528.1
Nitrosospira sp.
WP_151974286.1
Erythrobacter sp. EC-HK427
ANT64911.1
Prosthecochloris sp. CIB 2401
TCK07527.1
Marinobacterium mangrovicola
NOX08830.1
Gammaproteobacteria bacterium
MTI62595.1
Methylophaga sp.
MBW1679931.1
Deltaproteobacteria bacterium
MBL8599195.1
Devosia sp.
TAK84970.1
Betaproteobacteria bacterium
MBN9306564.1
Devosia sp.
CDC06865.1
Clostridium sp. CAG:343
###;others with WaaL
WP_182651118.1
Flavobacterium sp. SDK18b
MBN4079168.1
Beggiatoa alba
HCL28741.1
Candidatus Latescibacterota bacterium
MSR82318.1
Candidatus Latescibacterota bacterium
MBT4502535.1
Gemmatimonadota bacterium
NLT01624.1
Acholeplasmataceae bacterium
MBN1792893.1
Candidatus Woesearchaeota archaeon
WP_059678156.1
Exiguobacterium acetylicum
WP_051476318.1
Heyndrickxia ginsengihumi
WP_194703365.1
Fusibacter ferrireducens
MBQ9964162.1
Clostridia bacterium
MCD7856099.1
Clostridiales bacterium
HIZ20924.1
Bacillota bacterium
MBQ3893107.1
Mailhella sp.
WP_118246100.1
Clostridium sp. AM58-1XD
MCD8239511.1
Clostridiales bacterium
AWB96293.1
Agromyces badenianii
MBQ7037754.1
Clostridia bacterium
WP_232818695.1
Exiguobacterium flavidum
HCT63397.1
Erysipelotrichaceae bacterium
MBR5478559.1
Clostridia bacterium
MBQ9314244.1
Clostridia bacterium
NOZ86449.1
Deltaproteobacteria bacterium
MBP7040455.1
Anaerolineaceae bacterium
MBQ7737956.1
Oscillospiraceae bacterium
MBN2541345.1
bacterium
MAY27484.1
Polycyclovorans sp.
OHB20299.1
Parcubacteria group bacterium RIFCSPHIGHO2_01_FULL_47_10b
MBS4924345.1
Ruminococcus bicirculans (ex Wegman et al. 2014)
WP_194414874.1
Microbacterium yannicii
MBE6915952.1
Oscillospiraceae bacterium
MBR6208061.1
Oscillospiraceae bacterium
OKZ59018.1
Clostridiales bacterium 45_37
MBS6752517.1
Bacillota bacterium
PMI72553.1

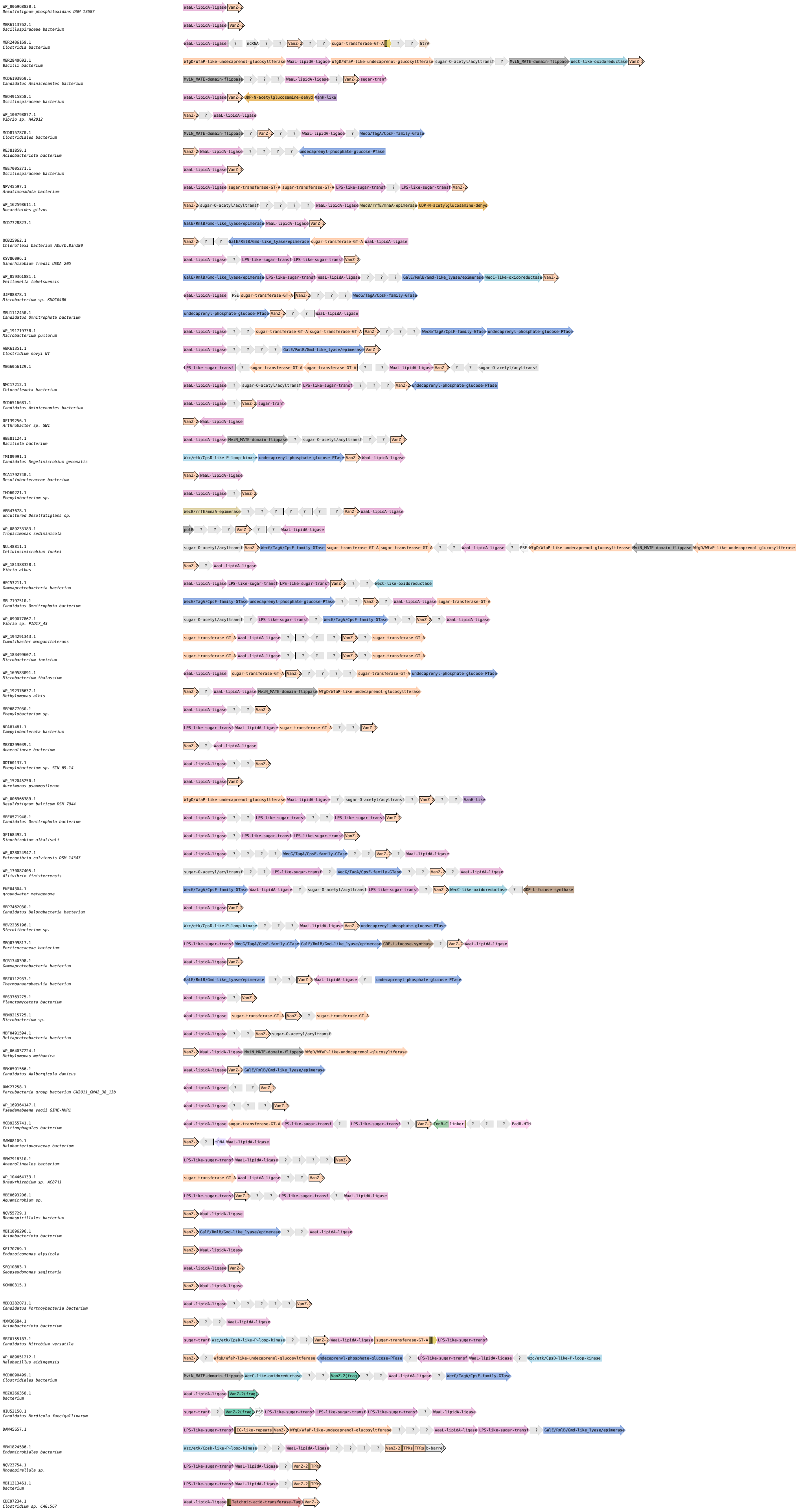

#;GNAT-associating sugar assembly pathway (GNAT here may be like the WecE-like GNAT). Direct fusions:

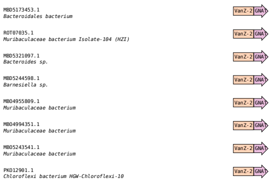

#;operonic associations

MBI2469461.1
Candidatus Rokuibacteriota bacterium

MBE6956793.1
Oscillospiraceae bacterium

MBR4865137.1
Clostridia bacterium

RSN69146.1
Actinomadura sp. WAC 06369

RAP77995.1
Paenibacillus montanisoli

WP_116479730.1
Ezakiella coagulans

HJE14714.1
Lapidilactobacillus dextrinicus

MBC1559294.1
Listeria booriae

MBC1562415.1
Listeria booriae

MBC1649367.1
Listeria booriae

APT19000.1
Amylolactobacillus amylophilus DSM 20533 = JCM 1125

MBQ3550123.1
Clostridia bacterium

MBR6994753.1
Ruminococcus sp.

WP_011522063.1
Candidatus Koribacter versatilis Ellin345

CCY05071.1
Eggerthella sp. CAG:1427

MBO7475253.1
Ruminococcus sp.

MBO5745542.1
Clostridia bacterium

WP_185347583.1
Listeria booriae

WP_185364015.1
Listeria booriae

WP_185406191.1
Listeria booriae

WP_185377051.1
Listeria booriae

WP_185362169.1
Listeria booriae

WP_185358144.1
Listeria booriae

WP_181603340.1
Clostridium intestinale

HJF19843.1
Enterococcus columbae

WP_016183985.1
Enterococcus columbae DSM 7374 = ATCC 51263

MBQ7715354.1
Clostridia bacterium

NLH64334.1
Erysipelotrichaceae bacterium

MBE7490399.1
Planctomycetota bacterium

KAA0547222.1
Bacillus sp. BGMRC 2118

WP_013488744.1

WP_195573008.1
Paenibacillus sp. 1001270B_150601_E10

HGY98100.1

MBQ7298273.1
Clostridia bacterium

MBR3840985.1
Erysipelotrichales bacterium

MBF1724677.1
Streptococcus sp.

MBQ2424996.1
Lachnospiraceae bacterium

WP_142640760.1
Psychrobacillus vulpis

MBQ8928291.1
Oscillospiraceae bacterium

MBQ7139743.1
Clostridia bacterium

OLT28817.1
Actinomadura sp. CNU-125

SFM90038.1
Psychrobacillus psychrodurans

MBE6659468.1
Oscillospiraceae bacterium

WP_169078189.1
Microcella alkalica

WP_068450421.1
Kurthia gibsonii

MBS4967735.1
Lachnospiraceae bacterium

MBS5729637.1
Clostridium sp.

WP_214627233.1
Paenibacillus agaridevorans

NUM47740.1
Anaerolineales bacterium

MBQ0139164.1
Candidatus Kurthia equi

WP_079560683.1
Lysinibacillus sp. AC-3

CDB34242.1
Eggerthella sp. CAG:209

WP_153834551.1
Gracilibacillus thailandensis

QTE71732.1
Clostridiales bacterium FE2011

NMP37531.1
Clostridiales bacterium

MBR5960870.1
Clostridia bacterium

WP_224845150.1

MRN52416.1
Paenibacillus monticola

WP_223068135.1
Paenibacillus caui

MBQ4043010.1
Clostridia bacterium

WP_116049542.1
Paenibacillus paeoniae

WP_087569964.1
Paenibacillus sp. MY03

WP_171655928.1
Paenibacillus foliorum

EHS58689.1
Paenibacillus sp. Aloe-11

MBR4030537.1
Clostridia bacterium

MBQ8813934.1
Lachnospiraceae bacterium

WP_078380781.1
Sutcliffiella halmapala

MBD2868301.1
Paenibacillus arenilitoris

MBO4676023.1
Oscillospiraceae bacterium

MBK9973446.1
Planctomycetota bacterium

WP_089819310.1
Halomicrobium zhouii

HCY78987.1
Clostridiales bacterium

OMF37214.1
Paenibacillus sp. FSL H8-0548

WP_076266289.1
Paenibacillus sp. FSL A5-0031

MBU6319986.1
Alphaproteobacteria bacterium

MBP7229687.1
Moraxellaceae bacterium

MBI9011309.1
Clostridiales bacterium

MBQ3483980.1
Clostridia bacterium

MBN2500350.1
Anaerolineales bacterium

MBN2000723.1
Candidatus Zhuqueibacterota bacterium

MBN8730183.1
Acidobacteriota bacterium

WP_051865692.1
Metabacillus indicus LMG 22858

TAE25507.1
Candidatus Kapaibacterium sp.

HAT56087.1
Veillonellaceae bacterium

WP_091071181.1
Paenibacillus sp. NFR01

EDS73215.1
Anaerofustis stercorihominis DSM 17244

CAH1215248.1
Paenibacillus plantiphilus

WP_029284961.1
Metabacillus indicus LMG 22858

WP_029567052.1
Metabacillus indicus

MBW4083389.1
Paenibacillus sp. S150

MBE6561169.1
Oscillospiraceae bacterium

WP_183599584.1
Paenibacillus phyllosphaerae

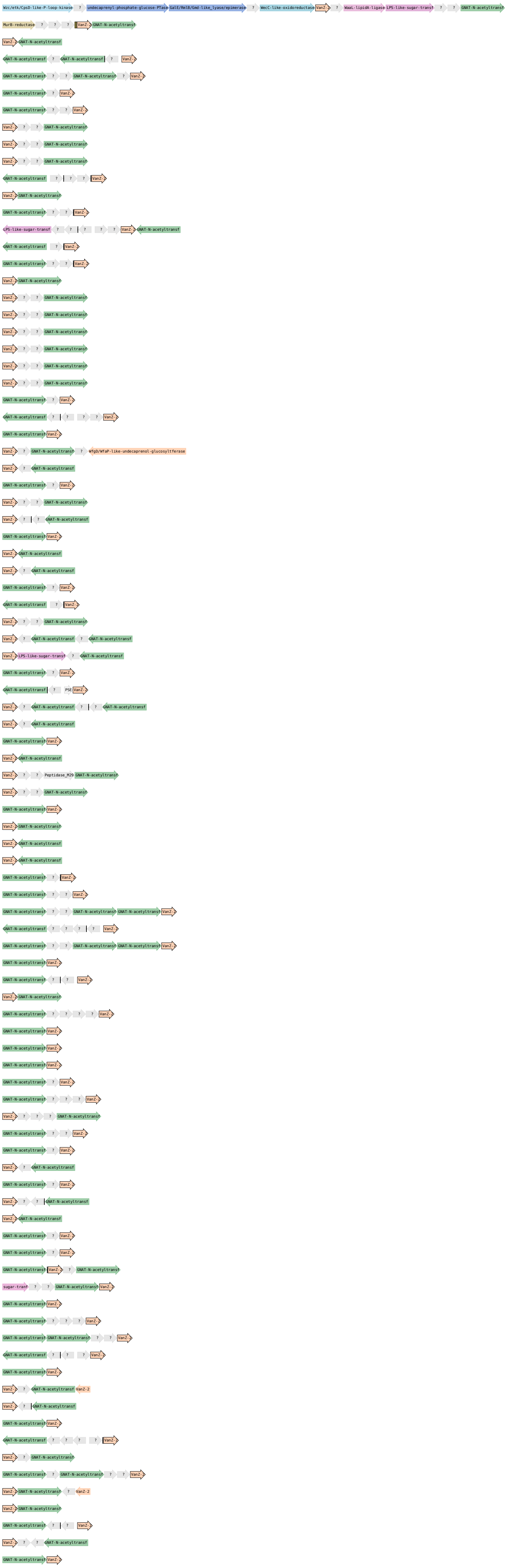

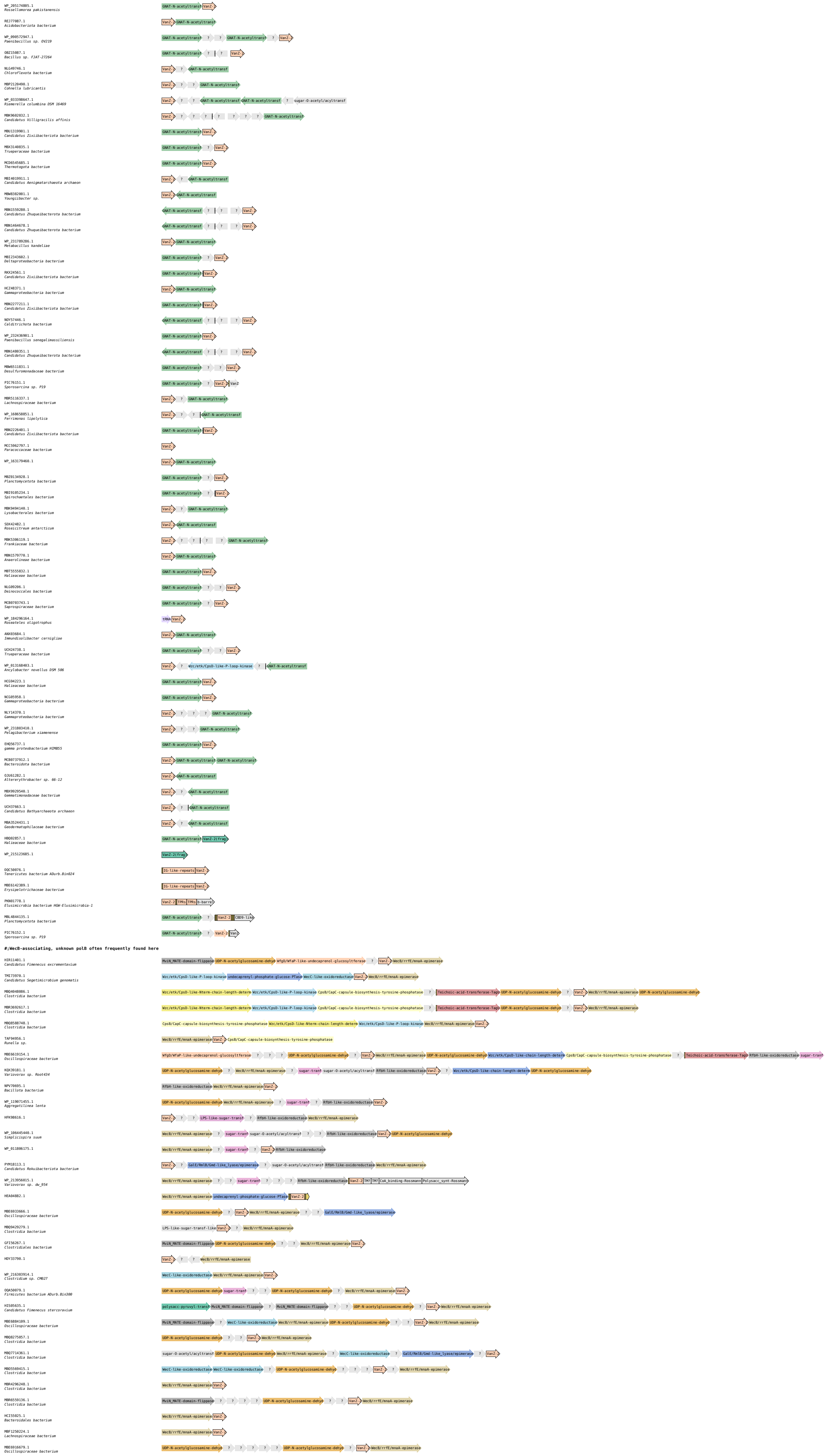
WP_205174805.1
Rossellomorea pakistanensis
REJ77087.1
Acidobacteriota bacterium
WP_090572947.1
Paenibacillus sp. OV219
OBZ15087.1
Bacillus sp. FJAT-27264
NLG49746.1
Chloroflexota bacterium
MBP2120490.1
Cohnella lubricantis
WP_033398647.1
Riemerella columbina DSM 16469
MBK9602032.1
Candidatus Villigracilis affinis
MBU1319901.1
Candidatus Zixiibacteriota bacterium
MBX3140835.1
Trueperaceae bacterium
MCD6545685.1
Thermotogota bacterium
MBI4019911.1
Candidatus Aenigmatarchaeota archaeon
MBW8382001.1
Youngiibacter sp.
MBN1559280.1
Candidatus Zhuqueibacterota bacterium
MBN1464678.1
Candidatus Zhuqueibacterota bacterium
WP_231789286.1
Metabacillus kandeliae
MBI2343602.1
Deltaproteobacteria bacterium
RKX24561.1
Candidatus Zixiibacteriota bacterium
HCZ48371.1
Gammaproteobacteria bacterium
MBN2277211.1
Candidatus Zixiibacteriota bacterium
NOY57446.1
Calditrichota bacterium
WP_232436901.1
Paenibacillus senegalimassiliensis
MBN1480351.1
Candidatus Zhuqueibacterota bacterium
MBW6511831.1
Desulfuromonadaceae bacterium
PIC76151.1
Sporosarcina sp. P19
MBR5116337.1
Lachnospiraceae bacterium
WP_168658851.1
Ferrimonas lipolytica
MBN2226401.1
Candidatus Zixiibacteriota bacterium
MCC5962797.1
Paracoccaceae bacterium
WP_163179460.1
MBZ0134928.1
Planctomycetota bacterium
MBI9105234.1
Spirochaetales bacterium
MBK9494140.1
Lysobacterales bacterium
SDX42482.1
Roseicitreum antarcticum
MBK5306119.1
Frankiaceae bacterium
MBN1579770.1
Anaerolineae bacterium
MBT5555832.1
Halieaceae bacterium
NLG09206.1
Deinococcales bacterium
MCB0703743.1
Saprospiraceae bacterium
WP_184296164.1
Roseateles oligotrophus
ANX03684.1
Immundisolibacter cernigliae
UCH24738.1
Trueperaceae bacterium
WP_013168403.1
Ancylobacter novellus DSM 506
HCG94223.1
Halieaceae bacterium
NCG05958.1
Gammaproteobacteria bacterium
NLY14370.1
Gammaproteobacteria bacterium
WP_231803410.1
Pelagibacterium xiamenense
EHQ56737.1
gamma proteobacterium HIMB55
MCB0737912.1
Bacteroidota bacterium
OJU61282.1
Altererythrobacter sp. 66-12
MBX9929540.1
Gemmatimonadaceae bacterium
UCH37663.1
Candidatus Bathyarchaeota archaeon
MBA3524431.1
Geodermatophilaceae bacterium
HBQ02857.1
Halieaceae bacterium
WP_215123685.1
OQC50076.1
Tenericutes bacterium ADurb.Bin024
MBE6142389.1
Erysipelotrichaceae bacterium
PKN01778.1
Elusimicrobia bacterium HGW-Elusimicrobia-1
MBL4844135.1
Planctomycetota bacterium
PIC76152.1
Sporosarcina sp. P19
#;WecB-associating, unknown polB often frequently found here
HIR11401.1
Candidatus Fimenecus excrementavium
TMI73970.1
Candidatus Segetimicrobium genomatis
MBQ4048086.1
Clostridia bacterium
MBR3692617.1
Clostridia bacterium
MBQ8588748.1
Clostridia bacterium
TAF94956.1
Runella sp.
MBE6619154.1
Oscillospiraceae bacterium
KQX39181.1
Variovorax sp. Root434
NPV70695.1
Bacillota bacterium
WP_119071455.1
Aggregatilinea lenta
HFK98616.1
WP_106445440.1
Simplicispira suum
WP_011806175.1
PYM18113.1
Candidatus Rokuibacteriota bacterium
WP_213956015.1
Variovorax sp. dw_954
HEA04882.1
MBE6933666.1
Oscillospiraceae bacterium
MBQ9429279.1
Clostridia bacterium
GFI56267.1
Clostridiales bacterium
HDY33790.1
WP_216303914.1
Clostridium sp. CM027
OQA50079.1
Firmicutes bacterium ADurb.Bin300
HIS05635.1
Candidatus Fimenecus stercoravium
MBE6884109.1
Oscillospiraceae bacterium
MBQ8275057.1
Clostridia bacterium
MBQ7714361.1
Clostridia bacterium
MBO5569415.1
Clostridia bacterium
MBR4296248.1
Clostridia bacterium
MBR6559136.1
Clostridia bacterium
HCI55025.1
Bacteroidales bacterium
MBF1250224.1
Lachnospiraceae bacterium
MBE6916679.1
Oscillospiraceae bacterium

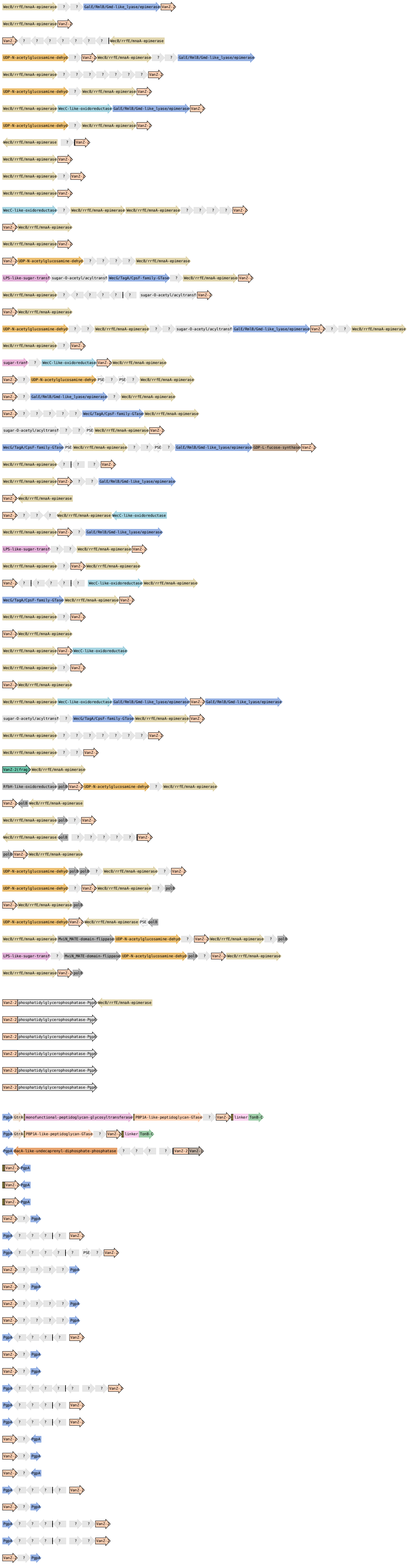

WP_026816608.1 *Arenimonas composti TR7-09 = DSM 18010*

MBR3865624.1 *Clostridia bacterium*

MBF4606072.1 *Curtobacterium sp. VKM Ac-1393*

MBE6047182.1 *Oscillospiraceae bacterium*

WP_119683162.1

MBC8560083.1 *Fumia xinanensis*

NLZ52048.1 *Thermoanaerobacteraceae bacterium*

HHU06685.1 *Clostridiales bacterium*

MBP5245504.1 *Clostridia bacterium*

MBO7275901.1 *Clostridia bacterium*

MBE6082652.1 *Oscillospiraceae bacterium*

WP_132014041.1 *Hydrogenispora ethanolica*

MBM3405145.1 *Bacteroidota bacterium*

MBQ9067226.1 *Oscillospiraceae bacterium*

MBO7625409.1 *Clostridia bacterium*

WP_128219467.1 *Nocardioides yefusunii*

MBN1126150.1 *Sedimentisphaerales bacterium*

MBQ7718835.1 *Clostridia bacterium*

NQU26395.1 *Candidatus Nealsonbacteria bacterium*

MBM4403008.1 *Candidatus Cloacimonadota bacterium*

OGS26672.1 *Elusimicrobia bacterium RIFOXYB2_FULL_48_7*

TLY42739.1 *Nitrospirota bacterium*

WP_185253535.1 *Nocardioides luti*

HHZ15869.1 *Candidatus Cloacimonadota bacterium*

MBP3258908.1 *Bacilli bacterium*

MBQ9469450.1 *Bacteroidales bacterium*

MBO8433179.1 *Candidatus Pullibacteroides excrementavium*

RZB29714.1 *Desulfobacteraceae bacterium Eth-SRB1*

MCB5284000.1 *Candidatus Cloacimonadota bacterium*

TVR69255.1 *Marinilabiliales bacterium*

MBN2413602.1 *Candidatus Zhuqueibacterota bacterium*

PKN78874.1 *Candidatus Cloacimonetes bacterium HGW-Cloacimonetes-1*

NLD47944.1 *Clostridiaceae bacterium*

MBS4014219.1 *Bacteroidota bacterium*

WP_152118925.1 *Halosegnis rubeus*

NVL90271.1 *Desulfobacterales bacterium*

PLX21662.1 *Salinivirgaceae bacterium*

HDQ00123.1 *bacterium*

MBT4958432.1 *Flavobacteriaceae bacterium*

RYG39207.1 *Burkholderiales bacterium*

NPV37611.1 *Bacteroidales bacterium*

WP_169853060.1 *Anaerohalosphaera lusitana*

MBN1186134.1 *Bacteroidales bacterium*

MBE6664172.1 *Bacteroidales bacterium*

RME91594.1 *Anaerolineae bacterium*

MBC8216959.1 *Planctomycetota bacterium*

MBO6957072.1 *Bacteroidales bacterium*

MBQ2945372.1 *Clostridia bacterium*

MBR5500019.1 *Clostridia bacterium*

MBQ2939412.1 *Clostridia bacterium*

MBQ8696711.1 *Clostridia bacterium*

MBR5569919.1 *Oscillospiraceae bacterium*

MBP5416516.1 *Clostridiales bacterium*

MBE6942968.1 *Oscillospiraceae bacterium*

MBP3377407.1 *Clostridia bacterium*

MBQ6272113.1 *Clostridiales bacterium*

MBQ9974237.1 *Oscillospiraceae bacterium*

HIV53815.1 *Candidatus Mediterraneibacter norwichensis*

###;pgpA: phosphatidylglycerophosphatase. Direct fusions:

HFU22806.1

HGB36577.1

RKZ81292.1 *Candidatus Hydrothermota bacterium*

MBP7703501.1 *Candidatus Hydrothermota bacterium*

MCD6087025.1 *Candidatus Hydrothermota bacterium*

HDL60512.1 *candidate division WOR-3 bacterium*

#;neighborhoods

MBK7633766.1 *Saprospiraceae bacterium*

MBK8517370.1 *Saprospiraceae bacterium*

HGC35080.1

HFK98859.1

NTW34899.1 *Syntrophobacteraceae bacterium*

NSW84830.1 *Syntrophobacteraceae bacterium*

PPD54593.1 *Methylotenera sp.*

RUO69835.1 *Pseudidiomarina sediminum*

HAD49256.1 *Idiomarina sp.*

MBN1634113.1 *Ignavibacteria bacterium*

NOT68865.1 *Methylophilaceae bacterium*

MBI5402370.1 *Ignavibacteriota bacterium*

MCE1165270.1 *Bacteroidota bacterium*

RUO52328.1 *Pseudidiomarina homiensis*

PCI60955.1 *Methylophilaceae bacterium*

OYY50185.1 *Methylophilales bacterium 28-44-11*

KPQ84507.1 *Idiomarinaceae bacterium HL-53*

WP_169931434.1 *Pseudidiomarina aestuarii*

PWW13300.1 *Pseudidiomarina maritima*

WP_099034002.1 *Lacimicrobium alkaliphilum*

NOT64860.1 *Methylotenera sp.*

WP_088331632.1 *Lacimicrobium sp. SS2-24*

WP_143236420.1 *Aliidiomarina halalkaliphila*

WP_029148443.1 *Methylophilus sp. 5*

KXS36314.1 *Idiomarina sp. T82-3*

MCC5854564.1 *Idiomarina sp.*

WP_140083069.1 *Methylophilus medardicus*

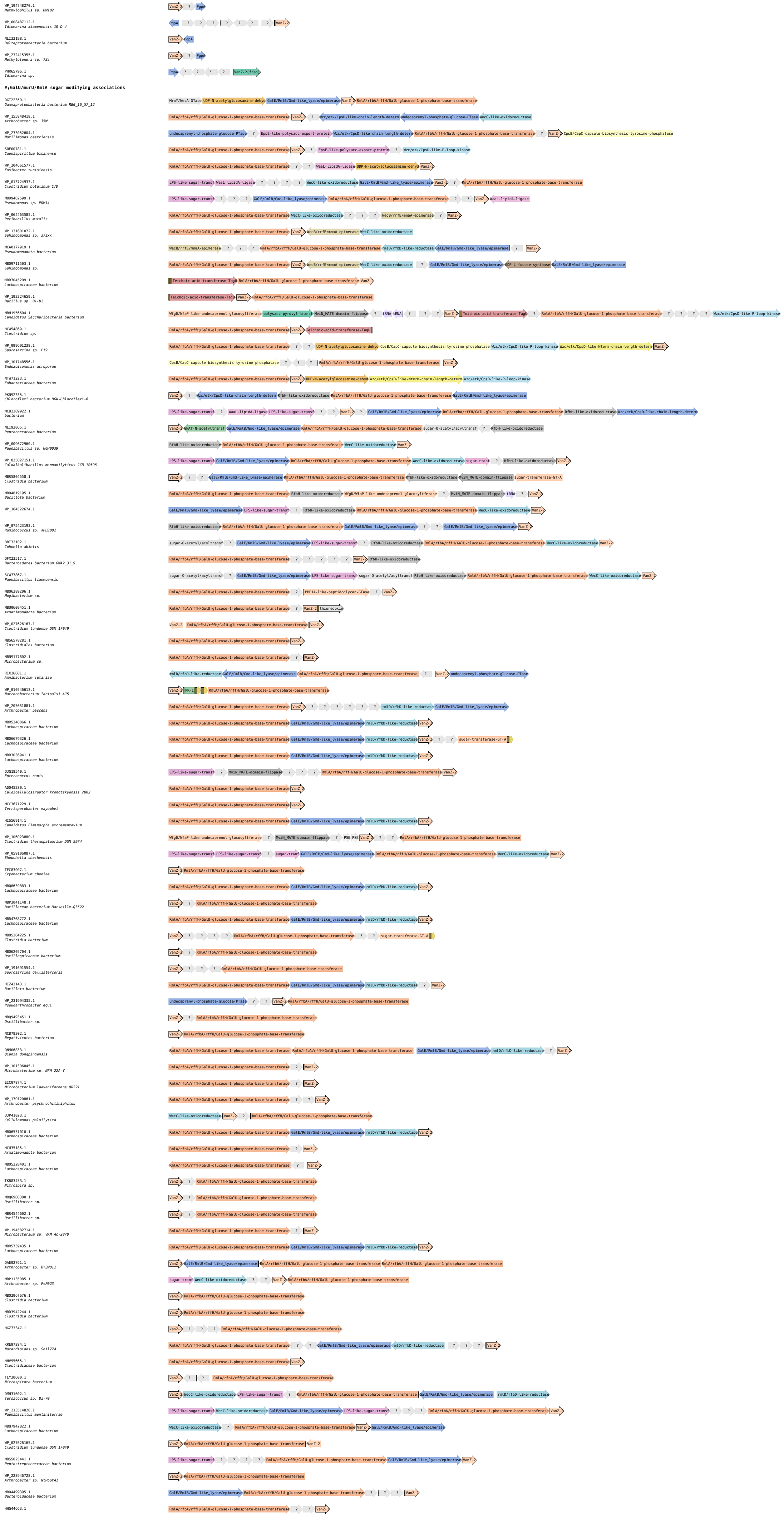

WP_194748270.1
Methylophilus sp. DW102
WP_008487112.1
Idiomarina xiamenensis 10-D-4
NLI32198.1
Deltaproteobacteria bacterium
WP_232415355.1
Methylotenera sp. 73s
PHR65796.1
Idiomarina sp.
#;GalU/murU/RmlA sugar modifying associations
OGT22359.1
Gammaproteobacteria bacterium RBG_16_57_12
WP_155848418.1
Arthrobacter sp. 35W
WP_233052604.1
Motilimonas cestriensis
SOE00781.1
Caenispirillum bisanense
WP_204661577.1
Fusibacter tunisiensis
WP_013724933.1
Clostridium botulinum C/D
MBD9482599.1
Pseudomonas sp. PDM14
WP_064463585.1
Peribacillus muralis
WP_131601072.1
Sphingomonas sp. 37zxx
MCA0177919.1
Pseudomonadota bacterium
MBD9711503.1
Sphingomonas sp.
MBR7045209.1
Lachnospiraceae bacterium
WP_193224659.1
Bacillus sp. B1-b2
MBH1956604.1
Candidatus Saccharibacteria bacterium
HCW54869.1
Clostridium sp.
WP_099691238.1
Sporosarcina sp. P19
WP_101748556.1
Endozoicomonas acroporae
NTW71223.1
Eubacteriaceae bacterium
PKN92335.1
Chloroflexi bacterium HGW-Chloroflexi-6
MCB2209922.1
bacterium
NLI92065.1
Peptococcaceae bacterium
WP_009672960.1
Paenibacillus sp. HGH0039
WP_025027151.1
Caldalkalibacillus mannanilyticus JCM 10596
MBR5804558.1
Clostridia bacterium
MBO4819195.1
Bacillota bacterium
WP_164522674.1
WP_075423193.1
Ruminococcus sp. XPD3002
BBI32102.1
Cohnella abietis
OFX23517.1
Bacteroidetes bacterium GWA2_31_9
SCW77867.1
Paenibacillus tianmuensis
MBQ6389206.1
Mogibacterium sp.
MBU0609451.1
Armatimonadota bacterium
WP_027626167.1
Clostridium lundense DSM 17049
MBS6578281.1
Clostridiales bacterium
MBN9177802.1
Microbacterium sp.
RIX26601.1
Amnibacterium setariae
WP_010546613.1
Natronobacterium lacisalsi AJ5
WP_205651881.1
Arthrobacter pascens
MBR5340066.1
Lachnospiraceae bacterium
MBQ6679326.1
Lachnospiraceae bacterium
MBR3036941.1
Lachnospiraceae bacterium
OJG18540.1
Enterococcus canis
ADQ45280.1
Caldicellulosiruptor kronotskyensis 2002
MCC3671229.1
Terrisporobacter mayombei
HIS56914.1
Candidatus Fimimorpha excrementavium
WP_106023880.1
Clostridium thermopalmarium DSM 5974
WP_059106087.1
Shouchella shacheensis
TFC83007.1
Cryobacterium cheniae
MBQ8639883.1
Lachnospiraceae bacterium
MBP3041148.1
Bacillaceae bacterium Marseille-Q3522
MBR4768772.1
Lachnospiraceae bacterium
MBO5204225.1
Clostridia bacterium
MBQ6205704.1
Oscillospiraceae bacterium
WP_191691554.1
Sporosarcina gallistercoris
HIZ43143.1
Bacillota bacterium
WP_231994335.1
Pseudarthrobacter equi
MBQ9493451.1
Oscillibacter sp.
NCB78302.1
Negativicutes bacterium
QNM06833.1
Qiania dongpingensis
WP_101306845.1
Microbacterium sp. NFH-22A-Y
EIC07874.1
Microbacterium laevaniformans OR221
WP_170120861.1
Arthrobacter psychrochitiniphilus
UJP41023.1
Cellulomonas palmilytica
MBQ6551010.1
Lachnospiraceae bacterium
HCU35185.1
Armatimonadota bacterium
MBO5228401.1
Lachnospiraceae bacterium
TKB83453.1
Nitrospira sp.
MBQ6986300.1
Oscillibacter sp.
MBR4544602.1
Oscillibacter sp.
WP_194582714.1
Microbacterium sp. VKM Ac-2870
MBR5739435.1
Lachnospiraceae bacterium
OAE82761.1
Arthrobacter sp. OY3WO11
MBP1135085.1
Arthrobacter sp. PvP023
MBQ2967676.1
Clostridia bacterium
MBR3942244.1
Clostridia bacterium
HGZ73347.1
KRE97284.1
Nocardioides sp. Soil774
HHV95665.1
Clostridiaceae bacterium
TLY30600.1
Nitrospirota bacterium
OMH31602.1
Tersicoccus sp. Bi-70
WP_213514820.1
Paenibacillus montaniterrae
MBQ7942822.1
Lachnospiraceae bacterium
WP_027626165.1
Clostridium lundense DSM 17049
MBS5025441.1
Peptostreptococcaceae bacterium
WP_223946728.1
Arthrobacter sp. NtRootA1
MBO4499395.1
Bacteroidaceae bacterium
HHG44663.1

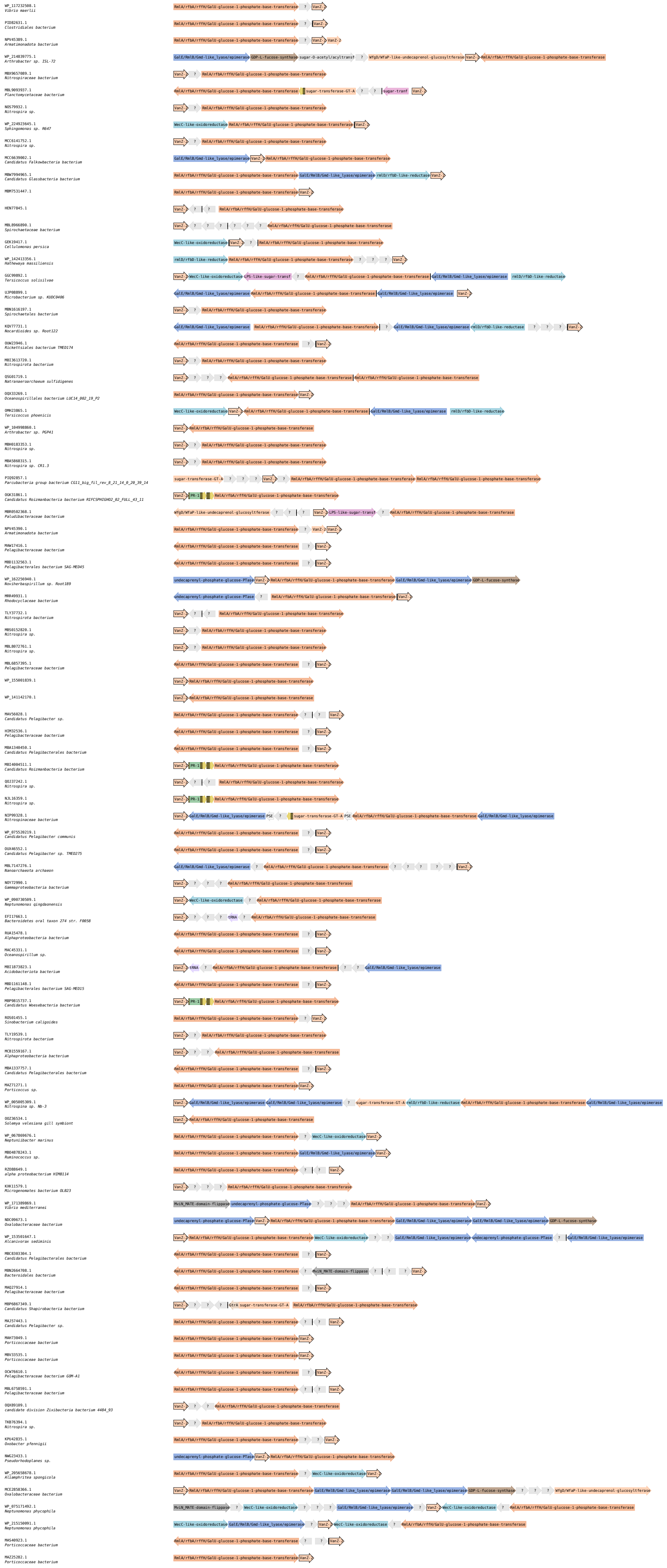

WP_117232508.1
Vibrio maerlii
PID82631.1
Clostridiales bacterium
NPV45389.1
Armatimonadota bacterium
WP_214839775.1
Arthrobacter sp. ISL-72
MBX9657089.1
Nitrospiraceae bacterium
MBL9093937.1
Planctomycetaceae bacterium
NOS79932.1
Nitrospira sp.
WP_224923645.1
Sphingomonas sp. R647
MCC6141752.1
Nitrospira sp.
MCC6639002.1
Candidatus Falkowbacteria bacterium
MBW7994965.1
Candidatus Glassbacteria bacterium
MBM7531447.1
HEN77845.1
MBL8966890.1
Spirochaetaceae bacterium
GEK19417.1
Cellulomonas persica
WP_142413356.1
Hathewaya massiliensis
GGC99892.1
Tersicoccus solisilvae
UJP08899.1
Microbacterium sp. KUDC0406
MBN1616197.1
Spirochaetales bacterium
KQV77731.1
Nocardioides sp. Root122
OUW23946.1
Rickettsiales bacterium TMED174
MBI3613720.1
Nitrospirota bacterium
QSG01719.1
Natranaeroarchaeum sulfidigenes
OQX33269.1
Oceanospirillales bacterium LUC14_002_19_P2
QMH23865.1
Tersicoccus phoenicis
WP_104998860.1
Arthrobacter sp. PGP41
MBH0183353.1
Nitrospira sp.
MBA5868315.1
Nitrospira sp. CR1.3
PIQ92857.1
Parcubacteria group bacterium CG11_big_fil_rev_8_21_14_0_20_39_14
OGK31861.1
Candidatus Roizmanbacteria bacterium RIFCSPHIGHO2_02_FULL_43_11
MBR8502368.1
Paludibacteraceae bacterium
NPV45390.1
Armatimonadota bacterium
MAW17416.1
Pelagibacteraceae bacterium
MBD1132563.1
Pelagibacterales bacterium SAG-MED45
WP_162256940.1
Noviherbaspirillum sp. Root189
MRR49931.1
Rhodocyclaceae bacterium
TLY37732.1
Nitrospirota bacterium
MBS0152820.1
Nitrospira sp.
MBL8072761.1
Nitrospira sp.
MBL6857395.1
Pelagibacteraceae bacterium
WP_155001839.1
WP_141142170.1
MAV56028.1
Candidatus Pelagibacter sp.
HIM32536.1
Pelagibacteraceae bacterium
MBA1340450.1
Candidatus Pelagibacterales bacterium
MBI4004511.1
Candidatus Roizmanbacteria bacterium
OOJ37242.1
Nitrospira sp.
NJL16359.1
Nitrospira sp.
NIP99328.1
Nitrospinaceae bacterium
WP_075520219.1
Candidatus Pelagibacter communis
OUX46552.1
Candidatus Pelagibacter sp. TMED275
MBL7147276.1
Nanoarchaeota archaeon
NOY72998.1
Gammaproteobacteria bacterium
WP_098730509.1
Neptunomonas qingdaonensis
EFI17663.1
Bacteroidetes oral taxon 274 str. F0058
RUA15478.1
Alphaproteobacteria bacterium
MAC45331.1
Oceanospirillum sp.
MBI1873823.1
Acidobacteriota bacterium
MBD1161148.1
Pelagibacterales bacterium SAG-MED15
MBP9815737.1
Candidatus Woesebacteria bacterium
ROS01455.1
Sinobacterium caligoides
TLY19539.1
Nitrospirota bacterium
MCB1559167.1
Alphaproteobacteria bacterium
MBA1337757.1
Candidatus Pelagibacterales bacterium
MAZ71271.1
Porticoccus sp.
WP_005005309.1
Nitrospina sp. Nb-3
OOZ36534.1
Solemya velesiana gill symbiont
WP_067869676.1
Neptunibacter marinus
MBO4878243.1
Ruminococcus sp.
RZO88649.1
alpha proteobacterium HIMB114
KKK11579.1
Microgenomates bacterium OLB23
WP_171389869.1
Vibrio mediterranei
NDC09673.1
Oxalobacteraceae bacterium
WP_153501647.1
Alcanivorax sediminis
MBC8303304.1
Candidatus Pelagibacterales bacterium
MBN2664708.1
Bacteroidales bacterium
MAQ27914.1
Pelagibacteraceae bacterium
MBP8867349.1
Candidatus Shapirobacteria bacterium
MAJ57443.1
Candidatus Pelagibacter sp.
MAH73049.1
Porticoccaceae bacterium
MBV33535.1
Porticoccaceae bacterium
OCW76610.1
Pelagibacteraceae bacterium GOM-A1
MBL6758591.1
Pelagibacteraceae bacterium
OGX89109.1
candidate division Zixibacteria bacterium 4484_93
TKB76394.1
Nitrospira sp.
KPU42835.1
Oxobacter pfennigii
NWG23433.1
Pseudorhodoplanes sp.
WP_205658678.1
Allamphritea spongicola
MCE2858366.1
Oxalobacteraceae bacterium
WP_075171492.1
Neptunomonas phycophila
WP_215150091.1
Neptunomonas phycophila
MAS40923.1
Porticoccaceae bacterium
MAZ25282.1
Porticoccaceae bacterium

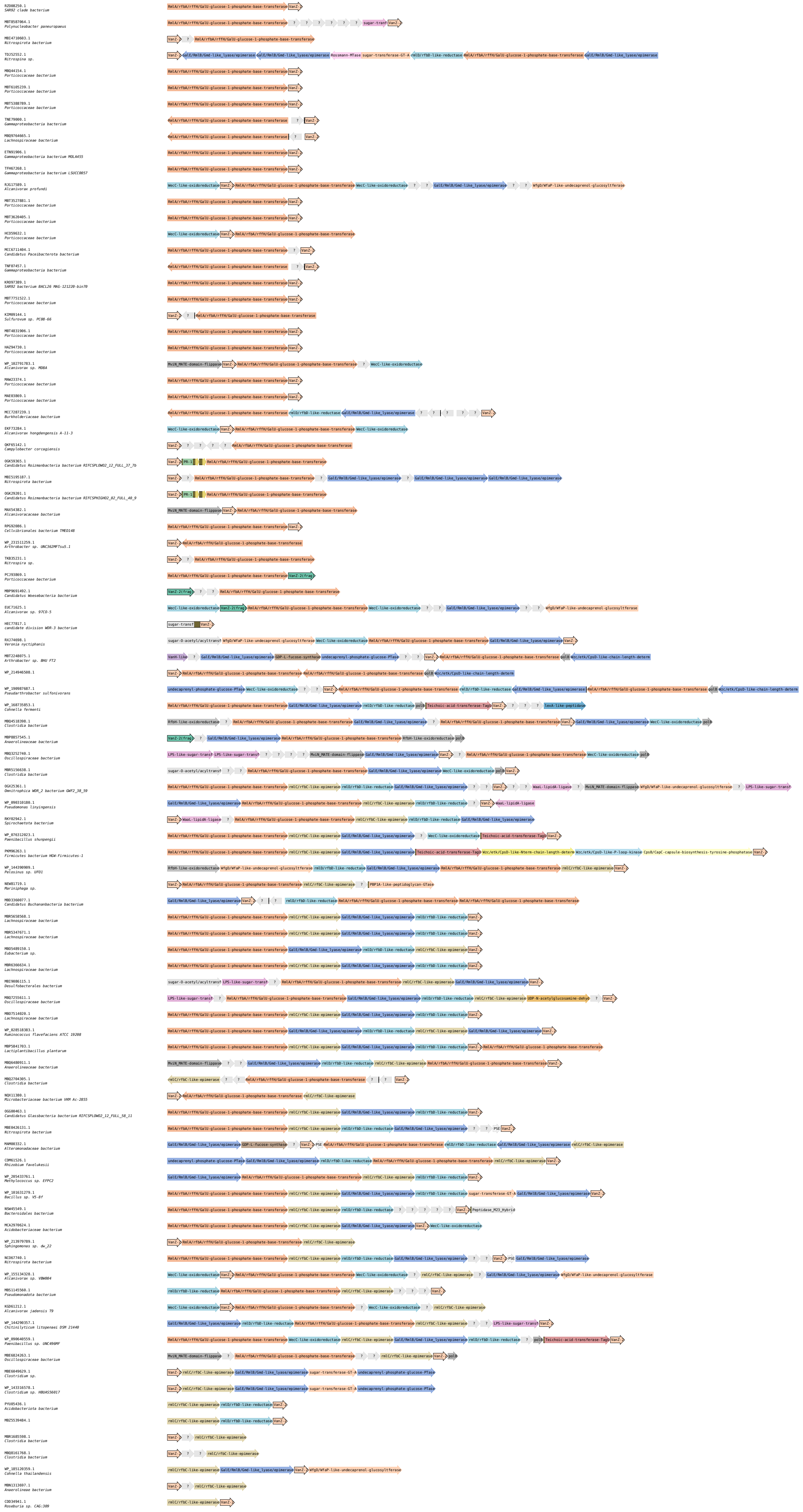

RZO08250.1 SAR92 clade bacterium
MBT8587064.1 Polynucleobacter paneuropaeus
MBI4710603.1 Nitrospirota bacterium
TDJ52552.1 Nitrospina sp.
MBQ44154.1 Porticoccaceae bacterium
MBT6105239.1 Porticoccaceae bacterium
MBT5388789.1 Porticoccaceae bacterium
TNE79000.1 Gammaproteobacteria bacterium
MBQ9764665.1 Lachnospiraceae bacterium
ETN91906.1 Gammaproteobacteria bacterium MOLA455
TFH67268.1 Gammaproteobacteria bacterium LSUCC0057
RJG17589.1 Alcanivorax profundi
MBT3527881.1 Porticoccaceae bacterium
MBT3620405.1 Porticoccaceae bacterium
HCO59632.1 Porticoccaceae bacterium
MCC6711404.1 Candidatus Paceibacterota bacterium
TNF07457.1 Gammaproteobacteria bacterium
KRO97389.1 SAR92 bacterium BACL26 MAG-121220-bin70
MBT7751522.1 Porticoccaceae bacterium
KIM09144.1 Sulfurovum sp. PC08-66
MBT4831906.1 Porticoccaceae bacterium
HAZ94730.1 Porticoccaceae bacterium
WP_102791783.1 Alcanivorax sp. MD8A
MAW23374.1 Porticoccaceae bacterium
MAE03869.1 Porticoccaceae bacterium
MCC7287239.1 Burkholderiaceae bacterium
EKF73284.1 Alcanivorax hongdengensis A-11-3
QKF65142.1 Campylobacter corcagiensis
OGK59365.1 Candidatus Roizmanbacteria bacterium RIFCSPLOWO2_12_FULL_37_7b
MBI5195187.1 Nitrospirota bacterium
OGK29201.1 Candidatus Roizmanbacteria bacterium RIFCSPHIGHO2_02_FULL_40_9
MAX54382.1 Alcanivoracaceae bacterium
RPG92886.1 Cellvibrionales bacterium TMED148
WP_231511259.1 Arthrobacter sp. UNC362MFTsu5.1
TKB35231.1 Nitrospira sp.
PCJ93869.1 Porticoccaceae bacterium
MBP9691492.1 Candidatus Woesebacteria bacterium
EUC71625.1 Alcanivorax sp. 97CO-5
HEC77817.1 candidate division WOR-3 bacterium
RXJ74698.1 Veronia nyctiphanis
MBT2248075.1 Arthrobacter sp. BHU FT2
WP_214946508.1
WP_190987687.1 Pseudarthrobacter sulfonivorans
WP_168735853.1 Cohnella fermenti
MBQ4518398.1 Clostridia bacterium
MBP8857545.1 Anaerolineaceae bacterium
MBQ3252740.1 Oscillospiraceae bacterium
MBR5156638.1 Clostridia bacterium
OGX25361.1 Omnitrophica WOR_2 bacterium GWF2_38_59
WP_090310180.1 Pseudomonas linyingensis
RKY02942.1 Spirochaetota bacterium
WP_076312023.1 Paenibacillus shunpengii
PKM96263.1 Firmicutes bacterium HGW-Firmicutes-1
WP_144390999.1 Pelosinus sp. UFO1
NEW81719.1 Mariniphaga sp.
MBD3360077.1 Candidatus Buchananbacteria bacterium
MBR5658568.1 Lachnospiraceae bacterium
MBR5347671.1 Lachnospiraceae bacterium
MBO5489150.1 Eubacterium sp.
MBR6366634.1 Lachnospiraceae bacterium
MBI9086115.1 Desulfobacterales bacterium
MBQ7255611.1 Oscillospiraceae bacterium
MBO7514020.1 Lachnospiraceae bacterium
WP_028518383.1 Ruminococcus flavefaciens ATCC 19208
MRP5841703.1 Lactiplantibacillus plantarum
MBQ6480911.1 Anaerolineaceae bacterium
MBQ2704305.1 Clostridia bacterium
NQX11380.1 Microbacteriaceae bacterium VKM Ac-2855
OGG80463.1 Candidatus Glassbacteria bacterium RIFCSPLOWO2_12_FULL_58_11
MBE0426131.1 Nitrospirota bacterium
MAM00332.1 Alteromonadaceae bacterium
CDM61526.1 Rhizobium favelukesii
WP_205433761.1 Methylococcus sp. EFPC2
WP_101631279.1 Bacillus sp. V5-8f
NSW45549.1 Bacteroidales bacterium
MCA2970624.1 Acidobacteriaceae bacterium
WP_213979789.1 Sphingomonas sp. dw_22
NCO67740.1 Nitrospirota bacterium
WP_155134328.1 Alcanivorax sp. VBW004
MBS1145560.1 Pseudomonadota bacterium
KGD61212.1 Alcanivorax jadensis T9
WP_144290357.1 Chitinilyticum litopenaei DSM 21440
WP_090640559.1 Paenibacillus sp. UNC496MF
MBE6824263.1 Oscillospiraceae bacterium
MBE6049629.1 Clostridium sp.
WP_143316578.1 Clostridium sp. HBUAS56017
PYU85436.1 Acidobacteriota bacterium
MBZ5539484.1
MBR1685598.1 Clostridia bacterium
MBQ8161768.1 Clostridia bacterium
WP_185120359.1 Cohnella thailandensis
MBN1313697.1 Anaerolineae bacterium
CDD34941.1 Roseburia sp. CAG:309

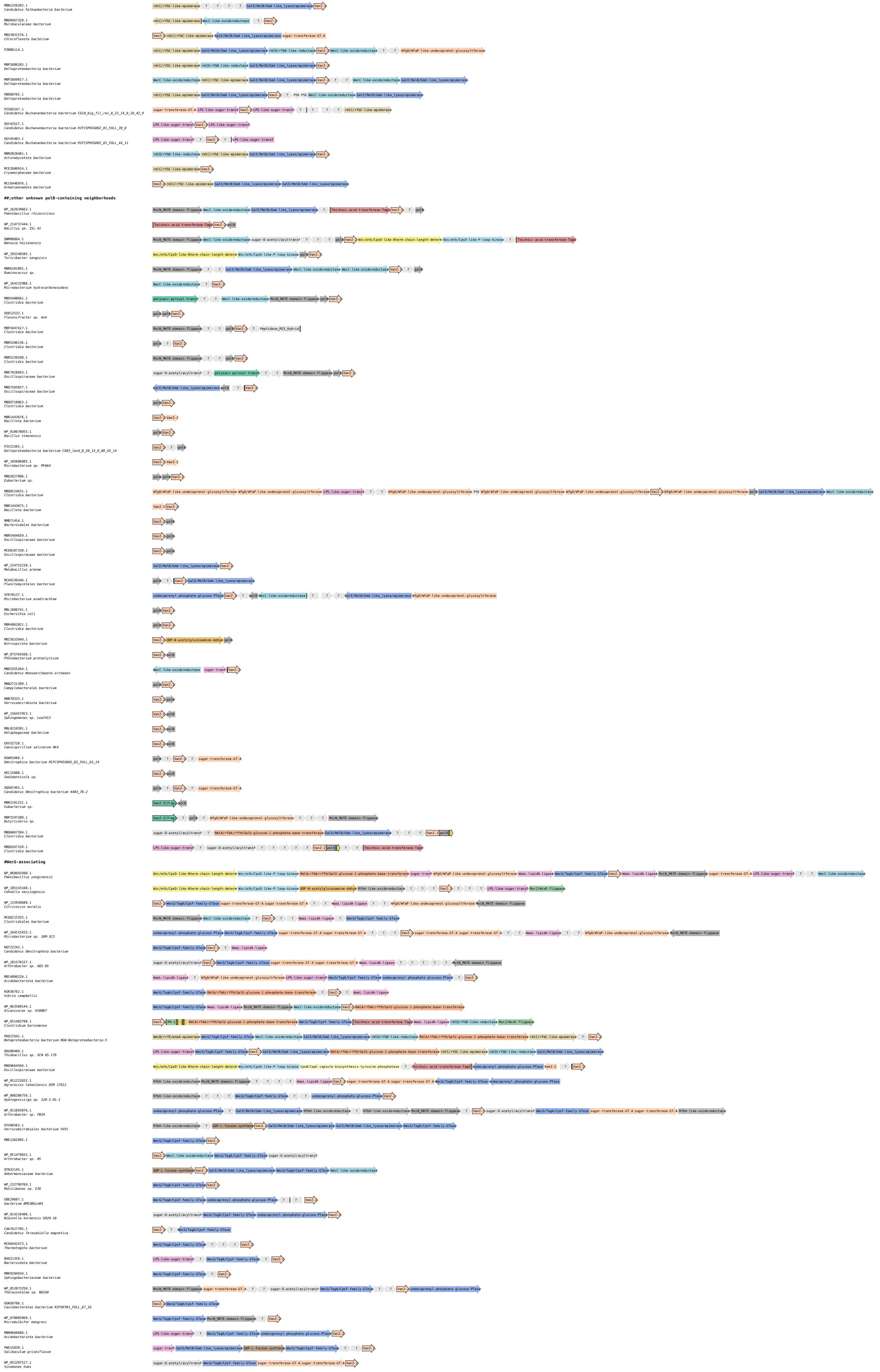

MBN1326202.1 Candidatus Falkowbacteria bacterium
rmlC/rfbC-like-epimerase ? ? ? ? GalE/RmlB/Gmd-like_lyase/epimerase VanZ
MBQ9467329.1 Muribaculaceae bacterium
rmlC/rfbC-like-epimerase WecC-like-oxidoreductase ? VanZ
MBX3031576.1 Chloroflexota bacterium
VanZ rmlC/rfbC-like-epimerase GalE/RmlB/Gmd-like_lyase/epimerase sugar-transferase-GT-A
PZN86114.1
rmlC/rfbC-like-epimerase GalE/RmlB/Gmd-like_lyase/epimerase rmlD/rfbD-like-reductase VanZ WecC-like-oxidoreductase ? ? WfgD/WfaP-like-undecaprenol-glucosyltferase
MBP2686265.1 Deltaproteobacteria bacterium
rmlC/rfbC-like-epimerase rmlD/rfbD-like-reductase GalE/RmlB/Gmd-like_lyase/epimerase VanZ
MBP2669017.1 Deltaproteobacteria bacterium
WecC-like-oxidoreductase rmlC/rfbC-like-epimerase GalE/RmlB/Gmd-like_lyase/epimerase VanZ ? ? WecC-like-oxidoreductase GalE/RmlB/Gmd-like_lyase/epimerase
HBD68702.1 Deltaproteobacteria bacterium
rmlC/rfbC-like-epimerase GalE/RmlB/Gmd-like_lyase/epimerase VanZ ? PSE PSE WecC-like-oxidoreductase GalE/RmlB/Gmd-like_lyase/epimerase
PIS05547.1 Candidatus Buchananbacteria bacterium CG10_big_fil_rev_8_21_14_0_10_42_9
sugar-transferase-GT-A LPS-like-sugar-transf VanZ LPS-like-sugar-transf ? ? ? ? rmlC/rfbC-like-epimerase
OGY43527.1 Candidatus Buchananbacteria bacterium RIFCSPHIGHO2_01_FULL_39_8
LPS-like-sugar-transf VanZ LPS-like-sugar-transf
OGY45483.1 Candidatus Buchananbacteria bacterium RIFCSPHIGHO2_01_FULL_44_11
LPS-like-sugar-transf ? VanZ ? LPS-like-sugar-transf
MBM2828481.1 Actinomycetota bacterium
rmlD/rfbD-like-reductase rmlC/rfbC-like-epimerase GalE/RmlB/Gmd-like_lyase/epimerase VanZ
MCE2686914.1 Cryomorphaceae bacterium
rmlC/rfbC-like-epimerase VanZ
MCC6446976.1 Armatimonadota bacterium
VanZ rmlC/rfbC-like-epimerase GalE/RmlB/Gmd-like_lyase/epimerase GalE/RmlB/Gmd-like_lyase/epimerase
##;other unknown polB-containing neighborhoods
WP_162639662.1 Paenibacillus rhizovicinus
MviN_MATE-domain-flippase WecC-like-oxidoreductase GalE/RmlB/Gmd-like_lyase/epimerase ? Teichoic-acid-transferase-TagO VanZ ? polB
WP_214737444.1 Bacillus sp. ISL-41
Teichoic-acid-transferase-TagO VanZ polB
QMU08804.1 Wansuia hejianensis
MviN_MATE-domain-flippase WecC-like-oxidoreductase sugar-O-acetyl/acyltransf ? ? ? polB VanZ Wzc/etk/CpsD-like-Nterm-chain-length-determ Wzc/etk/CpsD-like-P-loop-kinase ? Teichoic-acid-transferase-TagO
WP_195248585.1 Turicibacter sanguinis
Wzc/etk/CpsD-like-Nterm-chain-length-determ Wzc/etk/CpsD-like-P-loop-kinase polB VanZ
MBR6101891.1 Ruminococcus sp.
MviN_MATE-domain-flippase ? ? GalE/RmlB/Gmd-like_lyase/epimerase WecC-like-oxidoreductase WecC-like-oxidoreductase VanZ ? polB
WP_164232986.1 Microbacterium hydrocarbonoxydans
WecC-like-oxidoreductase ? VanZ
MBO5408661.1 Clostridia bacterium
polysacc-pyruvyl-transf ? ? WecC-like-oxidoreductase MviN_MATE-domain-flippase polB VanZ
OUO12532.1 Flavonifractor sp. An4
polB polB VanZ
MBP3447417.1 Clostridia bacterium
MviN_MATE-domain-flippase ? ? polB VanZ ? Peptidase_M23_Hybrid
MBR5280136.1 Clostridia bacterium
polB ? VanZ
MBR5239108.1 Clostridia bacterium
MviN_MATE-domain-flippase ? ? polB VanZ
MBE7010943.1 Oscillospiraceae bacterium
sugar-O-acetyl/acyltransf ? polysacc-pyruvyl-transf ? ? MviN_MATE-domain-flippase polB VanZ
MBQ7565657.1 Oscillospiraceae bacterium
GalE/RmlB/Gmd-like_lyase/epimerase polB ? VanZ
MBQ9718863.1 Clostridia bacterium
polB VanZ
MBR1443676.1 Bacillota bacterium
VanZ VanZ-2
WP_010678055.1 Bacillus timonensis
polB VanZ
PIV21565.1 Deltaproteobacteria bacterium CG03_land_8_20_14_0_80_45_14
VanZ ? polB
WP_105686085.1 Microbacterium sp. MYb64
VanZ VanZ-2
MBQ2627006.1 Eubacterium sp.
polB polB VanZ
MBQ8524631.1 Clostridia bacterium
WfgD/WfaP-like-undecaprenol-glucosyltferase WfgD/WfaP-like-undecaprenol-glucosyltferase LPS-like-sugar-transf ? ? WfgD/WfaP-like-undecaprenol-glucosyltferase PSE WfgD/WfaP-like-undecaprenol-glucosyltferase WfgD/WfaP-like-undecaprenol-glucosyltferase VanZ WfgD/WfaP-like-undecaprenol-glucosyltferase polB GalE/RmlB/Gmd-like_lyase/epimerase WecC-like-oxidoreductase
MBR1443675.1 Bacillota bacterium
VanZ-2 VanZ
NMB71454.1 Bacteroidales bacterium
VanZ polB
MBR5404659.1 Oscillospiraceae bacterium
VanZ polB
MCD8107330.1 Oscillospiraceae bacterium
VanZ polB
WP_224752238.1 Metabacillus arenae
GalE/RmlB/Gmd-like_lyase/epimerase VanZ
MCA9239346.1 Planctomycetales bacterium
polB ? VanZ GalE/RmlB/Gmd-like_lyase/epimerase
SFR78137.1 Microbacterium azadirachtae
undecaprenyl-phosphate-glucose-PTase VanZ ? polB WecC-like-oxidoreductase ? ? ? GalE/RmlB/Gmd-like_lyase/epimerase WfgD/WfaP-like-undecaprenol-glucosyltferase
MBL1006741.1 Escherichia coli
polB VanZ
MBR4962021.1 Clostridia bacterium
polB VanZ
MBI5633949.1 Nitrospirota bacterium
VanZ UDP-N-acetylglucosamine-dehyd polB
WP_075764568.1 Photobacterium proteolyticum
VanZ polB
MBD3355264.1 Candidatus Woesearchaeota archaeon
WecC-like-oxidoreductase sugar-tranf VanZ
MBN2721399.1 Campylobacterales bacterium
polB VanZ
NBB78335.1 Verrucomicrobiota bacterium
VanZ polB
WP_156457953.1 Sphingomonas sp. Leaf412
VanZ polB
MBL0210391.1 Holophagaceae bacterium
VanZ polB
EKV32728.1 Caenispirillum salinarum AK4
VanZ polB
OGW91666.1 Omnitrophica bacterium RIFCSPHIGHO2_02_FULL_63_14
polB ? VanZ ? sugar-transferase-GT-A
HEC15088.1 Sedimenticola sp.
VanZ polB
QQX87491.1 Candidatus Omnitrophica bacterium 4484_70.2
polB ? VanZ ? sugar-transferase-GT-A
MBR2191231.1 Eubacterium sp.
VanZ-2(frag) polB
MBP3197280.1 Butyrivibrio sp.
VanZ-2(frag) ? polB ? WfgD/WfaP-like-undecaprenol-glucosyltferase ? ? ? MviN_MATE-domain-flippase
MBQ6067394.1 Clostridia bacterium
sugar-O-acetyl/acyltransf ? RmlA/rfbA/rffH/GalU-glucose-1-phosphate-base-transferase GalE/RmlB/Gmd-like_lyase/epimerase ? ? ? VanZ-2 polB
MBQ6267310.1 Clostridia bacterium
LPS-like-sugar-transf ? sugar-O-acetyl/acyltransf ? ? ? ? ? VanZ-2 polB ? ? Teichoic-acid-transferase-TagO
#WecG-associating
WP_068693490.1 Paenibacillus yonginensis
Wzc/etk/CpsD-like-Nterm-chain-length-determ Wzc/etk/CpsD-like-P-loop-kinase RmlA/rfbA/rffH/GalU-glucose-1-phosphate-base-transferase sugar-tranf WfgD/WfaP-like-undecaprenol-glucosyltferase WaaL-lipidA-ligase WecG/TagA/CpsF-family-GTase VanZ WaaL-lipidA-ligase MviN_MATE-domain-flippase sugar-transferase-GT-A LPS-like-sugar-transf ? ? WecC-like-oxidoreductase
WP_185143168.1 Cohnella nanjingensis
Wzc/etk/CpsD-like-Nterm-chain-length-determ Wzc/etk/CpsD-like-P-loop-kinase UDP-N-acetylglucosamine-dehyd RfbH-like-oxidoreductase ? ? ? VanZ ? ? ? LPS-like-sugar-transf MurJ/WzxE-flippase
WP_115930689.1 Citricoccus muralis
VanZ WecG/TagA/CpsF-family-GTase sugar-transferase-GT-A sugar-transferase-GT-A ? ? WaaL-lipidA-ligase ? ? WfgD/WfaP-like-undecaprenol-glucosyltferase MviN_MATE-domain-flippase
MCD8215355.1 Clostridiales bacterium
MviN_MATE-domain-flippase WecC-like-oxidoreductase ? VanZ ? ? WaaL-lipidA-ligase ? WecG/TagA/CpsF-family-GTase
WP_164515433.1 Microbacterium sp. 10M-3C3
undecaprenyl-phosphate-glucose-PTase WecG/TagA/CpsF-family-GTase sugar-transferase-GT-A sugar-transferase-GT-A ? ? ? VanZ sugar-transferase-GT-A sugar-transferase-GT-A ? ? WaaL-lipidA-ligase ? ? WfgD/WfaP-like-undecaprenol-glucosyltferase MviN_MATE-domain-flippase
NQT22242.1 Candidatus Omnitrophota bacterium
WecG/TagA/CpsF-family-GTase VanZ ? WaaL-lipidA-ligase
WP_181578227.1 Arthrobacter sp. AD5-05
sugar-O-acetyl/acyltransf VanZ WecG/TagA/CpsF-family-GTase sugar-transferase-GT-A sugar-transferase-GT-A WaaL-lipidA-ligase ? ? ? ? ? MviN_MATE-domain-flippase
MBI4890226.1 Acidobacteriota bacterium
WaaL-lipidA-ligase ? WfgD/WfaP-like-undecaprenol-glucosyltferase LPS-like-sugar-transf WecG/TagA/CpsF-family-GTase undecaprenyl-phosphate-glucose-PTase ? VanZ
KGR36762.1 Vibrio campbellii
WecG/TagA/CpsF-family-GTase RmlA/rfbA/rffH/GalU-glucose-1-phosphate-base-transferase ? VanZ ? WaaL-lipidA-ligase
WP_063509144.1 Alcanivorax sp. HI0007
WecG/TagA/CpsF-family-GTase WaaL-lipidA-ligase MviN_MATE-domain-flippase WecC-like-oxidoreductase VanZ RmlA/rfbA/rffH/GalU-glucose-1-phosphate-base-transferase
WP_051483798.1 Clostridium bornimense
VanZ PR-1 RmlA/rfbA/rffH/GalU-glucose-1-phosphate-base-transferase WecG/TagA/CpsF-family-GTase Teichoic-acid-transferase-TagO WaaL-lipidA-ligase rmlD/rfbD-like-reductase MurJ/WzxE-flippase
PKO27641.1 Betaproteobacteria bacterium HGW-Betaproteobacteria-5
WecB/rffE/mnaA-epimerase WecG/TagA/CpsF-family-GTase WecC-like-oxidoreductase GalE/RmlB/Gmd-like_lyase/epimerase rmlD/rfbD-like-reductase RmlA/rfbA/rffH/GalU-glucose-1-phosphate-base-transferase rmlC/rfbC-like-epimerase ? VanZ
OOU88489.1 Thiobacillus sp. SCN 65-179
LPS-like-sugar-transf WecG/TagA/CpsF-family-GTase VanZ GalE/RmlB/Gmd-like_lyase/epimerase RmlA/rfbA/rffH/GalU-glucose-1-phosphate-base-transferase rmlC/rfbC-like-epimerase rmlD/rfbD-like-reductase GalE/RmlB/Gmd-like_lyase/epimerase
MBQ9664504.1 Oscillospiraceae bacterium
Wzc/etk/CpsD-like-Nterm-chain-length-determ Wzc/etk/CpsD-like-P-loop-kinase CpsB/CapC-capsule-biosynthesis-tyrosine-phosphatase ? Teichoic-acid-transferase-TagO undecaprenyl-phosphate-glucose-PTase VanZ-2 ? VanZ
WP_051222832.1 Agrococcus lahaulensis DSM 17612
RfbH-like-oxidoreductase MviN_MATE-domain-flippase ? ? ? ? WaaL-lipidA-ligase VanZ sugar-transferase-GT-A sugar-transferase-GT-A WecG/TagA/CpsF-family-GTase undecaprenyl-phosphate-glucose-PTase
WP_008286756.1 Hydrogenivirga sp. 128-5-R1-1
RfbH-like-oxidoreductase ? ? ? WecG/TagA/CpsF-family-GTase ? ? undecaprenyl-phosphate-glucose-PTase VanZ
WP_011093876.1 Arthrobacter sp. FB24
undecaprenyl-phosphate-glucose-PTase ? GalE/RmlB/Gmd-like_lyase/epimerase RfbH-like-oxidoreductase ? RfbH-like-oxidoreductase MviN_MATE-domain-flippase ? VanZ sugar-O-acetyl/acyltransf WecG/TagA/CpsF-family-GTase sugar-transferase-GT-A sugar-transferase-GT-A RfbH-like-oxidoreductase
OYV06562.1 Verrucomicrobiales bacterium VVV1
RfbH-like-oxidoreductase ? GDP-L-fucose-synthase VanZ GalE/RmlB/Gmd-like_lyase/epimerase GalE/RmlB/Gmd-like_lyase/epimerase WecG/TagA/CpsF-family-GTase
MBE1362995.1
WecG/TagA/CpsF-family-GTase VanZ
WP_051479032.1 Arthrobacter sp. H5
VanZ WecC-like-oxidoreductase WecG/TagA/CpsF-family-GTase sugar-O-acetyl/acyltransf
QTN32145.1 Akkermansiaceae bacterium
GDP-L-fucose-synthase VanZ GalE/RmlB/Gmd-like_lyase/epimerase WecG/TagA/CpsF-family-GTase WecC-like-oxidoreductase
WP_232799769.1 Motilimonas sp. E26
WecG/TagA/CpsF-family-GTase VanZ
GBE29607.1 bacterium BMS3Bbin04
WecG/TagA/CpsF-family-GTase undecaprenyl-phosphate-glucose-PTase ? ? VanZ
WP_014216406.1 Niastella koreensis GR20-10
sugar-O-acetyl/acyltransf WecG/TagA/CpsF-family-GTase undecaprenyl-phosphate-glucose-PTase VanZ
CAA7627705.1 Candidatus Terasakiella magnetica
VanZ ? WecG/TagA/CpsF-family-GTase
MCD6442473.1 Thermotogota bacterium
WecG/TagA/CpsF-family-GTase ? ? ? VanZ
NVO21359.1 Bacteroidota bacterium
LPS-like-sugar-transf ? WecG/TagA/CpsF-family-GTase ? VanZ
MBK9284934.1 Sphingobacteriaceae bacterium
WecG/TagA/CpsF-family-GTase ? VanZ
WP_052073250.1 Thalassotalea sp. ND16A
MviN_MATE-domain-flippase sugar-transferase-GT-A ? ? sugar-O-acetyl/acyltransf WecG/TagA/CpsF-family-GTase ? ? VanZ undecaprenyl-phosphate-glucose-PTase
OGN50706.1 Caulobacterales bacterium RIFOXYB1_FULL_67_16
VanZ WecG/TagA/CpsF-family-GTase
WP_078085969.1 Microbulbifer mangrovi
WecG/TagA/CpsF-family-GTase MviN_MATE-domain-flippase ? VanZ
MBN9660686.1 Acidobacteriota bacterium
LPS-like-sugar-transf ? WecG/TagA/CpsF-family-GTase undecaprenyl-phosphate-glucose-PTase VanZ
PWG15839.1 Salibaculum griseiflavum
sugar-tranf GalE/RmlB/Gmd-like_lyase/epimerase GDP-L-fucose-synthase WecG/TagA/CpsF-family-GTase ? ? VanZ
WP_052207517.1 Sinomonas humi
sugar-O-acetyl/acyltransf WecG/TagA/CpsF-family-GTase sugar-transferase-GT-A sugar-transferase-GT-A VanZ

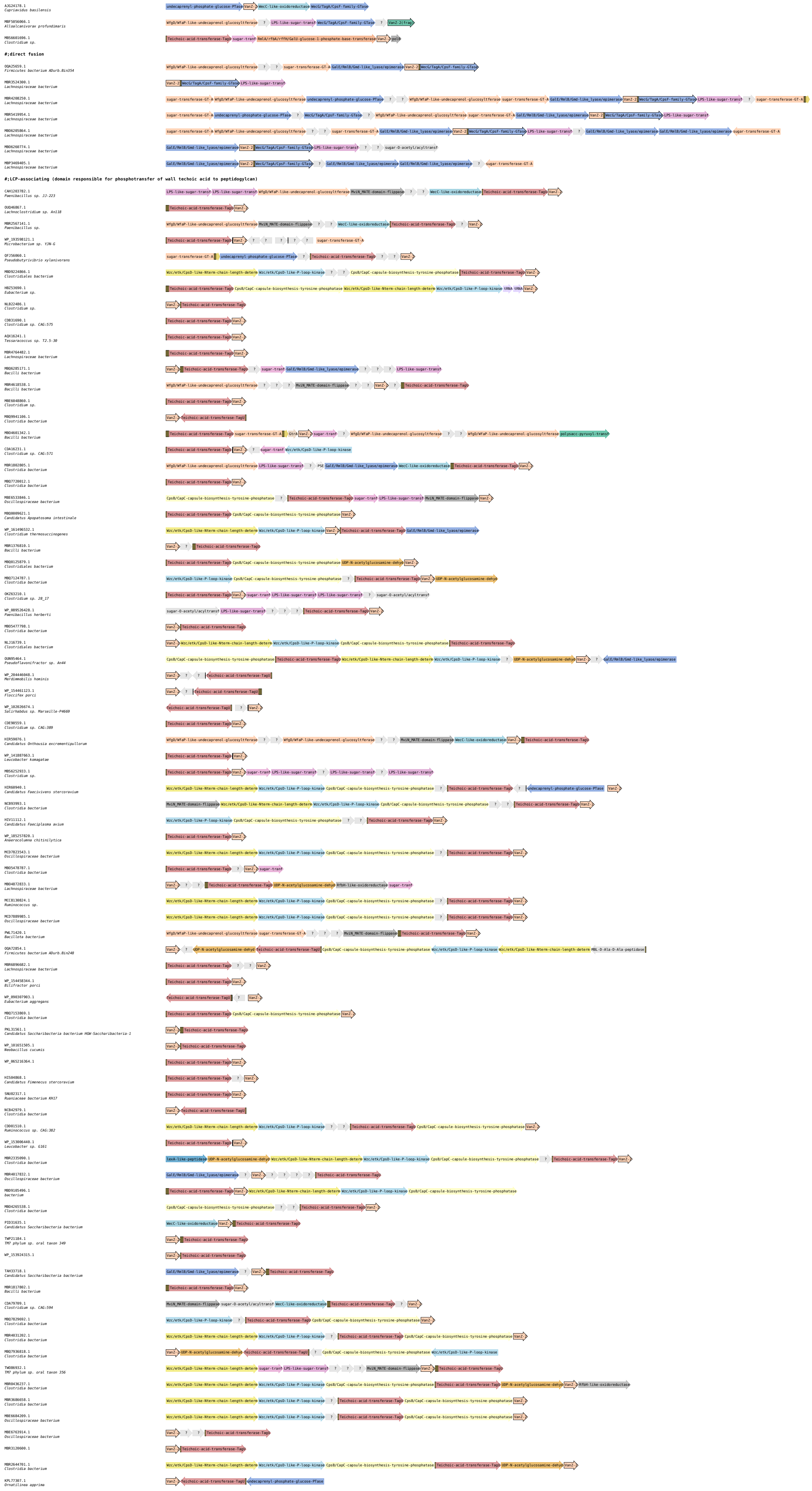

AJG24178.1
Cupriavidus basilensis
MBF5056066.1
Alloalcanivorax profundimaris
MBS6601696.1
Clostridium sp.
#;direct fusion
OQA25659.1
Firmicutes bacterium ADurb.Bin354
MBR3524300.1
Lachnospiraceae bacterium
MBR4208250.1
Lachnospiraceae bacterium
MBR5419954.1
Lachnospiraceae bacterium
MBO6205864.1
Lachnospiraceae bacterium
MBO6260774.1
Lachnospiraceae bacterium
MBP3469405.1
Lachnospiraceae bacterium
#;LCP-associating (domain responsible for phosphotransfer of wall techoic acid to peptidogylcan)
CAH1203782.1
Paenibacillus sp. JJ-223
OUQ46867.1
Lachnoclostridium sp. An118
MBR2567141.1
Paenibacillus sp.
WP_193598121.1
Microbacterium sp. YJN-G
QFJ56060.1
Pseudobutyrivibrio xylanivorans
MBD9224866.1
Clostridiales bacterium
HBZ53690.1
Eubacterium sp.
NLB22486.1
Clostridium sp.
CDB31690.1
Clostridium sp. CAG:575
AQX16241.1
Tessaracoccus sp. T2.5-30
MBR4764482.1
Lachnospiraceae bacterium
MBQ6285171.1
Bacilli bacterium
MBR4618538.1
Bacilli bacterium
MBE6048860.1
Clostridium sp.
MBQ9941106.1
Clostridia bacterium
MBO4601342.1
Bacilli bacterium
CDA16231.1
Clostridium sp. CAG:571
MBR1802805.1
Clostridia bacterium
MBQ7720012.1
Clostridia bacterium
MBE6533846.1
Oscillospiraceae bacterium
MBQ0009621.1
Candidatus Apopatosoma intestinale
WP_161496532.1
Clostridium thermosuccinogenes
MBR1376810.1
Bacilli bacterium
MBQ0125879.1
Clostridiales bacterium
MBQ7124787.1
Clostridia bacterium
OKZ63210.1
Clostridium sp. 28_17
WP_089526428.1
Paenibacillus herberti
MBO5477798.1
Clostridia bacterium
NLJ16739.1
Clostridiales bacterium
OUN95464.1
Pseudoflavonifractor sp. An44
WP_204446048.1
Merdimmobilis hominis
WP_154461123.1
Floccifex porci
WP_102026674.1
Salirhabdus sp. Marseille-P4669
CDE90559.1
Clostridium sp. CAG:389
HIR59076.1
Candidatus Onthousia excrementipullorum
WP_141887663.1
Leucobacter komagatae
MBS6252933.1
Clostridium sp.
HIR60940.1
Candidatus Faecivivens stercoravium
NCB93993.1
Clostridia bacterium
HIV11112.1
Candidatus Faeciplasma avium
WP_185257820.1
Anaerocolumna chitinilytica
MCD7823543.1
Oscillospiraceae bacterium
MBO5478787.1
Clostridia bacterium
MBO4872833.1
Lachnospiraceae bacterium
MCC8130824.1
Ruminococcus sp.
MCD7889985.1
Oscillospiraceae bacterium
PWL71420.1
Bacillota bacterium
OQA72854.1
Firmicutes bacterium ADurb.Bin248
MBR6896682.1
Lachnospiraceae bacterium
WP_154458344.1
Bilifractor porci
WP_090307903.1
Eubacterium aggregans
MBQ7153869.1
Clostridia bacterium
PKL31561.1
Candidatus Saccharibacteria bacterium HGW-Saccharibacteria-1
WP_101651505.1
Neobacillus cucumis
WP_065216364.1
HIS04868.1
Candidatus Fimenecus stercoravium
SNU02317.1
Ruaniaceae bacterium KH17
NCB42979.1
Clostridia bacterium
CDD01510.1
Ruminococcus sp. CAG:382
WP_153006440.1
Leucobacter sp. G161
MBR2335090.1
Clostridia bacterium
MBR4017832.1
Oscillospiraceae bacterium
MBD9105496.1
bacterium
MBO4265538.1
Clostridia bacterium
PID31635.1
Candidatus Saccharibacteria bacterium
TWP21184.1
TM7 phylum sp. oral taxon 349
WP_153924315.1
TAH33718.1
Candidatus Saccharibacteria bacterium
MBR1817802.1
Bacilli bacterium
CDA79709.1
Clostridium sp. CAG:594
MBQ7829692.1
Clostridia bacterium
MBR4031202.1
Clostridia bacterium
MBQ7936818.1
Clostridia bacterium
TWO86932.1
TM7 phylum sp. oral taxon 356
MBR0436237.1
Clostridia bacterium
MBR3686658.1
Clostridia bacterium
MBE6684209.1
Oscillospiraceae bacterium
MBE6763914.1
Oscillospiraceae bacterium
MBR3120600.1
MBR2644701.1
Clostridia bacterium
KPL77307.1
Ornatilinea apprima

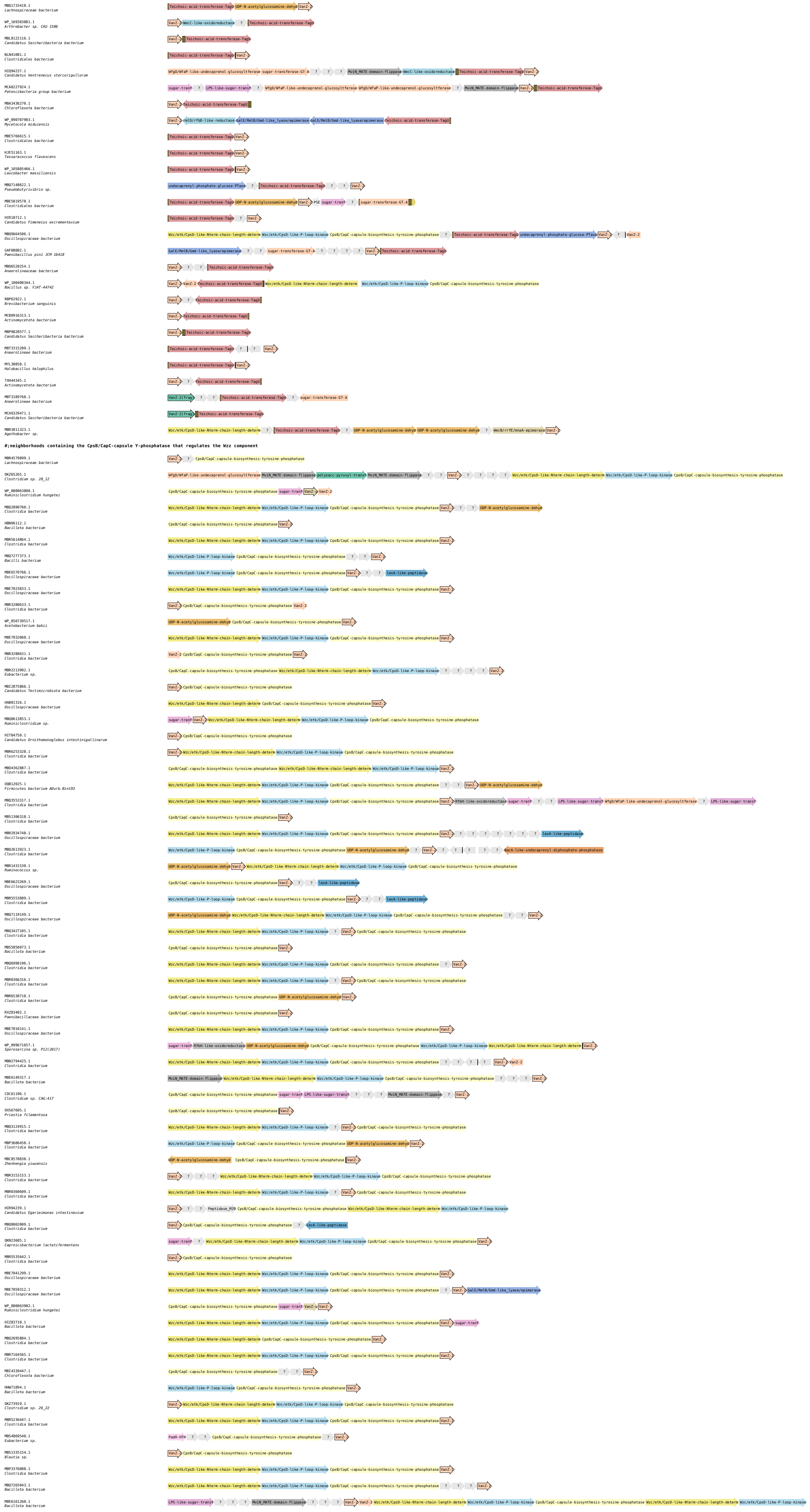
MBQ1733418.1 Lachnospiraceae bacterium
WP_169303881.1 Arthrobacter sp. CAU 1506
MBL8122116.1 Candidatus Saccharibacteria bacterium
NLN41081.1 Clostridiales bacterium
HIQ94237.1 Candidatus Ventrenecus stercoripullorum
MCA0227924.1 Patescibacteria group bacterium
MBA3436270.1 Chloroflexota bacterium
WP_090707903.1 Mycetocola miduiensis
MBE5766615.1 Clostridiales bacterium
HJE51163.1 Tessaracoccus flavescens
WP_105805466.1 Leucobacter massiliensis
MBQ7148622.1 Pseudobutyrivibrio sp.
MBE5819578.1 Clostridiales bacterium
HIR10712.1 Candidatus Fimenecus excrementavium
MBO9664506.1 Oscillospiraceae bacterium
GAF08082.1 Paenibacillus pini JCM 16418
MBQ6520254.1 Anaerolineaceae bacterium
WP_100400344.1 Bacillus sp. FJAT-44742
RBP62922.1 Brevibacterium sanguinis
MCB0916313.1 Actinomycetota bacterium
MBP9820577.1 Candidatus Saccharibacteria bacterium
MBT3315209.1 Anaerolineae bacterium
MYL30858.1 Halobacillus halophilus
TXH44345.1 Actinomycetota bacterium
MBT3189768.1 Anaerolineae bacterium
MCA9329471.1 Candidatus Saccharibacteria bacterium
MBR3811323.1 Agathobacter sp.
#;neighborhoods containing the CpsB/CapC-capsule Y-phosphatase that regulates the Wzz component
MBR4579899.1 Lachnospiraceae bacterium
OKZ65265.1 Clostridium sp. 28_12
WP_080063808.1 Ruminiclostridium hungatei
MBQ2890760.1 Clostridia bacterium
HBN96112.1 Bacillota bacterium
MBR5614864.1 Clostridia bacterium
MBQ7277373.1 Bacilli bacterium
MBE6570766.1 Oscillospiraceae bacterium
MBE7023833.1 Oscillospiraceae bacterium
MBR3280633.1 Clostridia bacterium
WP_050739517.1 Acetobacterium bakii
MBE7032060.1 Oscillospiraceae bacterium
MBR3280631.1 Clostridia bacterium
MBR2213992.1 Eubacterium sp.
MBI2875866.1 Candidatus Tectimicrobiota bacterium
HAB01316.1 Oscillospiraceae bacterium
MBQ8613853.1 Ruminiclostridium sp.
HIT84750.1 Candidatus Ornithomonoglobus intestinigallinarum
MBR6253328.1 Clostridia bacterium
MBQ4362887.1 Clostridia bacterium
OOB12025.1 Firmicutes bacterium ADurb.Bin193
MBQ3553317.1 Clostridia bacterium
MBS1306318.1 Clostridia bacterium
MBR2934748.1 Oscillospiraceae bacterium
MBQ2613923.1 Clostridia bacterium
MBR1431530.1 Ruminococcus sp.
MBE6623269.1 Oscillospiraceae bacterium
MBR5553889.1 Clostridia bacterium
MBQ7119149.1 Oscillospiraceae bacterium
MBQ3427105.1 Clostridia bacterium
MBS5856073.1 Bacillota bacterium
MBQ6998196.1 Clostridia bacterium
MBR0366316.1 Clostridia bacterium
MBR6530710.1 Clostridia bacterium
RXZ83402.1 Paenibacillaceae bacterium
MBE7010141.1 Oscillospiraceae bacterium
WP_099671657.1 Sporosarcina sp. P12(2017)
MBN2794425.1 Clostridia bacterium
MBE6149317.1 Bacillota bacterium
CDC61196.1 Clostridium sp. CAG:417
OXS67605.1 Priestia filamentosa
MBQ3124915.1 Clostridia bacterium
MBP3686458.1 Clostridia bacterium
MBC8578838.1 Zhenhengia yiwuensis
MBR3153153.1 Clostridia bacterium
MBR0360609.1 Clostridia bacterium
HIR94239.1 Candidatus Egerieimonas intestinavium
MBQ8082009.1 Clostridia bacterium
QMN23685.1 Caproicibacterium lactatifermentans
MBR5535642.1 Clostridia bacterium
MBE7041299.1 Oscillospiraceae bacterium
MBE7059312.1 Oscillospiraceae bacterium
WP_080063902.1 Ruminiclostridium hungatei
HIZ83710.1 Bacillota bacterium
MBQ2695804.1 Clostridia bacterium
MBR7164565.1 Clostridia bacterium
MBI4339447.1 Chloroflexota bacterium
HHW71894.1 Bacillota bacterium
OKZ73919.1 Clostridium sp. 26_22
MBR5236447.1 Clostridia bacterium
MBS4869540.1 Eubacterium sp.
MBS1335154.1 Blautia sp.
MBP3376808.1 Clostridia bacterium
MBQ7265043.1 Bacillota bacterium
MBE6161260.1 Bacillota bacterium

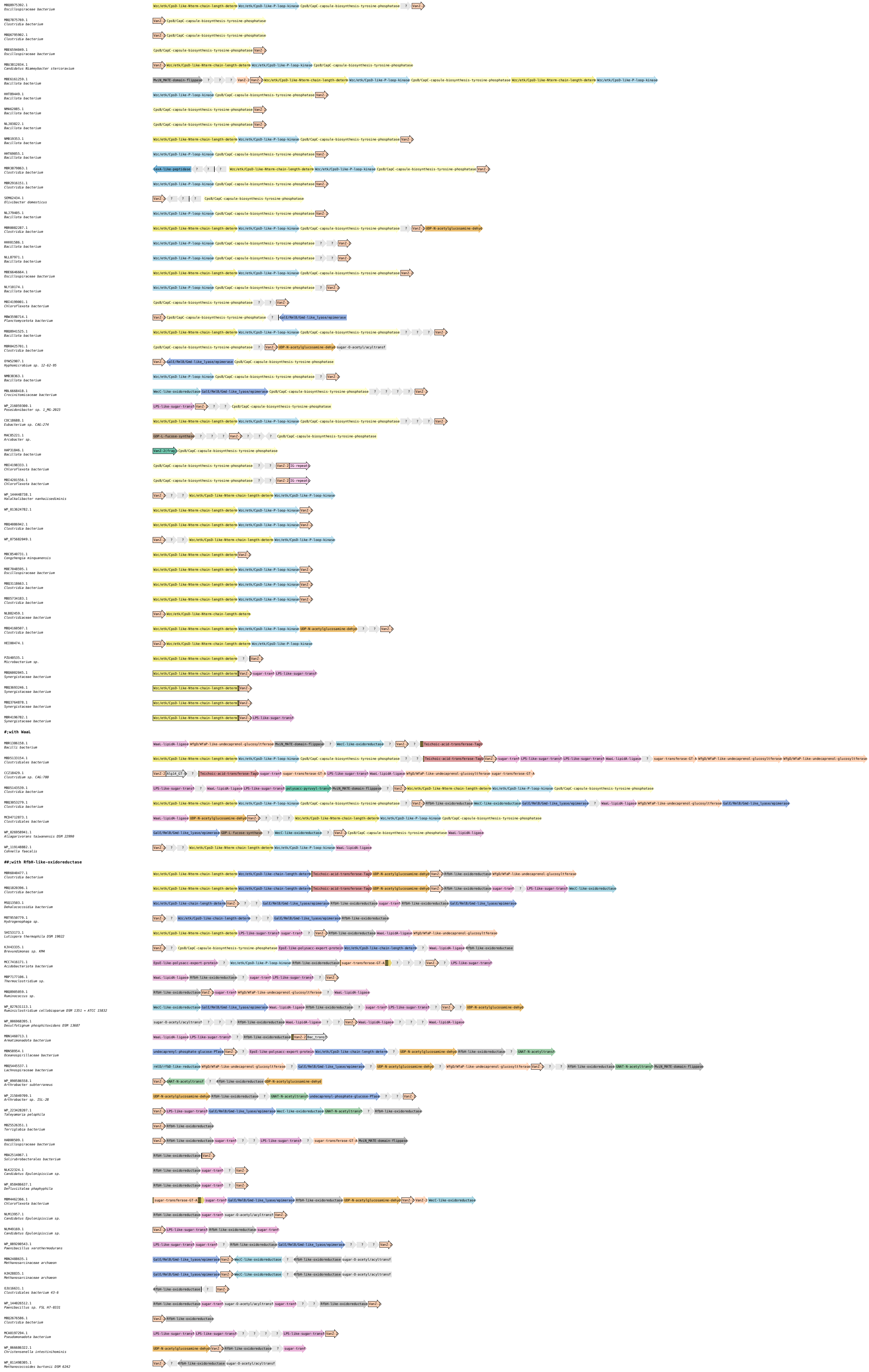
MBQ8975392.1
Oscillospiraceae bacterium
MBQ7875769.1
Clostridia bacterium
MBQ6795902.1
Clostridia bacterium
MBE6594049.1
Oscillospiraceae bacterium
MBU3812034.1
Candidatus Niameybacter stercoravium
MBE6161259.1
Bacillota bacterium
HHT89449.1
Bacillota bacterium
NMA62085.1
Bacillota bacterium
NLJ03822.1
Bacillota bacterium
NMB19353.1
Bacillota bacterium
HHT69055.1
Bacillota bacterium
MBR3879863.1
Clostridia bacterium
MBR2916151.1
Clostridia bacterium
SEM62434.1
Olivibacter domesticus
NLJ79405.1
Bacillota bacterium
MBR0082287.1
Clostridia bacterium
HHX01586.1
Bacillota bacterium
NLL87971.1
Bacillota bacterium
MBE6646664.1
Oscillospiraceae bacterium
NLY10174.1
Bacillota bacterium
MBI4199001.1
Chloroflexota bacterium
MBW3598714.1
Planctomycetota bacterium
MBQ8941525.1
Bacillota bacterium
MBR0425701.1
Clostridia bacterium
OYW52907.1
Hyphomicrobium sp. 12-62-95
NMB38363.1
Bacillota bacterium
MBL6668418.1
Crocinitomicaceae bacterium
WP_216059300.1
Poseidonibacter sp. 1_MG-2023
CDC18688.1
Eubacterium sp. CAG:274
MAC85221.1
Arcobacter sp.
HAP31846.1
Bacillota bacterium
MBI4198333.1
Chloroflexota bacterium
MBI4201556.1
Chloroflexota bacterium
WP_144448738.1
Halalkalibacter nanhaiisediminis
WP_013624782.1
MBQ4086942.1
Clostridia bacterium
WP_075682049.1
MBC8540731.1
Congzhengia minquanensis
MBE7048595.1
Oscillospiraceae bacterium
MBQ3118663.1
Clostridia bacterium
MBO5734183.1
Clostridia bacterium
NLB82459.1
Clostridiaceae bacterium
MBQ4160507.1
Clostridia bacterium
HEI00474.1
PZU40535.1
Microbacterium sp.
MBQ6002045.1
Synergistaceae bacterium
MBQ3693246.1
Synergistaceae bacterium
MBQ3764878.1
Synergistaceae bacterium
MBR4196782.1
Synergistaceae bacterium
#;with WaaL
MBR1386158.1
Bacilli bacterium
MBD5133154.1
Clostridiales bacterium
CCZ18429.1
Clostridium sp. CAG:780
MBO5143539.1
Clostridia bacterium
MBQ3053279.1
Clostridia bacterium
MCD4712873.1
Clostridiales bacterium
WP_026958941.1
Aliagarivorans taiwanensis DSM 22990
WP_119148882.1
Cohnella faecalis
##;with RfbH-like-oxidoreductase
MBR6040477.1
Clostridia bacterium
MBQ1820396.1
Clostridia bacterium
MSQ13503.1
Dehalococcoidia bacterium
MBT9550779.1
Hydrogenophaga sp.
SHI53173.1
Lutispora thermophila DSM 19022
KJV43335.1
Brevundimonas sp. KM4
MCC7416171.1
Acidobacteriota bacterium
MBP7177106.1
Thermoclostridium sp.
MBQ8905059.1
Ruminococcus sp.
WP_027631113.1
Ruminiclostridium cellobioparum DSM 1351 = ATCC 15832
WP_006968395.1
Desulfotignum phosphitoxidans DSM 13687
MBN1460713.1
Armatimonadota bacterium
MBN58954.1
Oceanospirillaceae bacterium
MBQ5445537.1
Lachnospiraceae bacterium
WP_090586558.1
Arthrobacter subterraneus
WP_215049709.1
Arthrobacter sp. ISL-28
WP_223428207.1
Tateyamaria pelophila
MBZ5526351.1
Terriglobia bacterium
HAB00509.1
Oscillospiraceae bacterium
MBA2514067.1
Solirubrobacterales bacterium
NLK22324.1
Candidatus Epulonipiscium sp.
WP_058486637.1
Defluviitalea phaphyphila
MBM4462366.1
Chloroflexota bacterium
NLM13957.1
Candidatus Epulonipiscium sp.
NLM49169.1
Candidatus Epulonipiscium sp.
WP_089200543.1
Paenibacillus xerothermodurans
MBN2488635.1
Methanosarcinaceae archaeon
HJH28835.1
Methanosarcinaceae archaeon
OJU16631.1
Clostridiales bacterium 43-6
WP_144026512.1
Paenibacillus sp. FSL H7-0331
MBQ2676586.1
Clostridia bacterium
MCA0197294.1
Pseudomonadota bacterium
WP_066686322.1
Christensenella intestinihominis
WP_011498305.1
Methanococcoides burtonii DSM 6242

MCA9246208.1
Planctomycetales bacterium

WP_118055257.1

MBD8942351.1
Clostridiales bacterium

MBC7076410.1
Syntrophomonadaceae bacterium

NOQ47838.1
Methanococcoides sp.

MBQ8349308.1
Ruminococcus sp.

MBA3687655.1
Chloroflexota bacterium

WP_101847233.1
Zhihengliuella sp. ISTPL4

MBR4425404.1
Oscillospiraceae bacterium

MCD4702846.1
Methanosarcinaceae archaeon

MBQ7128648.1
Clostridia bacterium

MBR1950359.1
Bacteroidales bacterium

WP_215701164.1
Clostridium sp. MCC353

MBQ7773172.1
Bacteroidales bacterium

MBM4462365.1
Chloroflexota bacterium

MBR2491344.1
Ruminiclostridium sp.

MBQ5784634.1
Bacteroidales bacterium

MBQ0110311.1
Candidatus Equicaccousia limihippi

WP_087862040.1
Brevefilum fermentans

MBE5958512.1
Lachnospiraceae bacterium

WP_145301982.1
Crateriforma conspicua

MBP3920599.1
Bacilli bacterium

WP_183775309.1
Catenibacillus scindens

MBN1296044.1
candidate division CSSED10-310 bacterium

RYD56689.1
Sphingobacteriales bacterium

MBE6681141.1
Oscillospiraceae bacterium

WP_050792197.1
Ruminococcus albus 8

KXK49315.1
Chloroflexi bacterium OLB13

HHM74088.1

NCD21747.1
Spartobacteria bacterium

MBN1355596.1
candidate division CSSED10-310 bacterium

WP_161407779.1
Paenibacillus silvestris

QQO11303.1
Breznakiella homolactica

MBN2243415.1
Acidobacteriota bacterium

QDU36070.1
Maioricimonas rarisocia

MCB7526393.1
Ruminococcus sp. TM463

MBK9123575.1
Candidatus Flexicrinis affinis

WP_206182320.1
Treponema medium

MBE6857488.1
Ruminococcus sp.

MBO5319330.1
Ruminococcus sp.

MBC7220204.1
Candidatus Bipolaricaulota bacterium

HIE52917.1
Armatimonadota bacterium

NLO42744.1
Bacteroidales bacterium

MBP2031237.1
Methanohalophilus levihalophilus

MBR4948327.1
Clostridiales bacterium

MBR6033925.1
Clostridia bacterium

MCA0337606.1
Actinomycetota bacterium

NCA81154.1
Opitutia bacterium

WP_012520637.1
Phenylobacterium zucineum HLK1

OLB56200.1
Nitrospirae bacterium 13_2_20CM_2_62_8

PNU18661.1
Geothermobacter hydrogeniphilus

MBL8553500.1
Phenylobacterium sp.

MBT8762800.1
Desulfovulcanus ferrireducens

EKS28266.1
Afipia felis ATCC 53690

MBU2136803.1
Alphaproteobacteria bacterium

WP_206157363.1
Mogibacterium kristiansenii

ODT85161.1
Phenylobacterium sp. SCN 70-31

MCD8554957.1
Seleniivibrio sp.

NWO07491.1
Alteromonadaceae bacterium

MBA4151180.1
Acinetobacter sp.

EHR72915.1
Burkholderiales bacterium JOSHI_001

TBV82207.1
Desulfobulbaceae bacterium

KAF0201134.1
Bacteroidota bacterium

MBI5074989.1
Nitrospirota bacterium

PIE68800.1
Deltaproteobacteria bacterium

OPY19806.1
Methanomethylovorans sp. PtaU1.Bin093

MBN1880142.1
candidate division CSSED10-310 bacterium

RZL48275.1
Variovorax sp.

MBR1909709.1
Lachnospiraceae bacterium

MBI5563193.1
Deltaproteobacteria bacterium

WP_062485657.1
Candidatus Nitrospira inopinata

WP_205234750.1
Desulfopila inferna

MCB2173674.1
bacterium

SCH65237.1
uncultured Ruminococcus sp.

MBQ6174890.1
Clostridia bacterium

#;with unknown polB

WP_077360339.1

OZB49286.1
Polynucleobacter sp. 39-45-136

##;GtrA-associated

MBQ6830182.1
Clostridia bacterium

NIQ78247.1
Anaerolineae bacterium

OIP79641.1
Parcubacteria group bacterium CG2_30_48_51

WP_118851944.1

TKJ29834.1
Chloroflexi bacterium B3_Chlor

WP_067948810.1
Agromyces sp. NDB4Y10

WP_154548502.1
Lactobacillus porci

WP_154548505.1
Lactobacillus porci

MBN2853826.1
Clostridia bacterium

WP_232496936.1
Agromyces humatus

OGV47271.1
Lentisphaerae bacterium GWF2_57_35

QNG36302.1
Geodermatophilaceae bacterium NBWT11

MBO4309929.1
Lachnospiraceae bacterium

MBQ1608038.1
Lachnospiraceae bacterium

WP_184198069.1
Armatimonas rosea

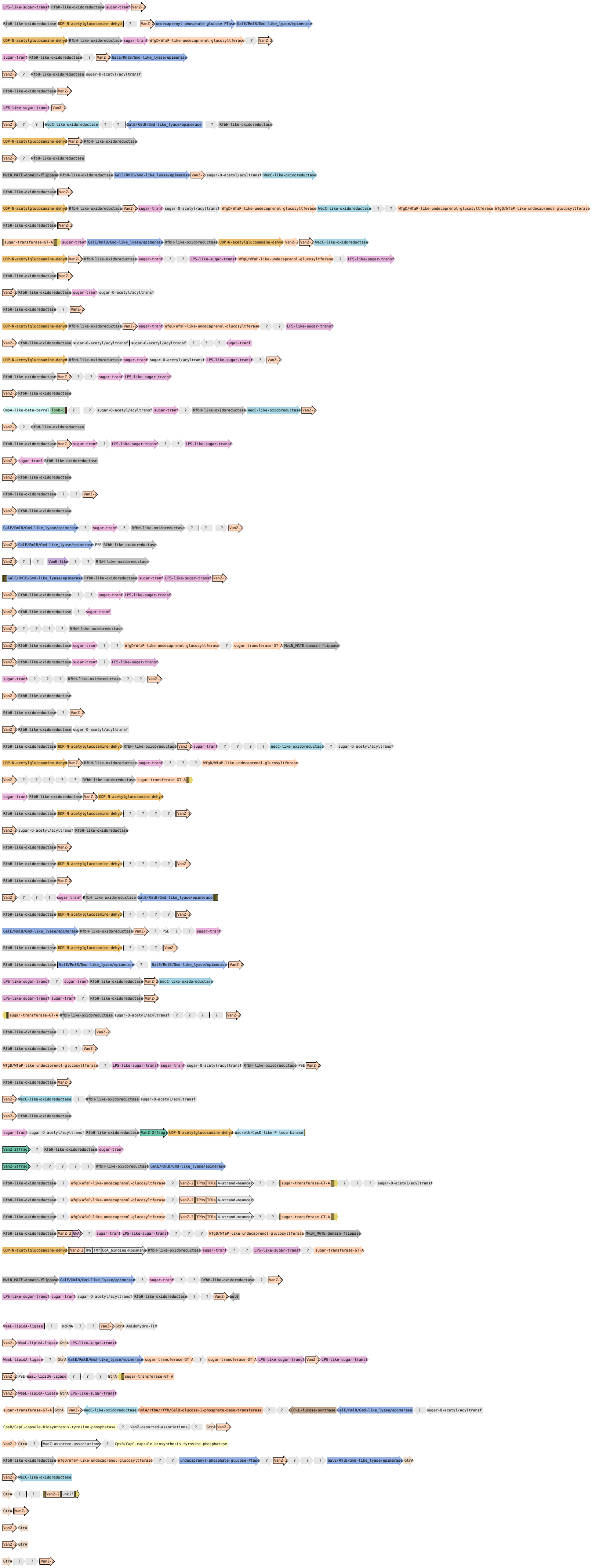

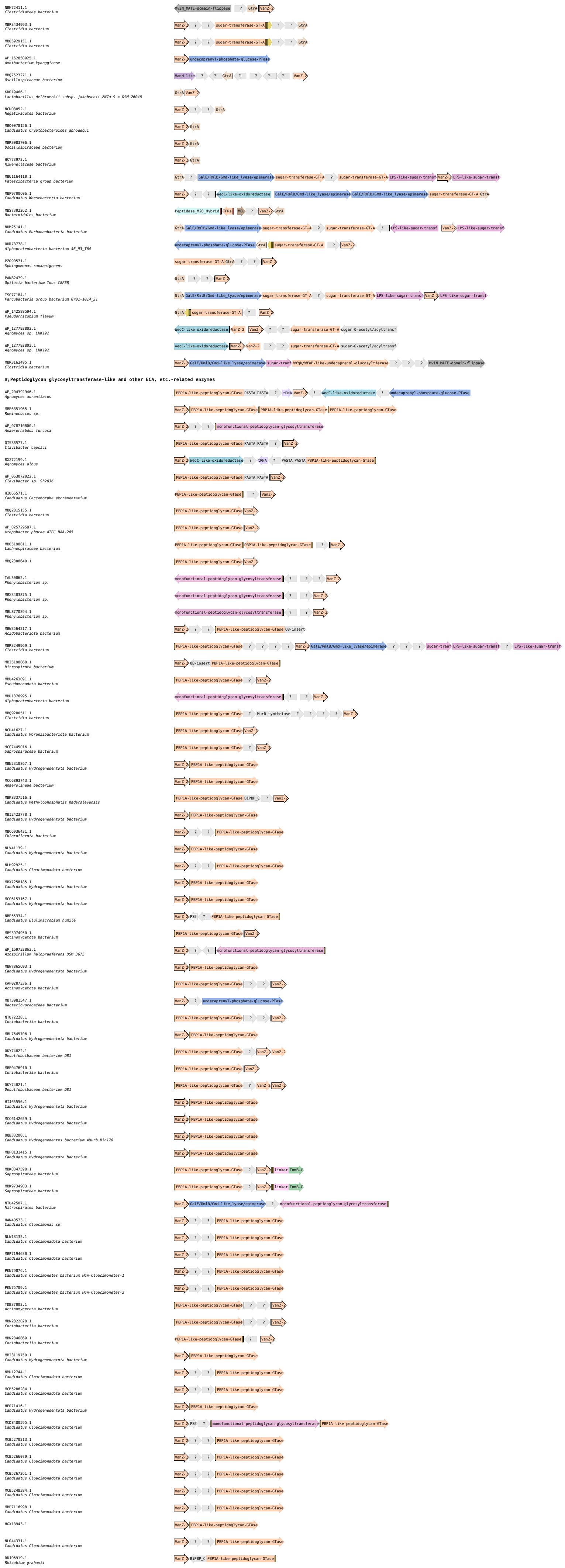
NBH72411.1 Clostridiaceae bacterium
MviN_MATE-domain-flippase ? GtrA VanZ-2
MBP3434993.1 Clostridia bacterium
VanZ-2 ? ? sugar-transferase-GT-A ? ? GtrA
MBO5929151.1 Clostridia bacterium
VanZ-2 ? ? sugar-transferase-GT-A ? ? GtrA
WP_162850925.1 Amnibacterium kyonggiense
VanZ-2 undecaprenyl-phosphate-glucose-PTase
MBQ7523271.1 Oscillospiraceae bacterium
VanH-like ? ? GtrA ? ? ? ? VanZ-2
KRO19466.1 Lactobacillus delbrueckii subsp. jakobsenii ZN7a-9 = DSM 26046
GtrA VanZ-2
NCD08852.1 Negativicutes bacterium
VanZ-2 ? ? GtrA
MBQ0078156.1 Candidatus Cryptobacteroides aphodequi
VanZ-2 GtrA
MBR3083706.1 Oscillospiraceae bacterium
VanZ-2 GtrA
HCY73973.1 Rikenellaceae bacterium
VanZ-2 GtrA
MBU1164110.1 Patescibacteria group bacterium
GtrA ? GalE/RmlB/Gmd-like_lyase/epimerase sugar-transferase-GT-A ? sugar-transferase-GT-A LPS-like-sugar-transf VanZ-2 LPS-like-sugar-transf
MBP9700606.1 Candidatus Woesebacteria bacterium
VanZ-2 ? ? WecC-like-oxidoreductase GalE/RmlB/Gmd-like_lyase/epimerase GalE/RmlB/Gmd-like_lyase/epimerase sugar-transferase-GT-A GtrA
MBS7302262.1 Bacteroidales bacterium
Peptidase_M28_Hybrid TPRs MBL ? VanZ-2 GtrA
NUM25141.1 Candidatus Buchananbacteria bacterium
GtrA GalE/RmlB/Gmd-like_lyase/epimerase sugar-transferase-GT-A ? sugar-transferase-GT-A ? LPS-like-sugar-transf VanZ-2 LPS-like-sugar-transf
OUR78778.1 Alphaproteobacteria bacterium 46_93_T64
undecaprenyl-phosphate-glucose-PTase GtrA sugar-transferase-GT-A ? VanZ-2
PZO90571.1 Sphingomonas sanxanigenens
sugar-transferase-GT-A GtrA ? ? VanZ-2
PAW82479.1 Opitutia bacterium Tous-C8FEB
GtrA ? ? VanZ-2
TSC77184.1 Parcubacteria group bacterium Gr01-1014_31
GtrA GalE/RmlB/Gmd-like_lyase/epimerase sugar-transferase-GT-A ? sugar-transferase-GT-A LPS-like-sugar-transf VanZ-2 LPS-like-sugar-transf
WP_142588594.1 Pseudorhizobium flavum
GtrA sugar-transferase-GT-A ? VanZ-2
WP_127792802.1 Agromyces sp. LHK192
WecC-like-oxidoreductase VanZ-2 VanZ-2 ? ? sugar-transferase-GT-A sugar-O-acetyl/acyltransf
WP_127792803.1 Agromyces sp. LHK192
WecC-like-oxidoreductase VanZ-2 VanZ-2 ? ? sugar-transferase-GT-A sugar-O-acetyl/acyltransf
MBR3163495.1 Clostridia bacterium
VanZ-2 GalE/RmlB/Gmd-like_lyase/epimerase sugar-tranf WfgD/WfaP-like-undecaprenol-glucosyltferase ? ? ? MviN_MATE-domain-flippase
#;Peptidoglycan glycosyltransferase-like and other ECA, etc.-related enzymes
WP_204392946.1 Agromyces aurantiacus
PBP1A-like-peptidoglycan-GTase PASTA PASTA ? tRNA VanZ-2 ? WecC-like-oxidoreductase ? undecaprenyl-phosphate-glucose-PTase
MBE6851965.1 Ruminococcus sp.
VanZ-2 PBP1A-like-peptidoglycan-GTase PBP1A-like-peptidoglycan-GTase PBP1A-like-peptidoglycan-GTase
WP_078710800.1 Anaerorhabdus furcosa
VanZ-2 ? ? monofunctional-peptidoglycan-glycosyltransferase
QIS38577.1 Clavibacter capsici
PBP1A-like-peptidoglycan-GTase PASTA PASTA ? VanZ-2
RXZ72199.1 Agromyces albus
VanZ-2 WecC-like-oxidoreductase ? tRNA ? PASTA PASTA PBP1A-like-peptidoglycan-GTase
WP_063072022.1 Clavibacter sp. Sh2036
PBP1A-like-peptidoglycan-GTase PASTA PASTA VanZ-2
HIU66571.1 Candidatus Caccomorpha excrementavium
PBP1A-like-peptidoglycan-GTase ? VanZ-2
MBQ2815155.1 Clostridia bacterium
PBP1A-like-peptidoglycan-GTase VanZ-2
WP_025729587.1 Atopobacter phocae ATCC BAA-285
PBP1A-like-peptidoglycan-GTase VanZ-2
MBO5198811.1 Lachnospiraceae bacterium
PBP1A-like-peptidoglycan-GTase PBP1A-like-peptidoglycan-GTase ? VanZ-2
MBQ2388640.1
PBP1A-like-peptidoglycan-GTase VanZ-2
TAL30862.1 Phenylobacterium sp.
monofunctional-peptidoglycan-glycosyltransferase ? ? ? VanZ-2
MBX3483875.1 Phenylobacterium sp.
monofunctional-peptidoglycan-glycosyltransferase ? ? VanZ-2
MBL8770894.1 Phenylobacterium sp.
monofunctional-peptidoglycan-glycosyltransferase ? ? VanZ-2
MBW3564217.1 Acidobacteriota bacterium
VanZ-2 ? ? PBP1A-like-peptidoglycan-GTase OB-insert
MBR3249969.1 Clostridia bacterium
PBP1A-like-peptidoglycan-GTase ? ? ? ? VanZ-2 GalE/RmlB/Gmd-like_lyase/epimerase ? ? ? sugar-tranf LPS-like-sugar-transf ? LPS-like-sugar-transf
MBI5198868.1 Nitrospirota bacterium
VanZ-2 OB-insert PBP1A-like-peptidoglycan-GTase
MBU4263091.1 Pseudomonadota bacterium
PBP1A-like-peptidoglycan-GTase ? VanZ-2
MBU1376995.1 Alphaproteobacteria bacterium
monofunctional-peptidoglycan-glycosyltransferase ? ? VanZ-2
MBQ9280511.1 Clostridia bacterium
PBP1A-like-peptidoglycan-GTase ? MurD-synthetase ? ? ? ? VanZ-2
NCU41627.1 Candidatus Moraniibacteriota bacterium
PBP1A-like-peptidoglycan-GTase VanZ-2
MCC7445016.1 Saprospiraceae bacterium
PBP1A-like-peptidoglycan-GTase ? VanZ-2
MBN2310867.1 Candidatus Hydrogenedentota bacterium
VanZ-2 PBP1A-like-peptidoglycan-GTase
MCC6893743.1 Anaerolineae bacterium
VanZ-2 PBP1A-like-peptidoglycan-GTase
MBK8337516.1 Candidatus Methylophosphatis haderslevensis
PBP1A-like-peptidoglycan-GTase BiPBP_C ? VanZ-2
MBI2423778.1 Candidatus Hydrogenedentota bacterium
VanZ-2 PBP1A-like-peptidoglycan-GTase
MBC6936431.1 Chloroflexota bacterium
VanZ-2 ? ? PBP1A-like-peptidoglycan-GTase
NLV41139.1 Candidatus Hydrogenedentota bacterium
VanZ-2 PBP1A-like-peptidoglycan-GTase
NLH92925.1 Candidatus Cloacimonadota bacterium
VanZ-2 ? ? PBP1A-like-peptidoglycan-GTase
MBX7258185.1 Candidatus Hydrogenedentota bacterium
VanZ-2 PBP1A-like-peptidoglycan-GTase
MCC6153167.1 Candidatus Hydrogenedentota bacterium
VanZ-2 PBP1A-like-peptidoglycan-GTase
NBP55334.1 Candidatus Elulimicrobium humile
VanZ-2 PSE ? PBP1A-like-peptidoglycan-GTase
MBS3974950.1 Actinomycetota bacterium
PBP1A-like-peptidoglycan-GTase VanZ-2
WP_169732863.1 Azospirillum halopraeferens DSM 3675
VanZ-2 ? ? monofunctional-peptidoglycan-glycosyltransferase
MBW7865693.1 Candidatus Hydrogenedentota bacterium
VanZ-2 PBP1A-like-peptidoglycan-GTase
KAF0207336.1 Actinomycetota bacterium
PBP1A-like-peptidoglycan-GTase ? ? VanZ-2
MBT3981547.1 Bacteriovoracaceae bacterium
VanZ-2 ? undecaprenyl-phosphate-glucose-PTase
NTU72228.1 Coriobacteriia bacterium
PBP1A-like-peptidoglycan-GTase ? ? VanZ-2
MBL7645706.1 Candidatus Hydrogenedentota bacterium
VanZ-2 PBP1A-like-peptidoglycan-GTase
OKY74822.1 Desulfobulbaceae bacterium DB1
PBP1A-like-peptidoglycan-GTase ? VanZ-2 VanZ-2
MBE0476910.1 Coriobacteriia bacterium
PBP1A-like-peptidoglycan-GTase VanZ-2
OKY74821.1 Desulfobulbaceae bacterium DB1
PBP1A-like-peptidoglycan-GTase ? VanZ-2 VanZ-2
HIJ65556.1 Candidatus Hydrogenedentota bacterium
VanZ-2 PBP1A-like-peptidoglycan-GTase
MCC6142659.1 Candidatus Hydrogenedentota bacterium
VanZ-2 PBP1A-like-peptidoglycan-GTase
OQB33200.1 Candidatus Hydrogenedentes bacterium ADurb.Bin170
VanZ-2 PBP1A-like-peptidoglycan-GTase
MBP8131415.1 Candidatus Hydrogenedentota bacterium
VanZ-2 PBP1A-like-peptidoglycan-GTase
MBK8347598.1 Saprospiraceae bacterium
PBP1A-like-peptidoglycan-GTase ? VanZ-2 linker TonB-C
MBK9734903.1 Saprospiraceae bacterium
PBP1A-like-peptidoglycan-GTase ? VanZ-2 linker TonB-C
NTU42507.1 Nitrospirales bacterium
VanZ-2 GalE/RmlB/Gmd-like_lyase/epimerase ? monofunctional-peptidoglycan-glycosyltransferase
HAN40573.1 Candidatus Cloacimonas sp.
VanZ-2 ? ? PBP1A-like-peptidoglycan-GTase
NLW18135.1 Candidatus Cloacimonadota bacterium
VanZ-2 ? ? PBP1A-like-peptidoglycan-GTase
MBP7194630.1 Candidatus Cloacimonadota bacterium
VanZ-2 ? ? PBP1A-like-peptidoglycan-GTase
PKN79876.1 Candidatus Cloacimonetes bacterium HGW-Cloacimonetes-1
VanZ-2 ? ? PBP1A-like-peptidoglycan-GTase
PKN75709.1 Candidatus Cloacimonetes bacterium HGW-Cloacimonetes-2
VanZ-2 ? ? PBP1A-like-peptidoglycan-GTase
TDB37002.1 Actinomycetota bacterium
PBP1A-like-peptidoglycan-GTase ? ? VanZ-2
MBN2822028.1 Coriobacteriia bacterium
PBP1A-like-peptidoglycan-GTase ? ? VanZ-2
MBN2846869.1 Coriobacteriia bacterium
PBP1A-like-peptidoglycan-GTase ? VanZ-2
MBI3119750.1 Candidatus Hydrogenedentota bacterium
VanZ-2 PBP1A-like-peptidoglycan-GTase
NMD12744.1 Candidatus Cloacimonadota bacterium
VanZ-2 ? ? PBP1A-like-peptidoglycan-GTase
MCB5286284.1 Candidatus Cloacimonadota bacterium
VanZ-2 ? ? PBP1A-like-peptidoglycan-GTase
HEO71416.1 Candidatus Hydrogenedentota bacterium
VanZ-2 PBP1A-like-peptidoglycan-GTase
MCD8480595.1 Candidatus Cloacimonadota bacterium
VanZ-2 PSE ? monofunctional-peptidoglycan-glycosyltransferase PBP1A-like-peptidoglycan-GTase
MCB5270213.1 Candidatus Cloacimonadota bacterium
VanZ-2 ? ? PBP1A-like-peptidoglycan-GTase
MCB5266079.1 Candidatus Cloacimonadota bacterium
VanZ-2 ? ? PBP1A-like-peptidoglycan-GTase
MCB5267261.1 Candidatus Cloacimonadota bacterium
VanZ-2 ? ? PBP1A-like-peptidoglycan-GTase
MCB5248384.1 Candidatus Cloacimonadota bacterium
VanZ-2 ? ? PBP1A-like-peptidoglycan-GTase
MBP7116998.1 Candidatus Cloacimonadota bacterium
VanZ-2 ? ? PBP1A-like-peptidoglycan-GTase
HGX18943.1
VanZ-2 PBP1A-like-peptidoglycan-GTase
NLO44331.1 Candidatus Cloacimonadota bacterium
VanZ-2 ? ? PBP1A-like-peptidoglycan-GTase
RDJ06919.1 Rhizobium grahamii
VanZ-2 BiPBP_C PBP1A-like-peptidoglycan-GTase

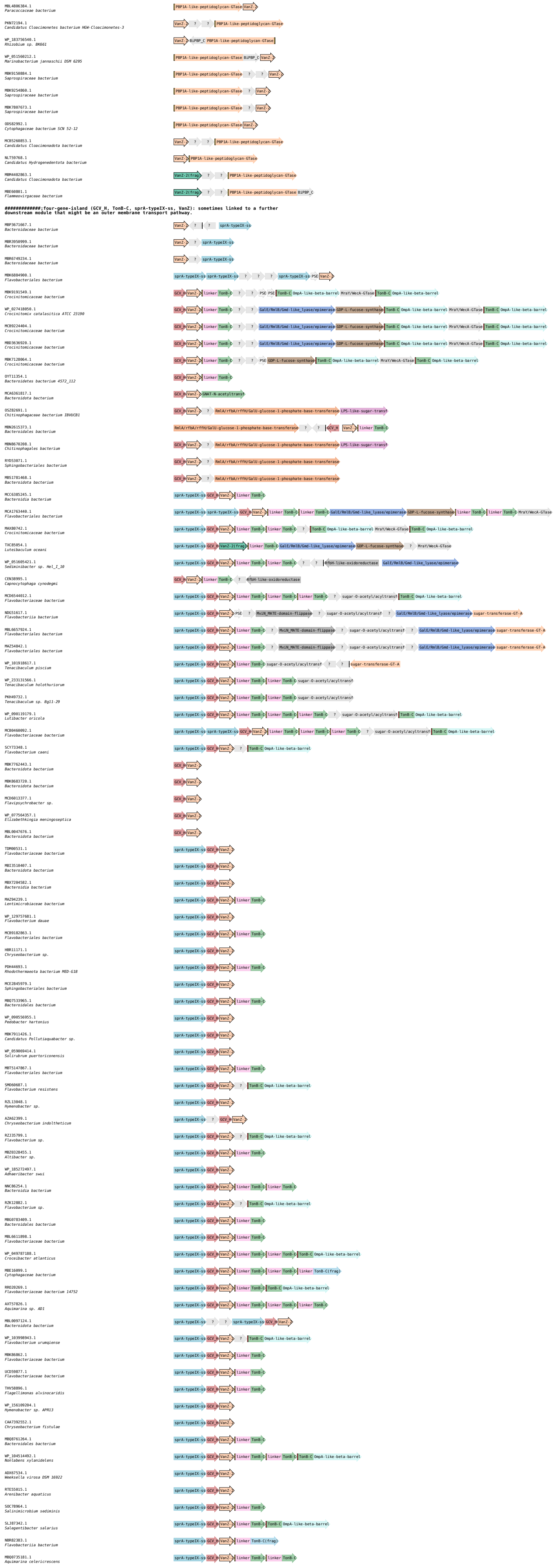
MBL4806384.1
Paracoccaceae bacterium
PBP1A-like-peptidoglycan-GTase VanZ-2
PKN72194.1
Candidatus Cloacimonetes bacterium HGW-Cloacimonetes-3
VanZ-2 ? ? PBP1A-like-peptidoglycan-GTase
WP_183756540.1
Rhizobium sp. BK661
VanZ-2 BiPBP_C PBP1A-like-peptidoglycan-GTase
WP_051560212.1
Marinobacterium jannaschii DSM 6295
PBP1A-like-peptidoglycan-GTase BiPBP_C VanZ-2
MBK9150884.1
Saprospiraceae bacterium
PBP1A-like-peptidoglycan-GTase ? ? VanZ-2
MBK9254860.1
Saprospiraceae bacterium
PBP1A-like-peptidoglycan-GTase ? VanZ-2
MBK7807673.1
Saprospiraceae bacterium
PBP1A-like-peptidoglycan-GTase ? VanZ-2
ODS82992.1
Cytophagaceae bacterium SCN 52-12
PBP1A-like-peptidoglycan-GTase VanZ-2
MCB5260853.1
Candidatus Cloacimonadota bacterium
VanZ-2 ? ? PBP1A-like-peptidoglycan-GTase
NLT59768.1
Candidatus Hydrogenedentota bacterium
VanZ-2 PBP1A-like-peptidoglycan-GTase
MBM4402863.1
Candidatus Cloacimonadota bacterium
VanZ-2(frag) ? ? PBP1A-like-peptidoglycan-GTase
MBE60801.1
Flammeovirgaceae bacterium
VanZ-2(frag) ? ? PBP1A-like-peptidoglycan-GTase BiPBP_C
#############;four-gene-island (GCV_H, TonB-C, sprA-typeIX-ss, VanZ): sometimes linked to a further downstream module that might be an outer membrane transport pathway.
MBP3671667.1
Bacteroidaceae bacterium
VanZ-2 ? ? sprA-typeIX-ss
MBR3950999.1
Bacteroidaceae bacterium
VanZ-2 ? sprA-typeIX-ss
MBR6749234.1
Bacteroidaceae bacterium
VanZ-2 ? sprA-typeIX-ss
MBK6884900.1
Flavobacteriales bacterium
sprA-typeIX-ss sprA-typeIX-ss ? ? ? sprA-typeIX-ss PSE VanZ-2
MBK9191549.1
Crocinitomicaceae bacterium
GCV_H VanZ-2 linker TonB-C ? ? PSE PSE TonB-C OmpA-like-beta-barrel MraY/WecA-GTase TonB-C OmpA-like-beta-barrel
WP_027418050.1
Crocinitomix catalasitica ATCC 23190
GCV_H VanZ-2 linker TonB-C ? ? GalE/RmlB/Gmd-like_lyase/epimerase GDP-L-fucose-synthase TonB-C OmpA-like-beta-barrel MraY/WecA-GTase TonB-C OmpA-like-beta-barrel
MCB9224404.1
Crocinitomicaceae bacterium
GCV_H VanZ-2 linker TonB-C ? ? GalE/RmlB/Gmd-like_lyase/epimerase GDP-L-fucose-synthase TonB-C OmpA-like-beta-barrel MraY/WecA-GTase TonB-C OmpA-like-beta-barrel
MBD3636920.1
Crocinitomicaceae bacterium
GCV_H VanZ-2 linker TonB-C ? ? GalE/RmlB/Gmd-like_lyase/epimerase GDP-L-fucose-synthase TonB-C OmpA-like-beta-barrel MraY/WecA-GTase TonB-C OmpA-like-beta-barrel
MBK7128064.1
Crocinitomicaceae bacterium
GCV_H VanZ-2 linker TonB-C ? ? PSE GDP-L-fucose-synthase TonB-C OmpA-like-beta-barrel MraY/WecA-GTase TonB-C OmpA-like-beta-barrel
OYT11354.1
Bacteroidetes bacterium 4572_112
GCV_H VanZ-2 linker TonB-C
MCA6361817.1
Bacteroidota bacterium
GCV_H VanZ-2 GNAT-N-acetyltransf
OSZ82691.1
Chitinophagaceae bacterium IBVUCB1
GCV_H VanZ-2 ? RmlA/rfbA/rffH/GalU-glucose-1-phosphate-base-transferase LPS-like-sugar-transf
MBN2615373.1
Bacteroidales bacterium
RmlA/rfbA/rffH/GalU-glucose-1-phosphate-base-transferase ? ? GCV_H VanZ-2 linker TonB-C
MBN8670208.1
Chitinophagales bacterium
GCV_H VanZ-2 ? RmlA/rfbA/rffH/GalU-glucose-1-phosphate-base-transferase LPS-like-sugar-transf
RYD53071.1
Sphingobacteriales bacterium
GCV_H VanZ-2 ? RmlA/rfbA/rffH/GalU-glucose-1-phosphate-base-transferase
MBS1781468.1
Bacteroidota bacterium
GCV_H VanZ-2 ? RmlA/rfbA/rffH/GalU-glucose-1-phosphate-base-transferase
MCC6385245.1
Bacteroidia bacterium
sprA-typeIX-ss GCV_H VanZ-2 linker TonB-C
MCA1763440.1
Flavobacteriales bacterium
sprA-typeIX-ss sprA-typeIX-ss GCV_H VanZ-2 linker TonB-C linker TonB-C GalE/RmlB/Gmd-like_lyase/epimerase GDP-L-fucose-synthase linker TonB-C linker TonB-C MraY/WecA-GTase
MAX80742.1
Crocinitomicaceae bacterium
sprA-typeIX-ss GCV_H VanZ-2 linker TonB-C linker TonB-C ? TonB-C OmpA-like-beta-barrel MraY/WecA-GTase TonB-C OmpA-like-beta-barrel
TXC85054.1
Luteibaculum oceani
sprA-typeIX-ss GCV_H VanZ-2(frag) linker TonB-C GalE/RmlB/Gmd-like_lyase/epimerase GDP-L-fucose-synthase ? MraY/WecA-GTase
WP_051605421.1
Sediminibacter sp. Hel_I_10
sprA-typeIX-ss GCV_H VanZ-2 linker TonB-C linker TonB-C ? ? RfbH-like-oxidoreductase GalE/RmlB/Gmd-like_lyase/epimerase
CEN38995.1
Capnocytophaga cynodegmi
GCV_H VanZ-2 linker TonB-C ? RfbH-like-oxidoreductase
MCD6544012.1
Flavobacteriaceae bacterium
sprA-typeIX-ss GCV_H VanZ-2 linker TonB-C linker TonB-C linker TonB-C ? sugar-O-acetyl/acyltransf TonB-C OmpA-like-beta-barrel
NDG51617.1
Flavobacteriia bacterium
sprA-typeIX-ss GCV_H VanZ-2 PSE ? MviN_MATE-domain-flippase ? sugar-O-acetyl/acyltransf ? GalE/RmlB/Gmd-like_lyase/epimerase sugar-transferase-GT-A
MBL6657924.1
Flavobacteriales bacterium
sprA-typeIX-ss GCV_H VanZ-2 linker TonB-C ? MviN_MATE-domain-flippase ? sugar-O-acetyl/acyltransf ? GalE/RmlB/Gmd-like_lyase/epimerase sugar-transferase-GT-A
MAZ54842.1
Flavobacteriales bacterium
sprA-typeIX-ss GCV_H VanZ-2 linker TonB-C ? MviN_MATE-domain-flippase ? sugar-O-acetyl/acyltransf ? GalE/RmlB/Gmd-like_lyase/epimerase sugar-transferase-GT-A
WP_101918617.1
Tenacibaculum piscium
sprA-typeIX-ss GCV_H VanZ-2 linker TonB-C sugar-O-acetyl/acyltransf ? ? sugar-transferase-GT-A
WP_233131566.1
Tenacibaculum holothuriorum
sprA-typeIX-ss GCV_H VanZ-2 linker TonB-C linker TonB-C sugar-O-acetyl/acyltransf
PKH49732.1
Tenacibaculum sp. Bg11-29
sprA-typeIX-ss GCV_H VanZ-2 linker TonB-C linker TonB-C sugar-O-acetyl/acyltransf
WP_090119179.1
Lutibacter oricola
sprA-typeIX-ss GCV_H VanZ-2 linker TonB-C linker TonB-C linker TonB-C ? sugar-O-acetyl/acyltransf TonB-C OmpA-like-beta-barrel
MCB0460092.1
Flavobacteriaceae bacterium
sprA-typeIX-ss sprA-typeIX-ss GCV_H VanZ-2 linker TonB-C linker TonB-C linker TonB-C ? sugar-O-acetyl/acyltransf TonB-C OmpA-like-beta-barrel
SCY73348.1
Flavobacterium caeni
sprA-typeIX-ss GCV_H VanZ-2 ? TonB-C OmpA-like-beta-barrel
MBK7762443.1
Bacteroidota bacterium
GCV_H VanZ-2
MBK8683720.1
Bacteroidota bacterium
GCV_H VanZ-2
MCD6013377.1
Flavipsychrobacter sp.
GCV_H VanZ-2
WP_077564357.1
Elizabethkingia meningoseptica
GCV_H VanZ-2
MBL0047676.1
Bacteroidota bacterium
GCV_H VanZ-2
TDM00531.1
Flavobacteriaceae bacterium
sprA-typeIX-ss GCV_H VanZ-2
MBI3510407.1
Bacteroidota bacterium
sprA-typeIX-ss GCV_H VanZ-2
MBX7204582.1
Bacteroidia bacterium
sprA-typeIX-ss GCV_H VanZ-2
MAZ94239.1
Lentimicrobiaceae bacterium
sprA-typeIX-ss GCV_H VanZ-2 linker TonB-C
WP_129757681.1
Flavobacterium dauae
sprA-typeIX-ss GCV_H VanZ-2
MCB9182863.1
Flavobacteriales bacterium
sprA-typeIX-ss GCV_H VanZ-2 linker TonB-C
HBR11171.1
Chryseobacterium sp.
sprA-typeIX-ss GCV_H VanZ-2
PDH44693.1
Rhodothermaeota bacterium MED-G18
sprA-typeIX-ss GCV_H VanZ-2 linker TonB-C
MCE2845979.1
Sphingobacteriales bacterium
sprA-typeIX-ss GCV_H VanZ-2
MBQ7533965.1
Bacteroidales bacterium
sprA-typeIX-ss GCV_H VanZ-2 linker TonB-C
WP_090556955.1
Pedobacter hartonius
sprA-typeIX-ss GCV_H VanZ-2
MBK7911426.1
Candidatus Pollutiaquabacter sp.
sprA-typeIX-ss GCV_H VanZ-2
WP_059069414.1
Solirubrum puertoriconensis
sprA-typeIX-ss GCV_H VanZ-2
MBT5147867.1
Flavobacteriales bacterium
sprA-typeIX-ss GCV_H VanZ-2 linker TonB-C
SMO60687.1
Flavobacterium resistens
sprA-typeIX-ss GCV_H VanZ-2 ? TonB-C OmpA-like-beta-barrel
RZL13048.1
Hymenobacter sp.
sprA-typeIX-ss GCV_H VanZ-2
AZA62399.1
Chryseobacterium indoltheticum
sprA-typeIX-ss ? GCV_H VanZ-2
RZJ35799.1
Flavobacterium sp.
sprA-typeIX-ss GCV_H VanZ-2 ? TonB-C OmpA-like-beta-barrel
MBZ0328455.1
Altibacter sp.
sprA-typeIX-ss GCV_H VanZ-2 linker TonB-C
WP_185272497.1
Adhaeribacter swui
sprA-typeIX-ss GCV_H VanZ-2
NNC86254.1
Bacteroidia bacterium
sprA-typeIX-ss GCV_H VanZ-2 linker TonB-C linker TonB-C
RZK12882.1
Flavobacterium sp.
sprA-typeIX-ss GCV_H VanZ-2 ? TonB-C OmpA-like-beta-barrel
MBG0783409.1
Bacteroidales bacterium
sprA-typeIX-ss GCV_H VanZ-2 linker TonB-C
MBL6611898.1
Flavobacteriaceae bacterium
sprA-typeIX-ss GCV_H VanZ-2 linker TonB-C
WP_049787188.1
Croceibacter atlanticus
sprA-typeIX-ss GCV_H VanZ-2 linker TonB-C linker TonB-C TonB-C OmpA-like-beta-barrel
MBE16099.1
Cytophagaceae bacterium
sprA-typeIX-ss GCV_H VanZ-2 linker TonB-C linker TonB-C linker TonB-C(frag)
RRO20269.1
Flavobacteriaceae bacterium 14752
sprA-typeIX-ss GCV_H VanZ-2 linker TonB-C TonB-C OmpA-like-beta-barrel
AXT57826.1
Aquimarina sp. AD1
sprA-typeIX-ss GCV_H VanZ-2 linker TonB-C linker TonB-C linker TonB-C
MBL0097124.1
Bacteroidota bacterium
sprA-typeIX-ss ? ? sprA-typeIX-ss GCV_H VanZ-2
WP_103998943.1
Flavobacterium urumqiense
sprA-typeIX-ss GCV_H VanZ-2 ? TonB-C OmpA-like-beta-barrel
MBK86862.1
Flavobacteriaceae bacterium
sprA-typeIX-ss GCV_H VanZ-2 linker TonB-C
UCD59877.1
Flavobacteriaceae bacterium
sprA-typeIX-ss GCV_H VanZ-2 linker TonB-C
THV58896.1
Flagellimonas alvinocaridis
sprA-typeIX-ss GCV_H VanZ-2 linker TonB-C
WP_156109204.1
Hymenobacter sp. APR13
sprA-typeIX-ss GCV_H VanZ-2
CAA7392552.1
Chryseobacterium fistulae
sprA-typeIX-ss GCV_H VanZ-2
MBQ8761264.1
Bacteroidales bacterium
sprA-typeIX-ss GCV_H VanZ-2 linker TonB-C
WP_104514492.1
Nonlabens xylanidelens
sprA-typeIX-ss GCV_H VanZ-2 linker TonB-C linker TonB-C TonB-C OmpA-like-beta-barrel
ADX67534.1
Weeksella virosa DSM 16922
sprA-typeIX-ss GCV_H VanZ-2
RTE55015.1
Arenibacter aquaticus
sprA-typeIX-ss GCV_H VanZ-2
SOC78964.1
Salinimicrobium sediminis
sprA-typeIX-ss GCV_H VanZ-2 linker TonB-C
SLJ87342.1
Salegentibacter salarius
sprA-typeIX-ss GCV_H VanZ-2 linker TonB-C TonB-C OmpA-like-beta-barrel
NBR82383.1
Flavobacteriia bacterium
sprA-typeIX-ss GCV_H VanZ-2 linker TonB-C(frag)
MBQ0735181.1
Aquimarina celericrescens
sprA-typeIX-ss GCV_H VanZ-2 linker TonB-C linker TonB-C

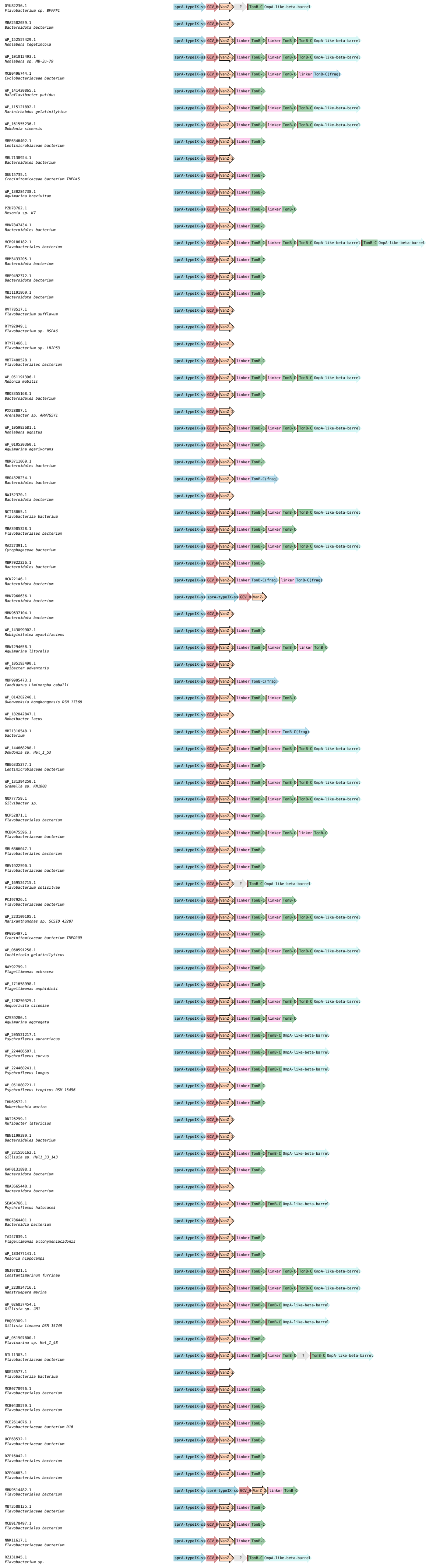
OYU82236.1
Flavobacterium sp. BFFFF1
sprA-typeIX-ss GCV_H VanZ ? TonB-C OmpA-like-beta-barrel
MBA2582039.1
Bacteroidota bacterium
sprA-typeIX-ss GCV_H VanZ
WP_152557429.1
Nonlabens tegetincola
sprA-typeIX-ss GCV_H VanZ linker TonB-C linker TonB-C TonB-C OmpA-like-beta-barrel
WP_101012493.1
Nonlabens sp. MB-3u-79
sprA-typeIX-ss GCV_H VanZ linker TonB-C linker TonB-C TonB-C OmpA-like-beta-barrel
MCB0496744.1
Cyclobacteriaceae bacterium
sprA-typeIX-ss GCV_H VanZ linker TonB-C linker TonB-C linker TonB-C(frag)
WP_141420865.1
Haloflavibacter putidus
sprA-typeIX-ss GCV_H VanZ linker TonB-C
WP_115121892.1
Marinirhabdus gelatinilytica
sprA-typeIX-ss GCV_H VanZ linker TonB-C linker TonB-C TonB-C OmpA-like-beta-barrel
WP_161555236.1
Dokdonia sinensis
sprA-typeIX-ss GCV_H VanZ linker TonB-C linker TonB-C TonB-C OmpA-like-beta-barrel
MBE6346402.1
Lentimicrobiaceae bacterium
sprA-typeIX-ss GCV_H VanZ linker TonB-C
MBL7138924.1
Bacteroidales bacterium
sprA-typeIX-ss GCV_H VanZ
OUU15735.1
Crocinitomicaceae bacterium TMED45
sprA-typeIX-ss GCV_H VanZ linker TonB-C
WP_130284738.1
Aquimarina brevivitae
sprA-typeIX-ss GCV_H VanZ linker TonB-C
PZD78762.1
Mesonia sp. K7
sprA-typeIX-ss GCV_H VanZ linker TonB-C linker TonB-C
MBW7847434.1
Bacteroidales bacterium
sprA-typeIX-ss GCV_H VanZ linker TonB-C
MCB9186182.1
Flavobacteriales bacterium
sprA-typeIX-ss GCV_H VanZ linker TonB-C linker TonB-C TonB-C OmpA-like-beta-barrel TonB-C OmpA-like-beta-barrel
MBM3433205.1
Bacteroidota bacterium
sprA-typeIX-ss GCV_H VanZ linker TonB-C
MBE9492372.1
Bacteroidota bacterium
sprA-typeIX-ss GCV_H VanZ linker TonB-C
MBI1191869.1
Bacteroidota bacterium
sprA-typeIX-ss GCV_H VanZ linker TonB-C
RVT78517.1
Flavobacterium sufflavum
sprA-typeIX-ss GCV_H VanZ
RTY92949.1
Flavobacterium sp. RSP46
sprA-typeIX-ss GCV_H VanZ
RTY71466.1
Flavobacterium sp. LB2P53
sprA-typeIX-ss GCV_H VanZ
MBT7488528.1
Flavobacteriales bacterium
sprA-typeIX-ss GCV_H VanZ linker TonB-C
WP_051191396.1
Mesonia mobilis
sprA-typeIX-ss GCV_H VanZ linker TonB-C linker TonB-C TonB-C OmpA-like-beta-barrel
MBQ3355168.1
Bacteroidales bacterium
sprA-typeIX-ss GCV_H VanZ linker TonB-C
PXX28887.1
Arenibacter sp. ARW7G5Y1
sprA-typeIX-ss GCV_H VanZ
WP_105983681.1
Nonlabens agnitus
sprA-typeIX-ss GCV_H VanZ linker TonB-C linker TonB-C TonB-C OmpA-like-beta-barrel
WP_010520360.1
Aquimarina agarivorans
sprA-typeIX-ss GCV_H VanZ linker TonB-C
MBR3711069.1
Bacteroidales bacterium
sprA-typeIX-ss GCV_H VanZ linker TonB-C
MBO4328234.1
Bacteroidales bacterium
sprA-typeIX-ss GCV_H VanZ linker TonB-C(frag)
NWJ52370.1
Bacteroidota bacterium
sprA-typeIX-ss GCV_H VanZ
NCT18065.1
Flavobacteriia bacterium
sprA-typeIX-ss GCV_H VanZ linker TonB-C linker TonB-C TonB-C OmpA-like-beta-barrel
MBA3985328.1
Flavobacteriales bacterium
sprA-typeIX-ss GCV_H VanZ linker TonB-C linker TonB-C
MAZ27391.1
Cytophagaceae bacterium
sprA-typeIX-ss GCV_H VanZ linker TonB-C linker TonB-C TonB-C OmpA-like-beta-barrel
MBR7022226.1
Bacteroidales bacterium
sprA-typeIX-ss GCV_H VanZ linker TonB-C
HCK22146.1
Bacteroidota bacterium
sprA-typeIX-ss GCV_H VanZ linker TonB-C(frag) linker TonB-C(frag)
MBK7966636.1
Bacteroidota bacterium
sprA-typeIX-ss sprA-typeIX-ss GCV_H VanZ
MBK9637104.1
Bacteroidota bacterium
sprA-typeIX-ss GCV_H VanZ
WP_143099902.1
Robiginitalea myxolifaciens
sprA-typeIX-ss GCV_H VanZ linker TonB-C
MBW1294658.1
Aquimarina litoralis
sprA-typeIX-ss GCV_H VanZ linker TonB-C linker TonB-C linker TonB-C
WP_105193490.1
Apibacter adventoris
sprA-typeIX-ss GCV_H VanZ
MBP9995473.1
Candidatus Limimorpha caballi
sprA-typeIX-ss GCV_H VanZ linker TonB-C(frag)
WP_014202246.1
Owenweeksia hongkongensis DSM 17368
sprA-typeIX-ss GCV_H VanZ linker TonB-C linker TonB-C
WP_182042847.1
Moheibacter lacus
sprA-typeIX-ss GCV_H VanZ
MBI1316548.1
bacterium
sprA-typeIX-ss GCV_H VanZ linker TonB-C linker TonB-C(frag)
WP_144668288.1
Dokdonia sp. Hel_I_53
sprA-typeIX-ss GCV_H VanZ linker TonB-C linker TonB-C TonB-C OmpA-like-beta-barrel
MBE6335277.1
Lentimicrobiaceae bacterium
sprA-typeIX-ss GCV_H VanZ linker TonB-C
WP_131394250.1
Gramella sp. KN1008
sprA-typeIX-ss GCV_H VanZ linker TonB-C linker TonB-C TonB-C OmpA-like-beta-barrel
NQX77759.1
Gilvibacter sp.
sprA-typeIX-ss GCV_H VanZ linker TonB-C linker TonB-C TonB-C OmpA-like-beta-barrel
NCP52871.1
Flavobacteriales bacterium
sprA-typeIX-ss GCV_H VanZ linker TonB-C
MCB0475596.1
Flavobacteriaceae bacterium
sprA-typeIX-ss GCV_H VanZ linker TonB-C linker TonB-C linker TonB-C
MBL6866047.1
Flavobacteriales bacterium
sprA-typeIX-ss GCV_H VanZ linker TonB-C
MBV1922590.1
Flavobacteriaceae bacterium
sprA-typeIX-ss GCV_H VanZ linker TonB-C
WP_169524715.1
Flavobacterium solisilvae
sprA-typeIX-ss GCV_H VanZ ? TonB-C OmpA-like-beta-barrel
PCJ97926.1
Flavobacteriaceae bacterium
sprA-typeIX-ss GCV_H VanZ linker TonB-C linker TonB-C
WP_223109105.1
Marixanthomonas sp. SCSIO 43207
sprA-typeIX-ss GCV_H VanZ linker TonB-C linker TonB-C TonB-C OmpA-like-beta-barrel
RPG86497.1
Crocinitomicaceae bacterium TMED209
sprA-typeIX-ss GCV_H VanZ linker TonB-C
WP_068591258.1
Cochleicola gelatinilyticus
sprA-typeIX-ss GCV_H VanZ linker TonB-C linker TonB-C TonB-C OmpA-like-beta-barrel
NAY92799.1
Flagellimonas ochracea
sprA-typeIX-ss GCV_H VanZ linker TonB-C
WP_171658998.1
Flagellimonas amphidinii
sprA-typeIX-ss GCV_H VanZ linker TonB-C
WP_128250325.1
Aequorivita ciconiae
sprA-typeIX-ss GCV_H VanZ linker TonB-C linker TonB-C TonB-C OmpA-like-beta-barrel
KZS39286.1
Aquimarina aggregata
sprA-typeIX-ss GCV_H VanZ linker TonB-C linker TonB-C
WP_205521217.1
Psychroflexus aurantiacus
sprA-typeIX-ss GCV_H VanZ linker TonB-C TonB-C OmpA-like-beta-barrel
WP_224486507.1
Psychroflexus curvus
sprA-typeIX-ss GCV_H VanZ linker TonB-C TonB-C OmpA-like-beta-barrel
WP_224460241.1
Psychroflexus longus
sprA-typeIX-ss GCV_H VanZ linker TonB-C TonB-C OmpA-like-beta-barrel
WP_051080721.1
Psychroflexus tropicus DSM 15496
sprA-typeIX-ss GCV_H VanZ linker TonB-C
THD69572.1
Robertkochia marina
sprA-typeIX-ss GCV_H VanZ linker TonB-C
RNI26299.1
Rufibacter latericius
sprA-typeIX-ss GCV_H VanZ
MBN1199389.1
Bacteroidales bacterium
sprA-typeIX-ss GCV_H VanZ
WP_231556162.1
Gillisia sp. Hel1_33_143
sprA-typeIX-ss GCV_H VanZ linker TonB-C TonB-C OmpA-like-beta-barrel
KAF0131898.1
Bacteroidota bacterium
sprA-typeIX-ss GCV_H VanZ linker TonB-C
MBA3665440.1
Bacteroidota bacterium
sprA-typeIX-ss GCV_H VanZ
SEA64766.1
Psychroflexus halocasei
sprA-typeIX-ss GCV_H VanZ linker TonB-C TonB-C OmpA-like-beta-barrel
MBC7864401.1
Bacteroidia bacterium
sprA-typeIX-ss GCV_H VanZ
TAI47039.1
Flagellimonas allohymeniacidonis
sprA-typeIX-ss GCV_H VanZ linker TonB-C
WP_183477141.1
Mesonia hippocampi
sprA-typeIX-ss GCV_H VanZ linker TonB-C
QNJ97821.1
Constantimarinum furrinae
sprA-typeIX-ss GCV_H VanZ linker TonB-C linker TonB-C TonB-C OmpA-like-beta-barrel
WP_223034716.1
Hanstruepera marina
sprA-typeIX-ss GCV_H VanZ linker TonB-C linker TonB-C TonB-C OmpA-like-beta-barrel
WP_026837454.1
Gillisia sp. JM1
sprA-typeIX-ss GCV_H VanZ linker TonB-C TonB-C OmpA-like-beta-barrel
EHQ03309.1
Gillisia limnaea DSM 15749
sprA-typeIX-ss GCV_H VanZ linker TonB-C TonB-C OmpA-like-beta-barrel
WP_051907800.1
Flavimarina sp. Hel_I_48
sprA-typeIX-ss GCV_H VanZ linker TonB-C
RTL11303.1
Flavobacteriaceae bacterium
sprA-typeIX-ss GCV_H VanZ linker TonB-C linker TonB-C ? TonB-C OmpA-like-beta-barrel
NDE28577.1
Flavobacteriia bacterium
sprA-typeIX-ss GCV_H VanZ
MCB0770976.1
Flavobacteriales bacterium
sprA-typeIX-ss GCV_H VanZ linker TonB-C
MCB0430579.1
Flavobacteriales bacterium
sprA-typeIX-ss GCV_H VanZ linker TonB-C
MCE2614076.1
Flavobacteriaceae bacterium D16
sprA-typeIX-ss GCV_H VanZ linker TonB-C
UCE68532.1
Flavobacteriaceae bacterium
sprA-typeIX-ss GCV_H VanZ linker TonB-C
RZP16042.1
Flavobacteriales bacterium
sprA-typeIX-ss GCV_H VanZ linker TonB-C
RZP04683.1
Flavobacteriales bacterium
sprA-typeIX-ss GCV_H VanZ linker TonB-C
MBK9514482.1
Flavobacteriales bacterium
sprA-typeIX-ss sprA-typeIX-ss GCV_H VanZ linker TonB-C
MBT3588125.1
Flavobacteriaceae bacterium
sprA-typeIX-ss GCV_H VanZ linker TonB-C
MCB9170497.1
Flavobacteriales bacterium
sprA-typeIX-ss GCV_H VanZ linker TonB-C
NNK11617.1
Flavobacteriaceae bacterium
sprA-typeIX-ss GCV_H VanZ linker TonB-C
RZJ31045.1
Flavobacterium sp.
sprA-typeIX-ss GCV_H VanZ ? TonB-C OmpA-like-beta-barrel

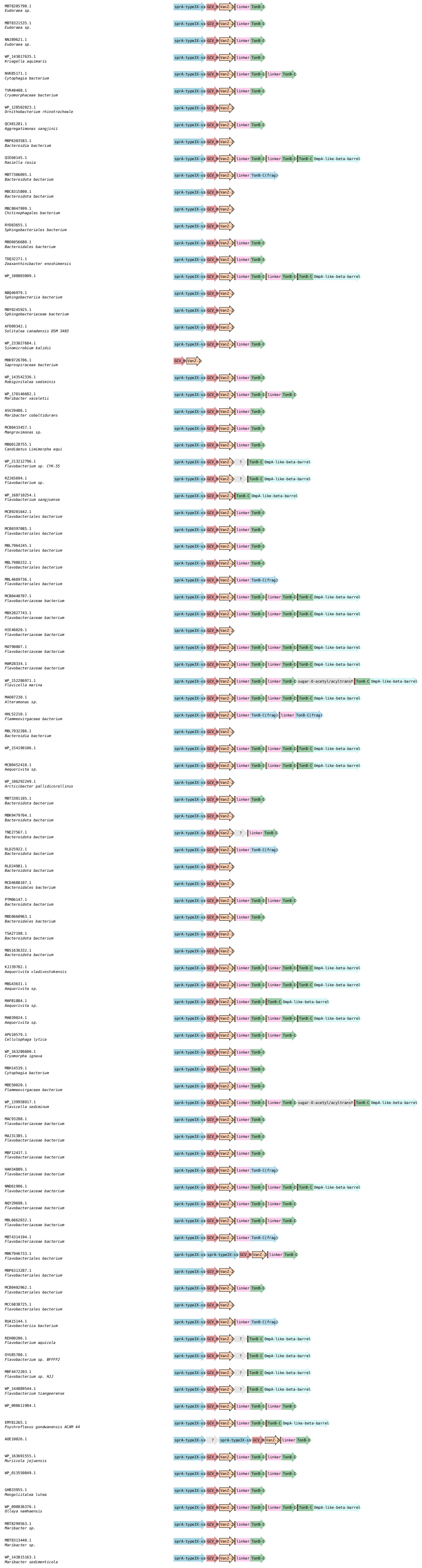
MBT8205790.1
Eudoraea sp.
MBT8321535.1
Eudoraea sp.
NNJ89621.1
Eudoraea sp.
WP_143017635.1
Kriegella aquimaris
NVK85171.1
Cytophagia bacterium
TVR40408.1
Cryomorphaceae bacterium
WP_128502023.1
Ornithobacterium rhinotracheale
QCX01201.1
Aggregatimonas sangjinii
MBP6303583.1
Bacteroidia bacterium
QIE60145.1
Rasiella rasia
MBT7306095.1
Bacteroidota bacterium
MBC8315800.1
Bacteroidota bacterium
MBC8047999.1
Chitinophagales bacterium
RYD83655.1
Sphingobacteriales bacterium
MBO6056680.1
Bacteroidales bacterium
TDQ32271.1
Zeaxanthinibacter enoshimensis
WP_100803009.1
NBQ46979.1
Sphingobacteriia bacterium
MBY0245925.1
Sphingobacteriaceae bacterium
AFD09342.1
Solitalea canadensis DSM 3403
WP_233027684.1
Sinomicrobium kalidii
MBK9726706.1
Saprospiraceae bacterium
WP_143542336.1
Robiginitalea sediminis
WP_170146682.1
Maribacter vaceletii
ASV29486.1
Maribacter cobaltidurans
MCB0433457.1
Mangrovimonas sp.
MBQ0128755.1
Candidatus Limimorpha equi
WP_213212796.1
Flavobacterium sp. CYK-55
RZJ65694.1
Flavobacterium sp.
WP_168710254.1
Flavobacterium sangjuense
MCB9201642.1
Flavobacteriales bacterium
MCB0397085.1
Flavobacteriales bacterium
MBL7964245.1
Flavobacteriales bacterium
MBL7980232.1
Flavobacteriales bacterium
MBL4669736.1
Flavobacteriales bacterium
MCB0440707.1
Flavobacteriaceae bacterium
MBX2827743.1
Flavobacteriaceae bacterium
HIE46020.1
Flavobacteriaceae bacterium
MAT90007.1
Flavobacteriaceae bacterium
MAM28334.1
Flavobacteriaceae bacterium
WP_152286971.1
Flavicella marina
MAO07230.1
Alteromonas sp.
HHL52210.1
Flammeovirgaceae bacterium
MBL7932286.1
Bacteroidia bacterium
WP_154190106.1
MCB0452410.1
Aequorivita sp.
WP_106292249.1
Arcticibacter pallidicorallinus
MBT3301105.1
Bacteroidota bacterium
MBK9479704.1
Bacteroidota bacterium
TNE27567.1
Bacteroidota bacterium
RLD25922.1
Bacteroidota bacterium
RLD24981.1
Bacteroidota bacterium
MCD4680107.1
Bacteroidales bacterium
PTM06147.1
Bacteroidota bacterium
MBE0660963.1
Bacteroidales bacterium
TSA27198.1
Bacteroidota bacterium
MBS1636332.1
Bacteroidota bacterium
KJJ39782.1
Aequorivita vladivostokensis
MBG43631.1
Aequorivita sp.
MAP81884.1
Aequorivita sp.
MAB39024.1
Aequorivita sp.
APU10579.1
Cellulophaga lytica
WP_163286600.1
Cryomorpha ignava
MBH14539.1
Cytophagia bacterium
MBE50020.1
Flammeovirgaceae bacterium
WP_139958917.1
Flavicella sediminum
MAC93288.1
Flavobacteriaceae bacterium
MAJ31385.1
Flavobacteriaceae bacterium
MBF12437.1
Flavobacteriaceae bacterium
HAH34889.1
Flavobacteriaceae bacterium
NND61906.1
Flavobacteriaceae bacterium
NQY29698.1
Flavobacteriaceae bacterium
MBL6662032.1
Flavobacteriaceae bacterium
MBT4314194.1
Flavobacteriaceae bacterium
MBK7946733.1
Flavobacteriales bacterium
MBP6313287.1
Flavobacteriales bacterium
MCB0402962.1
Flavobacteriales bacterium
MCC6838725.1
Flavobacteriales bacterium
RUA15144.1
Flavobacteriia bacterium
REH00206.1
Flavobacterium aquicola
OYU85700.1
Flavobacterium sp. BFFFF2
MBF4472203.1
Flavobacterium sp. HJJ
WP_144889544.1
Flavobacterium tiangeerense
WP_008611984.1
EMY81265.1
Psychroflexus gondwanensis ACAM 44
AOE10026.1
WP_163691555.1
Muriicola jejuensis
WP_013550849.1
GHB33955.1
Mongoliitalea lutea
WP_090836376.1
Olleya namhaensis
MBT8299563.1
Maribacter sp.
MBT8313440.1
Maribacter sp.
WP_143815163.1
Maribacter sedimenticola

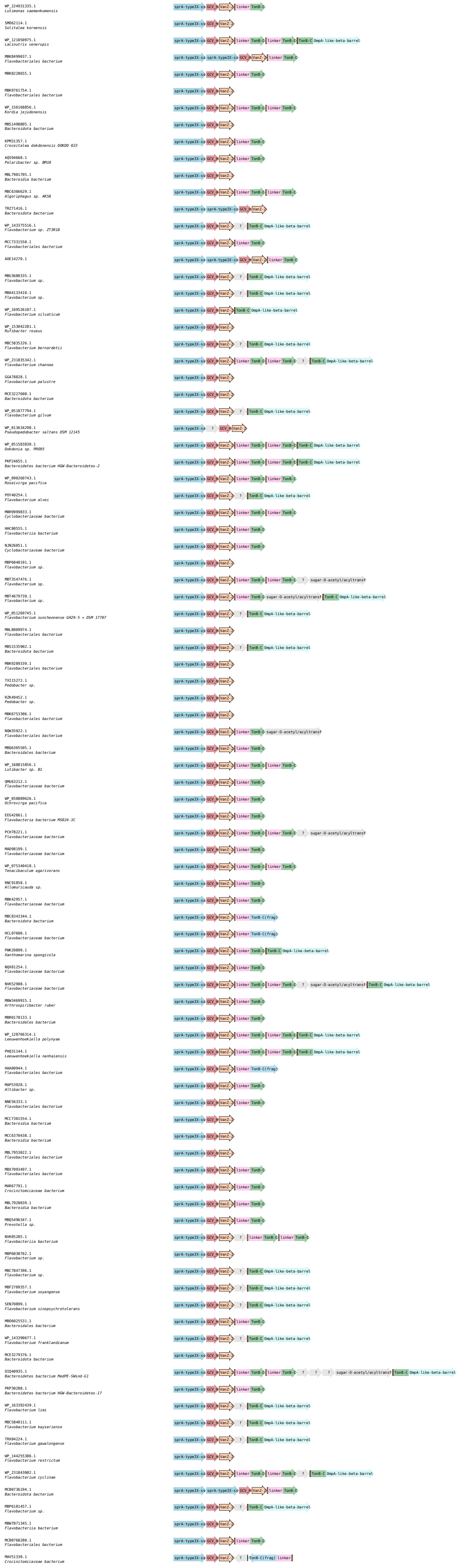

WP_224931335.1 Lutimonas saemankumensis
sprA-typeIX-ss GCV_H VanZ linker TonB-C
SMD62114.1 Solitalea koreensis
sprA-typeIX-ss GCV_H VanZ
WP_121050975.1 Lacinutrix venerupis
sprA-typeIX-ss GCV_H VanZ linker TonB-C linker TonB-C TonB-C OmpA-like-beta-barrel
MBK8499037.1 Flavobacteriales bacterium
sprA-typeIX-ss sprA-typeIX-ss GCV_H VanZ linker TonB-C
MBK8228655.1
sprA-typeIX-ss GCV_H VanZ linker TonB-C
MBK9761754.1 Flavobacteriales bacterium
sprA-typeIX-ss GCV_H VanZ
WP_156168856.1 Kordia jejudonensis
sprA-typeIX-ss GCV_H VanZ linker TonB-C linker TonB-C
MBS1498805.1 Bacteroidota bacterium
sprA-typeIX-ss GCV_H VanZ
KPM31357.1 Croceitalea dokdonensis DOKDO 023
sprA-typeIX-ss GCV_H VanZ linker TonB-C
AOS94660.1 Polaribacter sp. BM10
sprA-typeIX-ss GCV_H VanZ linker TonB-C
MBL7901705.1 Bacteroidia bacterium
sprA-typeIX-ss GCV_H VanZ
MBC6366629.1 Algoriphagus sp. AK58
sprA-typeIX-ss GCV_H VanZ linker TonB-C linker TonB-C
TRZ71416.1 Bacteroidota bacterium
sprA-typeIX-ss sprA-typeIX-ss GCV_H VanZ
WP_143375516.1 Flavobacterium sp. ZT3R18
sprA-typeIX-ss GCV_H VanZ ? TonB-C OmpA-like-beta-barrel
MCC7331550.1 Flavobacteriales bacterium
sprA-typeIX-ss GCV_H VanZ linker TonB-C
AOE14270.1
sprA-typeIX-ss sprA-typeIX-ss GCV_H VanZ linker TonB-C
MBU3680335.1 Flavobacterium sp.
sprA-typeIX-ss GCV_H VanZ ? TonB-C OmpA-like-beta-barrel
MBA4133410.1 Flavobacterium sp.
sprA-typeIX-ss GCV_H VanZ ? TonB-C OmpA-like-beta-barrel
WP_169526107.1 Flavobacterium silvaticum
sprA-typeIX-ss GCV_H VanZ TonB-C OmpA-like-beta-barrel
WP_153042281.1 Rufibacter roseus
sprA-typeIX-ss GCV_H VanZ
MBC5835226.1 Flavobacterium bernardetii
sprA-typeIX-ss GCV_H VanZ ? TonB-C OmpA-like-beta-barrel
WP_231835342.1 Flavobacterium channae
sprA-typeIX-ss GCV_H VanZ linker TonB-C linker TonB-C ? TonB-C OmpA-like-beta-barrel
GGA78828.1 Flavobacterium palustre
sprA-typeIX-ss GCV_H VanZ
MCE3227608.1 Bacteroidota bacterium
sprA-typeIX-ss GCV_H VanZ
WP_051877794.1 Flavobacterium gilvum
sprA-typeIX-ss GCV_H VanZ ? TonB-C OmpA-like-beta-barrel
WP_013634298.1 Pseudopedobacter saltans DSM 12145
sprA-typeIX-ss ? GCV_H VanZ
WP_051583830.1 Dokdonia sp. PRO95
sprA-typeIX-ss GCV_H VanZ linker TonB-C linker TonB-C TonB-C OmpA-like-beta-barrel
PKP24655.1 Bacteroidetes bacterium HGW-Bacteroidetes-2
sprA-typeIX-ss GCV_H VanZ linker TonB-C linker TonB-C TonB-C OmpA-like-beta-barrel
WP_090260743.1 Roseivirga pacifica
sprA-typeIX-ss GCV_H VanZ linker TonB-C linker TonB-C
POY40254.1 Flavobacterium alvei
sprA-typeIX-ss GCV_H VanZ ? TonB-C OmpA-like-beta-barrel
MBR9999833.1 Cyclobacteriaceae bacterium
sprA-typeIX-ss GCV_H VanZ linker TonB-C linker TonB-C
HHC80555.1 Flavobacteriia bacterium
sprA-typeIX-ss GCV_H VanZ linker TonB-C
NJN26051.1 Cyclobacteriaceae bacterium
sprA-typeIX-ss GCV_H VanZ linker TonB-C
MBP6040191.1 Flavobacterium sp.
sprA-typeIX-ss GCV_H VanZ
MBT3547476.1 Flavobacterium sp.
sprA-typeIX-ss GCV_H VanZ linker TonB-C linker TonB-C ? sugar-O-acetyl/acyltransf
MBT4679739.1 Flavobacterium sp.
sprA-typeIX-ss GCV_H VanZ linker TonB-C sugar-O-acetyl/acyltransf TonB-C OmpA-like-beta-barrel
WP_052269745.1 Flavobacterium suncheonense GH29-5 = DSM 17707
sprA-typeIX-ss GCV_H VanZ ? TonB-C OmpA-like-beta-barrel
MBL8009974.1 Flavobacteriales bacterium
sprA-typeIX-ss GCV_H VanZ
MBS1535902.1 Bacteroidota bacterium
sprA-typeIX-ss GCV_H VanZ ? TonB-C OmpA-like-beta-barrel
MBK9289339.1 Flavobacteriales bacterium
sprA-typeIX-ss GCV_H VanZ
TXI15272.1 Pedobacter sp.
sprA-typeIX-ss GCV_H VanZ
RZK49452.1 Pedobacter sp.
sprA-typeIX-ss GCV_H VanZ
MBK6753306.1 Flavobacteriales bacterium
sprA-typeIX-ss GCV_H VanZ
NQW35922.1 Flavobacteriales bacterium
sprA-typeIX-ss GCV_H VanZ linker TonB-C sugar-O-acetyl/acyltransf
MBQ6305505.1 Bacteroidales bacterium
sprA-typeIX-ss GCV_H VanZ linker TonB-C
WP_168815856.1 Lutibacter sp. B1
sprA-typeIX-ss GCV_H VanZ linker TonB-C linker TonB-C
OMJ63212.1 Flavobacteriaceae bacterium
sprA-typeIX-ss GCV_H VanZ linker TonB-C
WP_050809626.1 Ochrovirga pacifica
sprA-typeIX-ss GCV_H VanZ linker TonB-C
EEG42661.1 Flavobacteria bacterium MS024-3C
sprA-typeIX-ss GCV_H VanZ linker TonB-C
PCH78221.1 Flavobacteriaceae bacterium
sprA-typeIX-ss GCV_H VanZ linker TonB-C linker TonB-C ? sugar-O-acetyl/acyltransf
MAD98199.1 Flavobacteriaceae bacterium
sprA-typeIX-ss GCV_H VanZ linker TonB-C
WP_075340418.1 Tenacibaculum agarivorans
sprA-typeIX-ss GCV_H VanZ linker TonB-C linker TonB-C
RNC91858.1 Allomuricauda sp.
sprA-typeIX-ss GCV_H VanZ linker TonB-C
MBK42957.1 Flavobacteriaceae bacterium
sprA-typeIX-ss GCV_H VanZ linker TonB-C
MBC8343344.1 Bacteroidota bacterium
sprA-typeIX-ss GCV_H VanZ linker TonB-C(frag)
HCL07600.1 Flavobacteriaceae bacterium
sprA-typeIX-ss GCV_H VanZ linker TonB-C(frag)
PWK20899.1 Xanthomarina spongicola
sprA-typeIX-ss GCV_H VanZ linker TonB-C TonB-C OmpA-like-beta-barrel
NOX81254.1 Flavobacteriaceae bacterium
sprA-typeIX-ss GCV_H VanZ linker TonB-C
NVK52908.1 Flavobacteriaceae bacterium
sprA-typeIX-ss GCV_H VanZ linker TonB-C linker TonB-C ? sugar-O-acetyl/acyltransf TonB-C OmpA-like-beta-barrel
MBW3469915.1 Arthrospiribacter ruber
sprA-typeIX-ss GCV_H VanZ linker TonB-C
MBR0178133.1 Bacteroidales bacterium
sprA-typeIX-ss GCV_H VanZ linker TonB-C
WP_128766314.1 Leeuwenhoekiella polynyae
sprA-typeIX-ss GCV_H VanZ linker TonB-C linker TonB-C TonB-C OmpA-like-beta-barrel
PHQ31144.1 Leeuwenhoekiella nanhaiensis
sprA-typeIX-ss GCV_H VanZ linker TonB-C linker TonB-C TonB-C OmpA-like-beta-barrel
HAA00944.1 Flavobacteriales bacterium
sprA-typeIX-ss GCV_H VanZ linker TonB-C(frag)
MAP53928.1 Altibacter sp.
sprA-typeIX-ss GCV_H VanZ linker TonB-C
NNE56333.1 Flavobacteriales bacterium
sprA-typeIX-ss GCV_H VanZ linker TonB-C
MCC7301554.1 Bacteroidia bacterium
sprA-typeIX-ss GCV_H VanZ
MCC6370438.1 Bacteroidia bacterium
sprA-typeIX-ss GCV_H VanZ
MBL7953022.1 Flavobacteriales bacterium
sprA-typeIX-ss GCV_H VanZ
MBX7093497.1 Flavobacteriales bacterium
sprA-typeIX-ss GCV_H VanZ linker TonB-C
MAR67791.1 Crocinitomicaceae bacterium
sprA-typeIX-ss GCV_H VanZ linker TonB-C
MBL7928839.1 Bacteroidia bacterium
sprA-typeIX-ss GCV_H VanZ linker TonB-C
MBQ5496347.1 Prevotella sp.
sprA-typeIX-ss GCV_H VanZ linker TonB-C
NVK05285.1 Flavobacteriia bacterium
sprA-typeIX-ss GCV_H VanZ ? linker TonB-C linker TonB-C
MBP6038762.1 Flavobacterium sp.
sprA-typeIX-ss GCV_H VanZ
MBC7847396.1 Flavobacterium sp.
sprA-typeIX-ss GCV_H VanZ ? TonB-C OmpA-like-beta-barrel
MBP2709357.1 Flavobacterium soyangense
sprA-typeIX-ss GCV_H VanZ ? TonB-C OmpA-like-beta-barrel
SEN70899.1 Flavobacterium sinopsychrotolerans
sprA-typeIX-ss GCV_H VanZ ? TonB-C OmpA-like-beta-barrel
MBO6025531.1 Bacteroidales bacterium
sprA-typeIX-ss GCV_H VanZ linker TonB-C
WP_143390677.1 Flavobacterium franklandianum
sprA-typeIX-ss GCV_H VanZ ? TonB-C OmpA-like-beta-barrel
MCE3279376.1 Bacteroidota bacterium
sprA-typeIX-ss GCV_H VanZ
OIQ40935.1 Bacteroidetes bacterium MedPE-SWsnd-G1
sprA-typeIX-ss GCV_H VanZ linker TonB-C linker TonB-C ? ? ? sugar-O-acetyl/acyltransf TonB-C OmpA-like-beta-barrel
PKP30288.1 Bacteroidetes bacterium HGW-Bacteroidetes-17
sprA-typeIX-ss GCV_H VanZ linker TonB-C
WP_163392439.1 Flavobacterium limi
sprA-typeIX-ss GCV_H VanZ ? TonB-C OmpA-like-beta-barrel
MBC5840111.1 Flavobacterium kayseriense
sprA-typeIX-ss GCV_H VanZ ? TonB-C OmpA-like-beta-barrel
TRX04224.1 Flavobacterium gawalongense
sprA-typeIX-ss GCV_H VanZ ? TonB-C OmpA-like-beta-barrel
WP_144255386.1 Flavobacterium restrictum
sprA-typeIX-ss GCV_H VanZ
WP_231843002.1 Flavobacterium cyclinae
sprA-typeIX-ss GCV_H VanZ linker TonB-C linker TonB-C ? TonB-C OmpA-like-beta-barrel
MCB0736194.1 Bacteroidota bacterium
sprA-typeIX-ss sprA-typeIX-ss GCV_H VanZ linker TonB-C
MBP6181457.1 Flavobacterium sp.
sprA-typeIX-ss GCV_H VanZ ? TonB-C OmpA-like-beta-barrel
MBW7871345.1 Flavobacteriia bacterium
sprA-typeIX-ss GCV_H VanZ
MCB0760280.1 Flavobacteriales bacterium
sprA-typeIX-ss GCV_H VanZ linker TonB-C
MAV51330.1 Crocinitomicaceae bacterium
sprA-typeIX-ss GCV_H VanZ ? TonB-C(frag) linker

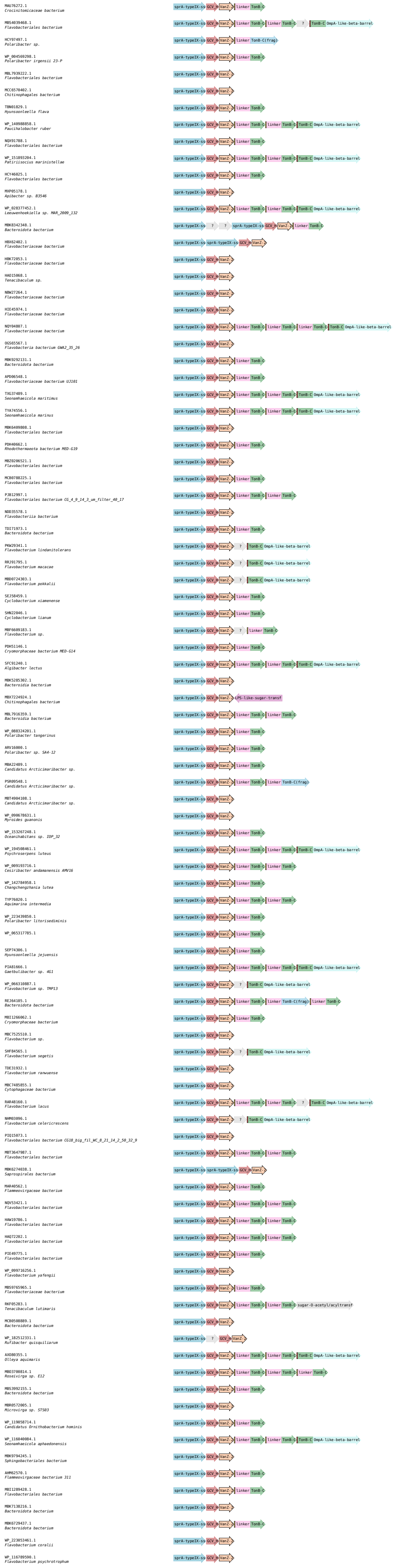
MAU76272.1
Crocinitomicaceae bacterium
MBS4039468.1
Flavobacteriales bacterium
HCY97497.1
Polaribacter sp.
WP_004569298.1
Polaribacter irgensii 23-P
MBL7939222.1
Flavobacteriales bacterium
MCC6570402.1
Chitinophagales bacterium
TBN01829.1
Hyunsoonleella flava
WP_140988858.1
Paucihalobacter ruber
NQX91788.1
Flavobacteriales bacterium
WP_151893204.1
Patiriisocius marinistellae
HCY46025.1
Flavobacteriales bacterium
MXP05178.1
Apibacter sp. B3546
WP_028377452.1
Leeuwenhoekiella sp. MAR_2009_132
MBK8342348.1
Bacteroidota bacterium
HBX62402.1
Flavobacteriaceae bacterium
HBK72053.1
Flavobacteriaceae bacterium
HAO15068.1
Tenacibaculum sp.
NBW27264.1
Flavobacteriaceae bacterium
HIE45974.1
Flavobacteriaceae bacterium
NQY04807.1
Flavobacteriaceae bacterium
OGS65567.1
Flavobacteria bacterium GWA2_35_26
MBK9292131.1
Bacteroidota bacterium
APD06548.1
Flavobacteriaceae bacterium UJ101
TXG37489.1
Seonamhaeicola maritimus
TYA74556.1
Seonamhaeicola marinus
MBK6409808.1
Flavobacteriales bacterium
PDH40662.1
Rhodothermaeota bacterium MED-G19
MBZ0206521.1
Flavobacteriales bacterium
MCB0788225.1
Flavobacteriales bacterium
PJB12997.1
Flavobacteriales bacterium CG_4_9_14_3_um_filter_40_17
NDD35578.1
Flavobacteriia bacterium
TDI71973.1
Bacteroidota bacterium
PKW29341.1
Flavobacterium lindanitolerans
RRJ91795.1
Flavobacterium macacae
MBD0724303.1
Flavobacterium pokkalii
SEJ58459.1
Cyclobacterium xiamenense
SHN22046.1
Cyclobacterium lianum
MBF6609183.1
Flavobacterium sp.
PDH51146.1
Cryomorphaceae bacterium MED-G14
SFC91240.1
Algibacter lectus
MBK5285302.1
Bacteroidia bacterium
MBX7224924.1
Chitinophagales bacterium
MBL7916359.1
Bacteroidia bacterium
WP_088324201.1
Polaribacter tangerinus
ARV16000.1
Polaribacter sp. SA4-12
MBA22489.1
Candidatus Arcticimaribacter sp.
PSR09548.1
Candidatus Arcticimaribacter sp.
MBT4904108.1
Candidatus Arcticimaribacter sp.
WP_090678631.1
Myroides guanonis
WP_153267248.1
Oceanihabitans sp. IOP_32
WP_194508461.1
Psychroserpens luteus
WP_009193716.1
Cesiribacter andamanensis AMV16
WP_142784958.1
Changchengzhania lutea
TYP76020.1
Aquimarina intermedia
WP_223439850.1
Polaribacter litorisediminis
WP_065317785.1
SEP74306.1
Hyunsoonleella jejuensis
PIA81666.1
Gaetbulibacter sp. 4G1
WP_066310887.1
Flavobacterium sp. TMP13
REJ64105.1
Bacteroidota bacterium
MBI1266062.1
Cryomorphaceae bacterium
MBC7525510.1
Flavobacterium sp.
SHF84565.1
Flavobacterium segetis
TDE31932.1
Flavobacterium ranwuense
MBC7485855.1
Cytophagaceae bacterium
RAR48160.1
Flavobacterium lacus
NHM03096.1
Flavobacterium celericrescens
PIQ15073.1
Flavobacteriales bacterium CG18_big_fil_WC_8_21_14_2_50_32_9
MBT3647987.1
Flavobacteriales bacterium
MBK6274038.1
Saprospirales bacterium
MAR40562.1
Flammeovirgaceae bacterium
NQV53421.1
Flavobacteriales bacterium
HAW19786.1
Flavobacteriales bacterium
HAQ72202.1
Flavobacteriales bacterium
PIE49775.1
Flavobacteriales bacterium
WP_099716256.1
Flavobacterium yafengii
MBS9765965.1
Flavobacteriaceae bacterium
RKF05283.1
Tenacibaculum lutimaris
MCB0508889.1
Bacteroidota bacterium
WP_182512331.1
Rufibacter quisquiliarum
AXO80355.1
Olleya aquimaris
MBO3700814.1
Roseivirga sp. E12
MBS3992155.1
Bacteroidota bacterium
MBR0572005.1
Microvirga sp. STS03
WP_119058714.1
Candidatus Ornithobacterium hominis
WP_116040084.1
Seonamhaeicola aphaedonensis
MBK9794245.1
Sphingobacteriales bacterium
AHM62570.1
Flammeovirgaceae bacterium 311
MBI1289428.1
Flavobacteriales bacterium
MBK7138216.1
Bacteroidota bacterium
MBK6729437.1
Bacteroidota bacterium
WP_223053461.1
Flavobacterium coralii
WP_116789590.1
Flavobacterium psychrotrophum
sprA-typeIX-ss
GCV_H
VanZ
linker
TonB-C
TonB-C(frag)
OmpA-like-beta-barrel
LPS-like-sugar-transf
sugar-O-acetyl/acyltransf
?

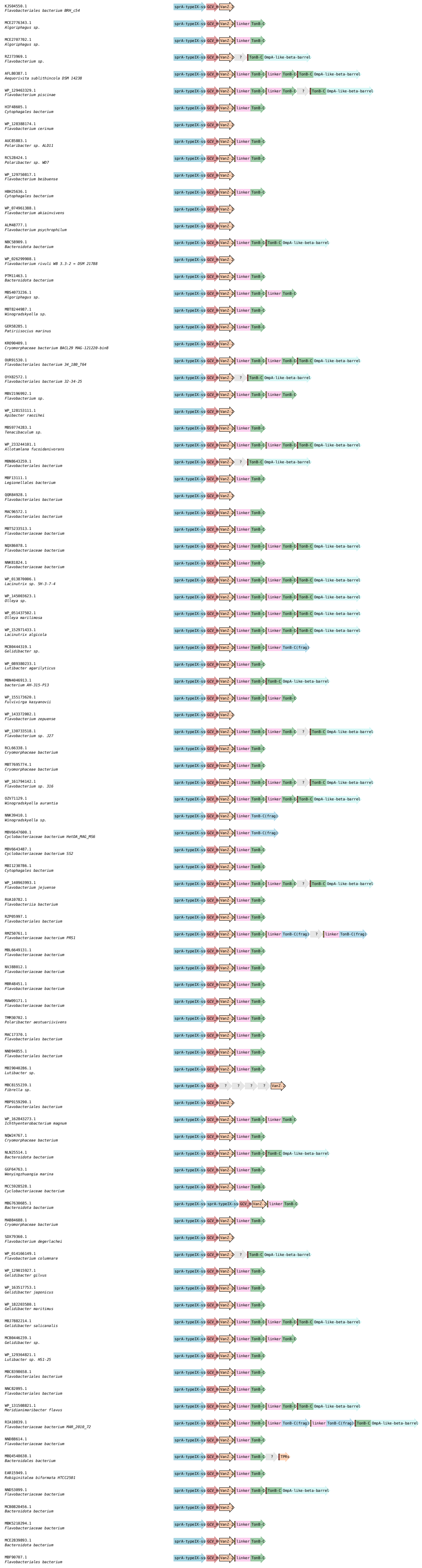
KJS04550.1 Flavobacteriales bacterium BRH_c54 sprA-typeIX-ss GCV_H VanZ-2
MCE2776343.1 Algoriphagus sp. sprA-typeIX-ss GCV_H VanZ-2 linker TonB-C
MCE2707702.1 Algoriphagus sp. sprA-typeIX-ss GCV_H VanZ-2 linker TonB-C
RZJ73969.1 Flavobacterium sp. sprA-typeIX-ss GCV_H VanZ-2 ? TonB-C OmpA-like-beta-barrel
AFL80387.1 Aequorivita sublithincola DSM 14238 sprA-typeIX-ss GCV_H VanZ-2 linker TonB-C linker TonB-C TonB-C OmpA-like-beta-barrel
WP_129463329.1 Flavobacterium piscinae sprA-typeIX-ss GCV_H VanZ-2 linker TonB-C linker TonB-C ? TonB-C OmpA-like-beta-barrel
HIF48605.1 Cytophagales bacterium sprA-typeIX-ss GCV_H VanZ-2 linker TonB-C
WP_128388174.1 Flavobacterium cerinum sprA-typeIX-ss GCV_H VanZ-2
AUC85883.1 Polaribacter sp. ALD11 sprA-typeIX-ss GCV_H VanZ-2 linker TonB-C
RCS28424.1 Polaribacter sp. WD7 sprA-typeIX-ss GCV_H VanZ-2 linker TonB-C
WP_129750817.1 Flavobacterium beibuense sprA-typeIX-ss GCV_H VanZ-2
HBH25636.1 Cytophagales bacterium sprA-typeIX-ss GCV_H VanZ-2 linker TonB-C
WP_074961388.1 Flavobacterium akiainvivens sprA-typeIX-ss GCV_H VanZ-2
ALM48777.1 Flavobacterium psychrophilum sprA-typeIX-ss GCV_H VanZ-2
NBC58909.1 Bacteroidota bacterium sprA-typeIX-ss GCV_H VanZ-2 linker TonB-C TonB-C OmpA-like-beta-barrel
WP_026299908.1 Flavobacterium rivuli WB 3.3-2 = DSM 21788 sprA-typeIX-ss GCV_H VanZ-2
PTM11463.1 Bacteroidota bacterium sprA-typeIX-ss GCV_H VanZ-2 linker TonB-C
MBS4073236.1 Algoriphagus sp. sprA-typeIX-ss GCV_H VanZ-2 linker TonB-C linker TonB-C
MBT8244987.1 Winogradskyella sp. sprA-typeIX-ss GCV_H VanZ-2 linker TonB-C
GER58285.1 Patiriisocius marinus sprA-typeIX-ss GCV_H VanZ-2 linker TonB-C
KRO90409.1 Cryomorphaceae bacterium BACL29 MAG-121220-bin8 sprA-typeIX-ss GCV_H VanZ-2
OUR91530.1 Flavobacteriales bacterium 34_180_T64 sprA-typeIX-ss GCV_H VanZ-2 linker TonB-C linker TonB-C TonB-C OmpA-like-beta-barrel
OYX82572.1 Flavobacteriales bacterium 32-34-25 sprA-typeIX-ss GCV_H VanZ-2 ? TonB-C OmpA-like-beta-barrel
MBV2196992.1 Flavobacterium sp. sprA-typeIX-ss GCV_H VanZ-2 linker TonB-C linker TonB-C
WP_128153111.1 Apibacter raozihei sprA-typeIX-ss GCV_H VanZ-2
MBS9774283.1 Tenacibaculum sp. sprA-typeIX-ss GCV_H VanZ-2 linker TonB-C
WP_233244101.1 Allotamlana fucoidanivorans sprA-typeIX-ss GCV_H VanZ-2 linker TonB-C linker TonB-C TonB-C OmpA-like-beta-barrel
MBN8643259.1 Flavobacteriales bacterium sprA-typeIX-ss GCV_H VanZ-2 ? TonB-C OmpA-like-beta-barrel
MBF13111.1 Legionellales bacterium sprA-typeIX-ss GCV_H VanZ-2 linker TonB-C
QQR84928.1 Flavobacteriales bacterium sprA-typeIX-ss GCV_H VanZ-2
MAC96572.1 Flavobacteriales bacterium sprA-typeIX-ss GCV_H VanZ-2 linker TonB-C
MBT5233513.1 Flavobacteriaceae bacterium sprA-typeIX-ss GCV_H VanZ-2 linker TonB-C
NQX86078.1 Flavobacteriaceae bacterium sprA-typeIX-ss GCV_H VanZ-2 linker TonB-C linker TonB-C TonB-C OmpA-like-beta-barrel
NNK81824.1 Flavobacteriaceae bacterium sprA-typeIX-ss GCV_H VanZ-2 linker TonB-C
WP_013870006.1 Lacinutrix sp. 5H-3-7-4 sprA-typeIX-ss GCV_H VanZ-2 linker TonB-C linker TonB-C TonB-C OmpA-like-beta-barrel
WP_145003623.1 Olleya sp. sprA-typeIX-ss GCV_H VanZ-2 linker TonB-C linker TonB-C TonB-C OmpA-like-beta-barrel
WP_051437502.1 Olleya marilimosa sprA-typeIX-ss GCV_H VanZ-2 linker TonB-C linker TonB-C TonB-C OmpA-like-beta-barrel
WP_152971433.1 Lacinutrix algicola sprA-typeIX-ss GCV_H VanZ-2 linker TonB-C linker TonB-C TonB-C OmpA-like-beta-barrel
MCB0444319.1 Gelidibacter sp. sprA-typeIX-ss GCV_H VanZ-2 linker TonB-C linker TonB-C(frag)
WP_089380233.1 Lutibacter agarilyticus sprA-typeIX-ss GCV_H VanZ-2 linker TonB-C
MBN4046913.1 bacterium AH-315-P13 sprA-typeIX-ss GCV_H VanZ-2 linker TonB-C TonB-C OmpA-like-beta-barrel
WP_155173620.1 Fulvivirga kasyanovii sprA-typeIX-ss GCV_H VanZ-2 linker TonB-C linker TonB-C
WP_143372002.1 Flavobacterium zepuense sprA-typeIX-ss GCV_H VanZ-2
WP_130733518.1 Flavobacterium sp. J27 sprA-typeIX-ss GCV_H VanZ-2 linker TonB-C linker TonB-C ? TonB-C OmpA-like-beta-barrel
RCL66338.1 Cryomorphaceae bacterium sprA-typeIX-ss GCV_H VanZ-2 linker TonB-C
MBT7695774.1 Cryomorphaceae bacterium sprA-typeIX-ss GCV_H VanZ-2 linker TonB-C
WP_161794142.1 Flavobacterium sp. 316 sprA-typeIX-ss GCV_H VanZ-2 linker TonB-C linker TonB-C ? TonB-C OmpA-like-beta-barrel
OZV71129.1 Winogradskyella aurantia sprA-typeIX-ss GCV_H VanZ-2 linker TonB-C linker TonB-C TonB-C OmpA-like-beta-barrel
NNK39410.1 Winogradskyella sp. sprA-typeIX-ss GCV_H VanZ-2 linker TonB-C(frag)
MBV6647600.1 Cyclobacteriaceae bacterium HetDA_MAG_MS6 sprA-typeIX-ss GCV_H VanZ-2 linker TonB-C(frag)
MBV6643487.1 Cyclobacteriaceae bacterium SS2 sprA-typeIX-ss GCV_H VanZ-2 linker TonB-C
MBI1230786.1 Cytophagales bacterium sprA-typeIX-ss GCV_H VanZ-2 linker TonB-C
WP_140963993.1 Flavobacterium jejuense sprA-typeIX-ss GCV_H VanZ-2 linker TonB-C linker TonB-C ? TonB-C OmpA-like-beta-barrel
RUA10782.1 Flavobacteriia bacterium sprA-typeIX-ss GCV_H VanZ-2 linker TonB-C
RZP05997.1 Flavobacteriales bacterium sprA-typeIX-ss GCV_H VanZ-2 linker TonB-C
RMZ50761.1 Flavobacteriaceae bacterium PRS1 sprA-typeIX-ss GCV_H VanZ-2 linker TonB-C linker TonB-C(frag) ? linker TonB-C(frag)
MBL6649131.1 Flavobacteriaceae bacterium sprA-typeIX-ss GCV_H VanZ-2 linker TonB-C
NVJ88012.1 Flavobacteriaceae bacterium sprA-typeIX-ss GCV_H VanZ-2 linker TonB-C
MBR48451.1 Flavobacteriaceae bacterium sprA-typeIX-ss GCV_H VanZ-2 linker TonB-C
MAW09171.1 Flavobacteriaceae bacterium sprA-typeIX-ss GCV_H VanZ-2 linker TonB-C
TMM30702.1 Polaribacter aestuariivivens sprA-typeIX-ss GCV_H VanZ-2 linker TonB-C
MAC17370.1 Flavobacteriales bacterium sprA-typeIX-ss GCV_H VanZ-2 linker TonB-C
NND94855.1 Flavobacteriales bacterium sprA-typeIX-ss GCV_H VanZ-2 linker TonB-C
MBI9040286.1 Lutibacter sp. sprA-typeIX-ss GCV_H VanZ-2 linker TonB-C
MBC8155239.1 Fibrella sp. sprA-typeIX-ss GCV_H ? ? ? ? VanZ-2
MBP9159290.1 Flavobacteriales bacterium sprA-typeIX-ss GCV_H VanZ-2
WP_162843273.1 Ichthyenterobacterium magnum sprA-typeIX-ss GCV_H VanZ-2 linker TonB-C linker TonB-C
NQW24767.1 Cryomorphaceae bacterium sprA-typeIX-ss GCV_H VanZ-2 linker TonB-C
NLN25514.1 Bacteroidota bacterium sprA-typeIX-ss GCV_H VanZ-2 linker TonB-C TonB-C OmpA-like-beta-barrel
GGF64763.1 Wenyingzhuangia marina sprA-typeIX-ss GCV_H VanZ-2 linker TonB-C
MCC5928528.1 Cyclobacteriaceae bacterium sprA-typeIX-ss GCV_H VanZ-2 linker TonB-C
MBG7630685.1 Bacteroidota bacterium sprA-typeIX-ss sprA-typeIX-ss GCV_H VanZ-2 linker TonB-C
MAB84688.1 Cryomorphaceae bacterium sprA-typeIX-ss GCV_H VanZ-2 linker TonB-C
SDX79360.1 Flavobacterium degerlachei sprA-typeIX-ss GCV_H VanZ-2
WP_014166149.1 Flavobacterium columnare sprA-typeIX-ss GCV_H VanZ-2 ? TonB-C OmpA-like-beta-barrel
WP_129015927.1 Gelidibacter gilvus sprA-typeIX-ss GCV_H VanZ-2 linker TonB-C
WP_163517753.1 Gelidibacter japonicus sprA-typeIX-ss GCV_H VanZ-2 linker TonB-C
WP_182203580.1 Gelidibacter maritimus sprA-typeIX-ss GCV_H VanZ-2 linker TonB-C
MBJ7882214.1 Gelidibacter salicanalis sprA-typeIX-ss GCV_H VanZ-2 linker TonB-C linker TonB-C TonB-C OmpA-like-beta-barrel
MCB0446239.1 Gelidibacter sp. sprA-typeIX-ss GCV_H VanZ-2 linker TonB-C linker TonB-C
WP_129364821.1 Lutibacter sp. HS1-25 sprA-typeIX-ss GCV_H VanZ-2 linker TonB-C
MBC8398658.1 Flavobacteriales bacterium sprA-typeIX-ss GCV_H VanZ-2 linker TonB-C
NNC82095.1 Flavobacteriales bacterium sprA-typeIX-ss GCV_H VanZ-2 linker TonB-C
WP_131508821.1 Meridianimaribacter flavus sprA-typeIX-ss GCV_H VanZ-2 linker TonB-C linker TonB-C TonB-C OmpA-like-beta-barrel
RIA10839.1 Flavobacteriaceae bacterium MAR_2010_72 sprA-typeIX-ss GCV_H VanZ-2 linker TonB-C linker TonB-C(frag) linker TonB-C(frag) TonB-C OmpA-like-beta-barrel
NND88614.1 Flavobacteriaceae bacterium sprA-typeIX-ss GCV_H VanZ-2 linker TonB-C
MBQ4548638.1 Bacteroidales bacterium sprA-typeIX-ss GCV_H VanZ-2 linker TonB-C ? TPRs
EAR15949.1 Robiginitalea biformata HTCC2501 sprA-typeIX-ss GCV_H VanZ-2 linker TonB-C
NND53099.1 Flavobacteriaceae bacterium sprA-typeIX-ss GCV_H VanZ-2 linker TonB-C TonB-C OmpA-like-beta-barrel
MCB0820456.1 Bacteroidota bacterium sprA-typeIX-ss GCV_H VanZ-2
MBK5210294.1 Flavobacteriaceae bacterium sprA-typeIX-ss GCV_H VanZ-2 linker TonB-C
MCE2839893.1 Bacteroidota bacterium sprA-typeIX-ss GCV_H VanZ-2 linker TonB-C
MBF90707.1 Flavobacteriales bacterium sprA-typeIX-ss GCV_H VanZ-2 linker TonB-C

MBC8464623.1
Bacteroidota bacterium

MBT8253698.1
Bacteroidia bacterium

MBT8257421.1
Bacteroidia bacterium

MAZ68001.1
Fidelibacterota bacterium

MBL7885582.1
Flavobacterium sp.

KRO67430.1
Cryomorphaceae bacterium BACL21 MAG-121220-bin10

MBG15026.1
Crocinitomicaceae bacterium

WP_205722010.1
Fulvivirga lutea

MBT8187474.1
Croceitalea sp.

WP_034234590.1
Arenibacter certesii

WP_129460013.1
Flavobacterium stagni

SHO72088.1
Flavobacterium cucumis

MAV00117.1
Flavobacteriaceae bacterium

MAN70956.1
Flavobacteriaceae bacterium

WP_215954846.1
Cellulophaga sp. 1_MG-2023

RZS96159.1
Cecembia calidifontis

HJH60842.1
Candidatus Limimorpha avicola

MXV37511.1
Flavobacteriaceae bacterium Ap0902

MBU3820923.1
Marixanthotalea marina

NNC95884.1
Chitinophagales bacterium

MCB0803022.1
Flavobacteriales bacterium

NMN32379.1

MBF6610773.1
Chryseobacterium sp.

RPG95042.1
Candidatus Pelagibacter sp. TMED253

MBT8270854.1
Bacteroidia bacterium

WP_181248534.1
Flavobacterium magnum

WP_051359866.1
Adhaeribacter aquaticus DSM 16391

NHM02240.1
Flavobacterium difficile

SDB57952.1
Flavobacteriaceae bacterium MAR_2010_188

MBT6169270.1
Flavobacteriaceae bacterium

WP_154364737.1
Maribacter luteus

WP_013305289.1

AXT18922.1
Flavobacteriaceae bacterium

MBU86606.1
Flavobacteriaceae bacterium

MBN2729057.1
Bacteroidales bacterium

WP_164721444.1
Maribacter sp. MJ134

WP_029447136.1
Cellulophaga baltica

EKF56582.1
Galbibacter marinus

ESU29524.1
Flavobacterium limnosediminis JC2902

WP_103726780.1
Flavobacterium croceum

MBR5377737.1
Bacteroidales bacterium

MBR5603743.1
Bacteroidales bacterium

MBL4709636.1
Flavobacteriales bacterium

NNE32618.1
Winogradskyella sp.

NND78088.1
Flavobacteriales bacterium

WP_155805525.1
Imtechella halotolerans

MBE2247652.1
Candidatus Competibacteraceae bacterium

OQD43960.1
Croceivirga radicis

WP_214112568.1
Aequorivita echinoideorum

TRZ45782.1
Robertkochia solimangrovi

WP_181858338.1
Winogradskyella arenosi

MBI2721811.1
Bacteroidota bacterium

MBU1011646.1
Bacteroidota bacterium

NNE02340.1
Eudoraea sp.

WP_163605680.1
Leptobacterium flavescens

HBH06296.1
Flavobacteriales bacterium

SMD34998.1
Reichenbachiella faecimaris

MBL6645875.1
Flavobacteriales bacterium

MBT6438109.1
Flavobacteriales bacterium

HFO54179.1

MBC7606474.1
Flavobacterium sp.

EDM44263.1
unidentified eubacterium SCB49

MBQ0147695.1
Candidatus Onthonaster equi

MBC7451550.1
Cytophaga sp.

KEO74641.1
Anditalea andensis

WP_223845556.1

MBT5402881.1
Crocinitomicaceae bacterium

WP_215967652.1
Oceanihabitans sp. 1_MG-2023

MBK7214727.1
Bacteroidales bacterium

CAG5086201.1
Parvicella tangerina

PHK28741.1
Nostoc linckia z16

MAN27421.1
Mesonia sp.

MBT8309560.1
Bacteroidia bacterium

WP_162984929.1
Mesonia aquimarina

PVY37228.1
Pontibacter virosus

MCB0456302.1
Flavobacteriaceae bacterium

MBU6204283.1
Bacteroidota bacterium

NVJ63485.1
Flavobacteriaceae bacterium

WP_162897274.1
Aquimarina sp. BL5

MBR06909.1
Rickettsiales bacterium

MBV7441066.1
Weeksellaceae bacterium TAE3-ERU29

MBL0054340.1
Bacteroidota bacterium

WP_051191514.1
Flavobacterium gelidilacus

OIQ16675.1
Lacinutrix sp. MedPE-SW

MBT8234888.1
Bacteroidia bacterium

MCB9426108.1
Flavobacteriales bacterium

NRA94134.1
Psychroserpens sp.

RXG24668.1
Leeuwenhoekiella aequorea

ARV09457.1
Winogradskyella sp. PC-19

MBT0813362.1

WP_106463078.1

TDY45783.1
Algoriphagus zhangzhouensis

PZR13820.1
Flavobacterium psychrophilum

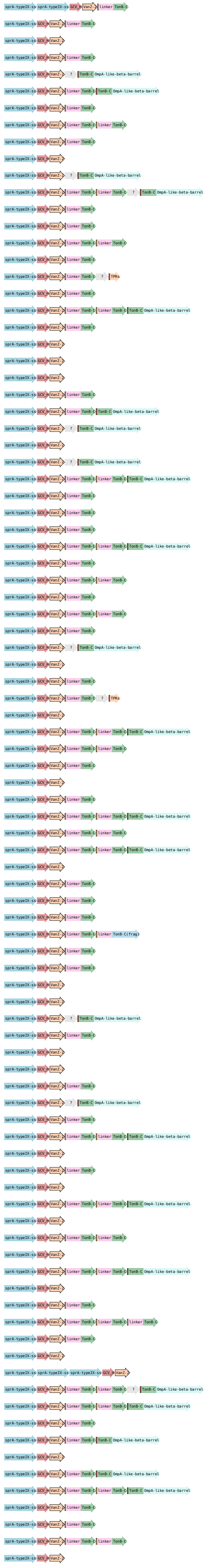

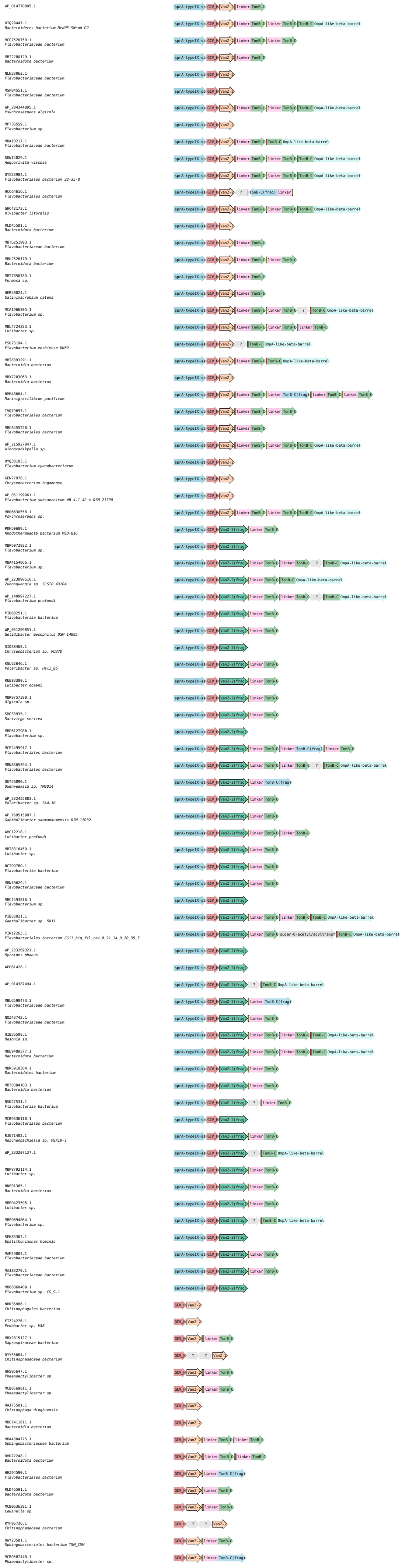
WP_014770805.1
OIQ29447.1
Bacteroidetes bacterium MedPE-SWsnd-G2
MCC7520759.1
Flavobacteriaceae bacterium
MBI2280129.1
Bacteroidota bacterium
NLN33062.1
Flavobacteriaceae bacterium
MSP84551.1
Flavobacteriaceae bacterium
WP_204344895.1
Psychroserpens algicola
MPT36559.1
Flavobacterium sp.
MBA10217.1
Flavobacteriaceae bacterium
SDW14829.1
Aequorivita viscosa
OYX23904.1
Flavobacteriales bacterium 32-35-8
HCC64616.1
Flavobacteriales bacterium
GHC42173.1
Ulvibacter litoralis
RLD45581.1
Bacteroidota bacterium
MBT6251993.1
Flavobacteriaceae bacterium
MBU2526179.1
Bacteroidota bacterium
MBT7850783.1
Formosa sp.
HER40824.1
Salinimicrobium catena
MCA1966305.1
Flavobacterium sp.
MBL4724333.1
Lutibacter sp.
ESU23194.1
Flavobacterium enshiense DK69
MBT8393291.1
Bacteroidia bacterium
MBX7203063.1
Bacteroidia bacterium
NMM48664.1
Marinigracilibium pacificum
TVQ79697.1
Flavobacteriales bacterium
MBC8455228.1
Flavobacteriales bacterium
WP_215927847.1
Winogradskyella sp.
OYQ38183.1
Flavobacterium cyanobacteriorum
GEN77978.1
Chryseobacterium hagamense
WP_051199961.1
Flavobacterium subsaxonicum WB 4.1-42 = DSM 21790
MBO6630558.1
Psychroserpens sp.
PDH50609.1
Rhodothermaeota bacterium MED-G16
MBP6072932.1
Flavobacterium sp.
MBA4154066.1
Flavobacterium sp.
WP_223090516.1
Zunongwangia sp. SCSIO 43204
WP_140997227.1
Flavobacterium profundi
PID68251.1
Flavobacteriia bacterium
WP_051209851.1
Gelidibacter mesophilus DSM 14095
SIQ38468.1
Chryseobacterium sp. RU37D
KGL62646.1
Polaribacter sp. Hel1_85
REE83300.1
Lutibacter oceani
MBR9757388.1
Algicola sp.
SMG33935.1
Marivirga sericea
MBP6127986.1
Flavobacterium sp.
MCE2495917.1
Flavobacteriales bacterium
MBN8565394.1
Flavobacteriales bacterium
OUT46890.1
Owenweeksia sp. TMED14
WP_232455883.1
Polaribacter sp. SA4-10
WP_169515987.1
Gaetbulibacter saemankumensis DSM 17032
AMC12218.1
Lutibacter profundi
MBT8316459.1
Lutibacter sp.
NCT09780.1
Flavobacteriia bacterium
MBN10629.1
Flavobacteriaceae bacterium
MBC7493816.1
Flavobacterium sp.
PIB33921.1
Gaetbulibacter sp. 5U11
PIR12263.1
Flavobacteriales bacterium CG11_big_fil_rev_8_21_14_0_20_35_7
WP_233269321.1
Myroides phaeus
APG61428.1
WP_014387494.1
MBL6590473.1
Flavobacteriaceae bacterium
NQZ43742.1
Flavobacteriaceae bacterium
HIB36508.1
Mesonia sp.
MBE9489377.1
Bacteroidota bacterium
MBR5910364.1
Bacteroidales bacterium
MBT8384103.1
Bacteroidia bacterium
NVK27531.1
Flavobacteriia bacterium
MCB9336110.1
Flavobacteriales bacterium
RJE71402.1
Reichenbachiella sp. MSK19-1
WP_233207137.1
MBP8792114.1
Lutibacter sp.
NNF01365.1
Bacteroidia bacterium
MBE0423595.1
Lutibacter sp.
MBF0694864.1
Flavobacterium sp.
SEH83363.1
Epilithonimonas hominis
MAM49804.1
Flavobacteriaceae bacterium
MAJ83270.1
Flavobacteriaceae bacterium
MBG6060409.1
Flavobacterium sp. CG_9.1
NBR36986.1
Chitinophagales bacterium
ETZ24276.1
Pedobacter sp. V48
MBX2815127.1
Saprospiraceae bacterium
RYY55684.1
Chitinophagaceae bacterium
HHS95647.1
Phaeodactylibacter sp.
MCB0569011.1
Phaeodactylibacter sp.
RAJ75501.1
Chitinophaga dinghuensis
MBC7411611.1
Bacteroidia bacterium
MBA4304725.1
Sphingobacteriaceae bacterium
RMD72248.1
Bacteroidota bacterium
HHZ94590.1
Flavobacteriales bacterium
RLD46591.1
Bacteroidota bacterium
MCB0636381.1
Lewinella sp.
RYF96736.1
Chitinophagaceae bacterium
OWY23581.1
Sphingobacteriales bacterium TSM_CSM
MCB0587448.1
Phaeodactylibacter sp.
sprA-typeIX-ss
GCV_H
VanZ
VanZ-2(frag)
linker
TonB-C
TonB-C(frag)
OmpA-like-beta-barrel
sugar-O-acetyl/acyltransf
?

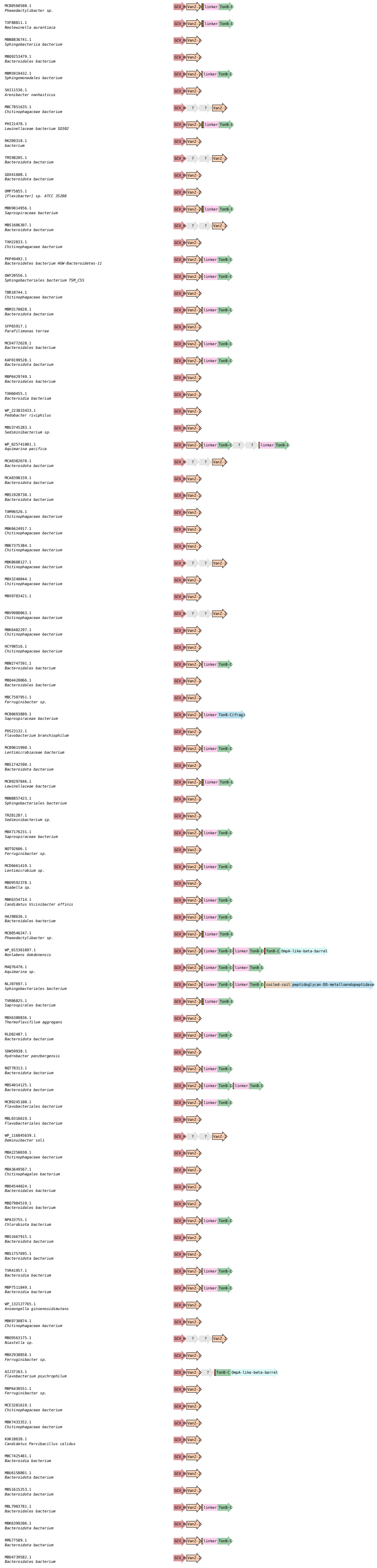
MCB0560508.1
Phaeodactylibacter sp.
GCV_H VanZ- linker TonB-C
TXF88811.1
Neolewinella aurantiaca
GCV_H VanZ- linker TonB-C
MBN8836741.1
Sphingobacteriia bacterium
GCV_H VanZ-
MBQ9253479.1
Bacteroidales bacterium
GCV_H VanZ-
MBM3919432.1
Sphingomonadales bacterium
GCV_H VanZ- linker TonB-C
SHJ11536.1
Arenibacter nanhaiticus
GCV_H VanZ-
MBC7851635.1
Chitinophagaceae bacterium
GCV_H ? ? VanZ-
PHI21478.1
Lewinellaceae bacterium SD302
GCV_H VanZ- linker TonB-C
RKZ09318.1
bacterium
GCV_H VanZ-
TMI98205.1
Bacteroidota bacterium
GCV_H ? ? VanZ-
GDX41608.1
Bacteroidota bacterium
GCV_H VanZ-
OMP75055.1
[Flexibacter] sp. ATCC 35208
GCV_H VanZ-
MBK9014956.1
Saprospiraceae bacterium
GCV_H VanZ- linker TonB-C
MBS1606307.1
Bacteroidota bacterium
GCV_H ? ? VanZ-
TXH22833.1
Chitinophagaceae bacterium
GCV_H VanZ-
PKP49492.1
Bacteroidetes bacterium HGW-Bacteroidetes-11
GCV_H VanZ- linker TonB-C
OWY20556.1
Sphingobacteriales bacterium TSM_CSS
GCV_H VanZ- linker TonB-C
TBR18744.1
Chitinophagaceae bacterium
GCV_H VanZ-
MBM3170828.1
Bacteroidota bacterium
GCV_H VanZ- linker TonB-C
SFP65917.1
Parafilimonas terrae
GCV_H VanZ-
MCD4772628.1
Bacteroidales bacterium
GCV_H VanZ- linker TonB-C
KAF0199528.1
Bacteroidota bacterium
GCV_H VanZ- linker TonB-C
MBP6429749.1
Bacteroidales bacterium
GCV_H VanZ-
TXH60455.1
Bacteroidia bacterium
GCV_H VanZ-
WP_223833433.1
Pedobacter riviphilus
GCV_H VanZ-
MBU3745283.1
Sediminibacterium sp.
GCV_H VanZ-
WP_025741001.1
Aquimarina pacifica
GCV_H VanZ- linker TonB-C ? ? linker TonB-C
MCA0382670.1
Bacteroidota bacterium
GCV_H ? ? VanZ-
MCA0398159.1
Bacteroidota bacterium
GCV_H VanZ-
MBS1928730.1
Bacteroidota bacterium
GCV_H VanZ-
TAM96526.1
Chitinophagaceae bacterium
GCV_H VanZ-
MBK6624917.1
Chitinophagaceae bacterium
GCV_H VanZ-
MBK7375384.1
Chitinophagaceae bacterium
GCV_H VanZ-
MBK8608127.1
Chitinophagaceae bacterium
GCV_H ? ? VanZ-
MBX3240044.1
Chitinophagaceae bacterium
GCV_H VanZ-
MBX9783421.1
GCV_H VanZ-
MBV9988063.1
Chitinophagaceae bacterium
GCV_H ? ? VanZ-
MBK6482297.1
Chitinophagaceae bacterium
GCV_H VanZ-
HCY90516.1
Chitinophagaceae bacterium
GCV_H VanZ-
MBN2747591.1
Bacteroidales bacterium
GCV_H VanZ- linker TonB-C
MBQ4420066.1
Bacteroidales bacterium
GCV_H VanZ-
MBC7507951.1
Ferruginibacter sp.
GCV_H VanZ-
MCB0693889.1
Saprospiraceae bacterium
GCV_H VanZ- linker TonB-C(frag)
PDS23132.1
Flavobacterium branchiophilum
GCV_H VanZ-
MCB9015990.1
Lentimicrobiaceae bacterium
GCV_H VanZ- linker TonB-C
MBS1742590.1
Bacteroidota bacterium
GCV_H VanZ-
MCB9297846.1
Lewinellaceae bacterium
GCV_H VanZ- linker TonB-C
MBN8857423.1
Sphingobacteriales bacterium
GCV_H VanZ-
TRZ81287.1
Sediminibacterium sp.
GCV_H VanZ-
MBX7176231.1
Saprospiraceae bacterium
GCV_H VanZ- linker TonB-C
NOT92606.1
Ferruginibacter sp.
GCV_H VanZ-
MCD6661419.1
Lentimicrobium sp.
GCV_H VanZ- linker TonB-C
MBO9592378.1
Niabella sp.
GCV_H VanZ-
MBK6354714.1
Candidatus Vicinibacter affinis
GCV_H VanZ- linker TonB-C
HAJ98636.1
Bacteroidales bacterium
GCV_H VanZ- linker TonB-C
MCB0546247.1
Phaeodactylibacter sp.
GCV_H VanZ- linker TonB-C
WP_015361697.1
Nonlabens dokdonensis
GCV_H VanZ- linker TonB-C linker TonB-C TonB-C OmpA-like-beta-barrel
MAQ76470.1
Aquimarina sp.
GCV_H VanZ- linker TonB-C linker TonB-C
NLJ07697.1
Sphingobacteriales bacterium
GCV_H VanZ- linker TonB-C linker TonB-C coiled-coil peptidoglycan-DD-metalloendopeptidase
TVR86825.1
Saprospirales bacterium
GCV_H VanZ- linker TonB-C
MBX6380836.1
Thermoflavifilum aggregans
GCV_H VanZ-
RLD82487.1
Bacteroidota bacterium
GCV_H VanZ- linker TonB-C
SDW59938.1
Hydrobacter penzbergensis
GCV_H VanZ-
NQT78313.1
Bacteroidota bacterium
GCV_H VanZ- linker TonB-C
MBS4014125.1
Bacteroidota bacterium
GCV_H VanZ- linker TonB-C linker TonB-C
MCB9245100.1
Flavobacteriales bacterium
GCV_H VanZ- linker TonB-C
MBL0316619.1
Flavobacteriales bacterium
GCV_H VanZ-
WP_116845639.1
Deminuibacter soli
GCV_H ? ? VanZ-
MBA2250030.1
Chitinophagaceae bacterium
GCV_H VanZ-
MBA3649567.1
Chitinophagales bacterium
GCV_H VanZ-
MBO4544024.1
Bacteroidales bacterium
GCV_H VanZ-
MBQ7984519.1
Bacteroidales bacterium
GCV_H VanZ-
NPA33755.1
Chlorobiota bacterium
GCV_H VanZ- linker TonB-C
MBS1667915.1
Bacteroidota bacterium
GCV_H VanZ-
MBS1757695.1
Bacteroidota bacterium
GCV_H VanZ-
TVR41957.1
Bacteroidia bacterium
GCV_H VanZ- linker TonB-C
MBP7511849.1
Bacteroidia bacterium
GCV_H VanZ- linker TonB-C
WP_132127765.1
Anseongella ginsenosidimutans
GCV_H VanZ-
MBK9730874.1
Chitinophagaceae bacterium
GCV_H VanZ-
MBO9563175.1
Niastella sp.
GCV_H ? ? VanZ-
MBX2938858.1
Ferruginibacter sp.
GCV_H VanZ-
AIJ37163.1
Flavobacterium psychrophilum
GCV_H VanZ- ? TonB-C OmpA-like-beta-barrel
MBP6430551.1
Ferruginibacter sp.
GCV_H VanZ-
MCE3281619.1
Chitinophagaceae bacterium
GCV_H VanZ-
MBK7433352.1
Chitinophagaceae bacterium
GCV_H VanZ-
KXK18638.1
Candidatus Parvibacillus calidus
GCV_H VanZ-
MBC7425481.1
Bacteroidia bacterium
GCV_H VanZ-
MBU6158801.1
Bacteroidota bacterium
GCV_H VanZ-
MBS1615253.1
Bacteroidota bacterium
GCV_H VanZ-
MBL7903781.1
Bacteroidales bacterium
GCV_H VanZ- linker TonB-C
MBK6399206.1
Bacteroidota bacterium
GCV_H VanZ-
RMG77589.1
Bacteroidota bacterium
GCV_H VanZ- linker TonB-C
MBO4739582.1
Bacteroidales bacterium
GCV_H VanZ-

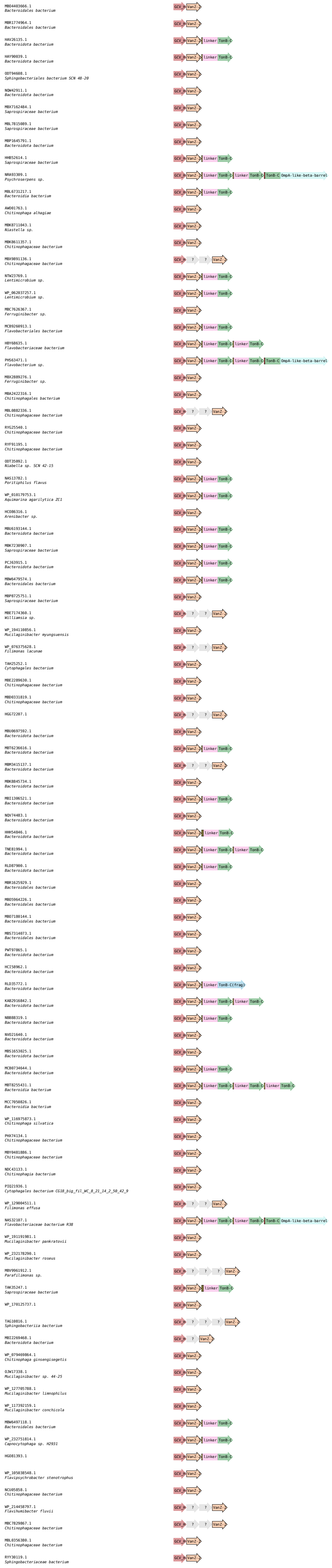
MBO4403666.1
Bacteroidales bacterium
GCV_H VanZ
MBR1774964.1
Bacteroidales bacterium
GCV_H VanZ
HAV26135.1
Bacteroidota bacterium
GCV_H VanZ linker TonB-C
HAY90039.1
Bacteroidota bacterium
GCV_H VanZ linker TonB-C
ODT94608.1
Sphingobacteriales bacterium SCN 48-20
GCV_H VanZ
NQW42911.1
Bacteroidota bacterium
GCV_H VanZ
MBX7162484.1
Saprospiraceae bacterium
GCV_H VanZ
MBL7815089.1
Saprospiraceae bacterium
GCV_H VanZ
MBP1645791.1
Bacteroidota bacterium
GCV_H VanZ
HHB52614.1
Saprospiraceae bacterium
GCV_H VanZ linker TonB-C
NRA93309.1
Psychroserpens sp.
GCV_H VanZ linker TonB-C linker TonB-C TonB-C OmpA-like-beta-barrel
MBL6731217.1
Bacteroidia bacterium
GCV_H VanZ linker TonB-C
AWO01763.1
Chitinophaga alhagiae
GCV_H VanZ
MBK8711043.1
Niastella sp.
GCV_H VanZ
MBK8611357.1
Chitinophagaceae bacterium
GCV_H VanZ
MBX9891136.1
Chitinophagaceae bacterium
GCV_H ? ? VanZ
NTW23769.1
Lentimicrobium sp.
GCV_H VanZ linker TonB-C
WP_062037257.1
Lentimicrobium sp.
GCV_H VanZ linker TonB-C
MBC7626367.1
Ferruginibacter sp.
GCV_H VanZ
MCB9260913.1
Flavobacteriales bacterium
GCV_H VanZ linker TonB-C
HBY68635.1
Flavobacteriaceae bacterium
GCV_H VanZ linker TonB-C linker TonB-C
PHS63471.1
Flavobacterium sp.
GCV_H VanZ linker TonB-C linker TonB-C TonB-C OmpA-like-beta-barrel
MBX2889276.1
Ferruginibacter sp.
GCV_H VanZ
MBA2422316.1
Chitinophagales bacterium
GCV_H VanZ
MBL0882336.1
Chitinophagaceae bacterium
GCV_H ? ? VanZ
RYG25540.1
Chitinophagaceae bacterium
GCV_H VanZ
RYF91195.1
Chitinophagaceae bacterium
GCV_H VanZ
ODT35092.1
Niabella sp. SCN 42-15
GCV_H VanZ
NAS13782.1
Poritiphilus flavus
GCV_H VanZ linker TonB-C
WP_010179753.1
Aquimarina agarilytica ZC1
GCV_H VanZ linker TonB-C
HCO86316.1
Arenibacter sp.
GCV_H VanZ
MBU6193144.1
Bacteroidota bacterium
GCV_H VanZ linker TonB-C
MBK7230907.1
Saprospiraceae bacterium
GCV_H VanZ linker TonB-C
PCJ63915.1
Bacteroidota bacterium
GCV_H VanZ linker TonB-C
MBW6479574.1
Bacteroidales bacterium
GCV_H VanZ linker TonB-C
MBP8725751.1
Saprospiraceae bacterium
GCV_H VanZ
MBE7174360.1
Williamsia sp.
GCV_H ? ? VanZ
WP_194110856.1
Mucilaginibacter myungsuensis
GCV_H VanZ
WP_076375628.1
Filimonas lacunae
GCV_H ? ? VanZ
TAH25252.1
Cytophagales bacterium
GCV_H VanZ
MBE2289630.1
Chitinophagaceae bacterium
GCV_H VanZ
MBD0331819.1
Chitinophagaceae bacterium
GCV_H VanZ
HGG72207.1
GCV_H ? ? VanZ
MBU0697592.1
Bacteroidota bacterium
GCV_H VanZ
MBT6236616.1
Bacteroidota bacterium
GCV_H VanZ linker TonB-C
MBM3415137.1
Bacteroidota bacterium
GCV_H ? ? VanZ
MBK8845734.1
Bacteroidota bacterium
GCV_H VanZ
MBI1306521.1
Bacteroidota bacterium
GCV_H VanZ linker TonB-C
NQV74483.1
Bacteroidota bacterium
GCV_H VanZ
HHH54846.1
Bacteroidota bacterium
GCV_H VanZ linker TonB-C
TNE81994.1
Bacteroidota bacterium
GCV_H VanZ linker TonB-C linker TonB-C
RLD87900.1
Bacteroidota bacterium
GCV_H VanZ linker TonB-C
MBR1625929.1
Bacteroidales bacterium
GCV_H VanZ
MBO5964226.1
Bacteroidales bacterium
GCV_H VanZ
MBO7180144.1
Bacteroidales bacterium
GCV_H VanZ
MBS7314073.1
Bacteroidales bacterium
GCV_H VanZ
PWT97865.1
Bacteroidota bacterium
GCV_H VanZ
HCI58962.1
Bacteroidota bacterium
GCV_H VanZ
RLD35772.1
Bacteroidota bacterium
GCV_H VanZ linker TonB-C(frag)
KAB2916842.1
Bacteroidota bacterium
GCV_H VanZ linker TonB-C linker TonB-C
NBB88319.1
Bacteroidota bacterium
GCV_H VanZ linker TonB-C
NVO21640.1
Bacteroidota bacterium
GCV_H VanZ
MBS1653025.1
Bacteroidota bacterium
GCV_H VanZ
MCB0734644.1
Bacteroidota bacterium
GCV_H VanZ linker TonB-C
MBT8255431.1
Bacteroidia bacterium
GCV_H VanZ linker TonB-C linker TonB-C linker TonB-C
MCC7050826.1
Bacteroidia bacterium
GCV_H VanZ
WP_116975873.1
Chitinophaga silvatica
GCV_H VanZ
PHX74134.1
Chitinophagaceae bacterium
GCV_H VanZ
MBY0481886.1
Chitinophagaceae bacterium
GCV_H VanZ
NDC43133.1
Chitinophagia bacterium
GCV_H VanZ
PIQ21936.1
Cytophagales bacterium CG18_big_fil_WC_8_21_14_2_50_42_9
GCV_H VanZ
WP_129004511.1
Filimonas effusa
GCV_H ? ? VanZ
NAS32107.1
Flavobacteriaceae bacterium R38
GCV_H VanZ linker TonB-C linker TonB-C TonB-C OmpA-like-beta-barrel
WP_191191981.1
Mucilaginibacter pankratovii
GCV_H VanZ
WP_232178290.1
Mucilaginibacter roseus
GCV_H VanZ
MBV9961912.1
Parafilimonas sp.
GCV_H ? ? ? VanZ
TAK35247.1
Saprospiraceae bacterium
GCV_H VanZ linker TonB-C
WP_170125737.1
GCV_H VanZ
TAG10816.1
Sphingobacteriia bacterium
GCV_H ? ? ? VanZ
MBI2269468.1
Bacteroidota bacterium
GCV_H ? VanZ
WP_079469864.1
Chitinophaga ginsengisegetis
GCV_H VanZ
OJW17338.1
Mucilaginibacter sp. 44-25
GCV_H VanZ
WP_127705788.1
Mucilaginibacter limnophilus
GCV_H VanZ
WP_117392159.1
Mucilaginibacter conchicola
GCV_H VanZ
MBW6497118.1
Bacteroidales bacterium
GCV_H VanZ linker TonB-C
WP_232751814.1
Capnocytophaga sp. H2931
GCV_H VanZ linker TonB-C
HGO81393.1
GCV_H VanZ linker TonB-C
WP_105038548.1
Flavipsychrobacter stenotrophus
GCV_H VanZ
NCU05858.1
Chitinophagaceae bacterium
GCV_H VanZ
WP_214458797.1
Flavihumibacter fluvii
GCV_H ? ? VanZ
MBC7829867.1
Chitinophagaceae bacterium
GCV_H ? ? VanZ
MBL0356380.1
Chitinophagaceae bacterium
GCV_H VanZ
RYY30119.1
Sphingobacteriaceae bacterium
GCV_H VanZ

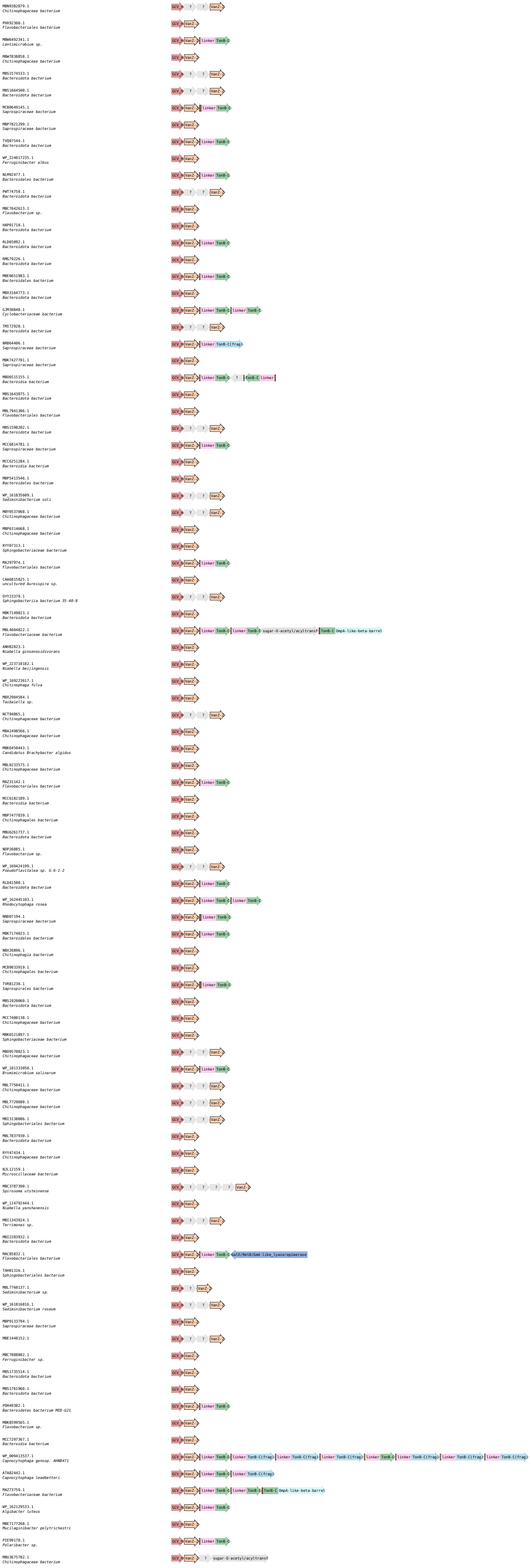
MBN9382879.1
Chitinophagaceae bacterium
GCV_H ? ? VanZ-2
PHX92360.1
Flavobacteriales bacterium
GCV_H VanZ-2
MBW6492341.1
Lentimicrobium sp.
GCV_H VanZ-2 linker TonB-C
MBW7838858.1
Chitinophagaceae bacterium
GCV_H VanZ-2
MBS1574533.1
Bacteroidota bacterium
GCV_H ? ? VanZ-2
MBS1664500.1
Bacteroidota bacterium
GCV_H ? ? VanZ-2
MCB0649145.1
Saprospiraceae bacterium
GCV_H VanZ-2 linker TonB-C
MBP7821299.1
Saprospiraceae bacterium
GCV_H VanZ-2
TVQ07544.1
Bacteroidota bacterium
GCV_H VanZ-2 linker TonB-C
WP_224017235.1
Ferruginibacter albus
GCV_H VanZ-2
NLM92477.1
Bacteroidales bacterium
GCV_H VanZ-2 linker TonB-C
PWT74750.1
Bacteroidota bacterium
GCV_H ? ? VanZ-2
MBC7642613.1
Flavobacterium sp.
GCV_H VanZ-2
HAP01710.1
Bacteroidota bacterium
GCV_H VanZ-2
RLD65092.1
Bacteroidota bacterium
GCV_H VanZ-2 linker TonB-C
RMG79226.1
Bacteroidota bacterium
GCV_H VanZ-2
MBE0651983.1
Bacteroidales bacterium
GCV_H VanZ-2 linker TonB-C
MBX3164773.1
Bacteroidota bacterium
GCV_H VanZ-2
GJM30848.1
Cyclobacteriaceae bacterium
GCV_H VanZ-2 linker TonB-C linker TonB-C
TMI72928.1
Bacteroidota bacterium
GCV_H ? ? VanZ-2
NRB64406.1
Saprospiraceae bacterium
GCV_H VanZ-2 linker TonB-C(frag)
MBK7427701.1
Saprospiraceae bacterium
GCV_H VanZ-2
MBO6515155.1
Bacteroidia bacterium
GCV_H VanZ-2 linker TonB-C ? TonB-C linker
MBS1643875.1
Bacteroidota bacterium
GCV_H VanZ-2
MBL7941306.1
Flavobacteriales bacterium
GCV_H VanZ-2
MBS1598202.1
Bacteroidota bacterium
GCV_H ? ? VanZ-2
MCC6814781.1
Saprospiraceae bacterium
GCV_H VanZ-2 linker TonB-C
MCC6251284.1
Bacteroidia bacterium
GCV_H VanZ-2
MBP5413546.1
Bacteroidales bacterium
GCV_H VanZ-2
WP_161835609.1
Sediminibacterium soli
GCV_H ? ? VanZ-2
MBY0537068.1
Chitinophagaceae bacterium
GCV_H ? ? VanZ-2
MBP6314668.1
Chitinophagaceae bacterium
GCV_H VanZ-2
RYY07313.1
Sphingobacteriaceae bacterium
GCV_H VanZ-2
MAJ97974.1
Flavobacteriales bacterium
GCV_H VanZ-2 linker TonB-C
CAA6815825.1
uncultured Aureispira sp.
GCV_H VanZ-2
OYY23379.1
Sphingobacteriia bacterium 35-40-8
GCV_H ? ? VanZ-2
MBK7149023.1
Bacteroidota bacterium
GCV_H VanZ-2
MBL4604822.1
Flavobacteriaceae bacterium
GCV_H VanZ-2 linker TonB-C linker TonB-C sugar-O-acetyl/acyltransf TonB-C OmpA-like-beta-barrel
ANH82823.1
Niabella ginsenosidivorans
GCV_H VanZ-2
WP_223710182.1
Niabella beijingensis
GCV_H VanZ-2
WP_169223617.1
Chitinophaga fulva
GCV_H VanZ-2
MBX2904584.1
Taibaiella sp.
GCV_H VanZ-2
NCT94865.1
Chitinophagaceae bacterium
GCV_H ? ? VanZ-2
MBA2498566.1
Chitinophagaceae bacterium
GCV_H VanZ-2
MBK6450443.1
Candidatus Brachybacter algidus
GCV_H VanZ-2
MBL0233575.1
Chitinophagaceae bacterium
GCV_H VanZ-2
MAZ31142.1
Flavobacteriales bacterium
GCV_H VanZ-2 linker TonB-C
MCC6182189.1
Bacteroidia bacterium
GCV_H VanZ-2
MBP7477839.1
Chitinophagales bacterium
GCV_H VanZ-2
MBU6261737.1
Bacteroidota bacterium
GCV_H VanZ-2
NDP26885.1
Flavobacterium sp.
GCV_H VanZ-2
WP_169424199.1
Pseudoflavitalea sp. G-6-1-2
GCV_H ? ? VanZ-2
RLD41500.1
Bacteroidota bacterium
GCV_H VanZ-2 linker TonB-C
WP_162445103.1
Rhodocytophaga rosea
GCV_H VanZ-2 linker TonB-C linker TonB-C
NND07194.1
Saprospiraceae bacterium
GCV_H VanZ-2 linker TonB-C
MBK7174023.1
Bacteroidales bacterium
GCV_H VanZ-2 linker TonB-C
NBX26896.1
Chitinophagia bacterium
GCV_H VanZ-2
MCB9033919.1
Chitinophagales bacterium
GCV_H VanZ-2
TVR81238.1
Saprospirales bacterium
GCV_H VanZ-2 linker TonB-C
MBS1920060.1
Bacteroidota bacterium
GCV_H VanZ-2
MCC7400138.1
Chitinophagaceae bacterium
GCV_H VanZ-2
MBK6521897.1
Sphingobacteriaceae bacterium
GCV_H VanZ-2
MBO9570823.1
Chitinophagaceae bacterium
GCV_H ? ? VanZ-2
WP_101333050.1
Brumimicrobium salinarum
GCV_H VanZ-2 linker TonB-C
MBL7750411.1
Chitinophagaceae bacterium
GCV_H ? ? VanZ-2
MBL7729680.1
Chitinophagaceae bacterium
GCV_H ? ? VanZ-2
MBI3138086.1
Sphingobacteriales bacterium
GCV_H ? ? VanZ-2
MBL7837930.1
Bacteroidota bacterium
GCV_H VanZ-2
RYY47434.1
Chitinophagaceae bacterium
GCV_H VanZ-2
NJL12159.1
Microscillaceae bacterium
GCV_H VanZ-2
MBC3787390.1
Spirosoma utsteinense
GCV_H ? ? ? ? VanZ-2
WP_114792444.1
Niabella yanshanensis
GCV_H VanZ-2
MBI1343924.1
Terrimonas sp.
GCV_H ? ? VanZ-2
MBI2283932.1
Bacteroidota bacterium
GCV_H VanZ-2
MAC85832.1
Flavobacteriales bacterium
GCV_H VanZ-2 linker TonB-C GalE/RmlB/Gmd-like_lyase/epimerase
TAH01316.1
Sphingobacteriales bacterium
GCV_H VanZ-2
MBL7760137.1
Sediminibacterium sp.
GCV_H ? VanZ-2
WP_161816816.1
Sediminibacterium roseum
GCV_H ? ? VanZ-2
MBP9133794.1
Saprospiraceae bacterium
GCV_H VanZ-2
MBE1448152.1
GCV_H ? ? VanZ-2
MBC7888802.1
Ferruginibacter sp.
GCV_H VanZ-2
MBS1735514.1
Bacteroidota bacterium
GCV_H VanZ-2
MBS1761960.1
Bacteroidota bacterium
GCV_H VanZ-2
PDH49382.1
Bacteroidetes bacterium MED-G21
GCV_H VanZ-2 linker TonB-C
MBK8599565.1
Flavobacterium sp.
GCV_H VanZ-2
MCC7297367.1
Bacteroidia bacterium
GCV_H VanZ-2
WP_009413537.1
Capnocytophaga genosp. AHN8471
GCV_H VanZ-2 linker TonB-C linker TonB-C(frag) linker TonB-C(frag) linker TonB-C(frag) linker TonB-C linker TonB-C(frag) linker TonB-C(frag) linker TonB-C(frag)
ATA82442.1
Capnocytophaga leadbetteri
GCV_H VanZ-2 linker TonB-C linker TonB-C(frag)
MAZ73759.1
Flavobacteriaceae bacterium
GCV_H VanZ-2 linker TonB-C linker TonB-C TonB-C OmpA-like-beta-barrel
WP_162129533.1
Algibacter luteus
GCV_H VanZ-2 linker TonB-C
MBE7177268.1
Mucilaginibacter polytrichastri
GCV_H VanZ-2
PIE99178.1
Polaribacter sp.
GCV_H VanZ-2 linker TonB-C
MBU3675782.1
Chitinophagaceae bacterium
GCV_H VanZ-2 ? sugar-O-acetyl/acyltransf

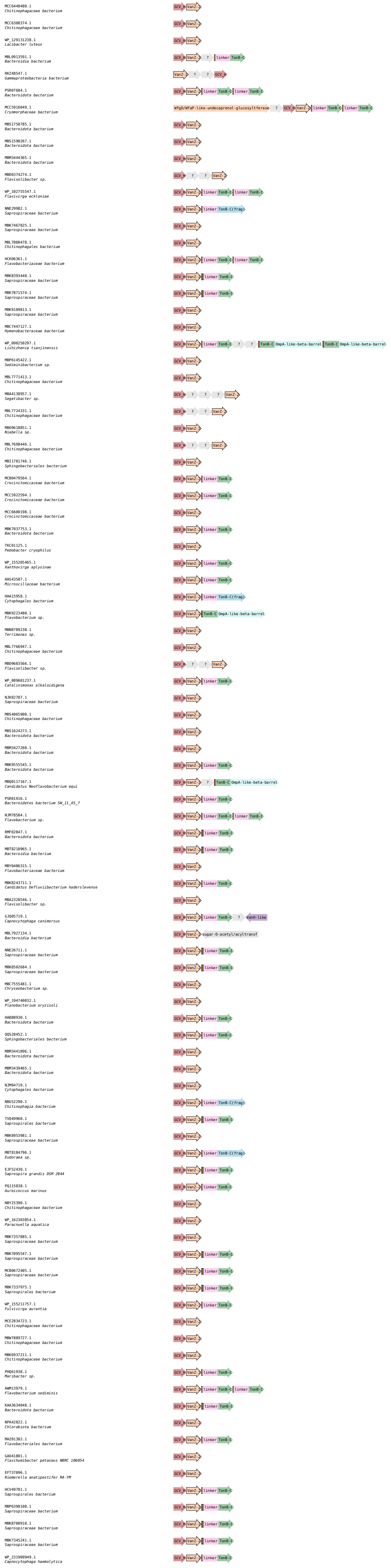
MCC6448480.1
Chitinophagaceae bacterium
GCV_H VanZ-2
MCC6308374.1
Chitinophagaceae bacterium
GCV_H VanZ-2
WP_129131238.1
Lacibacter luteus
GCV_H VanZ-2
MBL0913591.1
Bacteroidia bacterium
GCV_H VanZ-2 ? linker TonB-C
RKZ48547.1
Gammaproteobacteria bacterium
VanZ-2 ? ? GCV_H
PSR07684.1
Bacteroidota bacterium
GCV_H VanZ-2 linker TonB-C linker TonB-C
MCC5916049.1
Cryomorphaceae bacterium
WfgD/WfaP-like-undecaprenol-glucosyltferase ? GCV_H VanZ-2 linker TonB-C linker TonB-C
MBS1758785.1
Bacteroidota bacterium
GCV_H VanZ-2
MBS1590267.1
Bacteroidota bacterium
GCV_H VanZ-2
MBM3444365.1
Bacteroidota bacterium
GCV_H VanZ-2
MBD0374274.1
Flavisolibacter sp.
GCV_H ? ? VanZ-2
WP_102755547.1
Flavivirga eckloniae
GCV_H VanZ-2 linker TonB-C linker TonB-C
NNE29982.1
Saprospiraceae bacterium
GCV_H VanZ-2 linker TonB-C(frag)
MBK7467825.1
Saprospiraceae bacterium
GCV_H VanZ-2
MBL7800478.1
Chitinophagales bacterium
GCV_H VanZ-2
HCK06361.1
Flavobacteriaceae bacterium
GCV_H VanZ-2 linker TonB-C linker TonB-C
MBK8393448.1
Saprospiraceae bacterium
GCV_H VanZ-2 linker TonB-C
MBK7871574.1
Saprospiraceae bacterium
GCV_H VanZ-2 linker TonB-C
MBK9109813.1
Saprospiraceae bacterium
GCV_H VanZ-2
MBC7447127.1
Hymenobacteraceae bacterium
GCV_H VanZ-2
WP_090250297.1
Lishizhenia tianjinensis
GCV_H VanZ-2 linker TonB-C ? ? TonB-C OmpA-like-beta-barrel TonB-C OmpA-like-beta-barrel
MBP6145422.1
Sediminibacterium sp.
GCV_H VanZ-2
MBL7771413.1
Chitinophagaceae bacterium
GCV_H VanZ-2
MBA4138957.1
Segetibacter sp.
GCV_H ? ? ? VanZ-2
MBL7724331.1
Chitinophagaceae bacterium
GCV_H ? ? VanZ-2
MBO9618851.1
Niabella sp.
GCV_H VanZ-2
MBL7698446.1
Chitinophagaceae bacterium
GCV_H ? ? VanZ-2
MBI1781746.1
Sphingobacteriales bacterium
GCV_H VanZ-2
MCB0479564.1
Crocinitomicaceae bacterium
GCV_H VanZ-2 linker TonB-C
MCC5922594.1
Crocinitomicaceae bacterium
GCV_H VanZ-2 linker TonB-C
MCC6600198.1
Crocinitomicaceae bacterium
GCV_H VanZ-2
MBK7037753.1
Bacteroidota bacterium
GCV_H VanZ-2 linker TonB-C
TKC01125.1
Pedobacter cryophilus
GCV_H VanZ-2
WP_155205465.1
Xanthovirga aplysinae
GCV_H VanZ-2 linker TonB-C
HAS43587.1
Microscillaceae bacterium
GCV_H VanZ-2 linker TonB-C
HAA15958.1
Cytophagales bacterium
GCV_H VanZ-2 linker TonB-C(frag)
MBK9223480.1
Flavobacterium sp.
GCV_H VanZ-2 TonB-C OmpA-like-beta-barrel
MBN8789238.1
Terrimonas sp.
GCV_H VanZ-2
MBL7766947.1
Chitinophagaceae bacterium
GCV_H VanZ-2
MBO9683566.1
Flavisolibacter sp.
GCV_H ? ? VanZ-2
WP_089681237.1
Catalinimonas alkaloidigena
GCV_H VanZ-2 linker TonB-C
NJK82707.1
Saprospiraceae bacterium
GCV_H VanZ-2
MBS4065900.1
Chitinophagaceae bacterium
GCV_H VanZ-2
MBS1624273.1
Bacteroidota bacterium
GCV_H VanZ-2
MBM3427260.1
Bacteroidota bacterium
GCV_H VanZ-2
MBK9555545.1
Bacteroidota bacterium
GCV_H VanZ-2 linker TonB-C
MBQ0117167.1
Candidatus Neoflavobacterium equi
GCV_H VanZ-2 ? TonB-C OmpA-like-beta-barrel
PSR01916.1
Bacteroidetes bacterium SW_11_45_7
GCV_H VanZ-2 linker TonB-C
NJM78584.1
Flavobacterium sp.
GCV_H VanZ-2 linker TonB-C linker TonB-C
RMF02847.1
Bacteroidota bacterium
GCV_H VanZ-2 linker TonB-C
MBT8218965.1
Bacteroidia bacterium
GCV_H VanZ-2 linker TonB-C
MBY0486315.1
Flavobacteriaceae bacterium
GCV_H VanZ-2
MBK8243711.1
Candidatus Defluviibacterium haderslevense
GCV_H VanZ-2 linker TonB-C
MBA2328546.1
Flavisolibacter sp.
GCV_H VanZ-2
GJQ05719.1
Capnocytophaga canimorsus
GCV_H VanZ-2 linker TonB-C ? VanH-like
MBL7927134.1
Bacteroidia bacterium
GCV_H VanZ-2 sugar-O-acetyl/acyltransf
NNE26711.1
Saprospiraceae bacterium
GCV_H VanZ-2 linker TonB-C
MBK8502684.1
Saprospiraceae bacterium
GCV_H VanZ-2 linker TonB-C
MBC7555481.1
Chryseobacterium sp.
GCV_H VanZ-2
WP_194740032.1
Planobacterium oryzisoli
GCV_H VanZ-2
HAB88930.1
Bacteroidota bacterium
GCV_H VanZ-2 linker TonB-C
QQS28452.1
Sphingobacteriales bacterium
GCV_H VanZ-2 linker TonB-C
MBM3441896.1
Bacteroidota bacterium
GCV_H VanZ-2
MBM3439465.1
Bacteroidota bacterium
GCV_H VanZ-2
NJM94719.1
Cytophagales bacterium
GCV_H VanZ-2
NBU52290.1
Chitinophagia bacterium
GCV_H VanZ-2 linker TonB-C(frag)
TVQ49960.1
Saprospirales bacterium
GCV_H VanZ-2 linker TonB-C
MBK8953981.1
Saprospiraceae bacterium
GCV_H VanZ-2
MBT8184796.1
Eudoraea sp.
GCV_H VanZ-2 linker TonB-C(frag)
EJF52430.1
Saprospira grandis DSM 2844
GCV_H VanZ-2 linker TonB-C
PQJ15838.1
Aureicoccus marinus
GCV_H VanZ-2 linker TonB-C
NBY25390.1
Chitinophagaceae bacterium
GCV_H VanZ-2
WP_162303854.1
Paracnuella aquatica
GCV_H VanZ-2
MBK7357885.1
Saprospiraceae bacterium
GCV_H VanZ-2
MBK7095547.1
Saprospiraceae bacterium
GCV_H VanZ-2 linker TonB-C
MCB0672405.1
Saprospiraceae bacterium
GCV_H VanZ-2 linker TonB-C
MBK7337975.1
Saprospirales bacterium
GCV_H VanZ-2 linker TonB-C
WP_155211757.1
Fulvivirga aurantia
GCV_H VanZ-2 linker TonB-C
MCE2834723.1
Chitinophagaceae bacterium
GCV_H VanZ-2
MBW7889727.1
Chitinophagaceae bacterium
GCV_H VanZ-2
MBK6937211.1
Chitinophagaceae bacterium
GCV_H VanZ-2
PHQ61938.1
Maribacter sp.
GCV_H VanZ-2 linker TonB-C
AWM13979.1
Flavobacterium sediminis
GCV_H VanZ-2 linker TonB-C linker TonB-C
KAA3634048.1
Bacteroidota bacterium
GCV_H VanZ-2 linker TonB-C
NPA42822.1
Chlorobiota bacterium
GCV_H VanZ-2
MAZ01382.1
Flavobacteriales bacterium
GCV_H VanZ-2 linker TonB-C
GAO41801.1
Flavihumibacter petaseus NBRC 106054
GCV_H VanZ-2
EFT37096.1
Riemerella anatipestifer RA-YM
GCV_H VanZ-2
HCV49781.1
Saprospirales bacterium
GCV_H VanZ-2 linker TonB-C
MBP6398108.1
Saprospiraceae bacterium
GCV_H VanZ-2 linker TonB-C
MBK8700910.1
Saprospiraceae bacterium
GCV_H VanZ-2 linker TonB-C
MBK7345241.1
Saprospiraceae bacterium
GCV_H VanZ-2 linker TonB-C
WP_231909949.1
Capnocytophaga haemolytica
GCV_H VanZ-2 linker TonB-C

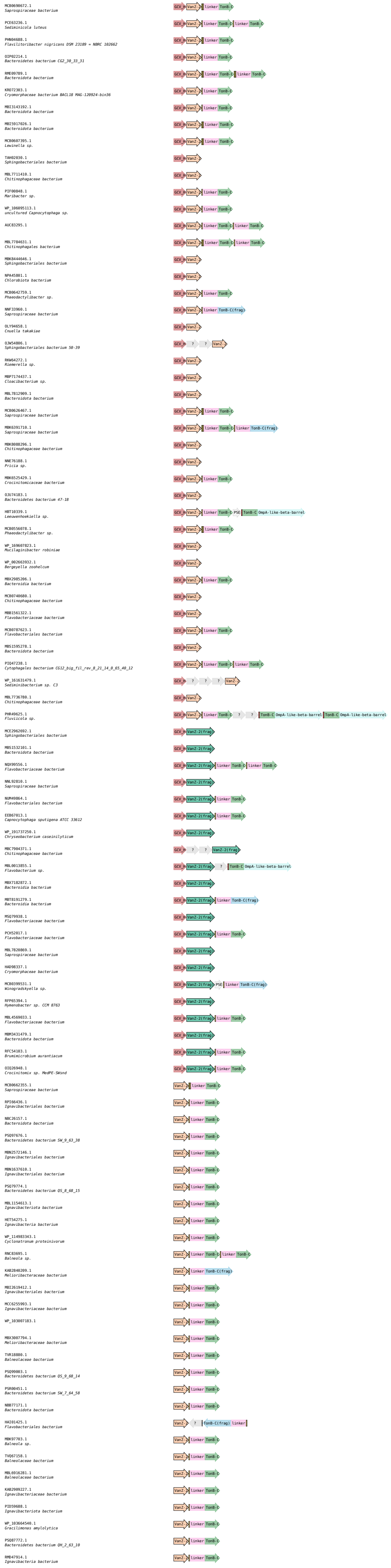
MCB0690672.1
Saprospiraceae bacterium
PCE63236.1
Sediminicola luteus
PHN04688.1
Flavilitoribacter nigricans DSM 23189 = NBRC 102662
OIP02214.1
Bacteroidetes bacterium CG2_30_33_31
RME09789.1
Bacteroidota bacterium
KRO72303.1
Cryomorphaceae bacterium BACL18 MAG-120924-bin36
MBI3143192.1
Bacteroidota bacterium
MBI5917026.1
Bacteroidota bacterium
MCB0607395.1
Lewinella sp.
TAH02030.1
Sphingobacteriales bacterium
MBL7711410.1
Chitinophagaceae bacterium
PIF00848.1
Maribacter sp.
WP_106095113.1
uncultured Capnocytophaga sp.
AUC83295.1
MBL7784631.1
Chitinophagales bacterium
MBK8444646.1
Sphingobacteriales bacterium
NPA45801.1
Chlorobiota bacterium
MCB0642759.1
Phaeodactylibacter sp.
NNF33960.1
Saprospiraceae bacterium
OLY94658.1
Cnuella takakiae
OJW54806.1
Sphingobacteriales bacterium 50-39
RKW64272.1
Riemerella sp.
MBP7174437.1
Cloacibacterium sp.
MBL7812909.1
Bacteroidota bacterium
MCB0626467.1
Saprospiraceae bacterium
MBK6391710.1
Saprospiraceae bacterium
MBK8088296.1
Chitinophagaceae bacterium
NNE76188.1
Pricia sp.
MBK6525429.1
Crocinitomicaceae bacterium
OJU74183.1
Bacteroidetes bacterium 47-18
HBT10339.1
Leeuwenhoekiella sp.
MCB0556078.1
Phaeodactylibacter sp.
WP_169607823.1
Mucilaginibacter robiniae
WP_002663932.1
Bergeyella zoohelcum
MBX2985206.1
Bacteroidia bacterium
MCB0740680.1
Chitinophagaceae bacterium
MBB1561322.1
Flavobacteriaceae bacterium
MCB0787623.1
Flavobacteriales bacterium
MBS1595278.1
Bacteroidota bacterium
PIQ47238.1
Cytophagales bacterium CG12_big_fil_rev_8_21_14_0_65_40_12
WP_161631479.1
Sediminibacterium sp. C3
MBL7736780.1
Chitinophagaceae bacterium
PHR49625.1
Fluviicola sp.
MCE2962692.1
Sphingobacteriales bacterium
MBS1532101.1
Bacteroidota bacterium
NQX99556.1
Flavobacteriaceae bacterium
NNL92810.1
Saprospiraceae bacterium
NUM49864.1
Flavobacteriales bacterium
EEB67013.1
Capnocytophaga sputigena ATCC 33612
WP_191737250.1
Chryseobacterium caseinilyticum
MBC7904371.1
Chitinophagaceae bacterium
MBL0013855.1
Flavobacterium sp.
MBX7182872.1
Bacteroidia bacterium
MBT8191279.1
Bacteroidia bacterium
MSQ79938.1
Flavobacteriaceae bacterium
PCH52017.1
Flavobacteriaceae bacterium
MBL7820869.1
Saprospiraceae bacterium
HAD98337.1
Cryomorphaceae bacterium
MCB0399531.1
Winogradskyella sp.
RFP65394.1
Hymenobacter sp. CCM 8763
MBL4569033.1
Flavobacteriaceae bacterium
MBM3431479.1
Bacteroidota bacterium
RFC54103.1
Brumimicrobium aurantiacum
OIQ26948.1
Crocinitomix sp. MedPE-SWsnd
MCB0662355.1
Saprospiraceae bacterium
RPI66436.1
Ignavibacteriales bacterium
NBC26157.1
Bacteroidota bacterium
PSQ97676.1
Bacteroidetes bacterium SW_9_63_38
MBN2572146.1
Ignavibacteriales bacterium
MBN1637610.1
Ignavibacteriales bacterium
PSQ79774.1
Bacteroidetes bacterium QS_8_68_15
MBL1154613.1
Ignavibacteriota bacterium
HET54275.1
Ignavibacteria bacterium
WP_114983343.1
Cyclonatronum proteinivorum
RNC83695.1
Balneola sp.
KAB2840209.1
Melioribacteraceae bacterium
MBI2619412.1
Ignavibacteriales bacterium
MCC6255993.1
Ignavibacteriaceae bacterium
WP_103007183.1
MBX3007794.1
Melioribacteraceae bacterium
TVR18880.1
Balneolaceae bacterium
PSQ99003.1
Bacteroidetes bacterium QS_9_68_14
PSR00451.1
Bacteroidetes bacterium SW_7_64_58
NBB77171.1
Bacteroidota bacterium
HAI01425.1
Flavobacteriales bacterium
MBK97703.1
Balneola sp.
TVQ67158.1
Balneolaceae bacterium
MBL6916281.1
Balneolaceae bacterium
KAB2909227.1
Ignavibacteriaceae bacterium
PID59688.1
Ignavibacteriota bacterium
WP_103664540.1
Gracilimonas amylolytica
PSQ87772.1
Bacteroidetes bacterium QH_2_63_10
RMD47914.1
Ignavibacteria bacterium

NLF41881.1	*Bacteroidales bacterium*	VanZ-2 linker TonB-C
MCC5905474.1	*Balneolaceae bacterium*	VanZ-2 linker TonB-C
GBD89590.1	*bacterium BMS3Abin04*	VanZ-2 linker TonB-C
KXK36929.1	*Bacteroidetes bacterium OLB9*	VanZ-2 linker TonB-C
GJQ22010.1	*Bacteroidia bacterium*	VanZ-2 linker TonB-C
CAG0939432.1	*Candidatus Brocadiaceae bacterium*	VanZ-2 linker TonB-C
MBI9070341.1	*Melioribacteraceae bacterium*	VanZ-2 linker TonB-C
WP_014560720.1	*Ignavibacterium album JCM 16511*	VanZ-2 linker TonB-C
MBI5663068.1	*Ignavibacterium album*	VanZ-2 linker TonB-C linker TonB-C
MBK8624398.1	*Saprospiraceae bacterium*	VanZ-2 linker TonB-C
MBX2976296.1	*Ignavibacteriaceae bacterium*	VanZ-2 linker TonB-C
MAC04938.1	*Balneola sp.*	VanZ-2 linker TonB-C linker TonB-C
MAL19252.1	*Balneola sp.*	VanZ-2 linker TonB-C
NCS89978.1	*Ignavibacteria bacterium*	VanZ-2 linker TonB-C
MBT8378568.1	*Ignavibacteria bacterium*	VanZ-2 linker TonB-C
NIT60887.1	*Fodinibius sp.*	VanZ-2 linker TonB-C
NWG28542.1	*Ignavibacteriaceae bacterium*	VanZ-2 linker TonB-C
MBZ0199793.1	*Ignavibacteriaceae bacterium*	VanZ-2 linker TonB-C
MCC6549406.1	*Ignavibacteriaceae bacterium*	VanZ-2 linker TonB-C
NBB75070.1	*Bacteroidota bacterium*	VanZ-2 linker TonB-C
NBV31133.1	*Bacteroidota bacterium*	VanZ-2 linker TonB-C
NLH60432.1	*Ignavibacteriales bacterium*	VanZ-2 linker TonB-C
HHD21969.1		VanZ-2 linker TonB-C(frag)
PIQ62278.1	*Bacteroidetes bacterium CG12_big_fil_rev_8_21_14_0_65_60_17*	VanZ-2 linker TonB-C
WP_101072864.1	*Rhodohalobacter barkolensis*	VanZ-2 linker TonB-C
TVR34720.1	*Balneolaceae bacterium*	VanZ-2 linker TonB-C
OGU29573.1	*Ignavibacteria bacterium GWA2_35_9*	VanZ-2 linker TonB-C
NBP81114.1	*bacterium*	VanZ-2 linker TonB-C(frag)
PIW69949.1	*Ignavibacteriales bacterium CG12_big_fil_rev_8_21_14_0_65_30_8*	VanZ-2 linker TonB-C
WP_012500964.1	*Chloroherpeton thalassium ATCC 35110*	linker TonB-C ? VanZ-2
TNE73968.1	*bacterium*	VanZ-2 linker TonB-C linker TonB-C
HAC16040.1	*Bacteroidota bacterium*	VanZ-2 linker TonB-C(frag)
MBN2731843.1	*Balneolaceae bacterium*	VanZ-2 linker TonB-C linker TonB-C
MBC8147033.1	*Bacteroidota bacterium*	VanZ-2 linker TonB-C
NBB99870.1	*Bacteroidota bacterium*	VanZ-2 linker TonB-C
MBM4169466.1	*Ignavibacteria bacterium*	VanZ-2 linker TonB-C
HED08897.1	*Ignavibacteria bacterium*	VanZ-2 linker TonB-C
MBK8621884.1	*Saprospiraceae bacterium*	VanZ-2 linker TonB-C
PSR03155.1	*Bacteroidetes bacterium QS_8_68_28*	VanZ-2 linker TonB-C
HFS67394.1	*Flavobacteriia bacterium*	VanZ-2 linker TonB-C linker TonB-C
NTV46145.1	*Chlorobiales bacterium*	linker TonB-C ? VanZ-2
MBK9459670.1	*Sphingobacteriales bacterium*	VanZ-2 linker TonB-C linker TonB-C
RCL67940.1	*Rhodothermaeota bacterium MED-G64*	VanZ-2 linker TonB-C
NTW49765.1	*Chlorobiales bacterium*	linker TonB-C ? VanZ-2
HHB79837.1	*Saprospiraceae bacterium*	VanZ-2 linker TonB-C
TDI71613.1	*Bacteroidota bacterium*	VanZ-2 linker TonB-C(frag) linker TonB-C
MBC8044143.1	*Chlorobiales bacterium*	linker TonB-C ? VanZ-2
HCD23624.1	*Flavobacteriaceae bacterium*	VanZ-2 linker TonB-C
MBI4417921.1	*Ignavibacteriales bacterium*	VanZ-2 linker TonB-C
MBT8386900.1	*Ignavibacteria bacterium*	VanZ-2 linker TonB-C
NNE48455.1	*Rhodothermales bacterium*	VanZ-2 linker TonB-C
PSQ89881.1	*Bacteroidetes bacterium QS_8_64_10*	VanZ-2 ? linker TonB-C
HFC01324.1	*Phaeodactylibacter sp.*	VanZ-2 linker TonB-C
MCD8528436.1	*Chitinophagales bacterium*	VanZ-2 linker TonB-C
MCB0537436.1	*Bacteroidota bacterium*	VanZ-2 linker TonB-C
MBT7704193.1	*Polaribacter sp.*	VanZ-2 linker TonB-C linker TonB-C
PCH67426.1	*Bacteroidota bacterium*	VanZ-2 linker TonB-C
SHL07890.1	*Rhodothermus profundi*	VanZ-2 linker TonB-C
MBK6362068.1	*Saprospiraceae bacterium*	VanZ-2 linker TonB-C
MBI3112153.1	*Ignavibacteriales bacterium*	VanZ-2 linker TonB-C
PAP81802.1	*Rubrivirga sp. SAORIC476*	VanZ-2 linker TonB-C linker TonB-C
MBX2871646.1	*Saprospiraceae bacterium*	VanZ-2 linker TonB-C
MBI1930909.1	*Ignavibacteriales bacterium*	VanZ-2 linker TonB-C linker TonB-C
MBT7242561.1	*Flavobacteriaceae bacterium*	VanZ-2 linker TonB-C
MBU1680396.1	*Bacteroidota bacterium*	VanZ-2 linker TonB-C
NMB82007.1	*Ignavibacteria bacterium*	VanZ-2 linker TonB-C
MCC5915207.1	*Balneolaceae bacterium*	VanZ-2 linker TonB-C
MBU1095384.1	*Bacteroidota bacterium*	VanZ-2(frag) linker TonB-C
MBL4586793.1	*Flavobacteriales bacterium*	VanZ-2(frag) ? linker TonB-C
SMO43141.1	*Gracilimonas mengyeensis*	VanZ-2(frag) linker TonB-C linker TonB-C
MCB0754477.1	*Flavobacteriales bacterium*	VanZ-2(frag) linker TonB-C linker TonB-C linker TonB-C(frag) TonB-C OmpA-like-beta-barrel TonB-C OmpA-like-beta-barrel
GJM33681.1	*Saprospiraceae bacterium*	VanZ-2(frag) linker TonB-C
MBI1939132.1	*Ignavibacteriales bacterium*	VanZ-2 linker TonB-C
NOX64942.1	*Chlorobiota bacterium*	VanZ-2 linker TonB-C
MBN1300439.1	*Melioribacteraceae bacterium*	VanZ-2 linker TonB-C
MBK7629584.1	*Ignavibacteriales bacterium*	VanZ-2 linker TonB-C
RJP59496.1	*Ignavibacteriales bacterium*	VanZ-2 linker TonB-C
GBD89187.1	*bacterium BMS3Abin03*	VanZ-2 linker TonB-C
MBZ0178830.1	*Melioribacteraceae bacterium*	VanZ-2 linker TonB-C
MBZ0183373.1	*Melioribacteraceae bacterium*	VanZ-2 linker TonB-C

#;DUF218 also in the context

TVR78628.1	*Chitinophagaceae bacterium*	DUF218 GCV_H VanZ-2 linker TonB-C linker TonB-C
NOX17310.1	*Chlorobiota bacterium*	VanZ-2 linker TonB-C ? DUF218
NLT50987.1	*Ignavibacteria bacterium*	VanZ-2 linker TonB-C ? DUF218
MBM4172030.1	*Ignavibacteria bacterium*	VanZ-2 linker TonB-C ? DUF218
GJQ64459.1	*Melioribacteraceae bacterium*	VanZ-2 linker TonB-C ? ? DUF218
MBK7106218.1	*Ignavibacteriota bacterium*	VanZ-2 linker TonB-C linker TonB-C ? DUF218
KUO62439.1	*bacterium BRH_c32*	VanZ-2 linker TonB-C DUF218
MBS4036244.1	*Ignavibacterium sp.*	VanZ-2 linker TonB-C DUF218
QQS38108.1	*Ignavibacteriales bacterium*	VanZ-2 linker TonB-C DUF218
MBL1211776.1	*Ignavibacteriota bacterium*	VanZ-2 linker TonB-C linker TonB-C
NWF50874.1	*Ignavibacteriaceae bacterium*	VanZ-2 linker TonB-C DUF218
MBP9119326.1	*Ignavibacterium sp.*	VanZ-2 linker TonB-C DUF218

MBU1100838.1
Bacteroidota bacterium

MBU0475511.1
Bacteroidota bacterium

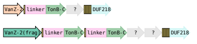

#;Patescibacterial-predominant association combining a peptidase (either Hybrid or Papain-like, fused to the same coiled-coil region), with a Lamin-Tail-Domain (IG-like), and VanZ. there is sometimes a further polysaccharide synthesis module (or parts of one) downstream

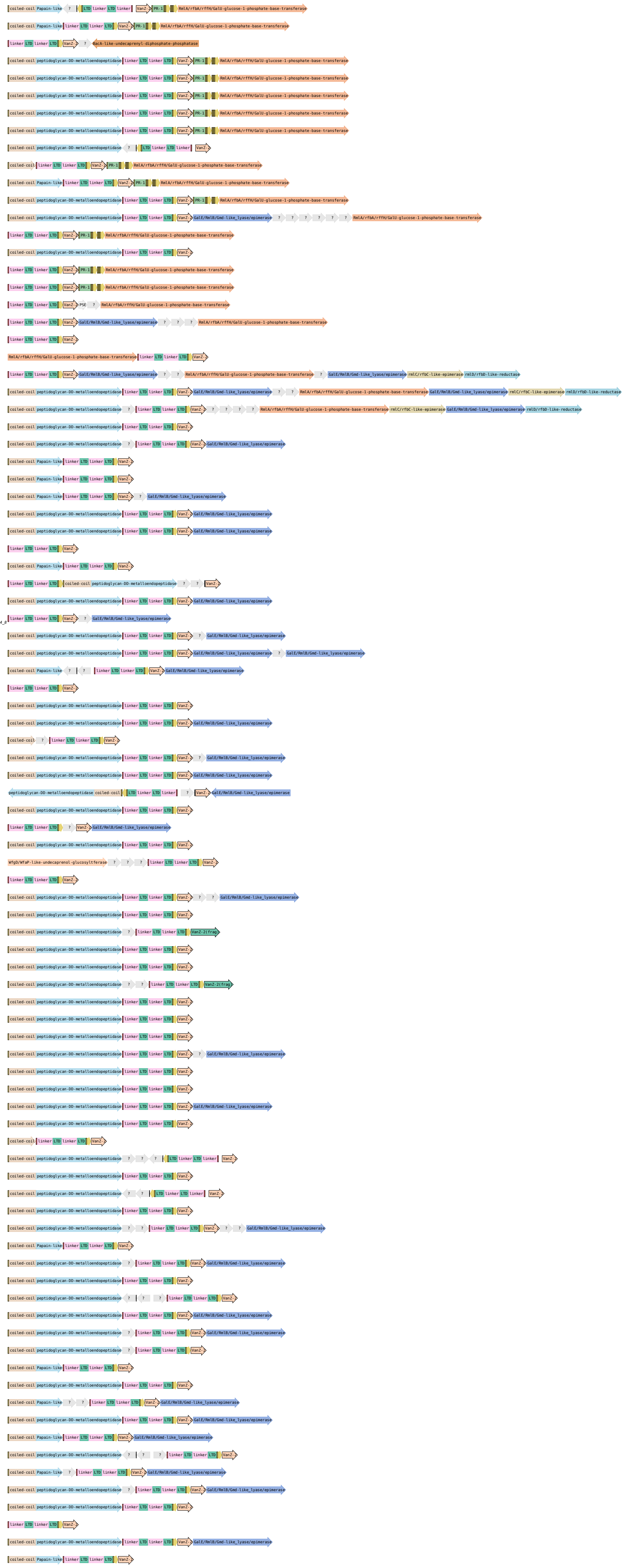

PIY71612.1
Candidatus Roizmanbacteria bacterium CG_4_10_14_0_8_um_filter_33_9

NMB84206.1
Candidatus Roizmanbacteria bacterium

NCN87770.1
Candidatus Paceibacterota bacterium

OGK18235.1
Candidatus Roizmanbacteria bacterium RIFCSPHIGHO2_01_FULL_39_8

OGK18574.1
Candidatus Roizmanbacteria bacterium RIFCSPHIGHO2_01_FULL_40_98

PIZ64014.1
Candidatus Roizmanbacteria bacterium CG_4_10_14_0_2_um_filter_39_13

OGK13262.1
Candidatus Roizmanbacteria bacterium RIFCSPHIGHO2_01_FULL_38_15

PIZ64791.1
Candidatus Roizmanbacteria bacterium CG_4_10_14_0_2_um_filter_36_9

OGG24394.1
Candidatus Gottesmanbacteria bacterium RIFCSPLOWO2_01_FULL_43_11b

OGK62015.1
Candidatus Roizmanbacteria bacterium RIFOXYA2_FULL_38_14

QQG44539.1
Candidatus Roizmanbacteria bacterium

OGK21236.1
Candidatus Roizmanbacteria bacterium RIFCSPHIGHO2_02_FULL_37_13b

KKP38503.1
Candidatus Woesebacteria bacterium GW2011_GWC2_33_12

OGK18939.1
Candidatus Roizmanbacteria bacterium RIFCSPHIGHO2_01_FULL_39_24

OGD90051.1
Candidatus Curtissbacteria bacterium RIFCSPHIGHO2_02_FULL_42_15

OGK14550.1
Candidatus Roizmanbacteria bacterium RIFCSPHIGHO2_01_FULL_37_16b

MBP6045091.1
Candidatus Woesebacteria bacterium

OGK15373.1
Candidatus Roizmanbacteria bacterium RIFCSPHIGHO2_01_FULL_39_12c

KKU09145.1
Candidatus Woesebacteria bacterium GW2011_GWB1_45_5

KKS87906.1
Parcubacteria group bacterium GW2011_GWC1_43_11

MBT4123891.1
Candidatus Paceibacterota bacterium

EKE12305.1
groundwater metagenome

KKQ91887.1
Candidatus Woesebacteria bacterium GW2011_GWB1_39_10

MBI5620980.1
Candidatus Gottesmanbacteria bacterium

QQS38703.1
Candidatus Woesebacteria bacterium

NMC36493.1
Candidatus Beckwithbacteria bacterium

KKQ95820.1
Candidatus Woesebacteria bacterium GW2011_GWA1_39_11b

RLC30880.1
Candidatus Woesebacteria bacterium

MBU0572453.1
Patescibacteria group bacterium

OQA93068.1
Microgenomates group bacterium ADurb.Bin219

OGM60697.1
Candidatus Woesebacteria bacterium RIFCSPLOWO2_01_FULL_39_10b

OGM21406.1
Candidatus Woesebacteria bacterium RIFCSPHIGHO2_01_FULL_38_9

MBP6994301.1
Candidatus Woesebacteria bacterium

OGM27149.1
Candidatus Woesebacteria bacterium RIFCSPHIGHO2_01_FULL_39_28

KKQ51325.1
Microgenomates group bacterium GW2011_GWC1_38_12

PIP52940.1
Candidatus Beckwithbacteria bacterium CG23_combo_of_CG06-09_8_20_14_all_34_8

MBN1168654.1
Candidatus Woesebacteria bacterium

KKR10709.1
Candidatus Woesebacteria bacterium GW2011_GWA1_39_21

PIS15330.1
Candidatus Shapirobacteria bacterium CG09_land_8_20_14_0_10_38_17

MBI2103782.1
Candidatus Woesebacteria bacterium

RJR29616.1
Candidatus Microgenomates bacterium

KKQ97445.1
Candidatus Woesebacteria bacterium GW2011_GWA1_39_12

KKR71071.1
Candidatus Woesebacteria bacterium GW2011_GWA2_40_7b

OGM25614.1
Candidatus Woesebacteria bacterium RIFCSPHIGHO2_01_FULL_39_95

NMB56833.1
Candidatus Beckwithbacteria bacterium

EKD62355.1
groundwater metagenome

EKE04954.1
groundwater metagenome

OGM61645.1
Candidatus Woesebacteria bacterium RIFCSPLOWO2_01_FULL_39_21

KKT85324.1
Parcubacteria group bacterium GW2011_GWA1_Parcubacteria_45_10

MBU0579519.1
Patescibacteria group bacterium

OGM76425.1
Candidatus Woesebacteria bacterium RIFOXYA1_FULL_40_18

MBM3205499.1
Candidatus Shapirobacteria bacterium

MBU3957293.1
Patescibacteria group bacterium

KKQ25217.1
Microgenomates group bacterium GW2011_GWC1_37_12b

OGG30002.1

OGG31579.1
Candidatus Gottesmanbacteria bacterium RIFCSPLOWO2_01_FULL_46_9

MBP7875618.1
Candidatus Woesebacteria bacterium

MBI2029701.1
Candidatus Gottesmanbacteria bacterium

OGG10534.1
Candidatus Gottesmanbacteria bacterium RIFCSPHIGHO2_01_FULL_43_15

MBN1263492.1
Candidatus Paceibacterota bacterium

OGM25255.1
Candidatus Woesebacteria bacterium RIFCSPHIGHO2_01_FULL_39_32

OGM57161.1
Candidatus Woesebacteria bacterium RIFCSPHIGHO2_12_FULL_46_16

KKT58509.1
Candidatus Amesbacteria bacterium GW2011_GWA1_44_24

AKM82873.1
Candidatus Woesebacteria bacterium GW2011_GWF1_31_35

KKP57446.1
Candidatus Gottesmanbacteria bacterium GW2011_GWA1_34_13

OGY14042.1
Candidatus Blackburnbacteria bacterium RIFCSPLOWO2_01_FULL_44_43

MBI4034819.1
Candidatus Chisholmbacteria bacterium

OGY16135.1
Candidatus Chisholmbacteria bacterium RIFCSPHIGHO2_01_FULL_49_18

KKR10880.1
Microgenomates group bacterium GW2011_GWA2_39_19

OGY18640.1
Candidatus Chisholmbacteria bacterium RIFCSPHIGHO2_01_FULL_52_32

OGD79027.1
Candidatus Collierbacteria bacterium RIFOXYB1_FULL_49_13

MCD6225706.1
bacterium

PIU02382.1
Candidatus Shapirobacteria bacterium CG09_land_8_20_14_0_10_49_15

NCN82571.1
Candidatus Paceibacterota bacterium

OGC98814.1
Candidatus Amesbacteria bacterium RIFCSPHIGHO2_01_FULL_47_34

HGI41181.1

KKP48244.1
Candidatus Woesebacteria bacterium GW2011_GWA2_33_28

MBM3208818.1
Candidatus Shapirobacteria bacterium

MBP7842613.1
Candidatus Woesebacteria bacterium

NCN06575.1
Candidatus Paceibacterota bacterium

RIK51215.1
Candidatus Microgenomates bacterium

HGP23976.1

MBP9670236.1
Candidatus Woesebacteria bacterium

KKU57015.1
Candidatus Amesbacteria bacterium GW2011_GWA2_47_11

KKU26284.1
Microgenomates group bacterium GW2011_GWC1_46_16

KKU87320.1
Microgenomates group bacterium GW2011_GWF2_47_9

MBP9781595.1
Candidatus Woesebacteria bacterium

MCB9801742.1
Pseudomonadales bacterium

AKM79579.1
Candidatus Beckwithbacteria bacterium GW2011_GWC1_49_16

MBU0974517.1
Patescibacteria group bacterium

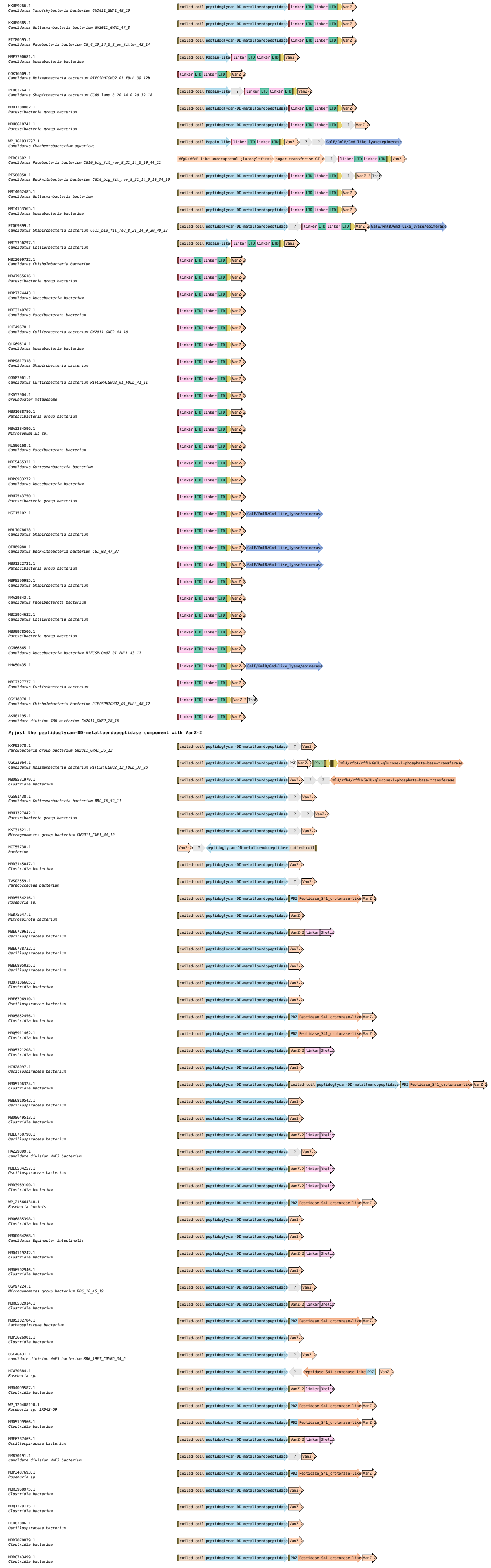
KKU89266.1
Candidatus Yanofskybacteria bacterium GW2011_GWA1_48_10
KKU80885.1
Candidatus Gottesmanbacteria bacterium GW2011_GWA1_47_8
PIY80595.1
Candidatus Pacebacteria bacterium CG_4_10_14_0_8_um_filter_42_14
MBP7700681.1
Candidatus Woesebacteria bacterium
OGK16609.1
Candidatus Roizmanbacteria bacterium RIFCSPHIGHO2_01_FULL_39_12b
PIU03764.1
Candidatus Shapirobacteria bacterium CG08_land_8_20_14_0_20_39_18
MBU1200802.1
Patescibacteria group bacterium
MBU0618741.1
Patescibacteria group bacterium
WP_161931797.1
Candidatus Chazhemtobacterium aquaticus
PIR61692.1
Candidatus Pacebacteria bacterium CG10_big_fil_rev_8_21_14_0_10_44_11
PIS08850.1
Candidatus Beckwithbacteria bacterium CG10_big_fil_rev_8_21_14_0_10_34_10
MBI4062485.1
Candidatus Gottesmanbacteria bacterium
MBI4153565.1
Candidatus Woesebacteria bacterium
PIQ69899.1
Candidatus Shapirobacteria bacterium CG11_big_fil_rev_8_21_14_0_20_40_12
MBI5356297.1
Candidatus Collierbacteria bacterium
MBI2009722.1
Candidatus Chisholmbacteria bacterium
MBW7955616.1
Patescibacteria group bacterium
MBP7774443.1
Candidatus Woesebacteria bacterium
MBT3249707.1
Candidatus Paceibacterota bacterium
KKT49670.1
Candidatus Collierbacteria bacterium GW2011_GWC2_44_18
QLG69614.1
Candidatus Woesebacteria bacterium
MBP9817318.1
Candidatus Shapirobacteria bacterium
OGD87061.1
Candidatus Curtissbacteria bacterium RIFCSPHIGHO2_01_FULL_41_11
EKD57904.1
groundwater metagenome
MBU1088786.1
Patescibacteria group bacterium
MBA3284596.1
Nitrosopumilus sp.
NLG06168.1
Candidatus Paceibacterota bacterium
MBI5465321.1
Candidatus Gottesmanbacteria bacterium
MBP6933272.1
Candidatus Woesebacteria bacterium
MBU2543750.1
Patescibacteria group bacterium
HGT15102.1
MBL7078628.1
Candidatus Shapirobacteria bacterium
OIN89980.1
Candidatus Beckwithbacteria bacterium CG1_02_47_37
MBU1322721.1
Patescibacteria group bacterium
MBP8590985.1
Candidatus Shapirobacteria bacterium
NMA29843.1
Candidatus Paceibacterota bacterium
MBI3954632.1
Candidatus Collierbacteria bacterium
MBU0978506.1
Patescibacteria group bacterium
OGM66665.1
Candidatus Woesebacteria bacterium RIFCSPLOWO2_01_FULL_43_11
HHA50435.1
MBI2327737.1
Candidatus Curtissbacteria bacterium
OGY18076.1
Candidatus Chisholmbacteria bacterium RIFCSPHIGHO2_01_FULL_48_12
AKM81195.1
candidate division TM6 bacterium GW2011_GWF2_28_16
#;just the peptidoglycan-DD-metalloendopeptidase component with VanZ-2
KKP93978.1
Parcubacteria group bacterium GW2011_GWA1_36_12
OGK33064.1
Candidatus Roizmanbacteria bacterium RIFCSPHIGHO2_12_FULL_37_9b
MBQ8531979.1
Clostridia bacterium
OGG01438.1
Candidatus Gottesmanbacteria bacterium RBG_16_52_11
MBU1327442.1
Patescibacteria group bacterium
KKT31621.1
Microgenomates group bacterium GW2011_GWF1_44_10
NCT55738.1
bacterium
MBR3145047.1
Clostridia bacterium
TVS02559.1
Paracoccaceae bacterium
MBD5554216.1
Roseburia sp.
HEB75647.1
Nitrospirota bacterium
MBE6729617.1
Oscillospiraceae bacterium
MBE6738732.1
Oscillospiraceae bacterium
MBE6805035.1
Oscillospiraceae bacterium
MBQ7106665.1
Clostridia bacterium
MBE6796910.1
Oscillospiraceae bacterium
MBO5852456.1
Clostridia bacterium
MBQ5911462.1
Clostridia bacterium
MBO5321208.1
Clostridia bacterium
HCH28097.1
Oscillospiraceae bacterium
MBO5106324.1
Clostridia bacterium
MBE6810542.1
Oscillospiraceae bacterium
MBQ8649513.1
Clostridia bacterium
MBE6750790.1
Oscillospiraceae bacterium
HAZ29899.1
candidate division WWE3 bacterium
MBE6534257.1
Oscillospiraceae bacterium
MBR3969100.1
Clostridia bacterium
WP_215664348.1
Roseburia hominis
MBQ6885398.1
Clostridia bacterium
MBQ0084268.1
Candidatus Equinaster intestinalis
MBQ4119242.1
Clostridia bacterium
MBR6502946.1
Clostridia bacterium
OGV97224.1
Microgenomates group bacterium RBG_16_45_19
MBR6532914.1
Clostridia bacterium
MBO5302784.1
Lachnospiraceae bacterium
MBP3626901.1
Clostridia bacterium
OGC46431.1
candidate division WWE3 bacterium RBG_19FT_COMBO_34_6
HCW30884.1
Roseburia sp.
MBR4099587.1
Clostridia bacterium
WP_120408190.1
Roseburia sp. 1XD42-69
MBO5199966.1
Clostridia bacterium
MBE6787465.1
Oscillospiraceae bacterium
NMB70191.1
candidate division WWE3 bacterium
MBP3487693.1
Roseburia sp.
MBR3960975.1
Clostridia bacterium
MBQ1279115.1
Clostridia bacterium
HCD82086.1
Oscillospiraceae bacterium
MBR7070879.1
Clostridia bacterium
MBR6743499.1
Clostridia bacterium

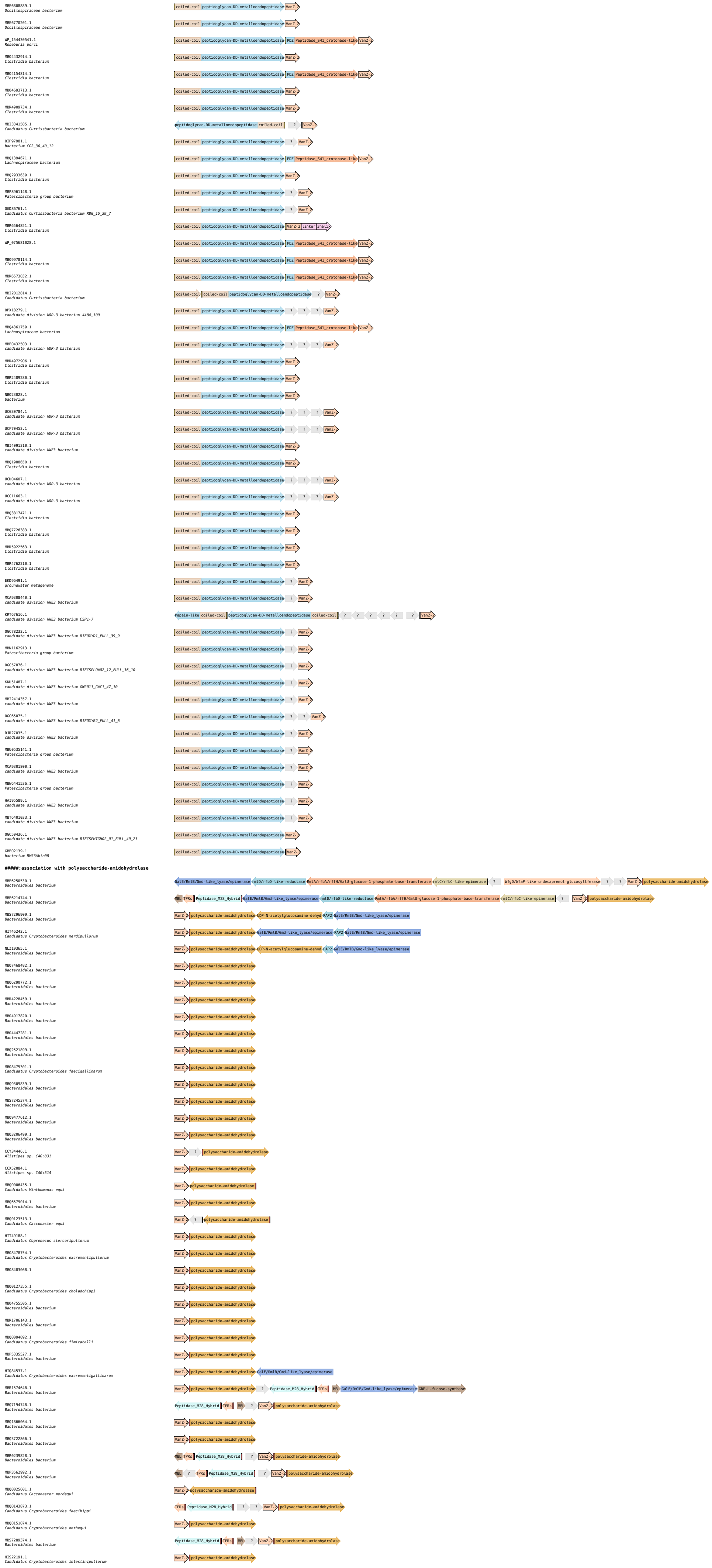
MBE6808889.1
Oscillospiraceae bacterium
MBE6770201.1
Oscillospiraceae bacterium
WP_154430541.1
Roseburia porci
MBO4432914.1
Clostridia bacterium
MBQ4154814.1
Clostridia bacterium
MBO4693713.1
Clostridia bacterium
MBR4909734.1
Clostridia bacterium
MBI3341585.1
Candidatus Curtissbacteria bacterium
OIP97981.1
bacterium CG2_30_40_12
MBQ1394671.1
Lachnospiraceae bacterium
MBQ2933639.1
Clostridia bacterium
MBP8961148.1
Patescibacteria group bacterium
OGD86761.1
Candidatus Curtissbacteria bacterium RBG_16_39_7
MBR6564851.1
Clostridia bacterium
WP_075681028.1
MBQ9978114.1
Clostridia bacterium
MBR6573032.1
Clostridia bacterium
MBI2012814.1
Candidatus Curtissbacteria bacterium
OPX18279.1
candidate division WOR-3 bacterium 4484_100
MBQ4361759.1
Lachnospiraceae bacterium
MBE0432503.1
candidate division WOR-3 bacterium
MBR4972906.1
Clostridia bacterium
MBR2489280.1
Clostridia bacterium
NBO23028.1
bacterium
UCG30784.1
candidate division WOR-3 bacterium
UCF70453.1
candidate division WOR-3 bacterium
MBI4091310.1
candidate division WWE3 bacterium
MBQ1988650.1
Clostridia bacterium
UCD04607.1
candidate division WOR-3 bacterium
UCC11663.1
candidate division WOR-3 bacterium
MBQ3817471.1
Clostridia bacterium
MBQ7726383.1
Clostridia bacterium
MBR5922563.1
Clostridia bacterium
MBR4762210.1
Clostridia bacterium
EKD96491.1
groundwater metagenome
MCA9308440.1
candidate division WWE3 bacterium
KRT67616.1
candidate division WWE3 bacterium CSP1-7
OGC78232.1
candidate division WWE3 bacterium RIFOXYD1_FULL_39_9
MBN1162913.1
Patescibacteria group bacterium
OGC57876.1
candidate division WWE3 bacterium RIFCSPLOWO2_12_FULL_36_10
KKU51487.1
candidate division WWE3 bacterium GW2011_GWC1_47_10
MBI2414357.1
candidate division WWE3 bacterium
OGC65075.1
candidate division WWE3 bacterium RIFOXYB2_FULL_41_6
RJR27035.1
candidate division WWE3 bacterium
MBU0535141.1
Patescibacteria group bacterium
MCA9301800.1
candidate division WWE3 bacterium
MBW6441536.1
Patescibacteria group bacterium
HAI95589.1
candidate division WWE3 bacterium
MBT6401033.1
candidate division WWE3 bacterium
OGC50436.1
candidate division WWE3 bacterium RIFCSPHIGHO2_01_FULL_40_23
GBE02139.1
bacterium BMS3Abin08
#####;association with polysaccharide-amidohydrolase
MBE6250530.1
Bacteroidales bacterium
MBE6214744.1
Bacteroidales bacterium
MBS7296909.1
Bacteroidales bacterium
HIT46242.1
Candidatus Cryptobacteroides merdipullorum
NLZ19365.1
Bacteroidales bacterium
MBQ7468482.1
Bacteroidales bacterium
MBQ6290772.1
Bacteroidales bacterium
MBR4228459.1
Bacteroidales bacterium
MBO4917820.1
Bacteroidales bacterium
MBO4447281.1
Bacteroidales bacterium
MBQ2521899.1
Bacteroidales bacterium
MBO8475301.1
Candidatus Cryptobacteroides faecigallinarum
MBQ9309839.1
Bacteroidales bacterium
MBS7245374.1
Bacteroidales bacterium
MBQ9477612.1
Bacteroidales bacterium
MBQ3206499.1
Bacteroidales bacterium
CCY34446.1
Alistipes sp. CAG:831
CCX52084.1
Alistipes sp. CAG:514
MBQ0006435.1
Candidatus Minthomonas equi
MBQ6579014.1
Bacteroidales bacterium
MBQ0123513.1
Candidatus Cacconaster equi
HIT49188.1
Candidatus Coprenecus stercoripullorum
MBO8478754.1
Candidatus Cryptobacteroides excrementipullorum
MBO8483068.1
MBQ0127355.1
Candidatus Cryptobacteroides choladohippi
MBO4755505.1
Bacteroidales bacterium
MBR1706143.1
Bacteroidales bacterium
MBQ0094092.1
Candidatus Cryptobacteroides fimicaballi
MBP5335527.1
Bacteroidales bacterium
HIQ84537.1
Candidatus Cryptobacteroides excrementigallinarum
MBR1574648.1
Bacteroidales bacterium
MBQ7194748.1
Bacteroidales bacterium
MBQ1866064.1
Bacteroidales bacterium
MBQ3722866.1
Bacteroidales bacterium
MBR0239828.1
Bacteroidales bacterium
MBP3562992.1
Bacteroidales bacterium
MBQ0025601.1
Candidatus Cacconaster merdequi
MBQ0143873.1
Candidatus Cryptobacteroides faecihippi
MBQ0151074.1
Candidatus Cryptobacteroides onthequi
MBS7289374.1
Bacteroidales bacterium
HIS22191.1
Candidatus Cryptobacteroides intestinipullorum

MBO4671474.1
Bacteroidales bacterium

MBP5637650.1
Bacteroidales bacterium

MBR1698654.1
Bacteroidales bacterium

MBO4428217.1
Bacteroidales bacterium

NLZ51076.1
Rikenellaceae bacterium

MBR0174543.1
Bacteroidales bacterium

MBR1575914.1
Bacteroidales bacterium

MBR4809026.1
Bacteroidales bacterium

MBR5385356.1
Bacteroidales bacterium

MBR5836554.1
Bacteroidales bacterium

MBO7192562.1
Bacteroidales bacterium

MBR6884057.1
Bacteroidales bacterium

MBQ8838403.1
Bacteroidales bacterium

MBQ9192838.1
Bacteroidales bacterium

CDD19099.1
Alistipes sp. CAG:435

MBQ1912402.1
Bacteroidales bacterium

MBQ7020062.1
Bacteroidales bacterium

MBE6242936.1
Bacteroidales bacterium

MBE6239985.1
Bacteroidales bacterium

MBE6217254.1
Bacteroidales bacterium

MBE6232953.1
Bacteroidales bacterium

MBE6234725.1
Bacteroidales bacterium

MBQ2371908.1
Bacteroidales bacterium

MBQ3607653.1
Bacteroidales bacterium

MBQ2918351.1
Bacteroidales bacterium

MBQ3711632.1
Bacteroidales bacterium

MBQ6199291.1
Bacteroidales bacterium

MBQ5983768.1
Bacteroidales bacterium

MBQ9548271.1
Bacteroidales bacterium

MBQ9463330.1
Bacteroidales bacterium

MBQ9652933.1
Bacteroidales bacterium

MBR1510520.1
Bacteroidales bacterium

MBR2064368.1
Bacteroidales bacterium

MBR3441464.1
Bacteroidales bacterium

MBP5486560.1
Bacteroidales bacterium

MBO7643628.1
Bacteroidales bacterium

MBO7396895.1
Bacteroidales bacterium

MBO6170428.1
Bacteroidales bacterium

MBO5876642.1
Bacteroidales bacterium

MBO5815184.1
Bacteroidales bacterium

MBO4756943.1
Bacteroidales bacterium

MBO4743768.1
Bacteroidales bacterium

MBR5567521.1
Bacteroidales bacterium

MBQ7749981.1
Bacteroidales bacterium

MBO4455287.1
Bacteroidales bacterium

MBQ3846393.1
Bacteroidales bacterium

MBR5042881.1
Bacteroidales bacterium

MBR4774532.1
Bacteroidales bacterium

MBO8444577.1
Candidatus Cryptobacteroides merdavium

MBO8456500.1
Candidatus Cryptobacteroides intestinigallinarum

MBQ1937324.1
Bacteroidales bacterium

MBO4476259.1
Bacteroidales bacterium

MBP5720537.1
Bacteroidales bacterium

MBQ6086500.1
Bacteroidales bacterium

MBS7291978.1
Bacteroidales bacterium

MBR6002466.1
Bacteroidales bacterium

MBR1501558.1
Bacteroidales bacterium

MBQ4389172.1
Bacteroidales bacterium

MBO6028894.1
Bacteroidales bacterium

MBR1926668.1
Bacteroidales bacterium

MBQ6761475.1
Bacteroidales bacterium

MBR6863565.1
Bacteroidales bacterium

NLX41504.1
Bacteroidales bacterium

MBQ0086085.1
Candidatus Cacconaster scatequi

MBR3292591.1
Bacteroidales bacterium

MBQ2150391.1
Bacteroidales bacterium

MBR6270099.1
Bacteroidales bacterium

MBR4212950.1
Bacteroidales bacterium

MCD8208606.1
Bacteroidales bacterium

MBQ0122544.1
Candidatus Cacconaster caballi

MBO8447647.1
Candidatus Cryptobacteroides merdigallinarum

MBO4341004.1
Bacteroidales bacterium

MBO4466649.1
Bacteroidales bacterium

MBR6345854.1
Bacteroidales bacterium

MBR5833191.1
Bacteroidales bacterium

MBQ7772123.1
Bacteroidales bacterium

MBR6212859.1
Bacteroidales bacterium

MBO8451958.1
Candidatus Cryptobacteroides intestinavium

MBR4167456.1
Bacteroidales bacterium

MBR6055453.1
Bacteroidales bacterium

MBQ9892347.1
Bacteroidales bacterium

MBO5635357.1
Bacteroidales bacterium

MBQ6179956.1
Bacteroidales bacterium

MBR5699949.1
Bacteroidales bacterium

MBO4761255.1
Bacteroidales bacterium

MBP5539069.1
Bacteroidales bacterium

MBQ6709399.1
Bacteroidales bacterium

MBO5311647.1
Bacteroidales bacterium

MBR5275246.1
Bacteroidales bacterium

MBR2855824.1
Bacteroidales bacterium

MBR4978096.1
Bacteroidales bacterium

MBR1956748.1
Bacteroidales bacterium

MBO5471172.1
Bacteroidales bacterium

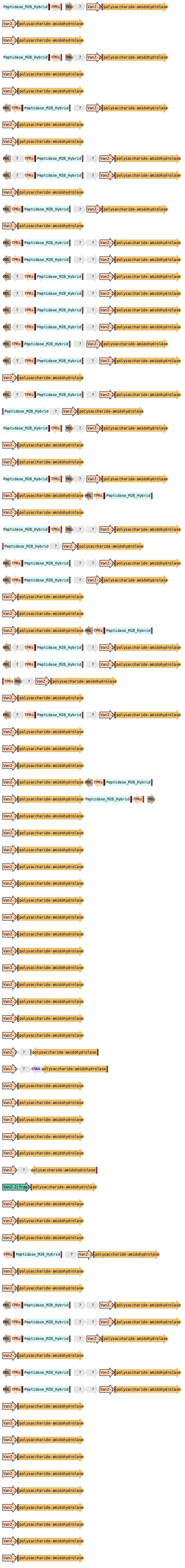

Accession	Organism	Gene neighbourhood
MBQ3251467.1	*Bacteroidales bacterium*	VanZ-2 · polysaccharide-amidohydrolase
MBQ9723335.1	*Bacteroidales bacterium*	VanZ-2 · polysaccharide-amidohydrolase
MBO8453857.1	*Candidatus Cryptobacteroides gallistercoris*	VanZ-2 · polysaccharide-amidohydrolase
MBR4298339.1	*Bacteroidales bacterium*	VanZ-2 · polysaccharide-amidohydrolase
MBQ7811487.1	*Bacteroidales bacterium*	VanZ-2 · polysaccharide-amidohydrolase
MBQ8170200.1	*Bacteroidales bacterium*	VanZ-2 · polysaccharide-amidohydrolase

####;VanY, with other exopolysaccharide synthesis/transport enzymes

Accession	Organism	Gene neighbourhood
MBQ6134791.1	*Bacilli bacterium*	MurE-synthetase · VanY · VanZ-2 · MviN_MATE-domain-flippase
NLJ32209.1	*Clostridiales bacterium*	VanZ-2 · Teichoic-acid-transferase-TagU · CpsB/CapC-capsule-biosynthesis-tyrosine-phosphatase · Wzc/etk/CpsD-like-P-loop-kinase · Wzc/etk/CpsD-like-Nterm-chain-length-determ · VanY

#;standalone VanY-VanZ-2 associations

Accession	Organism	Gene neighbourhood
MBM3937653.1	*Sphingomonadales bacterium*	GCV_H · VanZ-2 · VanY
GDX50254.1	*Bacteroidota bacterium*	VanZ-2 · VanY
NBX64033.1	*Bacteroidota bacterium*	GCV_H · VanZ-2 · VanY
MCE2877327.1	*Bacteroidota bacterium*	GCV_H · VanZ-2 · VanY
MBU9888543.1	*Candidatus Omnitrophota bacterium*	VanY · VanZ-2
EEG50205.1	*Blautia hydrogenotrophica DSM 10507*	VanZ-2 · VanY
OGX12737.1	*Omnitrophica bacterium RIFOXYB12_FULL_50_7*	VanY · VanZ-2
MBO5904364.1	*Clostridia bacterium*	VanY · VanZ-2
MBQ3318640.1	*Candidatus Saccharibacteria bacterium*	VanY · VanZ-2
MBQ4328086.1	*Clostridia bacterium*	VanY · VanZ-2
MBQ6321079.1	*Candidatus Saccharibacteria bacterium*	VanZ-2 · VanY
NLD51838.1	*Clostridiales bacterium*	VanY · VanY · ? · ? · VanZ-2
NCB34656.1	*Clostridia bacterium*	VanY · ? · ? · VanZ-2
HHX20644.1	*Clostridiales bacterium*	VanY · ? · ? · VanZ-2
NLO15697.1	*Clostridiales bacterium*	VanY · VanY · VanY · ? · ? · VanZ-2

#;association with other vancomycin-resistance genes

Accession	Organism	Gene neighbourhood
HBF86712.1	*Clostridiales bacterium*	MurF-UDP-N-acetylmuramoyl-tripeptide--D-alanyl-D-alanine-ligase · VanA/VanG · ? · VanZ-2
WP_163051250.1	*Amedibacterium intestinale*	MurF-UDP-N-acetylmuramoyl-tripeptide--D-alanyl-D-alanine-ligase · VanA/VanG · ? · VanT-N · ? · ? · VanZ-2
PYS41740.1	*Acidobacteriota bacterium*	VanZ-2 · ? · ? · VanA/VanG
PYS10621.1	*Acidobacteriota bacterium*	VanZ-2 · ? · ? · VanA/VanG
MBR1598791.1	*Lachnospiraceae bacterium*	VanZ-2 · VanA/VanG
MBO7202673.1	*Paludibacteraceae bacterium*	VanZ-2 · ? · ? · VanA/VanG
MBD9121835.1	*Catenibacterium mitsuokai*	VanZ-2 · VanX
MBR6801650.1	*Eubacteriaceae bacterium*	VanZ-2 · VanX
MBO7118715.1	*Bacteroidaceae bacterium*	VanZ-2 · VanX
WP_078683404.1	*Desulfobaculum bizertense DSM 18034*	VanZ-2 · VanH-like
OYW26225.1	*Caulobacter sp. 12-67-6*	VanH-like · VanZ-2
NQE63232.1	*Caulobacter sp. RHG1*	VanH-like · VanZ-2
WP_204497812.1	*Aquibacillus albus*	VanH-like · VanZ-2
MBU3924782.1	*Patescibacteria group bacterium*	VanH-like · VanZ-2
MBN2519725.1	*Bacteroidales bacterium*	VanH-like · PSE · VanZ-2
NBJ93037.1	*Parablautia muri*	VanH-like · ? · VanZ-2
OQB36280.1	*Candidatus Latescibacteria bacterium ADurb.Bin168*	VanH-like · ? · VanZ-2
MBP3203969.1	*Lachnospiraceae bacterium*	VanX · ? · VanZ-2

#####;fusions to DUF498, has an additional association with TPRs

Accession	Organism	Gene neighbourhood
MBD3236173.1	*Candidatus Eiseniibacteriota bacterium*	VanZ-2 DUF498 · TPRs
OQX92231.1	*candidate division Zixibacteria bacterium 4484_95*	VanZ-2 DUF498 · TPRs
MBM3287868.1	*Candidatus Eiseniibacteriota bacterium*	VanZ-2 DUF498 · TPRs
MBU1701955.1	*Candidatus Eiseniibacteriota bacterium*	VanZ-2 DUF498 · TPRs
MBP6875687.1	*Candidatus Eiseniibacteriota bacterium*	VanZ-2 DUF498 · TPRs
MBM3324589.1	*Calditrichota bacterium*	VanZ-2 DUF498 · TPRs
MBP6875319.1	*Candidatus Eiseniibacteriota bacterium*	VanZ-2 DUF498 · TPRs
MBU0508159.1	*bacterium*	VanZ-2 DUF498 · TPRs
MBP9026707.1	*Phycisphaerae bacterium*	VanZ-2 DUF498 · TPRs
HHE37782.1	*Candidatus Cloacimonadota bacterium*	TPRs · VanZ-2 DUF498 · ? · ? · ? · VanZ-2 DUF498 · TPRs
NQT34210.1	*bacterium*	VanZ-2 DUF498
RLC45853.1	*Candidatus Coatesbacteria bacterium*	VanZ-2 DUF498 · TPRs
PLX03894.1	*Marinilabiliales bacterium*	VanZ-2 DUF498 · TPRs
MCD4772239.1	*Bacteroidales bacterium*	VanZ-2 DUF498 · TPRs
MCD6594945.1	*bacterium*	VanZ-2 DUF498 · TPRs
MBL7190988.1	*bacterium*	VanZ-2 DUF498
NQT35455.1	*bacterium*	VanZ-2 DUF498
MBN1949534.1	*Candidatus Cloacimonadota bacterium*	VanZ-2 DUF498 · TPRs
MBL7032779.1	*Candidatus Delongbacteria bacterium*	VanZ-2 DUF498 · TPRs
MCD4651358.1	*Candidatus Cloacimonadota bacterium*	VanZ-2 DUF498 · TPRs
RLD90232.1	*Bacteroidota bacterium*	VanZ-2 DUF498 · TPRs
MBN2830181.1	*Candidatus Cloacimonadota bacterium*	VanZ-2 DUF498
MBE9467825.1	*Bacteroidota bacterium*	VanZ-2 DUF498 · TPRs
PLX11182.1	*Marinilabiliales bacterium*	VanZ-2 DUF498 · TPRs
MBN1950824.1	*Bacteroidales bacterium*	VanZ-2 DUF498
OFY38051.1	*Bacteroidetes bacterium GWF2_38_335*	VanZ-2 DUF498
MBP7463947.1	*Bacteroidales bacterium*	VanZ-2 DUF498 · TPRs
MBP8041875.1	*Bacteroidales bacterium*	VanZ-2 DUF498 · TPRs
HHE37778.1	*Candidatus Cloacimonadota bacterium*	TPRs · DUF498 VanZ-2 · ? · ? · ? · VanZ-2 DUF498 · TPRs
MBP7076979.1	*Bacteroidales bacterium*	VanZ-2 DUF498 · TPRs
MCD6579918.1	*bacterium*	VanZ-2 DUF498 · TPRs
PLX08147.1	*Marinilabiliales bacterium*	VanZ-2 DUF498
MBN2745927.1	*Bacteroidales bacterium*	VanZ-2 DUF498
MBL7103748.1	*Bacteroidales bacterium*	VanZ-2 DUF498
OQX70961.1	*Candidatus Cloacimonas sp. 4484_275*	VanZ-2 DUF498
NQU35349.1	*Bacteroidota bacterium*	VanZ-2 DUF498 · TPRs
MBU0616608.1	*Planctomycetota bacterium*	VanZ-2 DUF498 · TPRs
ROL61853.1	*Bacteroidetes/Chlorobi group bacterium ChocPot_Mid*	VanZ-2 DUF498

#####;fusion to PAP2; #;direct fusion

Accession	Organism	Gene neighbourhood
MCA9548502.1	*Myxococcales bacterium*	PAP2 VanZ-2
NLF14470.1	*Anaerolineaceae bacterium*	PAP2 VanZ-2
NJM40035.1	*Anaerolineae bacterium*	PAP2 VanZ-2
MBN1583366.1	*Anaerolineae bacterium*	PAP2 VanZ-2
MBN1937173.1	*Anaerolineae bacterium*	PAP2 VanZ-2
MCB0209914.1	*Anaerolineae bacterium*	PAP2 VanZ-2
MBE7556374.1	*Anaerolineales bacterium*	PAP2 VanZ-2
MCB0087348.1	*Caldilineaceae bacterium*	PAP2 VanZ-2
MCB0159621.1	*Caldilineaceae bacterium*	PAP2 VanZ-2
MCC6457057.1	*Caldilineaceae bacterium*	PAP2 VanZ-2

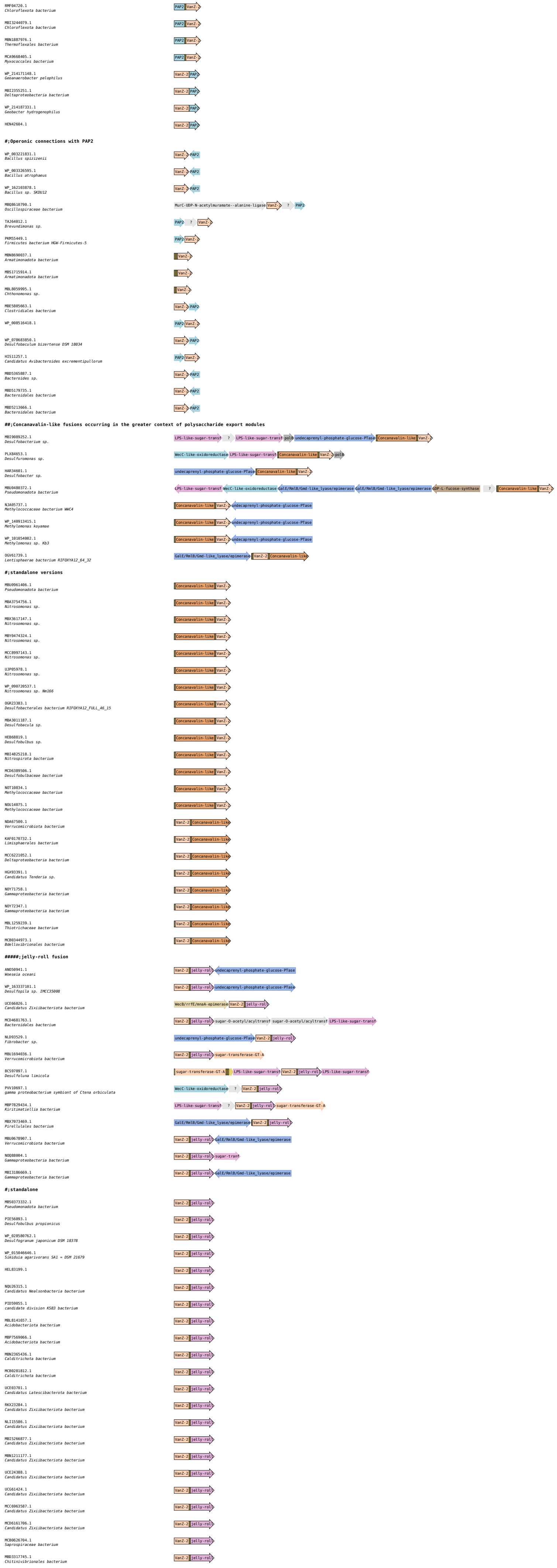
RMF04720.1 Chloroflexota bacterium
PAP2 VanZ-2
MBI3244079.1 Chloroflexota bacterium
PAP2 VanZ-2
MBN1887976.1 Thermoflexales bacterium
PAP2 VanZ-2
MCA9668405.1 Myxococcales bacterium
PAP2 VanZ-2
WP_214171148.1 Geoanaerobacter pelophilus
VanZ-2 PAP2
MBI2355251.1 Deltaproteobacteria bacterium
VanZ-2 PAP2
WP_214187331.1 Geobacter hydrogenophilus
VanZ-2 PAP2
HEN42604.1
VanZ-2 PAP2
#;Operonic connections with PAP2
WP_003221831.1 Bacillus spizizenii
VanZ-2 PAP2
WP_003326595.1 Bacillus atrophaeus
VanZ-2 PAP2
WP_162103878.1 Bacillus sp. SKDU12
VanZ-2 PAP2
MBQ8610790.1 Oscillospiraceae bacterium
MurC-UDP-N-acetylmuramate--alanine-ligase VanZ-2 ? PAP2
TAJ64012.1 Brevundimonas sp.
PAP2 ? VanZ-2
PKM55449.1 Firmicutes bacterium HGW-Firmicutes-5
PAP2 VanZ-2
MBN8690037.1 Armatimonadota bacterium
VanZ-2
MBS1715914.1 Armatimonadota bacterium
VanZ-2
MBL8059995.1 Chthonomonas sp.
VanZ-2
MBE5805663.1 Clostridiales bacterium
VanZ-2 PAP2
WP_008516418.1
PAP2 VanZ-2
WP_078683850.1 Desulfobaculum bizertense DSM 18034
VanZ-2 PAP2
HIS11257.1 Candidatus Avibacteroides excrementipullorum
PAP2 VanZ-2
MBD5365887.1 Bacteroides sp.
VanZ-2 PAP2
MBD5179735.1 Bacteroidales bacterium
VanZ-2 PAP2
MBD5213666.1 Bacteroidales bacterium
VanZ-2 PAP2
##;Concanavalin-like fusions occurring in the greater context of polysaccharide export modules
MBI9089252.1 Desulfobacterium sp.
LPS-like-sugar-transf ? LPS-like-sugar-transf polB undecaprenyl-phosphate-glucose-PTase Concanavalin-like VanZ-2
PLX84653.1 Desulfuromonas sp.
WecC-like-oxidoreductase LPS-like-sugar-transf Concanavalin-like VanZ-2 polB
HAR34601.1 Desulfobacter sp.
undecaprenyl-phosphate-glucose-PTase Concanavalin-like VanZ-2
MBU0480372.1 Pseudomonadota bacterium
LPS-like-sugar-transf WecC-like-oxidoreductase GalE/RmlB/Gmd-like_lyase/epimerase GalE/RmlB/Gmd-like_lyase/epimerase GDP-L-fucose-synthase ? Concanavalin-like VanZ-2
NJA05737.1 Methylococcaceae bacterium WWC4
Concanavalin-like VanZ-2 undecaprenyl-phosphate-glucose-PTase
WP_140913415.1 Methylomonas koyamae
Concanavalin-like VanZ-2 undecaprenyl-phosphate-glucose-PTase
WP_101054002.1 Methylomonas sp. Kb3
Concanavalin-like VanZ-2 undecaprenyl-phosphate-glucose-PTase
OGV61739.1 Lentisphaerae bacterium RIFOXYA12_64_32
GalE/RmlB/Gmd-like_lyase/epimerase VanZ-2 Concanavalin-like
#;standalone versions
MBU0961406.1 Pseudomonadota bacterium
Concanavalin-like VanZ-2
MBA3754756.1 Nitrosomonas sp.
Concanavalin-like VanZ-2
MBX3617147.1 Nitrosomonas sp.
Concanavalin-like VanZ-2
MBY0474324.1 Nitrosomonas sp.
Concanavalin-like VanZ-2
MCC8997143.1 Nitrosomonas sp.
Concanavalin-like VanZ-2
UJP05978.1 Nitrosomonas sp.
Concanavalin-like VanZ-2
WP_090720537.1 Nitrosomonas sp. Nm166
Concanavalin-like VanZ-2
OGR23383.1 Desulfobacterales bacterium RIFOXYA12_FULL_46_15
Concanavalin-like VanZ-2
MBA3011187.1 Desulfobacula sp.
Concanavalin-like VanZ-2
HEB68819.1 Desulfobulbus sp.
Concanavalin-like VanZ-2
MBI4825218.1 Nitrospirota bacterium
Concanavalin-like VanZ-2
MCD6389506.1 Desulfobulbaceae bacterium
Concanavalin-like VanZ-2
NOT10834.1 Methylococcaceae bacterium
Concanavalin-like VanZ-2
NOU14075.1 Methylococcaceae bacterium
Concanavalin-like VanZ-2
NDA67500.1 Verrucomicrobiota bacterium
VanZ-2 Concanavalin-like
KAF0170732.1 Limisphaerales bacterium
VanZ-2 Concanavalin-like
MCC6221052.1 Deltaproteobacteria bacterium
VanZ-2 Concanavalin-like
HGX93391.1 Candidatus Tenderia sp.
VanZ-2 Concanavalin-like
NOY71758.1 Gammaproteobacteria bacterium
VanZ-2 Concanavalin-like
NOY72347.1 Gammaproteobacteria bacterium
VanZ-2 Concanavalin-like
MBL1259239.1 Thiotrichaceae bacterium
VanZ-2 Concanavalin-like
MCB0344973.1 Bdellovibrionales bacterium
VanZ-2 Concanavalin-like
#####;jelly-roll fusion
ANO50941.1 Woeseia oceani
VanZ-2 jelly-roll undecaprenyl-phosphate-glucose-PTase
WP_163337101.1 Desulfopila sp. IMCC35008
VanZ-2 jelly-roll undecaprenyl-phosphate-glucose-PTase
UCE66026.1 Candidatus Zixiibacteriota bacterium
WecB/rrfE/mnaA-epimerase VanZ-2 jelly-roll
MCD4681763.1 Bacteroidales bacterium
VanZ-2 jelly-roll sugar-O-acetyl/acyltransf sugar-O-acetyl/acyltransf LPS-like-sugar-transf
NLD93529.1 Fibrobacter sp.
undecaprenyl-phosphate-glucose-PTase VanZ-2 jelly-roll
MBU1694036.1 Verrucomicrobiota bacterium
VanZ-2 jelly-roll sugar-transferase-GT-A
BCS97097.1 Desulfoluna limicola
sugar-transferase-GT-A LPS-like-sugar-transf VanZ-2 jelly-roll LPS-like-sugar-transf
PVV10697.1 gamma proteobacterium symbiont of Ctena orbiculata
WecC-like-oxidoreductase ? VanZ-2 jelly-roll
MBP7829434.1 Kiritimatiellia bacterium
LPS-like-sugar-transf ? VanZ-2 jelly-roll sugar-transferase-GT-A
MBX7073469.1 Pirellulales bacterium
GalE/RmlB/Gmd-like_lyase/epimerase VanZ-2 jelly-roll
MBU0678907.1 Verrucomicrobiota bacterium
VanZ-2 jelly-roll GalE/RmlB/Gmd-like_lyase/epimerase
NOQ88004.1 Gammaproteobacteria bacterium
VanZ-2 jelly-roll sugar-tranf
MBI3186669.1 Gammaproteobacteria bacterium
VanZ-2 jelly-roll GalE/RmlB/Gmd-like_lyase/epimerase
#;standalone
MBS0373332.1 Pseudomonadota bacterium
VanZ-2 jelly-roll
PIE56093.1 Desulfobulbus propionicus
VanZ-2 jelly-roll
WP_028580762.1 Desulfogranum japonicum DSM 18378
VanZ-2 jelly-roll
WP_015046646.1 Simiduia agarivorans SA1 = DSM 21679
VanZ-2 jelly-roll
HEL83199.1
VanZ-2 jelly-roll
NQU26315.1 Candidatus Nealsonbacteria bacterium
VanZ-2 jelly-roll
PID59055.1 candidate division KSB3 bacterium
VanZ-2 jelly-roll
MBL8141657.1 Acidobacteriota bacterium
VanZ-2 jelly-roll
MBP7569066.1 Acidobacteriota bacterium
VanZ-2 jelly-roll
MBN2365436.1 Calditrichota bacterium
VanZ-2 jelly-roll
MCB0281812.1 Calditrichota bacterium
VanZ-2 jelly-roll
UCE03701.1 Candidatus Latescibacterota bacterium
VanZ-2 jelly-roll
RKX23284.1 Candidatus Zixiibacteriota bacterium
VanZ-2 jelly-roll
NLI15586.1 Candidatus Zixiibacteriota bacterium
VanZ-2 jelly-roll
MBI5266877.1 Candidatus Zixiibacteriota bacterium
VanZ-2 jelly-roll
MBN1211177.1 Candidatus Zixiibacteriota bacterium
VanZ-2 jelly-roll
UCE24388.1 Candidatus Zixiibacteriota bacterium
VanZ-2 jelly-roll
UCG61424.1 Candidatus Zixiibacteriota bacterium
VanZ-2 jelly-roll
MCC6963587.1 Candidatus Zixiibacteriota bacterium
VanZ-2 jelly-roll
MCD6161706.1 Candidatus Zixiibacteriota bacterium
VanZ-2 jelly-roll
MCB0626704.1 Saprospiraceae bacterium
VanZ-2 jelly-roll
MBD3317745.1 Chitinivibrionales bacterium
VanZ-2 jelly-roll

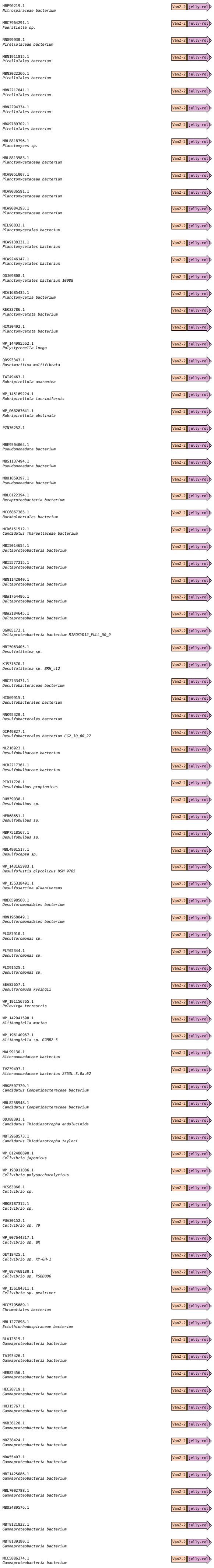
HBP90219.1
Nitrospiraceae bacterium
VanZ-2
jelly-rol
MBC7964291.1
Fuerstiella sp.
VanZ-2
jelly-rol
NND99930.1
Pirellulaceae bacterium
VanZ-2
jelly-rol
MBN1911815.1
Pirellulales bacterium
VanZ-2
jelly-rol
MBN2022266.1
Pirellulales bacterium
VanZ-2
jelly-rol
MBN2217841.1
Pirellulales bacterium
VanZ-2
jelly-rol
MBN2294334.1
Pirellulales bacterium
VanZ-2
jelly-rol
MBX9789702.1
Pirellulales bacterium
VanZ-2
jelly-rol
MBL8818796.1
Planctomyces sp.
VanZ-2
jelly-rol
MBL8813583.1
Planctomycetaceae bacterium
VanZ-2
jelly-rol
MCA9051007.1
Planctomycetaceae bacterium
VanZ-2
jelly-rol
MCA9036591.1
Planctomycetaceae bacterium
VanZ-2
jelly-rol
MCA9084293.1
Planctomycetaceae bacterium
VanZ-2
jelly-rol
NIL96832.1
Planctomycetales bacterium
VanZ-2
jelly-rol
MCA9138331.1
Planctomycetales bacterium
VanZ-2
jelly-rol
MCA9246147.1
Planctomycetales bacterium
VanZ-2
jelly-rol
QGJ69808.1
Planctomycetales bacterium 10988
VanZ-2
jelly-rol
MCA1685435.1
Planctomycetia bacterium
VanZ-2
jelly-rol
REK23786.1
Planctomycetota bacterium
VanZ-2
jelly-rol
HIM30492.1
Planctomycetota bacterium
VanZ-2
jelly-rol
WP_144995562.1
Polystyrenella longa
VanZ-2
jelly-rol
QDS93343.1
Roseimaritima multifibrata
VanZ-2
jelly-rol
TWT49463.1
Rubripirellula amarantea
VanZ-2
jelly-rol
WP_145169224.1
Rubripirellula lacrimiformis
VanZ-2
jelly-rol
WP_068267641.1
Rubripirellula obstinata
VanZ-2
jelly-rol
PZN76252.1
VanZ-2
jelly-rol
MBE9504064.1
Pseudomonadota bacterium
VanZ-2
jelly-rol
MBS1137494.1
Pseudomonadota bacterium
VanZ-2
jelly-rol
MBU1059297.1
Pseudomonadota bacterium
VanZ-2
jelly-rol
MBL0122394.1
Betaproteobacteria bacterium
VanZ-2
jelly-rol
MCC6867385.1
Burkholderiales bacterium
VanZ-2
jelly-rol
MCD6151512.1
Candidatus Tharpellaceae bacterium
VanZ-2
jelly-rol
MBI5014654.1
Deltaproteobacteria bacterium
VanZ-2
jelly-rol
MBI5577215.1
Deltaproteobacteria bacterium
VanZ-2
jelly-rol
MBN1142040.1
Deltaproteobacteria bacterium
VanZ-2
jelly-rol
MBW1764486.1
Deltaproteobacteria bacterium
VanZ-2
jelly-rol
MBW2184645.1
Deltaproteobacteria bacterium
VanZ-2
jelly-rol
OGR05172.1
Deltaproteobacteria bacterium RIFOXYD12_FULL_50_9
VanZ-2
jelly-rol
MBI5063405.1
Desulfatitalea sp.
VanZ-2
jelly-rol
KJS31570.1
Desulfatitalea sp. BRH_c12
VanZ-2
jelly-rol
MBC2733471.1
Desulfobacteraceae bacterium
VanZ-2
jelly-rol
HID69915.1
Desulfobacterales bacterium
VanZ-2
jelly-rol
NNK95320.1
Desulfobacterales bacterium
VanZ-2
jelly-rol
OIP49827.1
Desulfobacterales bacterium CG2_30_60_27
VanZ-2
jelly-rol
NLZ16923.1
Desulfobulbaceae bacterium
VanZ-2
jelly-rol
MCB2217361.1
Desulfobulbaceae bacterium
VanZ-2
jelly-rol
PID71728.1
Desulfobulbus propionicus
VanZ-2
jelly-rol
RUM39038.1
Desulfobulbus sp.
VanZ-2
jelly-rol
HEB68651.1
Desulfobulbus sp.
VanZ-2
jelly-rol
MBP7518567.1
Desulfobulbus sp.
VanZ-2
jelly-rol
MBL4901517.1
Desulfocapsa sp.
VanZ-2
jelly-rol
WP_143165983.1
Desulfofustis glycolicus DSM 9705
VanZ-2
jelly-rol
WP_155318491.1
Desulfosarcina alkanivorans
VanZ-2
jelly-rol
MBE0598560.1
Desulfuromonadales bacterium
VanZ-2
jelly-rol
MBN1958849.1
Desulfuromonadales bacterium
VanZ-2
jelly-rol
PLX87910.1
Desulfuromonas sp.
VanZ-2
jelly-rol
PLY02344.1
Desulfuromonas sp.
VanZ-2
jelly-rol
PLX91525.1
Desulfuromonas sp.
VanZ-2
jelly-rol
SEA82657.1
Desulfuromusa kysingii
VanZ-2
jelly-rol
WP_191156765.1
Pelovirga terrestris
VanZ-2
jelly-rol
WP_142941598.1
Aliikangiella marina
VanZ-2
jelly-rol
WP_196140967.1
Aliikangiella sp. G2MR2-5
VanZ-2
jelly-rol
MAL99130.1
Alteromonadaceae bacterium
VanZ-2
jelly-rol
TVZ39497.1
Alteromonadaceae bacterium 2753L.S.0a.02
VanZ-2
jelly-rol
MBK8507320.1
Candidatus Competibacteraceae bacterium
VanZ-2
jelly-rol
MBL8258948.1
Candidatus Competibacteraceae bacterium
VanZ-2
jelly-rol
ODJ88391.1
Candidatus Thiodiazotropha endolucinida
VanZ-2
jelly-rol
MBT2968573.1
Candidatus Thiodiazotropha taylori
VanZ-2
jelly-rol
WP_012486890.1
Cellvibrio japonicus
VanZ-2
jelly-rol
WP_193911086.1
Cellvibrio polysaccharolyticus
VanZ-2
jelly-rol
HCS63066.1
Cellvibrio sp.
VanZ-2
jelly-rol
MBK8187312.1
Cellvibrio sp.
VanZ-2
jelly-rol
PUA30152.1
Cellvibrio sp. 79
VanZ-2
jelly-rol
WP_007644317.1
Cellvibrio sp. BR
VanZ-2
jelly-rol
QEY18425.1
Cellvibrio sp. KY-GH-1
VanZ-2
jelly-rol
WP_087468180.1
Cellvibrio sp. PSBB006
VanZ-2
jelly-rol
WP_156184311.1
Cellvibrio sp. pealriver
VanZ-2
jelly-rol
MCC5795689.1
Chromatiales bacterium
VanZ-2
jelly-rol
MBL1277898.1
Ectothiorhodospiraceae bacterium
VanZ-2
jelly-rol
RLA12519.1
Gammaproteobacteria bacterium
VanZ-2
jelly-rol
TAJ93426.1
Gammaproteobacteria bacterium
VanZ-2
jelly-rol
HEB82456.1
Gammaproteobacteria bacterium
VanZ-2
jelly-rol
HEC28719.1
Gammaproteobacteria bacterium
VanZ-2
jelly-rol
HHJ15767.1
Gammaproteobacteria bacterium
VanZ-2
jelly-rol
NKB36128.1
Gammaproteobacteria bacterium
VanZ-2
jelly-rol
NOZ38424.1
Gammaproteobacteria bacterium
VanZ-2
jelly-rol
NRA55407.1
Gammaproteobacteria bacterium
VanZ-2
jelly-rol
MBI1425086.1
Gammaproteobacteria bacterium
VanZ-2
jelly-rol
MBL7002788.1
Gammaproteobacteria bacterium
VanZ-2
jelly-rol
MBO2489576.1
VanZ-2
jelly-rol
MBT8121822.1
Gammaproteobacteria bacterium
VanZ-2
jelly-rol
MBT8139180.1
Gammaproteobacteria bacterium
VanZ-2
jelly-rol
MCC5886274.1
Gammaproteobacteria bacterium
VanZ-2
jelly-rol

KPK59321.1	*Gammaproteobacteria bacterium SG8_31*	VanZ-2 jelly-roll
KPK52477.1	*Gammaproteobacteria bacterium SG8_31*	VanZ-2 jelly-roll
MBL4621177.1	*Immundisolibacteraceae bacterium*	VanZ-2 jelly-roll
WP_225314873.1		VanZ-2 jelly-roll
WP_012136474.1	*Marinobacter lipolyticus SM19*	VanZ-2 jelly-roll
RZA07721.1	*Moraxellaceae bacterium*	VanZ-2 jelly-roll
WP_101758188.1	*Oceanicoccus sp. KOV_DT_Chl*	VanZ-2 jelly-roll
NQZ30450.1	*Oceanospirillaceae bacterium*	VanZ-2 jelly-roll
NRA22502.1	*Oceanospirillaceae bacterium*	VanZ-2 jelly-roll
TNC79823.1	*Oleiphilus sp.*	VanZ-2 jelly-roll
KZY61725.1	*Oleiphilus sp. HI0065*	VanZ-2 jelly-roll
MBL4783034.1	*Porticoccaceae bacterium*	VanZ-2 jelly-roll
WP_028867737.1	*Psychromonas arctica DSM 14288*	VanZ-2 jelly-roll
WP_181817669.1	*Psychromonas sp. B3M02*	VanZ-2 jelly-roll
WP_101109521.1	*Psychromonas sp. psych-6C06*	VanZ-2 jelly-roll
WP_008046310.1	*Reinekea blandensis MED297*	VanZ-2 jelly-roll
WP_011468548.1	*Saccharophagus degradans 2-40*	VanZ-2 jelly-roll
MBM0108675.1	*Steroidobacter gossypii*	VanZ-2 jelly-roll
TVP85568.1	*Thioalkalivibrio sp.*	VanZ-2 jelly-roll
RDH81395.1	*endosymbiont of Galathealinum brachiosum*	VanZ-2 jelly-roll
EAW32270.1	*marine gamma proteobacterium HTCC2143*	VanZ-2 jelly-roll
WP_068514599.1	*Oleiphilus sp. HI0132*	VanZ-2 jelly-roll
NLW69441.1	*Bacteriovoracaceae bacterium*	VanZ-2 jelly-roll
MBF8258146.1	*Actinomycetota bacterium*	VanZ-2 jelly-roll
NPA94325.1	*Thermodesulfobacteriota bacterium*	VanZ-2 jelly-roll
MBK7141434.1	*bacterium*	VanZ-2 jelly-roll
MBK7090629.1	*bacterium*	VanZ-2 jelly-roll

####;fusion to zinc ribbon domains

TVQ52599.1	*Phycisphaerales bacterium*	Wzc/etk/CpsD-like-P-loop-kinase VanZ-2 ZnR ZnR
MBU3729300.1	*Phycisphaerales bacterium*	Wzc/etk/CpsD-like-P-loop-kinase VanZ-2 ZnR ZnR
MCA9291876.1	*Phycisphaerales bacterium*	Wzc/etk/CpsD-like-P-loop-kinase VanZ-2 ZnR ZnR
MCA9285549.1	*Phycisphaerales bacterium*	Wzc/etk/CpsD-like-P-loop-kinase VanZ-2 ZnR ZnR
PHX78948.1	*Planctomycetaceae bacterium*	VanZ-2 ZnR ZnR
TDJ57912.1	*Planctomycetota bacterium*	Wzc/etk/CpsD-like-P-loop-kinase VanZ-2 ZnR ZnR
NDG64290.1	*Planctomycetota bacterium*	Wzc/etk/CpsD-like-P-loop-kinase VanZ-2 ZnR ZnR

####;fusion to IG-like repeats, largely standalone on the genome

MBQ2736422.1	*Clostridia bacterium*	IG-like-repeats VanZ-2
MBQ7323482.1	*Clostridia bacterium*	IG-like-repeats VanZ-2
MBQ7375249.1	*Clostridia bacterium*	IG-like-repeats VanZ-2
MBQ7408419.1	*Clostridia bacterium*	IG-like-repeats VanZ-2
MBQ9715029.1	*Clostridia bacterium*	IG-like-repeats VanZ-2
MBR2371784.1	*Clostridia bacterium*	IG-like-repeats VanZ-2
MBR2340965.1	*Clostridia bacterium*	IG-like-repeats VanZ-2
MBR3875155.1	*Clostridia bacterium*	IG-like-repeats VanZ-2
MBR3864708.1	*Clostridia bacterium*	IG-like-repeats VanZ-2
MBR6788834.1	*Clostridia bacterium*	IG-like-repeats VanZ-2
MBR4419542.1	*Clostridia bacterium*	IG-like-repeats VanZ-2
MBO5457378.1	*Clostridia bacterium*	IG-like-repeats VanZ-2
MBE5765242.1	*Clostridiales bacterium*	IG-like-repeats VanZ-2
MBR2322863.1	*Clostridia bacterium*	IG-like-repeats VanZ-2
MBR6736800.1	*Clostridia bacterium*	IG-like-repeats VanZ-2
MBR3803934.1	*Clostridia bacterium*	IG-like-repeats VanZ-2
MBR5439406.1	*Clostridia bacterium*	IG-like-repeats VanZ-2
MBO5926628.1	*Clostridia bacterium*	IG-like-repeats VanZ-2
MBE5744100.1	*Clostridiales bacterium*	IG-like-repeats VanZ-2
MBR2933133.1	*Clostridia bacterium*	IG-like-repeats VanZ-2
MBR7135747.1	*Clostridia bacterium*	IG-like-repeats VanZ-2
MBS5859158.1	*Clostridium sp.*	IG-like-repeats VanZ-2
CCY03687.1	*Faecalibacterium sp. CAG:1138*	IG-like-repeats VanZ-2
CDB25131.1	*Firmicutes bacterium CAG:552*	IG-like-repeats VanZ-2
NCA95767.1	*Methanomicrobia archaeon*	IG-like-repeats VanZ-2
NLI94818.1	*Erysipelotrichaceae bacterium*	IG-like-repeats VanZ-2
MBM3909579.1	*Bacillota bacterium*	GalE/RmlB/Gmd-like_lyase/epimerase IG-like-repeats VanZ-2
MBR0294984.1	*Bacilli bacterium*	IG-like-repeats VanZ-2 VanZ-2
MBQ7994662.1	*Bacilli bacterium*	IG-like-repeats VanZ-2
MBO4666985.1	*Bacilli bacterium*	IG-like-repeats VanZ-2
HIT17423.1	*Candidatus Caccosoma faecigallinarum*	IG-like-repeats VanZ-2
MBO5711292.1	*Acholeplasmatales bacterium*	IG-like-repeats VanZ-2
RXZ57892.1	*Candidatus Borkfalkia ceftriaxoniphila*	IG-like-repeats VanZ-2
MBR7186830.1	*Clostridia bacterium*	IG-like-repeats VanZ-2
MBQ2894663.1	*Clostridia bacterium*	IG-like-repeats VanZ-2
MBQ8434369.1	*Clostridia bacterium*	IG-like-repeats VanZ-2
MBQ3001153.1	*Bacilli bacterium*	IG-like-repeats VanZ-2 IG-like-repeats VanZ-2
MBQ3001154.1	*Bacilli bacterium*	IG-like-repeats VanZ-2 IG-like-repeats VanZ-2
MBR1891088.1	*Clostridia bacterium*	IG-like-repeats VanZ-2
MBO4573634.1	*Clostridia bacterium*	IG-like-repeats VanZ-2
MBQ8141613.1	*Bacilli bacterium*	IG-like-repeats VanZ-2
NCA67321.1	*Clostridia bacterium*	IG-like-repeats VanZ-2
MBD5092519.1	*Clostridiales bacterium*	IG-like-repeats VanZ-2
MBP5445943.1	*Acholeplasmatales bacterium*	IG-like-repeats VanZ-2
MBQ8206927.1	*Bacilli bacterium*	IG-like-repeats VanZ-2
MBO5773107.1	*Clostridia bacterium*	IG-like-repeats VanZ-2
MBE7088347.1	*Clostridiales bacterium*	IG-like-repeats VanZ-2
MBQ4357430.1	*Acholeplasmatales bacterium*	IG-like-repeats VanZ-2
MBR3618022.1	*Acholeplasmatales bacterium*	IG-like-repeats VanZ-2
MBQ4124234.1	*bacterium*	IG-like-repeats VanZ-2
MBO8427801.1	*Candidatus Onthovivens merdipullorum*	IG-like-repeats VanZ-2
HBS10353.1	*Bacillota bacterium*	IG-like-repeats VanZ-2
HBS02399.1	*Bacillota bacterium*	IG-like-repeats VanZ-2
MBR1581561.1	*Bacilli bacterium*	IG-like-repeats VanZ-2
MBR2891860.1	*Bacilli bacterium*	IG-like-repeats VanZ-2
MBO7078936.1	*Bacilli bacterium*	IG-like-repeats VanZ-2
MBR2967651.1	*Clostridia bacterium*	Wzc/etk/CpsD-like-P-loop-kinase ? IG-like-repeats VanZ-2

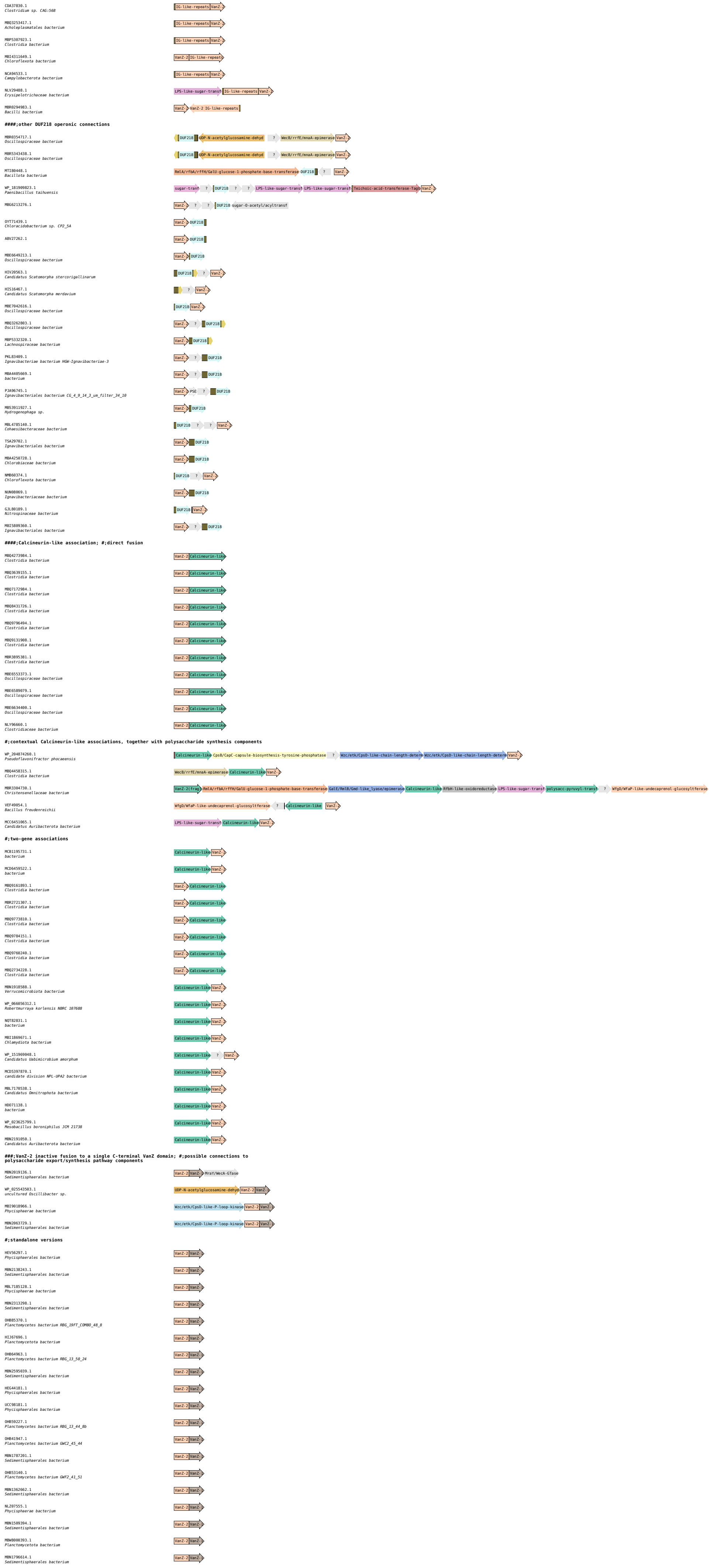

CDA37830.1
Clostridium sp. CAG:568
IG-like-repeats VanZ-2
MBQ3253417.1
Acholeplasmatales bacterium
IG-like-repeats VanZ-2
MBP5307923.1
Clostridia bacterium
IG-like-repeats VanZ-2
MBI4311649.1
Chloroflexota bacterium
VanZ-2 IG-like-repeats
NCA94533.1
Campylobacterota bacterium
IG-like-repeats VanZ-2
NLV29488.1
Erysipelotrichaceae bacterium
LPS-like-sugar-transf IG-like-repeats VanZ-2
MBR0294983.1
Bacilli bacterium
VanZ-2 VanZ-2 IG-like-repeats
####;other DUF218 operonic connections
MBR0354717.1
Oscillospiraceae bacterium
DUF218 UDP-N-acetylglucosamine-dehyd ? WecB/rrfE/mnaA-epimerase VanZ-2
MBR5343438.1
Oscillospiraceae bacterium
DUF218 UDP-N-acetylglucosamine-dehyd ? WecB/rrfE/mnaA-epimerase VanZ-2
MTI80448.1
Bacillota bacterium
RmlA/rfbA/rffH/GalU-glucose-1-phosphate-base-transferase DUF218 ? VanZ-2
WP_181909823.1
Paenibacillus taihuensis
sugar-tranf ? DUF218 ? ? LPS-like-sugar-transf LPS-like-sugar-transf Teichoic-acid-transferase-TagU VanZ-2
MBG6213276.1
VanZ-2 ? ? DUF218 sugar-O-acetyl/acyltransf
OYT71439.1
Chloracidobacterium sp. CP2_5A
VanZ-2 DUF218
ABV27262.1
VanZ-2 DUF218
MBE6649213.1
Oscillospiraceae bacterium
VanZ-2 DUF218
HIV20563.1
Candidatus Scatomorpha stercorigallinarum
DUF218 ? VanZ-2
HIS16467.1
Candidatus Scatomorpha merdavium
? VanZ-2
MBE7042616.1
Oscillospiraceae bacterium
DUF218 VanZ-2
MBQ3262803.1
Oscillospiraceae bacterium
VanZ-2 ? DUF218
MBP5332320.1
Lachnospiraceae bacterium
VanZ-2 DUF218
PKL83409.1
Ignavibacteriae bacterium HGW-Ignavibacteriae-3
VanZ-2 ? DUF218
MBA4405669.1
bacterium
VanZ-2 ? DUF218
PJA96745.1
Ignavibacteriales bacterium CG_4_9_14_3_um_filter_34_10
VanZ-2 PSE ? DUF218
MBS3911927.1
Hydrogenophaga sp.
VanZ-2 DUF218
MBL4785140.1
Cohaesibacteraceae bacterium
DUF218 ? ? VanZ-2
TSA29702.1
Ignavibacteriales bacterium
VanZ-2 DUF218
MBA4250728.1
Chlorobiaceae bacterium
VanZ-2 DUF218
NMB60374.1
Chloroflexota bacterium
DUF218 ? VanZ-2
NUN08069.1
Ignavibacteriaceae bacterium
VanZ-2 DUF218
GJL80189.1
Nitrospinaceae bacterium
DUF218 VanZ-2
MBI5809360.1
Ignavibacteriales bacterium
VanZ-2 ? DUF218
####;Calcineurin-like association; #;direct fusion
MBQ4273984.1
Clostridia bacterium
VanZ-2 Calcineurin-like
MBQ3639155.1
Clostridia bacterium
VanZ-2 Calcineurin-like
MBQ7172984.1
Clostridia bacterium
VanZ-2 Calcineurin-like
MBQ8431726.1
Clostridia bacterium
VanZ-2 Calcineurin-like
MBQ9796494.1
Clostridia bacterium
VanZ-2 Calcineurin-like
MBQ9131908.1
Clostridia bacterium
VanZ-2 Calcineurin-like
MBR3895381.1
Clostridia bacterium
VanZ-2 Calcineurin-like
MBE6553373.1
Oscillospiraceae bacterium
VanZ-2 Calcineurin-like
MBE6589079.1
Oscillospiraceae bacterium
VanZ-2 Calcineurin-like
MBE6634400.1
Oscillospiraceae bacterium
VanZ-2 Calcineurin-like
NLY96660.1
Clostridiaceae bacterium
VanZ-2 Calcineurin-like
#;contextual Calcineurin-like associations, together with polysaccharide synthesis components
WP_204874260.1
Pseudoflavonifractor phocaeensis
Calcineurin-like CpsB/CapC-capsule-biosynthesis-tyrosine-phosphatase ? Wzc/etk/CpsD-like-chain-length-determ Wzc/etk/CpsD-like-chain-length-determ VanZ-2
MBQ4458315.1
Clostridia bacterium
WecB/rrfE/mnaA-epimerase Calcineurin-like VanZ-2
MBR3304730.1
Christensenellaceae bacterium
VanZ-2(frag) RmlA/rfbA/rffH/GalU-glucose-1-phosphate-base-transferase GalE/RmlB/Gmd-like_lyase/epimerase Calcineurin-like RfbH-like-oxidoreductase LPS-like-sugar-transf polysacc-pyruvyl-transf ? WfgD/WfaP-like-undecaprenol-glucosyltferase
VEF49054.1
Bacillus freudenreichii
WfgD/WfaP-like-undecaprenol-glucosyltferase ? Calcineurin-like VanZ-2
MCC6451065.1
Candidatus Auribacterota bacterium
LPS-like-sugar-transf Calcineurin-like VanZ-2
#;two-gene associations
MCB1195731.1
bacterium
Calcineurin-like VanZ-2
MCD6459522.1
bacterium
Calcineurin-like VanZ-2
MBQ9161893.1
Clostridia bacterium
VanZ-2 Calcineurin-like
MBR2721307.1
Clostridia bacterium
VanZ-2 Calcineurin-like
MBQ9773810.1
Clostridia bacterium
VanZ-2 Calcineurin-like
MBQ9784151.1
Clostridia bacterium
VanZ-2 Calcineurin-like
MBQ9760240.1
Clostridia bacterium
VanZ-2 Calcineurin-like
MBQ2734228.1
Clostridia bacterium
VanZ-2 Calcineurin-like
MBN1918588.1
Verrucomicrobiota bacterium
Calcineurin-like VanZ-2
WP_066056312.1
Robertmurraya korlensis NBRC 107688
Calcineurin-like VanZ-2
NQT82831.1
bacterium
Calcineurin-like VanZ-2
MBI1869671.1
Chlamydiota bacterium
Calcineurin-like VanZ-2
WP_151969048.1
Candidatus Uabimicrobium amorphum
Calcineurin-like ? VanZ-2
MCD5397870.1
candidate division NPL-UPA2 bacterium
Calcineurin-like VanZ-2
MBL7170538.1
Candidatus Omnitrophota bacterium
Calcineurin-like VanZ-2
HDO71138.1
bacterium
Calcineurin-like VanZ-2
WP_023625799.1
Mesobacillus boroniphilus JCM 21738
Calcineurin-like VanZ-2
MBN2191050.1
Candidatus Auribacterota bacterium
Calcineurin-like VanZ-2
###;VanZ-2 inactive fusion to a single C-terminal VanZ domain; #;possible connections to polysaccharide export/synthesis pathway components
MBN2019136.1
Sedimentisphaerales bacterium
VanZ-2 VanZ-1 MraY/WecA-GTase
WP_025543503.1
uncultured Oscillibacter sp.
UDP-N-acetylglucosamine-dehyd VanZ-2 VanZ-1
MBI9018966.1
Phycisphaerae bacterium
Wzc/etk/CpsD-like-P-loop-kinase VanZ-2 VanZ-1
MBN2063729.1
Sedimentisphaerales bacterium
Wzc/etk/CpsD-like-P-loop-kinase VanZ-2 VanZ-1
#;standalone versions
HEV56297.1
Phycisphaerales bacterium
VanZ-2 VanZ-1
MBN2138243.1
Sedimentisphaerales bacterium
VanZ-2 VanZ-1
MBL7185128.1
Phycisphaerae bacterium
VanZ-2 VanZ-1
MBN2313298.1
Sedimentisphaerales bacterium
VanZ-2 VanZ-1
OHB85370.1
Planctomycetes bacterium RBG_19FT_COMBO_48_8
VanZ-2 VanZ-1
HIJ67696.1
Planctomycetota bacterium
VanZ-2 VanZ-1
OHB64963.1
Planctomycetes bacterium RBG_13_50_24
VanZ-2 VanZ-1
MBN2595039.1
Sedimentisphaerales bacterium
VanZ-2 VanZ-1
HEG44181.1
Phycisphaerales bacterium
VanZ-2 VanZ-1
UCC98181.1
Phycisphaerales bacterium
VanZ-2 VanZ-1
OHB59227.1
Planctomycetes bacterium RBG_13_44_8b
VanZ-2 VanZ-1
OHB41947.1
Planctomycetes bacterium GWC2_45_44
VanZ-2 VanZ-1
MBN1787201.1
Sedimentisphaerales bacterium
VanZ-2 VanZ-1
OHB53140.1
Planctomycetes bacterium GWF2_41_51
VanZ-2 VanZ-1
MBN1362662.1
Sedimentisphaerales bacterium
VanZ-2 VanZ-1
NLZ07555.1
Phycisphaerae bacterium
VanZ-2 VanZ-1
MBN1509394.1
Sedimentisphaerales bacterium
VanZ-2 VanZ-1
MBW8000393.1
Planctomycetota bacterium
VanZ-2 VanZ-1
MBN1796614.1
Sedimentisphaerales bacterium
VanZ-2 VanZ-1

Accession	Organism	Domain architecture
MBP3510054.1	*Oscillibacter sp.*	VanZ-2, VanZ-1
MBN2457166.1	*Sedimentisphaerales bacterium*	VanZ-2, VanZ-1
MBI4848980.1	*Nitrospirota bacterium*	VanZ-2, VanZ-1
MBI5056093.1	*Nitrospirota bacterium*	VanZ-2, VanZ-1
RKY13540.1	*Planctomycetota bacterium*	VanZ-2, VanZ-1
NLH15388.1	*Phycisphaerae bacterium*	VanZ-2, VanZ-1
UCE49224.1	*Phycisphaerales bacterium*	VanZ-2, VanZ-1
UCF00183.1	*Planctomycetota bacterium*	VanZ-2, VanZ-1
MBN1973076.1	*Sedimentisphaerales bacterium*	VanZ-2, VanZ-1
MBN2130057.1	*Sedimentisphaerales bacterium*	VanZ-2, VanZ-1
UCG47214.1	*Phycisphaerales bacterium*	VanZ-2, VanZ-1
MBN1846739.1	*Sedimentisphaerales bacterium*	VanZ-2, VanZ-1
MCD4830676.1	*Anaerohalosphaeraceae bacterium*	VanZ-2, VanZ-1
OHB57407.1	*Planctomycetes bacterium GWF2_50_10*	VanZ-2, VanZ-1
HDZ69249.1	*Phycisphaerales bacterium*	VanZ-2, VanZ-1
MCD6393918.1	*Planctomycetota bacterium*	VanZ-2, VanZ-1
HDS85592.1	*Phycisphaerales bacterium*	VanZ-2, VanZ-1
NQV36088.1	*Phycisphaeraceae bacterium*	VanZ-2, VanZ-1
MBN2271956.1	*Sedimentisphaerales bacterium*	VanZ-2, VanZ-1
MBN2588955.1	*Sedimentisphaerales bacterium*	VanZ-2, VanZ-1
HCO96786.1	*Phycisphaerales bacterium*	VanZ-2, VanZ-1
MBN2456508.1	*Sedimentisphaerales bacterium*	VanZ-2, VanZ-1
MBN2181966.1	*Sedimentisphaerales bacterium*	VanZ-2, VanZ-1
OHB62064.1	*Planctomycetes bacterium RBG_13_46_10*	VanZ-2, VanZ-1
UCC21823.1	*Planctomycetota bacterium*	VanZ-2, VanZ-1
MBC8469644.1	*Planctomycetota bacterium*	VanZ-2, VanZ-1
OHB63452.1	*Planctomycetes bacterium RBG_13_50_24*	VanZ-2, VanZ-1
NIP26881.1		VanZ-2, VanZ-1
KPL21755.1	*Phycisphaerae bacterium SM1_79*	VanZ-2, VanZ-1
HEX42335.1	*Phycisphaerales bacterium*	VanZ-2, VanZ-1
MBL7107435.1	*Phycisphaerae bacterium*	VanZ-2, VanZ-1
MBN1436759.1	*Sedimentisphaerales bacterium*	VanZ-2, VanZ-1
MBN2375946.1	*Sedimentisphaerales bacterium*	VanZ-2, VanZ-1
MBN1765229.1	*Sedimentisphaerales bacterium*	VanZ-2, VanZ-1
MBW8015131.1	*Planctomycetota bacterium*	VanZ-2, VanZ-1
OQA00154.1	*Planctomycetes bacterium ADurb.Bin401*	VanZ-2, VanZ-1
WP_051314940.1	*Alteribacter aurantiacus DSM 18675*	VanZ-i, VanZ-2
WP_183163865.1	*Alteribacter keqinensis*	VanZ-i, VanZ-2
WP_078578764.1	*Salipaludibacillus agaradhaerens*	VanZ-i, VanZ-2
TVP84723.1	*Alkalicoccus sp.*	VanZ-i, VanZ-2
WP_066156682.1	*Halalkalibacter krulwichiae*	VanZ-i, VanZ-2
WP_013171898.1	*[Bacillus] selenitireducens MLS10*	VanZ-i, VanZ-2

####;other fusions, beginning with cNMPBD+FHA

Accession	Organism	Domain architecture
MBM4455764.1	*Verrucomicrobiota bacterium*	VanZ-2, cNMPBD, FHA
NBR71730.1	*Pseudomonadota bacterium*	VanZ-2, cNMPBD, FHA
MSP87311.1	*Alphaproteobacteria bacterium*	VanZ-2, cNMPBD, FHA
MBM3597839.1	*Alphaproteobacteria bacterium*	VanZ-2, cNMPBD, FHA

#;cNMPBD

Accession	Organism	Domain architecture
MBM76508.1	*Pseudomonadota bacterium*	VanZ-2, cNMPBD, ??
MBK8986180.1	*Candidatus Leptovillus gracilis*	VanZ-2, cNMPBD
MBO94666.1	*Opitutales bacterium*	VanZ-2, cNMPBD
HIE97610.1	*Fuerstiella sp.*	VanZ-2, cNMPBD
PDH31503.1	*Puniceicoccaceae bacterium MED-G30*	VanZ-2, cNMPBD
WP_168442711.1		VanZ-2, cNMPBD
MAA79081.1	*Deltaproteobacteria bacterium*	VanZ-2, cNMPBD
WP_013043071.1	*Coraliomargarita akajimensis DSM 45221*	VanZ-2, cNMPBD
MBL8822333.1	*Planctomycetia bacterium*	VanZ-2, cNMPBD

#;TM+VanZ+TM+Papain-like

Accession	Organism	Domain architecture
HFG98258.1		VanZ-2, Papain-like
HHX58399.1	*Candidatus Moraniibacteriota bacterium*	VanZ-2, Papain-like
PIP27930.1	*Candidatus Moranbacteria bacterium CG23_combo_of_CG06-09_8_20_14_all_35_22*	VanZ-2, Papain-like
PIW94986.1	*Candidatus Moranbacteria bacterium CG_4_8_14_3_um_filter_34_16*	VanZ-2, Papain-like
KKQ12328.1	*Candidatus Moranbacteria bacterium GW2011_GWF1_36_78*	VanZ-2, Papain-like
MBT4251506.1	*bacterium*	VanZ-2, Papain-like
MBQ1547455.1	*Lachnospiraceae bacterium*	VanZ-2, Papain-like

#;VanZ-2+KTSC-like

Accession	Organism	Domain architecture
TCR85789.1	*Rhizobium sp. BK376*	VanZ-2, KTSC-like
WP_143102824.1	*Rhizobium sp. NFR07*	VanZ-2, KTSC-like
MCA0254997.1	*Pseudomonadota bacterium*	VanZ-2, KTSC-like
WP_183792036.1	*Allorhizobium borbori*	VanZ-2, KTSC-like

#;uncharacterized domain fusion

Accession	Organism	Domain architecture
MBW2242798.1	*Deltaproteobacteria bacterium*	VanZ-2, unk1?
MCA9512836.1	*Myxococcales bacterium*	VanZ-2, unk1?
MAE95303.1	*Deltaproteobacteria bacterium*	VanZ-2, unk1?
SEH07324.1	*Candidatus Venteria ishoeyi*	VanZ-2, unk1?
MBA2433488.1	*Chthoniobacterales bacterium*	VanZ-2, unk1?
MBK18239.1	*Rhodospirillaceae bacterium*	VanZ-2, unk1?
MBD3872265.1	*Candidatus Sulfomarinibacter sp. MAG AM1*	VanZ-2, unk1?
OQA26987.1	*Verrucomicrobia bacterium ADurb.Bin345*	VanZ-2, unk1?
MAG20691.1	*Fidelibacterota bacterium*	VanZ-2, unk1?
MBW2467366.1	*Deltaproteobacteria bacterium*	VanZ-2, unk1?
HIE11414.1	*Kiritimatiellia bacterium*	VanZ-2, unk1?
MBN1269705.1	*Kiritimatiellia bacterium*	VanZ-2, unk1?
NCC52676.1	*Spartobacteria bacterium*	VanZ-2, unk1?

#;TPR repeats and b-barrel

Accession	Organism	Domain architecture
MBD3272072.1	*Elusimicrobiota bacterium*	VanZ-2, TPRs, TPRs, b-barrel
MBU2567778.1	*Elusimicrobiota bacterium*	VanZ-2, TPRs, TPRs, b-barrel
MBV9080669.1	*Elusimicrobiota bacterium*	VanZ-2, TPRs, TPRs, b-barrel
MBN1621238.1	*Endomicrobiales bacterium*	VanZ-2, TPRs, TPRs, b-barrel
OVE78233.1	*bacterium F11*	VanZ-2, TPRs, TPRs, b-barrel

#;TPR repeats and b-meander

Accession	Organism	Domain architecture
MBY0246057.1	*Nitrospiraceae bacterium*	VanZ-2, TPRs, TPRs, 4-strand-meander
TNF38400.1	*Gammaproteobacteria bacterium*	VanZ-2, TPRs, TPRs, 4-strand-meander
MBI5039670.1	*Gammaproteobacteria bacterium*	VanZ-2, TPRs, TPRs, 4-strand-meander
MBZ0071686.1	*Gammaproteobacteria bacterium*	VanZ-2, TPRs, TPRs, 4-strand-meander

#;TPR repeats

Accession	Organism	Domains
TXT35449.1	*Planctomycetota bacterium*	VanZ-2 – TPRs
BCS35255.1	*Luteitalea sp. TBR-22*	VanZ-2 – TPRs

#;HUP-like fusion

Accession	Organism	Domains
NCN25062.1	*Candidatus Falkowbacteria bacterium*	VanZ-2 – QueH-Epoxyqueuosine-reductase_HUP
NTW22659.1	*Candidatus Falkowbacteria bacterium*	VanZ-2 – QueH-Epoxyqueuosine-reductase_HUP
MBA3047499.1	*Candidatus Falkowbacteria bacterium*	VanZ-2 – QueH-Epoxyqueuosine-reductase_HUP
MBD3247965.1	*Candidatus Falkowbacteria bacterium*	VanZ-2 – QueH-Epoxyqueuosine-reductase_HUP
PIV51342.1	*Candidatus Falkowbacteria bacterium CG02_land_8_20_14_3_00_36_14*	VanZ-2 – QueH-Epoxyqueuosine-reductase_HUP
KKR21380.1	*Parcubacteria group bacterium GW2011_GWE2_39_37*	VanZ-2 – QueH-Epoxyqueuosine-reductase_HUP

#;CBD9-like fusion

Accession	Organism	Domains
MBT3341723.1	*Gemmatimonadota bacterium*	VanZ-2 – CBD9-like
HBP23168.1	*Planctomycetota bacterium*	VanZ-2 – CBD9-like
RMG13590.1	*Planctomycetota bacterium*	VanZ-2 – CBD9-like
MCA8926164.1	*Planctomycetota bacterium*	VanZ-2 – CBD9-like
MBL4889782.1	*Candidatus Lindowiibacteriota bacterium*	VanZ-2 – CBD9-like
RMH56165.1	*Candidatus Hydrogenedentota bacterium*	VanZ-2 – CBD9-like
MBP7653293.1	*Candidatus Babelota bacterium*	VanZ-2 – CBD9-like

#;Sulfatase fusions

Accession	Organism	Domains
GAK54151.1	*Candidatus Moduliflexus flocculans*	PA14 – VanZ-2 – Sulfatase
GAK58465.1	*Candidatus Vecturithrix granuli*	PA14 – VanZ-2 – Sulfatase
PIE35816.1	*candidate division KSB3 bacterium*	PA14 – VanZ-2 – Sulfatase
MBE83733.1	*Gemmatimonadota bacterium*	VanZ-2 – Sulfatase
MBJ95594.1	*Rickettsiales bacterium*	VanZ-2 – Sulfatase

#;TM+VanZ+TM?+TM?+CoA_binding-Rossmann+Polysacc_synt-Rossmann

Accession	Organism	Domains
MBS0343515.1	*Pseudomonadota bacterium*	VanZ-2 – TM? – TM? – CoA_binding-Rossmann – Polysacc_synt-Rossmann
WP_213235659.1	*Fusibacter paucivorans*	VanZ-2 – TM? – TM? – CoA_binding-Rossmann – Polysacc_synt-Rossmann
HAO78777.1	*Limisphaerales bacterium*	VanZ-2 – TM? – TM? – CoA_binding-Rossmann – Bac-transf
ODT39826.1	*Microbacterium sp. SCN 70-200*	CoA_binding-Rossmann – Bac-transf – VanZ-2
MBN2620133.1	*candidate division WOR-3 bacterium*	Bac_transf – VanZ-2
OGC43294.1	*candidate division WOR-3 bacterium RBG_13_43_14*	Bac_transf – VanZ-2
VUZ84807.1	*Candidatus Methylomirabilis lanthanidiphila*	VanZ-2 – Bac_transf

#;GT-B fusion

Accession	Organism	Domains
PWM83348.1	*Clostridium sp.*	VanZ-2 – Alg14_GT-B
MBS5682186.1	*Clostridium sp.*	VanZ-2 – Alg14_GT-B

#;Radical-SAM fusion

Accession	Organism	Domains
TYB30702.1	*Candidatus Mcinerneyibacterium aminivorans*	VanZ-2 – Radical_SAM
HDS03078.1	*Bacillota bacterium*	VanZ-2 – Radical_SAM

#;OmpA-like b-barrel fusion

Accession	Organism	Domains
MBP9050919.1	*Alphaproteobacteria bacterium*	VanZ-2 – OmpA-like-beta-barrel
MCB1562657.1	*Alphaproteobacteria bacterium*	VanZ-2 – OmpA-like-beta-barrel

#;NUDIX fusion

Accession	Organism	Domains
MBR0224600.1	*Thermoguttaceae bacterium*	VanZ-2 – NUDIX
MBS7338419.1	*Thermoguttaceae bacterium*	VanZ-2 – NUDIX

#######;transfer to fungi, typified by YJR112W in S. cereviseae

Accession	Organism	Domains
KAF9701354.1	*Ascochyta lentis*	? – VanZ-2 – tail
KAF4493530.1	*Fusarium agapanthi*	?? – VanZ-2 – tail
KND93014.1	*Tolypocladium ophioglossoides CBS 100239*	disordered – VanZ-2 – tail
RMX96530.1	*Hortaea werneckii*	VanZ-2 – tail
KAG9192168.1	*Alternaria panax*	tail – VanZ-2 – tail
KAG9374154.1		tail – VanZ-2 – tail
KZZ90038.1	*Ascosphaera apis ARSEF 7405*	VanZ-2 – tail(strand-strand)
EMR67799.1	*Eutypa lata UCREL1*	VanZ-2 – tail(strand-strand)
EPE02373.1	*Ophiostoma piceae UAMH 11346*	VanZ-2 – tail(strand-strand)
RMJ23348.1	*Aspergillus sp. HF37*	VanZ-2 – tail(strand-strand)
OJD13848.1	*Emergomyces pasteurianus Ep9510*	VanZ-2 – tail(strand-strand)
OBZ70859.1	*Grifola frondosa*	VanZ-2 – tail(strand-strand)
OJD24398.1	*Blastomyces percursus*	VanZ-2 – tail(strand-strand)
PGH18119.1	*Helicocarpus griseus UAMH5409*	VanZ-2 – tail(strand-strand)
KXL44508.1	*Acidomyces sp. 'richmondensis'*	VanZ-2 – tail(strand-strand)
TQB67778.1	*Monascus purpureus*	VanZ-2 – tail(strand-strand)
RMD40198.1	*Chaetothyriales sp. CBS 134920*	VanZ-2 – tail(strand-strand)
KAB5526401.1	*Coniochaeta sp. 2T2.1*	VanZ-2 – tail(strand-strand)
PMD42458.1	*Hyaloscypha variabilis F*	VanZ-2 – tail(strand-strand)
KAF7510208.1	*Endocarpon pusillum*	VanZ-2 – tail(strand-strand)
TKA28006.1	*Salinomyces thailandicus*	VanZ-2 – tail(strand-strand)
OIW26764.1	*Coniochaeta ligniaria NRRL 30616*	VanZ-2 – tail(strand-strand)
KAH3645965.1		VanZ-2 – tail(strand-strand)
OQD77964.1	*Penicillium decumbens*	VanZ-2 – tail(strand-strand)
KAF2723422.1	*Polychaeton citri CBS 116435*	VanZ-2 – tail(strand-strand)
KAF7191651.1	*Pseudocercospora fuligena*	VanZ-2 – tail(strand-strand)
KIV78406.1	*Exophiala sideris*	VanZ-2 – tail(strand-strand)
RMZ87148.1	*Chaetothyriales sp. CBS 134916*	VanZ-2 – tail(strand-strand)
RYP04782.1	*Monosporascus ibericus*	VanZ-2 – tail(strand-strand)
KKY25082.1	*Phaeomoniella chlamydospora*	VanZ-2 – tail(strand-strand)
KAE9994549.1	*Venturia inaequalis*	VanZ-2 – tail(strand-strand)
KAH0538041.1	*Glutinoglossum americanum*	VanZ-2 – tail(strand-strand)
KAF2764286.1	*Teratosphaeria nubilosa*	VanZ-2 – tail(strand-strand)
KGO72953.1	*Penicillium italicum*	VanZ-2 – tail(strand-strand)
TID13962.1	*Venturia nashicola*	VanZ-2 – tail(strand-strand)
OQD85790.1	*Penicillium antarcticum*	VanZ-2 – tail(strand-strand)
KUM55914.1	*Penicillium freii*	VanZ-2 – tail(strand-strand)
QDS71349.1	*Venturia effusa*	VanZ-2 – tail(strand-strand)
QQK46898.1	*Penicillium digitatum*	VanZ-2 – tail(strand-strand)
KAH3684855.1	*Wickerhamomyces pijperi*	VanZ-2 – tail(strand-strand)
OBT75739.1	*Pseudogymnoascus sp. 05NY08*	VanZ-2 – tail(strand-strand)
KAF3386584.1	*Penicillium rolfsii*	VanZ-2 – tail(strand-strand)
KAF2234748.1	*Viridothelium virens*	VanZ-2 – tail(strand-strand)
EOD51821.1	*Neofusicoccum parvum UCRNP2*	VanZ-2 – tail(strand-strand)
PPJ57755.1	*Cercospora berteroae*	VanZ-2 – tail(strand-strand)
KAF2175557.1	*Zopfia rhizophila CBS 207.26*	VanZ-2 – tail(strand-strand)
KAG7103250.1	*Verticillium longisporum*	VanZ-2 – tail(strand-strand)
PKS11212.1	*Lomentospora prolificans*	VanZ-2 – tail(strand-strand)
KAF2459464.1	*Lineolata rhizophorae*	VanZ-2 – tail(strand-strand)
KAF2737964.1	*Polyplosphaeria fusca*	VanZ-2 – tail(strand-strand)
KAF2859608.1	*Piedraia hortae CBS 480.64*	VanZ-2 – tail(strand-strand)

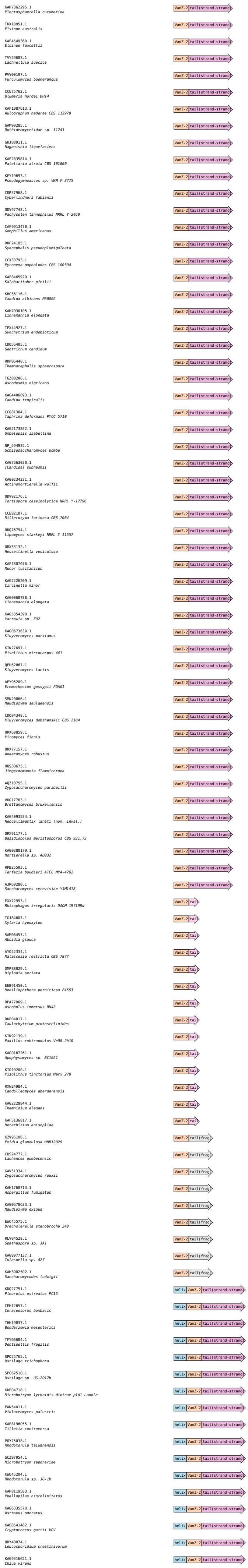
KAH7362295.1
Plectosphaerella cucumerina
VanZ-2 tail(strand-strand)
TKX18951.1
Elsinoe australis
VanZ-2 tail(strand-strand)
KAF4549360.1
Elsinoe fawcettii
VanZ-2 tail(strand-strand)
TVY59603.1
Lachnellula suecica
VanZ-2 tail(strand-strand)
PVV00197.1
Furculomyces boomerangus
VanZ-2 tail(strand-strand)
CCU75762.1
Blumeria hordei DH14
VanZ-2 tail(strand-strand)
KAF1987613.1
Aulographum hederae CBS 113979
VanZ-2 tail(strand-strand)
GAM90285.1
Dothideomycetidae sp. 11243
VanZ-2 tail(strand-strand)
GHJ88911.1
Naganishia liquefaciens
VanZ-2 tail(strand-strand)
KAF2835814.1
Patellaria atrata CBS 101060
VanZ-2 tail(strand-strand)
KFY19693.1
Pseudogymnoascus sp. VKM F-3775
VanZ-2 tail(strand-strand)
CDR37968.1
Cyberlindnera fabianii
VanZ-2 tail(strand-strand)
ODV97748.1
Pachysolen tannophilus NRRL Y-2460
VanZ-2 tail(strand-strand)
CAF9913470.1
Gomphillus americanus
VanZ-2 tail(strand-strand)
RKP24105.1
Syncephalis pseudoplumigaleata
VanZ-2 tail(strand-strand)
CCX33793.1
Pyronema omphalodes CBS 100304
VanZ-2 tail(strand-strand)
KAF8465929.1
Kalaharituber pfeilii
VanZ-2 tail(strand-strand)
KHC56116.1
Candida albicans P60002
VanZ-2 tail(strand-strand)
KAH7030105.1
Linnemannia elongata
VanZ-2 tail(strand-strand)
TPX44927.1
Synchytrium endobioticum
VanZ-2 tail(strand-strand)
CDO56405.1
Geotrichum candidum
VanZ-2 tail(strand-strand)
RKP06440.1
Thamnocephalis sphaerospora
VanZ-2 tail(strand-strand)
TGZ80206.1
Ascodesmis nigricans
VanZ-2 tail(strand-strand)
KAG4406893.1
Candida tropicalis
VanZ-2 tail(strand-strand)
CCG81304.1
Taphrina deformans PYCC 5710
VanZ-2 tail(strand-strand)
KAG2173852.1
Umbelopsis isabellina
VanZ-2 tail(strand-strand)
NP_594935.1
Schizosaccharomyces pombe
VanZ-2 tail(strand-strand)
KAG7663030.1
[Candida] subhashii
VanZ-2 tail(strand-strand)
KAG0234231.1
Actinomortierella wolfii
VanZ-2 tail(strand-strand)
ODV92176.1
Tortispora caseinolytica NRRL Y-17796
VanZ-2 tail(strand-strand)
CCE82107.1
Millerozyma farinosa CBS 7064
VanZ-2 tail(strand-strand)
ODQ76794.1
Lipomyces starkeyi NRRL Y-11557
VanZ-2 tail(strand-strand)
ORX53132.1
Hesseltinella vesiculosa
VanZ-2 tail(strand-strand)
KAF1807076.1
Mucor lusitanicus
VanZ-2 tail(strand-strand)
KAG2226209.1
Circinella minor
VanZ-2 tail(strand-strand)
KAG0068788.1
Linnemannia elongata
VanZ-2 tail(strand-strand)
KAG5354390.1
Yarrowia sp. E02
VanZ-2 tail(strand-strand)
KAG0673639.1
Kluyveromyces marxianus
VanZ-2 tail(strand-strand)
KIK27697.1
Pisolithus microcarpus 441
VanZ-2 tail(strand-strand)
QEU62867.1
Kluyveromyces lactis
VanZ-2 tail(strand-strand)
AEY95209.1
Eremothecium gossypii FDAG1
VanZ-2 tail(strand-strand)
SMN20866.1
Maudiozyma saulgeensis
VanZ-2 tail(strand-strand)
CDO94348.1
Kluyveromyces dobzhanskii CBS 2104
VanZ-2 tail(strand-strand)
ORX60859.1
Piromyces finnis
VanZ-2 tail(strand-strand)
ORX77157.1
Anaeromyces robustus
VanZ-2 tail(strand-strand)
RUS30673.1
Jimgerdemannia flammicorona
VanZ-2 tail(strand-strand)
AQZ18755.1
Zygosaccharomyces parabailii
VanZ-2 tail(strand-strand)
VUG17763.1
Brettanomyces bruxellensis
VanZ-2 tail(strand-strand)
KAG4093534.1
Neocallimastix lanati (nom. inval.)
VanZ-2 tail(strand-strand)
ORX91177.1
Basidiobolus meristosporus CBS 931.73
VanZ-2 tail(strand-strand)
KAG0380179.1
Mortierella sp. AD032
VanZ-2 tail(strand-strand)
RPB25503.1
Terfezia boudieri ATCC MYA-4762
VanZ-2 tail(strand-strand)
AJR69280.1
Saccharomyces cerevisiae YJM1418
VanZ-2 tail(strand-strand)
EXX72993.1
Rhizophagus irregularis DAOM 197198w
VanZ-2 tai
TGJ84607.1
Xylaria hypoxylon
VanZ-2 tai
SAM06457.1
Absidia glauca
VanZ-2 tai
AYO42334.1
Malassezia restricta CBS 7877
VanZ-2 tai
OMP88029.1
Diplodia seriata
VanZ-2 tai
EEB91450.1
Moniliophthora perniciosa FA553
VanZ-2 tai
RPA77969.1
Ascobolus immersus RN42
VanZ-2 tai
RKP04017.1
Caulochytrium protostelioides
VanZ-2 tai
KIK92139.1
Paxillus rubicundulus Ve08.2h10
VanZ-2 tai
KAG0167261.1
Apophysomyces sp. BC1021
VanZ-2 tai
KIO10200.1
Pisolithus tinctorius Marx 270
VanZ-2 tai
RXW24984.1
Candolleomyces aberdarensis
VanZ-2 tai
KAG2228844.1
Thamnidium elegans
VanZ-2 tai
KAF5136817.1
Metarhizium anisopliae
VanZ-2 tai
KZV95106.1
Exidia glandulosa HHB12029
VanZ-2 tail(frag)
CUS24772.1
Lachancea quebecensis
VanZ-2 tail(frag)
GAV51334.1
Zygosaccharomyces rouxii
VanZ-2 tail(frag)
KAH1760713.1
Aspergillus fumigatus
VanZ-2 tail(frag)
KAG0670633.1
Maudiozyma exigua
VanZ-2 tail(frag)
EWC45575.1
Drechslerella stenobrocha 248
VanZ-2 tail(frag)
RLV94528.1
Spathaspora sp. JA1
VanZ-2 tail(frag)
KAG8977137.1
Tulasnella sp. 427
VanZ-2 tail(frag)
KAH3902502.1
Saccharomycodes ludwigii
VanZ-2 tail(frag)
KDQ27751.1
Pleurotus ostreatus PC15
helix VanZ-2 tail(strand-strand)
CEH12657.1
Ceraceosorus bombacis
helix VanZ-2 tail(strand-strand)
THH19837.1
Bondarzewia mesenterica
helix VanZ-2 tail(strand-strand)
TFY66684.1
Dentipellis fragilis
helix VanZ-2 tail(strand-strand)
SPO25765.1
Ustilago trichophora
helix VanZ-2 tail(strand-strand)
SPC62510.1
Ustilago sp. UG-2017b
helix VanZ-2 tail(strand-strand)
KDE04718.1
Microbotryum lychnidis-dioicae p1A1 Lamole
helix VanZ-2 tail(strand-strand)
PWN54011.1
Violaceomyces palustris
helix VanZ-2 tail(strand-strand)
KAE8196055.1
Tilletia controversa
helix VanZ-2 tail(strand-strand)
POY75038.1
Rhodotorula taiwanensis
helix VanZ-2 tail(strand-strand)
SCZ97954.1
Microbotryum saponariae
helix VanZ-2 tail(strand-strand)
KWU45204.1
Rhodotorula sp. JG-1b
helix VanZ-2 tail(strand-strand)
KAH8119583.1
Phellopilus nigrolimitatus
helix VanZ-2 tail(strand-strand)
KAG6335570.1
Astraeus odoratus
helix VanZ-2 tail(strand-strand)
KAE8541482.1
Cryptococcus gattii VGV
helix VanZ-2 tail(strand-strand)
ORY40074.1
Leucosporidium creatinivorum
helix VanZ-2 tail(strand-strand)
KAG9316621.1
Chiua virens
helix VanZ-2 tail(strand-strand)

Accession	Organism	Domain architecture
KIJ67782.1	*Hydnomerulius pinastri MD-312*	helix VanZ-2 tail(strand-strand)
OCF30720.1	*Kwoniella heveanensis BCC8398*	helix VanZ-2 tail(strand-strand)
KXN90995.1	*Leucoagaricus sp. SymC.cos*	helix VanZ-2 tail(strand-strand)
KAF8973068.1	*Flammula alnicola*	helix VanZ-2 tail(strand-strand)
KAE8267843.1	*Tilletia walkeri*	helix VanZ-2 tail(strand-strand)
RDB25297.1	*Hypsizygus marmoreus*	helix VanZ-2 tail(strand-strand)
EPT04527.1	*Fomitopsis schrenkii*	helix VanZ-2 tail(strand-strand)
KAH0827867.1	*Lanmaoa asiatica*	helix VanZ-2 tail(strand-strand)
RPD57637.1	*Lentinus tigrinus ALCF2SS1-6*	helix VanZ-2 tail(strand-strand)
KIM83242.1	*Piloderma croceum F 1598*	helix VanZ-2 tail(strand-strand)
KIJ49552.1	*Sphaerobolus stellatus SS14*	helix VanZ-2 tail(strand-strand)
KAG9012765.1	*Tulasnella sp. JGI-2019a*	helix VanZ-2 tail(strand-strand)
KAH7916560.1	*Hygrophoropsis aurantiaca*	helix VanZ-2 tail(strand-strand)
EGU10877.1	*Rhodotorula toruloides ATCC 204091*	helix VanZ-2 tail(strand-strand)
PAV19030.1	*Pyrrhoderma noxium*	helix VanZ-2 tail(strand-strand)
OCH85092.1	*Obba rivulosa*	helix VanZ-2 tail(strand-strand)
KZS98687.1	*Sistotremastrum niveocremeum HHB9708*	helix VanZ-2 tail(strand-strand)
KAG5172993.1		helix VanZ-2 tail(strand-strand)
KAF8070675.1	*Lyophyllum atratum*	helix VanZ-2 tail(strand-strand)
KAF8554203.1	*Imleria badia*	helix VanZ-2 tail(strand-strand)
KAG8928776.1	*Tulasnella sp. 418*	helix VanZ-2 tail(strand-strand)
KAF5326148.1	*Tulosesus angulatus*	helix VanZ-2 tail(strand-strand)
KDQ10071.1	*Botryobasidium botryosum FD-172 SS1*	helix VanZ-2 tail(strand-strand)
THH07986.1	*Phellinidium pouzarii*	helix VanZ-2 tail(strand-strand)
KIY44424.1	*Fistulina hepatica ATCC 64428*	helix VanZ-2 tail(strand-strand)
KAG2015698.1	*Coprinopsis cinerea AmutBmut pab1-1*	helix VanZ-2 tail(strand-strand)
KAF8320714.1	*Clavulina sp. PMI_390*	helix VanZ-2 tail(strand-strand)
PBK91427.1	*Armillaria gallica*	helix VanZ-2 tail(strand-strand)
THH28744.1	*Antrodiella citrinella*	helix VanZ-2 tail(strand-strand)
KAF8236613.1	*Tricholoma matsutake 945*	helix VanZ-2 tail(strand-strand)
KAF9567967.1	*Agrocybe pediades*	helix VanZ-2 tail(strand-strand)
KZV59332.1	*Peniophora sp. CONT*	helix VanZ-2 tail(strand-strand)
TRM69510.1	*Schizophyllum amplum*	helix VanZ-2 tail(strand-strand)
EJD52655.1	*Auricularia subglabra TFB-10046 SS5*	helix VanZ-2 tail(strand-strand)
KIL65909.1	*Amanita muscaria Koide BX008*	helix VanZ-2 tail(strand-strand)
PPQ98772.1	*Panaeolus cyanescens*	helix VanZ-2 tail(strand-strand)
KAF9050015.1	*Panaeolus papilionaceus*	helix VanZ-2 tail(strand-strand)
KAF8197850.1	*Pholiota molesta*	helix VanZ-2 tail(strand-strand)
KIK67155.1	*Collybiopsis luxurians FD-317 M1*	helix VanZ-2 tail(strand-strand)
PPR05859.1	*Gymnopilus dilepis*	helix VanZ-2 tail(strand-strand)
KAF8635191.1	*Amanita inopinata Kibby_2008*	helix VanZ-2 tail(strand-strand)
CAD6580543.1	*Tremellales sp. Tagirdzhanova-0007*	helix VanZ-2 tail(strand-strand)
KIM26980.1	*Serendipita vermifera MAFF 305830*	helix VanZ-2 tail(strand-strand)
KDR75156.1	*Galerina marginata CBS 339.88*	helix VanZ-2 tail(strand-strand)
KAH8828277.1	*Flagelloscypha sp. PMI_526*	helix VanZ-2 tail(strand-strand)
KAG8826223.1	*Serendipita sp. 401*	helix VanZ-2 tail(strand-strand)
KAF9529236.1	*Crepidotus variabilis*	helix VanZ-2 tail(strand-strand)
CAE6402197.1	*Rhizoctonia solani*	helix VanZ-2 tail(strand-strand)
GAW04339.1	*Lentinula edodes*	helix VanZ-2 tail(strand-strand)
OCF58458.1	*Kwoniella mangroviensis CBS 10435*	helix VanZ-2 tail(strand-strand)
KAH8979207.1	*Lactarius akahatsu*	helix VanZ-2 tail(strand-strand)
KAH7108159.1	*Auriculariales sp. MPI-PUGE-AT-0066*	helix VanZ-2 tail(strand-strand)
PPQ68040.1	*Psilocybe cyanescens*	helix VanZ-2 tail(strand-strand)
KAG7579994.1	*Filobasidium floriforme*	helix VanZ-2 tail(strand-strand)
OQO01689.1	*Cryoendolithus antarcticus*	helix VanZ-2 tail(strand-strand)
KAF8650417.1	*Volvariella volvacea WC 439*	helix VanZ-2 tail(strand-strand)
KAG8949101.1	*Tulasnella sp. 424*	helix VanZ-2 tail(strand-strand)
TFK68688.1	*Pluteus cervinus*	helix VanZ-2 tail(strand-strand)
TFL05724.1	*Pterulicium gracile*	helix VanZ-2 tail(strand-strand)
KAF8919903.1	*Mucidula mucida*	helix VanZ-2 tail(strand-strand)
KZP05940.1	*Athelia psychrophila*	helix VanZ-2 tail(strand-strand)
PKI85114.1	*Malassezia vespertilionis*	helix VanZ-2 tail(strand-strand)
KIY74329.1	*Cylindrobasidium torrendii FP15055 ss-10*	helix VanZ-2 tail(strand-strand)
KAF5321403.1	*Psilocybe cf. subviscida*	helix VanZ-2 tail(strand-strand)
KAF9070092.1	*Rhodocollybia butyracea*	helix VanZ-2 tail(strand-strand)
KAF8608861.1	*Ceratobasidium sp. AG-I*	helix VanZ-2 tail(strand-strand)
RXK41768.1	*Tremella mesenterica*	helix VanZ-2 tail(strand-strand)
KAE9411210.1	*Gymnopus androsaceus JB14*	helix VanZ-2 tail(strand-strand)
KAG0142119.1	*Cronartium quercuum f. sp. fusiforme G11*	helix VanZ-2 tail(strand-strand)
KAG0642861.1	*Tuber brumale*	helix VanZ-2 tail(strand-strand)
SHO77590.1	*Malassezia sympodialis ATCC 42132*	helix VanZ-2 tail(strand-strand)
CDZ96501.1	*Phaffia rhodozyma*	helix VanZ-2 tail(strand-strand)
KAF0726906.1	*Aphanomyces euteiches*	VanZ-2
OQR92739.1		VanZ-2
KAF0700509.1	*Aphanomyces stellatus*	VanZ-2
XP_008607515.1	*Saprolegnia diclina VS20*	VanZ-2

#######;fusions to additional TM segments (relatively rare in comparison to VanZ-1);
#;TM+TM+VanZ-2+TM+TM

Accession	Organism	Domain architecture
MBI5099364.1	*Nitrospirota bacterium*	VanZ-2
MBI5740083.1	*Nitrospirota bacterium*	VanZ-2

#;TM+TM+TM+VanZ+TM

Accession	Organism	Domain architecture
GBE40841.1	*bacterium BMS3Bbin09*	VanZ-2
RJQ50085.1	*Nitrospiraceae bacterium*	VanZ-2
UCD36226.1	*Nitrospiraceae bacterium*	VanZ-2

#;5TM+VanZ

Accession	Organism	Domain architecture
MBI5705713.1	*Armatimonadota bacterium*	5TM VanZ-2
MCB0826264.1	*Armatimonadota bacterium*	5TM VanZ-2
QYK54111.1	*Fimbriimonadaceae bacterium*	5TM VanZ-2

#;TM+VanZ-2+disordered

Accession	Organism	Domain architecture
RDG34608.1	*Streptomyces corynorhini*	VanZ-2 disordered
RGD65726.1	*Lachnospiraceae bacterium OF09-6*	VanZ-2 disordered
NUO57330.1	*Hamadaea sp.*	VanZ-2 disordered
SEA03217.1	*Lachnospiraceae bacterium NK3A20*	VanZ-2 disordered
HIR12562.1	*Candidatus Choladousia intestinavium*	VanZ-2 disordered
MBO7711016.1	*Lachnospiraceae bacterium*	VanZ-2 disordered

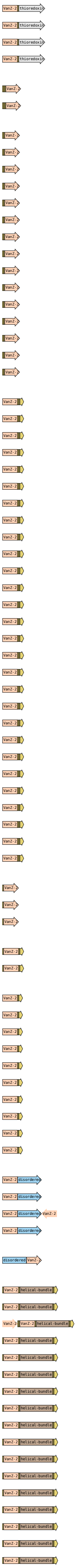

#;TM+VanZ+TM+thioredoxin

TDI33348.1
Acidobacteriota bacterium

NLO08023.1
candidate division WS1 bacterium

PYT08477.1
Acidobacteriota bacterium

MBI3450977.1
Acidobacteriota bacterium

#;TM+TM+TM+VanZ-2

QYK54794.1
Fimbriimonadaceae bacterium

MCC6402558.1
Fimbriimonadaceae bacterium

#;TM+TM+VanZ-2

MBQ2770605.1
Clostridia bacterium

WP_143009228.1
Alkalibacterium thalassium

MSS69919.1
Candidatus Latescibacterota bacterium

WP_197017726.1
Alkalibacterium sp. AK22

PWL71261.1

MXY44042.1
Dehalococcoidia bacterium

PWT95194.1
Bacteroidota bacterium

MBI2556720.1
Planctomycetota bacterium

MBN2205310.1
Thermoleophilia bacterium

CAB5118525.1
Olavius algarvensis associated proteobacterium Delta 3

HDL64448.1
Pseudomonadota bacterium

WP_051929592.1
Carnobacterium mobile DSM 4848

NCD19247.1
Actinomycetota bacterium

WP_051912428.1
Carnobacterium funditum DSM 5970

SIO18173.1
Carnobacterium alterfunditum

#;VanZ-2+TM+TM

PYM61160.1
Candidatus Rokuibacteriota bacterium

MBI3458213.1
Candidatus Rokuibacteriota bacterium

PYN38959.1
Candidatus Rokuibacteriota bacterium

HEU49820.1

PYN81019.1
Candidatus Rokuibacteriota bacterium

OLC17723.1
Candidatus Rokubacteria bacterium 13_1_40CM_69_27

MBI1962186.1
Candidatus Rokuibacteriota bacterium

MBI3625003.1
Candidatus Rokuibacteriota bacterium

MBI1893311.1
Candidatus Rokuibacteriota bacterium

MBI4012460.1
Candidatus Rokuibacteriota bacterium

MBI2468267.1
Candidatus Rokuibacteriota bacterium

MBI2461367.1
Candidatus Rokuibacteriota bacterium

MBI2154527.1
Candidatus Rokuibacteriota bacterium

OLD37401.1
Candidatus Rokubacteria bacterium 13_1_40CM_2_68_8

MBI4245581.1
Candidatus Rokuibacteriota bacterium

TMQ15735.1
Candidatus Rokuibacteriota bacterium

MBI4611651.1
Candidatus Rokuibacteriota bacterium

HGH12835.1

MBI2161127.1
Candidatus Rokuibacteriota bacterium

OGL10895.1
Candidatus Rokubacteria bacterium RIFCSPLOWO2_02_FULL_73_56

MBI1737388.1
Candidatus Rokuibacteriota bacterium

PYM84679.1
Candidatus Rokuibacteriota bacterium

HAM54850.1
Candidatus Rokuibacteriota bacterium

HEJ31679.1

TAJ98766.1
Candidatus Manganitrophaceae bacterium

MBI3802721.1
Candidatus Manganitrophaceae bacterium

HIE65182.1
Candidatus Manganitrophaceae bacterium

NOY84224.1
Candidatus Manganitrophaceae bacterium

#;TM+VanZ-2

WP_060799876.1
Peptoniphilus harei

MBW3622360.1
Armatimonadota bacterium

MBO4322001.1
Clostridia bacterium

#;TM+VanZ-2+TM

MBE5793437.1
Clostridiales bacterium

HIR52846.1
Candidatus Onthovicinus excrementipullorum

#;VanZ-2+TM

MCA1648579.1
Chloroflexota bacterium

MBP3486476.1
Oscillospiraceae bacterium

MAF65370.1
Planctomycetota bacterium

MSR61321.1
Planctomycetota bacterium

QDU66304.1
Engelhardtia mirabilis

HIG11899.1
Planctomycetota bacterium

MAB79024.1
Planctomycetota bacterium

MCA8980160.1
Planctomycetota bacterium

MBL8695170.1
Planctomycetota bacterium

MBL6720888.1
Planctomycetota bacterium

#;VanZ-2+disordered

MCB1360515.1
Paracoccaceae bacterium

MCB1396072.1
Paracoccaceae bacterium

MBS1813752.1
Acidobacteriota bacterium

WP_204651518.1
uncultured Gemmiger sp.

#;disordered+VanZ-2

OQY25151.1
Anaerolineaceae bacterium 4572_32.1

#;TM+VanZ-2+TM+helical-bundle+TM

REJ74666.1
Acidobacteriota bacterium

MBI4264976.1
Acidobacteriota bacterium

MCB1055780.1
Acidobacteriota bacterium

MBP7755118.1
Thermoanaerobaculia bacterium

MBA2304613.1
Acidobacteriota bacterium

MBA3295820.1
Acidobacteriota bacterium

MBL8142181.1
Acidobacteriota bacterium

MBI3263581.1
Acidobacteriota bacterium

MBY0495255.1
Cyanobacteriota bacterium

MBM40682.1
Acidobacteriota bacterium

MBQ00773.1
Acidobacteriota bacterium

RMH20134.1
Acidobacteriota bacterium

OFW08835.1
Acidobacteria bacterium RIFCSPLOWO2_02_FULL_67_36

MBM3809193.1
Acidimicrobiia bacterium

OFW46780.1
Acidobacteria bacterium RIFCSPLOWO2_12_FULL_67_14b

OFW00657.1
Acidobacteria bacterium RIFCSPLOWO2_02_FULL_68_18

MBI4888822.1
Acidobacteriota bacterium

MBW8894691.1 — VanZ-2 helical-bundle
Acidobacteriota bacterium

MBA2257885.1 — VanZ-2 helical-bundle
Acidobacteriota bacterium

MCA1650487.1 — VanZ-2 helical-bundle
Acidobacteriota bacterium

#;TM+TM+VanZ+TM+TM+TM

MBS29118.1 — VanZ-2
Alphaproteobacteria bacterium

MBL4720555.1 — VanZ-2
Alphaproteobacteria bacterium

MBO6783712.1 — VanZ-2
Alphaproteobacteria bacterium

MBP6819449.1 — VanZ-2
Ferrovibrio sp.

MBX3453310.1 — VanZ-2
Ferrovibrio sp.

MBT5107126.1 — VanZ-2
Rhodospirillaceae bacterium

NOX80585.1 — VanZ-2
Deltaproteobacteria bacterium

HBI15442.1 — VanZ-2

THB74555.1 — VanZ-2
Desulfobulbaceae bacterium

NOQ45828.1 — VanZ-2
Desulfobulbaceae bacterium

KJS00431.1 — VanZ-2
Desulfobulbaceae bacterium BRH_c16a

RJX23767.1 — VanZ-2
Desulfobulbus sp.

MBT8345919.1 — VanZ-2
Desulfofustis sp.

WP_163338278.1 — VanZ-2
Desulfopila sp. IMCC35008

# 15;TM+TM+TM+VanZ+TM+TM+TM

MBU0732788.1 — VanZ-2
Pseudomonadota bacterium

MBC8179326.1 — VanZ-2
Candidatus Desulfacyla euxinica

RLA99858.1 — VanZ-2
Deltaproteobacteria bacterium

MBN2254311.1 — VanZ-2
Deltaproteobacteria bacterium

MBW2354235.1 — VanZ-2
Deltaproteobacteria bacterium

MAF32938.1 — VanZ-2
Desulfobacter sp.

NNL74769.1 — VanZ-2
Desulfobacterales bacterium

UCD79122.1 — VanZ-2
Desulfobacterales bacterium

UCD33646.1 — VanZ-2
Desulfobacterales bacterium

RME07745.1 — VanZ-2
Anaerolineae bacterium

NPV40373.1 — VanZ-2
Anaerolineae bacterium

NMB59819.1 — VanZ-2
Chloroflexota bacterium

NLF51137.1 — VanZ-2
Leptolinea sp.

NMB54863.1 — VanZ-2
Leptolinea sp.

MBU2510993.1 — VanZ-2
bacterium

# 10;TM+TM+TM+VanZ+TM+TM

NTV84042.1 — VanZ-2
Bacteroidales bacterium

NNC84631.1 — VanZ-2
Bacteroidia bacterium

MBL7799148.1 — VanZ-2
Chitinophagales bacterium

NBU51361.1 — VanZ-2 sugar-transferase-GT-A
Chitinophagia bacterium

PHN04426.1 — VanZ-2
Flavilitoribacter nigricans DSM 23189 = NBRC 102662

MCB0520269.1 — LPS-like-sugar-transf VanZ-2
Saprospiraceae bacterium

RMG00576.1 — VanZ-2
Nitrospirota bacterium

MCB9865050.1 — VanZ-2
Phycisphaerales bacterium

OQY98902.1 — VanZ-2
Planctomycetes bacterium UTPLA1

MBI5762061.1 — VanZ-2
Planctomycetota bacterium

OGJ94200.1 — VanZ-2
Candidatus Raymondbacteria bacterium RIFOXYA2_FULL_49_16

MBN1129447.1 — VanZ-2
Chitinispirillaceae bacterium

MBN1307110.1 — VanZ-2
Chitinispirillaceae bacterium

MBN2037804.1 — VanZ-2
Chitinispirillaceae bacterium

MBD3347417.1 — VanZ-2
Chitinivibrionales bacterium

NLP03395.1 — VanZ-2
Fibrobacter sp.

MBF0430847.1 — VanZ-2
Fibrobacteria bacterium

##########;standalone or largely standalone versions on the genome

NUM35524.1 — VanZ-2
Candidatus Brocadiia bacterium

ACJ27947.1 — VanZ-2
Shewanella piezotolerans WP3

AZQ11439.1 — VanZ-2
Shewanella khirikhana

MCC5983948.1 — VanZ-2
Paracoccaceae bacterium

WP_101090034.1 — VanZ-2
Shewanella sp. Bg11-22

WP_161351465.1 — VanZ-2
Maritimibacter harenae

MBL6855414.1 — VanZ-2
Paracoccaceae bacterium

NOY45549.1 — VanZ-2
Deltaproteobacteria bacterium

RHS04985.1 — VanZ-2
Coprobacillus sp. AF13-4LB

MBO4392575.1 — VanZ-2
Clostridia bacterium

MBI1709820.1 — VanZ-2
Lactobacillus crispatus

SEI89794.1 — VanZ-2
Lachnospiraceae bacterium A10

HDX97997.1 — VanZ-2

MBE6635716.1 — VanZ-2
Oscillospiraceae bacterium

WP_014787162.1 — VanZ-2
Terriglobus roseus DSM 18391

HIU21414.1 — VanZ-2
Candidatus Limadaptatus stercorigallinarum

MBE5846707.1 — VanZ-2
Lachnospiraceae bacterium

MBE5833777.1 — VanZ-2
Butyrivibrio sp.

SHJ88965.1 — VanZ-2
Desulfofundulus thermosubterraneus DSM 16057

MBM7522434.1 — VanZ-2

WP_153724174.1 — VanZ-2
Heliorestis convoluta

PGL69610.1 — VanZ-2
Bacillus sp. AFS055030

MBM3846416.1 — VanZ-2
Verrucomicrobiota bacterium

PYM23294.1 — VanZ-2
Candidatus Rokuibacteriota bacterium

HEE72215.1 — VanZ-2

MBM3902565.1 — VanZ-2
Verrucomicrobiota bacterium

RLA87800.1 — VanZ-2
Deltaproteobacteria bacterium

HDX59766.1 — VanZ-2

MBQ6717575.1 — VanZ-2
Clostridia bacterium

MBP7059343.1 — VanZ-2
Lachnospiraceae bacterium

MBQ9030724.1 — VanZ-2
Parasporobacterium sp.

MBE6067091.1 — VanZ-2
Clostridium lundense

MCD8020728.1 — VanZ-2
Clostridiales bacterium

MBR4026926.1 — VanZ-2
Lachnospiraceae bacterium

APW62368.1 — VanZ-2
Paludisphaera borealis

OQC33552.1 — VanZ-2
Verrucomicrobia bacterium ADurb.Bin070

MBM6998996.1 — VanZ-2
bacterium

EEC56990.1 — VanZ-2
[Bacteroides] pectinophilus ATCC 43243

NOY40382.1 — VanZ-2
Planctomycetota bacterium

MBR0409659.1 — VanZ-2
Eubacterium sp.

MBO6214999.1 — VanZ-2
Lachnospiraceae bacterium

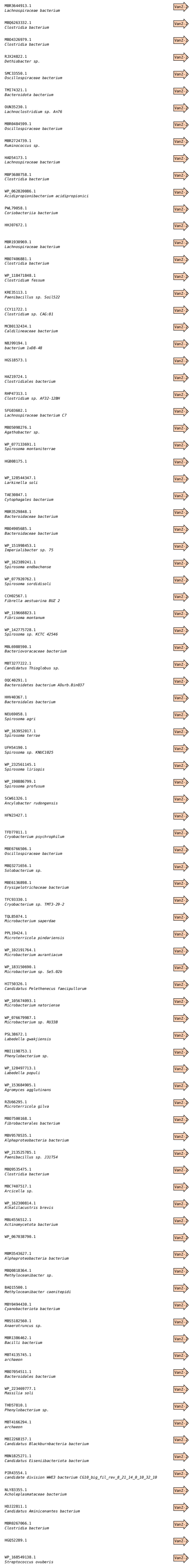
MBR3644913.1
Lachnospiraceae bacterium
MBQ6263332.1
Clostridia bacterium
MBO4326979.1
Clostridia bacterium
RJX24822.1
Dethiobacter sp.
SMC33550.1
Oscillospiraceae bacterium
TMI74321.1
Bacteroidota bacterium
OUN35230.1
Lachnoclostridium sp. An76
MBR0484599.1
Oscillospiraceae bacterium
MBR2724739.1
Ruminococcus sp.
HAD54173.1
Lachnospiraceae bacterium
MBP3680758.1
Clostridia bacterium
WP_062820086.1
Acidipropionibacterium acidipropionici
PWL79058.1
Coriobacteriia bacterium
HHJ07672.1
MBR1930969.1
Lachnospiraceae bacterium
MBO7406881.1
Clostridia bacterium
WP_118471848.1
Clostridium fessum
KRE35113.1
Paenibacillus sp. Soil522
CCY11722.1
Clostridium sp. CAG:81
MCB0132434.1
Caldilineaceae bacterium
NBJ99194.1
bacterium 1xD8-48
HGS18573.1
HAZ19724.1
Clostridiales bacterium
RHP47313.1
Clostridium sp. AF32-12BH
SFG03602.1
Lachnospiraceae bacterium C7
MBO5098276.1
Agathobacter sp.
WP_077133691.1
Spirosoma montaniterrae
HGB08175.1
WP_128544347.1
Larkinella soli
TAE30847.1
Cytophagales bacterium
MBR3529848.1
Bacteroidaceae bacterium
MBO4905685.1
Bacteroidaceae bacterium
WP_151998453.1
Imperialibacter sp. 75
WP_162389241.1
Spirosoma endbachense
WP_077920762.1
Spirosoma sordidisoli
CCH02567.1
Fibrella aestuarina BUZ 2
WP_119668823.1
Fibrisoma montanum
WP_142775728.1
Spirosoma sp. KCTC 42546
MBL6988590.1
Bacteriovoracaceae bacterium
MBT3277222.1
Candidatus Thioglobus sp.
OQC40291.1
Bacteroidetes bacterium ADurb.Bin037
HHV40367.1
Bacteroidales bacterium
NEU69058.1
Spirosoma agri
WP_163952017.1
Spirosoma terrae
UFH54190.1
Spirosoma sp. KNUC1025
WP_232561145.1
Spirosoma liriopis
WP_190886799.1
Spirosoma profusum
SCW61326.1
Ancylobacter rudongensis
HFN23427.1
TFD77011.1
Cryobacterium psychrophilum
MBE6766506.1
Oscillospiraceae bacterium
MBQ3271656.1
Solobacterium sp.
MBE6136898.1
Erysipelotrichaceae bacterium
TFC93330.1
Cryobacterium sp. TMT3-29-2
TQL85074.1
Microbacterium saperdae
PPL19424.1
Microterricola pindariensis
WP_102191764.1
Microbacterium aurantiacum
WP_183150690.1
Microbacterium sp. Se5.02b
HIT50326.1
Candidatus Pelethenecus faecipullorum
WP_105674093.1
Microbacterium natoriense
WP_076679987.1
Microbacterium sp. RU33B
PSL38672.1
Labedella gwakjiensis
MBI1198753.1
Phenylobacterium sp.
WP_128497713.1
Labedella populi
WP_153684905.1
Agromyces agglutinans
RZU66295.1
Microterricola gilva
MBO7500168.1
Fibrobacterales bacterium
MBV9570535.1
Alphaproteobacteria bacterium
WP_213525785.1
Paenibacillus sp. J31TS4
MBQ9535475.1
Clostridia bacterium
MBC7407517.1
Arcicella sp.
WP_162300814.1
Alkalilacustris brevis
MBU4556512.1
Actinomycetota bacterium
WP_067038790.1
MBM3543627.1
Alphaproteobacteria bacterium
MBQ0818364.1
Methyloceanibacter sp.
BAQ15500.1
Methyloceanibacter caenitepidi
MBY0494430.1
Cyanobacteriota bacterium
MBS5182560.1
Anaerotruncus sp.
MBR1386462.1
Bacilli bacterium
MBT4135745.1
archaeon
MBO7054511.1
Bacteroidales bacterium
WP_223469777.1
Massilia soli
THD57810.1
Phenylobacterium sp.
MBT4166294.1
archaeon
MBI2268157.1
Candidatus Blackburnbacteria bacterium
MBN1825271.1
Candidatus Eiseniibacteriota bacterium
PIR43554.1
candidate division WWE3 bacterium CG10_big_fil_rev_8_21_14_0_10_32_10
NLY83355.1
Acholeplasmataceae bacterium
HDJ22811.1
Candidatus Aminicenantes bacterium
MBR0267066.1
Clostridia bacterium
HGQ52289.1
WP_168549138.1
Streptococcus ovuberis

QCC76395.1	*Nocardioides daphniae*	VanZ-2
QMW80804.1	*Blautia producta*	VanZ-2
HAW15887.1	*Clostridiales bacterium*	VanZ-2
OUQ43411.1	*Drancourtella sp. An12*	VanZ-2
WP_161594088.1	*Microbacterium sp. RG1*	VanZ-2
EJP24459.1	*Streptococcus anginosus SK1138*	VanZ-2
OLD63075.1	*Acidobacteria bacterium 13_1_40CM_2_60_7*	VanZ-2
MBE5862323.1	*Lachnospiraceae bacterium*	VanZ-2
MBI3770806.1	*Deltaproteobacteria bacterium*	VanZ-2
NLB48205.1	*Microbacteriaceae bacterium*	VanZ-2
MBR6516360.1	*Bacilli bacterium*	VanZ-2
NLM65146.1	*Bacillota bacterium*	VanZ-2
TMA83065.1	*Deltaproteobacteria bacterium*	VanZ-2
MBR0446980.1	*Clostridia bacterium*	VanZ-2
MBR6326544.1	*Lachnospiraceae bacterium*	VanZ-2
HHW54759.1	*Bacillota bacterium*	VanZ-2
EJC65946.1	*Rhizobium leguminosarum bv. viciae WSM1455*	VanZ-2
MBQ4131709.1	*Clostridia bacterium*	VanZ-2
WP_118374223.1		VanZ-2
MBO5411672.1	*Clostridia bacterium*	VanZ-2
MTI94737.1	*Bacillota bacterium*	VanZ-2
NLY69581.1	*Clostridiales bacterium*	VanZ-2
ETD13724.1	*Streptococcus parasanguinis CC87K*	VanZ-2
HEE07772.1		VanZ-2
MCD7836033.1	*Lachnospiraceae bacterium*	VanZ-2
WP_013490228.1	*Evansella cellulosilytica DSM 2522*	VanZ-2
WP_195416391.1	*Enterocloster citroniae*	VanZ-2
PZU91692.1	*Chryseobacterium sp.*	VanZ-2
GGC33947.1	*Brevibacterium sediminis*	VanZ-2
MAY51278.1	*Microbacterium sp.*	VanZ-2
NDC37971.1	*Pseudomonadota bacterium*	VanZ-2
MBE6954709.1	*Oscillospiraceae bacterium*	VanZ-2
MBR3304747.1	*Christensenellaceae bacterium*	VanZ-2
MBR4733510.1	*Lachnospiraceae bacterium*	VanZ-2
TDI85178.1	*Caldithrix sp.*	VanZ-2
WP_105332378.1	*Bremerella cremea*	VanZ-2
HDZ09840.1	*Pseudohongiella sp.*	VanZ-2
MBQ2953158.1	*Clostridia bacterium*	VanZ-2
KAA0977921.1	*Paeniglutamicibacter gangotriensis*	VanZ-2
EEG48635.1	*Blautia hydrogenotrophica DSM 10507*	VanZ-2
MBA3242867.1	*Acidobacteriota bacterium*	VanZ-2
WP_192926427.1	*Microvirga tunisiensis*	VanZ-2
MCA1566929.1	*Acidobacteriota bacterium*	VanZ-2
MBR3503976.1	*Clostridia bacterium*	VanZ-2
WP_204502106.1	*Aquibacillus albus*	VanZ-2
MBP3580985.1	*Clostridia bacterium*	VanZ-2
WP_182436766.1	*Clostridium sp. OF09-36*	VanZ-2
MBA2502439.1	*Pyrinomonadaceae bacterium*	VanZ-2
MBR5313330.1	*Clostridia bacterium*	VanZ-2
PYS59037.1	*Acidobacteriota bacterium*	VanZ-2
WP_144209938.1	*Streptococcus constellatus*	VanZ-2
CDC43200.1	*Firmicutes bacterium CAG:424*	VanZ-2
MBB3731822.1	*Nonomuraea dietziae*	VanZ-2
MBQ2708550.1	*Clostridia bacterium*	VanZ-2
PYS44129.1	*Acidobacteriota bacterium*	VanZ-2
SEO19790.1	*Actinacidiphila rubida*	VanZ-2
MBU2520812.1	*Pseudomonadota bacterium*	VanZ-2
RLE33645.1	*Acidobacteriota bacterium*	VanZ-2
MBD7958268.1		VanZ-2
MBR3645130.1	*Lachnospiraceae bacterium*	VanZ-2
MBD9125409.1	*Lachnospiraceae bacterium*	VanZ-2
HBR57985.1	*Blastocatellia bacterium*	VanZ-2
PYJ61264.1	*Verrucomicrobiota bacterium*	VanZ-2
WP_106402105.1	*Actinocorallia populi*	VanZ-2
MBS0163831.1	*Nitrospira sp.*	VanZ-2
OBF41134.1	*Mycobacterium sp. ACS1612*	VanZ-2
HAP33560.1	*Lachnospiraceae bacterium*	VanZ-2
WP_231872708.1	*Microbacterium oleivorans*	VanZ-2
MCC5909584.1	*Clostridiaceae bacterium*	VanZ-2
MCD4685903.1	*Anaerolineae bacterium*	VanZ-2
MBD5554688.1	*Roseburia sp.*	VanZ-2
SUN62963.1	*Streptococcus hyointestinalis*	VanZ-2
MBR1477652.1	*Lachnospiraceae bacterium*	VanZ-2
WP_106215115.1	*Glaciihabitans tibetensis*	VanZ-2
MBW2613881.1	*Deltaproteobacteria bacterium*	VanZ-2
MBQ8233847.1	*Lachnospiraceae bacterium*	VanZ-2
GJQ27214.1	*Phycisphaerae bacterium*	VanZ-2
CCX41159.1	*Clostridium sp. CAG:1024*	VanZ-2
HIX06263.1	*Candidatus Allofournierella pullicola*	VanZ-2
HAC91930.1	*Planctomycetaceae bacterium*	VanZ-2
HBB19877.1	*Ruminococcus sp.*	VanZ-2
RFS87492.1	*Actinomadura spongiicola*	VanZ-2
MCD7725116.1	*Clostridiales bacterium*	VanZ-2
MCB4208594.1	*Arthrobacter sp. UM1*	VanZ-2
MCD7893497.1	*Erysipelotrichaceae bacterium*	VanZ-2
SEI99523.1	*Lachnospiraceae bacterium A10*	VanZ-2
MBR9974217.1	*Bacteroidota bacterium*	VanZ-2
MBQ3979577.1	*Lachnospiraceae bacterium*	VanZ-2
HAE60715.1	*Eubacteriaceae bacterium*	VanZ-2
NLK99583.1	*Clostridiales bacterium*	VanZ-2
MBE1446125.1	*Paenibacillus sp. OAS669*	VanZ-2
WP_102378958.1	*Raoultibacter timonensis*	VanZ-2
WP_132151882.1		VanZ-2

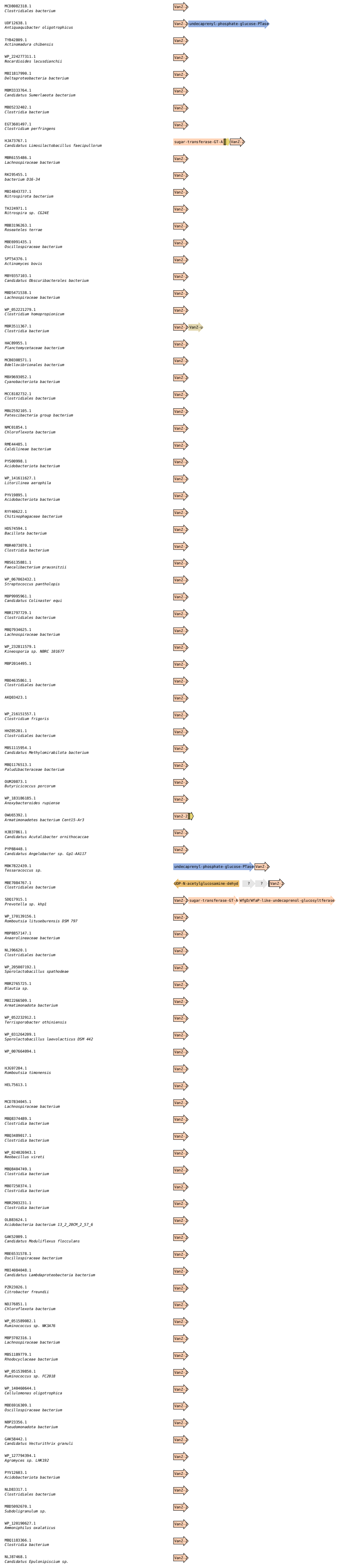

MCD8082318.1
Clostridiales bacterium
VanZ-2
UDF12638.1
Antiquaquibacter oligotrophicus
VanZ-2
undecaprenyl-phosphate-glucose-PTase
TYB42809.1
Actinomadura chibensis
VanZ-2
WP_224277311.1
Nocardioides lacusdianchii
VanZ-2
MBI1817990.1
Deltaproteobacteria bacterium
VanZ-2
MBM3333764.1
Candidatus Sumerlaeota bacterium
VanZ-2
MBO5232402.1
Clostridia bacterium
VanZ-2
EGT3601497.1
Clostridium perfringens
VanZ-2
HJA73767.1
Candidatus Limosilactobacillus faecipullorum
sugar-transferase-GT-A
VanZ-2
MBR6155486.1
Lachnospiraceae bacterium
VanZ-2
RKI95455.1
bacterium D16-34
VanZ-2
MBI4843737.1
Nitrospirota bacterium
VanZ-2
THJ24971.1
Nitrospira sp. CG24E
VanZ-2
MBB3196263.1
Roseateles terrae
VanZ-2
MBE6991435.1
Oscillospiraceae bacterium
VanZ-2
SPT54376.1
Actinomyces bovis
VanZ-2
MBY0357103.1
Candidatus Obscuribacterales bacterium
VanZ-2
MBD5471538.1
Lachnospiraceae bacterium
VanZ-2
WP_052221279.1
Clostridium homopropionicum
VanZ-2
MBR3511367.1
Clostridia bacterium
VanZ-2
VanZ-u
HAC89955.1
Planctomycetaceae bacterium
VanZ-2
MCB0308571.1
Bdellovibrionales bacterium
VanZ-2
MBX9693052.1
Cyanobacteriota bacterium
VanZ-2
MCC8182732.1
Clostridiales bacterium
VanZ-2
MBU2592105.1
Patescibacteria group bacterium
VanZ-2
NMC01854.1
Chloroflexota bacterium
VanZ-2
RME44485.1
Caldilineae bacterium
VanZ-2
PYS00998.1
Acidobacteriota bacterium
VanZ-2
WP_141611627.1
Litorilinea aerophila
VanZ-2
PYV19895.1
Acidobacteriota bacterium
VanZ-2
RYY40622.1
Chitinophagaceae bacterium
VanZ-2
HDS74594.1
Bacillota bacterium
VanZ-2
MBR4073070.1
Clostridia bacterium
VanZ-2
MBS6135881.1
Faecalibacterium prausnitzii
VanZ-2
WP_067063432.1
Streptococcus pantholopis
VanZ-2
MBP9995961.1
Candidatus Colinaster equi
VanZ-2
MBR1797729.1
Clostridiales bacterium
VanZ-2
MBQ7934625.1
Lachnospiraceae bacterium
VanZ-2
WP_232811579.1
Kineosporia sp. NBRC 101677
VanZ-2
MBP2014495.1
VanZ-2
MBO4635861.1
Clostridiales bacterium
VanZ-2
AKQ03423.1
VanZ-2
WP_216151557.1
Clostridium frigoris
VanZ-2
HHZ05201.1
Clostridiales bacterium
VanZ-2
MBS1115954.1
Candidatus Methylomirabilota bacterium
VanZ-2
MBQ1176513.1
Paludibacteraceae bacterium
VanZ-2
OUM20873.1
Butyricicoccus porcorum
VanZ-2
WP_183186185.1
Anoxybacteroides rupiense
VanZ-2
OWU65392.1
Armatimonadetes bacterium Cent15-Ar3
VanZ-2
HJB37861.1
Candidatus Acutalibacter ornithocaccae
VanZ-2
PYP88448.1
Candidatus Angelobacter sp. Gp1-AA117
VanZ-2
MBK7822439.1
Tessaracoccus sp.
undecaprenyl-phosphate-glucose-PTase
VanZ-2
MBE7084767.1
Clostridiales bacterium
UDP-N-acetylglucosamine-dehyd
?
?
VanZ-2
SDQ17915.1
Prevotella sp. khp1
VanZ-2
sugar-transferase-GT-A
WfgD/WfaP-like-undecaprenol-glucosyltferase
WP_170139156.1
Romboutsia lituseburensis DSM 797
VanZ-2
MBP8857147.1
Anaerolineaceae bacterium
VanZ-2
NLJ96620.1
Clostridiales bacterium
VanZ-2
WP_205007192.1
Sporolactobacillus spathodeae
VanZ-2
MBR2765725.1
Blautia sp.
VanZ-2
MBI2266509.1
Armatimonadota bacterium
VanZ-2
WP_052232912.1
Terrisporobacter othiniensis
VanZ-2
WP_031264209.1
Sporolactobacillus laevolacticus DSM 442
VanZ-2
WP_007664094.1
VanZ-2
HJG97204.1
Romboutsia timonensis
VanZ-2
HEL75613.1
VanZ-2
MCD7834045.1
Lachnospiraceae bacterium
VanZ-2
MBQ8374489.1
Clostridia bacterium
VanZ-2
MBQ3489017.1
Clostridia bacterium
VanZ-2
WP_024026943.1
Neobacillus vireti
VanZ-2
MBQ8404749.1
Clostridia bacterium
VanZ-2
MBO7250374.1
Clostridia bacterium
VanZ-2
MBR2903231.1
Clostridia bacterium
VanZ-2
OLB83624.1
Acidobacteria bacterium 13_2_20CM_2_57_6
VanZ-2
GAK52009.1
Candidatus Moduliflexus flocculans
VanZ-2
MBE6531578.1
Oscillospiraceae bacterium
VanZ-2
MBI4084048.1
Candidatus Lambdaproteobacteria bacterium
VanZ-2
PZR23026.1
Citrobacter freundii
VanZ-2
NDJ76851.1
Chloroflexota bacterium
VanZ-2
WP_051589082.1
Ruminococcus sp. NK3A76
VanZ-2
MBP3702316.1
Lachnospiraceae bacterium
VanZ-2
MBS1189779.1
Rhodocyclaceae bacterium
VanZ-2
WP_051539850.1
Ruminococcus sp. FC2018
VanZ-2
WP_140460644.1
Cellulomonas oligotrophica
VanZ-2
MBE6916309.1
Oscillospiraceae bacterium
VanZ-2
NBP23356.1
Pseudomonadota bacterium
VanZ-2
GAK58442.1
Candidatus Vecturithrix granuli
VanZ-2
WP_127794394.1
Agromyces sp. LHK192
VanZ-2
PYV12603.1
Acidobacteriota bacterium
VanZ-2
NLD83317.1
Clostridiales bacterium
VanZ-2
MBD5092670.1
Subdoligranulum sp.
VanZ-2
WP_120190627.1
Ammoniphilus oxalaticus
VanZ-2
MBQ1183366.1
Clostridia bacterium
VanZ-2
NLJ87468.1
Candidatus Epulonipiscium sp.
VanZ-2

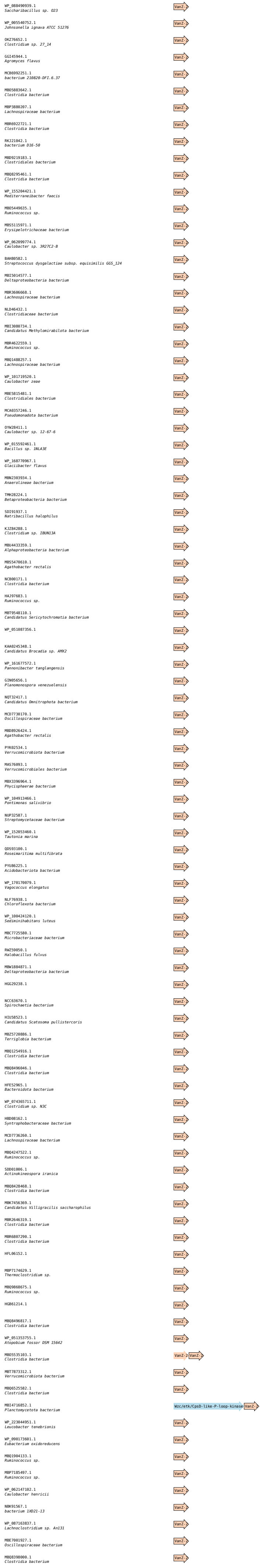
WP_088490939.1
Saccharibacillus sp. O23
VanZ-2
WP_005540752.1
Johnsonella ignava ATCC 51276
VanZ-2
OKZ76652.1
Clostridium sp. 27_14
VanZ-2
GGI45944.1
Agromyces flavus
VanZ-2
MCB6992251.1
bacterium 210820-DFI.6.37
VanZ-2
MBO5883642.1
Clostridia bacterium
VanZ-2
MBP3880207.1
Lachnospiraceae bacterium
VanZ-2
MBR6922721.1
Clostridia bacterium
VanZ-2
RKJ21042.1
bacterium D16-50
VanZ-2
MBD9219183.1
Clostridiales bacterium
VanZ-2
MBQ8295461.1
Clostridia bacterium
VanZ-2
WP_155204421.1
Mediterraneibacter faecis
VanZ-2
MBO5449635.1
Ruminococcus sp.
VanZ-2
MBS5115971.1
Erysipelotrichaceae bacterium
VanZ-2
WP_062099774.1
Caulobacter sp. 3R27C2-B
VanZ-2
BAH80582.1
Streptococcus dysgalactiae subsp. equisimilis GGS_124
VanZ-2
MBI5014577.1
Deltaproteobacteria bacterium
VanZ-2
MBR3606668.1
Lachnospiraceae bacterium
VanZ-2
NLD46432.1
Clostridiaceae bacterium
VanZ-2
MBI3080734.1
Candidatus Methylomirabilota bacterium
VanZ-2
MBR4622559.1
Ruminococcus sp.
VanZ-2
MBQ1488257.1
Lachnospiraceae bacterium
VanZ-2
WP_101719520.1
Caulobacter zeae
VanZ-2
MBE5815481.1
Clostridiales bacterium
VanZ-2
MCA0357246.1
Pseudomonadota bacterium
VanZ-2
OYW28411.1
Caulobacter sp. 12-67-6
VanZ-2
WP_015592461.1
Bacillus sp. 1NLA3E
VanZ-2
WP_168770967.1
Glaciibacter flavus
VanZ-2
MBN2303934.1
Anaerolineae bacterium
VanZ-2
TMH28224.1
Betaproteobacteria bacterium
VanZ-2
SDI91937.1
Natribacillus halophilus
VanZ-2
KJZ84288.1
Clostridium sp. IBUN13A
VanZ-2
MBU4433359.1
Alphaproteobacteria bacterium
VanZ-2
MBS5470610.1
Agathobacter rectalis
VanZ-2
NCB00171.1
Clostridia bacterium
VanZ-2
HAJ97683.1
Ruminococcus sp.
VanZ-2
MBT9548110.1
Candidatus Sericytochromatia bacterium
VanZ-2
WP_051087356.1
VanZ-2
KAA0245348.1
Candidatus Brocadia sp. AMX2
VanZ-2
WP_161677572.1
Pannonibacter tanglangensis
VanZ-2
GIN05656.1
Planomonospora venezuelensis
VanZ-2
NQT32417.1
Candidatus Omnitrophota bacterium
VanZ-2
MCD7730170.1
Oscillospiraceae bacterium
VanZ-2
MBD8926424.1
Agathobacter rectalis
VanZ-2
PYK02534.1
Verrucomicrobiota bacterium
VanZ-2
MAS76093.1
Verrucomicrobiales bacterium
VanZ-2
MBX3396964.1
Phycisphaerae bacterium
VanZ-2
WP_104913466.1
Pontimonas salivibrio
VanZ-2
NUP32587.1
Streptomycetaceae bacterium
VanZ-2
WP_152053460.1
Tautonia marina
VanZ-2
QDS93100.1
Roseimaritima multifibrata
VanZ-2
PYU86225.1
Acidobacteriota bacterium
VanZ-2
WP_170170079.1
Vagococcus elongatus
VanZ-2
NLF76938.1
Chloroflexota bacterium
VanZ-2
WP_100424120.1
Sediminihabitans luteus
VanZ-2
MBC7725580.1
Microbacteriaceae bacterium
VanZ-2
RWZ59050.1
Halobacillus fulvus
VanZ-2
MBW1884871.1
Deltaproteobacteria bacterium
VanZ-2
HGG29238.1
VanZ-2
NCC63670.1
Spirochaetia bacterium
VanZ-2
HIU58523.1
Candidatus Scatosoma pullistercoris
VanZ-2
MBZ5720886.1
Terriglobia bacterium
VanZ-2
MBQ1254916.1
Clostridia bacterium
VanZ-2
MBQ8496046.1
Clostridia bacterium
VanZ-2
HFE52965.1
Bacteroidota bacterium
VanZ-2
WP_074365711.1
Clostridium sp. N3C
VanZ-2
HBD08162.1
Syntrophobacteraceae bacterium
VanZ-2
MCD7736260.1
Lachnospiraceae bacterium
VanZ-2
MBQ4247522.1
Ruminococcus sp.
VanZ-2
SDD01006.1
Actinokineospora iranica
VanZ-2
MBQ8428468.1
Clostridia bacterium
VanZ-2
MBK7456369.1
Candidatus Villigracilis saccharophilus
VanZ-2
MBR2646319.1
Clostridia bacterium
VanZ-2
MBR6807290.1
Clostridia bacterium
VanZ-2
HFL06152.1
VanZ-2
MBP7174629.1
Thermoclostridium sp.
VanZ-2
MBQ9868675.1
Ruminococcus sp.
VanZ-2
HGB61214.1
VanZ-2
MBQ8496817.1
Clostridia bacterium
VanZ-2
WP_051353755.1
Atopobium fossor DSM 15642
VanZ-2
MBO5535103.1
Clostridia bacterium
VanZ-2
VanZ-2
MBT7873312.1
Verrucomicrobiota bacterium
VanZ-2
MBQ6525582.1
Clostridia bacterium
VanZ-2
MBI4716852.1
Planctomycetota bacterium
Wzc/etk/CpsD-like-P-loop-kinase
VanZ-2
WP_223044951.1
Leucobacter tenebrionis
VanZ-2
WP_090173601.1
Eubacterium oxidoreducens
VanZ-2
MBQ1904133.1
Ruminococcus sp.
VanZ-2
MBP7185497.1
Ruminococcus sp.
VanZ-2
WP_062147102.1
Caulobacter henricii
VanZ-2
NBK91567.1
bacterium 1XD21-13
VanZ-2
WP_087163837.1
Lachnoclostridium sp. An131
VanZ-2
MBE7001927.1
Oscillospiraceae bacterium
VanZ-2
MBQ8398000.1
Clostridia bacterium
VanZ-2

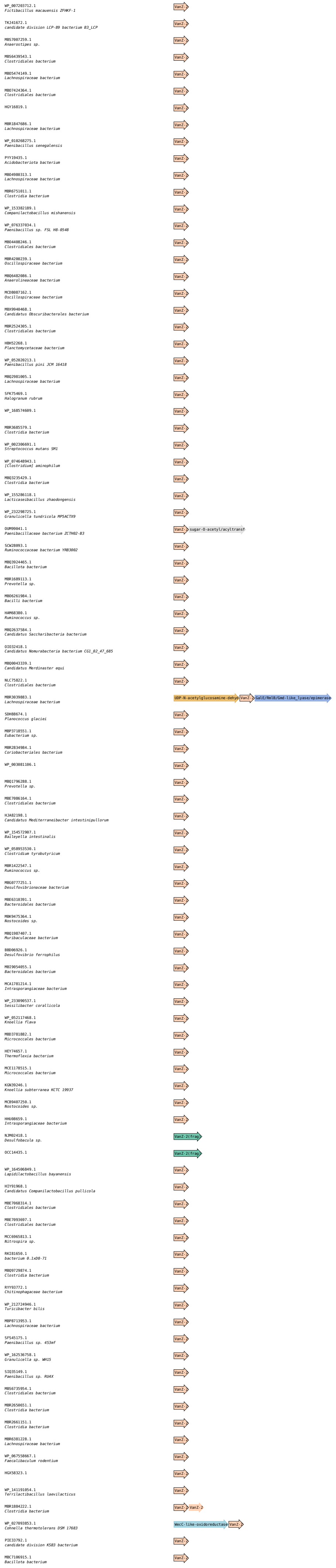

WP_007203712.1
Fictibacillus macauensis ZFHKF-1
VanZ-2
TKJ41672.1
candidate division LCP-89 bacterium B3_LCP
VanZ-2
MBS7007259.1
Anaerostipes sp.
VanZ-2
MBS6439543.1
Clostridiales bacterium
VanZ-2
MBO5474149.1
Lachnospiraceae bacterium
VanZ-2
MBO7424364.1
Clostridiales bacterium
VanZ-2
HGY16819.1
VanZ-2
MBR1847686.1
Lachnospiraceae bacterium
VanZ-2
WP_010268275.1
Paenibacillus senegalensis
VanZ-2
PYY19435.1
Acidobacteriota bacterium
VanZ-2
MBO4980313.1
Lachnospiraceae bacterium
VanZ-2
MBR6751011.1
Clostridia bacterium
VanZ-2
WP_153382189.1
Companilactobacillus mishanensis
VanZ-2
WP_076337034.1
Paenibacillus sp. FSL H8-0548
VanZ-2
MBO4408246.1
Clostridiales bacterium
VanZ-2
MBR4200239.1
Oscillospiraceae bacterium
VanZ-2
MBQ6482086.1
Anaerolineaceae bacterium
VanZ-2
MCD8087162.1
Oscillospiraceae bacterium
VanZ-2
MBX9940468.1
Candidatus Obscuribacterales bacterium
VanZ-2
MBR2524305.1
Clostridiales bacterium
VanZ-2
HBH52268.1
Planctomycetaceae bacterium
VanZ-2
WP_052020213.1
Paenibacillus pini JCM 16418
VanZ-2
MBQ2981005.1
Lachnospiraceae bacterium
VanZ-2
SFK75469.1
Halogranum rubrum
VanZ-2
WP_168574609.1
VanZ-2
MBR3685579.1
Clostridia bacterium
VanZ-2
WP_002306691.1
Streptococcus mutans SM1
VanZ-2
WP_074648943.1
[Clostridium] aminophilum
VanZ-2
MBQ3235429.1
Clostridia bacterium
VanZ-2
WP_155286118.1
Lacticaseibacillus zhaodongensis
VanZ-2
WP_232298725.1
Granulicella tundricola MP5ACTX9
VanZ-2
OUM99041.1
Paenibacillaceae bacterium ZCTH02-B3
VanZ-2
sugar-O-acetyl/acyltransf
SCW28093.1
Ruminococcaceae bacterium YRB3002
VanZ-2
MBQ3924465.1
Bacillota bacterium
VanZ-2
MBR1689113.1
Prevotella sp.
VanZ-2
MBO6261984.1
Bacilli bacterium
VanZ-2
HAM68380.1
Ruminococcus sp.
VanZ-2
MBQ2637584.1
Candidatus Saccharibacteria bacterium
VanZ-2
OIO32418.1
Candidatus Nomurabacteria bacterium CG1_02_47_685
VanZ-2
MBQ0043339.1
Candidatus Merdinaster equi
VanZ-2
NLC75822.1
Clostridiales bacterium
VanZ-2
MBR3039883.1
Lachnospiraceae bacterium
UDP-N-acetylglucosamine-dehyd
VanZ-2
GalE/RmlB/Gmd-like_lyase/epimerase
SDH88674.1
Planococcus glaciei
VanZ-2
MBP3718551.1
Eubacterium sp.
VanZ-2
MBR2834984.1
Coriobacteriales bacterium
VanZ-2
WP_003081106.1
VanZ-2
MBQ1796288.1
Prevotella sp.
VanZ-2
MBE7086164.1
Clostridiales bacterium
VanZ-2
HJA82198.1
Candidatus Mediterraneibacter intestinipullorum
VanZ-2
WP_154572907.1
Baileyella intestinalis
VanZ-2
WP_058953530.1
Clostridium tyrobutyricum
VanZ-2
MBR1422547.1
Ruminococcus sp.
VanZ-2
MBG0777251.1
Desulfovibrionaceae bacterium
VanZ-2
MBE6310391.1
Bacteroidales bacterium
VanZ-2
MBK9475364.1
Nostocoides sp.
VanZ-2
MBQ1987407.1
Muribaculaceae bacterium
VanZ-2
BBD06926.1
Desulfovibrio ferrophilus
VanZ-2
MBI9054055.1
Bacteroidales bacterium
VanZ-2
MCA1781214.1
Intrasporangiaceae bacterium
VanZ-2
WP_233090537.1
Sessilibacter corallicola
VanZ-2
WP_052117468.1
Knoellia flava
VanZ-2
MBD3781882.1
Micrococcales bacterium
VanZ-2
HEY74657.1
Thermoflexia bacterium
VanZ-2
MCE1178515.1
Micrococcales bacterium
VanZ-2
KGN39246.1
Knoellia subterranea KCTC 19937
VanZ-2
MCB9407250.1
Nostocoides sp.
VanZ-2
HHU08659.1
Intrasporangiaceae bacterium
VanZ-2
NJM02418.1
Desulfobacula sp.
VanZ-2(frag)
OCC14435.1
VanZ-2(frag)
WP_164506849.1
Lapidilactobacillus bayanensis
VanZ-2
HIY91968.1
Candidatus Companilactobacillus pullicola
VanZ-2
MBE7068314.1
Clostridiales bacterium
VanZ-2
MBE7093697.1
Clostridiales bacterium
VanZ-2
MCC6965813.1
Nitrospira sp.
VanZ-2
RKI81650.1
bacterium 0.1xD8-71
VanZ-2
MBQ9729874.1
Clostridia bacterium
VanZ-2
RYY93772.1
Chitinophagaceae bacterium
VanZ-2
WP_212724946.1
Turicibacter bilis
VanZ-2
MBP8713953.1
Lachnospiraceae bacterium
VanZ-2
SFS45175.1
Paenibacillus sp. 453mf
VanZ-2
WP_162536758.1
Granulicella sp. WH15
VanZ-2
SIQ35149.1
Paenibacillus sp. RU4X
VanZ-2
MBS6735954.1
Clostridiales bacterium
VanZ-2
MBR2650651.1
Clostridia bacterium
VanZ-2
MBR2661151.1
Clostridia bacterium
VanZ-2
MBR6381228.1
Lachnospiraceae bacterium
VanZ-2
WP_067558667.1
Faecalibaculum rodentium
VanZ-2
HGX58323.1
VanZ-2
WP_141191054.1
Terrilactibacillus laevilacticus
VanZ-2
MBR1884222.1
Clostridia bacterium
VanZ-2
VanZ-2
WP_027093853.1
Cohnella thermotolerans DSM 17683
WecC-like-oxidoreductase
VanZ-2
PIE33792.1
candidate division KSB3 bacterium
VanZ-2
MBC7106915.1
Bacillota bacterium
VanZ-2

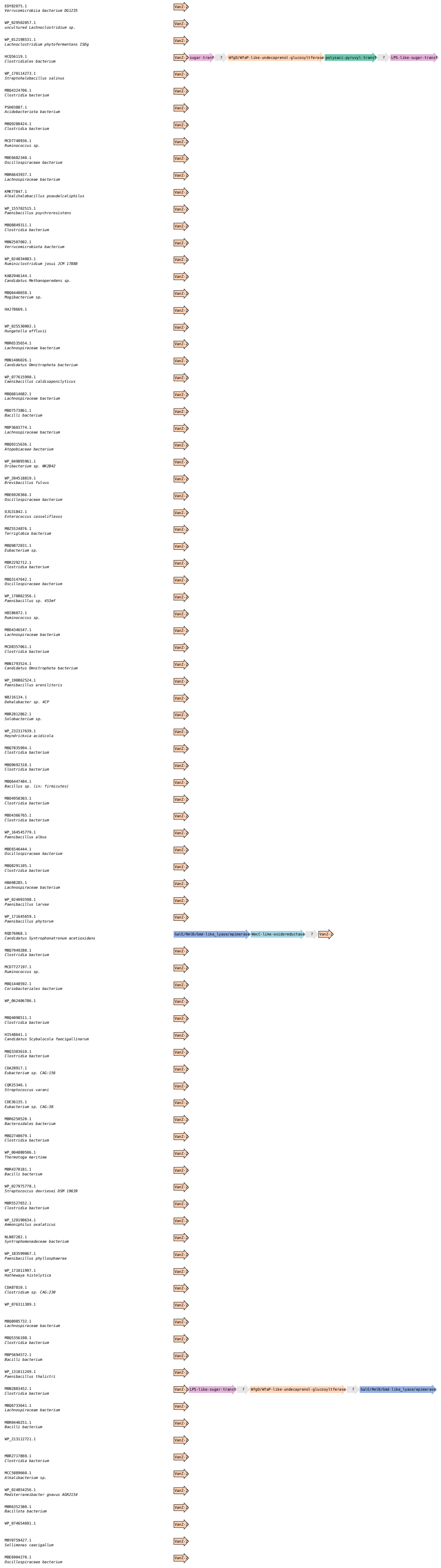
EDY82075.1
Verrucomicrobiia bacterium DG1235
VanZ-2
WP_029502057.1
uncultured Lachnoclostridium sp.
VanZ-2
WP_012198531.1
Lachnoclostridium phytofermentans ISDg
VanZ-2
HCQ56119.1
Clostridiales bacterium
VanZ-2
sugar-tranf
?
WfgD/WfaP-like-undecaprenol-glucosyltferase
polysacc-pyruvyl-transf
?
LPS-like-sugar-transf
WP_170114273.1
Streptohalobacillus salinus
VanZ-2
MBQ4324706.1
Clostridia bacterium
VanZ-2
PSH03887.1
Acidobacteriota bacterium
VanZ-2
MBQ9280424.1
Clostridia bacterium
VanZ-2
MCD7740936.1
Ruminococcus sp.
VanZ-2
MBE6682340.1
Oscillospiraceae bacterium
VanZ-2
MBR6643937.1
Lachnospiraceae bacterium
VanZ-2
KMK77847.1
Alkalihalobacillus pseudalcaliphilus
VanZ-2
WP_155702515.1
Paenibacillus psychroresistens
VanZ-2
MBQ8849311.1
Clostridia bacterium
VanZ-2
MBN2507002.1
Verrucomicrobiota bacterium
VanZ-2
WP_024834083.1
Ruminiclostridium josui JCM 17888
VanZ-2
KAB2946144.1
Candidatus Methanoperedens sp.
VanZ-2
MBQ6440658.1
Mogibacterium sp.
VanZ-2
HAJ78669.1
VanZ-2
WP_025530002.1
Hungatella effluvii
VanZ-2
MBR6535654.1
Lachnospiraceae bacterium
VanZ-2
MBN1406026.1
Candidatus Omnitrophota bacterium
VanZ-2
WP_077615990.1
Caenibacillus caldisaponilyticus
VanZ-2
MBQ6814682.1
Lachnospiraceae bacterium
VanZ-2
MBO7573861.1
Bacilli bacterium
VanZ-2
MBP3603774.1
Lachnospiraceae bacterium
VanZ-2
MBQ9315636.1
Atopobiaceae bacterium
VanZ-2
WP_049895961.1
Oribacterium sp. NK2B42
VanZ-2
WP_204518819.1
Brevibacillus fulvus
VanZ-2
MBE6920366.1
Oscillospiraceae bacterium
VanZ-2
OJG31842.1
Enterococcus casseliflavus
VanZ-2
MBZ5524876.1
Terriglobia bacterium
VanZ-2
MBQ9872031.1
Eubacterium sp.
VanZ-2
MBR2292712.1
Clostridia bacterium
VanZ-2
MBQ3147642.1
Oscillospiraceae bacterium
VanZ-2
WP_170862356.1
Paenibacillus sp. 453mf
VanZ-2
HBI86072.1
Ruminococcus sp.
VanZ-2
MBO4346547.1
Lachnospiraceae bacterium
VanZ-2
MCD8357061.1
Clostridia bacterium
VanZ-2
MBN1793524.1
Candidatus Omnitrophota bacterium
VanZ-2
WP_190862524.1
Paenibacillus arenilitoris
VanZ-2
NBJ16134.1
Dehalobacter sp. 4CP
VanZ-2
MBR2812862.1
Solobacterium sp.
VanZ-2
WP_232317639.1
Heyndrickxia acidicola
VanZ-2
MBQ7835994.1
Clostridia bacterium
VanZ-2
MBQ9692318.1
Clostridia bacterium
VanZ-2
MBQ6447484.1
Bacillus sp. (in: firmicutes)
VanZ-2
MBO4950303.1
Clostridia bacterium
VanZ-2
MBO4366765.1
Clostridia bacterium
VanZ-2
WP_164545779.1
Paenibacillus albus
VanZ-2
MBE6546444.1
Oscillospiraceae bacterium
VanZ-2
MBQ8291105.1
Clostridia bacterium
VanZ-2
HBA98285.1
Lachnospiraceae bacterium
VanZ-2
WP_024093598.1
Paenibacillus larvae
VanZ-2
WP_171645659.1
Paenibacillus phytorum
VanZ-2
RQD76068.1
Candidatus Syntrophonatronum acetioxidans
GalE/RmlB/Gmd-like_lyase/epimerase
WecC-like-oxidoreductase
?
VanZ-2
MBQ7949280.1
Clostridia bacterium
VanZ-2
MCD7727197.1
Ruminococcus sp.
VanZ-2
MBQ1448592.1
Coriobacteriales bacterium
VanZ-2
WP_062406786.1
VanZ-2
MBQ4098511.1
Clostridia bacterium
VanZ-2
HIS48641.1
Candidatus Scybalocola faecigallinarum
VanZ-2
MBQ3303610.1
Clostridia bacterium
VanZ-2
CDA28917.1
Eubacterium sp. CAG:156
VanZ-2
CQR25346.1
Streptococcus varani
VanZ-2
CDE36135.1
Eubacterium sp. CAG:38
VanZ-2
MBR6250520.1
Bacteroidales bacterium
VanZ-2
MBQ2740679.1
Clostridia bacterium
VanZ-2
WP_004080506.1
Thermotoga maritima
VanZ-2
MBR4378181.1
Bacilli bacterium
VanZ-2
WP_027975778.1
Streptococcus devriesei DSM 19639
VanZ-2
MBR5527652.1
Clostridia bacterium
VanZ-2
WP_120190634.1
Ammoniphilus oxalaticus
VanZ-2
NLN87282.1
Syntrophomonadaceae bacterium
VanZ-2
WP_183599067.1
Paenibacillus phyllosphaerae
VanZ-2
WP_171011997.1
Hathewaya histolytica
VanZ-2
CDA87810.1
Clostridium sp. CAG:230
VanZ-2
WP_076311389.1
VanZ-2
MBQ8985732.1
Lachnospiraceae bacterium
VanZ-2
MBQ5356198.1
Clostridia bacterium
VanZ-2
MBP5694572.1
Bacilli bacterium
VanZ-2
WP_131011249.1
Paenibacillus thalictri
VanZ-2
MBN2883452.1
Clostridia bacterium
VanZ-2
LPS-like-sugar-transf
?
WfgD/WfaP-like-undecaprenol-glucosyltferase
?
GalE/RmlB/Gmd-like_lyase/epimerase
MBQ6733641.1
Lachnospiraceae bacterium
VanZ-2
MBR0440251.1
Bacilli bacterium
VanZ-2
WP_213112721.1
VanZ-2
MBR2717869.1
Clostridia bacterium
VanZ-2
MCC5889660.1
Alkalibacterium sp.
VanZ-2
WP_024854256.1
Mediterraneibacter gnavus AGR2154
VanZ-2
MBR6352380.1
Bacillota bacterium
VanZ-2
WP_074654691.1
VanZ-2
MBY0759427.1
Sellimonas caecigallum
VanZ-2
MBE6904270.1
Oscillospiraceae bacterium
VanZ-2

MBP3568746.1	*Lachnospiraceae bacterium*	VanZ-2
HAM16791.1	*Eggerthellaceae bacterium*	VanZ-2
MBQ3027830.1	*Lachnospiraceae bacterium*	VanZ-2
HAZ91478.1	*Eubacterium sp.*	VanZ-2
MBQ8072742.1	*Clostridia bacterium*	VanZ-2
WP_213619968.1	*Paenibacillus sp. J22TS3*	VanZ-2
MBQ8726351.1	*Clostridia bacterium*	VanZ-2
WP_169028123.1	*Paenibacillus aquistagni*	VanZ-2
MBQ9795144.1	*Clostridia bacterium*	VanZ-2
WP_141432886.1	*Bacillus sp. 03113*	VanZ-2
MBQ9084470.1	*Clostridia bacterium*	VanZ-2
MBQ8406759.1	*Clostridia bacterium*	VanZ-2
MBQ9086106.1	*Clostridia bacterium*	VanZ-2
MBE5750841.1	*Clostridiales bacterium*	VanZ-2
MBQ9115948.1	*Clostridia bacterium*	VanZ-2
MBQ8288962.1	*Clostridia bacterium*	VanZ-2
HIR93994.1	*Candidatus Egerieimonas intestinavium*	VanZ-2
MBI4166413.1	*Acidobacteriota bacterium*	VanZ-2
MBP6590832.1	*Chitinophagaceae bacterium*	VanZ-2
WP_011886787.1	*Geobacillus thermodenitrificans*	VanZ-2
MBQ8140724.1	*Clostridia bacterium*	VanZ-2
MBQ9783139.1	*Clostridia bacterium*	VanZ-2
KJS17968.1	*Peptococcaceae bacterium BRH_c4b*	VanZ-2
WP_090975351.1	*Paenibacillus sp. CF384*	VanZ-2
MBQ7879648.1	*Clostridia bacterium*	VanZ-2
WP_102709225.1	*Paenibacillus castaneae*	VanZ-2
MBR3234325.1	*Atopobiaceae bacterium*	VanZ-2
MBS1575652.1	*Bacteroidota bacterium*	VanZ-2
MBN2031477.1	*bacterium*	VanZ-2
WP_185132452.1	*Cohnella zeiphila*	VanZ-2
WP_067980625.1	*Aerococcus urinaehominis*	VanZ-2
MBO6015273.1	*Lachnospiraceae bacterium*	VanZ-2
MBQ9927625.1	*Lachnospiraceae bacterium*	VanZ-2
MBE6917663.1	*Oscillospiraceae bacterium*	VanZ-2
MBR2283128.1	*Ruminococcus sp.*	VanZ-2
AKB29748.1	*Methanosarcina siciliae T4/M*	VanZ-2
MBO5337804.1	*Lachnospiraceae bacterium*	VanZ-2
WP_144355040.1	*Lactobacillus sp. LL6*	VanZ-2
WP_051233699.1	*Butyrivibrio sp. NC3005*	VanZ-2
MBO5056467.1	*Lachnospiraceae bacterium*	VanZ-2
NLV33855.1	*Clostridiaceae bacterium*	VanZ-2
KRK45132.1	*Dellaglioa algida DSM 15638*	VanZ-2
WP_214480811.1	*Bacillus sp. SM2101*	VanZ-2
WP_058920100.1	*Carnobacterium antarcticum*	VanZ-2
MBR5798483.1	*Lachnospiraceae bacterium*	VanZ-2
MBQ2473367.1	*Lachnospira sp.*	VanZ-2
MBR4183906.1	*Lachnospiraceae bacterium*	VanZ-2
MBR6222534.1	*Lachnospiraceae bacterium*	VanZ-2
MBQ5930429.1	*Clostridia bacterium*	VanZ-2
MBR2321591.1	*Clostridia bacterium*	VanZ-2
MBR6642474.1	*Lachnospiraceae bacterium*	VanZ-2
MBR2023380.1	*Clostridia bacterium*	VanZ-2
MBQ3824342.1	*Clostridia bacterium*	VanZ-2
MBE6599577.1	*Oscillospiraceae bacterium*	VanZ-2
WP_232698709.1	*Brevibacillus daliensis*	VanZ-2
MBQ3003481.1	*Clostridia bacterium*	VanZ-2
WP_015944805.1	*Desulfitobacterium hafniense DCB-2*	VanZ-2
WP_025087636.1	*Ligilactobacillus apodemi*	VanZ-2
WP_052008441.1	*Listeria aquatica*	VanZ-2
MBS5653181.1	*Eubacterium sp.*	VanZ-2
MBR6680286.1	*Clostridia bacterium*	VanZ-2
WP_223701263.1	*Sutcliffiella deserti*	VanZ-2
WP_156806456.1	*Streptococcus sp. S784/96/1*	VanZ-2
MBO5910607.1	*Elusimicrobiaceae bacterium*	WecC-like-oxidoreductase VanZ-2
MBQ3424688.1	*Clostridia bacterium*	VanZ-2
MBW1896884.1	*Deltaproteobacteria bacterium*	VanZ-2
WP_000919801.1	*Streptococcus agalactiae CCUG 37742*	VanZ-2
WP_028562335.1	*Paenibacillus pinihumi DSM 23905 = JCM 16419*	VanZ-2
WP_153724121.1	*Heliorestis convoluta*	VanZ-2
WP_216114476.1	*Clostridium algidicarnis*	VanZ-2
MBE6549024.1	*Oscillospiraceae bacterium*	VanZ-2
WP_184245319.1	*Gracilibacillus halotolerans*	VanZ-2
BBO92366.1	*Desulfosarcina ovata subsp. ovata*	VanZ-2
MSP91636.1	*Myxococcales bacterium*	VanZ-2
KKG56856.1	*Methanosarcina mazei*	VanZ-2
HII79465.1	*Methanosarcina sp.*	VanZ-2
AKB17622.1	*Methanosarcina sp. WWM596*	VanZ-2
WP_078579069.1	*Salipaludibacillus agaradhaerens*	VanZ-2
WP_183774930.1	*Catenibacillus scindens*	VanZ-2
WP_051671344.1	*Oribacterium sp. P6A1*	VanZ-2
WP_078036091.1	*Oribacterium sp. C9*	VanZ-2
MBC7527746.1	*Chthonomonadaceae bacterium*	VanZ-2
MBE6986442.1	*Oscillospiraceae bacterium*	VanZ-2
MBS6849214.1	*Ruminococcus sp.*	VanZ-2
MBQ8187829.1	*Clostridia bacterium*	VanZ-2
MBO4878620.1	*Ruminococcus sp.*	VanZ-2
WP_098517566.1	*Bacillus cereus*	VanZ-2
MBR1724186.1	*Ruminococcus sp.*	VanZ-2
HIT51153.1	*Candidatus Aveggerthella excrementigallinarum*	VanZ-2
NLZ15713.1	*Erysipelotrichaceae bacterium*	VanZ-2
WP_204412681.1	*Sutcliffiella tianshenii*	VanZ-2
MBQ8349127.1	*Ruminococcus sp.*	VanZ-2
MBE5928806.1	*Lachnospiraceae bacterium*	VanZ-2

HHU13182.1	*Clostridiaceae bacterium*	VanZ-2
HHT95567.1	*Clostridiaceae bacterium*	VanZ-2
MBE6862408.1	*Ruminococcus sp.*	VanZ-2
WP_078555925.1	*Alkalihalobacterium alkalicellulosilyticum*	VanZ-2
MBP8989845.1	*Clostridia bacterium*	VanZ-2
MBO7298130.1	*Clostridia bacterium*	VanZ-2
HIT18514.1	*Candidatus Fimivivens faecavium*	VanZ-2
OYT54012.1	*Candidatus Altiarchaeales archaeon ex4484_2*	VanZ-2
MBO5745212.1	*Clostridia bacterium*	VanZ-2
ETA74013.1	*Ligilactobacillus equi DPC 6820*	VanZ-2
MBR6966209.1	*Clostridia bacterium*	VanZ-2
OOO00011.1	*Epulopiscium sp. Nele67-Bin004*	VanZ-2
CDE81788.1	*Ruminococcus sp. CAG:353*	VanZ-2
CCZ82885.1	*Ruminococcus sp. CAG:254*	VanZ-2
MBR4576099.1	*Clostridia bacterium*	VanZ-2
MBR4234850.1	*Clostridia bacterium*	VanZ-2
MBQ5978057.1	*Oscillospiraceae bacterium*	VanZ-2
MBQ7131518.1	*Oscillospiraceae bacterium*	VanZ-2
WP_079439908.1	*Clostridium chromiireducens*	VanZ-2
WP_008087898.1	*Streptococcus ictaluri 707-05*	VanZ-2
MBR6626970.1	*Lachnospiraceae bacterium*	VanZ-2
PIR23944.1	*Deltaproteobacteria bacterium CG11_big_fil_rev_8_21_14_0_20_45_16*	VanZ-2
MBQ3409215.1	*Clostridia bacterium*	VanZ-2
WP_116877901.1	*Streptococcus chenjunshii*	VanZ-2
HCO28369.1	*Lachnospiraceae bacterium*	VanZ-2
WP_026827411.1	*Exiguobacterium sibiricum 7-3*	VanZ-2
MBR3035843.1	*Lachnospiraceae bacterium*	rmlD/rfbD-like-reductase VanZ-2
GFI16652.1	*Lachnospiraceae bacterium*	VanZ-2
NLN86703.1	*Syntrophomonadaceae bacterium*	VanZ-2
WP_216288534.1	*Clostridium psychrophilum*	VanZ-2
NLV20841.1	*Syntrophomonadaceae bacterium*	VanZ-2
TDR55538.1	*Listeria rocourtiae*	VanZ-2
MBR5997321.1	*Bacilli bacterium*	VanZ-2
GFI48330.1	*Lachnospiraceae bacterium*	VanZ-2
WP_168261247.1	*uncultured Streptococcus sp.*	VanZ-2
WP_205165927.1	*Alteribacter salitolerans*	VanZ-2
MBQ2896925.1	*Clostridia bacterium*	VanZ-2
WP_117642549.1	*uncultured Ligilactobacillus sp.*	VanZ-2
MBD5501054.1	*Lachnospiraceae bacterium*	VanZ-2
QCQ03586.1	*Ligilactobacillus animalis*	VanZ-2
MBR2460972.1	*Clostridia bacterium*	VanZ-2
HIX77797.1	*Candidatus Fusicatenibacter merdavium*	VanZ-2
MBR2085741.1	*Oscillospiraceae bacterium*	VanZ-2
MBO5938244.1	*Clostridia bacterium*	VanZ-2
WP_075670281.1	*Anaerostipes sp. 992a*	VanZ-2
MBQ0009026.1	*Candidatus Fiminaster equi*	VanZ-2
MBP1760781.1	*Bacillota bacterium*	VanZ-2
SNT92945.1	*Aristaeella hokkaidonensis*	VanZ-2
TMI89360.1	*Bacteroidota bacterium*	VanZ-2
WP_074714320.1	*Ruminococcus flavefaciens*	VanZ-2
MBE5851776.1	*Lachnospiraceae bacterium*	VanZ-2
WP_162303789.1	*Paracnuella aquatica*	WfgD/WfaP-like-undecaprenol-glucosyltferase VanZ-2
NLB45408.1	*Clostridiaceae bacterium*	VanZ-2
MBP5419959.1	*Bacteroidales bacterium*	VanZ-2
NLW12348.1	*Clostridiaceae bacterium*	VanZ-2
WP_204721393.1	*Hydrogenoanaerobacterium saccharovorans*	VanZ-2
MBZ5630119.1	*Terriglobia bacterium*	VanZ-2
WP_051217568.1	*Paenibacillus assamensis DSM 18201*	VanZ-2
MBS7341084.1	*Lachnospiraceae bacterium*	VanZ-2
WP_156284465.1	*Paenibacillus sp. NEAU-GSW1*	VanZ-2
MBQ8759169.1	*Clostridia bacterium*	VanZ-2
WP_224724646.1	*Paenibacillus vietnamensis*	VanZ-2
MBQ7486108.1	*Clostridia bacterium*	VanZ-2 VanZ-2
MBQ7374101.1	*Clostridia bacterium*	VanZ-2
MBQ9659585.1	*Clostridia bacterium*	VanZ-2
MBE5741722.1	*Clostridiales bacterium*	VanZ-2
NTW71242.1	*Eubacteriaceae bacterium*	VanZ-2
NMB53825.1	*Leptolinea sp.*	VanZ-2
MBP1585980.1	*Lachnospiraceae bacterium*	VanZ-2
RYY18037.1	*Chitinophagaceae bacterium*	VanZ-2
MXW34552.1	*Chloroflexota bacterium*	VanZ-2
MBW4838301.1	*Paenibacillaceae bacterium*	VanZ-2
MBM4064911.1	*Planctomycetota bacterium*	VanZ-2
MCC2230009.1	*Hominifimenecus microfluidus*	VanZ-2
MCC2230651.1	*Hominifimenecus microfluidus*	VanZ-2
PYX75389.1	*Acidobacteriota bacterium*	VanZ-2
WP_090108725.1	*Cohnella sp. OV330*	VanZ-2
MBE5807137.1	*Clostridiales bacterium*	VanZ-2
MBL7741095.1	*Chitinophagaceae bacterium*	VanZ-2
MBL7764165.1	*Chitinophagaceae bacterium*	VanZ-2
WP_099445978.1	*Caulobacter sp. B11*	VanZ-2
WP_184405433.1	*Geomicrobium halophilum*	VanZ-2
WP_204505207.1	*Phocaeicola plebeius*	VanZ-2
MBT5266185.1	*Rhodospirillaceae bacterium*	VanZ-2
WP_156606941.1	*Auraticoccus cholistanensis*	VanZ-2
HBP39137.1	*Clostridiales bacterium*	VanZ-2
MBP3479688.1	*Oscillospiraceae bacterium*	VanZ-2
MBQ7917497.1	*Clostridia bacterium*	VanZ-2
RZT21527.1	*Fictibacillus sp. BK138*	VanZ-2
PLS03333.1	*Neobacillus cucumis*	VanZ-2
RKJ48035.1	*bacterium 1XD42-54*	VanZ-2
MBS5398453.1	*Lachnospiraceae bacterium*	VanZ-2
NOX54760.1	*Planctomycetota bacterium*	VanZ-2

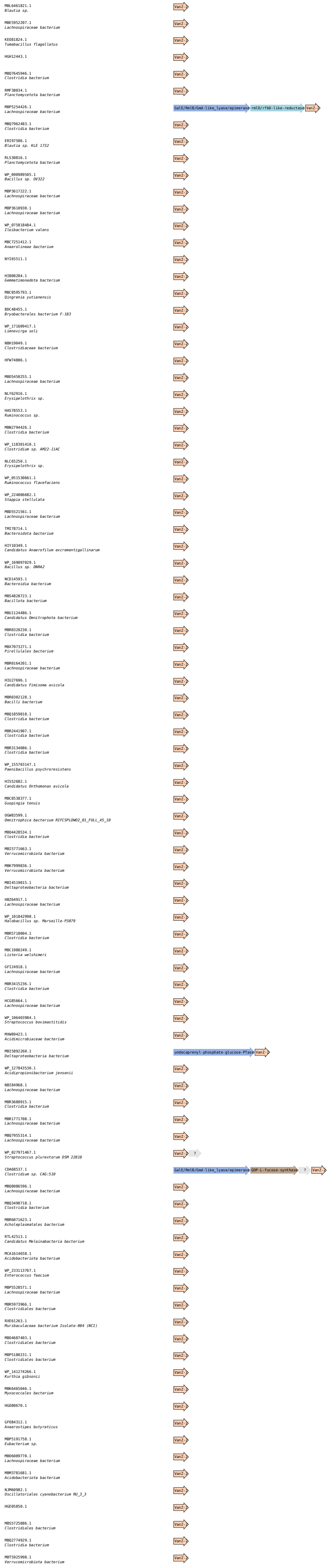
MBL6461821.1
Blautia sp.
VanZ-2
MBE5952207.1
Lachnospiraceae bacterium
VanZ-2
KEO81824.1
Tumebacillus flagellatus
VanZ-2
HGH12443.1
VanZ-2
MBQ7645946.1
Clostridia bacterium
VanZ-2
RMF38034.1
Planctomycetota bacterium
VanZ-2
MBP5254426.1
Lachnospiraceae bacterium
GalE/RmlB/Gmd-like_lyase/epimerase
rmlD/rfbD-like-reductase
VanZ-2
MBQ7962483.1
Clostridia bacterium
VanZ-2
ERI97586.1
Blautia sp. KLE 1732
VanZ-2
RLS30816.1
Planctomycetota bacterium
VanZ-2
WP_090989505.1
Bacillus sp. OV322
VanZ-2
MBP3617222.1
Lachnospiraceae bacterium
VanZ-2
MBP3610930.1
Lachnospiraceae bacterium
VanZ-2
WP_075818484.1
Ileibacterium valens
VanZ-2
MBC7251412.1
Anaerolineae bacterium
VanZ-2
NYI65511.1
VanZ-2
HIB00204.1
Gemmatimonadota bacterium
VanZ-2
MBC8595793.1
Qingrenia yutianensis
VanZ-2
BDC48455.1
Bryobacterales bacterium F-183
VanZ-2
WP_171609417.1
Limnovirga soli
VanZ-2
NBH19049.1
Clostridiaceae bacterium
VanZ-2
HFW74806.1
VanZ-2
MBO5450255.1
Lachnospiraceae bacterium
VanZ-2
NLY62916.1
Erysipelothrix sp.
VanZ-2
HAS78553.1
Ruminococcus sp.
VanZ-2
MBN2794426.1
Clostridia bacterium
VanZ-2
WP_118301410.1
Clostridium sp. AM22-11AC
VanZ-2
NLC65250.1
Erysipelothrix sp.
VanZ-2
WP_051530661.1
Ruminococcus flavefaciens
VanZ-2
WP_224806682.1
Stappia stellulata
VanZ-2
MBD5521561.1
Lachnospiraceae bacterium
VanZ-2
TMI78714.1
Bacteroidota bacterium
VanZ-2
HIY10349.1
Candidatus Anaerofilum excrementigallinarum
VanZ-2
WP_169097029.1
Bacillus sp. DNRA2
VanZ-2
NCD14593.1
Bacteroidia bacterium
VanZ-2
MBS4828723.1
Bacillota bacterium
VanZ-2
MBU1124486.1
Candidatus Omnitrophota bacterium
VanZ-2
MBR0320230.1
Clostridia bacterium
VanZ-2
MBX7073271.1
Pirellulales bacterium
VanZ-2
MBR0164201.1
Lachnospiraceae bacterium
VanZ-2
HIU27696.1
Candidatus Fimisoma avicola
VanZ-2
MBR0302128.1
Bacilli bacterium
VanZ-2
MBQ1859818.1
Clostridia bacterium
VanZ-2
MBR2441907.1
Clostridia bacterium
VanZ-2
MBR3134086.1
Clostridia bacterium
VanZ-2
WP_155703147.1
Paenibacillus psychroresistens
VanZ-2
HIS52682.1
Candidatus Onthomonas avicola
VanZ-2
MBC8538377.1
Guopingia tenuis
VanZ-2
OGW83599.1
Omnitrophica bacterium RIFCSPLOWO2_01_FULL_45_10
VanZ-2
MBQ4428534.1
Clostridia bacterium
VanZ-2
MBI5771663.1
Verrucomicrobiota bacterium
VanZ-2
MBK7999836.1
Verrucomicrobiota bacterium
VanZ-2
MBI4519015.1
Deltaproteobacteria bacterium
VanZ-2
HBZ64917.1
Lachnospiraceae bacterium
VanZ-2
WP_101842998.1
Halobacillus sp. Marseille-P3879
VanZ-2
MBR5718004.1
Clostridia bacterium
VanZ-2
MBC1980249.1
Listeria welshimeri
VanZ-2
GFI24918.1
Lachnospiraceae bacterium
VanZ-2
MBR3415236.1
Clostridia bacterium
VanZ-2
HCG85664.1
Lachnospiraceae bacterium
VanZ-2
WP_106403984.1
Streptococcus bovimastitidis
VanZ-2
MXW89423.1
Acidimicrobiaceae bacterium
VanZ-2
MBI5892260.1
Deltaproteobacteria bacterium
undecaprenyl-phosphate-glucose-PTase
VanZ-2
WP_127843530.1
Acidipropionibacterium jensenii
VanZ-2
NBI84968.1
Lachnospiraceae bacterium
VanZ-2
MBR3680915.1
Clostridia bacterium
VanZ-2
MBR1771708.1
Lachnospiraceae bacterium
VanZ-2
MBQ7955314.1
Lachnospiraceae bacterium
VanZ-2
WP_027971467.1
Streptococcus plurextorum DSM 22810
VanZ-2
?
CDA68537.1
Clostridium sp. CAG:510
GalE/RmlB/Gmd-like_lyase/epimerase
GDP-L-fucose-synthase
?
VanZ-2
MBQ8086596.1
Lachnospiraceae bacterium
VanZ-2
MBQ3490718.1
Clostridia bacterium
VanZ-2
MBR6071623.1
Acholeplasmatales bacterium
VanZ-2
RTL42513.1
Candidatus Melainabacteria bacterium
VanZ-2
MCA1614658.1
Acidobacteriota bacterium
VanZ-2
WP_233113767.1
Enterococcus faecium
VanZ-2
MBP5528571.1
Lachnospiraceae bacterium
VanZ-2
MBR5973966.1
Clostridiales bacterium
VanZ-2
RXE61263.1
Muribaculaceae bacterium Isolate-004 (NCI)
VanZ-2
MBO4687403.1
Clostridiales bacterium
VanZ-2
MBP5180231.1
Clostridiales bacterium
VanZ-2
WP_141274266.1
Kurthia gibsonii
VanZ-2
MBK6465046.1
Myxococcales bacterium
VanZ-2
HGO80670.1
VanZ-2
GFO84312.1
Anaerostipes butyraticus
VanZ-2
MBP5191758.1
Eubacterium sp.
VanZ-2
MBO6089770.1
Lachnospiraceae bacterium
VanZ-2
MBM3781681.1
Acidobacteriota bacterium
VanZ-2
NJM60982.1
Oscillatoriales cyanobacterium RU_3_3
VanZ-2
HGE05850.1
VanZ-2
MBS5725886.1
Clostridiales bacterium
VanZ-2
MBQ2774929.1
Clostridia bacterium
VanZ-2
MBT5925998.1
Verrucomicrobiota bacterium
VanZ-2

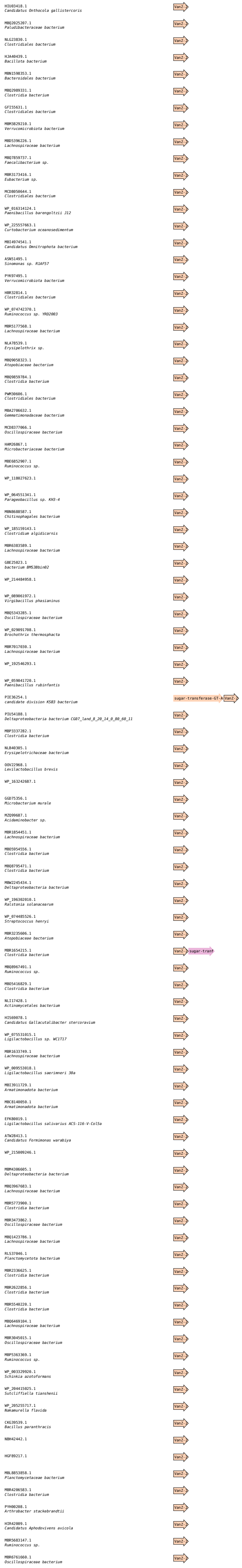
HIU03418.1
Candidatus Onthocola gallistercoris
VanZ-2
MBQ2025207.1
Paludibacteraceae bacterium
VanZ-2
NLG23830.1
Clostridiales bacterium
VanZ-2
HJA40439.1
Bacillota bacterium
VanZ-2
MBN1598353.1
Bacteroidales bacterium
VanZ-2
MBQ2989331.1
Clostridia bacterium
VanZ-2
GFI55631.1
Clostridiales bacterium
VanZ-2
MBM3829210.1
Verrucomicrobiota bacterium
VanZ-2
MBD5396226.1
Lachnospiraceae bacterium
VanZ-2
MBQ7859737.1
Faecalibacterium sp.
VanZ-2
MBR3173416.1
Eubacterium sp.
VanZ-2
MCD8050644.1
Clostridiales bacterium
VanZ-2
WP_016314124.1
Paenibacillus barengoltzii J12
VanZ-2
WP_225557663.1
Curtobacterium oceanosedimentum
VanZ-2
MBI4974541.1
Candidatus Omnitrophota bacterium
VanZ-2
ASN51495.1
Sinomonas sp. R1AF57
VanZ-2
PYK97495.1
Verrucomicrobiota bacterium
VanZ-2
HBR32814.1
Clostridiales bacterium
VanZ-2
WP_074742370.1
Ruminococcus sp. YRD2003
VanZ-2
MBR5177568.1
Lachnospiraceae bacterium
VanZ-2
NLA78539.1
Erysipelothrix sp.
VanZ-2
MBQ9058323.1
Atopobiaceae bacterium
VanZ-2
MBQ9859784.1
Clostridia bacterium
VanZ-2
PWM30606.1
Clostridiales bacterium
VanZ-2
MBA2706632.1
Gemmatimonadaceae bacterium
VanZ-2
MCD8377066.1
Oscillospiraceae bacterium
VanZ-2
HAM26867.1
Microbacteriaceae bacterium
VanZ-2
MBE6852907.1
Ruminococcus sp.
VanZ-2
WP_118027623.1
VanZ-2
WP_064551341.1
Parageobacillus sp. KH3-4
VanZ-2
MBN8688587.1
Chitinophagales bacterium
VanZ-2
WP_185159143.1
Clostridium algidicarnis
VanZ-2
MBR6383589.1
Lachnospiraceae bacterium
VanZ-2
GBE25023.1
bacterium BMS3Bbin02
VanZ-2
WP_214484958.1
VanZ-2
WP_089061972.1
Virgibacillus phasianinus
VanZ-2
MBQ5343285.1
Oscillospiraceae bacterium
VanZ-2
WP_029091708.1
Brochothrix thermosphacta
VanZ-2
MBR7017030.1
Lachnospiraceae bacterium
VanZ-2
WP_192546293.1
VanZ-2
WP_059041720.1
Paenibacillus rubinfantis
VanZ-2
PIE36254.1
candidate division KSB3 bacterium
sugar-transferase-GT-A
VanZ-2
PIU54188.1
Deltaproteobacteria bacterium CG07_land_8_20_14_0_80_60_11
VanZ-2
MBP3337282.1
Clostridia bacterium
VanZ-2
NLB40305.1
Erysipelotrichaceae bacterium
VanZ-2
OOV22968.1
Levilactobacillus brevis
VanZ-2
WP_163242687.1
VanZ-2
GGD75356.1
Microbacterium murale
VanZ-2
MZQ99687.1
Acidaminobacter sp.
VanZ-2
MBR1854451.1
Lachnospiraceae bacterium
VanZ-2
MBO5954556.1
Clostridia bacterium
VanZ-2
MBQ8795471.1
Clostridia bacterium
VanZ-2
MBW2245434.1
Deltaproteobacteria bacterium
VanZ-2
WP_196302010.1
Ralstonia solanacearum
VanZ-2
WP_074485526.1
Streptococcus henryi
VanZ-2
MBR3235606.1
Atopobiaceae bacterium
VanZ-2
MBR1654215.1
Clostridia bacterium
VanZ-2
sugar-tranf
MBQ8967491.1
Ruminococcus sp.
VanZ-2
MBO5416829.1
Clostridia bacterium
VanZ-2
NLI17428.1
Actinomycetales bacterium
VanZ-2
HIS69078.1
Candidatus Gallacutalibacter stercoravium
VanZ-2
WP_075531015.1
Ligilactobacillus sp. WC1T17
VanZ-2
MBR1633749.1
Lachnospiraceae bacterium
VanZ-2
WP_009553018.1
Ligilactobacillus saerimneri 30a
VanZ-2
MBI3911729.1
Armatimonadota bacterium
VanZ-2
MBC8140050.1
Armatimonadota bacterium
VanZ-2
EFK80019.1
Ligilactobacillus salivarius ACS-116-V-Col5a
VanZ-2
ATW28413.1
Candidatus Formimonas warabiya
VanZ-2
WP_215009246.1
VanZ-2
MBM4306605.1
Deltaproteobacteria bacterium
VanZ-2
MBQ3967683.1
Lachnospiraceae bacterium
VanZ-2
MBR5773900.1
Clostridia bacterium
VanZ-2
MBR3473862.1
Oscillospiraceae bacterium
VanZ-2
MBQ1423786.1
Lachnospiraceae bacterium
VanZ-2
RLS37046.1
Planctomycetota bacterium
VanZ-2
MBR2336625.1
Clostridia bacterium
VanZ-2
MBR2622856.1
Clostridia bacterium
VanZ-2
MBR5540220.1
Clostridia bacterium
VanZ-2
MBQ6469104.1
Lachnospiraceae bacterium
VanZ-2
MBR3045015.1
Oscillospiraceae bacterium
VanZ-2
MBP5363369.1
Ruminococcus sp.
VanZ-2
WP_003329920.1
Schinkia azotoformans
VanZ-2
WP_204415025.1
Sutcliffiella tianshenii
VanZ-2
WP_205255717.1
Nakamurella flavida
VanZ-2
CKG39539.1
Bacillus paranthracis
VanZ-2
NBH42442.1
VanZ-2
HGF89217.1
VanZ-2
MBL8853858.1
Planctomycetaceae bacterium
VanZ-2
MBR4206583.1
Clostridia bacterium
VanZ-2
PYH00208.1
Arthrobacter stackebrandtii
VanZ-2
HIR42009.1
Candidatus Aphodovivens avicola
VanZ-2
MBR5683147.1
Ruminococcus sp.
VanZ-2
MBR6761660.1
Oscillospiraceae bacterium
VanZ-2

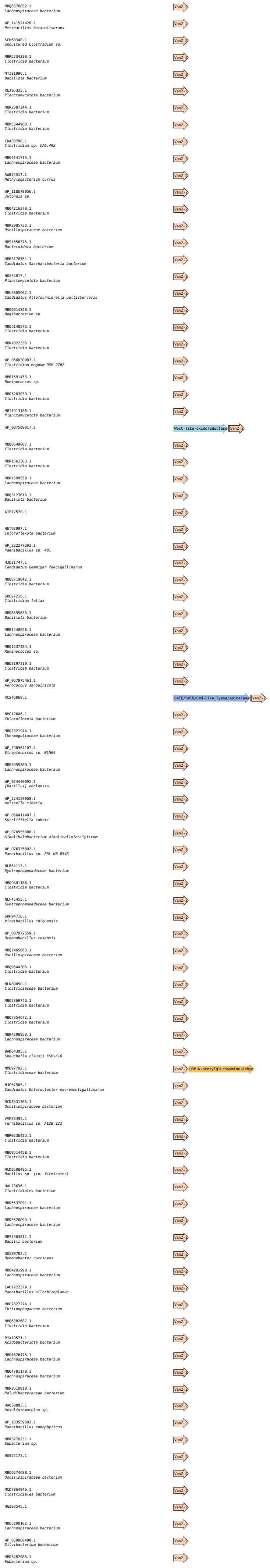
MBQ6376852.1
Lachnospiraceae bacterium
VanZ-2
WP_141521428.1
Peribacillus butanolivorans
VanZ-2
SCH68340.1
uncultured Clostridium sp.
VanZ-2
MBR5234220.1
Clostridia bacterium
VanZ-2
MTI81996.1
Bacillota bacterium
VanZ-2
REJ95335.1
Planctomycetota bacterium
VanZ-2
MBR2387244.1
Clostridia bacterium
VanZ-2
MBR5344908.1
Clostridia bacterium
VanZ-2
CDA30798.1
Clostridium sp. CAG:492
VanZ-2
MBQ9141715.1
Lachnospiraceae bacterium
VanZ-2
AWB24517.1
Methylobacterium currus
VanZ-2
WP_118678056.1
Jutongia sp.
VanZ-2
MBO4216370.1
Clostridia bacterium
VanZ-2
MBR2085733.1
Oscillospiraceae bacterium
VanZ-2
MBS1656375.1
Bacteroidota bacterium
VanZ-2
MBR3176761.1
Candidatus Saccharibacteria bacterium
VanZ-2
NOX54815.1
Planctomycetota bacterium
VanZ-2
MBU3805962.1
Candidatus Allofournierella pullistercoris
VanZ-2
MBQ6314320.1
Mogibacterium sp.
VanZ-2
MBO5148573.1
Clostridia bacterium
VanZ-2
MBR1822336.1
Clostridia bacterium
VanZ-2
WP_066630987.1
Clostridium magnum DSM 2767
VanZ-2
MBR1591453.1
Ruminococcus sp.
VanZ-2
MBO5203039.1
Clostridia bacterium
VanZ-2
MBI1913188.1
Planctomycetota bacterium
VanZ-2
WP_087508017.1
WecC-like-oxidoreductase
VanZ-2
MBQ8649007.1
Clostridia bacterium
VanZ-2
MBR1561503.1
Clostridia bacterium
VanZ-2
MBR1599559.1
Lachnospiraceae bacterium
VanZ-2
MBQ3123616.1
Bacillota bacterium
VanZ-2
AIF17570.1
VanZ-2
HET92097.1
Chloroflexota bacterium
VanZ-2
WP_232277393.1
Paenibacillus sp. 481
VanZ-2
HJD21747.1
Candidatus Gemmiger faecigallinarum
VanZ-2
MBQ8719042.1
Clostridia bacterium
VanZ-2
SHE97216.1
Clostridium fallax
VanZ-2
MBQ6555635.1
Bacillota bacterium
VanZ-2
MBR1440026.1
Lachnospiraceae bacterium
VanZ-2
MBQ1537484.1
Ruminococcus sp.
VanZ-2
MBQ8197219.1
Clostridia bacterium
VanZ-2
WP_067975461.1
Aerococcus sanguinicola
VanZ-2
RCS46069.1
GalE/RmlB/Gmd-like_lyase/epimerase
VanZ-2
NMC12606.1
Chloroflexota bacterium
VanZ-2
MBQ2821944.1
Thermoguttaceae bacterium
VanZ-2
WP_196967107.1
Streptococcus sp. NLN64
VanZ-2
MBE5959309.1
Lachnospiraceae bacterium
VanZ-2
WP_074440092.1
[Bacillus] enclensis
VanZ-2
WP_224159068.1
Weissella cibaria
VanZ-2
WP_066411407.1
Sutcliffiella cohnii
VanZ-2
WP_078555898.1
Alkalihalobacterium alkalicellulosilyticum
VanZ-2
WP_076335892.1
Paenibacillus sp. FSL H8-0548
VanZ-2
NLB54113.1
Syntrophomonadaceae bacterium
VanZ-2
MBQ9401186.1
Clostridia bacterium
VanZ-2
NLF45455.1
Syntrophomonadaceae bacterium
VanZ-2
SHH94716.1
Virgibacillus chiguensis
VanZ-2
WP_087972559.1
Oceanobacillus rekensis
VanZ-2
MBQ7465063.1
Oscillospiraceae bacterium
VanZ-2
MBQ9544385.1
Clostridia bacterium
VanZ-2
NLK89850.1
Clostridiaceae bacterium
VanZ-2
MBQ7369746.1
Clostridia bacterium
VanZ-2
MBQ7354672.1
Clostridia bacterium
VanZ-2
MBR4208958.1
Lachnospiraceae bacterium
VanZ-2
BAD64305.1
Shouchella clausii KSM-K16
VanZ-2
NMB97792.1
Clostridiaceae bacterium
VanZ-2
UDP-N-acetylglucosamine-dehyd
HJC67565.1
Candidatus Enterocloster excrementigallinarum
VanZ-2
MCD8331305.1
Oscillospiraceae bacterium
VanZ-2
VVM33495.1
Terribacillus sp. AE2B 122
VanZ-2
MBR0236425.1
Clostridia bacterium
VanZ-2
MBQ9514458.1
Clostridia bacterium
VanZ-2
MCD8508485.1
Bacillus sp. (in: firmicutes)
VanZ-2
HAL73636.1
Clostridiales bacterium
VanZ-2
MBD5537091.1
Lachnospiraceae bacterium
VanZ-2
MBD5520093.1
Lachnospiraceae bacterium
VanZ-2
MBS1363811.1
Bacilli bacterium
VanZ-2
OGX80763.1
Hymenobacter coccineus
VanZ-2
MBO4291990.1
Lachnospiraceae bacterium
VanZ-2
CAH1222379.1
Paenibacillus allorhizoplanae
VanZ-2
MBC7827274.1
Chitinophagaceae bacterium
VanZ-2
MBQ6382687.1
Clostridia bacterium
VanZ-2
PYX10571.1
Acidobacteriota bacterium
VanZ-2
MBO4626475.1
Lachnospiraceae bacterium
VanZ-2
MBO4701179.1
Lachnospiraceae bacterium
VanZ-2
MBR2618910.1
Paludibacteraceae bacterium
VanZ-2
HAU30883.1
Desulfotomaculum sp.
VanZ-2
WP_183559602.1
Paenibacillus endophyticus
VanZ-2
MBR3276231.1
Eubacterium sp.
VanZ-2
HGS25173.1
VanZ-2
MBQ6274688.1
Oscillospiraceae bacterium
VanZ-2
MCD7864946.1
Clostridiales bacterium
VanZ-2
HGZ03545.1
VanZ-2
MBO5208102.1
Lachnospiraceae bacterium
VanZ-2
WP_050060400.1
Silvibacterium bohemicum
VanZ-2
MBO5487085.1
Eubacterium sp.
VanZ-2

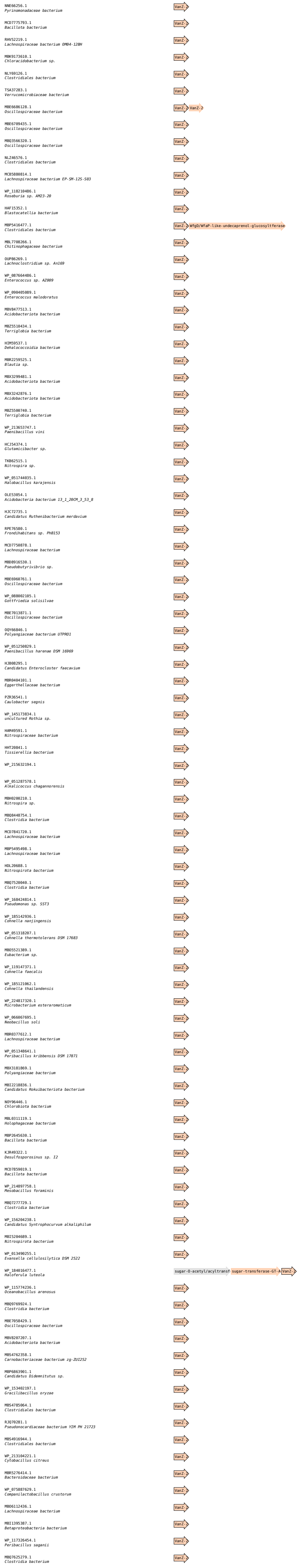
NNE66256.1
Pyrinomonadaceae bacterium
VanZ-2
MCD7775793.1
Bacillota bacterium
VanZ-2
RHV52219.1
Lachnospiraceae bacterium OM04-12BH
VanZ-2
MBK9173610.1
Chloracidobacterium sp.
VanZ-2
NLY69126.1
Clostridiales bacterium
VanZ-2
TSA37283.1
Verrucomicrobiaceae bacterium
VanZ-2
MBE6686128.1
Oscillospiraceae bacterium
VanZ-2
VanZ-2
MBE6709435.1
Oscillospiraceae bacterium
VanZ-2
MBQ3566320.1
Oscillospiraceae bacterium
VanZ-2
NLZ46576.1
Clostridiales bacterium
VanZ-2
MCB5880814.1
Lachnospiraceae bacterium EP-SM-12S-S03
VanZ-2
WP_118210486.1
Roseburia sp. AM23-20
VanZ-2
HAF15352.1
Blastocatellia bacterium
VanZ-2
MBP5416477.1
Clostridiales bacterium
VanZ-2
WfgD/WfaP-like-undecaprenol-glucosyltferase
MBL7708266.1
Chitinophagaceae bacterium
VanZ-2
OUP86269.1
Lachnoclostridium sp. An169
VanZ-2
WP_087664486.1
Enterococcus sp. AZ009
VanZ-2
WP_090405089.1
Enterococcus malodoratus
VanZ-2
MBV8477513.1
Acidobacteriota bacterium
VanZ-2
MBZ5510434.1
Terriglobia bacterium
VanZ-2
HIM59537.1
Dehalococcoidia bacterium
VanZ-2
MBR2259525.1
Blautia sp.
VanZ-2
MBX3299481.1
Acidobacteriota bacterium
VanZ-2
MBX3242876.1
Acidobacteriota bacterium
VanZ-2
MBZ5500740.1
Terriglobia bacterium
VanZ-2
WP_213653747.1
Paenibacillus vini
VanZ-2
HCJ54374.1
Glutamicibacter sp.
VanZ-2
TKB62515.1
Nitrospira sp.
VanZ-2
WP_051744035.1
Halobacillus karajensis
VanZ-2
OLE53054.1
Acidobacteria bacterium 13_1_20CM_3_53_8
VanZ-2
HJC72735.1
Candidatus Ruthenibacterium merdavium
VanZ-2
RPE76580.1
Frondihabitans sp. PhB153
VanZ-2
MCD7750878.1
Lachnospiraceae bacterium
VanZ-2
MBD8916530.1
Pseudobutyrivibrio sp.
VanZ-2
MBE6960761.1
Oscillospiraceae bacterium
VanZ-2
WP_088002105.1
Gottfriedia solisilvae
VanZ-2
MBE7013871.1
Oscillospiraceae bacterium
VanZ-2
OQY66846.1
Polyangiaceae bacterium UTPRO1
VanZ-2
WP_051250829.1
Paenibacillus harenae DSM 16969
VanZ-2
HJB08295.1
Candidatus Enterocloster faecavium
VanZ-2
MBR0404101.1
Eggerthellaceae bacterium
VanZ-2
PZR36541.1
Caulobacter segnis
VanZ-2
WP_145173834.1
uncultured Rothia sp.
VanZ-2
HAM49591.1
Nitrospiraceae bacterium
VanZ-2
HHT20841.1
Tissierellia bacterium
VanZ-2
WP_215632194.1
VanZ-2
WP_051287578.1
Alkalicoccus chagannorensis
VanZ-2
MBH0200210.1
Nitrospira sp.
VanZ-2
MBQ8448754.1
Clostridia bacterium
VanZ-2
MCD7841720.1
Lachnospiraceae bacterium
VanZ-2
MBP5495498.1
Lachnospiraceae bacterium
VanZ-2
HDL20688.1
Nitrospirota bacterium
VanZ-2
MBQ7520040.1
Clostridia bacterium
VanZ-2
WP_168424814.1
Pseudomonas sp. SST3
VanZ-2
WP_185142936.1
Cohnella nanjingensis
VanZ-2
WP_051318207.1
Cohnella thermotolerans DSM 17683
VanZ-2
MBO5521389.1
Eubacterium sp.
VanZ-2
WP_119147371.1
Cohnella faecalis
VanZ-2
WP_185121062.1
Cohnella thailandensis
VanZ-2
WP_224817320.1
Microbacterium esteraromaticum
VanZ-2
WP_066067695.1
Neobacillus soli
VanZ-2
MBR0377612.1
Lachnospiraceae bacterium
VanZ-2
WP_051348641.1
Peribacillus kribbensis DSM 17871
VanZ-2
MBX3181869.1
Polyangiaceae bacterium
VanZ-2
MBI2218836.1
Candidatus Rokuibacteriota bacterium
VanZ-2
NOY96446.1
Chlorobiota bacterium
VanZ-2
MBL0311119.1
Holophagaceae bacterium
VanZ-2
MBP2645630.1
Bacillota bacterium
VanZ-2
KJR49322.1
Desulfosporosinus sp. I2
VanZ-2
MCD7859019.1
Bacillota bacterium
VanZ-2
WP_214897758.1
Mesobacillus foraminis
VanZ-2
MBQ7277729.1
Clostridia bacterium
VanZ-2
WP_156204238.1
Candidatus Syntrophocurvum alkaliphilum
VanZ-2
MBI5204689.1
Nitrospirota bacterium
VanZ-2
WP_013490255.1
Evansella cellulosilytica DSM 2522
VanZ-2
WP_184016477.1
Haloferula luteola
sugar-O-acetyl/acyltransf
sugar-transferase-GT-A
VanZ-2
WP_115774236.1
Oceanobacillus arenosus
VanZ-2
MBQ9769924.1
Clostridia bacterium
VanZ-2
MBE7058429.1
Oscillospiraceae bacterium
VanZ-2
MBV8207207.1
Acidobacteriota bacterium
VanZ-2
MBS4762358.1
Carnobacteriaceae bacterium zg-ZUI252
VanZ-2
MBP6863901.1
Candidatus Didemnitutus sp.
VanZ-2
WP_153402197.1
Gracilibacillus oryzae
VanZ-2
MBS4785064.1
Clostridiales bacterium
VanZ-2
RJQ70281.1
Pseudonocardiaceae bacterium YIM PH 21723
VanZ-2
MBS4916944.1
Clostridiales bacterium
VanZ-2
WP_213104221.1
Cytobacillus citreus
VanZ-2
MBR5276414.1
Bacteroidaceae bacterium
VanZ-2
WP_075887629.1
Companilactobacillus crustorum
VanZ-2
MBO6112436.1
Lachnospiraceae bacterium
VanZ-2
MBI1395387.1
Betaproteobacteria bacterium
VanZ-2
WP_117326454.1
Peribacillus saganii
VanZ-2
MBQ7625279.1
Clostridia bacterium
VanZ-2

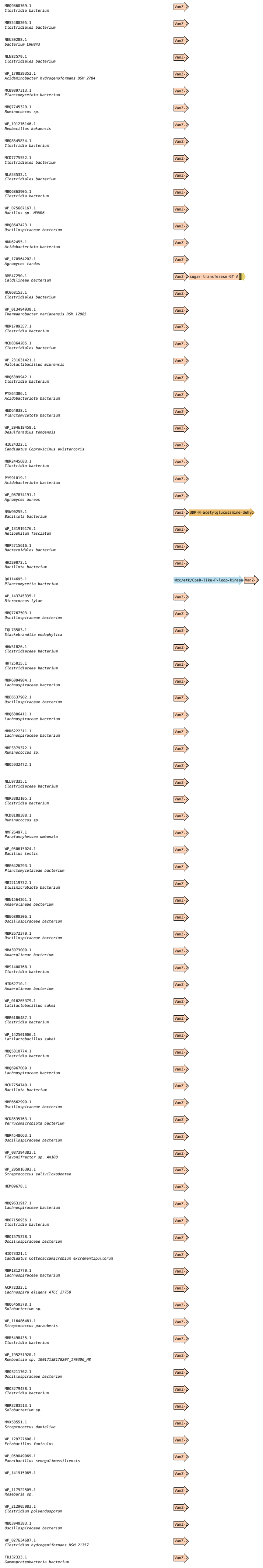

MBQ9860769.1
Clostridia bacterium
VanZ-2
MBS5480205.1
Clostridiales bacterium
VanZ-2
NEU30288.1
bacterium LRH843
VanZ-2
NLN82579.1
Clostridiales bacterium
VanZ-2
WP_170829352.1
Acidaminobacter hydrogenoformans DSM 2784
VanZ-2
MCB9897313.1
Planctomycetota bacterium
VanZ-2
MBQ7745329.1
Ruminococcus sp.
VanZ-2
WP_191276146.1
Neobacillus kokaensis
VanZ-2
MBQ8545834.1
Clostridia bacterium
VanZ-2
MCD7775552.1
Clostridiales bacterium
VanZ-2
NLA53532.1
Clostridiales bacterium
VanZ-2
MBQ6863905.1
Clostridia bacterium
VanZ-2
WP_075687167.1
Bacillus sp. MRMR6
VanZ-2
MBQ8647423.1
Oscillospiraceae bacterium
VanZ-2
NDD62455.1
Acidobacteriota bacterium
VanZ-2
WP_170964202.1
Agromyces tardus
VanZ-2
RME47290.1
Caldilineae bacterium
VanZ-2
sugar-transferase-GT-A
HCG68153.1
Clostridiales bacterium
VanZ-2
WP_013494938.1
Thermaerobacter marianensis DSM 12885
VanZ-2
MBR1709357.1
Clostridia bacterium
VanZ-2
MCD8364285.1
Clostridiales bacterium
VanZ-2
WP_231631421.1
Halolactibacillus miurensis
VanZ-2
MBQ6399942.1
Clostridia bacterium
VanZ-2
PYX64386.1
Acidobacteriota bacterium
VanZ-2
HED64038.1
Planctomycetota bacterium
VanZ-2
WP_204618458.1
Desulforadius tongensis
VanZ-2
HIU24322.1
Candidatus Coprovicinus avistercoris
VanZ-2
MBR2445683.1
Clostridia bacterium
VanZ-2
PYS91019.1
Acidobacteriota bacterium
VanZ-2
WP_067874191.1
Agromyces aureus
VanZ-2
NSW90255.1
Bacillota bacterium
VanZ-2
UDP-N-acetylglucosamine-dehyd
WP_131919176.1
Heliophilum fasciatum
VanZ-2
MBP5715616.1
Bacteroidales bacterium
VanZ-2
HHZ20072.1
Bacillota bacterium
VanZ-2
QOJ14695.1
Planctomycetia bacterium
Wzc/etk/CpsD-like-P-loop-kinase
VanZ-2
WP_143745335.1
Micrococcus lylae
VanZ-2
MBQ7767503.1
Oscillospiraceae bacterium
VanZ-2
TQL78503.1
Stackebrandtia endophytica
VanZ-2
HHW31826.1
Clostridiaceae bacterium
VanZ-2
HHT25015.1
Clostridiaceae bacterium
VanZ-2
MBR6094984.1
Lachnospiraceae bacterium
VanZ-2
MBE6537902.1
Oscillospiraceae bacterium
VanZ-2
MBQ6886411.1
Lachnospiraceae bacterium
VanZ-2
MBR6222311.1
Lachnospiraceae bacterium
VanZ-2
MBP3379372.1
Ruminococcus sp.
VanZ-2
MBQ5932472.1
VanZ-2
NLL97335.1
Clostridiaceae bacterium
VanZ-2
MBR3883105.1
Clostridia bacterium
VanZ-2
MCD8188388.1
Ruminococcus sp.
VanZ-2
NMF26497.1
Parafannyhessea umbonata
VanZ-2
WP_050615024.1
Bacillus testis
VanZ-2
MBE6426293.1
Planctomycetaceae bacterium
VanZ-2
MBI2119732.1
Elusimicrobiota bacterium
VanZ-2
MBN1564261.1
Anaerolineae bacterium
VanZ-2
MBE6800306.1
Oscillospiraceae bacterium
VanZ-2
MBR2672370.1
Oscillospiraceae bacterium
VanZ-2
MBA3073009.1
Anaerolineae bacterium
VanZ-2
MBS1400768.1
Clostridia bacterium
VanZ-2
HID62710.1
Anaerolineae bacterium
VanZ-2
WP_016265379.1
Latilactobacillus sakei
VanZ-2
MBR6186487.1
Clostridia bacterium
VanZ-2
WP_142501006.1
Latilactobacillus sakei
VanZ-2
MBQ5810774.1
Clostridia bacterium
VanZ-2
MBQ6967009.1
Lachnospiraceae bacterium
VanZ-2
MCD7754740.1
Bacillota bacterium
VanZ-2
MBE6662999.1
Oscillospiraceae bacterium
VanZ-2
MCD8535763.1
Verrucomicrobiota bacterium
VanZ-2
MBR4548663.1
Oscillospiraceae bacterium
VanZ-2
WP_087394382.1
Flavonifractor sp. An100
VanZ-2
WP_205016393.1
Streptococcus saliviloxodontae
VanZ-2
HEM09678.1
VanZ-2
MBQ9631917.1
Lachnospiraceae bacterium
VanZ-2
MBO7156936.1
Clostridia bacterium
VanZ-2
MBQ1575378.1
Oscillospiraceae bacterium
VanZ-2
HIQ73321.1
Candidatus Cottocaccamicrobium excrementipullorum
VanZ-2
MBR1812770.1
Lachnospiraceae bacterium
VanZ-2
ACR72333.1
Lachnospira eligens ATCC 27750
VanZ-2
MBQ6450378.1
Solobacterium sp.
VanZ-2
WP_116486481.1
Streptococcus parauberis
VanZ-2
MBR5498435.1
Clostridia bacterium
VanZ-2
WP_195251920.1
Romboutsia sp. 1001713B170207_170306_H8
VanZ-2
MBQ3211762.1
Oscillospiraceae bacterium
VanZ-2
MBQ3279438.1
Clostridia bacterium
VanZ-2
MBR3203513.1
Solobacterium sp.
VanZ-2
MVX58551.1
Streptococcus danieliae
VanZ-2
WP_129727608.1
Ectobacillus funiculus
VanZ-2
WP_059049969.1
Paenibacillus senegalimassiliensis
VanZ-2
WP_141915065.1
VanZ-2
WP_117922505.1
Roseburia sp.
VanZ-2
WP_212905083.1
Clostridium polyendosporum
VanZ-2
MBQ3940383.1
Oscillospiraceae bacterium
VanZ-2
WP_027634687.1
Clostridium hydrogeniformans DSM 21757
VanZ-2
TDJ32333.1
Gammaproteobacteria bacterium
VanZ-2

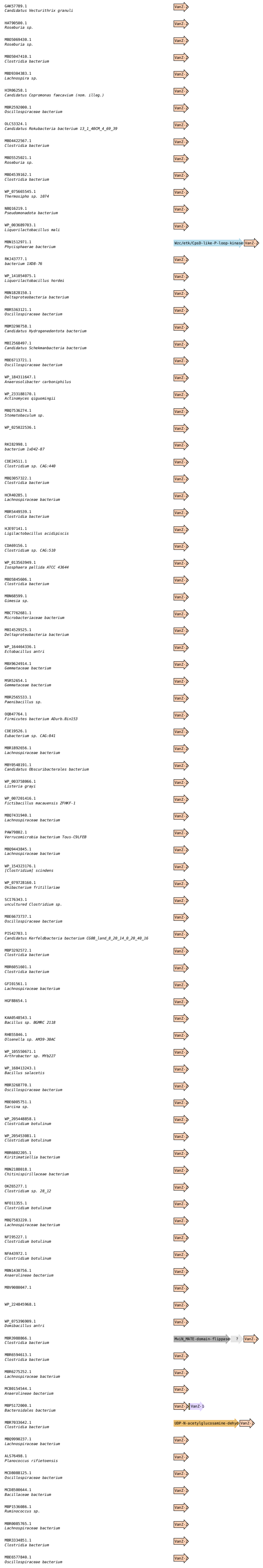
GAK57789.1
Candidatus Vecturithrix granuli
VanZ-2
HAT90500.1
Roseburia sp.
VanZ-2
MBO5069430.1
Roseburia sp.
VanZ-2
MBO5047410.1
Clostridia bacterium
VanZ-2
MBD9304383.1
Lachnospira sp.
VanZ-2
HIR06258.1
Candidatus Copromonas faecavium (nom. illeg.)
VanZ-2
MBR2592000.1
Oscillospiraceae bacterium
VanZ-2
OLC53324.1
Candidatus Rokubacteria bacterium 13_1_40CM_4_69_39
VanZ-2
MBO4422567.1
Clostridia bacterium
VanZ-2
MBO5525021.1
Roseburia sp.
VanZ-2
MBO4539162.1
Clostridia bacterium
VanZ-2
WP_075665545.1
Thermosipho sp. 1074
VanZ-2
NBQ16219.1
Pseudomonadota bacterium
VanZ-2
WP_003689703.1
Liquorilactobacillus mali
VanZ-2
MBN1512971.1
Phycisphaerae bacterium
Wzc/etk/CpsD-like-P-loop-kinase
VanZ-2
RKJ43777.1
bacterium 1XD8-76
VanZ-2
WP_141054075.1
Liquorilactobacillus hordei
VanZ-2
MBN1828150.1
Deltaproteobacteria bacterium
VanZ-2
MBR5363121.1
Oscillospiraceae bacterium
VanZ-2
MBM3290758.1
Candidatus Hydrogenedentota bacterium
VanZ-2
MBI2568497.1
Candidatus Schekmanbacteria bacterium
VanZ-2
MBE6713721.1
Oscillospiraceae bacterium
VanZ-2
WP_184311647.1
Anaerosolibacter carboniphilus
VanZ-2
WP_233188170.1
Actinomyces qiguomingii
VanZ-2
MBQ7536274.1
Stomatobaculum sp.
VanZ-2
WP_025022536.1
VanZ-2
RKI82998.1
bacterium 1xD42-87
VanZ-2
CDE24511.1
Clostridium sp. CAG:440
VanZ-2
MBQ3057322.1
Clostridia bacterium
VanZ-2
HCR40285.1
Lachnospiraceae bacterium
VanZ-2
MBR5449539.1
Clostridia bacterium
VanZ-2
HJE97141.1
Ligilactobacillus acidipiscis
VanZ-2
CDA69156.1
Clostridium sp. CAG:510
VanZ-2
WP_013563949.1
Isosphaera pallida ATCC 43644
VanZ-2
MBO5845606.1
Clostridia bacterium
VanZ-2
MBN68599.1
Gimesia sp.
VanZ-2
MBC7762681.1
Microbacteriaceae bacterium
VanZ-2
MBI4529525.1
Deltaproteobacteria bacterium
VanZ-2
WP_164464336.1
Ectobacillus antri
VanZ-2
MBX9624914.1
Gemmataceae bacterium
VanZ-2
MSR52654.1
Gemmataceae bacterium
VanZ-2
MBR2565533.1
Paenibacillus sp.
VanZ-2
OQB47764.1
Firmicutes bacterium ADurb.Bin153
VanZ-2
CDE19526.1
Eubacterium sp. CAG:841
VanZ-2
MBR1892656.1
Lachnospiraceae bacterium
VanZ-2
MBY0548191.1
Candidatus Obscuribacterales bacterium
VanZ-2
WP_003758066.1
Listeria grayi
VanZ-2
WP_007201416.1
Fictibacillus macauensis ZFHKF-1
VanZ-2
MBQ7431940.1
Lachnospiraceae bacterium
VanZ-2
PAW79802.1
Verrucomicrobia bacterium Tous-C9LFEB
VanZ-2
MBQ9443845.1
Lachnospiraceae bacterium
VanZ-2
WP_154323176.1
[Clostridium] scindens
VanZ-2
WP_079728160.1
Okibacterium fritillariae
VanZ-2
SCI76343.1
uncultured Clostridium sp.
VanZ-2
MBE6673737.1
Oscillospiraceae bacterium
VanZ-2
PIS42703.1
Candidatus Kerfeldbacteria bacterium CG08_land_8_20_14_0_20_40_16
VanZ-2
MBP3292572.1
Clostridia bacterium
VanZ-2
MBR6051601.1
Clostridia bacterium
VanZ-2
GFI01561.1
Lachnospiraceae bacterium
VanZ-2
HGF88654.1
VanZ-2
KAA0548543.1
Bacillus sp. BGMRC 2118
VanZ-2
RHB55846.1
Olsenella sp. AM39-30AC
VanZ-2
WP_105550671.1
Arthrobacter sp. MYb227
VanZ-2
WP_168413243.1
Bacillus salacetis
VanZ-2
MBR3268770.1
Oscillospiraceae bacterium
VanZ-2
MBE6005751.1
Sarcina sp.
VanZ-2
WP_205448858.1
Clostridium botulinum
VanZ-2
WP_205453081.1
Clostridium botulinum
VanZ-2
MBR6802205.1
Kiritimatiellia bacterium
VanZ-2
MBN2188018.1
Chitinispirillaceae bacterium
VanZ-2
OKZ65277.1
Clostridium sp. 28_12
VanZ-2
NFO11355.1
Clostridium botulinum
VanZ-2
MBQ7583220.1
Lachnospiraceae bacterium
VanZ-2
NFI95227.1
Clostridium botulinum
VanZ-2
NFA43972.1
Clostridium botulinum
VanZ-2
MBN1430756.1
Anaerolineae bacterium
VanZ-2
MBV9080047.1
VanZ-2
WP_224845968.1
VanZ-2
WP_075396909.1
Domibacillus antri
VanZ-2
MBR3988866.1
Clostridia bacterium
MviN_MATE-domain-flippase
?
VanZ-2
MBR6594613.1
Clostridia bacterium
VanZ-2
MBR6275252.1
Lachnospiraceae bacterium
VanZ-2
MCB0154544.1
Anaerolineae bacterium
VanZ-2
MBP5172000.1
Bacteroidales bacterium
VanZ-2
VanZ-1
MBR7033642.1
Clostridia bacterium
UDP-N-acetylglucosamine-dehyd
VanZ-2
MBQ9990237.1
Lachnospiraceae bacterium
VanZ-2
ALS76498.1
Planococcus rifietoensis
VanZ-2
MCD8088125.1
Oscillospiraceae bacterium
VanZ-2
MCD8500644.1
Bacillaceae bacterium
VanZ-2
MBP1536086.1
Ruminococcus sp.
VanZ-2
MBR0085765.1
Lachnospiraceae bacterium
VanZ-2
MBR3334851.1
Clostridia bacterium
VanZ-2
MBE6577840.1
Oscillospiraceae bacterium
VanZ-2

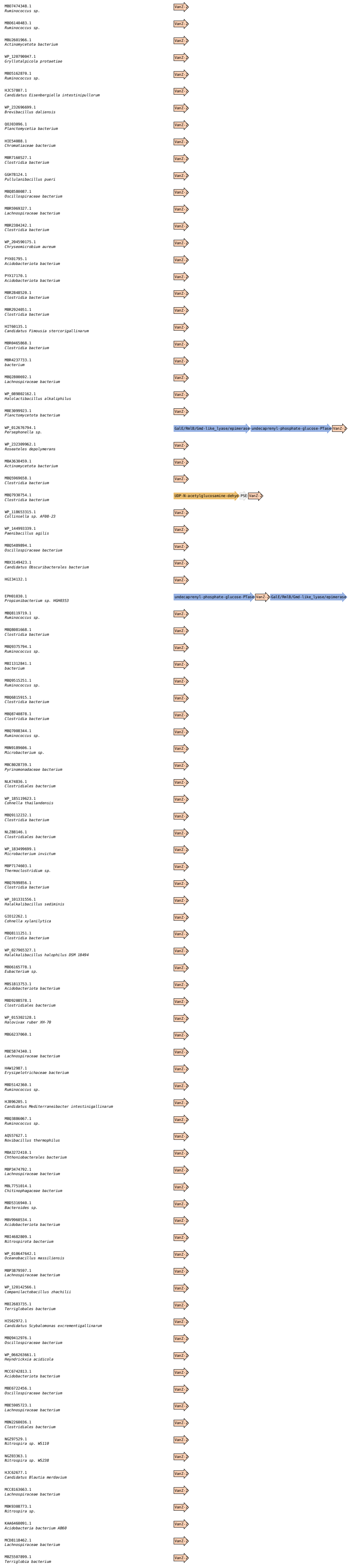
MBO7474348.1
Ruminococcus sp.
VanZ-2
MBO6140483.1
Ruminococcus sp.
VanZ-2
MBU2601966.1
Actinomycetota bacterium
VanZ-2
WP_120790047.1
Gryllotalpicola protaetiae
VanZ-2
MBO5162870.1
Ruminococcus sp.
VanZ-2
HJC57807.1
Candidatus Eisenbergiella intestinipullorum
VanZ-2
WP_232696699.1
Brevibacillus daliensis
VanZ-2
QOJ03096.1
Planctomycetia bacterium
VanZ-2
HIE54088.1
Chromatiaceae bacterium
VanZ-2
MBR7160527.1
Clostridia bacterium
VanZ-2
GGH78124.1
Pullulanibacillus pueri
VanZ-2
MBQ8580087.1
Oscillospiraceae bacterium
VanZ-2
MBR5969327.1
Lachnospiraceae bacterium
VanZ-2
MBR2384242.1
Clostridia bacterium
VanZ-2
WP_204590175.1
Chryseomicrobium aureum
VanZ-2
PYX01795.1
Acidobacteriota bacterium
VanZ-2
PYX17170.1
Acidobacteriota bacterium
VanZ-2
MBR2848520.1
Clostridia bacterium
VanZ-2
MBR2924051.1
Clostridia bacterium
VanZ-2
HIT60135.1
Candidatus Fimousia stercorigallinarum
VanZ-2
MBR0465868.1
Clostridia bacterium
VanZ-2
MBR4237733.1
bacterium
VanZ-2
MBQ2800692.1
Lachnospiraceae bacterium
VanZ-2
WP_089802162.1
Halolactibacillus alkaliphilus
VanZ-2
MBE3099923.1
Planctomycetota bacterium
VanZ-2
WP_012676794.1
Persephonella sp.
GalE/RmlB/Gmd-like_lyase/epimerase
undecaprenyl-phosphate-glucose-PTase
VanZ-2
WP_232309962.1
Roseateles depolymerans
VanZ-2
MBA3630459.1
Actinomycetota bacterium
VanZ-2
MBQ5969658.1
Clostridia bacterium
VanZ-2
MBQ7930754.1
Clostridia bacterium
UDP-N-acetylglucosamine-dehyd
PSE
VanZ-2
WP_118653315.1
Collinsella sp. AF08-23
VanZ-2
WP_144993339.1
Paenibacillus agilis
VanZ-2
MBQ5489894.1
Oscillospiraceae bacterium
VanZ-2
MBX3149423.1
Candidatus Obscuribacterales bacterium
VanZ-2
HGI34132.1
VanZ-2
EPH01030.1
Propionibacterium sp. HGH0353
undecaprenyl-phosphate-glucose-PTase
VanZ-2
GalE/RmlB/Gmd-like_lyase/epimerase
MBQ8119719.1
Ruminococcus sp.
VanZ-2
MBQ8081668.1
Clostridia bacterium
VanZ-2
MBQ9375794.1
Ruminococcus sp.
VanZ-2
MBI1312841.1
bacterium
VanZ-2
MBQ9515251.1
Ruminococcus sp.
VanZ-2
MBQ6815915.1
Clostridia bacterium
VanZ-2
MBQ8740878.1
Clostridia bacterium
VanZ-2
MBQ7008344.1
Ruminococcus sp.
VanZ-2
MBN9189606.1
Microbacterium sp.
VanZ-2
MBC8028739.1
Pyrinomonadaceae bacterium
VanZ-2
NLK74836.1
Clostridiales bacterium
VanZ-2
WP_185119623.1
Cohnella thailandensis
VanZ-2
MBQ9112232.1
Clostridia bacterium
VanZ-2
NLZ88146.1
Clostridiales bacterium
VanZ-2
WP_183499699.1
Microbacterium invictum
VanZ-2
MBP7174603.1
Thermoclostridium sp.
VanZ-2
MBQ7699856.1
Clostridia bacterium
VanZ-2
WP_101331556.1
Halalkalibacillus sediminis
VanZ-2
GIO12262.1
Cohnella xylanilytica
VanZ-2
MBQ8111251.1
Clostridia bacterium
VanZ-2
WP_027965327.1
Halalkalibacillus halophilus DSM 18494
VanZ-2
MBO6165778.1
Eubacterium sp.
VanZ-2
MBS1813753.1
Acidobacteriota bacterium
VanZ-2
MBD9208578.1
Clostridiales bacterium
VanZ-2
WP_015302128.1
Halovivax ruber XH-70
VanZ-2
MBG6237060.1
VanZ-2
MBE5874340.1
Lachnospiraceae bacterium
VanZ-2
HAW12987.1
Erysipelotrichaceae bacterium
VanZ-2
MBD5142360.1
Ruminococcus sp.
VanZ-2
HJB96205.1
Candidatus Mediterraneibacter intestinigallinarum
VanZ-2
MBQ3886067.1
Ruminococcus sp.
VanZ-2
AQS57627.1
Novibacillus thermophilus
VanZ-2
MBA3272410.1
Chthoniobacterales bacterium
VanZ-2
MBP3474792.1
Lachnospiraceae bacterium
VanZ-2
MBL7751014.1
Chitinophagaceae bacterium
VanZ-2
MBD5316940.1
Bacteroides sp.
VanZ-2
MBV9960534.1
Acidobacteriota bacterium
VanZ-2
MBI4682809.1
Nitrospirota bacterium
VanZ-2
WP_010647642.1
Oceanobacillus massiliensis
VanZ-2
MBP3879597.1
Lachnospiraceae bacterium
VanZ-2
WP_120142566.1
Companilactobacillus zhachilii
VanZ-2
MBI2683735.1
Terriglobales bacterium
VanZ-2
HIS62972.1
Candidatus Scybalomonas excrementigallinarum
VanZ-2
MBQ9412976.1
Oscillospiraceae bacterium
VanZ-2
WP_066263661.1
Heyndrickxia acidicola
VanZ-2
MCC6742813.1
Acidobacteriota bacterium
VanZ-2
MBE6722456.1
Oscillospiraceae bacterium
VanZ-2
MBE5905723.1
Lachnospiraceae bacterium
VanZ-2
MBN2260036.1
Clostridiales bacterium
VanZ-2
NGZ97529.1
Nitrospira sp. WS110
VanZ-2
NGZ03363.1
Nitrospira sp. WS238
VanZ-2
HJC62677.1
Candidatus Blautia merdavium
VanZ-2
MCC8163663.1
Lachnospiraceae bacterium
VanZ-2
MBK9308773.1
Nitrospira sp.
VanZ-2
KAA6460091.1
Acidobacteria bacterium AB60
VanZ-2
MCD8118462.1
Lachnospiraceae bacterium
VanZ-2
MBZ5507899.1
Terriglobia bacterium
VanZ-2

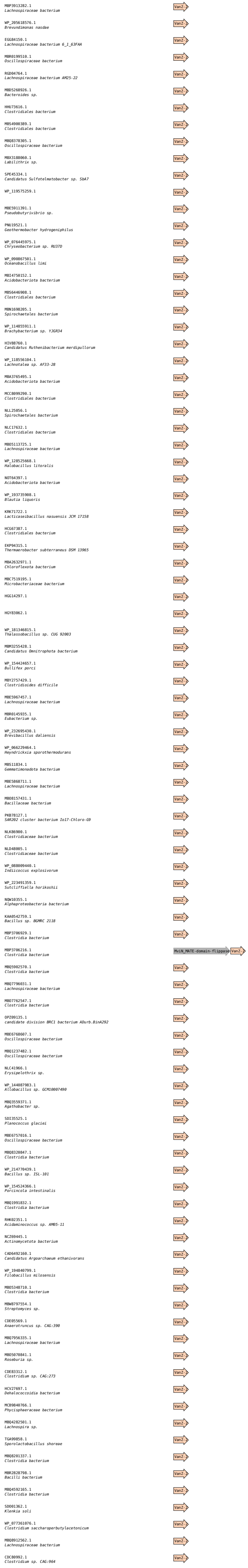

MBP3913282.1
Lachnospiraceae bacterium
VanZ-2
WP_205618576.1
Brevundimonas nasdae
VanZ-2
EGG84150.1
Lachnospiraceae bacterium 6_1_63FAA
VanZ-2
MBR0199510.1
Oscillospiraceae bacterium
VanZ-2
RGD04764.1
Lachnospiraceae bacterium AM25-22
VanZ-2
MBD5268926.1
Bacteroides sp.
VanZ-2
HHU73616.1
Clostridiales bacterium
VanZ-2
MBS4900389.1
Clostridiales bacterium
VanZ-2
MBQ8378305.1
Oscillospiraceae bacterium
VanZ-2
MBX3188060.1
Labilithrix sp.
VanZ-2
SPE45334.1
Candidatus Sulfotelmatobacter sp. SbA7
VanZ-2
WP_119575259.1
VanZ-2
MBE5911391.1
Pseudobutyrivibrio sp.
VanZ-2
PNU19521.1
Geothermobacter hydrogeniphilus
VanZ-2
WP_076445975.1
Chryseobacterium sp. RU37D
VanZ-2
WP_090867501.1
Oceanobacillus limi
VanZ-2
MBI4750152.1
Acidobacteriota bacterium
VanZ-2
MBS6446908.1
Clostridiales bacterium
VanZ-2
MBN1698205.1
Spirochaetales bacterium
VanZ-2
WP_114855911.1
Brachybacterium sp. YJGR34
VanZ-2
HIV88760.1
Candidatus Ruthenibacterium merdipullorum
VanZ-2
WP_118556104.1
Lachnotalea sp. AF33-28
VanZ-2
MBA3765495.1
Acidobacteriota bacterium
VanZ-2
MCC8099290.1
Clostridiales bacterium
VanZ-2
NLL25056.1
Spirochaetales bacterium
VanZ-2
NLC17632.1
Clostridiales bacterium
VanZ-2
MBO5113725.1
Lachnospiraceae bacterium
VanZ-2
WP_128525668.1
Halobacillus litoralis
VanZ-2
NOT64397.1
Acidobacteriota bacterium
VanZ-2
WP_193735908.1
Blautia liquoris
VanZ-2
KRK71722.1
Lacticaseibacillus nasuensis JCM 17158
VanZ-2
HCG67387.1
Clostridiales bacterium
VanZ-2
EKP94315.1
Thermaerobacter subterraneus DSM 13965
VanZ-2
MBA2632971.1
Chloroflexota bacterium
VanZ-2
MBC7519195.1
Microbacteriaceae bacterium
VanZ-2
HGG14297.1
VanZ-2
HGY83062.1
VanZ-2
WP_181346815.1
Thalassobacillus sp. CUG 92003
VanZ-2
MBM3255428.1
Candidatus Omnitrophota bacterium
VanZ-2
WP_154424657.1
Bullifex porci
VanZ-2
MBY2757429.1
Clostridioides difficile
VanZ-2
MBE5967457.1
Lachnospiraceae bacterium
VanZ-2
MBR0145935.1
Eubacterium sp.
VanZ-2
WP_232695430.1
Brevibacillus daliensis
VanZ-2
WP_066229464.1
Heyndrickxia sporothermodurans
VanZ-2
MBS11834.1
Gemmatimonadota bacterium
VanZ-2
MBE5868711.1
Lachnospiraceae bacterium
VanZ-2
MBO8157431.1
Bacillaceae bacterium
VanZ-2
PKB78127.1
SAR202 cluster bacterium Io17-Chloro-G9
VanZ-2
NLK86900.1
Clostridiaceae bacterium
VanZ-2
NLD48005.1
Clostridiaceae bacterium
VanZ-2
WP_088009440.1
Indiicoccus explosivorum
VanZ-2
WP_223491359.1
Sutcliffiella horikoshii
VanZ-2
NQW10355.1
Alphaproteobacteria bacterium
VanZ-2
KAA0542759.1
Bacillus sp. BGMRC 2118
VanZ-2
MBP3706929.1
Clostridia bacterium
VanZ-2
MBP3706216.1
Clostridia bacterium
MviN_MATE-domain-flippase
VanZ-2
MBQ5902570.1
Clostridia bacterium
VanZ-2
MBQ7796031.1
Lachnospiraceae bacterium
VanZ-2
MBO7762547.1
Clostridia bacterium
VanZ-2
OPZ09135.1
candidate division BRC1 bacterium ADurb.BinA292
VanZ-2
MBE6768607.1
Oscillospiraceae bacterium
VanZ-2
MBQ1237482.1
Oscillospiraceae bacterium
VanZ-2
NLC41966.1
Erysipelothrix sp.
VanZ-2
WP_144087983.1
Allobacillus sp. GCM10007490
VanZ-2
MBQ3559371.1
Agathobacter sp.
VanZ-2
SDI35525.1
Planococcus glaciei
VanZ-2
MBE6757016.1
Oscillospiraceae bacterium
VanZ-2
MBQ8320847.1
Clostridia bacterium
VanZ-2
WP_214770439.1
Bacillus sp. ISL-101
VanZ-2
WP_154524366.1
Porcincola intestinalis
VanZ-2
MBQ1991832.1
Clostridia bacterium
VanZ-2
RHK02351.1
Acidaminococcus sp. AM05-11
VanZ-2
NCZ69445.1
Actinomycetota bacterium
VanZ-2
CAD6492160.1
Candidatus Argoarchaeum ethanivorans
VanZ-2
WP_194840799.1
Filobacillus milosensis
VanZ-2
MBO5348710.1
Clostridia bacterium
VanZ-2
MBW8797554.1
Streptomyces sp.
VanZ-2
CDE05569.1
Anaerotruncus sp. CAG:390
VanZ-2
MBQ7956335.1
Lachnospiraceae bacterium
VanZ-2
MBO5070841.1
Roseburia sp.
VanZ-2
CDE83312.1
Clostridium sp. CAG:273
VanZ-2
HCV27697.1
Dehalococcoidia bacterium
VanZ-2
MCB9840766.1
Phycisphaeraceae bacterium
VanZ-2
MBQ4282501.1
Lachnospira sp.
VanZ-2
TGA99858.1
Sporolactobacillus shoreae
VanZ-2
MBQ8201337.1
Clostridia bacterium
VanZ-2
MBR2828798.1
Bacilli bacterium
VanZ-2
MBQ4592165.1
Clostridia bacterium
VanZ-2
SDO01362.1
Klenkia soli
VanZ-2
WP_077361076.1
Clostridium saccharoperbutylacetonicum
VanZ-2
MBQ8912562.1
Lachnospiraceae bacterium
VanZ-2
CDC80992.1
Clostridium sp. CAG:964
VanZ-2

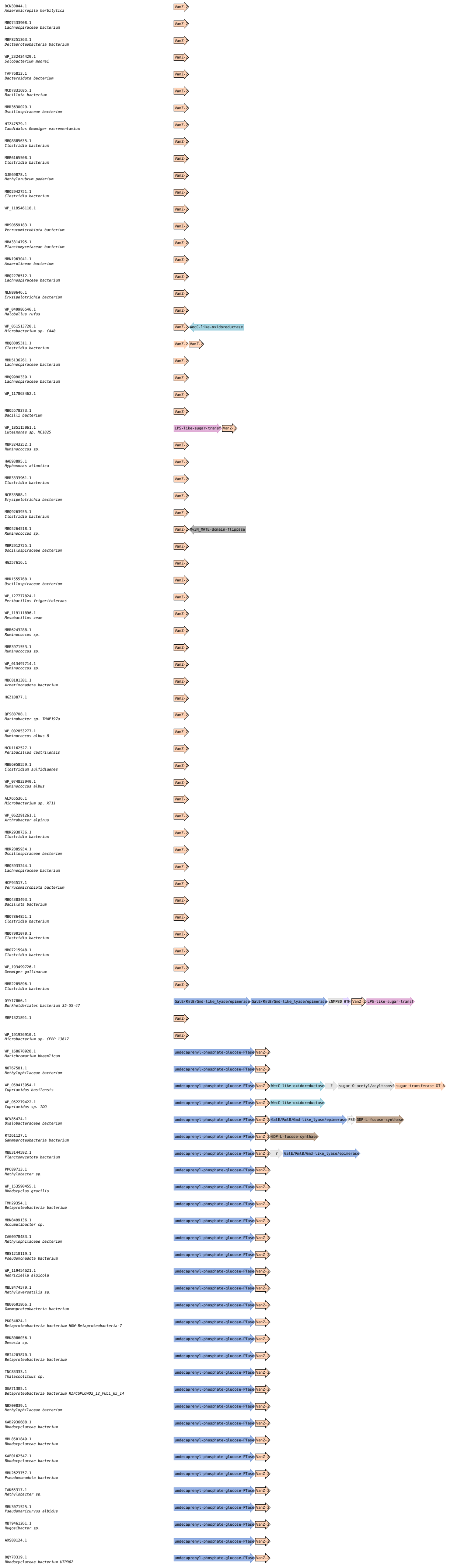
BCN30044.1
Anaeromicropila herbilytica
VanZ-2
MBQ7433908.1
Lachnospiraceae bacterium
VanZ-2
MBF8251363.1
Deltaproteobacteria bacterium
VanZ-2
WP_232424429.1
Solobacterium moorei
VanZ-2
TAF76813.1
Bacteroidota bacterium
VanZ-2
MCD7831685.1
Bacillota bacterium
VanZ-2
MBR3630029.1
Oscillospiraceae bacterium
VanZ-2
HIZ47579.1
Candidatus Gemmiger excrementavium
VanZ-2
MBQ8885635.1
Clostridia bacterium
VanZ-2
MBR6165508.1
Clostridia bacterium
VanZ-2
GJE69878.1
Methylorubrum podarium
VanZ-2
MBQ2942751.1
Clostridia bacterium
VanZ-2
WP_119546118.1
VanZ-2
MBS0659183.1
Verrucomicrobiota bacterium
VanZ-2
MBA3314795.1
Planctomycetaceae bacterium
VanZ-2
MBN1963041.1
Anaerolineae bacterium
VanZ-2
MBQ2276512.1
Lachnospiraceae bacterium
VanZ-2
NLN80646.1
Erysipelotrichia bacterium
VanZ-2
WP_049986546.1
Halobellus rufus
VanZ-2
WP_051513720.1
Microbacterium sp. C448
VanZ-2
WecC-like-oxidoreductase
MBQ8095311.1
Clostridia bacterium
VanZ-2
VanZ-2
MBD5136261.1
Lachnospiraceae bacterium
VanZ-2
MBQ9990339.1
Lachnospiraceae bacterium
VanZ-2
WP_117863462.1
VanZ-2
MBO5578273.1
Bacilli bacterium
VanZ-2
WP_185115061.1
Luteimonas sp. MC1825
LPS-like-sugar-transf
VanZ-2
MBP3243252.1
Ruminococcus sp.
VanZ-2
HAE93895.1
Hyphomonas atlantica
VanZ-2
MBR3333961.1
Clostridia bacterium
VanZ-2
NCB33588.1
Erysipelotrichia bacterium
VanZ-2
MBQ9263935.1
Clostridia bacterium
VanZ-2
MBO5264518.1
Ruminococcus sp.
VanZ-2
MviN_MATE-domain-flippase
MBR2912725.1
Oscillospiraceae bacterium
VanZ-2
HGZ57616.1
VanZ-2
MBR1555768.1
Oscillospiraceae bacterium
VanZ-2
WP_127777824.1
Peribacillus frigoritolerans
VanZ-2
WP_119111896.1
Mesobacillus zeae
VanZ-2
MBR6243288.1
Ruminococcus sp.
VanZ-2
MBR3971553.1
Ruminococcus sp.
VanZ-2
WP_013497714.1
Ruminococcus sp.
VanZ-2
MBC8101381.1
Armatimonadota bacterium
VanZ-2
HGZ10877.1
VanZ-2
QFS88708.1
Marinobacter sp. THAF197a
VanZ-2
WP_002853277.1
Ruminococcus albus 8
VanZ-2
MCD1162527.1
Peribacillus castrilensis
VanZ-2
MBE6058559.1
Clostridium sulfidigenes
VanZ-2
WP_074832940.1
Ruminococcus albus
VanZ-2
ALX65536.1
Microbacterium sp. XT11
VanZ-2
WP_062291261.1
Arthrobacter alpinus
VanZ-2
MBR2930736.1
Clostridia bacterium
VanZ-2
MBR2085934.1
Oscillospiraceae bacterium
VanZ-2
MBQ3933244.1
Lachnospiraceae bacterium
VanZ-2
HCF94517.1
Verrucomicrobiota bacterium
VanZ-2
MBQ4383493.1
Bacillota bacterium
VanZ-2
MBQ7864851.1
Clostridia bacterium
VanZ-2
MBQ7901070.1
Clostridia bacterium
VanZ-2
MBO7215948.1
Clostridia bacterium
VanZ-2
WP_193499726.1
Gemmiger gallinarum
VanZ-2
MBR2289896.1
Clostridia bacterium
VanZ-2
OYY17866.1
Burkholderiales bacterium 35-55-47
GalE/RmlB/Gmd-like_lyase/epimerase
GalE/RmlB/Gmd-like_lyase/epimerase
cNMPBD
HTH
VanZ-2
LPS-like-sugar-transf
MBP1321891.1
VanZ-2
WP_191926910.1
Microbacterium sp. CFBP 13617
VanZ-2
WP_168670928.1
Marichromatium bheemlicum
undecaprenyl-phosphate-glucose-PTase
VanZ-2
NOT67581.1
Methylophilaceae bacterium
undecaprenyl-phosphate-glucose-PTase
VanZ-2
WP_059413954.1
Cupriavidus basilensis
undecaprenyl-phosphate-glucose-PTase
VanZ-2
WecC-like-oxidoreductase
?
sugar-O-acetyl/acyltransf
sugar-transferase-GT-A
WP_052279422.1
Cupriavidus sp. IDO
undecaprenyl-phosphate-glucose-PTase
VanZ-2
WecC-like-oxidoreductase
NCV85474.1
Oxalobacteraceae bacterium
undecaprenyl-phosphate-glucose-PTase
VanZ-2
GalE/RmlB/Gmd-like_lyase/epimerase
PSE
GDP-L-fucose-synthase
RTZ61127.1
Gammaproteobacteria bacterium
undecaprenyl-phosphate-glucose-PTase
VanZ-2
GDP-L-fucose-synthase
MBE3144592.1
Planctomycetota bacterium
undecaprenyl-phosphate-glucose-PTase
VanZ-2
?
GalE/RmlB/Gmd-like_lyase/epimerase
PPC89713.1
Methylobacter sp.
undecaprenyl-phosphate-glucose-PTase
VanZ-2
WP_153590455.1
Rhodocyclus gracilis
undecaprenyl-phosphate-glucose-PTase
VanZ-2
TMH29354.1
Betaproteobacteria bacterium
undecaprenyl-phosphate-glucose-PTase
VanZ-2
MBN8499136.1
Accumulibacter sp.
undecaprenyl-phosphate-glucose-PTase
VanZ-2
CAG0978483.1
Methylophilaceae bacterium
undecaprenyl-phosphate-glucose-PTase
VanZ-2
MBS1210119.1
Pseudomonadota bacterium
undecaprenyl-phosphate-glucose-PTase
VanZ-2
WP_119454621.1
Henriciella algicola
undecaprenyl-phosphate-glucose-PTase
VanZ-2
MBL8474579.1
Methyloversatilis sp.
undecaprenyl-phosphate-glucose-PTase
VanZ-2
MBU0601866.1
Gammaproteobacteria bacterium
undecaprenyl-phosphate-glucose-PTase
VanZ-2
PKO34824.1
Betaproteobacteria bacterium HGW-Betaproteobacteria-7
undecaprenyl-phosphate-glucose-PTase
VanZ-2
MBK8086036.1
Devosia sp.
undecaprenyl-phosphate-glucose-PTase
VanZ-2
MBI4203870.1
Betaproteobacteria bacterium
undecaprenyl-phosphate-glucose-PTase
VanZ-2
TNC83333.1
Thalassolituus sp.
undecaprenyl-phosphate-glucose-PTase
VanZ-2
OGA71305.1
Betaproteobacteria bacterium RIFCSPLOWO2_12_FULL_65_14
undecaprenyl-phosphate-glucose-PTase
VanZ-2
NBX00839.1
Methylophilaceae bacterium
undecaprenyl-phosphate-glucose-PTase
VanZ-2
KAB2936688.1
Rhodocyclaceae bacterium
undecaprenyl-phosphate-glucose-PTase
VanZ-2
MBL8501849.1
Rhodocyclaceae bacterium
undecaprenyl-phosphate-glucose-PTase
VanZ-2
KAF0162547.1
Rhodocyclaceae bacterium
undecaprenyl-phosphate-glucose-PTase
VanZ-2
MBU2623757.1
Pseudomonadota bacterium
undecaprenyl-phosphate-glucose-PTase
VanZ-2
TAK65317.1
Methylobacter sp.
undecaprenyl-phosphate-glucose-PTase
VanZ-2
MBU3071525.1
Pseudomaricurvus albidus
undecaprenyl-phosphate-glucose-PTase
VanZ-2
MBT9461261.1
Rugosibacter sp.
undecaprenyl-phosphate-glucose-PTase
VanZ-2
AXS80124.1
undecaprenyl-phosphate-glucose-PTase
VanZ-2
OQY70319.1
Rhodocyclaceae bacterium UTPRO2
undecaprenyl-phosphate-glucose-PTase
VanZ-2

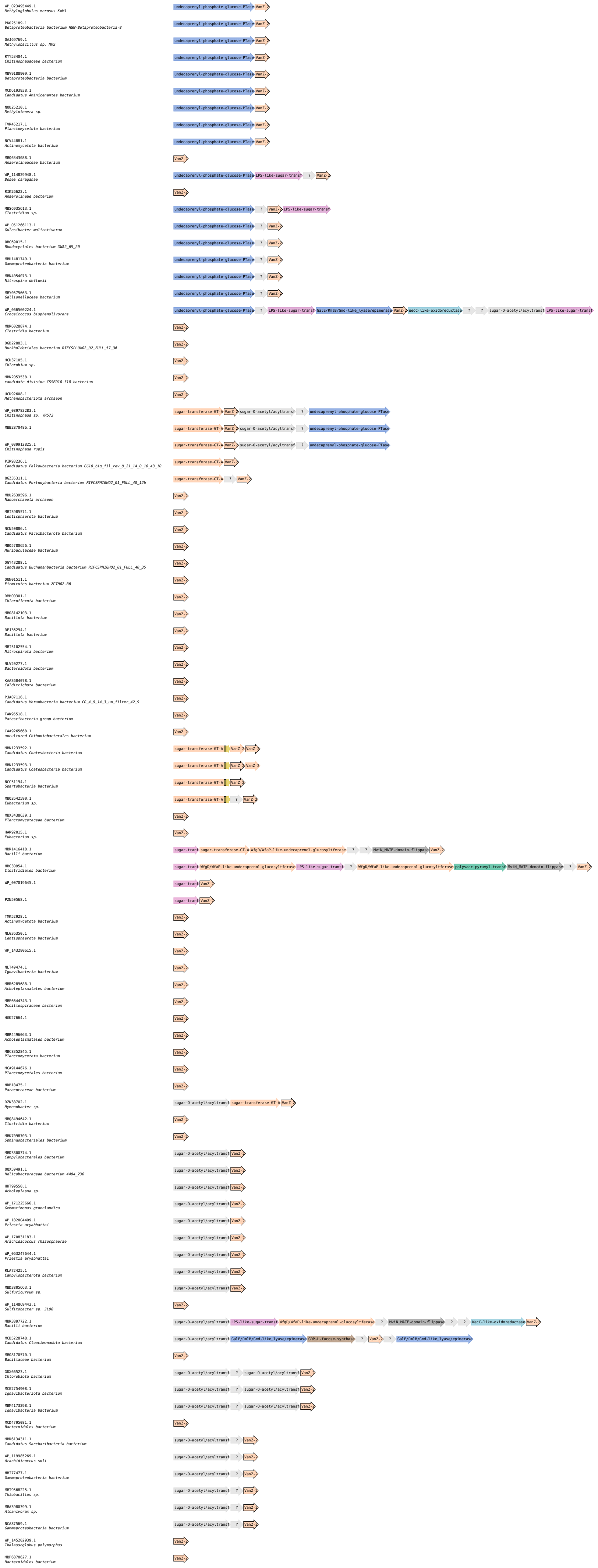
WP_023495449.1
Methyloglobulus morosus KoM1
undecaprenyl-phosphate-glucose-PTase VanZ
PKO25189.1
Betaproteobacteria bacterium HGW-Betaproteobacteria-8
undecaprenyl-phosphate-glucose-PTase VanZ
OAJ69769.1
Methylobacillus sp. MM3
undecaprenyl-phosphate-glucose-PTase VanZ
RYY53404.1
Chitinophagaceae bacterium
undecaprenyl-phosphate-glucose-PTase VanZ
MBV9188909.1
Betaproteobacteria bacterium
undecaprenyl-phosphate-glucose-PTase VanZ
MCD6193938.1
Candidatus Aminicenantes bacterium
undecaprenyl-phosphate-glucose-PTase VanZ
NOU25210.1
Methylotenera sp.
undecaprenyl-phosphate-glucose-PTase VanZ
TVR45217.1
Planctomycetota bacterium
undecaprenyl-phosphate-glucose-PTase VanZ
NCV44881.1
Actinomycetota bacterium
undecaprenyl-phosphate-glucose-PTase VanZ
MBQ6343088.1
Anaerolineaceae bacterium
VanZ
WP_114829948.1
Bosea caraganae
undecaprenyl-phosphate-glucose-PTase LPS-like-sugar-transf ? VanZ
RIK26622.1
Anaerolineae bacterium
VanZ
MBS6935613.1
Clostridium sp.
undecaprenyl-phosphate-glucose-PTase ? VanZ LPS-like-sugar-transf
WP_051266113.1
Gulosibacter molinativorax
undecaprenyl-phosphate-glucose-PTase ? VanZ
OHC69015.1
Rhodocyclales bacterium GWA2_65_20
undecaprenyl-phosphate-glucose-PTase ? VanZ
MBU1481749.1
Gammaproteobacteria bacterium
undecaprenyl-phosphate-glucose-PTase ? VanZ
MBN4054073.1
Nitrospira defluvii
undecaprenyl-phosphate-glucose-PTase ? VanZ
MBY0575663.1
Gallionellaceae bacterium
undecaprenyl-phosphate-glucose-PTase ? VanZ
WP_066560224.1
Croceicoccus bisphenolivorans
undecaprenyl-phosphate-glucose-PTase ? LPS-like-sugar-transf GalE/RmlB/Gmd-like_lyase/epimerase VanZ WecC-like-oxidoreductase ? ? sugar-O-acetyl/acyltransf LPS-like-sugar-transf
MBR6028874.1
Clostridia bacterium
VanZ
OGB22883.1
Burkholderiales bacterium RIFCSPLOWO2_02_FULL_57_36
VanZ
HCD37185.1
Chlorobium sp.
VanZ
MBN2053538.1
candidate division CSSED10-310 bacterium
VanZ
UCD92608.1
Methanobacteriota archaeon
VanZ
WP_089783283.1
Chitinophaga sp. YR573
sugar-transferase-GT-A VanZ sugar-O-acetyl/acyltransf ? undecaprenyl-phosphate-glucose-PTase
MBB2870486.1
sugar-transferase-GT-A VanZ sugar-O-acetyl/acyltransf ? undecaprenyl-phosphate-glucose-PTase
WP_089912825.1
Chitinophaga rupis
sugar-transferase-GT-A VanZ sugar-O-acetyl/acyltransf ? undecaprenyl-phosphate-glucose-PTase
PIR93236.1
Candidatus Falkowbacteria bacterium CG10_big_fil_rev_8_21_14_0_10_43_10
sugar-transferase-GT-A VanZ
OGZ35311.1
Candidatus Portnoybacteria bacterium RIFCSPHIGHO2_01_FULL_40_12b
sugar-transferase-GT-A ? VanZ
MBQ2630596.1
Nanoarchaeota archaeon
VanZ
MBI3985571.1
Lentisphaerota bacterium
VanZ
NCN50886.1
Candidatus Paceibacterota bacterium
VanZ
MBO5780656.1
Muribaculaceae bacterium
VanZ
OGY43288.1
Candidatus Buchananbacteria bacterium RIFCSPHIGHO2_01_FULL_40_35
VanZ
OUN81511.1
Firmicutes bacterium ZCTH02-B6
VanZ
RMH00301.1
Chloroflexota bacterium
VanZ
MBO8142103.1
Bacillota bacterium
VanZ
REJ36294.1
Bacillota bacterium
VanZ
MBI5102554.1
Nitrospirota bacterium
VanZ
NLV20277.1
Bacteroidota bacterium
VanZ
KAA3604078.1
Calditrichota bacterium
VanZ
PJA87116.1
Candidatus Moranbacteria bacterium CG_4_9_14_3_um_filter_42_9
VanZ
TAK95518.1
Patescibacteria group bacterium
VanZ
CAA9265668.1
uncultured Chthoniobacterales bacterium
VanZ
MBN1233592.1
Candidatus Coatesbacteria bacterium
sugar-transferase-GT-A VanZ-2 VanZ
MBN1233593.1
Candidatus Coatesbacteria bacterium
sugar-transferase-GT-A VanZ VanZ-2
NCC51194.1
Spartobacteria bacterium
sugar-transferase-GT-A VanZ
MBQ2642590.1
Eubacterium sp.
sugar-transferase-GT-A ? VanZ
MBX3438639.1
Planctomycetaceae bacterium
VanZ
HAR92015.1
Eubacterium sp.
VanZ
MBR1416418.1
Bacilli bacterium
sugar-transf sugar-transferase-GT-A WfgD/WfaP-like-undecaprenol-glucosyltferase ? ? MviN_MATE-domain-flippase VanZ
HBC30954.1
Clostridiales bacterium
sugar-transf WfgD/WfaP-like-undecaprenol-glucosyltferase LPS-like-sugar-transf ? WfgD/WfaP-like-undecaprenol-glucosyltferase polysacc-pyruvyl-transf MviN_MATE-domain-flippase ? VanZ
WP_007019645.1
sugar-transf VanZ
PZN50560.1
sugar-transf VanZ
TMK52928.1
Actinomycetota bacterium
VanZ
NLG36350.1
Lentisphaerota bacterium
VanZ
WP_143280615.1
VanZ
NLT49474.1
Ignavibacteria bacterium
VanZ
MBR6289688.1
Acholeplasmatales bacterium
VanZ
MBE6644343.1
Oscillospiraceae bacterium
VanZ
HGK27664.1
VanZ
MBR4496063.1
Acholeplasmatales bacterium
VanZ
MBC8352845.1
Planctomycetota bacterium
VanZ
MCA9144676.1
Planctomycetales bacterium
VanZ
NRB18475.1
Paracoccaceae bacterium
VanZ
RZK38702.1
Hymenobacter sp.
sugar-O-acetyl/acyltransf sugar-transferase-GT-A VanZ
MBQ8494642.1
Clostridia bacterium
VanZ
MBK7098703.1
Sphingobacteriales bacterium
VanZ
MBD3800374.1
Campylobacterales bacterium
sugar-O-acetyl/acyltransf VanZ
OQX59491.1
Helicobacteraceae bacterium 4484_230
sugar-O-acetyl/acyltransf VanZ
HHT99550.1
Acholeplasma sp.
sugar-O-acetyl/acyltransf VanZ
WP_171225666.1
Gemmatimonas groenlandica
sugar-O-acetyl/acyltransf VanZ
WP_182004409.1
Priestia aryabhattai
sugar-O-acetyl/acyltransf VanZ
WP_170831183.1
Arachidicoccus rhizosphaerae
sugar-O-acetyl/acyltransf VanZ
WP_063247644.1
Priestia aryabhattai
sugar-O-acetyl/acyltransf VanZ
RLA72425.1
Campylobacterota bacterium
sugar-O-acetyl/acyltransf VanZ
MBD3805663.1
Sulfuricurvum sp.
sugar-O-acetyl/acyltransf VanZ
WP_114869443.1
Sulfitobacter sp. JL08
VanZ
MBR3897722.1
Bacilli bacterium
sugar-O-acetyl/acyltransf LPS-like-sugar-transf WfgD/WfaP-like-undecaprenol-glucosyltferase ? MviN_MATE-domain-flippase ? ? WecC-like-oxidoreductase VanZ
MCB5228748.1
Candidatus Cloacimonadota bacterium
sugar-O-acetyl/acyltransf GalE/RmlB/Gmd-like_lyase/epimerase GDP-L-fucose-synthase ? VanZ ? GalE/RmlB/Gmd-like_lyase/epimerase
MBO8170570.1
Bacillaceae bacterium
VanZ
GDX66523.1
Chlorobiota bacterium
sugar-O-acetyl/acyltransf ? sugar-O-acetyl/acyltransf VanZ
MCE2754908.1
Ignavibacteriota bacterium
sugar-O-acetyl/acyltransf ? sugar-O-acetyl/acyltransf VanZ
MBM4173298.1
Ignavibacteria bacterium
sugar-O-acetyl/acyltransf ? sugar-O-acetyl/acyltransf VanZ
MCD4795081.1
Bacteroidales bacterium
VanZ
MBR6134311.1
Candidatus Saccharibacteria bacterium
sugar-O-acetyl/acyltransf ? VanZ
WP_119985269.1
Arachidicoccus soli
sugar-O-acetyl/acyltransf ? VanZ
HHI77477.1
Gammaproteobacteria bacterium
sugar-O-acetyl/acyltransf ? VanZ
MBT9568225.1
Thiobacillus sp.
sugar-O-acetyl/acyltransf ? VanZ
MBA3980399.1
Alcanivorax sp.
sugar-O-acetyl/acyltransf ? VanZ
NCA87569.1
Gammaproteobacteria bacterium
sugar-O-acetyl/acyltransf ? VanZ
WP_145202939.1
Thalassoglobus polymorphus
VanZ
MBP6870627.1
Bacteroidales bacterium
VanZ

EGW54253.1	*endosymbiont of Tevnia jerichonana (vent Tica)*	VanZ-2
MCB0277265.1	*Calditrichota bacterium*	VanZ-2
HAL92546.1	*Verrucomicrobiota bacterium*	VanZ-2
OIO75851.1	*Elusimicrobia bacterium CG1_02_37_114*	VanZ-2
PPD01216.1	*Methylotenera sp.*	LPS-like-sugar-transf; VanZ-2
MCC7429949.1	*bacterium*	VanZ-2; sugar-transferase-GT-A
MBD0379789.1		VanZ-2
NLX28060.1	*Bacteroidales bacterium*	VanZ-2
HGT29108.1		VanZ-2
MBS4961277.1	*Clostridiales bacterium*	VanZ-2
WP_006743928.1		VanZ-2; MurF-UDP-N-acetylmuramoyl-tripeptide--D-alanyl-D-alanine-ligase
TWJ16636.1	*Bacteroides zoogleoformans*	VanZ-2; MurF-UDP-N-acetylmuramoyl-tripeptide--D-alanyl-D-alanine-ligase
MBP3461102.1	*Bacilli bacterium*	VanZ-2
GBD41049.1	*bacterium HR39*	VanZ-2
WP_213050251.1	*Serpentinicella alkaliphila*	VanZ-2
EMG30314.1	*Campylobacter showae CC57C*	VanZ-2
NCC09942.1	*Bacteroidia bacterium*	VanZ-2; MurF-UDP-N-acetylmuramoyl-tripeptide--D-alanyl-D-alanine-ligase
MBE6287335.1	*Mediterranea massiliensis*	VanZ-2; MurF-UDP-N-acetylmuramoyl-tripeptide--D-alanyl-D-alanine-ligase
MBO7602487.1	*Bacteroidaceae bacterium*	VanZ-2; MurF-UDP-N-acetylmuramoyl-tripeptide--D-alanyl-D-alanine-ligase
MBR1542840.1	*Bacteroidaceae bacterium*	VanZ-2; MurF-UDP-N-acetylmuramoyl-tripeptide--D-alanyl-D-alanine-ligase
MBO7417814.1	*Bacteroidaceae bacterium*	VanZ-2; MurF-UDP-N-acetylmuramoyl-tripeptide--D-alanyl-D-alanine-ligase
MBQ8224050.1	*Bacteroides sp.*	VanZ-2; MurF-UDP-N-acetylmuramoyl-tripeptide--D-alanyl-D-alanine-ligase
NQW28925.1	*Ignavibacteria bacterium*	VanZ-2
NLJ08529.1	*Sphingobacteriales bacterium*	VanZ-2
KAB2894652.1	*Bacteroidota bacterium*	VanZ-2
MBK8974551.1	*Planctomycetota bacterium*	VanZ-2
WP_145343412.1	*Rosistilla ulvae*	VanZ-2
MBS1560484.1	*Bacteroidota bacterium*	VanZ-2
MCB9421270.1	*Ardenticatenaceae bacterium*	VanZ-2
MBR2043733.1	*Clostridia bacterium*	VanZ-2; UDP-N-acetylglucosamine-dehyd
MBL8151164.1	*Blastocatellia bacterium*	VanZ-2
MBK7996871.1	*Blastocatellia bacterium*	VanZ-2
MBR3383422.1	*Clostridia bacterium*	VanZ-2
MCB1264869.1	*Mycobacterium sp.*	VanZ-2
MBR6428911.1	*Clostridia bacterium*	VanZ-2
MBO4847824.1	*Clostridia bacterium*	VanZ-2
SDK31014.1	*Ferrimonas sediminum*	VanZ-2
WP_212918432.1	*Marinomonas sp. A3A*	VanZ-2
MBQ5445712.1	*Lachnospiraceae bacterium*	VanZ-2
MBE5942321.1	*Lachnospiraceae bacterium*	VanZ-2
MBQ4057517.1	*Lachnospiraceae bacterium*	VanZ-2
MBQ7574621.1	*Clostridia bacterium*	VanZ-2
WP_074732072.1	*Sharpea azabuensis*	VanZ-2
MBV9791326.1	*Chloroflexota bacterium*	VanZ-2
WP_143826237.1	*Scatolibacter rhodanostii*	VanZ-2
MBC7583694.1	*Tardiphaga sp.*	VanZ-2
MBC7580383.1	*Tardiphaga sp.*	VanZ-2
MBR0353315.1	*Oscillospiraceae bacterium*	VanZ-2
PPC74623.1	*Pokkaliibacter plantistimulans*	VanZ-2
HIT43985.1	*Candidatus Avacholeplasma faecigallinarum*	VanZ-2
MBR4332007.1	*Clostridia bacterium*	VanZ-2
MBI5095657.1	*Candidatus Hydrogenedentota bacterium*	VanZ-2
MBN1999137.1	*Candidatus Zhuqueibacterota bacterium*	VanZ-2
HEG71956.1		VanZ-2
WP_140010318.1	*Nocardioides plantarum*	VanZ-2
HBO13100.1	*Halieaceae bacterium*	VanZ-2
OGR29982.1	*Desulfuromonadales bacterium GWC2_61_20*	VanZ-2
RYD94120.1	*Sphingomonadales bacterium*	VanZ-2
MBO5375899.1	*Bacilli bacterium*	VanZ-2
MBQ2989531.1	*Clostridia bacterium*	VanZ-2
MCD8370942.1	*Clostridiales bacterium*	VanZ-2
MCC6805213.1	*Anaerolineae bacterium*	VanZ-2
MBS3166995.1	*Candidatus Woesearchaeota archaeon*	VanZ-2
UER55903.1	*Kineosporiaceae bacterium SCSIO 59966*	VanZ-2
MBQ2566427.1	*Bacteroidales bacterium*	VanZ-2
OGD83271.1	*Candidatus Collierbacteria bacterium RIFOXYD1_FULL_40_9*	VanZ-2
MBR6097216.1	*Candidatus Saccharibacteria bacterium*	VanZ-2
WP_007739258.1	*Halococcus thailandensis JCM 13552*	VanZ-2
WP_049998370.1	*Halococcus sediminicola*	VanZ-2
WP_145085224.1	*Sedimentibacter saalensis*	VanZ-2
MBS5518376.1	*Clostridiales bacterium*	VanZ-2
CAA2102422.1	*Methylobacterium bullatum*	VanZ-2
OGL87911.1	*Candidatus Uhrbacteria bacterium RIFCSPLOWO2_02_FULL_49_11*	VanZ-2
NBV13772.1	*Sphingobacteriia bacterium*	VanZ-2
MBI1742598.1	*Candidatus Acetothermia bacterium*	VanZ-2
WP_077731714.1	*Methylocaldum sp. GT1BB*	VanZ-2
MBR2455261.1	*Clostridia bacterium*	VanZ-2
QNV38797.1	*Rothia terrae*	VanZ-2
WP_205482682.1	*Rothia sp. ZJ932*	VanZ-2
HCN39715.1	*Rothia sp. (in: high G+C Gram-positive bacteria)*	VanZ-2
MCB9002483.1	*Bacteroidales bacterium*	VanZ-2
HHU80145.1	*Acholeplasmataceae bacterium*	VanZ-2
WP_013404202.1	*Caldicellulosiruptor hydrothermalis 108*	VanZ-2
MBP3648619.1	*Clostridia bacterium*	VanZ-2
HEB08872.1		VanZ-2
GBC91820.1	*bacterium HR15*	VanZ-2
WP_063975166.1	*Domibacillus aminovorans*	VanZ-2
NCC70492.1	*bacterium*	VanZ-2
MBP7080794.1	*Rhodocyclaceae bacterium*	VanZ-2
MXV92626.1	*Chloroflexota bacterium*	VanZ-2
WP_212607710.1		VanZ-2
MBB3935417.1	*Aureimonas phyllosphaerae*	VanZ-2
WP_170885600.1	*Alkalihalobacterium alkalicellulosilyticum*	VanZ-2

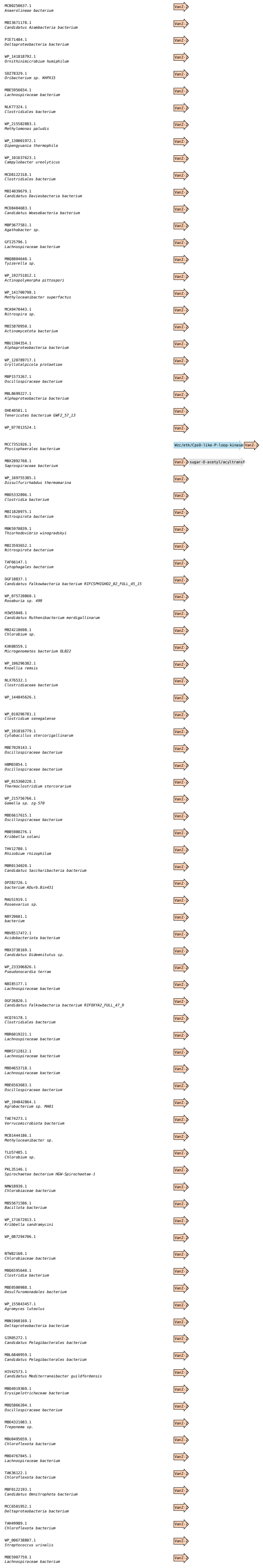
MCB0250637.1
Anaerolineae bacterium
VanZ-2
MBI3671178.1
Candidatus Azambacteria bacterium
VanZ-2
PIE71484.1
Deltaproteobacteria bacterium
VanZ-2
WP_141818792.1
Ornithinimicrobium humiphilum
VanZ-2
SDZ78329.1
Oribacterium sp. KHPX15
VanZ-2
MBE5956034.1
Lachnospiraceae bacterium
VanZ-2
NLK77324.1
Clostridiales bacterium
VanZ-2
WP_215582883.1
Methylomonas paludis
VanZ-2
WP_130001972.1
Qipengyuania thermophila
VanZ-2
WP_101637623.1
Campylobacter ureolyticus
VanZ-2
MCD8122318.1
Clostridiales bacterium
VanZ-2
MBI4039679.1
Candidatus Daviesbacteria bacterium
VanZ-2
MCD8484683.1
Candidatus Woesebacteria bacterium
VanZ-2
MBP3677581.1
Agathobacter sp.
VanZ-2
GFI25796.1
Lachnospiraceae bacterium
VanZ-2
MBQ8804640.1
Tyzzerella sp.
VanZ-2
WP_192751812.1
Actinopolymorpha pittospori
VanZ-2
WP_141700798.1
Methyloceanibacter superfactus
VanZ-2
MCA9470443.1
Nitrospira sp.
VanZ-2
MBI5870950.1
Actinomycetota bacterium
VanZ-2
MBU1304354.1
Alphaproteobacteria bacterium
VanZ-2
WP_120789717.1
Gryllotalpicola protaetiae
VanZ-2
MBP1573267.1
Oscillospiraceae bacterium
VanZ-2
MBL8699227.1
Alphaproteobacteria bacterium
VanZ-2
OHE40501.1
Tenericutes bacterium GWF2_57_13
VanZ-2
WP_077013524.1
VanZ-2
MCC7351926.1
Phycisphaerales bacterium
Wzc/etk/CpsD-like-P-loop-kinase
VanZ-2
MBX2892768.1
Saprospiraceae bacterium
VanZ-2
sugar-O-acetyl/acyltransf
WP_169755385.1
Dissulfurirhabdus thermomarina
VanZ-2
MBO5332896.1
Clostridia bacterium
VanZ-2
MBI1820975.1
Nitrospirota bacterium
VanZ-2
MBK5970839.1
Thiorhodovibrio winogradskyi
VanZ-2
MBI3593652.1
Nitrospirota bacterium
VanZ-2
TAF66147.1
Cytophagales bacterium
VanZ-2
OGF18837.1
Candidatus Falkowbacteria bacterium RIFCSPHIGHO2_02_FULL_45_15
VanZ-2
WP_075720860.1
Roseburia sp. 499
VanZ-2
HIW55048.1
Candidatus Ruthenibacterium merdigallinarum
VanZ-2
MBZ4218698.1
Chlorobium sp.
VanZ-2
KXK08559.1
Microgenomates bacterium OLB22
VanZ-2
WP_106296382.1
Knoellia remsis
VanZ-2
NLX76532.1
Clostridiaceae bacterium
VanZ-2
WP_144845626.1
VanZ-2
WP_010296781.1
Clostridium senegalense
VanZ-2
WP_191816779.1
Cytobacillus stercorigallinarum
VanZ-2
MBE7029143.1
Oscillospiraceae bacterium
VanZ-2
HBM03854.1
Oscillospiraceae bacterium
VanZ-2
WP_015360228.1
Thermoclostridium stercorarium
VanZ-2
WP_215756766.1
Gemella sp. zg-570
VanZ-2
MBE6617615.1
Oscillospiraceae bacterium
VanZ-2
MBB5980276.1
Kribbella solani
VanZ-2
THV12780.1
Rhizobium rhizophilum
VanZ-2
MBR0134020.1
Candidatus Saccharibacteria bacterium
VanZ-2
OPZ82726.1
bacterium ADurb.Bin431
VanZ-2
MAU51919.1
Roseovarius sp.
VanZ-2
NBY20601.1
bacterium
VanZ-2
MBV8517472.1
Acidobacteriota bacterium
VanZ-2
MBX3738169.1
Candidatus Didemnitutus sp.
VanZ-2
WP_233306826.1
Pseudonocardia terrae
VanZ-2
NBI85177.1
Lachnospiraceae bacterium
VanZ-2
OGF26820.1
Candidatus Falkowbacteria bacterium RIFOXYA2_FULL_47_9
VanZ-2
HCQ74178.1
Clostridiales bacterium
VanZ-2
MBR6019221.1
Lachnospiraceae bacterium
VanZ-2
MBR5712812.1
Lachnospiraceae bacterium
VanZ-2
MBO4653718.1
Lachnospiraceae bacterium
VanZ-2
MBE6563683.1
Oscillospiraceae bacterium
VanZ-2
WP_194842864.1
Agrobacterium sp. MA01
VanZ-2
TAE74273.1
Verrucomicrobiota bacterium
VanZ-2
MCB1444186.1
Methyloceanibacter sp.
VanZ-2
TLU57485.1
Chlorobium sp.
VanZ-2
PKL35146.1
Spirochaetae bacterium HGW-Spirochaetae-1
VanZ-2
NMW18930.1
Chlorobiaceae bacterium
VanZ-2
MBS5671586.1
Bacillota bacterium
VanZ-2
WP_171672013.1
Kribbella sandramycini
VanZ-2
WP_087294706.1
VanZ-2
NTW82160.1
Chlorobiaceae bacterium
VanZ-2
MBQ6595640.1
Clostridia bacterium
VanZ-2
MBE0500988.1
Desulfuromonadales bacterium
VanZ-2
WP_155843457.1
Agromyces luteolus
VanZ-2
MBN1960169.1
Deltaproteobacteria bacterium
VanZ-2
GIR05272.1
Candidatus Pelagibacterales bacterium
VanZ-2
MBL6840959.1
Candidatus Pelagibacterales bacterium
VanZ-2
HIV42573.1
Candidatus Mediterraneibacter guildfordensis
VanZ-2
MBO4919369.1
Erysipelotrichaceae bacterium
VanZ-2
MBQ5866204.1
Oscillospiraceae bacterium
VanZ-2
MBO4321083.1
Treponema sp.
VanZ-2
MBU0495659.1
Chloroflexota bacterium
VanZ-2
MBO4767045.1
Lachnospiraceae bacterium
VanZ-2
TAK36122.1
Chloroflexota bacterium
VanZ-2
MBF0122193.1
Candidatus Omnitrophota bacterium
VanZ-2
MCC6501952.1
Deltaproteobacteria bacterium
VanZ-2
TAH49989.1
Chloroflexota bacterium
VanZ-2
WP_006738807.1
Streptococcus urinalis
VanZ-2
MBE5907759.1
Lachnospiraceae bacterium
VanZ-2

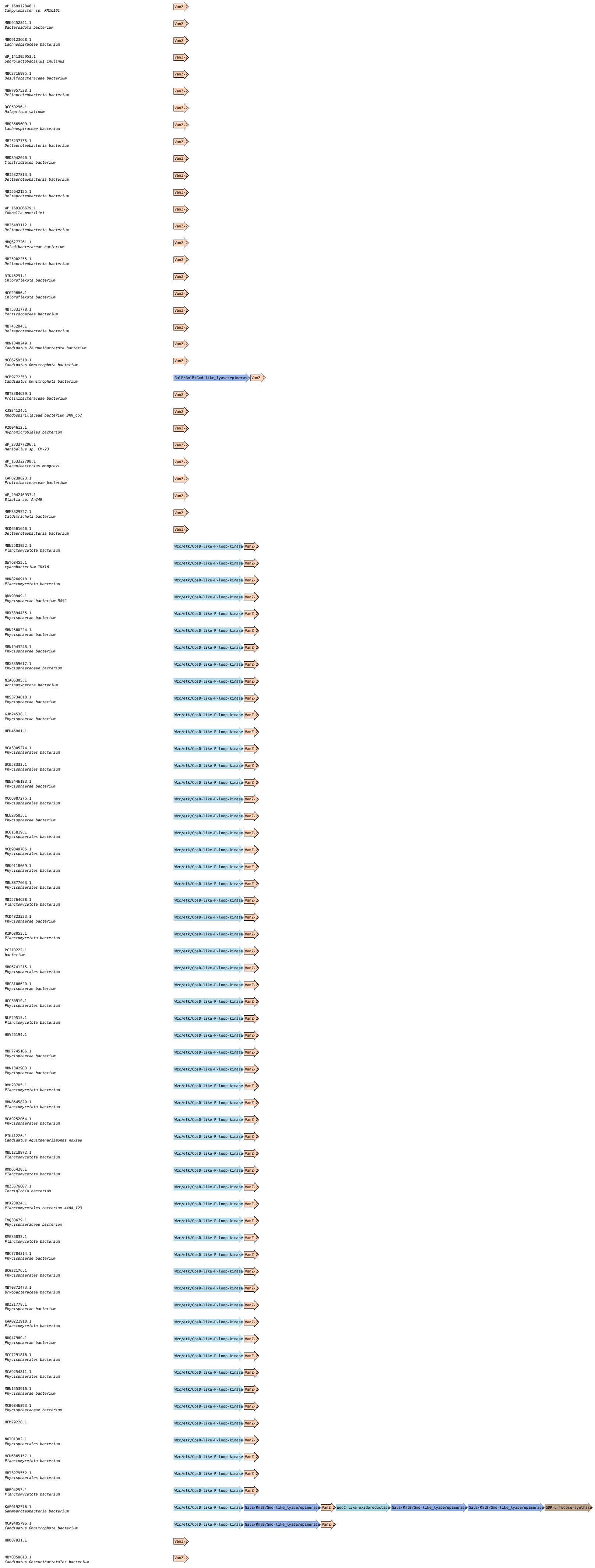
WP_169972846.1 Campylobacter sp. RM16191 VanZ-2
MBK9452841.1 Bacteroidota bacterium VanZ-2
MBQ9123668.1 Lachnospiraceae bacterium VanZ-2
WP_141305953.1 Sporolactobacillus inulinus VanZ-2
MBC2716985.1 Desulfobacteraceae bacterium VanZ-2
MBW7957528.1 Deltaproteobacteria bacterium VanZ-2
QCC50296.1 Halapricum salinum VanZ-2
MBQ3665609.1 Lachnospiraceae bacterium VanZ-2
MBI5237735.1 Deltaproteobacteria bacterium VanZ-2
MBD8942040.1 Clostridiales bacterium VanZ-2
MBI5327813.1 Deltaproteobacteria bacterium VanZ-2
MBI5642125.1 Deltaproteobacteria bacterium VanZ-2
WP_169306679.1 Cohnella pontilimi VanZ-2
MBI5493112.1 Deltaproteobacteria bacterium VanZ-2
MBQ6777261.1 Paludibacteraceae bacterium VanZ-2
MBI5902255.1 Deltaproteobacteria bacterium VanZ-2
RIK46291.1 Chloroflexota bacterium VanZ-2
HCG29666.1 Chloroflexota bacterium VanZ-2
MBT5331778.1 Porticoccaceae bacterium VanZ-2
MBT45204.1 Deltaproteobacteria bacterium VanZ-2
MBN1348249.1 Candidatus Zhuqueibacterota bacterium VanZ-2
MCC6759518.1 Candidatus Omnitrophota bacterium VanZ-2
MCB9772353.1 Candidatus Omnitrophota bacterium GalE/RmlB/Gmd-like_lyase/epimerase VanZ-2
MBT3384639.1 Prolixibacteraceae bacterium VanZ-2
KJS34124.1 Rhodospirillaceae bacterium BRH_c57 VanZ-2
PZO04612.1 Hyphomicrobiales bacterium VanZ-2
WP_233377206.1 Maribellus sp. CM-23 VanZ-2
WP_163322708.1 Draconibacterium mangrovi VanZ-2
KAF0239023.1 Prolixibacteraceae bacterium VanZ-2
WP_204246937.1 Blautia sp. An249 VanZ-2
MBM3329527.1 Calditrichota bacterium VanZ-2
MCD6561640.1 Deltaproteobacteria bacterium VanZ-2
MBN2583022.1 Planctomycetota bacterium Wzc/etk/CpsD-like-P-loop-kinase VanZ-2
OWY68455.1 cyanobacterium TDX16 Wzc/etk/CpsD-like-P-loop-kinase VanZ-2
MBK8266918.1 Planctomycetota bacterium Wzc/etk/CpsD-like-P-loop-kinase VanZ-2
QDV90949.1 Phycisphaerae bacterium RAS2 Wzc/etk/CpsD-like-P-loop-kinase VanZ-2
MBX3394435.1 Phycisphaerae bacterium Wzc/etk/CpsD-like-P-loop-kinase VanZ-2
MBN2560224.1 Phycisphaerae bacterium Wzc/etk/CpsD-like-P-loop-kinase VanZ-2
MBN1943248.1 Phycisphaerae bacterium Wzc/etk/CpsD-like-P-loop-kinase VanZ-2
MBX3359617.1 Phycisphaeraceae bacterium Wzc/etk/CpsD-like-P-loop-kinase VanZ-2
NIA06305.1 Actinomycetota bacterium Wzc/etk/CpsD-like-P-loop-kinase VanZ-2
MBS3734818.1 Phycisphaerae bacterium Wzc/etk/CpsD-like-P-loop-kinase VanZ-2
GJM24538.1 Phycisphaerae bacterium Wzc/etk/CpsD-like-P-loop-kinase VanZ-2
HEU46901.1 Wzc/etk/CpsD-like-P-loop-kinase VanZ-2
MCA3005274.1 Phycisphaerales bacterium Wzc/etk/CpsD-like-P-loop-kinase VanZ-2
UCE58333.1 Phycisphaerales bacterium Wzc/etk/CpsD-like-P-loop-kinase VanZ-2
MBN2446183.1 Phycisphaerae bacterium Wzc/etk/CpsD-like-P-loop-kinase VanZ-2
MCC6907275.1 Phycisphaerales bacterium Wzc/etk/CpsD-like-P-loop-kinase VanZ-2
NLE28583.1 Phycisphaerae bacterium Wzc/etk/CpsD-like-P-loop-kinase VanZ-2
UCG15819.1 Phycisphaerales bacterium Wzc/etk/CpsD-like-P-loop-kinase VanZ-2
MCB9849785.1 Phycisphaerales bacterium Wzc/etk/CpsD-like-P-loop-kinase VanZ-2
MBK9118669.1 Phycisphaerales bacterium Wzc/etk/CpsD-like-P-loop-kinase VanZ-2
MBL8877663.1 Phycisphaerales bacterium Wzc/etk/CpsD-like-P-loop-kinase VanZ-2
MBI5764638.1 Planctomycetota bacterium Wzc/etk/CpsD-like-P-loop-kinase VanZ-2
MCD4823323.1 Phycisphaerae bacterium Wzc/etk/CpsD-like-P-loop-kinase VanZ-2
RIK68053.1 Planctomycetota bacterium Wzc/etk/CpsD-like-P-loop-kinase VanZ-2
PCI10222.1 bacterium Wzc/etk/CpsD-like-P-loop-kinase VanZ-2
MBO6741215.1 Phycisphaerales bacterium Wzc/etk/CpsD-like-P-loop-kinase VanZ-2
MBC8106620.1 Phycisphaerae bacterium Wzc/etk/CpsD-like-P-loop-kinase VanZ-2
UCC30919.1 Phycisphaerales bacterium Wzc/etk/CpsD-like-P-loop-kinase VanZ-2
NLF29515.1 Planctomycetota bacterium Wzc/etk/CpsD-like-P-loop-kinase VanZ-2
HGV46194.1 Wzc/etk/CpsD-like-P-loop-kinase VanZ-2
MBP7745186.1 Phycisphaerae bacterium Wzc/etk/CpsD-like-P-loop-kinase VanZ-2
MBN1342903.1 Phycisphaerae bacterium Wzc/etk/CpsD-like-P-loop-kinase VanZ-2
RMH28705.1 Planctomycetota bacterium Wzc/etk/CpsD-like-P-loop-kinase VanZ-2
MBN8645829.1 Planctomycetota bacterium Wzc/etk/CpsD-like-P-loop-kinase VanZ-2
MCA9252064.1 Phycisphaerales bacterium Wzc/etk/CpsD-like-P-loop-kinase VanZ-2
PIU41226.1 Candidatus Aquitaenariimonas noxiae Wzc/etk/CpsD-like-P-loop-kinase VanZ-2
MBL1218872.1 Planctomycetota bacterium Wzc/etk/CpsD-like-P-loop-kinase VanZ-2
RMD65420.1 Planctomycetota bacterium Wzc/etk/CpsD-like-P-loop-kinase VanZ-2
MBZ5676607.1 Terriglobia bacterium Wzc/etk/CpsD-like-P-loop-kinase VanZ-2
OPX23924.1 Planctomycetales bacterium 4484_123 Wzc/etk/CpsD-like-P-loop-kinase VanZ-2
TVQ30679.1 Phycisphaeraceae bacterium Wzc/etk/CpsD-like-P-loop-kinase VanZ-2
RME36833.1 Planctomycetota bacterium Wzc/etk/CpsD-like-P-loop-kinase VanZ-2
MBC7784314.1 Phycisphaerae bacterium Wzc/etk/CpsD-like-P-loop-kinase VanZ-2
UCG32176.1 Phycisphaerales bacterium Wzc/etk/CpsD-like-P-loop-kinase VanZ-2
MBY0372473.1 Bryobacteraceae bacterium Wzc/etk/CpsD-like-P-loop-kinase VanZ-2
HDZ21778.1 Phycisphaerae bacterium Wzc/etk/CpsD-like-P-loop-kinase VanZ-2
KAA0221910.1 Planctomycetota bacterium Wzc/etk/CpsD-like-P-loop-kinase VanZ-2
NUQ47960.1 Phycisphaerae bacterium Wzc/etk/CpsD-like-P-loop-kinase VanZ-2
MCC7291816.1 Phycisphaerales bacterium Wzc/etk/CpsD-like-P-loop-kinase VanZ-2
MCA9254811.1 Phycisphaerales bacterium Wzc/etk/CpsD-like-P-loop-kinase VanZ-2
MBN1553916.1 Phycisphaerae bacterium Wzc/etk/CpsD-like-P-loop-kinase VanZ-2
MCB9846893.1 Phycisphaeraceae bacterium Wzc/etk/CpsD-like-P-loop-kinase VanZ-2
HFM79228.1 Wzc/etk/CpsD-like-P-loop-kinase VanZ-2
NOT01382.1 Phycisphaerales bacterium Wzc/etk/CpsD-like-P-loop-kinase VanZ-2
MCD6365157.1 Planctomycetota bacterium Wzc/etk/CpsD-like-P-loop-kinase VanZ-2
MBT3279552.1 Phycisphaerales bacterium Wzc/etk/CpsD-like-P-loop-kinase VanZ-2
NBB94253.1 Planctomycetota bacterium Wzc/etk/CpsD-like-P-loop-kinase VanZ-2
KAF0192576.1 Gammaproteobacteria bacterium Wzc/etk/CpsD-like-P-loop-kinase GalE/RmlB/Gmd-like_lyase/epimerase VanZ-2 WecC-like-oxidoreductase GalE/RmlB/Gmd-like_lyase/epimerase GalE/RmlB/Gmd-like_lyase/epimerase GDP-L-fucose-synthase
MCA9405796.1 Candidatus Omnitrophota bacterium Wzc/etk/CpsD-like-P-loop-kinase GalE/RmlB/Gmd-like_lyase/epimerase VanZ-2
HHO87931.1 VanZ-2
MBY0358013.1 Candidatus Obscuribacterales bacterium VanZ-2

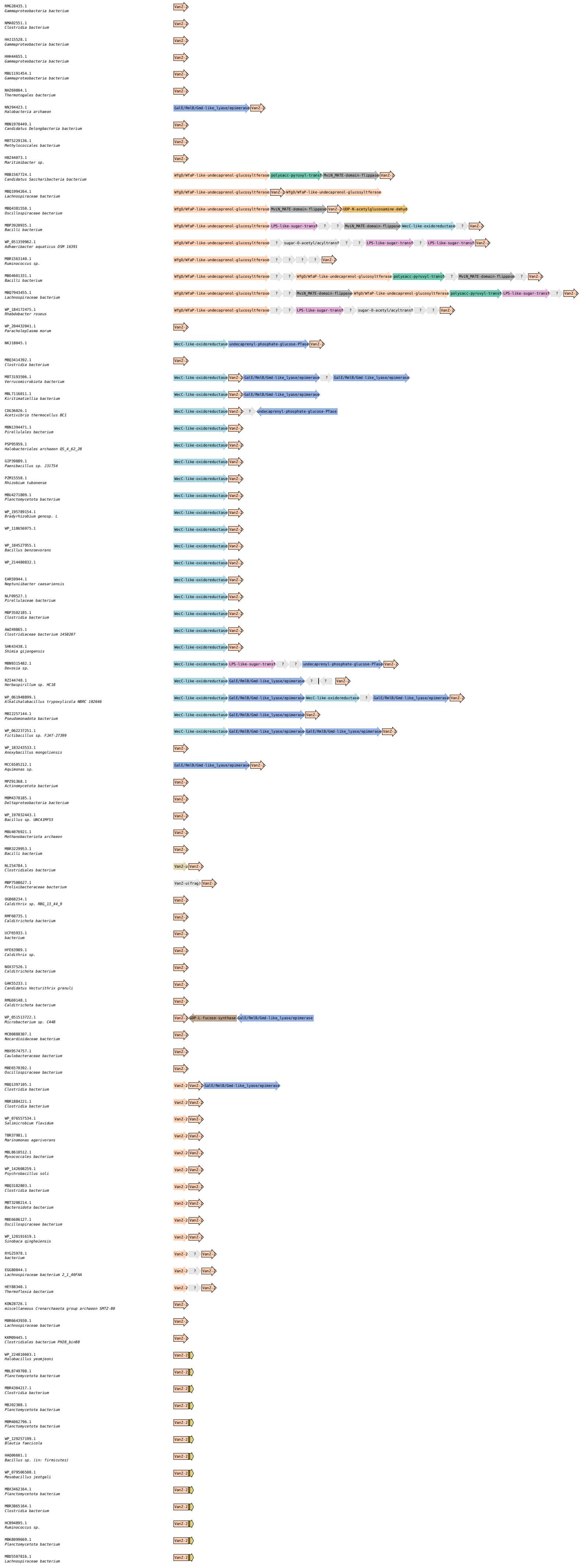
RMG28435.1
Gammaproteobacteria bacterium
VanZ
NMA82551.1
Clostridia bacterium
VanZ
HHJ15528.1
Gammaproteobacteria bacterium
VanZ
HHH44655.1
Gammaproteobacteria bacterium
VanZ
MBU1191454.1
Gammaproteobacteria bacterium
VanZ
NHZ69804.1
Thermotogales bacterium
VanZ
NNJ94423.1
Halobacteria archaeon
GalE/RmlB/Gmd-like_lyase/epimerase
VanZ
MBN1970449.1
Candidatus Delongbacteria bacterium
VanZ
MBT5229136.1
Methylococcales bacterium
VanZ
HBZ44073.1
Maritimibacter sp.
VanZ
MBB1567724.1
Candidatus Saccharibacteria bacterium
WfgD/WfaP-like-undecaprenol-glucosyltferase
polysacc-pyruvyl-transf
MviN_MATE-domain-flippase
VanZ
MBQ1994264.1
Lachnospiraceae bacterium
WfgD/WfaP-like-undecaprenol-glucosyltferase
VanZ
WfgD/WfaP-like-undecaprenol-glucosyltferase
MBQ4381550.1
Oscillospiraceae bacterium
WfgD/WfaP-like-undecaprenol-glucosyltferase
MviN_MATE-domain-flippase
VanZ
UDP-N-acetylglucosamine-dehyd
MBP3920935.1
Bacilli bacterium
WfgD/WfaP-like-undecaprenol-glucosyltferase
LPS-like-sugar-transf
?
?
MviN_MATE-domain-flippase
WecC-like-oxidoreductase
?
VanZ
WP_051359962.1
Adhaeribacter aquaticus DSM 16391
WfgD/WfaP-like-undecaprenol-glucosyltferase
?
sugar-O-acetyl/acyltransf
?
?
LPS-like-sugar-transf
?
LPS-like-sugar-transf
VanZ
MBR1563140.1
Ruminococcus sp.
WfgD/WfaP-like-undecaprenol-glucosyltferase
?
?
?
?
VanZ
MBO4601331.1
Bacilli bacterium
WfgD/WfaP-like-undecaprenol-glucosyltferase
?
?
WfgD/WfaP-like-undecaprenol-glucosyltferase
polysacc-pyruvyl-transf
?
MviN_MATE-domain-flippase
?
VanZ
MBQ7943455.1
Lachnospiraceae bacterium
WfgD/WfaP-like-undecaprenol-glucosyltferase
?
?
MviN_MATE-domain-flippase
WfgD/WfaP-like-undecaprenol-glucosyltferase
polysacc-pyruvyl-transf
LPS-like-sugar-transf
?
VanZ
WP_184172475.1
Rhabdobacter roseus
WfgD/WfaP-like-undecaprenol-glucosyltferase
?
?
LPS-like-sugar-transf
?
sugar-O-acetyl/acyltransf
?
?
VanZ
WP_204432041.1
Paracholeplasma morum
VanZ
NKJ18845.1
WecC-like-oxidoreductase
undecaprenyl-phosphate-glucose-PTase
VanZ
MBQ3414392.1
Clostridia bacterium
VanZ
MBT3193506.1
Verrucomicrobiota bacterium
WecC-like-oxidoreductase
VanZ
GalE/RmlB/Gmd-like_lyase/epimerase
?
GalE/RmlB/Gmd-like_lyase/epimerase
MBL7116011.1
Kiritimatiellia bacterium
WecC-like-oxidoreductase
VanZ
GalE/RmlB/Gmd-like_lyase/epimerase
CDG36026.1
Acetivibrio thermocellus BC1
WecC-like-oxidoreductase
VanZ
?
undecaprenyl-phosphate-glucose-PTase
MBN1394471.1
Pirellulales bacterium
WecC-like-oxidoreductase
VanZ
PSP95959.1
Halobacteriales archaeon QS_4_62_28
WecC-like-oxidoreductase
VanZ
GIP39889.1
Paenibacillus sp. J31TS4
WecC-like-oxidoreductase
VanZ
PZM15558.1
Rhizobium tubonense
WecC-like-oxidoreductase
VanZ
MBU4271809.1
Planctomycetota bacterium
WecC-like-oxidoreductase
VanZ
WP_195789154.1
Bradyrhizobium genosp. L
WecC-like-oxidoreductase
VanZ
WP_118656975.1
WecC-like-oxidoreductase
VanZ
WP_184527955.1
Bacillus benzoevorans
WecC-like-oxidoreductase
VanZ
WP_214480832.1
WecC-like-oxidoreductase
VanZ
EAR59944.1
Neptuniibacter caesariensis
WecC-like-oxidoreductase
VanZ
NLF09527.1
Pirellulaceae bacterium
WecC-like-oxidoreductase
VanZ
MBP3502185.1
Clostridia bacterium
WecC-like-oxidoreductase
VanZ
AWZ49865.1
Clostridiaceae bacterium 14S0207
WecC-like-oxidoreductase
VanZ
SHK43438.1
Shimia gijangensis
WecC-like-oxidoreductase
VanZ
MBN9315482.1
Devosia sp.
WecC-like-oxidoreductase
LPS-like-sugar-transf
?
?
undecaprenyl-phosphate-glucose-PTase
VanZ
RZI44748.1
Herbaspirillum sp. HC18
WecC-like-oxidoreductase
GalE/RmlB/Gmd-like_lyase/epimerase
?
?
VanZ
WP_061948899.1
Alkalihalobacillus trypoxylicola NBRC 102646
WecC-like-oxidoreductase
GalE/RmlB/Gmd-like_lyase/epimerase
WecC-like-oxidoreductase
?
GalE/RmlB/Gmd-like_lyase/epimerase
VanZ
MBI2257144.1
Pseudomonadota bacterium
WecC-like-oxidoreductase
GalE/RmlB/Gmd-like_lyase/epimerase
VanZ
WP_062237251.1
Fictibacillus sp. FJAT-27399
WecC-like-oxidoreductase
GalE/RmlB/Gmd-like_lyase/epimerase
GalE/RmlB/Gmd-like_lyase/epimerase
VanZ
WP_183243533.1
Anoxybacillus mongoliensis
VanZ
MCC6505212.1
Aquimonas sp.
GalE/RmlB/Gmd-like_lyase/epimerase
VanZ
MPZ91368.1
Actinomycetota bacterium
VanZ
MBM4370185.1
Deltaproteobacteria bacterium
VanZ
WP_197032443.1
Bacillus sp. UNC41MFS5
VanZ
MBU4076921.1
Methanobacteriota archaeon
VanZ
MBR3229953.1
Bacilli bacterium
VanZ
NLI54784.1
Clostridiales bacterium
VanZ-u
VanZ
MBP7508627.1
Prolixibacteraceae bacterium
VanZ-u(frag)
VanZ
OGB68234.1
Caldithrix sp. RBG_13_44_9
VanZ
RMF60735.1
Calditrichota bacterium
VanZ
UCF65933.1
bacterium
VanZ
HFE63989.1
Caldithrix sp.
VanZ
NOX37526.1
Calditrichota bacterium
VanZ
GAK55233.1
Candidatus Vecturithrix granuli
VanZ
RMG69148.1
Calditrichota bacterium
VanZ
WP_051513722.1
Microbacterium sp. C448
VanZ
GDP-L-fucose-synthase
GalE/RmlB/Gmd-like_lyase/epimerase
MCB0888307.1
Nocardioidaceae bacterium
VanZ
MBX9574757.1
Caulobacteraceae bacterium
VanZ
MBE6570392.1
Oscillospiraceae bacterium
VanZ
MBQ1397105.1
Clostridia bacterium
VanZ-2
VanZ
GalE/RmlB/Gmd-like_lyase/epimerase
MBR1884221.1
Clostridia bacterium
VanZ-2
VanZ
WP_076557534.1
Salimicrobium flavidum
VanZ-2
VanZ
TBR37981.1
Marinomonas agarivorans
VanZ-2
VanZ
MBL8610512.1
Myxococcales bacterium
VanZ-2
VanZ
WP_142608259.1
Psychrobacillus soli
VanZ-2
VanZ
MBQ3182803.1
Clostridia bacterium
VanZ-2
VanZ
MBT3208214.1
Bacteroidota bacterium
VanZ-2
VanZ
MBE6686127.1
Oscillospiraceae bacterium
VanZ-2
VanZ
WP_120191619.1
Sinobaca qinghaiensis
VanZ-2
VanZ
RYG25978.1
bacterium
VanZ-2
?
VanZ
EGG88844.1
Lachnospiraceae bacterium 2_1_46FAA
VanZ-2
?
VanZ
HEY88340.1
Thermoflexia bacterium
VanZ-2
?
VanZ
KON28726.1
miscellaneous Crenarchaeota group archaeon SMTZ-80
VanZ
MBR6643930.1
Lachnospiraceae bacterium
VanZ
KKM09445.1
Clostridiales bacterium PH28_bin88
VanZ
WP_224810603.1
Halobacillus yeomjeoni
VanZ-2
MBL8749708.1
Planctomycetota bacterium
VanZ-2
MBR4394217.1
Clostridia bacterium
VanZ-2
MBI02388.1
Planctomycetota bacterium
VanZ-2
MBM4062796.1
Planctomycetota bacterium
VanZ-2
WP_129257199.1
Blautia faecicola
VanZ-2
HAO06601.1
Bacillus sp. (in: firmicutes)
VanZ-2
WP_079506508.1
Mesobacillus jeotgali
VanZ-2
MBX3462164.1
Planctomycetota bacterium
VanZ-2
MBR3865164.1
Clostridia bacterium
VanZ-2
HCB94895.1
Ruminococcus sp.
VanZ-2
MBK8099669.1
Planctomycetota bacterium
VanZ-2
MBD5587816.1
Lachnospiraceae bacterium
VanZ-2

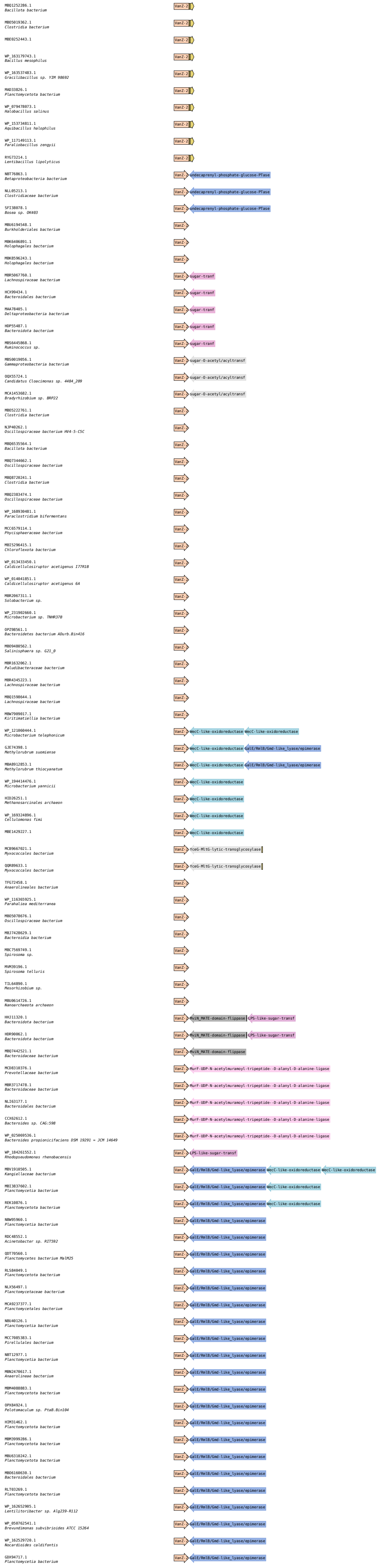
MBQ1252286.1
Bacillota bacterium
VanZ-2
MBO5019362.1
Clostridia bacterium
VanZ-2
MBE0252443.1
VanZ-2
WP_163179743.1
Bacillus mesophilus
VanZ-2
WP_163537483.1
Gracilibacillus sp. YIM 98692
VanZ-2
MAD33826.1
Planctomycetota bacterium
VanZ-2
WP_079478073.1
Halobacillus salinus
VanZ-2
WP_153734811.1
Aquibacillus halophilus
VanZ-2
WP_117149113.1
Paraliobacillus zengyii
VanZ-2
RYG73214.1
Lentibacillus lipolyticus
VanZ-2
NBT76863.1
Betaproteobacteria bacterium
VanZ-2
undecaprenyl-phosphate-glucose-PTase
NLL05213.1
Clostridiaceae bacterium
VanZ-2
undecaprenyl-phosphate-glucose-PTase
SFI38078.1
Bosea sp. OK403
VanZ-2
undecaprenyl-phosphate-glucose-PTase
MBU6194548.1
Burkholderiales bacterium
VanZ-2
MBK6406891.1
Holophagales bacterium
VanZ-2
MBK8596243.1
Holophagales bacterium
VanZ-2
MBR5067760.1
Lachnospiraceae bacterium
VanZ-2
sugar-tranf
HCX99434.1
Bacteroidales bacterium
VanZ-2
sugar-tranf
MAA78405.1
Deltaproteobacteria bacterium
VanZ-2
sugar-tranf
HDP55487.1
Bacteroidota bacterium
VanZ-2
sugar-tranf
MBS6445868.1
Ruminococcus sp.
VanZ-2
sugar-tranf
MBS0019056.1
Gammaproteobacteria bacterium
VanZ-2
sugar-O-acetyl/acyltransf
OQX55724.1
Candidatus Cloacimonas sp. 4484_209
VanZ-2
sugar-O-acetyl/acyltransf
MCA1453682.1
Bradyrhizobium sp. BRP22
VanZ-2
sugar-O-acetyl/acyltransf
MBO5222761.1
Clostridia bacterium
VanZ-2
NJP40262.1
Oscillospiraceae bacterium HV4-5-C5C
VanZ-2
MBQ6535564.1
Bacillota bacterium
VanZ-2
MBQ7344662.1
Oscillospiraceae bacterium
VanZ-2
MBQ8720241.1
Clostridia bacterium
VanZ-2
MBQ2383474.1
Oscillospiraceae bacterium
VanZ-2
WP_168930481.1
Paraclostridium bifermentans
VanZ-2
MCC6579114.1
Phycisphaeraceae bacterium
VanZ-2
MBI5296415.1
Chloroflexota bacterium
VanZ-2
WP_013433450.1
Caldicellulosiruptor acetigenus I77R1B
VanZ-2
WP_014041851.1
Caldicellulosiruptor acetigenus 6A
VanZ-2
MBR2067311.1
Solobacterium sp.
VanZ-2
WP_231902660.1
Microbacterium sp. TNHR37B
VanZ-2
OPZ98561.1
Bacteroidetes bacterium ADurb.Bin416
VanZ-2
MBO9480562.1
Salinisphaera sp. G21_0
VanZ-2
MBR1632062.1
Paludibacteraceae bacterium
VanZ-2
MBR4345223.1
Lachnospiraceae bacterium
VanZ-2
MBQ1598644.1
Lachnospiraceae bacterium
VanZ-2
MBW7909017.1
Kiritimatiellia bacterium
VanZ-2
WP_121060444.1
Microbacterium telephonicum
VanZ-2
WecC-like-oxidoreductase
WecC-like-oxidoreductase
GJE74398.1
Methylorubrum suomiense
VanZ-2
WecC-like-oxidoreductase
GalE/RmlB/Gmd-like_lyase/epimerase
MBA8912853.1
Methylorubrum thiocyanatum
VanZ-2
WecC-like-oxidoreductase
GalE/RmlB/Gmd-like_lyase/epimerase
WP_194414476.1
Microbacterium yannicii
VanZ-2
WecC-like-oxidoreductase
HID26251.1
Methanosarcinales archaeon
VanZ-2
WecC-like-oxidoreductase
WP_169324896.1
Cellulomonas fimi
VanZ-2
WecC-like-oxidoreductase
MBE1429227.1
VanZ-2
WecC-like-oxidoreductase
MCB9667021.1
Myxococcales bacterium
VanZ-2
YceG-MltG-lytic-transglycosylase
QQR89633.1
Myxococcales bacterium
VanZ-2
YceG-MltG-lytic-transglycosylase
TFG72458.1
Anaerolineales bacterium
VanZ-2
WP_116365925.1
Parahaliea mediterranea
VanZ-2
MBO5078676.1
Oscillospiraceae bacterium
VanZ-2
MBJ7428629.1
Bacteroidia bacterium
VanZ-2
MBC7569749.1
Spirosoma sp.
VanZ-2
MVM39196.1
Spirosoma telluris
VanZ-2
TIL64890.1
Mesorhizobium sp.
VanZ-2
MBU0614726.1
Nanoarchaeota archaeon
VanZ-2
HHJ11320.1
Bacteroidota bacterium
VanZ-2
MviN_MATE-domain-flippase
LPS-like-sugar-transf
HDR90062.1
Bacteroidota bacterium
VanZ-2
MviN_MATE-domain-flippase
LPS-like-sugar-transf
MBQ7442521.1
Bacteroidaceae bacterium
VanZ-2
MviN_MATE-domain-flippase
MCD8310376.1
Prevotellaceae bacterium
VanZ-2
MurF-UDP-N-acetylmuramoyl-tripeptide--D-alanyl-D-alanine-ligase
MBR3717478.1
Bacteroidaceae bacterium
VanZ-2
MurF-UDP-N-acetylmuramoyl-tripeptide--D-alanyl-D-alanine-ligase
NLI63177.1
Bacteroidales bacterium
VanZ-2
MurF-UDP-N-acetylmuramoyl-tripeptide--D-alanyl-D-alanine-ligase
CCX62612.1
Bacteroides sp. CAG:598
VanZ-2
MurF-UDP-N-acetylmuramoyl-tripeptide--D-alanyl-D-alanine-ligase
WP_025069536.1
Bacteroides propionicifaciens DSM 19291 = JCM 14649
VanZ-2
MurF-UDP-N-acetylmuramoyl-tripeptide--D-alanyl-D-alanine-ligase
WP_184261552.1
Rhodopseudomonas rhenobacensis
VanZ-2
LPS-like-sugar-transf
MBV1910505.1
Kangiellaceae bacterium
VanZ-2
GalE/RmlB/Gmd-like_lyase/epimerase
WecC-like-oxidoreductase
WecC-like-oxidoreductase
MBI3837602.1
Planctomycetia bacterium
VanZ-2
GalE/RmlB/Gmd-like_lyase/epimerase
WecC-like-oxidoreductase
REK10876.1
Planctomycetota bacterium
VanZ-2
GalE/RmlB/Gmd-like_lyase/epimerase
WecC-like-oxidoreductase
NBW95960.1
Planctomycetia bacterium
VanZ-2
GalE/RmlB/Gmd-like_lyase/epimerase
RDC48552.1
Acinetobacter sp. RIT592
VanZ-2
GalE/RmlB/Gmd-like_lyase/epimerase
QDT70560.1
Planctomycetes bacterium MalM25
VanZ-2
GalE/RmlB/Gmd-like_lyase/epimerase
RLS84049.1
Planctomycetota bacterium
VanZ-2
GalE/RmlB/Gmd-like_lyase/epimerase
NLX56497.1
Planctomycetaceae bacterium
VanZ-2
GalE/RmlB/Gmd-like_lyase/epimerase
MCA9237377.1
Planctomycetales bacterium
VanZ-2
GalE/RmlB/Gmd-like_lyase/epimerase
NBU40126.1
Planctomycetia bacterium
VanZ-2
GalE/RmlB/Gmd-like_lyase/epimerase
MCC7085383.1
Pirellulales bacterium
VanZ-2
GalE/RmlB/Gmd-like_lyase/epimerase
NBT12977.1
Planctomycetia bacterium
VanZ-2
GalE/RmlB/Gmd-like_lyase/epimerase
MBN2470617.1
Anaerolineae bacterium
VanZ-2
GalE/RmlB/Gmd-like_lyase/epimerase
MBM4088883.1
Planctomycetota bacterium
VanZ-2
GalE/RmlB/Gmd-like_lyase/epimerase
OPX84924.1
Pelotomaculum sp. PtaB.Bin104
VanZ-2
GalE/RmlB/Gmd-like_lyase/epimerase
HIM31462.1
Planctomycetota bacterium
VanZ-2
GalE/RmlB/Gmd-like_lyase/epimerase
MBM3999286.1
Planctomycetota bacterium
VanZ-2
GalE/RmlB/Gmd-like_lyase/epimerase
MBU6310242.1
Planctomycetota bacterium
VanZ-2
GalE/RmlB/Gmd-like_lyase/epimerase
MBO6160630.1
Bacteroidales bacterium
VanZ-2
GalE/RmlB/Gmd-like_lyase/epimerase
RLT03269.1
Planctomycetota bacterium
VanZ-2
GalE/RmlB/Gmd-like_lyase/epimerase
WP_162652905.1
Lentilitoribacter sp. Alg239-R112
VanZ-2
GalE/RmlB/Gmd-like_lyase/epimerase
WP_050762541.1
Brevundimonas subvibrioides ATCC 15264
VanZ-2
GalE/RmlB/Gmd-like_lyase/epimerase
WP_162529720.1
Nocardioides caldifontis
VanZ-2
GalE/RmlB/Gmd-like_lyase/epimerase
GDX94717.1
Planctomycetia bacterium
VanZ-2
GalE/RmlB/Gmd-like_lyase/epimerase

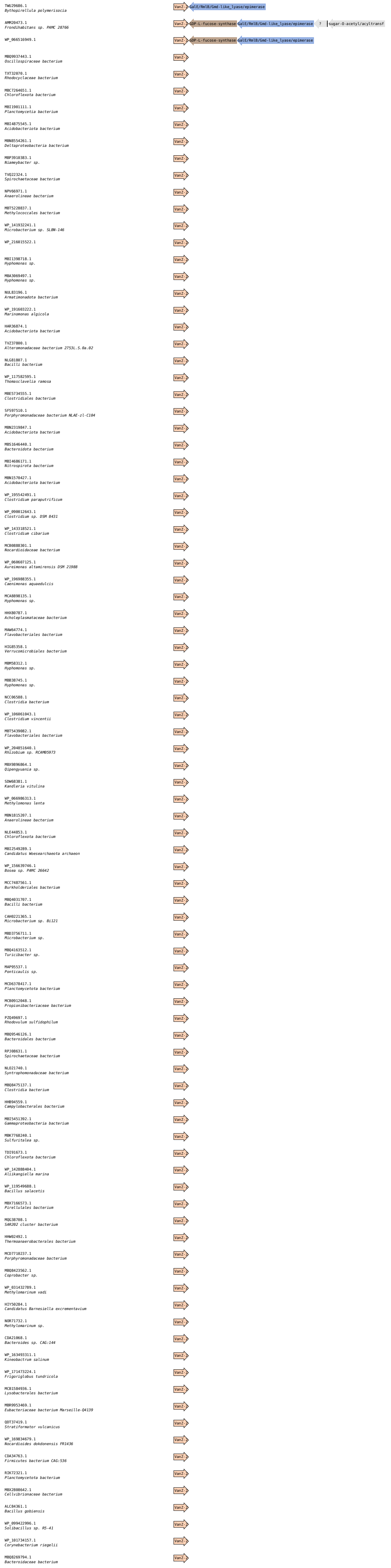

TWU29686.1
Bythopirellula polymerisocia
VanZ-2
GalE/RmlB/Gmd-like_lyase/epimerase
AMM20473.1
Frondihabitans sp. PAMC 28766
VanZ-2
GDP-L-fucose-synthase
GalE/RmlB/Gmd-like_lyase/epimerase
?
sugar-O-acetyl/acyltransf
WP_066516949.1
VanZ-2
GDP-L-fucose-synthase
GalE/RmlB/Gmd-like_lyase/epimerase
MBQ9937443.1
Oscillospiraceae bacterium
VanZ-2
TXT32070.1
Rhodocyclaceae bacterium
VanZ-2
MBC7264651.1
Chloroflexota bacterium
VanZ-2
MBI1901111.1
Planctomycetia bacterium
VanZ-2
MBI4875545.1
Acidobacteriota bacterium
VanZ-2
MBN8554261.1
Deltaproteobacteria bacterium
VanZ-2
MBP3910383.1
Niameybacter sp.
VanZ-2
TVQ22324.1
Spirochaetaceae bacterium
VanZ-2
NPV66971.1
Anaerolineae bacterium
VanZ-2
MBT5228837.1
Methylococcales bacterium
VanZ-2
WP_141932241.1
Microbacterium sp. SLBN-146
VanZ-2
WP_216015522.1
VanZ-2
MBI1398718.1
Hyphomonas sp.
VanZ-2
MBA3069497.1
Hyphomonas sp.
VanZ-2
NUL83196.1
Armatimonadota bacterium
VanZ-2
WP_191603222.1
Marinomonas algicola
VanZ-2
HAR36874.1
Acidobacteriota bacterium
VanZ-2
TVZ37800.1
Alteromonadaceae bacterium 2753L.S.0a.02
VanZ-2
NLG81807.1
Bacilli bacterium
VanZ-2
WP_117582595.1
Thomasclavelia ramosa
VanZ-2
MBE5734555.1
Clostridiales bacterium
VanZ-2
SFS97510.1
Porphyromonadaceae bacterium NLAE-zl-C104
VanZ-2
MBN2319847.1
Acidobacteriota bacterium
VanZ-2
MBS1646440.1
Bacteroidota bacterium
VanZ-2
MBI4686171.1
Nitrospirota bacterium
VanZ-2
MBN1570427.1
Acidobacteriota bacterium
VanZ-2
WP_195542491.1
Clostridium paraputrificum
VanZ-2
WP_090012643.1
Clostridium sp. DSM 8431
VanZ-2
WP_143318521.1
Clostridium cibarium
VanZ-2
MCB0888301.1
Nocardioidaceae bacterium
VanZ-2
WP_060607125.1
Aureimonas altamirensis DSM 21988
VanZ-2
WP_196988355.1
Caenimonas aquaedulcis
VanZ-2
MCA8898135.1
Hyphomonas sp.
VanZ-2
HHX80787.1
Acholeplasmataceae bacterium
VanZ-2
MAW64774.1
Flavobacteriales bacterium
VanZ-2
HIG85358.1
Verrucomicrobiales bacterium
VanZ-2
MBM58312.1
Hyphomonas sp.
VanZ-2
MBB38745.1
Hyphomonas sp.
VanZ-2
NCC06588.1
Clostridia bacterium
VanZ-2
WP_106061043.1
Clostridium vincentii
VanZ-2
MBT5439082.1
Flavobacteriales bacterium
VanZ-2
WP_204851640.1
Rhizobium sp. RCAM05973
VanZ-2
MBX9896864.1
Qipengyuania sp.
VanZ-2
SDW68381.1
Kandleria vitulina
VanZ-2
WP_066986313.1
Methylomonas lenta
VanZ-2
MBN1815207.1
Anaerolineae bacterium
VanZ-2
NLE44853.1
Chloroflexota bacterium
VanZ-2
MBI2549289.1
Candidatus Woesearchaeota archaeon
VanZ-2
WP_156639746.1
Bosea sp. PAMC 26642
VanZ-2
MCC7487561.1
Burkholderiales bacterium
VanZ-2
MBQ4031707.1
Bacilli bacterium
VanZ-2
CAH0221365.1
Microbacterium sp. Bi121
VanZ-2
MBD3756711.1
Microbacterium sp.
VanZ-2
MBQ4163512.1
Turicibacter sp.
VanZ-2
MAP95537.1
Ponticaulis sp.
VanZ-2
MCD6378417.1
Planctomycetota bacterium
VanZ-2
MCB0912048.1
Propionibacteriaceae bacterium
VanZ-2
PZQ49697.1
Rhodovulum sulfidophilum
VanZ-2
MBQ9546126.1
Bacteroidales bacterium
VanZ-2
RPJ08631.1
Spirochaetaceae bacterium
VanZ-2
NLO21740.1
Syntrophomonadaceae bacterium
VanZ-2
MBQ8475137.1
Clostridia bacterium
VanZ-2
HHB94559.1
Campylobacterales bacterium
VanZ-2
MBI5451392.1
Gammaproteobacteria bacterium
VanZ-2
MBK7768240.1
Sulfuritalea sp.
VanZ-2
TDI91673.1
Chloroflexota bacterium
VanZ-2
WP_142888404.1
Aliikangiella marina
VanZ-2
WP_119549688.1
Bacillus salacetis
VanZ-2
MBX7166573.1
Pirellulales bacterium
VanZ-2
MQG38708.1
SAR202 cluster bacterium
VanZ-2
HHW02492.1
Thermoanaerobacterales bacterium
VanZ-2
MCD7710237.1
Porphyromonadaceae bacterium
VanZ-2
MBQ8423562.1
Coprobacter sp.
VanZ-2
WP_031432789.1
Methylomarinum vadi
VanZ-2
HIY50284.1
Candidatus Barnesiella excrementavium
VanZ-2
NOR71732.1
Methylomarinum sp.
VanZ-2
CDA21068.1
Bacteroides sp. CAG:144
VanZ-2
WP_163493311.1
Kineobactrum salinum
VanZ-2
WP_171473224.1
Frigoriglobus tundricola
VanZ-2
MCB1584936.1
Lysobacterales bacterium
VanZ-2
MBR9953469.1
Eubacteriaceae bacterium Marseille-Q4139
VanZ-2
QDT37419.1
Stratiformator vulcanicus
VanZ-2
WP_169834679.1
Nocardioides dokdonensis FR1436
VanZ-2
CDA34763.1
Firmicutes bacterium CAG:536
VanZ-2
RIK72321.1
Planctomycetota bacterium
VanZ-2
MBX2808642.1
Cellvibrionaceae bacterium
VanZ-2
ALC84361.1
Bacillus gobiensis
VanZ-2
WP_099422996.1
Solibacillus sp. R5-41
VanZ-2
WP_101734157.1
Corynebacterium riegelii
VanZ-2
MBQ8269794.1
Bacteroidaceae bacterium
VanZ-2

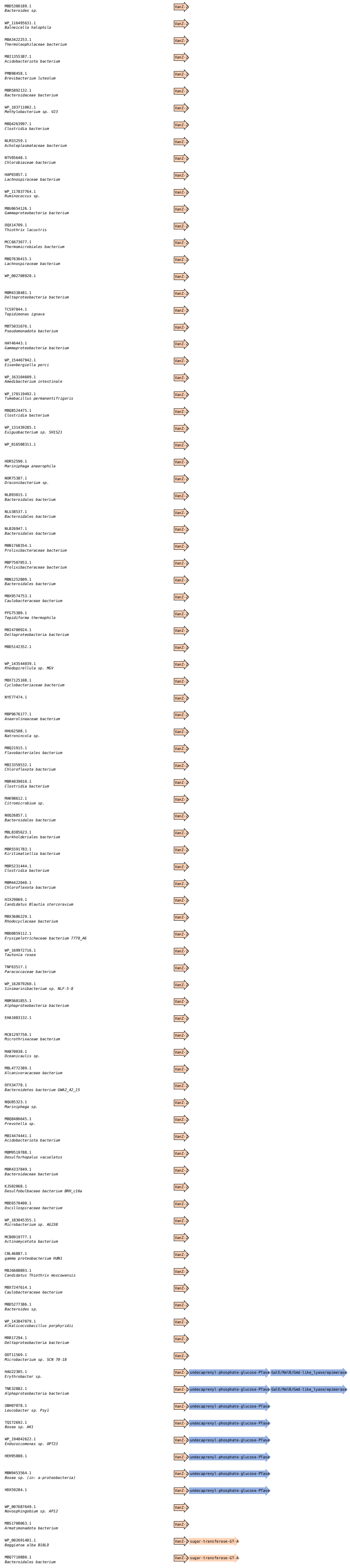

MBD5380189.1
Bacteroides sp.
VanZ-2
WP_116495631.1
Balneicella halophila
VanZ-2
MBA3422253.1
Thermoleophilaceae bacterium
VanZ-2
MBI1355387.1
Acidobacteriota bacterium
VanZ-2
PMB98458.1
Brevibacterium luteolum
VanZ-2
MBR5892132.1
Bacteroidaceae bacterium
VanZ-2
WP_103711002.1
Methylobacterium sp. V23
VanZ-2
MBQ4263997.1
Clostridia bacterium
VanZ-2
NLM33259.1
Acholeplasmataceae bacterium
VanZ-2
NTV05648.1
Chlorobiaceae bacterium
VanZ-2
HAP03857.1
Lachnospiraceae bacterium
VanZ-2
WP_117837764.1
Ruminococcus sp.
VanZ-2
MBU0654126.1
Gammaproteobacteria bacterium
VanZ-2
OQX14709.1
Thiothrix lacustris
VanZ-2
MCC6673677.1
Thermomicrobiales bacterium
VanZ-2
MBQ7636415.1
Lachnospiraceae bacterium
VanZ-2
WP_002708920.1
VanZ-2
MBM4338481.1
Deltaproteobacteria bacterium
VanZ-2
TCS97844.1
Tepidimonas ignava
VanZ-2
MBT5031676.1
Pseudomonadota bacterium
VanZ-2
HAY46443.1
Gammaproteobacteria bacterium
VanZ-2
WP_154467942.1
Eisenbergiella porci
VanZ-2
WP_163104609.1
Amedibacterium intestinale
VanZ-2
WP_170119492.1
Tumebacillus permanentifrigoris
VanZ-2
MBQ8524475.1
Clostridia bacterium
VanZ-2
WP_131439285.1
Exiguobacterium sp. SH1S21
VanZ-2
WP_016508311.1
VanZ-2
HDR52590.1
Mariniphaga anaerophila
VanZ-2
NOR75387.1
Draconibacterium sp.
VanZ-2
NLB93015.1
Bacteroidales bacterium
VanZ-2
NLU38537.1
Bacteroidales bacterium
VanZ-2
NLB26947.1
Bacteroidales bacterium
VanZ-2
MBN1768354.1
Prolixibacteraceae bacterium
VanZ-2
MBP7507053.1
Prolixibacteraceae bacterium
VanZ-2
MBN1252809.1
Bacteroidales bacterium
VanZ-2
MBX9574753.1
Caulobacteraceae bacterium
VanZ-2
PFG75380.1
Tepidiforma thermophila
VanZ-2
MBI4700924.1
Deltaproteobacteria bacterium
VanZ-2
MBD5142352.1
VanZ-2
WP_143544039.1
Rhodopirellula sp. MGV
VanZ-2
MBX7125108.1
Cyclobacteriaceae bacterium
VanZ-2
NYE77474.1
VanZ-2
MBP9676177.1
Anaerolineaceae bacterium
VanZ-2
HHU62508.1
Natronincola sp.
VanZ-2
MBQ21915.1
Flavobacteriales bacterium
VanZ-2
MBI3359532.1
Chloroflexota bacterium
VanZ-2
MBR4039010.1
Clostridia bacterium
VanZ-2
MAK98612.1
Citromicrobium sp.
VanZ-2
NOQ26857.1
Bacteroidales bacterium
VanZ-2
MBL8385623.1
Burkholderiales bacterium
VanZ-2
MBR5591783.1
Kiritimatiellia bacterium
VanZ-2
MBR5231444.1
Clostridia bacterium
VanZ-2
MBM4422040.1
Chloroflexota bacterium
VanZ-2
HIX29069.1
Candidatus Blautia stercoravium
VanZ-2
MBX3686229.1
Rhodocyclaceae bacterium
VanZ-2
MBD0859112.1
Erysipelotrichaceae bacterium 7770_A6
VanZ-2
WP_169972716.1
Tautonia rosea
VanZ-2
TNF63517.1
Paracoccaceae bacterium
VanZ-2
WP_162079260.1
Sinimarinibacterium sp. NLF-5-8
VanZ-2
MBM3601855.1
Alphaproteobacteria bacterium
VanZ-2
EHA1083132.1
VanZ-2
MCB1297750.1
Microthrixaceae bacterium
VanZ-2
MAB70038.1
Oceanicaulis sp.
VanZ-2
MBL4772389.1
Alcanivoracaceae bacterium
VanZ-2
OFX34778.1
Bacteroidetes bacterium GWA2_42_15
VanZ-2
NQU85323.1
Mariniphaga sp.
VanZ-2
MBQ8486645.1
Prevotella sp.
VanZ-2
MBI4474441.1
Acidobacteriota bacterium
VanZ-2
MBM9519788.1
Desulforhopalus vacuolatus
VanZ-2
MBR4337849.1
Bacteroidaceae bacterium
VanZ-2
KJS02068.1
Desulfobulbaceae bacterium BRH_c16a
VanZ-2
MBE6570400.1
Oscillospiraceae bacterium
VanZ-2
WP_183045355.1
Microbacterium sp. AG238
VanZ-2
MCB0919777.1
Actinomycetota bacterium
VanZ-2
CBL46887.1
gamma proteobacterium HdN1
VanZ-2
MBJ6608893.1
Candidatus Thiothrix moscowensis
VanZ-2
MBX7247614.1
Caulobacteraceae bacterium
VanZ-2
MBD5277386.1
Bacteroides sp.
VanZ-2
WP_143847079.1
Alkalicoccobacillus porphyridii
VanZ-2
MRR17294.1
Deltaproteobacteria bacterium
VanZ-2
ODT11569.1
Microbacterium sp. SCN 70-18
VanZ-2
HAU22305.1
Erythrobacter sp.
VanZ-2
undecaprenyl-phosphate-glucose-PTase
GalE/RmlB/Gmd-like_lyase/epimerase
TNE32882.1
Alphaproteobacteria bacterium
VanZ-2
undecaprenyl-phosphate-glucose-PTase
GalE/RmlB/Gmd-like_lyase/epimerase
UBH07078.1
Leucobacter sp. Psy1
VanZ-2
undecaprenyl-phosphate-glucose-PTase
TQI72692.1
Bosea sp. AK1
VanZ-2
undecaprenyl-phosphate-glucose-PTase
WP_194842622.1
Endozoicomonas sp. OPT23
VanZ-2
undecaprenyl-phosphate-glucose-PTase
HEH95880.1
VanZ-2
undecaprenyl-phosphate-glucose-PTase
MBN9453564.1
Bosea sp. (in: a-proteobacteria)
VanZ-2
undecaprenyl-phosphate-glucose-PTase
HDX59284.1
VanZ-2
undecaprenyl-phosphate-glucose-PTase
WP_007687649.1
Novosphingobium sp. AP12
VanZ-2
MBS1708063.1
Armatimonadota bacterium
VanZ-2
WP_002691401.1
Beggiatoa alba B18LD
VanZ-2
sugar-transferase-GT-A
MBQ7710880.1
Bacteroidales bacterium
VanZ-2
sugar-transferase-GT-A

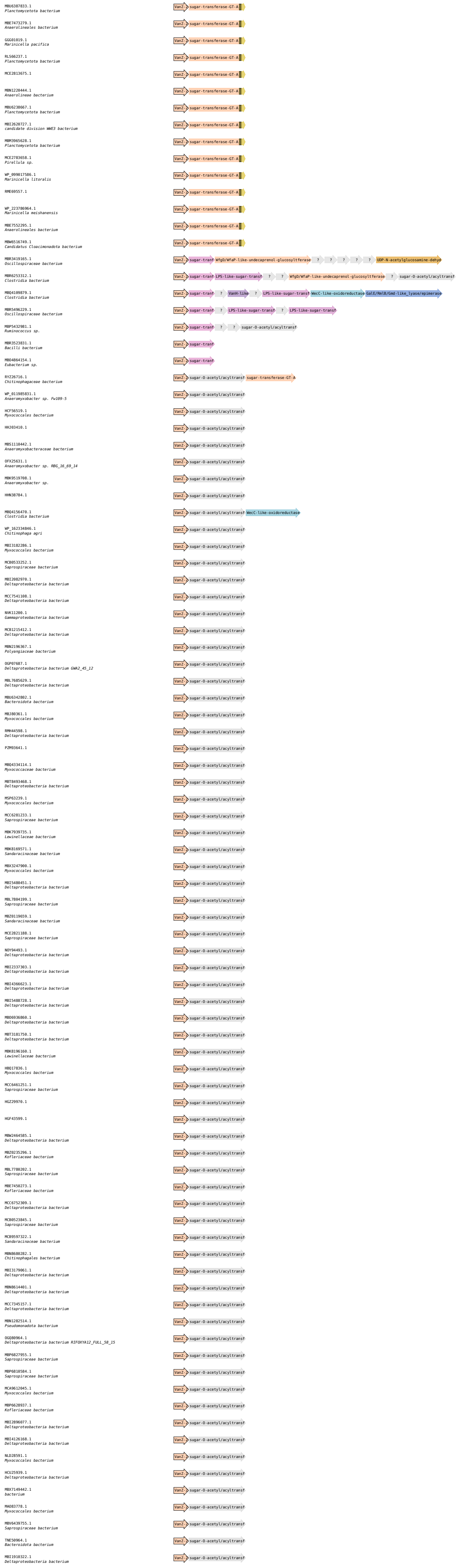

MBU6387833.1
Planctomycetota bacterium
VanZ-2 sugar-transferase-GT-A
MBE7473279.1
Anaerolineales bacterium
VanZ-2 sugar-transferase-GT-A
GGG01019.1
Marinicella pacifica
VanZ-2 sugar-transferase-GT-A
RLS66237.1
Planctomycetota bacterium
VanZ-2 sugar-transferase-GT-A
MCE2813675.1
VanZ-2 sugar-transferase-GT-A
MBN1220444.1
Anaerolineae bacterium
VanZ-2 sugar-transferase-GT-A
MBU6238667.1
Planctomycetota bacterium
VanZ-2 sugar-transferase-GT-A
MBI2620727.1
candidate division WWE3 bacterium
VanZ-2 sugar-transferase-GT-A
MBM3965628.1
Planctomycetota bacterium
VanZ-2 sugar-transferase-GT-A
MCE2783658.1
Pirellula sp.
VanZ-2 sugar-transferase-GT-A
WP_099017586.1
Marinicella litoralis
VanZ-2 sugar-transferase-GT-A
RME69557.1
VanZ-2 sugar-transferase-GT-A
WP_223786964.1
Marinicella meishanensis
VanZ-2 sugar-transferase-GT-A
MBE7552295.1
Anaerolineales bacterium
VanZ-2 sugar-transferase-GT-A
MBW6516749.1
Candidatus Cloacimonadota bacterium
VanZ-2 sugar-transferase-GT-A
MBR3419165.1
Oscillospiraceae bacterium
VanZ-2 sugar-tranf WfgD/WfaP-like-undecaprenol-glucosyltferase ? ? ? ? ? UDP-N-acetylglucosamine-dehyd
MBR6253312.1
Clostridia bacterium
VanZ-2 sugar-tranf LPS-like-sugar-transf ? ? WfgD/WfaP-like-undecaprenol-glucosyltferase ? sugar-O-acetyl/acyltransf
MBQ4109879.1
Clostridia bacterium
VanZ-2 sugar-tranf ? VanH-like ? LPS-like-sugar-transf WecC-like-oxidoreductase GalE/RmlB/Gmd-like_lyase/epimerase
MBR5496229.1
Oscillospiraceae bacterium
VanZ-2 sugar-tranf ? LPS-like-sugar-transf ? LPS-like-sugar-transf
MBP5432981.1
Ruminococcus sp.
VanZ-2 sugar-tranf ? ? sugar-O-acetyl/acyltransf
MBR3523831.1
Bacilli bacterium
VanZ-2 sugar-tranf
MBO4864154.1
Eubacterium sp.
VanZ-2 sugar-tranf
RYZ26716.1
Chitinophagaceae bacterium
VanZ-2 sugar-O-acetyl/acyltransf sugar-transferase-GT-A
WP_011985831.1
Anaeromyxobacter sp. Fw109-5
VanZ-2 sugar-O-acetyl/acyltransf
HCF56519.1
Myxococcales bacterium
VanZ-2 sugar-O-acetyl/acyltransf
HHJ03410.1
VanZ-2 sugar-O-acetyl/acyltransf
MBS1110442.1
Anaeromyxobacteraceae bacterium
VanZ-2 sugar-O-acetyl/acyltransf
OFX25631.1
Anaeromyxobacter sp. RBG_16_69_14
VanZ-2 sugar-O-acetyl/acyltransf
MBK9519708.1
Anaeromyxobacter sp.
VanZ-2 sugar-O-acetyl/acyltransf
HHN38784.1
VanZ-2 sugar-O-acetyl/acyltransf
MBQ4156470.1
Clostridia bacterium
VanZ-2 sugar-O-acetyl/acyltransf WecC-like-oxidoreductase
WP_162334846.1
Chitinophaga agri
VanZ-2 sugar-O-acetyl/acyltransf
MBI3182286.1
Myxococcales bacterium
VanZ-2 sugar-O-acetyl/acyltransf
MCB0533252.1
Saprospiraceae bacterium
VanZ-2 sugar-O-acetyl/acyltransf
MBI2082970.1
Deltaproteobacteria bacterium
VanZ-2 sugar-O-acetyl/acyltransf
MCC7541108.1
Deltaproteobacteria bacterium
VanZ-2 sugar-O-acetyl/acyltransf
NVK11200.1
Gammaproteobacteria bacterium
VanZ-2 sugar-O-acetyl/acyltransf
MCB1215412.1
Deltaproteobacteria bacterium
VanZ-2 sugar-O-acetyl/acyltransf
MBN2196367.1
Polyangiaceae bacterium
VanZ-2 sugar-O-acetyl/acyltransf
OGP07687.1
Deltaproteobacteria bacterium GWA2_45_12
VanZ-2 sugar-O-acetyl/acyltransf
MBL7685629.1
Deltaproteobacteria bacterium
VanZ-2 sugar-O-acetyl/acyltransf
MBU6342802.1
Bacteroidota bacterium
VanZ-2 sugar-O-acetyl/acyltransf
MBJ80361.1
Myxococcales bacterium
VanZ-2 sugar-O-acetyl/acyltransf
RMH44598.1
Deltaproteobacteria bacterium
VanZ-2 sugar-O-acetyl/acyltransf
PZM93641.1
VanZ-2 sugar-O-acetyl/acyltransf
MBQ4334114.1
Myxococcaceae bacterium
VanZ-2 sugar-O-acetyl/acyltransf
MBT8493468.1
Deltaproteobacteria bacterium
VanZ-2 sugar-O-acetyl/acyltransf
MSP63239.1
Myxococcales bacterium
VanZ-2 sugar-O-acetyl/acyltransf
MCC6281233.1
Saprospiraceae bacterium
VanZ-2 sugar-O-acetyl/acyltransf
MBK7939735.1
Lewinellaceae bacterium
VanZ-2 sugar-O-acetyl/acyltransf
MBK8169571.1
Sandaracinaceae bacterium
VanZ-2 sugar-O-acetyl/acyltransf
MBX3247900.1
Myxococcales bacterium
VanZ-2 sugar-O-acetyl/acyltransf
MBI5480451.1
Deltaproteobacteria bacterium
VanZ-2 sugar-O-acetyl/acyltransf
MBL7804199.1
Saprospiraceae bacterium
VanZ-2 sugar-O-acetyl/acyltransf
MBZ0119659.1
Sandaracinaceae bacterium
VanZ-2 sugar-O-acetyl/acyltransf
MCE2821188.1
Saprospiraceae bacterium
VanZ-2 sugar-O-acetyl/acyltransf
NOY94493.1
Deltaproteobacteria bacterium
VanZ-2 sugar-O-acetyl/acyltransf
MBI2337303.1
Deltaproteobacteria bacterium
VanZ-2 sugar-O-acetyl/acyltransf
MBI4366623.1
Deltaproteobacteria bacterium
VanZ-2 sugar-O-acetyl/acyltransf
MBI5488728.1
Deltaproteobacteria bacterium
VanZ-2 sugar-O-acetyl/acyltransf
MBO6936860.1
Deltaproteobacteria bacterium
VanZ-2 sugar-O-acetyl/acyltransf
MBT3181750.1
Deltaproteobacteria bacterium
VanZ-2 sugar-O-acetyl/acyltransf
MBK8196160.1
Lewinellaceae bacterium
VanZ-2 sugar-O-acetyl/acyltransf
HBQ17836.1
Myxococcales bacterium
VanZ-2 sugar-O-acetyl/acyltransf
MCC6461251.1
Saprospiraceae bacterium
VanZ-2 sugar-O-acetyl/acyltransf
HGZ29970.1
VanZ-2 sugar-O-acetyl/acyltransf
HGF43599.1
VanZ-2 sugar-O-acetyl/acyltransf
MBW2464585.1
Deltaproteobacteria bacterium
VanZ-2 sugar-O-acetyl/acyltransf
MBZ0235296.1
Kofleriaceae bacterium
VanZ-2 sugar-O-acetyl/acyltransf
MBL7780202.1
Saprospiraceae bacterium
VanZ-2 sugar-O-acetyl/acyltransf
MBE7450273.1
Kofleriaceae bacterium
VanZ-2 sugar-O-acetyl/acyltransf
MCC6752309.1
Deltaproteobacteria bacterium
VanZ-2 sugar-O-acetyl/acyltransf
MCB0523845.1
Saprospiraceae bacterium
VanZ-2 sugar-O-acetyl/acyltransf
MCB9597322.1
Sandaracinaceae bacterium
VanZ-2 sugar-O-acetyl/acyltransf
MBN8680282.1
Chitinophagales bacterium
VanZ-2 sugar-O-acetyl/acyltransf
MBI3179061.1
Deltaproteobacteria bacterium
VanZ-2 sugar-O-acetyl/acyltransf
MBN8614401.1
Deltaproteobacteria bacterium
VanZ-2 sugar-O-acetyl/acyltransf
MCC7345157.1
Deltaproteobacteria bacterium
VanZ-2 sugar-O-acetyl/acyltransf
MBN1282514.1
Pseudomonadota bacterium
VanZ-2 sugar-O-acetyl/acyltransf
OGQ80964.1
Deltaproteobacteria bacterium RIFOXYA12_FULL_58_15
VanZ-2 sugar-O-acetyl/acyltransf
MBP6827955.1
Saprospiraceae bacterium
VanZ-2 sugar-O-acetyl/acyltransf
MBP6810584.1
Saprospiraceae bacterium
VanZ-2 sugar-O-acetyl/acyltransf
MCA9612045.1
Myxococcales bacterium
VanZ-2 sugar-O-acetyl/acyltransf
MBP6628937.1
Kofleriaceae bacterium
VanZ-2 sugar-O-acetyl/acyltransf
MBI2896077.1
Deltaproteobacteria bacterium
VanZ-2 sugar-O-acetyl/acyltransf
MBI4126168.1
Deltaproteobacteria bacterium
VanZ-2 sugar-O-acetyl/acyltransf
NLD28591.1
Myxococcales bacterium
VanZ-2 sugar-O-acetyl/acyltransf
HCU25939.1
Deltaproteobacteria bacterium
VanZ-2 sugar-O-acetyl/acyltransf
MBX7149442.1
bacterium
VanZ-2 sugar-O-acetyl/acyltransf
MAO83778.1
Myxococcales bacterium
VanZ-2 sugar-O-acetyl/acyltransf
MBV6439755.1
Saprospiraceae bacterium
VanZ-2 sugar-O-acetyl/acyltransf
TNE50964.1
Bacteroidota bacterium
VanZ-2 sugar-O-acetyl/acyltransf
MBI1910322.1
Deltaproteobacteria bacterium
VanZ-2 sugar-O-acetyl/acyltransf

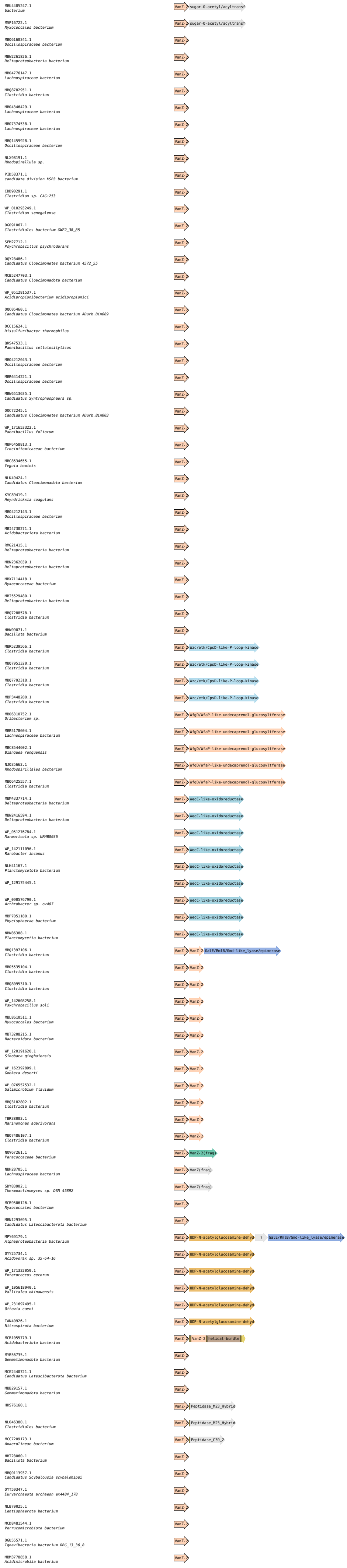
MBU4485247.1
bacterium
VanZ-2 sugar-O-acetyl/acyltransf
MSP16722.1
Myxococcales bacterium
VanZ-2 sugar-O-acetyl/acyltransf
MBQ6160341.1
Oscillospiraceae bacterium
VanZ-2
MBW2261826.1
Deltaproteobacteria bacterium
VanZ-2
MBO4776147.1
Lachnospiraceae bacterium
VanZ-2
MBQ8782951.1
Clostridia bacterium
VanZ-2
MBO4346429.1
Lachnospiraceae bacterium
VanZ-2
MBO7374538.1
Lachnospiraceae bacterium
VanZ-2
MBQ1459928.1
Oscillospiraceae bacterium
VanZ-2
NLX98191.1
Rhodopirellula sp.
VanZ-2
PID58371.1
candidate division KSB3 bacterium
VanZ-2
CDB90291.1
Clostridium sp. CAG:253
VanZ-2
WP_010293249.1
Clostridium senegalense
VanZ-2
OGO91067.1
Clostridiales bacterium GWF2_38_85
VanZ-2
SFM27712.1
Psychrobacillus psychrodurans
VanZ-2
OQY28486.1
Candidatus Cloacimonetes bacterium 4572_55
VanZ-2
MCB5247703.1
Candidatus Cloacimonadota bacterium
VanZ-2
WP_051281537.1
Acidipropionibacterium acidipropionici
VanZ-2
OQC05460.1
Candidatus Cloacimonetes bacterium ADurb.Bin089
VanZ-2
OCC15624.1
Dissulfuribacter thermophilus
VanZ-2
QKS47533.1
Paenibacillus cellulosilyticus
VanZ-2
MBO4212043.1
Oscillospiraceae bacterium
VanZ-2
MBR6414221.1
Oscillospiraceae bacterium
VanZ-2
MBW6513635.1
Candidatus Syntrophosphaera sp.
VanZ-2
OQC72245.1
Candidatus Cloacimonetes bacterium ADurb.Bin003
VanZ-2
WP_171653322.1
Paenibacillus foliorum
VanZ-2
MBP6458813.1
Crocinitomicaceae bacterium
VanZ-2
MBC8534655.1
Yeguia hominis
VanZ-2
NLK49424.1
Candidatus Cloacimonadota bacterium
VanZ-2
KYC89419.1
Heyndrickxia coagulans
VanZ-2
MBO4212143.1
Oscillospiraceae bacterium
VanZ-2
MBI4730271.1
Acidobacteriota bacterium
VanZ-2
RMG21415.1
Deltaproteobacteria bacterium
VanZ-2
MBN2362039.1
Deltaproteobacteria bacterium
VanZ-2
MBX7114418.1
Myxococcaceae bacterium
VanZ-2
MBI5529480.1
Deltaproteobacteria bacterium
VanZ-2
MBQ7288578.1
Clostridia bacterium
VanZ-2
HHW09071.1
Bacillota bacterium
VanZ-2
MBR5239566.1
Clostridia bacterium
VanZ-2 Wzc/etk/CpsD-like-P-loop-kinase
MBQ7951320.1
Clostridia bacterium
VanZ-2 Wzc/etk/CpsD-like-P-loop-kinase
MBQ7792318.1
Clostridia bacterium
VanZ-2 Wzc/etk/CpsD-like-P-loop-kinase
MBP3448280.1
Clostridia bacterium
VanZ-2 Wzc/etk/CpsD-like-P-loop-kinase
MBO6310752.1
Oribacterium sp.
VanZ-2 WfgD/WfaP-like-undecaprenol-glucosyltferase
MBR5178604.1
Lachnospiraceae bacterium
VanZ-2 WfgD/WfaP-like-undecaprenol-glucosyltferase
MBC8544602.1
Bianquea renquensis
VanZ-2 WfgD/WfaP-like-undecaprenol-glucosyltferase
NJO35662.1
Rhodospirillales bacterium
VanZ-2 WfgD/WfaP-like-undecaprenol-glucosyltferase
MBQ6425557.1
Clostridia bacterium
VanZ-2 WfgD/WfaP-like-undecaprenol-glucosyltferase
MBM4337714.1
Deltaproteobacteria bacterium
VanZ-2 WecC-like-oxidoreductase
MBW2416594.1
Deltaproteobacteria bacterium
VanZ-2 WecC-like-oxidoreductase
WP_051276784.1
Marmoricola sp. URHB0036
VanZ-2 WecC-like-oxidoreductase
WP_142111096.1
Rarobacter incanus
VanZ-2 WecC-like-oxidoreductase
NLH41167.1
Planctomycetota bacterium
VanZ-2 WecC-like-oxidoreductase
WP_129175445.1
VanZ-2 WecC-like-oxidoreductase
WP_090576790.1
Arthrobacter sp. ov407
VanZ-2 WecC-like-oxidoreductase
MBP7051180.1
Phycisphaerae bacterium
VanZ-2 WecC-like-oxidoreductase
NBW86388.1
Planctomycetia bacterium
VanZ-2 WecC-like-oxidoreductase
MBQ1397106.1
Clostridia bacterium
VanZ-2 VanZ-2 GalE/RmlB/Gmd-like_lyase/epimerase
MBO5535104.1
Clostridia bacterium
VanZ-2 VanZ-2
MBQ8095310.1
Clostridia bacterium
VanZ-2 VanZ-2
WP_142608258.1
Psychrobacillus soli
VanZ-2 VanZ-2
MBL8610511.1
Myxococcales bacterium
VanZ-2 VanZ-2
MBT3208215.1
Bacteroidota bacterium
VanZ-2 VanZ-2
WP_120191620.1
Sinobaca qinghaiensis
VanZ-2 VanZ-2
WP_162392899.1
Goekera deserti
VanZ-2 VanZ-2
WP_076557532.1
Salimicrobium flavidum
VanZ-2 VanZ-2
MBQ3182802.1
Clostridia bacterium
VanZ-2 VanZ-2
TBR38003.1
Marinomonas agarivorans
VanZ-2 VanZ-2
MBQ7486107.1
Clostridia bacterium
VanZ-2 VanZ-2
NQV67261.1
Paracoccaceae bacterium
VanZ-2 VanZ-2(frag)
NBH28705.1
Lachnospiraceae bacterium
VanZ-2 VanZ(frag)
SDY83902.1
Thermoactinomyces sp. DSM 45892
VanZ-2 VanZ(frag)
MCB9506126.1
Myxococcales bacterium
VanZ-2
MBN1293695.1
Candidatus Latescibacterota bacterium
VanZ-2
MPY69179.1
Alphaproteobacteria bacterium
VanZ-2 UDP-N-acetylglucosamine-dehyd ? GalE/RmlB/Gmd-like_lyase/epimerase
OYY25734.1
Acidovorax sp. 35-64-16
VanZ-2 UDP-N-acetylglucosamine-dehyd
WP_171332059.1
Enterococcus cecorum
VanZ-2 UDP-N-acetylglucosamine-dehyd
WP_105618940.1
Vallitalea okinawensis
VanZ-2 UDP-N-acetylglucosamine-dehyd
WP_231697495.1
Ottowia caeni
VanZ-2 UDP-N-acetylglucosamine-dehyd
TAN40926.1
Nitrospirota bacterium
VanZ-2 UDP-N-acetylglucosamine-dehyd
MCB1055779.1
Acidobacteriota bacterium
VanZ-2 VanZ-2 helical-bundle
MYB56735.1
Gemmatimonadota bacterium
VanZ-2
MCE2440721.1
Candidatus Latescibacterota bacterium
VanZ-2
MBB29157.1
Gemmatimonadota bacterium
VanZ-2
HHS76160.1
VanZ-2 Peptidase_M23_Hybrid
NLO46380.1
Clostridiales bacterium
VanZ-2 Peptidase_M23_Hybrid
MCC7209173.1
Anaerolineae bacterium
VanZ-2 Peptidase_C39_2
HHT28060.1
Bacillota bacterium
VanZ-2
MBQ0113937.1
Candidatus Scybalousia scybalohippi
VanZ-2
OYT59347.1
Euryarchaeota archaeon ex4484_178
VanZ-2
NLB70025.1
Lentisphaerota bacterium
VanZ-2
MCD8481544.1
Verrucomicrobiota bacterium
VanZ-2
OGU55571.1
Ignavibacteria bacterium RBG_13_36_8
VanZ-2
MBM3778858.1
Acidimicrobiia bacterium
VanZ-2

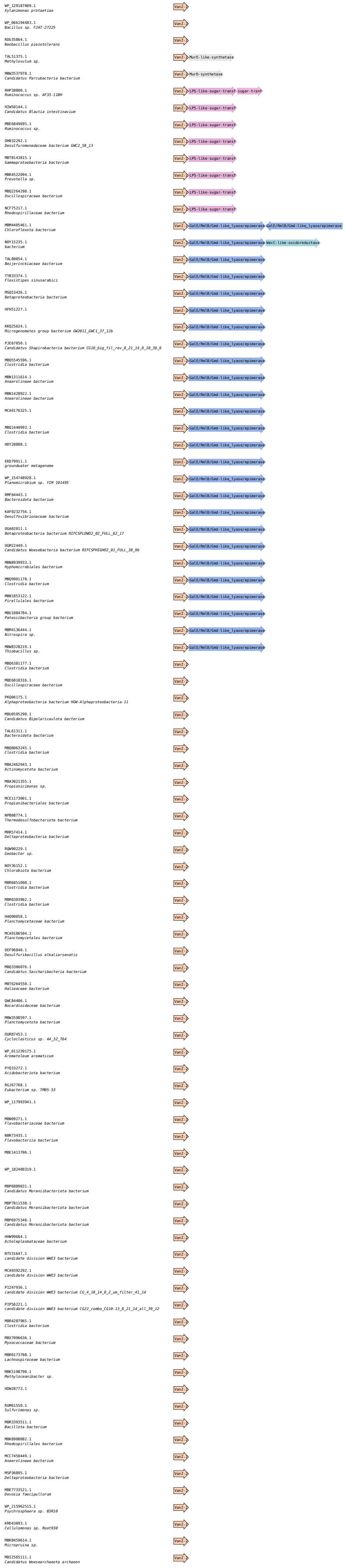

WP_129187009.1
Xylanimonas protaetiae
VanZ-2
WP_066194483.1
Bacillus sp. FJAT-27225
VanZ-2
RDU35864.1
Neobacillus piezotolerans
VanZ-2
TAL51375.1
Methylovulum sp.
VanZ-2
MurE-like-synthetase
MBW3537978.1
Candidatus Parcubacteria bacterium
VanZ-2
MurD-synthetase
RHP38800.1
Ruminococcus sp. AF33-11BH
VanZ-2
LPS-like-sugar-transf
sugar-tranf
HIW50144.1
Candidatus Blautia intestinavium
VanZ-2
LPS-like-sugar-transf
MBE6849695.1
Ruminococcus sp.
VanZ-2
LPS-like-sugar-transf
OHB32292.1
Desulfuromonadaceae bacterium GWC2_58_13
VanZ-2
LPS-like-sugar-transf
MBT8143815.1
Gammaproteobacteria bacterium
VanZ-2
LPS-like-sugar-transf
MBR4522094.1
Prevotella sp.
VanZ-2
LPS-like-sugar-transf
MBQ2264298.1
Oscillospiraceae bacterium
VanZ-2
LPS-like-sugar-transf
NCF75217.1
Rhodospirillaceae bacterium
VanZ-2
LPS-like-sugar-transf
MBM4405461.1
Chloroflexota bacterium
VanZ-2
GalE/RmlB/Gmd-like_lyase/epimerase
GalE/RmlB/Gmd-like_lyase/epimerase
NOY15235.1
bacterium
VanZ-2
GalE/RmlB/Gmd-like_lyase/epimerase
WecC-like-oxidoreductase
TAL80054.1
Beijerinckiaceae bacterium
VanZ-2
GalE/RmlB/Gmd-like_lyase/epimerase
TYB33374.1
Flexistipes sinusarabici
VanZ-2
GalE/RmlB/Gmd-like_lyase/epimerase
MSQ53436.1
Betaproteobacteria bacterium
VanZ-2
GalE/RmlB/Gmd-like_lyase/epimerase
HFH51227.1
VanZ-2
GalE/RmlB/Gmd-like_lyase/epimerase
KKQ25624.1
Microgenomates group bacterium GW2011_GWC1_37_12b
VanZ-2
GalE/RmlB/Gmd-like_lyase/epimerase
PJE67050.1
Candidatus Shapirobacteria bacterium CG10_big_fil_rev_8_21_14_0_10_36_6
VanZ-2
GalE/RmlB/Gmd-like_lyase/epimerase
MBQ5545596.1
Clostridia bacterium
VanZ-2
GalE/RmlB/Gmd-like_lyase/epimerase
MBN1311614.1
Anaerolineae bacterium
VanZ-2
GalE/RmlB/Gmd-like_lyase/epimerase
MBN1428922.1
Anaerolineae bacterium
VanZ-2
GalE/RmlB/Gmd-like_lyase/epimerase
MCA9176325.1
VanZ-2
GalE/RmlB/Gmd-like_lyase/epimerase
MBQ1440993.1
Clostridia bacterium
VanZ-2
GalE/RmlB/Gmd-like_lyase/epimerase
HDY20808.1
VanZ-2
GalE/RmlB/Gmd-like_lyase/epimerase
EKD79911.1
groundwater metagenome
VanZ-2
GalE/RmlB/Gmd-like_lyase/epimerase
WP_154748928.1
Planomicrobium sp. YIM 101495
VanZ-2
GalE/RmlB/Gmd-like_lyase/epimerase
RMF04443.1
Bacteroidota bacterium
VanZ-2
GalE/RmlB/Gmd-like_lyase/epimerase
KAF0232756.1
Desulfovibrionaceae bacterium
VanZ-2
GalE/RmlB/Gmd-like_lyase/epimerase
OGA02011.1
Betaproteobacteria bacterium RIFCSPLOWO2_02_FULL_62_17
VanZ-2
GalE/RmlB/Gmd-like_lyase/epimerase
OGM22449.1
Candidatus Woesebacteria bacterium RIFCSPHIGHO2_01_FULL_38_9b
VanZ-2
GalE/RmlB/Gmd-like_lyase/epimerase
MBN8939933.1
Hyphomicrobiales bacterium
VanZ-2
GalE/RmlB/Gmd-like_lyase/epimerase
MBQ9901178.1
Clostridia bacterium
VanZ-2
GalE/RmlB/Gmd-like_lyase/epimerase
MBN1853122.1
Pirellulales bacterium
VanZ-2
GalE/RmlB/Gmd-like_lyase/epimerase
MBU1084784.1
Patescibacteria group bacterium
VanZ-2
GalE/RmlB/Gmd-like_lyase/epimerase
MBM4136444.1
Nitrospira sp.
VanZ-2
GalE/RmlB/Gmd-like_lyase/epimerase
MBW8328219.1
Thiobacillus sp.
VanZ-2
GalE/RmlB/Gmd-like_lyase/epimerase
MBQ6381177.1
Clostridia bacterium
VanZ-2
MBE6818316.1
Oscillospiraceae bacterium
VanZ-2
PKQ06175.1
Alphaproteobacteria bacterium HGW-Alphaproteobacteria-11
VanZ-2
MBU0595298.1
Candidatus Bipolaricaulota bacterium
VanZ-2
TAL61311.1
Bacteroidota bacterium
VanZ-2
MBQ8063245.1
Clostridia bacterium
VanZ-2
MBA2462943.1
Actinomycetota bacterium
VanZ-2
MBA3021355.1
Propionicimonas sp.
VanZ-2
MCE1173001.1
Propionibacteriales bacterium
VanZ-2
NPB08774.1
Thermodesulfobacteriota bacterium
VanZ-2
MRR57414.1
Deltaproteobacteria bacterium
VanZ-2
RQW90229.1
Geobacter sp.
VanZ-2
NOY36152.1
Chlorobiota bacterium
VanZ-2
MBR6051098.1
Clostridia bacterium
VanZ-2
MBR0303962.1
Clostridia bacterium
VanZ-2
HAO00058.1
Planctomycetaceae bacterium
VanZ-2
MCA9106504.1
Planctomycetales bacterium
VanZ-2
OEF96840.1
Desulfuribacillus alkaliarsenatis
VanZ-2
MBQ3306076.1
Candidatus Saccharibacteria bacterium
VanZ-2
MBT6264550.1
Halieaceae bacterium
VanZ-2
QWC84406.1
Nocardioidaceae bacterium
VanZ-2
MBW3598597.1
Planctomycetota bacterium
VanZ-2
OUR87453.1
Cycloclasticus sp. 44_32_T64
VanZ-2
WP_011239175.1
Aromatoleum aromaticum
VanZ-2
PYQ33272.1
Acidobacteriota bacterium
VanZ-2
RGJ67768.1
Eubacterium sp. TM05-53
VanZ-2
WP_117993941.1
VanZ-2
MBN09271.1
Flavobacteriaceae bacterium
VanZ-2
NBR73435.1
Flavobacteriia bacterium
VanZ-2
MBE1413706.1
VanZ-2
WP_182400319.1
VanZ-2
MBP6889031.1
Candidatus Moraniibacteriota bacterium
VanZ-2
MBP7811538.1
Candidatus Moraniibacteriota bacterium
VanZ-2
MBP6975348.1
Candidatus Moraniibacteriota bacterium
VanZ-2
HHW99684.1
Acholeplasmataceae bacterium
VanZ-2
NTV31647.1
candidate division WWE3 bacterium
VanZ-2
MCA9392292.1
candidate division WWE3 bacterium
VanZ-2
PIZ47936.1
candidate division WWE3 bacterium CG_4_10_14_0_2_um_filter_41_14
VanZ-2
PIP56221.1
candidate division WWE3 bacterium CG22_combo_CG10-13_8_21_14_all_39_12
VanZ-2
MBR4287965.1
Clostridia bacterium
VanZ-2
MBX7096636.1
Myxococcaceae bacterium
VanZ-2
MBR0173708.1
Lachnospiraceae bacterium
VanZ-2
MBK5198790.1
Methyloceanibacter sp.
VanZ-2
HDW28773.1
VanZ-2
RUM61550.1
Sulfurimonas sp.
VanZ-2
MBR3393511.1
Bacillota bacterium
VanZ-2
MBK8908082.1
Rhodospirillales bacterium
VanZ-2
MCC7450449.1
Anaerolineae bacterium
VanZ-2
MSP36895.1
Deltaproteobacteria bacterium
VanZ-2
MBE7733521.1
Devosia faecipullorum
VanZ-2
WP_215962515.1
Psychrosphaera sp. B3R10
VanZ-2
KRD43093.1
Cellulomonas sp. Root930
VanZ-2
MBK8459614.1
Micropruina sp.
VanZ-2
MBI2565111.1
Candidatus Woesearchaeota archaeon
VanZ-2

NLM38886.1	*Bacillota bacterium*	VanZ-2
NLM54177.1	*Bacillota bacterium*	VanZ-2
KLU40130.1	*Peptococcaceae bacterium 1109*	VanZ-2
PHS27257.1	*Robiginitomaculum sp.*	VanZ-2
MBP5993301.1	*Candidatus Moraniibacteriota bacterium*	VanZ-2
MBP5476183.1	*Paludibacteraceae bacterium*	VanZ-2
MBR6355194.1	*Paludibacteraceae bacterium*	VanZ-2
NQT95495.1	*Candidatus Omnitrophota bacterium*	VanZ-2
MBE6871681.1	*Oscillospiraceae bacterium*	VanZ-2
WP_144176117.1	*Denitromonas ohlonensis*	VanZ-2
KAA3653874.1	*Pseudomonadota bacterium*	VanZ-2
WP_011872936.1	*Saccharopolyspora erythraea*	VanZ-2
WP_144353513.1	*Paenibacillus darwinianus*	VanZ-2
MBR3101508.1	*Muribaculaceae bacterium*	VanZ-2
NLX94531.1	*Clostridiales bacterium*	VanZ-2
WP_214359702.1	*Denitromonas iodatirespirans*	VanZ-2
MBO6251327.1	*Muribaculaceae bacterium*	VanZ-2
PKM50160.1	*Firmicutes bacterium HGW-Firmicutes-7*	VanZ-2
WP_161606275.1	*Microlunatus speluncae*	VanZ-2
WP_185022659.1	*Curtobacterium sp. PhB115*	VanZ-2
MBI3407876.1	*Planctomycetota bacterium*	VanZ-2
MBC9872726.1		VanZ-2
NLJ74384.1	*Bacillota bacterium*	VanZ-2
WP_075571017.1	*Salinicola socius*	VanZ-2
HHW45421.1	*Clostridiales bacterium*	VanZ-2
MBP7335419.1	*Niveispirillum sp.*	VanZ-2
WP_089219304.1		VanZ-2
MBQ8942445.1	*Bacillota bacterium*	VanZ-2
OYT42408.1	*Candidatus Aenigmarchaeota archaeon ex4484_224*	VanZ-2
MBI2094670.1	*Candidatus Omnitrophota bacterium*	VanZ-2
MBI4352838.1	*Candidatus Omnitrophota bacterium*	VanZ-2
MBN2392193.1	*Anaerolineae bacterium*	VanZ-2
OIO38580.1	*Candidatus Omnitrophica bacterium CG1_02_46_14*	VanZ-2
PHQ59489.1	*Robiginitomaculum sp.*	VanZ-2
HIR07897.1	*Candidatus Pullichristensenella stercoripullorum*	VanZ-2
MCB5230424.1	*Candidatus Cloacimonadota bacterium*	VanZ-2
MBK1832843.1	*Roseibacillus ishigakijimensis*	VanZ-2
MCD8175677.1	*Phascolarctobacterium sp.*	VanZ-2
TAM48346.1	*Acidobacteriota bacterium*	VanZ-2
MBI2213703.1	*Acidobacteriota bacterium*	VanZ-2
MBQ3215951.1	*Oscillospiraceae bacterium*	VanZ-2
MCD6450374.1		VanZ-2
MBP1620203.1	*Acidobacteriota bacterium*	VanZ-2
MBZ4656946.1	*Methermicoccus sp.*	VanZ-2
MBQ8190081.1	*Lachnospiraceae bacterium*	VanZ-2
MBP7798169.1	*Thermoanaerobaculaceae bacterium*	VanZ-2
WP_214056900.1	*Nocardioides aquaticus*	VanZ-2
MBQ3489749.1	*Clostridia bacterium*	VanZ-2
KDA53821.1	*Thermoanaerobaculum aquaticum*	VanZ-2
MBQ6702837.1	*Clostridia bacterium*	VanZ-2
MBE6533385.1	*Oscillospiraceae bacterium*	VanZ-2
MBI3136667.1	*Bacteroidota bacterium*	VanZ-2
WP_191014735.1	*Treponema zioleckii*	VanZ-2
MBN1876449.1	*Anaerolineae bacterium*	VanZ-2
NLI98989.1	*bacterium*	VanZ-2
TKJ38014.1	*candidate division TA06 bacterium B3_TA06*	VanZ-2
MBN2378394.1	*candidate division WOR-3 bacterium*	VanZ-2
WP_079544561.1	*Bradyrhizobium lablabi*	VanZ-2
MBC7550282.1	*Cellulomonas sp.*	VanZ-2
MBS3973427.1	*Actinomycetota bacterium*	VanZ-2
EGG80842.2	*Lachnospiraceae bacterium 2_1_46FAA*	VanZ-2
HEY88338.1	*Thermoflexia bacterium*	VanZ-2
RYG25979.1	*bacterium*	VanZ-2
MBN2612338.1	*Bacteroidales bacterium*	VanZ-2
MBR3431157.1	*Clostridia bacterium*	VanZ-2
MBP7957618.1	*Ottowia sp.*	VanZ-2
MCA9116954.1	*Planctomycetaceae bacterium*	VanZ-2
WP_103953511.1	*Lachnospira multipara*	VanZ-2
CDB67783.1	*Eubacterium sp. CAG:252*	VanZ-2
MBS6266231.1	*Clostridium sp.*	VanZ-2
NLK04177.1	*Clostridiales bacterium*	VanZ-2
WP_194611369.1	*Clostridium vitabionis*	VanZ-2
MBQ8279067.1	*Roseburia sp.*	VanZ-2
HAP21346.1	*Lachnospiraceae bacterium*	VanZ-2
MBS6467205.1	*Clostridium sp.*	VanZ-2
RHR47116.1	*Clostridiaceae bacterium AF18-31LB*	VanZ-2
MBC8571990.1	*Jingyaoa shaoxingensis*	VanZ-2
WP_118383140.1	*Dorea sp. AM58-8*	VanZ-2
MBQ2922643.1	*Tyzzerella sp.*	VanZ-2
WP_087161285.1	*Lachnoclostridium sp. An169*	VanZ-2
HIQ74168.1	*Candidatus Cottocaccamicrobium excrementipullorum*	VanZ-2
MCC8061279.1	*Clostridiales bacterium*	VanZ-2
HIR14612.1	*Candidatus Choladousia intestinavium*	VanZ-2
WP_099386827.1	*Agathobacter ruminis*	VanZ-2
HBN56597.1	*Lachnospiraceae bacterium*	VanZ-2
MBQ6843564.1	*Agathobacter sp.*	VanZ-2
MBQ4559519.1	*Tyzzerella sp.*	VanZ-2
MBQ6812851.1	*Agathobacter sp.*	VanZ-2
MBO5245701.1	*Eubacterium sp.*	VanZ-2
MBO5239756.1	*Lachnospiraceae bacterium*	VanZ-2
WP_013976467.1	*Clostridium sp. SY8519*	VanZ-2
MBQ2287269.1	*Lachnospiraceae bacterium*	VanZ-2
MBS6680147.1	*Roseburia sp.*	VanZ-2

Accession	Organism	Gene
HIZ08674.1	*Candidatus Eubacterium avistercoris*	VanZ
MBQ4231821.1	*Lachnospiraceae bacterium*	VanZ
MBP7320632.1	*Lachnospiraceae bacterium*	VanZ
MBQ9764714.1	*Lachnospiraceae bacterium*	VanZ
HIW41895.1	*Candidatus Mediterraneibacter vanvlietii*	VanZ
HIR87711.1	*Candidatus Fimimorpha faecalis*	VanZ
MBQ2577216.1	*Lachnospiraceae bacterium*	VanZ
HCS04625.1	*Eubacterium sp.*	VanZ
WP_087154964.1		VanZ
MBS6914385.1	*Clostridium sp.*	VanZ
MBR3646268.1	*Lachnospiraceae bacterium*	VanZ
RHQ42176.1	*Roseburia sp. AF25-25LB*	VanZ
CDA14607.1	*Firmicutes bacterium CAG:212*	VanZ
SDW63671.1	*Lachnospiraceae bacterium KHCPX20*	VanZ
MBO8463860.1	*Candidatus Scybalomonas excrementavium*	VanZ
SFQ32906.1	*Lachnospiraceae bacterium XBB1006*	VanZ
HJA12426.1	*Candidatus Mediterraneibacter merdipullorum*	VanZ
HIT06117.1	*Candidatus Scybalocola faecipullorum*	VanZ
MBO5155227.1	*Eubacterium sp.*	VanZ
MBQ8306879.1	*Blautia sp.*	VanZ
MBQ9119867.1	*Lachnospiraceae bacterium*	VanZ
MBQ8797962.1	*Lachnospiraceae bacterium*	VanZ
WP_120408549.1		VanZ
WP_006780903.1	*Hungatella hathewayi*	VanZ
MBO5159539.1	*Lachnospiraceae bacterium*	VanZ
WP_006862798.1	*Marvinbryantia formatexigens*	VanZ
CCY87303.1	*Clostridium sp. CAG:149*	VanZ
MBE5962480.1	*Lachnospiraceae bacterium*	VanZ
MTK10418.1	*Hungatella sp.*	VanZ
MBR6536886.1	*Lachnospiraceae bacterium*	VanZ
MBS6642559.1	*Clostridiaceae bacterium*	VanZ
MBQ7535603.1	*Stomatobaculum sp.*	VanZ
OKZ91683.1	*Coprococcus sp. CAG:131_42_139*	VanZ
SFN82571.1	*Anaerocolumna aminovalerica*	VanZ
WP_118316179.1		VanZ
EOS34232.1	*Lachnospiraceae bacterium M18-1*	VanZ
WP_005541617.1		VanZ
MBP5490969.1	*Lachnospiraceae bacterium*	VanZ
WP_009263585.1	*Faecalimonas umbilicata*	VanZ
WP_117970038.1		VanZ
MBQ3406055.1	*Lachnospiraceae bacterium*	VanZ
MBO4997131.1	*Lachnospira sp.*	VanZ
MBO5279333.1	*Lachnospiraceae bacterium*	VanZ
MBO6106058.1	*Stomatobaculum sp.*	VanZ
WP_074716841.1	*Lachnobacterium bovis DSM 14045*	VanZ
MBA4688418.1	*Candidatus Galacturonibacter soehngenii*	VanZ
WP_007593503.1	*uncultured Lachnoanaerobaculum sp.*	VanZ
MBQ7507042.1	*Lachnospiraceae bacterium*	VanZ
MBQ4284047.1	*Lachnospira sp.*	VanZ
MBE5902121.1	*Lachnospiraceae bacterium*	VanZ
CUQ77596.1	*Lachnospira eligens*	VanZ
MBS7060556.1	*Eubacterium sp.*	VanZ
CCY69395.1	*Eubacterium sp. CAG:161*	VanZ
MBR9938318.1	*Oscillospiraceae bacterium Marseille-Q3528*	VanZ
MBQ8281031.1	*Lachnospiraceae bacterium*	VanZ
HIR76095.1	*Candidatus Choladousia intestinipullorum*	VanZ
WP_078767151.1	*Eubacterium uniforme*	VanZ
HJA19169.1	*Candidatus Mediterraneibacter ornithocaccae*	VanZ
HJB17738.1	*Candidatus Bariatricus faecipullorum*	VanZ
HIZ13557.1	*Candidatus Mediterraneibacter stercorigallinarum*	VanZ
MBE5887630.1	*Lachnospiraceae bacterium*	VanZ
MBR3154211.1	*Lachnospiraceae bacterium*	VanZ
MCC8141903.1	*Lachnospiraceae bacterium*	VanZ
EEC58251.1	*[Bacteroides] pectinophilus ATCC 43243*	VanZ
WP_074650225.1	*[Clostridium] aminophilum*	VanZ
MBR6230741.1	*Lachnospiraceae bacterium*	VanZ
BCN29832.1	*Anaeromicropila herbilytica*	VanZ
WP_066549846.1	*uncultured Enterocloster sp.*	VanZ
MBO5261365.1	*Coprococcus sp.*	VanZ
MBQ6442591.1	*Lachnospiraceae bacterium*	VanZ
MCC8018036.1	*Lachnospiraceae bacterium*	VanZ
WP_118555821.1		VanZ
HBD66300.1	*Eubacterium sp.*	VanZ
PNH20660.1	*Lachnospiraceae bacterium*	VanZ
WP_031557603.1	*Lachnospira multipara LB2003*	VanZ
MBO4787555.1	*Lachnospiraceae bacterium*	VanZ
HJC06311.1	*Candidatus Enterocloster excrementipullorum*	VanZ
MBN1526517.1	*Candidatus Omnitrophota bacterium*	VanZ
MCA1651279.1	*Acidobacteriota bacterium*	VanZ
ERI76600.1	*[Clostridium] symbiosum ATCC 14940*	VanZ
MBQ0035017.1	*Candidatus Colicola faecequi*	VanZ
MBU4184975.1	*Pseudomonadota bacterium*	VanZ
WP_088572176.1	*Thermoflexus hugenholtzii JAD2*	VanZ
MBN8590878.1	*Anaerolineae bacterium*	VanZ
MBR6190939.1	*Prevotella sp.*	VanZ
TSA28157.1	*bacterium*	VanZ
MBS7223178.1	*Megasphaera sp.*	VanZ
OGM88994.1	*Candidatus Woesebacteria bacterium RIFOXYD1_FULL_46_19*	VanZ
MBR3646225.1	*Lachnospiraceae bacterium*	VanZ
TCU91106.1	*Roseateles saccharophilus*	VanZ
RNC67583.1	*Desulfuromonadales bacterium*	VanZ
WP_214176080.1	*Geomobilimonas luticola*	VanZ
HIT80103.1	*Candidatus Faecivivens stercorigallinarum*	VanZ

MBP7928233.1	*Patescibacteria group bacterium*	VanZ-2
MBL0142039.1	*Betaproteobacteria bacterium*	VanZ-2
MBR5651046.1	*Bacteroidales bacterium*	VanZ-2
MBR6173450.1	*Eubacterium sp.*	VanZ-2
WP_008310957.1	*Haloarcula sp. CGMCC 1.6347*	VanZ-2
MBR1882593.1	*Muribaculaceae bacterium*	VanZ-2
WP_194697428.1	*Nocardioides agariphilus*	VanZ-2
WP_183815881.1	*Parvularcula dongshanensis*	VanZ-2
MBU1203787.1	*Nanoarchaeota archaeon*	VanZ-2
RTL33872.1	*Burkholderiales bacterium*	VanZ-2
MBP0969952.1	*Oscillospiraceae bacterium*	VanZ-2
MBR5065464.1	*Oscillospiraceae bacterium*	VanZ-2
WP_004593891.1	*Haloarcula japonica*	VanZ-2
WP_155475214.1	*Heliobacterium mobile*	VanZ-2
WP_051938248.1	*Ghiorsea bivora*	VanZ-2
MCD6591072.1	*Candidatus Aenigmatarchaeota archaeon*	VanZ-2
WP_153169855.1	*Thauera sp. 2A1*	VanZ-2
UCC93792.1	*Thermoplasmata archaeon*	VanZ-2
WP_101349237.1	*Haloarcula sp. Atlit-47R*	VanZ-2
OYV23352.1	*Methylococcaceae bacterium NSO1*	VanZ-2
MBU2569886.1	*Gammaproteobacteria bacterium*	VanZ-2
PKM38116.1	*Gammaproteobacteria bacterium HGW-Gammaproteobacteria-10*	VanZ-2
MBX2903299.1	*Chitinophagales bacterium*	VanZ-2
NLV68001.1	*Spirochaetota bacterium*	VanZ-2
MBN9062890.1	*Hyphomicrobiales bacterium*	VanZ-2
MBU1782773.1	*bacterium*	VanZ-2
MBE6048957.1	*Clostridium sp.*	VanZ-2
HDD05194.1	*Candidatus Aenigmatarchaeota archaeon*	VanZ-2
MBR6903195.1	*Clostridia bacterium*	VanZ-2
MBF0484698.1	*Candidatus Omnitrophota bacterium*	VanZ-2
PIP19864.1	*Candidatus Sherwoodlollariibacterium unditelluris*	VanZ-2
OGX37703.1	*Omnitrophica WOR_2 bacterium RIFCSPHIGHO2_02_FULL_50_17*	VanZ-2
MBN1585979.1	*Candidatus Omnitrophota bacterium*	VanZ-2
RJP29054.1	*Candidatus Omnitrophota bacterium*	VanZ-2
NLX80054.1	*Proteiniphilum sp.*	VanZ-2
QAT16764.1	*Candidatus Velamenicoccus archaeovorus*	VanZ-2
MBN2330627.1	*Candidatus Aenigmatarchaeota archaeon*	VanZ-2
TRZ96143.1	*bacterium*	VanZ-2
MBF0531558.1	*Candidatus Omnitrophota bacterium*	VanZ-2
MBR2339112.1	*Clostridia bacterium*	VanZ-2
OGX02385.1	*Omnitrophica WOR_2 bacterium GWA2_44_7*	VanZ-2
KPK97115.1	*Omnitrophica WOR_2 bacterium SM23_72*	VanZ-2
OGW96656.1	*Omnitrophica WOR_2 bacterium GWA2_45_18*	VanZ-2
MBL7070017.1	*Candidatus Omnitrophota bacterium*	VanZ-2
OGX08778.1	*Omnitrophica WOR_2 bacterium GWA2_47_8*	VanZ-2
MBM3255119.1	*Candidatus Omnitrophota bacterium*	VanZ-2
MBM3251629.1	*Candidatus Omnitrophota bacterium*	VanZ-2
MCA9405872.1	*Candidatus Omnitrophota bacterium*	VanZ-2
MBU4343367.1	*Candidatus Omnitrophota bacterium*	VanZ-2
OIP72447.1	*Oscillatoriales cyanobacterium CG2_30_40_61*	VanZ-2
MBP6342916.1	*Candidatus Omnitrophota bacterium*	VanZ-2
WP_213623113.1		VanZ-2
OGX26380.1	*Omnitrophica WOR_2 bacterium RIFCSPHIGHO2_02_FULL_48_11*	VanZ-2
MBI4982469.1	*Candidatus Omnitrophota bacterium*	VanZ-2
MBL7092312.1	*Candidatus Omnitrophota bacterium*	VanZ-2
MBU1726705.1	*Candidatus Omnitrophota bacterium*	VanZ-2
KJJ85589.1	*Candidatus Omnitrophus magneticus*	VanZ-2
OGX54140.1	*Omnitrophica WOR_2 bacterium RIFOXYB2_FULL_45_11*	VanZ-2
OGW76599.1	*Omnitrophica bacterium RIFCSPHIGHO2_02_FULL_46_20*	VanZ-2
OGX44394.1	*Omnitrophica WOR_2 bacterium RIFCSPLOWO2_12_FULL_51_8*	VanZ-2
MBN1913335.1	*Candidatus Omnitrophota bacterium*	VanZ-2
MBU0571210.1	*Candidatus Omnitrophota bacterium*	VanZ-2
MBP9854883.1	*Candidatus Omnitrophota bacterium*	VanZ-2
NLT73560.1	*Chloroflexota bacterium*	VanZ-2
NLE91468.1	*Candidatus Undivivens industriae*	VanZ-2
PIP67832.1	*Candidatus Omnitrophica bacterium CG22_combo_CG10-13_8_21_14_all_43_16*	VanZ-2
RKY42869.1	*Candidatus Makaraimicrobium thalassicum*	VanZ-2
MCA9394024.1	*Candidatus Omnitrophota bacterium*	VanZ-2
OGX15752.1	*Omnitrophica WOR_2 bacterium RBG_13_41_10*	VanZ-2
NQT32599.1	*Candidatus Omnitrophota bacterium*	VanZ-2
OGX27026.1	*Omnitrophica WOR_2 bacterium RIFCSPHIGHO2_02_FULL_45_21*	VanZ-2
MBN1869090.1	*Candidatus Omnitrophota bacterium*	VanZ-2
NQT75559.1	*Candidatus Omnitrophota bacterium*	VanZ-2
OGX41790.1	*Omnitrophica WOR_2 bacterium RIFCSPLOWO2_12_FULL_51_24*	VanZ-2
MBU1124644.1	*Candidatus Omnitrophota bacterium*	VanZ-2
MBR3245117.1	*Parasporobacterium sp.*	VanZ-2
MCA9409023.1	*Candidatus Omnitrophota bacterium*	VanZ-2
MBU1888183.1	*Candidatus Omnitrophota bacterium*	VanZ-2
MBU1061227.1	*Candidatus Omnitrophota bacterium*	VanZ-2
OGP19180.1	*Deltaproteobacteria bacterium GWA2_65_63*	VanZ-2
MBN2213252.1	*Bacteroidales bacterium*	VanZ-2
MBL7994835.1	*bacterium*	VanZ-2
NUN44627.1	*bacterium*	VanZ-2
NUM79212.1	*bacterium*	VanZ-2
NLC17988.1	*Clostridiales bacterium*	VanZ-2
MBO5088334.1	*Lachnospiraceae bacterium*	VanZ-2
MBQ9885672.1	*Lachnospiraceae bacterium*	VanZ-2
HIS47117.1	*Candidatus Scybalocola faecigallinarum*	VanZ-2
CDA28402.1	*Eubacterium sp. CAG:156*	VanZ-2
MBP3325970.1	*Coprococcus sp.*	VanZ-2
QNM09008.1	*Wansuia hejianensis*	VanZ-2
MBE5921671.1	*Lachnospiraceae bacterium*	VanZ-2
CCY61884.1	*Clostridium sp. CAG:264*	VanZ-2

NLK27460.1<br>*Clostridiales bacterium*	VanZ
HHT97345.1<br>*Clostridiales bacterium*	VanZ
MBQ3515467.1<br>*Lachnospiraceae bacterium*	VanZ
MBP3339717.1<br>*Lachnospiraceae bacterium*	VanZ
HIT89477.1<br>*Candidatus Merdenecus merdavium*	VanZ
HIS31523.1<br>*Candidatus Limivivens intestinipullorum*	VanZ
MBO5197964.1<br>*Lachnospiraceae bacterium*	VanZ
HIR24206.1<br>*Candidatus Egerieimonas faecigallinarum*	VanZ
MBS5060415.1<br>*Roseburia hominis*	VanZ
MBQ9983028.1<br>*Lachnospiraceae bacterium*	VanZ
NBH98856.1<br>*Lachnospiraceae bacterium*	VanZ
NCB91351.1<br>*Clostridia bacterium*	VanZ
HIU67664.1<br>*Candidatus Caccomorpha excrementavium*	VanZ
WP_028529063.1<br>*Ruminococcus gauvreauii*	VanZ
HIV40046.1<br>*Candidatus Blautia stercorigallinarum*	VanZ
MBO5373779.1<br>*Lachnospiraceae bacterium*	VanZ
WP_115482054.1<br>*Anaerosacchariphilus polymeriproducens*	VanZ
HIV22782.1<br>*Candidatus Merdiplasma excrementigallinarum*	VanZ
OGW34134.1<br>*Nitrospirae bacterium GWC2_56_14*	VanZ
UCC62920.1<br>*Anaerolineae bacterium*	VanZ
MBE6016759.1<br>*Lachnospiraceae bacterium*	VanZ
WP_152529543.1<br>*Aliihoeflea sp. 2WW*	VanZ
MBW1937045.1<br>*Deltaproteobacteria bacterium*	VanZ
MBE6329009.1<br>*Bacteroidales bacterium*	VanZ
MBI5538433.1<br>*Bacteroidia bacterium*	VanZ
UCG81745.1<br>*Desulfobacterales bacterium*	VanZ
MAW49714.1<br>*Flavobacteriaceae bacterium*	VanZ
RPG70669.1<br>*Flavobacteriaceae bacterium TMED120*	VanZ
MBL30703.1<br>*Flavobacteriaceae bacterium*	VanZ
KAF0218555.1<br>*Geobacteraceae bacterium*	VanZ
WP_014551667.1<br>*Geobacter sulfurreducens*	VanZ
WP_012648071.1<br>*Geotalea daltonii FRC-32*	VanZ
WP_068655898.1<br>*Paenibacillus crassostreae*	VanZ
WP_102246998.1<br>*Pseudazoarcus pumilus*	VanZ
CDA67622.1<br>*Clostridium sp. CAG:510*	VanZ
MBD5500926.1<br>*Lachnospiraceae bacterium*	VanZ
MCC6484813.1<br>*Armatimonadota bacterium*	VanZ
MBO4282440.1<br>*Bacteroidales bacterium*	VanZ
MBO5538119.1<br>*Prevotella sp.*	VanZ
MCD8151365.1<br>*Clostridiales bacterium*	VanZ
NLK94564.1<br>*Clostridiales bacterium*	VanZ
WP_015424428.1<br>*Candidatus Cloacimonas acidaminovorans str. Evry*	VanZ
WP_077598107.1<br>*Olsenella urininfantis*	VanZ
WP_087225023.1<br>*Olsenella sp. An270*	VanZ
WP_087217049.1<br>*Olsenella sp. An285*	VanZ
MBS6401059.1<br>*Atopobiaceae bacterium*	VanZ
WP_087320705.1<br>*Olsenella sp. An290*	VanZ
MBP3884278.1<br>*Olsenella sp.*	VanZ
HBH96229.1<br>*Candidatus Omnitrophota bacterium*	VanZ
MSP70932.1<br>*Myxococcales bacterium*	VanZ
MBN1872359.1<br>*Candidatus Omnitrophota bacterium*	VanZ
MBW7944358.1<br>*Patescibacteria group bacterium*	VanZ
HGK50783.1	VanZ
WP_087837895.1<br>*Pigmentiphaga sp. NML030171*	VanZ
NQT46219.1<br>*Candidatus Omnitrophota bacterium*	VanZ
MBI4343945.1<br>*Candidatus Omnitrophota bacterium*	VanZ
NPA13218.1<br>*Aquificota bacterium*	VanZ
MBI4597762.1<br>*Candidatus Omnitrophota bacterium*	VanZ
MBP1675655.1<br>*Bacteroidota bacterium*	VanZ
MBQ6453078.1<br>*Coriobacteriales bacterium*	VanZ
MBN2347243.1<br>*Bacteroidales bacterium*	VanZ
PYR32256.1<br>*Acidobacteriota bacterium*	VanZ
MBQ3419453.1<br>*Erysipelotrichaceae bacterium*	VanZ
WP_118365725.1<br>*Amedibacillus dolichus*	VanZ
MCC5877637.1<br>*Candidatus Sumerlaeia bacterium*	VanZ
QHS17306.1<br>*haloarchaeon 3A1-DGR*	VanZ
MBE7056984.1<br>*Oscillospiraceae bacterium*	VanZ
OHD17488.1<br>*Spirochaetes bacterium GWB1_60_80*	VanZ
WP_051527885.1<br>*Balneatrix alpica*	VanZ
WP_015435734.1<br>*Azoarcus sp. KH32C*	VanZ
NOT24681.1<br>*Acidobacteriota bacterium*	VanZ
MCC7043398.1<br>*Acidobacteriota bacterium*	VanZ
MBB3346298.1<br>*Sphingomonas sp. BK069*	VanZ
BBF44101.1<br>*Lachnospiraceae bacterium KM106-2*	VanZ
PZN82915.1	VanZ
ODS57007.1<br>*Acidobacteria bacterium SCN 69-37*	VanZ
MCB1857805.1<br>*Gammaproteobacteria bacterium*	VanZ
HAU86482.1<br>*Lachnospiraceae bacterium*	VanZ
MBO5055572.1<br>*Lachnospiraceae bacterium*	VanZ
MBI4265611.1<br>*Acidobacteriota bacterium*	VanZ
MBI2174808.1<br>*Candidatus Omnitrophota bacterium*	VanZ
WP_213340265.1<br>*Ancylobacter lacus*	VanZ
WP_102628989.1<br>*Halomonas heilongjiangensis*	VanZ
HIT34453.1<br>*Candidatus Faecousia intestinigallinarum*	VanZ
HAJ57384.1<br>*Candidatus Omnitrophota bacterium*	VanZ
PIY82402.1<br>*Candidatus Omnitrophica bacterium CG_4_10_14_0_8_um_filter_44_12*	VanZ
KON28731.1<br>*miscellaneous Crenarchaeota group archaeon SMTZ-80*	VanZ
MBQ7324830.1<br>*Clostridia bacterium*	VanZ
MBW6440793.1<br>*Patescibacteria group bacterium*	VanZ
MBQ0035642.1<br>*Candidatus Colivicinus equi*	VanZ
MBO7677737.1<br>*Erysipelotrichaceae bacterium*	VanZ
MBQ2657488.1<br>*Erysipelotrichaceae bacterium*	VanZ
MBR6233255.1<br>*Erysipelotrichaceae bacterium*	VanZ

OZA20638.1	*Novosphingobium sp. 17-62-19*	VanZ-2
WP_183613896.1	*Novosphingobium hassiacum*	VanZ-2
MCD6476459.1	*Anaerolineaceae bacterium*	VanZ-2
WP_060826131.1	*Sulfurospirillum cavolei*	VanZ-2
MBO5454571.1	*Clostridia bacterium*	VanZ-2
WP_232366977.1		VanZ-2
NLG73740.1	*Chloroflexota bacterium*	VanZ-2
OGS02171.1	*Elusimicrobia bacterium RIFOXYA12_FULL_49_49*	VanZ-2
MBP0958267.1	*Oscillospiraceae bacterium*	VanZ-2
WP_105043666.1	*Rubritalea profundi*	VanZ-2
OQW76920.1	*Proteobacteria bacterium ST_bin11*	VanZ-2
WP_143184396.1	*Rubritalea squalenifaciens DSM 18772*	VanZ-2
WP_006968381.1	*Desulfotignum phosphitoxidans DSM 13687*	VanZ-2
CDE53932.1	*Roseburia sp. CAG:303*	VanZ-2
WP_163319124.1	*Dysgonomonas sp. 520*	VanZ-2
MBM64455.1	*Acidobacteriota bacterium*	VanZ-2
TDI34457.1	*Acidobacteriota bacterium*	VanZ-2
MBR3771254.1	*Clostridium sp.*	VanZ-2
OFW05908.1	*Acidobacteria bacterium RIFCSPLOWO2_02_FULL_68_18*	VanZ-2
MCC7123333.1	*Acidobacteriota bacterium*	VanZ-2
MCC7240562.1	*Acidobacteriota bacterium*	VanZ-2
MBI2219845.1	*Acidobacteriota bacterium*	VanZ-2
MBE40754.1	*Acidobacteriota bacterium*	VanZ-2
MXZ72519.1	*Acidobacteriota bacterium*	VanZ-2
MPY86648.1	*Luteitalea sp.*	VanZ-2
TDI22796.1	*Acidobacteriota bacterium*	VanZ-2
MCC7177163.1	*Acidobacteriota bacterium*	VanZ-2
MBM3750873.1	*Acidimicrobiia bacterium*	VanZ-2
MBI1874650.1	*Acidobacteriota bacterium*	VanZ-2
MBS1818720.1	*Acidobacteriota bacterium*	VanZ-2
MSO83169.1	*Acidobacteriota bacterium*	VanZ-2
MBK9240632.1	*Acidobacteriota bacterium*	VanZ-2
HIC58135.1	*Acidobacteriota bacterium*	VanZ-2
MBQ8848958.1	*Clostridia bacterium*	VanZ-2
OFW15892.1	*Acidobacteria bacterium RIFCSPLOWO2_02_FULL_67_21*	VanZ-2
MCC7008379.1	*Acidobacteriota bacterium*	VanZ-2
MBP7778758.1	*Acidobacteriota bacterium*	VanZ-2
MCE2514424.1	*Acidobacteriota bacterium*	VanZ-2
MBL02074.1	*Acidobacteriota bacterium*	VanZ-2
MBF8300180.1	*Acidobacteriota bacterium*	VanZ-2
MYI74414.1	*Acidobacteriota bacterium*	VanZ-2
MBQ9072182.1	*Bacilli bacterium*	VanZ-2
MBR2801600.1	*Erysipelotrichaceae bacterium*	VanZ-2
NLC70879.1	*Desulfuromonadaceae bacterium*	VanZ-2
WP_145288421.1	*Salinicoccus cyprini*	VanZ-2
MCA1585466.1	*Acidobacteriota bacterium*	VanZ-2
MBM3260531.1	*Candidatus Tanganyikabacteria bacterium*	VanZ-2
MBR3770245.1	*Lachnospiraceae bacterium*	VanZ-2
MCB1875175.1	*Chromatiales bacterium*	VanZ-2
KKM09446.1	*Clostridiales bacterium PH28_bin88*	VanZ-2
MBS5645646.1	*Clostridiales bacterium*	VanZ-2
MBS6477905.1	*Collinsella sp.*	VanZ-2
MBQ3293862.1	*Candidatus Saccharibacteria bacterium*	VanZ-2
MBK6588477.1	*Acidobacteriota bacterium*	VanZ-2
TDL79023.1	*Peribacillus frigoritolerans*	VanZ-2
MBR5779660.1	*Clostridia bacterium*	VanZ-2
MBQ6831820.1	*Oscillospiraceae bacterium*	VanZ-2
HIA54317.1	*Candidatus Melainabacteria bacterium*	VanZ-2
PIW28897.1	*Rhodobacterales bacterium CG15_BIG_FIL_POST_REV_8_21_14_020_59_13*	VanZ-2
MBO6214271.1	*Lachnospiraceae bacterium*	VanZ-2
PYM90773.1	*Candidatus Rokuibacteriota bacterium*	VanZ-2
MBE5968254.1	*Lachnospiraceae bacterium*	VanZ-2
WP_007700617.1	*Halovivax asiaticus JCM 14624*	VanZ-2
HBR82387.1	*Enterococcus sp.*	VanZ-2
MCA9185493.1	*Planctomycetales bacterium*	VanZ-2
PZR73745.1	*Chthoniobacterales bacterium*	VanZ-2
MBQ9734758.1	*Clostridia bacterium*	VanZ-2
MBQ3589763.1	*Clostridia bacterium*	VanZ-2
MBP7416952.1	*Pyrinomonadaceae bacterium*	VanZ-2
MCA9132455.1	*Planctomycetales bacterium*	VanZ-2
MBQ8913637.1	*Lachnospiraceae bacterium*	VanZ-2
WP_215191958.1	*Exiguobacterium sp. s7*	VanZ-2
WP_215142192.1	*Exiguobacterium qingdaonense*	VanZ-2
MBE7020679.1	*Oscillospiraceae bacterium*	VanZ-2
HJC35792.1	*Candidatus Merdibacter merdavium*	VanZ-2
WP_015723677.1	*Desulfobulbus propionicus DSM 2032*	VanZ-2
MBI1886312.1	*Chloroflexota bacterium*	VanZ-2
MBI4508639.1	*Deltaproteobacteria bacterium*	VanZ-2
HDS15132.1	*Pseudomonadota bacterium*	VanZ-2
MBR5745754.1	*Clostridia bacterium*	VanZ-2
MBR5859477.1	*Clostridia bacterium*	VanZ-2
NDC87261.1	*Bacteroidota bacterium*	VanZ-2
MBC7390982.1	*Cytophagales bacterium*	VanZ-2
WP_193638163.1	*Nocardioides malaquae*	VanZ-2
MBR4950383.1	*Clostridia bacterium*	VanZ-2
MBO4594222.1	*Clostridia bacterium*	VanZ-2
NQV05925.1	*bacterium*	VanZ-2
OUV37780.1	*Opitutia bacterium TMED102*	VanZ-2
UCG49201.1	*Phycisphaerales bacterium*	VanZ-2
MBQ7432629.1	*Lachnospiraceae bacterium*	VanZ-2
MBD9185066.1	*Clostridiales bacterium*	VanZ-2
WP_090872243.1	*Oceanobacillus limi*	VanZ-2
MBD9185235.1	*Clostridiales bacterium*	VanZ-2

MBQ2453517.1	*Lachnospiraceae bacterium*	VanZ-2
MBV8519102.1	*Acidobacteriota bacterium*	VanZ-2
OGY88961.1	*Candidatus Komeilibacteria bacterium RIFCSPHIGHO2_01_FULL_52_14*	VanZ-2
MBP6911759.1	*Candidatus Paceibacterota bacterium*	VanZ-2
NLM57731.1	*Clostridium sp.*	VanZ-2
ODS96690.1	*Lautropia sp. SCN 69-89*	VanZ-2
MBR3560402.1	*Oscillospiraceae bacterium*	VanZ-2
ADV83962.1	*Terriglobus saanensis SP1PR4*	VanZ-2
KAA3619081.1	*Pseudomonadota bacterium*	VanZ-2
MBF0373016.1	*Alphaproteobacteria bacterium*	VanZ-2
WP_156289491.1	*Oceanobacillus salinisoli*	VanZ-2
MBK4796144.1	*Enterococcus faecium*	VanZ-2
MBQ4600431.1	*Oscillospiraceae bacterium*	VanZ-2
MBE6551989.1	*Oscillospiraceae bacterium*	VanZ-2
WP_089383536.1	*Halorubrum vacuolatum*	VanZ-2
MBQ7782855.1	*Oscillospiraceae bacterium*	VanZ-2
QLQ10610.1	*Nocardioidaceae bacterium*	VanZ-2
QRN85730.1	*Clostridia bacterium*	VanZ-2
MBR3149914.1	*Eubacterium sp.*	VanZ-2
MBQ0102478.1	*Candidatus Colimorpha enterica*	VanZ-2
MBO5184954.1	*Clostridia bacterium*	VanZ-2
MBN1920490.1	*Anaerolineae bacterium*	VanZ-2
MBE5926734.1	*Lachnospiraceae bacterium*	VanZ-2
MBI3969271.1	*Chloroflexota bacterium*	VanZ-2
MBX3256833.1	*Chitinophagaceae bacterium*	VanZ-2
MBD5295158.1	*Bacteroides sp.*	VanZ-2
MCD8316126.1	*Eggerthellaceae bacterium*	VanZ-2
MBE6806972.1	*Oscillospiraceae bacterium*	VanZ-2
MBZ0189970.1	*Candidatus Obscuribacterales bacterium*	VanZ-2
HER51434.1		VanZ-2
MBE5898526.1	*Lachnospiraceae bacterium*	VanZ-2
NLW22018.1	*Tissierellia bacterium*	VanZ-2
MCA1622419.1	*Acidobacteriota bacterium*	VanZ-2
MBR4635944.1	*Clostridia bacterium*	VanZ-2
MBO4351981.1	*Eggerthellaceae bacterium*	VanZ-2
MBP5092072.1	*Bacilli bacterium*	VanZ-2
MCB1023751.1	*Acidobacteriota bacterium*	VanZ-2
MBR1968712.1	*Clostridia bacterium*	VanZ-2
HID64574.1	*Anaerolineae bacterium*	VanZ-2
WP_206391564.1	*Cytobacillus horneckiae*	VanZ-2
MBP7290259.1	*Nannocystaceae bacterium*	VanZ-2
WP_231716098.1		VanZ-2
MCD8132803.1	*Clostridiales bacterium*	VanZ-2
MCC6129815.1	*Acidobacteriota bacterium*	VanZ-2
MCC8150993.1	*Lachnospiraceae bacterium*	VanZ-2
MBK05348.1	*Deltaproteobacteria bacterium*	VanZ-2
MBR1970721.1	*Clostridia bacterium*	VanZ-2
WP_059103630.1	*Shouchella shacheensis*	VanZ-2
WP_008442261.1	*Halorubrum sp. GN11GM_10-3_MGM*	VanZ-2
WP_004427353.1	*Alkalihalobacillus alcalophilus*	VanZ-2
MBT7651436.1	*Opitutia bacterium*	VanZ-2
MBQ8359027.1	*Oscillospiraceae bacterium*	VanZ-2
MBL4576151.1	*Opitutaceae bacterium*	VanZ-2
MCB0838294.1	*Bacteroidota bacterium*	VanZ-2
WP_090082740.1	*Halpernia frigidisoli*	VanZ-2
MBF0341779.1	*Magnetococcales bacterium*	VanZ-2
MBA3961247.1	*Chthoniobacterales bacterium*	VanZ-2
MBQ7410907.1	*Clostridia bacterium*	VanZ-2
MBQ7386600.1	*Clostridia bacterium*	VanZ-2
HIY25649.1	*Candidatus Acutalibacter pullistercoris*	VanZ-2
MBS7374514.1	*Muribaculaceae bacterium*	VanZ-2
MBK5191274.1	*Methanosarcinales archaeon*	VanZ-2
MBQ6890891.1	*Oscillospiraceae bacterium*	VanZ-2
HAR0974533.1	*Enterococcus faecium*	VanZ-2
MBQ4337901.1	*Clostridia bacterium*	VanZ-2
MBR7927211.1	*Aerococcaceae bacterium zg-ZUI334*	VanZ-2
MBC7238319.1	*Chloroflexota bacterium*	VanZ-2
WP_051456711.1	*Ruminococcus flavefaciens 007c*	VanZ-2
MBP7473434.1	*Prevotella sp.*	VanZ-2
NQV28353.1	*Rhodopirellula sp.*	VanZ-2
NLG50820.1	*Chloroflexota bacterium*	VanZ-2
MBQ9663271.1	*Oscillospiraceae bacterium*	VanZ-2
MBQ9686315.1	*Oscillospiraceae bacterium*	VanZ-2
OJU40533.1	*Microbacterium sp. 69-10*	VanZ-2
MAE63789.1	*Phycisphaeraceae bacterium*	VanZ-2
MBI2872068.1	*Chloroflexota bacterium*	VanZ-2
HHN33412.1		VanZ-2
MBC7814767.1	*Anaerolineae bacterium*	VanZ-2
WP_066363367.1	*Neobacillus drentensis*	VanZ-2
WP_007085014.1	*Neobacillus bataviensis LMG 21833*	VanZ-2
MBL4616401.1	*Robiginitomaculum sp.*	VanZ-2
MBQ8277470.1	*Bacteroidaceae bacterium*	VanZ-2
MBQ7036019.1	*Clostridia bacterium*	VanZ-2
WP_191737873.1	*Chryseobacterium caseinilyticum*	VanZ-2
MBK6980211.1	*Betaproteobacteria bacterium*	VanZ-2
WP_140922339.1	*Apilactobacillus timberlakei*	VanZ-2
MBQ3602120.1	*Lachnospiraceae bacterium*	VanZ-2
HEB54007.1	*bacterium*	VanZ-2
MBQ8244470.1	*Oscillospiraceae bacterium*	VanZ-2
MBQ9842344.1	*Clostridia bacterium*	VanZ-2
MBY0274013.1		VanZ-2
MBQ3149998.1	*Clostridia bacterium*	VanZ-2
WP_168914832.1	*Microcella flavibacter*	VanZ-2

MBR0367994.1	*Clostridia bacterium*	VanZ-2
MBR3900980.1	*Ruminococcus sp.*	VanZ-2
WP_013708989.1	*Coriobacterium glomerans PW2*	VanZ-2
MBR5683492.1	*Ruminococcus sp.*	VanZ-2
KUP08419.1	*Bacillus coahuilensis m2-6*	VanZ-2
WP_190997076.1		VanZ-2
MAM53302.1	*Microbacterium sp.*	VanZ-2
MBI5416237.1	*Candidatus Omnitrophota bacterium*	VanZ-2
MBP5378269.1	*Ruminococcus sp.*	VanZ-2
WP_168241154.1		VanZ-2
MBR3817136.1	*Clostridia bacterium*	VanZ-2
WP_130416204.1	*Xylanimonas ulmi*	VanZ-2
MBR7063266.1	*Clostridia bacterium*	VanZ-2
MBE6141176.1	*Bacillota bacterium*	VanZ-2
MBP7694696.1	*Anaerolineales bacterium*	VanZ-2
WP_154532595.1	*Inconstantimicrobium porci*	VanZ-2
MBR2888895.1	*Oscillospiraceae bacterium*	VanZ-2
HBJ36174.1	*Planctomycetaceae bacterium*	VanZ-2
MBR2880196.1	*Oscillospiraceae bacterium*	VanZ-2
MBD8944737.1	*Oscillospiraceae bacterium*	VanZ-2
QPQ36688.1	*Lysinibacillus sp. JNUCC-52*	VanZ-2
MCB9234991.1	*Bacteroidia bacterium*	VanZ-2
KJS88012.1	*Desulfosporosinus sp. BICA1-9*	VanZ-2
MBR2937282.1	*Oscillospiraceae bacterium*	VanZ-2
MBK9449537.1	*Bacteroidota bacterium*	VanZ-2
MBO4869689.1	*Clostridia bacterium*	VanZ-2
NLW70923.1	*Eubacteriaceae bacterium*	VanZ-2
MBQ2715633.1	*Clostridia bacterium*	VanZ-2
NCB71750.1	*Clostridia bacterium*	VanZ-2
MBR2215473.1	*Selenomonadaceae bacterium*	VanZ-2
MBP3926974.1	*Clostridium sp.*	VanZ-2
MBE6050217.1	*Clostridium sp.*	VanZ-2
WP_169361223.1	*Psychrobacillus sp. BL-248-WT-3*	VanZ-2
MBR4287715.1	*Clostridia bacterium*	VanZ-2
NLI67682.1	*Bacilli bacterium*	VanZ-2
NMA52656.1	*Peptococcaceae bacterium*	VanZ-2
MBR4473163.1	*Oscillospiraceae bacterium*	VanZ-2
WP_051659081.1	*Acholeplasma hippikon*	VanZ-2
HIS18082.1	*Candidatus Coprovivens excrementavium*	VanZ-2
NNC98856.1	*Gammaproteobacteria bacterium*	VanZ-2
WP_216289280.1	*Clostridium psychrophilum*	VanZ-2
WP_212905168.1	*Clostridium polyendosporum*	VanZ-2
MBR4456399.1	*Solobacterium sp.*	VanZ-2
RJG51673.1	*Motilimonas pumila*	VanZ-2
WP_184310921.1	*Anaerosolibacter carboniphilus*	VanZ-2
MBM4442199.1	*Candidatus Rokuibacteriota bacterium*	VanZ-2
AIQ63806.1	*Paenibacillus stellifer*	VanZ-2
OPY59277.1	*Pelotomaculum sp. PtaU1.Bin035*	VanZ-2
WP_184324535.1	*Brachybacterium aquaticum*	VanZ-2
PYV57989.1	*Acidobacteriota bacterium*	VanZ-2
HIW57301.1	*Bacillota bacterium*	VanZ-2
WP_008288403.1	*Hydrogenivirga sp. 128-5-R1-1*	VanZ-2
HJB04737.1	*Candidatus Merdibacter merdigallinarum*	VanZ-2
HIU80674.1	*Candidatus Coproplasma excrementipullorum*	VanZ-2
MBR5570795.1	*Oscillospiraceae bacterium*	VanZ-2
MBQ4097587.1	*Clostridia bacterium*	VanZ-2
MBD5137184.1	*Lachnospiraceae bacterium*	VanZ-2
MBO5274971.1	*Clostridia bacterium*	VanZ-2
TNF85770.1	*Gammaproteobacteria bacterium*	VanZ-2
MBQ2658042.1	*Erysipelotrichaceae bacterium*	VanZ-2
MBS5451708.1	*Coriobacteriia bacterium*	VanZ-2
MBQ5865330.1	*Oscillospiraceae bacterium*	VanZ-2
GHC78564.1	*Nocardiopsis terrae*	VanZ-2
HIE95801.1	*Fuerstiella sp.*	VanZ-2
MBO5049409.1	*Oscillospiraceae bacterium*	VanZ-2
MBU6213440.1	*Actinomycetales bacterium*	VanZ-2
MBU3964982.1	*Patescibacteria group bacterium*	VanZ-2
MBC8117162.1	*Planctomycetaceae bacterium*	VanZ-2
WP_079442226.1	*Clostridium chromiireducens*	VanZ-2
WP_052019637.1	*Paenibacillus sp. JCM 10914*	VanZ-2
MBP3361031.1	*Clostridia bacterium*	VanZ-2
HHZ11682.1	*Acholeplasmataceae bacterium*	VanZ-2
MBP3597515.1		VanZ-2
MBQ4137636.1	*Clostridia bacterium*	VanZ-2
MBR5267087.1	*Lachnospiraceae bacterium*	VanZ-2
OGP71110.1	*Deltaproteobacteria bacterium RBG_13_58_19*	VanZ-2
MBZ4647637.1	*Clostridia bacterium*	VanZ-2
MBO7611901.1	*Candidatus Ruminimicrobiellum bubulum*	VanZ-2
WP_023562120.1	*Actinoplanes friuliensis DSM 7358*	VanZ-2
MBN4074890.1	*bacterium AH-315-E09*	VanZ-2
WP_163539554.1	*Gracilibacillus sp. YIM 98692*	VanZ-2
OGN50867.1	*Caulobacterales bacterium RIFOXYB1_FULL_67_16*	VanZ-2
MBL4823047.1	*Colwellia sp.*	VanZ-2
MBT9500128.1	*Burkholderiaceae bacterium*	VanZ-2
HCQ83296.1	*Verrucomicrobiales bacterium*	VanZ-2
WP_003423178.1	*Clostridium butyricum*	VanZ-2
TDP75515.1	*Brachybacterium sp. AG952*	VanZ-2
MBR5337841.1	*Lachnospiraceae bacterium*	VanZ-2
MBN2669618.1	*Bacteroidales bacterium*	VanZ-2
PYV85248.1	*Acidobacteriota bacterium*	VanZ-2
WP_012799411.1	*Slackia heliotrinireducens*	VanZ-2
MCC5970117.1	*Pararhodobacter sp.*	VanZ-2
OGO76767.1	*Clostridiales bacterium GWB2_37_7*	VanZ-2

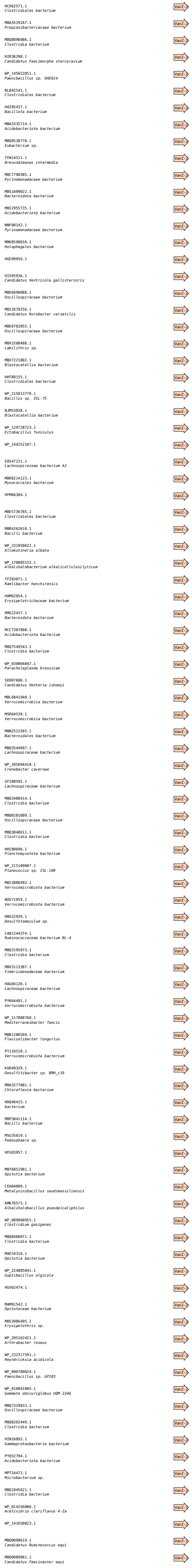
HCO62571.1
Clostridiales bacterium
VanZ
MBA3529167.1
Propionibacteriaceae bacterium
VanZ
MBQ8896086.1
Clostridia bacterium
VanZ
HIR36290.1
Candidatus Faecimorpha stercoravium
VanZ
WP_145022051.1
Paenibacillus sp. OAE614
VanZ
NLB42541.1
Clostridiales bacterium
VanZ
HAI85437.1
Bacillota bacterium
VanZ
MBA3335714.1
Acidobacteriota bacterium
VanZ
MBQ9530770.1
Eubacterium sp.
VanZ
TFW14511.1
Brevundimonas intermedia
VanZ
MBC7798385.1
Pyrinomonadaceae bacterium
VanZ
MBS1609022.1
Bacteroidota bacterium
VanZ
MBI2955735.1
Acidobacteriota bacterium
VanZ
NNF00142.1
Pyrinomonadaceae bacterium
VanZ
MBK8598816.1
Holophagales bacterium
VanZ
HGE99956.1
VanZ
HIS95936.1
Candidatus Ventricola gallistercoris
VanZ
MBE6690088.1
Oscillospiraceae bacterium
VanZ
MBI2679256.1
Candidatus Korobacter versatilis
VanZ
MBE6702855.1
Oscillospiraceae bacterium
VanZ
MBX3208488.1
Labilithrix sp.
VanZ
MBX7221802.1
Blastocatellia bacterium
VanZ
HHT89155.1
Clostridiales bacterium
VanZ
WP_215013770.1
Bacillus sp. ISL-75
VanZ
NJM53838.1
Blastocatellia bacterium
VanZ
WP_129728723.1
Ectobacillus funiculus
VanZ
WP_144252187.1
VanZ
EOS47231.1
Lachnospiraceae bacterium A2
VanZ
MBK8214123.1
Myxococcales bacterium
VanZ
HFM66304.1
VanZ
MBE5736785.1
Clostridiales bacterium
VanZ
MBR4262610.1
Bacilli bacterium
VanZ
WP_231950622.1
Allokutzneria albata
VanZ
WP_170885532.1
Alkalihalobacterium alkalicellulosilyticum
VanZ
TFZ03071.1
Ramlibacter henchirensis
VanZ
HAM62654.1
Erysipelotrichaceae bacterium
VanZ
RMG22437.1
Bacteroidota bacterium
VanZ
MCC7307898.1
Acidobacteriota bacterium
VanZ
MBQ7549543.1
Clostridia bacterium
VanZ
WP_030004047.1
Paracholeplasma brassicae
VanZ
SEH07680.1
Candidatus Venteria ishoeyi
VanZ
MBL6841940.1
Verrucomicrobiia bacterium
VanZ
MSR64539.1
Verrucomicrobiia bacterium
VanZ
MBN2521565.1
Bacteroidales bacterium
VanZ
MBQ3544697.1
Lachnospiraceae bacterium
VanZ
WP_205694418.1
Crenobacter cavernae
VanZ
GFI08595.1
Lachnospiraceae bacterium
VanZ
MBQ3488414.1
Clostridia bacterium
VanZ
MBQ6281689.1
Oscillospiraceae bacterium
VanZ
MBQ3848611.1
Clostridia bacterium
VanZ
HHI80696.1
Planctomycetota bacterium
VanZ
WP_215109007.1
Planococcus sp. ISL-109
VanZ
MBI3880492.1
Verrucomicrobiota bacterium
VanZ
NOS71959.1
Verrucomicrobiota bacterium
VanZ
HBX22939.1
Desulfotomaculum sp.
VanZ
CAB1244374.1
Ruminococcaceae bacterium BL-4
VanZ
MBQ3195073.1
Clostridia bacterium
VanZ
MBX3113387.1
Fimbriimonadaceae bacterium
VanZ
HAG04126.1
Lachnospiraceae bacterium
VanZ
PYK64491.1
Verrucomicrobiota bacterium
VanZ
WP_117888760.1
Mediterraneibacter faecis
VanZ
MBB1286569.1
Flavisolibacter longurius
VanZ
PYJ16510.1
Verrucomicrobiota bacterium
VanZ
KUO49329.1
Desulfitibacter sp. BRH_c19
VanZ
MBA3277001.1
Chloroflexia bacterium
VanZ
HDQ46415.1
bacterium
VanZ
MBP3841114.1
Bacilli bacterium
VanZ
MSU35619.1
Pedosphaera sp.
VanZ
HFG83957.1
VanZ
MBT6851981.1
Opitutia bacterium
VanZ
CEA04009.1
Metalysinibacillus saudimassiliensis
VanZ
KMK76573.1
Alkalihalobacillus pseudalcaliphilus
VanZ
WP_089968955.1
Clostridium gasigenes
VanZ
MBQ8406071.1
Clostridia bacterium
VanZ
MAE54316.1
Opitutia bacterium
VanZ
WP_224885041.1
Guptibacillus algicola
VanZ
HGV62474.1
VanZ
MAM91542.1
Opitutaceae bacterium
VanZ
MBS3986495.1
Erysipelothrix sp.
VanZ
WP_205162421.1
Arthrobacter roseus
VanZ
WP_232317591.1
Heyndrickxia acidicola
VanZ
WP_090789024.1
Paenibacillus sp. GP183
VanZ
WP_010041005.1
Gemmata obscuriglobus UQM 2246
VanZ
MBQ7329833.1
Oscillospiraceae bacterium
VanZ
MBQ8202449.1
Clostridia bacterium
VanZ
HIN16892.1
Gammaproteobacteria bacterium
VanZ
PYQ52794.1
Acidobacteriota bacterium
VanZ
MPT14472.1
Microbacterium sp.
VanZ
MBQ1945621.1
Clostridia bacterium
VanZ
WP_014256006.1
Acetivibrio clariflavus 4-2a
VanZ
WP_141650823.1
VanZ
MBQ0098619.1
Candidatus Ruminococcus equi
VanZ
MBQ0089961.1
Candidatus Faecinaster equi
VanZ

WP_185147894.1	*Peribacillus simplex*	VanZ-2
MBR3327342.1	*Atopobiaceae bacterium*	VanZ-2
MBD3175515.1	*Armatimonadia bacterium*	VanZ-2
PRR83073.1	*Clostridium vincentii*	VanZ-2
WP_170287774.1	*Arthrobacter citreus*	VanZ-2
MBQ8089078.1	*Clostridia bacterium*	VanZ-2
MBQ8797266.1	*Oscillospiraceae bacterium*	VanZ-2
MBQ7975554.1	*Clostridia bacterium*	VanZ-2
NDB18079.1	*Actinomycetota bacterium*	VanZ-2
MBP3898921.1	*Mogibacterium sp.*	VanZ-2
MBQ8606491.1	*Clostridia bacterium*	VanZ-2
CEA04705.1	*Metalysinibacillus saudimassiliensis*	VanZ-2
HJC02064.1	*Candidatus Gemmiger stercoripullorum*	VanZ-2
WP_119631578.1	*Methylocaldum marinum*	VanZ-2
WP_104057671.1	*Jeotgalibacillus proteolyticus*	VanZ-2
NLW45703.1	*Syntrophomonadaceae bacterium*	VanZ-2
HJD22271.1	*Candidatus Gemmiger faecigallinarum*	VanZ-2
RIK75684.1	*Planctomycetota bacterium*	VanZ-2
MBQ8724503.1	*Oscillospiraceae bacterium*	VanZ-2
NME84290.1	*Clostridium sp. SM-530-WT-3G*	VanZ-2
HJB41438.1	*Candidatus Gemmiger avicola*	VanZ-2
WP_140753194.1	*Mesorhizobium sp. B4-1-3*	VanZ-2
HBR11608.1	*Chryseobacterium sp.*	VanZ-2
NOY54080.1	*Deltaproteobacteria bacterium*	VanZ-2
MBP3739048.1	*Muribaculaceae bacterium*	VanZ-2
WP_191203738.1		VanZ-2
MCD6518896.1	*Anaerolineae bacterium*	VanZ-2
MBQ6876279.1	*Lachnospiraceae bacterium*	VanZ-2
MBP3309249.1	*Ruminococcus sp.*	VanZ-2
WP_164473095.1	*Clostridium sp. E02*	VanZ-2
MBQ5953867.1	*Lachnospiraceae bacterium*	VanZ-2
MBN2208322.1	*Candidatus Coatesbacteria bacterium*	VanZ-2
MBO7610330.1	*Muribaculaceae bacterium*	VanZ-2
WP_075878154.1	*Merdibacter massiliensis*	VanZ-2
WP_118752070.1	*Clostridium sp. AM29-11AC*	VanZ-2
MBN1680378.1	*Anaerolineae bacterium*	VanZ-2
MBE6801418.1	*Oscillospiraceae bacterium*	VanZ-2
MBR2900900.1	*Clostridia bacterium*	VanZ-2
MBR1815981.1	*Lachnospiraceae bacterium*	VanZ-2
MBQ6546026.1	*Lachnospiraceae bacterium*	VanZ-2
MBM3885541.1	*Gemmatimonadota bacterium*	VanZ-2
RLC87341.1	*Chloroflexota bacterium*	VanZ-2
HJH11752.1	*Metalysinibacillus jejuensis*	VanZ-2
MBQ3140606.1	*Clostridia bacterium*	VanZ-2
CCY79542.1	*Mycoplasma sp. CAG:877*	VanZ-2
NLF21482.1	*Clostridiaceae bacterium*	VanZ-2
MBQ7063931.1	*Bacillota bacterium*	VanZ-2
MBR6595977.1	*Oscillospiraceae bacterium*	VanZ-2
ATU05502.1	*Candidatus Gracilibacteria bacterium HOT-871*	VanZ-2
MBP6890391.1	*Veillonella sp.*	VanZ-2
WP_223193071.1	*Paenibacillus sedimenti*	VanZ-2
GDY54324.1	*Streptomyces violaceusniger*	VanZ-2
SCI96088.1	*uncultured Ruminococcus sp.*	VanZ-2
WP_143276286.1	*Brachybacterium nesterenkovii*	VanZ-2
WP_003614434.1	*Methylosinus trichosporium OB3b*	VanZ-2
MBR4109012.1	*Oscillospiraceae bacterium*	VanZ-2
WP_130805623.1	*Senegalia massiliensis*	VanZ-2
WP_067121554.1	*Microbacterium sp. H83*	VanZ-2
MBQ9360427.1	*Lachnospiraceae bacterium*	VanZ-2
WP_026906798.1	*Paucisalibacillus globulus DSM 18846*	VanZ-2
HCW52726.1	*Clostridium sp.*	VanZ-2
CCX93814.1	*Firmicutes bacterium CAG:110*	VanZ-2
MBQ8689083.1	*Clostridia bacterium*	VanZ-2
MBE6836203.1	*Oscillospiraceae bacterium*	VanZ-2
MBR6120327.1	*Oscillospiraceae bacterium*	VanZ-2
MBR4881932.1	*Clostridia bacterium*	VanZ-2
WP_162911939.1	*Streptococcus respiraculi*	VanZ-2
MBR5447962.1	*Clostridia bacterium*	VanZ-2
PYN60833.1	*Candidatus Rokuibacteriota bacterium*	VanZ-2
MBR5314118.1	*Clostridia bacterium*	VanZ-2
WP_213620428.1		VanZ-2
AIY03628.1	*Arthrobacter sp. PAMC 25486*	VanZ-2
PYM77439.1	*Candidatus Rokuibacteriota bacterium*	VanZ-2
MBO4338916.1	*Clostridia bacterium*	VanZ-2
MCD6284244.1	*bacterium*	VanZ-2
MBM4315205.1	*Deltaproteobacteria bacterium*	VanZ-2
MBO5169633.1	*Oscillospiraceae bacterium*	VanZ-2
MBR5529579.1	*Oscillospiraceae bacterium*	VanZ-2
MBL7003706.1	*Gammaproteobacteria bacterium*	VanZ-2
WP_002610101.1	*[Clostridium] innocuum*	VanZ-2
MBR3319651.1	*Candidatus Saccharibacteria bacterium*	VanZ-2
MBO5110065.1	*Clostridia bacterium*	VanZ-2
MBN2107207.1	*Deltaproteobacteria bacterium*	VanZ-2
MBR5389093.1	*Candidatus Saccharibacteria bacterium*	VanZ-2
WP_128215770.1	*Clostridium manihotivorum*	VanZ-2
WP_144844855.1	*Paenibacillus cremeus*	VanZ-2
MBM6967920.1	*Pseudoramibacter alactolyticus*	VanZ-2
MBI5788116.1	*Candidatus Schekmanbacteria bacterium*	VanZ-2
MBX3727799.1	*Candidatus Sumerlaeia bacterium*	VanZ-2
MBT5620698.1	*Verrucomicrobiota bacterium*	VanZ-2
WP_117419471.1	*Lacrimispora amygdalina*	VanZ-2
TWU36140.1	*Novipirellula artificiosorum*	VanZ-2
HIQ68655.1	*Candidatus Faecousia excrementigallinarum*	VanZ-2

WP_191751312.1	*Clostridium gallinarum*	VanZ-2
PIQ24586.1	*bacterium (Candidatus Blackallbacteria) CG18_big_fil_WC_8_21_14_2_50_49_26*	VanZ-2
EEG29272.1	*[Clostridium] methylpentosum DSM 5476*	VanZ-2
MBQ7781247.1	*Lachnospiraceae bacterium*	VanZ-2
WP_184677038.1	*Algisphaera agarilytica*	VanZ-2
WP_010532494.1	*Lentibacillus jeotgali*	VanZ-2
MBS5330942.1	*Subdoligranulum variabile*	VanZ-2
HGQ52720.1		VanZ-2
PWL89059.1	*Escherichia coli*	VanZ-2
WP_204908662.1		VanZ-2
MBF0433717.1	*Fibrobacteria bacterium*	VanZ-2
WP_212695105.1	*Vallitalea pronyensis*	VanZ-2
MBQ8496821.1	*Clostridia bacterium*	VanZ-2
PKM88851.1	*Firmicutes bacterium HGW-Firmicutes-12*	VanZ-2
WP_090236959.1	*Lentibacillus halodurans*	VanZ-2
RZD38504.1	*Methanobacteriota archaeon*	VanZ-2
MCC8091021.1	*Oscillospiraceae bacterium*	VanZ-2
WP_117679817.1	*Collinsella tanakaei*	VanZ-2
MBE7059395.1		VanZ-2
SEI91984.1	*Bhargavaea ginsengi*	VanZ-2
MBO5316968.1	*Oscillospiraceae bacterium*	VanZ-2
MBR5368671.1	*Lachnospiraceae bacterium*	VanZ-2
MBO5837209.1	*Oscillospiraceae bacterium*	VanZ-2
MSO24196.1	*Acidobacteriota bacterium*	VanZ-2
WP_195419681.1	*Collinsella sp. D33t1_170424_A12*	VanZ-2
MBI2495404.1	*Candidatus Omnitrophota bacterium*	VanZ-2
NLA76262.1	*Clostridiales bacterium*	VanZ-2
NLB38036.1	*Clostridiales bacterium*	VanZ-2
WP_026890659.1	*[Clostridium] aerotolerans DSM 5434*	VanZ-2
MBO5869026.1	*Oscillospiraceae bacterium*	VanZ-2
MBS6842859.1	*Clostridiales bacterium*	VanZ-2
WP_156281297.1	*Paenibacillus sp. NEAU-GSW1*	VanZ-2
MBS5873107.1	*Clostridiales bacterium*	VanZ-2
NLP34123.1	*Clostridiales bacterium*	VanZ-2
TDQ55949.1	*Phaeovulum veldkampii DSM 11550*	VanZ-2
MBS5618811.1	*Clostridiales bacterium*	VanZ-2
HHX56330.1	*Clostridiales bacterium*	VanZ-2
NQS77043.1	*Candidatus Syntrophopropionicum ammoniitolerans*	VanZ-2
MBU6143912.1	*Acidobacteriota bacterium*	VanZ-2
HHZ14427.1	*Clostridiales bacterium*	VanZ-2
RHF37630.1	*Thomasclavelia ramosa*	VanZ-2
MBP3338535.1	*Lachnospiraceae bacterium*	VanZ-2
WP_008825591.1	*Haloplasma contractile SSD-17B*	VanZ-2
MBP3878534.1	*Lachnospiraceae bacterium*	VanZ-2
OAB45182.1	*Paenibacillus antarcticus*	VanZ-2
MBR6880928.1	*Clostridiales bacterium*	VanZ-2
MBV9083288.1	*Acidobacteriaceae bacterium*	VanZ-2
MBV8809672.1	*Acidobacteriaceae bacterium*	VanZ-2
MBQ8234474.1	*Bacilli bacterium*	VanZ-2
MBP5608680.1	*Lachnospiraceae bacterium*	VanZ-2
NJM78306.1	*Acaryochloridaceae cyanobacterium RU_4_10*	VanZ-2
CAB5091893.1	*Olavius algarvensis associated proteobacterium Delta 3*	VanZ-2
MBR4618506.1	*Bacilli bacterium*	VanZ-2
MBP1591111.1	*Oscillospiraceae bacterium*	VanZ-2
MCC6106822.1	*Atopobium sp.*	VanZ-2
MBP8968526.1	*Lachnospiraceae bacterium*	VanZ-2
MBA4808496.1	*Brevundimonas sp.*	VanZ-2
WP_144743202.1	*Enorma burkinafasonensis*	VanZ-2
NCD06751.1	*Spirochaetia bacterium*	VanZ-2
MBX3339833.1	*Nitrospira sp.*	VanZ-2
WP_204671037.1	*Enorma phocaeensis*	VanZ-2
MBN1602328.1	*Chitinispirillaceae bacterium*	VanZ-2
RMH37585.1	*Nitrospirota bacterium*	VanZ-2
WP_016213785.1	*Eubacterium sp. 14-2*	VanZ-2
WP_153406657.1	*Gracilibacillus oryzae*	VanZ-2
WP_163538620.1	*Gracilibacillus sp. YIM 98692*	VanZ-2
MBN2913530.1	*Oscillospiraceae bacterium*	VanZ-2
MBA2487129.1	*Nitrospira sp.*	VanZ-2
RZD37532.1	*Methanobacteriota archaeon*	VanZ-2
MBP5600971.1	*Lachnospiraceae bacterium*	VanZ-2
MBE6921985.1	*Oscillospiraceae bacterium*	VanZ-2
MBP5553504.1	*Lachnospiraceae bacterium*	VanZ-2
MBI3609967.1	*Nitrospirota bacterium*	VanZ-2
OPY14604.1	*Syntrophus sp. PtaB.Bin138*	VanZ-2
MCA9531264.1	*Myxococcales bacterium*	VanZ-2
KQY23227.1	*Cellulomonas sp. Root485*	VanZ-2
MBZ5536005.1	*Terriglobia bacterium*	VanZ-2
MBR1709894.1	*Clostridia bacterium*	VanZ-2
NLX25000.1	*Lentisphaerota bacterium*	VanZ-2
PKN68404.1	*Deltaproteobacteria bacterium HGW-Deltaproteobacteria-12*	VanZ-2
WP_127579812.1	*Paenibacillus koleovorans NBRC 103111*	VanZ-2
MBS5002142.1	*Holdemania filiformis*	VanZ-2
WP_025803272.1	*Campylobacter corcagiensis*	VanZ-2
MBI4584590.1	*Planctomycetota bacterium*	VanZ-2
MBD5087546.1	*Clostridiales bacterium*	VanZ-2
MBT3468036.1	*Opitutia bacterium*	VanZ-2
MBP0981230.1	*Oscillospiraceae bacterium*	VanZ-2
MBO5953688.1	*Oscillospiraceae bacterium*	VanZ-2
MBO6148836.1	*Lachnospiraceae bacterium*	VanZ-2
MBK6796450.1	*Acidobacteriota bacterium*	VanZ-2
QTA83644.1	*Desulfonema limicola*	VanZ-2
MBQ3902674.1	*Lachnospiraceae bacterium*	VanZ-2
WP_183192440.1	*Brevibacillus fluminis*	VanZ-2

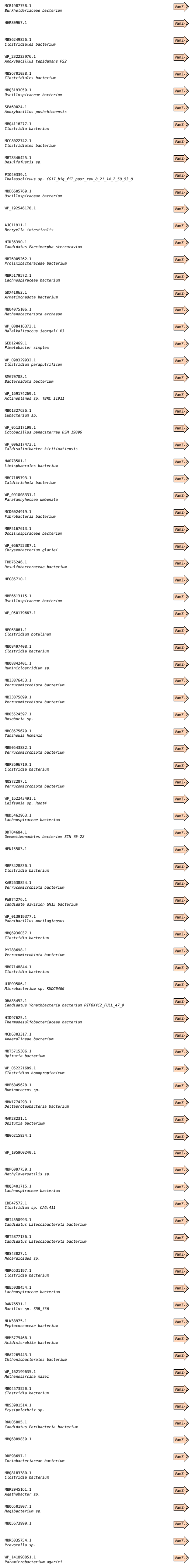
MCB1987758.1
Burkholderiaceae bacterium
VanZ-2
HHR80967.1
VanZ-2
MBS6249826.1
Clostridiales bacterium
VanZ-2
WP_232223976.1
Anoxybacillus tepidamans PS2
VanZ-2
MBS6701038.1
Clostridiales bacterium
VanZ-2
MBQ3193059.1
Oscillospiraceae bacterium
VanZ-2
SFA60024.1
Anoxybacillus pushchinoensis
VanZ-2
MBQ4116277.1
Clostridia bacterium
VanZ-2
MCC8022742.1
Clostridiales bacterium
VanZ-2
MBT8346425.1
Desulfofustis sp.
VanZ-2
PIQ40339.1
Thalassolituus sp. CG17_big_fil_post_rev_8_21_14_2_50_53_8
VanZ-2
MBE6605769.1
Oscillospiraceae bacterium
VanZ-2
WP_192546178.1
VanZ-2
AJC11911.1
Berryella intestinalis
VanZ-2
HIR36390.1
Candidatus Faecimorpha stercoravium
VanZ-2
MBT6005262.1
Prolixibacteraceae bacterium
VanZ-2
MBR5179572.1
Lachnospiraceae bacterium
VanZ-2
GDX41062.1
Armatimonadota bacterium
VanZ-2
MBU4075106.1
Methanobacteriota archaeon
VanZ-2
WP_008416373.1
Halalkalicoccus jeotgali B3
VanZ-2
GEB12469.1
Pimelobacter simplex
VanZ-2
WP_099329932.1
Clostridium paraputrificum
VanZ-2
RMG70708.1
Bacteroidota bacterium
VanZ-2
WP_169174269.1
Actinoplanes sp. TBRC 11911
VanZ-2
MBQ1327636.1
Eubacterium sp.
VanZ-2
WP_051317199.1
Ectobacillus panaciterrae DSM 19096
VanZ-2
WP_006317473.1
Caldisalinibacter kiritimatiensis
VanZ-2
HAO78501.1
Limisphaerales bacterium
VanZ-2
MBC7185793.1
Calditrichota bacterium
VanZ-2
WP_091008331.1
Parafannyhessea umbonata
VanZ-2
MCD6024919.1
Fibrobacteria bacterium
VanZ-2
MBP5167613.1
Oscillospiraceae bacterium
VanZ-2
WP_066752387.1
Chryseobacterium glaciei
VanZ-2
THB76246.1
Desulfobacteraceae bacterium
VanZ-2
HEG85710.1
VanZ-2
MBE6613115.1
Oscillospiraceae bacterium
VanZ-2
WP_050179663.1
VanZ-2
NFG63061.1
Clostridium botulinum
VanZ-2
MBQ8497408.1
Clostridia bacterium
VanZ-2
MBQ8842401.1
Ruminiclostridium sp.
VanZ-2
MBI3876453.1
Verrucomicrobiota bacterium
VanZ-2
MBI3875899.1
Verrucomicrobiota bacterium
VanZ-2
MBO5524597.1
Roseburia sp.
VanZ-2
MBC8575679.1
Yanshouia hominis
VanZ-2
MBE0543882.1
Verrucomicrobiota bacterium
VanZ-2
MBP3696719.1
Clostridia bacterium
VanZ-2
NOS72207.1
Verrucomicrobiota bacterium
VanZ-2
WP_162243491.1
Leifsonia sp. Root4
VanZ-2
MBD5462963.1
Lachnospiraceae bacterium
VanZ-2
ODT04684.1
Gemmatimonadetes bacterium SCN 70-22
VanZ-2
HEN15503.1
VanZ-2
MBP3428830.1
Clostridia bacterium
VanZ-2
KAB2638854.1
Verrucomicrobiota bacterium
VanZ-2
PWB74276.1
candidate division GN15 bacterium
VanZ-2
WP_013919377.1
Paenibacillus mucilaginosus
VanZ-2
MBQ6936037.1
Clostridia bacterium
VanZ-2
PYI88698.1
Verrucomicrobiota bacterium
VanZ-2
MBO7148844.1
Clostridia bacterium
VanZ-2
UJP09506.1
Microbacterium sp. KUDC0406
VanZ-2
OHA85452.1
Candidatus Yonathbacteria bacterium RIFOXYC2_FULL_47_9
VanZ-2
HID97625.1
Thermodesulfobacteriaceae bacterium
VanZ-2
MCD6303317.1
Anaerolineae bacterium
VanZ-2
MBT5715306.1
Opitutia bacterium
VanZ-2
WP_052221689.1
Clostridium homopropionicum
VanZ-2
MBE6845628.1
Ruminococcus sp.
VanZ-2
MBW1774293.1
Deltaproteobacteria bacterium
VanZ-2
MAK28231.1
Opitutia bacterium
VanZ-2
MBG6215824.1
VanZ-2
WP_105960240.1
VanZ-2
MBP6097759.1
Methyloversatilis sp.
VanZ-2
MBQ3401715.1
Lachnospiraceae bacterium
VanZ-2
CDE47572.1
Clostridium sp. CAG:411
VanZ-2
MBI4550993.1
Candidatus Latescibacterota bacterium
VanZ-2
MBT5877136.1
Candidatus Latescibacterota bacterium
VanZ-2
MBS43027.1
Nocardioides sp.
VanZ-2
MBR6531197.1
Clostridia bacterium
VanZ-2
MBE5938454.1
Lachnospiraceae bacterium
VanZ-2
RAN76531.1
Bacillus sp. SRB_336
VanZ-2
NLW38975.1
Peptococcaceae bacterium
VanZ-2
MBM3779468.1
Acidimicrobiia bacterium
VanZ-2
MBA2269443.1
Chthoniobacterales bacterium
VanZ-2
WP_162199635.1
Methanosarcina mazei
VanZ-2
MBQ4573520.1
Clostridia bacterium
VanZ-2
MBS3991514.1
Erysipelothrix sp.
VanZ-2
RKU05805.1
Candidatus Poribacteria bacterium
VanZ-2
MBQ6889839.1
VanZ-2
RRF98697.1
Coriobacteriaceae bacterium
VanZ-2
MBQ8183380.1
Clostridia bacterium
VanZ-2
MBR2045161.1
Agathobacter sp.
VanZ-2
MBQ6501807.1
Mogibacterium sp.
VanZ-2
MBQ5673999.1
VanZ-2
MBR5035754.1
Prevotella sp.
VanZ-2
WP_141898851.1
Paramicrobacterium agarici
VanZ-2

Accession	Organism	Gene
WP_076708681.1	*Microbacterium oleivorans*	VanZ-2
MBQ8858212.1	*Clostridia bacterium*	VanZ-2
MBR1738375.1	*Bacillota bacterium*	VanZ-2
MBI4856666.1	*Acetobacterium woodii*	VanZ-2
WP_090855401.1	*Litchfieldia salsa*	VanZ-2
WP_066299163.1	*Arthrobacter luteolus*	VanZ-2
MCD8288414.1	*Prevotella sp.*	VanZ-2
MBR3025940.1	*Oscillospiraceae bacterium*	VanZ-2
NLD72847.1	*Chloroflexota bacterium*	VanZ-2
TMA56874.1	*Deltaproteobacteria bacterium*	VanZ-2
ERH12934.1	*halophilic archaeon J07HB67*	VanZ-2
MBR3949992.1	*Oscillospiraceae bacterium*	VanZ-2
MBS5950512.1	*Clostridium sp.*	VanZ-2
WP_117518593.1	*Absiella sp. AM29-15*	VanZ-2
MBQ8503446.1	*Clostridia bacterium*	VanZ-2
MBR2311148.1	*Oscillospiraceae bacterium*	VanZ-2
MBS3972791.1	*Erysipelotrichia bacterium*	VanZ-2
MBP3314201.1	*Clostridia bacterium*	VanZ-2
MBQ1917002.1	*Lachnospiraceae bacterium*	VanZ-2
GJE59445.1	*Methylobacterium trifolii*	VanZ-2
ESS03591.1	*uncultured archaeon A07HR67*	VanZ-2
HHT42230.1	*Bacillota bacterium*	VanZ-2
MBQ8510920.1	*Clostridia bacterium*	VanZ-2
MBQ8617854.1	*Clostridia bacterium*	VanZ-2
NNK14293.1	*Desulfofustis sp.*	VanZ-2
MBN1202425.1	*Anaerolineae bacterium*	VanZ-2
SDI41195.1	*Natribacillus halophilus*	VanZ-2
MCB0185592.1	*Caldilineaceae bacterium*	VanZ-2
MBQ8474728.1	*Clostridia bacterium*	VanZ-2
MBR3809634.1	*Clostridia bacterium*	VanZ-2
WP_193726879.1	*Paenibacillus guangzhouensis*	VanZ-2
WP_193762061.1	*Microbacterium oxydans*	VanZ-2
WP_163179781.1	*Bacillus mesophilus*	VanZ-2
MBV8555372.1	*Planctomycetaceae bacterium*	VanZ-2
MCA8997908.1	*Planctomycetaceae bacterium*	VanZ-2
MBL6427632.1	*Maritimibacter sp.*	VanZ-2
SBS73510.1	*uncultured Microbacterium sp.*	VanZ-2
MBQ3782385.1	*Lachnospiraceae bacterium*	VanZ-2
MBO9541585.1	*bacterium*	VanZ-2
MBS1251108.1	*Anaerolineales bacterium*	VanZ-2
MBR7185045.1	*Clostridia bacterium*	VanZ-2
WP_223722639.1	*Microbacterium sp. cx-55*	VanZ-2
MBQ7857375.1	*Oscillospiraceae bacterium*	VanZ-2
AEB08897.1	*Desulfobacca acetoxidans DSM 11109*	VanZ-2
MBQ8076242.1	*Oscillospiraceae bacterium*	VanZ-2
WP_183339115.1	*Microlunatus antarcticus*	VanZ-2
AWK50143.1	*Clostridium beijerinckii*	VanZ-2
MCD6286789.1	*Anaerolineae bacterium*	VanZ-2
WP_131172824.1	*Propioniciclava tarda*	VanZ-2
TEU14172.1	*Anaerolineales bacterium*	VanZ-2
MCA9261189.1	*Planctomycetales bacterium*	VanZ-2
WP_100161475.1	*Pseudooceanicola sp.*	VanZ-2
WP_170100215.1	*Trichococcus patagoniensis*	VanZ-2
MBQ7964890.1	*Ruminococcus sp.*	VanZ-2
MBQ6353677.1	*Lachnospiraceae bacterium*	VanZ-2
WP_104055256.1	*Arthrobacter sp. 9E16*	VanZ-2
MBO7176852.1	*Clostridia bacterium*	VanZ-2
MBQ7801963.1	*Oscillospiraceae bacterium*	VanZ-2
MBR3096181.1	*Clostridia bacterium*	VanZ-2
MBQ5565518.1	*Clostridia bacterium*	VanZ-2
MBQ6386455.1	*Lachnospiraceae bacterium*	VanZ-2
MBQ8909724.1	*Oscillospiraceae bacterium*	VanZ-2
MBQ1567906.1	*Erysipelotrichaceae bacterium*	VanZ-2
MCD8218939.1	*Ruminococcus sp.*	VanZ-2
MRX54227.1	*Metabacillus idriensis*	VanZ-2
HGP68391.1		VanZ-2
MCA9039194.1		VanZ-2
MCA9049419.1	*Planctomycetaceae bacterium*	VanZ-2
WP_101521347.1	*Kineobactrum sediminis*	VanZ-2
MBQ8001649.1	*Ruminococcus sp.*	VanZ-2
WP_119850457.1	*Paenibacillus lautus*	VanZ-2
MBQ4472677.1	*Lachnospiraceae bacterium*	VanZ-2
HID08531.1	*Armatimonadota bacterium*	VanZ-2
MBN2264570.1	*Candidatus Aminicenantes bacterium*	VanZ-2
PYR26694.1	*Acidobacteriota bacterium*	VanZ-2
MBI4753675.1	*Candidatus Desantisbacteria bacterium*	VanZ-2
MBP7962911.1	*Caldilineaceae bacterium*	VanZ-2
NBR65070.1	*Actinomycetota bacterium*	VanZ-2
RJP78928.1	*Candidatus Zixiibacteriota bacterium*	VanZ-2
MBX5493261.1	*Chloroflexota bacterium*	VanZ-2
MBD3177907.1	*Armatimonadia bacterium*	VanZ-2
MBE0644040.1	*Bacteroidota bacterium*	VanZ-2
RZM08825.1	*Sphingomonas sp.*	VanZ-2
MBQ4342899.1	*Erysipelotrichaceae bacterium*	VanZ-2
HCY89066.1	*Chitinophagaceae bacterium*	VanZ-2
WP_119386283.1		VanZ-2
MBT5018270.1	*Planctomicrobium sp.*	VanZ-2
WP_116418014.1	*Subtercola boreus*	VanZ-2
MBS6602005.1	*Clostridium sp.*	VanZ-2
WP_184715571.1	*Streptosporangium saharense*	VanZ-2
EFI41577.1	*Peptoniphilus sp. oral taxon 386 str. F0131*	VanZ-2
AIE85699.1	*Fimbriimonas ginsengisoli Gsoil 348*	VanZ-2
HGF89681.1		VanZ-2

MBQ7825637.1	*Clostridia bacterium*	VanZ-2
HCG98525.1	*Actinomycetota bacterium*	VanZ-2
WP_163948611.1	*Paenibacillus sp. SYP-B3998*	VanZ-2
NQU92507.1	*Bacteroidota bacterium*	VanZ-2
WP_119072180.1	*Aggregatilinea lenta*	VanZ-2
HIS60309.1	*Candidatus Faecousia faecipullorum*	VanZ-2
MAP63515.1	*Microbacterium sp.*	VanZ-2
MBQ1843842.1	*Lachnospiraceae bacterium*	VanZ-2
MBQ8056975.1	*Ruminococcus sp.*	VanZ-2
WP_016205155.1		VanZ-2
CDD53200.1	*Ruminococcus sp. CAG:379*	VanZ-2
MBF0627225.1	*Magnetococcales bacterium*	VanZ-2
WP_213493777.1	*Telmatocola sphagniphila*	VanZ-2
NCU25594.1	*Candidatus Nomurabacteria bacterium*	VanZ-2
MBU6276871.1	*Planctomycetota bacterium*	VanZ-2
MBP1980460.1		VanZ-2
MBM3216037.1	*Candidatus Poribacteria bacterium*	VanZ-2
MBD9035003.1	*Prevotella sp.*	VanZ-2
MBR5868018.1	*Clostridia bacterium*	VanZ-2
MBI2999506.1	*Deltaproteobacteria bacterium*	VanZ-2
MBR6413931.1	*Oscillospiraceae bacterium*	VanZ-2
MYE32856.1	*Chloroflexota bacterium*	VanZ-2
MBR5518373.1	*Clostridia bacterium*	VanZ-2
NQW18410.1	*Chloroflexota bacterium*	VanZ-2
PYN92541.1	*Candidatus Rokuibacteriota bacterium*	VanZ-2
MBS1911669.1	*Bacteroidota bacterium*	VanZ-2
RAY12976.1	*Actinomadura craniellae*	VanZ-2
MBO5921048.1	*Bacteroidaceae bacterium*	VanZ-2
MBN9200177.1	*Microbacterium aurantiacum*	VanZ-2
HBJ76490.1	*Porphyromonadaceae bacterium*	VanZ-2
MBR0133957.1	*Candidatus Saccharibacteria bacterium*	VanZ-2
MBR1939124.1	*Candidatus Saccharibacteria bacterium*	VanZ-2
MCC7145877.1	*Phycisphaeraceae bacterium*	VanZ-2
WP_224129287.1	*Nocardioides sp. TRM66260-LWL*	VanZ-2
MBR6134128.1	*Candidatus Saccharibacteria bacterium*	VanZ-2
MBM3333213.1	*Candidatus Sumerlaeota bacterium*	VanZ-2
MBQ7940636.1	*Clostridia bacterium*	VanZ-2
MCD6386462.1	*Candidatus Sumerlaeota bacterium*	VanZ-2
WP_193308553.1	*Halorubrum halophilum*	VanZ-2
WP_075217588.1	*Mongoliimonas terrestris*	VanZ-2
WP_145056214.1	*Lignipirellula cremea*	VanZ-2
MBR5121762.1	*Oscillospiraceae bacterium*	VanZ-2
HGU00732.1		VanZ-2
HJB26585.1	*Bacillota bacterium*	VanZ-2
KHF39292.1	*Halalkalibacter okhensis*	VanZ-2
CAA9246011.1	*uncultured Cytophagales bacterium*	VanZ-2
MBQ7668711.1	*Clostridia bacterium*	VanZ-2
MBR2778883.1	*Bacillota bacterium*	VanZ-2
MBO5128947.1	*Oscillospiraceae bacterium*	VanZ-2
HGV67495.1		VanZ-2
MBQ5995120.1	*Clostridia bacterium*	VanZ-2
MBP9581221.1	*Ignavibacterium sp.*	VanZ-2
MBQ7047752.1	*Clostridia bacterium*	VanZ-2
MBQ7116509.1	*Clostridia bacterium*	VanZ-2
MBP3523664.1	*Clostridia bacterium*	VanZ-2
MBO5836821.1	*Oscillospiraceae bacterium*	VanZ-2
MBO6301058.1	*Ruminiclostridium sp.*	VanZ-2
HIU48490.1	*Candidatus Avimonoglobus intestinipullorum*	VanZ-2
MBP3656224.1	*Clostridia bacterium*	VanZ-2
MBA4148510.1	*Verrucomicrobiota bacterium*	VanZ-2
WP_098996035.1	*Bacillus sp. AFS037270*	VanZ-2
MBO5475865.1	*Bacilli bacterium*	VanZ-2
MBP5193976.1	*Clostridia bacterium*	VanZ-2
WP_051075123.1		VanZ-2
MBQ6273535.1	*Oscillospiraceae bacterium*	VanZ-2
MBI5775540.1	*Verrucomicrobiota bacterium*	VanZ-2
GFI43420.1	*Lachnospiraceae bacterium*	VanZ-2
MBQ9975308.1	*Clostridia bacterium*	VanZ-2
OGP85826.1	*Deltaproteobacteria bacterium RBG_16_58_17*	VanZ-2
HBT19530.1	*Clostridiaceae bacterium*	VanZ-2
WP_215635674.1	*Clostridium baratii*	VanZ-2
MCD6050031.1	*Verrucomicrobiota bacterium*	VanZ-2
HFW62296.1		VanZ-2
MBE6683874.1	*Oscillospiraceae bacterium*	VanZ-2
MBP5165802.1	*Oscillospiraceae bacterium*	VanZ-2
NLD46037.1	*Clostridiaceae bacterium*	VanZ-2
HHI34403.1		VanZ-2
MBR2464660.1	*Clostridia bacterium*	VanZ-2
MBQ2932397.1	*Clostridia bacterium*	VanZ-2
MBC8062723.1	*Clostridiaceae bacterium*	VanZ-2
PYU02467.1	*Acidobacteriota bacterium*	VanZ-2
NMM23952.1	*Phycicoccus sp.*	VanZ-2
TAE17266.1	*Bacteroidota bacterium*	VanZ-2
WP_061858826.1	*Clostridium thermopalmarium*	VanZ-2
MBQ2853888.1	*Oscillospiraceae bacterium*	VanZ-2
KAA3613778.1	*Calditrichota bacterium*	VanZ-2
KAA3662577.1	*Calditrichota bacterium*	VanZ-2
WP_008908085.1	*Caloramator australicus RC3*	VanZ-2
PYQ38617.1	*Acidobacteriota bacterium*	VanZ-2
WP_141190936.1	*Terrilactibacillus laevilacticus*	VanZ-2
MBQ3888312.1	*Clostridia bacterium*	VanZ-2
MAD23532.1	*Verrucomicrobiales bacterium*	VanZ-2
MBE6741545.1	*Oscillospiraceae bacterium*	VanZ-2

HEQ79025.1	*Methanobacteriota archaeon*	VanZ-2
MBO5440371.1	*Clostridia bacterium*	VanZ-2
WP_181347891.1	*Thalassobacillus sp. CUG 92003*	VanZ-2
OKZ69930.1	*Clostridiales bacterium 52_15*	VanZ-2
HIR24904.1	*Candidatus Egerieimonas faecigallinarum*	VanZ-2
NOT46472.1	*Acidobacteriota bacterium*	VanZ-2
MCE3275785.1	*Propionibacteriaceae bacterium*	VanZ-2
MBN1537308.1	*Anaerolineales bacterium*	VanZ-2
HHX22719.1	*Thermoanaerobacterales bacterium*	VanZ-2
HJC21581.1	*Candidatus Butyricicoccus avicola*	VanZ-2
HIH44997.1	*Candidatus Methanoperedenaceae archaeon*	VanZ-2
QTD51249.1	*Sulfidibacter corallicola*	VanZ-2
NLH00673.1	*Clostridiales bacterium*	VanZ-2
MBR2340506.1	*Clostridia bacterium*	VanZ-2
RJL36187.1	*Bailinhaonella thermotolerans*	VanZ-2
NQU94849.1	*Candidatus Omnitrophota bacterium*	VanZ-2
WP_028822305.1	*Propionimicrobium lymphophilum DSM 4903*	VanZ-2
MBQ6789559.1	*Clostridia bacterium*	VanZ-2
NLL31471.1	*Clostridiales bacterium*	VanZ-2
MBO5484064.1	*Lachnospiraceae bacterium*	VanZ-2
HIU95066.1	*Candidatus Aphodomorpha intestinavium*	VanZ-2
ANU63826.1	*Muribaculum intestinale*	VanZ-2
MBQ5800331.1	*Clostridia bacterium*	VanZ-2
MBS5794048.1	*Clostridiales bacterium*	VanZ-2
MBP5135301.1	*Paludibacteraceae bacterium*	VanZ-2
MBE6796009.1	*Oscillospiraceae bacterium*	VanZ-2
REJ75358.1	*Acidobacteriota bacterium*	VanZ-2
NLW73280.1	*Clostridiales bacterium*	VanZ-2
MBQ9171851.1	*Candidatus Saccharibacteria bacterium*	VanZ-2
WP_106507089.1	*Brachybacterium timonense*	VanZ-2
WP_232236042.1	*Fannyhessea vaginae*	VanZ-2
WP_025689869.1	*Paenibacillus zanthoxyli JH29*	VanZ-2
WP_163253086.1	*Clostridium sporogenes*	VanZ-2
HHT54456.1	*Clostridiales bacterium*	VanZ-2
OGW46726.1	*Nitrospirae bacterium RBG_13_41_22*	VanZ-2
PKB65067.1	*SAR202 cluster bacterium Io17-Chloro-G2*	VanZ-2
MSQ83718.1	*Myxococcales bacterium*	VanZ-2
WP_051292621.1	*Olivibacter sitiensis DSM 17696*	VanZ-2
MBX5440410.1	*Solirubrobacteraceae bacterium*	VanZ-2
QQS46406.1	*Acidobacteriota bacterium*	VanZ-2
WP_224811323.1		VanZ-2
MBQ1224195.1	*Oscillospiraceae bacterium*	VanZ-2
NLC76013.1	*Clostridia bacterium*	VanZ-2
WP_088249235.1		VanZ-2
MBO5248659.1	*Clostridia bacterium*	VanZ-2
MBQ8773766.1	*Muribaculaceae bacterium*	VanZ-2
WP_204590544.1	*Chryseomicrobium aureum*	VanZ-2
WP_169360592.1	*Psychrobacillus sp. BL-248-WT-3*	VanZ-2
AUD65249.1	*Tenericutes bacterium MZ-XQ*	VanZ-2
MBQ3306664.1	*Bacilli bacterium*	VanZ-2
MBQ5412774.1	*Oscillospiraceae bacterium*	VanZ-2
MBQ0120081.1	*Candidatus Sodaliphilus limicaballi*	VanZ-2
MBV9301538.1	*Acidobacteriaceae bacterium*	VanZ-2
WP_106210644.1	*Glaciihabitans tibetensis*	VanZ-2
WP_118918980.1	*Neobacillus notoginsengisoli*	VanZ-2
MBS7642295.1	*Candidatus Bathyarchaeota archaeon*	VanZ-2
MBM4147325.1	*Nitrospira sp.*	VanZ-2
MCD7737832.1	*Lachnospiraceae bacterium*	VanZ-2
MBQ7790152.1	*Bacilli bacterium*	VanZ-2
OUU39171.1	*Verrucomicrobia bacterium TMED56*	VanZ-2
MBO5265578.1	*Ruminococcus sp.*	VanZ-2
MBQ6651003.1	*Atopobiaceae bacterium*	VanZ-2
WP_087430297.1	*Collinsella sp. An307*	VanZ-2
WP_204566048.1		VanZ-2
MBQ5512871.1	*Bacteroidales bacterium*	VanZ-2
MBP3674295.1	*Oscillospiraceae bacterium*	VanZ-2
MBP0969065.1	*Oscillospiraceae bacterium*	VanZ-2
HIV68318.1	*Candidatus Butyricicoccus stercorigallinarum*	VanZ-2
WP_011324044.1	*Natronomonas pharaonis DSM 2160*	VanZ-2
MBR4656323.1	*Oscillospiraceae bacterium*	VanZ-2
WP_051348734.1	*Peribacillus kribbensis DSM 17871*	VanZ-2
MBQ9168432.1	*Oscillospiraceae bacterium*	VanZ-2
PKQ62350.1	*Labilibaculum manganireducens*	VanZ-2
HET76444.1		VanZ-2
WP_163290950.1	*Diaminobutyricibacter tongyongensis*	VanZ-2
MBD5371004.1	*Bacteroides sp.*	VanZ-2
WP_006039455.1	*Paenibacillus curdlanolyticus YK9*	VanZ-2
MBQ0155817.1	*Candidatus Scybalocola fimicaballi*	VanZ-2
ATX79377.1	*Mariprofundus aestuarium*	VanZ-2
MBQ6625650.1	*Clostridia bacterium*	VanZ-2
KGG79630.1	*Caloranaerobacter azorensis H53214*	VanZ-2
MBQ4609532.1	*Clostridia bacterium*	VanZ-2
MBQ4155748.1	*Clostridia bacterium*	VanZ-2
WP_080062592.1	*Ruminiclostridium hungatei*	VanZ-2
MBQ3126979.1	*Clostridia bacterium*	VanZ-2
OGW29761.1	*Nitrospirae bacterium GWC2_42_7*	VanZ-2
MBD8962074.1	*Oscillospiraceae bacterium*	VanZ-2
MXR70753.1	*Shewanella insulae*	VanZ-2
MBI5187071.1	*Nitrospinota bacterium*	VanZ-2
NLH59532.1	*Clostridiales bacterium*	VanZ-2
MBS7364471.1	*Paludibacteraceae bacterium*	VanZ-2
HBL40495.1	*Oscillospiraceae bacterium*	VanZ-2
NRB79549.1	*Saccharospirillaceae bacterium*	VanZ-2

MBQ7088158.1	*Clostridia bacterium*	VanZ-2
MBX3194352.1	*Microbacteriaceae bacterium*	VanZ-2
MBQ9551455.1	*Clostridia bacterium*	VanZ-2
WP_066368676.1	*Neobacillus fumarioli NBRC 102428*	VanZ-2
NOR71288.1	*Methylomarinum sp.*	VanZ-2
WP_197025981.1	*Nocardioides sp. URHA0020*	VanZ-2
MBR2942577.1	*Clostridia bacterium*	VanZ-2
MBR2474446.1	*Clostridia bacterium*	VanZ-2
NLK23005.1	*Clostridiales bacterium*	VanZ-2
SPT79246.1	*Niallia circulans*	VanZ-2
MBQ8067671.1	*Solobacterium sp.*	VanZ-2
MBK9468316.1	*Gammaproteobacteria bacterium*	VanZ-2
HIY66210.1	*Candidatus Agrococcus pullicola*	VanZ-2
MBR6562996.1	*Clostridia bacterium*	VanZ-2
HJF51298.1	*Butyricicoccus pullicaecorum*	VanZ-2
MBO5195730.1	*Clostridia bacterium*	VanZ-2
GGB55183.1	*Lentibacillus populi*	VanZ-2
MBQ9083773.1	*Clostridia bacterium*	VanZ-2
NLX22573.1	*Phycisphaerae bacterium*	VanZ-2
MBO0851419.1	*Pseudonocardia sp.*	VanZ-2
WP_191282381.1	*Pseudolysinimonas yzui*	VanZ-2
MBP3939349.1	*Clostridia bacterium*	VanZ-2
NLL05454.1	*Clostridiaceae bacterium*	VanZ-2
MBQ8626051.1	*Agathobacter sp.*	VanZ-2
MBM3324865.1	*Calditrichota bacterium*	VanZ-2
AGW12322.1	*Megalodesulfovibrio gigas DSM 1382 = ATCC 19364*	VanZ-2
WP_164553391.1	*Brevibacillus marinus*	VanZ-2
NLI61698.1	*Clostridiales bacterium*	VanZ-2
WP_152910541.1	*Pseudactinotalea sp. HY160*	VanZ-2
WP_068679178.1	*Paenibacillus oryzae*	VanZ-2
NLU24692.1	*Clostridiales bacterium*	VanZ-2
WP_015255085.1	*Thermobacillus sp.*	VanZ-2
HHW46974.1	*Clostridiales bacterium*	VanZ-2
MBD5087618.1	*Clostridiales bacterium*	VanZ-2
MBZ5648460.1	*Terriglobia bacterium*	VanZ-2
NLY66869.1	*Tissierellia bacterium*	VanZ-2
HIA13529.1	*Nitrospirales bacterium*	VanZ-2
AKU98621.1	*Labilithrix luteola*	VanZ-2
WP_068759222.1	*Turicibacter sp. H121*	VanZ-2
WP_066727141.1	*Desulfuromonas sp. DDH964*	VanZ-2
PYV34002.1	*Acidobacteriota bacterium*	VanZ-2
MBR4240749.1	*Eubacterium sp.*	VanZ-2
MBI9087777.1	*Desulfobacterium sp.*	VanZ-2
PYS70866.1	*Acidobacteriota bacterium*	VanZ-2
MBR6258226.1	*Lachnospiraceae bacterium*	VanZ-2
WP_205136126.1	*Virgibacillus halotolerans*	VanZ-2
WP_027638755.1	*Clostridium cadaveris*	VanZ-2
RPH95992.1	*Candidatus Zhuqueibacterota bacterium*	VanZ-2
NLP48325.1	*Clostridiales bacterium*	VanZ-2
UCG11375.1	*Deltaproteobacteria bacterium*	VanZ-2
MCC6420294.1	*Gemmataceae bacterium*	VanZ-2
WP_215007982.1		VanZ-2
MBS5679213.1	*Clostridiales bacterium*	VanZ-2
WP_213649918.1	*Paenibacillus sp. J23TS9*	VanZ-2
WP_204598095.1	*Clostridium pascui*	VanZ-2
MBK9214842.1	*Chloracidobacterium sp.*	VanZ-2
WP_077864340.1	*Clostridium saccharobutylicum*	VanZ-2
MCA9284644.1	*Phycisphaerales bacterium*	VanZ-2
MBO4406878.1	*Clostridia bacterium*	VanZ-2
NLX31040.1	*Deltaproteobacteria bacterium*	VanZ-2
MBD3639011.1	*Crocinitomicaceae bacterium*	VanZ-2
WP_129438090.1	*Paenibacillus protaetiae*	VanZ-2
CDA88954.1	*Ruminococcus sp. CAG:563*	VanZ-2
WP_145281173.1	*Pirellulimonas nuda*	VanZ-2
RME02089.1	*Deltaproteobacteria bacterium*	VanZ-2
NPV68772.1	*Anaerolineae bacterium*	VanZ-2
WP_012876901.1	*Xylanimonas cellulosilytica DSM 15894*	VanZ-2
MCC7208486.1	*Anaerolineae bacterium*	VanZ-2
MBL9080450.1	*Planctomycetales bacterium*	VanZ-2
HCL89814.1	*Candidatus Sediminicultor tertius*	VanZ-2
MBS1705199.1	*Armatimonadota bacterium*	VanZ-2
MBI3468645.1	*Planctomycetota bacterium*	VanZ-2
HEY64494.1	*Caldilineae bacterium*	VanZ-2
PWT74123.1	*Chloroflexota bacterium*	VanZ-2
MBQ4059411.1	*Lachnospiraceae bacterium*	VanZ-2
CUU33840.1	*Armatimonadetes bacterium GXS*	VanZ-2
ESS12948.1	*uncultured archaeon A07HR60*	VanZ-2
WP_168689135.1	*Leifsonia sp. PS1209*	VanZ-2
MBM4246404.1	*Deltaproteobacteria bacterium*	VanZ-2
MBQ1821965.1	*Lachnospiraceae bacterium*	VanZ-2
NLE51680.1	*Chloroflexota bacterium*	VanZ-2
MBR0294028.1	*Acidaminococcaceae bacterium*	VanZ-2
WP_216156628.1	*Polynucleobacter sp. P1-05-14*	VanZ-2
NLZ80619.1	*Clostridiales bacterium*	VanZ-2
MBA2241394.1	*Chthoniobacterales bacterium*	VanZ-2
HIE09415.1	*Armatimonadota bacterium*	VanZ-2
WP_026545079.1	*Arthrobacter sp. 35/47*	VanZ-2
MBA3833078.1	*Chthoniobacterales bacterium*	VanZ-2
WP_214482133.1	*Bacillus sp. SM2101*	VanZ-2
MBG30379.1	*Opitutia bacterium*	VanZ-2
WP_078391300.1	*Shouchella patagoniensis*	VanZ-2
OLO38074.1	*Alkalihalophilus pseudofirmus*	VanZ-2
MBC7315065.1	*Chloroflexota bacterium*	VanZ-2

MBI5355910.1
Candidatus Aenigmatarchaeota archaeon VanZ-2

PYI93127.1
Verrucomicrobiota bacterium VanZ-2

PYK39897.1
Verrucomicrobiota bacterium VanZ-2

MBI4220880.1
Chloroflexota bacterium VanZ-2

MBM3873978.1
Verrucomicrobiota bacterium VanZ-2

MBU2339912.1
Alphaproteobacteria bacterium VanZ-2

RKW54376.1
Lachnospiraceae bacterium VanZ-2

MBE0555989.1
Pseudomonadota bacterium VanZ-2

WP_191781462.1
Bacillus sp. PS06 VanZ-2

MBU0664585.1
Pseudomonadota bacterium VanZ-2

WP_067610544.1
Erythrobacter sp. QSSC1-22B VanZ-2

RLI72594.1
Archaeoglobales archaeon VanZ-2

MBU1205494.1
Pseudomonadota bacterium VanZ-2

MBU4235456.1
Pseudomonadota bacterium VanZ-2

MTK06033.1
Hungatella sp. VanZ-2

BAL57215.1 VanZ-2

WP_023457568.1
Asticcacaulis sp. AC466 VanZ-2

HHV33848.1
Acholeplasma sp. VanZ-2

PNE09698.1
Beijerinckiaceae bacterium VanZ-2

WP_102401316.1
Haloimpatiens massiliensis VanZ-2

REJ08817.1
Halobacillus trueperi VanZ-2

MBS3090633.1
Candidatus Pacearchaeota archaeon VanZ-2

SIH38409.1
Mycobacteroides abscessus subsp. abscessus VanZ-2

WP_117412296.1
Breoghania sp. L-A4 VanZ-2

MBM3809546.1
Acidimicrobiia bacterium VanZ-2

WP_008881390.1
Geobacillus thermodenitrificans VanZ-2

WP_144329908.1
Geobacillus sp. LEMMJ02 VanZ-2

MBC7766022.1
Clostridia bacterium VanZ-2

MBL8306050.1
Rubrivivax sp. VanZ-2

MBR4672595.1
Bacilli bacterium VanZ-2

MBP3444986.1
Bacilli bacterium VanZ-2

MBP3635027.1
Bacilli bacterium VanZ-2

MBT5001068.1
Tateyamaria sp. VanZ-2

WP_030094875.1
Mycobacteroides chelonae VanZ-2

MBE1366038.1 VanZ-2

HCX72938.1
Candidatus Cloacimonas sp. VanZ-2

OUU58812.1
Verrucomicrobia bacterium TMED60 VanZ-2

MBQ2661401.1
Clostridia bacterium VanZ-2

MBQ9825746.1
Bacillota bacterium VanZ-2

MBQ6126127.1
Erysipelotrichaceae bacterium VanZ-2

MBR3840314.1
Erysipelotrichales bacterium VanZ-2

HBK84783.1
Bacillota bacterium VanZ-2

WP_062051759.1
Bacillus sp. JCM 19034 VanZ-2

WP_183596689.1
Paenibacillus phyllosphaerae VanZ-2

MBQ9461810.1
Clostridia bacterium VanZ-2

MBA3620356.1
Acidothermales bacterium VanZ-2

MAG20045.1
archaeon VanZ-2

MBQ8330729.1
Lachnospiraceae bacterium VanZ-2

MBQ9907717.1
Oscillospiraceae bacterium VanZ-2

WP_058628969.1
Microbacterium testaceum VanZ-2

MBR1761780.1
Eubacterium sp. VanZ-2

WP_104133362.1
Cryobacterium sp. M91 VanZ-2

MBA3466620.1
Gemmatimonadaceae bacterium VanZ-2

MBR6229656.1
Eubacterium sp. VanZ-2

MBP3479721.1
Oscillospiraceae bacterium VanZ-2

MBO5495037.1
Eubacterium sp. VanZ-2

MBR5755914.1
Erysipelotrichaceae bacterium VanZ-2

WP_077612373.1
Clostridium sp. Marseille-P2415 VanZ-2

MBE6871779.1
Oscillospiraceae bacterium VanZ-2

SEO55305.1
Denitrobacterium detoxificans VanZ-2

MBQ7499943.1
Clostridia bacterium VanZ-2

MBA2569016.1
Actinomycetota bacterium VanZ-2

WP_006717249.1
Desulfitobacterium metallireducens DSM 15288 VanZ-2

MBR6681807.1
Clostridia bacterium VanZ-2

MBE6734367.1
Oscillospiraceae bacterium VanZ-2

MBR5619787.1
Clostridia bacterium VanZ-2

PZR75792.1
Chthoniobacterales bacterium VanZ-2

HBE71406.1
Planctomycetaceae bacterium VanZ-2

MBN2578218.1
Pirellulales bacterium VanZ-2

ACD50090.1 VanZ-2

MBI1756299.1
Fimbriimonas ginsengisoli VanZ-2

WP_215041654.1
Bacillus sp. ISL-18 VanZ-2

MBR4305104.1
Clostridia bacterium VanZ-2

MBX9722463.1
Candidatus Obscuribacterales bacterium VanZ-2

PLS79911.1
Chloroflexota bacterium VanZ-2

MCA8946844.1
Planctomycetota bacterium VanZ-2

PNT94002.1
Clostridium thermosuccinogenes VanZ-2

HCI59952.1
Ruminococcus sp. VanZ-2

MCA9107787.1
Planctomycetales bacterium VanZ-2

MBR4484535.1
Erysipelotrichaceae bacterium VanZ-2

RLS44359.1
Planctomycetota bacterium VanZ-2

WP_163209754.1
Clostridium sporogenes VanZ-2

TFH46985.1
ANME-2 cluster archaeon VanZ-2

MBP3893168.1
Atopobiaceae bacterium VanZ-2

WP_169361320.1
Psychrobacillus sp. BL-248-WT-3 VanZ-2

MBA3270396.1
Acidobacteriota bacterium VanZ-2

WP_193853439.1
cf. Phormidesmis sp. LEGE 11477 VanZ-2

WP_066172507.1
Bacillus marinisedimentorum VanZ-2

MQY58017.1
Clostridia bacterium VanZ-2

MSQ02913.1
Myxococcales bacterium VanZ-2

WP_063966345.1
Domibacillus aminovorans VanZ-2

MBO5539732.1
Muribaculaceae bacterium VanZ-2

NLO65465.1
Bacillota bacterium VanZ-2

MBP5561608.1	*Muribaculaceae bacterium*	VanZ-2
NLX42360.1	*Chloroflexota bacterium*	VanZ-2
WP_132093863.1	*Caulobacter sp. BK020*	VanZ-2
MBQ8472478.1	*Bacilli bacterium*	VanZ-2
MBQ3080911.1	*Clostridia bacterium*	VanZ-2
MBA3313330.1	*Planctomycetaceae bacterium*	VanZ-2
WP_224839733.1	*Sutcliffiella tianshenii*	VanZ-2
MBQ4105311.1	*Clostridia bacterium*	VanZ-2
HBO43323.1	*Planctomycetaceae bacterium*	VanZ-2
MBL8166307.1	*Anaerolineae bacterium*	VanZ-2
MBC7241627.1	*Anaerolineae bacterium*	VanZ-2
HER43935.1	*Candidatus Eiseniibacteriota bacterium*	VanZ-2
HAD58136.1	*Planctomycetaceae bacterium*	VanZ-2
MBQ2093384.1	*Ruminococcus sp.*	VanZ-2
NIL97329.1	*Planctomycetales bacterium*	VanZ-2
MBQ4647737.1	*Clostridia bacterium*	VanZ-2
MBD9158509.1	*Clostridiales bacterium*	VanZ-2
MBI1799086.1	*Candidatus Eiseniibacteriota bacterium*	VanZ-2
WP_182167703.1	*Stappia albiluteola*	VanZ-2
GGN03603.1	*Terrabacter tumescens*	VanZ-2
WP_154311379.1		VanZ-2
WP_224874932.1	*Rossellomorea aquimaris*	VanZ-2
MBF0270944.1	*Magnetococcales bacterium*	VanZ-2
TYS18548.1	*Rossellomorea vietnamensis*	VanZ-2
RJQ54379.1	*Actinomycetota bacterium*	VanZ-2
MBQ1950807.1	*Clostridia bacterium*	VanZ-2
WP_224521860.1	*Rossellomorea vietnamensis*	VanZ-2
EOS44513.1	*Lachnospiraceae bacterium A2*	VanZ-2
WP_027630911.1	*Ruminiclostridium cellobioparum DSM 1351 = ATCC 15832*	VanZ-2
MBN2273193.1	*Bacteroidales bacterium*	VanZ-2
HGX16401.1		VanZ-2
MBQ3705081.1	*Clostridia bacterium*	VanZ-2
WP_216298530.1		VanZ-2
MBQ3046536.1	*Clostridia bacterium*	VanZ-2
SEI72422.1	*Lachnospiraceae bacterium A10*	VanZ-2
MBU4514428.1	*Bacillota bacterium*	VanZ-2
NLX59175.1	*Phycisphaerae bacterium*	VanZ-2
MBQ9534427.1	*Clostridia bacterium*	VanZ-2
MBR6168957.1	*Candidatus Saccharibacteria bacterium*	VanZ-2
MBM3717002.1	*Actinomycetota bacterium*	VanZ-2
MBR5669473.1	*Candidatus Saccharibacteria bacterium*	VanZ-2
CDC36442.1	*Butyrivibrio sp. CAG:318*	VanZ-2
WP_077715266.1	*Desulforamulus ferrireducens*	VanZ-2
WP_181905772.1	*Microbacterium bovistercoris*	VanZ-2
MBC7812491.1	*Anaerolineae bacterium*	VanZ-2
WP_130146572.1	*Pseudoalteromonas sp. CO325X*	VanZ-2
WP_182102436.1	*Niallia taxi*	VanZ-2
MBR2741110.1	*Oscillospiraceae bacterium*	VanZ-2
WP_191741777.1	*Clostridium butanoliproducens*	VanZ-2
NLC06039.1	*Erysipelothrix sp.*	VanZ-2
NJN14405.1	*Planctomycetota bacterium*	VanZ-2
MAR11310.1	*Blastopirellula sp.*	VanZ-2
MBP6619143.1	*Leadbetterella sp.*	VanZ-2
HHS07767.1		VanZ-2
MBR5616745.1	*Oscillospiraceae bacterium*	VanZ-2
WP_010077636.1	*Clostridium cellulovorans 743B*	VanZ-2
MCC6641147.1	*Deltaproteobacteria bacterium*	VanZ-2
NLP12282.1	*bacterium*	VanZ-2
MBQ8891944.1	*Bacilli bacterium*	VanZ-2
MBI3988501.1	*Candidatus Methylomirabilota bacterium*	VanZ-2
GAO31306.1	*Geofilum rubicundum JCM 15548*	VanZ-2
MBR4059755.1	*Lachnospiraceae bacterium*	VanZ-2
MBR6527256.1	*Lachnospiraceae bacterium*	VanZ-2
MBD8976083.1	*Veillonella magna*	VanZ-2
OQX22831.1	*Desulfobacteraceae bacterium IS3*	VanZ-2
PYS52966.1	*Acidobacteriota bacterium*	VanZ-2
MBL0699307.1	*Desulfobacterales bacterium*	VanZ-2
MBR2868239.1	*Clostridia bacterium*	VanZ-2
WP_061325863.1	*Clostridium sporogenes*	VanZ-2
WP_191698240.1	*Solibacillus sp. FSL W8-0372*	VanZ-2
MBR2942866.1	*Clostridia bacterium*	VanZ-2
WP_011879310.1	*Desulforamulus reducens MI-1*	VanZ-2
MCC5697517.1	*Klebsiella pneumoniae*	VanZ-2
HIU34850.1	*Candidatus Pullichristensenella excrementigallinarum*	VanZ-2
MSU23027.1	*Opitutus sp.*	VanZ-2
WP_012635776.1	*Halothermothrix orenii H 168*	VanZ-2
PWR00133.1	*Leucothrix pacifica*	VanZ-2
MBC7591500.1	*Salinibacterium sp.*	VanZ-2
MBI2150643.1	*Acidobacteriota bacterium*	VanZ-2
MBI2835696.1	*Acidobacteriota bacterium*	VanZ-2
MBW2991726.1	*Candidatus Woesearchaeota archaeon*	VanZ-2
MBI3669834.1	*Acidobacteriota bacterium*	VanZ-2
MBP1587980.1	*Clostridia bacterium*	VanZ-2
MBM3326374.1	*Calditrichota bacterium*	VanZ-2
MBR2902324.1	*Clostridia bacterium*	VanZ-2
MBF1116172.1	*Solobacterium sp.*	VanZ-2
MCC6798964.1	*Anaerolineae bacterium*	VanZ-2
PYJ71170.1	*Verrucomicrobiota bacterium*	VanZ-2
WP_016428167.1	*Paenisporosarcina sp. FSL H8-0542*	VanZ-2
MBX3332161.1	*Nitrospira sp.*	VanZ-2
WP_104328227.1	*Rathayibacter sp. AY1A3*	VanZ-2
MBR2067119.1	*Solobacterium sp.*	VanZ-2
MCC6237981.1	*Dehalococcoidia bacterium*	VanZ-2

MBZ5527248.1	*Terriglobia bacterium*	VanZ
WP_183108769.1	*Thermohalobacter berrensis*	VanZ
HHX55782.1	*Clostridiales bacterium*	VanZ
MCB2107313.1	*Paracoccaceae bacterium*	VanZ
MBE7033589.1	*Oscillospiraceae bacterium*	VanZ
MBQ8569806.1	*Oscillospiraceae bacterium*	VanZ
NLZ83778.1	*Clostridiales bacterium*	VanZ
EUJ41378.1	*Brochothrix campestris FSL F6-1037*	VanZ
MBM7657471.1	*Sporolactobacillus spathodeae*	VanZ
MBU2055645.1	*Pseudomonadota bacterium*	VanZ
MBE3576182.1	*Limnochordales bacterium*	VanZ
MCA3179534.1	*Burkholderiales bacterium*	VanZ
WP_162370710.1	*Anaerotalea alkaliphila*	VanZ
MCB5262803.1	*Candidatus Cloacimonadota bacterium*	VanZ
NDC24190.1	*Pseudomonadota bacterium*	VanZ
MBS4015648.1	*Candidatus Latescibacterota bacterium*	VanZ
MBO5895818.1	*Clostridia bacterium*	VanZ
MBQ6523640.1	*Atopobiaceae bacterium*	VanZ
MAW28146.1	*Gammaproteobacteria bacterium*	VanZ
HHJ52577.1	*Caldithrix abyssi*	VanZ
WP_154893465.1		VanZ
MBR9943456.1	*Clostridiaceae bacterium Marseille-Q3526*	VanZ
WP_193470150.1		VanZ
WP_076249582.1	*Paenibacillus sp. FSL H8-0104*	VanZ
WP_119820968.1	*Halalkaliarchaeum desulfuricum*	VanZ
HAO99905.1	*Fibrobacterota bacterium*	VanZ
CAG7619762.1	*Paenibacillus allorhizosphaerae*	VanZ
PAW63072.1	*Verrucomicrobiia bacterium Tous-C4TDCM*	VanZ
MBX3279448.1	*Acidobacteriota bacterium*	VanZ
WP_214426503.1	*Paenarthrobacter sp. DKR-5*	VanZ
NLM49843.1	*Clostridiaceae bacterium*	VanZ
MBJ06853.1	*Verrucomicrobiaceae bacterium*	VanZ
MBL59190.1	*Verrucomicrobiales bacterium*	VanZ
HAM72924.1	*Verrucomicrobiales bacterium*	VanZ
MBV9110140.1	*Gemmatimonadota bacterium*	VanZ
MBE6159679.1	*Lactobacillales bacterium*	VanZ
KRM73061.1	*Lacticaseibacillus brantae DSM 23927*	VanZ
WP_169923669.1	*Propionicimonas paludicola*	VanZ
MBU6282265.1	*bacterium*	VanZ
HCD43721.1	*Lachnoclostridium sp.*	VanZ
NLT14532.1	*Clostridiales bacterium*	VanZ
MBK8466949.1	*Chloracidobacterium sp.*	VanZ
NKB76419.1	*Gammaproteobacteria bacterium*	VanZ
MBI4364628.1	*Candidatus Latescibacterota bacterium*	VanZ
MSQ07853.1	*Dehalococcoidia bacterium*	VanZ
MBR5561411.1	*Clostridia bacterium*	VanZ
NMB43099.1	*Clostridiales bacterium*	VanZ
WP_013703217.1	*Marinithermus hydrothermalis DSM 14884*	VanZ
OPX96715.1	*Syntrophorhabdus sp. PtaB.Bin006*	VanZ
WP_015010714.1	*Amphibacillus xylanus NBRC 15112*	VanZ
NLL22284.1	*Tissierellia bacterium*	VanZ
SCK04643.1	*uncultured Clostridium sp.*	VanZ
TLY17775.1	*Nitrospirota bacterium*	VanZ
WP_181677440.1	*Listeria rustica*	VanZ
MCB1363055.1	*Paracoccaceae bacterium*	VanZ
MBQ7375973.1	*Clostridia bacterium*	VanZ
MBR5301381.1	*Clostridia bacterium*	VanZ
NBD39185.1	*Verrucomicrobiota bacterium*	VanZ
MBR3416933.1	*Clostridia bacterium*	VanZ
RCL35634.1	*Puniceicoccaceae bacterium*	VanZ
WP_224268114.1	*Haloprofundus salinisoli*	VanZ
MCD8144816.1	*Oscillospiraceae bacterium*	VanZ
PGZ91738.1	*Bacillus sp. AFS029533*	VanZ
NTW70816.1	*Eubacteriaceae bacterium*	VanZ
TET66404.1	*Candidatus Zixiibacteriota bacterium*	VanZ
MBD3354670.1	*Candidatus Woesearchaeota archaeon*	VanZ
WP_115862003.1	*Lysobacter silvisoli*	VanZ
MCD7872134.1	*Clostridiales bacterium*	VanZ
TDI08775.1	*Acidobacteriota bacterium*	VanZ
WP_088088102.1	*Bacillus sp. OV166*	VanZ
SFD05372.1	*Bosea sp. CRIB-10*	VanZ
MBR2740694.1	*Oscillospiraceae bacterium*	VanZ
KKM10243.1	*Clostridiales bacterium PH28_bin88*	VanZ
BAL78824.1	*Bradyrhizobium cosmicum*	VanZ
WP_191813251.1	*Cytobacillus sp. FSL K6-0265*	VanZ
WP_015395611.1	*Clostridium sp. YIM B02555*	VanZ
NLN83344.1	*Bacillota bacterium*	VanZ
SIO46197.1	*Bradyrhizobium erythrophlei*	VanZ
KMK77387.1	*Alkalihalobacillus pseudalcaliphilus*	VanZ
MAN62651.1	*Parvibaculum sp.*	VanZ
MBR2951510.1	*Clostridia bacterium*	VanZ
MBS1640877.1	*Bacteroidota bacterium*	VanZ
MBR3599975.1	*Lachnospiraceae bacterium*	VanZ
PKN65227.1	*Deltaproteobacteria bacterium HGW-Deltaproteobacteria-15*	VanZ
WP_128905705.1	*Halorubrum amylolyticum*	VanZ
WP_052015917.1	*Halalkalibacter hemicellulosilyticusJCM 9152*	VanZ
PYV71596.1	*Acidobacteriota bacterium*	VanZ
MAL85354.1	*Opitutia bacterium*	VanZ
WP_105957423.1	*Alkalicoccus urumqiensis*	VanZ
HFN11631.1		VanZ
TCO12987.1	*Camelimonas lactis*	VanZ
NNV09120.1	*Geobacillus sp. MMMUD3*	VanZ
WP_120704465.1	*Rhizobium jaguaris*	VanZ

MBX3155492.1	*Deltaproteobacteria bacterium*	VanZ-2
AOX46934.1	*Microbacterium sp. BH-3-3-3*	VanZ-2
MBR2717394.1	*Oscillospiraceae bacterium*	VanZ-2
WP_213518025.1	*Paenibacillus montaniterrae*	VanZ-2
HAC35008.1	*Gammaproteobacteria bacterium*	VanZ-2
WP_130436025.1	*Cuneatibacter caecimuris*	VanZ-2
MBL7859069.1	*Cyclobacteriaceae bacterium*	VanZ-2
OUS29477.1	*Gammaproteobacteria bacterium 45_16_T64*	VanZ-2
WP_223036664.1	*Crassaminicella profunda*	VanZ-2
WP_145261851.1	*Calycomorphotria hydatis*	VanZ-2
MBR3883529.1	*Bacteroidaceae bacterium*	VanZ-2
MBR1865992.1	*Lachnospiraceae bacterium*	VanZ-2
WP_024030799.1	*Neobacillus vireti*	VanZ-2
WP_089973609.1	*Clostridium gasigenes*	VanZ-2
MBO4593338.1	*Bacteroidaceae bacterium*	VanZ-2
WP_090843812.1	*Alkalicoccus daliensis*	VanZ-2
MCC7390781.1	*Candidatus Sumerlaeota bacterium*	VanZ-2
MBE7047357.1	*Oscillospiraceae bacterium*	VanZ-2
WP_181602103.1	*Clostridium intestinale*	VanZ-2
RMH36472.1	*Nitrospirota bacterium*	VanZ-2
MBR3131141.1	*Candidatus Saccharibacteria bacterium*	VanZ-2
MBQ8620944.1	*Oscillospiraceae bacterium*	VanZ-2
MBQ9268837.1	*Oscillospiraceae bacterium*	VanZ-2
OQB51400.1	*Firmicutes bacterium ADurb.Bin146*	VanZ-2
HIX15876.1	*Candidatus Hungatella pullicola*	VanZ-2
MBQ8263746.1	*Oscillospiraceae bacterium*	VanZ-2
MBQ7653474.1	*Clostridia bacterium*	VanZ-2
TXH97805.1	*Rheinheimera sp.*	VanZ-2
MBQ6709041.1	*Clostridia bacterium*	VanZ-2
MBQ6645127.1	*Clostridia bacterium*	VanZ-2
OQB64204.1	*Tenericutes bacterium ADurb.Bin140*	VanZ-2
MBQ9859118.1	*Clostridia bacterium*	VanZ-2
RZV60809.1	*Flavobacteriaceae bacterium*	VanZ-2
HCW73774.1	*Clostridiaceae bacterium*	VanZ-2
MBD5158096.1	*Butyrivibrio sp.*	VanZ-2
WP_131441850.1	*Clostridium perfringens*	VanZ-2
NLM23759.1	*Bacillota bacterium*	VanZ-2
MBQ4527436.1	*Clostridia bacterium*	VanZ-2
MBM3212625.1	*Candidatus Poribacteria bacterium*	VanZ-2
NLO40098.1	*Ruminiclostridium sp.*	VanZ-2
MBM3295356.1		VanZ-2
WP_120989151.1	*Arthrobacter sp. AG1021*	VanZ-2
WP_066679144.1	*Clostridium septicum*	VanZ-2
RCK80788.1	*Candidatus Ozemobacter sibiricus*	VanZ-2
NLV24009.1	*Deltaproteobacteria bacterium*	VanZ-2
MBQ17909.1	*Planctomycetaceae bacterium*	VanZ-2
PIR95097.1	*Candidatus Falkowbacteria bacterium CG10_big_fil_rev_8_21_14_0_10_37_6*	VanZ-2
UCG38272.1	*bacterium*	VanZ-2
WP_185127932.1	*Cohnella zeiphila*	VanZ-2
ANM28793.1	*Acidobacteria bacterium Mor1*	VanZ-2
MBD3348686.1	*Candidatus Effluviviaceae Genus IV sp.*	VanZ-2
MBQ4612484.1	*Clostridia bacterium*	VanZ-2
MTI18456.1	*Pseudovibrio flavus*	VanZ-2
RPI87229.1	*Planctomycetaceae bacterium*	VanZ-2
WP_214779808.1	*Bacillus sp. ISL-45*	VanZ-2
AMM42152.1	*Candidatus Desulfofervidus auxilii*	VanZ-2
MBP6749830.1	*Lysobacteraceae bacterium*	VanZ-2
MCA9749324.1	*Romboutsia sp.*	VanZ-2
MBT4885622.1	*Planctomycetaceae bacterium*	VanZ-2
MCB0327582.1	*Bdellovibrionales bacterium*	VanZ-2
MBK28839.1	*Verrucomicrobiales bacterium*	VanZ-2
MBR5101923.1	*Muribaculaceae bacterium*	VanZ-2
RKR94192.1	*Williamsia muralis*	VanZ-2
EAR25591.1	*marine actinobacterium PHSC20C1*	VanZ-2
WP_152729377.1	*Goekera deserti*	VanZ-2
NDH16712.1	*Opitutia bacterium*	VanZ-2
MCC6824981.1	*Acidobacteriota bacterium*	VanZ-2
MBI2516802.1	*Opitutia bacterium*	VanZ-2
MBQ1317398.1	*Lachnospiraceae bacterium*	VanZ-2
MBJ2194195.1	*Muribaculaceae bacterium*	VanZ-2
MBE6713310.1	*Oscillospiraceae bacterium*	VanZ-2
UCF88743.1	*bacterium*	VanZ-2
NOZ40523.1	*Planctomycetota bacterium*	VanZ-2
MCD4769226.1	*Bacteroidales bacterium*	VanZ-2
WP_162369067.1	*Anaerotalea alkaliphila*	VanZ-2
WP_010243062.1	*Acetivibrio cellulolyticus CD2*	VanZ-2
WP_002596607.1	*Clostridium thermobutyricum*	VanZ-2
WP_205170867.1	*Rossellomorea pakistanensis*	VanZ-2
MBQ4425239.1	*Lachnospiraceae bacterium*	VanZ-2
MCB1254660.1	*Austwickia sp.*	VanZ-2
NCX77132.1	*Pseudomonadota bacterium*	VanZ-2
WP_169907537.1	*Priestia abyssalis*	VanZ-2
WP_168630271.1	*Cellulomonas denverensis*	VanZ-2
HIV75239.1	*Candidatus Pseudogracilibacillus intestinigallinarum*	VanZ-2
MCB1132710.1	*Verrucomicrobiia bacterium*	VanZ-2
MBQ2960086.1	*Oscillospiraceae bacterium*	VanZ-2
MBI5836283.1	*Candidatus Eiseniibacteriota bacterium*	VanZ-2
WP_204542744.1	*Peribacillus deserti*	VanZ-2
MBP6576876.1	*Chryseobacterium sp.*	VanZ-2
WP_010632056.1	*Sporolactobacillus vineae DSM 21990 = SL153*	VanZ-2
OLC84215.1	*Acidobacteria bacterium 13_1_40CM_4_57_6*	VanZ-2
WP_224839854.1	*Sutcliffiella tianshenii*	VanZ-2
WP_131445994.1	*Flaviaesturariibacter flavus*	VanZ-2

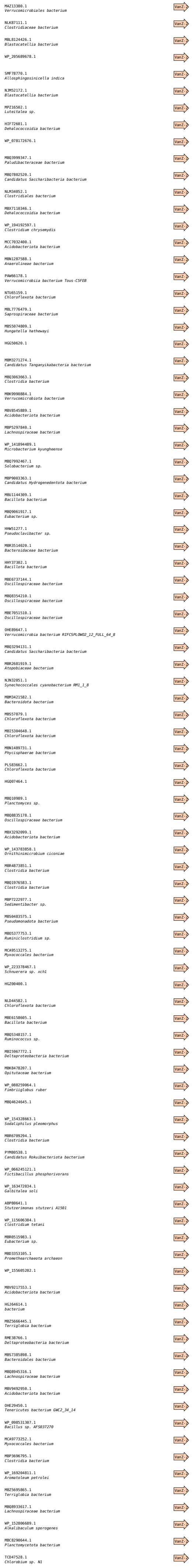
MAZ13380.1
Verrucomicrobiales bacterium
VanZ-2
NLK87111.1
Clostridiaceae bacterium
VanZ-2
MBL8124426.1
Blastocatellia bacterium
VanZ-2
WP_205689678.1
VanZ-2
SMF78770.1
Allosphingosinicella indica
VanZ-2
NJM52172.1
Blastocatellia bacterium
VanZ-2
MPZ16502.1
Luteitalea sp.
VanZ-2
HIF72601.1
Dehalococcoidia bacterium
VanZ-2
WP_078172676.1
VanZ-2
MBQ3999347.1
Paludibacteraceae bacterium
VanZ-2
MBQ7802520.1
Candidatus Saccharibacteria bacterium
VanZ-2
NLM34052.1
Clostridiales bacterium
VanZ-2
MBX7110346.1
Dehalococcoidia bacterium
VanZ-2
WP_194192597.1
Clostridium chrysemydis
VanZ-2
MCC7032400.1
Acidobacteriota bacterium
VanZ-2
MBN1287588.1
Anaerolineae bacterium
VanZ-2
PAW66178.1
Verrucomicrobiia bacterium Tous-C5FEB
VanZ-2
NTU65159.1
Chloroflexota bacterium
VanZ-2
MBL7776479.1
Saprospiraceae bacterium
VanZ-2
MBS5074809.1
Hungatella hathewayi
VanZ-2
HGG50620.1
VanZ-2
MBM3271274.1
Candidatus Tanganyikabacteria bacterium
VanZ-2
MBQ3063663.1
Clostridia bacterium
VanZ-2
MBK9990884.1
Verrucomicrobiota bacterium
VanZ-2
MBV8545889.1
Acidobacteriota bacterium
VanZ-2
MBP5297840.1
Lachnospiraceae bacterium
VanZ-2
WP_141894489.1
Microbacterium kyungheense
VanZ-2
MBQ7992467.1
Solobacterium sp.
VanZ-2
MBP9003363.1
Candidatus Hydrogenedentota bacterium
VanZ-2
MBU1144309.1
Bacillota bacterium
VanZ-2
MBQ9061917.1
Eubacterium sp.
VanZ-2
HHW51277.1
Pseudoclavibacter sp.
VanZ-2
MBR3514020.1
Bacteroidaceae bacterium
VanZ-2
HHY37302.1
Bacillota bacterium
VanZ-2
MBE6737144.1
Oscillospiraceae bacterium
VanZ-2
MBQ8354210.1
Oscillospiraceae bacterium
VanZ-2
MBE7051510.1
Oscillospiraceae bacterium
VanZ-2
OHE89647.1
Verrucomicrobia bacterium RIFCSPLOWO2_12_FULL_64_8
VanZ-2
MBQ3294131.1
Candidatus Saccharibacteria bacterium
VanZ-2
MBR2681919.1
Atopobiaceae bacterium
VanZ-2
NJN32051.1
Synechococcales cyanobacterium RM1_1_8
VanZ-2
MBM3421582.1
Bacteroidota bacterium
VanZ-2
MBS57879.1
Chloroflexota bacterium
VanZ-2
MBI5304648.1
Chloroflexota bacterium
VanZ-2
MBN1489731.1
Phycisphaerae bacterium
VanZ-2
PLS83662.1
Chloroflexota bacterium
VanZ-2
HGQ07464.1
VanZ-2
MBQ10989.1
Planctomyces sp.
VanZ-2
MBQ8835178.1
Oscillospiraceae bacterium
VanZ-2
MBX3292099.1
Acidobacteriota bacterium
VanZ-2
WP_143783858.1
Ornithinimicrobium ciconiae
VanZ-2
MBR4873851.1
Clostridia bacterium
VanZ-2
MBQ1976583.1
Clostridia bacterium
VanZ-2
MBP7222977.1
Sedimentibacter sp.
VanZ-2
MBS0483575.1
Pseudomonadota bacterium
VanZ-2
MBO5377753.1
Ruminiclostridium sp.
VanZ-2
MCA9513275.1
Myxococcales bacterium
VanZ-2
WP_223378467.1
Schnuerera sp. xch1
VanZ-2
HGZ00400.1
VanZ-2
NLD44582.1
Chloroflexota bacterium
VanZ-2
MBE6158605.1
Bacillota bacterium
VanZ-2
MBQ5348157.1
Ruminococcus sp.
VanZ-2
MBI5967772.1
Deltaproteobacteria bacterium
VanZ-2
MBK8478207.1
Opitutaceae bacterium
VanZ-2
WP_088259064.1
Fimbriiglobus ruber
VanZ-2
MBQ4624645.1
VanZ-2
WP_154328663.1
Sodaliphilus pleomorphus
VanZ-2
MBR6709294.1
Clostridia bacterium
VanZ-2
PYM80538.1
Candidatus Rokuibacteriota bacterium
VanZ-2
WP_066245121.1
Fictibacillus phosphorivorans
VanZ-2
WP_163472034.1
Galbitalea soli
VanZ-2
ABP80641.1
Stutzerimonas stutzeri A1501
VanZ-2
WP_115606384.1
Clostridium tetani
VanZ-2
MBR0515983.1
Eubacterium sp.
VanZ-2
MBD3353105.1
Promethearchaeota archaeon
VanZ-2
WP_155605202.1
VanZ-2
MBV9217553.1
Acidobacteriota bacterium
VanZ-2
HGJ64614.1
bacterium
VanZ-2
MBZ5666445.1
Terriglobia bacterium
VanZ-2
RME38766.1
Deltaproteobacteria bacterium
VanZ-2
MBS7385898.1
Bacteroidales bacterium
VanZ-2
MBQ8945316.1
Lachnospiraceae bacterium
VanZ-2
MBV9492950.1
Acidobacteriota bacterium
VanZ-2
OHE29450.1
Tenericutes bacterium GWC2_34_14
VanZ-2
WP_098531307.1
Bacillus sp. AFS037270
VanZ-2
MCA9773252.1
Myxococcales bacterium
VanZ-2
MBP3696795.1
Clostridia bacterium
VanZ-2
WP_169204811.1
Aromatoleum petrolei
VanZ-2
MBZ5695865.1
Terriglobia bacterium
VanZ-2
MBQ8933617.1
Lachnospiraceae bacterium
VanZ-2
WP_152806689.1
Alkalibaculum sporogenes
VanZ-2
MBC8290644.1
Planctomycetota bacterium
VanZ-2
TCD47528.1
Chlorobium sp. N1
VanZ-2

WP_068694799.1	*Paenibacillus yonginensis*	VanZ-2
MBF0926919.1	*Clostridiales bacterium*	VanZ-2
MBQ6323095.1	*Lachnospiraceae bacterium*	VanZ-2
RHA09759.1		VanZ-2
WP_015596042.1		VanZ-2
MBS0632831.1	*Verrucomicrobiota bacterium*	VanZ-2
MBQ0069454.1	*Candidatus Sodaliphilus fimicaballi*	VanZ-2
WP_196790897.1	*Motilibacter aurantiacus*	VanZ-2
MAA99450.1	*Stappia sp.*	VanZ-2
WP_143236770.1	*Actinomyces urinae*	VanZ-2
MBQ9522895.1	*Paludibacteraceae bacterium*	VanZ-2
MBI1292694.1	*bacterium*	VanZ-2
MBS1402652.1	*Oscillospiraceae bacterium*	VanZ-2
MBN2251052.1	*Candidatus Altiarchaeota archaeon*	VanZ-2
MBA4380686.1	*Anaerolinea sp.*	VanZ-2
MBQ7941551.1	*Muribaculaceae bacterium*	VanZ-2
MBP3894366.1	*Atopobiaceae bacterium*	VanZ-2
WP_102693638.1	*Rummeliibacillus pycnus*	VanZ-2
PIN91444.1	*Candidatus Pacearchaeota archaeon CG10_big_fil_rev_8_21_14_0_10_32_14*	VanZ-2
MBQ7162316.1	*Bacteroidales bacterium*	VanZ-2
WP_152150330.1	*Bacillus aerolatus*	VanZ-2
QQS39840.1	*Acidobacteriota bacterium*	VanZ-2
HIY61952.1	*Candidatus Mediterraneibacter stercoripullorum*	VanZ-2
MBP5361493.1	*Ruminococcus sp.*	VanZ-2
WP_078697874.1	*Caloramator quimbayensis*	VanZ-2
MBQ9200855.1	*Bacteroidales bacterium*	VanZ-2
WP_203839879.1	*Winogradskya humida*	VanZ-2
MBN2696386.1	*Bacilli bacterium*	VanZ-2
MBE2199296.1	*Anaerolinea sp.*	VanZ-2
MSU67783.1	*Opitutaceae bacterium*	VanZ-2
WP_058613853.1	*Microbacterium testaceum*	VanZ-2
MBE7471195.1	*Anaerolineales bacterium*	VanZ-2
HCT91153.1		VanZ-2
MBP2011526.1		VanZ-2
MBN1069467.1	*Clostridium botulinum*	VanZ-2
MBA2725421.1	*Actinomycetota bacterium*	VanZ-2
HDI51283.1	*Bacteroidota bacterium*	VanZ-2
MBD5159710.1	*Ruminococcus sp.*	VanZ-2
WP_212996343.1	*Winogradskya consettensis*	VanZ-2
WP_027724808.1	*Tuberibacillus calidus DSM 17572*	VanZ-2
WP_090798244.1	*Paenibacillus sp. GP183*	VanZ-2
NQW45090.1	*Deltaproteobacteria bacterium*	VanZ-2
NLM31834.1	*Acholeplasmataceae bacterium*	VanZ-2
MBQ6630720.1	*Romboutsia sp.*	VanZ-2
MBP1744918.1	*Bacillota bacterium*	VanZ-2
MAV85483.1	*Gammaproteobacteria bacterium*	VanZ-2
MCC5980377.1	*Oceanicaulis sp.*	VanZ-2
MBQ1424337.1	*Lachnospiraceae bacterium*	VanZ-2
NQX10680.1	*Microbacteriaceae bacterium VKM Ac-2855*	VanZ-2
NLF48561.1	*Acholeplasmataceae bacterium*	VanZ-2
MBP6691722.1	*Lysobacterales bacterium*	VanZ-2
MBO5171086.1	*Oscillospiraceae bacterium*	VanZ-2
WP_215759238.1	*Hallella faecis*	VanZ-2
MBE2224219.1	*Anaerolineae bacterium*	VanZ-2
MBA3453499.1	*Deltaproteobacteria bacterium*	VanZ-2
HDH58028.1	*Bacteroidota bacterium*	VanZ-2
PSQ07328.1	*Halobacteriales archaeon QS_5_70_15*	VanZ-2
MBP6822768.1	*Acidobacteriota bacterium*	VanZ-2
MBX3234928.1	*Nitrospiraceae bacterium*	VanZ-2
MBQ6839513.1	*Oscillospiraceae bacterium*	VanZ-2
MCB1105902.1	*Cephaloticoccus sp.*	VanZ-2
MBQ9843743.1	*Oscillospiraceae bacterium*	VanZ-2
KGN83084.1	*Porphyromonadaceae bacterium COT-184 OH4590*	VanZ-2
MBK8465576.1	*Chloracidobacterium sp.*	VanZ-2
MBM3801222.1	*Acidimicrobiia bacterium*	VanZ-2
OGF25092.1	*Candidatus Falkowbacteria bacterium RIFOXYA2_FULL_47_19*	VanZ-2
WP_143698139.1	*Vibrio sp. qd031*	VanZ-2
MBN1396953.1	*Bacteroidota bacterium*	VanZ-2
RPJ74460.1	*Desulfobacteraceae bacterium*	VanZ-2
WP_078813729.1	*Prosthecobacter debontii*	VanZ-2
HAR20012.1	*Cytophagales bacterium*	VanZ-2
MBY5161275.1	*Salsipaludibacter albus*	VanZ-2
MBP3661252.1	*Oscillospiraceae bacterium*	VanZ-2
NND78995.1	*Maribacter sp.*	VanZ-2
MBR4108944.1	*Oscillospiraceae bacterium*	VanZ-2
MBP3798650.1	*Ruminococcus sp.*	VanZ-2
TVR04800.1	*Deltaproteobacteria bacterium*	VanZ-2
MBQ7916482.1	*Bacillota bacterium*	VanZ-2
BAR60385.1	*Bradyrhizobium diazoefficiens*	VanZ-2
CCX42999.1	*Clostridium sp. CAG:1024*	VanZ-2
WP_182089669.1	*Clostridium sp.*	VanZ-2
HIU15464.1	*Candidatus Ventricola intestinavium*	VanZ-2
RLA20783.1	*Gammaproteobacteria bacterium*	VanZ-2
MBO5488976.1	*Eubacterium sp.*	VanZ-2
WP_154481449.1	*Anaerosalibacter bizertensis*	VanZ-2
MBV9865818.1	*Abditibacteriaceae bacterium*	VanZ-2
WP_074366463.1	*Clostridium sp. N3C*	VanZ-2
MCC6508721.1	*Pirellulaceae bacterium*	VanZ-2
MBL9207961.1	*Opitutaceae bacterium*	VanZ-2
MBQ0795952.1	*Zhongshania sp.*	VanZ-2
WP_164104134.1	*Candidatus Laterigemmans baculatus*	VanZ-2
NMB01386.1	*Bacillota bacterium*	VanZ-2
WP_192496624.1	*Pseudoclavibacter sp. CFCC 13796*	VanZ-2

MBO6728918.1
Maricaulis sp. VanZ-2

WP_003540886.1
Desulfotomaculum nigrificans CO-1-SRB VanZ-2

MBQ9762012.1
Oscillospiraceae bacterium VanZ-2

NOX98022.1
Nitrospirota bacterium VanZ-2

MBO5346654.1
Lachnospiraceae bacterium VanZ-2

MCA9462871.1
Nitrospira sp. VanZ-2

TVQ34861.1
Spirochaetaceae bacterium VanZ-2

UCC43722.1
Candidatus Zixiibacteriota bacterium VanZ-2

SMF20828.1
Alteromonadaceae bacterium Bs31 VanZ-2

WP_233245463.1
Salinibacterium hongtaonis VanZ-2

WP_050754385.1
Gleimia coleocanis DSM 15436 VanZ-2

MBR3474818.1
Oscillospiraceae bacterium VanZ-2

MBI5964139.1
Chloroflexota bacterium VanZ-2

MBP3815313.1
Bacillota bacterium VanZ-2

MBE6677644.1
Oscillospiraceae bacterium VanZ-2

MBR3227462.1
Erysipelotrichaceae bacterium VanZ-2

MBQ6521156.1
Anaerolineaceae bacterium VanZ-2

POH21348.1
Fructilactobacillus sanfranciscensis VanZ-2

MBL9192934.1
Opitutaceae bacterium VanZ-2

PIQ90626.1
Candidatus Omnitrophica bacterium CG11_big_fil_rev_8_21_14_0_20_41_12 VanZ-2

OGL39255.1
Candidatus Schekmanbacteria bacterium GWA2_38_9 VanZ-2

MBR4897804.1
Prevotella sp. VanZ-2

MBQ7886652.1
Clostridia bacterium VanZ-2

MBK7487783.1
Nitrospira sp. VanZ-2

MBR6786700.1
Clostridia bacterium VanZ-2

RMD82997.1
Lentisphaerota bacterium VanZ-2

MBI2800414.1
Gammaproteobacteria bacterium VanZ-2

HBC47930.1
Candidatus Zixiibacteriota bacterium VanZ-2

MBQ8099223.1
Bacteroidaceae bacterium VanZ-2

WP_141784795.1 VanZ-2

MBQ6937418.1
Clostridia bacterium VanZ-2

WP_156484828.1
Bhargavaea cecembensis VanZ-2

WP_192016853.1
Photobacterium arenosum VanZ-2

MCA0351554.1
Chloroflexota bacterium VanZ-2

QOJ33742.1
Nitrospira sp. VanZ-2

GJM45423.1
Gemmatimonadota bacterium VanZ-2

WP_027250980.1
Photobacterium halotolerans DSM 18316 VanZ-2

HID40293.1
Calditrichota bacterium VanZ-2

PZQ87836.1
Leifsonia xyli VanZ-2

MBR2862093.1
Bacteroidaceae bacterium VanZ-2

MPZ99583.1
Dehalococcoidia bacterium VanZ-2

WP_161456933.1
Photobacterium halotolerans VanZ-2

MCA9085402.1
Planctomycetaceae bacterium VanZ-2

MBQ5733997.1
Lachnospiraceae bacterium VanZ-2

QDV67459.1
Rosistilla carotiformis VanZ-2

MBQ3599802.1
Lachnospiraceae bacterium VanZ-2

MCA9048029.1
Planctomycetaceae bacterium VanZ-2

MCD7838139.1
Clostridiales bacterium VanZ-2

NNF00040.1
Pyrinomonadaceae bacterium VanZ-2

MBR5516047.1
Clostridia bacterium VanZ-2

NPV45065.1
Bacillota bacterium VanZ-2

MBP3854898.1
Ruminiclostridium sp. VanZ-2

MBZ0113157.1
Thermoanaerobaculia bacterium VanZ-2

WP_144795918.1
Microbacterium paludicola VanZ-2

MBR3973392.1
Oscillospiraceae bacterium VanZ-2

KXG44166.1
Tepidibacillus decaturensis VanZ-2

WP_156609418.1
Auraticoccus cholistanensis VanZ-2

RJR39801.1
Deltaproteobacteria bacterium VanZ-2

MBR0431648.1
Candidatus Saccharibacteria bacterium VanZ-2

NBH32859.1
Clostridiaceae bacterium VanZ-2

HHY10275.1
Bacillota bacterium VanZ-2

MBO0800523.1
Blastocatellia bacterium VanZ-2

MBX3448082.1
Planctomycetaceae bacterium VanZ-2

MBR9955082.1
Eubacteriaceae bacterium Marseille-Q4139 VanZ-2

MBE5942084.1
Lachnospiraceae bacterium VanZ-2

TRZ81512.1
bacterium VanZ-2

MCA9328472.1
Candidatus Saccharibacteria bacterium VanZ-2

MBR4723949.1
Clostridia bacterium VanZ-2

MBU3758957.1
Candidatus Omnitrophota bacterium VanZ-2

MBQ2553686.1
Prevotella sp. VanZ-2

MBO4825055.1
Lachnospiraceae bacterium VanZ-2

HHX61645.1
Candidatus Epulonipiscium sp. VanZ-2

MBW1744688.1
Deltaproteobacteria bacterium VanZ-2

NBB80529.1
Verrucomicrobiota bacterium VanZ-2

HBN46754.1
Prevotella sp. VanZ-2

MBL9122930.1
Planctomycetaceae bacterium VanZ-2

HIU41425.1
Candidatus Egerieicola faecale VanZ-2

HIY89852.1
Candidatus Merdibacter merdipullorum VanZ-2

OSX94480.1
Bacillus mycoides VanZ-2

KPJ55460.1
Planctomycetes bacterium DG_58 VanZ-2

MBQ8579684.1
Oscillospiraceae bacterium VanZ-2

WP_088119899.1
Bacillus anthracis VanZ-2

WP_153552550.1
Halomicrobium sp. LC1Hm VanZ-2

PIV58027.1
Bacteroidetes bacterium CG02_land_8_20_14_3_00_31_25 VanZ-2

MBR3319886.1
Candidatus Saccharibacteria bacterium VanZ-2

NLX11514.1
Chloroflexota bacterium VanZ-2

MBO6795974.1
Maricaulis sp. VanZ-2

TXI84321.1
Crocinitomicaceae bacterium VanZ-2

MBR2263743.1
Bacillota bacterium VanZ-2

MYL53186.1
Pontibacillus yanchengensis VanZ-2

WP_051497038.1
Pseudomonas sp. BAY1663 VanZ-2

MBO5024160.1
Clostridia bacterium VanZ-2

MBU0903925.1
Bacillota bacterium VanZ-2

MCB2141186.1<br>*bacterium*	VanZ-2
MBR0511186.1<br>*Ruminococcus sp.*	VanZ-2
WP_185174083.1<br>*Rothia nasimurium*	VanZ-2
MCD7859453.1<br>*Bacillota bacterium*	VanZ-2
MBE6853960.1<br>*Ruminococcus sp.*	VanZ-2
MBX9718279.1<br>*Microbacteriaceae bacterium*	VanZ-2
MCB1982927.1<br>*Rhodoferax sp.*	VanZ-2
MBQ1930655.1<br>*Lachnospiraceae bacterium*	VanZ-2
NLB48424.1<br>*Erysipelotrichia bacterium*	VanZ-2
WP_106110128.1<br>*uncultured Actinomyces sp.*	VanZ-2
MBL6765855.1<br>*Verrucomicrobiia bacterium*	VanZ-2
MBE6858187.1<br>*Ruminococcus sp.*	VanZ-2
WP_013981112.1<br>*Eggerthella sp. YY7918*	VanZ-2
MBN2581595.1<br>*Planctomycetota bacterium*	VanZ-2
MBP0021048.1<br>*Cyanobacteria bacterium SBLK*	VanZ-2
MBQ7466765.1<br>*Clostridia bacterium*	VanZ-2
MBU1937678.1<br>*bacterium*	VanZ-2
MBR3661387.1<br>*Bacilli bacterium*	VanZ-2
OGP60794.1<br>*Deltaproteobacteria bacterium RBG_13_61_14*	VanZ-2
WP_214779617.1	VanZ-2
KZE63295.1<br>*Fictibacillus phosphorivorans*	VanZ-2
MBR1565360.1<br>*Oscillospiraceae bacterium*	VanZ-2
HDL89965.1<br>*Thermodesulforhabdus norvegica*	VanZ-2
NOR45019.1<br>*Candidatus Delongbacteria bacterium*	VanZ-2
MBV7411270.1<br>*Dermabacteraceae bacterium TAE3-ERU27*	VanZ-2
MBK7933002.1<br>*Acidobacteriota bacterium*	VanZ-2
MBU0973173.1<br>*Pseudomonadota bacterium*	VanZ-2
MBQ7678091.1<br>*Lachnospiraceae bacterium*	VanZ-2
MBU8906500.1<br>*Desertibacillus haloalkaliphilus*	VanZ-2
SCY49198.1<br>*Lachnospiraceae bacterium XPB1003*	VanZ-2
MBO8174239.1<br>*Thermococcus sp.*	VanZ-2
MBS1242720.1<br>*Nitrospirota bacterium*	VanZ-2
MBN9631792.1<br>*Actinomycetota bacterium*	VanZ-2
NBD35571.1<br>*Chloroflexota bacterium*	VanZ-2
MBN2429117.1<br>*Deltaproteobacteria bacterium*	VanZ-2
MBQ9347216.1<br>*Oscillibacter sp.*	VanZ-2
MBA2764877.1<br>*Thermoleophilaceae bacterium*	VanZ-2
RLI32346.1<br>*Candidatus Bathyarchaeota archaeon*	VanZ-2
PYV41126.1<br>*Acidobacteriota bacterium*	VanZ-2
MBL6724877.1<br>*Rubripirellula sp.*	VanZ-2
WP_079428197.1<br>*Clostridium oryzae*	VanZ-2
WP_063389006.1<br>*Aeribacillus sp. FSL K6-3256*	VanZ-2
BBU41221.1<br>*Aeribacillus pallidus*	VanZ-2
TDI66736.1<br>*Alphaproteobacteria bacterium*	VanZ-2
MBQ6405587.1<br>*Prevotella sp.*	VanZ-2
WP_132057930.1<br>*Halorussus amylolyticus*	VanZ-2
WP_066398205.1<br>*Cytobacillus eiseniae*	VanZ-2
MBR2048440.1<br>*Oscillospiraceae bacterium*	VanZ-2
PIE03060.1<br>*Acidobacteriota bacterium*	VanZ-2
WP_099422584.1	VanZ-2
MBS5794910.1<br>*Clostridiales bacterium*	VanZ-2
MBD5226279.1<br>*Bacteroidales bacterium*	VanZ-2
NLO62857.1<br>*Clostridiaceae bacterium*	VanZ-2
WP_155845895.1	VanZ-2
RKX30113.1<br>*Verrucomicrobiota bacterium*	VanZ-2
MBR3995829.1<br>*Clostridia bacterium*	VanZ-2
PWM65638.1<br>*Clostridiales bacterium*	VanZ-2
WP_028393453.1<br>*Peribacillus huizhouensis*	VanZ-2
MBD5303147.1<br>*Bacteroides sp.*	VanZ-2
WP_078545783.1<br>*Litchfieldia alkalitelluris*	VanZ-2
MBR1673939.1<br>*Eubacterium sp.*	VanZ-2
MBI2207444.1<br>*Candidatus Rokuibacteriota bacterium*	VanZ-2
MCB9739904.1<br>*Deltaproteobacteria bacterium*	VanZ-2
EOS51735.1<br>*Lachnospiraceae bacterium MD335*	VanZ-2
MBP6963087.1<br>*Armatimonadota bacterium*	VanZ-2
PLX10874.1<br>*Marinilabiliales bacterium*	VanZ-2
MBQ0071700.1<br>*Candidatus Physcosoma equi*	VanZ-2
MBL8455512.1<br>*Zoogloeaceae bacterium*	VanZ-2
WP_012872188.1<br>*Sphaerobacter thermophilus*	VanZ-2
MBR1937657.1<br>*Spirochaetales bacterium*	VanZ-2
WP_171050969.1<br>*Bacillus sp. BHET2*	VanZ-2
WP_194561994.1<br>*Savagea serpentis*	VanZ-2
MBM3500769.1<br>*Armatimonadota bacterium*	VanZ-2
MBV8772901.1<br>*Deltaproteobacteria bacterium*	VanZ-2
MBP7119215.1<br>*Candidatus Woesebacteria bacterium*	VanZ-2
HGH00262.1	VanZ-2
MBE9508350.1<br>*Chloroflexota bacterium*	VanZ-2
WP_003353474.1<br>*Bacillus smithii*	VanZ-2
MCA9390600.1<br>*candidate division WWE3 bacterium*	VanZ-2
WP_185277887.1<br>*Leifsonia shinshuensis*	VanZ-2
NYJ19964.1<br>*Leifsonia psychrotolerans*	VanZ-2
WP_152110923.1<br>*Bacillus sp. B1-b2*	VanZ-2
MBM7611072.1	VanZ-2
WP_025157118.1<br>*Leifsonia aquatica H1aii*	VanZ-2
WP_164515427.1<br>*Microbacterium sp. 10M-3C3*	VanZ-2
MBM68421.1<br>*Haliea sp.*	VanZ-2
OLD57506.1<br>*Acidobacteria bacterium 13_1_40CM_2_56_5*	VanZ-2
WP_233313739.1<br>*Niallia sp. BSM11*	VanZ-2
WP_223438620.1<br>*Metabacillus dongyingensis*	VanZ-2
WP_013388749.1<br>*Ilyobacter polytropus DSM 2926*	VanZ-2
MBW1835685.1<br>*Deltaproteobacteria bacterium*	VanZ-2
MCC6124897.1<br>*Pirellulales bacterium*	VanZ-2
PYP68037.1<br>*Gemmatimonadota bacterium*	VanZ-2

MBC7288610.1	*Armatimonadota bacterium*	VanZ-2
KAB7707361.1	*Bacillus aerolatus*	VanZ-2
WP_009819121.1	*Roseovarius sp. 217*	VanZ-2
MBR3679939.1	*Oscillospiraceae bacterium*	VanZ-2
MBN2290642.1	*Candidatus Glassbacteria bacterium*	VanZ-2
MBR4422376.1	*Erysipelotrichaceae bacterium*	VanZ-2
MCA0396409.1	*Bacteroidota bacterium*	VanZ-2
CCY66702.1	*Clostridium sp. CAG:678*	VanZ-2
PKL14074.1	*Spirochaetae bacterium HGW-Spirochaetae-8*	VanZ-2
PKL26844.1	*Spirochaetae bacterium HGW-Spirochaetae-2*	VanZ-2
MBC7724991.1	*Microbacteriaceae bacterium*	VanZ-2
WP_007203251.1	*Fictibacillus macauensis ZFHKF-1*	VanZ-2
POZ90607.1	*Petrotoga sp. SL27*	VanZ-2
WP_224721968.1	*Paenibacillus vietnamensis*	VanZ-2
RLE43096.1	*Candidatus Woesearchaeota archaeon*	VanZ-2
TDO45639.1	*Kribbella sp. VKM Ac-2571*	VanZ-2
MBQ1725072.1	*Muribaculaceae bacterium*	VanZ-2
OIO46446.1	*Parcubacteria group bacterium CG1_02_37_51*	VanZ-2
OGJ12863.1	*Candidatus Pacearchaeota archaeon RBG_19FT_COMBO_34_9*	VanZ-2
NLJ35736.1	*candidate division WS1 bacterium*	VanZ-2
WP_205182169.1	*Neobacillus cucumis*	VanZ-2
WP_191202096.1		VanZ-2
HIB84315.1	*Chromatiaceae bacterium*	VanZ-2
UCD50632.1	*Phycisphaerales bacterium*	VanZ-2
MBQ1822887.1	*Lachnospiraceae bacterium*	VanZ-2
MBP2423361.1		VanZ-2
WP_127602361.1	*Paenibacillus chibensis*	VanZ-2
NLE61255.1	*Planctomycetota bacterium*	VanZ-2
MBQ2953321.1	*Clostridia bacterium*	VanZ-2
MBQ4187797.1	*Bacillota bacterium*	VanZ-2
WP_225006121.1		VanZ-2
WP_192879357.1	*Microbulbifer sp. A4B17*	VanZ-2
MBL4828666.1	*Aliivibrio sp.*	VanZ-2
MBP3558752.1	*Thermoguttaceae bacterium*	VanZ-2
MBI9014130.1	*Clostridiales bacterium*	VanZ-2
MCB1724316.1	*Gammaproteobacteria bacterium*	VanZ-2
MBI5263384.1	*Bradyrhizobium sp.*	VanZ-2
MBQ6554594.1	*Bacillota bacterium*	VanZ-2
MBO5805126.1	*Bacteroidales bacterium*	VanZ-2
MCB0036833.1	*Anaerolineales bacterium*	VanZ-2
QDU61648.1	*Kolteria novifilia*	VanZ-2
MBQ7793038.1	*Clostridia bacterium*	VanZ-2
WP_059399634.1	*Azospirillum brasilense*	VanZ-2
WP_170130627.1	*Sphaerotilus hippei*	VanZ-2
HBT22593.1	*Verrucomicrobiales bacterium*	VanZ-2
MBK5097076.1	*Gemmatimonadota bacterium*	VanZ-2
HIV96778.1	*Candidatus Agathobaculum stercoravium*	VanZ-2
THC45089.1	*Massilia sp. Mn16-1_5*	VanZ-2
MBL9086378.1	*Planctomycetia bacterium*	VanZ-2
TNF01888.1	*Deltaproteobacteria bacterium*	VanZ-2
MBR2786198.1	*Clostridia bacterium*	VanZ-2
HBK04081.1	*Clostridiales bacterium*	VanZ-2
HCR04918.1	*Gemmatimonadota bacterium*	VanZ-2
MCC6108419.1	*Denitrobacterium sp.*	VanZ-2
HHU56331.1	*Acholeplasmataceae bacterium*	VanZ-2
MBI4040301.1	*Deltaproteobacteria bacterium*	VanZ-2
NLE39153.1	*Pirellulaceae bacterium*	VanZ-2
HCR65291.1	*Oceanicaulis sp.*	VanZ-2
MBL8951355.1	*Myxococcaceae bacterium*	VanZ-2
MBE34809.1	*Opitutaceae bacterium*	VanZ-2
MBK7511932.1	*Chloracidobacterium sp.*	VanZ-2
WP_204202751.1	*Bacillus suaedaesalsae*	VanZ-2
MBQ9979469.1	*Oscillospiraceae bacterium*	VanZ-2
WP_154308216.1	*Metabacillus lacus*	VanZ-2
WP_067297766.1	*Marinobacterium profundum*	VanZ-2
MBR6492958.1	*Paludibacteraceae bacterium*	VanZ-2
MBR3184333.1	*Bacillota bacterium*	VanZ-2
PKO20593.1	*Chloroflexi bacterium HGW-Chloroflexi-1*	VanZ-2
WP_232429754.1	*Janthinobacterium sp. CG3*	VanZ-2
RME48427.1	*Chloroflexota bacterium*	VanZ-2
MBC7261796.1	*Chloroflexota bacterium*	VanZ-2
MBP1699321.1	*Deltaproteobacteria bacterium*	VanZ-2
NLA75750.1	*Deltaproteobacteria bacterium*	VanZ-2
NMC56968.1	*Eubacteriaceae bacterium*	VanZ-2
HAT16343.1	*Gemmatimonadota bacterium*	VanZ-2
NLP08236.1	*Clostridiaceae bacterium*	VanZ-2
MBN8604061.1	*Planctomycetota bacterium*	VanZ-2
PTY03231.1	*Opitutaceae bacterium EW11*	VanZ-2
NUQ65729.1	*Pirellulales bacterium*	VanZ-2
NCU42070.1	*Candidatus Moraniibacteriota bacterium*	VanZ-2
MAI34544.1	*Rhodopirellula sp.*	VanZ-2
MBQ9032520.1	*Parasporobacterium sp.*	VanZ-2
EMZ23546.1		VanZ-2
KKI99386.1	*Prochlorothrix hollandica PCC 9006 = CALU 1027*	VanZ-2
NLH55109.1	*Acholeplasmataceae bacterium*	VanZ-2
WP_204955961.1	*Metabacillus crassostreae*	VanZ-2
MBR2967665.1	*Clostridia bacterium*	VanZ-2
WP_168880676.1	*Flammeovirga agarivorans*	VanZ-2
WP_224136496.1	*Metabacillus rhizolycopersici*	VanZ-2
PZN36548.1		VanZ-2
NLC62905.1	*Thermoanaerobacterales bacterium*	VanZ-2
MCA9899857.1	*Anaerolineales bacterium*	VanZ-2
OGS36505.1	*Elusimicrobia bacterium RIFOXYB2_FULL_49_7*	VanZ-2

WP_212695696.1	*Vallitalea pronyensis*	VanZ-2
TNF32824.1	*Deltaproteobacteria bacterium*	VanZ-2
WP_051350731.1	*Caloramator proteoclasticus DSM 10124*	VanZ-2
MBQ1827030.1	*Erysipelotrichaceae bacterium*	VanZ-2
MBE2232904.1	*Anaerolinea sp.*	VanZ-2
TVQ12884.1	*Balneolaceae bacterium*	VanZ-2
WP_140870019.1		VanZ-2
MBW2001687.1	*Deltaproteobacteria bacterium*	VanZ-2
WP_161836786.1	*Anaerocolumna sedimenticola*	VanZ-2
HHS81387.1		VanZ-2
MBX7103577.1	*Gemmataceae bacterium*	VanZ-2
WP_170290211.1	*Arthrobacter citreus*	VanZ-2
MBP3268351.1	*Ruminococcus sp.*	VanZ-2
MCB0340204.1	*Bdellovibrionales bacterium*	VanZ-2
HGU54682.1		VanZ-2
WP_077025411.1	*Fuerstiella marisgermanici*	VanZ-2
MBO5029326.1	*Muribaculaceae bacterium*	VanZ-2
WP_170258140.1	*Rhizobium leguminosarum*	VanZ-2
WP_100401489.1	*Bacillus sp. FJAT-42315*	VanZ-2
MBX3427344.1	*Pirellulales bacterium*	VanZ-2
MBO7472898.1	*Ruminococcus sp.*	VanZ-2
MBJ81703.1	*Acidimicrobiaceae bacterium*	VanZ-2
WP_161831837.1	*Turicibacter sp. KK003*	VanZ-2
MBR4991018.1	*Oscillospiraceae bacterium*	VanZ-2
KPL20735.1	*Anaerolineae bacterium SM23_84*	VanZ-2
MBR1476294.1	*Muribaculaceae bacterium*	VanZ-2
TAH63430.1	*Anaerolineaceae bacterium*	VanZ-2
MBO4908184.1	*Lachnospiraceae bacterium*	VanZ-2
MBI3320733.1	*Candidatus Omnitrophota bacterium*	VanZ-2
MBL0062470.1	*bacterium*	VanZ-2
WP_005212972.1	*Clostridium celatum*	VanZ-2
MBR7075982.1		VanZ-2
MCB9223704.1	*Crocinitomicaceae bacterium*	VanZ-2
MBC8127800.1	*Verrucomicrobiales bacterium*	VanZ-2
MBN2859311.1	*Sphaerochaetaceae bacterium*	VanZ-2
MBE6821257.1	*Oscillospiraceae bacterium*	VanZ-2
NQT38581.1	*Planctomycetota bacterium*	VanZ-2
PRR81748.1	*Clostridium vincentii*	VanZ-2
MBR4027415.1	*Lachnospiraceae bacterium*	VanZ-2
MBI1279574.1	*Anaerolineaceae bacterium*	VanZ-2
WP_185201415.1	*Glaciihabitans sp. INWT7*	VanZ-2
MBI4643376.1	*Deltaproteobacteria bacterium*	VanZ-2
MBO5410560.1	*Clostridia bacterium*	VanZ-2
QPC48451.1	*Mangrovibacillus cuniculi*	VanZ-2
WP_204553682.1	*Bacillus ectoiniformans*	VanZ-2
WP_204592333.1		VanZ-2
OPZ34498.1	*Tenericutes bacterium ADurb.BinA155*	VanZ-2
WP_214476474.1	*Cytobacillus gottheilii*	VanZ-2
WP_223701078.1	*Sutcliffiella deserti*	VanZ-2
MBR6784805.1	*Clostridia bacterium*	VanZ-2
MCB0825126.1	*Armatimonadota bacterium*	VanZ-2
HEY72695.1	*Thermoflexia bacterium*	VanZ-2
MCC8175177.1	*Bacteroidales bacterium*	VanZ-2
OLE53494.1	*Acidobacteria bacterium 13_1_20CM_3_53_8*	VanZ-2
MBR6726513.1	*Clostridia bacterium*	VanZ-2
MBQ1206479.1	*Clostridia bacterium*	VanZ-2
WP_181706370.1	*Chthonobacter rhizosphaerae*	VanZ-2
MBI5190177.1	*Nitrospirota bacterium*	VanZ-2
WP_223695170.1	*Leifsonia poae*	VanZ-2
MBQ8026516.1	*Clostridia bacterium*	VanZ-2
MBS5566007.1	*Bacillota bacterium*	VanZ-2
MBT8351187.1	*Deltaproteobacteria bacterium*	VanZ-2
MCC6857082.1	*Bryobacterales bacterium*	VanZ-2
NOZ05474.1	*Chloroflexota bacterium*	VanZ-2
NLD35663.1	*Desulfatiglans sp.*	VanZ-2
MBE6116011.1	*Erysipelotrichaceae bacterium*	VanZ-2
HEC40462.1	*bacterium*	VanZ-2
MBR9988392.1	*Gemmatimonadota bacterium*	VanZ-2
WP_196828532.1	*Frigoribacterium sp. CG_9.8*	VanZ-2
HID46300.1	*Chromatiaceae bacterium*	VanZ-2
NOY88926.1	*FCB group bacterium*	VanZ-2
WP_215077408.1		VanZ-2
OAH54444.1	*Domibacillus aminovorans*	VanZ-2
MBC8583975.1	*Youxingia wuxianensis*	VanZ-2
WP_076110725.1	*Paenibacillus sp. FSL H3-0319*	VanZ-2
MBK6698018.1	*Myxococcales bacterium*	VanZ-2
MBO4453278.1	*Clostridia bacterium*	VanZ-2
NLL34373.1	*Clostridiales bacterium*	VanZ-2
WP_063661000.1	*Comamonas thiooxydans*	VanZ-2
MBO4517380.1	*Clostridia bacterium*	VanZ-2
MBR3326454.1	*Atopobiaceae bacterium*	VanZ-2
WP_152661231.1	*Halioglobus maricola*	VanZ-2
MCB0206070.1	*Anaerolineae bacterium*	VanZ-2
WP_171042302.1		VanZ-2
MBI3636218.1	*Candidatus Rokuibacteriota bacterium*	VanZ-2
WP_184215512.1	*Microbacterium marinum*	VanZ-2
MBK8013649.1	*Deltaproteobacteria bacterium*	VanZ-2
WP_185137037.1	*Cohnella xylanilytica*	VanZ-2
WP_185192844.1	*Halobellus ruber*	VanZ-2
NQS76946.1	*Candidatus Syntrophopropionicum ammoniitolerans*	VanZ-2
WP_009491669.1	*Microvirga lotononidis*	VanZ-2
HHU37556.1	*Treponema sp.*	VanZ-2
HDP95271.1	*Candidatus Aminicenantes bacterium*	VanZ-2

MBD5310364.1	*Bacteroides sp.*	VanZ-2
PKM66581.1	*Firmicutes bacterium HGW-Firmicutes-19*	VanZ-2
MBR4859080.1	*Clostridia bacterium*	VanZ-2
MBC8877064.1	*Planctomycetota bacterium*	VanZ-2
MBU50154.1	*Deltaproteobacteria bacterium*	VanZ-2
QII83146.1	*Jeotgalibaca arthritidis*	VanZ-2
WP_145090045.1	*Rosistilla carotiformis*	VanZ-2
HEN17766.1		VanZ-2
MBR4128315.1	*Bacteroidaceae bacterium*	VanZ-2
MBQ9533747.1	*Prevotella sp.*	VanZ-2
HFQ81083.1	*Desulfobacterales bacterium*	VanZ-2
NJN38367.1	*Acaryochloridaceae cyanobacterium CSU_3_4*	VanZ-2
MBQ4632343.1	*Prevotella sp.*	VanZ-2
HDD25247.1	*Chloroflexota bacterium*	VanZ-2
WP_010267339.1	*Paenibacillus senegalensis*	VanZ-2
WP_025777206.1	*Brevibacterium sp. VCM10*	VanZ-2
PKM86039.1	*Firmicutes bacterium HGW-Firmicutes-10*	VanZ-2
MBC7225537.1	*Anaerolineae bacterium*	VanZ-2
MBN2419751.1	*Deltaproteobacteria bacterium*	VanZ-2
WP_143132307.1	*Clostridioides difficile*	VanZ-2
MCB1058912.1	*Calditrichota bacterium*	VanZ-2
MBP9865136.1	*Candidatus Omnitrophota bacterium*	VanZ-2
MBL8082426.1	*Candidatus Obscuribacter sp.*	VanZ-2
WP_003351780.1	*Bacillus methanolicus PB1*	VanZ-2
WP_100332462.1	*Bacillus xiapuensis*	VanZ-2
AFY94488.1	*Chamaesiphon minutus PCC 6605*	VanZ-2
MBN2397858.1	*Deltaproteobacteria bacterium*	VanZ-2
MBP3388041.1	*Clostridia bacterium*	VanZ-2
WP_119320473.1	*Capsulimonas corticalis*	VanZ-2
MBU0507741.1	*bacterium*	VanZ-2
MBP5642122.1	*Paludibacteraceae bacterium*	VanZ-2
MBO4855442.1	*Candidatus Saccharibacteria bacterium*	VanZ-2
OQA18838.1	*Firmicutes bacterium ADurb.Bin354*	VanZ-2
MBR1875222.1	*Candidatus Saccharibacteria bacterium*	VanZ-2
WP_164779538.1	*Paenibacillus kobensis NBRC 15729*	VanZ-2
HBH54860.1	*Planctomycetaceae bacterium*	VanZ-2
MBP6577646.1	*Chryseobacterium sp.*	VanZ-2
MBN1418966.1	*Planctomycetota bacterium*	VanZ-2
RKD30123.1	*Thermohalobacter berrensis*	VanZ-2
MBQ5687321.1	*Ruminococcus sp.*	VanZ-2
WP_103206738.1	*Microbacterium sp. AG157*	VanZ-2
WP_006076553.1	*Halococcus saccharolyticus DSM 5350*	VanZ-2
TGX80810.1	*Palleniella muris*	VanZ-2
MBU4086807.1	*Nanoarchaeota archaeon*	VanZ-2
MBL7993188.1	*Candidatus Kapaibacterium sp.*	VanZ-2
NMC15547.1	*Chloroflexota bacterium*	VanZ-2
TAL67739.1	*Bacteroidota bacterium*	VanZ-2
VVB61035.1	*uncultured archaeon*	VanZ-2
MBQ2990921.1	*Clostridia bacterium*	VanZ-2
TRL41958.1	*Rhizobium straminoryzae*	VanZ-2
MBQ1409809.1	*Oscillospiraceae bacterium*	VanZ-2
MBO5006830.1	*Clostridia bacterium*	VanZ-2
MBE5929019.1	*Lachnospiraceae bacterium*	VanZ-2
HIW84543.1	*Candidatus Dorea gallistercoris*	VanZ-2
MCC7205130.1	*Phycisphaeraceae bacterium*	VanZ-2
WP_128476488.1	*Halorussus pelagicus*	VanZ-2
MCD6510500.1	*Thermoprotei archaeon*	VanZ-2
HAJ27611.1	*Syntrophus sp. (in: bacteria)*	VanZ-2
MBM4200810.1	*Gammaproteobacteria bacterium*	VanZ-2
MBQ3497657.1	*Clostridia bacterium*	VanZ-2
WP_115864039.1	*Halorussus litoreus*	VanZ-2
CCZ52245.1	*Clostridium sp. CAG:75*	VanZ-2
WP_075617194.1	*Paenisporosarcina indica*	VanZ-2
MBV8907261.1	*Terriglobia bacterium*	VanZ-2
WP_106011034.1	*Clostridium luticellarii*	VanZ-2
MBQ1344353.1	*Candidatus Saccharibacteria bacterium*	VanZ-2
MBQ2637798.1	*Candidatus Saccharibacteria bacterium*	VanZ-2
MBR5562215.1	*Clostridia bacterium*	VanZ-2
MBQ3274997.1	*Candidatus Saccharibacteria bacterium*	VanZ-2
MBR4865584.1	*Clostridia bacterium*	VanZ-2
MBM4258399.1	*Deltaproteobacteria bacterium*	VanZ-2
WP_023394029.1	*Candidatus Halobonum tyrrellensis G22*	VanZ-2
MYE22993.1	*Gammaproteobacteria bacterium*	VanZ-2
OFV93276.1	*Acidobacteria bacterium RIFCSPLOWO2_02_FULL_64_15*	VanZ-2
MBP7936795.1	*Phycisphaerae bacterium*	VanZ-2
WP_026563123.1	*Bacillus sp. J37*	VanZ-2
MBO2481699.1		VanZ-2
HDS00823.1	*Candidatus Zixiibacteriota bacterium*	VanZ-2
WP_013386857.1	*Ilyobacter polytropus DSM 2926*	VanZ-2
RIK58968.1	*Candidatus Zhuqueibacterota bacterium*	VanZ-2
MBN2528630.1	*Deltaproteobacteria bacterium*	VanZ-2
MBE9548365.1	*Pseudomonadota bacterium*	VanZ-2
WP_091002331.1		VanZ-2
WP_105185194.1	*Micropruina glycogenica*	VanZ-2
MBK6808131.1	*Sandaracinaceae bacterium*	VanZ-2
RZO61391.1	*Sandaracinaceae bacterium*	VanZ-2
WP_101518682.1	*Pseudohalioglobus lutimaris*	VanZ-2
MBS7297711.1	*Eubacteriales bacterium*	VanZ-2
TVQ00534.1	*Balneolaceae bacterium*	VanZ-2
WP_115797611.1	*Halorussus sp. JP-T4*	VanZ-2
MBQ8514805.1	*Ruminococcus sp.*	VanZ-2
MBD8043079.1	*Arthrobacter pullicola*	VanZ-2
MBX3235424.1	*Nitrospiraceae bacterium*	VanZ-2

Accession	Organism	Gene
MBQ7822438.1	*Clostridia bacterium*	VanZ-2
MBE7480918.1	*Polyangiaceae bacterium*	VanZ-2
MBP7822497.1	*Candidatus Moraniibacteriota bacterium*	VanZ-2
QQR77210.1	*Candidatus Moraniibacteriota bacterium*	VanZ-2
GBD32633.1	*bacterium HR33*	VanZ-2
MBD7949266.1	*Oerskovia rustica*	VanZ-2
WP_162656666.1	*Tuwongella immobilis*	VanZ-2
MBK8089053.1	*Chitinophagaceae bacterium*	VanZ-2
PIZ98484.1	*Candidatus Komeilibacteria bacterium CG_4_10_14_0_2_um_filter_37_10*	VanZ-2
MBC8384420.1	*Candidatus Cloacimonadota bacterium*	VanZ-2
WP_099598576.1	*Reichenbachiella sp. 5M10*	VanZ-2
MBD8924639.1	*Agathobacter rectalis*	VanZ-2
WP_013457096.1	*Oceanithermus profundus DSM 14977*	VanZ-2
MCD6482952.1	*Candidatus Aenigmatarchaeota archaeon*	VanZ-2
CDK00004.1	*Microbacterium sp. C448*	VanZ-2
MBR2695024.1	*Thermoguttaceae bacterium*	VanZ-2
MBQ3784153.1	*Lachnospiraceae bacterium*	VanZ-2
MBQ8015032.1	*Clostridia bacterium*	VanZ-2
MBP8988721.1	*Clostridia bacterium*	VanZ-2
MCD6149893.1	*bacterium*	VanZ-2
MCA9576817.1	*Myxococcales bacterium*	VanZ-2
MBU6162635.1	*Myxococcales bacterium*	VanZ-2
HEY57732.1	*Anaerolineae bacterium*	VanZ-2
MBE6659655.1	*Oscillospiraceae bacterium*	VanZ-2
MBN2564771.1	*Candidatus Eiseniibacteriota bacterium*	VanZ-2
MBE5997194.1	*Lachnospiraceae bacterium*	VanZ-2
WP_169566285.1	*Microbacterium luticocti DSM 19459*	VanZ-2
MBO5914669.1	*Clostridia bacterium*	VanZ-2
MBR6445880.1	*Prevotella sp.*	VanZ-2
MBI5555820.1	*Elusimicrobiota bacterium*	VanZ-2
MBR0523895.1	*Prevotella sp.*	VanZ-2
MBA1147635.1	*Ectothiorhodospiraceae bacterium WFHF3C12*	VanZ-2
OQA22218.1	*Chloroflexi bacterium ADurb.Bin360*	VanZ-2
MBS1801742.1	*Acidobacteriota bacterium*	VanZ-2
MBP3614621.1	*Bacteroidaceae bacterium*	VanZ-2
HFX15792.1		VanZ-2
WP_026488365.1	*Butyrivibrio sp. XBB1001*	VanZ-2
MBN1509907.1	*Sedimentisphaerales bacterium*	VanZ-2
MBE6843968.1	*Ruminococcus sp.*	VanZ-2
MBO6201262.1	*Chryseobacterium sp.*	VanZ-2
NNF76430.1	*Hyphomicrobiales bacterium*	VanZ-2
MBQ3842266.1	*Ruminiclostridium sp.*	VanZ-2
RKZ74618.1	*Candidatus Parabeggiatoa sp. nov. 3*	VanZ-2
MBN1995718.1	*Anaerolineae bacterium*	VanZ-2
MBO8428980.1	*Candidatus Egerieousia excrementavium*	VanZ-2
HHU06562.1	*Clostridiales bacterium*	VanZ-2
WP_161919119.1	*Pallidibacillus pasinlerensis*	VanZ-2
HIX82300.1	*Candidatus Erysipelatoclostridium merdavium*	VanZ-2
MBX7241481.1	*Bacteroidia bacterium*	VanZ-2
TLM97384.1	*Actinomycetota bacterium*	VanZ-2
EQD07894.1	*Neisseria meningitidis NM151*	VanZ-2
OSC63213.1	*Streptomyces sp. 4F*	VanZ-2
WP_087298585.1	*uncultured Thomasclavelia sp.*	VanZ-2
MBR2280379.1	*Ruminococcus sp.*	VanZ-2
WP_087372416.1		VanZ-2
MBP3765549.1	*Bacilli bacterium*	VanZ-2
GEC96512.1	*Zoogloea ramigera*	VanZ-2
MCA9075823.1	*Planctomycetaceae bacterium*	VanZ-2
MBO6006981.1	*Lachnospiraceae bacterium*	VanZ-2
MBD5541081.1	*Lachnospiraceae bacterium*	VanZ-2
MBN1592850.1	*Candidatus Coatesbacteria bacterium*	VanZ-2
MBQ3202844.1	*Clostridia bacterium*	VanZ-2
MBT5923373.1	*Cellvibrionales bacterium*	VanZ-2
MCC6614294.1	*Anaerolineae bacterium*	VanZ-2
WP_103425706.1	*Salinigranum rubrum*	VanZ-2
PSP56067.1	*Halobacteriales archaeon QS_1_67_19*	VanZ-2
MBQ2973608.1	*Clostridia bacterium*	VanZ-2
MBI3406547.1	*Acidobacteriota bacterium*	VanZ-2
MBR4280776.1	*Clostridia bacterium*	VanZ-2
GIO41686.1	*Paenibacillus apis*	VanZ-2
RME01794.1	*Calditrichota bacterium*	VanZ-2
CDB44978.1	*Phascolarctobacterium faecium*	VanZ-2
QDT11051.1	*Stieleria marina*	VanZ-2
WP_129486203.1	*Fusibacter sp. A2*	VanZ-2
MBQ3044521.1	*Clostridia bacterium*	VanZ-2
MBC9897817.1		VanZ-2
NLY47654.1	*Clostridiales bacterium*	VanZ-2
MBQ4297885.1	*Clostridia bacterium*	VanZ-2
MBO5502094.1	*Clostridia bacterium*	VanZ-2
MCC8073429.1	*Clostridiales bacterium*	VanZ-2
MBW1979837.1	*Deltaproteobacteria bacterium*	VanZ-2
MBQ3583554.1	*Lachnospiraceae bacterium*	VanZ-2
NLY48398.1		VanZ-2
MBR6696715.1	*Lachnospiraceae bacterium*	VanZ-2
WP_060679353.1	*Virgibacillus halodenitrificans JCM 12304*	VanZ-2
MCB0271483.1	*Bdellovibrionales bacterium*	VanZ-2
WP_162355999.1	*Paenibacillus lycopersici*	VanZ-2
NLP28725.1	*Clostridia bacterium*	VanZ-2
MBQ3090880.1	*Oscillospiraceae bacterium*	VanZ-2
MCA3239902.1	*Rubrivivax sp.*	VanZ-2
MPQ43319.1	*Clostridium tarantellae*	VanZ-2
MCC7360219.1	*Anaerolineales bacterium*	VanZ-2
OQA39550.1	*Parcubacteria group bacterium ADurb.Bin316*	VanZ-2

WP_170184242.1
Glutamicibacter uratoxydans VanZ-2

MBQ1476918.1
Erysipelotrichaceae bacterium VanZ-2

HDR14263.1
Desulfobacteraceae bacterium VanZ-2

MBR7185342.1
Clostridia bacterium VanZ-2

NLI86100.1
Propionibacterium sp. VanZ-2

NNG01775.1
Desulfobacteraceae bacterium VanZ-2

WP_063182644.1
Paenibacillus elgii VanZ-2

GBF77913.1
Paenibacillus sp. 598K VanZ-2

MBE6927427.1
Oscillospiraceae bacterium VanZ-2

GEP70386.1
Cellulomonas soli VanZ-2

MBM3765623.1
Acidobacteriota bacterium VanZ-2

MBN9523916.1
bacterium VanZ-2

MBO6046312.1
Erysipelotrichaceae bacterium VanZ-2

WP_100075439.1
Chryseobacterium camelliae VanZ-2

NQS88309.1
Patescibacteria group bacterium VanZ-2

MBN9501143.1
Armatimonadota bacterium VanZ-2

TAJ11216.1
Marinilabiliaceae bacterium JC017 VanZ-2

NLL46703.1
Clostridiales bacterium VanZ-2

WP_075981908.1
Bacillus massilionigeriensis VanZ-2

NGY80496.1
Priestia megaterium VanZ-2

MBO4609643.1
Lachnospiraceae bacterium VanZ-2

HGF34892.1 VanZ-2

PVC76053.1
Priestia megaterium VanZ-2

MBS1957577.1
Cyanobacteria bacterium SZAS-4 VanZ-2

MCD6014995.1
Solirubrobacterales bacterium VanZ-2

HGS38960.1 VanZ-2

HJA46021.1
Candidatus Phascolarctobacterium stercoravium VanZ-2

WP_027448670.1
Pontibacillus marinus BH030004 = DSM 16465 VanZ-2

ALS30176.1
Paenibacillus sp. 32O-W VanZ-2

TBN53285.1
Hansschlegelia quercus VanZ-2

NMM38589.1
Glaciimonas sp. VanZ-2

MBQ5824680.1
Clostridia bacterium VanZ-2

MBM3151956.1
Chloroflexota bacterium VanZ-2

MBM7106954.1
Brevibacillus laterosporus VanZ-2

MCB0711238.1
Ignavibacteriota bacterium VanZ-2

MBC8249266.1
Anaerolineales bacterium VanZ-2

HCO19819.1
Flavobacteriaceae bacterium VanZ-2

WP_090833307.1
Bacillus sp. cl95 VanZ-2

NLJ15051.1
Ignavibacteria bacterium VanZ-2

NCQ18827.1
Ignavibacteria bacterium VanZ-2

MBF5080550.1
Quadrisphaera sp. INWT6 VanZ-2

WP_169212468.1 VanZ-2

HAL63096.1
Clostridiales bacterium VanZ-2

UCD25229.1
Gemmatimonadota bacterium VanZ-2

TQV61901.1
Sulfurovum sp. VanZ-2

HHT08459.1
Clostridiales bacterium VanZ-2

MBQ4316877.1
Clostridia bacterium VanZ-2

WP_216020706.1
Colwellia sp. E2M01 VanZ-2

NUN94116.1
Verrucomicrobiia bacterium VanZ-2

MBF0429758.1
Fibrobacteria bacterium VanZ-2

MCC5992073.1
Paracoccaceae bacterium VanZ-2

MBK8871752.1
Elusimicrobiota bacterium VanZ-2

OQA89749.1
Elusimicrobia bacterium ADurb.Bin231 VanZ-2

WP_153722056.1
Sporosarcina cascadiensis VanZ-2

MBX3751359.1
Opitutaceae bacterium VanZ-2

MBI9056616.1
Labilibaculum sp. VanZ-2

MBO4824975.1
Lachnospiraceae bacterium VanZ-2

WP_007983629.1
Haladaptatus paucihalophilus DX253 VanZ-2

MBI3993007.1
Candidatus Lambdaproteobacteria bacterium VanZ-2

OQA63558.1
Firmicutes bacterium ADurb.Bin262 VanZ-2

MBO5090942.1
Clostridia bacterium VanZ-2

MBO5059843.1
Clostridia bacterium VanZ-2

MBS6280470.1
Lachnospiraceae bacterium VanZ-2

MBR6097706.1
Candidatus Saccharibacteria bacterium VanZ-2

WP_192168611.1
Aeromicrobium sp. CFBP 8757 VanZ-2

TAE93352.1
Verrucomicrobiota bacterium VanZ-2

AKU90835.1
Vulgatibacter incomptus VanZ-2

WP_183592062.1
Nocardioides soli VanZ-2

MCB1759992.1
Gammaproteobacteria bacterium VanZ-2

MBH1986402.1
Burkholderiales bacterium VanZ-2

MBI3329535.1
Nitrospinota bacterium VanZ-2

MBR4164222.1
Solobacterium sp. VanZ-2

MBQ9684388.1
Candidatus Saccharibacteria bacterium VanZ-2

MBP3311491.1
Butyricicoccus sp. VanZ-2

MBR5507134.1
Clostridia bacterium VanZ-2

MBQ3352615.1
Candidatus Saccharibacteria bacterium VanZ-2

MBL8784977.1
Deltaproteobacteria bacterium VanZ-2

MBK6683421.1
Deltaproteobacteria bacterium VanZ-2

MBR6748486.1
Clostridia bacterium VanZ-2

WP_080454964.1
Roseovarius sp. A-2 VanZ-2

MBQ8391019.1
Clostridia bacterium VanZ-2

RWZ54482.1
Halobacillus fulvus VanZ-2

MBD3249190.1
Candidatus Woesearchaeota archaeon VanZ-2

WP_151636457.1
Noviherbaspirillum aerium VanZ-2

MSA69798.1
Holdemania massiliensis VanZ-2

MCC8043057.1
Oscillospiraceae bacterium VanZ-2

CAA9261265.1
uncultured Chloroflexia bacterium VanZ-2

MCB9557469.1
Deltaproteobacteria bacterium VanZ-2

MBR3780829.1
Clostridia bacterium VanZ-2

MBW8882484.1
Asticcacaulis sp. VanZ-2

MBR3900861.1
Ruminococcus sp. VanZ-2

WP_051176421.1
Luteimonas mephitis VanZ-2

WP_215219328.1
Novilysobacter luteus VanZ-2

THJ16462.1	*Nitrospira sp. CG24D*	VanZ-2
MCA9298993.1	*Phycisphaerales bacterium*	VanZ-2
NLG10374.1	*Coriobacteriaceae bacterium*	VanZ-2
PYL05221.1	*Verrucomicrobiota bacterium*	VanZ-2
OQW85731.1	*Rhodoferax ferrireducens*	VanZ-2
MBF0944945.1	*Alloprevotella sp.*	VanZ-2
TBW58774.1	*Marinobacter halodurans*	VanZ-2
MCB1045509.1	*Acidobacteriota bacterium*	VanZ-2
MBR6580920.1	*Ruminococcus sp.*	VanZ-2
WP_014902895.1	*Desulfosporosinus meridiei DSM 13257*	VanZ-2
KUO53634.1	*Desulfitibacter sp. BRH_c19*	VanZ-2
NLX60910.1	*Tissierellia bacterium*	VanZ-2
NNM29098.1	*Akkermansiaceae bacterium*	VanZ-2
MBO0698507.1	*Zavarzinella sp.*	VanZ-2
MBP5624765.1	*Lachnospiraceae bacterium*	VanZ-2
NWF74777.1	*Nitrospirota bacterium*	VanZ-2
MCD6578706.1	*bacterium*	VanZ-2
WP_063779851.1	*Microvirga massiliensis*	VanZ-2
MBR6119679.1	*Oscillospiraceae bacterium*	VanZ-2
WP_196002049.1	*Clostridium sp. 1001271B_151109_B4*	VanZ-2
MCB2230546.1	*bacterium*	VanZ-2
MBQ6269631.1	*Bacteroidota bacterium*	VanZ-2
UCF79394.1	*Candidatus Eiseniibacteriota bacterium*	VanZ-2
OLS25922.1	*Candidatus Heimdallarchaeota archaeon LC_3*	VanZ-2
WP_152052073.1	*Tautonia marina*	VanZ-2
WP_120512931.1	*Photobacterium salinisoli*	VanZ-2
PRB71307.1	*Arthrobacter sp. MYb213*	VanZ-2
HAN87019.1	*Bacillota bacterium*	VanZ-2
MBR5531486.1	*Bacteroidaceae bacterium*	VanZ-2
MBW1686681.1	*Deltaproteobacteria bacterium*	VanZ-2
WP_192029184.1	*Pseudomarimonas arenosa*	VanZ-2
MBP5226782.1	*Kiritimatiellia bacterium*	VanZ-2
AWZ49979.1	*Clostridiaceae bacterium 14S0207*	VanZ-2
NIP13776.1	*Pseudomonadales bacterium*	VanZ-2
NBJ75864.1		VanZ-2
HBT77061.1	*Planctomycetaceae bacterium*	VanZ-2
WP_101642364.1	*Peribacillus deserti*	VanZ-2
WP_012778137.1	*Hirschia baltica ATCC 49814*	VanZ-2
TVS08947.1	*Planctomycetaceae bacterium*	VanZ-2
WP_006783514.1	*Turicibacter sanguinis*	VanZ-2
WP_224630744.1	*Mesorhizobium sp. CA10*	VanZ-2
WP_051413866.1	*Planococcus glaciei CHR43*	VanZ-2
MBA29403.1	*Hyphomonadaceae bacterium*	VanZ-2
MBC7644545.1	*Thermoleophilia bacterium*	VanZ-2
MBG87717.1	*Verrucomicrobiales bacterium*	VanZ-2
RSK29455.1	*Bacillus sp. HMF5848*	VanZ-2
MBN8592079.1	*Anaerolineae bacterium*	VanZ-2
MBO7337909.1	*Paludibacteraceae bacterium*	VanZ-2
HIU29499.1	*Candidatus Egerieisoma faecipullorum*	VanZ-2
WP_144233650.1	*Oceanidesulfovibrio marinus*	VanZ-2
MBI1255994.1	*Chloroflexota bacterium*	VanZ-2
MCA9098798.1	*Planctomycetaceae bacterium*	VanZ-2
NCQ24838.1	*Roseovarius sp.*	VanZ-2
MBM6985165.1	*Acidaminococcaceae bacterium*	VanZ-2
MBQ2903140.1	*Clostridia bacterium*	VanZ-2
NLM25458.1	*Bacillota bacterium*	VanZ-2
NQX02861.1	*bacterium*	VanZ-2
MBS5426186.1	*Phascolarctobacterium succinatutens*	VanZ-2
MBV6499626.1	*Prosthecobacter sp.*	VanZ-2
MBQ7017258.1	*Bacillota bacterium*	VanZ-2
WP_163241020.1		VanZ-2
MCC7139060.1	*Planctomycetota bacterium*	VanZ-2
UCF34167.1	*Phycisphaerales bacterium*	VanZ-2
MBO4753955.1	*Bacteroidales bacterium*	VanZ-2
MBF1591114.1	*Hoylesella shahii*	VanZ-2
TET33539.1	*Planctomycetota bacterium*	VanZ-2
MBO5664461.1	*Bacteroidales bacterium*	VanZ-2
MCA6439346.1	*Chitinophagaceae bacterium*	VanZ-2
MBQ7690041.1	*Muribaculaceae bacterium*	VanZ-2
MBN1756542.1	*bacterium*	VanZ-2
WP_102215636.1	*Gleimia hominis*	VanZ-2
MBC8290490.1	*Planctomycetota bacterium*	VanZ-2
WP_068487681.1	*Planococcus maritimus*	VanZ-2
WP_231592758.1	*Bacillus glycinifermentans*	VanZ-2
WP_075690435.1	*Bacillus sp. MRMR6*	VanZ-2
MBT3310514.1	*Desulfobacteraceae bacterium*	VanZ-2
NLD71970.1	*Chloroflexota bacterium*	VanZ-2
MBR2471486.1	*Clostridia bacterium*	VanZ-2
MBN1938889.1	*Candidatus Aminicenantes bacterium*	VanZ-2
MBN2626546.1	*Spirochaetales bacterium*	VanZ-2
WP_091058796.1	*Opitutus sp. GAS368*	VanZ-2
MBT3272472.1	*Spirochaetales bacterium*	VanZ-2
MBH65752.1	*Chloroflexota bacterium*	VanZ-2
MBD5310643.1	*Bacteroides sp.*	VanZ-2
WP_051259545.1	*Epilithonimonas tenax DSM 16811*	VanZ-2
MBT3480994.1	*Opitutales bacterium*	VanZ-2
HIS55613.1	*Candidatus Fimimorpha excrementavium*	VanZ-2
MBA4220206.1	*Methylobacterium sp.*	VanZ-2
HGW54232.1		VanZ-2
HDQ72234.1	*Chloroflexota bacterium*	VanZ-2
MBE7049320.1	*Oscillospiraceae bacterium*	VanZ-2
WP_190580951.1		VanZ-2
MBM3666190.1	*Actinomycetota bacterium*	VanZ-2

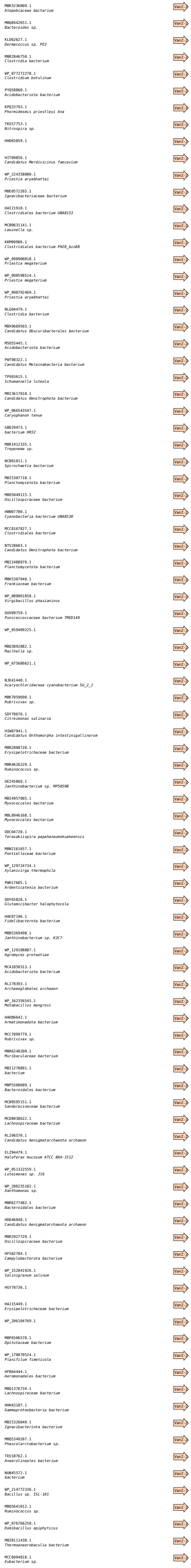

MBR3236009.1
Atopobiaceae bacterium
MBQ8442651.1
Bacteroides sp.
KLO62627.1
Dermacoccus sp. PE3
MBR2846750.1
Clostridia bacterium
WP_077272278.1
Clostridium botulinum
PYQ58868.1
Acidobacteriota bacterium
KPQ33793.1
Phormidesmis priestleyi Ana
TKS57753.1
Nitrospira sp.
HHD65059.1
HIT09856.1
Candidatus Merdivicinus faecavium
WP_224358080.1
Priestia aryabhattai
MBE0572265.1
Ignavibacteriaceae bacterium
HAI21918.1
Clostridiales bacterium UBA8153
MCB0631141.1
Lewinella sp.
KKM09906.1
Clostridiales bacterium PH28_bin88
WP_098900818.1
Priestia megaterium
WP_098598514.1
Priestia megaterium
WP_098792469.1
Priestia aryabhattai
NLG04479.1
Clostridia bacterium
MBX9669503.1
Candidatus Obscuribacterales bacterium
MSO55445.1
Acidobacteriota bacterium
PWT98322.1
Candidatus Melainabacteria bacterium
TPX03615.1
Schumannella luteola
MBI3617018.1
Candidatus Omnitrophota bacterium
WP_066543547.1
Caryophanon tenue
GBD29473.1
bacterium HR32
MBR1912335.1
Treponema sp.
NCB01011.1
Spirochaetia bacterium
MBI5307718.1
Planctomycetota bacterium
MBO5049115.1
Oscillospiraceae bacterium
HBN07700.1
Cyanobacteria bacterium UBA8530
MCC8167827.1
Clostridiales bacterium
NTV28603.1
Candidatus Omnitrophota bacterium
MBI2488979.1
Planctomycetota bacterium
MBK5307948.1
Frankiaceae bacterium
WP_089061858.1
Virgibacillus phasianinus
OUV89759.1
Puniceicoccaceae bacterium TMED149
WP_059409225.1
MBQ3892882.1
Mailhella sp.
WP_075686621.1
NJK41440.1
Acaryochloridaceae cyanobacterium SU_2_1
MBK7059690.1
Rubrivivax sp.
SDY70676.1
Citreimonas salinaria
HIW87941.1
Candidatus Onthomorpha intestinigallinarum
MBR2808728.1
Erysipelotrichaceae bacterium
MBR4626229.1
Ruminococcus sp.
OEZ45869.1
Janthinobacterium sp. MP5059B
MBI4957005.1
Myxococcales bacterium
MBL8946168.1
Myxococcales bacterium
ODC04720.1
Terasakiispira papahanaumokuakeensis
MBN2161657.1
Pontiellaceae bacterium
WP_129724734.1
Xylanivirga thermophila
PWH17685.1
Ardenticatenia bacterium
QDY65828.1
Glutamicibacter halophytocola
HAE87196.1
Fidelibacterota bacterium
MBB5369498.1
Janthinobacterium sp. K2C7
WP_129188887.1
Agromyces protaetiae
MCA1850313.1
Acidobacteriota bacterium
RLI78393.1
Archaeoglobales archaeon
WP_162356545.1
Metabacillus mangrovi
HAH86642.1
Armatimonadota bacterium
MCC7099779.1
Rubrivivax sp.
MBR6248200.1
Muribaculaceae bacterium
MBI1270891.1
bacterium
MBP5500689.1
Bacteroidales bacterium
MCB9595151.1
Sandaracinaceae bacterium
MCD8038622.1
Lachnospiraceae bacterium
RLI96570.1
Candidatus Aenigmatarchaeota archaeon
ELZ94479.1
Haloferax mucosum ATCC BAA-1512
WP_051322559.1
Luteimonas sp. J16
WP_206235182.1
Xanthomonas sp.
MBR6277482.1
Bacteroidales bacterium
HDD46048.1
Candidatus Aenigmatarchaeota archaeon
MBR2927729.1
Oscillospiraceae bacterium
HFS82784.1
Campylobacterota bacterium
WP_152041926.1
Salinigranum salinum
HGY78736.1
HAJ15449.1
Erysipelotrichaceae bacterium
WP_206104769.1
MBP6506578.1
Opitutaceae bacterium
WP_170070524.1
Planifilum fimeticola
HFB64444.1
Aeromonadales bacterium
MBQ1376734.1
Lachnospiraceae bacterium
HHH43187.1
Gammaproteobacteria bacterium
MBI5326040.1
Ignavibacteriota bacterium
MBQ5349207.1
Phascolarctobacterium sp.
TEU18762.1
Anaerolineales bacterium
NUN45572.1
bacterium
WP_214772336.1
Bacillus sp. ISL-101
MBQ5641012.1
Ruminococcus sp.
WP_076766250.1
Domibacillus epiphyticus
MBZ0111438.1
Thermoanaerobaculia bacterium
MCC6094818.1
Eubacterium sp.

MCA9205647.1	*Planctomycetales bacterium*	VanZ-2
RKL63341.1	*Thermoanaerobacteraceae bacterium SP2*	VanZ-2
WP_185233078.1	*Teredinibacter franksiae*	VanZ-2
MBR0231054.1	*Clostridia bacterium*	VanZ-2
MBQ8928697.1	*Oscillospiraceae bacterium*	VanZ-2
WP_141191160.1	*Terrilactibacillus laevilacticus*	VanZ-2
WP_027368968.1	*Desulfocurvibacter africanus subsp. africanus DSM 2603*	VanZ-2
MBE6297154.1	*Bacteroidales bacterium*	VanZ-2
MBE6293367.1	*Bacteroidales bacterium*	VanZ-2
WP_196989326.1	*Panacibacter microcysteis*	VanZ-2
MBP8972280.1	*Anaerolineae bacterium*	VanZ-2
MBQ8218028.1	*Oscillospiraceae bacterium*	VanZ-2
OGE80691.1	*Candidatus Doudnabacteria bacterium RIFCSPHIGHO2_01_FULL_43_23*	VanZ-2
WP_101748754.1	*Endozoicomonas acroporae*	VanZ-2
WP_066015790.1	*Endozoicomonas atrinae*	VanZ-2
KEI72721.1	*Endozoicomonas elysicola*	VanZ-2
MBQ6947476.1	*Clostridia bacterium*	VanZ-2
WP_119547920.1	*Bacillus salacetis*	VanZ-2
AMO55447.1	*Endozoicomonas montiporae CL-33*	VanZ-2
WP_025488841.1	*Eisenbergiella massiliensis*	VanZ-2
MBK5225667.1	*Thermoleophilia bacterium*	VanZ-2
TAJ98659.1	*Candidatus Manganitrophaceae bacterium*	VanZ-2
MBN2003488.1	*Anaerolineae bacterium*	VanZ-2
MBE0429886.1	*Thermoleophilia bacterium*	VanZ-2
HAP79824.1	*Ruminococcus sp.*	VanZ-2
WP_026698842.1	*Alkalicoccus chagannorensis*	VanZ-2
RLC46896.1	*Candidatus Cloacimonadota bacterium*	VanZ-2
HIW15614.1	*Bacillota bacterium*	VanZ-2
WP_170181310.1	*Chlorobaculum thiosulfatiphilum*	VanZ-2
NLI69240.1	*Bacillota bacterium*	VanZ-2
HAJ73828.1	*Lachnospiraceae bacterium*	VanZ-2
WP_224846048.1		VanZ-2
MBQ9268578.1	*Oscillospiraceae bacterium*	VanZ-2
MBL4699706.1	*Phycisphaeraceae bacterium*	VanZ-2
MBM4761511.1	*Bacillus sp. B15-48*	VanZ-2
HGC58691.1		VanZ-2
MBQ7801472.1	*Oscillospiraceae bacterium*	VanZ-2
MBI3605029.1	*Nitrospirota bacterium*	VanZ-2
NBX75604.1	*Pseudomonadota bacterium*	VanZ-2
MCC7080474.1	*Burkholderiales bacterium*	VanZ-2
WP_155669057.1	*Ornithinibacillus caprae*	VanZ-2
CDE07658.1	*Prevotella sp. CAG:485*	VanZ-2
HCK53952.1	*Planctomycetaceae bacterium*	VanZ-2
MBN1221058.1	*Anaerolineae bacterium*	VanZ-2
WP_099092331.1	*Polynucleobacter wuianus*	VanZ-2
MBO7581441.1	*Bacteroidaceae bacterium*	VanZ-2
MBP3231414.1	*Anaerovibrio sp.*	VanZ-2
WP_204759015.1	*Limosilactobacillus reuteri*	VanZ-2
WP_205197750.1	*Aeromicrobium sp. YIM 150415*	VanZ-2
KRF20527.1	*Nocardioides sp. Soil797*	VanZ-2
OFX52369.1	*Bacteroidetes bacterium GWA2_30_7*	VanZ-2
MBU3943595.1	*Pseudomonadota bacterium*	VanZ-2
HJB61394.1	*Candidatus Ruminococcus gallistercoris*	VanZ-2
MBR6811570.1	*Candidatus Saccharibacteria bacterium*	VanZ-2
HDU69611.1		VanZ-2
HAD08177.1	*Porticoccaceae bacterium*	VanZ-2
MAG93903.1	*Planctomycetaceae bacterium*	VanZ-2
MBM3307557.1		VanZ-2
MBQ4549986.1	*Oscillospiraceae bacterium*	VanZ-2
MBR1801808.1	*Candidatus Saccharibacteria bacterium*	VanZ-2
MBR4933498.1	*Clostridia bacterium*	VanZ-2
MCD8281647.1	*Prevotella sp.*	VanZ-2
MBQ8051956.1	*Bacteroidaceae bacterium*	VanZ-2
WP_029626608.1		VanZ-2
MCA9755860.1	*Candidatus Eiseniibacteriota bacterium*	VanZ-2
RLB00368.1	*Deltaproteobacteria bacterium*	VanZ-2
MBO7304712.1	*Clostridia bacterium*	VanZ-2
WP_169799209.1	*Nocardioides jensenii JCM 1364 = NBRC 14755*	VanZ-2
RZJ84305.1	*Massilia sp.*	VanZ-2
NQW21049.1	*Chloroflexota bacterium*	VanZ-2
WP_051428284.1	*Bacillus sp. J33*	VanZ-2
PWC42156.1	*Azospirillum sp. TSO22-1*	VanZ-2
SCX20645.1	*Ruminococcaceae bacterium P7*	VanZ-2
RYF10479.1	*Oxalobacteraceae bacterium*	VanZ-2
TFG02177.1	*Promethearchaeota archaeon*	VanZ-2
WP_007640278.1	*Paraglaciecola psychrophila 170*	VanZ-2
MBQ7634904.1	*Bacteroidaceae bacterium*	VanZ-2
MBX9607941.1	*Gammaproteobacteria bacterium*	VanZ-2
NLC55728.1	*Armatimonadota bacterium*	VanZ-2
MCB1746675.1	*Gammaproteobacteria bacterium*	VanZ-2
ETI94822.1	*Intestinibacter bartlettii DORA_8_9*	VanZ-2
HHS71958.1		VanZ-2
MBP1649797.1	*Bacteroidota bacterium*	VanZ-2
MBW3670532.1	*Acidobacteriota bacterium*	VanZ-2
NMA37485.1	*Papillibacter sp.*	VanZ-2
MBP3567938.1	*Lachnospiraceae bacterium*	VanZ-2
WP_088007073.1	*Indiicoccus explosivorum*	VanZ-2
MBV8186214.1	*Alphaproteobacteria bacterium*	VanZ-2
MBM6593012.1	*Microvirga pudoricolor*	VanZ-2
MBD8654678.1	*Oxalobacteraceae sp. CFBP 13730*	VanZ-2
HIR97326.1	*Candidatus Merdisoma faecalis*	VanZ-2
MBQ8641500.1	*Clostridia bacterium*	VanZ-2
SIO34394.1	*Bradyrhizobium erythrophlei*	VanZ-2

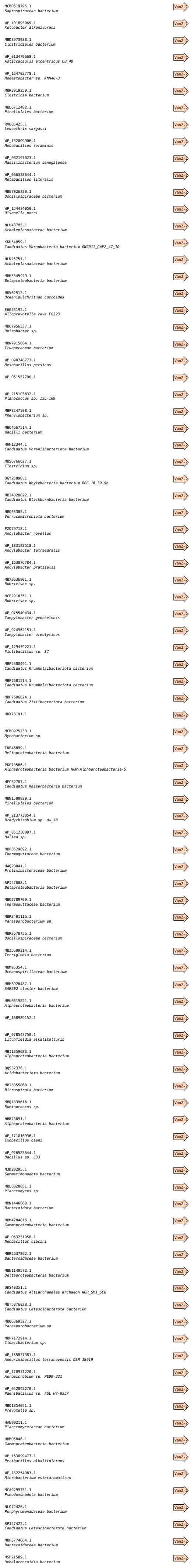

MCB0519795.1
Saprospiraceae bacterium
WP_101895969.1
Ketobacter alkanivorans
MBD8973988.1
Clostridiales bacterium
WP_013479668.1
Asticcacaulis excentricus CB 48
WP_164702778.1
Modestobacter sp. KNN46-3
MBR3019259.1
Clostridia bacterium
MBL6712482.1
Pirellulales bacterium
RVU85425.1
Leucothrix sargassi
WP_132009906.1
Mesobacillus foraminis
WP_062197023.1
Massilibacterium senegalense
WP_066328644.1
Metabacillus litoralis
MBE7026220.1
Oscillospiraceae bacterium
WP_154434050.1
Olsenella porci
NLU43785.1
Acholeplasmataceae bacterium
KKU54859.1
Candidatus Moranbacteria bacterium GW2011_GWE2_47_10
NLD25757.1
Acholeplasmataceae bacterium
MBM3345929.1
Betaproteobacteria bacterium
NDV62512.1
Oceanipulchritudo coccoides
EHG23192.1
Alloprevotella rava F0323
MBC7956337.1
Rhizobacter sp.
MBW7915684.1
Trueperaceae bacterium
WP_090748773.1
Mesobacillus persicus
WP_051557700.1
WP_215103632.1
Planococcus sp. ISL-109
MBP8247508.1
Phenylobacterium sp.
MBO4667514.1
Bacilli bacterium
HHH12344.1
Candidatus Moraniibacteriota bacterium
MBS6766627.1
Clostridium sp.
OGY25098.1
Candidatus Woykebacteria bacterium RBG_16_39_9b
MBI4028822.1
Candidatus Blackburnbacteria bacterium
NBQ65385.1
Verrucomicrobiota bacterium
PZQ79718.1
Ancylobacter novellus
WP_183188518.1
Ancylobacter tetraedralis
WP_163076784.1
Ancylobacter pratisalsi
MBX3636901.1
Rubrivivax sp.
MCE2916351.1
Rubrivivax sp.
WP_075540434.1
Campylobacter geochelonis
WP_024962151.1
Campylobacter ureolyticus
WP_129479221.1
Fictibacillus sp. S7
MBP2680491.1
Candidatus Krumholzibacteriota bacterium
MBP2681514.1
Candidatus Krumholzibacteriota bacterium
MBP7696824.1
Candidatus Zixiibacteriota bacterium
HDX73191.1
MCB0925233.1
Mycobacterium sp.
TNE46899.1
Deltaproteobacteria bacterium
PKP70566.1
Alphaproteobacteria bacterium HGW-Alphaproteobacteria-5
HEC32787.1
Candidatus Kaiserbacteria bacterium
MBN1590929.1
Pirellulales bacterium
WP_213773854.1
Bradyrhizobium sp. dw_78
WP_051230097.1
Haliea sp.
MBP3529692.1
Thermoguttaceae bacterium
HAQ20841.1
Prolixibacteraceae bacterium
RPI47608.1
Betaproteobacteria bacterium
MBQ2789709.1
Thermoguttaceae bacterium
MBR3401116.1
Parasporobacterium sp.
MBR3678756.1
Oscillospiraceae bacterium
MBZ5699214.1
Terriglobia bacterium
MBM95354.1
Oceanospirillaceae bacterium
MBM3926487.1
SAR202 cluster bacterium
MBU6319821.1
Alphaproteobacteria bacterium
WP_168989152.1
WP_078543750.1
Litchfieldia alkalitelluris
MBI1359683.1
Alphaproteobacteria bacterium
QQS32376.1
Acidobacteriota bacterium
MBI5855868.1
Nitrospirota bacterium
MBQ1839616.1
Ruminococcus sp.
NBR78891.1
Alphaproteobacteria bacterium
WP_171016936.1
Exobacillus caeni
WP_026583644.1
Bacillus sp. J33
NJD20295.1
Gemmatimonadota bacterium
MBL8820051.1
Planctomyces sp.
MBN1446868.1
Bacteroidota bacterium
MBM4204816.1
Gammaproteobacteria bacterium
WP_063251958.1
Neobacillus niacini
MBR2637862.1
Bacteroidaceae bacterium
MBN1140572.1
Deltaproteobacteria bacterium
ODS40351.1
Candidatus Altiarchaeales archaeon WOR_SM1_SCG
MBT5876828.1
Candidatus Latescibacterota bacterium
MBQ6369327.1
Parasporobacterium sp.
MBP7172914.1
Cloacibacterium sp.
WP_155837381.1
Aneurinibacillus terranovensis DSM 18919
WP_170931220.1
Aeromicrobium sp. PE09-221
WP_052092270.1
Paenibacillus sp. FSL H7-0357
MBQ1854051.1
Prevotella sp.
HAN99211.1
Planctomycetaceae bacterium
HHM05846.1
Gammaproteobacteria bacterium
WP_163099473.1
Peribacillus alkalitolerans
WP_182254063.1
Microbacterium esteraromaticum
MCA0299751.1
Pseudomonadota bacterium
NLO72426.1
Porphyromonadaceae bacterium
RPJ47422.1
Candidatus Latescibacterota bacterium
MBP3774664.1
Bacteroidaceae bacterium
MSP21589.1
Dehalococcoidia bacterium

MBR3161110.1	*Bacilli bacterium*	VanZ-2
CAA6827490.1	*uncultured Aureispira sp.*	VanZ-2
EKD31336.1	*groundwater metagenome*	VanZ-2
MBN1566155.1	*Anaerolineae bacterium*	VanZ-2
MBQ7751363.1	*Clostridia bacterium*	VanZ-2
MBI9098428.1	*Spirochaetaceae bacterium*	VanZ-2
RLD50386.1	*Bacteroidota bacterium*	VanZ-2
HCQ33216.1	*Verrucomicrobiales bacterium*	VanZ-2
NLX35585.1	*Chloroflexota bacterium*	VanZ-2
WP_008787540.1	*Coprobacillus cateniformis JCM 10604*	VanZ-2
OHC42894.1	*Rhodanobacter sp. RIFOXYA1_FULL_67_6*	VanZ-2
MBS7125469.1	*Coprobacillus sp.*	VanZ-2
MBT8490246.1	*Deltaproteobacteria bacterium*	VanZ-2
WP_066141318.1	*Haladaptatus sp. R4*	VanZ-2
MCB9415353.1	*Actinomycetota bacterium*	VanZ-2
WP_162802952.1	*Ornithinimicrobium avium*	VanZ-2
WP_231701343.1	*Clostridium botulinum*	VanZ-2
MBI4395970.1	*Elusimicrobiota bacterium*	VanZ-2
HEI73726.1		VanZ-2
NOQ96982.1	*Calditrichia bacterium*	VanZ-2
MBD5198641.1	*Bacteroidales bacterium*	VanZ-2
MBD5234720.1	*Bacteroidales bacterium*	VanZ-2
MBS4974374.1	*Clostridium celatum*	VanZ-2
NQY50005.1	*Colwellia sp.*	VanZ-2
NQZ26903.1	*Colwellia sp.*	VanZ-2
MBR4460027.1	*Paludibacteraceae bacterium*	VanZ-2
MBC8485517.1	*Bacteroidota bacterium*	VanZ-2
MCA9062632.1	*Planctomycetaceae bacterium*	VanZ-2
WP_182769991.1	*Shewanella sp. SG41-4*	VanZ-2
WP_076416183.1	*Shewanella sp. UCD-KL12*	VanZ-2
NKQ41419.1	*Sulfurovum sp.*	VanZ-2
KRG85279.1	*Stenotrophomonas acidaminiphila*	VanZ-2
MBM4432032.1	*Chloroflexota bacterium*	VanZ-2
HEC66892.1	*bacterium*	VanZ-2
WP_075184956.1		VanZ-2
MBD3400835.1	*Candidatus Coatesbacteria bacterium*	VanZ-2
MBW1998095.1	*Deltaproteobacteria bacterium*	VanZ-2
WP_232402746.1	*Aeromicrobium wangtongii*	VanZ-2
NBS31945.1	*Planctomycetia bacterium*	VanZ-2
MBP7267776.1	*Eubacterium sp.*	VanZ-2
MBM3125251.1	*Chloroflexota bacterium*	VanZ-2
MBQ9351795.1	*Phyllobacterium sp.*	VanZ-2
PWB39132.1	*Parcubacteria group bacterium*	VanZ-2
WP_223262285.1	*Variovorax beijingensis*	VanZ-2
NLC43830.1	*Clostridiales bacterium*	VanZ-2
WP_098529030.1	*Bacillus sp. AFS037270*	VanZ-2
MBI5646323.1	*Ignavibacteriota bacterium*	VanZ-2
WP_090832601.1		VanZ-2
WP_089764553.1	*Halobellus clavatus*	VanZ-2
NTV79234.1	*Clostridiales bacterium*	VanZ-2
MBU1801933.1	*Actinomycetota bacterium*	VanZ-2
MBV8544827.1	*Acidobacteriota bacterium*	VanZ-2
MBD5469833.1	*Lachnospiraceae bacterium*	VanZ-2
WP_213531961.1	*Paenibacillus sp. J45TS6*	VanZ-2
MCC7341367.1	*Bryobacterales bacterium*	VanZ-2
MBP7141459.1	*Opitutaceae bacterium*	VanZ-2
WP_006830405.1	*Planococcus antarcticus DSM 14505*	VanZ-2
WP_099263875.1	*Rhodopirellula bahusiensis*	VanZ-2
HHY82008.1	*Clostridiales bacterium*	VanZ-2
HBA93417.1	*Oscillospiraceae bacterium*	VanZ-2
MBS3736413.1	*Candidatus Bipolaricaulota bacterium*	VanZ-2
WP_033827525.1	*Bacillus andreraoultii*	VanZ-2
WP_033827617.1	*Bacillus andreraoultii*	VanZ-2
MBO4501512.1	*Clostridia bacterium*	VanZ-2
KAB2647871.1	*Verrucomicrobiota bacterium*	VanZ-2
NLJ54089.1	*Intrasporangiaceae bacterium*	VanZ-2
RXE72558.1	*Muribaculaceae bacterium Isolate-013 (NCI)*	VanZ-2
PON12416.1	*Candidatus Entotheonella serta*	VanZ-2
MBM3222336.1	*Candidatus Tectimicrobiota bacterium*	VanZ-2
MBR0302775.1	*Clostridia bacterium*	VanZ-2
MCA9473120.1	*Nitrospira sp.*	VanZ-2
HAE44142.1	*Lachnospiraceae bacterium*	VanZ-2
MBI3242833.1	*Chloroflexota bacterium*	VanZ-2
WP_213528216.1	*Paenibacillus cisolokensis*	VanZ-2
OGV57444.1	*Lentisphaerae bacterium RIFOXYA12_FULL_48_11*	VanZ-2
RMG23899.1	*Armatimonadota bacterium*	VanZ-2
NMC82206.1	*Armatimonadota bacterium*	VanZ-2
MBP3701911.1	*Lachnospiraceae bacterium*	VanZ-2
MCC7119664.1	*Anaerolineales bacterium*	VanZ-2
WP_162317782.1	*Haloarcula limicola*	VanZ-2
MBI2867995.1	*Chloroflexota bacterium*	VanZ-2
WP_051404915.1	*Peribacillus huizhouensis*	VanZ-2
WP_214476337.1	*Cytobacillus gottheilii*	VanZ-2
PKK86915.1	*Tenericutes bacterium HGW-Tenericutes-8*	VanZ-2
THG45303.1	*Muribaculum caecicola*	VanZ-2
PWU11289.1	*Verrucomicrobiota bacterium*	VanZ-2
QNI90137.1	*Synechococcus sp. ROS8604*	VanZ-2
HAV14358.1	*Opitutia bacterium*	VanZ-2
MBN1670730.1	*Kiritimatiellia bacterium*	VanZ-2
PJA81226.1	*Nitrospirae bacterium CG_4_9_14_3_um_filter_51_5*	VanZ-2
TFG63862.1	*Nitrospirales bacterium*	VanZ-2
WP_169098689.1		VanZ-2
MBP3399630.1	*Erysipelotrichaceae bacterium*	VanZ-2

OGR27406.1 *Desulfobacca sp. RBG_16_60_12* — VanZ-2

MBD3866491.1 *Candidatus Polarisedimenticola svalbardensis* — VanZ-2

MBD5284749.1 *Bacteroides sp.* — VanZ-2

MBU0936493.1 *Spirochaetota bacterium* — VanZ-2

MBU0928095.1 *Spirochaetota bacterium* — VanZ-2

WP_065853335.1 — VanZ-2

MBD3371507.1 *Candidatus Coatesbacteria bacterium* — VanZ-2

MBD5321989.1 *Bacteroides sp.* — VanZ-2

AAS96891.1 *Nitratidesulfovibrio vulgaris str. Hildenborough* — VanZ-2

WP_155306943.1 *Desulfosarcina widdelii* — VanZ-2

MCC7474090.1 *Pirellulales bacterium* — VanZ-2

MBP1755820.1 *Bacillota bacterium* — VanZ-2

MBL0187172.1 *Candidatus Obscuribacter sp.* — VanZ-2

SDW33247.1 *Paenibacillus sp. PDC88* — VanZ-2

MBM4053924.1 *Planctomycetota bacterium* — VanZ-2

NIK23017.1 *Paenibacillus lupini* — VanZ-2

UCE08110.1 *bacterium* — VanZ-2

RUM37025.1 *Desulfobulbus sp.* — VanZ-2

MBE4907751.1 — VanZ-2

MYB75863.1 *Chloroflexota bacterium* — VanZ-2

MBC7841566.1 *Gemmatimonadaceae bacterium* — VanZ-2

MBE1353457.1 — VanZ-2

MBT9524031.1 *Rhizobacter sp.* — VanZ-2

MBT4224879.1 *Opitutia bacterium* — VanZ-2

TAK63425.1 *Bacteroidota bacterium* — VanZ-2

MBZ0256218.1 *bacterium* — VanZ-2

MBF2036996.1 *Leptolyngbyaceae cyanobacterium T60_A2020_046* — VanZ-2

NPA85606.1 *Thermoproteota archaeon* — VanZ-2

WP_105013014.1 *Salinibacter sp. 10B* — VanZ-2

MBQ4381423.1 *Oscillospiraceae bacterium* — VanZ-2

MBI3482713.1 *Bacteroidota bacterium* — VanZ-2

MBZ0253877.1 *Deltaproteobacteria bacterium* — VanZ-2

RPI04419.1 *Zetaproteobacteria bacterium* — VanZ-2

MBO7145222.1 *Salinivirgaceae bacterium* — VanZ-2

MBX3618653.1 *Rhizobacter sp.* — VanZ-2

MBX3119439.1 *Fimbriimonadaceae bacterium* — VanZ-2

MBX7142257.1 *Chitinophagales bacterium* — VanZ-2

MBP9891343.1 *Planctomycetota bacterium* — VanZ-2

MBN1810172.1 *Planctomycetota bacterium* — VanZ-2

MBN1441790.1 *Planctomycetota bacterium* — VanZ-2

AUN37641.1 — VanZ-2

WP_026574152.1 *Bacillus sp. UNC438CL73TsuS30* — VanZ-2

MBO4251935.1 *Clostridia bacterium* — VanZ-2

VFB17928.1 *Pseudomonas fragi* — VanZ-2

MCA8944494.1 *Planctomycetota bacterium* — VanZ-2

MBI2193836.1 *Planctomycetota bacterium* — VanZ-2

MBD3414162.1 *Candidatus Aminicenantes bacterium* — VanZ-2

WP_197046687.1 *Oceanobacillus salinisoli* — VanZ-2

MBO4999583.1 *Bacteroidaceae bacterium* — VanZ-2

MBK7722868.1 *Austwickia sp.* — VanZ-2

KPV95848.1 *Pseudoalteromonas sp. P1-9* — VanZ-2

RQV96739.1 *bacterium* — VanZ-2

TNF49488.1 *bacterium* — VanZ-2

MBS3812245.1 *Candidatus Bipolaricaulota bacterium* — VanZ-2

MBC7587178.1 *Chitinophagaceae bacterium* — VanZ-2

WP_161694198.1 *Paenibacillus sacheonensis* — VanZ-2

MBR5455477.1 *Bacteroidaceae bacterium* — VanZ-2

MBO4827717.1 *Prevotella sp.* — VanZ-2

MBQ6807921.1 *Bacillota bacterium* — VanZ-2

HHX43029.1 *Chloroflexota bacterium* — VanZ-2

WP_168926907.1 *Shewanella donghaensis* — VanZ-2

MBO6032682.1 *Prevotella sp.* — VanZ-2

MBS6207340.1 *Bacillota bacterium* — VanZ-2

GAL05235.1 *Photobacterium aphoticum* — VanZ-2

WP_203926299.1 *Virgisporangium ochraceum* — VanZ-2

WP_204012354.1 *Virgisporangium aurantiacum* — VanZ-2

MCA8988444.1 *Planctomycetaceae bacterium* — VanZ-2

MBX3083222.1 *Anaerolineae bacterium* — VanZ-2

MBR1837936.1 *Bacteroidaceae bacterium* — VanZ-2

NKB81724.1 *Nitrospirales bacterium* — VanZ-2

MBQ4625902.1 *Clostridia bacterium* — VanZ-2

MBA34061.1 — VanZ-2

NMA28222.1 *Burkholderiales bacterium* — VanZ-2

WP_089745551.1 *Granulicatella balaenopterae* — VanZ-2

SFG97476.1 *Neptunomonas qingdaonensis* — VanZ-2

MBM3317521.1 *Candidatus Eiseniibacteriota bacterium* — VanZ-2

WP_223592230.1 — VanZ-2

WP_145266580.1 *Tautonia plasticadhaerens* — VanZ-2

WP_213773879.1 *Bradyrhizobium sp. dw_78* — VanZ-2

CDE45666.1 *Clostridium sp. CAG:411* — VanZ-2

MBQ7175110.1 *Lachnospiraceae bacterium* — VanZ-2

WP_224136571.1 *Metabacillus rhizolycopersici* — VanZ-2

MBR4749972.1 *Abditibacteriota bacterium* — VanZ-2

OGF97735.1 *Candidatus Glassbacteria bacterium RBG_16_58_8* — VanZ-2

UCD66696.1 *Deltaproteobacteria bacterium* — VanZ-2

MCE2740381.1 *Sphingobacteriales bacterium* — VanZ-2

MBR5416270.1 *Thermoguttaceae bacterium* — VanZ-2

WP_182869309.1 *Bradyrhizobium diazoefficiens* — VanZ-2

QQS61104.1 *Candidatus Moraniibacteriota bacterium* — VanZ-2

WP_105332011.1 *Bremerella cremea* — VanZ-2

OUE08216.1 *Clavibacter michiganensis* — VanZ-2

MBQ9358590.1 *Abditibacteriota bacterium* — VanZ-2

MBQ8860143.1 *Ruminococcus sp.* — VanZ-2

Accession	Organism	Gene
MCC6001502.1	*Pararhodobacter sp.*	VanZ-2
MBS6453382.1	*Butyrivibrio sp.*	VanZ-2
MBR3567099.1	*Salinivirgaceae bacterium*	VanZ-2
MBO7476568.1	*Salinivirgaceae bacterium*	VanZ-2
MBQ3100065.1	*Clostridia bacterium*	VanZ-2
WP_101690402.1	*Dysgonomonas massiliensis*	VanZ-2
MPY66771.1	*Deinococcus terrestris*	VanZ-2
WP_205596755.1	*Halostella salina*	VanZ-2
WP_131924303.1	*Hazenella coriacea*	VanZ-2
MBP1542013.1	*Prevotella sp.*	VanZ-2
HIP94388.1	*Leucothrix sp.*	VanZ-2
KPK76548.1	*candidate division Zixibacteria bacterium SM23_73*	VanZ-2
MBU0729924.1		VanZ-2
MBM4398195.1	*Deltaproteobacteria bacterium*	VanZ-2
WP_204457062.1	*Barnesiella sp. CU968*	VanZ-2
MCB1552432.1	*Lysobacterales bacterium*	VanZ-2
MBD5257315.1	*Barnesiella sp.*	VanZ-2
MBD5479967.1	*Lachnospiraceae bacterium*	VanZ-2
MBP1735339.1	*Deltaproteobacteria bacterium*	VanZ-2
MBP3299208.1	*Muribaculaceae bacterium*	VanZ-2
WP_051620845.1	*Paenibacillus sp. UNC451MF*	VanZ-2
MBT3234689.1	*Bdellovibrionales bacterium*	VanZ-2
WP_205525337.1	*Novosphingobium sp. THN1*	VanZ-2
WP_195600596.1	*Longibaculum muris*	VanZ-2
MBX9679434.1	*Gemmataceae bacterium*	VanZ-2
MBO4813551.1	*Muribaculaceae bacterium*	VanZ-2
MBW2232911.1	*Deltaproteobacteria bacterium*	VanZ-2
MBK6910301.1	*bacterium*	VanZ-2
MBC7207110.1	*Methyloversatilis sp.*	VanZ-2
MBQ1722452.1	*Muribaculaceae bacterium*	VanZ-2
MBM3621355.1	*Alphaproteobacteria bacterium*	VanZ-2
MBN9600162.1	*Afipia sp.*	VanZ-2
GFI07205.1	*Muribaculaceae bacterium*	VanZ-2
HIW08672.1	*Candidatus Faecalibacterium intestinigallinarum*	VanZ-2
MBW2738767.1	*Deltaproteobacteria bacterium*	VanZ-2
RKY87766.1	*Candidatus Zhuqueibacterota bacterium*	VanZ-2
PYQ34119.1	*Acidobacteriota bacterium*	VanZ-2
MBN2013392.1	*Candidatus Zhuqueibacterota bacterium*	VanZ-2
MCB1069888.1	*Kiritimatiellia bacterium*	VanZ-2
PKN39529.1	*Deltaproteobacteria bacterium HGW-Deltaproteobacteria-20*	VanZ-2
PIE08047.1	*Rhodobacterales bacterium*	VanZ-2
MQA67389.1	*Alphaproteobacteria bacterium*	VanZ-2
MCB1436336.1	*Rhodobiaceae bacterium*	VanZ-2
PYQ52877.1	*Acidobacteriota bacterium*	VanZ-2
NLH92000.1	*Atopobium sp.*	VanZ-2
OQA85752.1	*bacterium ADurb.Bin236*	VanZ-2
MBS6445291.1	*Ruminococcus sp.*	VanZ-2
MBI5468406.1	*Deltaproteobacteria bacterium*	VanZ-2
NLC95392.1	*Bacilli bacterium*	VanZ-2
PYU25533.1	*Acidobacteriota bacterium*	VanZ-2
MBR4966001.1	*Lachnospiraceae bacterium*	VanZ-2
MBP3398169.1	*Erysipelotrichaceae bacterium*	VanZ-2
MBI4549395.1	*Candidatus Omnitrophota bacterium*	VanZ-2
WP_141245192.1	*Mesorhizobium sp. WSM3860*	VanZ-2
KAB2660610.1	*Verrucomicrobiota bacterium*	VanZ-2
MBQ9982509.1	*Oscillospiraceae bacterium*	VanZ-2
MBP3412072.1	*Oscillospiraceae bacterium*	VanZ-2
WP_163300918.1	*Desulfolutivibrio sulfodismutans DSM 3696*	VanZ-2
MBE0503140.1	*Desulfuromonadales bacterium*	VanZ-2
PLX78723.1	*Desulfuromonas sp.*	VanZ-2
MBE6985685.1	*Oscillospiraceae bacterium*	VanZ-2
CUH41818.1	*Ruegeria atlantica*	VanZ-2
MBU3906811.1	*Nanoarchaeota archaeon*	VanZ-2
MSQ15886.1	*Dehalococcoidia bacterium*	VanZ-2
MCB1277490.1	*Prosthecobacter sp.*	VanZ-2
NNL07558.1	*Gammaproteobacteria bacterium*	VanZ-2
WP_052302377.1	*Bacillus sp. SG-1*	VanZ-2
PRZ01466.1	*Marinilabilia salmonicolor*	VanZ-2
MBO5999006.1	*Lachnospiraceae bacterium*	VanZ-2
MBD0291008.1	*Thermoleophilia bacterium*	VanZ-2
NKB64615.1	*Gammaproteobacteria bacterium*	VanZ-2
PYR66874.1	*Acidobacteriota bacterium*	VanZ-2
GJL49384.1	*Nitrospirales bacterium*	VanZ-2
RPI69598.1	*Geobacteraceae bacterium*	VanZ-2
KPL13655.1	*Bacteroides sp. SM23_62*	VanZ-2
WP_152587517.1	*Microvirga thermotolerans*	VanZ-2
WP_115360022.1	*Sporosarcina pasteurii*	VanZ-2
MBR6459921.1	*Actinomycetaceae bacterium*	VanZ-2
MBM3792142.1	*Acidobacteriota bacterium*	VanZ-2
MBS0331918.1	*Pseudomonadota bacterium*	VanZ-2
TFH87631.1	*Billgrantia azerbaijanica*	VanZ-2
MBI1320727.1	*Candidatus Hydrogenedens sp.*	VanZ-2
MBS3957058.1	*Clostridiales bacterium*	VanZ-2
WP_009332182.1		VanZ-2
MBW4095832.1	*Acidobacteriota bacterium*	VanZ-2
MBN2720556.1	*Pseudomonadota bacterium*	VanZ-2
MBI2767077.1	*Chloroflexota bacterium*	VanZ-2
MCB9308671.1	*Lewinellaceae bacterium*	VanZ-2
OFV68228.1	*Candidatus Syntrophoarchaeum caldarius*	VanZ-2
HAW08573.1	*Bacteroidota bacterium*	VanZ-2
MCB1049794.1	*Acidobacteriota bacterium*	VanZ-2
MBX3128829.1	*Polyangiaceae bacterium*	VanZ-2
OAI45724.1	*Nitrospira sp. SCGC AG-212-E16*	VanZ-2

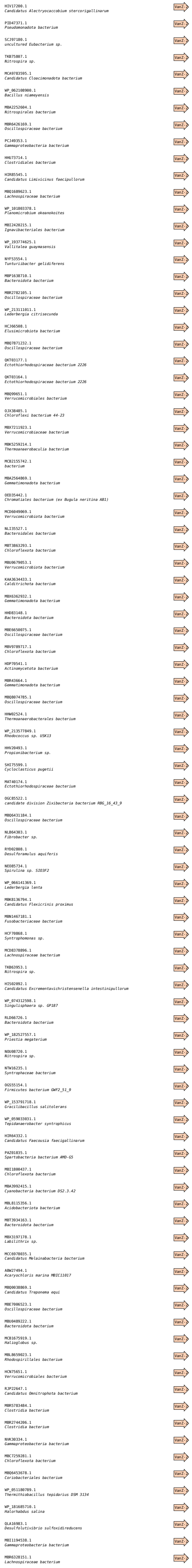
HIV17200.1
Candidatus Alectryocaccobium stercorigallinarum
VanZ-2
PID47371.1
Pseudomonadota bacterium
VanZ-2
SCJ97180.1
uncultured Eubacterium sp.
VanZ-2
TKB75807.1
Nitrospira sp.
VanZ-2
MCA9783595.1
Candidatus Cloacimonadota bacterium
VanZ-2
WP_062108900.1
Bacillus niameyensis
VanZ-2
MBA2252604.1
Nitrospirales bacterium
VanZ-2
MBR6426169.1
Oscillospiraceae bacterium
VanZ-2
PCJ49353.1
Gammaproteobacteria bacterium
VanZ-2
HHU73714.1
Clostridiales bacterium
VanZ-2
HIR85545.1
Candidatus Limivicinus faecipullorum
VanZ-2
MBQ1689623.1
Lachnospiraceae bacterium
VanZ-2
WP_101803378.1
Planomicrobium okeanokoites
VanZ-2
MBI2420215.1
Ignavibacteriales bacterium
VanZ-2
WP_193774625.1
Vallitalea guaymasensis
VanZ-2
NYF53554.1
Tunturiibacter gelidiferens
VanZ-2
MBP1638710.1
Bacteroidota bacterium
VanZ-2
MBR2782105.1
Oscillospiraceae bacterium
VanZ-2
WP_213111011.1
Lederbergia citrisecunda
VanZ-2
HCJ66508.1
Elusimicrobiota bacterium
VanZ-2
MBQ7871232.1
Oscillospiraceae bacterium
VanZ-2
QKT03177.1
Ectothiorhodospiraceae bacterium 2226
VanZ-2
QKT03164.1
Ectothiorhodospiraceae bacterium 2226
VanZ-2
MBQ99651.1
Verrucomicrobiales bacterium
VanZ-2
OJX38485.1
Chloroflexi bacterium 44-23
VanZ-2
MBX7211923.1
Verrucomicrobiaceae bacterium
VanZ-2
MBK5259214.1
Thermoanaerobaculia bacterium
VanZ-2
MCB2155742.1
bacterium
VanZ-2
MBA2564869.1
Gemmatimonadota bacterium
VanZ-2
OED35442.1
Chromatiales bacterium (ex Bugula neritina AB1)
VanZ-2
MCD6049069.1
Verrucomicrobiota bacterium
VanZ-2
NLI35527.1
Bacteroidales bacterium
VanZ-2
MBT3863293.1
Chloroflexota bacterium
VanZ-2
MBU0679053.1
Verrucomicrobiota bacterium
VanZ-2
KAA3634433.1
Calditrichota bacterium
VanZ-2
MBX6362932.1
Gemmatimonadota bacterium
VanZ-2
HHD83148.1
Bacteroidota bacterium
VanZ-2
MBE6650075.1
Oscillospiraceae bacterium
VanZ-2
MBV9789717.1
Chloroflexota bacterium
VanZ-2
HDP70541.1
Actinomycetota bacterium
VanZ-2
MBR43664.1
Gemmatimonadota bacterium
VanZ-2
MBQ8074785.1
Oscillospiraceae bacterium
VanZ-2
HHW02524.1
Thermoanaerobacterales bacterium
VanZ-2
WP_213577849.1
Rhodococcus sp. USK13
VanZ-2
HHV20493.1
Propionibacterium sp.
VanZ-2
SHI75599.1
Cycloclasticus pugetii
VanZ-2
MAT40174.1
Ectothiorhodospiraceae bacterium
VanZ-2
OGC85522.1
candidate division Zixibacteria bacterium RBG_16_43_9
VanZ-2
MBQ6431184.1
Oscillospiraceae bacterium
VanZ-2
NLB64303.1
Fibrobacter sp.
VanZ-2
RYD02808.1
Desulforamulus aquiferis
VanZ-2
NEO85734.1
Spirulina sp. SIO3F2
VanZ-2
WP_066141369.1
Lederbergia lenta
VanZ-2
MBK8136794.1
Candidatus Flexicrinis proximus
VanZ-2
MBN1467181.1
Fusobacteriaceae bacterium
VanZ-2
HCF70868.1
Syntrophomonas sp.
VanZ-2
MCD8378896.1
Lachnospiraceae bacterium
VanZ-2
TKB63953.1
Nitrospira sp.
VanZ-2
HIS02092.1
Candidatus Excrementavichristensenella intestinipullorum
VanZ-2
WP_074312598.1
Singulisphaera sp. GP187
VanZ-2
RLD66726.1
Bacteroidota bacterium
VanZ-2
WP_182527557.1
Priestia megaterium
VanZ-2
NOU08720.1
Nitrospira sp.
VanZ-2
NTW16235.1
Syntrophaceae bacterium
VanZ-2
OGS55154.1
Firmicutes bacterium GWF2_51_9
VanZ-2
WP_153791718.1
Gracilibacillus salitolerans
VanZ-2
WP_059033031.1
Tepidanaerobacter syntrophicus
VanZ-2
HIR64332.1
Candidatus Faecousia faecigallinarum
VanZ-2
PAZ01835.1
Spartobacteria bacterium AMD-G5
VanZ-2
MBI1800437.1
Chloroflexota bacterium
VanZ-2
MBA3992415.1
Cyanobacteria bacterium DS2.3.42
VanZ-2
MBL8115356.1
Acidobacteriota bacterium
VanZ-2
MBT3934163.1
Bacteroidota bacterium
VanZ-2
MBX3197178.1
Labilithrix sp.
VanZ-2
MCC6978035.1
Candidatus Melainabacteria bacterium
VanZ-2
ABW27494.1
Acaryochloris marina MBIC11017
VanZ-2
MBQ0038869.1
Candidatus Treponema equi
VanZ-2
MBE7006523.1
Oscillospiraceae bacterium
VanZ-2
MBU0489222.1
Bacteroidota bacterium
VanZ-2
MCB1675919.1
Halioglobus sp.
VanZ-2
MBL8659023.1
Rhodospirillales bacterium
VanZ-2
HCN75651.1
Verrucomicrobiales bacterium
VanZ-2
RJP22647.1
Candidatus Omnitrophota bacterium
VanZ-2
MBR5783484.1
Clostridia bacterium
VanZ-2
MBR2744206.1
Clostridia bacterium
VanZ-2
NVK30334.1
Gammaproteobacteria bacterium
VanZ-2
MBC7259281.1
Chloroflexota bacterium
VanZ-2
MBQ6453678.1
Coriobacteriales bacterium
VanZ-2
WP_051180789.1
Thermithiobacillus tepidarius DSM 3134
VanZ-2
WP_181685710.1
Halorhabdus salina
VanZ-2
QLA16983.1
Desulfolutivibrio sulfoxidireducens
VanZ-2
MBI1194538.1
Gammaproteobacteria bacterium
VanZ-2
MBR6328151.1
Lachnospiraceae bacterium
VanZ-2

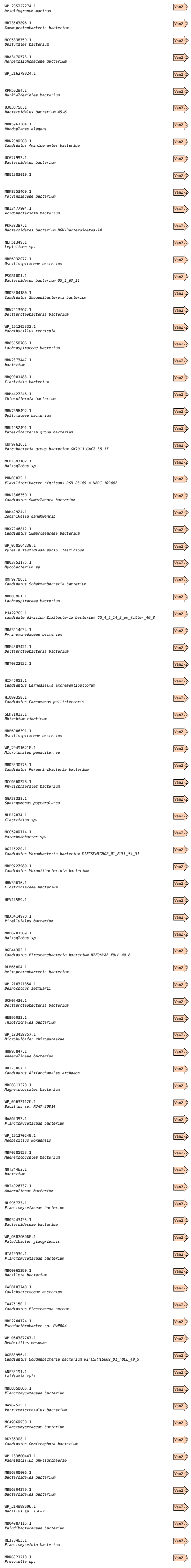

WP_205222274.1
Desulfogranum marinum
MBT3563896.1
Gammaproteobacteria bacterium
MCC5838759.1
Opitutales bacterium
MBA3470573.1
Herpetosiphonaceae bacterium
WP_216278924.1
RPH59294.1
Burkholderiales bacterium
OJU38758.1
Bacteroidales bacterium 45-6
MBK5961304.1
Rhodoplanes elegans
MBN2399568.1
Candidatus Aminicenantes bacterium
UCG27992.1
Bacteroidales bacterium
MBE1383010.1
MBK8253460.1
Polyangiaceae bacterium
MBI3477804.1
Acidobacteriota bacterium
PKP38387.1
Bacteroidetes bacterium HGW-Bacteroidetes-14
NLF51349.1
Leptolinea sp.
MBE6932077.1
Oscillospiraceae bacterium
PSQ81001.1
Bacteroidetes bacterium QS_1_63_11
MBD3384180.1
Candidatus Zhuqueibacterota bacterium
MBW2513967.1
Deltaproteobacteria bacterium
WP_191202332.1
Paenibacillus terricola
MBO5550706.1
Lachnospiraceae bacterium
MBN2373447.1
bacterium
MBQ9081483.1
Clostridia bacterium
MBM4427246.1
Chloroflexota bacterium
MBW7896492.1
Opitutaceae bacterium
MBU1952491.1
Patescibacteria group bacterium
KKP97610.1
Parcubacteria group bacterium GW2011_GWC2_36_17
MCB1697102.1
Halioglobus sp.
PHN05825.1
Flavilitoribacter nigricans DSM 23189 = NBRC 102662
MBN1866350.1
Candidatus Sumerlaeota bacterium
RDH42924.1
Zooshikella ganghwensis
MBX7246812.1
Candidatus Sumerlaeaceae bacterium
WP_058564230.1
Xylella fastidiosa subsp. fastidiosa
MBU3751175.1
Mycobacterium sp.
RMF92708.1
Candidatus Schekmanbacteria bacterium
NBH83961.1
Lachnospiraceae bacterium
PJA29765.1
candidate division Zixibacteria bacterium CG_4_9_14_3_um_filter_46_8
MBA3514634.1
Pyrinomonadaceae bacterium
MBM4303421.1
Deltaproteobacteria bacterium
MBT0822932.1
HIX46052.1
Candidatus Barnesiella excrementipullorum
HIU90359.1
Candidatus Caccomonas pullistercoris
SEH71032.1
Rhizobium tibeticum
MBE6986391.1
Oscillospiraceae bacterium
WP_204916218.1
Microlunatus panaciterrae
MBD3330775.1
Candidatus Peregrinibacteria bacterium
MCC6360228.1
Phycisphaerales bacterium
GGA38338.1
Sphingomonas psychrolutea
NLB19874.1
Clostridium sp.
MCC5989714.1
Pararhodobacter sp.
OGI15220.1
Candidatus Moranbacteria bacterium RIFCSPHIGHO2_01_FULL_54_31
MBP9727980.1
Candidatus Moraniibacteriota bacterium
HHW30616.1
Clostridiaceae bacterium
HFV14589.1
MBX3414970.1
Pirellulales bacterium
MBP6701569.1
Halioglobus sp.
OGF44393.1
Candidatus Firestonebacteria bacterium RIFOXYA2_FULL_40_8
RLB65004.1
Deltaproteobacteria bacterium
WP_216321854.1
Deinococcus aestuarii
UCH07430.1
Deltaproteobacteria bacterium
HEB99032.1
Thiotrichales bacterium
WP_183458357.1
Microbulbifer rhizosphaerae
HHN93847.1
Anaerolineae bacterium
HDI73067.1
Candidatus Altiarchaeales archaeon
MBF0611328.1
Magnetococcales bacterium
WP_066321126.1
Bacillus sp. FJAT-29814
HAA62392.1
Planctomycetaceae bacterium
WP_191270240.1
Neobacillus kokaensis
MBF0285923.1
Magnetococcales bacterium
NQT34462.1
bacterium
MBI4926737.1
Anaerolineae bacterium
NLS95773.1
Planctomycetaceae bacterium
MBQ3243435.1
Bacteroidaceae bacterium
WP_068706068.1
Paludibacter jiangxiensis
HIA19536.1
Planctomycetaceae bacterium
MBQ0065290.1
Bacillota bacterium
KAF0183748.1
Caulobacteraceae bacterium
TAA75150.1
Candidatus Electronema aureum
MBP2264724.1
Pseudarthrobacter sp. PvP004
WP_066387767.1
Neobacillus mesonae
OGE83956.1
Candidatus Doudnabacteria bacterium RIFCSPHIGHO2_01_FULL_49_9
ANF33191.1
Leifsonia xyli
MBL8850665.1
Planctomycetaceae bacterium
HAV62525.1
Verrucomicrobiales bacterium
MCA9069938.1
Planctomycetaceae bacterium
RKY36308.1
Candidatus Omnitrophota bacterium
WP_183600447.1
Paenibacillus phyllosphaerae
MBE6300006.1
Bacteroidales bacterium
MBE6304279.1
Bacteroidales bacterium
WP_214998606.1
Bacillus sp. ISL-7
MBO4987115.1
Paludibacteraceae bacterium
REJ70463.1
Planctomycetota bacterium
MBR6321218.1
Prevotella sp.

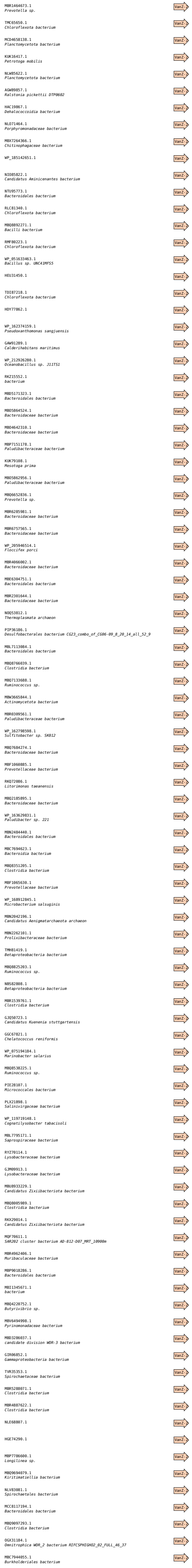
MBR1464673.1
Prevotella sp.
VanZ-2
TMC65650.1
Chloroflexota bacterium
VanZ-2
MCD4658138.1
Planctomycetota bacterium
VanZ-2
KUK16417.1
Petrotoga mobilis
VanZ-2
NLW85622.1
Planctomycetota bacterium
VanZ-2
AGW89857.1
Ralstonia pickettii DTP0602
VanZ-2
HAC19867.1
Dehalococcoidia bacterium
VanZ-2
NLO71464.1
Porphyromonadaceae bacterium
VanZ-2
MBX7264366.1
Chitinophagaceae bacterium
VanZ-2
WP_185142651.1
VanZ-2
NIO85822.1
Candidatus Aminicenantes bacterium
VanZ-2
NTU95773.1
Bacteroidales bacterium
VanZ-2
RLC81340.1
Chloroflexota bacterium
VanZ-2
MBQ8892271.1
Bacilli bacterium
VanZ-2
RMF80223.1
Chloroflexota bacterium
VanZ-2
WP_051633463.1
Bacillus sp. UNC41MFS5
VanZ-2
HEU31450.1
VanZ-2
TDI87218.1
Chloroflexota bacterium
VanZ-2
HDY77862.1
VanZ-2
WP_162374159.1
Pseudoxanthomonas sangjuensis
VanZ-2
GAW91289.1
Calderihabitans maritimus
VanZ-2
WP_212926280.1
Oceanobacillus sp. J11TS1
VanZ-2
RKZ15552.1
bacterium
VanZ-2
MBD5171323.1
Bacteroidales bacterium
VanZ-2
MBO5864524.1
Bacteroidaceae bacterium
VanZ-2
MBO4642310.1
Bacteroidaceae bacterium
VanZ-2
MBP7151178.1
Paludibacteraceae bacterium
VanZ-2
KUK79108.1
Mesotoga prima
VanZ-2
MBO5862956.1
Paludibacteraceae bacterium
VanZ-2
MBQ6652836.1
Prevotella sp.
VanZ-2
MBR6285981.1
Bacteroidaceae bacterium
VanZ-2
MBR6757565.1
Bacteroidaceae bacterium
VanZ-2
WP_205946514.1
Floccifex porci
VanZ-2
MBR4066002.1
Bacteroidaceae bacterium
VanZ-2
MBE6304751.1
Bacteroidales bacterium
VanZ-2
MBR2301644.1
Bacteroidaceae bacterium
VanZ-2
NOQ53812.1
Thermoplasmata archaeon
VanZ-2
PIP36186.1
Desulfobacterales bacterium CG23_combo_of_CG06-09_8_20_14_all_52_9
VanZ-2
MBL7113084.1
Bacteroidales bacterium
VanZ-2
MBQ8766039.1
Clostridia bacterium
VanZ-2
MBQ7133688.1
Ruminococcus sp.
VanZ-2
MBW3665844.1
Actinomycetota bacterium
VanZ-2
MBR0309561.1
Paludibacteraceae bacterium
VanZ-2
WP_162798598.1
Sulfitobacter sp. SK012
VanZ-2
MBQ7684274.1
Bacteroidaceae bacterium
VanZ-2
MBF1060885.1
Prevotellaceae bacterium
VanZ-2
RKQ72086.1
Litorimonas taeanensis
VanZ-2
MBQ2185895.1
Bacteroidaceae bacterium
VanZ-2
WP_163629831.1
Paludibacter sp. 221
VanZ-2
MBN2484440.1
Bacteroidales bacterium
VanZ-2
MBC7694623.1
Bacteroidia bacterium
VanZ-2
MBQ8351205.1
Clostridia bacterium
VanZ-2
MBF1065630.1
Prevotellaceae bacterium
VanZ-2
WP_168912845.1
Microbacterium salsuginis
VanZ-2
MBN2042196.1
Candidatus Aenigmatarchaeota archaeon
VanZ-2
MBN2262101.1
Prolixibacteraceae bacterium
VanZ-2
TMH81419.1
Betaproteobacteria bacterium
VanZ-2
MBQ8825203.1
Ruminococcus sp.
VanZ-2
NBS82808.1
Betaproteobacteria bacterium
VanZ-2
MBR1539761.1
Clostridia bacterium
VanZ-2
GJQ50723.1
Candidatus Kuenenia stuttgartensis
VanZ-2
GGC67821.1
Chelatococcus reniformis
VanZ-2
WP_075194184.1
Marinobacter salarius
VanZ-2
MBQ8538225.1
Ruminococcus sp.
VanZ-2
PIE28107.1
Micrococcales bacterium
VanZ-2
PLX21898.1
Salinivirgaceae bacterium
VanZ-2
WP_119719148.1
Cognatilysobacter tabacisoli
VanZ-2
MBL7795171.1
Saprospiraceae bacterium
VanZ-2
RYZ70114.1
Lysobacteraceae bacterium
VanZ-2
GJM09913.1
Lysobacteraceae bacterium
VanZ-2
MBU8933229.1
Candidatus Zixiibacteriota bacterium
VanZ-2
MBQ8005989.1
Clostridia bacterium
VanZ-2
RKX29014.1
Candidatus Zixiibacteriota bacterium
VanZ-2
MQF70611.1
SAR202 cluster bacterium AD-812-D07_MRT_10900m
VanZ-2
MBR4962406.1
Muribaculaceae bacterium
VanZ-2
MBP9018286.1
Bacteroidales bacterium
VanZ-2
MBI1345671.1
bacterium
VanZ-2
MBQ4220752.1
Butyrivibrio sp.
VanZ-2
MBV6494998.1
Pyrinomonadaceae bacterium
VanZ-2
MBD3286037.1
candidate division WOR-3 bacterium
VanZ-2
GIR06852.1
Gammaproteobacteria bacterium
VanZ-2
TVR35353.1
Spirochaetaceae bacterium
VanZ-2
MBR5288071.1
Clostridia bacterium
VanZ-2
MBR4887622.1
Clostridia bacterium
VanZ-2
NLE68807.1
VanZ-2
HGE74290.1
VanZ-2
MBP7786600.1
Longilinea sp.
VanZ-2
MBQ9694079.1
Kiritimatiellia bacterium
VanZ-2
NLV83081.1
Spirochaetales bacterium
VanZ-2
MCC8117194.1
Bacteroidales bacterium
VanZ-2
MBQ9097293.1
Clostridia bacterium
VanZ-2
OGX31184.1
Omnitrophica WOR_2 bacterium RIFCSPHIGHO2_02_FULL_46_37
VanZ-2
MBC7944055.1
Burkholderiales bacterium
VanZ-2

MBQ7508519.1
Spirochaetales bacterium VanZ-2

MBD0276008.1
Acetobacteraceae bacterium VanZ-2

MBR5308138.1
Clostridia bacterium VanZ-2

MBD5261738.1
Bacteroides sp. VanZ-2

MBT5877271.1
Candidatus Latescibacterota bacterium VanZ-2

SEA31009.1
Rubrimonas cliftonensis VanZ-2

MBI9061080.1
Marinilabiliaceae bacterium VanZ-2

MCA0243530.1
Pseudomonadota bacterium VanZ-2

MBU1231544.1
Pseudomonadota bacterium VanZ-2

WP_015709680.1
Treponema primitia ZAS-2 VanZ-2

MBQ2313709.1
Treponema sp. VanZ-2

HHI92123.1
Gammaproteobacteria bacterium VanZ-2

SLM14490.1
uncultured spirochete VanZ-2

MBD5358628.1
Bacteroides sp. VanZ-2

MBQ9069572.1
Eggerthellaceae bacterium VanZ-2

MBS1216639.1
Pseudomonadota bacterium VanZ-2

HED12970.1
Gammaproteobacteria bacterium VanZ-2

PYQ26349.1
Acidobacteriota bacterium VanZ-2

MBQ7147047.1
Lachnospiraceae bacterium VanZ-2

OQA27808.1
Verrucomicrobia bacterium ADurb.Bin345 VanZ-2

MAV74865.1
Halieaceae bacterium VanZ-2

MBE9516130.1
Pseudomonadota bacterium VanZ-2

HDH97152.1
Pseudomonadota bacterium VanZ-2

MCA8977813.1
Planctomycetota bacterium VanZ-2

TLZ62062.1
Methanobacteriota archaeon VanZ-2

TLZ70794.1
Methanobacteriota archaeon VanZ-2

MBP7951177.1
Verrucomicrobiales bacterium VanZ-2

NJR50965.1
Leptolyngbyaceae cyanobacterium CSU_1_3 VanZ-2

MBQ4224719.1
Oscillospiraceae bacterium VanZ-2

RLC50646.1
Candidatus Cloacimonadota bacterium VanZ-2

WP_231784220.1
Lentibacillus sp. JNUCC-1 VanZ-2

PIQ88328.1
Candidatus Ghiorseimicrobium undicola VanZ-2

MCB1095408.1
Verrucomicrobiia bacterium VanZ-2

MBI4729426.1
Acidobacteriota bacterium VanZ-2

MBN2366711.1
Calditrichota bacterium VanZ-2

MBR3495073.1
Clostridia bacterium VanZ-2

NUQ82927.1
Bacteroidota bacterium VanZ-2

UCC84997.1
Gemmatimonadota bacterium VanZ-2

MBC7666996.1
Caulobacter sp. VanZ-2

GBU08692.1
Bacteroidales bacterium VanZ-2

MCC6864916.1
Ignavibacteria bacterium VanZ-2

WP_079415880.1 VanZ-2

WP_034223596.1
Arenimonas donghaensis DSM 18148 = HO3-R19 VanZ-2

MBA3434405.1
Actinomycetota bacterium VanZ-2

RPI14235.1
Ignavibacteriota bacterium VanZ-2

PHS22296.1
Robiginitomaculum sp. VanZ-2

MCB0702138.1
Ignavibacteriota bacterium VanZ-2

MBD3410041.1
Ignavibacteriales bacterium VanZ-2

MBK8283446.1
Ahniella sp. VanZ-2

MBM3435612.1
Bacteroidota bacterium VanZ-2

MBE0540853.1
Verrucomicrobiota bacterium VanZ-2

MBS1946706.1
Bacteroidota bacterium VanZ-2

PIU45328.1
Ignavibacteriales bacterium CG07_land_8_20_14_0_80_59_12 VanZ-2

KAA9154531.1
Microbacterium lushaniae VanZ-2

MBI4328946.1
Chloroflexota bacterium VanZ-2

MBN2840798.1
Coriobacteriia bacterium VanZ-2

MBC7978728.1
Myxococcales bacterium VanZ-2

WP_011187059.1
Leifsonia xyli subsp. xyli VanZ-2

MBK5257952.1
Thermoanaerobaculia bacterium VanZ-2

PAZ00369.1
Spartobacteria bacterium AMD-G4 VanZ-2

RJP69614.1
Candidatus Abyssobacteria bacterium SURF_17 VanZ-2

KNC19855.1
Arthrobacter sp. RIT-PI-e VanZ-2

MBQ3577873.1
Bacillota bacterium VanZ-2

MBO5386732.1
Lachnospiraceae bacterium VanZ-2

MSR77454.1
Candidatus Taenariivivens baikalensis VanZ-2

MBA3627298.1
Chloroflexota bacterium VanZ-2

WP_027358105.1
Desulforegula conservatrix Mb1Pa VanZ-2

MBQ0110400.1
Candidatus Equicaccousia limihippi VanZ-2

PAW65540.1
Opitutia bacterium Tous-C1TDCM VanZ-2

MBT4758591.1
Opitutia bacterium VanZ-2

NOQ46855.1
Desulfobulbaceae bacterium VanZ-2

MBT6338334.1
Desulfobacula sp. VanZ-2

MCC6396143.1
Bacteroidota bacterium VanZ-2

MBF4510614.1
Aeromicrobium sp. VanZ-2

HFU30108.1 VanZ-2

MBT3175816.1
Desulfobacula sp. VanZ-2

MBR7092291.1
Clostridia bacterium VanZ-2

RZB31090.1
Desulfobacteraceae bacterium Eth-SRB1 VanZ-2

MCD4781043.1
Candidatus Omnitrophota bacterium VanZ-2

RJQ76698.1
Desulfobacteraceae bacterium VanZ-2

PKP06067.1
Bacteroidetes bacterium HGW-Bacteroidetes-5 VanZ-2

PKO96602.1
Bacteroidetes bacterium HGW-Bacteroidetes-7 VanZ-2

OGQ95778.1
Deltaproteobacteria bacterium RIFOXYD12_FULL_57_12 VanZ-2

MBQ2608236.1
Paludibacteraceae bacterium VanZ-2

HAS21418.1
Lachnospiraceae bacterium VanZ-2

MBB1575455.1
Flavobacteriaceae bacterium VanZ-2

MBN1543682.1
Candidatus Zhuqueibacterota bacterium VanZ-2

MBD3378175.1
Candidatus Zhuqueibacterota bacterium VanZ-2

CDE17496.1
Eubacterium sp. CAG:841 VanZ-2

MBU3923139.1
Patescibacteria group bacterium VanZ-2

MBW2516480.1
Deltaproteobacteria bacterium VanZ-2

RBP46737.1
Garciella nitratireducens VanZ-2

HIS51682.1
Candidatus Onthomonas avicola VanZ-2

MBW1809514.1	*Deltaproteobacteria bacterium*	VanZ-2
HJC44936.1	*Candidatus Faecalibacterium faecigallinarum*	VanZ-2
VTR67827.1	*Desulfosarcina cetonica*	VanZ-2
OUU06986.1	*Verrucomicrobia bacterium TMED40*	VanZ-2
QQS52669.1	*Bacteroidota bacterium*	VanZ-2
ACD83380.1	*Methylacidiphilum infernorum V4*	VanZ-2
MBN1626419.1	*Deltaproteobacteria bacterium*	VanZ-2
MBM4356411.1	*Deltaproteobacteria bacterium*	VanZ-2
MBM2805559.1	*Deltaproteobacteria bacterium*	VanZ-2
HGY09699.1	*Oceanithermus profundus*	VanZ-2
MBI4746766.1	*Deltaproteobacteria bacterium*	VanZ-2
WP_119847303.1	*Paenibacillus lautus*	VanZ-2
MBI1997168.1	*Deltaproteobacteria bacterium*	VanZ-2
HIE26398.1	*Candidatus Poribacteria bacterium*	VanZ-2
MBI2485567.1	*Deltaproteobacteria bacterium*	VanZ-2
WP_213644043.1	*Paenibacillus lautus*	VanZ-2
OGI16424.1	*Candidatus Moranbacteria bacterium RIFCSPHIGHO2_02_FULL_40_12b*	VanZ-2
MBR0280837.1	*Oscillibacter sp.*	VanZ-2
TXI34196.1	*Candidatus Moraniibacteriota bacterium*	VanZ-2
PIF03343.1	*Draconibacterium sp.*	VanZ-2
MBR5090906.1	*Ruminiclostridium sp.*	VanZ-2
QSG01324.1	*Natranaeroarchaeum sulfidigenes*	VanZ-2
WP_151680545.1	*Weizmannia acidilactici*	VanZ-2
RLB19559.1	*Deltaproteobacteria bacterium*	VanZ-2
HIX73718.1	*Candidatus Parabacteroides intestinipullorum*	VanZ-2
MBP3871064.1	*Faecalicoccus sp.*	VanZ-2
MBB2986926.1	*Terracoccus luteus*	VanZ-2
MAZ95816.1	*Planctomycetaceae bacterium*	VanZ-2
HIC89868.1	*Anaerolineae bacterium*	VanZ-2
MBC7821801.1	*Planctomycetaceae bacterium*	VanZ-2
WP_077539916.1	*Sedimentisphaera cyanobacteriorum*	VanZ-2
MBC8207702.1	*Candidatus Desulfatifera sulfidica*	VanZ-2
NOQ82888.1	*Myxococcales bacterium*	VanZ-2
MBZ0295494.1	*Anaerolineae bacterium*	VanZ-2
MCC7423955.1	*Planctomycetaceae bacterium*	VanZ-2
HGY64813.1		VanZ-2
MCA9099527.1	*Planctomycetales bacterium*	VanZ-2
MCA9188435.1	*Planctomycetales bacterium*	VanZ-2
MCD6495295.1	*Candidatus Bipolaricaulota bacterium*	VanZ-2
MBL8773429.1	*Phenylobacterium sp.*	VanZ-2
POH60662.1	*Arthrobacter glacialis*	VanZ-2
MBY0318911.1	*Reyranella sp.*	VanZ-2
GEP60037.1	*Reyranella soli*	VanZ-2
SJM71738.1	*Arthrobacter rhombi*	VanZ-2
WP_076235986.1	*Paenibacillus sp. FSL H7-0331*	VanZ-2
RLS22460.1	*Planctomycetota bacterium*	VanZ-2
MBI4767897.1	*Deltaproteobacteria bacterium*	VanZ-2
MCC7061678.1	*Planctomycetota bacterium*	VanZ-2
MBF0198038.1	*Planctomycetota bacterium*	VanZ-2
MCB9892162.1	*Planctomycetota bacterium*	VanZ-2
MBM3474481.1	*Armatimonadota bacterium*	VanZ-2
MCB0045378.1	*Caldilineaceae bacterium*	VanZ-2
HIR10357.1	*Candidatus Avoscillospira stercoripullorum*	VanZ-2
MBR3313650.1	*Atopobiaceae bacterium*	VanZ-2
MBU0617900.1	*Planctomycetota bacterium*	VanZ-2
MCD8025405.1	*Clostridiales bacterium*	VanZ-2
MBQ2770610.1	*Clostridia bacterium*	VanZ-2
TAK57883.1	*Dehalococcoidia bacterium*	VanZ-2
MBE2216731.1	*Ignavibacteria bacterium*	VanZ-2
MBH1988476.1	*Myxococcaceae bacterium*	VanZ-2
ANG62493.1	*Marinobacterium aestuarii*	VanZ-2
ESQ94100.1	*Asticcacaulis benevestitus DSM 16100 = ATCC BAA-896*	VanZ-2
WP_052039395.1	*uncultured Parvimonas sp.*	VanZ-2
MBH81021.1	*Gammaproteobacteria bacterium*	VanZ-2
MBQ4129488.1	*Ruminococcus sp.*	VanZ-2
MBK9248886.1	*Ignavibacteria bacterium*	VanZ-2
MBL8015858.1	*Ignavibacteria bacterium*	VanZ-2
HEL19423.1		VanZ-2
MBT8477601.1	*Gemmatimonadota bacterium*	VanZ-2
MBM2813588.1	*Ignavibacteria bacterium*	VanZ-2
MCC6789897.1	*Hyphomonadaceae bacterium*	VanZ-2
MCA9606667.1	*Myxococcales bacterium*	VanZ-2
MCB1153325.1	*bacterium*	VanZ-2
MBS6491079.1	*Bacillota bacterium*	VanZ-2
MBR2489065.1	*Clostridia bacterium*	VanZ-2
PLX98922.1	*Desulfuromonas sp.*	VanZ-2
MBF0623126.1	*Magnetococcales bacterium*	VanZ-2
MBM4175039.1	*Ignavibacteria bacterium*	VanZ-2
PLX86141.1	*Desulfuromonas sp.*	VanZ-2
RKZ88238.1	*Candidatus Parabeggiatoa sp. nov. 1*	VanZ-2
MBP5551954.1	*Spirochaetales bacterium*	VanZ-2
MBQ7272411.1	*Bacteroidales bacterium*	VanZ-2
WP_010960897.1	*Methylococcus capsulatus*	VanZ-2
WP_190775176.1		VanZ-2
MBI5568034.1	*Chloroflexota bacterium*	VanZ-2
MBE2284781.1	*Prosthecobacter sp.*	VanZ-2
MBQ4568759.1	*Ruminococcus sp.*	VanZ-2
MBI4300266.1	*Chloroflexota bacterium*	VanZ-2
MCD7815676.1	*Bacteroides sp.*	VanZ-2
WP_163210485.1	*Bacteroides sp. 519*	VanZ-2
MBR3640091.1	*Clostridia bacterium*	VanZ-2
WP_066109271.1		VanZ-2
MTI71647.1	*Bacillota bacterium*	VanZ-2

MBI2855567.1	*Chloroflexota bacterium*	VanZ-2
MBM3819668.1	*Acidimicrobiia bacterium*	VanZ-2
MBI2904710.1	*Chloroflexota bacterium*	VanZ-2
KPK86251.1	*Bacteroides sp. SM23_62_1*	VanZ-2
MTI67183.1	*Bacillota bacterium*	VanZ-2
ABD25544.1	*Novosphingobium aromaticivorans DSM 12444*	VanZ-2
MBQ3636326.1	*Bacteroidales bacterium*	VanZ-2
MBV6420528.1	*Ignavibacteriaceae bacterium*	VanZ-2
WP_205233361.1	*Desulfopila inferna*	VanZ-2
MCC6521378.1	*Polyangiaceae bacterium*	VanZ-2
HCV43138.1	*Bacteroidota bacterium*	VanZ-2
ERG90595.1	*Haloquadratum walsbyi J07HQW1*	VanZ-2
NLO60907.1	*Spirochaetales bacterium*	VanZ-2
ERG96259.1	*Haloquadratum walsbyi J07HQW2*	VanZ-2
RPI67123.1	*Ignavibacteriota bacterium*	VanZ-2
MBN2814131.1	*Bacteroidales bacterium*	VanZ-2
MBQ4332052.1	*Spirochaetaceae bacterium*	VanZ-2
MBI3171901.1	*Hydrocarboniphaga effusa*	VanZ-2
BCS94384.1	*Desulfoluna limicola*	VanZ-2
MCB2199801.1	*bacterium*	VanZ-2
MBF37766.1	*Idiomarinaceae bacterium*	VanZ-2
MBO24250.1	*Verrucomicrobiales bacterium*	VanZ-2
NTW05889.1	*Peptococcaceae bacterium*	VanZ-2
WP_170964437.1	*Natronomonas salsuginis*	VanZ-2
NTU89318.1	*Actinomycetota bacterium*	VanZ-2
HCY77736.1	*Ignavibacteriales bacterium*	VanZ-2
KGM36746.1	*Streptococcus sinensis*	VanZ-2
WP_153764583.1	*Endozoicomonas sp. OPT23*	VanZ-2
WP_183373996.1	*Faecalicoccus acidiformans*	VanZ-2
MBE7477644.1	*Ignavibacteriales bacterium*	VanZ-2
MYD11856.1	*Chloroflexota bacterium*	VanZ-2
MBS3772299.1	*Bacteroidales bacterium*	VanZ-2
MBO4988134.1	*Lachnospiraceae bacterium*	VanZ-2
MBP8976122.1	*Bacteroidota bacterium*	VanZ-2
HGT16310.1		VanZ-2
RPI78343.1	*Chloroflexota bacterium*	VanZ-2
MCD6388808.1	*Desulfobulbaceae bacterium*	VanZ-2
WP_162616487.1	*Xylanimonas allomyrinae*	VanZ-2
MBD5183754.1	*Bacteroidales bacterium*	VanZ-2
OQY16034.1	*Desulfobacterium sp. 4572_20*	VanZ-2
WP_075886246.1	*Faecalibaculum rodentium*	VanZ-2
MBN8457749.1	*Verrucomicrobiota bacterium*	VanZ-2
MBW6486664.1	*Syntrophobacterales bacterium*	VanZ-2
BAW00119.1	*Lysobacter enzymogenes*	VanZ-2
MAX57503.1	*Chloroflexota bacterium*	VanZ-2
MBG7606424.1	*Verrucomicrobiota bacterium*	VanZ-2
NLD23944.1	*Bacteroidales bacterium*	VanZ-2
UCC40385.1	*Candidatus Aminicenantes bacterium*	VanZ-2
MBK7229277.1	*Ignavibacteriales bacterium*	VanZ-2
OFW63870.1	*Actinobacteria bacterium RBG_19FT_COMBO_36_27*	VanZ-2
MBQ9227537.1	*Eubacterium sp.*	VanZ-2
WP_117201779.1		VanZ-2
THB72765.1	*Desulfobacteraceae bacterium*	VanZ-2
RPI98522.1	*Desulfobacteraceae bacterium*	VanZ-2
MBR3942184.1	*Clostridia bacterium*	VanZ-2
HAF30143.1	*Bacteroidales bacterium*	VanZ-2
MBQ6670823.1	*Bacillota bacterium*	VanZ-2
MBR5577448.1	*Lachnospiraceae bacterium*	VanZ-2
OGD63447.1	*Candidatus Beckwithbacteria bacterium RBG_13_42_9*	VanZ-2
MBO7301595.1	*Bacteroidaceae bacterium*	VanZ-2
OLP52614.1	*Xaviernesmea rhizosphaerae*	VanZ-2
SPD75161.1	*uncultured Desulfobacterium sp.*	VanZ-2
MXX19309.1	*Dehalococcoidia bacterium*	VanZ-2
TVR45219.1	*Puniceicoccaceae bacterium*	VanZ-2
MBQ0048515.1	*Candidatus Minthosoma equi*	VanZ-2
MAH40557.1	*Puniceicoccaceae bacterium*	VanZ-2
WP_233288317.1		VanZ-2
MBR1499784.1	*Bacteroidaceae bacterium*	VanZ-2
MCB2147760.1	*Deltaproteobacteria bacterium*	VanZ-2
MBP6215956.1	*Luteimonas sp.*	VanZ-2
MBQ2003004.1	*Bacteroidaceae bacterium*	VanZ-2
NCB19640.1	*Bacteroidia bacterium*	VanZ-2
MBT5901621.1	*Opitutaceae bacterium*	VanZ-2
NBQ58803.1	*Opitutaceae bacterium*	VanZ-2
WP_131330902.1	*Rhizosphaericola mali*	VanZ-2
WP_101232856.1	*Colwellia sp. 12G3*	VanZ-2
MBD5249913.1	*Barnesiella sp.*	VanZ-2
CDB68447.1	*Eubacterium sp. CAG:252*	VanZ-2
HAO62557.1	*Porphyromonadaceae bacterium*	VanZ-2
MBK6432244.1	*Candidatus Amarolinea dominans*	VanZ-2
MBI4595389.1	*Candidatus Tectimicrobiota bacterium*	VanZ-2
MBX3100518.1	*Salinibacterium sp.*	VanZ-2
MBD3236991.1	*Candidatus Eiseniibacteriota bacterium*	VanZ-2
MBI5183203.1	*Nitrospinota bacterium*	VanZ-2
MBN2716236.1	*Deltaproteobacteria bacterium*	VanZ-2
MBN2493061.1	*Deltaproteobacteria bacterium*	VanZ-2
NLD09225.1	*Lysobacteraceae bacterium*	VanZ-2
MBQ6480930.1	*Anaerolineaceae bacterium*	VanZ-2
KKT36671.1	*Candidatus Gottesmanbacteria bacterium GW2011_GWB1_44_11c*	VanZ-2
TLD43978.1	*Accumulibacter sp.*	VanZ-2
MBR7041352.1	*Clostridia bacterium*	VanZ-2
WP_169980727.1	*Tautonia rosea*	VanZ-2
MBS1508301.1	*Bacteroidota bacterium*	VanZ-2

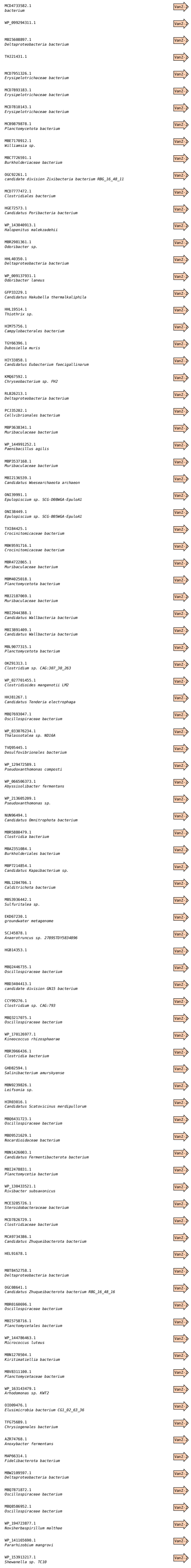
MCD4733582.1
bacterium
WP_099294311.1
MBI5608897.1
Deltaproteobacteria bacterium
THJ21431.1
MCD7951326.1
Erysipelotrichaceae bacterium
MCD7893183.1
Erysipelotrichaceae bacterium
MCD7810143.1
Erysipelotrichaceae bacterium
MCB9879878.1
Planctomycetota bacterium
MBE7170912.1
Williamsia sp.
MBC7726591.1
Burkholderiaceae bacterium
OGC92261.1
candidate division Zixibacteria bacterium RBG_16_48_11
MCD7777472.1
Clostridiales bacterium
HGE72573.1
Candidatus Poribacteria bacterium
WP_143040913.1
Halopenitus malekzadehii
MBR2981361.1
Odoribacter sp.
HHL40350.1
Deltaproteobacteria bacterium
WP_009137931.1
Odoribacter laneus
GFP33229.1
Candidatus Hakubella thermalkaliphila
HHL19514.1
Thiothrix sp.
HIM75756.1
Campylobacterales bacterium
TGY66396.1
Dubosiella muris
HIY33858.1
Candidatus Eubacterium faecigallinarum
KMQ67592.1
Chryseobacterium sp. FH2
RLB26213.1
Deltaproteobacteria bacterium
PCJ35202.1
Cellvibrionales bacterium
MBP3638341.1
Muribaculaceae bacterium
WP_144991252.1
Paenibacillus agilis
MBP3537168.1
Muribaculaceae bacterium
MBI2136539.1
Candidatus Woesearchaeota archaeon
ONI39991.1
Epulopiscium sp. SCG-D08WGA-EpuloA1
ONI38449.1
Epulopiscium sp. SCG-B05WGA-EpuloA1
TXI84425.1
Crocinitomicaceae bacterium
MBK9591716.1
Crocinitomicaceae bacterium
MBR4722865.1
Muribaculaceae bacterium
MBM4025018.1
Planctomycetota bacterium
MBJ2187069.1
Muribaculaceae bacterium
MBI2944388.1
Candidatus Wallbacteria bacterium
MBI3891409.1
Candidatus Wallbacteria bacterium
MBL9077315.1
Planctomycetota bacterium
OKZ91313.1
Clostridium sp. CAG:307_30_263
WP_027701455.1
Clostridioides mangenotii LM2
HHJ81267.1
Candidatus Tenderia electrophaga
MBQ7693047.1
Oscillospiraceae bacterium
WP_033076234.1
Thalassotalea sp. ND16A
TVQ95445.1
Desulfovibrionales bacterium
WP_129472589.1
Pseudoxanthomonas composti
WP_066506373.1
Abyssisolibacter fermentans
WP_213605209.1
Pseudoxanthomonas sp.
NUN96494.1
Candidatus Omnitrophota bacterium
MBR5880479.1
Clostridia bacterium
MBA2351084.1
Burkholderiales bacterium
MBP7214854.1
Candidatus Kapaibacterium sp.
MBL1204706.1
Calditrichota bacterium
MBS3936442.1
Sulfuritalea sp.
EKD67230.1
groundwater metagenome
SCJ45878.1
Anaerotruncus sp. 2789STDY5834896
HGB14353.1
MBQ2446735.1
Oscillospiraceae bacterium
MBD3404413.1
candidate division GN15 bacterium
CCY99276.1
Clostridium sp. CAG:793
MBQ3217075.1
Oscillospiraceae bacterium
WP_170126977.1
Kineococcus rhizosphaerae
MBR3966436.1
Clostridia bacterium
GHD82594.1
Salinibacterium amurskyense
MBN9239826.1
Leifsonia sp.
HIR03016.1
Candidatus Scatovicinus merdipullorum
MBQ6431723.1
Oscillospiraceae bacterium
MBO9521629.1
Nocardioidaceae bacterium
MBN1426003.1
Candidatus Fermentibacterota bacterium
MBI2478831.1
Planctomycetia bacterium
WP_130433521.1
Rivibacter subsaxonicus
MCE3285726.1
Steroidobacteraceae bacterium
MCD7826729.1
Clostridiaceae bacterium
MCA9734386.1
Candidatus Zhuqueibacterota bacterium
HEL91678.1
MBT8452758.1
Deltaproteobacteria bacterium
OGC08641.1
Candidatus Zhuqueibacterota bacterium RBG_16_48_16
MBR0160696.1
Oscillospiraceae bacterium
MBI5758716.1
Planctomycetales bacterium
WP_144786463.1
Micrococcus luteus
MBN1270504.1
Kiritimatiellia bacterium
MBV8311100.1
Planctomycetaceae bacterium
WP_163143479.1
Arhodomonas sp. KWT2
OIO09476.1
Elusimicrobia bacterium CG1_02_63_36
TFG75689.1
Chrysiogenales bacterium
AZR74768.1
Anoxybacter fermentans
MAP66314.1
Fidelibacterota bacterium
MBW2109597.1
Deltaproteobacteria bacterium
MBQ7871872.1
Oscillospiraceae bacterium
MBQ8586952.1
Oscillospiraceae bacterium
WP_194723877.1
Noviherbaspirillum malthae
WP_141165690.1
Pararhizobium mangrovi
WP_153913217.1
Shewanella sp. TC10

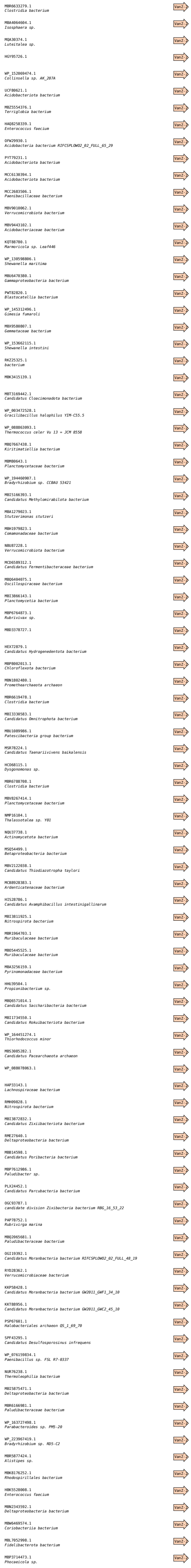
MBR6633279.1
Clostridia bacterium
MBA4064604.1
Isosphaera sp.
MQA30374.1
Luteitalea sp.
HGY95726.1
WP_152069474.1
Collinsella sp. AK_207A
UCF80621.1
Acidobacteriota bacterium
MBZ5554376.1
Terriglobia bacterium
HAQ8258339.1
Enterococcus faecium
OFW29930.1
Acidobacteria bacterium RIFCSPLOWO2_02_FULL_65_29
PYT79231.1
Acidobacteriota bacterium
MCC6130394.1
Acidobacteriota bacterium
MCC2683506.1
Paenibacillaceae bacterium
MBV9010062.1
Verrucomicrobiota bacterium
MBV9443102.1
Acidobacteriaceae bacterium
KQT88780.1
Marmoricola sp. Leaf446
WP_130598806.1
Shewanella maritima
MBU6470380.1
Gammaproteobacteria bacterium
PWT82820.1
Blastocatellia bacterium
WP_145312496.1
Gimesia fumaroli
MBX9580807.1
Gemmataceae bacterium
WP_153662115.1
Shewanella intestini
RKZ25325.1
bacterium
MBK3415139.1
MBT3169442.1
Candidatus Cloacimonadota bacterium
WP_003472528.1
Gracilibacillus halophilus YIM-C55.5
WP_088863093.1
Thermococcus celer Vu 13 = JCM 8558
MBQ7667438.1
Kiritimatiellia bacterium
MBM80643.1
Planctomycetaceae bacterium
WP_194460907.1
Bradyrhizobium sp. CCBAU 53421
MBI5166393.1
Candidatus Methylomirabilota bacterium
MBA1279023.1
Stutzerimonas stutzeri
MBH1979823.1
Comamonadaceae bacterium
NBU87228.1
Verrucomicrobiota bacterium
MCD6589312.1
Candidatus Fermentibacteraceae bacterium
MBQ6404075.1
Oscillospiraceae bacterium
MBI3866143.1
Planctomycetia bacterium
MBP6764873.1
Rubrivivax sp.
MBD3378727.1
HEX72879.1
Candidatus Hydrogenedentota bacterium
MBP8002013.1
Chloroflexota bacterium
MBN1802480.1
Promethearchaeota archaeon
MBR6619478.1
Clostridia bacterium
MBI3330583.1
Candidatus Omnitrophota bacterium
MBU1089986.1
Patescibacteria group bacterium
MSR78224.1
Candidatus Taenariivivens baikalensis
HCO68115.1
Dysgonomonas sp.
MBR6788708.1
Clostridia bacterium
MBV8267414.1
Planctomycetaceae bacterium
NMP16104.1
Thalassotalea sp. Y01
NQU37738.1
Actinomycetota bacterium
MSQ54499.1
Betaproteobacteria bacterium
MBV2122038.1
Candidatus Thiodiazotropha taylori
MCB8928383.1
Ardenticatenaceae bacterium
HIS28786.1
Candidatus Avamphibacillus intestinigallinarum
MBI3811925.1
Nitrospirota bacterium
MBR1964703.1
Muribaculaceae bacterium
MBO5445525.1
Muribaculaceae bacterium
MBA3256159.1
Pyrinomonadaceae bacterium
HHU39504.1
Propionibacterium sp.
MBQ6571014.1
Candidatus Saccharibacteria bacterium
MBI1734550.1
Candidatus Rokuibacteriota bacterium
WP_164451274.1
Thiorhodococcus minor
MBS3085282.1
Candidatus Pacearchaeota archaeon
WP_088078063.1
HAP33143.1
Lachnospiraceae bacterium
RMH09828.1
Nitrospirota bacterium
MBI3872832.1
Candidatus Zixiibacteriota bacterium
RME27640.1
Deltaproteobacteria bacterium
MBB14598.1
Candidatus Poribacteria bacterium
MBP7612986.1
Paludibacter sp.
PLX24452.1
Candidatus Parcubacteria bacterium
OGC93787.1
candidate division Zixibacteria bacterium RBG_16_53_22
PAP78752.1
Rubrivirga marina
MBQ2065681.1
Paludibacteraceae bacterium
OGI19392.1
Candidatus Moranbacteria bacterium RIFCSPLOWO2_02_FULL_48_19
RYD28362.1
Verrucomicrobiaceae bacterium
KKP58428.1
Candidatus Moranbacteria bacterium GW2011_GWF1_34_10
KKT88956.1
Candidatus Moranbacteria bacterium GW2011_GWC2_45_10
PSP67601.1
Halobacteriales archaeon QS_1_69_70
SPF43295.1
Candidatus Desulfosporosinus infrequens
WP_076159834.1
Paenibacillus sp. FSL R7-0337
NUR76238.1
Thermoleophilia bacterium
MBI5875471.1
Deltaproteobacteria bacterium
MBR6166981.1
Paludibacteraceae bacterium
WP_163727498.1
Parabacteroides sp. PM5-20
WP_223967419.1
Bradyrhizobium sp. RD5-C2
MBR5877424.1
Alistipes sp.
MBK8176252.1
Rhodospirillales bacterium
HBK5528008.1
Enterococcus faecium
MBN2343592.1
Deltaproteobacteria bacterium
MBW6469574.1
Coriobacteriia bacterium
MBL7052998.1
Fidelibacterota bacterium
MBP3714473.1
Phocaeicola sp.

MBR2682773.1	*Atopobiaceae bacterium*	VanZ-2
HEC35512.1	*Anaerolineae bacterium*	VanZ-2
ODU51728.1	*Lysobacteraceae bacterium SCN 69-48*	VanZ-2
HBI57837.1	*Porphyromonadaceae bacterium*	VanZ-2
NPV09435.1	*Anaerolineae bacterium*	VanZ-2
MBA3874846.1	*Anaerolineae bacterium*	VanZ-2
MBQ6658757.1	*Prevotella sp.*	VanZ-2
MCD4833208.1	*Bacteroidales bacterium*	VanZ-2
MBL7201031.1	*Anaerolineae bacterium*	VanZ-2
MBA2644528.1	*Solirubrobacterales bacterium*	VanZ-2
MBW1793374.1	*Deltaproteobacteria bacterium*	VanZ-2
MBJ7390505.1	*Chthoniobacterales bacterium*	VanZ-2
WP_184014986.1	*Haloferula luteola*	VanZ-2
HHW49074.1	*Clostridiaceae bacterium*	VanZ-2
MBW8850735.1	*Lysobacterales bacterium*	VanZ-2
MBO6288004.1	*Prevotella sp.*	VanZ-2
WP_130603752.1	*Litorilituus sediminis*	VanZ-2
MBR4140607.1	*Campylobacter sp.*	VanZ-2
MBW2260949.1	*Deltaproteobacteria bacterium*	VanZ-2
MBT3314905.1	*Anaerolineae bacterium*	VanZ-2
MBW2427413.1	*Deltaproteobacteria bacterium*	VanZ-2
OFY58651.1	*Bacteroidetes bacterium RBG_19FT_COMBO_42_10*	VanZ-2
WP_170251266.1	*Rhizobium sp. SEMIA 4085*	VanZ-2
MBR2133453.1	*Eubacterium sp.*	VanZ-2
PSQ62762.1	*Bacteroidetes bacterium QH_1_61_8*	VanZ-2
MBS7367562.1	*Prevotella sp.*	VanZ-2
WP_011971589.1	*Alkaliphilus metalliredigens QYMF*	VanZ-2
OFX46822.1	*Bacteroidetes bacterium GWA2_33_15*	VanZ-2
MCD8202218.1	*Prevotella sp.*	VanZ-2
MCD5416237.1	*Candidatus Bipolaricaulota bacterium*	VanZ-2
MBR5852187.1	*Bacteroidaceae bacterium*	VanZ-2
MBS7408510.1	*Prevotellamassilia sp.*	VanZ-2
HIG79032.1	*Cycloclasticus sp.*	VanZ-2
MBG70591.1	*Roseibacillus sp.*	VanZ-2
NLF04457.1	*Actinomycetales bacterium*	VanZ-2
RJR50607.1	*Desulfobacteraceae bacterium*	VanZ-2
HGV28651.1		VanZ-2
NBH13388.1	*Lachnospiraceae bacterium*	VanZ-2
MBU2528024.1	*bacterium*	VanZ-2
WP_064749385.1	*Lysobacter firmicutimachus*	VanZ-2
BCT92417.1	*Noviluteimonas caseinilytica*	VanZ-2
SEQ37617.1	*Lachnospiraceae bacterium RM5*	VanZ-2
NCD34588.1	*Spartobacteria bacterium*	VanZ-2
KRC41376.1	*Phenylobacterium sp. Root77*	VanZ-2
NVK00913.1	*Oceanospirillaceae bacterium*	VanZ-2
MBW3659159.1	*Actinomycetota bacterium*	VanZ-2
MBZ0157392.1	*Candidatus Nitrobium versatile*	VanZ-2
MBD5174404.1	*Bacteroidales bacterium*	VanZ-2
MBS1730332.1	*Bacteroidota bacterium*	VanZ-2
WP_142532840.1		VanZ-2
OQC27275.1	*Verrucomicrobia bacterium ADurb.Bin063*	VanZ-2
MBM2840628.1	*Bacteroidota bacterium*	VanZ-2
RLL56046.1	*Mariprofundus sp. EBB-1*	VanZ-2
MBE7495248.1	*Verrucomicrobiaceae bacterium*	VanZ-2
MCC3197686.1		VanZ-2
MBE6295919.1	*Bacteroidales bacterium*	VanZ-2
NLX18824.1	*Desulfobulbus sp.*	VanZ-2
WP_213298513.1	*Acidovorax sp. CCYZU-2555*	VanZ-2
NLG49109.1	*Chloroflexota bacterium*	VanZ-2
NBC84273.1	*Bacteroidota bacterium*	VanZ-2
MBI4547897.1	*Ignavibacteriota bacterium*	VanZ-2
WP_052012330.1	*Alicyclobacillus hesperidum URH17-3-68*	VanZ-2
OJW76173.1	*Spirosoma sp. 48-14*	VanZ-2
TNF38489.1	*Bacteroidota bacterium*	VanZ-2
NLM56049.1	*Clostridiales bacterium*	VanZ-2
WP_204713098.1	*Faecalitalea cylindroides*	VanZ-2
MBI3195742.1	*Ignavibacteriota bacterium*	VanZ-2
WP_162026045.1	*Lentimonas sp. CC8*	VanZ-2
WP_059057625.1	*Halobacterium hubeiense*	VanZ-2
RMD78749.1	*Gammaproteobacteria bacterium*	VanZ-2
NLN23132.1	*Clostridiales bacterium*	VanZ-2
MBE0572716.1		VanZ-2
NBX76887.1	*Pseudomonadota bacterium*	VanZ-2
KPK86975.1	*Bacteroides sp. SM23_62_1*	VanZ-2
MBN2735802.1	*Spirochaetales bacterium*	VanZ-2
WP_051564145.1	*Desulfovermiculus halophilus DSM 18834*	VanZ-2
MBR2281305.1	*Spirochaetales bacterium*	VanZ-2
WP_152666496.1		VanZ-2
MBO4280064.1	*Spirochaetales bacterium*	VanZ-2
MBI5648904.1	*Chloroflexota bacterium*	VanZ-2
NLN41536.1	*Clostridiales bacterium*	VanZ-2
MCE2503875.1	*Chlorobiota bacterium*	VanZ-2
PYN74576.1	*Candidatus Rokuibacteriota bacterium*	VanZ-2
MBD5354702.1	*Bacteroides sp.*	VanZ-2
MBD5270094.1	*Bacteroides sp.*	VanZ-2
QDX91878.1	*Brevibacillus laterosporus*	VanZ-2
HEC44053.1	*Bacteroides sp.*	VanZ-2
PLX86179.1	*Desulfuromonas sp.*	VanZ-2
MBE3064960.1	*Spirochaetota bacterium*	VanZ-2
MBU1168444.1	*Pseudomonadota bacterium*	VanZ-2
NCB03352.1	*Spirochaetia bacterium*	VanZ-2
MBP6300293.1	*Arenimonas sp.*	VanZ-2
MBQ6570605.1	*Clostridia bacterium*	VanZ-2

Accession	Organism	Gene
MBI1805348.1	*Ignavibacteria bacterium*	VanZ-2
PSP80459.1	*Halobacteriales archaeon QS_1_68_20*	VanZ-2
WP_002701928.1	*Treponema saccharophilum*	VanZ-2
RMD89251.1	*Alphaproteobacteria bacterium*	VanZ-2
MBO6543948.1	*Alphaproteobacteria bacterium*	VanZ-2
MBQ4192679.1	*Clostridia bacterium*	VanZ-2
MBP9986757.1	*Candidatus Equibacterium intestinale*	VanZ-2
NLO12840.1	*Clostridiales bacterium*	VanZ-2
TGY97922.1	*Petralouisia muris*	VanZ-2
TAD75904.1	*Oscillatoriales cyanobacterium*	VanZ-2
MBG6053915.1		VanZ-2
MAT74913.1	*Candidatus Poribacteria bacterium*	VanZ-2
MBC8120438.1	*Gloeobacterales cyanobacterium ES-bin-141*	VanZ-2
NJO89283.1	*Chloroflexia bacterium*	VanZ-2
UCE77363.1	*Gammaproteobacteria bacterium*	VanZ-2
GHE79939.1	*Camelimonas fluminis*	VanZ-2
AQR63374.1	*Brevundimonas sp. LM2*	VanZ-2
HCQ54438.1	*Brevundimonas diminuta*	VanZ-2
TAJ19820.1	*Dehalococcoidia bacterium*	VanZ-2
MCD8486989.1	*Desertifilum sp.*	VanZ-2
MBS4932638.1	*Clostridiales bacterium*	VanZ-2
TVR53488.1	*Gemmatimonadales bacterium*	VanZ-2
HBG49567.1	*Cyanobacteria bacterium UBA9971*	VanZ-2
NUO62703.1	*Gemmatimonadaceae bacterium*	VanZ-2
HIW32538.1		VanZ-2
OIO37333.1	*Candidatus Omnitrophica bacterium CG1_02_49_10*	VanZ-2
MBC7473133.1	*Candidatus Sericytochromatia bacterium*	VanZ-2
MSQ42333.1	*Dehalococcoidia bacterium*	VanZ-2
MBX9877461.1		VanZ-2
NIP57933.1	*Gemmatimonadota bacterium*	VanZ-2
WP_215608090.1	*Leptothoe spongobia TAU-MAC 1115*	VanZ-2
WP_168663212.1		VanZ-2
MBI4456929.1	*Acidobacteriota bacterium*	VanZ-2
MBQ0831630.1	*Marinobacter sp.*	VanZ-2
MBM4158279.1	*Ignavibacteria bacterium*	VanZ-2
WP_023460189.1	*Asticcacaulis sp. AC466*	VanZ-2
AHI28565.1	*Marinobacter similis*	VanZ-2
MCC6731222.1	*Chthonomonadales bacterium*	VanZ-2
WP_224412694.1	*Oscillatoria salina IIICB1*	VanZ-2
WP_066227518.1	*Heyndrickxia sporothermodurans*	VanZ-2
WP_184773707.1	*Aminobacter carboxidus*	VanZ-2
WP_010933181.1	*Chlorobaculum tepidum TLS*	VanZ-2
RXK82481.1	*Chlorobaculum sp. 24CR*	VanZ-2
WP_100637959.1	*Marinobacter salexigens*	VanZ-2
WP_066268980.1	*Heyndrickxia acidicola*	VanZ-2
MBV9045381.1	*Alphaproteobacteria bacterium*	VanZ-2
NJM98785.1	*Phormidesmis sp. RL_2_1*	VanZ-2
MBP3365409.1	*Treponema sp.*	VanZ-2
TCR76683.1	*Rhizobium sp. BK376*	VanZ-2
MBX6313082.1	*Isosphaeraceae bacterium*	VanZ-2
MBR4661084.1	*Bacteroidales bacterium*	VanZ-2
UCH15655.1	*Bacteroidales bacterium*	VanZ-2
KPQ26660.1	*Marinobacter excellens HL-55*	VanZ-2
WP_100689029.1	*Marinobacter aromaticivorans*	VanZ-2
MBR1376718.1	*Bacilli bacterium*	VanZ-2
MCD6565454.1	*Bacteroidales bacterium*	VanZ-2
MBQ9136314.1	*Lachnospiraceae bacterium*	VanZ-2
WP_128560811.1	*Methylobacterium crusticola*	VanZ-2
HJC37924.1	*Candidatus Mediterraneibacter faecigallinarum*	VanZ-2
MBL8850013.1	*Planctomycetaceae bacterium*	VanZ-2
WP_205451607.1	*Methylomonas sp. EFPC1*	VanZ-2
MBO5789879.1	*Clostridia bacterium*	VanZ-2
WP_033158205.1	*Methylomonas sp. WH-1*	VanZ-2
MBN9580056.1	*Afipia sp.*	VanZ-2
MBQ4021032.1	*Bacteroidales bacterium*	VanZ-2
RLD35215.1	*Bacteroidota bacterium*	VanZ-2
TML32735.1	*Actinomycetota bacterium*	VanZ-2
WP_120499796.1	*Roseovarius sp. EL26*	VanZ-2
OMH25774.1	*Motiliproteus sp. MSK22-1*	VanZ-2
WP_190998282.1	*Peribacillus faecalis*	VanZ-2
MBL9174043.1		VanZ-2
HCM13143.1	*Lachnospiraceae bacterium*	VanZ-2
WP_117445610.1	*Faecalicoccus pleomorphus*	VanZ-2
WP_015752403.1	*Desulfohalobium retbaense DSM 5692*	VanZ-2
PPK97403.1	*Kineococcus xinjiangensis*	VanZ-2
NLF65775.1	*Chloroflexota bacterium*	VanZ-2
HHH81993.1	*Chloroflexota bacterium*	VanZ-2
RPI66732.1	*Ignavibacteriales bacterium*	VanZ-2
WP_205179762.1	*Neobacillus cucumis*	VanZ-2
RLE53519.1	*Candidatus Methanomethylicota archaeon*	VanZ-2
MBK8090601.1	*Verrucomicrobiaceae bacterium*	VanZ-2
PIE58315.1	*Desulfobulbus propionicus*	VanZ-2
PID71515.1	*Desulfobulbus propionicus*	VanZ-2
WP_205242355.1	*Desulfobulbus alkaliphilus*	VanZ-2
MBP1648560.1	*Bacteroidota bacterium*	VanZ-2
MBP1658071.1	*Bacteroidota bacterium*	VanZ-2
MBD5204738.1	*Bacteroidales bacterium*	VanZ-2
CAA9470726.1	*uncultured Solirubrobacteraceae bacterium*	VanZ-2
MBU6181261.1	*Verrucomicrobiota bacterium*	VanZ-2
MBW2465915.1	*Deltaproteobacteria bacterium*	VanZ-2
WP_144816722.1	*Aerolutibacter ruishenii*	VanZ-2
MBR2285144.1	*Paludibacteraceae bacterium*	VanZ-2
PLX50252.1	*Desulfobulbaceae bacterium*	VanZ-2

NBC48576.1	*Gammaproteobacteria bacterium*	VanZ-2
HHU59117.1	*Bacteroidales bacterium*	VanZ-2
MBI4811642.1	*Ignavibacteriales bacterium*	VanZ-2
MBI4824725.1	*Nitrospirota bacterium*	VanZ-2
OIP93068.1	*Syntrophaceae bacterium CG2_30_58_14*	VanZ-2
MZP82656.1	*Bacteroidales bacterium*	VanZ-2
CAA6810818.1	*uncultured Thiotrichaceae bacterium*	VanZ-2
HBH47510.1	*Bacteroidales bacterium*	VanZ-2
WP_008730248.1	*Actinomyces massiliensis*	VanZ-2
MCB0186639.1	*Caldilineaceae bacterium*	VanZ-2
MCA1793397.1	*Desulfobacteraceae bacterium*	VanZ-2
WP_162987509.1	*Metabacillus litoralis*	VanZ-2
MCB0033819.1	*Anaerolineales bacterium*	VanZ-2
WP_232145563.1	*Luteimonas sp. Y-2-2-4F*	VanZ-2
OGQ21100.1	*Deltaproteobacteria bacterium RBG_16_71_12*	VanZ-2
MBQ3021780.1	*Clostridia bacterium*	VanZ-2
HIC96750.1	*Candidatus Bipolaricaulota bacterium*	VanZ-2
MBR5295838.1	*Clostridia bacterium*	VanZ-2
WP_068335981.1	*Photobacterium jeanii*	VanZ-2
WP_195963813.1	*Clostridium cuniculi*	VanZ-2
KPP96148.1	*Bacteroidetes bacterium HLUCCA01*	VanZ-2
MBF1079044.1	*Prevotellaceae bacterium*	VanZ-2
MBQ8736352.1	*Bacteroidaceae bacterium*	VanZ-2
MBM3948453.1	*SAR202 cluster bacterium*	VanZ-2
WP_051215035.1	*Granulicoccus phenolivorans DSM 17626 = NBRC 107789 = JCM 15570*	VanZ-2
WP_144414928.1	*Photobacterium swingsii*	VanZ-2
MCE1188364.1	*Ignavibacteria bacterium*	VanZ-2
NCO11498.1	*Candidatus Pacearchaeota archaeon*	VanZ-2
WP_152118889.1	*Halosegnis rubeus*	VanZ-2
KKS31755.1	*Candidatus Collierbacteria bacterium GW2011_GWB2_42_12*	VanZ-2
MCD6098196.1	*bacterium*	VanZ-2
OGY74518.1	*Candidatus Jacksonbacteria bacterium RIFOXYA2_FULL_43_12*	VanZ-2
WP_076533203.1	*Roseovarius nanhaiticus*	VanZ-2
WP_161340453.1	*Sneathiella chungangensis*	VanZ-2
HHO48258.1	*Desulfobacteraceae bacterium*	VanZ-2
MBF0121481.1	*Desulfobacterales bacterium*	VanZ-2
KLU62687.1	*Peptococcaceae bacterium CEB3*	VanZ-2
NQU94952.1	*Candidatus Omnitrophota bacterium*	VanZ-2
MBW6493198.1	*Burkholderiaceae bacterium*	VanZ-2
MSU66041.1	*Opitutus sp.*	VanZ-2
MBR3370161.1	*Paracoccaceae bacterium*	VanZ-2
TET33450.1	*Anaerolineales bacterium*	VanZ-2
MBW2606264.1	*Deltaproteobacteria bacterium*	VanZ-2
MCC5834116.1	*Opitutales bacterium*	VanZ-2
PAW72300.1	*Opitutia bacterium Tous-C5TDCM*	VanZ-2
MBP5569713.1	*Prevotella sp.*	VanZ-2
WP_104343326.1	*Clavibacter michiganensis*	VanZ-2
HIO93455.1	*Leucothrix mucor*	VanZ-2
MBR1548154.1	*Prevotella sp.*	VanZ-2
OFX13079.1	*Armatimonadetes bacterium RBG_16_58_9*	VanZ-2
WP_141432848.1	*Bacillus sp. 03113*	VanZ-2
MBN1177586.1	*Anaerolineae bacterium*	VanZ-2
MBW1675448.1	*Deltaproteobacteria bacterium*	VanZ-2
MBC7250452.1	*Anaerolineae bacterium*	VanZ-2
MBI2398653.1	*Lysobacterales bacterium*	VanZ-2
MBR0527391.1	*Prevotella sp.*	VanZ-2
MBQ6187302.1	*Prevotella sp.*	VanZ-2
MBA3055523.1	*Sphingomonadales bacterium*	VanZ-2
MBE6692586.1	*Oscillospiraceae bacterium*	VanZ-2
MBI5168520.1	*Candidatus Eiseniibacteriota bacterium*	VanZ-2
MBW2502103.1	*Deltaproteobacteria bacterium*	VanZ-2
MBR3284174.1	*Alloprevotella sp.*	VanZ-2
NLT41372.1	*Anaerolineae bacterium*	VanZ-2
WP_118045181.1	*Blautia obeum*	VanZ-2
RKV71931.1	*Alloprevotella sp.*	VanZ-2
NIA19962.1	*Lysobacteraceae bacterium*	VanZ-2
HBR71701.1	*Candidatus Moraniibacteriota bacterium*	VanZ-2
NET33617.1	*Cyanothece sp. SIO1E1*	VanZ-2
MBT6210456.1	*Woeseia sp.*	VanZ-2
EKE11769.1	*groundwater metagenome*	VanZ-2
MBO7675592.1	*Atopobiaceae bacterium*	VanZ-2
MBQ7063414.1	*Bacillota bacterium*	VanZ-2
MBF0599243.1	*Atopobiaceae bacterium FL090493*	VanZ-2
HGV98493.1		VanZ-2
WP_194538250.1	*Thermogemmata fonticola*	VanZ-2
MBR4814449.1	*Paludibacteraceae bacterium*	VanZ-2
MBM4350228.1	*Deltaproteobacteria bacterium*	VanZ-2
MBD3238257.1	*Candidatus Moraniibacteriota bacterium*	VanZ-2
MBI2439026.1	*Candidatus Moraniibacteriota bacterium*	VanZ-2
WP_206037643.1	*Rhodococcus sp. HNM0569*	VanZ-2
WP_212548324.1		VanZ-2
MBP1592196.1	*Oscillospiraceae bacterium*	VanZ-2
MBQ3930060.1	*Paludibacteraceae bacterium*	VanZ-2
MBQ4036104.1	*Paludibacteraceae bacterium*	VanZ-2
WP_105213637.1	*Pseudoalteromonas sp. T1lg22*	VanZ-2
MBD3180922.1	*Candidatus Poribacteria bacterium*	VanZ-2
HAM49620.1	*Nitrospiraceae bacterium*	VanZ-2
NOZ85357.1	*Deltaproteobacteria bacterium*	VanZ-2
MBA4075932.1	*Cyanobacteria bacterium PR.023*	VanZ-2
MBL4869864.1		VanZ-2
WP_068264175.1	*Rubripirellula obstinata*	VanZ-2
RMF21288.1	*Deltaproteobacteria bacterium*	VanZ-2
WP_169641087.1	*Roseobacter ponti*	VanZ-2

NOY64674.1	*Nitrospirota bacterium*	VanZ-2
MBS7347125.1	*Muribaculaceae bacterium*	VanZ-2
WP_161158600.1	*Vibrio nitrifigilis*	VanZ-2
MBP2692077.1	*Muribaculaceae bacterium*	VanZ-2
WP_184091901.1	*Anaerocolumna cellulosilytica*	VanZ-2
MBQ7351488.1	*Clostridia bacterium*	VanZ-2
MBO5052670.1	*Muribaculaceae bacterium*	VanZ-2
MBI3595651.1	*Nitrospirota bacterium*	VanZ-2
MBL0336333.1	*Chitinophagaceae bacterium*	VanZ-2
MAI78768.1	*Deltaproteobacteria bacterium*	VanZ-2
MBM3333675.1	*Candidatus Sumerlaeota bacterium*	VanZ-2
MBQ5697289.1	*Muribaculaceae bacterium*	VanZ-2
MBS0265781.1	*Planctomycetota bacterium*	VanZ-2
MBN1477738.1	*Candidatus Sumerlaeota bacterium*	VanZ-2
MBU0984979.1	*Candidatus Zixiibacteriota bacterium*	VanZ-2
MBJ2166517.1	*Muribaculaceae bacterium*	VanZ-2
WP_066790429.1	*Rummeliibacillus stabekisii*	VanZ-2
WP_076570706.1	*Salimicrobium salexigens*	VanZ-2
MBD3382898.1	*Candidatus Zixiibacteriota bacterium*	VanZ-2
NMC43310.1	*Candidatus Zixiibacteriota bacterium*	VanZ-2
GBE16530.1	*bacterium BMS3Abin15*	VanZ-2
OGW34196.1	*Nitrospirae bacterium GWC2_56_14*	VanZ-2
WP_100374125.1	*Bacillus sp. FJAT-45037*	VanZ-2
PWI20238.1	*Streptomyces sp. Act143*	VanZ-2
MBM4002967.1	*Planctomycetota bacterium*	VanZ-2
WP_059755618.1	*Thiobacillus denitrificans*	VanZ-2
WP_130532358.1	*Pseudoxanthomonas winnipegensis*	VanZ-2
MBL8723861.1	*Planctomycetota bacterium*	VanZ-2
KKP92480.1	*Parcubacteria group bacterium GW2011_GWA1_36_12*	VanZ-2
EKD22067.1	*groundwater metagenome*	VanZ-2
MBI3317401.1	*Candidatus Omnitrophota bacterium*	VanZ-2
HHX65368.1	*Chloroflexota bacterium*	VanZ-2
MBN1687634.1	*Candidatus Omnitrophota bacterium*	VanZ-2
HAV93028.1	*candidate division WOR-3 bacterium*	VanZ-2
WP_161596972.1	*Rhizobium glycinendophyticum*	VanZ-2
MCD6040897.1	*Burkholderiales bacterium*	VanZ-2
PKO61091.1	*Betaproteobacteria bacterium HGW-Betaproteobacteria-17*	VanZ-2
ODS22743.1	*Candidatus Endobugula sertula*	VanZ-2
MBR3606481.1	*Lachnospiraceae bacterium*	VanZ-2
KPJ63519.1	*candidate division KD3-62 bacterium DG_56*	VanZ-2
MBC7858295.1	*Burkholderiaceae bacterium*	VanZ-2
WP_183376408.1	*Helcobacillus massiliensis*	VanZ-2
HJG37985.1	*Enorma phocaeensis*	VanZ-2
NIR53156.1	*Candidatus Zhuqueibacterota bacterium*	VanZ-2
HCU64296.1	*Rheinheimera sp.*	VanZ-2
MBQ3140191.1	*Ruminococcus sp.*	VanZ-2
MBI3350785.1	*Burkholderiales bacterium*	VanZ-2
MBL8864900.1	*Planctomycetia bacterium*	VanZ-2
WP_127588423.1		VanZ-2
MBN2009518.1	*Candidatus Zhuqueibacterota bacterium*	VanZ-2
WP_171198210.1	*Nakamurella aerolata*	VanZ-2
MAC34104.1	*Haliea sp.*	VanZ-2
MBP6850632.1	*Rhodoferax sp.*	VanZ-2
WP_120745390.1	*Paenibacillus ginsengarvi*	VanZ-2
WP_196788540.1	*Motilibacter rhizosphaerae*	VanZ-2
NLN93595.1	*Candidatus Hydrogenedens sp.*	VanZ-2
WP_088653618.1	*Geofilum rhodophaeum*	VanZ-2
NLW14748.1	*Erysipelothrix sp.*	VanZ-2
WP_011466577.1	*Saccharophagus degradans 2-40*	VanZ-2
MBL6599918.1	*Alphaproteobacteria bacterium*	VanZ-2
MBQ8994286.1	*Oscillospiraceae bacterium*	VanZ-2
RJS95000.1	*Salinisphaera sp. Q1T1-3*	VanZ-2
MBP3942175.1	*Rhinopithecimicrobium faecis*	VanZ-2
OQB22676.1	*candidate division BRC1 bacterium ADurb.Bin183*	VanZ-2
QWH85245.1	*Bacillus mycoides*	VanZ-2
UCH12049.1	*Candidatus Omnitrophota bacterium*	VanZ-2
CAA9340593.1	*uncultured Lysobacter sp.*	VanZ-2
WP_129072498.1	*Salinibacterium sp. UTAS2018*	VanZ-2
WP_213518130.1	*Paenibacillus montaniterrae*	VanZ-2
GIE20058.1	*Winogradskya humida*	VanZ-2
MBR5272497.1	*Clostridia bacterium*	VanZ-2
MBR1749337.1	*Ruminococcus sp.*	VanZ-2
HBB87153.1	*Blastocatellia bacterium*	VanZ-2
MBD0858619.1	*Erysipelotrichaceae bacterium 7770_A6*	VanZ-2
HDP98332.1	*bacterium*	VanZ-2
MBI5965735.1	*Chloroflexota bacterium*	VanZ-2
MBP5550477.1	*Bacilli bacterium*	VanZ-2
MCA0345894.1	*Actinomycetota bacterium*	VanZ-2
MBP3595753.1	*Lachnospiraceae bacterium*	VanZ-2
MBR0419681.1	*Erysipelotrichaceae bacterium*	VanZ-2
OPZ33204.1	*Tenericutes bacterium ADurb.BinA124*	VanZ-2
MBR0461721.1	*Erysipelotrichaceae bacterium*	VanZ-2
MBS0460770.1	*Pseudomonadota bacterium*	VanZ-2
MBO4388722.1	*Spirochaetales bacterium*	VanZ-2
HGR87707.1		VanZ-2
MBI5352067.1	*Chloroflexota bacterium*	VanZ-2
MBI5840288.1	*Chloroflexota bacterium*	VanZ-2
TNE54718.1	*Bacteroidota bacterium*	VanZ-2
MBI3175870.1	*Chloroflexota bacterium*	VanZ-2
MBP6177026.1	*Anaerolineales bacterium*	VanZ-2
MBM3263106.1	*Candidatus Zixiibacteriota bacterium*	VanZ-2
MCC7394326.1	*Sphingomonadaceae bacterium*	VanZ-2
MBA3251121.1	*Geodermatophilaceae bacterium*	VanZ-2

WP_027860418.1
Marmoricola sp. URHB0036

MBA3417439.1
Geodermatophilaceae bacterium

MBE2185188.1
Rhodothermales bacterium

WP_030482908.1
Nocardioides aequoreus

MCA1710726.1
Actinomycetota bacterium

MBC7715928.1
Rhizobacter sp.

MBW6456087.1
Trueperaceae bacterium

WP_144395449.1
Pleionea sediminis

WP_130981999.1
Glaciihabitans arcticus

WP_007202760.1
Fictibacillus macauensis ZFHKF-1

MBR4444812.1
Solobacterium sp.

WP_101567373.1
Metabacillus idriensis

WP_117152512.1
Paraliobacillus quinghaiensis

MBQ4255202.1
Bacilli bacterium

WP_162909592.1
Aggregatilinea lenta

MBW6516218.1
Candidatus Cloacimonadota bacterium

MBN2685282.1
Pontiellaceae bacterium

HCE10822.1
Oxalobacteraceae bacterium

MCA9057615.1
Planctomycetaceae bacterium

WP_099326921.1
uncultured Candidatus Kuenenia sp.

WP_075091222.1
Planctomyces sp. SH-PL14

MCB1848634.1
Halieaceae bacterium

MBR3354720.1
Oscillospiraceae bacterium

MBP7355655.1
Longilinea sp.

WP_145172814.1
Gimesia aquarii

MBC8385234.1
Candidatus Cloacimonadota bacterium

RRN69854.1
Agrobacterium deltaense

WP_034210151.1
Arenimonas metalli CF5-1

NOZ28100.1
Chloroflexota bacterium

RMD67705.1
Candidatus Pacearchaeota archaeon

MBS9768337.1
Flavobacteriaceae bacterium

MBQ4562584.1
Clostridia bacterium

NUQ42922.1
Calditrichaceae bacterium

AJD89630.1
Jeotgalibacillus malaysiensis

MBR2240904.1
Clostridia bacterium

HAP03570.1
Lachnospiraceae bacterium

MBU1186165.1
Acidobacteriota bacterium

PWT92431.1
Blastocatellia bacterium

PKF59115.1
Alteromonadales bacterium alter-6D02

MBZ5498756.1
Terriglobia bacterium

NRB22716.1
Shewanella sp.

MAJ70163.1
Alteromonadaceae bacterium

MBD5494779.1
Lachnospiraceae bacterium

MBK7393186.1
Chloracidobacterium sp.

WP_119481881.1
Amnibacterium setariae

MBQ8996914.1
Candidatus Saccharibacteria bacterium

MBA4176882.1
Leptothrix sp. (in: b-proteobacteria)

MBP1623334.1
Acidobacteriota bacterium

ERL53347.1
Halomonas huangheensis

ONI60555.1
Leifsonia sp. ALI-44-B

OUS11610.1
Gammaproteobacteria bacterium 53_120_T64

MBN2423398.1
Calditrichaceae bacterium

MBI3651295.1
Acidobacteriota bacterium

OIQ47455.1
Gammaproteobacteria bacterium MedPE

MBA3767858.1
Acidobacteriota bacterium

MBI1184582.1
bacterium

MBX7105633.1
Gemmataceae bacterium

MCB0284475.1
Calditrichota bacterium

WP_089400597.1
Noviherbaspirillum humi

MBL1135257.1
Chloroflexota bacterium

MBL8023769.1
Elusimicrobiota bacterium

MBT4867015.1
Planctomycetaceae bacterium

WP_183167680.1

MBI4116744.1
Candidatus Pacearchaeota archaeon

CDZ24476.1
[Clostridium] cellulosi

NQX88996.1
Halioglobus sp.

MBV7328698.1
Chloroflexi bacterium TSY

MBD3261343.1
Candidatus Altiarchaeales archaeon

MBX3625020.1
Rhizobacter sp.

NCA80485.1
Sphingobacteriia bacterium

PKB57860.1
SAR202 cluster bacterium Casp-Chloro-G3

CAA0080757.1
BD1-7 clade bacterium

MCA9245572.1
Planctomycetales bacterium

MCA9269013.1
Planctomycetales bacterium

MBQ4465755.1
Oscillospiraceae bacterium

KZD16979.1
Candidatus Fermentibacter danicus

MBN1435108.1
Candidatus Fermentibacterales bacterium

MBR0343425.1
Oscillospiraceae bacterium

PCJ63668.1
Candidatus Hydrogenedentota bacterium

HEK52577.1

WP_009623273.1
Desulfosporosinus sp. OT

MBQ6846852.1
Oscillospiraceae bacterium

MBC7900311.1
Pyrinomonadaceae bacterium

HGM87377.1

MBK8068793.1
Rhodanobacteraceae bacterium

MBK6436464.1
Rhodanobacteraceae bacterium

WP_181841208.1
Emticicia sp. BO119

WP_141006169.1
Nocardioides humi

MBI4530187.1
Candidatus Latescibacterota bacterium

MBL4763005.1
Gammaproteobacteria bacterium

MBD3333779.1
candidate division GN15 bacterium

MBQ9850792.1
Clostridia bacterium

MBL7136722.1
Fidelibacterota bacterium

MBC7542216.1	*Candidatus Sericytochromatia bacterium*	VanZ-2
MBY8991814.1	*Promethearchaeota archaeon*	VanZ-2
TMG84861.1	*Betaproteobacteria bacterium*	VanZ-2
MBP8251710.1	*Herpetosiphon sp.*	VanZ-2
WP_141017487.1	*Azoarcus sp. DD4*	VanZ-2
MBV7435286.1	*Cardiobacteriaceae bacterium TAE3-ERU3*	VanZ-2
MBF8269681.1	*Gammaproteobacteria bacterium*	VanZ-2
WP_140959732.1	*Aestuariibacter sp. GS-14*	VanZ-2
QAY95397.1	*Methylovirgula ligni*	VanZ-2
AER54711.1	*Pseudoxanthomonas spadix BD-a59*	VanZ-2
WP_162350007.1	*Pseudoxanthomonas gei*	VanZ-2
MBQ6529061.1	*Clostridia bacterium*	VanZ-2
WP_183640449.1	*Pseudoxanthomonas sp. PXM05*	VanZ-2
MBO6297619.1	*Lachnospiraceae bacterium*	VanZ-2
WP_066462633.1	*Caryophanon latum*	VanZ-2
MBQ9413927.1	*Clostridia bacterium*	VanZ-2
PIS37886.1	*Nitrospirae bacterium CG08_land_8_20_14_0_20_52_24*	VanZ-2
MCC6544008.1	*Nitrospirota bacterium*	VanZ-2
MBU6480832.1	*Nitrospirota bacterium*	VanZ-2
HBE73406.1	*Candidatus Zixiibacteriota bacterium*	VanZ-2
WP_232307160.1		VanZ-2
WP_028401764.1	*Ectobacillus panaciterrae DSM 19096*	VanZ-2
UCD95124.1	*Candidatus Zixiibacteriota bacterium*	VanZ-2
MBQ2399183.1	*Muribaculaceae bacterium*	VanZ-2
MBS4042900.1	*Chitinophagaceae bacterium*	VanZ-2
MCD6150148.1	*Candidatus Tharpellaceae bacterium*	VanZ-2
MCD6117766.1	*bacterium*	VanZ-2
MBP3459923.1	*Lachnospiraceae bacterium*	VanZ-2
WP_204114937.1	*Shimia biformata*	VanZ-2
MBU0894477.1	*Nanoarchaeota archaeon*	VanZ-2
MBP3535789.1	*Muribaculaceae bacterium*	VanZ-2
MBI1727695.1	*Candidatus Rokuibacteriota bacterium*	VanZ-2
MBS7354259.1	*Erysipelotrichaceae bacterium*	VanZ-2
ROT05425.1	*Muribaculaceae bacterium Isolate-037 (Harlan)*	VanZ-2
MCB1684894.1	*Pseudomonadales bacterium*	VanZ-2
PYM49608.1	*Candidatus Rokuibacteriota bacterium*	VanZ-2
MBT0748838.1		VanZ-2
TET87468.1	*Sulfurovum sp.*	VanZ-2
HAI84711.1	*Chitinophagaceae bacterium*	VanZ-2
MBE6695847.1	*Oscillospiraceae bacterium*	VanZ-2
UCD35107.1	*Nitrospiraceae bacterium*	VanZ-2
RJQ43301.1	*Nitrospiraceae bacterium*	VanZ-2
WP_075706530.1	*Vibrio panuliri*	VanZ-2
MCC8174523.1	*Odoribacter sp.*	VanZ-2
AJS61540.1	*Paenibacillus sp. IHBB 10380*	VanZ-2
KIT56577.1	*Vibrio parahaemolyticus EN9701072*	VanZ-2
WP_013554051.1	*Nitratifractor salsuginis DSM 16511*	VanZ-2
WP_120031423.1		VanZ-2
HAS63618.1	*Vibrio sp.*	VanZ-2
MBQ7631198.1	*Paludibacteraceae bacterium*	VanZ-2
SIO59342.1	*Singulisphaera sp. GP187*	VanZ-2
QQR78118.1	*Candidatus Moraniibacteriota bacterium*	VanZ-2
WP_194436496.1	*Vibrio fluminensis*	VanZ-2
MBW8054041.1	*Nitrospira sp.*	VanZ-2
MBK1668321.1	*Rhodovibrio sodomensis*	VanZ-2
MCD6594176.1	*bacterium*	VanZ-2
WP_079557042.1	*Alkalitalea saponilacus*	VanZ-2
MBN1226905.1	*Deltaproteobacteria bacterium*	VanZ-2
MBI2361772.1	*Elusimicrobiota bacterium*	VanZ-2
NOS80709.1	*Nitrospira sp.*	VanZ-2
HAQ63832.1	*Oscillospiraceae bacterium*	VanZ-2
RGU59640.1	*Paraprevotella clara*	VanZ-2
TAK39034.1	*Lysobacteraceae bacterium*	VanZ-2
NLZ97184.1	*Candidatus Magasanikiibacteriota bacterium*	VanZ-2
MBI4832086.1	*Candidatus Lindowiibacteriota bacterium*	VanZ-2
TFH02726.1	*Calditrichales bacterium*	VanZ-2
NLO79079.1	*Lysobacteraceae bacterium*	VanZ-2
MCC6624773.1	*Deltaproteobacteria bacterium*	VanZ-2
MBL0135386.1	*Chitinophagaceae bacterium*	VanZ-2
WP_068025636.1	*Rhodoplanes sp. Z2-YC6860*	VanZ-2
HIC93986.1	*Anaerolineae bacterium*	VanZ-2
MBU6294875.1	*Planctomycetota bacterium*	VanZ-2
WP_078736395.1	*Porphyromonas circumdentaria*	VanZ-2
MBD3367802.1	*Candidatus Effluviviaceae Genus V sp.*	VanZ-2
WP_170266559.1	*Brevifollis gellanilyticus*	VanZ-2
MBL0709604.1	*Colwellia sp.*	VanZ-2
OAH08894.1	*Rhodobacteraceae bacterium SB2*	VanZ-2
MCB1568522.1	*Lysobacterales bacterium*	VanZ-2
WP_193214645.1	*Luteolibacter marinus*	VanZ-2
WP_204094596.1	*Amycolatopsis sp. FDAARGOS 1241*	VanZ-2
MBW2205891.1	*Deltaproteobacteria bacterium*	VanZ-2
PIU51913.1	*Candidatus Desantisbacteria bacterium CG07_land_8_20_14_0_80_39_15*	VanZ-2
GBE52928.1	*bacterium BMS3Bbin14*	VanZ-2
MBN1640731.1	*Anaerolineae bacterium*	VanZ-2
MBW2406616.1	*Deltaproteobacteria bacterium*	VanZ-2
MBQ8301777.1	*Clostridia bacterium*	VanZ-2
MBP3849774.1	*Prevotella sp.*	VanZ-2
MBU3819207.1	*Candidatus Faecalibacterium intestinavium*	VanZ-2
MBQ5656111.1	*Bacteroidaceae bacterium*	VanZ-2
NDD10666.1	*Paracoccaceae bacterium*	VanZ-2
PLY06430.1	*Arcobacter sp.*	VanZ-2
MBQ4115982.1	*Clostridia bacterium*	VanZ-2
WP_087148621.1	*Crenothrix polyspora*	VanZ-2

MBS3740056.1	*Candidatus Bipolaricaulota bacterium*	VanZ
MBR1799886.1	*Bacteroidaceae bacterium*	VanZ
HCY41546.1	*Prolixibacteraceae bacterium*	VanZ
OGP59587.1	*Deltaproteobacteria bacterium RBG_13_49_15*	VanZ
SDN31416.1	*Alkalicoccus daliensis*	VanZ
MBE2215843.1	*Opitutaceae bacterium*	VanZ
MBT5739059.1	*Planctomycetota bacterium*	VanZ
MBX3037740.1	*Anaerolineales bacterium*	VanZ
MBA2428219.1	*Thermoleophilaceae bacterium*	VanZ
WP_088865294.1	*Thermococcus barossii*	VanZ
UCC89248.1	*Anaerolineales bacterium*	VanZ
MCA9981583.1	*Anaerolineales bacterium*	VanZ
MBQ4095132.1	*Oscillospiraceae bacterium*	VanZ
MCB0077771.1	*Anaerolineales bacterium*	VanZ
MCC6259883.1	*Anaerolineales bacterium*	VanZ
TML20485.1	*Actinomycetota bacterium*	VanZ
MCC8088172.1	*Rikenellaceae bacterium*	VanZ
MCB8976133.1	*Ardenticatenaceae bacterium*	VanZ
NOR84107.1	*Ardenticatenales bacterium*	VanZ
MBD3365952.1	*candidate division WWE3 bacterium*	VanZ
MCD7962567.1	*Rikenellaceae bacterium*	VanZ
MBN1273967.1	*Candidatus Aminicenantes bacterium*	VanZ
MCB2194468.1	*Bacteroidota bacterium*	VanZ
MCB0738647.1	*Bacteroidota bacterium*	VanZ
MBP3786176.1	*Bacteroidaceae bacterium*	VanZ
HEY54392.1	*Caldilineae bacterium*	VanZ
OPY00671.1	*Syntrophorhabdus sp. PtaB.Bin047*	VanZ
OPY70243.1	*Syntrophorhabdaceae bacterium PtaU1.Bin034*	VanZ
MBW7888294.1	*Bacteroidota bacterium*	VanZ
TET25924.1	*Candidatus Aminicenantes bacterium*	VanZ
VVB78770.1	*uncultured archaeon*	VanZ
MBN8458502.1	*Verrucomicrobiota bacterium*	VanZ
HHP71090.1		VanZ
MBD5189393.1	*Bacteroidales bacterium*	VanZ
HIY21107.1	*Candidatus Flavonifractor merdigallinarum*	VanZ
MBQ9300867.1	*Clostridia bacterium*	VanZ
PBH77411.1	*Clostridioides difficile*	VanZ
MBP1679263.1	*Bacteroidota bacterium*	VanZ
HGI22077.1		VanZ
MBA2751266.1	*Actinomycetota bacterium*	VanZ
WP_205228645.1	*Desulfobulbus rhabdoformis*	VanZ
MBN1181785.1	*Bacteroidales bacterium*	VanZ
MBL7825229.1	*Saprospiraceae bacterium*	VanZ
MBF0264069.1	*Gammaproteobacteria bacterium*	VanZ
MBI4340761.1	*Candidatus Omnitrophota bacterium*	VanZ
RLC75846.1	*Chloroflexota bacterium*	VanZ
WP_205758737.1	*Lysobacter sp. HDW10*	VanZ
HIE48965.1	*Gammaproteobacteria bacterium*	VanZ
MBN1182150.1	*Bacteroidales bacterium*	VanZ
MBN1143132.1	*Bacteroidales bacterium*	VanZ
MBN1988188.1	*Bacteroidales bacterium*	VanZ
MBE6719458.1	*Oscillospiraceae bacterium*	VanZ
MBN2482178.1	*Bacteroidales bacterium*	VanZ
MBO4674490.1	*Elusimicrobiaceae bacterium*	VanZ
MBN2764475.1	*Bacteroidales bacterium*	VanZ
MCB1226557.1	*Verrucomicrobiales bacterium*	VanZ
MAU59437.1	*Parvibaculum sp.*	VanZ
OYY07820.1	*Sphingobacteriia bacterium 35-36-14*	VanZ
NIK13133.1	*Alkalibacillus almallahensis*	VanZ
TAD91333.1	*Bacteroidota bacterium*	VanZ
MBC7492694.1	*Novosphingobium sp.*	VanZ
WP_169801141.1	*Halalkalibacter krulwichiae*	VanZ
MCC7196208.1	*Gemmatimonadaceae bacterium*	VanZ
HAL56733.1	*Bacteroidota bacterium*	VanZ
MBE9504113.1	*Pseudomonadota bacterium*	VanZ
MBE9537195.1	*Pseudomonadota bacterium*	VanZ
MBI2165137.1	*Chloroflexota bacterium*	VanZ
MBN2874291.1	*Spirochaetales bacterium*	VanZ
MXZ86367.1	*Acidimicrobiia bacterium*	VanZ
MBQ3707949.1	*Clostridia bacterium*	VanZ
WP_231705784.1	*Effusibacillus lacus*	VanZ
HEC44182.1	*Bacteroides sp.*	VanZ
WP_162937331.1	*Indioceanicola profundi*	VanZ
MBO7214802.1	*Clostridia bacterium*	VanZ
HBA35536.1	*Gammaproteobacteria bacterium*	VanZ
MBI5350245.1	*Chloroflexota bacterium*	VanZ
WP_099615785.1	*Marinobacter sp.*	VanZ
SKC07403.1	*Lachnospiraceae bacterium*	VanZ
MBQ6000542.1	*Clostridia bacterium*	VanZ
WP_074791180.1	*Haloferax larsenii*	VanZ
MBA4311289.1	*Chlorobiaceae bacterium*	VanZ
MBD3241636.1	*Chitinivibrionales bacterium*	VanZ
MBU0581278.1	*Candidatus Margulisiibacteriota bacterium*	VanZ
MBO9368144.1	*Chloroflexota bacterium*	VanZ
HIF23445.1	*Gemmatimonadota bacterium*	VanZ
WP_089736205.1	*Haloarchaeobius iranensis*	VanZ
HGB40811.1		VanZ
MBX9690621.1	*Candidatus Obscuribacterales bacterium*	VanZ
HAZ49425.1	*Cyanobacteria bacterium UBA11371*	VanZ
MBX3132949.1	*Gemmatimonadaceae bacterium*	VanZ
OGN83430.1	*Chloroflexi bacterium GWC2_73_18*	VanZ
MBX7119060.1	*Gemmatimonadaceae bacterium*	VanZ
MBX2812924.1	*Myxococcales bacterium*	VanZ

OUV60145.1	*Candidatus Pelagibacter sp. TMED128*	VanZ-2
MBA3852871.1	*Gemmatimonas sp.*	VanZ-2
WP_128965139.1	*Bradyrhizobium guangdongense*	VanZ-2
NEQ53681.1	*Leptolyngbya sp. SIO3F4*	VanZ-2
EGQ40626.1	*Candidatus Nanosalinarum sp. J07AB56*	VanZ-2
MBX3172834.1	*Gemmatimonadaceae bacterium*	VanZ-2
MBA2452804.1	*Chloroflexia bacterium*	VanZ-2
HGZ22787.1		VanZ-2
MBN1982887.1	*Chitinivibrionales bacterium*	VanZ-2
AYM52942.1		VanZ-2
OPY19242.1	*Syntrophus sp. PtaB.Bin075*	VanZ-2
MBL0332363.1	*Chlorobiota bacterium*	VanZ-2
NPA36490.1	*Chlorobiota bacterium*	VanZ-2
OGU15811.1	*Geobacteraceae bacterium GWC2_53_11*	VanZ-2
MCA9621845.1	*Myxococcales bacterium*	VanZ-2
PLY12322.1	*Desulfuromonas sp.*	VanZ-2
MBR2695590.1	*Candidatus Saccharibacteria bacterium*	VanZ-2
CDF22068.1	*Prevotella sp. CAG:617*	VanZ-2
WP_049968052.1	*Haloferax sp. Atlit-19N*	VanZ-2
WP_215937627.1	*Winogradskyella psychrotolerans*	VanZ-2
MCB9715801.1	*Myxococcales bacterium*	VanZ-2
WP_106524745.1	*Taibaiella chishuiensis*	VanZ-2
PYK10973.1	*Verrucomicrobiota bacterium*	VanZ-2
WP_004057880.1	*Haloferax mediterranei ATCC 33500*	VanZ-2
MBE0597061.1	*Desulfuromonadales bacterium*	VanZ-2
WP_130390255.1	*Cupriavidus agavae*	VanZ-2
MBI4770163.1	*Chloroflexota bacterium*	VanZ-2
WP_183349443.1	*Desulfoprunum benzoelyticum*	VanZ-2
MBK9266601.1	*Polyangiaceae bacterium*	VanZ-2
MBE6593873.1	*Oscillospiraceae bacterium*	VanZ-2
WP_162361064.1		VanZ-2
MBS3803754.1	*Oleiphilaceae bacterium*	VanZ-2
MCC6477181.1	*bacterium*	VanZ-2
WP_141622218.1	*Marilutibacter alkalisoli*	VanZ-2
MCE2659873.1	*Rubrivivax sp.*	VanZ-2
OQX63024.1	*Desulfococcus sp. 4484_241*	VanZ-2
MBX7190410.1	*Sandaracinaceae bacterium*	VanZ-2
MBO5009958.1	*Clostridia bacterium*	VanZ-2
MBP3574713.1	*Prevotella sp.*	VanZ-2
WP_087196849.1	*Rubneribacter badeniensis*	VanZ-2
MBR6998457.1	*Prevotella sp.*	VanZ-2
MYI83016.1	*Chloroflexota bacterium*	VanZ-2
WP_119117599.1	*Peribacillus asahii*	VanZ-2
WP_090764957.1	*Bacillus sp. OK048*	VanZ-2
NTU50198.1	*Desulfobulbaceae bacterium*	VanZ-2
MCA9483980.1	*Nitrospina sp.*	VanZ-2
PLX49710.1	*Desulfobulbaceae bacterium*	VanZ-2
HCC52221.1	*Porphyromonadaceae bacterium*	VanZ-2
PZC49395.1	*Chloroflexota bacterium*	VanZ-2
OGW18066.1	*Nitrospinae bacterium RIFCSPLOWO2_12_FULL_45_22*	VanZ-2
PHS19811.1	*Kangiella sp.*	VanZ-2
MCD4690830.1	*bacterium*	VanZ-2
MBI9085381.1	*Desulfobacterales bacterium*	VanZ-2
MBH39453.1	*Chloroflexota bacterium*	VanZ-2
MBA31335.1	*Chloroflexota bacterium*	VanZ-2
MBC7364338.1	*Candidatus Aminicenantes bacterium*	VanZ-2
MBS7377528.1	*Paludibacteraceae bacterium*	VanZ-2
MBP3587196.1	*Paludibacteraceae bacterium*	VanZ-2
MCA9928177.1	*Anaerolineales bacterium*	VanZ-2
MBP5453080.1	*Lachnospiraceae bacterium*	VanZ-2
MBN1318394.1	*Anaerolineales bacterium*	VanZ-2
MBR1878912.1	*Paludibacteraceae bacterium*	VanZ-2
MBK8616347.1	*Candidatus Villigracilis proximus*	VanZ-2
MBF8256112.1	*Anaerolineales bacterium*	VanZ-2
NIM92547.1	*Anaerolineales bacterium*	VanZ-2
MBX9654276.1	*bacterium*	VanZ-2
MBW2601882.1	*Deltaproteobacteria bacterium*	VanZ-2
MBW2238118.1	*Deltaproteobacteria bacterium*	VanZ-2
MBW2221901.1	*Deltaproteobacteria bacterium*	VanZ-2
PIU51614.1	*Candidatus Desantisbacteria bacterium CG07_land_8_20_14_0_80_39_15*	VanZ-2
MBR2121403.1	*Lachnospiraceae bacterium*	VanZ-2
MBI4928456.1	*Anaerolineae bacterium*	VanZ-2
TMQ50530.1	*Candidatus Eiseniibacteriota bacterium*	VanZ-2
MAZ34142.1	*Thalassospira sp.*	VanZ-2
MBP7862925.1	*bacterium*	VanZ-2
MBP7669127.1	*Candidatus Eiseniibacteriota bacterium*	VanZ-2
MBP1724907.1	*Deltaproteobacteria bacterium*	VanZ-2
WP_160912589.1	*Halobacillus litoralis*	VanZ-2
MBN2803578.1	*Deltaproteobacteria bacterium*	VanZ-2
MBS1242367.1	*Nitrospirota bacterium*	VanZ-2
GBE17712.1	*archaeon BMS3Abin16*	VanZ-2
MBQ4511723.1	*Anaerolineaceae bacterium*	VanZ-2
TFF94100.1	*Promethearchaeota archaeon*	VanZ-2
PLX13588.1	*Marinilabiliales bacterium*	VanZ-2
HCP08656.1	*Candidatus Moraniibacteriota bacterium*	VanZ-2
UCE63958.1	*Nitrospirota bacterium*	VanZ-2
HFC76640.1	*Candidatus Moraniibacteriota bacterium*	VanZ-2
HBG86311.1	*Marinilabiliaceae bacterium*	VanZ-2
SOD55486.1	*Pseudoxanthomonas wuyuanensis*	VanZ-2
MBN1214039.1	*Promethearchaeota archaeon*	VanZ-2
NTW46133.1	*Candidatus Moraniibacteriota bacterium*	VanZ-2
KAB2336741.1	*Cytobacillus depressus*	VanZ-2
MBK7142409.1	*bacterium*	VanZ-2

NTV33234.1	*Deltaproteobacteria bacterium*	VanZ-2
MBU1999279.1	*Candidatus Omnitrophota bacterium*	VanZ-2
MBU4376570.1	*Candidatus Omnitrophota bacterium*	VanZ-2
MBL8508564.1	*Chitinimonas sp.*	VanZ-2
HIQ79774.1	*Candidatus Scatavimonas merdigallinarum*	VanZ-2
MCC3222564.1		VanZ-2
NCO89268.1	*Candidatus Roizmanbacteria bacterium*	VanZ-2
HDZ90405.1	*Deltaproteobacteria bacterium*	VanZ-2
RLB89282.1	*Deltaproteobacteria bacterium*	VanZ-2
RLB84158.1	*Deltaproteobacteria bacterium*	VanZ-2
MBQ9045314.1	*Oscillospiraceae bacterium*	VanZ-2
RLB54552.1	*Deltaproteobacteria bacterium*	VanZ-2
MBO5797608.1	*Clostridia bacterium*	VanZ-2
MCD6291955.1	*Candidatus Tharpella sp.*	VanZ-2
WP_066060476.1	*Candidatus Desulfofervidus auxilii*	VanZ-2
KKO20438.1	*Candidatus Brocadia fulgida*	VanZ-2
RZJ64010.1	*Flavobacterium sp.*	VanZ-2
OGJ22513.1	*Candidatus Pacebacteria bacterium RIFCSPHIGHO2_01_FULL_46_10*	VanZ-2
MBN3038311.1	*Candidatus Omnitrophota bacterium*	VanZ-2
MBO5379994.1	*Clostridia bacterium*	VanZ-2
MBY0528463.1	*Gemmataceae bacterium*	VanZ-2
MBQ6980537.1	*Clostridia bacterium*	VanZ-2
HEB56388.1	*Gammaproteobacteria bacterium*	VanZ-2
HGZ84502.1		VanZ-2
MBI1388069.1	*bacterium*	VanZ-2
EDP69871.1	*Flavobacteriales bacterium ALC-1*	VanZ-2
MBL7058285.1	*Patescibacteria group bacterium*	VanZ-2
MBK9021077.1	*Sulfuritalea sp.*	VanZ-2
MBU4142609.1	*Patescibacteria group bacterium*	VanZ-2
SDO03189.1	*Halobacillus aidingensis*	VanZ-2
MBO5099388.1	*Clostridia bacterium*	VanZ-2
MBL8328200.1	*Rubrivivax sp.*	VanZ-2
MCD7806910.1	*Lachnospiraceae bacterium*	VanZ-2
MCA9731091.1	*Candidatus Zhuqueibacterota bacterium*	VanZ-2
HAU59315.1	*Opitutia bacterium*	VanZ-2
MBR6766321.1	*Clostridia bacterium*	VanZ-2
MBZ0073042.1	*Gammaproteobacteria bacterium*	VanZ-2
NLN50657.1	*Acholeplasmataceae bacterium*	VanZ-2
MBR3969995.1	*Ruminococcus sp.*	VanZ-2
MCE3262796.1	*Pseudoduganella sp.*	VanZ-2
MBQ6366078.1	*Oscillospiraceae bacterium*	VanZ-2
PYX93746.1	*Acidobacteriota bacterium*	VanZ-2
MBQ7259960.1	*Lachnospiraceae bacterium*	VanZ-2
WP_119771213.1	*Noviherbaspirillum saxi*	VanZ-2
MBA2302014.1	*Acidobacteriota bacterium*	VanZ-2
HGW00168.1		VanZ-2
HGS53195.1		VanZ-2
SFE12575.1	*Marinobacter sp. DSM 26671*	VanZ-2
WP_100813267.1	*Microbacterium lacus*	VanZ-2
WP_068418850.1	*Planctomyces sp. SH-PL62*	VanZ-2
MBR3696656.1	*Clostridia bacterium*	VanZ-2
WP_214448787.1	*Flavihumibacter rivuli*	VanZ-2
WP_099917056.1	*Massilia psychrophila*	VanZ-2
RJX20519.1	*Desulfobulbus sp.*	VanZ-2
MBI2973106.1	*Armatimonadota bacterium*	VanZ-2
HHS61073.1		VanZ-2
BCS34148.1	*Luteitalea sp. TBR-22*	VanZ-2
MBN8444119.1	*Gammaproteobacteria bacterium*	VanZ-2
MBI4660044.1	*Verrucomicrobiota bacterium*	VanZ-2
MBN1408142.1	*Calditrichaceae bacterium*	VanZ-2
MBR2020221.1	*Clostridia bacterium*	VanZ-2
MBL8303097.1	*Ideonella sp.*	VanZ-2
RKY25182.1	*Planctomycetota bacterium*	VanZ-2
WP_015756150.1		VanZ-2
HGW78030.1		VanZ-2
MBN2444239.1	*Spirochaetales bacterium*	VanZ-2
WP_088853283.1	*Thermococcus pacificus*	VanZ-2
RMF74578.1	*Planctomycetota bacterium*	VanZ-2
WP_143579972.1	*Thalassotalea sp. PS06*	VanZ-2
MBC7645598.1	*Deinococcales bacterium*	VanZ-2
MBD3265827.1	*bacterium*	VanZ-2
WP_027022542.1	*Conchiformibius steedae DSM 2580*	VanZ-2
MBI3188522.1	*Gammaproteobacteria bacterium*	VanZ-2
WP_119154851.1	*Caldimonas tepidiphila*	VanZ-2
MBL8732623.1	*Planctomycetota bacterium*	VanZ-2
MBM3960862.1	*Planctomycetota bacterium*	VanZ-2
NDB95943.1	*Verrucomicrobiota bacterium*	VanZ-2
MBI9014092.1	*Clostridiales bacterium*	VanZ-2
VFK24847.1		VanZ-2
TMH18462.1	*Betaproteobacteria bacterium*	VanZ-2
NBQ33980.1	*Gammaproteobacteria bacterium*	VanZ-2
HEJ72360.1		VanZ-2
MBU6174282.1	*Planctomycetota bacterium*	VanZ-2
MBS1271599.1		VanZ-2
NOZ52889.1	*Gammaproteobacteria bacterium*	VanZ-2
HCL05798.1	*Chitinophagaceae bacterium*	VanZ-2
WP_007606700.1	*Bradyrhizobium sp. WSM471*	VanZ-2
NQU10004.1	*bacterium*	VanZ-2
HIZ06028.1	*Candidatus Phocaeicola gallistercoris*	VanZ-2
NLI16722.1	*Candidatus Zixiibacteriota bacterium*	VanZ-2
MBT33478.1	*Thalassovita sp.*	VanZ-2
WP_068713919.1	*Vibrio tritonius*	VanZ-2
MBQ4204583.1	*Thermoguttaceae bacterium*	VanZ-2

EDQ03228.1	*Sulfitobacter indolifex HEL-45*	VanZ-2
MBL4907814.1	*Sneathiella sp.*	VanZ-2
MBJ7258927.1	*Chthoniobacterales bacterium*	VanZ-2
NRB70831.1	*Lysobacterales bacterium*	VanZ-2
HIC34536.1	*Gammaproteobacteria bacterium*	VanZ-2
MBM6861153.1	*Clostridium saudiense*	VanZ-2
MBP6649370.1	*Bacteroidia bacterium*	VanZ-2
MBD3168088.1	*Candidatus Zixiibacteriota bacterium*	VanZ-2
MCB1607139.1	*Lysobacterales bacterium*	VanZ-2
WP_151736527.1	*Paenibacillus tengchongensis*	VanZ-2
NCC98238.1	*Bacteroidia bacterium*	VanZ-2
KPL04420.1	*candidate division Zixibacteria bacterium SM23_73_2*	VanZ-2
WP_152335399.1	*Salipiger sp. HF18*	VanZ-2
PKP22905.1	*Bacteroidetes bacterium HGW-Bacteroidetes-21*	VanZ-2
WP_121093163.1	*Roseinatronobacter ekhonensis*	VanZ-2
MBL4853690.1	*Robiginitomaculum sp.*	VanZ-2
OYT15627.1	*Bacteroidetes bacterium 4572_77*	VanZ-2
MBA11115.1	*Gammaproteobacteria bacterium*	VanZ-2
HEB92646.1	*Gammaproteobacteria bacterium*	VanZ-2
QGK70813.1	*Allosaccharopolyspora coralli*	VanZ-2
MBX2992719.1	*Bacteroidota bacterium*	VanZ-2
MBN2851683.1	*Clostridia bacterium*	VanZ-2
PYJ93016.1	*Verrucomicrobiota bacterium*	VanZ-2
TAG07873.1	*Verrucomicrobiota bacterium*	VanZ-2
MBQ6750139.1	*Bacteroidaceae bacterium*	VanZ-2
KAB2647126.1	*Verrucomicrobiota bacterium*	VanZ-2
MBU0764320.1	*Bacteroidota bacterium*	VanZ-2
MBI5692285.1	*Verrucomicrobiota bacterium*	VanZ-2
MBT6166532.1	*Verrucomicrobiota bacterium*	VanZ-2
MBU1693541.1	*Verrucomicrobiota bacterium*	VanZ-2
MBR2154162.1	*Bacteroidaceae bacterium*	VanZ-2
MBO5275459.1	*Clostridia bacterium*	VanZ-2
MBM2845635.1	*Bacteroidota bacterium*	VanZ-2
OED44321.1	*Endozoicomonas sp. (ex Bugula neritina AB1)*	VanZ-2
HCL67437.1	*Rhizobium sp.*	VanZ-2
TET54720.1	*Anaerolineales bacterium*	VanZ-2
NLB03381.1	*Bacteroidales bacterium*	VanZ-2
NLI87531.1	*Bacteroidales bacterium*	VanZ-2
KAB2926113.1	*Bacteroidota bacterium*	VanZ-2
MBR0384176.1	*Eubacteriaceae bacterium*	VanZ-2
GFZ33095.1	*Clostridium zeae*	VanZ-2
NLK15185.1	*Bacteroidales bacterium*	VanZ-2
MBL9126605.1	*Verrucomicrobiales bacterium*	VanZ-2
MBQ8353407.1	*Spirochaetaceae bacterium*	VanZ-2
WP_062261902.1	*Endozoicomonas arenosclerae*	VanZ-2
RMF17409.1	*Gammaproteobacteria bacterium*	VanZ-2
PYN12492.1	*Candidatus Rokuibacteriota bacterium*	VanZ-2
NLY07752.1	*Spirochaetales bacterium*	VanZ-2
MBD5231883.1	*Bacteroidales bacterium*	VanZ-2
NLT93169.1	*Actinomycetota bacterium*	VanZ-2
MBN2519122.1	*Bacteroidales bacterium*	VanZ-2
KOG48037.1	*Streptomyces varsoviensis*	VanZ-2
MBU1053597.1	*Pseudomonadota bacterium*	VanZ-2
MCD6332347.1	*Bacteroidales bacterium*	VanZ-2
HBS27776.1	*Gammaproteobacteria bacterium*	VanZ-2
MCD6347301.1	*Bacteroidales bacterium*	VanZ-2
PSP47570.1	*Halobacteriales archaeon QH_7_69_31*	VanZ-2
WP_074908783.1	*Proteiniclasticum ruminis*	VanZ-2
MBP0013369.1	*Roseofilum sp. SID3*	VanZ-2
MBN8936626.1	*Hyphomicrobiales bacterium*	VanZ-2
MBS3807089.1	*Bacteroidales bacterium*	VanZ-2
NER37302.1	*Oscillatoria sp. SIO1A7*	VanZ-2
MBW4678949.1	*Microcoleus vaginatus WJT46-NPBG5*	VanZ-2
WP_074119769.1	*Bradyrhizobium sp. AS23.2*	VanZ-2
SIO46712.1	*Bradyrhizobium erythrophlei*	VanZ-2
MQY56733.1	*bacterium*	VanZ-2
TSA20533.1	*bacterium*	VanZ-2
SEM37613.1	*Bosea lupini*	VanZ-2
MBQ6836011.1	*Clostridia bacterium*	VanZ-2
MBU6452063.1	*Cyanobacteria bacterium REEB67*	VanZ-2
MBE0574469.1	*Desulfuromonadales bacterium*	VanZ-2
WP_075597011.1	*Leptolyngbya sp. 'hensonii'*	VanZ-2
NJP09032.1	*Leptolyngbyaceae cyanobacterium RU_5_1*	VanZ-2
WP_106338260.1	*Alsobacter soli*	VanZ-2
TKX42552.1	*Halorubrum sp. ARQ200*	VanZ-2
TFG54554.1	*Hyphomicrobiales bacterium*	VanZ-2
MBW6463079.1	*Bacillota bacterium*	VanZ-2
MBE6014419.1	*Lachnospiraceae bacterium*	VanZ-2
MBR9825140.1	*Alphaproteobacteria bacterium*	VanZ-2
MBI1329046.1	*Alphaproteobacteria bacterium*	VanZ-2
MBA2589912.1	*Alphaproteobacteria bacterium*	VanZ-2
MBD5403962.1	*Treponema sp.*	VanZ-2
MBU5452460.1	*Pseudoflavonifractor sp. MSJ-30*	VanZ-2
MBF0358995.1	*Magnetococcales bacterium*	VanZ-2
MBQ9848190.1	*Clostridia bacterium*	VanZ-2
MBE9100169.1	*filamentous cyanobacterium LEGE 07170*	VanZ-2
WP_006020132.1	*Afipia broomeae ATCC 49717*	VanZ-2
MCB8965208.1	*Bacteroidales bacterium*	VanZ-2
GJD77926.1	*Methylobacterium gregans*	VanZ-2
MBU4264051.1	*Pseudomonadota bacterium*	VanZ-2
WP_195780930.1	*Priestia megaterium*	VanZ-2
MCD8386723.1	*Bacteroidales bacterium*	VanZ-2
KXB48550.1	*Bacteroidales bacterium KA00344*	VanZ-2

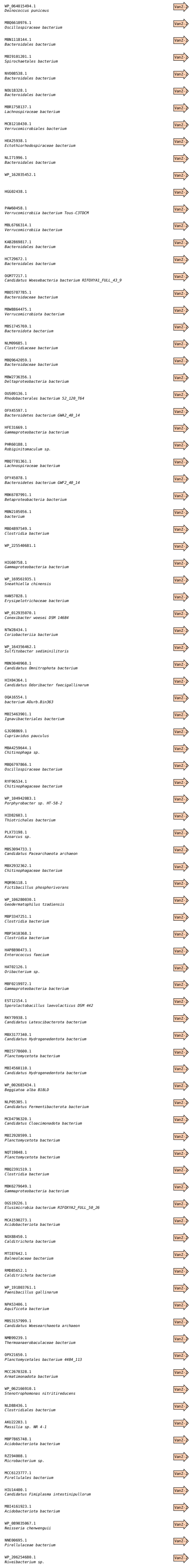

WP_064015494.1
Deinococcus puniceus
VanZ-2
MBQ6610976.1
Oscillospiraceae bacterium
VanZ-2
MBN1118144.1
Bacteroidales bacterium
VanZ-2
MBI9101201.1
Spirochaetales bacterium
VanZ-2
NVO08538.1
Bacteroidales bacterium
VanZ-2
NOU18328.1
Bacteroidales bacterium
VanZ-2
MBR1758137.1
Lachnospiraceae bacterium
VanZ-2
MCB1210430.1
Verrucomicrobiales bacterium
VanZ-2
HEA25938.1
Ectothiorhodospiraceae bacterium
VanZ-2
NLI71996.1
Bacteroidales bacterium
VanZ-2
WP_162035452.1
VanZ-2
HGG02438.1
VanZ-2
PAW60458.1
Verrucomicrobiia bacterium Tous-C3TDCM
VanZ-2
MBL6766314.1
Verrucomicrobiia bacterium
VanZ-2
KAB2869817.1
Bacteroidales bacterium
VanZ-2
HCT29672.1
Bacteroidales bacterium
VanZ-2
OGM77217.1
Candidatus Woesebacteria bacterium RIFOXYA1_FULL_43_9
VanZ-2
MBO5787785.1
Bacteroidaceae bacterium
VanZ-2
MBW8864475.1
Verrucomicrobiota bacterium
VanZ-2
MBS1745769.1
Bacteroidota bacterium
VanZ-2
NLM09685.1
Clostridiaceae bacterium
VanZ-2
MBQ9642059.1
Bacteroidaceae bacterium
VanZ-2
MBW2736356.1
Deltaproteobacteria bacterium
VanZ-2
OUS09136.1
Rhodobacterales bacterium 52_120_T64
VanZ-2
OFX45597.1
Bacteroidetes bacterium GWA2_40_14
VanZ-2
HFE31669.1
Gammaproteobacteria bacterium
VanZ-2
PHR60188.1
Robiginitomaculum sp.
VanZ-2
MBQ7781361.1
Lachnospiraceae bacterium
VanZ-2
OFY45078.1
Bacteroidetes bacterium GWF2_40_14
VanZ-2
MBK6787991.1
Betaproteobacteria bacterium
VanZ-2
MBN2105056.1
bacterium
VanZ-2
MBO4897549.1
Clostridia bacterium
VanZ-2
WP_225540681.1
VanZ-2
HIG60758.1
Gammaproteobacteria bacterium
VanZ-2
WP_169561935.1
Sneathiella chinensis
VanZ-2
HAN57828.1
Erysipelotrichaceae bacterium
VanZ-2
WP_012935070.1
Conexibacter woesei DSM 14684
VanZ-2
NTW28434.1
Coriobacteriia bacterium
VanZ-2
WP_164356462.1
Sulfitobacter sediminilitoris
VanZ-2
MBN3040968.1
Candidatus Omnitrophota bacterium
VanZ-2
HIX04364.1
Candidatus Odoribacter faecigallinarum
VanZ-2
OQA16554.1
bacterium ADurb.Bin363
VanZ-2
MBI5463901.1
Ignavibacteriales bacterium
VanZ-2
GJG98869.1
Cupriavidus pauculus
VanZ-2
MBA4259644.1
Chitinophaga sp.
VanZ-2
MBQ6797866.1
Oscillospiraceae bacterium
VanZ-2
RYF96534.1
Chitinophagaceae bacterium
VanZ-2
WP_104942083.1
Porphyrobacter sp. HT-58-2
VanZ-2
HID82603.1
Thiotrichales bacterium
VanZ-2
PLX73198.1
Azoarcus sp.
VanZ-2
MBS3094733.1
Candidatus Pacearchaeota archaeon
VanZ-2
MBX2932362.1
Chitinophagaceae bacterium
VanZ-2
MQR96118.1
Fictibacillus phosphorivorans
VanZ-2
WP_106280030.1
Geodermatophilus tzadiensis
VanZ-2
MBP3347251.1
Clostridia bacterium
VanZ-2
MBP3410368.1
Clostridia bacterium
VanZ-2
HAP8890473.1
Enterococcus faecium
VanZ-2
HAT02126.1
Oribacterium sp.
VanZ-2
MBF0219972.1
Gammaproteobacteria bacterium
VanZ-2
EST12154.1
Sporolactobacillus laevolacticus DSM 442
VanZ-2
RKY70938.1
Candidatus Latescibacterota bacterium
VanZ-2
MBX3177340.1
Candidatus Hydrogenedentota bacterium
VanZ-2
MBI5778600.1
Planctomycetota bacterium
VanZ-2
MBI4560110.1
Candidatus Hydrogenedentota bacterium
VanZ-2
WP_002683434.1
Beggiatoa alba B18LD
VanZ-2
NLP05305.1
Candidatus Fermentibacterota bacterium
VanZ-2
MCD4796320.1
Candidatus Cloacimonadota bacterium
VanZ-2
MBI2920599.1
Planctomycetota bacterium
VanZ-2
NQT19848.1
Planctomycetota bacterium
VanZ-2
MBQ2391519.1
Clostridia bacterium
VanZ-2
MBK6279649.1
Gammaproteobacteria bacterium
VanZ-2
OGS19226.1
Elusimicrobia bacterium RIFOXYA2_FULL_50_26
VanZ-2
MCA1590273.1
Acidobacteriota bacterium
VanZ-2
NOX88450.1
Calditrichota bacterium
VanZ-2
MTI87642.1
Balneolaceae bacterium
VanZ-2
RMD85652.1
Calditrichota bacterium
VanZ-2
WP_191803761.1
Paenibacillus gallinarum
VanZ-2
NPA53406.1
Aquificota bacterium
VanZ-2
MBS3157999.1
Candidatus Woesearchaeota archaeon
VanZ-2
NMB99239.1
Thermoanaerobaculaceae bacterium
VanZ-2
OPX21650.1
Planctomycetales bacterium 4484_113
VanZ-2
MCC2670328.1
Armatimonadota bacterium
VanZ-2
WP_062166910.1
Stenotrophomonas nitritireducens
VanZ-2
NLD88436.1
Clostridiales bacterium
VanZ-2
AKU22203.1
Massilia sp. NR 4-1
VanZ-2
MBP7865748.1
Acidobacteriota bacterium
VanZ-2
RZI94008.1
Microbacterium sp.
VanZ-2
MCC6123777.1
Pirellulales bacterium
VanZ-2
HIU14400.1
Candidatus Fimiplasma intestinipullorum
VanZ-2
MBI4161923.1
Acidobacteriota bacterium
VanZ-2
WP_089035067.1
Neisseria chenwenguii
VanZ-2
NNE00695.1
Pirellulaceae bacterium
VanZ-2
WP_206254680.1
Niveibacterium sp.
VanZ-2

WP_143837541.1	*Aliiglaciecola sp. M165*	VanZ-2
NOT43811.1	*Acidobacteriota bacterium*	VanZ-2
TDI33987.1	*Acidobacteriota bacterium*	VanZ-2
MBL6644024.1	*Flavobacteriaceae bacterium*	VanZ-2
HAV41923.1	*Acidobacteriota bacterium*	VanZ-2
PYU89170.1	*Acidobacteriota bacterium*	VanZ-2
MBK9434752.1	*Micrococcales bacterium*	VanZ-2
WP_052077324.1	*Thalassobacter sp. 16PALIMAR09*	VanZ-2
MBL0167978.1	*Propionivibrio sp.*	VanZ-2
PYS95225.1	*Acidobacteriota bacterium*	VanZ-2
PWT88489.1	*Acidobacteriota bacterium*	VanZ-2
WP_062421050.1	*Leptolinea tardivitalis*	VanZ-2
MCA1556137.1	*Acidobacteriota bacterium*	VanZ-2
MCA9741707.1	*Candidatus Zhuqueibacterota bacterium*	VanZ-2
MBN2089317.1	*Candidatus Zhuqueibacterota bacterium*	VanZ-2
MBS1324840.1	*Oscillospiraceae bacterium*	VanZ-2
MBL8314343.1	*Rubrivivax sp.*	VanZ-2
MBU4202303.1	*Candidatus Altiarchaeota archaeon*	VanZ-2
MBT9568766.1	*Thiobacillus sp.*	VanZ-2
MBC7880406.1	*Gloeobacterales cyanobacterium ES-bin-313*	VanZ-2
PIP80129.1	*Gammaproteobacteria bacterium CG22_combo_CG10-13_8_21_14_all_40_8*	VanZ-2
PKM09462.1	*Gammaproteobacteria bacterium HGW-Gammaproteobacteria-5*	VanZ-2
KPF52825.1	*beta proteobacterium AAP51*	VanZ-2
MBA2476713.1	*Actinomycetota bacterium*	VanZ-2
KOR28811.1	*Achromatium sp. WMS1*	VanZ-2
OGT28291.1	*Gammaproteobacteria bacterium RBG_16_66_13*	VanZ-2
MCD6569173.1	*Deltaproteobacteria bacterium*	VanZ-2
WP_145082378.1	*Aureliella helgolandensis*	VanZ-2
PKM95486.1	*Firmicutes bacterium HGW-Firmicutes-1*	VanZ-2
RLB11502.1	*Deltaproteobacteria bacterium*	VanZ-2
MCD6328179.1	*bacterium*	VanZ-2
NTW96200.1	*Erysipelotrichaceae bacterium*	VanZ-2
RUM68693.1	*Sulfurovum sp.*	VanZ-2
MBL8300398.1	*Rhodanobacteraceae bacterium*	VanZ-2
TNE52469.1	*Deltaproteobacteria bacterium*	VanZ-2
MBR2860882.1	*Clostridia bacterium*	VanZ-2
MBE7055624.1	*Oscillospiraceae bacterium*	VanZ-2
WP_143525060.1	*Labilibacter marinus*	VanZ-2
MBI1996517.1	*Deltaproteobacteria bacterium*	VanZ-2
MBN1492724.1	*Candidatus Omnitrophota bacterium*	VanZ-2
MBI2346158.1	*Deltaproteobacteria bacterium*	VanZ-2
HBM35331.1	*Sulfuricurvum sp.*	VanZ-2
MBI3755175.1		VanZ-2
MBT6691359.1	*Candidatus Parcubacteria bacterium*	VanZ-2
GBE28406.1	*bacterium BMS3Bbin03*	VanZ-2
OGI21850.1	*Candidatus Moranbacteria bacterium RIFCSPHIGHO2_01_FULL_55_24*	VanZ-2
MBM3481434.1	*Alphaproteobacteria bacterium*	VanZ-2
WP_140944920.1	*Candidatus Thermokryptus mobilis*	VanZ-2
HGX05257.1		VanZ-2
WP_162454757.1	*Pseudoxanthomonas kalamensis DSM 18571*	VanZ-2
GJL55055.1	*Nitrospirales bacterium*	VanZ-2
MBN1833327.1	*Deltaproteobacteria bacterium*	VanZ-2
MBN1850336.1	*Deltaproteobacteria bacterium*	VanZ-2
OGW54612.1	*Nitrospirae bacterium RIFCSPLOWO2_02_42_7*	VanZ-2
WP_140726875.1	*Pseudomonas sp. Hp2*	VanZ-2
MBR1731600.1	*Ruminococcus sp.*	VanZ-2
HCR71308.1	*Anaerolineae bacterium*	VanZ-2
HEY43463.1	*Anaerolineae bacterium*	VanZ-2
HCA05001.1	*Oscillospiraceae bacterium*	VanZ-2
MBO5268185.1	*Muribaculaceae bacterium*	VanZ-2
MBW1644846.1	*Deltaproteobacteria bacterium*	VanZ-2
MCB1667062.1	*Pseudomonadales bacterium*	VanZ-2
MCB0857384.1	*Solirubrobacterales bacterium*	VanZ-2
MBW1853803.1	*Deltaproteobacteria bacterium*	VanZ-2
MBA4410698.1	*Odoribacter sp.*	VanZ-2
MBX3235108.1	*Nitrospiraceae bacterium*	VanZ-2
MBN1873384.1	*Anaerolineae bacterium*	VanZ-2
MBL7034019.1	*Candidatus Delongbacteria bacterium*	VanZ-2
MBU0944040.1	*Pseudomonadota bacterium*	VanZ-2
MBQ7842827.1	*Odoribacter sp.*	VanZ-2
MBT7073828.1	*Anaerolineae bacterium*	VanZ-2
MBW2579966.1	*Deltaproteobacteria bacterium*	VanZ-2
WP_013445559.1	*Paludibacter propionicigenes WB4*	VanZ-2
MCC7446176.1	*Anaerolineae bacterium*	VanZ-2
MBQ6626238.1	*Ruminococcus sp.*	VanZ-2
RME84505.1	*Zetaproteobacteria bacterium*	VanZ-2
NDP22899.1	*Paludibacter sp.*	VanZ-2
MBA3059316.1	*Rhodoferax sp.*	VanZ-2
MBP6610153.1	*Paludibacter sp.*	VanZ-2
MBN2765079.1	*Paludibacteraceae bacterium*	VanZ-2
HHD32291.1		VanZ-2
MBA5866923.1	*Nitrospira sp. CR1.3*	VanZ-2
TQL62128.1		VanZ-2
MBR6117789.1	*Paludibacteraceae bacterium*	VanZ-2
RLG56313.1	*Candidatus Hydrothermarchaeota archaeon*	VanZ-2
MBA2792854.1	*Thermoleophilaceae bacterium*	VanZ-2
MCA9992378.1	*Anaerolineales bacterium*	VanZ-2
MCB8916261.1	*Thermoleophilales bacterium*	VanZ-2
WP_023399590.1	*Pseudoalteromonas sp. Of7M-16*	VanZ-2
NIM90116.1	*Candidatus Aminicenantes bacterium*	VanZ-2
MBC8335336.1	*Candidatus Desulfolinea nitratireducens*	VanZ-2
MBN2416573.1	*bacterium*	VanZ-2
MAR34014.1	*Chloroflexota bacterium*	VanZ-2

MBO5182753.1	*Paraprevotella sp.*	VanZ-2
MBP5288821.1	*Clostridia bacterium*	VanZ-2
MBN2543541.1	*bacterium*	VanZ-2
NPA95777.1	*Thermodesulfobacteriota bacterium*	VanZ-2
HBC21687.1	*Porphyromonadaceae bacterium*	VanZ-2
RME75026.1	*Chloroflexota bacterium*	VanZ-2
THB79747.1	*Desulfobulbaceae bacterium*	VanZ-2
NWG01537.1	*Syntrophaceae bacterium*	VanZ-2
MBD9230197.1	*Prevotella sp.*	VanZ-2
WP_068673101.1	*Oceanobacillus sp. Castelsardo*	VanZ-2
MBQ2196693.1	*Prevotella sp.*	VanZ-2
MBQ8656151.1	*Prevotella sp.*	VanZ-2
KAA0284703.1	*Chloroflexota bacterium*	VanZ-2
WP_078693554.1		VanZ-2
MBR2102982.1	*Prevotella sp.*	VanZ-2
MYH40719.1	*Chloroflexota bacterium*	VanZ-2
MBR1414352.1	*Prevotella sp.*	VanZ-2
WP_170123478.1	*Plasticicumulans acidivorans*	VanZ-2
MBP7767847.1	*Prevotella sp.*	VanZ-2
WP_049926550.1		VanZ-2
MBN1134900.1	*Methanosarcinaceae archaeon*	VanZ-2
WP_143083184.1	*Alteribacillus iranensis*	VanZ-2
MBI9068073.1	*Salinivirgaceae bacterium*	VanZ-2
QGX94848.1	*Haloplanus rallus*	VanZ-2
MBK8720924.1	*Saprospiraceae bacterium*	VanZ-2
MBQ7502820.1	*bacterium*	VanZ-2
MBA3485924.1	*Lysobacter sp.*	VanZ-2
WP_163339741.1	*Desulfopila sp. IMCC35008*	VanZ-2
NOR25600.1	*Desulforhopalus sp.*	VanZ-2
MCD4687894.1	*Desulfuromonadaceae bacterium*	VanZ-2
WP_026908908.1	*Paucisalibacillus globulus DSM 18846*	VanZ-2
WP_008319586.1	*Haloferax mucosum ATCC BAA-1512*	VanZ-2
WP_014732687.1	*Haloferax mediterranei ATCC 33500*	VanZ-2
OGU56091.1	*Ignavibacteria bacterium GWF2_33_9*	VanZ-2
MCB9639865.1	*Myxococcales bacterium*	VanZ-2
WP_142714873.1	*Fodinibius sediminis*	VanZ-2
MBC8590908.1	*Wansuia hejianensis*	VanZ-2
MCA9616348.1		VanZ-2
MBS6627921.1	*Clostridiales bacterium*	VanZ-2
ELX13030.1	*Janthinobacterium sp. HH01*	VanZ-2
WP_155876218.1	*Desulfuromonas sp. AOP6*	VanZ-2
MBU95191.1	*Balneola sp.*	VanZ-2
NGP77315.1	*Halalkalibaculum roseum*	VanZ-2
MBQ7352939.1	*Clostridia bacterium*	VanZ-2
HHE07882.1	*Chlorobaculum parvum*	VanZ-2
MBO4419490.1	*Oscillospiraceae bacterium*	VanZ-2
MBS1968587.1	*Chloroflexi bacterium SZAS-1*	VanZ-2
MBD8512855.1	*Photobacterium arenosum*	VanZ-2
HHS10221.1		VanZ-2
NNE08174.1	*Gemmatimonadota bacterium*	VanZ-2
UCE18016.1	*Gemmatimonadota bacterium*	VanZ-2
OFV66250.1	*Candidatus Syntrophoarchaeum butanivorans*	VanZ-2
NLE88838.1	*Myxococcales bacterium*	VanZ-2
TDA67037.1	*Clostridia bacterium*	VanZ-2
MBQ9205771.1	*Treponema sp.*	VanZ-2
MBQ7986958.1	*Bacteroidaceae bacterium*	VanZ-2
MBW4511396.1	*Scytonematopsis contorta HA4267-MV1*	VanZ-2
MBR5899005.1	*Muribaculaceae bacterium*	VanZ-2
MAE56767.1	*Methanobacteriota archaeon*	VanZ-2
MBX3707958.1	*Pseudomonadales bacterium*	VanZ-2
OUV90188.1	*Puniceicoccaceae bacterium TMED149*	VanZ-2
MBM3859920.1	*Verrucomicrobiota bacterium*	VanZ-2
WP_089669571.1	*Halobacterium jilantaiense*	VanZ-2
MBS0011069.1	*Bacteroidales bacterium*	VanZ-2
MBL7975246.1	*Candidatus Kapaibacterium sp.*	VanZ-2
MCD6217390.1	*bacterium*	VanZ-2
HFK23938.1		VanZ-2
RHC45448.1	*Dorea formicigenerans*	VanZ-2
OIO71667.1	*Zetaproteobacteria bacterium CG1_02_53_45*	VanZ-2
KGO99518.1	*Lysobacter defluvii IMMIB APB-9 = DSM 18482*	VanZ-2
NIN34974.1	*Gammaproteobacteria bacterium*	VanZ-2
PKO56706.1	*Betaproteobacteria bacterium HGW-Betaproteobacteria-21*	VanZ-2
MBR3091490.1	*Bacteroidaceae bacterium*	VanZ-2
MCA9555359.1	*Myxococcales bacterium*	VanZ-2
MAE04902.1	*Nitrospinota bacterium*	VanZ-2
WP_143907273.1	*Tomitella fengzijianii*	VanZ-2
MBQ4277051.1	*Bacteroidaceae bacterium*	VanZ-2
NNG12891.1	*Halobacteria archaeon*	VanZ-2
WP_224766751.1	*Janibacter melonis*	VanZ-2
MBI4427841.1	*Ignavibacteriales bacterium*	VanZ-2
MBC7574119.1	*Herminiimonas sp.*	VanZ-2
MBR6891668.1	*Bacteroidaceae bacterium*	VanZ-2
PIE51432.1	*Candidatus Fermentibacterota bacterium*	VanZ-2
MCB1504466.1	*Hyphomicrobiaceae bacterium*	VanZ-2
MBD3893195.1	*Hydrogenophaga sp.*	VanZ-2
MBI4754052.1	*Betaproteobacteria bacterium*	VanZ-2
MBS3896355.1	*Silanimonas sp.*	VanZ-2
NUM79957.1	*bacterium*	VanZ-2
NUS39433.1	*Lysobacter sp.*	VanZ-2
MBU4304068.1	*Candidatus Omnitrophota bacterium*	VanZ-2
MBK8832972.1	*Candidatus Opimibacter skivensis*	VanZ-2
MSQ40861.1	*Dehalococcoidia bacterium*	VanZ-2
MBQ7225821.1	*Clostridia bacterium*	VanZ-2

MCC8147141.1	*Bacteroidales bacterium*	VanZ-2
PLX24248.1	*Marinilabiliales bacterium*	VanZ-2
MBO5220240.1	*Clostridia bacterium*	VanZ-2
MBQ2690847.1	*Clostridia bacterium*	VanZ-2
MAN92486.1	*Hyphomonadaceae bacterium*	VanZ-2
AUJ71366.1	*Pseudoalteromonas sp. NC201*	VanZ-2
MCD6080323.1	*Candidatus Omnitrophota bacterium*	VanZ-2
WP_015778742.1	*Cryptobacterium curtum*	VanZ-2
BAH54589.1	*Rhodococcus opacus B4*	VanZ-2
MBJ7284066.1	*Akkermansiaceae bacterium*	VanZ-2
MBT4516702.1	*Candidatus Komeilibacteria bacterium*	VanZ-2
MBL4776070.1	*Mariprofundus sp.*	VanZ-2
WP_008296707.1	*Bhargavaea cecembensis DSE10*	VanZ-2
CCX63633.1	*Prevotella sp. CAG:1058*	VanZ-2
MBC7720450.1	*Chitinophagaceae bacterium*	VanZ-2
MBN8706164.1	*Bacteroidota bacterium*	VanZ-2
MBQ9812758.1	*Thermoguttaceae bacterium*	VanZ-2
MBF0369859.1	*Magnetococcales bacterium*	VanZ-2
WP_232588087.1	*Halobacterium noricense*	VanZ-2
MBF0584235.1	*Magnetococcales bacterium*	VanZ-2
MAP21516.1	*Alteromonadaceae bacterium*	VanZ-2
HIT15387.1	*Candidatus Avimuribaculum pullicola*	VanZ-2
MBN1948326.1	*Candidatus Cloacimonadota bacterium*	VanZ-2
WP_190531311.1		VanZ-2
WP_143730394.1	*Microbulbifer sp. GL-2*	VanZ-2
WP_015246533.1	*Singulisphaera acidiphila DSM 18658*	VanZ-2
MBV5318143.1	*Desulfobulbaceae bacterium*	VanZ-2
MBX2858989.1	*Cellvibrionaceae bacterium*	VanZ-2
NBK69839.1		VanZ-2
OYV05393.1	*Verrucomicrobiales bacterium VVV1*	VanZ-2
KAB2891172.1	*Desulfobulbaceae bacterium*	VanZ-2
MBU1202908.1	*Patescibacteria group bacterium*	VanZ-2
NBQ40445.1	*Alphaproteobacteria bacterium*	VanZ-2
MBQ9485247.1	*Clostridia bacterium*	VanZ-2
MCA9833127.1	*Thermomicrobiales bacterium*	VanZ-2
MCD6406098.1	*Planctomycetota bacterium*	VanZ-2
MRR09118.1	*bacterium*	VanZ-2
MBP5134780.1	*Paludibacteraceae bacterium*	VanZ-2
OZA30452.1	*Hydrogenophilales bacterium 17-61-9*	VanZ-2
OGX38225.1	*Omnitrophica WOR_2 bacterium RIFCSPHIGHO2_02_FULL_52_10*	VanZ-2
MBP5306679.1	*Paludibacteraceae bacterium*	VanZ-2
ROL59330.1	*Bacteroidetes/Chlorobi group bacterium ChocPot_Mid*	VanZ-2
HAE58167.1	*Anaerolineae bacterium*	VanZ-2
MBP8945682.1	*Paludibacteraceae bacterium*	VanZ-2
PID49034.1	*Pseudomonadota bacterium*	VanZ-2
NQV19830.1	*Rhodospirillales bacterium*	VanZ-2
MBS7237313.1	*Bacteroidaceae bacterium*	VanZ-2
MBC7308529.1	*Nostocoides sp.*	VanZ-2
HGY06636.1		VanZ-2
MBE6540865.1	*Oscillospiraceae bacterium*	VanZ-2
WP_144992253.1	*Polystyrenella longa*	VanZ-2
HCW91261.1	*Marinobacter sp.*	VanZ-2
MBI4494785.1	*Chloroflexota bacterium*	VanZ-2
MBI1795462.1		VanZ-2
TXT16935.1	*Erysipelotrichaceae bacterium*	VanZ-2
MBL0349432.1	*Elusimicrobiota bacterium*	VanZ-2
WP_009788018.1	*Lyngbya sp. PCC 8106*	VanZ-2
MBN2682932.1	*Bacteroidales bacterium*	VanZ-2
HID50654.1	*Anaerolineae bacterium*	VanZ-2
MBR1446758.1	*Alloprevotella sp.*	VanZ-2
NNE06883.1	*Lysobacterales bacterium*	VanZ-2
WP_154739042.1	*Hyphomicrobium album*	VanZ-2
MBM4071029.1	*Planctomycetota bacterium*	VanZ-2
RMD45910.1	*Candidatus Pacearchaeota archaeon*	VanZ-2
MBO5104374.1	*Ruminococcus sp.*	VanZ-2
WP_223047210.1	*Leptothermofonsia sichuanensis E412*	VanZ-2
MBQ8075104.1	*Oscillospiraceae bacterium*	VanZ-2
HCI54726.1	*Bacteroidales bacterium*	VanZ-2
MBI4650119.1	*Candidatus Desantisbacteria bacterium*	VanZ-2
MBN1922904.1	*Anaerolineae bacterium*	VanZ-2
MBS3093296.1	*Candidatus Pacearchaeota archaeon*	VanZ-2
MBT1702225.1	*Chryseosolibacter indicus*	VanZ-2
NND73242.1	*Rhodothermales bacterium*	VanZ-2
MBO4925161.1	*Clostridia bacterium*	VanZ-2
MBR1832364.1	*Ruminiclostridium sp.*	VanZ-2
WP_195711844.1		VanZ-2
MBN9076806.1	*Hyphomicrobiales bacterium*	VanZ-2
MBK6425732.1	*Blastocatellia bacterium*	VanZ-2
MCD6262127.1	*Deltaproteobacteria bacterium*	VanZ-2
NOZ62334.1	*Calditrichota bacterium*	VanZ-2
MBL8493356.1	*Rhodocyclaceae bacterium*	VanZ-2
MBC8468992.1	*Planctomycetota bacterium*	VanZ-2
RKX17372.1	*Candidatus Zixiibacteriota bacterium*	VanZ-2
RKX18500.1	*Candidatus Zixiibacteriota bacterium*	VanZ-2
MQA90778.1	*Gemmatimonas sp.*	VanZ-2
NTV46862.1	*Chlorobiales bacterium*	VanZ-2
HER07746.1	*Bacteroides sp.*	VanZ-2
MBQ2443884.1	*Clostridia bacterium*	VanZ-2
MBI3772282.1	*Gammaproteobacteria bacterium*	VanZ-2
MBS1787193.1	*Acidobacteriota bacterium*	VanZ-2
NIM99207.1	*Candidatus Zixiibacteriota bacterium*	VanZ-2
MBR3544461.1	*Oscillospiraceae bacterium*	VanZ-2
MBW8784303.1	*Novosphingobium sp.*	VanZ-2

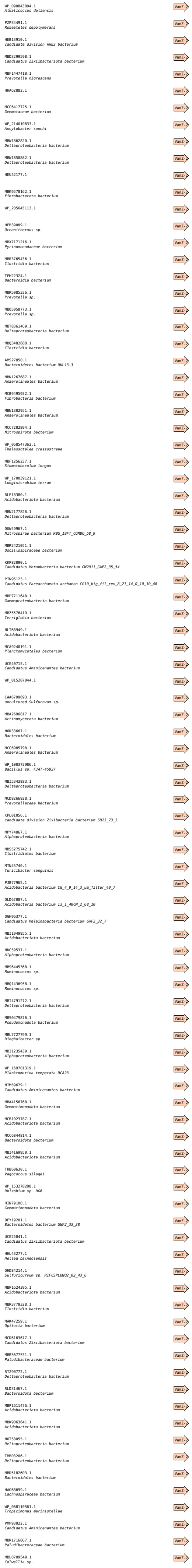
WP_090843804.1
Alkalicoccus daliensis
VanZ-2
PZP34491.1
Roseateles depolymerans
VanZ-2
HEB13910.1
candidate division WWE3 bacterium
VanZ-2
MBD3299598.1
Candidatus Zixiibacteriota bacterium
VanZ-2
MBF1447410.1
Prevotella nigrescens
VanZ-2
HHA62882.1
VanZ-2
MCC6417725.1
Gemmataceae bacterium
VanZ-2
WP_214010837.1
Ancylobacter sonchi
VanZ-2
MBW1862820.1
Deltaproteobacteria bacterium
VanZ-2
MBW1850882.1
Deltaproteobacteria bacterium
VanZ-2
HEU52177.1
VanZ-2
MBK9578162.1
Fibrobacterota bacterium
VanZ-2
WP_205645113.1
VanZ-2
HFB39089.1
Oceanithermus sp.
VanZ-2
MBX7171216.1
Pyrinomonadaceae bacterium
VanZ-2
MBR3765436.1
Clostridia bacterium
VanZ-2
TFH22324.1
Bacteroidia bacterium
VanZ-2
MBR5085336.1
Prevotella sp.
VanZ-2
MBO5058773.1
Prevotella sp.
VanZ-2
MBT8361469.1
Deltaproteobacteria bacterium
VanZ-2
MBQ3482688.1
Clostridia bacterium
VanZ-2
AMS27850.1
Bacteroidetes bacterium UKL13-3
VanZ-2
MBN1267687.1
Anaerolineales bacterium
VanZ-2
MCB9495932.1
Fibrobacteria bacterium
VanZ-2
MBN1302951.1
Anaerolineales bacterium
VanZ-2
MCC7202894.1
Nitrospirota bacterium
VanZ-2
WP_068547362.1
Thalassotalea crassostreae
VanZ-2
MBF1256237.1
Stomatobaculum longum
VanZ-2
WP_170039121.1
Longimicrobium terrae
VanZ-2
RLE18380.1
Acidobacteriota bacterium
VanZ-2
MBN2177826.1
Deltaproteobacteria bacterium
VanZ-2
OGW49967.1
Nitrospirae bacterium RBG_19FT_COMBO_58_9
VanZ-2
MBR2421051.1
Oscillospiraceae bacterium
VanZ-2
KKP82090.1
Candidatus Moranbacteria bacterium GW2011_GWF2_35_54
VanZ-2
PIN95123.1
Candidatus Pacearchaeota archaeon CG10_big_fil_rev_8_21_14_0_10_30_48
VanZ-2
MBP7711648.1
Gammaproteobacteria bacterium
VanZ-2
MBZ5576419.1
Terriglobia bacterium
VanZ-2
NLT68949.1
Acidobacteriota bacterium
VanZ-2
MCA9240191.1
Planctomycetales bacterium
VanZ-2
UCE40715.1
Candidatus Aminicenantes bacterium
VanZ-2
WP_015207044.1
VanZ-2
CAA6799693.1
uncultured Sulfurovum sp.
VanZ-2
MBA2696817.1
Actinomycetota bacterium
VanZ-2
NOR33667.1
Bacteroidales bacterium
VanZ-2
MCC6985798.1
Anaerolineales bacterium
VanZ-2
WP_100372986.1
Bacillus sp. FJAT-45037
VanZ-2
MBI5343883.1
Deltaproteobacteria bacterium
VanZ-2
MCD8266928.1
Prevotellaceae bacterium
VanZ-2
KPL01056.1
candidate division Zixibacteria bacterium SM23_73_3
VanZ-2
MPY74867.1
Alphaproteobacteria bacterium
VanZ-2
MBS5275742.1
Clostridiales bacterium
VanZ-2
MTN45740.1
Turicibacter sanguinis
VanZ-2
PJB77965.1
Acidobacteria bacterium CG_4_9_14_3_um_filter_49_7
VanZ-2
OLD67087.1
Acidobacteria bacterium 13_1_40CM_2_68_10
VanZ-2
OGH96377.1
Candidatus Melainabacteria bacterium GWF2_32_7
VanZ-2
MBI1949955.1
Acidobacteriota bacterium
VanZ-2
NDC59537.1
Alphaproteobacteria bacterium
VanZ-2
MBS6445368.1
Ruminococcus sp.
VanZ-2
MBQ1436958.1
Ruminococcus sp.
VanZ-2
MBI4791272.1
Deltaproteobacteria bacterium
VanZ-2
MBS0479876.1
Pseudomonadota bacterium
VanZ-2
MBL7727799.1
Dinghuibacter sp.
VanZ-2
MBI1235439.1
Alphaproteobacteria bacterium
VanZ-2
WP_169701319.1
Planktomarina temperata RCA23
VanZ-2
NIM59679.1
Candidatus Aminicenantes bacterium
VanZ-2
MBA4156768.1
Gemmatimonadota bacterium
VanZ-2
MCB1023787.1
Acidobacteriota bacterium
VanZ-2
MCC6844814.1
Bacteroidota bacterium
VanZ-2
MBI4169950.1
Acidobacteriota bacterium
VanZ-2
THB60630.1
Vagococcus silagei
VanZ-2
WP_153270208.1
Rhizobium sp. BG6
VanZ-2
HIN79100.1
Gemmatimonadota bacterium
VanZ-2
OFY19201.1
Bacteroidetes bacterium GWF2_33_38
VanZ-2
UCE25041.1
Candidatus Zixiibacteriota bacterium
VanZ-2
HHL43277.1
Hellea balneolensis
VanZ-2
OHD84214.1
Sulfuricurvum sp. RIFCSPLOWO2_02_43_6
VanZ-2
MBP1624395.1
Acidobacteriota bacterium
VanZ-2
MBR3779328.1
Clostridia bacterium
VanZ-2
MAK47259.1
Opitutia bacterium
VanZ-2
MCD6163677.1
Candidatus Zixiibacteriota bacterium
VanZ-2
MBR5677531.1
Paludibacteraceae bacterium
VanZ-2
RTZ90772.1
Deltaproteobacteria bacterium
VanZ-2
RLD31467.1
Bacteroidota bacterium
VanZ-2
MBP1611476.1
Acidobacteriota bacterium
VanZ-2
MBK9063641.1
Acidobacteriota bacterium
VanZ-2
NOT58055.1
Deltaproteobacteria bacterium
VanZ-2
TMB03206.1
Deltaproteobacteria bacterium
VanZ-2
MBD5182603.1
Bacteroidales bacterium
VanZ-2
HAG68699.1
Lachnospiraceae bacterium
VanZ-2
WP_068110561.1
Tropicimonas marinistellae
VanZ-2
PMP93922.1
Candidatus Aminicenantes bacterium
VanZ-2
MBR1716067.1
Paludibacteraceae bacterium
VanZ-2
MBL0709549.1
Colwellia sp.
VanZ-2

MBF2062947.1	*Calothrix sp. C42_A2020_038*	VanZ-2
NCD25051.1	*Deltaproteobacteria bacterium*	VanZ-2
RLD84820.1	*Bacteroidota bacterium*	VanZ-2
MBR2592557.1	*Oscillospiraceae bacterium*	VanZ-2
RLD75344.1	*Bacteroidota bacterium*	VanZ-2
MBW4550395.1	*Aphanocapsa sp. GSE-SYN-MK-11-07L*	VanZ-2
PHS30913.1	*Sulfurovum sp.*	VanZ-2
MBI4519997.1	*Gemmatimonadota bacterium*	VanZ-2
MBU6443724.1	*Alphaproteobacteria bacterium*	VanZ-2
MCA1789454.1	*Thioalkalivibrio sp.*	VanZ-2
NHT74930.1	*Ferranicluibacter rubi*	VanZ-2
MBW3534715.1	*Gemmatimonadota bacterium*	VanZ-2
WP_195248659.1	*Turicibacter sanguinis*	VanZ-2
RMG88674.1	*Bacteroidota bacterium*	VanZ-2
MBI5050668.1	*Nitrospirota bacterium*	VanZ-2
MBU1565975.1	*Pseudomonadota bacterium*	VanZ-2
UCD18589.1	*Candidatus Zixiibacteriota bacterium*	VanZ-2
KZZ73426.1		VanZ-2
MBW3628730.1	*Gemmatimonadota bacterium*	VanZ-2
RMH72539.1	*Gemmatimonadota bacterium*	VanZ-2
HHK95908.1		VanZ-2
MCB0717277.1	*Bacteroidota bacterium*	VanZ-2
MBT3206031.1	*Gammaproteobacteria bacterium*	VanZ-2
OJT97636.1	*Alphaproteobacteria bacterium 65-7*	VanZ-2
SNR23103.1	*Halorubrum ezzemoulense*	VanZ-2
WP_181833280.1	*Bacillus taeanensis*	VanZ-2
MBD1148974.1	*Pelagibacterales bacterium SAG-MED30*	VanZ-2
MBE31309.1	*Acidiferrobacteraceae bacterium*	VanZ-2
MBW3569631.1	*Gemmatimonadota bacterium*	VanZ-2
WP_194120618.1	*Dyadobacter subterraneus*	VanZ-2
UCF66189.1	*Acidobacteriota bacterium*	VanZ-2
MBN1105354.1	*Deltaproteobacteria bacterium*	VanZ-2
PCH92924.1	*Paracoccaceae bacterium*	VanZ-2
TDI45399.1	*Acidobacteriota bacterium*	VanZ-2
WP_005002965.1	*Nitrococcus mobilis Nb-231*	VanZ-2
OJW12698.1	*Planctomycetales bacterium 71-10*	VanZ-2
HAP35767.1	*Bacteroidota bacterium*	VanZ-2
MCB0019559.1	*Anaerolineales bacterium*	VanZ-2
GAY30246.1	*Prevotella sp. MGM2*	VanZ-2
OKL54140.1	*Bowdeniella nasicola*	VanZ-2
MBR6594338.1	*Clostridia bacterium*	VanZ-2
MBD0338020.1	*Thermoleophilia bacterium*	VanZ-2
MCA9961789.1	*Anaerolineales bacterium*	VanZ-2
MBA2599586.1	*Actinomycetota bacterium*	VanZ-2
MBI4441641.1	*Candidatus Woesearchaeota archaeon*	VanZ-2
HAV27434.1	*Lachnospiraceae bacterium*	VanZ-2
MBV6397512.1	*Anaerolineales bacterium*	VanZ-2
KUK39669.1	*Synergistales bacterium 54_24*	VanZ-2
MBS5436795.1	*Prevotella sp.*	VanZ-2
MBO9489895.1	*Endozoicomonas sp. G2_1*	VanZ-2
MBN2555143.1	*Anaerolineales bacterium*	VanZ-2
MBU2446461.1	*Bacteroidota bacterium*	VanZ-2
RSX57397.1	*Bifidobacterium samirii*	VanZ-2
MBE6319229.1	*Bacteroidales bacterium*	VanZ-2
MBM2847831.1	*Anaerolineales bacterium*	VanZ-2
HHD75245.1	*Campylobacterales bacterium*	VanZ-2
NQZ22539.1	*Colwellia sp.*	VanZ-2
OGW36449.1	*Nitrospirae bacterium GWD2_57_9*	VanZ-2
UCD67253.1	*Betaproteobacteria bacterium*	VanZ-2
WP_115734112.1	*Aminobacter aminovorans*	VanZ-2
MBW8889730.1	*Fibrobacterota bacterium*	VanZ-2
TFH49766.1	*Bacteroidia bacterium*	VanZ-2
MBR6978758.1	*Prevotella sp.*	VanZ-2
HEX61946.1		VanZ-2
PIN93448.1	*Candidatus Pacearchaeota archaeon CG10_big_fil_rev_8_21_14_0_10_31_24*	VanZ-2
KKS21608.1	*candidate division WWE3 bacterium GW2011_GWA1_41_8*	VanZ-2
NUM47834.1	*Anaerolineales bacterium*	VanZ-2
SEO79328.1	*Rhodospirillales bacterium URHD0017*	VanZ-2
WP_106483761.1	*Bacteroides sp. Marseille-P3684*	VanZ-2
HCW43828.1	*Planctomycetota bacterium*	VanZ-2
MBM3831931.1	*Verrucomicrobiota bacterium*	VanZ-2
MBU1739067.1	*Pseudomonadota bacterium*	VanZ-2
BAN33875.1	*Sulfuricella denitrificans skB26*	VanZ-2
MBW1758802.1	*Deltaproteobacteria bacterium*	VanZ-2
MCD6353062.1	*Pseudomonadota bacterium*	VanZ-2
PHR93570.1	*Robiginitomaculum sp.*	VanZ-2
MBQ8315804.1	*Lachnospiraceae bacterium*	VanZ-2
WP_002641269.1	*Simonsiella muelleri*	VanZ-2
MBU1343092.1	*Pseudomonadota bacterium*	VanZ-2
RLA47723.1	*Gammaproteobacteria bacterium*	VanZ-2
HHV58971.1	*Clostridiaceae bacterium*	VanZ-2
MCA0250999.1	*Actinomycetota bacterium*	VanZ-2
MBE0584100.1	*Desulfofustis sp.*	VanZ-2
MBQ6931778.1	*Clostridia bacterium*	VanZ-2
MBU0985911.1	*Pseudomonadota bacterium*	VanZ-2
NBU75106.1	*Planctomycetota bacterium*	VanZ-2
MBK8806529.1	*Bacteroidales bacterium*	VanZ-2
OQY91350.1	*Anaerolineae bacterium UTCFX1*	VanZ-2
WP_005032836.1	*Holophaga foetida DSM 6591*	VanZ-2
WP_223928654.1	*Prevotella lacticifex*	VanZ-2
MBU0682372.1	*Pseudomonadota bacterium*	VanZ-2
RJO63810.1	*Candidatus Omnitrophota bacterium*	VanZ-2
MBD5345585.1	*Bacteroides sp.*	VanZ-2

MBW2412106.1	*Deltaproteobacteria bacterium*	VanZ-2
MCD8185011.1	*Rikenellaceae bacterium*	VanZ-2
WP_077317529.1	*Virgibacillus proomii*	VanZ-2
HCQ31546.1	*Candidatus Collierbacteria bacterium*	VanZ-2
HGR72933.1		VanZ-2
MCB1601663.1	*Lysobacterales bacterium*	VanZ-2
KKT73714.1	*Candidatus Collierbacteria bacterium GW2011_GWB1_44_6*	VanZ-2
NLF51224.1	*Leptolinea sp.*	VanZ-2
WP_143585730.1	*Thalassomonas sp. M1454*	VanZ-2
PWM73222.1	*Oscillospiraceae bacterium*	VanZ-2
MBO6064907.1	*Lachnospiraceae bacterium*	VanZ-2
HGT02266.1		VanZ-2
MBN1790035.1	*Bacteroidales bacterium*	VanZ-2
NPU95621.1	*Candidatus Omnitrophota bacterium*	VanZ-2
MBW7861909.1	*Rhodocyclaceae bacterium*	VanZ-2
MCB9377401.1	*Holophagales bacterium*	VanZ-2
ANY85110.1	*Microvirga ossetica*	VanZ-2
RLI24954.1	*Candidatus Bathyarchaeota archaeon*	VanZ-2
NQZ06402.1	*Algicola sp.*	VanZ-2
HEB12201.1	*Actinomycetota bacterium*	VanZ-2
MBN8582275.1	*Anaerolineae bacterium*	VanZ-2
MBK8958406.1	*Pseudomonadota bacterium*	VanZ-2
MBQ3841178.1	*Ruminiclostridium sp.*	VanZ-2
MBI3275827.1	*Methylocystis sp.*	VanZ-2
MBI2912042.1	*Chloroflexota bacterium*	VanZ-2
ETR76235.1	*Afipia sp. P52-10*	VanZ-2
MBI5533909.1	*Deltaproteobacteria bacterium*	VanZ-2
WP_195542729.1	*Massiliimalia timonensis*	VanZ-2
OGP32267.1	*Deltaproteobacteria bacterium GWC2_42_11*	VanZ-2
CAA9362159.1	*uncultured Gemmatimonadota bacterium*	VanZ-2
PKN36163.1	*Deltaproteobacteria bacterium HGW-Deltaproteobacteria-19*	VanZ-2
MBR5659705.1	*Lachnospiraceae bacterium*	VanZ-2
MBI2784309.1	*Gammaproteobacteria bacterium*	VanZ-2
NBP68511.1	*Cytophagia bacterium*	VanZ-2
MBE6823271.1	*Oscillospiraceae bacterium*	VanZ-2
MAZ52844.1	*Trueperaceae bacterium*	VanZ-2
MBD3379520.1	*Candidatus Omnitrophota bacterium*	VanZ-2
MCB4792531.1	*Candidatus Liberimonas magnetica*	VanZ-2
TYS78500.1	*Rossellomorea aquimaris*	VanZ-2
MBF0677899.1	*Devosia sp.*	VanZ-2
NIS59891.1	*Pseudomonadota bacterium*	VanZ-2
MBR2259037.1	*Blautia sp.*	VanZ-2
MBR1594284.1	*Alloprevotella sp.*	VanZ-2
TMQ63070.1	*Candidatus Eiseniibacteriota bacterium*	VanZ-2
NJD08140.1	*Methylococcaceae bacterium*	VanZ-2
NLM85628.1	*Clostridiales bacterium*	VanZ-2
MBS1538697.1	*Bacteroidota bacterium*	VanZ-2
MBW6457958.1	*FCB group bacterium*	VanZ-2
WP_089809485.1	*Halogeometricum rufum*	VanZ-2
WP_006053483.1	*Halogeometricum borinquense*	VanZ-2
NPA45469.1	*Chlorobiota bacterium*	VanZ-2
RLA48914.1	*Gammaproteobacteria bacterium*	VanZ-2
NJD57442.1	*Nitrospirota bacterium*	VanZ-2
PCC98441.1	*Halopseudomonas pelagia*	VanZ-2
NDL69899.1	*Vreelandella alkaliphila*	VanZ-2
PCJ22053.1	*Gammaproteobacteria bacterium*	VanZ-2
TML83523.1	*Actinomycetota bacterium*	VanZ-2
PWT72130.1	*Pseudomonadota bacterium*	VanZ-2
HIG07136.1	*Methylococcaceae bacterium*	VanZ-2
RJG05776.1	*Noviherbaspirillum cavernae*	VanZ-2
TAK11641.1	*Anaerolineae bacterium*	VanZ-2
NLN98277.1	*Eubacteriaceae bacterium*	VanZ-2
MCA9452927.1	*Nitrospira sp.*	VanZ-2
HAV75885.1	*Anaerolineae bacterium*	VanZ-2
MCB1622759.1	*Thiothrix sp.*	VanZ-2
MBA2579855.1	*Thermoleophilaceae bacterium*	VanZ-2
WP_140767730.1	*Mesorhizobium sp. B2-1-3A*	VanZ-2
MBT5506989.1		VanZ-2
MBP5770820.1		VanZ-2
WP_103081031.1		VanZ-2
MBI1761092.1	*Acidobacteriota bacterium*	VanZ-2
NOU14445.1	*Methylococcaceae bacterium*	VanZ-2
MBQ7204058.1	*Eubacterium sp.*	VanZ-2
MBX2841568.1	*Flammeovirgaceae bacterium*	VanZ-2
WP_076766417.1	*Domibacillus epiphyticus*	VanZ-2
MBS3080927.1	*Candidatus Pacearchaeota archaeon*	VanZ-2
OGR12681.1	*Desulfobacula sp. GWF2_41_7*	VanZ-2
WP_051012267.1	*Desulfobacula toluolica Tol2*	VanZ-2
WP_164362431.1	*Sulfuriroseicoccus oceanibius*	VanZ-2
MBI5136106.1	*Nitrospirota bacterium*	VanZ-2
MBN1472252.1	*Syntrophaceae bacterium*	VanZ-2
MBQ6436480.1	*bacterium*	VanZ-2
MBI5957052.1	*Chloroflexota bacterium*	VanZ-2
PCJ46065.1	*Moraxellaceae bacterium*	VanZ-2
EQI35727.1	*Clostridioides difficile Y184*	VanZ-2
RMH20679.1	*Gammaproteobacteria bacterium*	VanZ-2
MBP7435269.1	*Bacteroidales bacterium*	VanZ-2
RLC50248.1	*Candidatus Cloacimonadota bacterium*	VanZ-2
MBJ7721999.1		VanZ-2
OQB01153.1	*Spirochaetes bacterium ADurb.Bin215*	VanZ-2
MBP7859883.1	*Patescibacteria group bacterium*	VanZ-2
MBI5824417.1	*Chloroflexota bacterium*	VanZ-2
PCK08162.1	*Alteromonadaceae bacterium*	VanZ-2

MBO5099787.1	*Treponema sp.*	VanZ-2
WP_052002723.1	*Caldisalinibacter kiritimatiensis*	VanZ-2
MBQ1815562.1	*Ruminococcus sp.*	VanZ-2
MBI4535110.1	*Ignavibacteriota bacterium*	VanZ-2
MBQ3388838.1	*Thermoguttaceae bacterium*	VanZ-2
RJS98322.1	*Halococcus sp. IIIV-5B*	VanZ-2
MBC7720802.1	*Chitinophagaceae bacterium*	VanZ-2
WP_009948111.1	*Saccharopolyspora erythraea*	VanZ-2
TAN58079.1	*Patescibacteria group bacterium*	VanZ-2
MBF8292618.1	*Steroidobacteraceae bacterium*	VanZ-2
WP_012486975.1	*Cellvibrio japonicus*	VanZ-2
MBR0192970.1	*Thermoguttaceae bacterium*	VanZ-2
KKS65576.1	*Parcubacteria group bacterium GW2011_GWB1_42_6*	VanZ-2
MBR1381810.1	*Paludibacteraceae bacterium*	VanZ-2
EMA44665.1	*Halobiforma nitratireducens JCM 10879*	VanZ-2
MBF0096824.1	*Magnetococcales bacterium*	VanZ-2
KKT34666.1	*Parcubacteria group bacterium GW2011_GWA2_44_12*	VanZ-2
MBF0277601.1	*SAR324 cluster bacterium*	VanZ-2
KKS26180.1	*Parcubacteria group bacterium GW2011_GWA2_42_11*	VanZ-2
WP_130635273.1	*Alteromonas sp. KUL49*	VanZ-2
OGI06105.1	*Candidatus Margulisbacteria bacterium GWF2_35_9*	VanZ-2
MBQ6796444.1	*Clostridia bacterium*	VanZ-2
MBA2478854.1	*Sporichthyaceae bacterium*	VanZ-2
MBL8544352.1	*Hyphomonadaceae bacterium*	VanZ-2
MBQ8956709.1	*Lachnospiraceae bacterium*	VanZ-2
TMH86799.1	*Betaproteobacteria bacterium*	VanZ-2
WP_059283553.1	*Bacillus coahuilensis m2-6*	VanZ-2
NNC38392.1	*Hyphomonadaceae bacterium*	VanZ-2
CCX90118.1	*Firmicutes bacterium CAG:110*	VanZ-2
MAY35766.1	*Spongiibacteraceae bacterium*	VanZ-2
MBR2549687.1	*Clostridiales bacterium*	VanZ-2
WP_089752231.1	*Halobacillus alkaliphilus*	VanZ-2
MCD4760272.1	*bacterium*	VanZ-2
MCE1199757.1	*Marinilabiliales bacterium*	VanZ-2
NTW30553.1	*Candidatus Moraniibacteriota bacterium*	VanZ-2
NOR81610.1	*Methyloprofundus sp.*	VanZ-2
PHR30535.1	*Desulfotalea sp.*	VanZ-2
WP_080522556.1	*Methyloprofundus sedimenti*	VanZ-2
WP_193214183.1	*Luteolibacter marinus*	VanZ-2
TMH66485.1	*Betaproteobacteria bacterium*	VanZ-2
PKQ15436.1	*Actinobacteria bacterium HGW-Actinobacteria-7*	VanZ-2
MBA4054010.1	*Marivirga sp.*	VanZ-2
MBE6943102.1	*Oscillospiraceae bacterium*	VanZ-2
MBI3579591.1	*Ignavibacteriales bacterium*	VanZ-2
MBR4187607.1	*Bacteroidaceae bacterium*	VanZ-2
MBK6470320.1	*Betaproteobacteria bacterium*	VanZ-2
OIO69280.1	*Zetaproteobacteria bacterium CG1_02_55_237*	VanZ-2
MBQ0048935.1	*Candidatus Physcousia equi*	VanZ-2
NLW80923.1	*Desulfovibrionales bacterium*	VanZ-2
MBI2400310.1	*Deltaproteobacteria bacterium*	VanZ-2
MXV74284.1	*Candidatus Poribacteria bacterium*	VanZ-2
MBI3801091.1		VanZ-2
PSP81894.1	*Halobacteriales archaeon QS_1_68_17*	VanZ-2
MBQ6831939.1	*Oscillospiraceae bacterium*	VanZ-2
MBU1863320.1	*Candidatus Omnitrophota bacterium*	VanZ-2
HHL42962.1	*Hellea balneolensis*	VanZ-2
MBK6329498.1	*Bacteroidota bacterium*	VanZ-2
RBS72666.1	*Enterococcus faecium*	VanZ-2
MBV8631645.1	*Silvibacterium sp.*	VanZ-2
MBQ8975118.1	*Oscillospiraceae bacterium*	VanZ-2
NTW34435.1	*Bacteroidota bacterium*	VanZ-2
MBI4970262.1	*Candidatus Omnitrophota bacterium*	VanZ-2
MBI2624003.1	*Candidatus Parcubacteria bacterium*	VanZ-2
MBI2625598.1	*Candidatus Parcubacteria bacterium*	VanZ-2
QNI56175.1	*Synechococcus sp. BIOS-E4-1*	VanZ-2
OLE20647.1	*Betaproteobacteria bacterium 13_1_20CM_3_63_8*	VanZ-2
GFI46216.1	*Lachnospiraceae bacterium*	VanZ-2
NUN69236.1	*Bacteroidota bacterium*	VanZ-2
MBJ2183291.1	*Muribaculaceae bacterium*	VanZ-2
MCC6699709.1	*Candidatus Hydrogenedentota bacterium*	VanZ-2
MBL6432673.1	*Muribaculaceae bacterium*	VanZ-2
MBV1883977.1	*Pseudomonadales bacterium*	VanZ-2
PJE57872.1	*Candidatus Portnoybacteria bacterium CG10_big_fil_rev_8_21_14_0_10_36_7*	VanZ-2
HIX66530.1		VanZ-2
MBL8003661.1	*Candidatus Kapaibacterium sp.*	VanZ-2
MBQ4918110.1	*Muribaculaceae bacterium*	VanZ-2
PKB78924.1	*SAR202 cluster bacterium Io17-Chloro-G9*	VanZ-2
KAF0095057.1	*Puniceicoccaceae bacterium 5H*	VanZ-2
RKY67489.1	*Candidatus Latescibacterota bacterium*	VanZ-2
MBV1919966.1	*Pseudomonadales bacterium*	VanZ-2
GIS34960.1	*Gammaproteobacteria bacterium*	VanZ-2
MBQ4344440.1	*Muribaculaceae bacterium*	VanZ-2
MBR0432820.1	*Bacteroidaceae bacterium*	VanZ-2
WP_062154643.1	*Beggiatoa leptomitoformis*	VanZ-2
RTL16479.1	*Burkholderiales bacterium*	VanZ-2
MBS3768786.1	*Bacteroidales bacterium*	VanZ-2
KXK11767.1	*Chloroflexi bacterium OLB14*	VanZ-2
NKZ26004.1	*Gemella sanguinis*	VanZ-2
MBC8342783.1	*Bacteroidota bacterium*	VanZ-2
PTX98360.1	*Spartobacteria bacterium LR76*	VanZ-2
TAK08414.1	*bacterium*	VanZ-2
MBO4944856.1	*Muribaculaceae bacterium*	VanZ-2
MBC7984215.1	*Steroidobacteraceae bacterium*	VanZ-2

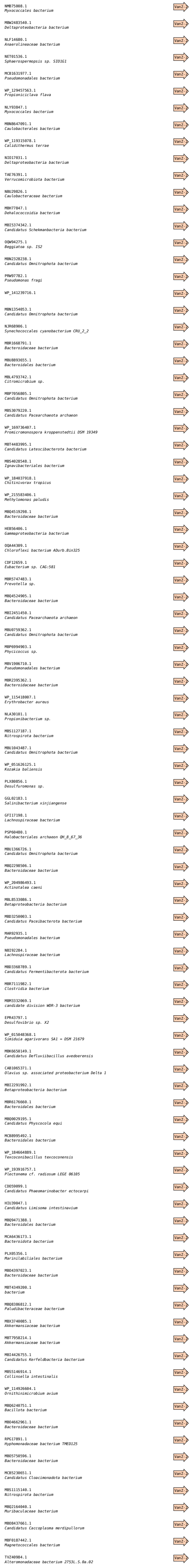
NMB75008.1
Myxococcales bacterium
VanZ-2
MBW2483540.1
Deltaproteobacteria bacterium
VanZ-2
NLF14680.1
Anaerolineaceae bacterium
VanZ-2
NET01536.1
Sphaerospermopsis sp. SIO1G1
VanZ-2
MCB1631977.1
Pseudomonadales bacterium
VanZ-2
WP_129457563.1
Propioniciclava flava
VanZ-2
NLY93847.1
Myxococcales bacterium
VanZ-2
MBN8647091.1
Caulobacterales bacterium
VanZ-2
WP_119315078.1
Calidithermus terrae
VanZ-2
NIO17031.1
Deltaproteobacteria bacterium
VanZ-2
TAE76391.1
Verrucomicrobiota bacterium
VanZ-2
NBU29826.1
Caulobacteraceae bacterium
VanZ-2
MBH77847.1
Dehalococcoidia bacterium
VanZ-2
MBI5374342.1
Candidatus Schekmanbacteria bacterium
VanZ-2
OQW94275.1
Beggiatoa sp. IS2
VanZ-2
MBN2328238.1
Candidatus Omnitrophota bacterium
VanZ-2
PRW97782.1
Pseudomonas fragi
VanZ-2
WP_141239716.1
VanZ-2
MBN1354053.1
Candidatus Omnitrophota bacterium
VanZ-2
NJR68906.1
Synechococcales cyanobacterium CRU_2_2
VanZ-2
MBR1668791.1
Bacteroidaceae bacterium
VanZ-2
MBU8893655.1
Bacteroidales bacterium
VanZ-2
MBL4793742.1
Citromicrobium sp.
VanZ-2
MBP7056805.1
Candidatus Omnitrophota bacterium
VanZ-2
MBS3079220.1
Candidatus Pacearchaeota archaeon
VanZ-2
WP_169736407.1
Promicromonospora kroppenstedtii DSM 19349
VanZ-2
MBT4483995.1
Candidatus Latescibacterota bacterium
VanZ-2
MBS4028548.1
Ignavibacteriales bacterium
VanZ-2
WP_184037918.1
Chitinivorax tropicus
VanZ-2
WP_215583406.1
Methylomonas paludis
VanZ-2
MBQ4519298.1
Bacteroidaceae bacterium
VanZ-2
HEB56406.1
Gammaproteobacteria bacterium
VanZ-2
OQA44309.1
Chloroflexi bacterium ADurb.Bin325
VanZ-2
CDF12659.1
Eubacterium sp. CAG:581
VanZ-2
MBR5747483.1
Prevotella sp.
VanZ-2
MBQ4524905.1
Bacteroidaceae bacterium
VanZ-2
MBI2451450.1
Candidatus Pacearchaeota archaeon
VanZ-2
MBU0759362.1
Candidatus Omnitrophota bacterium
VanZ-2
MBP6994903.1
Phycicoccus sp.
VanZ-2
MBV1906710.1
Pseudomonadales bacterium
VanZ-2
MBR2395362.1
Bacteroidaceae bacterium
VanZ-2
WP_115418007.1
Erythrobacter aureus
VanZ-2
NLA30101.1
Propionibacterium sp.
VanZ-2
MBS1127187.1
Nitrospirota bacterium
VanZ-2
MBU1043487.1
Candidatus Omnitrophota bacterium
VanZ-2
WP_051626125.1
Kozakia baliensis
VanZ-2
PLX80856.1
Desulfuromonas sp.
VanZ-2
GGL02183.1
Salinibacterium xinjiangense
VanZ-2
GFI17198.1
Lachnospiraceae bacterium
VanZ-2
PSP60480.1
Halobacteriales archaeon QH_8_67_36
VanZ-2
MBU1366726.1
Candidatus Omnitrophota bacterium
VanZ-2
MBQ2298506.1
Bacteroidaceae bacterium
VanZ-2
WP_204986493.1
Actinotalea caeni
VanZ-2
MBL8533086.1
Betaproteobacteria bacterium
VanZ-2
MBD3250003.1
Candidatus Paceibacterota bacterium
VanZ-2
MAR92935.1
Pseudomonadales bacterium
VanZ-2
NBI92284.1
Lachnospiraceae bacterium
VanZ-2
MBD3368789.1
Candidatus Fermentibacterota bacterium
VanZ-2
MBR7111982.1
Clostridia bacterium
VanZ-2
MBM3332069.1
candidate division WOR-3 bacterium
VanZ-2
EPR43797.1
Desulfovibrio sp. X2
VanZ-2
WP_015048368.1
Simiduia agarivorans SA1 = DSM 21679
VanZ-2
MBK6650149.1
Candidatus Defluviibacillus avedoerensis
VanZ-2
CAB1065371.1
Olavius sp. associated proteobacterium Delta 1
VanZ-2
MBI2291992.1
Betaproteobacteria bacterium
VanZ-2
MBR6176660.1
Bacteroidales bacterium
VanZ-2
MBQ0029195.1
Candidatus Physcocola equi
VanZ-2
MCB8995492.1
Bacteroidales bacterium
VanZ-2
WP_184664889.1
Texcoconibacillus texcoconensis
VanZ-2
WP_193916757.1
Plectonema cf. radiosum LEGE 06105
VanZ-2
CDO59099.1
Candidatus Phaeomarinobacter ectocarpi
VanZ-2
HIU39047.1
Candidatus Limisoma intestinavium
VanZ-2
MBQ9471388.1
Bacteroidales bacterium
VanZ-2
MCA6436173.1
Bacteroidota bacterium
VanZ-2
PLX05356.1
Marinilabiliales bacterium
VanZ-2
MBO4397023.1
Bacteroidaceae bacterium
VanZ-2
MBT4349200.1
bacterium
VanZ-2
MBQ8386812.1
Paludibacteraceae bacterium
VanZ-2
MBX3740085.1
Akkermansiaceae bacterium
VanZ-2
MBT7958214.1
Akkermansiaceae bacterium
VanZ-2
MBI4426755.1
Candidatus Kerfeldbacteria bacterium
VanZ-2
MBS5146914.1
Collinsella intestinalis
VanZ-2
WP_114926604.1
Ornithinimicrobium avium
VanZ-2
MBQ6240751.1
Bacillota bacterium
VanZ-2
MBO4662961.1
Bacteroidaceae bacterium
VanZ-2
RPG17891.1
Hyphomonadaceae bacterium TMED125
VanZ-2
MBO5750596.1
Bacteroidaceae bacterium
VanZ-2
MCB5230651.1
Candidatus Cloacimonadota bacterium
VanZ-2
MBS1115140.1
Nitrospirota bacterium
VanZ-2
MBQ2164040.1
Muribaculaceae bacterium
VanZ-2
MBO8437661.1
Candidatus Caccoplasma merdipullorum
VanZ-2
MBF0187442.1
Magnetococcales bacterium
VanZ-2
TVZ40984.1
Alteromonadaceae bacterium 2753L.S.0a.02
VanZ-2

MBC8002178.1	*Verrucomicrobiales bacterium*	VanZ-2
MBM4402107.1	*Candidatus Cloacimonadota bacterium*	VanZ-2
MBN2780747.1	*Fidelibacterota bacterium*	VanZ-2
MBR1786287.1	*Paludibacteraceae bacterium*	VanZ-2
RCK77373.1	*Ignavibacteriota bacterium*	VanZ-2
WP_079525714.1	*Solibacillus isronensis*	VanZ-2
MBJ12009.1	*Fidelibacterota bacterium*	VanZ-2
MCE3003173.1	*Lysobacteraceae bacterium*	VanZ-2
NIB99859.1	*Halobacterium sp. R2-5*	VanZ-2
PUA29099.1	*Cellvibrio sp. 79*	VanZ-2
MBI3307523.1	*Candidatus Omnitrophota bacterium*	VanZ-2
HIP83173.1	*Desulfocapsa sulfexigens*	VanZ-2
MBO7260304.1	*Bacteroidaceae bacterium*	VanZ-2
MBI3317629.1	*Candidatus Omnitrophota bacterium*	VanZ-2
MCE1154852.1	*Bacteroidales bacterium*	VanZ-2
MBN2460010.1	*Candidatus Cloacimonadota bacterium*	VanZ-2
WP_143564277.1	*Shewanella hanedai*	VanZ-2
MCE2868912.1	*Lysobacteraceae bacterium*	VanZ-2
MBQ6959315.1	*Bacteroidales bacterium*	VanZ-2
MBR5854757.1	*Paludibacteraceae bacterium*	VanZ-2
MBQ7901907.1	*Clostridia bacterium*	VanZ-2
KYF72278.1	*Sorangium cellulosum*	VanZ-2
MBI5965278.1	*Chloroflexota bacterium*	VanZ-2
MBO4689683.1	*Paludibacteraceae bacterium*	VanZ-2
MBV5306502.1	*Desulfobulbaceae bacterium*	VanZ-2
MCB1331813.1	*Maritimibacter sp.*	VanZ-2
MBS4001503.1	*Desulfobulbaceae bacterium*	VanZ-2
PCJ16949.1	*Candidatus Cloacimonadota bacterium*	VanZ-2
MBI5751658.1	*Hydrogenophilales bacterium*	VanZ-2
WP_003763643.1	*Neisseria sicca*	VanZ-2
TVR53589.1	*Spirochaetaceae bacterium*	VanZ-2
WP_066204037.1	*Bacillus sp. FJAT-27225*	VanZ-2
WP_003756262.1	*Neisseria sicca*	VanZ-2
NLG19963.1	*Actinomycetales bacterium*	VanZ-2
HCM15601.1	*Candidatus Cloacimonas sp.*	VanZ-2
MBP5458448.1	*Paludibacteraceae bacterium*	VanZ-2
HBI14567.1	*Desulfobulbaceae bacterium*	VanZ-2
NDC30114.1	*Bacteroidota bacterium*	VanZ-2
MBP6344697.1	*Neisseriaceae bacterium*	VanZ-2
PWB59288.1	*Nitrosomonadales bacterium*	VanZ-2
WP_077036665.1	*Pelomonas sp. KK5*	VanZ-2
ROL60245.1	*Bacteroidetes/Chlorobi group bacterium MS-B_bin-24*	VanZ-2
MAH52862.1	*Pseudomonadota bacterium*	VanZ-2
MBX3466375.1	*Planctomycetota bacterium*	VanZ-2
TFB11597.1	*Fidelibacterota bacterium MT.SAG.2*	VanZ-2
MBQ2687622.1	*Clostridia bacterium*	VanZ-2
MBP5575721.1	*Treponema sp.*	VanZ-2
MBQ5379963.1	*Paraprevotella sp.*	VanZ-2
MBQ7528923.1	*bacterium*	VanZ-2
MBQ8282218.1	*Paraprevotella sp.*	VanZ-2
SDZ73741.1	*Lachnospiraceae bacterium NK3A20*	VanZ-2
RLD13355.1	*Candidatus Zhuqueibacterota bacterium*	VanZ-2
MBC8210374.1	*Gammaproteobacteria bacterium*	VanZ-2
OQY08172.1	*Desulfobacteraceae bacterium 4572_123*	VanZ-2
NOZ47971.1	*Chlorobiota bacterium*	VanZ-2
HHN34787.1		VanZ-2
MBC8184664.1	*Candidatus Zhuqueibacterota bacterium*	VanZ-2
MBR1508470.1	*Eubacterium sp.*	VanZ-2
WP_089804644.1	*Halogeometricum rufum*	VanZ-2
MBR4120169.1	*Spirochaetales bacterium*	VanZ-2
MBK8575872.1	*Elusimicrobiota bacterium*	VanZ-2
HBK58318.1	*Spirochaetaceae bacterium*	VanZ-2
RLA16363.1	*Gammaproteobacteria bacterium*	VanZ-2
MBI2793417.1	*Ignavibacteria bacterium*	VanZ-2
MCB4756429.1	*Candidatus Obscuribacterium magneticum*	VanZ-2
MBI2907369.1	*Chloroflexota bacterium*	VanZ-2
MBN1894060.1	*bacterium*	VanZ-2
HCW55866.1	*Erysipelotrichaceae bacterium*	VanZ-2
MBJ7521214.1	*Solirubrobacteraceae bacterium*	VanZ-2
NTW51100.1	*Chlorobiaceae bacterium*	VanZ-2
NNJ25309.1	*Alienimonas chondri*	VanZ-2
MBP7652016.1	*Candidatus Babelota bacterium*	VanZ-2
MBO4696652.1	*Lachnospiraceae bacterium*	VanZ-2
MBI4477540.1	*Acidobacteriota bacterium*	VanZ-2
HHC07042.1		VanZ-2
MBQ2730512.1	*Clostridia bacterium*	VanZ-2
MBR2350281.1	*Clostridia bacterium*	VanZ-2
MBN9298662.1	*Filimonas sp.*	VanZ-2
MZP83069.1	*Bacteroidales bacterium*	VanZ-2
MTI68266.1	*Bacillota bacterium*	VanZ-2
CCZ20195.1	*Candidatus Apopatosoma intestinale*	VanZ-2
MCD6306806.1	*Deltaproteobacteria bacterium*	VanZ-2
NJO14434.1	*Thioploca sp.*	VanZ-2
PIV71464.1	*Rhodocyclales bacterium CG17_big_fil_post_rev_8_21_14_2_50_68_7*	VanZ-2
OIP62887.1	*Nitrospirae bacterium CG2_30_70_394*	VanZ-2
MBE6350720.1	*Spirochaetaceae bacterium*	VanZ-2
RZO95761.1	*Gammaproteobacteria bacterium*	VanZ-2
WP_014514832.1	*Thermus sp.*	VanZ-2
UCH36712.1	*Armatimonadota bacterium*	VanZ-2
MCC5964666.1	*Natronohydrobacter sp.*	VanZ-2
WP_011944135.1	*Thermotoga sp. 38H-to*	VanZ-2
HCE15006.1	*Clostridiales bacterium*	VanZ-2
RKX24270.1	*Candidatus Zixiibacteriota bacterium*	VanZ-2

MBC77674.1	*Halobacteriovoraceae bacterium*	VanZ-2
WP_170185525.1	*Lapillicoccus jejuensis*	VanZ-2
WP_067298202.1	*Marinobacterium profundum*	VanZ-2
RPI02722.1	*Calditrichota bacterium*	VanZ-2
WP_052242334.1	*Novosphingobium sp. ES2-1*	VanZ-2
MBQ9695904.1	*Oscillospiraceae bacterium*	VanZ-2
MBD5265010.1	*Bacteroides sp.*	VanZ-2
WP_009133016.1	*Alistipes indistinctus*	VanZ-2
MBU3839477.1	*Candidatus Ruminococcus intestinipullorum*	VanZ-2
PIN77019.1	*Candidatus Woesearchaeota archaeon CG10_big_fil_rev_8_21_14_0_10_34_8*	VanZ-2
MBN1132223.1	*Bacteroidales bacterium*	VanZ-2
RKY33271.1	*Candidatus Omnitrophota bacterium*	VanZ-2
MCD8215026.1	*Clostridiales bacterium*	VanZ-2
HIZ62325.1	*Candidatus Gemmiger avistercoris*	VanZ-2
KJE45782.1		VanZ-2
OYW78926.1	*Asticcacaulis sp. 32-58-5*	VanZ-2
OIP82797.1	*Porphyromonadaceae bacterium CG2_30_38_12*	VanZ-2
PYX98444.1	*Acidobacteriota bacterium*	VanZ-2
MBC7964481.1	*Fuerstiella sp.*	VanZ-2
MBP1864788.1	*Clostridium tetanomorphum*	VanZ-2
KPK07496.1	*Anaerolineae bacterium SG8_19*	VanZ-2
ANX13724.1	*Fictibacillus arsenicus*	VanZ-2
NLK67103.1	*Campylobacteraceae bacterium*	VanZ-2
MBU1298635.1	*Bacteroidota bacterium*	VanZ-2
MBI9054708.1	*Bacteroidales bacterium*	VanZ-2
MCB1837417.1	*Alcanivoracaceae bacterium*	VanZ-2
NDB95617.1	*Verrucomicrobiota bacterium*	VanZ-2
WP_214393197.1	*Aminobacter anthyllidis*	VanZ-2
MBS2017324.1	*Deltaproteobacteria bacterium*	VanZ-2
NDA98532.1	*Flavobacteriia bacterium*	VanZ-2
MCA0179682.1	*Actinomycetota bacterium*	VanZ-2
MBL1430393.1	*Robiginitomaculum sp.*	VanZ-2
WP_100156787.1	*Snodgrassella alvi*	VanZ-2
MBP1713639.1	*Deltaproteobacteria bacterium*	VanZ-2
MBQ7421714.1	*Prevotella sp.*	VanZ-2
WP_213167493.1		VanZ-2
MBQ7438099.1	*Oscillospiraceae bacterium*	VanZ-2
HIR28042.1	*Candidatus Choladousia intestinigallinarum*	VanZ-2
WP_007505269.1	*Caldalkalibacillus thermarum TA2.A1*	VanZ-2
MAE70276.1	*Gemmatimonadota bacterium*	VanZ-2
WP_162213207.1	*Nostocoides australiense Ben110*	VanZ-2
MSO22577.1	*Acidobacteriota bacterium*	VanZ-2
HGH43132.1		VanZ-2
MSQ61575.1	*Dehalococcoidia bacterium*	VanZ-2
MAU69185.1	*Gammaproteobacteria bacterium*	VanZ-2
EEY06621.1	*Brucella pinnipedialis M163/99/10*	VanZ-2
MBI5267620.1	*Candidatus Zixiibacteriota bacterium*	VanZ-2
QAA76335.1	*Candidatus Bipolaricaulis sibiricus*	VanZ-2
PCJ63086.1	*Planctomycetota bacterium*	VanZ-2
MBR6290562.1	*Clostridia bacterium*	VanZ-2
PID88858.1	*Bacteroidia bacterium*	VanZ-2
MBQ01607.1	*Acidobacteriota bacterium*	VanZ-2
MBI5868365.1	*Candidatus Zixiibacteriota bacterium*	VanZ-2
MCD6380261.1	*bacterium*	VanZ-2
MBM4382553.1	*Deltaproteobacteria bacterium*	VanZ-2
MBU4308781.1	*Nanoarchaeota archaeon*	VanZ-2
WP_204669701.1		VanZ-2
MBS7240527.1	*Acetatifactor sp.*	VanZ-2
NBP87718.1	*Planctomycetia bacterium*	VanZ-2
MBN2117198.1	*Anaerolineales bacterium*	VanZ-2
GGA35138.1	*Pelagibacterium lentulum*	VanZ-2
MBK9725552.1	*Candidatus Lutibacillus vidarii*	VanZ-2
MBN1598481.1	*Bacteroidales bacterium*	VanZ-2
MBN8445621.1	*Gammaproteobacteria bacterium*	VanZ-2
RPI29126.1	*Acidobacteriota bacterium*	VanZ-2
MBE0674700.1	*Bacteroidales bacterium*	VanZ-2
RPI20246.1	*Acidobacteriota bacterium*	VanZ-2
NDJ78220.1	*Chloroflexota bacterium*	VanZ-2
MCC7046258.1	*Alphaproteobacteria bacterium*	VanZ-2
MCB0833674.1	*Bacteroidota bacterium*	VanZ-2
MCC7295686.1	*Acidobacteriota bacterium*	VanZ-2
WP_204119356.1	*Lacticaseibacillus suilingensis*	VanZ-2
MBA3726524.1	*Armatimonadota bacterium*	VanZ-2
HIQ53945.1	*Halopseudomonas pachastrellae*	VanZ-2
NBQ55589.1	*Verrucomicrobiota bacterium*	VanZ-2
MCB2197330.1	*Bacteroidota bacterium*	VanZ-2
HHN75411.1	*Acidobacteriota bacterium*	VanZ-2
OUW81277.1	*Gammaproteobacteria bacterium TMED222*	VanZ-2
RAL56365.1	*Candidatus Gracilibacteria bacterium GN02-873*	VanZ-2
MBA3509035.1	*Thermoleophilaceae bacterium*	VanZ-2
MAI89259.1	*Ponticaulis sp.*	VanZ-2
MXW25341.1	*Dehalococcoidia bacterium*	VanZ-2
MBS1269384.1	*Gammaproteobacteria bacterium*	VanZ-2
PUA31529.1	*Candidatus Terraquivivens tikiterensis*	VanZ-2
MCD6424493.1	*Anaerolineales bacterium*	VanZ-2
NOQ25996.1	*Bacteroidales bacterium*	VanZ-2
WP_190558660.1	*Anabaena sphaerica FACHB-251*	VanZ-2
MBI2412767.1	*Deltaproteobacteria bacterium*	VanZ-2
MBL7972507.1	*Prolixibacteraceae bacterium*	VanZ-2
MBI2092913.1	*Deltaproteobacteria bacterium*	VanZ-2
MBA3600789.1	*Acidobacteriota bacterium*	VanZ-2
SEH47685.1	*Ruminococcus flavefaciens*	VanZ-2
WP_163577139.1	*Halomonas faecis*	VanZ-2

MBL8013699.1	*Candidatus Omnitrophota bacterium*	VanZ-2
MCD6250426.1	*Candidatus Zixiibacteriota bacterium*	VanZ-2
PXF57515.1	*Deltaproteobacteria bacterium*	VanZ-2
HFD13605.1	*Crenotrichaceae bacterium*	VanZ-2
MBN1221919.1	*Candidatus Aminicenantes bacterium*	VanZ-2
WP_163935973.1	*Paraferrimonas sp. SM1919*	VanZ-2
HJA29012.1	*Candidatus Olsenella pullicola*	VanZ-2
MBC8333900.1	*Candidatus Desulfolinea nitratireducens*	VanZ-2
HCN06061.1	*Bacteroidota bacterium*	VanZ-2
WP_154646226.1	*Timonella senegalensis JC301*	VanZ-2
SYZ73928.1	*Candidatus Zixiibacteriota bacterium*	VanZ-2
RUA03313.1	*Deltaproteobacteria bacterium*	VanZ-2
EQB63025.1	*candidate division Zixibacteria bacterium RBG-1*	VanZ-2
HEB35929.1	*Candidatus Aminicenantes bacterium*	VanZ-2
OQA99949.1	*Bacteroidetes bacterium ADurb.Bin217*	VanZ-2
OFX19933.1	*Bacteroidetes bacterium GWA2_31_9b*	VanZ-2
OFX47616.1	*Bacteroidetes bacterium GWA2_33_15*	VanZ-2
MBI4481631.1	*Acidobacteriota bacterium*	VanZ-2
MBA4186827.1	*Planctomycetaceae bacterium*	VanZ-2
RUM71386.1	*Sulfurovum sp.*	VanZ-2
WP_087377080.1	*Anaeromassilibacillus sp. An172*	VanZ-2
OFX85956.1	*Bacteroidetes bacterium GWE2_32_14*	VanZ-2
WP_060759160.1	*Propionibacterium freudenreichii*	VanZ-2
TFH05662.1	*Spirochaetales bacterium*	VanZ-2
OFY27660.1	*Bacteroidetes bacterium GWF2_35_48*	VanZ-2
OFY34460.1	*Bacteroidetes bacterium GWF2_38_335*	VanZ-2
HBG76096.1	*Clostridiales bacterium*	VanZ-2
PKP39210.1	*Bacteroidetes bacterium HGW-Bacteroidetes-15*	VanZ-2
WP_060572619.1		VanZ-2
MBR5596277.1	*Lachnospiraceae bacterium*	VanZ-2
MBT8396017.1	*Gemmatimonadota bacterium*	VanZ-2
MBN9210812.1	*Microbacterium sp.*	VanZ-2
MBT8441582.1	*Gammaproteobacteria bacterium*	VanZ-2
RMF28725.1	*Chloroflexota bacterium*	VanZ-2
MBI3931911.1	*Acidobacteriota bacterium*	VanZ-2
MBN9534562.1	*Alphaproteobacteria bacterium*	VanZ-2
RPH62967.1	*Chloroflexota bacterium*	VanZ-2
OHB32271.1	*Desulfuromonadaceae bacterium GWC2_58_13*	VanZ-2
WP_152482211.1		VanZ-2
MBQ7546440.1	*Clostridia bacterium*	VanZ-2
MBQ7939528.1	*Clostridia bacterium*	VanZ-2
ACL71210.1	*Thioalkalivibrio sulfidiphilus HL-EbGr7*	VanZ-2
ONI63285.1	*Microbacterium sp. CSI-V*	VanZ-2
MCD6268756.1	*Candidatus Tharpella sp.*	VanZ-2
MBI9032700.1	*bacterium*	VanZ-2
WP_204395690.1	*Agromyces aurantiacus*	VanZ-2
MBI3074301.1	*Deltaproteobacteria bacterium*	VanZ-2
MBR6513652.1	*Clostridia bacterium*	VanZ-2
MBS3819806.1	*bacterium*	VanZ-2
MSO53259.1	*Acidobacteriota bacterium*	VanZ-2
MBU1690376.1	*Gammaproteobacteria bacterium*	VanZ-2
WP_128218890.1		VanZ-2
MCB2219458.1	*Bacteroidota bacterium*	VanZ-2
OGT95171.1	*Gemmatimonadetes bacterium GWC2_71_9*	VanZ-2
MBI5235832.1	*Deltaproteobacteria bacterium*	VanZ-2
RUM45802.1	*Hydrogenimonas sp.*	VanZ-2
MBI5498313.1	*Deltaproteobacteria bacterium*	VanZ-2
KEQ19592.1	*Endozoicomonas numazuensis*	VanZ-2
MBE3116287.1	*Candidatus Bathyarchaeota archaeon*	VanZ-2
MBI5378629.1	*Nitrospirota bacterium*	VanZ-2
MBV6647302.1	*Cyclobacteriaceae bacterium HetDA_MAG_MS6*	VanZ-2
RPJ86064.1	*Acidobacteriota bacterium*	VanZ-2
MBE0640004.1	*Bacteroidales bacterium*	VanZ-2
MBW3541026.1	*Planctomycetota bacterium*	VanZ-2
NOZ91495.1	*Campylobacterota bacterium*	VanZ-2
MBC7941353.1	*Rubrivivax sp.*	VanZ-2
MBA2440664.1	*Thermoleophilaceae bacterium*	VanZ-2
HHS98267.1	*Chloroflexota bacterium*	VanZ-2
OHB85153.1	*Planctomycetes bacterium RBG_16_64_12*	VanZ-2
MBK84860.1	*Gammaproteobacteria bacterium*	VanZ-2
MBK5295063.1	*Terriglobia bacterium*	VanZ-2
WP_196259239.1	*Pelagibacterium limicola*	VanZ-2
OGP66562.1	*Deltaproteobacteria bacterium RBG_13_47_9*	VanZ-2
MBC7757113.1	*Methylotenera sp.*	VanZ-2
WP_182239871.1	*Colwellia sp. MB02u-10*	VanZ-2
PYV44499.1	*Acidobacteriota bacterium*	VanZ-2
MBA3785178.1	*Acidobacteriota bacterium*	VanZ-2
MBM4343639.1	*Deltaproteobacteria bacterium*	VanZ-2
PIP98266.1	*Rhodobacterales bacterium CG18_big_fil_WC_8_21_14_2_50_71_9*	VanZ-2
MBW2978064.1	*Candidatus Woesearchaeota archaeon*	VanZ-2
WP_162891361.1	*Aeromicrobium sp. A1-2*	VanZ-2
OHE21769.1	*Syntrophobacterales bacterium RBG_19FT_COMBO_59_10*	VanZ-2
MBO5238190.1	*Lachnospiraceae bacterium*	VanZ-2
AFV97061.1	*Candidatus Sulfuricurvum sp. RIFRC-1*	VanZ-2
MBN2617681.1	*Spirochaetales bacterium*	VanZ-2
AOE06383.1		VanZ-2
MBV6520869.1	*Gemmatimonadaceae bacterium*	VanZ-2
KPL17588.1	*Bacteroides sp. SM23_62*	VanZ-2
WP_087376922.1	*Anaeromassilibacillus sp. An172*	VanZ-2
NCB85461.1	*Bacteroidia bacterium*	VanZ-2
WP_162199861.1	*Pyrinomonas methylaliphatogenes*	VanZ-2
HIM93682.1	*Campylobacterales bacterium*	VanZ-2
MAJ83873.1	*Gammaproteobacteria bacterium*	VanZ-2

MBD3234894.1	*Candidatus Zixiibacteriota bacterium*	VanZ-
NRA26522.1	*Opitutales bacterium*	VanZ-
RLG32415.1	*Methanosarcinales archaeon*	VanZ-
MCC6707730.1	*Gammaproteobacteria bacterium*	VanZ-
MBC7797763.1	*Pyrinomonadaceae bacterium*	VanZ-
HEC83050.1	*Bacillota bacterium*	VanZ-
MBP6002356.1	*Pyrinomonadaceae bacterium*	VanZ-
MBK6286468.1	*Gammaproteobacteria bacterium*	VanZ-
TVP96352.1	*Roseinatronobacter sp.*	VanZ-
MCD6320476.1	*Candidatus Desulfofervidaceae bacterium*	VanZ-
MBW1765357.1	*Deltaproteobacteria bacterium*	VanZ-
HIU67914.1	*Candidatus Caccomorpha excrementavium*	VanZ-
MBR4776877.1	*Lachnospiraceae bacterium*	VanZ-
MBW2078929.1	*Deltaproteobacteria bacterium*	VanZ-
MBI5219130.1	*Bacteroidia bacterium*	VanZ-
MBI5232109.1	*Coriobacteriales bacterium*	VanZ-
MBU0544454.1	*Pseudomonadota bacterium*	VanZ-
HGY57031.1	*Caldithrix abyssi*	VanZ-
WP_154692641.1	*Novosphingobium sp. Gsoil 351*	VanZ-
QCD45181.1	*Campylobacter mucosalis CCUG 21559*	VanZ-
MCA9485482.1	*Nanoarchaeota archaeon*	VanZ-
NIA08562.1	*Nitrospiraceae bacterium*	VanZ-
MCC6925062.1	*Novosphingobium sp.*	VanZ-
MCC6827193.1	*Novosphingobium sp.*	VanZ-
MBP7474419.1	*Pyrinomonadaceae bacterium*	VanZ-
TVP60448.1	*Gemmatimonadales bacterium*	VanZ-
MBD3223347.1	*Caldithrix sp.*	VanZ-
MBA4160581.1	*Novosphingobium sp.*	VanZ-
TNF46979.1	*Bacteroidota bacterium*	VanZ-
NLA94121.1	*Bacteroidales bacterium*	VanZ-
PIN78001.1	*Candidatus Woesearchaeota archaeon CG10_big_fil_rev_8_21_14_0_10_34_12*	VanZ-
MBK7051803.1	*Candidatus Rhodoferax bjergmarkensis*	VanZ-
WP_128101620.1	*Paenibacillus sp. DCT19*	VanZ-
HDY87631.1	*bacterium*	VanZ-
HDV38965.1		VanZ-
RME29446.1	*Candidatus Zixiibacteriota bacterium*	VanZ-
MCE1185692.1	*Rhodocyclales bacterium*	VanZ-
ERG99686.1	*Haloquadratum sp. J07HQX50*	VanZ-
MBS0305049.1	*Pseudomonadota bacterium*	VanZ-
WP_153796124.1	*Foetidibacter luteolus*	VanZ-
MCC6935019.1	*Thermomicrobiales bacterium*	VanZ-
HIO66078.1	*Planctomycetota bacterium*	VanZ-
MBT3270238.1	*Candidatus Poribacteria bacterium*	VanZ-
MBK8229950.1	*Candidatus Eiseniibacteriota bacterium*	VanZ-
HHQ44734.1	*Candidatus Altiarchaeales archaeon*	VanZ-
MBN1951103.1	*Bacteroidales bacterium*	VanZ-
MBS0482935.1	*Pseudomonadota bacterium*	VanZ-
MAM71453.1	*Gammaproteobacteria bacterium*	VanZ-
MBS0575514.1	*Pseudomonadota bacterium*	VanZ-
NLA49817.1	*Bacteroidales bacterium*	VanZ-
WP_115751218.1	*Oceanobacillus chungangensis*	VanZ-
MQY53532.1	*Bacillota bacterium*	VanZ-
NLK03616.1	*Clostridiales bacterium*	VanZ-
MBP7272672.1	*Saprospiraceae bacterium*	VanZ-
MBT3637528.1	*Opitutia bacterium*	VanZ-
QDU25175.1	*Anatilimnocola aggregata*	VanZ-
HDP74450.1	*Bacteroidales bacterium*	VanZ-
MCC2674911.1	*Ramlibacter sp.*	VanZ-
MBO5597843.1	*Oribacterium sp.*	VanZ-
MCA9810801.1	*Candidatus Dadabacteria bacterium*	VanZ-
NTU54493.1	*Chlorobiaceae bacterium*	VanZ-
RRJ29590.1	*Halocatena pleomorpha*	VanZ-
WP_183970281.1	*Quisquiliibacterium transsilvanicum*	VanZ-
PIR17139.1	*Deltaproteobacteria bacterium CG11_big_fil_rev_8_21_14_0_20_49_13*	VanZ-
MBR5817159.1	*Clostridia bacterium*	VanZ-
MBE9531401.1	*Pseudomonadota bacterium*	VanZ-
PCI76905.1	*SAR86 cluster bacterium*	VanZ-
GBD07542.1	*bacterium HR21*	VanZ-
NLY38263.1	*Bacillota bacterium*	VanZ-
MBF2025230.1	*Leptolyngbyaceae cyanobacterium C42_A2020_001*	VanZ-
HID86033.1	*Anaerolineae bacterium*	VanZ-
MBD3295918.1	*Candidatus Omnitrophota bacterium*	VanZ-
KPK88538.1	*Deltaproteobacteria bacterium SM23_61*	VanZ-
MBI2106155.1	*Candidatus Woesearchaeota archaeon*	VanZ-
MBW4692455.1	*Lyngbya sp. HA4199-MV5*	VanZ-
MBM9554004.1		VanZ-
GBE09203.1	*bacterium BMS3Abin11*	VanZ-
MSW84816.1	*Actinomycetota bacterium*	VanZ-
MBR3154649.1	*Lachnospiraceae bacterium*	VanZ-
TMM13856.1	*Actinomycetota bacterium*	VanZ-
GJE24366.1	*Methylobacterium mesophilicum*	VanZ-
MBE7184166.1	*Methylobacterium mesophilicum*	VanZ-
GJD96537.1	*Methylobacterium iners*	VanZ-
MBC8277880.1	*FCB group bacterium*	VanZ-
WP_066021807.1	*Clostridium sp. CT7*	VanZ-
MBS0262949.1	*Planctomycetota bacterium*	VanZ-
MBI2507924.1	*Candidatus Woesearchaeota archaeon*	VanZ-
HAM30559.1	*Erysipelotrichaceae bacterium*	VanZ-
NQW26417.1	*Flammeovirgaceae bacterium*	VanZ-
NLK18061.1	*Clostridiales bacterium*	VanZ-
MAG45381.1	*Nanoarchaeota archaeon*	VanZ-
OYD15630.1	*candidate division WOR-3 bacterium JGI_Cruoil_03_51_56*	VanZ-
MBI5353714.1	*Chloroflexota bacterium*	VanZ-

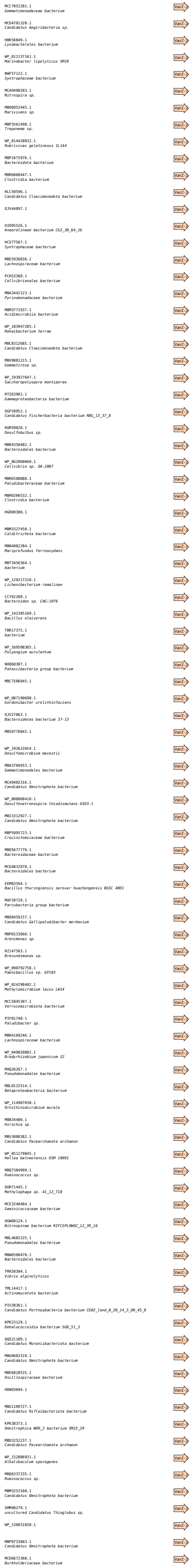
MCC7052281.1
Gemmatimonadaceae bacterium
MCD4701320.1
Candidatus Aegiribacteria sp.
HBK56849.1
Lysobacterales bacterium
WP_012137161.1
Marinobacter lipolyticus SM19
NWF57122.1
Syntrophaceae bacterium
MCA9498203.1
Nitrospira sp.
MBO6852445.1
Marivivens sp.
MBP3561498.1
Treponema sp.
WP_014428032.1
Rubrivivax gelatinosus IL144
MBP1675976.1
Bacteroidota bacterium
MBR6808447.1
Clostridia bacterium
RLC50596.1
Candidatus Cloacimonadota bacterium
OJV44897.1
OIO95526.1
Anaerolineae bacterium CG2_30_64_16
HCS77567.1
Syntrophaceae bacterium
MBE5936836.1
Lachnospiraceae bacterium
PCH53360.1
Cellvibrionales bacterium
MBA3442123.1
Pyrinomonadaceae bacterium
MBM3771937.1
Acidimicrobiia bacterium
WP_183947285.1
Rehaibacterium terrae
MBC8312685.1
Candidatus Cloacimonadota bacterium
MBV9881215.1
Gemmatirosa sp.
WP_193927647.1
Saccharopolyspora montiporae
RTZ63901.1
Gammaproteobacteria bacterium
OGF59952.1
Candidatus Fischerbacteria bacterium RBG_13_37_8
RUM39020.1
Desulfobulbus sp.
MBR4350402.1
Bacteroidales bacterium
WP_062060660.1
Cellvibrio sp. OA-2007
MBR6508888.1
Paludibacteraceae bacterium
MBR0206532.1
Clostridia bacterium
HGR00386.1
MBM3327450.1
Calditrichota bacterium
MBN4082284.1
Mariprofundus ferrooxydans
MBT3456364.1
bacterium
WP_129217210.1
Lichenibacterium ramalinae
CCY92209.1
Bacteroides sp. CAG:1076
WP_142305169.1
Bacillus oleivorans
TBR17375.1
bacterium
WP_169508385.1
Polyangium aurulentum
NOQ68387.1
Patescibacteria group bacterium
MBC7586945.1
WP_087190698.1
Gordonibacter urolithinfaciens
OJV27063.1
Bacteroidetes bacterium 37-13
MBS9776842.1
WP_192622654.1
Desulfomicrobium macestii
MBA3760453.1
Gemmatimonadales bacterium
MCA9402316.1
Candidatus Omnitrophota bacterium
WP_008868410.1
Desulfonatronospira thiodismutans ASO3-1
MBI3312927.1
Candidatus Omnitrophota bacterium
MBP5995723.1
Crocinitomicaceae bacterium
MBO5677776.1
Bacteroidaceae bacterium
MCD4832970.1
Bacteroidales bacterium
EEM83594.1
Bacillus thuringiensis serovar huazhongensis BGSC 4BD1
MAF20729.1
Parcubacteria group bacterium
MBO8459157.1
Candidatus Gallipaludibacter merdavium
MBP6533660.1
Arenimonas sp.
RZJ47503.1
Brevundimonas sp.
WP_090792758.1
Paenibacillus sp. GP183
WP_024298402.1
Methylomicrobium lacus LW14
MCC5845307.1
Verrucomicrobiota bacterium
PIF01748.1
Paludibacter sp.
MBR4169246.1
Lachnospiraceae bacterium
WP_049820002.1
Bradyrhizobium japonicum 22
MAQ26267.1
Pseudomonadales bacterium
MBL0122514.1
Betaproteobacteria bacterium
WP_114907038.1
Ornithinimicrobium murale
MBB34400.1
Hirschia sp.
MBS3088382.1
Candidatus Pacearchaeota archaeon
WP_051279845.1
Hellea balneolensis DSM 19091
MBQ7384999.1
Ruminococcus sp.
OUR71445.1
Methylophaga sp. 41_12_T18
MCE3248484.1
Geminicoccaceae bacterium
OGW09124.1
Nitrospinae bacterium RIFCSPLOWO2_12_39_16
MBL4682225.1
Pseudomonadales bacterium
MBW6500470.1
Bacteroidales bacterium
TMX50304.1
Vibrio alginolyticus
TML14417.1
Actinomycetota bacterium
PIV38361.1
Candidatus Portnoybacteria bacterium CG02_land_8_20_14_3_00_45_8
KPK23129.1
Dehalococcoidia bacterium SG8_51_3
QQS21189.1
Candidatus Moraniibacteriota bacterium
MBU0683319.1
Candidatus Omnitrophota bacterium
MBE6828535.1
Oscillospiraceae bacterium
HDW93694.1
MBU1109727.1
Candidatus Rifleibacteriota bacterium
KPK38373.1
Omnitrophica WOR_2 bacterium SM23_29
MBD3252237.1
Candidatus Pacearchaeota archaeon
WP_152806931.1
Alkalibaculum sporogenes
MBQ6337235.1
Ruminococcus sp.
MBM3253160.1
Candidatus Omnitrophota bacterium
SMM98279.1
uncultured Candidatus Thioglobus sp.
WP_130031830.1
MBP9733603.1
Candidatus Omnitrophota bacterium
MCD6672368.1
Burkholderiaceae bacterium

MBQ3816086.1	*Clostridia bacterium*	VanZ-2
MBS5736603.1	*Clostridiales bacterium*	VanZ-2
MBQ2581582.1	*Ruminococcus sp.*	VanZ-2
MBI2630401.1	*Candidatus Pacearchaeota archaeon*	VanZ-2
MBS1152647.1	*Myxococcaceae bacterium*	VanZ-2
KAB1659346.1	*Pseudoclavibacter sp. CFCC 11306*	VanZ-2
NBV50594.1	*bacterium*	VanZ-2
MBE7202003.1	*Parafilimonas terrae*	VanZ-2
EGR2744379.1	*Vibrio parahaemolyticus*	VanZ-2
PZU92164.1	*Chelatococcus sp.*	VanZ-2
WP_232547802.1	*Propioniciclava soli*	VanZ-2
MBR99090.1	*Flavobacteriaceae bacterium*	VanZ-2
MCB1615359.1	*Pseudomonadales bacterium*	VanZ-2
MCC7548640.1	*Burkholderiales bacterium*	VanZ-2
WP_204105442.1	*Spirulina sp. CCY15215*	VanZ-2
MBK7327664.1	*Candidatus Amarobacter glycogenicus*	VanZ-2
WP_190764611.1	*Spongiibacter pelagi*	VanZ-2
MBS7352563.1	*Muribaculaceae bacterium*	VanZ-2
OYT89688.1	*Burkholderiales bacterium PBB6*	VanZ-2
HCS47475.1	*Candidatus Aminicenantes bacterium*	VanZ-2
MBW4470688.1	*Stenomitos rutilans HA7619-LM2*	VanZ-2
MBX3600927.1	*Rubrivivax sp.*	VanZ-2
WP_231914749.1	*Methylomonas sp. DH-1*	VanZ-2
MBQ1902440.1	*Lachnospiraceae bacterium*	VanZ-2
MBI4945599.1	*Bacteroidota bacterium*	VanZ-2
MBI2042998.1	*Candidatus Pacearchaeota archaeon*	VanZ-2
MBP48038.1	*Myxococcales bacterium*	VanZ-2
MBK6835936.1	*Bacteroidota bacterium*	VanZ-2
MBS4932350.1	*Clostridiales bacterium*	VanZ-2
MCD8212373.1	*Oscillospiraceae bacterium*	VanZ-2
TLZ49072.1	*Methanobacteriota archaeon*	VanZ-2
HAN50340.1	*Lachnospiraceae bacterium*	VanZ-2
WP_156464131.1	*Devosia sp. Leaf420*	VanZ-2
WP_068257047.1	*Janibacter corallicola NBRC 107790*	VanZ-2
HIJ95690.1	*Desulfuromonadales bacterium*	VanZ-2
NNE56882.1	*Hellea sp.*	VanZ-2
MBR4862986.1	*Oscillospiraceae bacterium*	VanZ-2
MBE6806043.1	*Oscillospiraceae bacterium*	VanZ-2
RYE57758.1	*Hyphomicrobiales bacterium*	VanZ-2
MBI1260867.1	*Hyphomicrobiales bacterium*	VanZ-2
KPK94891.1	*bacterium SM23_31*	VanZ-2
WP_183907818.1	*Simiduia aestuariiviva*	VanZ-2
WP_011735639.1	*Pelobacter propionicus DSM 2379*	VanZ-2
EEK83855.1	*Bacillus cereus ATCC 4342*	VanZ-2
WP_182677308.1	*Shewanella sp. SR44-3*	VanZ-2
MBT4825725.1	*Flavobacteriaceae bacterium*	VanZ-2
MBI4115117.1	*Candidatus Omnitrophota bacterium*	VanZ-2
MCC6789908.1	*Hyphomonadaceae bacterium*	VanZ-2
MBN1500932.1	*Spirochaetota bacterium*	VanZ-2
HGU27375.1		VanZ-2
WP_105726981.1		VanZ-2
KJS36589.1	*Hyphomonas sp. BRH_c22*	VanZ-2
MBQ7281027.1	*Bacteroidales bacterium*	VanZ-2
HHN00803.1		VanZ-2
RUM40939.1	*Desulfocapsa sp.*	VanZ-2
PIE60117.1		VanZ-2
HEC13795.1	*Acidiferrobacteraceae bacterium*	VanZ-2
MBP2639076.1	*Bacillota bacterium*	VanZ-2
WP_131448843.1	*Flaviaesturariibacter flavus*	VanZ-2
MBI3312397.1	*Candidatus Omnitrophota bacterium*	VanZ-2
MBI3331296.1	*Candidatus Omnitrophota bacterium*	VanZ-2
MBR3837426.1	*Clostridia bacterium*	VanZ-2
WP_115008509.1	*Moraxella lacunata*	VanZ-2
NWF91912.1	*Syntrophaceae bacterium*	VanZ-2
OON93860.1	*Epulopiscium sp. AS2M-Bin002*	VanZ-2
MBK8971814.1	*Hahellaceae bacterium*	VanZ-2
MBX3518103.1	*Rhodospirillales bacterium*	VanZ-2
MBI2870298.1	*Candidatus Omnitrophota bacterium*	VanZ-2
NOW41723.1		VanZ-2
WP_130067633.1	*Bacillus albus*	VanZ-2
MBS3757356.1	*Desulfobacterales bacterium*	VanZ-2
MBQ9300681.1	*Clostridia bacterium*	VanZ-2
WP_185448694.1		VanZ-2
MBI1953893.1	*Candidatus Omnitrophota bacterium*	VanZ-2
MBP6973051.1	*Syntrophorhabdus sp.*	VanZ-2
MBS1768938.1	*Acidobacteriota bacterium*	VanZ-2
HBN25944.1	*Desulfobacteraceae bacterium*	VanZ-2
HHV65413.1	*Peptococcaceae bacterium*	VanZ-2
MBQ3811513.1	*Kiritimatiellia bacterium*	VanZ-2
MCD6413737.1	*Elusimicrobiota bacterium*	VanZ-2
MBP7829120.1	*Kiritimatiellia bacterium*	VanZ-2
GIC77827.1	*Moritella sp. F1*	VanZ-2
OLD40685.1	*Armatimonadetes bacterium 13_1_40CM_3_65_7*	VanZ-2
MBI3287235.1	*Chloroflexota bacterium*	VanZ-2
MBQ9951656.1	*Clostridia bacterium*	VanZ-2
MTI13678.1	*Sansalvadorimonas verongulae*	VanZ-2
WP_066354228.1	*Aliarcobacter skirrowii*	VanZ-2
MCB9367480.1	*Calditrichota bacterium*	VanZ-2
PKN58567.1	*Deltaproteobacteria bacterium HGW-Deltaproteobacteria-14*	VanZ-2
TDU88250.1	*Kribbella voronezhensis*	VanZ-2
NLK07537.1	*Bacillota bacterium*	VanZ-2
OGP10262.1	*Deltaproteobacteria bacterium GWA2_42_85*	VanZ-2
PIU57623.1	*Deltaproteobacteria bacterium CG07_land_8_20_14_0_80_38_7*	VanZ-2

NLW50567.1	*Candidatus Brocadiaceae bacterium*	VanZ-2
NTU98049.1	*Chlorobiaceae bacterium*	VanZ-2
PPC91160.1	*Methylobacter sp.*	VanZ-2
MBW2568970.1	*Deltaproteobacteria bacterium*	VanZ-2
MBN1117468.1	*Bacteroidales bacterium*	VanZ-2
NOU38644.1	*Ferruginibacter sp.*	VanZ-2
MBM7573938.1		VanZ-2
MBN1522589.1	*Candidatus Auribacterota bacterium*	VanZ-2
NBS19151.1	*Flavobacteriia bacterium*	VanZ-2
MBQ3284374.1	*Ruminococcus sp.*	VanZ-2
MBP1709023.1	*Deltaproteobacteria bacterium*	VanZ-2
WP_116008984.1	*Thalassotalea euphylliae*	VanZ-2
MBO7514868.1	*Lachnospiraceae bacterium*	VanZ-2
OGI18461.1	*Candidatus Melainabacteria bacterium RIFOXYA2_FULL_32_9*	VanZ-2
HHM01770.1	*Caldithrix abyssi*	VanZ-2
WP_206242177.1	*Novosphingobium terrae*	VanZ-2
NBR84599.1	*Verrucomicrobiota bacterium*	VanZ-2
MBA3365462.1	*Actinomycetota bacterium*	VanZ-2
MBD3347478.1	*Chitinivibrionales bacterium*	VanZ-2
NLX12801.1	*Phycisphaerales bacterium*	VanZ-2
WP_115493358.1	*Alteriqipengyuania lutimaris*	VanZ-2
NBQ24125.1	*Verrucomicrobiota bacterium*	VanZ-2
MBO6668037.1	*Parvibaculum sp.*	VanZ-2
MBQ6386726.1	*Lachnospiraceae bacterium*	VanZ-2
MBI5406323.1	*Nitrospirota bacterium*	VanZ-2
MBI4743506.1	*Actinomycetota bacterium*	VanZ-2
HEB65901.1	*Chloroflexota bacterium*	VanZ-2
MBI5443974.1	*Deltaproteobacteria bacterium*	VanZ-2
MBI5048417.1	*Deltaproteobacteria bacterium*	VanZ-2
UCG55922.1	*Phycisphaerales bacterium*	VanZ-2
THD74254.1	*Phenylobacterium sp.*	VanZ-2
MBI5182202.1	*Nitrospirota bacterium*	VanZ-2
MBR2586842.1	*Candidatus Saccharibacteria bacterium*	VanZ-2
RLF13394.1	*Thermoprotei archaeon*	VanZ-2
NLH80165.1	*Phyllobacteriaceae bacterium*	VanZ-2
MBI1910473.1	*Deltaproteobacteria bacterium*	VanZ-2
AQS33366.1		VanZ-2
HEU50518.1		VanZ-2
MBQ0729604.1	*Oleispira antarctica*	VanZ-2
MBU0761497.1	*Candidatus Altiarchaeota archaeon*	VanZ-2
RNA67606.1	*Alteribacter keqinensis*	VanZ-2
MBD5227703.1	*Bacteroidales bacterium*	VanZ-2
MBI3981276.1	*Gemmatimonadota bacterium*	VanZ-2
MBI4542664.1	*Gemmatimonadota bacterium*	VanZ-2
NJD62242.1	*Deltaproteobacteria bacterium*	VanZ-2
MBS1794357.1	*Acidobacteriota bacterium*	VanZ-2
OQX99957.1	*Bacteroidetes bacterium 4572_128*	VanZ-2
MBW2995865.1	*Candidatus Woesearchaeota archaeon*	VanZ-2
MBX2819030.1	*Rhodothermaceae bacterium*	VanZ-2
HAL61871.1	*Chloroflexota bacterium*	VanZ-2
WP_190547908.1		VanZ-2
MBA62546.1	*Planctomycetaceae bacterium*	VanZ-2
ODV00326.1	*Rubrivivax sp. SCN 70-15*	VanZ-2
MBD5163813.1	*Bacteroidales bacterium*	VanZ-2
OQB29375.1	*Bacteroidetes bacterium ADurb.Bin174*	VanZ-2
RKE70457.1	*Pseudorhodoplanes sinuspersici*	VanZ-2
CCZ90567.1	*Clostridium sp. CAG:167*	VanZ-2
WP_026679480.1	*Fictibacillus gelatini DSM 15865*	VanZ-2
MCD6354924.1	*Prolixibacteraceae bacterium*	VanZ-2
QZT36015.1	*Halosquirtibacter xylanolyticus*	VanZ-2
QZE13982.1	*Halosquirtibacter laminarini*	VanZ-2
MBR6044585.1	*Ruminococcus sp.*	VanZ-2
MBA3532163.1	*Ardenticatenales bacterium*	VanZ-2
NBC19624.1	*Alphaproteobacteria bacterium*	VanZ-2
MBQ0103186.1	*Candidatus Colivivens caballi*	VanZ-2
MBN1528338.1	*Thermoleophilaceae bacterium*	VanZ-2
WP_087998760.1	*Gottfriedia solisilvae*	VanZ-2
OAI45034.1	*Rhizomicrobium sp. SCGC AG-212-E05*	VanZ-2
NIM01551.1	*Acidobacteriota bacterium*	VanZ-2
KAA0255171.1	*Acidobacteriota bacterium*	VanZ-2
OYT42167.1	*Candidatus Altiarchaeales archaeon ex4484_43*	VanZ-2
WP_129077176.1	*Anaerobacillus alkaliphilus*	VanZ-2
MBL4871949.1	*Paracoccaceae bacterium*	VanZ-2
HAK42970.1	*Clostridium sp.*	VanZ-2
WP_090794109.1	*Paenibacillus sp. GP183*	VanZ-2
OUU20654.1	*Planctomycetia bacterium TMED53*	VanZ-2
MBL8055753.1	*Anaerolineales bacterium*	VanZ-2
PYQ45391.1	*Acidobacteriota bacterium*	VanZ-2
MBN9585716.1	*Afipia sp.*	VanZ-2
MBS3099306.1	*Candidatus Pacearchaeota archaeon*	VanZ-2
MBP9713560.1	*Sterolibacterium sp.*	VanZ-2
MBW3652655.1	*Actinomycetota bacterium*	VanZ-2
MBS3099596.1	*Candidatus Pacearchaeota archaeon*	VanZ-2
MBM52251.1	*Acidobacteriota bacterium*	VanZ-2
MBX3643851.1	*Rubrivivax sp.*	VanZ-2
MBN2533851.1		VanZ-2
WP_196119934.1		VanZ-2
MBR3008043.1	*Stomatobaculum sp.*	VanZ-2
CAG0951135.1	*Anaerolineae bacterium*	VanZ-2
PPC76337.1	*Pokkaliibacter plantistimulans*	VanZ-2
NRA39413.1	*Planctomycetota bacterium*	VanZ-2
WP_195171009.1	*Phototrophicus methaneseepsis*	VanZ-2
NCB07691.1	*Bacteroidia bacterium*	VanZ-2

MBA2481031.1	*Planctomycetota bacterium*	VanZ-2
HBO03426.1	*Eubacterium sp.*	VanZ-2
HCP50284.1	*Gammaproteobacteria bacterium*	VanZ-2
MBK13940.1	*Porticoccaceae bacterium*	VanZ-2
MBU1248708.1	*Pseudomonadota bacterium*	VanZ-2
PYN16248.1	*Candidatus Rokuibacteriota bacterium*	VanZ-2
PJA42038.1	*Lysobacterales bacterium CG_4_9_14_3_um_filter_62_6*	VanZ-2
WP_129203730.1	*Ammoniphilus sp. CFH 90114*	VanZ-2
HBH60900.1	*Nitrospiraceae bacterium*	VanZ-2
WP_025876738.1	*Prevotella corporis*	VanZ-2
HBZ33498.1	*Bacteroidales bacterium*	VanZ-2
MBL8753227.1	*Planctomycetota bacterium*	VanZ-2
MBD5337772.1	*Bacteroides sp.*	VanZ-2
MBK5278968.1	*Bacteroidia bacterium*	VanZ-2
MCC7001101.1	*Gemmatimonadaceae bacterium*	VanZ-2
MBR4946098.1	*Kiritimatiellia bacterium*	VanZ-2
MCC6992217.1	*Acidobacteriota bacterium*	VanZ-2
MBS0213597.1	*Pseudomonadota bacterium*	VanZ-2
MBQ7428847.1	*Butyrivibrio sp.*	VanZ-2
MBQ9600814.1	*Neisseriaceae bacterium*	VanZ-2
MCD7809739.1	*Erysipelotrichaceae bacterium*	VanZ-2
MCB1902181.1	*Rhodocyclaceae bacterium*	VanZ-2
SJN17553.1	*Mycetocola reblochoni REB411*	VanZ-2
MBV6414734.1	*Lysobacterales bacterium*	VanZ-2
MBI4095343.1	*Nanobdellati archaeon*	VanZ-2
GBE04839.1	*bacterium BMS3Abin10*	VanZ-2
MBP9690351.1	*Bacteroidia bacterium*	VanZ-2
MBS4042306.1	*Chitinophagaceae bacterium*	VanZ-2
HIW85164.1	*Candidatus Eubacterium faecipullorum*	VanZ-2
MBP5763532.1	*Bacteroidales bacterium*	VanZ-2
MBF6560091.1	*Candidatus Binataceae bacterium*	VanZ-2
MSQ46212.1	*Ignavibacteria bacterium*	VanZ-2
MBP7838445.1	*Bacteroidales bacterium*	VanZ-2
NTW45151.1	*Anaerolineaceae bacterium*	VanZ-2
WP_105053318.1	*Psychrosphaera saromensis*	VanZ-2
MBU6311988.1	*Actinomycetales bacterium*	VanZ-2
HHO55866.1	*Trueperaceae bacterium*	VanZ-2
MBQ9568767.1	*Alloprevotella sp.*	VanZ-2
KAF0239164.1	*Chitinophagaceae bacterium*	VanZ-2
TEX49498.1	*Actinomycetales bacterium mxb001*	VanZ-2
NRA61015.1	*Psychrobium sp.*	VanZ-2
RKY77442.1	*Candidatus Zhuqueibacterota bacterium*	VanZ-2
WP_090243487.1		VanZ-2
TCP32812.1	*Sphingomonas sp. BK235*	VanZ-2
WP_154646580.1	*Alloprevotella tannerae*	VanZ-2
MBO9717414.1	*Pseudoxanthomonas sp.*	VanZ-2
WP_182732393.1	*Pseudoalteromonas rhizosphaerae*	VanZ-2
MAU90376.1	*Gammaproteobacteria bacterium*	VanZ-2
MBR1455661.1	*Oscillospiraceae bacterium*	VanZ-2
MBP7891793.1	*Arenimonas sp.*	VanZ-2
WP_183961441.1	*Chiayiivirga flava*	VanZ-2
MBI4323773.1	*Chloroflexota bacterium*	VanZ-2
MBA3770348.1	*Blastocatellia bacterium*	VanZ-2
MBE6760114.1	*Oscillospiraceae bacterium*	VanZ-2
OGU44067.1	*Ignavibacteria bacterium GWC2_56_12*	VanZ-2
GBE20586.1	*archaeon BMS3Abin17*	VanZ-2
MAN52029.1	*Marinimicrobium sp.*	VanZ-2
MBU4347033.1	*Patescibacteria group bacterium*	VanZ-2
KKU18352.1	*Candidatus Pacebacteria bacterium GW2011_GWA1_46_10*	VanZ-2
TAJ82609.1	*Gallionellaceae bacterium*	VanZ-2
MBO4453449.1	*Paludibacteraceae bacterium*	VanZ-2
MBC7592701.1	*Aeromicrobium sp.*	VanZ-2
MBI3304752.1	*Candidatus Parcubacteria bacterium*	VanZ-2
MBI5118835.1	*Candidatus Poribacteria bacterium*	VanZ-2
MBR2944521.1	*Clostridia bacterium*	VanZ-2
MBU0706790.1	*Patescibacteria group bacterium*	VanZ-2
HGX09881.1		VanZ-2
EDY81969.1	*Verrucomicrobiia bacterium DG1235*	VanZ-2
MBC3191391.1	*Pseudonocardia sp. C8*	VanZ-2
MBR1928606.1	*Paludibacteraceae bacterium*	VanZ-2
RYF68253.1	*Comamonadaceae bacterium*	VanZ-2
MBK7877659.1	*Planctomycetota bacterium*	VanZ-2
MBQ8100957.1	*Paludibacteraceae bacterium*	VanZ-2
QDO89449.1	*Ornithinimicrobium ciconiae*	VanZ-2
MAK60074.1	*Ponticaulis sp.*	VanZ-2
MBC7638261.1	*Actinomycetota bacterium*	VanZ-2
MBQ8337110.1	*Bacteroidaceae bacterium*	VanZ-2
MBN1462087.1	*Paludibacteraceae bacterium*	VanZ-2
MCB9002501.1	*Bacteroidales bacterium*	VanZ-2
MBU3853433.1	*Candidatus Paraprevotella stercoravium*	VanZ-2
MBB5955813.1	*Saccharothrix tamanrassetensis*	VanZ-2
MBL8518153.1	*Betaproteobacteria bacterium*	VanZ-2
MBI4239620.1	*Candidatus Uhrbacteria bacterium*	VanZ-2
WP_002667123.1		VanZ-2
MBU0467180.1	*Nanoarchaeota archaeon*	VanZ-2
GIL35618.1	*Phycicoccus sp. DTK01*	VanZ-2
WP_233341953.1	*Parolsenella massiliensis*	VanZ-2
MYD96456.1	*Gammaproteobacteria bacterium*	VanZ-2
MCB1796072.1	*Candidatus Competibacteraceae bacterium*	VanZ-2
MBO5975337.1	*Paludibacteraceae bacterium*	VanZ-2
MCC6960726.1	*Dehalococcoidia bacterium*	VanZ-2
KAB2314742.1	*Betaproteobacteria bacterium SCN2*	VanZ-2
WP_051437767.1	*Eubacterium xylanophilum ATCC 35991*	VanZ-2

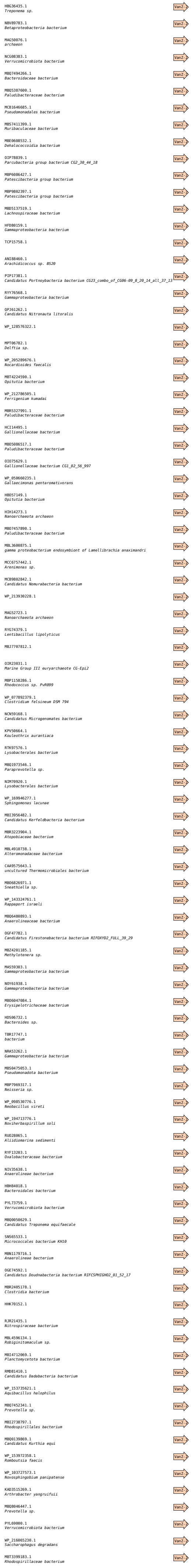
HBG36435.1
Treponema sp.
NBV89783.1
Betaproteobacteria bacterium
MAG50076.1
archaeon
NCG08383.1
Verrucomicrobiota bacterium
MBQ7494266.1
Bacteroidaceae bacterium
MBQ5387600.1
Paludibacteraceae bacterium
MCB1646685.1
Pseudomonadales bacterium
MBS7411399.1
Muribaculaceae bacterium
MBE0608532.1
Dehalococcoidia bacterium
OIP78839.1
Parcubacteria group bacterium CG2_30_44_18
MBP6086427.1
Patescibacteria group bacterium
MBP9802397.1
Patescibacteria group bacterium
MBD5137519.1
Lachnospiraceae bacterium
HFD80159.1
Gammaproteobacteria bacterium
TCP15758.1
ANI88460.1
Arachidicoccus sp. BS20
PIP17381.1
Candidatus Portnoybacteria bacterium CG23_combo_of_CG06-09_8_20_14_all_37_13
RYY76568.1
Gammaproteobacteria bacterium
QPJ61262.1
Candidatus Nitronauta litoralis
WP_128576322.1
MPT06782.1
Delftia sp.
WP_205289676.1
Nocardioides faecalis
MBT4224590.1
Opitutia bacterium
WP_212786505.1
Ferrigenium kumadai
MBR5327991.1
Paludibacteraceae bacterium
HCI14495.1
Gallionellaceae bacterium
MBO5086517.1
Paludibacteraceae bacterium
OIO75629.1
Gallionellaceae bacterium CG1_02_56_997
WP_050660235.1
Gallaecimonas pentaromativorans
HBO57149.1
Opitutia bacterium
HIH14273.1
Nanoarchaeota archaeon
MBO7457890.1
Paludibacteraceae bacterium
MBL3600875.1
gamma proteobacterium endosymbiont of Lamellibrachia anaximandri
MCC6757442.1
Arenimonas sp.
MCB9802842.1
Candidatus Nomurabacteria bacterium
WP_213930228.1
MAG52723.1
Nanoarchaeota archaeon
RYG74379.1
Lentibacillus lipolyticus
MBJ7707812.1
OIR23031.1
Marine Group III euryarchaeote CG-Epi2
MBP1158286.1
Rhodococcus sp. PvR099
WP_077892379.1
Clostridium felsineum DSM 794
NCN59168.1
Candidatus Microgenomates bacterium
KPV50664.1
Kouleothrix aurantiaca
RTK97576.1
Lysobacterales bacterium
MBQ1973546.1
Paraprevotella sp.
NIM70920.1
Lysobacterales bacterium
WP_169946277.1
Sphingomonas lacunae
MBI3956482.1
Candidatus Kerfeldbacteria bacterium
MBR3223904.1
Atopobiaceae bacterium
MBL4910738.1
Alteromonadaceae bacterium
CAA9575643.1
uncultured Thermomicrobiales bacterium
MBO6826971.1
Sneathiella sp.
WP_143324761.1
Rappaport israeli
MBQ6480893.1
Anaerolineaceae bacterium
OGF47782.1
Candidatus Firestonebacteria bacterium RIFOXYD2_FULL_39_29
MBZ4201185.1
Methylotenera sp.
MAS59303.1
Gammaproteobacteria bacterium
NOY61938.1
Gammaproteobacteria bacterium
MBO6047084.1
Erysipelotrichaceae bacterium
HDS06732.1
Bacteroides sp.
TBR17747.1
bacterium
NRA53262.1
Gammaproteobacteria bacterium
MBS0475053.1
Pseudomonadota bacterium
MBP7969317.1
Neisseria sp.
WP_098530776.1
Neobacillus vireti
WP_194713776.1
Noviherbaspirillum soli
RUO28065.1
Aliidiomarina sedimenti
RYF13203.1
Oxalobacteraceae bacterium
NIV35638.1
Anaerolineae bacterium
HBH84018.1
Bacteroidales bacterium
PYL73759.1
Verrucomicrobiota bacterium
MBQ0050629.1
Candidatus Treponema equifaecale
SNS65533.1
Micrococcales bacterium KH10
MBN1179716.1
Anaerolineae bacterium
OGE74592.1
Candidatus Doudnabacteria bacterium RIFCSPHIGHO2_01_52_17
MBR2405178.1
Clostridia bacterium
HHK70152.1
RJR21435.1
Nitrospiraceae bacterium
MBL4596134.1
Robiginitomaculum sp.
MBI4712069.1
Planctomycetota bacterium
RMD81410.1
Candidatus Dadabacteria bacterium
WP_153735621.1
Aquibacillus halophilus
MBQ7452341.1
Prevotella sp.
MBI2738797.1
Rhodospirillales bacterium
MBQ0139869.1
Candidatus Kurthia equi
WP_153972358.1
Romboutsia faecis
WP_103727573.1
Novosphingobium panipatense
KAD3515269.1
Arthrobacter yangruifuii
MBQ8046447.1
Prevotella sp.
PYL69000.1
Verrucomicrobiota bacterium
WP_216065230.1
Saccharophagus degradans
MBT3399183.1
Rhodospirillaceae bacterium

Accession	Organism	Gene
WP_117704675.1	*Eubacterium sp. OM08-24*	VanZ-2
MBZ0094166.1	*Sulfuricella sp.*	VanZ-2
HIF12372.1	*Dehalococcoidia bacterium*	VanZ-2
MBL0710306.1	*Colwellia sp.*	VanZ-2
TRZ92451.1	*bacterium*	VanZ-2
UCE72154.1	*Nitrospiraceae bacterium*	VanZ-2
WP_140818483.1		VanZ-2
UCH82025.1	*Nitrospiraceae bacterium*	VanZ-2
PLK50065.1	*Uliginosibacterium sp. TH139*	VanZ-2
OHB88713.1	*Planctomycetes bacterium RIFCSPHIGHO2_02_FULL_50_42*	VanZ-2
KAF1071634.1	*Variovorax sp.*	VanZ-2
WP_115717470.1	*Gallaecimonas mangrovi*	VanZ-2
RPH62582.1	*Acidobacteriota bacterium*	VanZ-2
MBA2793290.1	*Thermoleophilaceae bacterium*	VanZ-2
WP_171460926.1	*Vitreoscilla sp. C1*	VanZ-2
RNL67669.1	*Zhongshania marina*	VanZ-2
OFY62863.1	*Bacteroidetes bacterium RBG_13_43_22*	VanZ-2
MCC6300691.1	*Anaerolineales bacterium*	VanZ-2
MBI9031806.1	*bacterium*	VanZ-2
WP_170864683.1	*Halogranum amylolyticum*	VanZ-2
NBX42481.1	*Paracoccaceae bacterium*	VanZ-2
MAB04556.1	*Paracoccaceae bacterium*	VanZ-2
KPL18377.1	*candidate division Zixibacteria bacterium SM23_81*	VanZ-2
MBN1525817.1	*Spirochaetales bacterium*	VanZ-2
WP_015215349.1	*Anabaena sp. PCC 7938*	VanZ-2
MCD8302182.1	*Prevotellaceae bacterium*	VanZ-2
PYK80227.1	*Verrucomicrobiota bacterium*	VanZ-2
MCA9464807.1	*Nitrospira sp.*	VanZ-2
MBI2818776.1	*Acidobacteriota bacterium*	VanZ-2
MAA89431.1	*Deltaproteobacteria bacterium*	VanZ-2
MBE6469105.1	*Coriobacteriaceae bacterium*	VanZ-2
NSW94982.1	*Bacteroidales bacterium*	VanZ-2
RLC29668.1	*Deltaproteobacteria bacterium*	VanZ-2
RPI86780.1	*Planctomycetaceae bacterium*	VanZ-2
MBI4826682.1	*Nitrospirota bacterium*	VanZ-2
MBN2430398.1	*Acidobacteriota bacterium*	VanZ-2
NLD48450.1	*Clostridiaceae bacterium*	VanZ-2
MBT5052573.1	*Gammaproteobacteria bacterium*	VanZ-2
MBN9588544.1	*Alphaproteobacteria bacterium*	VanZ-2
MBX2818423.1	*Rhodothermaceae bacterium*	VanZ-2
MBP1610430.1	*Acidobacteriota bacterium*	VanZ-2
MBM3687753.1	*Actinomycetota bacterium*	VanZ-2
MBN2475617.1	*Pirellulales bacterium*	VanZ-2
MBU2136772.1	*Alphaproteobacteria bacterium*	VanZ-2
RLT41417.1	*Chloroflexota bacterium*	VanZ-2
MBN2014895.1	*Candidatus Altiarchaeota archaeon*	VanZ-2
RPI90112.1	*Chloroflexota bacterium*	VanZ-2
MBV8193149.1	*Alphaproteobacteria bacterium*	VanZ-2
MBA2243616.1	*Gemmatimonadota bacterium*	VanZ-2
MBV9331220.1	*Alphaproteobacteria bacterium*	VanZ-2
MBI2388371.1	*Deltaproteobacteria bacterium*	VanZ-2
MBV9063841.1	*Alphaproteobacteria bacterium*	VanZ-2
MQC17451.1	*Chloroflexota bacterium*	VanZ-2
MBI5187938.1	*Nitrospirota bacterium*	VanZ-2
RLE93463.1	*Thermoprotei archaeon*	VanZ-2
TAL80166.1	*Bacteroidota bacterium*	VanZ-2
MBI5234422.1	*Deltaproteobacteria bacterium*	VanZ-2
MBQ8302183.1	*Clostridia bacterium*	VanZ-2
MBQ7322524.1	*Clostridia bacterium*	VanZ-2
OIN85234.1	*Alphaproteobacteria bacterium CG1_02_46_17*	VanZ-2
WP_077216103.1		VanZ-2
HAM69006.1	*Ruminococcus sp.*	VanZ-2
MBE3138460.1	*Actinomycetota bacterium*	VanZ-2
WP_101759551.1	*Oceanicoccus sp. KOV_DT_Chl*	VanZ-2
MBS3151011.1	*Candidatus Woesearchaeota archaeon*	VanZ-2
MBD3321592.1	*Chitinivibrionales bacterium*	VanZ-2
MBU0651231.1	*bacterium*	VanZ-2
MCB1037527.1	*Acidobacteriota bacterium*	VanZ-2
MBI5589741.1	*Deltaproteobacteria bacterium*	VanZ-2
MBD3292075.1	*Armatimonadia bacterium*	VanZ-2
OYW50809.1	*Novosphingobium sp. 12-62-10*	VanZ-2
WP_005612240.1	*[Ruminococcus] lactaris*	VanZ-2
MBR1393622.1	*Ruminococcus sp.*	VanZ-2
RPI20721.1	*Chloroflexota bacterium*	VanZ-2
WP_161719537.1	*Novosphingobium ovatum*	VanZ-2
MBD3793456.1	*Campylobacterales bacterium*	VanZ-2
HHS50138.1	*Candidatus Zixiibacteriota bacterium*	VanZ-2
MBV8453429.1	*Deltaproteobacteria bacterium*	VanZ-2
HDR91371.1	*Candidatus Zixiibacteriota bacterium*	VanZ-2
HHH19054.1	*Campylobacterales bacterium*	VanZ-2
MBE0472740.1	*Rhodoferax sp.*	VanZ-2
OGW47231.1	*Nitrospirae bacterium RBG_19FT_COMBO_58_9*	VanZ-2
MBP3495412.1	*Clostridia bacterium*	VanZ-2
MBG0860974.1	*Bacteroidales bacterium*	VanZ-2
WP_077052957.1		VanZ-2
MBW1894397.1	*Deltaproteobacteria bacterium*	VanZ-2
MBK7132166.1	*Bacteroidales bacterium*	VanZ-2
MBK7711209.1	*Bacteroidales bacterium*	VanZ-2
MBK8102790.1	*Cellvibrionales bacterium*	VanZ-2
MBQ8039166.1	*Lachnospiraceae bacterium*	VanZ-2
OGT79490.1	*Gammaproteobacteria bacterium RIFCSPLOWO2_02_FULL_61_13*	VanZ-2
MBW2219059.1	*Deltaproteobacteria bacterium*	VanZ-2
WP_224316413.1	*Microvirga puerhi*	VanZ-2

MBW2415271.1	*Deltaproteobacteria bacterium*	VanZ-2
MBO1223727.1	*Candidatus Scalindua sediminis*	VanZ-2
STD71124.1	*Dermacoccus nishinomiyaensis*	VanZ-2
MBA2720861.1	*Chloroflexota bacterium*	VanZ-2
MCD6276118.1	*Thermoplasmata archaeon*	VanZ-2
WP_204239083.1	*Microvirga arvi*	VanZ-2
WP_233053552.1	*Motilimonas cestriensis*	VanZ-2
MBN1779516.1	*bacterium*	VanZ-2
TWU59998.1	*Rubripirellula tenax*	VanZ-2
UCC94907.1	*Candidatus Omnitrophota bacterium*	VanZ-2
MBN2348292.1	*Bacteroidales bacterium*	VanZ-2
MCD6418151.1	*bacterium*	VanZ-2
PYJ88374.1	*Verrucomicrobiota bacterium*	VanZ-2
NTU69016.1	*Chlorobiaceae bacterium*	VanZ-2
NNF04036.1	*Rhodothermales bacterium*	VanZ-2
WP_196410804.1		VanZ-2
NLN28519.1	*Bacillota bacterium*	VanZ-2
NLB34571.1	*Elusimicrobiota bacterium*	VanZ-2
OGP65962.1	*Deltaproteobacteria bacterium RBG_13_53_10*	VanZ-2
KXK49697.1	*Candidatus Tepidiaquacellales bacterium OLB5*	VanZ-2
MBP6856846.1	*Candidatus Pacearchaeota archaeon*	VanZ-2
MBQ9021370.1	*Eggerthellaceae bacterium*	VanZ-2
MBI3175472.1	*Chloroflexota bacterium*	VanZ-2
MBI3151485.1	*Chloroflexota bacterium*	VanZ-2
MBT8341554.1	*Desulfatitalea sp.*	VanZ-2
MBP3503022.1	*Clostridia bacterium*	VanZ-2
MBU2102797.1	*Candidatus Omnitrophota bacterium*	VanZ-2
HHH39060.1	*Sedimenticola sp.*	VanZ-2
MAF32817.1	*Desulfobacter sp.*	VanZ-2
NOY06455.1	*Chlorobiota bacterium*	VanZ-2
ESW84066.1	*Mesorhizobium sp. LSJC280B00*	VanZ-2
WP_163485287.1		VanZ-2
MBS66621.1	*Gammaproteobacteria bacterium*	VanZ-2
OGS02699.1	*Elusimicrobia bacterium RIFOXYA12_FULL_49_49*	VanZ-2
NBC15999.1	*Bacteroidota bacterium*	VanZ-2
TPN76052.1	*Mesorhizobium sp. CU3*	VanZ-2
WP_063676881.1	*Bradyrhizobium neotropicale*	VanZ-2
MBK7650915.1	*Flammeovirgaceae bacterium*	VanZ-2
PIP42608.1	*Desulfobacterales bacterium CG23_combo_of_CG06-09_8_20_14_all_51_8*	VanZ-2
RZA06648.1	*Moraxellaceae bacterium*	VanZ-2
MBD3278852.1	*Candidatus Aegiribacteria sp.*	VanZ-2
WP_223088392.1		VanZ-2
PCI41137.1	*Moraxellaceae bacterium*	VanZ-2
MBI5945308.1	*Chloroflexota bacterium*	VanZ-2
WP_025035201.1	*Bradyrhizobium sp. DOA9*	VanZ-2
MBQ6046222.1	*Bacteroidales bacterium*	VanZ-2
MBP1663364.1	*Bacteroidota bacterium*	VanZ-2
HHD82679.1	*Bacteroidota bacterium*	VanZ-2
MBK8472070.1	*Sphingobacteriales bacterium*	VanZ-2
MBF0184504.1	*Magnetococcales bacterium*	VanZ-2
MBX9687589.1	*Candidatus Obscuribacterales bacterium*	VanZ-2
MBK6577846.1	*Sandaracinaceae bacterium*	VanZ-2
WP_116775323.1	*Sphingobacterium corticibacter*	VanZ-2
NLE48838.1	*Sandaracinaceae bacterium*	VanZ-2
MBM4408531.1	*Chloroflexota bacterium*	VanZ-2
HHX39664.1	*Armatimonadota bacterium*	VanZ-2
MBE7039480.1	*Oscillospiraceae bacterium*	VanZ-2
MBQ6146817.1	*Clostridia bacterium*	VanZ-2
MBC8175115.1	*Fidelibacterota bacterium*	VanZ-2
HHU34637.1	*Bacteroidota bacterium*	VanZ-2
MBE5970348.1	*Lachnospiraceae bacterium*	VanZ-2
MBC8128281.1	*Verrucomicrobiales bacterium*	VanZ-2
HGR87067.1		VanZ-2
WP_027182676.1	*Desulfovibrio inopinatus DSM 10711*	VanZ-2
MBM7690705.1	*Peribacillus deserti*	VanZ-2
NTU93244.1	*Chlorobiaceae bacterium*	VanZ-2
WP_067677566.1	*Erythrobacter sp. HI0063*	VanZ-2
WP_075958461.1	*uncultured Ornithinimicrobium sp.*	VanZ-2
MBK7498290.1	*Ignavibacteriales bacterium*	VanZ-2
HEA46225.1	*Candidatus Pacearchaeota archaeon*	VanZ-2
WP_225073538.1	*Desulfuromonas sp. CSMB_57*	VanZ-2
WP_067660422.1	*Ferrimonas marina*	VanZ-2
WP_163835275.1	*Spartinivicinus ruber*	VanZ-2
QLH40087.1	*Defluviicoccus sp.*	VanZ-2
MBQ7760774.1	*Clostridia bacterium*	VanZ-2
MBP7216987.1	*Candidatus Omnitrophota bacterium*	VanZ-2
MBI4415560.1	*Methanobacteriota archaeon*	VanZ-2
OGO20009.1	*Chloroflexi bacterium RBG_16_48_8*	VanZ-2
RME70060.1	*Verrucomicrobiota bacterium*	VanZ-2
MBE5783910.1	*Clostridiales bacterium*	VanZ-2
WP_216054817.1	*Psychrosphaera sp. F3M07*	VanZ-2
MBR57468.1	*Myxococcales bacterium*	VanZ-2
MQA02722.1	*Streptosporangiales bacterium*	VanZ-2
WP_089281179.1	*Anaerovirgula multivorans*	VanZ-2
WP_232387322.1	*Methylobacterium currus*	VanZ-2
MCA0363985.1	*Bacteroidota bacterium*	VanZ-2
MBM3252362.1	*Candidatus Omnitrophota bacterium*	VanZ-2
MBP7088898.1	*Candidatus Omnitrophota bacterium*	VanZ-2
MBU0548684.1	*Candidatus Omnitrophota bacterium*	VanZ-2
SUA36490.1	*Neisseria zoodegmatis*	VanZ-2
MBI3479959.1	*Nitrosomonadales bacterium*	VanZ-2
UCF37188.1	*Acidobacteriota bacterium*	VanZ-2
MAM16069.1	*Flavobacteriaceae bacterium*	VanZ-2

WP_161602035.1	*Aliiglaciecola lipolytica E3*	VanZ-2
MBU1121525.1	*Candidatus Omnitrophota bacterium*	VanZ-2
WP_191311953.1	*Paracoccus aerius*	VanZ-2
GBF74561.1	*Paenibacillus sp. 598K*	VanZ-2
WP_051600871.1	*Eubacterium sp. AB3007*	VanZ-2
TXH75116.1	*Thiothrix sp.*	VanZ-2
MBU4251751.1	*Candidatus Omnitrophota bacterium*	VanZ-2
MBO8439719.1	*Candidatus Aphodosoma intestinipullorum*	VanZ-2
AST94365.1	*Sutcliffiella cohnii*	VanZ-2
SDZ68157.1	*Evansella caseinilytica*	VanZ-2
MBQ2944647.1	*Ruminococcus sp.*	VanZ-2
WP_225008473.1	*Novosphingobium percolationis*	VanZ-2
TML16186.1	*Actinomycetota bacterium*	VanZ-2
PKB19532.1	*Novosphingobium kunmingense*	VanZ-2
TNC81828.1	*Oleiphilus sp.*	VanZ-2
HCU65396.1	*Rheinheimera sp.*	VanZ-2
MCA9704942.1	*Myxococcales bacterium*	VanZ-2
SFK94125.1	*Paenibacillus sp. 1_12*	VanZ-2
NOT33602.1	*Candidatus Eiseniibacteriota bacterium*	VanZ-2
KUK79466.1	*Microgenomates bacterium 39_7*	VanZ-2
GBD96523.1	*bacterium BMS3Abin06*	VanZ-2
NGY88500.1	*Priestia megaterium*	VanZ-2
MCB0856300.1	*Bacteroidota bacterium*	VanZ-2
MBM3303548.1	*Candidatus Aenigmatarchaeota archaeon*	VanZ-2
WP_164218316.1	*Virgibacillus sp. YIM 98842*	VanZ-2
MBE7027532.1	*Oscillospiraceae bacterium*	VanZ-2
MAX12574.1	*Fidelibacterota bacterium*	VanZ-2
MBT2969087.1	*Candidatus Thiodiazotropha taylori*	VanZ-2
WP_152146384.1	*Alkalilimnicola sp. S0819*	VanZ-2
HDH10662.1	*Nitrospirota bacterium*	VanZ-2
MBQ6257086.1	*Clostridia bacterium*	VanZ-2
MBI4051858.1	*Elusimicrobiota bacterium*	VanZ-2
MBI5573025.1	*Elusimicrobiota bacterium*	VanZ-2
WP_078717424.1	*Paucidesulfovibrio gracilis DSM 16080*	VanZ-2
MBO0749260.1	*Porphyrobacter sp.*	VanZ-2
MBR8829361.1	*Gomphosphaeria aponina SAG 52.96 = DSM 107014*	VanZ-2
MCB1714946.1	*Candidatus Competibacteraceae bacterium*	VanZ-2
MCD6423461.1	*Elusimicrobiota bacterium*	VanZ-2
MBR2024501.1	*Clostridia bacterium*	VanZ-2
MBS3086361.1	*Candidatus Pacearchaeota archaeon*	VanZ-2
MBU0491373.1	*Chloroflexota bacterium*	VanZ-2
MCA1561557.1	*Acidobacteriota bacterium*	VanZ-2
MBC7696714.1	*Bacteroidia bacterium*	VanZ-2
MBX9673533.1	*Candidatus Obscuribacterales bacterium*	VanZ-2
MBE7013378.1	*Oscillospiraceae bacterium*	VanZ-2
OGR89658.1	*Elusimicrobia bacterium RIFCSPLOWO2_01_FULL_59_12*	VanZ-2
WP_129948412.1	*Parashewanella hymeniacidonis*	VanZ-2
MBR9978693.1	*Bacteroidota bacterium*	VanZ-2
MBI5174456.1	*Candidatus Melainabacteria bacterium*	VanZ-2
WP_128990005.1	*Stenotrophomonas sp. MA5*	VanZ-2
PID28811.1	*Candidatus Cloacimonadota bacterium*	VanZ-2
MBT8442872.1	*Gammaproteobacteria bacterium*	VanZ-2
MBT7952152.1	*Gammaproteobacteria bacterium*	VanZ-2
MBM3749193.1	*Acidobacteriota bacterium*	VanZ-2
MBM3690380.1	*Actinomycetota bacterium*	VanZ-2
MBI3608418.1	*Nitrospirota bacterium*	VanZ-2
MBP5153189.1	*Lachnospiraceae bacterium*	VanZ-2
WP_010175620.1	*Bacillus coahuilensis m2-6*	VanZ-2
MBT6723291.1	*Planctomycetaceae bacterium*	VanZ-2
MBV8557827.1	*Planctomycetaceae bacterium*	VanZ-2
MBQ7388577.1	*Paludibacteraceae bacterium*	VanZ-2
MBI1812047.1	*Nitrospirota bacterium*	VanZ-2
MBM3746089.1	*Acidobacteriota bacterium*	VanZ-2
NLA42437.1	*Smithella sp.*	VanZ-2
KFZ44959.1	*Smithella sp. D17*	VanZ-2
MBQ5592416.1	*Clostridia bacterium*	VanZ-2
HHV37363.1	*Candidatus Cloacimonadota bacterium*	VanZ-2
HDH05678.1	*Nitrospirota bacterium*	VanZ-2
OAH06926.1	*Rhodobacteraceae bacterium SB2*	VanZ-2
MBP1686183.1	*Deltaproteobacteria bacterium*	VanZ-2
MBU2637758.1	*Nanoarchaeota archaeon*	VanZ-2
PIE56774.1	*Desulfobulbus propionicus*	VanZ-2
MBT3188603.1	*Anaerolineae bacterium*	VanZ-2
MBT4362973.1	*Desulfobacteraceae bacterium*	VanZ-2
NMD01796.1	*Bacteroidales bacterium*	VanZ-2
TCN77660.1	*Shewanella fodinae*	VanZ-2
MBP7564049.1	*Candidatus Cloacimonadota bacterium*	VanZ-2
MAA65924.1	*Alteromonadaceae bacterium*	VanZ-2
NRA75682.1	*Planctomycetota bacterium*	VanZ-2
WP_027516161.1	*Bradyrhizobium sp.*	VanZ-2
OUU04356.1	*Flammeovirgaceae bacterium TMED32*	VanZ-2
MBR1809072.1	*Paludibacteraceae bacterium*	VanZ-2
MBE8717992.1	*Cellvibrio polysaccharolyticus*	VanZ-2
MBA4342543.1	*Methylibium sp.*	VanZ-2
WP_029867971.1	*Rhizobium sp. IY2*	VanZ-2
MBA3013630.1	*Desulfobulbaceae bacterium*	VanZ-2
WP_116689069.1	*Pelagibaculum spongiae*	VanZ-2
MBX9614998.1	*Caulobacteraceae bacterium*	VanZ-2
NRA82320.1	*Gammaproteobacteria bacterium*	VanZ-2
MBP8665573.1	*Syntrophaceae bacterium*	VanZ-2
HGX20173.1		VanZ-2
WP_232334338.1	*Oceanobacillus sp. HCA-5259*	VanZ-2
MBN1805954.1	*Sedimentisphaerales bacterium*	VanZ-2

MBI5947884.1	*Chloroflexota bacterium*	VanZ-2
MBK8468885.1	*Actinomycetales bacterium*	VanZ-2
CDE03674.1	*Anaerotruncus sp. CAG:390*	VanZ-2
UCH77804.1	*Candidatus Coatesbacteria bacterium*	VanZ-2
WP_012800970.1	*Kangiella koreensis DSM 16069*	VanZ-2
MCB5251290.1	*Candidatus Cloacimonadota bacterium*	VanZ-2
MBX9964618.1	*Burkholderiales bacterium*	VanZ-2
MBR3878061.1	*Bacteroidaceae bacterium*	VanZ-2
MCB1667843.1	*Pseudomonadales bacterium*	VanZ-2
MCE1195957.1	*bacterium*	VanZ-2
MBI2880416.1	*Candidatus Tectimicrobiota bacterium*	VanZ-2
HID03798.1	*Desulfobacterales bacterium*	VanZ-2
MBL8821070.1	*Planctomycetia bacterium*	VanZ-2
MCC7352766.1	*Anaerolineae bacterium*	VanZ-2
MBQ0157757.1	*Candidatus Equimonas enterica*	VanZ-2
WP_164045532.1	*Rhizobium leguminosarum*	VanZ-2
PIZ83818.1	*Candidatus Pacearchaeota archaeon CG_4_10_14_0_2_um_filter_05_32_18*	VanZ-2
ETX01765.1	*Candidatus Entotheonella factor*	VanZ-2
MBS5789933.1	*Fusobacterium sp.*	VanZ-2
MBR2635036.1	*Clostridia bacterium*	VanZ-2
MBN8995710.1	*Hyphomicrobiales bacterium*	VanZ-2
MBR7165800.1	*Clostridia bacterium*	VanZ-2
WP_128189028.1	*Agrococcus sediminis*	VanZ-2
TKD70956.1	*Guptibacillus hwajinpoensis*	VanZ-2
MBQ77823.1	*Cellvibrionales bacterium*	VanZ-2
PYJ41547.1	*Verrucomicrobiota bacterium*	VanZ-2
MBL8894021.1	*Hyphomicrobiales bacterium*	VanZ-2
MBU2068423.1	*Patescibacteria group bacterium*	VanZ-2
MBO5673304.1	*Paludibacteraceae bacterium*	VanZ-2
MBR0201409.1	*Bacteroidaceae bacterium*	VanZ-2
GDY21313.1	*Verrucomicrobiota bacterium*	VanZ-2
TAG57196.1	*Cytophagales bacterium*	VanZ-2
KPK55207.1	*Thiotrichales bacterium SG8_50*	VanZ-2
WP_026692789.1	*Peribacillus kribbensis DSM 17871*	VanZ-2
MYA16244.1	*Gammaproteobacteria bacterium*	VanZ-2
MCD6256422.1	*Deltaproteobacteria bacterium*	VanZ-2
HII14898.1	*Nanoarchaeota archaeon*	VanZ-2
MBR6102139.1	*Ruminococcus sp.*	VanZ-2
RJP50376.1	*Anaerolineaceae bacterium*	VanZ-2
WP_231507663.1	*Bacillus sp. UNC41MFS5*	VanZ-2
SON56846.1	*Hartmannibacter diazotrophicus*	VanZ-2
MBB25445.1	*Geminicoccus sp.*	VanZ-2
NWG14448.1	*Acidobacteriota bacterium*	VanZ-2
MBR1586092.1	*Clostridia bacterium*	VanZ-2
NQT16622.1	*Planctomycetota bacterium*	VanZ-2
WP_182503455.1	*Peribacillus huizhouensis*	VanZ-2
MBL9115808.1	*Verrucomicrobiaceae bacterium*	VanZ-2
MBV9463377.1	*Verrucomicrobiia bacterium*	VanZ-2
NOZ95852.1	*Acidobacteriota bacterium*	VanZ-2
HCQ00424.1	*Candidatus Latescibacterota bacterium*	VanZ-2
MBN1756383.1	*bacterium*	VanZ-2
WP_008527394.1	*Rhizobium sp. Pop5*	VanZ-2
NLE97283.1	*Propionibacterium sp.*	VanZ-2
MCD7721016.1	*Prevotellaceae bacterium*	VanZ-2
HGX97123.1		VanZ-2
MCD9017040.1	*Parachryseolinea silvisoli*	VanZ-2
OHB84775.1	*Planctomycetes bacterium RBG_19FT_COMBO_48_8*	VanZ-2
MBQ3674227.1	*Campylobacter sp.*	VanZ-2
OYW66090.1	*Hydrogenophilales bacterium 12-64-13*	VanZ-2
MBR9702384.1	*Candidatus Pacearchaeota archaeon*	VanZ-2
WP_161496696.1	*Clostridium thermosuccinogenes*	VanZ-2
MBK6886054.1	*Nostocoides sp.*	VanZ-2
TDJ61381.1	*Pseudomonadota bacterium*	VanZ-2
MBD3389149.1	*Candidatus Altiarchaeales archaeon*	VanZ-2
MBS1328126.1	*Oscillospiraceae bacterium*	VanZ-2
WP_064648887.1	*Rhizobium sp. WYCCWR10014*	VanZ-2
MBR0351153.1	*Clostridia bacterium*	VanZ-2
WP_232788374.1	*Macromonas nakdongensis*	VanZ-2
MBQ6906907.1	*Clostridia bacterium*	VanZ-2
MBK9099206.1	*bacterium*	VanZ-2
HHS14327.1	*bacterium*	VanZ-2
WP_104148549.1	*Brevibacillus laterosporus*	VanZ-2
MCA1555026.1	*Chloroflexota bacterium*	VanZ-2
TDL88194.1	*Vibrio vulnificus*	VanZ-2
MBE9511010.1	*Bacteroidota bacterium*	VanZ-2
MBU1669185.1	*bacterium*	VanZ-2
MBK6764212.1	*Micrococcales bacterium*	VanZ-2
WP_006503149.1	*Austwickia chelonae*	VanZ-2
MBI4093311.1	*Candidatus Kerfeldbacteria bacterium*	VanZ-2
MAH03623.1	*Candidatus Pacearchaeota archaeon*	VanZ-2
OFY83257.1	*Bacteroidetes bacterium RBG_19FT_COMBO_42_7*	VanZ-2
MBC6427399.1	*Ekhidna sp.*	VanZ-2
MCA1979046.1	*Thiobacillus sp.*	VanZ-2
MBM3934939.1	*SAR202 cluster bacterium*	VanZ-2
MCC6927496.1	*Gemmatimonadaceae bacterium*	VanZ-2
MBS1168968.1	*Pseudomonadota bacterium*	VanZ-2
MBS1180308.1	*Pseudomonadota bacterium*	VanZ-2
GAV25394.1	*Carboxydothermus islandicus*	VanZ-2
MAG02412.1	*Candidatus Pacearchaeota archaeon*	VanZ-2
HDY75424.1	*Fidelibacterota bacterium*	VanZ-2
NLU23067.1	*Phycisphaerae bacterium*	VanZ-2
OVE83128.1	*Natronolimnobius baerhuensis*	VanZ-2
GIR15951.1	*Pseudomonadota bacterium*	VanZ-2

MBN2625547.1	*Spirochaetales bacterium*	VanZ-2
WP_119382825.1	*Clavibacter lycopersici*	VanZ-2
WP_104055236.1	*Arthrobacter sp. 9E16*	VanZ-2
MBN2419411.1	*Deltaproteobacteria bacterium*	VanZ-2
CDE87387.1	*Prevotella sp. CAG:891*	VanZ-2
UCD38004.1	*Fidelibacterota bacterium*	VanZ-2
OIP36125.1	*Candidatus Desantisbacteria bacterium CG2_30_40_21*	VanZ-2
MBN2351118.1	*Spirochaetales bacterium*	VanZ-2
MBC8205080.1	*FCB group bacterium*	VanZ-2
WP_205665317.1	*Guptibacillus hwajinpoensis*	VanZ-2
WP_068318512.1	*Aliiruegeria sabulilitoris*	VanZ-2
PIQ75014.1	*Candidatus Portnoybacteria bacterium CG11_big_fil_rev_8_21_14_0_20_40_15*	VanZ-2
HIF54015.1	*Methylococcaceae bacterium*	VanZ-2
MBN2408462.1	*Candidatus Aminicenantes bacterium*	VanZ-2
MBK8541841.1	*Candidatus Epilinea brevis*	VanZ-2
MBP9698182.1	*Candidatus Moraniibacteriota bacterium*	VanZ-2
MCC6948865.1	*Bradyrhizobiaceae bacterium*	VanZ-2
NNE86082.1	*Alphaproteobacteria bacterium*	VanZ-2
WP_194968354.1	*Methylobacter sp. BlB1*	VanZ-2
MBC7624372.1	*Burkholderiales bacterium*	VanZ-2
OGH98697.1	*Candidatus Margulisbacteria bacterium GWD2_39_127*	VanZ-2
WP_077589937.1	*Planococcus lenghuensis*	VanZ-2
MBM4334062.1	*Deltaproteobacteria bacterium*	VanZ-2
MBW2452748.1	*Deltaproteobacteria bacterium*	VanZ-2
MBN9444425.1	*Bosea sp. (in: a-proteobacteria)*	VanZ-2
MBE6982021.1	*Oscillospiraceae bacterium*	VanZ-2
MBI5969815.1	*Deltaproteobacteria bacterium*	VanZ-2
GBE04009.1	*bacterium BMS3Abin09*	VanZ-2
MBT5571224.1	*Alphaproteobacteria bacterium*	VanZ-2
PYI80320.1	*Verrucomicrobiota bacterium*	VanZ-2
TVQ15597.1	*Bacteroidota bacterium*	VanZ-2
HDQ71810.1	*Chloroflexota bacterium*	VanZ-2
WP_117157320.1		VanZ-2
MBP9852171.1	*Candidatus Paceibacterota bacterium*	VanZ-2
MBI2844429.1	*Armatimonadota bacterium*	VanZ-2
HCA52740.1	*Mycobacterium sp.*	VanZ-2
WP_090790362.1	*Paenibacillus sp. GP183*	VanZ-2
KUJ80806.1		VanZ-2
NQS97800.1	*Candidatus Tariuqbacter arcticus*	VanZ-2
WP_232319803.1	*Prosthecochloris sp. HL-130-GSB*	VanZ-2
MCB1629563.1	*Lysobacterales bacterium*	VanZ-2
AQS33822.1		VanZ-2
HIQ30248.1	*Candidatus Caldarchaeum subterraneum*	VanZ-2
RLF97870.1	*Candidatus Wolframiiraptor sp.*	VanZ-2
NQV19510.1	*Armatimonadota bacterium*	VanZ-2
RPJ22933.1	*Chloroflexota bacterium*	VanZ-2
TET45663.1	*candidate division TA06 bacterium*	VanZ-2
MAF17517.1	*Marinomonas sp.*	VanZ-2
NCO33440.1	*Armatimonadota bacterium*	VanZ-2
NIP82829.1	*Gemmatimonadota bacterium*	VanZ-2
RZK10289.1	*Flavobacterium sp.*	VanZ-2
MBV1731120.1	*Hydrogenophaga sp.*	VanZ-2
MBX3500884.1	*Alphaproteobacteria bacterium*	VanZ-2
HIS81967.1	*Candidatus Scatomonas merdavium*	VanZ-2
MBN1192448.1	*Coriobacteriia bacterium*	VanZ-2
MBA3583005.1	*Gemmatimonadota bacterium*	VanZ-2
MBI5804843.1	*candidate division TA06 bacterium*	VanZ-2
MBI5582548.1	*Deltaproteobacteria bacterium*	VanZ-2
WP_162039014.1		VanZ-2
HIZ97669.1	*Candidatus Janibacter merdipullorum*	VanZ-2
MBI5588647.1	*Deltaproteobacteria bacterium*	VanZ-2
MCE1274784.1	*Chlorobiales bacterium*	VanZ-2
RLD46330.1	*Bacteroidota bacterium*	VanZ-2
NJK90904.1	*Blastochloris sp.*	VanZ-2
PIN87992.1	*Candidatus Woesearchaeota archaeon CG10_big_fil_rev_8_21_14_0_10_32_24*	VanZ-2
RME40790.1	*Deltaproteobacteria bacterium*	VanZ-2
MCC7277110.1	*Chromatiaceae bacterium*	VanZ-2
HIF10202.1	*Sneathiellales bacterium*	VanZ-2
RTL50704.1	*Bradyrhizobiaceae bacterium*	VanZ-2
WP_025761732.1	*Dyadobacter tibetensis Y620-1*	VanZ-2
PCI42878.1	*Alphaproteobacteria bacterium*	VanZ-2
MBC7461922.1	*Thermoleophilia bacterium*	VanZ-2
NPA68795.1	*Chlorobiota bacterium*	VanZ-2
MBN1799130.1	*Spirochaetales bacterium*	VanZ-2
RYD99286.1	*Sphingomonadales bacterium*	VanZ-2
MBC8278342.1	*FCB group bacterium*	VanZ-2
MBN1841424.1	*Deltaproteobacteria bacterium*	VanZ-2
MBN1882957.1	*Candidatus Zymogenaceae bacterium*	VanZ-2
WP_007420195.1	*Idiomarina sp. A28L*	VanZ-2
MBJ7452715.1	*Blastococcus sp.*	VanZ-2
OYD17011.1	*candidate division WOR-3 bacterium JGI_Cruoil_03_44_89*	VanZ-2
MBG6185010.1		VanZ-2
PKO17600.1	*candidate division BRC1 bacterium HGW-BRC1-1*	VanZ-2
RYZ21710.1	*Chitinophagaceae bacterium*	VanZ-2
WP_050931684.1	*Aestuariivita boseongensis*	VanZ-2
MBK7863377.1	*Archangiaceae bacterium*	VanZ-2
CAA6821393.1	*uncultured Sulfurovum sp.*	VanZ-2
MBA3261660.1	*Thermoleophilaceae bacterium*	VanZ-2
MBS6445092.1	*Ruminococcus sp.*	VanZ-2
WP_190615290.1	*Tepiditoga spiralis*	VanZ-2
MBQ8010791.1	*Oscillospiraceae bacterium*	VanZ-2
NOU42854.1	*Methyloglobulus sp.*	VanZ-2
MBS3068157.1	*Candidatus Micrarchaeota archaeon*	VanZ-2

MBT3449701.1	*Bacteroidota Order II. bacterium*	VanZ-2
NLX10326.1	*Chloroflexota bacterium*	VanZ-2
PIS22659.1	*candidate division WWE3 bacterium CG08_land_8_20_14_0_20_41_10*	VanZ-2
HFD15439.1	*Rhodospirillales bacterium*	VanZ-2
MBD3288396.1	*Candidatus Zhuqueibacterota bacterium*	VanZ-2
MBN2166667.1	*Marinilabiliaceae bacterium*	VanZ-2
PIP01690.1	*Zetaproteobacteria bacterium CG23_combo_of_CG06-09_8_20_14_all_54_7*	VanZ-2
MBQ0008312.1	*Candidatus Minthenecus merdequi*	VanZ-2
MBX9928927.1	*Gemmatimonadaceae bacterium*	VanZ-2
HAX61430.1	*Elusimicrobiota bacterium*	VanZ-2
MBO4367952.1	*Clostridia bacterium*	VanZ-2
MBR4572683.1	*Prevotella sp.*	VanZ-2
MBU4439465.1	*Bacillota bacterium*	VanZ-2
MBP6735340.1	*Chromatiaceae bacterium*	VanZ-2
NIM50254.1	*Gemmatimonadales bacterium*	VanZ-2
TXT50670.1	*Spirochaetota bacterium*	VanZ-2
OGT12030.1	*Gallionellales bacterium RIFCSPHIGHO2_02_FULL_57_16*	VanZ-2
MBL8096148.1	*Anaerolineales bacterium*	VanZ-2
HGS98086.1		VanZ-2
WP_232054634.1	*Laribacter hongkongensis*	VanZ-2
RUA17672.1	*Clostridia bacterium*	VanZ-2
WP_223798576.1	*Sphingomonas nostoxanthinifaciens*	VanZ-2
NLG56447.1	*Rhodococcus sp. (in: high G+C Gram-positive bacteria)*	VanZ-2
WP_127820330.1	*Microbacterium sp. CPCC 204701*	VanZ-2
NRB17872.1	*Paracoccaceae bacterium*	VanZ-2
WP_163876394.1	*Parapedobacter sp. SGR-10*	VanZ-2
MBE9520964.1	*Pseudomonadota bacterium*	VanZ-2
NIO05117.1	*Pseudomonadota bacterium*	VanZ-2
NIQ37676.1	*Pseudomonadota bacterium*	VanZ-2
NBX93371.1	*Pseudomonadota bacterium*	VanZ-2
HHP61870.1		VanZ-2
MBK6742999.1	*Hydrogenophilales bacterium*	VanZ-2
MBW2617092.1	*Deltaproteobacteria bacterium*	VanZ-2
MBP7965377.1	*Paludibacteraceae bacterium*	VanZ-2
MBP6935043.1	*Paludibacteraceae bacterium*	VanZ-2
MBP6370037.1	*Paludibacteraceae bacterium*	VanZ-2
NIP85367.1	*Planctomycetales bacterium*	VanZ-2
MBC7617686.1	*Rhodoferax sp.*	VanZ-2
MBN8572419.1	*Candidatus Kapaibacterium sp.*	VanZ-2
TAJ92707.1	*Gammaproteobacteria bacterium*	VanZ-2
MBU0953321.1	*Nanoarchaeota archaeon*	VanZ-2
NOT18724.1	*Sideroxydans sp.*	VanZ-2
MBP7933335.1	*Phycisphaerae bacterium*	VanZ-2
MCB1993397.1	*Geminicoccaceae bacterium*	VanZ-2
MBK8093660.1	*Verrucomicrobiaceae bacterium*	VanZ-2
MBU1103711.1	*Nanoarchaeota archaeon*	VanZ-2
WP_142905080.1	*Exilibacterium tricleocarpae*	VanZ-2
MBM3430728.1	*Bacteroidota bacterium*	VanZ-2
MBE6574403.1	*Oscillospiraceae bacterium*	VanZ-2
HGW68217.1		VanZ-2
MBU6301456.1	*Verrucomicrobiota bacterium*	VanZ-2
MBI1794978.1	*Chloroflexota bacterium*	VanZ-2
NBC48947.1	*Gammaproteobacteria bacterium*	VanZ-2
HHH13848.1	*Thiolapillus brandeum*	VanZ-2
RMH60382.1	*Zetaproteobacteria bacterium*	VanZ-2
PHS72990.1	*Cycloclasticus sp.*	VanZ-2
RFC48289.1	*Verrucomicrobiota bacterium*	VanZ-2
RJP85192.1	*Desulfobacteraceae bacterium*	VanZ-2
MBI4759926.1	*Chloroflexota bacterium*	VanZ-2
WP_143071476.1	*Tranquillimonas rosea*	VanZ-2
MBI5295426.1	*Chloroflexota bacterium*	VanZ-2
MBP6411472.1	*Pseudarcicella sp.*	VanZ-2
HEA67213.1	*Desulfobacterales bacterium*	VanZ-2
MBS6848388.1	*Ruminococcus sp.*	VanZ-2
WP_089199210.1	*Paenibacillus xerothermodurans*	VanZ-2
MBQ9975923.1	*Clostridia bacterium*	VanZ-2
MCD8095886.1	*Ruminococcus sp.*	VanZ-2
HIG43464.1	*Gammaproteobacteria bacterium*	VanZ-2
MBK8292187.1	*Flammeovirgaceae bacterium*	VanZ-2
PIP07363.1	*Syntrophobacteraceae bacterium CG23_combo_of_CG06-09_8_20_14_all_50_8*	VanZ-2
NCC73298.1	*Sphingobacteriia bacterium*	VanZ-2
RLC84692.1	*Chloroflexota bacterium*	VanZ-2
MBX3717553.1	*Burkholderiales bacterium*	VanZ-2
OUM94911.1	*Paenibacillaceae bacterium ZCTH02-B3*	VanZ-2
MBT9613394.1	*Burkholderiales bacterium*	VanZ-2
NNF60882.1	*Gammaproteobacteria bacterium*	VanZ-2
MBN2492825.1	*Planctomycetota bacterium*	VanZ-2
MBU0530528.1	*Nanoarchaeota archaeon*	VanZ-2
MBE6724786.1	*Oscillospiraceae bacterium*	VanZ-2
MBM3452025.1	*Bacteroidota bacterium*	VanZ-2
WP_118534667.1	*Roseburia sp. AM51-8*	VanZ-2
WP_028239925.1	*Stutzerimonas azotifigens DSM 17556*	VanZ-2
OYU93078.1	*Bacteroidetes bacterium B1(2017)*	VanZ-2
OHB94111.1	*Planctomycetes bacterium RIFCSPHIGHO2_12_FULL_52_36*	VanZ-2
WP_061341084.1	*Stutzerimonas balearica*	VanZ-2
MAO32259.1	*Crocinitomicaceae bacterium*	VanZ-2
OYU97312.1	*Bacteroidetes bacterium B1(2017)*	VanZ-2
MBQ0753912.1	*Gammaproteobacteria bacterium*	VanZ-2
WP_190571632.1	*Alkalinema sp. FACHB-956*	VanZ-2
NWF72997.1	*Nitrospirota bacterium*	VanZ-2
MBR3018981.1	*Clostridia bacterium*	VanZ-2
MBP7518042.1	*Desulfobulbus sp.*	VanZ-2
MBQ9440159.1	*Paludibacteraceae bacterium*	VanZ-2

EZH65363.1	*Bacillaceae bacterium JMAK1*	VanZ-2
MCD6665618.1	*Hydrogenophaga sp.*	VanZ-2
MBU4182625.1	*Gammaproteobacteria bacterium*	VanZ-2
MBL7747189.1	*Chitinophagaceae bacterium*	VanZ-2
TAD73613.1	*Oscillatoriales cyanobacterium*	VanZ-2
RKZ29792.1	*bacterium*	VanZ-2
MBP6903156.1	*Burkholderiaceae bacterium*	VanZ-2
PZN10888.1		VanZ-2
WP_015416159.1	*Pseudodesulfovibrio piezophilus C1TLV30*	VanZ-2
KJV10190.1	*Elstera litoralis*	VanZ-2
MBK9140007.1	*Verrucomicrobiota bacterium*	VanZ-2
MCA8987996.1	*Planctomycetaceae bacterium*	VanZ-2
MBI5408077.1	*Nitrospirota bacterium*	VanZ-2
MBI5479098.1	*Deltaproteobacteria bacterium*	VanZ-2
BAZ30280.1	*Cylindrospermum sp. NIES-4074*	VanZ-2
OQA47024.1	*Chloroflexi bacterium ADurb.Bin325*	VanZ-2
NIP98153.1	*Akkermansiaceae bacterium*	VanZ-2
MBQ4507661.1	*Paludibacteraceae bacterium*	VanZ-2
MBI9017238.1	*Phycisphaerae bacterium*	VanZ-2
MBI3292297.1	*Elusimicrobiota bacterium*	VanZ-2
MBI3297949.1	*Elusimicrobiota bacterium*	VanZ-2
HIR36967.1	*Candidatus Limisoma gallistercoris*	VanZ-2
MBK8556341.1	*Lewinellaceae bacterium*	VanZ-2
OIQ42603.1	*Gammaproteobacteria bacterium MedPE*	VanZ-2
UCD07734.1	*Candidatus Aenigmatarchaeota archaeon*	VanZ-2
HDY68846.1	*Candidatus Scalindua sp.*	VanZ-2
MBA2361244.1	*Actinomycetota bacterium*	VanZ-2
NLU25022.1	*Clostridiales bacterium*	VanZ-2
OUV63645.1	*Gammaproteobacteria bacterium TMED119*	VanZ-2
MBR5376765.1	*Lachnospiraceae bacterium*	VanZ-2
MSW96107.1	*Actinomycetota bacterium*	VanZ-2
MBO0863239.1	*Chloracidobacterium sp.*	VanZ-2
WP_215965103.1	*Psychrosphaera sp. 1_MG-2023*	VanZ-2
MBW8010001.1	*Chloroflexota bacterium*	VanZ-2
PWL70101.1	*Clostridiales bacterium*	VanZ-2
BCS80446.1	*Caldicellulosiruptor diazotrophicus*	VanZ-2
OGV40670.1	*Lentisphaerae bacterium GWF2_57_35*	VanZ-2
MQY62706.1	*Calditrichota bacterium*	VanZ-2
WP_101597502.1	*Bacillus sp. M6-12*	VanZ-2
NHN38659.1	*Pseudomaricurvus alcaniphilus*	VanZ-2
OGY30287.1	*Candidatus Woykebacteria bacterium RIFCSPHIGHO2_02_FULL_43_16b*	VanZ-2
MBL7160559.1	*Candidatus Aenigmatarchaeota archaeon*	VanZ-2
WP_214300631.1	*Pelotalea chapellei*	VanZ-2
MBN8682534.1	*Chitinophagales bacterium*	VanZ-2
TAL32901.1	*Phenylobacterium sp.*	VanZ-2
MCA4133073.1	*Arthrobacter sp. M4*	VanZ-2
GAP72949.1	*Candidatus Symbiothrix dinenymphae*	VanZ-2
MBO7393497.1	*Abditibacteriota bacterium*	VanZ-2
WP_127693663.1	*Neptunomonas sp. XY-337*	VanZ-2
MBI3615413.1	*Candidatus Omnitrophota bacterium*	VanZ-2
RLD16550.1	*Caldisericota bacterium*	VanZ-2
PKN94401.1	*Chloroflexi bacterium HGW-Chloroflexi-6*	VanZ-2
PWM40150.1	*Clostridiales bacterium*	VanZ-2
MBI4974228.1	*Candidatus Omnitrophota bacterium*	VanZ-2
TXH70187.1	*Thiothrix sp.*	VanZ-2
NNJ72531.1	*Enterobacterales bacterium*	VanZ-2
UIP57898.1	*Agromyces marinus*	VanZ-2
GJL71885.1	*Nitrosomonas sp.*	VanZ-2
WP_079688713.1	*Ohtaekwangia koreensis*	VanZ-2
OUM96472.1	*Thermobacillus sp. ZCTH02-B1*	VanZ-2
MBL8510888.1	*Betaproteobacteria bacterium*	VanZ-2
MCD8538780.1	*Leadbetterella sp.*	VanZ-2
WP_141107756.1	*Butyrivibrio sp. YAB3001*	VanZ-2
MBR1792742.1	*Bacteroidales bacterium*	VanZ-2
NEQ70807.1	*Symploca sp. SIO2D2*	VanZ-2
MBN2120007.1	*Candidatus Omnitrophota bacterium*	VanZ-2
WP_015539347.1	*Gordonibacter pamelaeae*	VanZ-2
SMN15282.1	*uncultured Candidatus Thioglobus sp.*	VanZ-2
WP_066015308.1	*Endozoicomonas atrinae*	VanZ-2
MBI28793.1	*Pelagibacteraceae bacterium*	VanZ-2
WP_224798078.1	*Idiomarina abyssalis*	VanZ-2
WP_216050195.1	*Aliiglaciecola sp. NS0011-25*	VanZ-2
HAQ62023.1	*Candidatus Delongbacteria bacterium*	VanZ-2
MCB2213176.1	*bacterium*	VanZ-2
MBQ7707101.1	*Lachnospiraceae bacterium*	VanZ-2
WP_064503537.1	*uncultured Paracoccus sp.*	VanZ-2
MBO8168470.1	*Thermoanaerobacteraceae bacterium*	VanZ-2
NBH82366.1	*Lachnospiraceae bacterium*	VanZ-2
TDI90813.1	*Caldithrix sp.*	VanZ-2
MBI5073420.1	*Candidatus Woesearchaeota archaeon*	VanZ-2
MCB0258596.1	*Calditrichota bacterium*	VanZ-2
MBL4880163.1	*Oleispira sp.*	VanZ-2
MCC6411710.1	*Saprospiraceae bacterium*	VanZ-2
MAP96449.1	*Ponticaulis sp.*	VanZ-2
MCB9308353.1	*Lewinellaceae bacterium*	VanZ-2
UCC91569.1	*Candidatus Aenigmatarchaeota archaeon*	VanZ-2
WP_233382670.1	*Methylobacterium sp. C25*	VanZ-2
WP_015933600.1	*Methylobacterium nodulans ORS 2060*	VanZ-2
NQV63787.1	*SAR86 cluster bacterium*	VanZ-2
WP_183457497.1	*Mesorhizobium huakuii*	VanZ-2
OPL19766.1	*Candidatus Aegiribacteria sp. MLS_C*	VanZ-2
PZO81573.1	*Mesorhizobium amorphae*	VanZ-2
WP_027046624.1	*Mesorhizobium sp. 131-3-5*	VanZ-2

KQC11819.1	*Candidatus Cloacimonas sp. SDB*	VanZ-2
HIS86503.1	*Candidatus Faecivicinus avistercoris*	VanZ-2
MBQ9519498.1	*Bacillota bacterium*	VanZ-2
NDC74355.1	*bacterium*	VanZ-2
WP_161299898.1	*Streptomyces sp. SID5785*	VanZ-2
HDX41852.1		VanZ-2
MBI3093769.1	*Rhodocyclales bacterium*	VanZ-2
WP_026997516.1	*Flectobacillus major DSM 103*	VanZ-2
MBT6031712.1	*Kordiimonadaceae bacterium*	VanZ-2
MBP9906552.1	*Rhodoferax sp.*	VanZ-2
MCE1169179.1		VanZ-2
WP_102195618.1	*Companilactobacillus nuruki*	VanZ-2
WP_181975063.1	*Clostridium sp. AF36-4*	VanZ-2
MCD6182066.1	*Candidatus Cloacimonadota bacterium*	VanZ-2
MBO6158077.1	*Bacillota bacterium*	VanZ-2
NCP86516.1	*bacterium*	VanZ-2
WP_130631031.1		VanZ-2
TFG86470.1	*Hyphomicrobiales bacterium*	VanZ-2
WP_214160589.1	*Kineosporia corallincola*	VanZ-2
TCR93391.1	*Rhizobium sp. BK376*	VanZ-2
MBR7153883.1	*Paludibacteraceae bacterium*	VanZ-2
WP_013808628.1		VanZ-2
MBI4924062.1	*Devosia nanyangense*	VanZ-2
PKF56302.1	*Alteromonadales bacterium alter-6D02*	VanZ-2
WP_013344397.1	*Ferrimonas balearica*	VanZ-2
KRP35768.1	*Opitutaceae bacterium BACL24 MAG-120322-bin51*	VanZ-2
WP_011771023.1	*Psychromonas ingrahamii 37*	VanZ-2
WP_101102808.1	*Macromonas bipunctata*	VanZ-2
QTV80599.1	*Microbacterium sp. NIBRBAC000506063*	VanZ-2
PPD35647.1	*Methylomonas sp.*	VanZ-2
NUE00933.1	*Haloferacaceae archaeon YAN*	VanZ-2
MBI3115264.1	*Candidatus Kerfeldbacteria bacterium*	VanZ-2
ODS42241.1	*Candidatus Altiarchaeales archaeon IMC4*	VanZ-2
MBT3952393.1	*Rhodobacterales bacterium*	VanZ-2
MBS6520396.1	*Clostridiales bacterium*	VanZ-2
HIX92133.1	*Bacillota bacterium*	VanZ-2
MBF0913393.1	*Candidatus Altimarinota bacterium*	VanZ-2
MBN36224.1	*Rhodospirillaceae bacterium*	VanZ-2
MBQ7333586.1	*Clostridia bacterium*	VanZ-2
MBJ48304.1	*Fidelibacterota bacterium*	VanZ-2
NWF52173.1	*Nitrospirota bacterium*	VanZ-2
HIZ54184.1	*Candidatus Enterococcus avicola*	VanZ-2
WP_066638871.1	*Desulfolucanica intricata*	VanZ-2
MCB2203883.1	*bacterium*	VanZ-2
WP_024516074.1	*Bradyrhizobium sp. Tv2a-2*	VanZ-2
MBK6661579.1	*Candidatus Amarobacillus elongatus*	VanZ-2
MBF1114471.1	*Solobacterium sp.*	VanZ-2
WP_225112389.1	*Bradyrhizobium sp. NBAIM32*	VanZ-2
MCD6517076.1	*Candidatus Aminicenantes bacterium*	VanZ-2
WP_023496206.1		VanZ-2
CAA6826637.1	*uncultured Sulfurovum sp.*	VanZ-2
MBX5469125.1	*Thermoleophilaceae bacterium*	VanZ-2
WP_164664337.1	*Sulfurovum sp. bin170*	VanZ-2
MBK8814178.1	*Methylococcaceae bacterium*	VanZ-2
GJM16235.1	*Thermodesulfobacteriota bacterium*	VanZ-2
NOQ36349.1	*Methylococcaceae bacterium*	VanZ-2
WP_012083428.1	*Sulfurovum sp. NBC37-1*	VanZ-2
AQQ55083.1	*Planococcus lenghuensis*	VanZ-2
WP_184241530.1	*Micrococcus cohnii*	VanZ-2
MBJ7247887.1	*Thermoleophilia bacterium*	VanZ-2
MBI3885810.1	*Opitutia bacterium*	VanZ-2
KPJ50557.1	*candidate division TA06 bacterium DG_26*	VanZ-2
RUM63112.1	*Sulfurospirillum sp.*	VanZ-2
TMH45400.1	*Betaproteobacteria bacterium*	VanZ-2
VVB53666.1	*uncultured archaeon*	VanZ-2
RPJ24707.1	*Chloroflexota bacterium*	VanZ-2
WP_204147197.1	*Marinomonas ostreistagni*	VanZ-2
MCC6920536.1	*Alphaproteobacteria bacterium*	VanZ-2
MQC16739.1	*Chloroflexota bacterium*	VanZ-2
MQC17053.1	*Chloroflexota bacterium*	VanZ-2
MBI4774033.1	*Deltaproteobacteria bacterium*	VanZ-2
NIR46579.1	*Gemmatimonadota bacterium*	VanZ-2
MQC25534.1	*Chloroflexota bacterium*	VanZ-2
MBE6023148.1	*Cellulosilyticum sp.*	VanZ-2
WP_101794807.1	*Porphyrobacter sp. TH134*	VanZ-2
WP_027107770.1	*Lacticigenium naphthae DSM 19658*	VanZ-2
WP_120018224.1	*Mesorhizobium waimense*	VanZ-2
RMF57677.1	*Bacteroidota bacterium*	VanZ-2
SFH17946.1	*Desulfotruncus arcticus DSM 17038*	VanZ-2
MCD5390127.1	*Candidatus Paceibacterota bacterium*	VanZ-2
MBI4409102.1	*Gemmatimonadota bacterium*	VanZ-2
SHP76595.1	*Mycobacteroides abscessus subsp. abscessus*	VanZ-2
HHD79251.1	*Campylobacterota bacterium*	VanZ-2
MBE1483621.1		VanZ-2
MBM4193792.1	*Gemmatimonadota bacterium*	VanZ-2
OFW34899.1	*Candidatus Aquicultor primus*	VanZ-2
MBK5946393.1	*Phaeovulum veldkampii DSM 11550*	VanZ-2
MBW3660092.1	*Gemmatimonadota bacterium*	VanZ-2
HCR48691.1	*Bacteroidota bacterium*	VanZ-2
WP_011324208.1	*Natronomonas pharaonis DSM 2160*	VanZ-2
AIA12905.1		VanZ-2
OJX34854.1	*Flavobacteriia bacterium 40-80*	VanZ-2
HIP41219.1	*Campylobacterales bacterium*	VanZ-2

RYF44029.1	*Comamonadaceae bacterium*	VanZ-2
MBC8185619.1	*Candidatus Zhuqueibacterota bacterium*	VanZ-2
MCC6772906.1	*Gemmatimonadaceae bacterium*	VanZ-2
RKX91468.1	*Spirochaetota bacterium*	VanZ-2
MBR2133809.1	*Eubacterium sp.*	VanZ-2
MBW1635591.1	*Deltaproteobacteria bacterium*	VanZ-2
NQT23923.1	*Candidatus Zhuqueibacterota bacterium*	VanZ-2
HHA15139.1		VanZ-2
MBV9528389.1	*Sphingomonas sp.*	VanZ-2
MBS0369065.1	*Pseudomonadota bacterium*	VanZ-2
MBE9573175.1	*Pseudomonadota bacterium*	VanZ-2
NPA30733.1	*Chloroflexota bacterium*	VanZ-2
MBE9539082.1	*Pseudomonadota bacterium*	VanZ-2
HHP93467.1		VanZ-2
MBQ1528439.1	*Candidatus Saccharibacteria bacterium*	VanZ-2
TVP43499.1	*Halomonas sp.*	VanZ-2
PIV89210.1	*Hydrogenophilales bacterium CG17_big_fil_post_rev_8_21_14_2_50_63_12*	VanZ-2
RMH34411.1	*Gammaproteobacteria bacterium*	VanZ-2
MBV1822060.1	*Bacteroidales bacterium MSK.15.36*	VanZ-2
MBR5717999.1	*Clostridia bacterium*	VanZ-2
MBT16540.1	*Verrucomicrobiales bacterium*	VanZ-2
MBP7368574.1	*Paludibacteraceae bacterium*	VanZ-2
MAH36027.1	*Verrucomicrobiales bacterium*	VanZ-2
MBP6231018.1	*Paludibacteraceae bacterium*	VanZ-2
MBE3039734.1	*Chloroflexota bacterium*	VanZ-2
MBK7256609.1	*Ignavibacteriota bacterium*	VanZ-2
WP_170012200.1	*Starkeya sp. ORNL1*	VanZ-2
HGF02389.1		VanZ-2
MBT4928615.1	*Cellvibrionales bacterium*	VanZ-2
RYD17121.1	*Verrucomicrobiaceae bacterium*	VanZ-2
PHR59461.1	*Arcobacter sp.*	VanZ-2
MBF0235215.1	*Desulfamplus sp.*	VanZ-2
MBU1290028.1	*Patescibacteria group bacterium*	VanZ-2
MAH13726.1	*Verrucomicrobiota bacterium*	VanZ-2
MBQ0118677.1	*Candidatus Colicola coprequi*	VanZ-2
WP_027288564.1	*Rhodovibrio salinarum*	VanZ-2
MBI5381763.1	*Opitutia bacterium*	VanZ-2
MBG30274.1	*Opitutia bacterium*	VanZ-2
OGS41067.1	*Elusimicrobia bacterium RIFOXYD12_FULL_66_9*	VanZ-2
MBQ6449502.1	*bacterium*	VanZ-2
MBP5473463.1	*Bacteroidales bacterium*	VanZ-2
MCB9430139.1	*Actinomycetota bacterium*	VanZ-2
HCX98510.1	*Bacteroidales bacterium*	VanZ-2
MBI2804626.1	*Planctomycetota bacterium*	VanZ-2
MBD5156833.1	*Butyrivibrio sp.*	VanZ-2
NOY39279.1	*Nitrospirota bacterium*	VanZ-2
MCA9035926.1	*Planctomycetaceae bacterium*	VanZ-2
MBM3679809.1	*Actinomycetota bacterium*	VanZ-2
MBI5194273.1	*Nitrospirota bacterium*	VanZ-2
WP_009487479.1		VanZ-2
MBT3239795.1	*Chloroflexota bacterium*	VanZ-2
NQV00997.1	*Parcubacteria group bacterium*	VanZ-2
WP_115138106.1	*Shewanella submarina*	VanZ-2
MBI5739379.1	*Nitrospirota bacterium*	VanZ-2
WP_104203390.1	*Billgrantia saliphila*	VanZ-2
MBB1284892.1	*Flavisolibacter longurius*	VanZ-2
WP_128519567.1	*Absicoccus porci*	VanZ-2
UCG77404.1	*Nitrospirota bacterium*	VanZ-2
KPK40351.1	*Gammaproteobacteria bacterium SG8_47*	VanZ-2
MCA9836043.1	*Trueperaceae bacterium*	VanZ-2
MBQ8821264.1	*Lachnospiraceae bacterium*	VanZ-2
NLF71866.1	*Candidatus Anammoximicrobium sp.*	VanZ-2
MBF0217813.1	*Candidatus Omnitrophota bacterium*	VanZ-2
NJD36040.1	*Betaproteobacteria bacterium*	VanZ-2
MBF0478373.1	*Candidatus Omnitrophota bacterium*	VanZ-2
MBQ7919822.1	*Lachnospiraceae bacterium*	VanZ-2
MCB1842069.1	*Halioglobus sp.*	VanZ-2
MCA9396567.1	*Candidatus Omnitrophota bacterium*	VanZ-2
GBE15788.1	*bacterium BMS3Abin14*	VanZ-2
UCD14874.1	*Candidatus Omnitrophota bacterium*	VanZ-2
WP_140808075.1	*Mesorhizobium sp. B2-7-1*	VanZ-2
MBL8514155.1	*Betaproteobacteria bacterium*	VanZ-2
HGE55781.1		VanZ-2
PSB12266.1	*Pleurocapsa sp. CCALA 161*	VanZ-2
PKN81982.1	*Chloroflexi bacterium HGW-Chloroflexi-9*	VanZ-2
MBQ6222199.1	*Solobacterium sp.*	VanZ-2
MBD9074513.1	*bacterium*	VanZ-2
WP_141699464.1	*Candidatus Marithrix sp. Canyon 246*	VanZ-2
MBC7790593.1	*Gemmatimonadaceae bacterium*	VanZ-2
MBV9190770.1	*Betaproteobacteria bacterium*	VanZ-2
MBQ3000026.1	*Clostridia bacterium*	VanZ-2
MBU0634347.1	*Candidatus Omnitrophota bacterium*	VanZ-2
WP_009745341.1		VanZ-2
MBL8523187.1	*Betaproteobacteria bacterium*	VanZ-2
HFM98582.1		VanZ-2
MBK9983250.1	*Candidatus Opimibacter skivensis*	VanZ-2
MBI4846260.1	*Candidatus Omnitrophota bacterium*	VanZ-2
MBL8320030.1	*Burkholderiaceae bacterium*	VanZ-2
MBD1934644.1	*Trichocoleus sp. FACHB-69*	VanZ-2
WP_051171138.1	*Neomelitea salexigens DSM 19753*	VanZ-2
MBI3737057.1	*Candidatus Sumerlaeota bacterium*	VanZ-2
MCC6386290.1	*Dehalococcoidia bacterium*	VanZ-2
NBH48815.1		VanZ-2

MBU4334000.1	*Candidatus Omnitrophota bacterium*	VanZ-2
WP_153393499.1	*Ornithinicoccus halotolerans*	VanZ-2
OQX79587.1	*Candidatus Omnitrophica bacterium 4484_70.1*	VanZ-2
OGX07615.1	*Omnitrophica WOR_2 bacterium GWA2_37_7*	VanZ-2
PZO47699.1	*Phormidesmis priestleyi*	VanZ-2
MBR3564885.1	*Paludibacteraceae bacterium*	VanZ-2
MBR4704018.1	*Paludibacteraceae bacterium*	VanZ-2
MBR6787762.1	*Clostridia bacterium*	VanZ-2
MBR9706704.1	*Candidatus Pacearchaeota archaeon*	VanZ-2
MBI1883039.1	*Chlamydiota bacterium*	VanZ-2
MCB1619089.1	*Pseudomonadales bacterium*	VanZ-2
MBN9254998.1	*Mesorhizobium sp.*	VanZ-2
PLX88050.1	*Desulfuromonas sp.*	VanZ-2
MBU1102741.1	*Patescibacteria group bacterium*	VanZ-2
WP_215975124.1	*Gilvimarinus sp. 1_MG-2023*	VanZ-2
MBN8563720.1	*Leptolyngbya sp. UWPOB_LEPTO1*	VanZ-2
MCB0885477.1	*Propionibacteriaceae bacterium*	VanZ-2
MBD5245840.1	*Barnesiella sp.*	VanZ-2
WP_183521114.1	*Mameliella alba*	VanZ-2
OGW62123.1	*Nitrospirae bacterium RIFCSPHIGHO2_01_FULL_66_17*	VanZ-2
TAN01384.1	*Rhizobiaceae bacterium*	VanZ-2
MBC7961812.1	*Deltaproteobacteria bacterium*	VanZ-2
MCB1829513.1	*Gammaproteobacteria bacterium*	VanZ-2
MCB1786769.1	*Gammaproteobacteria bacterium*	VanZ-2
MCB1790490.1	*Gammaproteobacteria bacterium*	VanZ-2
GDX24992.1	*Actinomycetes bacterium*	VanZ-2
MBW8911210.1	*Sphingomonas sp.*	VanZ-2
MBK9741136.1	*Actinomycetota bacterium*	VanZ-2
MBI4961802.1	*Desulfomonile tiedjei*	VanZ-2
PIV10102.1	*Candidatus Portnoybacteria bacterium CG03_land_8_20_14_0_80_41_10*	VanZ-2
MBI4849558.1	*Nitrospirota bacterium*	VanZ-2
MBL7155011.1	*Candidatus Portnoybacteria bacterium*	VanZ-2
MBM3125619.1	*Chloroflexota bacterium*	VanZ-2
HID56283.1	*Candidatus Poribacteria bacterium*	VanZ-2
MCD8365848.1	*Clostridiales bacterium*	VanZ-2
MBP96364.1	*Candidatus Poribacteria bacterium*	VanZ-2
WP_013293794.1	*Gallionella capsiferriformans ES-2*	VanZ-2
WP_007935588.1		VanZ-2
RYF85744.1	*Chitinophagaceae bacterium*	VanZ-2
OYY46470.1	*Gallionellales bacterium 35-53-114*	VanZ-2
HFQ88612.1	*Desulfobulbus sp.*	VanZ-2
MCA9151255.1	*Planctomycetales bacterium*	VanZ-2
ANE53599.1	*Flavisolibacter tropicus*	VanZ-2
NBT90768.1	*Verrucomicrobiota bacterium*	VanZ-2
KPJ61134.1	*Planctomycetes bacterium DG_23*	VanZ-2
GBD43771.1	*bacterium HR40*	VanZ-2
MBM4207712.1	*Gammaproteobacteria bacterium*	VanZ-2
RMG15113.1	*Planctomycetota bacterium*	VanZ-2
WP_102414515.1		VanZ-2
NIA13493.1	*Nitrospiraceae bacterium*	VanZ-2
MBR6777419.1	*Clostridia bacterium*	VanZ-2
WP_013178248.1	*Truepera radiovictrix*	VanZ-2
MZQ49093.1	*Bacteroidales bacterium*	VanZ-2
WP_224810678.1		VanZ-2
MCD7738845.1	*Lachnospiraceae bacterium*	VanZ-2
WP_013555806.1	*Deinococcus maricopensis DSM 21211*	VanZ-2
OGY91713.1	*Candidatus Komeilibacteria bacterium RIFCSPLOWO2_01_FULL_53_11*	VanZ-2
PCJ68364.1	*Rhodobiaceae bacterium*	VanZ-2
NKX40573.1	*Rhodobacteraceae bacterium R_SAG2*	VanZ-2
NLD70241.1	*Limnobacter sp.*	VanZ-2
GGK63061.1	*Ornithinimicrobium pekingense*	VanZ-2
OHC43468.1	*Rhodobacteraceae bacterium GWE1_64_9*	VanZ-2
MBR9842182.1	*Paracoccaceae bacterium*	VanZ-2
MBI2426976.1	*Candidatus Kerfeldbacteria bacterium*	VanZ-2
MBK5243904.1	*Eubacteriaceae bacterium*	VanZ-2
TFG90400.1	*Syntrophobacterales bacterium*	VanZ-2
MBL9189386.1	*Opitutaceae bacterium*	VanZ-2
UCD71937.1	*Syntrophobacterales bacterium*	VanZ-2
MCA9356948.1	*Candidatus Kaiserbacteria bacterium*	VanZ-2
WP_062582837.1	*Rhizobium sp. Leaf321*	VanZ-2
WP_087143083.1	*Crenothrix polyspora*	VanZ-2
MBA2433149.1	*Chthoniobacterales bacterium*	VanZ-2
HGB57188.1		VanZ-2
WP_191619679.1	*Pelagicoccus enzymogenes*	VanZ-2
MSQ09672.1	*Dehalococcoidia bacterium*	VanZ-2
WP_170303583.1	*Reyranella soli*	VanZ-2
GGD04573.1	*Pyruvatibacter mobilis*	VanZ-2
MBQ2915451.1	*Clostridia bacterium*	VanZ-2
MBR4598898.1	*Treponema sp.*	VanZ-2
RZP02249.1	*Gammaproteobacteria bacterium*	VanZ-2
MBQ8896613.1	*Clostridia bacterium*	VanZ-2
QOX78554.1	*Trichlorobacter lovleyi*	VanZ-2
MBD7978437.1	*Serpens gallinarum*	VanZ-2
MBP1603243.1	*Acidobacteriota bacterium*	VanZ-2
WP_066875309.1	*Clostridium mediterraneense*	VanZ-2
OGV72529.1	*Methylophilales bacterium RIFCSPHIGHO2_02_FULL_57_10*	VanZ-2
PIE63236.1	*Desulfobacter postgatei*	VanZ-2
OGR28294.1	*Desulfobacca sp. RBG_16_60_12*	VanZ-2
MBL6637994.1	*Planktomarina sp.*	VanZ-2
MCC3193545.1		VanZ-2
WP_162317773.1	*Haloarcula limicola*	VanZ-2
MBS0660448.1	*Verrucomicrobiota bacterium*	VanZ-2
OWU82806.1	*Phaeobacter sp. 22II1-1F12B*	VanZ-2

OFW10976.1	*Acidobacteria bacterium RIFCSPLOWO2_02_FULL_67_36*	VanZ-2
MBO5298634.1	*Clostridia bacterium*	VanZ-2
WP_183103132.1	*Mitsuaria sp. WAJ17*	VanZ-2
MBL9131954.1	*Verrucomicrobiaceae bacterium*	VanZ-2
TDJ44266.1	*Gammaproteobacteria bacterium*	VanZ-2
MBK7353354.1	*Nitrosomonas sp.*	VanZ-2
MBO5071574.1	*Eubacterium sp.*	VanZ-2
MCE1158630.1	*Spirochaetia bacterium*	VanZ-2
HCV97739.1	*Verrucomicrobiales bacterium*	VanZ-2
MBR2284174.1	*Ruminococcus sp.*	VanZ-2
WP_127710653.1	*Novosphingobium umbonatum*	VanZ-2
KHS41742.1	*Novosphingobium subterraneum*	VanZ-2
MBC7781819.1	*Burkholderiales bacterium*	VanZ-2
OYY58952.1	*Hydrogenophilales bacterium 28-61-11*	VanZ-2
MBN9146595.1	*Novosphingobium sp.*	VanZ-2
WP_131445272.1	*Parasulfuritortus cantonensis*	VanZ-2
WP_168942079.1	*Parazoarcus communis*	VanZ-2
WP_223696785.1	*Kinneretia aquatilis*	VanZ-2
WP_116984010.1	*Emticicia sp. C21*	VanZ-2
WP_051259432.1	*Schaalia suimastitidis DSM 15538*	VanZ-2
WP_181843047.1	*Emticicia sp. BO119*	VanZ-2
MBJ7417756.1	*Niveispirillum sp.*	VanZ-2
RLA10584.1	*Gammaproteobacteria bacterium*	VanZ-2
HID40173.1	*Calditrichota bacterium*	VanZ-2
MBE9573151.1	*Pseudomonadota bacterium*	VanZ-2
HEC80035.1	*Bacillota bacterium*	VanZ-2
NLZ54892.1	*Thermoanaerobacteraceae bacterium*	VanZ-2
MBA4070522.1	*Gemmatimonas sp.*	VanZ-2
WP_010888656.1	*Deinococcus radiodurans*	VanZ-2
MBS0253958.1	*Pseudomonadota bacterium*	VanZ-2
WP_160896891.1	*Pseudooceanicola endophyticus*	VanZ-2
MBS1214197.1	*Pseudomonadota bacterium*	VanZ-2
MBX6332948.1	*Gemmatimonadaceae bacterium*	VanZ-2
TYB82714.1	*Kosmotoga sp.*	VanZ-2
MBX3120200.1	*Fimbriimonadaceae bacterium*	VanZ-2
MBS2015458.1	*Deltaproteobacteria bacterium*	VanZ-2
MBP7321792.1	*Deltaproteobacteria bacterium*	VanZ-2
MBE0499023.1	*Campylobacterales bacterium*	VanZ-2
TNF52548.1	*bacterium*	VanZ-2
WP_142877666.1	*Mesorhizobium sp. WSM4310*	VanZ-2
MSQ68300.1	*Gammaproteobacteria bacterium*	VanZ-2
OGS46762.1	*Elusimicrobia bacterium RIFOXYD2_FULL_34_15*	VanZ-2
HBR96635.1	*Gammaproteobacteria bacterium*	VanZ-2
MBA3560878.1	*Gemmatimonadaceae bacterium*	VanZ-2
MBQ0082444.1	*Candidatus Colousia faecequi*	VanZ-2
MBV9775127.1	*Gemmatimonadota bacterium*	VanZ-2
WP_013658308.1	*Cellulosilyticum sp. ST5*	VanZ-2
MBU2589738.1	*Nanoarchaeota archaeon*	VanZ-2
MBN4055116.1	*bacterium AH-315-K03*	VanZ-2
MBI5886239.1	*Deltaproteobacteria bacterium*	VanZ-2
MBM3604541.1	*Alphaproteobacteria bacterium*	VanZ-2
MBO6765552.1	*Maricaulis sp.*	VanZ-2
MBP7759827.1	*Alphaproteobacteria bacterium*	VanZ-2
MBI2408524.1	*Gemmatimonadota bacterium*	VanZ-2
MBN1326771.1	*Candidatus Cloacimonadota bacterium*	VanZ-2
MBU6373337.1	*Alphaproteobacteria bacterium*	VanZ-2
MBK5201062.1	*Spirochaetaceae bacterium*	VanZ-2
MBD3262963.1	*Candidatus Woesearchaeota archaeon*	VanZ-2
OSM04326.1	*Magnetofaba australis IT-1*	VanZ-2
GDX87144.1	*Gemmatimonadota bacterium*	VanZ-2
MBT3338784.1	*Anaerolineae bacterium*	VanZ-2
PWL32877.1	*Fluviicola sp. XM-24bin1*	VanZ-2
MBF0192973.1	*Magnetococcales bacterium*	VanZ-2
MBE1415953.1		VanZ-2
MBQ60547.1	*Gammaproteobacteria bacterium*	VanZ-2
OQY40377.1	*Spirochaetaceae bacterium 4572_7*	VanZ-2
MBI4209107.1	*Deltaproteobacteria bacterium*	VanZ-2
WP_049741833.1	*Brevibacillus reuszeri*	VanZ-2
MCB1590827.1	*Alphaproteobacteria bacterium*	VanZ-2
RMH15029.1	*Gemmatimonadota bacterium*	VanZ-2
OQB90800.1	*Verrucomicrobia bacterium ADurb.Bin122*	VanZ-2
WP_119082626.1	*Altererythrobacter sp. B11*	VanZ-2
ROT95125.1	*Altererythrobacter sp. FM1*	VanZ-2
HIC44806.1	*Sulfurimonas sp.*	VanZ-2
MBL8902507.1	*Hyphomicrobiales bacterium*	VanZ-2
MBL8331902.1	*Rubrivivax sp.*	VanZ-2
HCO49649.1	*Spirochaetaceae bacterium*	VanZ-2
MBN2586756.1	*Candidatus Fermentibacteraceae bacterium*	VanZ-2
MRR13621.1		VanZ-2
MCE2690192.1	*Rubrivivax sp.*	VanZ-2
HAE21855.1	*Spirochaetaceae bacterium*	VanZ-2
NOT10363.1	*Methylococcaceae bacterium*	VanZ-2
WP_212658076.1	*Devosia litorisediminis*	VanZ-2
NKB66477.1	*Candidatus Latescibacterota bacterium*	VanZ-2
WP_162393544.1	*Goekera deserti*	VanZ-2
WP_170955948.1	*Cohaesibacter gelatinilyticus*	VanZ-2
OPX23192.1	*Candidatus Latescibacteria bacterium 4484_107*	VanZ-2
HBM62068.1	*Citreicella sp.*	VanZ-2
MBI5938965.1	*Caulobacterales bacterium*	VanZ-2
MBS3963251.1	*Methylomonas sp.*	VanZ-2
MBQ2030958.1	*Lachnospiraceae bacterium*	VanZ-2
OYV20843.1	*Methylococcaceae bacterium NSP1-2*	VanZ-2
MBR4101608.1	*Oscillospiraceae bacterium*	VanZ-2

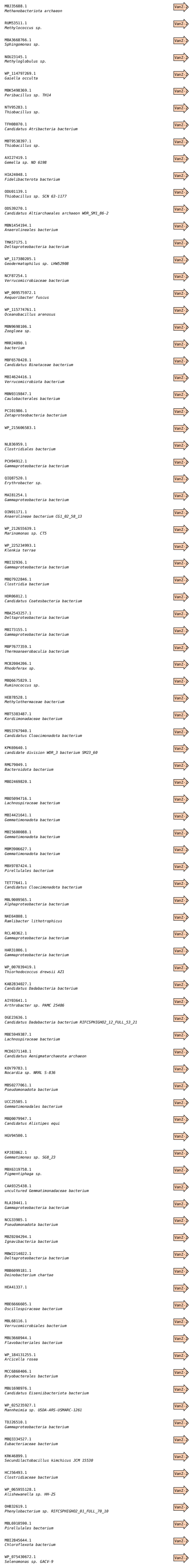
MBJ35688.1
Methanobacteriota archaeon
VanZ-2
RUM53511.1
Methylococcus sp.
VanZ-2
MBA3668766.1
Sphingomonas sp.
VanZ-2
NOU23145.1
Methyloglobulus sp.
VanZ-2
WP_114797269.1
Gaiella occulta
VanZ-2
MBK5498369.1
Peribacillus sp. TH14
VanZ-2
NTV95283.1
Thiobacillus sp.
VanZ-2
TFH08070.1
Candidatus Atribacteria bacterium
VanZ-2
MBT9538397.1
Thiobacillus sp.
VanZ-2
AXI27419.1
Gemella sp. ND 6198
VanZ-2
HIA24048.1
Fidelibacterota bacterium
VanZ-2
ODU01139.1
Thiobacillus sp. SCN 63-1177
VanZ-2
ODS39270.1
Candidatus Altiarchaeales archaeon WOR_SM1_86-2
VanZ-2
MBN1454194.1
Anaerolineales bacterium
VanZ-2
TMA57175.1
Deltaproteobacteria bacterium
VanZ-2
WP_117380205.1
Geodermatophilus sp. LHW52908
VanZ-2
NCF87254.1
Verrucomicrobiaceae bacterium
VanZ-2
WP_009575972.1
Aequoribacter fuscus
VanZ-2
WP_115774761.1
Oceanobacillus arenosus
VanZ-2
MBN9698106.1
Zoogloea sp.
VanZ-2
MRR24090.1
bacterium
VanZ-2
MBF6570428.1
Candidatus Binataceae bacterium
VanZ-2
MBI4624416.1
Verrucomicrobiota bacterium
VanZ-2
MBN9319847.1
Caulobacterales bacterium
VanZ-2
PCI01986.1
Zetaproteobacteria bacterium
VanZ-2
WP_215606583.1
VanZ-2
NLB36959.1
Clostridiales bacterium
VanZ-2
PCH94912.1
Gammaproteobacteria bacterium
VanZ-2
QIQ87520.1
Erythrobacter sp.
VanZ-2
MAI81254.1
Gammaproteobacteria bacterium
VanZ-2
OIN91171.1
Anaerolineae bacterium CG1_02_58_13
VanZ-2
WP_212655639.1
Marinomonas sp. CT5
VanZ-2
WP_225234993.1
Klenkia terrae
VanZ-2
MBI32936.1
Gammaproteobacteria bacterium
VanZ-2
MBQ7922846.1
Clostridia bacterium
VanZ-2
HDR06012.1
Candidatus Coatesbacteria bacterium
VanZ-2
MBA2543257.1
Deltaproteobacteria bacterium
VanZ-2
MBI73155.1
Gammaproteobacteria bacterium
VanZ-2
MBP7677359.1
Thermoanaerobaculia bacterium
VanZ-2
MCB2004206.1
Rhodoferax sp.
VanZ-2
MBQ6675829.1
Ruminococcus sp.
VanZ-2
HEB78528.1
Methylothermaceae bacterium
VanZ-2
MBT5383487.1
Kordiimonadaceae bacterium
VanZ-2
MBS3767940.1
Candidatus Cloacimonadota bacterium
VanZ-2
KPK69640.1
candidate division WOR_3 bacterium SM23_60
VanZ-2
RMG79049.1
Bacteroidota bacterium
VanZ-2
MBO2469820.1
VanZ-2
MBO5094716.1
Lachnospiraceae bacterium
VanZ-2
MBI4421641.1
Gemmatimonadota bacterium
VanZ-2
MBI5600088.1
Gemmatimonadota bacterium
VanZ-2
MBM3906627.1
Gemmatimonadota bacterium
VanZ-2
MBX9787424.1
Pirellulales bacterium
VanZ-2
TET77641.1
Candidatus Cloacimonadota bacterium
VanZ-2
MBL9009565.1
Alphaproteobacteria bacterium
VanZ-2
NKE64808.1
Ramlibacter lithotrophicus
VanZ-2
RCL40362.1
Gammaproteobacteria bacterium
VanZ-2
HAR31006.1
Gammaproteobacteria bacterium
VanZ-2
WP_007039419.1
Thiorhodococcus drewsii AZ1
VanZ-2
KAB2834027.1
Candidatus Dadabacteria bacterium
VanZ-2
AIY03641.1
Arthrobacter sp. PAMC 25486
VanZ-2
OGE23636.1
Candidatus Dadabacteria bacterium RIFCSPHIGHO2_12_FULL_53_21
VanZ-2
MBE5949387.1
Lachnospiraceae bacterium
VanZ-2
MCD6371148.1
Candidatus Aenigmatarchaeota archaeon
VanZ-2
KOV79783.1
Nocardia sp. NRRL S-836
VanZ-2
MBS0277061.1
Pseudomonadota bacterium
VanZ-2
UCC25505.1
Gemmatimonadales bacterium
VanZ-2
MBQ0079947.1
Candidatus Alistipes equi
VanZ-2
HGV94500.1
VanZ-2
KPJ83062.1
Gemmatimonas sp. SG8_23
VanZ-2
MBX6319758.1
Pigmentiphaga sp.
VanZ-2
CAA9325438.1
uncultured Gemmatimonadaceae bacterium
VanZ-2
RLA19441.1
Gammaproteobacteria bacterium
VanZ-2
NCG33985.1
Pseudomonadota bacterium
VanZ-2
MBZ0204294.1
Ignavibacteria bacterium
VanZ-2
MBW2214022.1
Deltaproteobacteria bacterium
VanZ-2
MBB6099181.1
Deinobacterium chartae
VanZ-2
HEA41337.1
VanZ-2
MBE6666605.1
Oscillospiraceae bacterium
VanZ-2
MBL68116.1
Verrucomicrobiales bacterium
VanZ-2
MBU3660944.1
Flavobacteriales bacterium
VanZ-2
WP_184131255.1
Arcicella rosea
VanZ-2
MCC6860406.1
Bryobacterales bacterium
VanZ-2
MBU1698976.1
Candidatus Eiseniibacteriota bacterium
VanZ-2
WP_025235927.1
Mannheimia sp. USDA-ARS-USMARC-1261
VanZ-2
TDJ26510.1
Gammaproteobacteria bacterium
VanZ-2
MBQ3334527.1
Eubacteriaceae bacterium
VanZ-2
KRK46899.1
Secundilactobacillus kimchicus JCM 15530
VanZ-2
HCJ56493.1
Clostridiaceae bacterium
VanZ-2
WP_065955128.1
Alishewanella sp. HH-ZS
VanZ-2
OHB32619.1
Phenylobacterium sp. RIFCSPHIGHO2_01_FULL_70_10
VanZ-2
MBL6910590.1
Pirellulales bacterium
VanZ-2
MBI2845644.1
Chloroflexota bacterium
VanZ-2
WP_075430672.1
Selenomonas sp. GACV-9
VanZ-2

MBR4012863.1	*Clostridia bacterium*	VanZ-2
WP_182216719.1	*Colwellia sp. BRX8-2*	VanZ-2
NNF59110.1		VanZ-2
MBM3161463.1	*Bacteroidota bacterium*	VanZ-2
HAC78786.1	*Deltaproteobacteria bacterium*	VanZ-2
HEB98503.1	*Thiotrichales bacterium*	VanZ-2
KXH77763.1	*Candidatus Thorarchaeota archaeon SMTZ1-83*	VanZ-2
MCD6501032.1	*bacterium*	VanZ-2
WP_065694196.1	*Rhizobium sp. AC44/96*	VanZ-2
WP_007812994.1	*Rhizobium sp. CF142*	VanZ-2
MBT3568976.1	*Opitutia bacterium*	VanZ-2
WP_192118336.1	*Rhizobium sp. CFBP 8762*	VanZ-2
MBP8233471.1	*Rhizorhabdus sp.*	VanZ-2
MBT0801439.1		VanZ-2
MSU51919.1	*Opitutaceae bacterium*	VanZ-2
ADE55186.1	*Coraliomargarita akajimensis DSM 45221*	VanZ-2
OFY66448.1	*Bacteroidetes bacterium RBG_13_43_22*	VanZ-2
NVN87175.1	*Rhodopseudomonas sp.*	VanZ-2
QJE73858.1	*Aerophototrophica crusticola*	VanZ-2
NVK24809.1	*Gammaproteobacteria bacterium*	VanZ-2
MBV9122826.1	*Planctomycetota bacterium*	VanZ-2
MBS0205143.1	*Planctomycetota bacterium*	VanZ-2
MBF0221016.1	*Desulfobulbaceae bacterium*	VanZ-2
MBI5932887.1		VanZ-2
MBI4579977.1	*Planctomycetota bacterium*	VanZ-2
MCA1764868.1	*Desulfobulbaceae bacterium*	VanZ-2
WP_027497986.1	*Rhodococcus sp. JG-3*	VanZ-2
GIS53748.1	*bacterium*	VanZ-2
WP_008714413.1	*Rhodococcus sp. AW25M09*	VanZ-2
OPZ94194.1	*Planctomycetes bacterium ADurb.Bin412*	VanZ-2
MBO5573590.1	*Clostridium sp.*	VanZ-2
MCA9213649.1	*Planctomycetales bacterium*	VanZ-2
WP_194136990.1	*Salipiger mangrovisoli*	VanZ-2
MBQ9471809.1	*Ruminococcus sp.*	VanZ-2
RKZ33935.1	*bacterium*	VanZ-2
WP_068427063.1	*Rhodococcus sp. B10*	VanZ-2
HCA50784.1	*Planctomycetaceae bacterium*	VanZ-2
MBI4709269.1	*Candidatus Portnoybacteria bacterium*	VanZ-2
MBQ5420783.1	*Clostridium sp.*	VanZ-2
MBV1873321.1	*Gammaproteobacteria bacterium*	VanZ-2
RKD75238.1	*Sinobaca qinghaiensis*	VanZ-2
PSQ27281.1	*Halobacteriales archaeon QS_9_68_17*	VanZ-2
NCN85536.1	*Sphingomonadales bacterium*	VanZ-2
WP_051319135.1	*Chitinimonas koreensis*	VanZ-2
MCA9806085.1	*Cyanobacteria bacterium HKST-UBA02*	VanZ-2
MCB1774517.1	*Gammaproteobacteria bacterium*	VanZ-2
MCB1763626.1	*Gammaproteobacteria bacterium*	VanZ-2
MBM3136136.1	*Chloroflexota bacterium*	VanZ-2
NLK27154.1	*Clostridiales bacterium*	VanZ-2
MCB1752587.1	*Gammaproteobacteria bacterium*	VanZ-2
MCC7121875.1	*Gammaproteobacteria bacterium*	VanZ-2
WP_100914138.1	*Pseudoalteromonas spongiae*	VanZ-2
NOR11321.1	*Desulfovibrionaceae bacterium*	VanZ-2
MBL38632.1	*Lysobacterales bacterium*	VanZ-2
WP_034133551.1	*Pseudomonas azotoformans*	VanZ-2
NQT95799.1	*Candidatus Omnitrophota bacterium*	VanZ-2
NQZ67699.1	*Lentisphaeria bacterium*	VanZ-2
WP_231723824.1	*Gemella sp. oral taxon 928*	VanZ-2
KPK52042.1	*Myxococcales bacterium SG8_38_1*	VanZ-2
OON99442.1	*Epulopiscium sp. Nele67-Bin004*	VanZ-2
PIP25697.1	*Candidatus Moranbacteria bacterium CG23_combo_of_CG06-09_8_20_14_all_41_28*	VanZ-2
ARU29986.1	*Cellvibrio sp. PSBB006*	VanZ-2
MBP9903199.1	*Verrucomicrobiota bacterium*	VanZ-2
KPK37983.1	*Omnitrophica WOR_2 bacterium SM23_29*	VanZ-2
HGH07843.1		VanZ-2
MBK7282454.1	*Candidatus Aalborgicola defluviihabitans*	VanZ-2
MBQ8832530.1	*Paludibacteraceae bacterium*	VanZ-2
MBQ7438397.1	*Paludibacteraceae bacterium*	VanZ-2
MBQ3673624.1	*Paludibacteraceae bacterium*	VanZ-2
TAH50911.1	*Betaproteobacteria bacterium*	VanZ-2
NPA74865.1	*Methanobacteriota archaeon*	VanZ-2
MBU1045389.1	*Candidatus Omnitrophota bacterium*	VanZ-2
MBU1007091.1	*Candidatus Omnitrophota bacterium*	VanZ-2
MBQ3998196.1	*Paludibacteraceae bacterium*	VanZ-2
RMF90618.1	*Methanobacteriota archaeon*	VanZ-2
RKW12179.1	*Capnocytophaga sp.*	VanZ-2
MBU0549823.1	*Candidatus Omnitrophota bacterium*	VanZ-2
HAY2527104.1	*Enterococcus faecium*	VanZ-2
PYX84806.1	*Acidobacteriota bacterium*	VanZ-2
MBN2484179.1	*Candidatus Omnitrophota bacterium*	VanZ-2
AXJ96646.1	*Sphingomonas sp. FARSPH*	VanZ-2
MBQ9322296.1	*Eubacterium sp.*	VanZ-2
MBD1160495.1	*Pelagibacterales bacterium SAG-MED14*	VanZ-2
MCA0427265.1	*Bacteroidota bacterium*	VanZ-2
HEC99963.1	*Pseudomonadota bacterium*	VanZ-2
MBI4698131.1	*Nitrospirota bacterium*	VanZ-2
WP_051218886.1	*Oceanobacter kriegii DSM 6294*	VanZ-2
OGQ93349.1	*Deltaproteobacteria bacterium RIFOXYA12_FULL_61_11*	VanZ-2
MBC7160185.1	*Porphyrobacter sp.*	VanZ-2
CDD46016.1	*Firmicutes bacterium CAG:534*	VanZ-2
MBB89209.1	*Methanobacteriota archaeon*	VanZ-2
OYU36185.1	*Novosphingobium sp. PASSN1*	VanZ-2
OGP18698.1	*Deltaproteobacteria bacterium GWA2_55_10*	VanZ-2

MCB2080036.1	*Novosphingobium sp.*	VanZ-2
MBP8260374.1	*Verrucomicrobiota bacterium*	VanZ-2
MCD6532245.1	*bacterium*	VanZ-2
MBO4280662.1	*Lachnospiraceae bacterium*	VanZ-2
RVT54531.1	*Rubrivivax albus*	VanZ-2
MBV15012.1	*Methanobacteriota archaeon*	VanZ-2
MBK6609717.1	*Sphingobacteriales bacterium*	VanZ-2
MCE2787397.1	*Sphingobacteriales bacterium*	VanZ-2
MBF0413860.1	*Desulfamplus sp.*	VanZ-2
MBP7825848.1	*Verrucomicrobiota bacterium*	VanZ-2
KXJ40332.1	*Methylothermaceae bacteria B42*	VanZ-2
NLV92572.1	*Bacillota bacterium*	VanZ-2
MBQ2727448.1	*Clostridia bacterium*	VanZ-2
MBL8110668.1	*Anaerolineales bacterium*	VanZ-2
WP_142585892.1	*uncultured Methylobacterium sp.*	VanZ-2
MBL8078491.1	*Anaerolineales bacterium*	VanZ-2
WP_152274731.1	*Arthrobacter koreensis*	VanZ-2
MBE7186341.1	*Methylobacterium mesophilicum*	VanZ-2
WP_099952815.1	*Methylobacterium currus*	VanZ-2
GJD45213.1	*Methylobacterium cerastii*	VanZ-2
MBQ7069707.1	*Ruminococcus sp.*	VanZ-2
WP_183430139.1	*Mesorhizobium sp. RMAD-H1*	VanZ-2
MBR5379401.1	*Clostridia bacterium*	VanZ-2
MBR0190252.1	*Clostridia bacterium*	VanZ-2
NBS98259.1	*Betaproteobacteria bacterium*	VanZ-2
MBD3823204.1	*Campylobacterota bacterium*	VanZ-2
TMI03593.1	*Betaproteobacteria bacterium*	VanZ-2
WP_024708620.1	*Martelella sp. AD-3*	VanZ-2
WP_231858875.1	*Haloquadratum walsbyi DSM 16790*	VanZ-2
WP_182121655.1	*Neisseria shayeganii*	VanZ-2
WP_170107721.1	*Photobacterium lipolyticum*	VanZ-2
WP_062689499.1	*Photobacterium sanguinicancri*	VanZ-2
WP_213887474.1	*Pseudohalocynthiibacter aestuariivivens*	VanZ-2
HIG75316.1	*Bacteroidota bacterium*	VanZ-2
PCI70050.1	*Piscirickettsiaceae bacterium*	VanZ-2
HIF35645.1	*Candidatus Thioglobus sp.*	VanZ-2
MBN2117658.1	*Anaerolineales bacterium*	VanZ-2
WP_184463725.1	*Zhongshania antarctica*	VanZ-2
MBW2059092.1	*Deltaproteobacteria bacterium*	VanZ-2
NPA06571.1	*Chloroflexota bacterium*	VanZ-2
WP_064198403.1	*Emticicia oligotrophica*	VanZ-2
SHH62154.1	*Bradyrhizobium erythrophlei*	VanZ-2
MBQ0789211.1	*Cycloclasticus sp.*	VanZ-2
MBP2043116.1		VanZ-2
MBJ7553070.1	*Marinomonas spartinae*	VanZ-2
OTE96502.1	*Crenothrix sp. D3*	VanZ-2
NMC86633.1	*Anaerolineaceae bacterium*	VanZ-2
MBR4993266.1	*Lachnospiraceae bacterium*	VanZ-2
MBQ3326356.1	*Clostridia bacterium*	VanZ-2
KAF0107388.1	*Anaerolineaceae bacterium*	VanZ-2
MBN9056710.1	*Hyphomicrobiales bacterium*	VanZ-2
HIS84924.1	*Candidatus Faecivicinus avistercoris*	VanZ-2
RPI36702.1	*Nitrospiraceae bacterium*	VanZ-2
TCD47107.1	*Chlorobium sp. N1*	VanZ-2
MBK6871596.1	*Kineosporiaceae bacterium*	VanZ-2
TMI84617.1	*Bacteroidota bacterium*	VanZ-2
KAF0814087.1	*Andreprevotia sp. IGB-42*	VanZ-2
WP_127766260.1	*Hwanghaeella grinnelliae*	VanZ-2
WP_014086451.1	*Pseudogulbenkiania sp. NH8B*	VanZ-2
MBA2342190.1	*Thermoleophilaceae bacterium*	VanZ-2
HHH19126.1	*Campylobacterales bacterium*	VanZ-2
MBN8609378.1	*Deltaproteobacteria bacterium*	VanZ-2
UCF83693.1	*Desulfobacteraceae bacterium*	VanZ-2
MBX3038964.1	*Anaerolineales bacterium*	VanZ-2
CCY67658.1	*Clostridium sp. CAG:678*	VanZ-2
MCB1586721.1	*Lysobacterales bacterium*	VanZ-2
MBE3570840.1	*Bacillales bacterium*	VanZ-2
SOE22376.1	*Spirosomataceae bacterium TFI 002*	VanZ-2
WP_191773973.1	*Devosia oryzisoli*	VanZ-2
MBW3551880.1	*Gemmatimonadota bacterium*	VanZ-2
HID94818.1	*Candidatus Latescibacterota bacterium*	VanZ-2
MBA2602472.1	*Acidobacteriota bacterium*	VanZ-2
WP_182545091.1	*Halosaccharopolyspora lacisalsi*	VanZ-2
MBQ8747729.1	*Clostridia bacterium*	VanZ-2
MBW8816064.1	*Caulobacterales bacterium*	VanZ-2
HBI94556.1	*Terrisporobacter hibernicus*	VanZ-2
MBK5189185.1	*Gemmatimonadaceae bacterium*	VanZ-2
MBI1905315.1	*Rhodocyclales bacterium*	VanZ-2
PIP29592.1	*Candidatus Kuenenbacteria bacterium CG23_combo_of_CG06-09_8_20_14_all_36_9*	VanZ-2
MCD4743542.1	*Desulfobacteraceae bacterium*	VanZ-2
RYF95676.1	*Caulobacteraceae bacterium*	VanZ-2
MBI4320198.1	*Chloroflexota bacterium*	VanZ-2
NLM14101.1	*Candidatus Epulonipiscium sp.*	VanZ-2
NUS62688.1	*Saccharothrix sp.*	VanZ-2
WP_213166970.1	*Alkalicella caledoniensis*	VanZ-2
OON91442.1	*Epulopiscium sp. Nele67-Bin001*	VanZ-2
NGX96023.1	*Candidatus Afipia apatlaquensis*	VanZ-2
MBK6656184.1	*Zoogloea sp.*	VanZ-2
WP_025659121.1	*Rhizobium sp. IBUN*	VanZ-2
MBQ1550670.1	*Bacilli bacterium*	VanZ-2
MCB9113045.1	*Anaerolineales bacterium*	VanZ-2
OHD94579.1	*Sulfurimonas sp. GWF2_37_8*	VanZ-2
NQT96294.1	*Fidelibacterota bacterium*	VanZ-2

KPV40370.1	*Thiohalorhabdus denitrificans*	VanZ-2
WP_007602939.1	*Bradyrhizobium sp. WSM1253*	VanZ-2
WP_010898450.1	*Halalkalibacterium halodurans*	VanZ-2
WP_144234760.1	*Oceanidesulfovibrio marinus*	VanZ-2
MBR3429335.1	*Clostridia bacterium*	VanZ-2
RLG59771.1	*Candidatus Hydrothermarchaeota archaeon*	VanZ-2
MBV6505187.1	*Syntrophorhabdaceae bacterium*	VanZ-2
MBA2390361.1	*Geodermatophilaceae bacterium*	VanZ-2
MBT3217142.1	*Fidelibacterota bacterium*	VanZ-2
MBE2223028.1	*Anaerolineae bacterium*	VanZ-2
WP_100319152.1	*Roseinatronobacter bogoriensis DSM 18756*	VanZ-2
ETW98418.1	*Candidatus Entotheonella gemina*	VanZ-2
ESP95171.1	*Pseudoalteromonas luteoviolacea 2ta16*	VanZ-2
MBQ0114418.1	*Candidatus Sodaliphilus aphodohippi*	VanZ-2
MCA9902828.1	*Anaerolineae bacterium*	VanZ-2
BCA53199.1	*Nitrospira sp. KM1*	VanZ-2
MBE6939520.1	*Oscillospiraceae bacterium*	VanZ-2
AEN05911.1	*halophilic archaeon DL31*	VanZ-2
MCD4715150.1	*Desulfobacterales bacterium*	VanZ-2
WP_010127717.1	*Sphingomonas sp. KC8*	VanZ-2
WP_171131874.1	*Sphingomonas sp. ID1715*	VanZ-2
OIR19018.1	*Marine Group III euryarchaeote CG-Epi1*	VanZ-2
UGS36230.1	*Capillimicrobium parvum*	VanZ-2
MBI5746800.1	*Nitrospirota bacterium*	VanZ-2
MBO5213257.1	*Clostridia bacterium*	VanZ-2
OIP50426.1	*Desulfobacterales bacterium CG2_30_60_27*	VanZ-2
HIU55066.1	*Candidatus Gallibacteroides avistercoris*	VanZ-2
MBU0831943.1	*Alphaproteobacteria bacterium*	VanZ-2
MBQ3633852.1	*Paludibacteraceae bacterium*	VanZ-2
MBK9444341.1	*Candidatus Rhodoferax mariagerensis*	VanZ-2
WP_225255050.1	*Jidongwangia harbinensis*	VanZ-2
MBD3264374.1	*Candidatus Omnitrophota bacterium*	VanZ-2
MBE1382586.1		VanZ-2
MCB1704813.1	*Halioglobus sp.*	VanZ-2
MBN1272585.1	*Candidatus Aminicenantes bacterium*	VanZ-2
MBR95035.1	*Micrococcales bacterium*	VanZ-2
WP_006524145.1	*Desulfoscipio gibsoniae DSM 7213*	VanZ-2
WP_153822137.1	*Polyangium spumosum*	VanZ-2
WP_075049358.1	*Ignicoccus islandicus DSM 13165*	VanZ-2
MBP6635377.1	*Paludibacter sp.*	VanZ-2
WP_143957961.1	*Rhodoligotrophos appendicifer*	VanZ-2
OIP87246.1	*Syntrophaceae bacterium CG2_30_49_12*	VanZ-2
MBB1091577.1	*Rhodopseudomonas palustris*	VanZ-2
WP_160971303.1	*Pseudomaricurvus sp. HS19*	VanZ-2
WP_223981694.1	*Cupriavidus pampae*	VanZ-2
MBI5874232.1	*Candidatus Omnitrophota bacterium*	VanZ-2
MBM3248879.1	*Candidatus Omnitrophota bacterium*	VanZ-2
MBL8169377.1	*Acidobacteriota bacterium*	VanZ-2
MBL4607293.1	*Pseudomonadales bacterium*	VanZ-2
UJA19544.1	*Thermoleophilia bacterium SCSIO 60948*	VanZ-2
MBP6920344.1	*Candidatus Omnitrophota bacterium*	VanZ-2
NOL47459.1	*Synechococcus sp. MIT S9220*	VanZ-2
MBU3958683.1	*Candidatus Omnitrophota bacterium*	VanZ-2
QQG39951.1	*Candidatus Aenigmatarchaeota archaeon*	VanZ-2
OPX29671.1	*Candidatus Omnitrophica bacterium 4484_171*	VanZ-2
MBS1137799.1	*Pseudomonadota bacterium*	VanZ-2
MBQ3866540.1	*Clostridia bacterium*	VanZ-2
HJH49692.1	*Merdimonas faecis*	VanZ-2
MBI08725.1	*Rhodospirillaceae bacterium*	VanZ-2
MBR4835919.1	*Clostridia bacterium*	VanZ-2
WP_169546226.1	*Sneathiella aquimaris*	VanZ-2
OQX20111.1	*Desulfobulbaceae bacterium A2*	VanZ-2
HIA97831.1	*Marine Group III euryarchaeote*	VanZ-2
WP_119334576.1	*Hydrogenophilus thermoluteolus*	VanZ-2
MBR1757931.1	*Lachnospiraceae bacterium*	VanZ-2
WP_168907994.1	*Paenibacillus albicereus*	VanZ-2
MBC8551783.1	*Candidatus Brocadiales bacterium*	VanZ-2
MBP7947860.1	*Verrucomicrobiota bacterium*	VanZ-2
WP_162271563.1	*Gemmata sp. SH-PL17*	VanZ-2
WP_152475793.1	*Roseovarius sp. THAF9*	VanZ-2
KPK63934.1	*candidate division WOR_3 bacterium SM23_42*	VanZ-2
PYJ65186.1	*Verrucomicrobiota bacterium*	VanZ-2
MCC6415201.1	*Opitutaceae bacterium*	VanZ-2
OQX15783.1	*Desulfobulbaceae bacterium A2*	VanZ-2
MBL8757815.1	*Phycisphaerae bacterium*	VanZ-2
MBR6820068.1	*Bacteroidaceae bacterium*	VanZ-2
OLA72004.1	*Ruminococcus sp. 37_24*	VanZ-2
MBE6595799.1	*Oscillospiraceae bacterium*	VanZ-2
PCI14615.1	*Thiotrichales bacterium*	VanZ-2
MCA9114083.1	*Planctomycetaceae bacterium*	VanZ-2
MCB0922530.1	*Actinomycetota bacterium*	VanZ-2
MBI5879410.1	*Chloroflexota bacterium*	VanZ-2
NLK81440.1	*Bacteroidales bacterium*	VanZ-2
MBP7146811.1	*Acidobacteriota bacterium*	VanZ-2
UCG44023.1	*candidate division WOR-3 bacterium*	VanZ-2
MBG7602772.1	*Gammaproteobacteria bacterium*	VanZ-2
MCA9264407.1	*Planctomycetales bacterium*	VanZ-2
MBD3672608.1	*Planctomycetaceae bacterium*	VanZ-2
OGP05530.1	*Deltaproteobacteria bacterium GWA2_38_16*	VanZ-2
HHM98526.1		VanZ-2
MCC5879733.1	*Idiomarina sp.*	VanZ-2
HAW46916.1	*Roseovarius sp.*	VanZ-2
GIS22389.1	*Gammaproteobacteria bacterium*	VanZ-2

NNC66477.1
Gammaproteobacteria bacterium — VanZ-2

MBV7323297.1 — VanZ-2

MBM3133781.1
Chloroflexota bacterium — VanZ-2

WP_050069896.1
Anaerosalibacter massiliensis — VanZ-2

MBA2537867.1
Actinomycetota bacterium — VanZ-2

WP_162883754.1
Chromatocurvus halotolerans — VanZ-2

PHS34057.1
Sulfurovum sp. — VanZ-2

MCB2082095.1
Rickettsiales bacterium — VanZ-2

WP_051461461.1
Tomitella biformata — VanZ-2

HHU14041.1
Lentisphaerota bacterium — VanZ-2

TMJ97945.1
Actinomycetota bacterium — VanZ-2

WP_127699221.1
Rheinheimera riviphila — VanZ-2

WP_051202740.1
Sediminimonas qiaohouensis DSM 21189 — VanZ-2

RME55102.1
Candidatus Woesearchaeota archaeon — VanZ-2

HJC42307.1
Candidatus Mediterraneibacter gallistercoris — VanZ-2

PYS24984.1
Acidobacteriota bacterium — VanZ-2

MBE9529534.1
Pseudomonadota bacterium — VanZ-2

MBS0463393.1
Pseudomonadota bacterium — VanZ-2

WP_051395296.1 — VanZ-2

MAA93038.1
Rheinheimera sp. — VanZ-2

PIQ87963.1
Candidatus Omnitrophica bacterium CG11_big_fil_rev_8_21_14_0_20_43_6 — VanZ-2

HAM16208.1
Eggerthellaceae bacterium — VanZ-2

MBQ1898678.1
Ruminococcus sp. — VanZ-2

NUQ39643.1
Caldilineales bacterium — VanZ-2

MBW3618353.1
Pseudomonadota bacterium — VanZ-2

NDQ57682.1
Acidipila sp. — VanZ-2

MCB2182790.1
Desulfobulbaceae bacterium — VanZ-2

WP_068231243.1
Rheinheimera sp. EpRS3 — VanZ-2

WP_205311018.1
Rheinheimera maricola — VanZ-2

PCH62997.1
SAR86 cluster bacterium — VanZ-2

MBQ7300419.1
Clostridia bacterium — VanZ-2

HBH34932.1
Gammaproteobacteria bacterium — VanZ-2

NRB38177.1
Pseudomonadales bacterium — VanZ-2

WP_066575106.1
Sphingomonas koreensis — VanZ-2

TKB66481.1
Nitrospira sp. — VanZ-2

PYV12244.1
Acidobacteriota bacterium — VanZ-2

MBI3008911.1
Candidatus Omnitrophota bacterium — VanZ-2

MBF0253965.1
Candidatus Omnitrophota bacterium — VanZ-2

HIH42244.1
Candidatus Woesearchaeota archaeon — VanZ-2

MBQ4394306.1
Paludibacteraceae bacterium — VanZ-2

TIC81470.1
Crenobacter intestini — VanZ-2

QCD47390.1
Campylobacter rectus — VanZ-2

WP_169972207.1
Campylobacter californiensis — VanZ-2

MBE6976862.1
Oscillospiraceae bacterium — VanZ-2

UCG62917.1
Candidatus Zixiibacteriota bacterium — VanZ-2

MBV9150553.1
Alphaproteobacteria bacterium — VanZ-2

KPK25773.1
Desulfobacterales bacterium SG8_35_2 — VanZ-2

MBK6443312.1
Candidatus Phosphoribacter baldrii — VanZ-2

BCW90681.1
Alphaproteobacteria bacterium SO-S41 — VanZ-2

PIE68226.1
Deltaproteobacteria bacterium — VanZ-2

HAL29416.1
Coriobacteriia bacterium — VanZ-2

MBI4714453.1
Nitrospirota bacterium — VanZ-2

MBC7265788.1
Coriobacteriia bacterium — VanZ-2

TET19886.1
Candidatus Bathyarchaeota archaeon — VanZ-2

MBU6327783.1
Verrucomicrobiota bacterium — VanZ-2

HAD27971.1
Paracoccaceae bacterium — VanZ-2

MBX9740378.1
Beijerinckiaceae bacterium — VanZ-2

MBN1333753.1
Synergistales bacterium — VanZ-2

MBI0474743.1
Sphingomonas sp. MA1305 — VanZ-2

WP_101509708.1
Thermotoga sp. KOL6 — VanZ-2

MBL1279989.1
Fluviicola sp. — VanZ-2

NRA11662.1
Crocinitomicaceae bacterium — VanZ-2

MBM4249612.1
Methanobacteriota archaeon — VanZ-2

MBL4862341.1
Crocinitomicaceae bacterium — VanZ-2

MBU2008589.1
Chloroflexota bacterium — VanZ-2

MBW6460951.1
Bacteroidales bacterium — VanZ-2

MBQ4274198.1
Clostridia bacterium — VanZ-2

MBU1255741.1
Patescibacteria group bacterium — VanZ-2

OLD72151.1
Ignavibacteria bacterium 13_1_40CM_2_61_4 — VanZ-2

MBX7084335.1
Nannocystaceae bacterium — VanZ-2

TMV84711.1
Thioclava sp. BHET1 — VanZ-2

HIF27294.1
Fidelibacterota bacterium — VanZ-2

MCB0216356.1
Chloroflexota bacterium — VanZ-2

MBQ4511022.1
Clostridia bacterium — VanZ-2

WP_215678907.1
Roseburia inulinivorans — VanZ-2

MCA9517001.1
Myxococcales bacterium — VanZ-2

HET88483.1 — VanZ-2

PYJ10859.1
Verrucomicrobiota bacterium — VanZ-2

MCC7268357.1
Caulobacteraceae bacterium — VanZ-2

MBE3072645.1
Acidobacteriota bacterium — VanZ-2

WP_052190573.1
Chitinibacter sp. ZOR0017 — VanZ-2

MBA4376791.1
Anaerolinea sp. — VanZ-2

WP_114917695.1 — VanZ-2

MBM4185050.1
Gemmatimonadota bacterium — VanZ-2

MCA1784187.1
Intrasporangiaceae bacterium — VanZ-2

MBR6646760.1
Clostridia bacterium — VanZ-2

MSR06191.1
Gemmatimonadota bacterium — VanZ-2

MCE1246028.1
Bacillota bacterium — VanZ-2

WP_184421476.1
Thalassotalea piscium — VanZ-2

WP_119322338.1
Capsulimonas corticalis — VanZ-2

MBL8423363.1
Candidatus Accumulibacter phosphatis — VanZ-2

NLV48411.1
Clostridiaceae bacterium — VanZ-2

OJU05860.1
Rhizobium sp. 63-7 — VanZ-2

WP_090743499.1
Mesobacillus persicus — VanZ-2

MBI4731096.1
Chloroflexota bacterium — VanZ-2

NTU97973.1
Chlorobiaceae bacterium — VanZ-2

HHV11241.1
Clostridiales bacterium — VanZ-2

WP_169149761.1
Azoarcus sp. TTM-91 — VanZ-2

MBI3913685.1
Chloroflexota bacterium — VanZ-2

MBO6234556.1 — VanZ-2

OIQ00760.1
Zetaproteobacteria bacterium CG2_30_46_52 — VanZ-2

WP_182542924.1
Halosaccharopolyspora lacisalsi — VanZ-2

WP_169074298.1
Thalassotalea algicola — VanZ-2

MCB1339730.1
Pseudooceanicola sp. — VanZ-2

OGB43248.1
Burkholderiales bacterium RIFOXYC2_FULL_59_8 — VanZ-2

PZU36055.1
Microbacterium sp. — VanZ-2

MBC7373048.1
Frankiales bacterium — VanZ-2

MBT7655577.1
Flavobacteriales bacterium — VanZ-2

MBO7649596.1
Lachnospiraceae bacterium — VanZ-2

OGF16431.1
Candidatus Eisenbacteria bacterium RBG_16_71_46 — VanZ-2

NDC76200.1
bacterium — VanZ-2

WP_002304666.1
Enterococcus faecium — VanZ-2

MBV8568876.1
Methylobacteriaceae bacterium — VanZ-2

WP_066055746.1
Robertmurraya korlensis NBRC 107688 — VanZ-2

MBM3234243.1
Candidatus Pacearchaeota archaeon — VanZ-2

MBM2827450.1
Dehalococcoidia bacterium — VanZ-2

MUV07242.1
Planococcaceae bacterium Storch 2/2-2 — VanZ-2

MBP8173852.1
Aeromonadaceae bacterium — VanZ-2

WP_106136972.1
uncultured Spirosoma sp. — VanZ-2

WP_100345655.1
Compostimonas suwonensis — VanZ-2

NMB61124.1
Chloroflexota bacterium — VanZ-2

MBX3683097.1
Thauera sp. — VanZ-2

NBO91889.1
Planctomycetia bacterium — VanZ-2

WP_051772066.1
Saccharothrix sp. NRRL B-16314 — VanZ-2

MBS3171563.1
Candidatus Woesearchaeota archaeon — VanZ-2

WP_118885971.1
Arcobacter suis — VanZ-2

NTS39659.1
Flavisolibacter longurius — VanZ-2

MBI5710395.1
Candidatus Eiseniibacteriota bacterium — VanZ-2

WP_156861442.1
Oceanicaulis alexandrii DSM 11625 — VanZ-2

MAW92963.1
Methanobacteriota archaeon — VanZ-2

RUM50276.1
Hydrogenothermus sp. — VanZ-2

HGS05800.1 — VanZ-2

MBN51033.1
Spongiibacteraceae bacterium — VanZ-2

WP_193178263.1
Oricola nitratireducens — VanZ-2

OGP25557.1
Deltaproteobacteria bacterium GWB2_55_19 — VanZ-2

MAV49163.1
Pelagibacteraceae bacterium — VanZ-2

MCD6450885.1
Acidobacteriota bacterium — VanZ-2

OGA49790.1
Betaproteobacteria bacterium RIFCSPLOWO2_12_FULL_62_13 — VanZ-2

WP_003047577.1
Thermus aquaticus Y51MC23 — VanZ-2

MBR6338501.1
Ruminococcus sp. — VanZ-2

WP_154149705.1
Roseovarius bejariae — VanZ-2

HIN79626.1
Planctomycetota bacterium — VanZ-2

MBX2886212.1
Granulosicoccus sp. — VanZ-2

MBN1517026.1
Candidatus Sumerlaeota bacterium — VanZ-2

AQS28163.1 — VanZ-2

MBM2835248.1
Candidatus Brocadiaceae bacterium — VanZ-2

MBL1244337.1
Sulfurimonas sp. — VanZ-2

MBV12492.1
Flavobacteriaceae bacterium — VanZ-2

KXT78254.1
Streptococcus sp. DD13 — VanZ-2

OGA36400.1
Betaproteobacteria bacterium RIFCSPLOWO2_12_FULL_64_23 — VanZ-2

MBM3916508.1
Sphingomonadales bacterium — VanZ-2

NTV79626.1
Clostridiales bacterium — VanZ-2

MBQ3101233.1
Clostridia bacterium — VanZ-2

HDQ99050.1
candidate division WOR-3 bacterium — VanZ-2

MQN29233.1 — VanZ-2

MBT4974719.1
Gammaproteobacteria bacterium — VanZ-2

MBN1694365.1
candidate division WOR-3 bacterium — VanZ-2

NPA51672.1
Aquificota bacterium — VanZ-2

MBR4555400.1
Ruminococcus sp. — VanZ-2

HHR83572.1 — VanZ-2

MCA9196179.1
Planctomycetales bacterium — VanZ-2

MBW3665675.1
Actinomycetota bacterium — VanZ-2

WP_105213355.1
Pseudoalteromonas sp. T1lg88 — VanZ-2

MBM3249463.1
Candidatus Omnitrophota bacterium — VanZ-2

MBQ4089589.1
Clostridia bacterium — VanZ-2

MBU1048586.1
Candidatus Bipolaricaulota bacterium — VanZ-2

MCC7246780.1
Saprospiraceae bacterium — VanZ-2

MBE5868497.1
Lachnospiraceae bacterium — VanZ-2

NRA40988.1
Pseudomonadales bacterium — VanZ-2

WP_161990616.1
Candidatus Methylobacter oryzae — VanZ-2

TXT23887.1
Gallionellaceae bacterium — VanZ-2

ARP50376.1
Ruminococcaceae bacterium CPB6 — VanZ-2

MBF0219919.1
Gammaproteobacteria bacterium — VanZ-2

MBU4274076.1
Planctomycetota bacterium — VanZ-2

MBD5390447.1
bacterium — VanZ-2

MBN9147564.1
Nitrobacter sp. — VanZ-2

WP_004336346.1
Thauera linaloolentis — VanZ-2

MBR9922967.1
Bacteroidota bacterium — VanZ-2

WP_025785869.1
Sporosarcina sp. D27 — VanZ-2

MCD6679548.1
Burkholderiaceae bacterium — VanZ-2

MBA3025880.1
Sulfurimonas sp. — VanZ-2

EOS25566.1
Lachnospiraceae bacterium 3-1 — VanZ-2

WP_223303269.1 — VanZ-2

MBQ2067408.1
Paludibacteraceae bacterium — VanZ-2

QLQ08982.1
Anaerolineae bacterium — VanZ-2

WP_105253901.1
Pseudoalteromonas sp. T1lg75 — VanZ-2

KPK00399.1	*Nitrospira bacterium SG8_35_4*	VanZ-2
PAW76465.1	*Verrucomicrobia bacterium Tous-C9LFEB*	VanZ-2
MBI5509243.1	*Deltaproteobacteria bacterium*	VanZ-2
NOR72827.1	*Mariprofundaceae bacterium*	VanZ-2
WP_224664160.1	*Mesorhizobium sp. ES1-1*	VanZ-2
MBD9098421.1	*bacterium*	VanZ-2
HIQ28108.1	*Sulfurovum sp.*	VanZ-2
MBX9859843.1	*Sphingomonas sp.*	VanZ-2
EAW31725.1	*marine gamma proteobacterium HTCC2143*	VanZ-2
MBQ8055527.1	*Paludibacteraceae bacterium*	VanZ-2
CVI65577.1	*Clostridiales bacterium CHKCI001*	VanZ-2
MAI06278.1	*Rhodospirillaceae bacterium*	VanZ-2
HHR45918.1		VanZ-2
MBI2672033.1	*Candidatus Woesearchaeota archaeon*	VanZ-2
MCB8997724.1	*Actinomycetota bacterium*	VanZ-2
MBT5419569.1	*Candidatus Cloacimonadota bacterium*	VanZ-2
WP_212547949.1	*Pseudoalteromonas sp. BDTF-M6*	VanZ-2
NUN16681.1	*Myxococcales bacterium*	VanZ-2
ALZ77825.1	*Rheinheimera sp. F8*	VanZ-2
MBR3646942.1	*Paludibacteraceae bacterium*	VanZ-2
RZO16236.1	*SAR86 cluster bacterium*	VanZ-2
MCD4828068.1	*Candidatus Cloacimonadota bacterium*	VanZ-2
MBV8591803.1	*Acetobacteraceae bacterium*	VanZ-2
NQZ82139.1	*Colwellia sp.*	VanZ-2
MBK8433145.1	*Candidatus HaderslevielIa danica*	VanZ-2
RJP53194.1	*Anaerolineaceae bacterium*	VanZ-2
MBF0237488.1	*SAR324 cluster bacterium*	VanZ-2
MBK7027141.1	*Candidatus Aalborgiella aggregata*	VanZ-2
MCD7740840.1	*Ruminococcus sp.*	VanZ-2
MBW8295699.1	*Sphingopyxis sp.*	VanZ-2
WP_193023420.1	*Thalassotalea sp. LPB0316*	VanZ-2
WP_223072739.1	*Erythrobacter sp. SCSIO 43205*	VanZ-2
NCP22976.1	*Erythrobacter sp.*	VanZ-2
WP_104530442.1	*Blastococcus saxobsidens*	VanZ-2
WP_154245792.1	*Kangiella sp. HZ709*	VanZ-2
HHS03164.1		VanZ-2
WP_024299092.1	*Methylomicrobium lacus LW14*	VanZ-2
MBF8267867.1	*Dehalococcoidia bacterium*	VanZ-2
CCQ11100.1	*Pseudoalteromonas luteoviolacea B = ATCC 29581*	VanZ-2
MBA4416669.1	*Syntrophus sp. (in: bacteria)*	VanZ-2
WP_193954702.1	*Pontibacterium sinense*	VanZ-2
MBC8414793.1	*bacterium*	VanZ-2
MBQ9197456.1	*Clostridia bacterium*	VanZ-2
HAJ12101.1	*Comamonadaceae bacterium*	VanZ-2
NOQ35151.1	*Methylococcaceae bacterium*	VanZ-2
MBV8651299.1	*Alphaproteobacteria bacterium*	VanZ-2
WP_106334914.1	*Alsobacter soli*	VanZ-2
WP_143250706.1		VanZ-2
HAB66242.1	*Bacillota bacterium*	VanZ-2
EFP62684.1	*Erysipelotrichaceae bacterium 3_1_53*	VanZ-2
NBR33899.1	*Paracoccaceae bacterium*	VanZ-2
WP_161972969.1	*Halostella pelagica*	VanZ-2
WP_065791624.1	*Pseudoalteromonas luteoviolacea*	VanZ-2
HAB39108.1	*Paracoccaceae bacterium*	VanZ-2
GGX92966.1	*Vogesella alkaliphila*	VanZ-2
WP_063377861.1	*Pseudoalteromonas luteoviolacea NCIMB 1942*	VanZ-2
TNE73608.1	*Bacteroidota bacterium*	VanZ-2
WP_171578579.1	*Bradyrhizobium australiense*	VanZ-2
WP_079568389.1	*Bradyrhizobium erythrophlei*	VanZ-2
KPJ93393.1	*Gemmatimonas sp. SG8_17*	VanZ-2
MBQ6395138.1	*Atopobiaceae bacterium*	VanZ-2
WP_128965192.1	*Bradyrhizobium guangdongense*	VanZ-2
GAC42618.1	*Paenibacillus popilliae ATCC 14706*	VanZ-2
MBW1721280.1	*Deltaproteobacteria bacterium*	VanZ-2
WP_162143281.1	*Chitinilyticum aquatile DSM 21506*	VanZ-2
WP_161853158.1	*Bradyrhizobium sp. CCBAU 051011*	VanZ-2
MBA3773569.1	*Ramlibacter sp.*	VanZ-2
MBQ8687310.1	*Ruminococcus sp.*	VanZ-2
MBI3962103.1	*Deinococcus sp.*	VanZ-2
WP_012816949.1	*Zymomonas mobilis*	VanZ-2
MAL68543.1	*Porticoccaceae bacterium*	VanZ-2
OGW16440.1	*Nitrospinae bacterium RIFCSPLOWO2_12_FULL_45_22*	VanZ-2
MBL8960171.1	*Gemmatimonadota bacterium*	VanZ-2
MBN1656275.1	*Deltaproteobacteria bacterium*	VanZ-2
MCE2865126.1	*Cryomorphaceae bacterium*	VanZ-2
MBI5416624.1	*Candidatus Poribacteria bacterium*	VanZ-2
MBQ0798480.1	*Porticoccaceae bacterium*	VanZ-2
WP_152421082.1	*Natronolimnohabitans innermongolicus JCM 12255*	VanZ-2
PYN22788.1	*Candidatus Rokuibacteriota bacterium*	VanZ-2
WP_029582189.1	*Bradyrhizobium sp. URHD0069*	VanZ-2
WP_183409130.1	*Litorivivens lipolytica*	VanZ-2
NMD30376.1	*Chloroflexota bacterium*	VanZ-2
MBN2049159.1	*Spirochaetales bacterium*	VanZ-2
HIP02895.1	*Campylobacterales bacterium*	VanZ-2
MBE1543406.1		VanZ-2
PTL36840.1	*Candidatus Methylomirabilis limnetica*	VanZ-2
WP_024517515.1	*Bradyrhizobium sp. Tv2a-2*	VanZ-2
RLS34069.1	*Planctomycetota bacterium*	VanZ-2
MBL8454183.1	*Zoogloea sp.*	VanZ-2
WP_140904490.1	*Brucella gallinifaecis*	VanZ-2
MBC7561332.1	*Gemmatimonadaceae bacterium*	VanZ-2
WP_008082576.1	*Aciduliprofundum boonei T469*	VanZ-2
NRB78881.1	*Saccharospirillaceae bacterium*	VanZ-2

MBA2572672.1	*Gemmatimonadota bacterium*	VanZ
MBT3738448.1	*Fidelibacterota bacterium*	VanZ
WP_224879632.1	*Guptibacillus algicola*	VanZ
WP_062283796.1	*Rhizobium sp. Root274*	VanZ
RMH96391.1	*Calditrichota bacterium*	VanZ
MBO5374427.1	*Clostridia bacterium*	VanZ
NUP89441.1	*Candidatus Sumerlaeia bacterium*	VanZ
MBO5397324.1	*Clostridia bacterium*	VanZ
OGH26881.1		VanZ
MBF6559028.1	*Candidatus Binataceae bacterium*	VanZ
HHS43677.1		VanZ
WP_153526532.1	*Brucella sp. 2280*	VanZ
CAI09507.1	*Aromatoleum aromaticum EbN1*	VanZ
MBL4688554.1	*Nannocystaceae bacterium*	VanZ
MBR2377574.1	*Clostridia bacterium*	VanZ
MBC8199056.1	*Candidatus Desulfaltia bathyphila*	VanZ
MBQ5926974.1	*Clostridia bacterium*	VanZ
MBO0753680.1	*Bradyrhizobiaceae bacterium*	VanZ
WP_076922886.1	*Pseudoalteromonas haloplanktis*	VanZ
NNF07188.1	*Candidatus Eiseniibacteriota bacterium*	VanZ
CDD85440.1	*Collinsella sp. CAG:289*	VanZ
HAQ37205.1	*Saprospirales bacterium*	VanZ
MBE6130202.1	*Erysipelotrichaceae bacterium*	VanZ
ODQ37732.1	*Mannheimia haemolytica*	VanZ
WP_068131955.1	*Roseimaritima ulvae*	VanZ
MBL7141986.1	*Patescibacteria group bacterium*	VanZ
MBD89253.1	*Deltaproteobacteria bacterium*	VanZ
KIR01335.1	*Lachnospiraceae bacterium TWA4*	VanZ
MBZ5493298.1	*Terriglobia bacterium*	VanZ
TXH54338.1	*Bacteroidia bacterium*	VanZ
OYU73870.1	*Alphaproteobacteria bacterium PA3*	VanZ
MBQ7377125.1	*Clostridia bacterium*	VanZ
MBQ82985.1	*Gammaproteobacteria bacterium*	VanZ
KRM94460.1	*Lentilactobacillus senioris DSM 24302 = JCM 17472*	VanZ
MBM3621133.1	*Alphaproteobacteria bacterium*	VanZ
PRY90303.1	*Donghicola tyrosinivorans*	VanZ
NOY84787.1	*Candidatus Manganitrophaceae bacterium*	VanZ
BCS53540.1	*Geobacter sp. SVR*	VanZ
PCI21420.1	*Piscirickettsiaceae bacterium*	VanZ
PZR41649.1	*Azospira oryzae*	VanZ
HHA48915.1		VanZ
WP_182941015.1	*Gluconacetobacter johannae DSM 13595*	VanZ
OIO71940.1	*Zetaproteobacteria bacterium CG1_02_49_23*	VanZ
WP_161822032.1	*Sporotomaculum syntrophicum*	VanZ
WP_104526103.1	*Blastococcus atacamensis*	VanZ
WP_169779192.1	*Campylobacter curvus*	VanZ
MBN8956504.1	*Hyphomicrobiales bacterium*	VanZ
WP_130254097.1	*Pseudoalteromonas phenolica*	VanZ
KRT77619.1	*Candidatus Sysuimicrobiota bacterium CSP1-3*	VanZ
MBA4692224.1	*SAR86 cluster bacterium*	VanZ
MBI1836928.1	*Flavobacteriia bacterium*	VanZ
MCC6776623.1	*Hyphomicrobiales bacterium*	VanZ
MBA4267615.1	*Comamonadaceae bacterium*	VanZ
WP_169767067.1	*Campylobacter curvus*	VanZ
NLV64659.1	*Synergistaceae bacterium*	VanZ
NQY35229.1	*Alteromonadaceae bacterium*	VanZ
HAT42794.1	*Rheinheimera sp.*	VanZ
ABX43645.1	*Lachnoclostridium phytofermentans ISDg*	VanZ
MBW7876214.1	*Candidatus Cloacimonadota bacterium*	VanZ
WP_130671738.1	*Rhizobium leguminosarum*	VanZ
HJF93448.1	*Lachnoclostridium phocaeense*	VanZ
MBF0439249.1	*Magnetococcales bacterium*	VanZ
MBX3596679.1	*Rhizobiaceae bacterium*	VanZ
MBF8274405.1	*Magnetococcales bacterium*	VanZ
WP_170179574.1	*Solirubrobacter pauli*	VanZ
MBT7451281.1	*Rhodospirillaceae bacterium*	VanZ
RMG92435.1	*Zetaproteobacteria bacterium*	VanZ
MBK0867230.1	*Saccharopolyspora galaxeae*	VanZ
WP_213339926.1	*Ancylobacter lacus*	VanZ
MAK14203.1	*Pseudomonadota bacterium*	VanZ
MBL8140242.1	*Acidobacteriota bacterium*	VanZ
MBP8228623.1	*Rheinheimera sp.*	VanZ
OYY93237.1	*Hydrogenophilales bacterium 28-61-23*	VanZ
MBQ4054808.1	*Clostridia bacterium*	VanZ
MBE7037844.1	*Oscillospiraceae bacterium*	VanZ
WP_144222667.1	*Mesorhizobium amorphae*	VanZ
MBA3896207.1	*Sphingomonadaceae bacterium*	VanZ
HHV43567.1	*Bacillota bacterium*	VanZ
MBN1471442.1	*Syntrophaceae bacterium*	VanZ
MBP7571147.1	*Acidobacteriota bacterium*	VanZ
WP_155263506.1	*Sphingomonas segetis*	VanZ
MBP5275160.1	*Abditibacteriota bacterium*	VanZ
MBD3246648.1	*Candidatus Omnitrophota bacterium*	VanZ
NBS12748.1	*Gammaproteobacteria bacterium*	VanZ
MAH12879.1	*Verrucomicrobiota bacterium*	VanZ
MBI4015143.1	*Candidatus Aenigmatarchaeota archaeon*	VanZ
WP_214736765.1	*Bacillus sp. ISL-41*	VanZ
WP_027312663.1	*Balneatrix alpica*	VanZ
WP_156839278.1	*Novosphingobium aquimarinum*	VanZ
PKQ29872.1	*Actinobacteria bacterium HGW-Actinobacteria-10*	VanZ
WP_067912838.1	*Novosphingobium fuchskuhlense*	VanZ
OGP18434.1	*Deltaproteobacteria bacterium GWA2_55_82*	VanZ
MBI9106135.1	*Spirochaetales bacterium*	VanZ

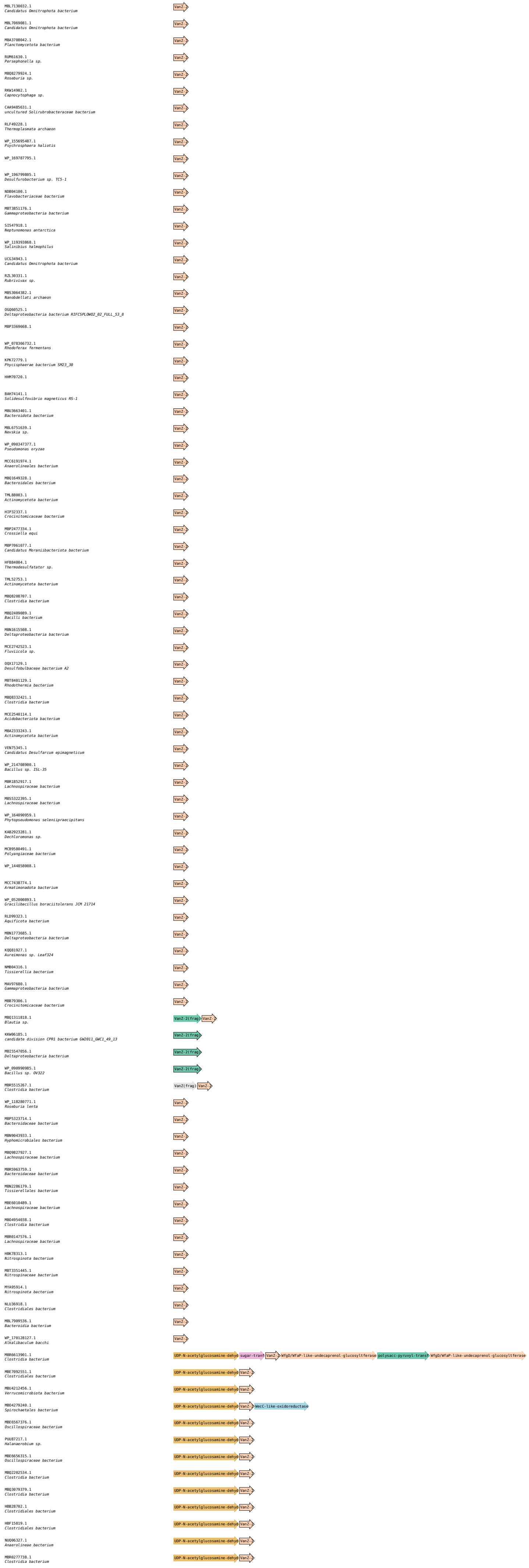

MBL7130032.1
Candidatus Omnitrophota bacterium
VanZ-2
MBL7069081.1
Candidatus Omnitrophota bacterium
VanZ-2
MBA3708042.1
Planctomycetota bacterium
VanZ-2
RUM61630.1
Persephonella sp.
VanZ-2
MBQ8279924.1
Roseburia sp.
VanZ-2
RKW14902.1
Capnocytophaga sp.
VanZ-2
CAA9485631.1
uncultured Solirubrobacteraceae bacterium
VanZ-2
RLF49228.1
Thermoplasmata archaeon
VanZ-2
WP_155695487.1
Psychrosphaera haliotis
VanZ-2
WP_169787795.1
VanZ-2
WP_196799805.1
Desulfurobacterium sp. TC5-1
VanZ-2
NDB04100.1
Flavobacteriaceae bacterium
VanZ-2
MBT3851176.1
Gammaproteobacteria bacterium
VanZ-2
SIS47918.1
Neptunomonas antarctica
VanZ-2
WP_119393868.1
Salinibius halmophilus
VanZ-2
UCG34943.1
Candidatus Omnitrophota bacterium
VanZ-2
RZL30331.1
Rubrivivax sp.
VanZ-2
MBS3064382.1
Nanobdellati archaeon
VanZ-2
OGQ60525.1
Deltaproteobacteria bacterium RIFCSPLOWO2_02_FULL_53_8
VanZ-2
MBP3369668.1
VanZ-2
WP_078366732.1
Rhodoferax fermentans
VanZ-2
KPK72779.1
Phycisphaerae bacterium SM23_30
VanZ-2
HHM70720.1
VanZ-2
BAH74141.1
Solidesulfovibrio magneticus RS-1
VanZ-2
MBU3663401.1
Bacteroidota bacterium
VanZ-2
MBL6751639.1
Nevskia sp.
VanZ-2
WP_090347377.1
Pseudomonas oryzae
VanZ-2
MCC6191974.1
Anaerolineales bacterium
VanZ-2
MBQ1649328.1
Bacteroidales bacterium
VanZ-2
TML88003.1
Actinomycetota bacterium
VanZ-2
HIP32337.1
Crocinitomicaceae bacterium
VanZ-2
MBP2477334.1
Crossiella equi
VanZ-2
MBP7061077.1
Candidatus Moraniibacteriota bacterium
VanZ-2
HFB84004.1
Thermodesulfatator sp.
VanZ-2
TML52753.1
Actinomycetota bacterium
VanZ-2
MBQ8208707.1
Clostridia bacterium
VanZ-2
MBQ2409089.1
Bacilli bacterium
VanZ-2
MBN1615508.1
Deltaproteobacteria bacterium
VanZ-2
MCE2742523.1
Fluviicola sp.
VanZ-2
OQX17129.1
Desulfobulbaceae bacterium A2
VanZ-2
MBT8401129.1
Rhodothermia bacterium
VanZ-2
MBQ8332421.1
Clostridia bacterium
VanZ-2
MCE2540114.1
Acidobacteriota bacterium
VanZ-2
MBA2333243.1
Actinomycetota bacterium
VanZ-2
VEN75345.1
Candidatus Desulfarcum epimagneticum
VanZ-2
WP_214708900.1
Bacillus sp. ISL-35
VanZ-2
MBR1852917.1
Lachnospiraceae bacterium
VanZ-2
MBS5322395.1
Lachnospiraceae bacterium
VanZ-2
WP_164090959.1
Phytopseudomonas seleniipraecipitans
VanZ-2
KAB2923281.1
Dechloromonas sp.
VanZ-2
MCB9580491.1
Polyangiaceae bacterium
VanZ-2
WP_144858008.1
VanZ-2
MCC7438774.1
Armatimonadota bacterium
VanZ-2
WP_052000893.1
Gracilibacillus boraciitolerans JCM 21714
VanZ-2
RLD99323.1
Aquificota bacterium
VanZ-2
MBN1773685.1
Deltaproteobacteria bacterium
VanZ-2
KQQ81927.1
Aureimonas sp. Leaf324
VanZ-2
NMB04316.1
Tissierellia bacterium
VanZ-2
MAV97680.1
Gammaproteobacteria bacterium
VanZ-2
MBB79306.1
Crocinitomicaceae bacterium
VanZ-2
MBQ1311818.1
Blautia sp.
VanZ-2(frag)
VanZ-2
KKW06185.1
candidate division CPR1 bacterium GW2011_GWC1_49_13
VanZ-2(frag)
MBI5547056.1
Deltaproteobacteria bacterium
VanZ-2(frag)
WP_090990985.1
Bacillus sp. OV322
VanZ-2(frag)
MBR5515267.1
Clostridia bacterium
VanZ(frag)
VanZ-2
WP_118280771.1
Roseburia lenta
VanZ-2
MBP5323714.1
Bacteroidaceae bacterium
VanZ-2
MBN9043933.1
Hyphomicrobiales bacterium
VanZ-2
MBQ9827927.1
Lachnospiraceae bacterium
VanZ-2
MBR5963759.1
Bacteroidaceae bacterium
VanZ-2
MBN2286179.1
Tissierellales bacterium
VanZ-2
MBE6010489.1
Lachnospiraceae bacterium
VanZ-2
MBO4954038.1
Clostridia bacterium
VanZ-2
MBR0147576.1
Lachnospiraceae bacterium
VanZ-2
HBK78313.1
Nitrospinota bacterium
VanZ-2
MBT3351445.1
Nitrospinaceae bacterium
VanZ-2
MYA95914.1
Nitrospinota bacterium
VanZ-2
NLU36918.1
Clostridiales bacterium
VanZ-2
MBL7909536.1
Bacteroidia bacterium
VanZ-2
WP_170128127.1
Alkalibaculum bacchi
VanZ-2
MBR6613901.1
Clostridia bacterium
UDP-N-acetylglucosamine-dehyd
sugar-tranf
VanZ-2
WfgD/WfaP-like-undecaprenol-glucosyltferase
polysacc-pyruvyl-transf
WfgD/WfaP-like-undecaprenol-glucosyltferase
MBE7092551.1
Clostridiales bacterium
UDP-N-acetylglucosamine-dehyd
VanZ-2
MBU4212456.1
Verrucomicrobiota bacterium
UDP-N-acetylglucosamine-dehyd
VanZ-2
MBO4279240.1
Spirochaetales bacterium
UDP-N-acetylglucosamine-dehyd
VanZ-2
WecC-like-oxidoreductase
MBE6567376.1
Oscillospiraceae bacterium
UDP-N-acetylglucosamine-dehyd
VanZ-2
PUU87217.1
Halanaerobium sp.
UDP-N-acetylglucosamine-dehyd
VanZ-2
MBE6656315.1
Oscillospiraceae bacterium
UDP-N-acetylglucosamine-dehyd
VanZ-2
MBQ2202534.1
Clostridia bacterium
UDP-N-acetylglucosamine-dehyd
VanZ-2
MBQ3079379.1
Clostridia bacterium
UDP-N-acetylglucosamine-dehyd
VanZ-2
HBB28702.1
Clostridiales bacterium
UDP-N-acetylglucosamine-dehyd
VanZ-2
HBF15819.1
Clostridiales bacterium
UDP-N-acetylglucosamine-dehyd
VanZ-2
NUQ06327.1
Anaerolineae bacterium
UDP-N-acetylglucosamine-dehyd
VanZ-2
MBR0277738.1
Clostridia bacterium
UDP-N-acetylglucosamine-dehyd
VanZ-2

Accession	Organism	Gene neighborhood
MBL8210455.1	*Bryobacterales bacterium*	UDP-N-acetylglucosamine-dehyd, VanZ-2
MBM4445723.1	*Chloroflexota bacterium*	UDP-N-acetylglucosamine-dehyd, VanZ-2
MBR6795791.1	*Clostridia bacterium*	UDP-N-acetylglucosamine-dehyd, VanZ-2
MBC8273881.1	*Chloroflexota bacterium*	UDP-N-acetylglucosamine-dehyd, VanZ-2
MBR6644208.1	*Clostridia bacterium*	UDP-N-acetylglucosamine-dehyd, VanZ-2
MBQ7600524.1	*Clostridia bacterium*	UDP-N-acetylglucosamine-dehyd, VanZ-2
MBR4500357.1	*Clostridia bacterium*	UDP-N-acetylglucosamine-dehyd, VanZ-2
MBR6535095.1	*Clostridia bacterium*	UDP-N-acetylglucosamine-dehyd, VanZ-2
OGO02805.1	*Chloroflexi bacterium RBG_13_53_26*	UDP-N-acetylglucosamine-dehyd, VanZ-2
MBQ9735730.1	*Clostridia bacterium*	UDP-N-acetylglucosamine-dehyd, VanZ-2
MBQ2253021.1	*Clostridia bacterium*	UDP-N-acetylglucosamine-dehyd, VanZ-2
MBU1020167.1	*Bacillota bacterium*	UDP-N-acetylglucosamine-dehyd, VanZ-2
NLH06188.1	*Chloroflexota bacterium*	UDP-N-acetylglucosamine-dehyd, VanZ-2
NLL70472.1	*Candidatus Epulonipiscium sp.*	UDP-N-acetylglucosamine-dehyd, VanZ-2
TXI66628.1	*Limnohabitans sp.*	UDP-N-acetylglucosamine-dehyd, VanZ-2
MBQ8909977.1	*Oscillospiraceae bacterium*	VanZ-2
MBQ8554806.1	*Clostridia bacterium*	VanZ-2
MBR0446790.1	*Oscillospiraceae bacterium*	VanZ-2
OQB14397.1	*Firmicutes bacterium ADurb.Bin193*	VanZ-2
MBI5068803.1	*Deltaproteobacteria bacterium*	GalE/RmlB/Gmd-like_lyase/epimerase, VanZ-2, WecC-like-oxidoreductase, GalE/RmlB/Gmd-like_lyase/epimerase, GDP-L-fucose-synthase
WP_091134045.1	*Microvirga guangxiensis*	sugar-transferase-GT-A, VanZ-2
NLK97154.1	*Candidatus Epulonipiscium sp.*	YceG-MltG-lytic-transglycosylase, LPS-like-sugar-transf, ?, VanZ-2
OGC47456.1	*candidate division WWE3 bacterium RIFCSPHIGHO2_01_FULL_42_13*	VanZ-2, ?, ?, GalE/RmlB/Gmd-like_lyase/epimerase, sugar-transferase-GT-A, GalE/RmlB/Gmd-like_lyase/epimerase
MBD5328081.1	*Bacteroides sp.*	VanZ-2
WP_232594693.1	*Marinibactrum halimedae*	YceG-MltG-lytic-transglycosylase, ?, VanZ-2
HEY84295.1	*Chloroflexota bacterium*	VanZ-2
MBM95198.1	*Oceanospirillaceae bacterium*	VanZ-2
MBQ2694262.1	*Clostridia bacterium*	VanZ-2
SFK48628.1	*Lachnospiraceae bacterium KH1T2*	VanZ-2
MBL9169539.1	*Verrucomicrobiales bacterium*	VanZ-2
MBD5315007.1	*Bacteroides sp.*	VanZ-2
MCB1094500.1	*Verrucomicrobiia bacterium*	VanZ-2
MBM4293927.1	*Deltaproteobacteria bacterium*	VanZ-2
MBL9143618.1	*Verrucomicrobiaceae bacterium*	VanZ-2
NLS78900.1	*Chloroflexota bacterium*	VanZ-2
MBO6116600.1	*Ruminococcus sp.*	VanZ-2
MBR5472968.1	*Clostridia bacterium*	VanZ-2
MBR3299941.1	*Clostridia bacterium*	VanZ-2
MBQ9354857.1	*Clostridia bacterium*	VanZ-2
MBO4734492.1	*Clostridia bacterium*	VanZ-2
MBC5987513.1		VanZ-2
MBR5152319.1	*Clostridia bacterium*	VanZ-2
MBI3353330.1	*Nitrospirota bacterium*	VanZ-2
RKZ17724.1	*bacterium*	VanZ-2
MBM4117495.1	*bacterium*	VanZ-2
UCF05632.1	*bacterium*	VanZ-2
MCB1161097.1	*bacterium*	VanZ-2
MBN1164543.1	*Candidatus Krumholzibacteriota bacterium*	VanZ-2
UCE03993.1	*Candidatus Latescibacterota bacterium*	VanZ-2
MBU8922164.1	*Candidatus Latescibacterota bacterium*	VanZ-2
MBN2185334.1	*Candidatus Krumholzibacteriota bacterium*	VanZ-2
OQX86369.1	*Candidatus Latescibacteria bacterium 4484_7*	VanZ-2
NIM18967.1	*Candidatus Latescibacterota bacterium*	VanZ-2
UCG52530.1	*Candidatus Latescibacterota bacterium*	VanZ-2
UCH82770.1	*Candidatus Latescibacterota bacterium*	VanZ-2
MBC8366552.1	*bacterium*	VanZ-2
MBN2170561.1	*Candidatus Krumholzibacteriota bacterium*	VanZ-2
OIO60993.1	*Proteobacteria bacterium CG1_02_64_396*	VanZ-2
WP_213410918.1	*Xylanibacillus composti*	VanZ-2
WP_089198330.1	*Paenibacillus xerothermodurans*	VanZ-2
WP_141333722.1	*Paenibacillus validus*	VanZ-2
WP_013917610.1	*Paenibacillus mucilaginosus*	VanZ-2
MBQ8593461.1	*Bacteroidaceae bacterium*	VanZ-2
MBP3237998.1	*Lachnospiraceae bacterium*	VanZ-2
MCC8072429.1	*Bacteroidales bacterium*	VanZ-2
NDF59517.1	*Crocinitomicaceae bacterium*	VanZ-2
NLF78311.1	*Chloroflexota bacterium*	VanZ-2
PHR56072.1	*Robiginitomaculum sp.*	VanZ-2
MBR0133308.1	*Lachnospiraceae bacterium*	VanZ-2
EGG81491.1	*Lachnospiraceae bacterium 2_1_46FAA*	VanZ-2
RGS82608.1	*Coprococcus sp. AF21-14LB*	VanZ-2
MBM4214868.1	*Gammaproteobacteria bacterium*	LolA-outer-membrane-chaperone, VanZ-2
MBH03492.1	*Lysobacterales bacterium*	LolA-outer-membrane-chaperone, VanZ-2
TDJ41708.1	*Gammaproteobacteria bacterium*	LolA-outer-membrane-chaperone, VanZ-2
NOX70934.1	*Gammaproteobacteria bacterium*	LolA-outer-membrane-chaperone, VanZ-2
MBT3992486.1	*Gammaproteobacteria bacterium*	LolA-outer-membrane-chaperone, VanZ-2
MAD91787.1	*Gammaproteobacteria bacterium*	LolA-outer-membrane-chaperone, VanZ-2
MBT7275880.1	*Woeseiaceae bacterium*	LolA-outer-membrane-chaperone, VanZ-2
TDJ43232.1		LolA-outer-membrane-chaperone, VanZ-2
TDJ12897.1	*Gammaproteobacteria bacterium*	LolA-outer-membrane-chaperone, VanZ-2
NBX43834.1	*Gammaproteobacteria bacterium*	LolA-outer-membrane-chaperone, VanZ-2
MCC7200882.1	*Gammaproteobacteria bacterium*	LolA-outer-membrane-chaperone, VanZ-2
NCU00745.1	*Candidatus Macondimonas diazotrophica*	LolA-outer-membrane-chaperone, VanZ-2
NCF72627.1	*Gammaproteobacteria bacterium*	LolA-outer-membrane-chaperone, VanZ-2
MBX5460251.1	*Steroidobacteraceae bacterium*	LolA-outer-membrane-chaperone, VanZ-2
MBV6417477.1	*Steroidobacteraceae bacterium*	LolA-outer-membrane-chaperone, VanZ-2
MBG60920.1	*Woeseiaceae bacterium*	LolA-outer-membrane-chaperone, VanZ-2
MBX3702810.1	*Steroidobacteraceae bacterium*	LolA-outer-membrane-chaperone, VanZ-2
KPK59875.1	*Gammaproteobacteria bacterium SG8_31*	LolA-outer-membrane-chaperone, VanZ-2
MBM5812703.1	*Gammaproteobacteria bacterium*	LolA-outer-membrane-chaperone, VanZ-2
MCC7259163.1	*Gammaproteobacteria bacterium*	LolA-outer-membrane-chaperone, VanZ-2
RZV37825.1	*Chromatiales bacterium*	LolA-outer-membrane-chaperone, VanZ-2
RZO85501.1	*Oceanococcus sp.*	LolA-outer-membrane-chaperone, VanZ-2

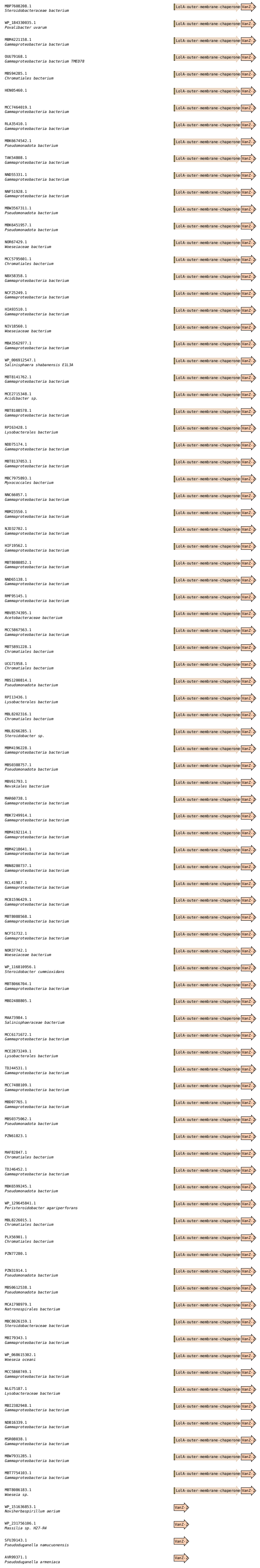

MBP7608208.1
Steroidobacteraceae bacterium
LolA-outer-membrane-chaperone
VanZ-2
WP_184330035.1
Povalibacter uvarum
LolA-outer-membrane-chaperone
VanZ-2
MBM4221158.1
Gammaproteobacteria bacterium
LolA-outer-membrane-chaperone
VanZ-2
OUU79168.1
Gammaproteobacteria bacterium TMED78
LolA-outer-membrane-chaperone
VanZ-2
MBS94285.1
Chromatiales bacterium
LolA-outer-membrane-chaperone
VanZ-2
HEN05460.1
LolA-outer-membrane-chaperone
VanZ-2
MCC7464019.1
Gammaproteobacteria bacterium
LolA-outer-membrane-chaperone
VanZ-2
RLA35410.1
Gammaproteobacteria bacterium
LolA-outer-membrane-chaperone
VanZ-2
MBK6674542.1
Pseudomonadota bacterium
LolA-outer-membrane-chaperone
VanZ-2
TAK54808.1
Gammaproteobacteria bacterium
LolA-outer-membrane-chaperone
VanZ-2
NND55331.1
Gammaproteobacteria bacterium
LolA-outer-membrane-chaperone
VanZ-2
NNF51928.1
Gammaproteobacteria bacterium
LolA-outer-membrane-chaperone
VanZ-2
MBW3567311.1
Pseudomonadota bacterium
LolA-outer-membrane-chaperone
VanZ-2
MBK6451957.1
Pseudomonadota bacterium
LolA-outer-membrane-chaperone
VanZ-2
NOR67429.1
Woeseiaceae bacterium
LolA-outer-membrane-chaperone
VanZ-2
MCC5795601.1
Chromatiales bacterium
LolA-outer-membrane-chaperone
VanZ-2
NBX58358.1
Gammaproteobacteria bacterium
LolA-outer-membrane-chaperone
VanZ-2
NCF25249.1
Gammaproteobacteria bacterium
LolA-outer-membrane-chaperone
VanZ-2
HIA93510.1
Gammaproteobacteria bacterium
LolA-outer-membrane-chaperone
VanZ-2
NIV18560.1
Woeseiaceae bacterium
LolA-outer-membrane-chaperone
VanZ-2
MBA3562977.1
Gammaproteobacteria bacterium
LolA-outer-membrane-chaperone
VanZ-2
WP_006912547.1
Salinisphaera shabanensis E1L3A
LolA-outer-membrane-chaperone
VanZ-2
MBT8141762.1
Gammaproteobacteria bacterium
LolA-outer-membrane-chaperone
VanZ-2
MCE2715348.1
Acidibacter sp.
LolA-outer-membrane-chaperone
VanZ-2
MBT8108578.1
Gammaproteobacteria bacterium
LolA-outer-membrane-chaperone
VanZ-2
RPI63428.1
Lysobacterales bacterium
LolA-outer-membrane-chaperone
VanZ-2
NDD75174.1
Gammaproteobacteria bacterium
LolA-outer-membrane-chaperone
VanZ-2
MBT8137053.1
Gammaproteobacteria bacterium
LolA-outer-membrane-chaperone
VanZ-2
MBC7975893.1
Myxococcales bacterium
LolA-outer-membrane-chaperone
VanZ-2
NNC66057.1
Gammaproteobacteria bacterium
LolA-outer-membrane-chaperone
VanZ-2
MBM23550.1
Gammaproteobacteria bacterium
LolA-outer-membrane-chaperone
VanZ-2
NJD32702.1
Gammaproteobacteria bacterium
LolA-outer-membrane-chaperone
VanZ-2
HIF19562.1
Gammaproteobacteria bacterium
LolA-outer-membrane-chaperone
VanZ-2
MBT8080852.1
Gammaproteobacteria bacterium
LolA-outer-membrane-chaperone
VanZ-2
NND65138.1
Gammaproteobacteria bacterium
LolA-outer-membrane-chaperone
VanZ-2
RMF95145.1
Gammaproteobacteria bacterium
LolA-outer-membrane-chaperone
VanZ-2
MBV8574395.1
Acetobacteraceae bacterium
LolA-outer-membrane-chaperone
VanZ-2
MCC5867563.1
Gammaproteobacteria bacterium
LolA-outer-membrane-chaperone
VanZ-2
MBT5891228.1
Chromatiales bacterium
LolA-outer-membrane-chaperone
VanZ-2
UCG71958.1
Chromatiales bacterium
LolA-outer-membrane-chaperone
VanZ-2
MBS1200814.1
Pseudomonadota bacterium
LolA-outer-membrane-chaperone
VanZ-2
RPI13436.1
Lysobacterales bacterium
LolA-outer-membrane-chaperone
VanZ-2
MBL8202316.1
Chromatiales bacterium
LolA-outer-membrane-chaperone
VanZ-2
MBL8266285.1
Steroidobacter sp.
LolA-outer-membrane-chaperone
VanZ-2
MBM4196228.1
Gammaproteobacteria bacterium
LolA-outer-membrane-chaperone
VanZ-2
MBS0388757.1
Pseudomonadota bacterium
LolA-outer-membrane-chaperone
VanZ-2
MBV61793.1
Nevskiales bacterium
LolA-outer-membrane-chaperone
VanZ-2
MAR60738.1
Gammaproteobacteria bacterium
LolA-outer-membrane-chaperone
VanZ-2
MBK7249914.1
Gammaproteobacteria bacterium
LolA-outer-membrane-chaperone
VanZ-2
MBM4192114.1
Gammaproteobacteria bacterium
LolA-outer-membrane-chaperone
VanZ-2
MBM4218641.1
Gammaproteobacteria bacterium
LolA-outer-membrane-chaperone
VanZ-2
MBN8280737.1
Gammaproteobacteria bacterium
LolA-outer-membrane-chaperone
VanZ-2
RCL41987.1
Gammaproteobacteria bacterium
LolA-outer-membrane-chaperone
VanZ-2
MCB1596429.1
Gammaproteobacteria bacterium
LolA-outer-membrane-chaperone
VanZ-2
MBT8088568.1
Gammaproteobacteria bacterium
LolA-outer-membrane-chaperone
VanZ-2
NCF51732.1
Gammaproteobacteria bacterium
LolA-outer-membrane-chaperone
VanZ-2
NOR37742.1
Woeseiaceae bacterium
LolA-outer-membrane-chaperone
VanZ-2
WP_116810956.1
Steroidobacter cummioxidans
LolA-outer-membrane-chaperone
VanZ-2
MBT8066704.1
Gammaproteobacteria bacterium
LolA-outer-membrane-chaperone
VanZ-2
MBO2488805.1
LolA-outer-membrane-chaperone
VanZ-2
MAA73984.1
Salinisphaeraceae bacterium
LolA-outer-membrane-chaperone
VanZ-2
MCC6171672.1
Gammaproteobacteria bacterium
LolA-outer-membrane-chaperone
VanZ-2
MCE2873249.1
Lysobacterales bacterium
LolA-outer-membrane-chaperone
VanZ-2
TDJ44531.1
Gammaproteobacteria bacterium
LolA-outer-membrane-chaperone
VanZ-2
MCC7488109.1
Gammaproteobacteria bacterium
LolA-outer-membrane-chaperone
VanZ-2
MBD07765.1
Gammaproteobacteria bacterium
LolA-outer-membrane-chaperone
VanZ-2
MBS0375062.1
Pseudomonadota bacterium
LolA-outer-membrane-chaperone
VanZ-2
PZN61823.1
LolA-outer-membrane-chaperone
VanZ-2
MAF82847.1
Chromatiales bacterium
LolA-outer-membrane-chaperone
VanZ-2
TDJ46452.1
Gammaproteobacteria bacterium
LolA-outer-membrane-chaperone
VanZ-2
MBK6599245.1
Pseudomonadota bacterium
LolA-outer-membrane-chaperone
VanZ-2
WP_129645841.1
Peristeroidobacter agariperforans
LolA-outer-membrane-chaperone
VanZ-2
MBL8226015.1
Chromatiales bacterium
LolA-outer-membrane-chaperone
VanZ-2
PLX56901.1
Chromatiales bacterium
LolA-outer-membrane-chaperone
VanZ-2
PZN77280.1
LolA-outer-membrane-chaperone
VanZ-2
PZN31914.1
Pseudomonadota bacterium
LolA-outer-membrane-chaperone
VanZ-2
MBS0612538.1
Pseudomonadota bacterium
LolA-outer-membrane-chaperone
VanZ-2
MCA1798979.1
Natronospirales bacterium
LolA-outer-membrane-chaperone
VanZ-2
MBC8026159.1
Steroidobacteraceae bacterium
LolA-outer-membrane-chaperone
VanZ-2
MBI79343.1
Gammaproteobacteria bacterium
LolA-outer-membrane-chaperone
VanZ-2
WP_068615302.1
Woeseia oceani
LolA-outer-membrane-chaperone
VanZ-2
MCC5860749.1
Gammaproteobacteria bacterium
LolA-outer-membrane-chaperone
VanZ-2
NLG75187.1
Lysobacteraceae bacterium
LolA-outer-membrane-chaperone
VanZ-2
MBI2382948.1
Gammaproteobacteria bacterium
LolA-outer-membrane-chaperone
VanZ-2
NDB16339.1
Gammaproteobacteria bacterium
LolA-outer-membrane-chaperone
VanZ-2
MSR08038.1
Gammaproteobacteria bacterium
LolA-outer-membrane-chaperone
VanZ-2
MBW7931285.1
Gammaproteobacteria bacterium
LolA-outer-membrane-chaperone
VanZ-2
MBT7754103.1
Gammaproteobacteria bacterium
LolA-outer-membrane-chaperone
VanZ-2
MBT8086183.1
Woeseia sp.
LolA-outer-membrane-chaperone
VanZ-2
WP_151636853.1
Noviherbaspirillum aerium
VanZ-2
WP_231756106.1
Massilia sp. H27-R4
VanZ-2
SFU39143.1
Pseudoduganella namucuonensis
VanZ-2
AVR99371.1
Pseudoduganella armeniaca
VanZ-2

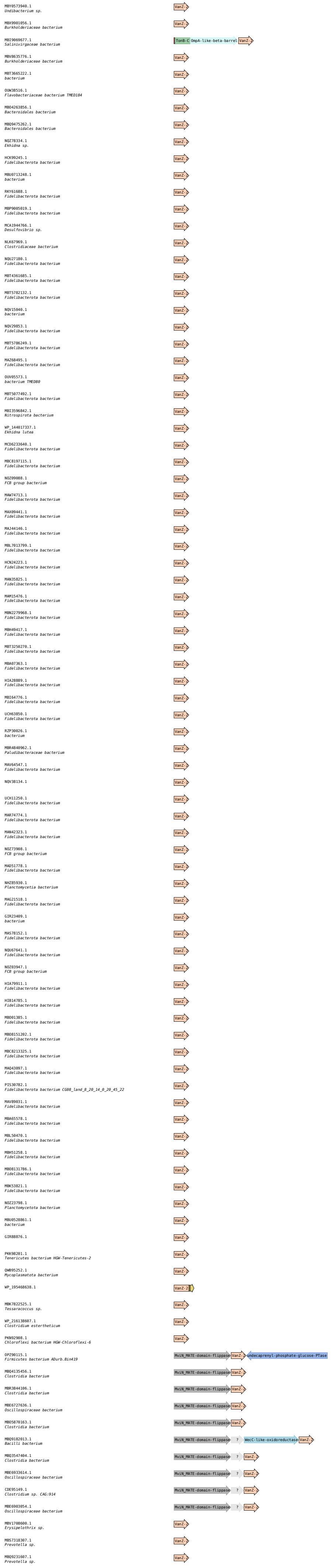
MBY0573940.1
Undibacterium sp.
VanZ-2
MBX9901056.1
Burkholderiaceae bacterium
VanZ-2
MBI9069677.1
Salinivirgaceae bacterium
TonB-C
OmpA-like-beta-barrel
VanZ-2
MBV8635776.1
Burkholderiaceae bacterium
VanZ-2
MBT3665222.1
bacterium
VanZ-2
OUW38516.1
Flavobacteriaceae bacterium TMED184
VanZ-2
MBO4263856.1
Bacteroidales bacterium
VanZ-2
MBQ9475262.1
Bacteroidales bacterium
VanZ-2
NQZ78334.1
Ekhidna sp.
VanZ-2
HCK99245.1
Fidelibacterota bacterium
VanZ-2
MBU0713248.1
bacterium
VanZ-2
RKY61688.1
Fidelibacterota bacterium
VanZ-2
MBP9005019.1
Fidelibacterota bacterium
VanZ-2
MCA1944766.1
Desulfovibrio sp.
VanZ-2
NLK67969.1
Clostridiaceae bacterium
VanZ-2
NQU27180.1
Fidelibacterota bacterium
VanZ-2
MBT4361685.1
Fidelibacterota bacterium
VanZ-2
MBT5782132.1
Fidelibacterota bacterium
VanZ-2
NQV15040.1
bacterium
VanZ-2
NQV29853.1
Fidelibacterota bacterium
VanZ-2
MBT5786249.1
Fidelibacterota bacterium
VanZ-2
MAZ68495.1
Fidelibacterota bacterium
VanZ-2
OUV05573.1
bacterium TMED80
VanZ-2
MBT5077492.1
Fidelibacterota bacterium
VanZ-2
MBI3596842.1
Nitrospirota bacterium
VanZ-2
WP_144017337.1
Ekhidna lutea
VanZ-2
MCD6233640.1
Fidelibacterota bacterium
VanZ-2
MBC8197115.1
Fidelibacterota bacterium
VanZ-2
NOZ09088.1
FCB group bacterium
VanZ-2
MAW74713.1
Fidelibacterota bacterium
VanZ-2
MAX09441.1
Fidelibacterota bacterium
VanZ-2
MAJ44146.1
Fidelibacterota bacterium
VanZ-2
MBL7013799.1
Fidelibacterota bacterium
VanZ-2
HCN24223.1
Fidelibacterota bacterium
VanZ-2
MAN35825.1
Fidelibacterota bacterium
VanZ-2
MAM15476.1
Fidelibacterota bacterium
VanZ-2
MBN2279968.1
Fidelibacterota bacterium
VanZ-2
MBH49417.1
Fidelibacterota bacterium
VanZ-2
MBT3250270.1
Fidelibacterota bacterium
VanZ-2
MBA07363.1
Fidelibacterota bacterium
VanZ-2
HIA28889.1
Fidelibacterota bacterium
VanZ-2
MBI64776.1
Fidelibacterota bacterium
VanZ-2
UCH63850.1
Fidelibacterota bacterium
VanZ-2
RZP30026.1
bacterium
VanZ-2
MBR4840962.1
Paludibacteraceae bacterium
VanZ-2
MAV64547.1
Fidelibacterota bacterium
VanZ-2
NQV38134.1
VanZ-2
UCH11250.1
Fidelibacterota bacterium
VanZ-2
MAR74774.1
Fidelibacterota bacterium
VanZ-2
MAN42323.1
Fidelibacterota bacterium
VanZ-2
NOZ73908.1
FCB group bacterium
VanZ-2
MAD51778.1
Fidelibacterota bacterium
VanZ-2
NHZ85930.1
Planctomycetia bacterium
VanZ-2
MAG21518.1
Fidelibacterota bacterium
VanZ-2
GIR23409.1
bacterium
VanZ-2
MAS78152.1
Fidelibacterota bacterium
VanZ-2
NQU67641.1
Fidelibacterota bacterium
VanZ-2
NOZ03947.1
FCB group bacterium
VanZ-2
HIA79911.1
Fidelibacterota bacterium
VanZ-2
HIB14785.1
Fidelibacterota bacterium
VanZ-2
MBO01385.1
Fidelibacterota bacterium
VanZ-2
MBO8151202.1
Fidelibacterota bacterium
VanZ-2
MBC8213325.1
Fidelibacterota bacterium
VanZ-2
MAQ43097.1
Fidelibacterota bacterium
VanZ-2
PIS30782.1
Fidelibacterota bacterium CG08_land_8_20_14_0_20_45_22
VanZ-2
MAV89031.1
Fidelibacterota bacterium
VanZ-2
MBA65578.1
Fidelibacterota bacterium
VanZ-2
MBL50470.1
Fidelibacterota bacterium
VanZ-2
MBH51258.1
Fidelibacterota bacterium
VanZ-2
MBO8131786.1
Fidelibacterota bacterium
VanZ-2
MBK53821.1
Fidelibacterota bacterium
VanZ-2
NOZ23798.1
Planctomycetota bacterium
VanZ-2
MBU0528861.1
bacterium
VanZ-2
GIR88876.1
VanZ-2
PKK98201.1
Tenericutes bacterium HGW-Tenericutes-2
VanZ-2
QWB95252.1
Mycoplasmatota bacterium
VanZ-2
WP_195468638.1
VanZ-2
MBK7822525.1
Tessaracoccus sp.
VanZ-2
WP_216138607.1
Clostridium estertheticum
VanZ-2
PKN92908.1
Chloroflexi bacterium HGW-Chloroflexi-6
VanZ-2
OPZ90115.1
Firmicutes bacterium ADurb.Bin419
MviN_MATE-domain-flippase
VanZ-2
undecaprenyl-phosphate-glucose-PTase
MBQ4135456.1
Clostridia bacterium
MviN_MATE-domain-flippase
VanZ-2
MBR3844106.1
Clostridia bacterium
MviN_MATE-domain-flippase
VanZ-2
MBE6727636.1
Oscillospiraceae bacterium
MviN_MATE-domain-flippase
VanZ-2
MBO5870163.1
Clostridia bacterium
MviN_MATE-domain-flippase
VanZ-2
MBQ9182013.1
Bacilli bacterium
MviN_MATE-domain-flippase
?
WecC-like-oxidoreductase
VanZ-2
MBQ3547404.1
Clostridia bacterium
MviN_MATE-domain-flippase
?
VanZ-2
MBE6933614.1
Oscillospiraceae bacterium
MviN_MATE-domain-flippase
?
VanZ-2
CDE95149.1
Clostridium sp. CAG:914
MviN_MATE-domain-flippase
?
VanZ-2
MBE6903054.1
Oscillospiraceae bacterium
MviN_MATE-domain-flippase
?
VanZ-2
MBV1708600.1
Erysipelothrix sp.
VanZ-2
MBS7318307.1
Prevotella sp.
VanZ-2
MBQ9231607.1
Prevotella sp.
VanZ-2

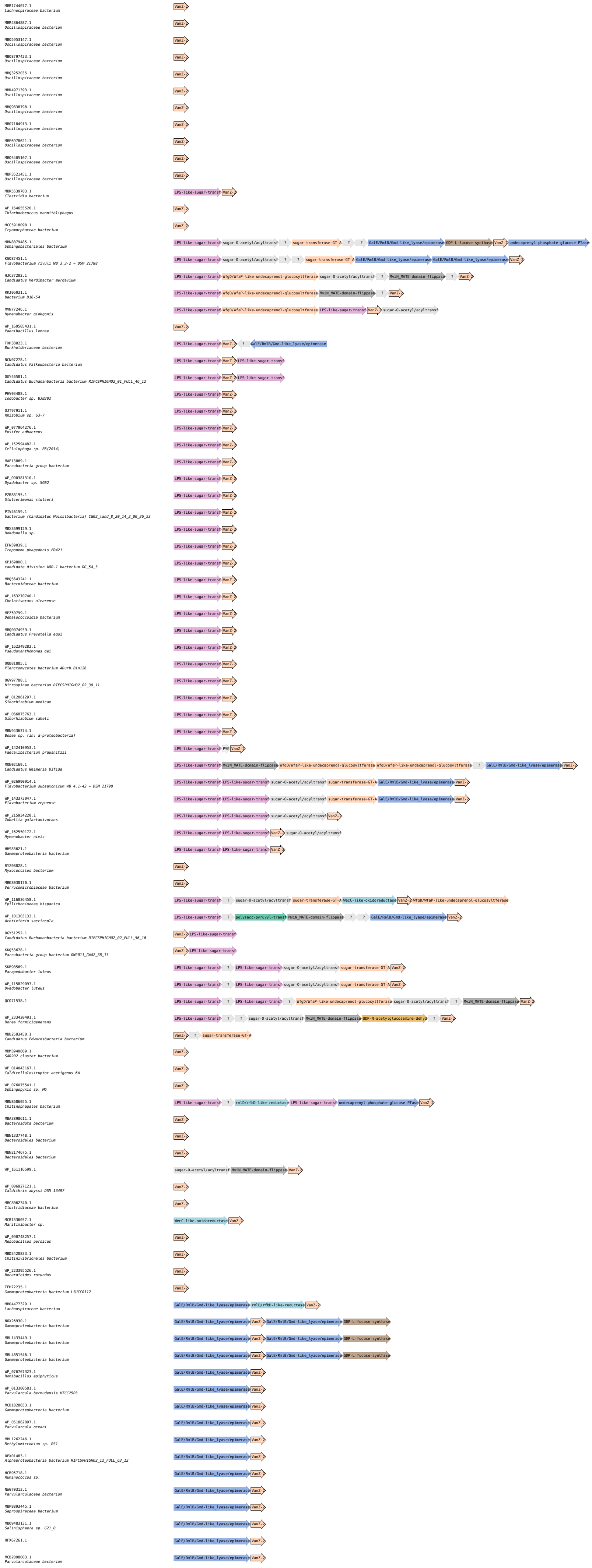
MBR1744077.1
Lachnospiraceae bacterium
VanZ-2
MBR4864887.1
Oscillospiraceae bacterium
VanZ-2
MBO5953147.1
Oscillospiraceae bacterium
VanZ-2
MBQ8797423.1
Oscillospiraceae bacterium
VanZ-2
MBQ3252035.1
Oscillospiraceae bacterium
VanZ-2
MBR4971393.1
Oscillospiraceae bacterium
VanZ-2
MBQ9838798.1
Oscillospiraceae bacterium
VanZ-2
MBO7184913.1
Oscillospiraceae bacterium
VanZ-2
MBE6978621.1
Oscillospiraceae bacterium
VanZ-2
MBQ5405107.1
Oscillospiraceae bacterium
VanZ-2
MBP3521451.1
Oscillospiraceae bacterium
VanZ-2
MBR5539703.1
Clostridia bacterium
LPS-like-sugar-transf VanZ-2
WP_164655520.1
Thiorhodococcus mannitoliphagus
VanZ-2
MCC5918098.1
Cryomorphaceae bacterium
VanZ-2
MBN8879485.1
Sphingobacteriales bacterium
LPS-like-sugar-transf sugar-O-acetyl/acyltransf ? sugar-transferase-GT-A ? ? GalE/RmlB/Gmd-like_lyase/epimerase GDP-L-fucose-synthase VanZ-2 undecaprenyl-phosphate-glucose-PTase
KGO87451.1
Flavobacterium rivuli WB 3.3-2 = DSM 21788
LPS-like-sugar-transf sugar-O-acetyl/acyltransf ? ? sugar-transferase-GT-A GalE/RmlB/Gmd-like_lyase/epimerase GalE/RmlB/Gmd-like_lyase/epimerase VanZ-2
HJC37202.1
Candidatus Merdibacter merdavium
LPS-like-sugar-transf WfgD/WfaP-like-undecaprenol-glucosyltferase sugar-O-acetyl/acyltransf ? MviN_MATE-domain-flippase ? VanZ-2
RKJ06031.1
bacterium D16-54
LPS-like-sugar-transf WfgD/WfaP-like-undecaprenol-glucosyltferase MviN_MATE-domain-flippase ? VanZ-2
MVN77246.1
Hymenobacter ginkgonis
LPS-like-sugar-transf WfgD/WfaP-like-undecaprenol-glucosyltferase LPS-like-sugar-transf VanZ-2 sugar-O-acetyl/acyltransf
WP_169505431.1
Paenibacillus lemnae
VanZ-2
TXH38023.1
Burkholderiaceae bacterium
LPS-like-sugar-transf VanZ-2 ? GalE/RmlB/Gmd-like_lyase/epimerase
NCN07278.1
Candidatus Falkowbacteria bacterium
LPS-like-sugar-transf VanZ-2 LPS-like-sugar-transf
OGY46581.1
Candidatus Buchananbacteria bacterium RIFCSPHIGHO2_01_FULL_46_12
LPS-like-sugar-transf VanZ-2 LPS-like-sugar-transf
PHV03488.1
Iodobacter sp. BJB302
LPS-like-sugar-transf VanZ-2
OJT97911.1
Rhizobium sp. 63-7
LPS-like-sugar-transf VanZ-2
WP_077964276.1
Ensifer adhaerens
LPS-like-sugar-transf VanZ-2
WP_152594482.1
Cellulophaga sp. E6(2014)
LPS-like-sugar-transf VanZ-2
MAF13869.1
Parcubacteria group bacterium
LPS-like-sugar-transf VanZ-2
WP_090381310.1
Dyadobacter sp. SG02
LPS-like-sugar-transf VanZ-2
PZR88195.1
Stutzerimonas stutzeri
LPS-like-sugar-transf VanZ-2
PIV46159.1
bacterium (Candidatus Moisslbacteria) CG02_land_8_20_14_3_00_36_53
LPS-like-sugar-transf VanZ-2
MBX3699129.1
Dokdonella sp.
LPS-like-sugar-transf VanZ-2
EFW39039.1
Treponema phagedenis F0421
LPS-like-sugar-transf VanZ-2
KPJ69800.1
candidate division WOR-1 bacterium DG_54_3
LPS-like-sugar-transf VanZ-2
MBQ5643241.1
Bacteroidaceae bacterium
LPS-like-sugar-transf VanZ-2
WP_163270740.1
Chelativorans alearense
LPS-like-sugar-transf VanZ-2
MPZ50799.1
Dehalococcoidia bacterium
LPS-like-sugar-transf VanZ-2
MBQ0074939.1
Candidatus Prevotella equi
LPS-like-sugar-transf VanZ-2
WP_162349282.1
Pseudoxanthomonas gei
LPS-like-sugar-transf VanZ-2
OQB81885.1
Planctomycetes bacterium ADurb.Bin126
LPS-like-sugar-transf VanZ-2
OGV97788.1
Nitrospinae bacterium RIFCSPHIGHO2_02_39_11
LPS-like-sugar-transf VanZ-2
WP_012061297.1
Sinorhizobium medicae
LPS-like-sugar-transf VanZ-2
WP_066875763.1
Sinorhizobium saheli
LPS-like-sugar-transf VanZ-2
MBN9436374.1
Bosea sp. (in: a-proteobacteria)
LPS-like-sugar-transf VanZ-2
WP_142410953.1
Faecalibacterium prausnitzii
LPS-like-sugar-transf PSE VanZ-2
MQN02169.1
Candidatus Weimeria bifida
LPS-like-sugar-transf MviN_MATE-domain-flippase WfgD/WfaP-like-undecaprenol-glucosyltferase WfgD/WfaP-like-undecaprenol-glucosyltferase ? GalE/RmlB/Gmd-like_lyase/epimerase VanZ-2
WP_026990914.1
Flavobacterium subsaxonicum WB 4.1-42 = DSM 21790
LPS-like-sugar-transf LPS-like-sugar-transf sugar-O-acetyl/acyltransf sugar-transferase-GT-A GalE/RmlB/Gmd-like_lyase/epimerase VanZ-2
WP_143373047.1
Flavobacterium zepuense
LPS-like-sugar-transf LPS-like-sugar-transf sugar-O-acetyl/acyltransf sugar-transferase-GT-A GalE/RmlB/Gmd-like_lyase/epimerase VanZ-2
WP_215934220.1
Zobellia galactanivorans
LPS-like-sugar-transf LPS-like-sugar-transf sugar-O-acetyl/acyltransf VanZ-2
WP_162550172.1
Hymenobacter nivis
LPS-like-sugar-transf LPS-like-sugar-transf VanZ-2 sugar-O-acetyl/acyltransf
HHS83621.1
Gammaproteobacteria bacterium
LPS-like-sugar-transf LPS-like-sugar-transf VanZ-2
RYZ08828.1
Myxococcales bacterium
VanZ-2
MBK8038170.1
Verrucomicrobiaceae bacterium
VanZ-2
WP_116036458.1
Epilithonimonas hispanica
LPS-like-sugar-transf ? sugar-O-acetyl/acyltransf sugar-transferase-GT-A WecC-like-oxidoreductase VanZ-2 WfgD/WfaP-like-undecaprenol-glucosyltferase
WP_101303133.1
Acetivibrio saccincola
LPS-like-sugar-transf ? polysacc-pyruvyl-transf MviN_MATE-domain-flippase ? ? GalE/RmlB/Gmd-like_lyase/epimerase VanZ-2
OGY51252.1
Candidatus Buchananbacteria bacterium RIFCSPHIGHO2_02_FULL_56_16
VanZ-2 LPS-like-sugar-transf
KKQ53678.1
Parcubacteria group bacterium GW2011_GWA2_38_13
VanZ-2 LPS-like-sugar-transf
SKB98569.1
Parapedobacter luteus
LPS-like-sugar-transf ? LPS-like-sugar-transf sugar-O-acetyl/acyltransf sugar-transferase-GT-A VanZ-2
WP_115829097.1
Dyadobacter luteus
LPS-like-sugar-transf ? LPS-like-sugar-transf sugar-O-acetyl/acyltransf sugar-transferase-GT-A VanZ-2
QCO71538.1
LPS-like-sugar-transf ? LPS-like-sugar-transf ? WfgD/WfaP-like-undecaprenol-glucosyltferase sugar-O-acetyl/acyltransf ? MviN_MATE-domain-flippase VanZ-2
WP_233420491.1
Dorea formicigenerans
LPS-like-sugar-transf ? ? sugar-O-acetyl/acyltransf MviN_MATE-domain-flippase UDP-N-acetylglucosamine-dehyd ? VanZ-2
MBU2593450.1
Candidatus Edwardsbacteria bacterium
VanZ-2 ? sugar-transferase-GT-A
MBM3940889.1
SAR202 cluster bacterium
VanZ-2
WP_014043167.1
Caldicellulosiruptor acetigenus 6A
VanZ-2
WP_076075541.1
Sphingopyxis sp. MG
VanZ-2
MBN8686055.1
Chitinophagales bacterium
LPS-like-sugar-transf ? rmlD/rfbD-like-reductase LPS-like-sugar-transf undecaprenyl-phosphate-glucose-PTase VanZ-2
MBA3898611.1
Bacteroidota bacterium
VanZ-2
MBN1337748.1
Bacteroidales bacterium
VanZ-2
MBN2174675.1
Bacteroidales bacterium
VanZ-2
WP_161116599.1
sugar-O-acetyl/acyltransf MviN_MATE-domain-flippase VanZ-2
WP_006927121.1
Caldithrix abyssi DSM 13497
VanZ-2
MBC8062340.1
Clostridiaceae bacterium
VanZ-2
MCB1336057.1
Maritimibacter sp.
WecC-like-oxidoreductase VanZ-2
WP_090748257.1
Mesobacillus persicus
VanZ-2
MBD3420833.1
Chitinivibrionales bacterium
VanZ-2
WP_223395526.1
Nocardioides rotundus
VanZ-2
TFH72235.1
Gammaproteobacteria bacterium LSUCC0112
VanZ-2
MBO4477329.1
Lachnospiraceae bacterium
GalE/RmlB/Gmd-like_lyase/epimerase rmlD/rfbD-like-reductase VanZ-2
NOX26930.1
Gammaproteobacteria bacterium
GalE/RmlB/Gmd-like_lyase/epimerase VanZ-2 GalE/RmlB/Gmd-like_lyase/epimerase GDP-L-fucose-synthase
MBL1433449.1
Gammaproteobacteria bacterium
GalE/RmlB/Gmd-like_lyase/epimerase VanZ-2 GalE/RmlB/Gmd-like_lyase/epimerase GDP-L-fucose-synthase
MBL4851546.1
Gammaproteobacteria bacterium
GalE/RmlB/Gmd-like_lyase/epimerase VanZ-2 GalE/RmlB/Gmd-like_lyase/epimerase GDP-L-fucose-synthase
WP_076767323.1
Domibacillus epiphyticus
GalE/RmlB/Gmd-like_lyase/epimerase VanZ-2
WP_013300581.1
Parvularcula bermudensis HTCC2503
GalE/RmlB/Gmd-like_lyase/epimerase VanZ-2
MCB1828653.1
Gammaproteobacteria bacterium
GalE/RmlB/Gmd-like_lyase/epimerase VanZ-2
WP_051882097.1
Parvularcula oceani
GalE/RmlB/Gmd-like_lyase/epimerase VanZ-2
MBL1262246.1
Methylomicrobium sp. RS1
GalE/RmlB/Gmd-like_lyase/epimerase VanZ-2
OFX01483.1
Alphaproteobacteria bacterium RIFCSPHIGHO2_12_FULL_63_12
GalE/RmlB/Gmd-like_lyase/epimerase VanZ-2
HCB95718.1
Ruminococcus sp.
GalE/RmlB/Gmd-like_lyase/epimerase VanZ-2
NWG70313.1
Parvularculaceae bacterium
GalE/RmlB/Gmd-like_lyase/epimerase VanZ-2
MBP8893445.1
Saprospiraceae bacterium
GalE/RmlB/Gmd-like_lyase/epimerase VanZ-2
MBO9483131.1
Salinisphaera sp. G21_0
GalE/RmlB/Gmd-like_lyase/epimerase VanZ-2
HFX67261.1
GalE/RmlB/Gmd-like_lyase/epimerase VanZ-2
MCB2098003.1
Parvularculaceae bacterium
GalE/RmlB/Gmd-like_lyase/epimerase VanZ-2

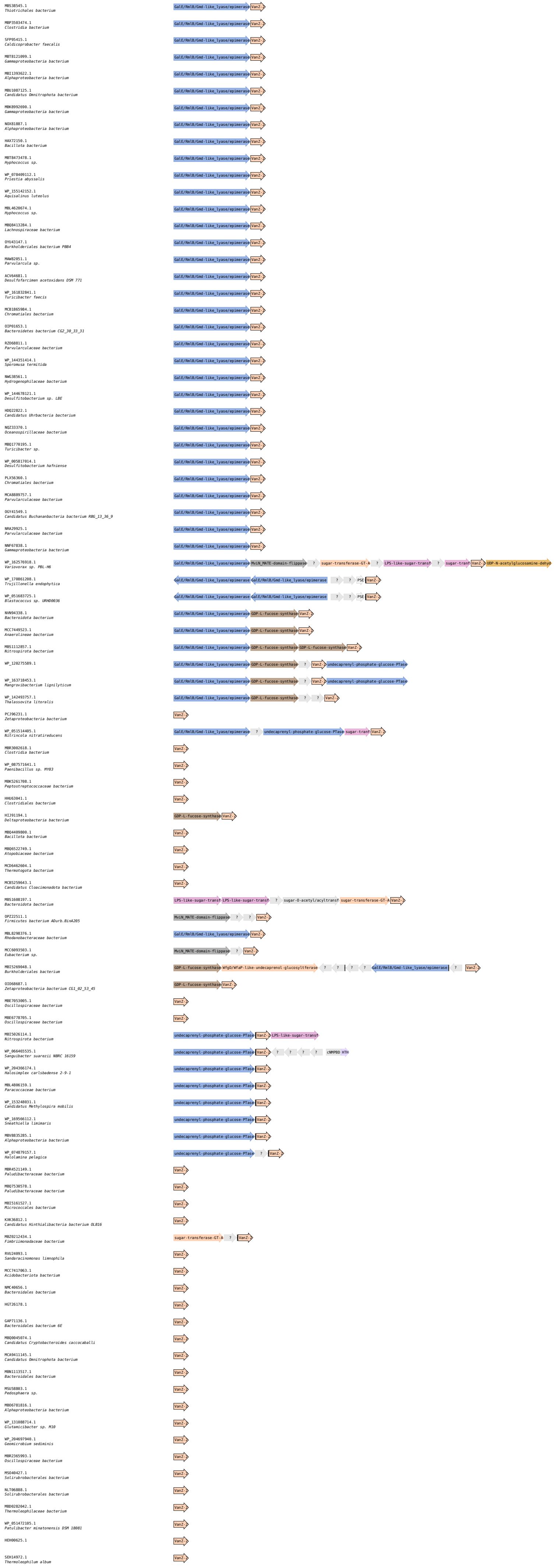

MBS38545.1
Thiotrichales bacterium
MBP3503474.1
Clostridia bacterium
SFP95415.1
Caldicoprobacter faecalis
MBT8121099.1
Gammaproteobacteria bacterium
MBI1393622.1
Alphaproteobacteria bacterium
MBU1087125.1
Candidatus Omnitrophota bacterium
MBK8992690.1
Gammaproteobacteria bacterium
NOX81887.1
Alphaproteobacteria bacterium
HAX72150.1
Bacillota bacterium
MBT8473478.1
Hyphococcus sp.
WP_078409112.1
Priestia abyssalis
WP_155142152.1
Aquisalinus luteolus
MBL4620674.1
Hyphococcus sp.
MBQ8413284.1
Lachnospiraceae bacterium
OYU43147.1
Burkholderiales bacterium PBB4
MAW82051.1
Parvularcula sp.
ACV64681.1
Desulfofarcimen acetoxidans DSM 771
WP_161832841.1
Turicibacter faecis
MCB1865984.1
Chromatiales bacterium
OIP01653.1
Bacteroidetes bacterium CG2_30_33_31
RZO68811.1
Parvularculaceae bacterium
WP_144351414.1
Sporomusa termitida
NWG38561.1
Hydrogenophilaceae bacterium
WP_144678121.1
Desulfitobacterium sp. LBE
HDQ22822.1
Candidatus Uhrbacteria bacterium
NQZ33370.1
Oceanospirillaceae bacterium
MBQ1770195.1
Turicibacter sp.
WP_005817014.1
Desulfitobacterium hafniense
PLX56360.1
Chromatiales bacterium
MCA8889757.1
Parvularculaceae bacterium
OGY41549.1
Candidatus Buchananbacteria bacterium RBG_13_36_9
NRA29925.1
Parvularculaceae bacterium
NNF67838.1
Gammaproteobacteria bacterium
WP_162576918.1
Variovorax sp. PBL-H6
WP_170861208.1
Trujillonella endophytica
WP_051683725.1
Blastococcus sp. URHD0036
NVN94338.1
Bacteroidota bacterium
MCC7449523.1
Anaerolineae bacterium
MBS1112857.1
Nitrospirota bacterium
WP_120275589.1
WP_163718453.1
Mangrovibacterium lignilyticum
WP_142493757.1
Thalassovita litoralis
PCJ96231.1
Zetaproteobacteria bacterium
WP_051514405.1
Nitrincola nitratireducens
MBR3002618.1
Clostridia bacterium
WP_087571641.1
Paenibacillus sp. MY03
MBK5261708.1
Peptostreptococcaceae bacterium
HHU63041.1
Clostridiales bacterium
HIJ91194.1
Deltaproteobacteria bacterium
MBQ4409800.1
Bacillota bacterium
MBQ6522749.1
Atopobiaceae bacterium
MCD6462604.1
Thermotogota bacterium
MCB5259643.1
Candidatus Cloacimonadota bacterium
MBS1608197.1
Bacteroidota bacterium
OPZ22511.1
Firmicutes bacterium ADurb.BinA205
MBL8298376.1
Rhodanobacteraceae bacterium
MCC6093503.1
Eubacterium sp.
MBI5269048.1
Burkholderiales bacterium
OIO68687.1
Zetaproteobacteria bacterium CG1_02_53_45
MBE7053005.1
Oscillospiraceae bacterium
MBE6778705.1
Oscillospiraceae bacterium
MBI5026114.1
Nitrospirota bacterium
WP_066465535.1
Sanguibacter suarezii NBRC 16159
WP_204366174.1
Halosimplex carlsbadense 2-9-1
MBL4806159.1
Paracoccaceae bacterium
WP_153248031.1
Candidatus Methylospira mobilis
WP_169566112.1
Sneathiella limimaris
MBV8835285.1
Alphaproteobacteria bacterium
WP_074879157.1
Halolamina pelagica
MBR4521149.1
Paludibacteraceae bacterium
MBQ7530578.1
Paludibacteraceae bacterium
MBI5161527.1
Micrococcales bacterium
KXK36812.1
Candidatus Hinthialibacteria bacterium OLB16
MBZ0212434.1
Fimbriimonadaceae bacterium
RVU24093.1
Sandaracinomonas limnophila
MCC7417063.1
Acidobacteriota bacterium
NMC40656.1
Bacteroidales bacterium
HGT26178.1
GAP71136.1
Bacteroidales bacterium 6E
MBQ0045074.1
Candidatus Cryptobacteroides caccocaballi
MCA9411145.1
Candidatus Omnitrophota bacterium
MBN1113517.1
Bacteroidales bacterium
MSU58803.1
Pedosphaera sp.
MBO6781816.1
Alphaproteobacteria bacterium
WP_131088714.1
Glutamicibacter sp. M10
WP_204697940.1
Geomicrobium sediminis
MBR2365993.1
Oscillospiraceae bacterium
MSO40427.1
Solirubrobacterales bacterium
NLT06888.1
Solirubrobacterales bacterium
MBD0282042.1
Thermoleophilaceae bacterium
WP_051472105.1
Patulibacter minatonensis DSM 18081
HEH00625.1
SEH14972.1
Thermoleophilum album

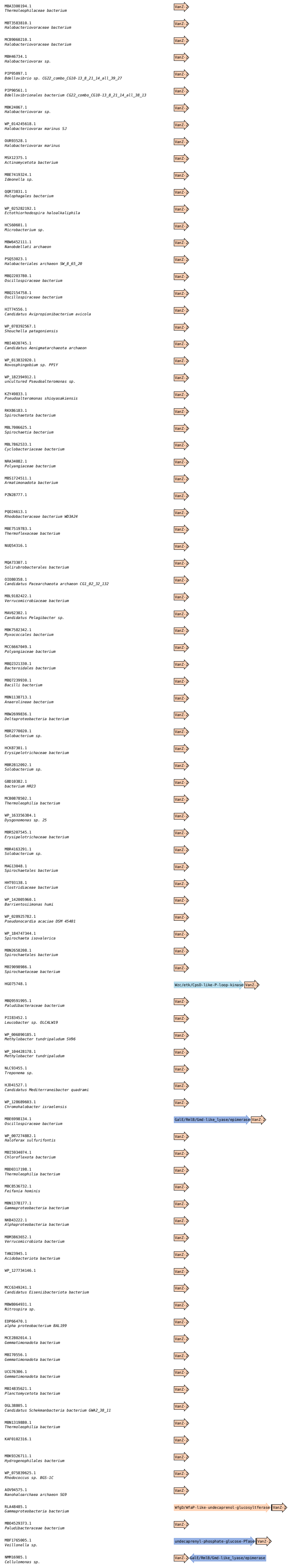
MBA3300194.1
Thermoleophilaceae bacterium
VanZ-2
MBT3583810.1
Halobacteriovoraceae bacterium
VanZ-2
MCB9060210.1
Halobacteriovoraceae bacterium
VanZ-2
MBH46734.1
Halobacteriovorax sp.
VanZ-2
PIP95097.1
Bdellovibrio sp. CG22_combo_CG10-13_8_21_14_all_39_27
VanZ-2
PIP90561.1
Bdellovibrionales bacterium CG22_combo_CG10-13_8_21_14_all_38_13
VanZ-2
MBK24067.1
Halobacteriovorax sp.
VanZ-2
WP_014245618.1
Halobacteriovorax marinus SJ
VanZ-2
OUR93528.1
Halobacteriovorax marinus
VanZ-2
MSX12375.1
Actinomycetota bacterium
VanZ-2
MBE7419324.1
Ideonella sp.
VanZ-2
QQR73831.1
Holophagales bacterium
VanZ-2
WP_025282192.1
Ectothiorhodospira haloalkaliphila
VanZ-2
HCS60601.1
Microbacterium sp.
VanZ-2
MBW6452111.1
Nanobdellati archaeon
VanZ-2
PSQ53023.1
Halobacteriales archaeon SW_8_65_20
VanZ-2
MBQ2203780.1
Oscillospiraceae bacterium
VanZ-2
MBQ2154758.1
Oscillospiraceae bacterium
VanZ-2
HIT74556.1
Candidatus Avipropionibacterium avicola
VanZ-2
WP_078392567.1
Shouchella patagoniensis
VanZ-2
MBI4020745.1
Candidatus Aenigmatarchaeota archaeon
VanZ-2
WP_013832020.1
Novosphingobium sp. PP1Y
VanZ-2
WP_182394912.1
uncultured Pseudoalteromonas sp.
VanZ-2
KZY49833.1
Pseudoalteromonas shioyasakiensis
VanZ-2
RKX86183.1
Spirochaetota bacterium
VanZ-2
MBL7006625.1
Spirochaetia bacterium
VanZ-2
MBL7862533.1
Cyclobacteriaceae bacterium
VanZ-2
NRA34082.1
Polyangiaceae bacterium
VanZ-2
MBS1724511.1
Armatimonadota bacterium
VanZ-2
PZN28777.1
VanZ-2
PQO24613.1
Rhodobacteraceae bacterium WD3A24
VanZ-2
MBE7519783.1
Thermoflexaceae bacterium
VanZ-2
NUQ54316.1
VanZ-2
MQA73307.1
Solirubrobacterales bacterium
VanZ-2
OIO80358.1
Candidatus Pacearchaeota archaeon CG1_02_32_132
VanZ-2
MBL9182422.1
Verrucomicrobiaceae bacterium
VanZ-2
MAV62302.1
Candidatus Pelagibacter sp.
VanZ-2
MBK7582342.1
Myxococcales bacterium
VanZ-2
MCC6667049.1
Polyangiaceae bacterium
VanZ-2
MBQ2321330.1
Bacteroidales bacterium
VanZ-2
MBQ7239930.1
Bacilli bacterium
VanZ-2
MBN1138713.1
Anaerolineae bacterium
VanZ-2
MBW2699836.1
Deltaproteobacteria bacterium
VanZ-2
MBR2770020.1
Solobacterium sp.
VanZ-2
HCK87301.1
Erysipelotrichaceae bacterium
VanZ-2
MBR2812092.1
Solobacterium sp.
VanZ-2
GBD10382.1
bacterium HR23
VanZ-2
MCB0878502.1
Thermoleophilia bacterium
VanZ-2
WP_163356384.1
Dysgonomonas sp. 25
VanZ-2
MBR5207545.1
Erysipelotrichaceae bacterium
VanZ-2
MBR4163291.1
Solobacterium sp.
VanZ-2
MAG13048.1
Spirochaetales bacterium
VanZ-2
HHT93138.1
Clostridiaceae bacterium
VanZ-2
WP_142005960.1
Barrientosiimonas humi
VanZ-2
WP_028925782.1
Pseudonocardia acaciae DSM 45401
VanZ-2
WP_184747344.1
Spirochaeta isovalerica
VanZ-2
MBN2658208.1
Spirochaetales bacterium
VanZ-2
MBI9098986.1
Spirochaetaceae bacterium
VanZ-2
HGO75748.1
Wzc/etk/CpsD-like-P-loop-kinase
VanZ-2
MBQ9591995.1
Paludibacteraceae bacterium
VanZ-2
PII83452.1
Leucobacter sp. OLCALW19
VanZ-2
WP_006890185.1
Methylobacter tundripaludum SV96
VanZ-2
WP_104428178.1
Methylobacter tundripaludum
VanZ-2
NLC93455.1
Treponema sp.
VanZ-2
HJD41527.1
Candidatus Mediterraneibacter quadrami
VanZ-2
WP_128689603.1
Chromohalobacter israelensis
VanZ-2
MBE6998134.1
Oscillospiraceae bacterium
GalE/RmlB/Gmd-like_lyase/epimerase
VanZ-2
WP_007274882.1
Haloferax sulfurifontis
VanZ-2
MBI5934074.1
Chloroflexota bacterium
VanZ-2
MBD0317198.1
Thermoleophilia bacterium
VanZ-2
MBC8536732.1
Feifania hominis
VanZ-2
MBN1378177.1
Gammaproteobacteria bacterium
VanZ-2
NKB43222.1
Alphaproteobacteria bacterium
VanZ-2
MBM3863652.1
Verrucomicrobiota bacterium
VanZ-2
TAN23945.1
Acidobacteriota bacterium
VanZ-2
WP_127734146.1
VanZ-2
MCC6349241.1
Candidatus Eiseniibacteriota bacterium
VanZ-2
MBW8064931.1
Nitrospira sp.
VanZ-2
EDP66470.1
alpha proteobacterium BAL199
VanZ-2
MCE2802014.1
Gemmatimonadota bacterium
VanZ-2
MBI70556.1
Gemmatimonadota bacterium
VanZ-2
UCG76306.1
Gemmatimonadota bacterium
VanZ-2
MBI4835621.1
Planctomycetota bacterium
VanZ-2
OGL38805.1
Candidatus Schekmanbacteria bacterium GWA2_38_11
VanZ-2
MBN1319880.1
Thermoleophilia bacterium
VanZ-2
KAF0102316.1
VanZ-2
MBK9326711.1
Hydrogenophilales bacterium
VanZ-2
WP_075839625.1
Rhodococcus sp. BGS-1C
VanZ-2
AOV94575.1
Nanohaloarchaea archaeon SG9
VanZ-2
RLA48485.1
Gammaproteobacteria bacterium
WfgD/WfaP-like-undecaprenol-glucosyltferase
VanZ-2
MBO4529373.1
Paludibacteraceae bacterium
VanZ-2
MBF1765005.1
Veillonella sp.
undecaprenyl-phosphate-glucose-PTase
VanZ-2
NMM16985.1
Cellulomonas sp.
VanZ-2
GalE/RmlB/Gmd-like_lyase/epimerase

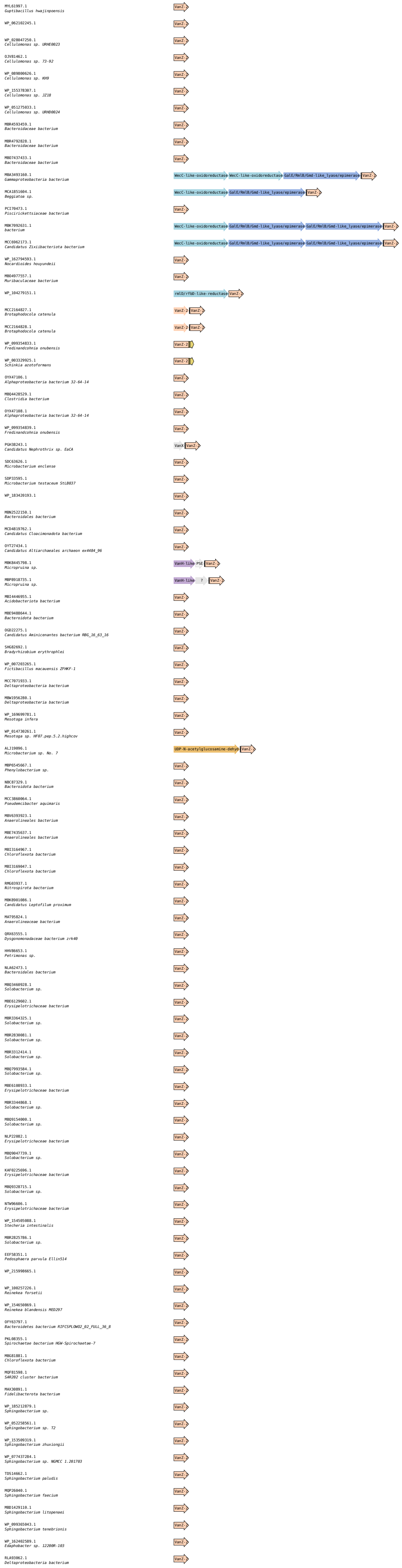

MYL61997.1
Guptibacillus hwajinpoensis
VanZ-2
WP_062102245.1
VanZ-2
WP_028047250.1
Cellulomonas sp. URHE0023
VanZ-2
OJV81462.1
Cellulomonas sp. 73-92
VanZ-2
WP_089800626.1
Cellulomonas sp. KH9
VanZ-2
WP_155378307.1
Cellulomonas sp. JZ18
VanZ-2
WP_051275033.1
Cellulomonas sp. URHD0024
VanZ-2
MBR4593459.1
Bacteroidaceae bacterium
VanZ-2
MBR4792828.1
Bacteroidaceae bacterium
VanZ-2
MBO7437433.1
Bacteroidaceae bacterium
VanZ-2
MBA3493160.1
Gammaproteobacteria bacterium
WecC-like-oxidoreductase
WecC-like-oxidoreductase
GalE/RmlB/Gmd-like_lyase/epimerase
VanZ-2
MCA1851604.1
Beggiatoa sp.
WecC-like-oxidoreductase
GalE/RmlB/Gmd-like_lyase/epimerase
VanZ-2
PCI70473.1
Piscirickettsiaceae bacterium
VanZ-2
MBK7092631.1
bacterium
WecC-like-oxidoreductase
GalE/RmlB/Gmd-like_lyase/epimerase
GalE/RmlB/Gmd-like_lyase/epimerase
VanZ-2
MCC6962173.1
Candidatus Zixiibacteriota bacterium
WecC-like-oxidoreductase
GalE/RmlB/Gmd-like_lyase/epimerase
GalE/RmlB/Gmd-like_lyase/epimerase
VanZ-2
WP_162794593.1
Nocardioides houyundeii
VanZ-2
MBO4977557.1
Muribaculaceae bacterium
VanZ-2
WP_104279151.1
rmlD/rfbD-like-reductase
VanZ-2
MCC2164827.1
Brotaphodocola catenula
VanZ-2
VanZ-2
MCC2164828.1
Brotaphodocola catenula
VanZ-2
VanZ-2
WP_099354833.1
Fredinandcohnia onubensis
VanZ-2
WP_003329925.1
Schinkia azotoformans
VanZ-2
OYX47106.1
Alphaproteobacteria bacterium 32-64-14
VanZ-2
MBQ4428529.1
Clostridia bacterium
VanZ-2
OYX47108.1
Alphaproteobacteria bacterium 32-64-14
VanZ-2
WP_099354839.1
Fredinandcohnia onubensis
VanZ-2
PGH38243.1
Candidatus Nephrothrix sp. EaCA
VanX
VanZ-2
SDC63626.1
Microbacterium enclense
VanZ-2
SDP33595.1
Microbacterium testaceum StLB037
VanZ-2
WP_183420193.1
VanZ-2
MBN2522150.1
Bacteroidales bacterium
VanZ-2
MCD4819762.1
Candidatus Cloacimonadota bacterium
VanZ-2
OYT27434.1
Candidatus Altiarchaeales archaeon ex4484_96
VanZ-2
MBK8445798.1
Micropruina sp.
VanH-like
PSE
VanZ-2
MBP8918735.1
Micropruina sp.
VanH-like
?
VanZ-2
MBI4446955.1
Acidobacteriota bacterium
VanZ-2
MBE9488644.1
Bacteroidota bacterium
VanZ-2
OGD22275.1
Candidatus Aminicenantes bacterium RBG_16_63_16
VanZ-2
SHG82692.1
Bradyrhizobium erythrophlei
VanZ-2
WP_007203265.1
Fictibacillus macauensis ZFHKF-1
VanZ-2
MCC7071933.1
Deltaproteobacteria bacterium
VanZ-2
MBW1956280.1
Deltaproteobacteria bacterium
VanZ-2
WP_169699781.1
Mesotoga infera
VanZ-2
WP_014730261.1
Mesotoga sp. HF07.pep.5.2.highcov
VanZ-2
ALJ19096.1
Microbacterium sp. No. 7
UDP-N-acetylglucosamine-dehyd
VanZ-2
MBP6545667.1
Phenylobacterium sp.
VanZ-2
NBC87329.1
Bacteroidota bacterium
VanZ-2
MCC3860064.1
Pseudemcibacter aquimaris
VanZ-2
MBV6393923.1
Anaerolineales bacterium
VanZ-2
MBE7435637.1
Anaerolineales bacterium
VanZ-2
MBI3164967.1
Chloroflexota bacterium
VanZ-2
MBI3169047.1
Chloroflexota bacterium
VanZ-2
RMG03937.1
Nitrospirota bacterium
VanZ-2
MBK8901086.1
Candidatus Leptofilum proximum
VanZ-2
MAT95824.1
Anaerolineaceae bacterium
VanZ-2
QRX63555.1
Dysgonomonadaceae bacterium zrk40
VanZ-2
HHV86653.1
Petrimonas sp.
VanZ-2
NLA62473.1
Bacteroidales bacterium
VanZ-2
MBQ3460928.1
Solobacterium sp.
VanZ-2
MBE6129602.1
Erysipelotrichaceae bacterium
VanZ-2
MBR3364325.1
Solobacterium sp.
VanZ-2
MBR2830081.1
Solobacterium sp.
VanZ-2
MBR3312414.1
Solobacterium sp.
VanZ-2
MBQ7993584.1
Solobacterium sp.
VanZ-2
MBE6108933.1
Erysipelotrichaceae bacterium
VanZ-2
MBR3344868.1
Solobacterium sp.
VanZ-2
MBQ9154000.1
Solobacterium sp.
VanZ-2
NLP22082.1
Erysipelotrichaceae bacterium
VanZ-2
MBQ9047739.1
Solobacterium sp.
VanZ-2
KAF0225696.1
Erysipelotrichaceae bacterium
VanZ-2
MBQ9328715.1
Solobacterium sp.
VanZ-2
NTW96606.1
Erysipelotrichaceae bacterium
VanZ-2
WP_154505088.1
Stecheria intestinalis
VanZ-2
MBR2825786.1
Solobacterium sp.
VanZ-2
EEF58351.1
Pedosphaera parvula Ellin514
VanZ-2
WP_215998665.1
VanZ-2
WP_100257226.1
Reinekea forsetii
VanZ-2
WP_154650869.1
Reinekea blandensis MED297
VanZ-2
OFY63797.1
Bacteroidetes bacterium RIFCSPLOWO2_02_FULL_36_8
VanZ-2
PKL08355.1
Spirochaetae bacterium HGW-Spirochaetae-7
VanZ-2
MBG81881.1
Chloroflexota bacterium
VanZ-2
MQF81598.1
SAR202 cluster bacterium
VanZ-2
MAX30891.1
Fidelibacterota bacterium
VanZ-2
WP_185212879.1
Sphingobacterium sp.
VanZ-2
WP_052258561.1
Sphingobacterium sp. T2
VanZ-2
WP_153509319.1
Sphingobacterium zhuxiongii
VanZ-2
WP_077437284.1
Sphingobacterium sp. NGMCC 1.201703
VanZ-2
TDS14662.1
Sphingobacterium paludis
VanZ-2
MQP26040.1
Sphingobacterium faecium
VanZ-2
MBD1429110.1
Sphingobacterium litopenaei
VanZ-2
WP_099365043.1
Sphingobacterium tenebrionis
VanZ-2
WP_162402589.1
Edaphobacter sp. 12200R-103
VanZ-2
RLA93062.1
Deltaproteobacteria bacterium
VanZ-2

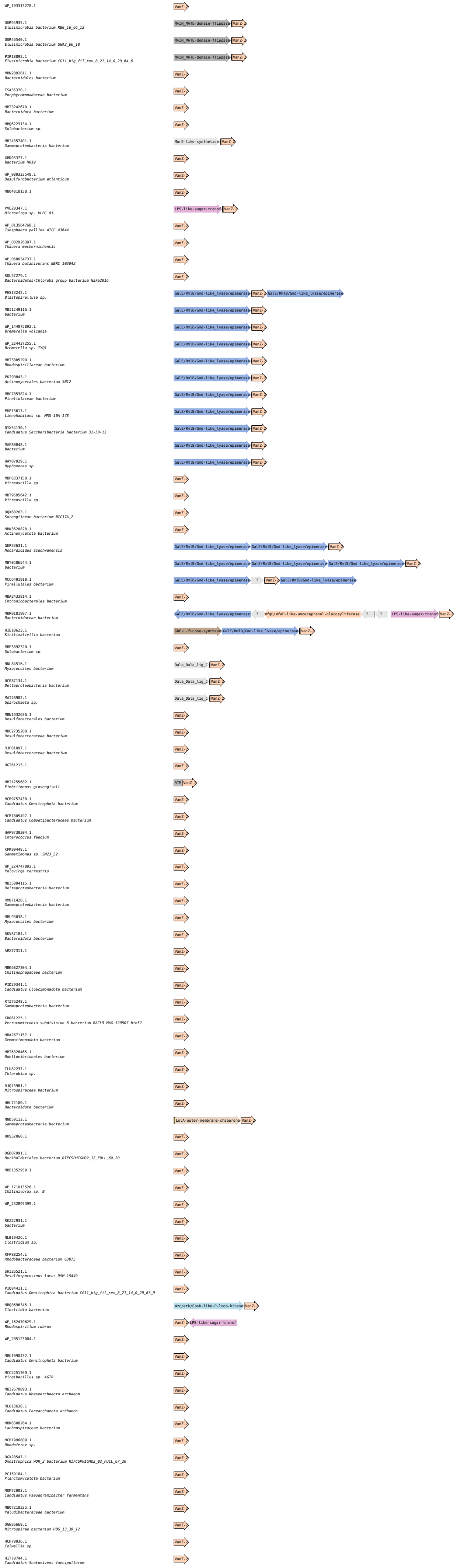
WP_103313278.1
VanZ
OGR94935.1
Elusimicrobia bacterium RBG_16_66_12
MviN_MATE-domain-flippase
VanZ
OGR46540.1
Elusimicrobia bacterium GWA2_66_18
MviN_MATE-domain-flippase
VanZ
PIR18892.1
Elusimicrobia bacterium CG11_big_fil_rev_8_21_14_0_20_64_6
MviN_MATE-domain-flippase
VanZ
MBN2892811.1
Bacteroidales bacterium
VanZ
TSA35370.1
Porphyromonadaceae bacterium
VanZ
MBT3242679.1
Bacteroidota bacterium
VanZ
MBQ6223134.1
Solobacterium sp.
VanZ
MBI4357401.1
Gammaproteobacteria bacterium
MurE-like-synthetase
VanZ
GBD03377.1
bacterium HR19
VanZ
WP_089323548.1
Desulfurobacterium atlanticum
VanZ
MBO4818138.1
VanZ
PVE20347.1
Microvirga sp. KLBC 81
LPS-like-sugar-transf
VanZ
WP_013564768.1
Isosphaera pallida ATCC 43644
VanZ
WP_002926397.1
Thauera mechernichensis
VanZ
WP_068634737.1
Thauera butanivorans NBRC 103042
VanZ
ROL57279.1
Bacteroidetes/Chlorobi group bacterium Naka2016
VanZ
PHS13242.1
Blastopirellula sp.
GalE/RmlB/Gmd-like_lyase/epimerase
VanZ
GalE/RmlB/Gmd-like_lyase/epimerase
MBI1249116.1
bacterium
GalE/RmlB/Gmd-like_lyase/epimerase
VanZ
WP_144975092.1
Bremerella volcania
GalE/RmlB/Gmd-like_lyase/epimerase
VanZ
WP_224437255.1
Bremerella sp. TYQ1
GalE/RmlB/Gmd-like_lyase/epimerase
VanZ
MBT3885290.1
Rhodospirillaceae bacterium
GalE/RmlB/Gmd-like_lyase/epimerase
VanZ
PKI90843.1
Actinomycetales bacterium SN12
GalE/RmlB/Gmd-like_lyase/epimerase
VanZ
MBC7853824.1
Pirellulaceae bacterium
GalE/RmlB/Gmd-like_lyase/epimerase
VanZ
PUE13617.1
Limnohabitans sp. MMS-10A-178
GalE/RmlB/Gmd-like_lyase/epimerase
VanZ
OYX54130.1
Candidatus Saccharibacteria bacterium 32-50-13
GalE/RmlB/Gmd-like_lyase/epimerase
VanZ
MAF80848.1
bacterium
GalE/RmlB/Gmd-like_lyase/epimerase
VanZ
HAY07929.1
Hyphomonas sp.
GalE/RmlB/Gmd-like_lyase/epimerase
VanZ
MBP6337150.1
Vitreoscilla sp.
VanZ
MBT9595642.1
Vitreoscilla sp.
VanZ
OQX68263.1
Sorangiineae bacterium NIC37A_2
VanZ
MBW3620828.1
Actinomycetota bacterium
VanZ
GEP33631.1
Nocardioides szechwanensis
GalE/RmlB/Gmd-like_lyase/epimerase
GalE/RmlB/Gmd-like_lyase/epimerase
VanZ
MBY0586544.1
bacterium
GalE/RmlB/Gmd-like_lyase/epimerase
GalE/RmlB/Gmd-like_lyase/epimerase
GalE/RmlB/Gmd-like_lyase/epimerase
VanZ
MCC6491910.1
Pirellulales bacterium
GalE/RmlB/Gmd-like_lyase/epimerase
?
VanZ
GalE/RmlB/Gmd-like_lyase/epimerase
MBA2433814.1
Chthoniobacterales bacterium
VanZ
MBR0181997.1
Bacteroidaceae bacterium
GalE/RmlB/Gmd-like_lyase/epimerase
?
WfgD/WfaP-like-undecaprenol-glucosyltferase
?
?
LPS-like-sugar-transf
VanZ
HIE10623.1
Kiritimatiellia bacterium
GDP-L-fucose-synthase
GalE/RmlB/Gmd-like_lyase/epimerase
VanZ
MBP3892320.1
Solobacterium sp.
VanZ
NNL84516.1
Myxococcales bacterium
Dala_Dala_lig_C
VanZ
UCE87134.1
Deltaproteobacteria bacterium
Dala_Dala_lig_C
VanZ
MAI26902.1
Spirochaeta sp.
Dala_Dala_lig_C
VanZ
MBN1932636.1
Desulfobacterales bacterium
VanZ
MBC2735200.1
Desulfobacteraceae bacterium
VanZ
RJP81097.1
Desulfobacteraceae bacterium
VanZ
HGT61215.1
VanZ
MBI1755682.1
Fimbriimonas ginsengisoli
STM
VanZ
MCB9757438.1
Candidatus Omnitrophota bacterium
VanZ
MCB1805497.1
Candidatus Competibacteraceae bacterium
VanZ
HAP9739304.1
Enterococcus faecium
VanZ
KPK80448.1
Gemmatimonas sp. SM23_52
VanZ
WP_224747093.1
Pelovirga terrestris
VanZ
MBI5894115.1
Deltaproteobacteria bacterium
VanZ
RMD72428.1
Gammaproteobacteria bacterium
VanZ
MBL93038.1
Myxococcales bacterium
VanZ
RKV87184.1
Bacteroidota bacterium
VanZ
ARX77311.1
VanZ
MBK6827304.1
Chitinophagaceae bacterium
VanZ
PID29341.1
Candidatus Cloacimonadota bacterium
VanZ
RTZ76248.1
Gammaproteobacteria bacterium
VanZ
KRO61225.1
Verrucomicrobia subdivision 6 bacterium BACL9 MAG-120507-bin52
VanZ
MBA2671157.1
Gemmatimonadota bacterium
VanZ
MBT6326465.1
Bdellovibrionales bacterium
VanZ
TLU82237.1
Chlorobium sp.
VanZ
RJO13981.1
Nitrospiraceae bacterium
VanZ
HHL72100.1
Bacteroidota bacterium
VanZ
NND59112.1
Gammaproteobacteria bacterium
LolA-outer-membrane-chaperone
VanZ
HHS52860.1
VanZ
OGB07991.1
Burkholderiales bacterium RIFCSPHIGHO2_12_FULL_69_20
VanZ
MBE1352959.1
VanZ
WP_171013526.1
Chitinivorax sp. B
VanZ
WP_232897399.1
VanZ
RKZ22931.1
bacterium
VanZ
NLB19426.1
Clostridium sp.
VanZ
RFP88254.1
Rhodobacteraceae bacterium 63075
VanZ
SHI26521.1
Desulfosporosinus lacus DSM 15449
VanZ
PIZ84411.1
Candidatus Omnitrophica bacterium CG11_big_fil_rev_8_21_14_0_20_63_9
VanZ
MBQ8696345.1
Clostridia bacterium
Wzc/etk/CpsD-like-P-loop-kinase
VanZ
WP_162470629.1
Rhodospirillum rubrum
VanZ
LPS-like-sugar-transf
WP_205123004.1
VanZ
MBU1090432.1
Candidatus Omnitrophota bacterium
VanZ
MCC2251369.1
Virgibacillus sp. AGTR
VanZ
MBI2670893.1
Candidatus Woesearchaeota archaeon
VanZ
RLG13638.1
Candidatus Pacearchaeota archaeon
VanZ
MBR6308264.1
Lachnospiraceae bacterium
VanZ
MCB1996809.1
Rhodoferax sp.
VanZ
OGX28547.1
Omnitrophica WOR_2 bacterium RIFCSPHIGHO2_02_FULL_67_20
VanZ
PCJ59184.1
Planctomycetota bacterium
VanZ
MQM72003.1
Candidatus Pseudoramibacter fermentans
VanZ
MBQ7210325.1
Paludibacteraceae bacterium
VanZ
OGW36669.1
Nitrospirae bacterium RBG_13_39_12
VanZ
HCH70936.1
Colwellia sp.
VanZ
HIT70744.1
Candidatus Scatovivens faecipullorum
VanZ

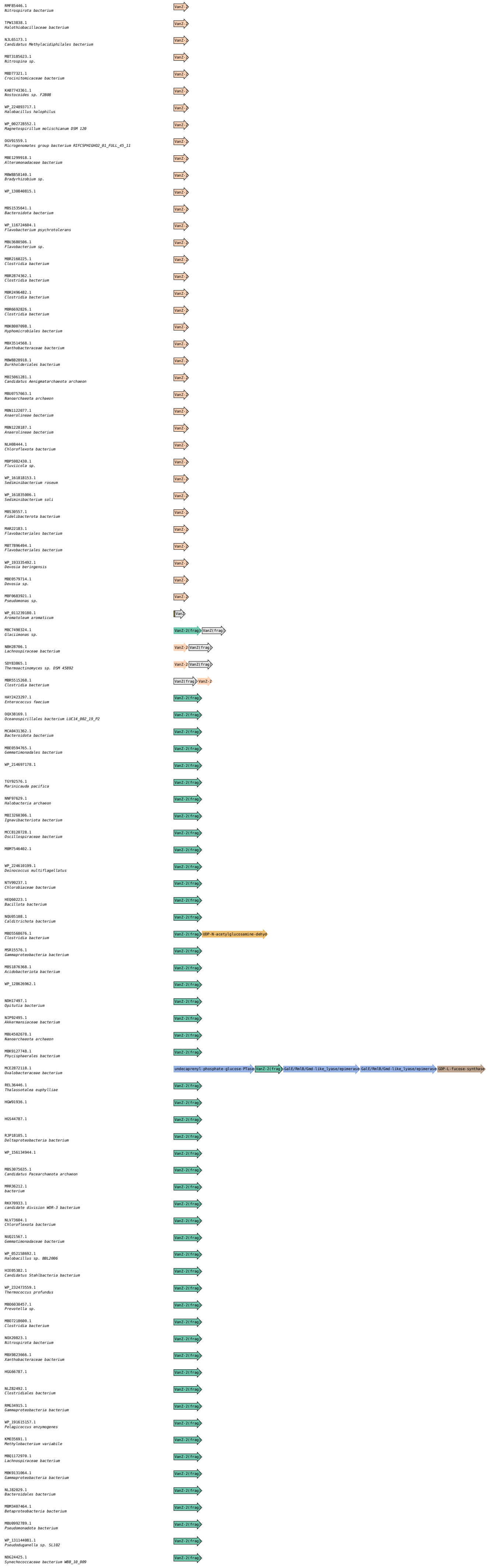
RMF85446.1
Nitrospirota bacterium
VanZ-2
TPW13838.1
Halothiobacillaceae bacterium
VanZ-2
NJL65173.1
Candidatus Methylacidiphilales bacterium
VanZ-2
MBT3185623.1
Nitrospina sp.
VanZ-2
MBD77321.1
Crocinitomicaceae bacterium
VanZ-2
KAB7743361.1
Nostocoides sp. F2B08
VanZ-2
WP_224893717.1
Halobacillus halophilus
VanZ-2
WP_002728552.1
Magnetospirillum molischianum DSM 120
VanZ-2
OGV91559.1
Microgenomates group bacterium RIFCSPHIGHO2_01_FULL_45_11
VanZ-2
MBE1299918.1
Alteromonadaceae bacterium
VanZ-2
MBW8858140.1
Bradyrhizobium sp.
VanZ-2
WP_130840815.1
VanZ-2
MBS1535641.1
Bacteroidota bacterium
VanZ-2
WP_116724604.1
Flavobacterium psychrotolerans
VanZ-2
MBU3680506.1
Flavobacterium sp.
VanZ-2
MBR2160225.1
Clostridia bacterium
VanZ-2
MBR2874362.1
Clostridia bacterium
VanZ-2
MBR2496482.1
Clostridia bacterium
VanZ-2
MBR6692826.1
Clostridia bacterium
VanZ-2
MBK8007098.1
Hyphomicrobiales bacterium
VanZ-2
MBX3514568.1
Xanthobacteraceae bacterium
VanZ-2
MBW8828918.1
Burkholderiales bacterium
VanZ-2
MBI5061281.1
Candidatus Aenigmatarchaeota archaeon
VanZ-2
MBU0757663.1
Nanoarchaeota archaeon
VanZ-2
MBN1122077.1
Anaerolineae bacterium
VanZ-2
MBN1228187.1
Anaerolineae bacterium
VanZ-2
NLH08444.1
Chloroflexota bacterium
VanZ-2
MBP5982430.1
Fluviicola sp.
VanZ-2
WP_161818153.1
Sediminibacterium roseum
VanZ-2
WP_161835006.1
Sediminibacterium soli
VanZ-2
MBS30557.1
Fidelibacterota bacterium
VanZ-2
MAR22183.1
Flavobacteriales bacterium
VanZ-2
MBT7896494.1
Flavobacteriales bacterium
VanZ-2
WP_193335492.1
Devosia beringensis
VanZ-2
MBE0579714.1
Devosia sp.
VanZ-2
MBF0683921.1
Pseudomonas sp.
VanZ-2
WP_011239180.1
Aromatoleum aromaticum
VanZ
MBC7490324.1
Glaciimonas sp.
VanZ-2(frag) VanZ(frag)
NBH28706.1
Lachnospiraceae bacterium
VanZ-2 VanZ(frag)
SDY83865.1
Thermoactinomyces sp. DSM 45892
VanZ-2 VanZ(frag)
MBR5515268.1
Clostridia bacterium
VanZ(frag) VanZ-2
HAY2423297.1
Enterococcus faecium
VanZ-2(frag)
OQX38169.1
Oceanospirillales bacterium LUC14_002_19_P2
VanZ-2(frag)
MCA0431362.1
Bacteroidota bacterium
VanZ-2(frag)
MBE0594765.1
Gemmatimonadales bacterium
VanZ-2(frag)
WP_214697178.1
VanZ-2(frag)
TGY92576.1
Marinicauda pacifica
VanZ-2(frag)
NNF97629.1
Halobacteria archaeon
VanZ-2(frag)
MBI3260306.1
Ignavibacteriota bacterium
VanZ-2(frag)
MCC8120728.1
Oscillospiraceae bacterium
VanZ-2(frag)
MBM7546402.1
VanZ-2(frag)
WP_224610199.1
Deinococcus multiflagellatus
VanZ-2(frag)
NTV99237.1
Chlorobiaceae bacterium
VanZ-2(frag)
HEQ60223.1
Bacillota bacterium
VanZ-2(frag)
NQU05108.1
Calditrichota bacterium
VanZ-2(frag)
MBO5568676.1
Clostridia bacterium
VanZ-2(frag) UDP-N-acetylglucosamine-dehyd
MSR15576.1
Gammaproteobacteria bacterium
VanZ-2(frag)
MBS1876368.1
Acidobacteriota bacterium
VanZ-2(frag)
WP_128626962.1
VanZ-2(frag)
NDH17497.1
Opitutia bacterium
VanZ-2(frag)
NIP92495.1
Akkermansiaceae bacterium
VanZ-2(frag)
MBU4502678.1
Nanoarchaeota archaeon
VanZ-2(frag)
MBK9127748.1
Phycisphaerales bacterium
VanZ-2(frag)
MCE2872118.1
Oxalobacteraceae bacterium
undecaprenyl-phosphate-glucose-PTase VanZ-2(frag) GalE/RmlB/Gmd-like_lyase/epimerase GalE/RmlB/Gmd-like_lyase/epimerase GDP-L-fucose-synthase
REL36446.1
Thalassotalea euphylliae
VanZ-2(frag)
HGW91936.1
VanZ-2(frag)
HGS44787.1
VanZ-2(frag)
RJP18105.1
Deltaproteobacteria bacterium
VanZ-2(frag)
WP_156134944.1
VanZ-2(frag)
MBS3075635.1
Candidatus Pacearchaeota archaeon
VanZ-2(frag)
MRR36212.1
bacterium
VanZ-2(frag)
RKX70933.1
candidate division WOR-3 bacterium
VanZ-2(frag)
NLV73604.1
Chloroflexota bacterium
VanZ-2(frag)
NUQ21567.1
Gemmatimonadaceae bacterium
VanZ-2(frag)
WP_052158692.1
Halobacillus sp. BBL2006
VanZ-2(frag)
HIE05382.1
Candidatus Stahlbacteria bacterium
VanZ-2(frag)
WP_232473559.1
Thermococcus profundus
VanZ-2(frag)
MBO6030457.1
Prevotella sp.
VanZ-2(frag)
MBO7218600.1
Clostridia bacterium
VanZ-2(frag)
NOX20823.1
Nitrospirota bacterium
VanZ-2(frag)
MBX9823666.1
Xanthobacteraceae bacterium
VanZ-2(frag)
HGG66787.1
VanZ-2(frag)
NLZ82492.1
Clostridiales bacterium
VanZ-2(frag)
RMG34915.1
Gammaproteobacteria bacterium
VanZ-2(frag)
WP_191615157.1
Pelagicoccus enzymogenes
VanZ-2(frag)
KMO35691.1
Methylobacterium variabile
VanZ-2(frag)
MBQ1172970.1
Lachnospiraceae bacterium
VanZ-2(frag)
MBK9131064.1
Gammaproteobacteria bacterium
VanZ-2(frag)
NLJ82029.1
Bacteroidales bacterium
VanZ-2(frag)
MBM3407464.1
Betaproteobacteria bacterium
VanZ-2(frag)
MBU0992789.1
Pseudomonadota bacterium
VanZ-2(frag)
WP_131144081.1
Pseudoduganella sp. SL102
VanZ-2(frag)
NDG24425.1
Synechococcaceae bacterium WBB_10_009
VanZ-2(frag)

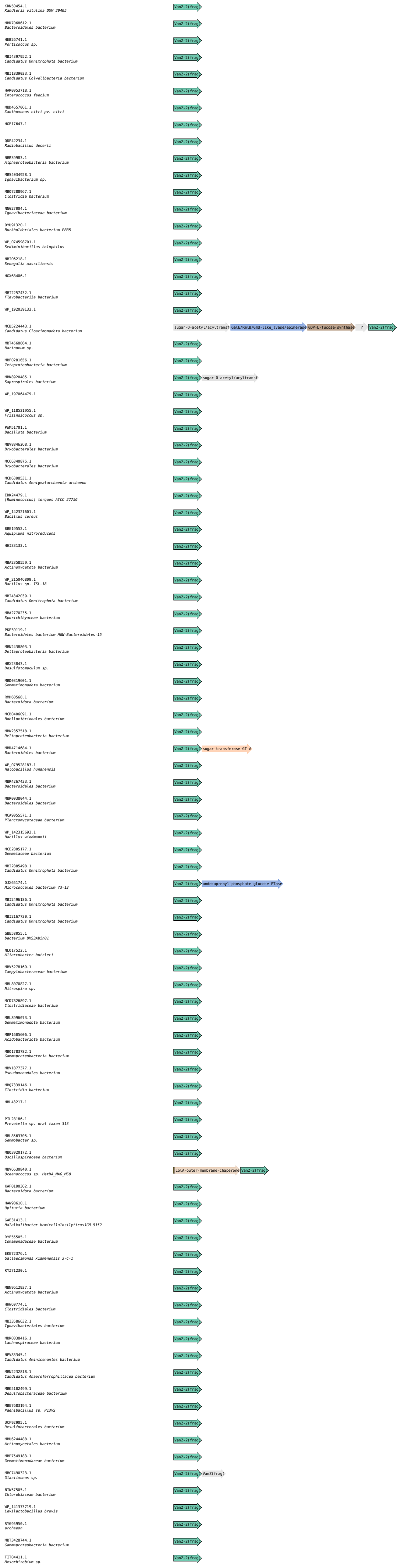
KRN50454.1
Kandleria vitulina DSM 20405
VanZ-2(frag
MBR7068612.1
Bacteroidales bacterium
VanZ-2(frag
HEB26741.1
Porticoccus sp.
VanZ-2(frag
MBI4397952.1
Candidatus Omnitrophota bacterium
VanZ-2(frag
MBI1839023.1
Candidatus Colwellbacteria bacterium
VanZ-2(frag
HAR0953718.1
Enterococcus faecium
VanZ-2(frag
MBD4657061.1
Xanthomonas citri pv. citri
VanZ-2(frag
HGE17647.1
VanZ-2(frag
QDP42234.1
Radiobacillus deserti
VanZ-2(frag
NBR39983.1
Alphaproteobacteria bacterium
VanZ-2(frag
MBS4034928.1
Ignavibacterium sp.
VanZ-2(frag
MBO7288967.1
Clostridia bacterium
VanZ-2(frag
NNG27004.1
Ignavibacteriaceae bacterium
VanZ-2(frag
OYU91320.1
Burkholderiales bacterium PBB5
VanZ-2(frag
WP_074598701.1
Sediminibacillus halophilus
VanZ-2(frag
NBI06218.1
Senegalia massiliensis
VanZ-2(frag
HGX68406.1
VanZ-2(frag
MBI2257432.1
Flavobacteriia bacterium
VanZ-2(frag
WP_192039133.1
VanZ-2(frag
MCB5224443.1
Candidatus Cloacimonadota bacterium
sugar-O-acetyl/acyltransf
GalE/RmlB/Gmd-like_lyase/epimerase
GDP-L-fucose-synthase
?
VanZ-2(frag)
MBT4568864.1
Marinovum sp.
VanZ-2(frag
MBF0281656.1
Zetaproteobacteria bacterium
VanZ-2(frag
MBK8920485.1
Saprospirales bacterium
VanZ-2(frag
sugar-O-acetyl/acyltransf
WP_197064479.1
VanZ-2(frag
WP_118521955.1
Frisingicoccus sp.
VanZ-2(frag
PWM51701.1
Bacillota bacterium
VanZ-2(frag
MBV8846268.1
Bryobacterales bacterium
VanZ-2(frag
MCC6340875.1
Bryobacterales bacterium
VanZ-2(frag
MCD6398531.1
Candidatus Aenigmatarchaeota archaeon
VanZ-2(frag
EDK24479.1
[Ruminococcus] torques ATCC 27756
VanZ-2(frag
WP_142321601.1
Bacillus cereus
VanZ-2(frag
BBE19552.1
Aquipluma nitroreducens
VanZ-2(frag
HHI33133.1
VanZ-2(frag
MBA2358559.1
Actinomycetota bacterium
VanZ-2(frag
WP_215046809.1
Bacillus sp. ISL-18
VanZ-2(frag
MBI4342039.1
Candidatus Omnitrophota bacterium
VanZ-2(frag
MBA2770235.1
Sporichthyaceae bacterium
VanZ-2(frag
PKP39119.1
Bacteroidetes bacterium HGW-Bacteroidetes-15
VanZ-2(frag
MBN2438803.1
Deltaproteobacteria bacterium
VanZ-2(frag
HBX23843.1
Desulfotomaculum sp.
VanZ-2(frag
MBD0319601.1
Gemmatimonadota bacterium
VanZ-2(frag
RMH60568.1
Bacteroidota bacterium
VanZ-2(frag
MCB0406091.1
Bdellovibrionales bacterium
VanZ-2(frag
MBW2357518.1
Deltaproteobacteria bacterium
VanZ-2(frag
MBR4714684.1
Bacteroidales bacterium
VanZ-2(frag
sugar-transferase-GT-A
WP_079528183.1
Halobacillus hunanensis
VanZ-2(frag
MBR4267433.1
Bacteroidales bacterium
VanZ-2(frag
MBR0038044.1
Bacteroidales bacterium
VanZ-2(frag
MCA9055571.1
Planctomycetaceae bacterium
VanZ-2(frag
WP_142315693.1
Bacillus wiedmannii
VanZ-2(frag
MCE2805177.1
Gemmataceae bacterium
VanZ-2(frag
MBI2885498.1
Candidatus Omnitrophota bacterium
VanZ-2(frag
OJX65174.1
Micrococcales bacterium 73-13
VanZ-2(frag
undecaprenyl-phosphate-glucose-PTase
MBI2496186.1
Candidatus Omnitrophota bacterium
VanZ-2(frag
MBI2167730.1
Candidatus Omnitrophota bacterium
VanZ-2(frag
GBE58055.1
bacterium BMS3Abin01
VanZ-2(frag
NLO17522.1
Aliarcobacter butzleri
VanZ-2(frag
MBV5278169.1
Campylobacteraceae bacterium
VanZ-2(frag
MBL8070827.1
Nitrospira sp.
VanZ-2(frag
MCD7826897.1
Clostridiaceae bacterium
VanZ-2(frag
MBL8996073.1
Gemmatimonadota bacterium
VanZ-2(frag
MBP1605606.1
Acidobacteriota bacterium
VanZ-2(frag
MBQ1783782.1
Gammaproteobacteria bacterium
VanZ-2(frag
MBV1877377.1
Pseudomonadales bacterium
VanZ-2(frag
MBQ7339146.1
Clostridia bacterium
VanZ-2(frag
HHL43217.1
VanZ-2(frag
PTL28186.1
Prevotella sp. oral taxon 313
VanZ-2(frag
MBL8563705.1
Gemmobacter sp.
VanZ-2(frag
MBQ3920172.1
Oscillospiraceae bacterium
VanZ-2(frag
MBV6630840.1
Oceanococcus sp. HetDA_MAG_MS8
LolA-outer-membrane-chaperone
VanZ-2(frag
KAF0190362.1
Bacteroidota bacterium
VanZ-2(frag
HAW98610.1
Opitutia bacterium
VanZ-2(frag
GAE31413.1
Halalkalibacter hemicellulosilyticusJCM 9152
VanZ-2(frag
RYF55505.1
Comamonadaceae bacterium
VanZ-2(frag
EKE72376.1
Gallaecimonas xiamenensis 3-C-1
VanZ-2(frag
RYZ71230.1
VanZ-2(frag
MBN9612937.1
Actinomycetota bacterium
VanZ-2(frag
HHW69774.1
Clostridiales bacterium
VanZ-2(frag
MBI3586632.1
Ignavibacteriales bacterium
VanZ-2(frag
MBR0038416.1
Lachnospiraceae bacterium
VanZ-2(frag
NPV83345.1
Candidatus Aminicenantes bacterium
VanZ-2(frag
MBN2232818.1
Candidatus Anaeroferrophillacea bacterium
VanZ-2(frag
MBK5102499.1
Desulfobacteraceae bacterium
VanZ-2(frag
MBE7683194.1
Paenibacillus sp. P13VS
VanZ-2(frag
UCF92905.1
Desulfobacterales bacterium
VanZ-2(frag
MBU6244488.1
Actinomycetales bacterium
VanZ-2(frag
MBP7549183.1
Gemmatimonadaceae bacterium
VanZ-2(frag
MBC7490323.1
Glaciimonas sp.
VanZ-2(frag
VanZ(frag)
NTW57505.1
Chlorobiaceae bacterium
VanZ-2(frag
WP_141373719.1
Levilactobacillus brevis
VanZ-2(frag
RYG95950.1
archaeon
VanZ-2(frag
MBT3428744.1
Gammaproteobacteria bacterium
VanZ-2(frag
TIT04411.1
Mesorhizobium sp.
VanZ-2(frag

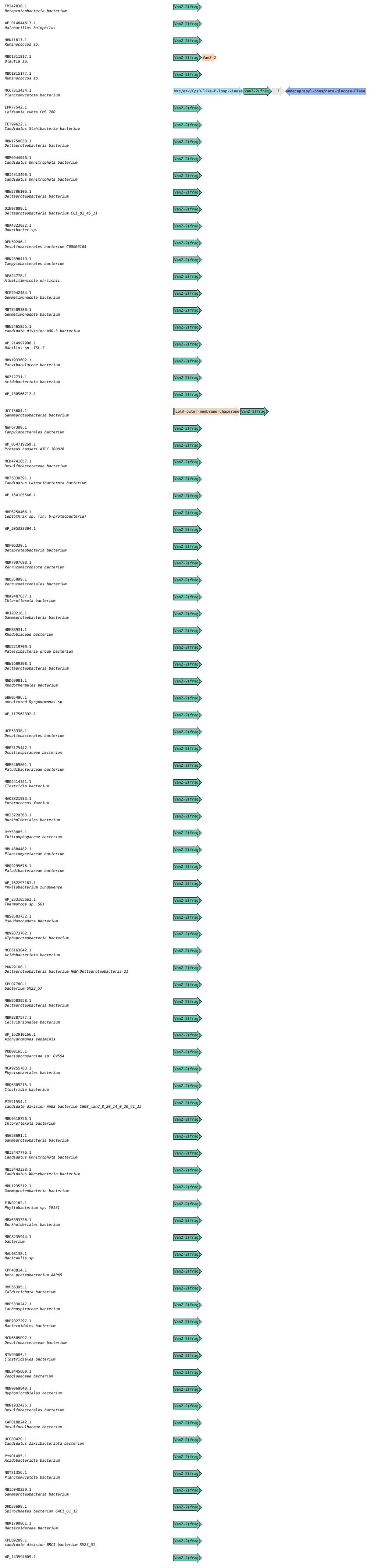
TMI42838.1
Betaproteobacteria bacterium
VanZ-2(frag)
WP_014644613.1
Halobacillus halophilus
VanZ-2(frag)
HBN11617.1
Ruminococcus sp.
VanZ-2(frag)
MBQ1311817.1
Blautia sp.
VanZ-2(frag) VanZ-2
MBQ1815177.1
Ruminococcus sp.
VanZ-2(frag)
MCC7313434.1
Planctomycetota bacterium
Wzc/etk/CpsD-like-P-loop-kinase VanZ-2(frag) ? undecaprenyl-phosphate-glucose-PTase
EPR77542.1
Leifsonia rubra CMS 76R
VanZ-2(frag)
TET99622.1
Candidatus Stahlbacteria bacterium
VanZ-2(frag)
MBW1750038.1
Deltaproteobacteria bacterium
VanZ-2(frag)
MBP6944046.1
Candidatus Omnitrophota bacterium
VanZ-2(frag)
MBI4313490.1
Candidatus Omnitrophota bacterium
VanZ-2(frag)
MBW2706106.1
Deltaproteobacteria bacterium
VanZ-2(frag)
OIN97009.1
Deltaproteobacteria bacterium CG1_02_45_11
VanZ-2(frag)
MBA4323032.1
Odoribacter sp.
VanZ-2(frag)
OEU59246.1
Desulfobacterales bacterium C00003104
VanZ-2(frag)
MBN2896419.1
Campylobacterales bacterium
VanZ-2(frag)
RFA24778.1
Alkalilimnicola ehrlichii
VanZ-2(frag)
MCE2942484.1
Gemmatimonadota bacterium
VanZ-2(frag)
MBT8489380.1
Gemmatimonadota bacterium
VanZ-2(frag)
MBN2465855.1
candidate division WOR-3 bacterium
VanZ-2(frag)
WP_214997908.1
Bacillus sp. ISL-7
VanZ-2(frag)
MBV1933602.1
Parvibaculaceae bacterium
VanZ-2(frag)
NOZ12731.1
Acidobacteriota bacterium
VanZ-2(frag)
WP_130566712.1
VanZ-2(frag)
UCC15604.1
Gammaproteobacteria bacterium
LolA-outer-membrane-chaperone VanZ-2(frag)
NWF67309.1
Campylobacterales bacterium
VanZ-2(frag)
WP_064719269.1
Proteus hauseri ATCC 700826
VanZ-2(frag)
MCD4741857.1
Desulfobacteraceae bacterium
VanZ-2(frag)
MBT5830391.1
Candidatus Latescibacterota bacterium
VanZ-2(frag)
WP_164105546.1
VanZ-2(frag)
MBP6250466.1
Leptothrix sp. (in: b-proteobacteria)
VanZ-2(frag)
WP_205323304.1
VanZ-2(frag)
NDF06330.1
Betaproteobacteria bacterium
VanZ-2(frag)
MBK7997698.1
Verrucomicrobiota bacterium
VanZ-2(frag)
MBO35099.1
Verrucomicrobiales bacterium
VanZ-2(frag)
MBA2487837.1
Chloroflexota bacterium
VanZ-2(frag)
HHJ20218.1
Gammaproteobacteria bacterium
VanZ-2(frag)
HBM88931.1
Rhodobiaceae bacterium
VanZ-2(frag)
MBU2219709.1
Patescibacteria group bacterium
VanZ-2(frag)
MBW2609398.1
Deltaproteobacteria bacterium
VanZ-2(frag)
NND69981.1
Rhodothermales bacterium
VanZ-2(frag)
SBW05496.1
uncultured Dysgonomonas sp.
VanZ-2(frag)
WP_117562392.1
VanZ-2(frag)
UCE53338.1
Desulfobacterales bacterium
VanZ-2(frag)
MBR3175442.1
Oscillospiraceae bacterium
VanZ-2(frag)
MBR5469801.1
Paludibacteraceae bacterium
VanZ-2(frag)
MBO4414341.1
Clostridia bacterium
VanZ-2(frag)
HAQ3821983.1
Enterococcus faecium
VanZ-2(frag)
MBI3229363.1
Burkholderiales bacterium
VanZ-2(frag)
RYY53985.1
Chitinophagaceae bacterium
VanZ-2(frag)
MBL4884482.1
Planctomycetaceae bacterium
VanZ-2(frag)
MBQ9295676.1
Paludibacteraceae bacterium
VanZ-2(frag)
WP_162293161.1
Phyllobacterium zundukense
VanZ-2(frag)
WP_233185662.1
Thermotoga sp. SG1
VanZ-2(frag)
MBS0503732.1
Pseudomonadota bacterium
VanZ-2(frag)
MBV9373762.1
Alphaproteobacteria bacterium
VanZ-2(frag)
MCC6163842.1
Acidobacteriota bacterium
VanZ-2(frag)
PKN29168.1
Deltaproteobacteria bacterium HGW-Deltaproteobacteria-21
VanZ-2(frag)
KPL07780.1
bacterium SM23_57
VanZ-2(frag)
MBW2683958.1
Deltaproteobacteria bacterium
VanZ-2(frag)
MBK8287577.1
Cellvibrionales bacterium
VanZ-2(frag)
WP_162910166.1
Azohydromonas sediminis
VanZ-2(frag)
PUB08165.1
Paenisporosarcina sp. OV554
VanZ-2(frag)
MCA9255783.1
Phycisphaerales bacterium
VanZ-2(frag)
MBQ6805215.1
Clostridia bacterium
VanZ-2(frag)
PIS21554.1
candidate division WWE3 bacterium CG08_land_8_20_14_0_20_41_15
VanZ-2(frag)
MBU0510756.1
Chloroflexota bacterium
VanZ-2(frag)
HGG58691.1
Gammaproteobacteria bacterium
VanZ-2(frag)
MBI2447776.1
Candidatus Omnitrophota bacterium
VanZ-2(frag)
MBI3443338.1
Candidatus Woesebacteria bacterium
VanZ-2(frag)
MBU1235312.1
Gammaproteobacteria bacterium
VanZ-2(frag)
EJN02162.1
Phyllobacterium sp. YR531
VanZ-2(frag)
MBX6393336.1
Burkholderiales bacterium
VanZ-2(frag)
MBC8235944.1
bacterium
VanZ-2(frag)
MAL08138.1
Maricaulis sp.
VanZ-2(frag)
KPF48814.1
beta proteobacterium AAP65
VanZ-2(frag)
RMF56395.1
Calditrichota bacterium
VanZ-2(frag)
MBP5330247.1
Lachnospiraceae bacterium
VanZ-2(frag)
MBP7027297.1
Bacteroidales bacterium
VanZ-2(frag)
MCD6585097.1
Desulfobacteraceae bacterium
VanZ-2(frag)
NTV90085.1
Clostridiales bacterium
VanZ-2(frag)
MBL8445669.1
Zoogloeaceae bacterium
VanZ-2(frag)
MBN9069840.1
Hyphomicrobiales bacterium
VanZ-2(frag)
MBN1932425.1
Desulfobacterales bacterium
VanZ-2(frag)
KAF0188242.1
Desulfobulbaceae bacterium
VanZ-2(frag)
UCC80420.1
Candidatus Zixiibacteriota bacterium
VanZ-2(frag)
PYV01495.1
Acidobacteriota bacterium
VanZ-2(frag)
NOT31356.1
Planctomycetota bacterium
VanZ-2(frag)
MBI5040229.1
Gammaproteobacteria bacterium
VanZ-2(frag)
OHD33698.1
Spirochaetes bacterium GWC1_61_12
VanZ-2(frag)
MBR1790861.1
Bacteroidaceae bacterium
VanZ-2(frag)
KPL09209.1
candidate division BRC1 bacterium SM23_51
VanZ-2(frag)
WP_143594609.1
VanZ-2(frag)

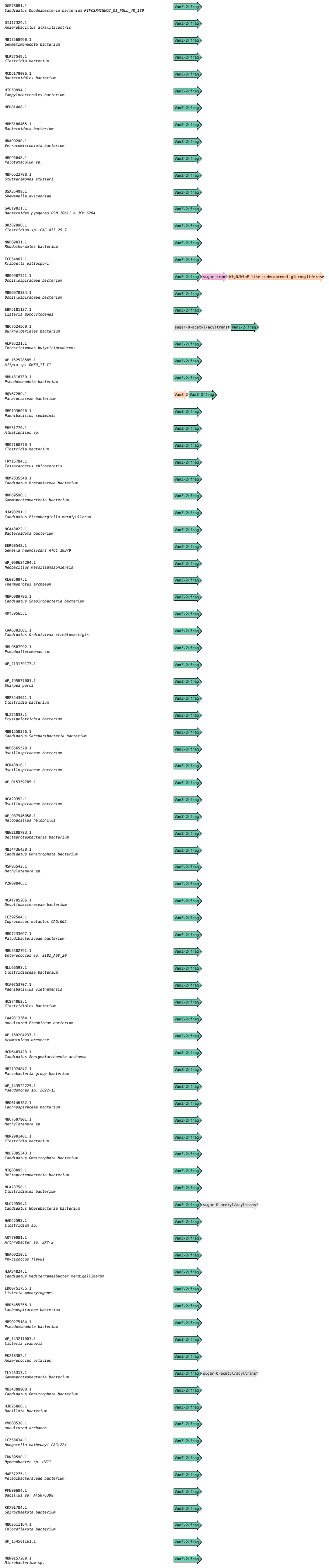

OGE78881.1
Candidatus Doudnabacteria bacterium RIFCSPHIGHO2_01_FULL_48_180
VanZ-2(frag)
OIJ17329.1
Anaerobacillus alkalilacustris
VanZ-2(frag)
MBI3568990.1
Gemmatimonadota bacterium
VanZ-2(frag)
NLP27549.1
Clostridia bacterium
VanZ-2(frag)
MCD6179086.1
Bacteroidales bacterium
VanZ-2(frag)
HIP50994.1
Campylobacterales bacterium
VanZ-2(frag)
HEU81408.1
VanZ-2(frag)
MBM3186465.1
Bacteroidota bacterium
VanZ-2(frag)
NDA09240.1
Verrucomicrobiota bacterium
VanZ-2(frag)
HBC93648.1
Pelotomaculum sp.
VanZ-2(frag)
MBF6622788.1
Stutzerimonas stutzeri
VanZ-2(frag)
QSX35409.1
Shewanella avicenniae
VanZ-2(frag)
GAE19011.1
Bacteroides pyogenes DSM 20611 = JCM 6294
VanZ-2(frag)
OKZ82990.1
Clostridium sp. CAG_433_25_7
VanZ-2(frag)
NNE69831.1
Rhodothermales bacterium
VanZ-2(frag)
TCC54967.1
Kribbella pittospori
VanZ-2(frag)
MBQ9907241.1
Oscillospiraceae bacterium
VanZ-2(frag)
sugar-tranf
WfgD/WfaP-like-undecaprenol-glucosyltferase
MBE6978384.1
Oscillospiraceae bacterium
VanZ-2(frag)
EBF5181137.1
Listeria monocytogenes
VanZ-2(frag)
MBC7624569.1
Burkholderiales bacterium
sugar-O-acetyl/acyltransf
VanZ-2(frag)
ALP95231.1
Intestinimonas butyriciproducens
VanZ-2(frag)
WP_152528585.1
Afipia sp. OHSU_II-C1
VanZ-2(frag)
MBU4316739.1
Pseudomonadota bacterium
VanZ-2(frag)
NQV67260.1
Paracoccaceae bacterium
VanZ-2
VanZ-2(frag)
MBP1936028.1
Paenibacillus sediminis
VanZ-2(frag)
PHS31770.1
Alkaliphilus sp.
VanZ-2(frag)
MBQ7160370.1
Clostridia bacterium
VanZ-2(frag)
TRY16784.1
Tessaracoccus rhinocerotis
VanZ-2(frag)
MBM2835548.1
Candidatus Brocadiaceae bacterium
VanZ-2(frag)
NDH69590.1
Gammaproteobacteria bacterium
VanZ-2(frag)
HJA93291.1
Candidatus Eisenbergiella merdipullorum
VanZ-2(frag)
HCA43022.1
Bacteroidota bacterium
VanZ-2(frag)
EER68548.1
Gemella haemolysans ATCC 10379
VanZ-2(frag)
WP_090634294.1
Neobacillus massiliamazoniensis
VanZ-2(frag)
RLG85097.1
Thermoprotei archaeon
VanZ-2(frag)
MBP6989786.1
Candidatus Shapirobacteria bacterium
VanZ-2(frag)
RKY59565.1
VanZ-2(frag)
KAA6302681.1
Candidatus Ordinivivax streblomastigis
VanZ-2(frag)
MBL0687992.1
Pseudoalteromonas sp.
VanZ-2(frag)
WP_213139177.1
VanZ-2(frag)
WP_195837001.1
Sharpea porci
VanZ-2(frag)
MBP3443041.1
Clostridia bacterium
VanZ-2(frag)
NLZ75833.1
Erysipelotrichia bacterium
VanZ-2(frag)
MBB1558276.1
Candidatus Saccharibacteria bacterium
VanZ-2(frag)
MBE6665529.1
Oscillospiraceae bacterium
VanZ-2(frag)
HCR43918.1
Oscillospiraceae bacterium
VanZ-2(frag)
WP_015359785.1
VanZ-2(frag)
HCA29352.1
Oscillospiraceae bacterium
VanZ-2(frag)
WP_087946058.1
Halobacillus halophilus
VanZ-2(frag)
MBW2108783.1
Deltaproteobacteria bacterium
VanZ-2(frag)
MBI4436430.1
Candidatus Omnitrophota bacterium
VanZ-2(frag)
MSP86542.1
Methylotenera sp.
VanZ-2(frag)
PZN09846.1
VanZ-2(frag)
MCA1795206.1
Desulfobacteraceae bacterium
VanZ-2(frag)
CCZ92504.1
Coprococcus eutactus CAG:665
VanZ-2(frag)
MBO7233697.1
Paludibacteraceae bacterium
VanZ-2(frag)
MBU5582791.1
Enterococcus sp. S181_ASV_20
VanZ-2(frag)
NLL66593.1
Clostridiaceae bacterium
VanZ-2(frag)
MCA0753707.1
Paenibacillus vietnamensis
VanZ-2(frag)
HCS74062.1
Clostridiales bacterium
VanZ-2(frag)
CAA9313364.1
uncultured Frankineae bacterium
VanZ-2(frag)
WP_169204237.1
Aromatoleum bremense
VanZ-2(frag)
MCD6483423.1
Candidatus Aenigmatarchaeota archaeon
VanZ-2(frag)
MBI1974847.1
Parcubacteria group bacterium
VanZ-2(frag)
WP_143522725.1
Pseudomonas sp. 2822-15
VanZ-2(frag)
MBO6146782.1
Lachnospiraceae bacterium
VanZ-2(frag)
MBC7697901.1
Methylotenera sp.
VanZ-2(frag)
MBR2901401.1
Clostridia bacterium
VanZ-2(frag)
MBL7085343.1
Candidatus Omnitrophota bacterium
VanZ-2(frag)
NIQ88895.1
Deltaproteobacteria bacterium
VanZ-2(frag)
NLA73758.1
Clostridiales bacterium
VanZ-2(frag)
RLC29556.1
Candidatus Woesebacteria bacterium
VanZ-2(frag)
sugar-O-acetyl/acyltransf
HAK42598.1
Clostridium sp.
VanZ-2(frag)
AOY70081.1
Arthrobacter sp. ZXY-2
VanZ-2(frag)
NHA69210.1
Phycicoccus flavus
VanZ-2(frag)
HJA34824.1
Candidatus Mediterraneibacter merdigallinarum
VanZ-2(frag)
EDO0751755.1
Listeria monocytogenes
VanZ-2(frag)
MBD5455356.1
Lachnospiraceae bacterium
VanZ-2(frag)
MBS0275184.1
Pseudomonadota bacterium
VanZ-2(frag)
WP_143211003.1
Listeria ivanovii
VanZ-2(frag)
PKZ16382.1
Anaerococcus octavius
VanZ-2(frag)
TLY45313.1
Gammaproteobacteria bacterium
VanZ-2(frag)
sugar-O-acetyl/acyltransf
MBI4308900.1
Candidatus Omnitrophota bacterium
VanZ-2(frag)
HJB26868.1
Bacillota bacterium
VanZ-2(frag)
VVB88538.1
uncultured archaeon
VanZ-2(frag)
CCZ58634.1
Hungatella hathewayi CAG:224
VanZ-2(frag)
TDN39590.1
Hymenobacter sp. UV11
VanZ-2(frag)
MAE37275.1
Pelagibacteraceae bacterium
VanZ-2(frag)
PFN80604.1
Bacillus sp. AFS076308
VanZ-2(frag)
RKX91784.1
Spirochaetota bacterium
VanZ-2(frag)
MBU2611164.1
Chloroflexota bacterium
VanZ-2(frag)
WP_154581161.1
VanZ-2(frag)
MBN9157289.1
Microbacterium sp.
VanZ-2(frag)

Accession	Organism	Genes
OPZ94790.1	*Firmicutes bacterium ADurb.Bin419*	VanZ-2(frag
HAM1618252.1	*Listeria monocytogenes*	VanZ-2(frag
MBM3186948.1	*Bacteroidota bacterium*	VanZ-2(frag
MBL8362329.1	*Rubrivivax sp.*	VanZ-2(frag
HBZ49822.1	*Halieaceae bacterium*	VanZ-2(frag
MCB9247515.1	*Ignavibacteriales bacterium*	VanZ-2(frag
MBQ2891830.1	*Bacilli bacterium*	VanZ-2(frag
PJO45056.1	*Lysinibacillus xylanilyticus*	VanZ-2(frag
MBU2577595.1	*Patescibacteria group bacterium*	VanZ-2(frag, GalE/RmlB/Gmd-like_lyase/epimerase
PPC94682.1	*Methylotenera sp.*	VanZ-2(frag
MBQ0140643.1	*Candidatus Kurthia equi*	VanZ-2(frag
GEO63407.1	*Companilactobacillus nantensis*	VanZ-2(frag
WP_163540081.1	*Gracilibacillus sp. YIM 98692*	VanZ-2(frag
MBX9720207.1	*Candidatus Obscuribacterales bacterium*	VanZ-2(frag
EUJ37898.1	*Brochothrix thermosphacta DSM 20171 = FSL F6-1036*	VanZ-2(frag
OGR96173.1	*Elusimicrobia bacterium RIFCSPLOWO2_01_FULL_64_13*	VanZ-2(frag
MBR1533881.1	*Ruminococcus sp.*	VanZ-2(frag
MBO6038443.1	*Acidaminococcaceae bacterium*	VanZ-2(frag
HHY74732.1	*Bacillus sp. (in: firmicutes)*	VanZ-2(frag
MBE6352910.1	*Treponema bryantii*	VanZ-2(frag
KRN38790.1	*Lactiplantibacillus plantarum*	VanZ-2(frag
MBT7527846.1	*Rhodospirillales bacterium*	VanZ-2(frag
MBR0456912.1	*Bacillota bacterium*	VanZ-2(frag
WP_233403514.1	*Bacillus halotolerans*	VanZ-2(frag
RMG98046.1	*Candidatus Dadabacteria bacterium*	VanZ-2(frag
MBQ4604930.1	*Clostridia bacterium*	VanZ-2(frag
HGF27442.1		VanZ-2(frag
NNE43630.1	*Gemmatimonadota bacterium*	VanZ-2(frag
MBI3006288.1	*Ignavibacteriales bacterium*	VanZ-2(frag
MBL0067099.1	*Chitinophagaceae bacterium*	VanZ-2(frag
RJP52189.1	*Anaerolineaceae bacterium*	VanZ-2(frag
MCB1893077.1	*Rhodocyclaceae bacterium*	VanZ-2(frag
MBQ6894344.1	*Clostridia bacterium*	VanZ-2(frag
AXA35590.1	*Candidatus Sumerlaea chitinivorans*	VanZ-2(frag
MBO4782742.1	*Lachnospiraceae bacterium*	VanZ-2(frag
MBI4218871.1	*Chloroflexota bacterium*	VanZ-2(frag
MBP8065962.1	*Flavobacterium sp.*	VanZ-2(frag, ?, TonB-C, OmpA-like-beta-barrel
MBQ9042808.1	*Eggerthellaceae bacterium*	VanZ-2(frag
MBP7897238.1	*Gemmiger sp.*	VanZ-2(frag
HBG20169.1	*Desulfobulbaceae bacterium*	VanZ-2(frag
HHY42101.1	*Thermoanaerobacterales bacterium*	VanZ-2(frag
EHZ8140203.1	*Listeria innocua*	VanZ-2(frag
WP_223443389.1	*Metabacillus dongyingensis*	VanZ-2(frag
NIW80004.1	*Calditrichia bacterium*	VanZ-2(frag
MCC6353224.1	*Verrucomicrobiia bacterium*	VanZ-2(frag
SHE23773.1	*methanotrophic endosymbiont of Bathymodiolus puteoserpentis (Logatchev)*	VanZ-2(frag
TAN62501.1	*bacterium*	VanZ-2(frag
HBG13540.1	*Clostridium sp.*	VanZ-2(frag
MBQ1935757.1	*Clostridia bacterium*	VanZ-2(frag
NLM12025.1	*Clostridiaceae bacterium*	VanZ-2(frag
MBQ1878076.1	*Erysipelotrichaceae bacterium*	VanZ-2(frag
MBO7376468.1	*Clostridia bacterium*	VanZ-2(frag
EAG4133268.1	*Listeria monocytogenes*	VanZ-2(frag
MBF0951856.1	*Alloprevotella tannerae*	VanZ-2(frag
MBP3351958.1	*Lachnospiraceae bacterium*	VanZ-2(frag
TSA66015.1	*Sediminibacterium sp.*	VanZ-2(frag
HDO77752.1	*Actinomycetota bacterium*	VanZ-2
HDO63329.1	*Methanomicrobia archaeon*	VanZ-2
DAJ01095.1		VanZ-2
POE64844.1		?, VanZ-2

######;Some VanZ-like sequences defy easy characterization, based on sequence analysis, as either VanZ-1 or VanZ-2. At the same time, there is little evidence to support the establishment of a third, monophyletic family. The sequences below are of interest due to their contextual associations; however, their precise affinities are unclear.; ####;VanY associations

Accession	Organism	Genes
SFR92433.1		VanY, VanZ-u
WP_168908164.1	*Paenibacillus albicereus*	VanY, VanZ-u
WP_192703613.1	*Paenibacillus sp. OAS669*	VanY, VanZ-u
WP_066367266.1	*Bacillus sp. FJAT-27264*	VanY, VanZ-u
WP_214718356.1	*Bacillus sp. ISL-40*	VanY, VanZ-u
WP_116188895.1	*Paenibacillus taihuensis*	VanZ-u, VanY
MBD3783651.1	*Micrococcales bacterium*	VanY, VanZ-u
WP_129519727.1		VanY, VanZ-u

#;association with other vancomycin-resistance genes

Accession	Organism	Genes
MBN9139290.1	*Micrococcales bacterium*	VanH-like, VanA/VanG, VanZ-u

####;Calcineurin domain association

Accession	Organism	Genes
MBQ4111607.1	*Clostridia bacterium*	VanZ-u, disordered, Calcineurin
MBP3665517.1	*Clostridia bacterium*	VanZ-u, Calcineurin
MBE6586709.1	*Oscillospiraceae bacterium*	VanZ-u, Calcineurin
MBQ3507311.1	*Clostridia bacterium*	VanZ-u, Calcineurin
MBR6782413.1	*Clostridia bacterium*	VanZ-u, Calcineurin
MBR6782505.1	*Clostridia bacterium*	VanZ-u, Calcineurin
MBQ7910327.1	*Clostridia bacterium*	VanZ-u, Calcineurin
MBR5124198.1	*Clostridia bacterium*	VanZ-u, Calcineurin
MBS7224669.1	*Clostridiaceae bacterium*	VanZ-u, Calcineurin
MBD8986896.1	*Clostridiales bacterium*	VanZ-u, Calcineurin
MBE6652470.1	*Oscillospiraceae bacterium*	VanZ-u, Calcineurin
MBR3866968.1	*Butyricicoccus sp.*	Calcineurin, ?, VanZ-u
MBQ9779601.1	*Clostridia bacterium*	VanZ-u, Calcineurin
MBP3312604.1	*Butyricicoccus sp.*	VanZ-u, Calcineurin
MBE6557741.1	*Oscillospiraceae bacterium*	VanZ-u, Calcineurin

###################;Wok-DUF2238 associations; ###Schlafen fusion (sometimes the Wok is inactive)

Accession	Organism	Genes
ETA68262.1	*Methanolobus tindarius DSM 2278*	Wok-DUF2238, Schlafen
MBU2576944.1	*Nanoarchaeota archaeon*	Wok-DUF2238, Schlafen
MAG24016.1	*Candidatus Pacearchaeota archaeon*	Wok-DUF2238, Schlafen
MSS74459.1	*Candidatus Pacearchaeota archaeon*	Wok-DUF2238, Schlafen
PIN94654.1	*Candidatus Pacearchaeota archaeon CG10_big_fil_rev_8_21_14_0_10_30_48*	Wok-DUF2238, Schlafen

RMD66190.1
Candidatus Pacearchaeota archaeon
Wok-DUF2238 Schlafen

MAG07701.1
Candidatus Pacearchaeota archaeon
Wok-DUF2238 Schlafen

MBS3067016.1
Candidatus Pacearchaeota archaeon
Wok-DUF2238 Schlafen

MBM3231807.1
Candidatus Pacearchaeota archaeon
Wok-DUF2238 Schlafen

MAG38222.1
Candidatus Pacearchaeota archaeon
Wok-DUF2238 Schlafen

WP_172361051.1
Wok-DUF2238 Schlafen

MBS3071365.1
Candidatus Pacearchaeota archaeon
Wok-DUF2238 Schlafen

MBT7102568.1
archaeon
Wok-DUF2238 Schlafen

MBS3082295.1
Candidatus Pacearchaeota archaeon
Wok-DUF2238 Schlafen

RJQ29329.1
Candidatus Parcubacteria bacterium
Schlafen Wok-DUF2238

NLF33478.1
Thermoplasmatales archaeon
Wok-DUF2238-i Schlafen

OPY31377.1
Methanomassiliicoccales archaeon PtaU1.Bin124
Wok-DUF2238-i Schlafen

MBS3065874.1
Candidatus Pacearchaeota archaeon
Wok-DUF2238-i Schlafen

HIG51900.1
Candidatus Pacearchaeota archaeon
Wok-DUF2238-i Schlafen

MBS3076040.1
Candidatus Pacearchaeota archaeon
Wok-DUF2238-i Schlafen

MBR9706537.1
Candidatus Pacearchaeota archaeon
Wok-DUF2238-i Schlafen

MBS3079082.1
Candidatus Pacearchaeota archaeon
Wok-DUF2238-i Schlafen

MBL7053569.1
Candidatus Portnoybacteria bacterium
Wok-DUF2238-i Schlafen

MBT4135243.1
archaeon
Wok-DUF2238-i Schlafen

MBU2053337.1
Nanoarchaeota archaeon
Wok-DUF2238-i Schlafen

MAG10719.1
Candidatus Pacearchaeota archaeon
Wok-DUF2238-i Schlafen

MBU2104262.1
Nanoarchaeota archaeon
Wok-DUF2238-i Schlafen

MBT4376662.1
archaeon
Wok-DUF2238-i Schlafen

PJC45383.1
Candidatus Pacearchaeota archaeon CG_4_9_14_0_2_um_filter_30_8
Wok-DUF2238-i Schlafen

YdjM associations, often accompanied by a second, inactive copy of the Lipocone

WP_008526393.1
Halorhabdus tiamatea SARL4B
YdjM-hydrolase Wok-DUF2238

WP_153550183.1
Candidatus Nanohalobium constans
Calcineurin YdjM-hydrolase Wok-DUF2238-i Wok-DUF2238

WP_006076767.1
Halococcus saccharolyticus DSM 5350
YdjM-hydrolase Wok-DUF2238 Wok-DUF2238-i

ELY58995.1
Natronolimnohabitans innermongolicus JCM 12255
YdjM-hydrolase Wok-DUF2238-i Wok-DUF2238

EMA37356.1
Halobiforma nitratireducens JCM 10879
YdjM-hydrolase Wok-DUF2238-i Wok-DUF2238

ELY56171.1
Natronococcus amylolyticus DSM 10524
Wok-DUF2238-i Wok-DUF2238

AUX08480.1
Halalkaliarchaeum desulfuricum
YdjM-hydrolase Wok-DUF2238-i Wok-DUF2238

RLE39227.1
Candidatus Woesearchaeota archaeon
Wok-DUF2238 YdjM-hydrolase

EJN59115.1
Halogranum salarium B-1
Wok-DUF2238-i Wok-DUF2238

SFR62914.1
Halogeometricum rufum
YdjM-hydrolase Wok-DUF2238

PSP61336.1
Halobacteriales archaeon QH_8_64_26
YdjM-hydrolase PSE Wok-DUF2238

WP_225935269.1
Halobaculum roseum
YdjM-hydrolase Wok-DUF2238

WP_179259880.1
Natrinema halophilum
YdjM-hydrolase Wok-DUF2238

WP_012795185.1
Halorhabdus utahensis DSM 12940
YdjM-hydrolase Wok-DUF2238

PSQ36991.1
Halobacteriales archaeon SW_12_69_24
YdjM-hydrolase PSE Wok-DUF2238

WP_144261909.1
Haloglomus irregulare
YdjM-hydrolase Wok-DUF2238-i Wok-DUF2238

WP_210408892.1
Halorhabdus rudnickae
Wok-DUF2238-i Wok-DUF2238 YdjM-hydrolase

WP_006078157.1
Halococcus saccharolyticus DSM 5350
YdjM-hydrolase Wok-DUF2238-i Wok-DUF2238

WP_152942876.1
Natronorubrum aibiense
YdjM-hydrolase Wok-DUF2238-i Wok-DUF2238

WP_230892819.1
Halobacterium sp. KA-6
YdjM-hydrolase Wok-DUF2238-i Wok-DUF2238

HII89315.1
Candidatus Woesearchaeota archaeon
YdjM-hydrolase Wok-DUF2238

WP_049893779.1
Salinarchaeum sp. Harcht-Bsk1
YdjM-hydrolase Wok-DUF2238-i Wok-DUF2238

QKQ98409.1
Candidatus Nanohaloarchaea archaeon
Wok-DUF2238-i Wok-DUF2238 YdjM-hydrolase

AGB16092.1
Halovivax ruber XH-70
YdjM-hydrolase Wok-DUF2238-i Wok-DUF2238

WP_020221326.1
Halarchaeum acidiphilum MH1-52-1
Wok-DUF2238-i Wok-DUF2238

WP_014039924.1
Haloarcula sp. KBTZ06
YdjM-hydrolase Wok-DUF2238-i Wok-DUF2238

WP_089817340.1
Halomicrobium zhouii
YdjM-hydrolase Wok-DUF2238-i Wok-DUF2238

WP_013199415.1
Halalkalicoccus jeotgali B3
Wok-DUF2238-i Wok-DUF2238 YdjM-hydrolase

WP_162315909.1
Haloarcula limicola
YdjM-hydrolase Wok-DUF2238-i Wok-DUF2238

WP_066380923.1
Halalkalicoccus paucihalophilus
Wok-DUF2238-i Wok-DUF2238 YdjM-hydrolase

WP_159762221.1
Halovenus carboxidivorans
YdjM-hydrolase Wok-DUF2238-i Wok-DUF2238

WP_049993684.1
Halapricum salinum
YdjM-hydrolase Wok-DUF2238

WP_158056442.1
Halorussus halophilus
YdjM-hydrolase Wok-DUF2238-i Wok-DUF2238

WP_114578091.1
Saliphagus infecundisoli
YdjM-hydrolase Wok-DUF2238-i Wok-DUF2238

WP_158204049.1
YdjM-hydrolase Wok-DUF2238

ELZ34039.1
Halogeometricum pallidum JCM 14848
YdjM-hydrolase Wok-DUF2238

WP_145848451.1
Salarchaeum sp. JOR-1
YdjM-hydrolase Wok-DUF2238-i Wok-DUF2238

WP_023393198.1
Candidatus Halobonum tyrrellensis G22
YdjM-hydrolase Wok-DUF2238

WP_200899149.1
Halanaeroarchaeum sulfurireducens
YdjM-hydrolase Wok-DUF2238

PSQ06382.1
Halobacteriales archaeon QS_5_68_33
YdjM-hydrolase ? PSE PSE Wok-DUF2238

WP_233560590.1
Halobellus sp. Atlit-38R
YdjM-hydrolase Wok-DUF2238

WP_089732386.1
Haloarchaeobius iranensis
YdjM-hydrolase Wok-DUF2238

WP_232571429.1
Halobacterium litoreum
YdjM-hydrolase Wok-DUF2238

WP_170216850.1
Halobellus limi
YdjM-hydrolase Wok-DUF2238

WP_227373560.1
Haladaptatus halobius
YdjM-hydrolase Wok-DUF2238 Wok-DUF2238-i

WP_226013402.1
Halomicrobium salinisoli
YdjM-hydrolase Wok-DUF2238

WP_079233474.1
Halolamina sp. CBA1230
YdjM-hydrolase Wok-DUF2238

GGN92048.1
Haloarcula pellucida
YdjM-hydrolase ? Wok-DUF2238

WP_053948782.1
Halolamina sediminis
YdjM-hydrolase Wok-DUF2238

WP_153555050.1
Halomicrobium sp. LC1Hm
YdjM-hydrolase ? Wok-DUF2238

WP_170092908.1
Halomicrobium mukohataei
YdjM-hydrolase ? Wok-DUF2238

WP_121823036.1
Halostella salina
YdjM-hydrolase ? Wok-DUF2238

WP_070364327.1
Halodesulfurarchaeum formicicum
YdjM-hydrolase Wok-DUF2238

MBU2104294.1
Nanoarchaeota archaeon
Wok-DUF2238 YdjM-hydrolase

MAG11168.1
Candidatus Pacearchaeota archaeon
Wok-DUF2238 YdjM-hydrolase

WP_122105250.1
Halorubrum glutamatedens
Wok-DUF2238-i Wok-DUF2238

WP_224448518.1
Haloprofundus salilacus
YdjM-hydrolase Wok-DUF2238

SFG62632.1
Halopelagius inordinatus
YdjM-hydrolase Wok-DUF2238

WP_157511272.1
YdjM-hydrolase Wok-DUF2238-i Wok-DUF2238

WP_166042654.1
Halorubellus sp. JP-L1
YdjM-hydrolase Wok-DUF2238

MBI1970802.1
Candidatus Woesearchaeota archaeon
YdjM-hydrolase Wok-DUF2238

WP_154550925.1
Halorhabdus sp. CBA1104
YdjM-hydrolase Wok-DUF2238

MBS3760721.1
Halodesulfurarchaeum sp.
YdjM-hydrolase Wok-DUF2238

WP_071932531.1
Halodesulfurarchaeum formicicum
YdjM-hydrolase Wok-DUF2238

MBN1456205.1
Methanomicrobia archaeon
Wok-DUF2238 YdjM-hydrolase

WP_220582483.1
YdjM-hydrolase Wok-DUF2238

WP_145845737.1
Salarchaeum sp. JOR-1
YdjM-hydrolase Wok-DUF2238

######;fusion to MTase-TYW3

MCD6146998.1	*Thermoplasmata archaeon*	Wok-DUF2238 MTase-TYW3
HDS59825.1	*Thermoplasmatales archaeon*	Wok-DUF2238 MTase-TYW3
MCD6172083.1	*Thermoplasmata archaeon*	Wok-DUF2238 MTase-TYW3
MCD6474517.1	*Thermoplasmata archaeon*	Wok-DUF2238 MTase-TYW3
MCD6222157.1	*Thermoplasmata archaeon*	Wok-DUF2238 MTase-TYW3
HHF56638.1	*Thermoplasmatales archaeon*	Wok-DUF2238 MTase-TYW3

###;rhomboid adjacent

MBE6062438.1	*Clostridium butyricum*	Rhomboid Wok-DUF2238
KJZ82704.1	*Clostridium sp. IBUN22A*	Rhomboid Wok-DUF2238
WP_195264872.1	*Clostridium sp. DSM 100503*	Rhomboid Wok-DUF2238
NFO11782.1	*Clostridium botulinum*	Rhomboid Wok-DUF2238
MBQ6819892.1	*Clostridium sp.*	Rhomboid Wok-DUF2238
WP_219777683.1	*Clostridium weizhouense*	Rhomboid Wok-DUF2238
WP_069679028.1	*Clostridium taeniosporum*	Rhomboid Wok-DUF2238
WP_195343337.1	*Clostridium paraputrificum*	Rhomboid Wok-DUF2238
WP_209796157.1	*Clostridium moniliforme*	Rhomboid Wok-DUF2238
WP_194192271.1	*Clostridium chrysemydis*	Rhomboid Wok-DUF2238
NLK23525.1	*Clostridiales bacterium*	Rhomboid Wok-DUF2238
WP_090014486.1	*Clostridium sp. DSM 8431*	Rhomboid Wok-DUF2238
WP_195467792.1	*Clostridium sp. D43t1_170807_H7*	?? Rhomboid DUF2388
MBS5948867.1	*Clostridium sp.*	Rhomboid Wok-DUF2238
SUQ50597.1	*Clostridium neonatale*	Rhomboid Wok-DUF2238
ATD54587.1	*Clostridium chauvoei*	Rhomboid Wok-DUF2238
RPJ27926.1	*Chloroflexota bacterium*	GT4 Wok-DUF2238 ? Rhomboid
NLK96153.1	*Clostridiales bacterium*	Rhomboid Wok-DUF2238
MBN1384207.1	*Elusimicrobiota bacterium*	Rhomboid Wok-DUF2238
MCD6367773.1	*Candidatus Aenigmatarchaeota archaeon*	Rhomboid ? Wok-DUF2238(frag)

############;patatin, rare

HIW25486.1	*Bacillota bacterium*	Patatin DUF6363 Wok-DUF2238
MBO5375366.1	*Bacilli bacterium*	Wok-DUF2238 Patatin DUF6363
SHI61626.1	*Thermoclostridium caenicola*	Patatin Wok-DUF2238
MBI2397074.1	*Lysobacterales bacterium*	Patatin Wok-DUF2238
MBK6726967.1	*Lysobacterales bacterium*	Patatin Wok-DUF2238
MBR5232077.1	*Clostridia bacterium*	Patatin DUF6363 ? Wok-DUF2238
MBQ1945919.1	*Clostridia bacterium*	Patatin DUF6363 ? Wok-DUF2238
MCD6212195.1	*Sulfurovum sp.*	Patatin Wok-DUF2238

#######;Synaptojanin-like associations

WP_204458820.1	*Agromyces cerinus subsp. nitratus*	Synaptojanin-like Wok-DUF2238
WP_083391810.1	*Microbacterium sp. LCT-H2*	GTA Synaptojanin-like Wok-DUF2238

#;comes with GTA

WP_136030637.1	*Microbacterium sp. PF5*	GTA Synaptojanin-like Wok-DUF2238
WP_205781281.1	*Microbacterium sp. Gd 4-13*	GTA Synaptojanin-like Wok-DUF2238
WP_121057382.1	*Microbacterium telephonicum*	GTA Synaptojanin-like Wok-DUF2238 GT4
WP_230111832.1	*Microbacterium foliorum*	GTA Synaptojanin-like Wok-DUF2238
WP_135118723.1	*Leifsonia flava*	Synaptojanin-like Wok-DUF2238 GTA ABHydrolase
WP_023951930.1	*Microbacterium paludicola*	GTA Synaptojanin-like Wok-DUF2238
WP_055820445.1	*Leifsonia sp. Root60*	Synaptojanin-like Wok-DUF2238 GTA ABHydrolase
WP_207169359.1	*Nesterenkonia sp. E16_10*	GTA Synaptojanin-like Wok-DUF2238
WP_139200475.1	*Curtobacterium sp. MMLR14_010*	GTA Synaptojanin-like Wok-DUF2238
WP_067876671.1	*Agromyces aureus*	Synaptojanin-like Wok-DUF2238 GTA
WP_096223471.1	*Glutamicibacter sp. BW78*	Synaptojanin-like Wok-DUF2238 GTA
WP_058724553.1	*Curtobacterium luteum*	GTA Synaptojanin-like Wok-DUF2238
WP_224778715.1	*Leucobacter sp. Psy1*	GTA Synaptojanin-like Wok-DUF2238
WP_092504809.1	*Agrococcus jejuensis*	GTA Synaptojanin-like Wok-DUF2238
WP_192594683.1	*Nesterenkonia lutea*	GTA Synaptojanin-like Wok-DUF2238
WP_111106050.1	*Curtobacterium sp. MCLR17_007*	GTA Synaptojanin-like Wok-DUF2238
WP_136362705.1	*Microbacterium oleivorans*	GTA Synaptojanin-like Wok-DUF2238
WP_183357111.1	*Garicola koreensis*	GTA Synaptojanin-like Wok-DUF2238
WP_133542269.1	*Microbacterium sp. BK668*	GTA Synaptojanin-like Wok-DUF2238
SBN61571.1	*Curtobacterium sp. 9128*	ABHydrolase GTA Synaptojanin-like Wok-DUF2238
WP_141399258.1	*Curtobacterium sp. 'Ferrero'*	GTA Synaptojanin-like Wok-DUF2238
SDO62110.1	*Microbacterium sp. ru370.1*	GTA Synaptojanin-like Wok-DUF2238
WP_076703747.1	*Tersicoccus phoenicis*	ABHydrolase ? ? ? GTA Synaptojanin-like Wok-DUF2238
WP_215485930.1	*Microbacterium flavum*	GTA Synaptojanin-like Wok-DUF2238
WP_161449795.1	*Glutamicibacter soli*	GTA Synaptojanin-like Wok-DUF2238
WP_147037710.1	*Microbacterium aerolatum*	GTA Synaptojanin-like Wok-DUF2238
WP_019178853.1	*Microbacterium yannicii PS01*	GTA Synaptojanin-like Wok-DUF2238
WP_092960989.1	*Falsiroseomonas stagni DSM 19981*	Calcineurin ? Wok-DUF2238 Synaptojanin-like
WP_147050852.1	*Microbacterium saccharophilum*	GTA Synaptojanin-like Wok-DUF2238

#;with GNAT

WP_207455547.1	*Herbiconiux sp. SYSU D00978*	GNAT Synaptojanin-like Wok-DUF2238

#;NUDIX instead of GTA?

WP_127049513.1	*Labedella endophytica*	NUDIX Synaptojanin-like Wok-DUF2238

#;just Synaptojanin-like

OLE17557.1	*Actinobacteria bacterium 13_1_20CM_4_68_12*	Synaptojanin-like Wok-DUF2238 Wok-DUF2238-i
OUP77991.1	*Erysipelatoclostridium sp. An173*	Wok-DUF2238 Synaptojanin-like
WP_218463591.1	*Maritimibacter sp. DP1N21-5*	Synaptojanin-like Wok-DUF2238
WP_224821981.1	*Pseudooceanicola nanhaiensis*	Synaptojanin-like Wok-DUF2238
MBY0350149.1	*Tabrizicola sp.*	Synaptojanin-like Wok-DUF2238
WP_161352014.1	*Maritimibacter harenae*	Synaptojanin-like Wok-DUF2238
WP_198916893.1	*Palleronia pontilimi*	Synaptojanin-like Wok-DUF2238
MBU0470215.1	*Nanoarchaeota archaeon*	Wok-DUF2238 ? Synaptojanin-like
NKB38710.1	*Gammaproteobacteria bacterium*	Synaptojanin-like ? Wok-DUF2238
MBS3165750.1	*Candidatus Woesearchaeota archaeon*	Synaptojanin-like Wok-DUF2238
HHX82564.1	*Pseudomonadaceae bacterium*	Wok-DUF2238 Synaptojanin-like
CUI97503.1	*Cognatishimia activa*	Synaptojanin-like Wok-DUF2238
WP_116085682.1	*Tropicimonas sp. IMCC34011*	Synaptojanin-like Wok-DUF2238
MAM71007.1	*Gammaproteobacteria bacterium*	Wok-DUF2238 Synaptojanin-like
WP_007256319.1	*Oceanicola granulosus HTCC2516*	Synaptojanin-like Wok-DUF2238
QVL45522.1	*Methylophilaceae bacterium*	Wok-DUF2238 ? Synaptojanin-like
WP_224503914.1	*Celeribacter litoreus*	Synaptojanin-like Wok-DUF2238
OGT66390.1	*Gammaproteobacteria bacterium RIFCSPLOWO2_01_FULL_47_190*	Wok-DUF2238 Synaptojanin-like
WP_111366667.1	*Aquicoccus porphyridii*	Synaptojanin-like Wok-DUF2238
WP_085847839.1	*Pacificibacter marinus*	Synaptojanin-like Wok-DUF2238
MBR2573703.1	*Loktanella sp.*	Synaptojanin-like Wok-DUF2238

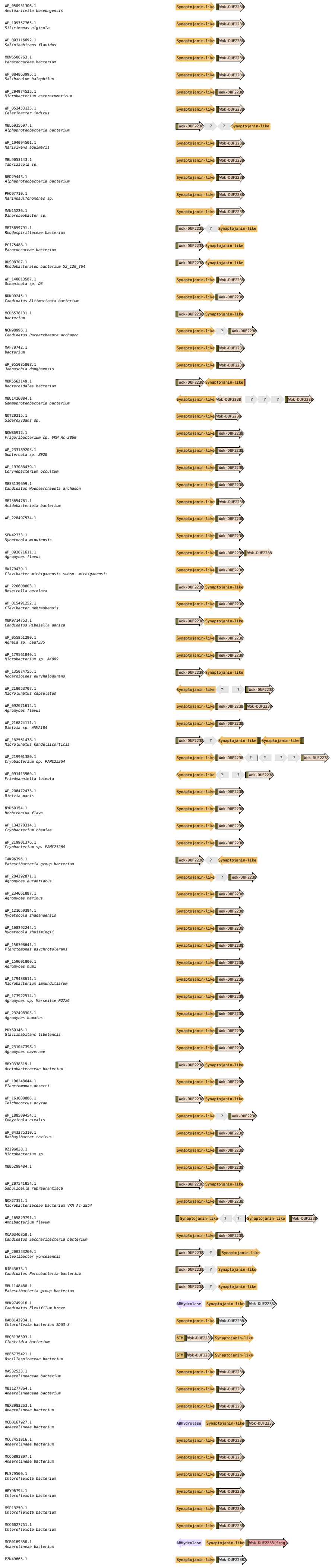

#########;NUDIX and carotenoid biosynthesis module association

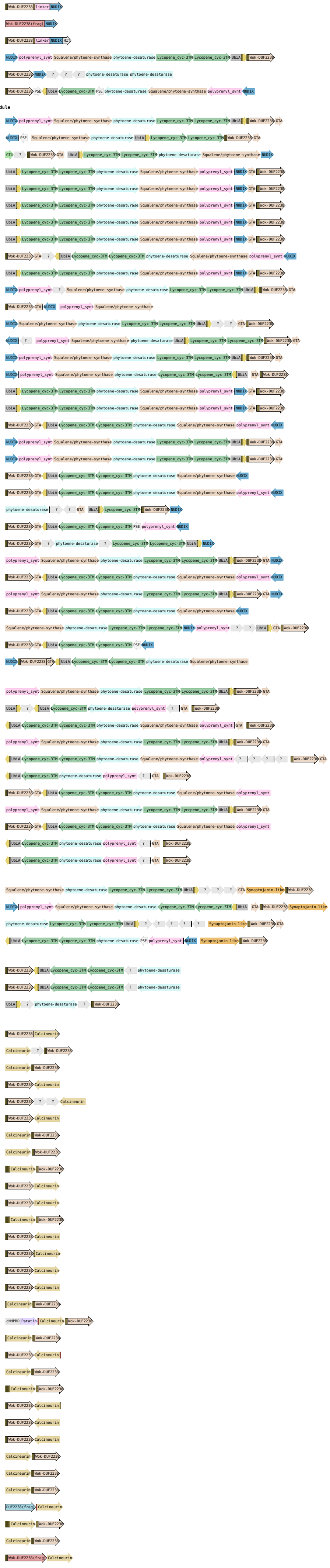

HBB61962.1
Lachnospiraceae bacterium

NLH94775.1
Synergistaceae bacterium

WP_160642928.1
Neglectibacter sp. X4

WP_137772835.1
Citricoccus sp. SGAir0253

WP_198665169.1
Tropicimonas sp. IMCC34011

WP_181753540.1
Aeromicrobium phoceense

#;NUDIX and GTA and adjacent and carotenoid biosynthesis module

NUL46519.1
Cellulosimicrobium funkei

WP_227718647.1
Micrococcus sp. FDAARGOS_333

GGD30957.1
Nocardioides daphniae

WP_227902384.1
Arthrobacter sp. YD2

WP_170290149.1
Arthrobacter gandavensis

WP_055239256.1
Arthrobacter sp. Edens01

CEA08968.1
Arthrobacter saudimassiliensis

WP_066296224.1
Arthrobacter luteolus

WP_154795054.1
Occultella kanbiaonis

WP_229964806.1
Arthrobacter sp. zg-Y820

WP_156249983.1
Pseudactinotalea terrae

WP_221652514.1
Actinotalea ferrariae

WP_146340242.1
Nesterenkonia sp. NBAIMH1

WP_230297523.1
Micrococcus luteus

WP_010143016.1
Citricoccus zhacaiensis

WP_051299141.1
Arthrobacter castelli DSM 16402

WP_019481477.1
Arthrobacter sp. Bz4

WP_171583889.1
Arthrobacter sp. 260

WP_207949895.1
Nocardioides ochotonae

MBD8062467.1
Oceanitalea stevensii

WP_139071948.1
Georgenia wutianyii

WP_124237621.1
Aeromicrobium camelliae

TWD14513.1
Marihabitans asiaticum

WP_168582955.1
Gephyromycinifex aptenodytis

WP_111250248.1

WP_204142491.1
Janibacter endophyticus

WP_143784725.1
Ornithinimicrobium ciconiae

WP_199286825.1
Nocardioides houyundeii

WP_141817082.1
Ornithinimicrobium humiphilum

WP_193638541.1
Nocardioides malaquae

WP_159458051.1
Brachybacterium nesterenkovii

QCX29217.1
Nocardioides jishulii

WP_213553288.1
Nocardioides faecalis

#;just GTA with carotenoid biosynthesis module

WP_205754370.1
Arthrobacter sp. CAU 1506

WP_157571289.1
Kocuria rosea subsp. polaris

WP_127573323.1
Georgenia faecalis

WP_152815960.1
Georgenia subflava

WP_125775666.1
Antribacter gilvus

GGG52858.1
Kocuria dechangensis

WP_231245222.1
Nocardioides sp. cx-173

WP_126039930.1
Flaviflexus salsibiostraticola

WP_106849060.1
Blastococcus sp. Marseille-P5729

WP_062736185.1
Kocuria turfanensis

WP_143709841.1
Kocuria flava

#;with Synaptojanin-like-like and GTA

WP_092666355.1
Agrococcus carbonis

WP_051441898.1
Arthrobacter sp. H14

MBM6620675.1
Micrococcaceae bacterium RIT 802

WP_128497480.1
Labedella populi

#;just carotenoid biosynthesis module

WP_156188060.1
Arsenicicoccus sp. oral taxon 190

WP_029210760.1
Arsenicicoccus bolidensis

MBP7832676.1
Candidatus Levyibacteriota bacterium

##########;calcineurin association

KUK59775.1
Methanoculleus marisnigri

MCC6095415.1
Eubacterium sp.

MCD8356697.1
Clostridia bacterium

MBS7403162.1
Oscillospiraceae bacterium

MBQ8357985.1
Clostridia bacterium

MBP8639924.1
Oscillospiraceae bacterium

HIR76785.1
Candidatus Choladousia intestinipullorum

MBR2459783.1
Clostridia bacterium

WP_199222872.1
Rhodohalobacter mucosus

SCL74929.1
Methanoculleus chikugoensis

MBI1968476.1
Candidatus Woesearchaeota archaeon

OQY66018.1
Polyangiaceae bacterium UTPRO1

MBS3148577.1
Candidatus Woesearchaeota archaeon

WP_128694472.1
Methanoculleus taiwanensis

PKL56936.1
Methanomicrobiales archaeon HGW-Methanomicrobiales-6

WP_014372611.1
Paenibacillus mucilaginosus

HCX63651.1
Eubacteriaceae bacterium

WP_150863411.1
Marinihelvus fidelis

MBP2649085.1
Gemmatimonadota bacterium

WP_004037788.1
Methanofollis liminatans DSM 4140

MBN2311918.1
Candidatus Hydrogenedentota bacterium

MCC6766288.1
Deltaproteobacteria bacterium

OPY43724.1
Methanoregulaceae archaeon PtaU1.Bin059

WP_081890480.1
Paenibacillus tyrfis

MBO5797844.1
Clostridia bacterium

MBR3768502.1
Clostridia bacterium

WP_190613958.1
Tepiditoga spiralis

OQA66041.1
Firmicutes bacterium ADurb.Bin262

NMA87602.1
Methanoculleus bourgensis

WP_019892043.1
Allobaculum stercoricanis DSM 13633

MRR07162.1
Deltaproteobacteria bacterium

MBP7411724.1
Methanoculleus sp.

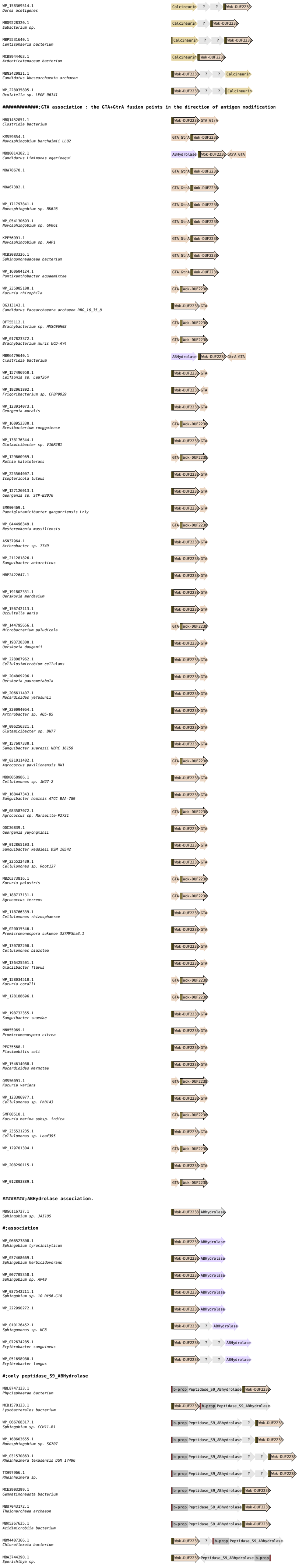
WP_158369514.1 Dorea acetigenes | Calcineurin ? ? Wok-DUF2238
MBQ9228320.1 Eubacterium sp. | Calcineurin ? Wok-DUF2238
MBP5531640.1 Lentisphaeria bacterium | Calcineurin ? ? Wok-DUF2238
MCB8944463.1 Ardenticatenaceae bacterium | Calcineurin Wok-DUF2238
MBN2420831.1 Candidatus Woesearchaeota archaeon | Wok-DUF2238 ? ? Calcineurin
WP_228035805.1 Oculatella sp. LEGE 06141 | Wok-DUF2238 ? ? Calcineurin
#############;GTA association : the GTA+GtrA fusion points in the direction of antigen modification
MBQ1452051.1 Clostridia bacterium | Wok-DUF2238 GTA GtrA
KMS59854.1 Novosphingobium barchaimii LL02 | GTA GtrA Wok-DUF2238
MBQ0014302.1 Candidatus Limimonas egerieequi | ABHydrolase Wok-DUF2238 GtrA GTA
NOW78670.1 | GTA GtrA Wok-DUF2238
NOW67382.1 | GTA GtrA Wok-DUF2238
WP_171797841.1 Novosphingobium sp. BK626 | GTA GtrA Wok-DUF2238
WP_054130693.1 Novosphingobium sp. GV061 | GTA GtrA Wok-DUF2238
KPF56991.1 Novosphingobium sp. AAP1 | GTA GtrA Wok-DUF2238
MCB2083326.1 Sphingomonadaceae bacterium | GTA GtrA Wok-DUF2238
WP_160604124.1 Pontixanthobacter aquaemixtae | GTA GtrA Wok-DUF2238
WP_235005100.1 Kocuria rhizophila | GTA Wok-DUF2238
OGJ13143.1 Candidatus Pacearchaeota archaeon RBG_16_35_8 | Wok-DUF2238 GTA
OFT55112.1 Brachybacterium sp. HMSC06H03 | GTA Wok-DUF2238
WP_017823372.1 Brachybacterium muris UCD-AY4 | GTA Wok-DUF2238
MBR6479640.1 Clostridia bacterium | ABHydrolase Wok-DUF2238 GtrA GTA
WP_157496950.1 Leifsonia sp. Leaf264 | Wok-DUF2238 GTA
WP_192061802.1 Frigoribacterium sp. CFBP9029 | Wok-DUF2238 GTA
WP_123914073.1 Georgenia muralis | Wok-DUF2238 GTA
WP_160952330.1 Brevibacterium rongguiense | GTA Wok-DUF2238
WP_138176344.1 Glutamicibacter sp. V16R2B1 | Wok-DUF2238 GTA
WP_129660969.1 Rothia halotolerans | GTA Wok-DUF2238
WP_225564007.1 Isoptericola luteus | Wok-DUF2238 GTA
WP_127126013.1 Georgenia sp. SYP-B2076 | Wok-DUF2238 GTA
EMR00469.1 Paeniglutamicibacter gangotriensis Lz1y | Wok-DUF2238 GTA
WP_044496349.1 Nesterenkonia massiliensis | GTA Wok-DUF2238
ASN37964.1 Arthrobacter sp. 7749 | Wok-DUF2238 GTA
WP_211281826.1 Sanguibacter antarcticus | Wok-DUF2238 GTA
MBP2422647.1 | Wok-DUF2238 GTA
WP_191802331.1 Oerskovia merdavium | Wok-DUF2238 GTA
WP_156742113.1 Occultella aeris | Wok-DUF2238 GTA
WP_144795656.1 Microbacterium paludicola | GTA Wok-DUF2238
WP_193720300.1 Oerskovia douganii | Wok-DUF2238 GTA
WP_228087962.1 Cellulosimicrobium cellulans | Wok-DUF2238 GTA
WP_204809206.1 Oerskovia paurometabola | Wok-DUF2238 GTA
WP_206611407.1 Nocardioides yefusunii | Wok-DUF2238 GTA
WP_220094064.1 Arthrobacter sp. AQ5-05 | Wok-DUF2238 GTA
WP_096256321.1 Glutamicibacter sp. BW77 | Wok-DUF2238 GTA
WP_157607330.1 Sanguibacter suarezii NBRC 16159 | Wok-DUF2238 GTA
WP_021011402.1 Agrococcus pavilionensis RW1 | GTA Wok-DUF2238
MBD8058986.1 Cellulomonas sp. JH27-2 | Wok-DUF2238 GTA
WP_168447343.1 Sanguibacter hominis ATCC BAA-789 | Wok-DUF2238 GTA
WP_083587072.1 Agrococcus sp. Marseille-P2731 | GTA Wok-DUF2238
QDC26839.1 Georgenia yuyongxinii | Wok-DUF2238 GTA
WP_012865103.1 Sanguibacter keddieii DSM 10542 | Wok-DUF2238 GTA
WP_235522439.1 Cellulomonas sp. Root137 | Wok-DUF2238 GTA
MBZ6373816.1 Kocuria palustris | GTA Wok-DUF2238
WP_188717131.1 Agrococcus terreus | GTA Wok-DUF2238
WP_118766339.1 Cellulomonas rhizosphaerae | Wok-DUF2238 GTA
WP_020015546.1 Promicromonospora sukumoe 327MFSha3.1 | Wok-DUF2238 GTA
WP_130782200.1 Cellulomonas biazotea | Wok-DUF2238 GTA
WP_136425501.1 Glaciibacter flavus | Wok-DUF2238 GTA
WP_158034510.1 Kocuria coralli | GTA Wok-DUF2238
WP_128188696.1 | GTA Wok-DUF2238
WP_198732355.1 Sanguibacter suaedae | Wok-DUF2238 GTA
NNH55069.1 Promicromonospora citrea | Wok-DUF2238 GTA
PFG35568.1 Flavimobilis soli | Wok-DUF2238 GTA
WP_154614088.1 Nocardioides marmotae | Wok-DUF2238 GTA
QMS56091.1 Kocuria varians | GTA Wok-DUF2238
WP_123306977.1 Cellulomonas sp. PhB143 | Wok-DUF2238 GTA
SMF08510.1 Kocuria marina subsp. indica | GTA Wok-DUF2238
WP_235521235.1 Cellulomonas sp. Leaf395 | Wok-DUF2238 GTA
WP_129701304.1 | GTA Wok-DUF2238
WP_208290115.1 | Wok-DUF2238 GTA
WP_012803889.1 | GTA Wok-DUF2238
########;ABHydrolase association.
MBG6116727.1 Sphingobium sp. JAI105 | Wok-DUF2238 ABhydrolase
#;association
WP_066523808.1 Sphingobium tyrosinilyticum | Wok-DUF2238 ABHydrolase
WP_037468669.1 Sphingobium herbicidovorans | Wok-DUF2238 ABHydrolase
WP_007705358.1 Sphingobium sp. AP49 | Wok-DUF2238 ABHydrolase
WP_037542211.1 Sphingobium sp. 10 DY56-G10 | Wok-DUF2238 ABHydrolase
WP_222990272.1 | Wok-DUF2238 ABHydrolase
WP_010126452.1 Sphingomonas sp. KC8 | Wok-DUF2238 ? ABHydrolase
WP_072674285.1 Erythrobacter sanguineus | Wok-DUF2238 ? ? ABHydrolase
WP_051698988.1 Erythrobacter longus | Wok-DUF2238 ? ? ABHydrolase
#;only peptidase_S9_ABHydrolase
MBL8747133.1 Phycisphaerae bacterium | b-prop Peptidase_S9_ABhydrolase Wok-DUF2238
MCB1570123.1 Lysobacterales bacterium | Wok-DUF2238 b-prop Peptidase_S9_ABhydrolase
WP_066768317.1 Sphingobium sp. CCH11-B1 | b-prop Peptidase_S9_ABhydrolase ? Wok-DUF2238
WP_168603655.1 Novosphingobium sp. SG707 | b-prop Peptidase_S9_ABhydrolase ? Wok-DUF2238
WP_031570863.1 Rheinheimera texasensis DSM 17496 | b-prop Peptidase_S9_ABhydrolase ? ? Wok-DUF2238
TXH97966.1 Rheinheimera sp. | b-prop Peptidase_S9_ABhydrolase ? ? Wok-DUF2238
MCE2903299.1 Gemmatimonadota bacterium | b-prop Peptidase_S9_ABhydrolase Wok-DUF2238
MBU7043172.1 Theionarchaea archaeon | b-prop Peptidase_S9_ABhydrolase Wok-DUF2238
MBK5267635.1 Acidimicrobiia bacterium | b-prop Peptidase_S9_ABhydrolase Wok-DUF2238
MBM4407366.1 Chloroflexota bacterium | Wok-DUF2238 ? b-prop Peptidase_S9_ABhydrolase
MBA3744290.1 Sporichthya sp. | Wok-DUF2238 Peptidase_S9_ABhydrolase b-prop

MBA3383384.1
Actinomycetota bacterium
Wok-DUF2238(frag) b-prop Peptidase_S9_ABHydrolase

#;two gene association

EPD51490.1
Paenisporosarcina sp. HGH0030
Wok-DUF2238 ABHydrolase

MBS3135573.1
Candidatus Woesearchaeota archaeon
Wok-DUF2238 ABHydrolase

WP_231936731.1
Mesotoga infera
Wok-DUF2238 ABHydrolase

MBQ7596625.1
Clostridia bacterium
Wok-DUF2238 ABHydrolase

WP_091026193.1
Nocardioides szechwanensis
Wok-DUF2238 ABHydrolase

NLM31288.1
Acholeplasmataceae bacterium
ABHydrolase Wok-DUF2238

PHQ62968.1
Sphingobium sp.
Wok-DUF2238 ABHydrolase

WP_143160899.1
Pollutimonas bauzanensis
Wok-DUF2238 ABHydrolase

WP_025292501.1
Sphingomonas sanxanigenens DSM 19645 = NX02
Wok-DUF2238 ABHydrolase

MBT8143038.1
Gammaproteobacteria bacterium
ABHydrolase Wok-DUF2238

MBN2198126.1
Candidatus Wolfebacteria bacterium
Wok-DUF2238 ABHydrolase

SMN17043.1
uncultured Candidatus Thioglobus sp.
ABHydrolase Wok-DUF2238

MBR2126107.1
Akkermansia sp.
ABHydrolase ? ABHydrolase Wok-DUF2238

WP_053950992.1
Candidatus Thioglobus autotrophicus
ABHydrolase Wok-DUF2238

HHZ18701.1
Acholeplasmataceae bacterium
ABHydrolase Wok-DUF2238

WP_104432892.1
Kineococcus xinjiangensis
ABHydrolase Wok-DUF2238

WP_069386585.1
Cellulosimicrobium cellulans
ABHydrolase Wok-DUF2238

KGH47168.1
Modestobacter caceresii
Wok-DUF2238 ABHydrolase

HJB19053.1
Candidatus Bariatricus faecipullorum
ABHydrolase ? tRNA Wok-DUF2238

WP_050697659.1
Anaeromassilibacillus senegalensis
Wok-DUF2238 ? ? ABHydrolase

WP_076071661.1
Sphingomonas montana
Wok-DUF2238 ? ABHydrolase

WP_157216903.1
Flavisphingomonas formosensis
Wok-DUF2238 ? ? ABHydrolase

MBS3127792.1
Candidatus Woesearchaeota archaeon
ABHydrolase ? ? Wok-DUF2238

VUX55448.1
uncultured Woeseiaceae bacterium
Wok-DUF2238 ? ABHydrolase

NVB79092.1
Kofleriaceae bacterium
ABHydrolase ? ? Wok-DUF2238

MBS3175620.1
Candidatus Woesearchaeota archaeon
ABHydrolase ? ? Wok-DUF2238

WP_218974716.1
Streptomyces sp. NP160
ABHydrolase ? Wok-DUF2238

MBV9329257.1
Chloroflexota bacterium
ABHydrolase ? Wok-DUF2238

WP_188893260.1
Microlunatus endophyticus
ABHydrolase ? Wok-DUF2238

WP_204534783.1
Arthrobacter tumbae
Wok-DUF2238 ? ABHydrolase

WP_056548971.1
Arthrobacter sp. Soil782
Wok-DUF2238 ? ? ABHydrolase

KKR03726.1
Parcubacteria group bacterium GW2011_GWF2_39_13b
Wok-DUF2238 ? GT4 ABHydrolase

MBV9582049.1
Chloroflexota bacterium
ABHydrolase ? Wok-DUF2238

MCA1648553.1
Chloroflexota bacterium
ABHydrolase ? Wok-DUF2238

NOJ31466.1
Candidatus Nitrosocosmicus sp.
Wok-DUF2238 ABHydrolase

WP_143018028.1
Belnapia rosea
ABHydrolase ? Wok-DUF2238

#############;comes with second, inactive copy

SFG40631.1
Halopelagius inordinatus
Wok-DUF2238-i Wok-DUF2238

WP_020447318.1
Salinarchaeum sp. Harcht-Bsk1
Wok-DUF2238-i Wok-DUF2238

PSQ47968.1
Halobacteriales archaeon SW_7_65_23
Wok-DUF2238-i Wok-DUF2238

ELY58950.1
Natronolimnohabitans innermongolicus JCM 12255
Wok-DUF2238-i Wok-DUF2238

ELY98665.1
Natrialba asiatica DSM 12278
Wok-DUF2238-i Wok-DUF2238

WP_049990112.1
Natrinema salifodinae
Wok-DUF2238-i Wok-DUF2238

WP_235856258.1
Methanolobus halotolerans
Wok-DUF2238-i Wok-DUF2238

WP_091710736.1
Methanolobus vulcani
Wok-DUF2238-i Wok-DUF2238

NEU58059.1
Halorussus sp. MSC15.2
Wok-DUF2238-i Wok-DUF2238

WP_049922394.1
Halopiger djelfimassiliensis
Wok-DUF2238-i Wok-DUF2238

WP_084006234.1
Methanohalophilus portucalensis FDF-1
Wok-DUF2238-i Wok-DUF2238

WP_054862153.1
Natronolimnobius baerhuensis
Wok-DUF2238-i Wok-DUF2238

WP_091936803.1
Methanolobus profundi
Wok-DUF2238-i Wok-DUF2238

WP_015233554.1
Wok-DUF2238-i Wok-DUF2238

WP_227356761.1
Haladaptatus salinisoli
Wok-DUF2238-i Wok-DUF2238

WP_159484348.1
Halorubrum sp. JWXQ-INN 858
Wok-DUF2238-i Wok-DUF2238

NHX37461.1
Halolamina sp. R1-12
Wok-DUF2238-i Wok-DUF2238

WP_226778592.1
Natrinema zhouii
Wok-DUF2238-i Wok-DUF2238

WP_049954332.1
Halostagnicola larsenii XH-48
Wok-DUF2238-i Wok-DUF2238

WP_130170158.1
Natrinema altunense
Wok-DUF2238-i Wok-DUF2238

PSP27596.1
Halobacteriales archaeon QH_2_65_14
Wok-DUF2238-i Wok-DUF2238

WP_004216062.1
Natrialba magadii ATCC 43099
Wok-DUF2238-i Wok-DUF2238

WP_006432140.1
Natrinema versiforme JCM 10478
Wok-DUF2238-i Wok-DUF2238

WP_090309187.1
Natronorubrum texcoconense
Wok-DUF2238-i Wok-DUF2238

ELZ58992.1
Halorubrum distributum JCM 10118
Wok-DUF2238-i Wok-DUF2238

QLG29447.1
Halorarum halophilum
Wok-DUF2238-i Wok-DUF2238

WP_114578944.1
Saliphagus sp. LR7
Wok-DUF2238-i Wok-DUF2238

WP_167937536.1
Natronococcus sp. JC468
Wok-DUF2238-i Wok-DUF2238

WP_179168094.1
Wok-DUF2238-i Wok-DUF2238

SIR85093.1
Haladaptatus litoreus
Wok-DUF2238-i Wok-DUF2238

MBB6646564.1
Halobellus ruber
Wok-DUF2238-i Wok-DUF2238

WP_075937154.1
Halosegnis longus
Wok-DUF2238-i Wok-DUF2238

WP_220617756.1
Haloarcula rubra
Wok-DUF2238-i Wok-DUF2238

WP_170082880.1
Haloarcula rubripromontorii
Wok-DUF2238-i Wok-DUF2238

WP_178333365.1
Natronomonas salina
Wok-DUF2238-i Wok-DUF2238

WP_197427993.1
Halogeometricum sp. CBA1124
Wok-DUF2238-i Wok-DUF2238

WP_121599443.1
Halorubrum sp. ASP121
Wok-DUF2238-i Wok-DUF2238

HIH98457.1
Thermoplasmata archaeon
Wok-DUF2238-i Wok-DUF2238

WP_157525953.1
Halorientalis sp. IM1011
Wok-DUF2238-i Wok-DUF2238

MBR3553606.1
Clostridia bacterium
Wok-DUF2238-i Wok-DUF2238

MBR5409398.1
Clostridia bacterium
Wok-DUF2238-i Wok-DUF2238

MBR5423080.1
Clostridia bacterium
Wok-DUF2238-i Wok-DUF2238

MBR5825477.1
Clostridia bacterium
Wok-DUF2238-i Wok-DUF2238

MBQ7688260.1
Clostridia bacterium
Wok-DUF2238-i Wok-DUF2238

MBQ7637697.1
Clostridia bacterium
Wok-DUF2238-i Wok-DUF2238

MBQ1553336.1
Clostridia bacterium
Wok-DUF2238-i Wok-DUF2238

MBR5427089.1
Clostridia bacterium
Wok-DUF2238-i Wok-DUF2238

WP_112080505.1
Halorubrum sp. 48-1-W
Wok-DUF2238-i Wok-DUF2238

WP_156588133.1
Halorubrum sp. CBA1125
Wok-DUF2238-i Wok-DUF2238

WP_156588081.1
Halorubrum sp. CBA1125
Wok-DUF2238-i Wok-DUF2238 disordered

MBT3771994.1
Methanobacteriota archaeon
Wok-DUF2238-i Wok-DUF2238 2TM

RUA02550.1
Candidatus Poseidoniales archaeon
Wok-DUF2238-i Wok-DUF2238 2TM

MBL6882431.1
Candidatus Poseidoniaceae archaeon
Wok-DUF2238-i Wok-DUF2238 2TM

MBI5289281.1
Chloroflexota bacterium
Wok-DUF2238-i Wok-DUF2238

WP_009487085.1
Wok-DUF2238-i Wok-DUF2238

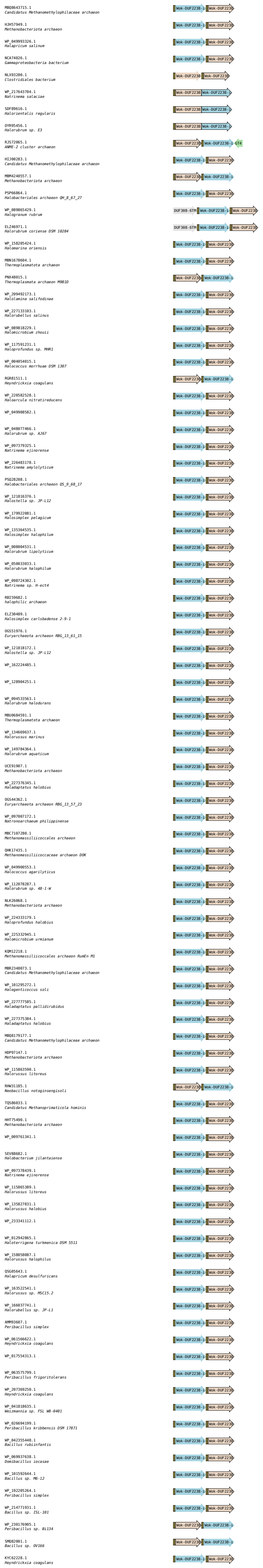
MBQ8643715.1
Candidatus Methanomethylophilaceae archaeon
HJH57949.1
Methanobacteriota archaeon
WP_049993326.1
Halapricum salinum
NCA74026.1
Gammaproteobacteria bacterium
NLX93200.1
Clostridiales bacterium
WP_217643704.1
Natrinema salaciae
SDF89616.1
Halorientalis regularis
OYR95456.1
Halorubrum sp. E3
RJS72065.1
ANME-2 cluster archaeon
HIJ00283.1
Candidatus Methanomethylophilaceae archaeon
MBM4240557.1
Methanobacteriota archaeon
PSP66864.1
Halobacteriales archaeon QH_8_67_27
WP_089865429.1
Halogranum rubrum
ELZ46971.1
Halorubrum coriense DSM 10284
WP_158205424.1
Halomarina oriensis
MBN1678604.1
Thermoplasmatota archaeon
PNX48015.1
Thermoplasmata archaeon M9B1D
WP_209492173.1
Halolamina salifodinae
WP_227133103.1
Halorubellus salinus
WP_089818229.1
Halomicrobium zhouii
WP_117591231.1
Haloprofundus sp. MHR1
WP_004054015.1
Halococcus morrhuae DSM 1307
RGR81511.1
Heyndrickxia coagulans
WP_220582528.1
Haloarcula nitratireducens
WP_049988582.1
WP_048077466.1
Halorubrum sp. AJ67
WP_097379325.1
Natrinema ejinorense
WP_226483178.1
Natrinema amylolyticum
PSQ28208.1
Halobacteriales archaeon QS_9_68_17
WP_121816376.1
Halostella sp. JP-L12
WP_179922081.1
Halosimplex pelagicum
WP_135364535.1
Halosimplex halophilum
WP_008004531.1
Halorubrum lipolyticum
WP_050033033.1
Halorubrum halophilum
WP_098724302.1
Natrinema sp. H-ect4
RBI59682.1
halophilic archaeon
ELZ30489.1
Halosimplex carlsbadense 2-9-1
OGS51976.1
Euryarchaeota archaeon RBG_13_61_15
WP_121818172.1
Halostella sp. JP-L12
WP_162224485.1
WP_128904251.1
WP_094533563.1
Halorubrum halodurans
MBU0684591.1
Thermoplasmatota archaeon
WP_134669637.1
Halorussus marinus
WP_149784364.1
Halorubrum aquaticum
UCE91907.1
Methanobacteriota archaeon
WP_227376345.1
Haladaptatus halobius
OGS44362.1
Euryarchaeota archaeon RBG_13_57_23
WP_097007172.1
Natronoarchaeum philippinense
MBC7107280.1
Methanomassiliicoccales archaeon
QHK17435.1
Methanomassiliicoccaceae archaeon DOK
WP_049900553.1
Halococcus agarilyticus
WP_112078287.1
Halorubrum sp. 48-1-W
NLK26068.1
Methanobacteriota archaeon
WP_224333179.1
Haloprofundus halobius
WP_225332945.1
Halomicrobium urmianum
KQM12218.1
Methanomassiliicoccales archaeon RumEn M1
MBR2348073.1
Candidatus Methanomethylophilaceae archaeon
WP_181295272.1
Halegenticoccus soli
WP_227777585.1
Haladaptatus pallidirubidus
WP_227375384.1
Haladaptatus halobius
MBQ8179177.1
Candidatus Methanomethylophilaceae archaeon
HDP97147.1
Methanobacteriota archaeon
WP_115863590.1
Halorussus litoreus
RHW31185.1
Neobacillus notoginsengisoli
TOS86033.1
Candidatus Methanoprimaticola hominis
HHT75490.1
Methanobacteriota archaeon
WP_009761341.1
SEV86682.1
Halobacterium jilantaiense
WP_097378439.1
Natrinema ejinorense
WP_115865389.1
Halorussus litoreus
WP_135827831.1
Halorussus halobius
WP_233341112.1
WP_012942865.1
Haloterrigena turkmenica DSM 5511
WP_158058087.1
Halorussus halophilus
QSG05643.1
Halapricum desulfuricans
WP_163522541.1
Halorussus sp. MSC15.2
WP_166037741.1
Halorubellus sp. JP-L1
AMM93807.1
Peribacillus simplex
WP_061566622.1
Heyndrickxia coagulans
WP_017554313.1
WP_063575799.1
Peribacillus frigoritolerans
WP_207369250.1
Heyndrickxia coagulans
WP_041818635.1
Weizmannia sp. FSL W8-0401
WP_026694199.1
Peribacillus kribbensis DSM 17871
WP_042355448.1
Bacillus rubiinfantis
WP_069937638.1
Domibacillus iocasae
WP_101592644.1
Bacillus sp. MG-12
WP_192205264.1
Peribacillus simplex
WP_214771931.1
Bacillus sp. ISL-101
WP_230176905.1
Peribacillus sp. Bi134
SMD82001.1
Bacillus sp. OV166
KYC62228.1
Heyndrickxia coagulans
DUF308-6TM
Wok-DUF2238-1
Wok-DUF2238
GT4

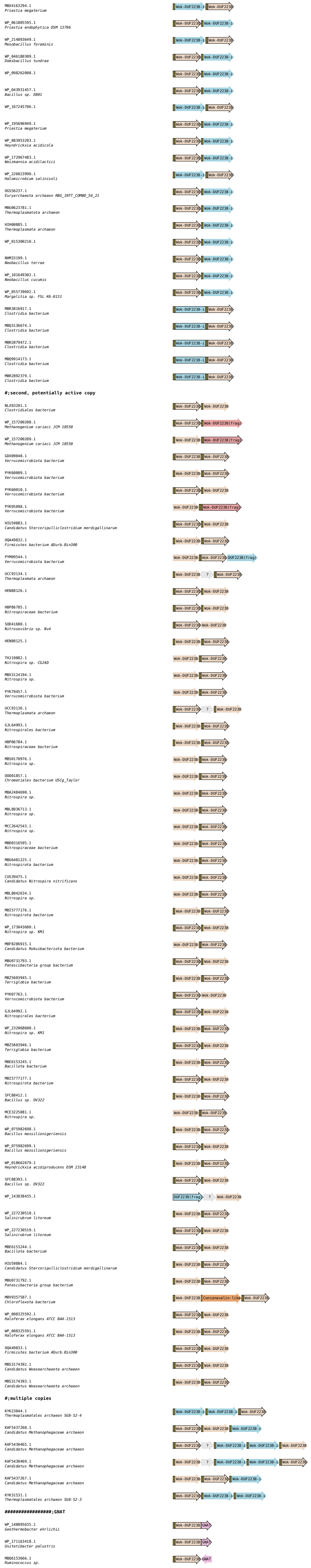
MBX4163294.1
Priestia megaterium
Wok-DUF2238-i Wok-DUF2238
WP_061805595.1
Priestia endophytica DSM 13796
Wok-DUF2238 Wok-DUF2238-i
WP_214893649.1
Mesobacillus foraminis
Wok-DUF2238-i Wok-DUF2238
WP_046180309.1
Domibacillus tundrae
Wok-DUF2238 Wok-DUF2238-i
WP_098262008.1
Wok-DUF2238 Wok-DUF2238-i
WP_043931457.1
Bacillus sp. EB01
Wok-DUF2238 Wok-DUF2238-i
WP_167245706.1
Wok-DUF2238-i Wok-DUF2238
WP_195696949.1
Priestia megaterium
Wok-DUF2238 Wok-DUF2238-i
WP_083953203.1
Heyndrickxia acidicola
Wok-DUF2238 Wok-DUF2238-i
WP_172967483.1
Weizmannia acidilactici
Wok-DUF2238 Wok-DUF2238-i
WP_226023990.1
Halomicrobium salinisoli
Wok-DUF2238-i Wok-DUF2238
OGS56237.1
Euryarchaeota archaeon RBG_19FT_COMBO_56_21
Wok-DUF2238 Wok-DUF2238-i
MBU0623781.1
Thermoplasmatota archaeon
Wok-DUF2238 Wok-DUF2238-i
HIH00885.1
Thermoplasmata archaeon
Wok-DUF2238 Wok-DUF2238-i
WP_015300210.1
Wok-DUF2238 Wok-DUF2238-i
NHM33199.1
Neobacillus terrae
Wok-DUF2238 Wok-DUF2238-i
WP_101649302.1
Neobacillus cucumis
Wok-DUF2238 Wok-DUF2238-i
WP_055739692.1
Margalitia sp. FSL K6-0131
Wok-DUF2238 Wok-DUF2238-i
MBR3816917.1
Clostridia bacterium
Wok-DUF2238-i Wok-DUF2238
MBQ3136674.1
Clostridia bacterium
Wok-DUF2238-i Wok-DUF2238
MBR2079472.1
Clostridia bacterium
Wok-DUF2238-i Wok-DUF2238
MBQ9914173.1
Clostridia bacterium
Wok-DUF2238-i Wok-DUF2238
MBR2892379.1
Clostridia bacterium
Wok-DUF2238-i Wok-DUF2238
#;second, potentially active copy
NLX93201.1
Clostridiales bacterium
Wok-DUF2238 Wok-DUF2238
WP_157200208.1
Methanogenium cariaci JCM 10550
Wok-DUF2238 Wok-DUF2238(frag)
WP_157200209.1
Methanogenium cariaci JCM 10550
Wok-DUF2238 Wok-DUF2238(frag)
GDX09048.1
Verrucomicrobiota bacterium
Wok-DUF2238 Wok-DUF2238
PYK60009.1
Verrucomicrobiota bacterium
Wok-DUF2238 Wok-DUF2238
PYK60010.1
Verrucomicrobiota bacterium
Wok-DUF2238 Wok-DUF2238
PYK95098.1
Verrucomicrobiota bacterium
Wok-DUF2238 Wok-DUF2238(frag)
HIU59883.1
Candidatus Stercoripulliclostridium merdigallinarum
Wok-DUF2238 Wok-DUF2238
OQA49832.1
Firmicutes bacterium ADurb.Bin300
Wok-DUF2238 Wok-DUF2238
PYM09544.1
Verrucomicrobiota bacterium
Wok-DUF2238 Wok-DUF2238 DUF2238(frag)
UCC93134.1
Thermoplasmata archaeon
Wok-DUF2238 ? Wok-DUF2238
HEN80126.1
Wok-DUF2238 Wok-DUF2238
HBP86785.1
Nitrospiraceae bacterium
Wok-DUF2238 Wok-DUF2238
SOD41680.1
Nitrosovibrio sp. Nv4
Wok-DUF2238 Wok-DUF2238
HEN80125.1
Wok-DUF2238 Wok-DUF2238
THJ19882.1
Nitrospira sp. CG24D
Wok-DUF2238 Wok-DUF2238
MBX3124194.1
Nitrospira sp.
Wok-DUF2238 Wok-DUF2238
PYK79457.1
Verrucomicrobiota bacterium
Wok-DUF2238 Wok-DUF2238
UCC93136.1
Thermoplasmata archaeon
Wok-DUF2238 ? Wok-DUF2238
GJL64993.1
Nitrospirales bacterium
Wok-DUF2238 Wok-DUF2238
HBP86784.1
Nitrospiraceae bacterium
Wok-DUF2238 Wok-DUF2238
MBS0170976.1
Nitrospira sp.
Wok-DUF2238 Wok-DUF2238
OOO01857.1
Chromatiales bacterium USCg_Taylor
Wok-DUF2238 Wok-DUF2238
MBA2484690.1
Nitrospira sp.
Wok-DUF2238 Wok-DUF2238
MBL8036713.1
Nitrospira sp.
Wok-DUF2238 Wok-DUF2238
MCC2642543.1
Nitrospira sp.
Wok-DUF2238 Wok-DUF2238
MBD0316595.1
Nitrospiraceae bacterium
Wok-DUF2238 Wok-DUF2238
MBU6481225.1
Nitrospirota bacterium
Wok-DUF2238 Wok-DUF2238
CUS39475.1
Candidatus Nitrospira nitrificans
Wok-DUF2238 Wok-DUF2238
MBL8042634.1
Nitrospira sp.
Wok-DUF2238 Wok-DUF2238
MBI5777176.1
Nitrospirota bacterium
Wok-DUF2238 Wok-DUF2238
WP_173043680.1
Nitrospira sp. KM1
Wok-DUF2238 Wok-DUF2238
MBF8286915.1
Candidatus Rokuibacteriota bacterium
Wok-DUF2238 Wok-DUF2238
MBU0731793.1
Patescibacteria group bacterium
Wok-DUF2238 Wok-DUF2238
MBZ5603945.1
Terriglobia bacterium
Wok-DUF2238 Wok-DUF2238
PYK07763.1
Verrucomicrobiota bacterium
Wok-DUF2238 Wok-DUF2238
GJL64992.1
Nitrospirales bacterium
Wok-DUF2238 Wok-DUF2238
WP_232068608.1
Nitrospira sp. KM1
Wok-DUF2238 Wok-DUF2238
MBZ5603946.1
Terriglobia bacterium
Wok-DUF2238 Wok-DUF2238
MBE6153245.1
Bacillota bacterium
Wok-DUF2238 Wok-DUF2238
MBI5777177.1
Nitrospirota bacterium
Wok-DUF2238 Wok-DUF2238
SFC88412.1
Bacillus sp. OV322
Wok-DUF2238 Wok-DUF2238
MCE3225081.1
Nitrospira sp.
Wok-DUF2238 Wok-DUF2238
WP_075982698.1
Bacillus massilionigeriensis
Wok-DUF2238 Wok-DUF2238
WP_075982699.1
Bacillus massilionigeriensis
Wok-DUF2238 Wok-DUF2238
WP_018662679.1
Heyndrickxia acidiproducens DSM 23148
Wok-DUF2238 Wok-DUF2238
SFC88393.1
Bacillus sp. OV322
Wok-DUF2238 Wok-DUF2238
WP_143838435.1
DUF2238(frag) ? Wok-DUF2238
WP_227230518.1
Salinirubrum litoreum
Wok-DUF2238 Wok-DUF2238
WP_227230519.1
Salinirubrum litoreum
Wok-DUF2238 Wok-DUF2238
MBE6153244.1
Bacillota bacterium
Wok-DUF2238 Wok-DUF2238
HIU59884.1
Candidatus Stercoripulliclostridium merdigallinarum
Wok-DUF2238 Wok-DUF2238
MBU0731792.1
Patescibacteria group bacterium
Wok-DUF2238 Wok-DUF2238
MBV9357587.1
Chloroflexota bacterium
Wok-DUF2238 Concanavalin-like Wok-DUF2238
WP_008325592.1
Haloferax elongans ATCC BAA-1513
Wok-DUF2238 Wok-DUF2238
WP_008325591.1
Haloferax elongans ATCC BAA-1513
Wok-DUF2238 Wok-DUF2238
OQA49833.1
Firmicutes bacterium ADurb.Bin300
Wok-DUF2238 Wok-DUF2238
MBS3174392.1
Candidatus Woesearchaeota archaeon
Wok-DUF2238 Wok-DUF2238
MBS3174393.1
Candidatus Woesearchaeota archaeon
Wok-DUF2238 Wok-DUF2238
#;multiple copies
KYK23044.1
Thermoplasmatales archaeon SG8-52-4
Wok-DUF2238-i Wok-DUF2238-i Wok-DUF2238
KAF5437268.1
Candidatus Methanophagaceae archaeon
Wok-DUF2238 Wok-DUF2238 Wok-DUF2238-i
KAF5430465.1
Candidatus Methanophagaceae archaeon
Wok-DUF2238 ? Wok-DUF2238-i Wok-DUF2238-i Wok-DUF2238
KAF5430469.1
Candidatus Methanophagaceae archaeon
Wok-DUF2238 ? Wok-DUF2238-i Wok-DUF2238-i Wok-DUF2238
KAF5437267.1
Candidatus Methanophagaceae archaeon
Wok-DUF2238 Wok-DUF2238 Wok-DUF2238-i
KYK31531.1
Thermoplasmatales archaeon SG8-52-3
Wok-DUF2238 Wok-DUF2238-i Wok-DUF2238-i
################;GNAT
WP_148895635.1
Geothermobacter ehrlichii
Wok-DUF2238 GNAT
WP_171163418.1
Usitatibacter palustris
Wok-DUF2238 GNAT
MBQ6153666.1
Ruminococcus sp.
Wok-DUF2238 GNAT

WP_068623176.1
Trichococcus ilyis

WP_087029980.1
Trichococcus palustris

WP_108032068.1
Trichococcus patagoniensis

WP_068561733.1
Trichococcus flocculiformis

OGU35605.1
Ignavibacteria bacterium GWA2_36_19

WP_119657583.1
Hymenobacter rubripertinctus

WP_054957885.1
Paenibacillus dakarensis

HIS85603.1
Candidatus Faecivicinus avistercoris

NNH22337.1
Pseudokineococcus marinus

CDA80550.1
Clostridium sp. CAG:594

WP_168972664.1
Clostridium cadaveris

HIU39770.1
Candidatus Aphodocola excrementigallinarum

WP_151903104.1
Corynebacterium urogenitale

#########;GT-4 association

MBR2265180.1
Paludibacteraceae bacterium

MBR4563506.1
Paludibacteraceae bacterium

UAL07814.1
Candidatus Methanogranum gryphiswaldense

MBS3084855.1
Candidatus Pacearchaeota archaeon

OHA14919.1
Candidatus Tagabacteria bacterium RIFCSPLOWO2_01_FULL_39_11

KHO48793.1
archaeon GW2011_AR1

OGH72445.1
Candidatus Magasanikbacteria bacterium RIFCSPHIGHO2_02_FULL_44_13

HAV78155.1
Anaerolineae bacterium

MBC7450237.1
Cytophaga sp.

MBK6342166.1
Flavobacteriales bacterium

AGI47093.1
Thermoplasmatales archaeon BRNA1

PIN90077.1
Candidatus Pacearchaeota archaeon CG10_big_fil_rev_8_21_14_0_10_32_14

OGN05648.1
Candidatus Yanofskybacteria bacterium RIFCSPHIGHO2_01_FULL_44_17

###########;other rare associations; ##;PAP2 fusions

MCC6792234.1
Thermomicrobiales bacterium

MCA9864200.1
Thermomicrobiales bacterium

PWB43846.1
Dehalococcoidia bacterium

MCC6791977.1
Thermomicrobiales bacterium

MCC7362922.1
Dehalococcoidia bacterium

MBX3072087.1
Thermomicrobiales bacterium

##;HAD association

WP_144405704.1
Aliterella atlantica CENA595

WP_214430857.1
Dendronalium phyllosphericum CENA369

WP_190491553.1
Microcoleus sp. FACHB-831

WP_009633646.1
Synechocystis sp. PCC 7509

MBW4620732.1
Cyanosarcina radialis HA8281-LM2

##;DUF218 fusion

MBQ9431174.1
Kiritimatiellia bacterium

MBN1618527.1
Candidatus Dojkabacteria bacterium

##; CDP-alcohol phosphatidyltransferase fusion

MCD7756484.1
Bacillota bacterium

##;TM module fusions

MBQ0136231.1
Candidatus Limimonas coprohippi

MBT5281840.1
Methanobacteriota archaeon

PIN90018.1
Candidatus Pacearchaeota archaeon CG10_big_fil_rev_8_21_14_0_10_32_14

MBW3540768.1
Planctomycetota bacterium

MBI4152004.1
Candidatus Woesearchaeota archaeon

WP_143406895.1
Methanonatronarchaeum thermophilum

BBA47077.1
Bifidobacterium bifidum LMG 13195

MBP7932492.1
Candidatus Syntrophosphaera sp.

MBW6514868.1
Candidatus Syntrophosphaera sp.

TFH03672.1
Spirochaetales bacterium

QHK18290.1
Methanomassiliicoccaceae archaeon DOK

MCD8294616.1
Clostridia bacterium

QLH75123.1
Methanomassiliicoccales archaeon

MBQ7861784.1
Clostridia bacterium

MBS3817221.1
Thermoplasmatota archaeon

MBS3790668.1
Thermoplasmatota archaeon

NTU99067.1
Candidatus Falkowbacteria bacterium

MBI4779041.1
Candidatus Falkowbacteria bacterium

PWI48475.1
Candidatus Heimdallarchaeota archaeon B3_Heim

##other misc fusions

MBL8925290.1
Pseudonocardia sp.

OGH69923.1
Candidatus Magasanikbacteria bacterium RIFCSPHIGHO2_01_FULL_47_8

MSU56085.1
Candidatus Taylorbacteria bacterium

WP_203892608.1

CPM80085.1
Bordetella pertussis

MBE6957471.1
Oscillospiraceae bacterium

MBQ7176933.1
Victivallales bacterium

TAL01349.1
Verrucomicrobiota bacterium

SFI28877.1
Jannaschia pohangensis

SCH24153.1
uncultured Eubacterium sp.

CAG0951764.1
Phycisphaerales bacterium

OGF24999.1
Candidatus Falkowbacteria bacterium RIFOXYB2_FULL_34_18

CAE7148932.1
Symbiodinium pilosum

THJ23699.1
Nitrospira sp. CG24D

MBR9691535.1
Candidatus Woesearchaeota archaeon

MBP3405656.1
Kiritimatiellia bacterium

MBV9357588.1
Chloroflexota bacterium

MBN2251035.1
Candidatus Altiarchaeota archaeon

####################;standalone

HHN36860.1

MBQ7650205.1
Victivallales bacterium

CNE07418.1
Mycobacterium tuberculosis

EGY51328.1
Neisseria shayeganii 871

MBA2313649.1
Actinomycetota bacterium

HIX46938.1
Candidatus Borkfalkia faecigallinarum

MBQ8522299.1
Clostridia bacterium

SCJ94434.1
uncultured Clostridium sp.

MBY0274907.1
Candidatus Binatia bacterium

GGL21580.1
Planomonospora parontospora subsp. antibiotica

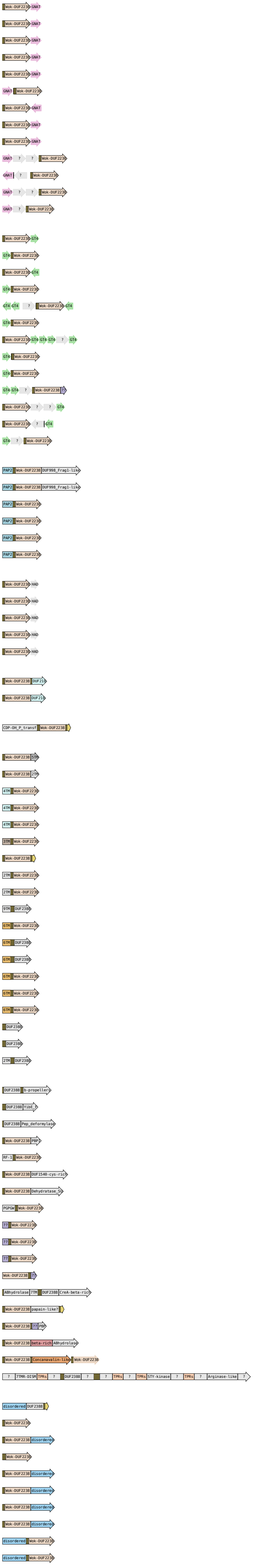

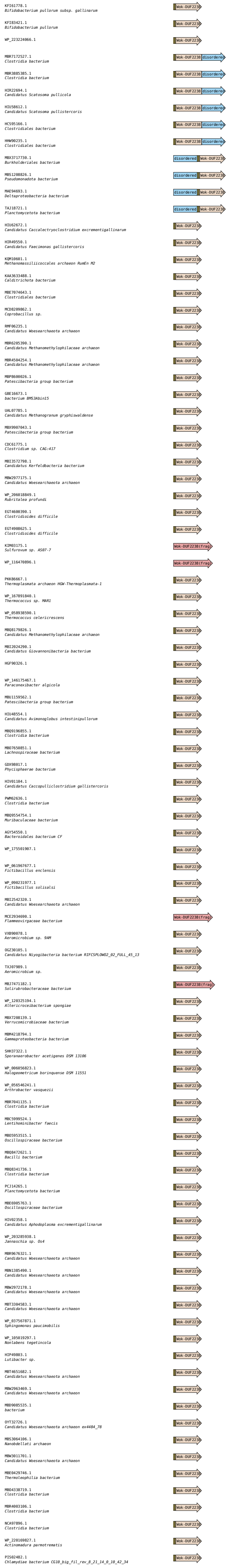
KFI61778.1
Bifidobacterium pullorum subsp. gallinarum
Wok-DUF2238
KFI83421.1
Bifidobacterium pullorum
Wok-DUF2238
WP_223224066.1
Wok-DUF2238
MBR7172527.1
Clostridia bacterium
Wok-DUF2238
disordered
MBR3885385.1
Clostridia bacterium
Wok-DUF2238
disordered
HIR22694.1
Candidatus Scatosoma pullicola
Wok-DUF2238
disordered
HIU58612.1
Candidatus Scatosoma pullistercoris
Wok-DUF2238
disordered
HCS95166.1
Clostridiales bacterium
Wok-DUF2238
disordered
HHW90235.1
Clostridiales bacterium
Wok-DUF2238
disordered
MBX3717730.1
Burkholderiales bacterium
disordered
Wok-DUF2238
MBS1208826.1
Pseudomonadota bacterium
disordered
Wok-DUF2238
MAE94693.1
Deltaproteobacteria bacterium
disordered
Wok-DUF2238
TAJ18721.1
Planctomycetota bacterium
disordered
Wok-DUF2238
HIU62672.1
Candidatus Caccalectryoclostridium excrementigallinarum
Wok-DUF2238
HIR49550.1
Candidatus Faecimonas gallistercoris
Wok-DUF2238
KQM10601.1
Methanomassiliicoccales archaeon RumEn M2
Wok-DUF2238
KAA3633488.1
Calditrichota bacterium
Wok-DUF2238
MBE7074643.1
Clostridiales bacterium
Wok-DUF2238
MCD8209862.1
Coprobacillus sp.
Wok-DUF2238
RMF06235.1
Candidatus Woesearchaeota archaeon
Wok-DUF2238
MBR6205390.1
Candidatus Methanomethylophilaceae archaeon
Wok-DUF2238
MBR4504254.1
Candidatus Methanomethylophilaceae archaeon
Wok-DUF2238
MBP8600026.1
Patescibacteria group bacterium
Wok-DUF2238
GBE16673.1
bacterium BMS3Abin15
Wok-DUF2238
UAL07785.1
Candidatus Methanogranum gryphiswaldense
Wok-DUF2238
MBX9907043.1
Patescibacteria group bacterium
Wok-DUF2238
CDC61775.1
Clostridium sp. CAG:417
Wok-DUF2238
MBI3572798.1
Candidatus Kerfeldbacteria bacterium
Wok-DUF2238
MBW2977175.1
Candidatus Woesearchaeota archaeon
Wok-DUF2238
WP_206018849.1
Rubritalea profundi
Wok-DUF2238
EGT4600390.1
Clostridioides difficile
Wok-DUF2238
EGT4908625.1
Clostridioides difficile
Wok-DUF2238
KIM03175.1
Sulfurovum sp. AS07-7
Wok-DUF2238(frag)
WP_116470896.1
Wok-DUF2238(frag)
PKK86667.1
Thermoplasmata archaeon HGW-Thermoplasmata-1
Wok-DUF2238
WP_167891840.1
Thermococcus sp. MAR1
Wok-DUF2238
WP_058938590.1
Thermococcus celericrescens
Wok-DUF2238
MBQ8179826.1
Candidatus Methanomethylophilaceae archaeon
Wok-DUF2238
MBI2024290.1
Candidatus Giovannonibacteria bacterium
Wok-DUF2238
HGF90326.1
Wok-DUF2238
WP_146175467.1
Paraconexibacter algicola
Wok-DUF2238
MBU1159562.1
Patescibacteria group bacterium
Wok-DUF2238
HIU48554.1
Candidatus Avimonoglobus intestinipullorum
Wok-DUF2238
MBQ9196855.1
Clostridia bacterium
Wok-DUF2238
MBO7650851.1
Lachnospiraceae bacterium
Wok-DUF2238
GDX98017.1
Phycisphaerae bacterium
Wok-DUF2238
HIV01104.1
Candidatus Caccopulliclostridium gallistercoris
Wok-DUF2238
PWM62636.1
Clostridia bacterium
Wok-DUF2238
MBQ9554754.1
Muribaculaceae bacterium
Wok-DUF2238
AGY54550.1
Bacteroidales bacterium CF
Wok-DUF2238
WP_175501907.1
Wok-DUF2238
WP_061967677.1
Fictibacillus enclensis
Wok-DUF2238
WP_090231977.1
Fictibacillus solisalsi
Wok-DUF2238
MBI2542320.1
Candidatus Woesearchaeota archaeon
Wok-DUF2238
MCE2934690.1
Flammeovirgaceae bacterium
Wok-DUF2238(frag)
VXB90078.1
Aeromicrobium sp. 9AM
Wok-DUF2238
OGZ30105.1
Candidatus Niyogibacteria bacterium RIFCSPLOWO2_02_FULL_45_13
Wok-DUF2238
TXJ07989.1
Aeromicrobium sp.
Wok-DUF2238
MBJ7471182.1
Solirubrobacteraceae bacterium
Wok-DUF2238(frag)
WP_120325194.1
Altericroceibacterium spongiae
Wok-DUF2238
MBX7208139.1
Verrucomicrobiaceae bacterium
Wok-DUF2238
MBM4218794.1
Gammaproteobacteria bacterium
Wok-DUF2238
SHH37322.1
Sporanaerobacter acetigenes DSM 13106
Wok-DUF2238
WP_006056823.1
Halogeometricum borinquense DSM 11551
Wok-DUF2238
WP_056546241.1
Arthrobacter vasquezii
Wok-DUF2238
MBR7041135.1
Clostridia bacterium
Wok-DUF2238
MBC5999524.1
Lentihominibacter faecis
Wok-DUF2238
MBO5953515.1
Oscillospiraceae bacterium
Wok-DUF2238
MBQ8472621.1
Bacilli bacterium
Wok-DUF2238
MBQ8341736.1
Clostridia bacterium
Wok-DUF2238
PCJ14265.1
Planctomycetota bacterium
Wok-DUF2238
MBE6905763.1
Oscillospiraceae bacterium
Wok-DUF2238
HIV02358.1
Candidatus Aphodoplasma excrementigallinarum
Wok-DUF2238
WP_203285938.1
Jannaschia sp. Os4
Wok-DUF2238
MBR9676321.1
Candidatus Woesearchaeota archaeon
Wok-DUF2238
MBN1385490.1
Candidatus Woesearchaeota archaeon
Wok-DUF2238
MBW2972178.1
Candidatus Woesearchaeota archaeon
Wok-DUF2238
MBT3304583.1
Candidatus Woesearchaeota archaeon
Wok-DUF2238
WP_037567871.1
Sphingomonas paucimobilis
Wok-DUF2238
WP_105019297.1
Nonlabens tegetincola
Wok-DUF2238
HIP49803.1
Lutibacter sp.
Wok-DUF2238
MBT4651682.1
Candidatus Woesearchaeota archaeon
Wok-DUF2238
MBW2963469.1
Candidatus Woesearchaeota archaeon
Wok-DUF2238
MBD9085535.1
bacterium
Wok-DUF2238
OYT32726.1
Candidatus Woesearchaeota archaeon ex4484_78
Wok-DUF2238
MBS3064106.1
Nanobdellati archaeon
Wok-DUF2238
MBW3011701.1
Candidatus Woesearchaeota archaeon
Wok-DUF2238
MBE0429746.1
Thermoleophilia bacterium
Wok-DUF2238
MBO4338719.1
Clostridia bacterium
Wok-DUF2238
MBR4003106.1
Clostridia bacterium
Wok-DUF2238
NCA97896.1
Clostridia bacterium
Wok-DUF2238
WP_220169827.1
Actinomadura parmotrematis
Wok-DUF2238
PIS02482.1
Chlamydiae bacterium CG10_big_fil_rev_8_21_14_0_10_42_34
Wok-DUF2238

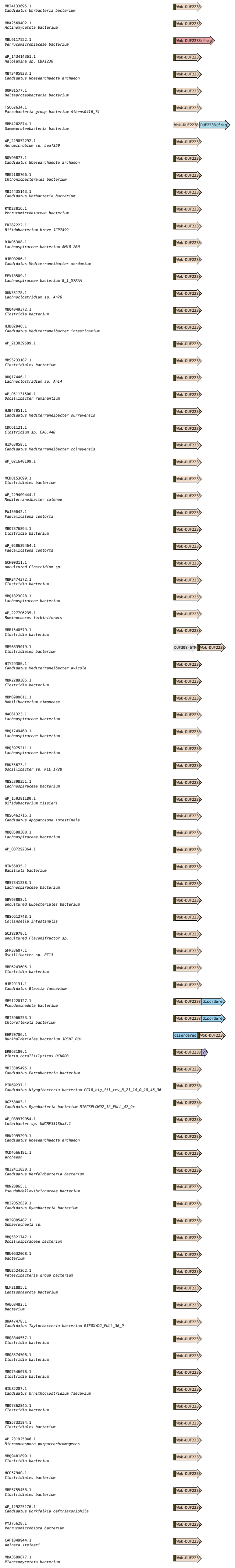
MBI4133695.1
Candidatus Uhrbacteria bacterium
Wok-DUF2238
MBA2569402.1
Actinomycetota bacterium
Wok-DUF2238
MBL9117552.1
Verrucomicrobiaceae bacterium
Wok-DUF2238(frag)
WP_143414361.1
Halolamina sp. CBA1230
Wok-DUF2238
MBT3405933.1
Candidatus Woesearchaeota archaeon
Wok-DUF2238
QQR81577.1
Deltaproteobacteria bacterium
Wok-DUF2238
TSC62634.1
Parcubacteria group bacterium Athens0416_74
Wok-DUF2238
MBM4202874.1
Gammaproteobacteria bacterium
Wok-DUF2238
DUF2238(frag)
WP_229052292.1
Aeromicrobium sp. Leaf350
Wok-DUF2238
NQV90877.1
Candidatus Woesearchaeota archaeon
Wok-DUF2238
MBE2180766.1
Chthoniobacterales bacterium
Wok-DUF2238
MBI4435143.1
Candidatus Uhrbacteria bacterium
Wok-DUF2238
RYD23016.1
Verrucomicrobiaceae bacterium
Wok-DUF2238
ERI87222.1
Bifidobacterium breve JCP7499
Wok-DUF2238
RJW05308.1
Lachnospiraceae bacterium AM40-2BH
Wok-DUF2238
HJB00286.1
Candidatus Mediterraneibacter merdavium
Wok-DUF2238
EFV18509.1
Lachnospiraceae bacterium 8_1_57FAA
Wok-DUF2238
OUN35178.1
Lachnoclostridium sp. An76
Wok-DUF2238
MBQ4049372.1
Clostridia bacterium
Wok-DUF2238
HJB82940.1
Candidatus Mediterraneibacter intestinavium
Wok-DUF2238
WP_213039589.1
Wok-DUF2238
MBS5733187.1
Clostridiales bacterium
Wok-DUF2238
OUQ17446.1
Lachnoclostridium sp. An14
Wok-DUF2238
WP_051131500.1
Oscillibacter ruminantium
Wok-DUF2238
HJB47051.1
Candidatus Mediterraneibacter surreyensis
Wok-DUF2238
CDC61121.1
Clostridium sp. CAG:448
Wok-DUF2238
HIX63058.1
Candidatus Mediterraneibacter colneyensis
Wok-DUF2238
WP_021648109.1
Wok-DUF2238
MCD8153699.1
Clostridiales bacterium
Wok-DUF2238
WP_229409444.1
Mediterraneibacter catenae
Wok-DUF2238
PWJ50042.1
Faecalicatena contorta
Wok-DUF2238
MBQ7376894.1
Clostridia bacterium
Wok-DUF2238
WP_050639464.1
Faecalicatena contorta
Wok-DUF2238
SCH00311.1
uncultured Clostridium sp.
Wok-DUF2238
MBR2474372.1
Clostridia bacterium
Wok-DUF2238
MBQ1823028.1
Lachnospiraceae bacterium
Wok-DUF2238
WP_227706235.1
Ruminococcus turbiniformis
Wok-DUF2238
MBR1540579.1
Clostridia bacterium
Wok-DUF2238
MBS6839019.1
Clostridiales bacterium
DUF308-6TM
Wok-DUF2238
HIY29306.1
Candidatus Mediterraneibacter avicola
Wok-DUF2238
MBR2289385.1
Clostridia bacterium
Wok-DUF2238
MBM6990011.1
Mobilibacterium timonense
Wok-DUF2238
HAC61323.1
Lachnospiraceae bacterium
Wok-DUF2238
MBQ1749460.1
Lachnospiraceae bacterium
Wok-DUF2238
MBQ3975211.1
Lachnospiraceae bacterium
Wok-DUF2238
ERK55673.1
Oscillibacter sp. KLE 1728
Wok-DUF2238
MBS5398351.1
Lachnospiraceae bacterium
Wok-DUF2238
WP_150381180.1
Bifidobacterium tissieri
Wok-DUF2238
MBS6462715.1
Candidatus Apopatosoma intestinale
Wok-DUF2238
MBQ8598380.1
Lachnospiraceae bacterium
Wok-DUF2238
WP_087292364.1
Wok-DUF2238
HIW56935.1
Bacillota bacterium
Wok-DUF2238
MBS7341238.1
Lachnospiraceae bacterium
Wok-DUF2238
SBV93888.1
uncultured Eubacteriales bacterium
Wok-DUF2238
MBS6612748.1
Collinsella intestinalis
Wok-DUF2238
SCJ82979.1
uncultured Flavonifractor sp.
Wok-DUF2238
SFP33607.1
Oscillibacter sp. PC13
Wok-DUF2238
MBP6243605.1
Clostridia bacterium
Wok-DUF2238
HJB29131.1
Candidatus Blautia faecavium
Wok-DUF2238
MBS1220127.1
Pseudomonadota bacterium
Wok-DUF2238
disordered
MBI3966253.1
Chloroflexota bacterium
Wok-DUF2238
disordered
EHR70706.1
Burkholderiales bacterium JOSHI_001
disordered
Wok-DUF2238
ERB63180.1
Vibrio coralliilyticus OCN008
Wok-DUF2238
??
MBI3305495.1
Candidatus Parcubacteria bacterium
Wok-DUF2238
PIR69237.1
Candidatus Niyogibacteria bacterium CG10_big_fil_rev_8_21_14_0_10_46_36
Wok-DUF2238
OGZ58003.1
Candidatus Ryanbacteria bacterium RIFCSPLOWO2_12_FULL_47_9c
Wok-DUF2238
WP_089979954.1
Luteibacter sp. UNCMF331Sha3.1
Wok-DUF2238
MBW2999299.1
Candidatus Woesearchaeota archaeon
Wok-DUF2238
MCD4666191.1
archaeon
Wok-DUF2238
MBI2411030.1
Candidatus Kerfeldbacteria bacterium
Wok-DUF2238
MBN20965.1
Pseudobdellovibrionaceae bacterium
Wok-DUF2238
MBI2052639.1
Candidatus Ryanbacteria bacterium
Wok-DUF2238
MBI9095487.1
Sphaerochaeta sp.
Wok-DUF2238
MBQ5321747.1
Oscillospiraceae bacterium
Wok-DUF2238
MBU0632068.1
bacterium
Wok-DUF2238
MBU2524362.1
Patescibacteria group bacterium
Wok-DUF2238
NLF21885.1
Lentisphaerota bacterium
Wok-DUF2238
MAE68482.1
bacterium
Wok-DUF2238
OHA47478.1
Candidatus Taylorbacteria bacterium RIFOXYD2_FULL_36_9
Wok-DUF2238
MBQ8844557.1
Clostridia bacterium
Wok-DUF2238
MBQ8574508.1
Clostridia bacterium
Wok-DUF2238
MBQ7546078.1
Clostridia bacterium
Wok-DUF2238
HIU82207.1
Candidatus Ornithoclostridium faecavium
Wok-DUF2238
MBQ7362845.1
Clostridia bacterium
Wok-DUF2238
MBS5733584.1
Clostridiales bacterium
Wok-DUF2238
WP_231925046.1
Micromonospora purpureochromogenes
Wok-DUF2238
MBQ9481899.1
Clostridia bacterium
Wok-DUF2238
HCG57940.1
Clostridiales bacterium
Wok-DUF2238
MBE5755458.1
Clostridiales bacterium
Wok-DUF2238
WP_129225176.1
Candidatus Borkfalkia ceftriaxoniphila
Wok-DUF2238
PYJ75628.1
Verrucomicrobiota bacterium
Wok-DUF2238
CAF1049944.1
Adineta steineri
Wok-DUF2238
MBA3699877.1
Planctomycetota bacterium
Wok-DUF2238

MBQ8425888.1	*Clostridia bacterium*	Wok-DUF2238
HIZ09266.1	*Candidatus Borkfalkia avicola*	Wok-DUF2238
MBS1313766.1	*Clostridia bacterium*	Wok-DUF2238
NNE93270.1	*Verrucomicrobiales bacterium*	Wok-DUF2238
HHU04795.1	*Fastidiosipila sp.*	Wok-DUF2238
MBL8858999.1	*Planctomycetota bacterium*	Wok-DUF2238
NLC89236.1	*Clostridiaceae bacterium*	Wok-DUF2238
WP_133517314.1	*Methanimicrococcus blatticola*	Wok-DUF2238
MBR1987501.1	*Clostridia bacterium*	Wok-DUF2238
MBE5747596.1	*Clostridiales bacterium*	Wok-DUF2238
MBQ7830992.1	*Clostridia bacterium*	Wok-DUF2238
MBQ3020040.1	*Clostridia bacterium*	Wok-DUF2238
MBQ9728354.1	*Clostridia bacterium*	Wok-DUF2238
QNF46090.1		Wok-DUF2238
WP_094533893.1	*Halorubrum halodurans*	Wok-DUF2238
WP_157267618.1	*Azohydromonas aeria*	Wok-DUF2238
MCB1232316.1	*Verrucomicrobiia bacterium*	Wok-DUF2238
WP_064735972.1	*Azohydromonas australica DSM 1124*	Wok-DUF2238
CAA9479472.1	*uncultured Solirubrobacteraceae bacterium*	Wok-DUF2238
WP_204548061.1		Wok-DUF2238
WP_110017297.1	*Plasticicumulans acidivorans*	Wok-DUF2238
MBA2855566.1		Wok-DUF2238
WP_126004250.1	*Sphingomonas koreensis*	Wok-DUF2238
MBU3671089.1	*Nevskiaceae bacterium*	Wok-DUF2238
MBT5048074.1	*Rhodospirillaceae bacterium*	Wok-DUF2238
PCJ38378.1	*Cellvibrionales bacterium*	Wok-DUF2238
KJS09945.1	*Gammaproteobacteria bacterium BRH_c0*	Wok-DUF2238
WP_052260628.1	*Photobacterium gaetbulicola*	Wok-DUF2238
WP_139531491.1	*Klebsiella michiganensis*	Wok-DUF2238
WP_088167741.1	*Klebsiella michiganensis*	Wok-DUF2238
MBU6211728.1	*Gammaproteobacteria bacterium*	Wok-DUF2238
MCB2117978.1	*Paracoccaceae bacterium*	Wok-DUF2238
KFN40146.1	*Sulfuricurvum sp. MLSB*	Wok-DUF2238
WP_066057047.1	*Halioglobus sp. HI00S01*	Wok-DUF2238
WP_132811499.1	*Sulfuricurvum sp. IAE1*	Wok-DUF2238
WP_169948286.1		Wok-DUF2238
WP_220341794.1	*Mumia sp. zg.B17*	Wok-DUF2238
MBM2809918.1	*Chloroflexota bacterium*	Wok-DUF2238
WP_051707235.1	*Nocardioides aequoreus*	Wok-DUF2238
WP_134129642.1	*Kribbella pratensis*	Wok-DUF2238
MBA3876563.1	*Anaerolinea sp.*	Wok-DUF2238
WP_214347881.1	*Cellulomonas fulva*	Wok-DUF2238
OQY81759.1	*Chloroflexi bacterium UTCFX4*	Wok-DUF2238
WP_025654360.1	*Faecalicatena contorta*	Wok-DUF2238
SHH94912.1	*Sporobacter termitidis DSM 10068*	Wok-DUF2238
HJA13239.1	*Candidatus Mediterraneibacter merdipullorum*	Wok-DUF2238
HIT10569.1	*Candidatus Onthousia faecigallinarum*	Wok-DUF2238
WP_021725455.1	*Olsenella profusa F0195*	Wok-DUF2238
MBR3098632.1	*Clostridia bacterium*	Wok-DUF2238
WP_229740704.1	*Ornithinibacillus halotolerans*	Wok-DUF2238
WP_101879528.1	*Mediterraneibacter gnavus*	Wok-DUF2238
MBS1348116.1	*Bacillota bacterium*	Wok-DUF2238
MBS7331738.1	*Eubacteriales bacterium*	Wok-DUF2238
MBS7217952.1	*Oscillospiraceae bacterium*	Wok-DUF2238
MBR3169995.1	*Lachnospiraceae bacterium*	Wok-DUF2238
WP_235837616.1	*Clostridium sp. Marseille-P3244*	Wok-DUF2238
MBR6399252.1	*Lachnospiraceae bacterium*	Wok-DUF2238
MBD5084401.1	*Clostridiales bacterium*	Wok-DUF2238
MBQ8447443.1	*Clostridia bacterium*	Wok-DUF2238
MBQ9746806.1	*Clostridia bacterium*	Wok-DUF2238
WP_216240908.1	*uncultured Faecalicatena sp.*	Wok-DUF2238
MBP7388261.1	*Gemmiger sp.*	Wok-DUF2238
WP_138284998.1	*Bifidobacterium bifidum*	Wok-DUF2238
OQM66042.1	*Bifidobacterium bifidum*	Wok-DUF2238
WP_033510118.1	*Bifidobacterium bifidum*	Wok-DUF2238
NBI11175.1	*Colidextribacter sp. OB.20*	Wok-DUF2238
MBR6727389.1	*Clostridia bacterium*	Wok-DUF2238
QSI27625.1	*Erysipelotrichaceae bacterium 66202529*	Wok-DUF2238
MBQ8585376.1	*Butyricicoccus sp.*	Wok-DUF2238
RKI68813.1	*bacterium 1xD42-67*	Wok-DUF2238
WP_233108411.1	*[Clostridium] innocuum*	Wok-DUF2238
MBS5041085.1	*Erysipelotrichaceae bacterium*	Wok-DUF2238
CVH77139.1	*Coriobacteriaceae bacterium CHKCI002*	Wok-DUF2238
EFP60590.1	*Erysipelotrichaceae bacterium 3_1_53*	Wok-DUF2238
ESL14106.1		Wok-DUF2238
WP_227115296.1	*Gordonibacter urolithinfaciens*	Wok-DUF2238
HJF64368.1	*Gordonibacter urolithinfaciens*	Wok-DUF2238
VUZ28281.1	*Acetobacterium wieringae*	Wok-DUF2238
WP_009588511.1	*Clostridium sp. 10cd**	Wok-DUF2238
WP_122644743.1	*Luxibacter massiliensis*	Wok-DUF2238
NBK14527.1		Wok-DUF2238
HIV24680.1	*Candidatus Scatomonas pullistercoris*	Wok-DUF2238
MBQ7859210.1	*Faecalibacterium sp.*	Wok-DUF2238
MCD7894512.1	*Erysipelotrichaceae bacterium*	Wok-DUF2238
WP_107631004.1	*Agathobaculum massiliense*	Wok-DUF2238
CDE82972.1	*Clostridium sp. CAG:273*	Wok-DUF2238
HIR14867.1	*Candidatus Choladousia intestinavium*	Wok-DUF2238
CCX64332.1	*Firmicutes bacterium CAG:791*	Wok-DUF2238
MCD7735222.1	*Clostridiales bacterium*	Wok-DUF2238
MBD9008858.1	*Clostridiales bacterium*	Wok-DUF2238
MBQ4353515.1	*Clostridia bacterium*	Wok-DUF2238
HIX47471.1	*Candidatus Mediterraneibacter caccavium*	Wok-DUF2238
WP_008728725.1	*[Clostridium] innocuum 2959*	Wok-DUF2238

WP_201783927.1	*Tractidigestivibacter scatoligenes*	Wok-DUF2238
WP_002606394.1	*[Clostridium] innocuum 2959*	Wok-DUF2238
WP_217793502.1	*[Clostridium] innocuum*	Wok-DUF2238
MBR0127842.1	*Bacillota bacterium*	Wok-DUF2238
WP_044904527.1	*[Clostridium] innocuum*	Wok-DUF2238
WP_117443265.1	*Clostridium sp. DFI.1.208*	Wok-DUF2238
MBS6179697.1	*Erysipelotrichaceae bacterium*	Wok-DUF2238
HIT63851.1	*Candidatus Ventrimonas merdavium*	Wok-DUF2238
MBU2159181.1	*Patescibacteria group bacterium*	Wok-DUF2238
NIP58646.1	*Gemmatimonadota bacterium*	Wok-DUF2238
TCS78807.1	*Muricomes intestini*	Wok-DUF2238
HIV22512.1	*Candidatus Merdiplasma excrementigallinarum*	Wok-DUF2238
MBR2984465.1	*Clostridia bacterium*	Wok-DUF2238
MBP3501462.1	*Oscillospiraceae bacterium*	Wok-DUF2238
MBR3043649.1	*Oscillospiraceae bacterium*	Wok-DUF2238
WP_204245182.1	*Anaeromassilibacillus sp. An172*	Wok-DUF2238
HIV98814.1	*Candidatus Ornithospirochaeta avicola*	Wok-DUF2238
WP_087157157.1	*Lachnoclostridium sp. An181*	Wok-DUF2238
HIQ78564.1	*Candidatus Scatomorpha intestinavium*	Wok-DUF2238
QNM09245.1	*Wansuia hejianensis*	Wok-DUF2238
MBO7358737.1	*Clostridia bacterium*	Wok-DUF2238
CDE17393.1	*Eubacterium sp. CAG:841*	Wok-DUF2238
MBR3995757.1	*Clostridia bacterium*	Wok-DUF2238
HJB13922.1	*Candidatus Oscillibacter excrementigallinarum*	Wok-DUF2238
MBS5576406.1	*Bacillota bacterium*	Wok-DUF2238
HJC14019.1	*Candidatus Agathobaculum intestinigallinarum*	Wok-DUF2238
MBE6564548.1	*Oscillospiraceae bacterium*	Wok-DUF2238
MBQ6859784.1	*Clostridia bacterium*	Wok-DUF2238
MBR3554954.1	*Oscillospiraceae bacterium*	Wok-DUF2238
WP_207752011.1		Wok-DUF2238
MBQ8025494.1	*Oscillospiraceae bacterium*	Wok-DUF2238
MBR2010613.1	*Clostridia bacterium*	Wok-DUF2238
MBR3059290.1	*Oscillospiraceae bacterium*	Wok-DUF2238
HIT71668.1	*Candidatus Scatovivens faecipullorum*	Wok-DUF2238
MBO5166795.1	*Lachnospiraceae bacterium*	Wok-DUF2238
WP_148567824.1	*Acetobacterium paludosum*	Wok-DUF2238
MBD5092568.1	*Clostridiales bacterium*	Wok-DUF2238
MBQ8281112.1	*Lachnospiraceae bacterium*	Wok-DUF2238
HBM99422.1	*Ruminococcus sp.*	Wok-DUF2238
MBS5330982.1	*Subdoligranulum variabile*	Wok-DUF2238
MBQ8523955.1	*Clostridia bacterium*	Wok-DUF2238
MBP1544615.1	*Oscillospiraceae bacterium*	Wok-DUF2238
HJD22377.1	*Candidatus Gemmiger faecigallinarum*	Wok-DUF2238
MBS1402264.1	*Oscillospiraceae bacterium*	Wok-DUF2238
MBQ7821691.1	*Clostridia bacterium*	Wok-DUF2238
HIV17032.1	*Candidatus Alectryocaccobium stercorigallinarum*	Wok-DUF2238
MBE6681387.1	*Oscillospiraceae bacterium*	Wok-DUF2238
MBR4872390.1	*Clostridia bacterium*	Wok-DUF2238
MCD8129415.1	*Oscillospiraceae bacterium*	Wok-DUF2238
MCC8078401.1	*Oscillospiraceae bacterium*	Wok-DUF2238
MCD7903344.1	*Oscillospiraceae bacterium*	Wok-DUF2238
MCD7852412.1	*Oscillospiraceae bacterium*	Wok-DUF2238
MCD8358036.1	*Oscillospiraceae bacterium*	Wok-DUF2238
MBP3464198.1	*Clostridia bacterium*	Wok-DUF2238
MBO4949744.1	*Clostridia bacterium*	Wok-DUF2238
MBQ9069734.1	*Clostridia bacterium*	Wok-DUF2238
MBQ8094870.1	*Clostridia bacterium*	Wok-DUF2238
MBI4855919.1	*Acetobacterium woodii*	Wok-DUF2238
MBQ8509081.1	*Clostridia bacterium*	Wok-DUF2238
MBQ8394745.1	*Clostridia bacterium*	Wok-DUF2238
MBR7099976.1	*Clostridia bacterium*	Wok-DUF2238
HIS73075.1	*Candidatus Scatomorpha pullicola*	Wok-DUF2238
MBQ8620339.1	*Clostridia bacterium*	Wok-DUF2238
MBO4631481.1	*Lentisphaeria bacterium*	Wok-DUF2238
MBQ9902769.1	*Clostridia bacterium*	Wok-DUF2238
MBQ3483516.1	*Clostridia bacterium*	Wok-DUF2238
MBR6274131.1	*Lachnospiraceae bacterium*	Wok-DUF2238
WP_102372462.1	*Enorma phocaeensis*	Wok-DUF2238
MBD8923668.1	*bacterium*	Wok-DUF2238
MBP1562389.1	*Oscillospiraceae bacterium*	Wok-DUF2238
CDE74327.1	*Clostridium sp. CAG:451*	Wok-DUF2238
PWM49823.1	*Bacillota bacterium*	Wok-DUF2238
HJB05590.1	*Candidatus Merdibacter merdigallinarum*	Wok-DUF2238
MBR6761241.1	*Oscillospiraceae bacterium*	Wok-DUF2238
WP_027295140.1	*Robinsoniella peoriensis*	Wok-DUF2238
MBR5302411.1	*Clostridia bacterium*	Wok-DUF2238
MBQ1554910.1	*Clostridia bacterium*	Wok-DUF2238
QUA53328.1	*Aristaeella lactis*	Wok-DUF2238
MBQ7880086.1	*Clostridia bacterium*	Wok-DUF2238
WP_207741538.1		Wok-DUF2238
MBQ3934430.1	*Clostridia bacterium*	Wok-DUF2238
WP_235418104.1	*Faecalicatena contorta*	Wok-DUF2238
MBQ5824748.1	*Clostridia bacterium*	Wok-DUF2238
MBQ9948770.1	*Oscillospiraceae bacterium*	Wok-DUF2238
WP_089275967.1	*[Clostridium] innocuum*	Wok-DUF2238
MBR2929642.1	*Clostridia bacterium*	Wok-DUF2238
WP_013758087.1	*Treponema brennaborense DSM 12168*	Wok-DUF2238
MBO4941700.1	*Clostridia bacterium*	Wok-DUF2238
MBO5999355.1	*Lachnospiraceae bacterium*	Wok-DUF2238
HIY96684.1	*Candidatus Borkfalkia excrementigallinarum*	Wok-DUF2238
MBP8715534.1	*Lachnospiraceae bacterium*	Wok-DUF2238
OUQ08329.1	*Erysipelatoclostridium sp. An15*	Wok-DUF2238
WP_147534274.1	*Bacillus marasmi*	Wok-DUF2238

WP_009139716.1	*Slackia piriformis*	Wok-DUF2238
MBQ9784509.1	*Clostridia bacterium*	Wok-DUF2238
MBQ6380862.1	*Clostridia bacterium*	Wok-DUF2238
MBQ8753216.1	*Clostridia bacterium*	Wok-DUF2238
MBQ8841179.1	*Ruminiclostridium sp.*	Wok-DUF2238
MBE6634848.1	*Oscillospiraceae bacterium*	Wok-DUF2238
WP_177268823.1	*Lentihominibacter sp.*	Wok-DUF2238
MBP3591399.1	*Clostridia bacterium*	Wok-DUF2238
MBQ3306703.1	*Bacilli bacterium*	Wok-DUF2238
MBR5261400.1	*Oscillospiraceae bacterium*	Wok-DUF2238
MBQ7965539.1	*Ruminococcus sp.*	Wok-DUF2238
MBQ8229314.1	*Clostridia bacterium*	Wok-DUF2238
MBS7157307.1	*Collinsella sp.*	Wok-DUF2238
MBS5428518.1	*Bacillota bacterium*	Wok-DUF2238
MBO4216710.1	*Clostridia bacterium*	Wok-DUF2238
HIT11234.1	*Candidatus Pelethosoma merdigallinarum*	Wok-DUF2238
MBR3210719.1	*Bacilli bacterium*	Wok-DUF2238
MBQ7096936.1	*Clostridia bacterium*	Wok-DUF2238
NVD96176.1	*Enterococcus faecium*	Wok-DUF2238
WP_129224720.1	*Candidatus Borkfalkia ceftriaxoniphila*	Wok-DUF2238
CDB88599.1	*Firmicutes bacterium CAG:170*	Wok-DUF2238
MBQ8861949.1	*Clostridia bacterium*	Wok-DUF2238
WP_231577062.1	*Salinicoccus sediminis*	Wok-DUF2238
WP_156921180.1	*Brochothrix campestris*	Wok-DUF2238
MBQ8535467.1	*Bacilli bacterium*	Wok-DUF2238
WP_113921126.1	*Alkalibaculum bacchi*	Wok-DUF2238
MBQ9955543.1	*Eggerthellaceae bacterium*	Wok-DUF2238
MBR2046743.1	*Agathobacter sp.*	Wok-DUF2238
MBR6949549.1	*Bacilli bacterium*	Wok-DUF2238
MBQ2910495.1	*Bacilli bacterium*	Wok-DUF2238
MBR2546800.1	*Eubacterium sp.*	Wok-DUF2238
WP_094886721.1	*Virgibacillus indicus*	Wok-DUF2238
WP_069817237.1	*Staphylococcus equorum*	Wok-DUF2238
WP_002506301.1	*Staphylococcus equorum*	Wok-DUF2238
MBO7342042.1	*Clostridia bacterium*	Wok-DUF2238
MBR1975043.1	*Clostridia bacterium*	Wok-DUF2238
WP_016623178.1		Wok-DUF2238
NLL62753.1	*Oscillospiraceae bacterium*	Wok-DUF2238
MBS7315010.1	*Clostridiaceae bacterium*	Wok-DUF2238
MBE6146593.1	*Bacillota bacterium*	Wok-DUF2238
WP_028273934.1	*Atopococcus tabaci DSM 17538*	Wok-DUF2238
MBR3679550.1	*Oscillospiraceae bacterium*	Wok-DUF2238
GAY78231.1	*Sporolactobacillus inulinus*	Wok-DUF2238
WP_070703704.1	*Enterococcus sp. HMSC076E04*	Wok-DUF2238
MBE6888940.1	*Oscillospiraceae bacterium*	Wok-DUF2238
WP_186578499.1	*Aquibacillus kalidii*	Wok-DUF2238
MBQ3419802.1	*Erysipelotrichaceae bacterium*	Wok-DUF2238
MBS5164510.1	*Butyricicoccus pullicaecorum*	Wok-DUF2238
MBQ8930680.1	*Oscillospiraceae bacterium*	Wok-DUF2238
MBQ4542750.1	*Clostridia bacterium*	Wok-DUF2238
MBR6703041.1	*Clostridia bacterium*	Wok-DUF2238
WP_146499670.1	*Planomicrobium sp. CPCC 101110*	Wok-DUF2238
WP_071393361.1	*Bacillus tuaregi*	Wok-DUF2238
MBQ6841541.1	*Bacilli bacterium*	Wok-DUF2238
MBQ2875578.1	*Clostridia bacterium*	Wok-DUF2238
WP_102028780.1	*Salirhabdus sp. Marseille-P4669*	Wok-DUF2238
MBQ1948424.1	*Clostridia bacterium*	Wok-DUF2238
WP_136137355.1	*Vagococcus silagei*	Wok-DUF2238
WP_204243870.1	*Thomasclavelia spiroformis*	Wok-DUF2238
MBO5906951.1	*Clostridia bacterium*	Wok-DUF2238
MBS7306785.1	*Eubacteriales bacterium*	Wok-DUF2238
WP_225418446.1	*Lacticaseibacillus daqingensis*	Wok-DUF2238
MBO5769871.1	*Spirochaetales bacterium*	Wok-DUF2238
CDF11917.1	*Mycoplasma sp. CAG:776*	Wok-DUF2238
WP_010014482.1	*Loigolactobacillus coryniformis*	Wok-DUF2238
WP_010010492.1	*Loigolactobacillus coryniformis subsp. coryniformis KCTC 3167 = DSM 20001*	Wok-DUF2238
CCY80078.1	*Mycoplasma sp. CAG:877*	Wok-DUF2238
MBE6148092.1	*Bacillota bacterium*	Wok-DUF2238
MBR1708730.1	*Clostridia bacterium*	Wok-DUF2238
MBE6156053.1	*Bacillota bacterium*	Wok-DUF2238
MBO4686740.1	*Lachnospiraceae bacterium*	Wok-DUF2238
WP_120142104.1	*Companilactobacillus zhachilii*	Wok-DUF2238
MBR6505345.1	*Candidatus Saccharibacteria bacterium*	Wok-DUF2238
HHX06865.1	*Erysipelothrix sp.*	Wok-DUF2238
CDE95011.1	*Clostridium sp. CAG:914*	Wok-DUF2238
MBQ8528378.1	*Clostridia bacterium*	Wok-DUF2238
MBQ7140242.1	*Bacilli bacterium*	Wok-DUF2238
WP_125569133.1	*Companilactobacillus insicii*	Wok-DUF2238
HAX73032.1	*Bacillota bacterium*	Wok-DUF2238
MBQ6236303.1	*Clostridia bacterium*	Wok-DUF2238
HIX07942.1	*Candidatus Borkfalkia faecipullorum*	Wok-DUF2238
NBI88327.1	*Lachnospiraceae bacterium*	Wok-DUF2238
MBQ8178121.1	*Clostridia bacterium*	Wok-DUF2238
MBR6740488.1	*Clostridia bacterium*	Wok-DUF2238
MBO7132180.1	*Candidatus Saccharibacteria bacterium*	Wok-DUF2238
MBQ9313614.1	*Clostridia bacterium*	Wok-DUF2238
MBQ2677351.1	*Bacillota bacterium*	Wok-DUF2238
MBQ8993882.1	*Turicibacter sp.*	Wok-DUF2238
TGY96648.1	*Petralouisia muris*	Wok-DUF2238
MBE6151237.1	*Bacillota bacterium*	Wok-DUF2238
MBR2258073.1	*Blautia sp.*	Wok-DUF2238
MBQ9064490.1	*Blautia sp.*	Wok-DUF2238
MBQ3298227.1	*Bacilli bacterium*	Wok-DUF2238

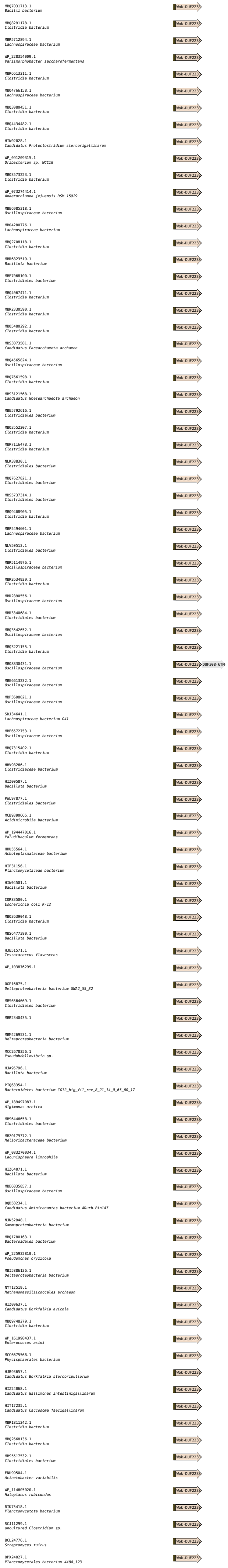
MBQ7031713.1
Bacilli bacterium
Wok-DUF2238
MBQ8291178.1
Clostridia bacterium
Wok-DUF2238
MBR5712894.1
Lachnospiraceae bacterium
Wok-DUF2238
WP_228354009.1
Variimorphobacter saccharofermentans
Wok-DUF2238
MBR6613211.1
Clostridia bacterium
Wok-DUF2238
MBO4766158.1
Lachnospiraceae bacterium
Wok-DUF2238
MBQ3080451.1
Clostridia bacterium
Wok-DUF2238
MBQ4434482.1
Clostridia bacterium
Wok-DUF2238
HIW02028.1
Candidatus Protoclostridium stercorigallinarum
Wok-DUF2238
WP_091209315.1
Oribacterium sp. WCC10
Wok-DUF2238
MBQ3573223.1
Clostridia bacterium
Wok-DUF2238
WP_073274414.1
Anaerocolumna jejuensis DSM 15929
Wok-DUF2238
MBE6985318.1
Oscillospiraceae bacterium
Wok-DUF2238
MBO4280776.1
Lachnospiraceae bacterium
Wok-DUF2238
MBQ2708118.1
Clostridia bacterium
Wok-DUF2238
MBR6823519.1
Bacillota bacterium
Wok-DUF2238
MBE7068100.1
Clostridiales bacterium
Wok-DUF2238
MBQ4067471.1
Clostridia bacterium
Wok-DUF2238
MBR2330590.1
Clostridia bacterium
Wok-DUF2238
MBO5480292.1
Clostridia bacterium
Wok-DUF2238
MBS3073581.1
Candidatus Pacearchaeota archaeon
Wok-DUF2238
MBQ4565824.1
Oscillospiraceae bacterium
Wok-DUF2238
MBQ7661598.1
Clostridia bacterium
Wok-DUF2238
MBS3121568.1
Candidatus Woesearchaeota archaeon
Wok-DUF2238
MBE5792616.1
Clostridiales bacterium
Wok-DUF2238
MBQ3552207.1
Clostridia bacterium
Wok-DUF2238
MBR7116478.1
Clostridia bacterium
Wok-DUF2238
NLK38830.1
Clostridiales bacterium
Wok-DUF2238
MBQ7627821.1
Clostridiales bacterium
Wok-DUF2238
MBS5737314.1
Clostridiales bacterium
Wok-DUF2238
MBQ9408905.1
Clostridia bacterium
Wok-DUF2238
MBP5494601.1
Lachnospiraceae bacterium
Wok-DUF2238
NLV50513.1
Clostridiales bacterium
Wok-DUF2238
MBR5114976.1
Oscillospiraceae bacterium
Wok-DUF2238
MBR2634929.1
Clostridia bacterium
Wok-DUF2238
MBR2890556.1
Oscillospiraceae bacterium
Wok-DUF2238
MBR3340684.1
Clostridiales bacterium
Wok-DUF2238
MBQ3542652.1
Oscillospiraceae bacterium
Wok-DUF2238
MBQ3221155.1
Clostridia bacterium
Wok-DUF2238
MBQ8830431.1
Oscillospiraceae bacterium
Wok-DUF2238
DUF308-6TM
MBE6613232.1
Oscillospiraceae bacterium
Wok-DUF2238
MBP3698021.1
Oscillospiraceae bacterium
Wok-DUF2238
SDJ34641.1
Lachnospiraceae bacterium G41
Wok-DUF2238
MBE6572753.1
Oscillospiraceae bacterium
Wok-DUF2238
MBQ7315402.1
Clostridia bacterium
Wok-DUF2238
HHV98266.1
Clostridiaceae bacterium
Wok-DUF2238
HIZ00587.1
Bacillota bacterium
Wok-DUF2238
PWL97877.1
Clostridiales bacterium
Wok-DUF2238
MCB9390665.1
Acidimicrobiia bacterium
Wok-DUF2238
WP_194447016.1
Paludibaculum fermentans
Wok-DUF2238
HHU55564.1
Acholeplasmataceae bacterium
Wok-DUF2238
HIF31156.1
Planctomycetaceae bacterium
Wok-DUF2238
HIW04501.1
Bacillota bacterium
Wok-DUF2238
CQR83500.1
Escherichia coli K-12
Wok-DUF2238
MBQ3639048.1
Clostridia bacterium
Wok-DUF2238
MBS6477380.1
Bacillota bacterium
Wok-DUF2238
HJE51571.1
Tessaracoccus flavescens
Wok-DUF2238
WP_103876299.1
Wok-DUF2238
OGP16875.1
Deltaproteobacteria bacterium GWA2_55_82
Wok-DUF2238
MBS6564669.1
Clostridiales bacterium
Wok-DUF2238
MBR2340435.1
Wok-DUF2238
MBM4269531.1
Deltaproteobacteria bacterium
Wok-DUF2238
MCC2678356.1
Pseudobdellovibrio sp.
Wok-DUF2238
HJA95796.1
Bacillota bacterium
Wok-DUF2238
PIQ63354.1
Bacteroidetes bacterium CG12_big_fil_rev_8_21_14_0_65_60_17
Wok-DUF2238
WP_189497083.1
Algimonas arctica
Wok-DUF2238
MBS6446658.1
Clostridiales bacterium
Wok-DUF2238
MBZ0179372.1
Melioribacteraceae bacterium
Wok-DUF2238
WP_083270034.1
Lacunisphaera limnophila
Wok-DUF2238
HIZ64071.1
Bacillota bacterium
Wok-DUF2238
MBE6835857.1
Oscillospiraceae bacterium
Wok-DUF2238
OQB58234.1
Candidatus Aminicenantes bacterium ADurb.Bin147
Wok-DUF2238
NJN52948.1
Gammaproteobacteria bacterium
Wok-DUF2238
MBQ1780163.1
Bacteroidales bacterium
Wok-DUF2238
WP_225932810.1
Pseudomonas oryzicola
Wok-DUF2238
MBI5886136.1
Deltaproteobacteria bacterium
Wok-DUF2238
NYT12519.1
Methanomassiliicoccales archaeon
Wok-DUF2238
HIZ09637.1
Candidatus Borkfalkia avicola
Wok-DUF2238
MBQ9748279.1
Clostridia bacterium
Wok-DUF2238
WP_161998437.1
Enterococcus asini
Wok-DUF2238
MCC6675568.1
Phycisphaerales bacterium
Wok-DUF2238
HJB93657.1
Candidatus Borkfalkia stercoripullorum
Wok-DUF2238
HIZ24068.1
Candidatus Gallimonas intestinigallinarum
Wok-DUF2238
HIT17235.1
Candidatus Caccosoma faecigallinarum
Wok-DUF2238
MBR1811242.1
Clostridia bacterium
Wok-DUF2238
MBQ2668136.1
Clostridia bacterium
Wok-DUF2238
MBS5517532.1
Clostridiales bacterium
Wok-DUF2238
ENU99504.1
Acinetobacter variabilis
Wok-DUF2238
WP_114605020.1
Haloplanus rubicundus
Wok-DUF2238
RIK75418.1
Planctomycetota bacterium
Wok-DUF2238
SCJ11299.1
uncultured Clostridium sp.
Wok-DUF2238
BCL24776.1
Streptomyces tuirus
Wok-DUF2238
OPX24027.1
Planctomycetales bacterium 4484_123
Wok-DUF2238

OGY58859.1	*Candidatus Colwellbacteria bacterium RIFCSPHIGHO2_12_FULL_44_17*	Wok-DUF2238
MBO5398162.1	*Clostridia bacterium*	Wok-DUF2238
WP_081283990.1	*Stenotrophomonas maltophilia*	Wok-DUF2238
WP_073275224.1	*Microbulbifer donghaiensis*	Wok-DUF2238
MBK7169734.1	*Gammaproteobacteria bacterium*	Wok-DUF2238
MBQ6165284.1	*Clostridia bacterium*	Wok-DUF2238
WP_124080687.1	*Pigmentiphaga humi*	Wok-DUF2238
MCA9364642.1	*Candidatus Moraniibacteriota bacterium*	Wok-DUF2238
MBP3327815.1	*Clostridia bacterium*	Wok-DUF2238
MBE6016873.1	*Lachnospiraceae bacterium*	Wok-DUF2238
MBE7076927.1	*Clostridiales bacterium*	Wok-DUF2238
HIZ72289.1	*Candidatus Gallimonas intestinavium*	Wok-DUF2238
MBW2387272.1	*Deltaproteobacteria bacterium*	Wok-DUF2238
RQD82818.1	*Methanosalsum natronophilum*	Wok-DUF2238
HHX78507.1	*Acholeplasmataceae bacterium*	Wok-DUF2238
MBQ9162431.1	*Clostridia bacterium*	Wok-DUF2238
MBP5585571.1	*Lentisphaeria bacterium*	Wok-DUF2238
MBR3564893.1	*Paludibacteraceae bacterium*	Wok-DUF2238
MBH0112993.1	*Novosphingobium aureum*	Wok-DUF2238
HBG37382.1	*Treponema sp.*	Wok-DUF2238
WP_177577049.1	*Hydrogeniiclostridium mannosilyticum*	Wok-DUF2238
EDS18617.1	*Thomasclavelia ramosa DSM 1402*	Wok-DUF2238
WP_080064693.1	*Ruminiclostridium hungatei*	Wok-DUF2238
HHU07935.1	*Clostridiaceae bacterium*	Wok-DUF2238
MBT7875131.1	*Verrucomicrobiota bacterium*	Wok-DUF2238
WP_224361627.1	*Hyalangium versicolor*	Wok-DUF2238
MBL4871794.1	*Robiginitomaculum sp.*	Wok-DUF2238
MBN2024308.1	*Pirellulales bacterium*	Wok-DUF2238
QQK06933.1	*Miniphocaeibacter halophilus*	Wok-DUF2238
MBS5384028.1	*Eubacterium sp.*	Wok-DUF2238
WP_211221950.1	*Lacticigenium naphthae DSM 19658*	Wok-DUF2238
NLA82496.1	*Clostridiaceae bacterium*	Wok-DUF2238
MBI2651468.1	*Candidatus Woesearchaeota archaeon*	Wok-DUF2238
MCB1843465.1	*Halioglobus sp.*	Wok-DUF2238
WP_145027638.1	*Caulifigura coniformis*	Wok-DUF2238
PIN93918.1	*Candidatus Pacearchaeota archaeon CG10_big_fil_rev_8_21_14_0_10_31_24*	Wok-DUF2238
OGQ55934.1	*Deltaproteobacteria bacterium RIFCSPLOWO2_02_FULL_53_8*	Wok-DUF2238
HGW74847.1		Wok-DUF2238
WP_231568067.1	*Sphingobium herbicidovorans*	Wok-DUF2238
MBB5948401.1		Wok-DUF2238
PNP94918.1	*Listeria newyorkensis*	Wok-DUF2238
MBP7791989.1	*Candidatus Goldiibacteriota bacterium*	Wok-DUF2238
MBP7587798.1	*Thermoanaerobaculia bacterium*	Wok-DUF2238
OKZ68990.1	*Clostridiales bacterium 52_15*	Wok-DUF2238
MCC2136903.1	*Hominenteromicrobium mulieris*	Wok-DUF2238
TMK96760.1	*Actinomycetota bacterium*	Wok-DUF2238
WP_201586187.1	*Psychrobacter phenylpyruvicus*	Wok-DUF2238
ERI98714.1	*Clostridium sp. ATCC 29733*	Wok-DUF2238
WP_066602444.1	*Desulfomicrobium orale DSM 12838*	Wok-DUF2238
HED36219.1	*Gammaproteobacteria bacterium*	Wok-DUF2238
UCG70017.1	*Thermoplasmata archaeon*	Wok-DUF2238
HCU69816.1	*Desulfomicrobium sp.*	Wok-DUF2238
HHX22044.1	*Clostridiales bacterium*	Wok-DUF2238
SDY38777.1	*Tessaracoccus flavus*	Wok-DUF2238
MBI2627139.1	*Parcubacteria group bacterium*	Wok-DUF2238
WP_235874600.1	*Pseudomonas sp. JV551A1*	Wok-DUF2238
MBS5571627.1	*Clostridiales bacterium*	Wok-DUF2238
MBR2391694.1	*Clostridia bacterium*	Wok-DUF2238
HAE87545.1	*Fidelibacterota bacterium*	Wok-DUF2238
MBX3365396.1	*Phycisphaeraceae bacterium*	Wok-DUF2238
MBQ9692580.1	*Clostridia bacterium*	Wok-DUF2238
PLX42997.1	*Deltaproteobacteria bacterium*	Wok-DUF2238
HCZ17191.1	*Accumulibacter sp.*	Wok-DUF2238
HCA46462.1	*Armatimonadota bacterium*	Wok-DUF2238
MBQ7338668.1	*Clostridia bacterium*	Wok-DUF2238
CDD01896.1	*Ruminococcus sp. CAG:382*	Wok-DUF2238
WP_145168968.1		Wok-DUF2238
HIU39810.1	*Candidatus Aphodocola excrementigallinarum*	Wok-DUF2238
MBW6442372.1	*Patescibacteria group bacterium*	Wok-DUF2238
MBR5862550.1	*Bacteroidales bacterium*	Wok-DUF2238
QKV56310.1	*Dechloromonas sp.*	Wok-DUF2238
WP_087239517.1	*Anaeromassilibacillus sp. An200*	Wok-DUF2238
WP_202748214.1	*Clostridium rhizosphaerae*	Wok-DUF2238
PKN52355.1	*Deltaproteobacteria bacterium HGW-Deltaproteobacteria-13*	Wok-DUF2238
GGS60381.1	*Streptomyces cinerochromogenes*	Wok-DUF2238
TMP90853.1	*Verrucomicrobiota bacterium*	Wok-DUF2238
MBQ3253981.1	*Acholeplasmatales bacterium*	Wok-DUF2238
WP_051646898.1	*Sharpea azabuensis DSM 18934*	Wok-DUF2238
MSR75293.1	*Planctomycetota bacterium*	Wok-DUF2238
WP_188983733.1	*Pseudomonas matsuisoli*	Wok-DUF2238
MCB5267530.1	*Candidatus Cloacimonadota bacterium*	Wok-DUF2238
NLO70266.1	*Porphyromonadaceae bacterium*	Wok-DUF2238
NLM33413.1	*Acholeplasmataceae bacterium*	Wok-DUF2238
MBR2338355.1	*Clostridia bacterium*	Wok-DUF2238
MBS7300325.1	*Eubacteriales bacterium*	Wok-DUF2238
MBP7016318.1	*Smithella sp.*	Wok-DUF2238
MBQ8749486.1	*Clostridia bacterium*	Wok-DUF2238
MBN1470881.1	*Syntrophaceae bacterium*	Wok-DUF2238
NLT70436.1	*Verrucomicrobiaceae bacterium*	Wok-DUF2238
MBE6964742.1	*Oscillospiraceae bacterium*	Wok-DUF2238
HIV63628.1	*Bacillota bacterium*	Wok-DUF2238
MBO7762204.1	*Clostridia bacterium*	Wok-DUF2238
HEB91397.1	*Deltaproteobacteria bacterium*	Wok-DUF2238

WP_142941157.1		Wok-DUF2238
PWL47897.1	*Clostridiales bacterium*	Wok-DUF2238
MBK8069549.1	*Rhodanobacteraceae bacterium*	Wok-DUF2238
MBS6510765.1	*Clostridiales bacterium*	Wok-DUF2238
MBR4919043.1	*Bacteroidales bacterium*	Wok-DUF2238
OQC29531.1	*Deltaproteobacteria bacterium ADurb.Bin072*	Wok-DUF2238
PZO82127.1	*Mesorhizobium amorphae*	Wok-DUF2238
NTU93667.1	*Chlorobiaceae bacterium*	Wok-DUF2238
MBP3284178.1	*Clostridia bacterium*	Wok-DUF2238
MBP6508058.1	*Opitutaceae bacterium*	Wok-DUF2238
WP_087936614.1	*Massilimicrobiota sp. An80*	Wok-DUF2238
MAG92145.1	*Planctomycetaceae bacterium*	Wok-DUF2238
MCB1243749.1	*Verrucomicrobiia bacterium*	Wok-DUF2238
MBL8850904.1	*Planctomycetaceae bacterium*	Wok-DUF2238
WP_006057362.1	*Holdemania filiformis*	Wok-DUF2238
MBQ8420392.1	*Clostridia bacterium*	Wok-DUF2238
WP_092593010.1	*Acidaminobacter hydrogenoformans DSM 2784*	Wok-DUF2238
MBP6059496.1	*Candidatus Saccharicenans sp.*	Wok-DUF2238
WP_072682999.1	*uncultured Holdemania sp.*	Wok-DUF2238
WP_106064023.1	*Clostridium liquoris*	Wok-DUF2238
MBS4955911.1	*Clostridium sp.*	Wok-DUF2238
TDI50734.1	*Acidobacteriota bacterium*	Wok-DUF2238
MBQ2794160.1	*Clostridia bacterium*	Wok-DUF2238
CAH1214238.1	*Paenibacillus allorhizoplanae*	Wok-DUF2238
WP_117895485.1	*Holdemania filiformis*	Wok-DUF2238
MBP6913439.1	*Candidatus Levyibacteriota bacterium*	Wok-DUF2238
WP_004029600.1	*Methanobacterium formicicum DSM 3637*	Wok-DUF2238
MBI5453228.1	*Deltaproteobacteria bacterium*	Wok-DUF2238
HHY64034.1	*Clostridiaceae bacterium*	Wok-DUF2238
WP_171650470.1	*Paenibacillus foliorum*	Wok-DUF2238
MBQ7523952.1	*Oscillospiraceae bacterium*	Wok-DUF2238
NCF27401.1	*Gammaproteobacteria bacterium*	Wok-DUF2238
HBR08923.1	*Clostridiales bacterium*	Wok-DUF2238
NLM15714.1	*Clostridiaceae bacterium*	Wok-DUF2238
NBX45573.1	*Gammaproteobacteria bacterium*	Wok-DUF2238
MBE9529347.1	*Pseudomonadota bacterium*	Wok-DUF2238
EHJ33628.1	*Lacticaseibacillus rhamnosus ATCC 21052*	Wok-DUF2238
WP_107789980.1	*Nitrosomonas nitrosa*	Wok-DUF2238
MBP8606900.1	*Clostridia bacterium*	Wok-DUF2238
HIY45614.1	*Candidatus Borkfalkia excrementipullorum*	Wok-DUF2238
MBO5046554.1	*Clostridia bacterium*	Wok-DUF2238
MBA3654701.1	*Actinomycetota bacterium*	Wok-DUF2238
HBV68870.1	*Clostridiales bacterium*	Wok-DUF2238
MBQ9792528.1	*Clostridia bacterium*	Wok-DUF2238
NCB85712.1	*Bacteroidia bacterium*	Wok-DUF2238
UCE31196.1	*Burkholderiales bacterium*	Wok-DUF2238
UCE05651.1	*bacterium*	Wok-DUF2238
WP_051620592.1	*Paenibacillus sp. UNC451MF*	Wok-DUF2238
OGO93192.1	*Clostridiales bacterium GWF2_38_85*	Wok-DUF2238
PWL43040.1	*Bacillota bacterium*	Wok-DUF2238
MBI9102385.1	*Spirochaetales bacterium*	Wok-DUF2238
WP_044279060.1	*Candidatus Cloacimonas acidaminovorans str. Evry*	Wok-DUF2238
WP_110839657.1	*Paenibacillus flagellatus*	Wok-DUF2238
MBQ6171692.1	*Clostridia bacterium*	Wok-DUF2238
WP_052963039.1		Wok-DUF2238
HHV33593.1	*Acholeplasma sp.*	Wok-DUF2238
WP_170157317.1	*Roseimicrobium gellanilyticum*	Wok-DUF2238
GIE07102.1	*Paractinoplanes durhamensis*	Wok-DUF2238
MBM7524454.1		Wok-DUF2238
MBW6520201.1	*Desulfoarculaceae bacterium*	Wok-DUF2238
MBS3099706.1	*Candidatus Pacearchaeota archaeon*	Wok-DUF2238
MBO5711354.1	*Acholeplasmatales bacterium*	Wok-DUF2238
PIN86091.1	*Candidatus Woesearchaeota archaeon CG10_big_fil_rev_8_21_14_0_10_44_13*	Wok-DUF2238
MBR1534861.1	*Ruminococcus sp.*	Wok-DUF2238
MBR3083456.1	*Oscillospiraceae bacterium*	Wok-DUF2238
WP_129969244.1	*Allopusillimonas soli*	Wok-DUF2238
EEI18758.1	*Lentilactobacillus buchneri ATCC 11577*	Wok-DUF2238
WP_003677623.1	*Loigolactobacillus coryniformis subsp. coryniformis CECT 5711*	Wok-DUF2238
WP_017930091.1	*Robiginitomaculum antarcticum DSM 21748*	Wok-DUF2238
WP_192700719.1	*Paenibacillus sp. OAS669*	Wok-DUF2238
MBR4546720.1	*Paludibacteraceae bacterium*	Wok-DUF2238
WP_196370823.1	*Acholeplasma laidlawii*	Wok-DUF2238
MBS6628935.1	*Clostridiales bacterium*	Wok-DUF2238
WP_138189910.1	*Culicoidibacter larvae*	Wok-DUF2238
TXI69719.1	*Limnohabitans sp.*	Wok-DUF2238
MBL0134542.1	*Chitinophagaceae bacterium*	Wok-DUF2238
MBQ6018810.1	*Clostridia bacterium*	Wok-DUF2238
NLX82882.1	*Clostridiales bacterium*	Wok-DUF2238
WP_154467452.1	*Eisenbergiella porci*	Wok-DUF2238
MBE6911491.1	*Oscillospiraceae bacterium*	Wok-DUF2238
ABC78009.1	*Syntrophus aciditrophicus SB*	Wok-DUF2238
MBU3056890.1	*Pseudomonas indica*	Wok-DUF2238
HIJ00808.1	*Candidatus Methanomethylophilaceae archaeon*	Wok-DUF2238
MBR4069395.1	*Clostridia bacterium*	Wok-DUF2238
MBM4363854.1	*Deltaproteobacteria bacterium*	Wok-DUF2238
QOJ17453.1	*Phycisphaeraceae bacterium*	Wok-DUF2238
MBT9612363.1	*Burkholderiales bacterium*	Wok-DUF2238
NIO41737.1	*Burkholderiales bacterium*	Wok-DUF2238
MSU34556.1	*Pedosphaera sp.*	Wok-DUF2238
WP_185394342.1	*Listeria fleischmannii*	Wok-DUF2238
HCS75518.1	*Clostridiales bacterium*	Wok-DUF2238
MBR5233218.1	*Clostridia bacterium*	Wok-DUF2238
MBO5170385.1	*Oscillospiraceae bacterium*	Wok-DUF2238

HHU80248.1	*Acholeplasmataceae bacterium*	Wok-DUF2238
MBN2931133.1	*Catenibacterium mitsuokai*	Wok-DUF2238
NCH38828.1	*Cronobacter sakazakii*	Wok-DUF2238
MBK1855944.1	*Oceaniferula flava*	Wok-DUF2238
WP_167223244.1	*Pelagibius litoralis*	Wok-DUF2238
MBI4413300.1	*Candidatus Methylomirabilota bacterium*	Wok-DUF2238
WP_209581323.1	*Methanobacterium aggregans*	Wok-DUF2238
MBO7526731.1	*Clostridia bacterium*	Wok-DUF2238
MBQ8891023.1	*Clostridia bacterium*	Wok-DUF2238
MBS3141934.1	*Candidatus Woesearchaeota archaeon*	Wok-DUF2238
WP_010962076.1	*Methylococcus capsulatus*	Wok-DUF2238
MBA2414212.1	*Burkholderiaceae bacterium*	Wok-DUF2238
WP_204434557.1	*Paracholeplasma morum*	Wok-DUF2238
MCC6558888.1	*Polyangiaceae bacterium*	Wok-DUF2238
RPH53919.1	*Lysobacterales bacterium*	Wok-DUF2238
MBZ5631598.1	*Terriglobia bacterium*	Wok-DUF2238
MCB5247291.1	*Candidatus Cloacimonadota bacterium*	Wok-DUF2238
MBQ1552014.1	*Clostridia bacterium*	Wok-DUF2238
MBD3248417.1	*Candidatus Woesearchaeota archaeon*	Wok-DUF2238
MBM6798066.1	*Coprobacillus cateniformis*	Wok-DUF2238
[truncated: 504,892 more chars]
